# Metabolic pattern across energy imbalance: An exploratory metabolomics study of female body weight extremes including anorexia nervosa and athletes

**DOI:** 10.1113/EP092957

**Published:** 2026-07-14

**Authors:** Sonja Lackner, Olli Kärkkäinen, Sabrina Mörkl, Wolfram Müller, Alfred Fürhapter‐Rieger, Kati Hanhineva, Sandra Johanna Holasek

**Affiliations:** ^1^ Division of Pathophysiology and Immunology, Otto Loewi Research Center Medical University of Graz Graz Austria; ^2^ Afekta Technologies Ltd Kuopio Finland; ^3^ School of Pharmacy University of Eastern Finland Kuopio Finland; ^4^ Department of Psychiatry and Psychotherapeutic Medicine Medical University of Graz Graz Austria; ^5^ Department of Medical Psychology, Psychosomatics and Psychotherapy Medical University of Graz Graz Austria; ^6^ Division of Biophysics, Gottfried Schatz Research Center Medical University of Graz Graz Austria; ^7^ Division of Physiology, Otto Loewi Research Center Medical University of Graz Graz Austria; ^8^ Food Sciences Unit, Department of Life Technologies University of Turku Turku Finland; ^9^ Institute of Public Health and Clinical Nutrition School of Medicine University of Eastern Finland Kuopio Finland

**Keywords:** anorexia nervosa, athletes, body composition, energy balance, lysophospholipids, plasma metabolomics, relative energy deficiency in sports (REDs)

## Abstract

In anorexia nervosa (AN), prolonged metabolic adaptation to severe energy deficiency can impair treatment efficacy. Similarly, low energy availability poses a recognized risk to athletes, potentially compromising health and performance. This cross‐sectional study aimed to characterize the plasma metabolic pattern of females with varying physical conditions, including AN patients, athletes, and normal weight, overweight and obese females. Non‐targeted metabolomics was applied to plasma samples from 107 females aged 18–40 years. Body fat was assessed via ultrasound and bioimpedance analysis. Nutritional intake, physical activity and clinical parameters were evaluated through standardized assessments. Sixty metabolites related to lipid, phospholipid and amino acid metabolism differed significantly between groups (ANOVA, false discovery rate *q* < 0.05). AN patients showed reduced long‐chain free fatty acids, medium‐chain acylcarnitines, etherphospholipids and branched‐chain amino acids (BCAAs) (Cohen's *d* < −0.5), alongside increased lysophospholipids and glycerophosphocholine (*d* > 0.6). Athletes exhibited a similar though less pronounced pattern, with preserved BCAA levels, while overweight and obese individuals displayed opposing patterns. Metabolites including fatty acids, acylcarnitines, BCAAs and phospholipids positively correlated with body fat (*r*_s_ > 0.25, *q* < 0.05), whereas lysophospholipids and glycerophosphocholine correlated negatively (*r*_s_ < −0.25, *q* < 0.05). The metabolic pattern in AN may be consistent with metabolism alterations in energy‐deficiency states, which may be associated with structural tissue loss. Athletes demonstrated similar shifts that may reflect adaptive responses to potential low energy availability, while overweight and obese profiles were consistent with energy surplus. These findings emphasize associations of systemic metabolism adaptations in energy‐imbalance states and highlight the importance of adequate energy intake in vulnerable groups to maintain metabolic and structural integrity and prevent adverse health consequences.

## Abstract (highlights)

- **What is the central question of this study?**
  Energy balance is reflected in plasma metabolic profiles: what are the distinct signatures across young females spanning energy deficiency, athletic high energy turnover and energy surplus?
- **What is the main finding and its importance?**
  Non‐targeted plasma metabolomics revealed partially convergent metabolic features in athletes and anorexia nervosa, characterized by increased lysophospholipids and reduced lipid‐related metabolites, whereas overweight and obesity showed opposing profiles, distinguishing energy‐deficient from energy‐surplus states. These exploratory findings suggest systemic adaptions to energy imbalance and highlight the importance of adequate energy intake in vulnerable groups to maintain metabolic and structural integrity.

## 1 INTRODUCTION

The lifetime prevalence of anorexia nervosa (AN) is estimated to be up to 4% of females and 0.3% of males (van Eeden et al., 2021). This severe disorder is associated with high relapse and mortality rates, reaching up to 10% (Papadopoulos et al., 2009). Core features of AN include persistent restriction of food intake leading to severe underweight, intense fear of weight gain, behavior that interferes with weight restoration, and a distorted perception of body weight or shape (American Psychiatric Association, 2022). Environmental, neurobiological and genetic factors contribute to the onset and maintenance of AN (Bulik et al., 2006). Despite remarkable research efforts, the biological mechanisms of AN remain incompletely understood, and no medications are effective in its treatment. A better understanding of metabolic and cellular mechanisms associated with extreme weight loss may help identify novel targets for therapeutic interventions.

Chronic energy deficiency is associated with weight loss and mobilization of alternative energy sources. While body fat stores are typically utilized first (Sakers et al., 2022), depletion beyond a critical point can result in breakdown of lean tissue such as muscle or organ mass, resulting in cachexia (Rohm et al., 2019). Interestingly, some AN patients appear to retain normal subcutaneous fat layers despite severely reduced body mass index (BMI), suggesting preferential degradation of other tissues (Lackner et al., 2019). In severe energy deficiency states, key physiological systems – including fertility and reproductivity (Boutari et al., 2020), bone metabolism (Tenforde, 2019) and the immune system (Lee & Dixit, 2020) – are downregulated.

These pathophysiological consequences of energy deficiency are not limited to individuals with AN but are increasingly recognized in sports medicine under the term ‘relative energy deficiency in sports’ (REDs) (Mountjoy et al., 2023). REDs results from low energy availability (LEA), defined as insufficient energy availability for physiological function after accounting for exercise energy expenditure. LEA is associated with inadequate energy intake relative to expenditure, often due to intensive training and combined with reduced appetite, time constraints or disordered eating behaviours. It affects endocrine function (Dipla et al., 2021) and impairs physical, mental and bone health as well as metabolic regulation (Dipla et al., 2021; Melin et al., 2019). Depending on the type of sport, the prevalence among female athletes ranges from 23% to 79.5% (Mountjoy et al., 2023).

In clinical practice, BMI and body weight changes are commonly used to monitor nutritional status and therapy progress. However, body composition, a key determinant of metabolic health (Goossens, 2017), remains often unconsidered. To understand the physiological consequences of energy imbalance more comprehensively, the integration of body composition analysis within metabolomic profiling is essential. Metabolomics, the systematic analysis of small molecules in biological samples, offers a powerful tool to investigate these adaptions, particularly in conditions such as AN, obesity and athletic training where profound lifestyle‐driven metabolic shifts occur (Newgard, 2017). Moreover, identifying early biomarkers for energy deficiency is crucial for improving treatment strategies in both AN (Himmerich & Treasure, 2024) and REDs (Melin et al., 2019), enabling earlier and more targeted interventions.

In this exploratory cross‐sectional study, we performed non‐targeted metabolomics on plasma samples from young adult white females of varying physiques including individuals with AN, athletes, normal weight controls, overweight and obesity. The aim was to compare metabolic profiles across these groups and to explore associations between plasma metabolites, body fat, and dietary intake, based on the hypothesis that specific metabolic patterns are associated with differences in energy balance and related physiological adaptions.

## 2 METHODS

### 2.1 Ethical approval

The study was conducted in accordance with the principles of the *Declaration of Helsinki* as revised in 2013. The study protocol and all procedures were approved by the Ethics Committee of the Medical University of Graz, Austria (approval reference number: MUG‐26–383ex13/14). All participants provided written informed consent prior to inclusion. The study was not registered in a public database, as it was a non‐interventional, cross‐sectional observational study.

### 2.2 Participants

This cross‐sectional study enrolled females from various energy‐status groups: AN patients, athletes, normal weight, overweight, and obese individuals, classified according to WHO BMI categories. We recruited AN patients, diagnosed by experienced clinical psychiatrists in structured diagnostic interviews, from three psychiatric clinics, athletes from local level ball sports teams, and the other groups through local universities and word‐of mouth in Graz, Austria from October 2014 until November 2015. Participants were examined overnight fasted (at least 12 h of fasting) and were instructed to abstain from exercise the evening before the investigation. Inclusion criteria comprised women aged 18–40 years, AN diagnosis according to ICD‐10 (World Health Organization, 2016) and athletes training at least 7 h weekly with regular competition participation. Exclusion criteria included antibiotic/antifungal treatment, prebiotic or probiotic use, acute/chronic diseases or infections within the previous 2 months, alcohol/drug dependence, major cognitive deficits, life‐threatening condition in AN, history of digestive diseases or gastrointestinal surgery (except appendectomy), and current pregnancy or breastfeeding.

### 2.3 Anthropometry and body composition assessment

Key anthropometric measures were determined following established recommendations of the International Society for the Advancement of Kinanthropometry.

#### 2.3.1 Ultrasound measurement

Subcutaneous adipose tissue (SAT) patterning was measured using ultrasound (US) (Müller et al., 2016), accurately and reliably (Müller et al., 2020) determining thin and thick fat layers in various individuals, including athletes and obese (Störchle et al., 2017). Measurements were taken at eight representative body sites using the GE Logiq US system (GE Healthcare, Milwaukee, WI, USA) with a linear probe (L8‐18i RS, operated at 8–16 MHz), analysed with the semiautomatic NISOS‐BCAv4.2 software (NISOS GmbH, Graz, Austria). Measures were International Association of Sciences in Medicine and Sports (IASMS)‐certified (International Association of Sciences in Medicine and Sports (IASMS), Graz, Austria). The total SAT at the eight sites was calculated (*D*_INCL_) for further analysis. Notably, the term ‘body fat’ is widely used although there are strong arguments to refer to adipose tissue preferentially (Clarys et al., 2010).

#### 2.3.2 Bioelectrical impedance analysis

Single frequency bioelectrical impedance analysis (BIA) (BIA 101, Akern, Pontassieve, Italy) was conducted at 50 kHz, following standard protocols (Kyle et al., 2004). Fat‐free mass (FFM) was calculated using the equation of Sun et al. (2003) and total body fat (TBF) was derived by subtracting FFM from body weight.

### 2.4 Lifestyle assessment: Smoking, physical activity, basal metabolic rate, menstrual cycle status and nutritional assessment

Smoking behaviour was determined by the Fagerström questionnaire (Fagerstrom, 1978) and physical activity levels by the International Physical Activity Questionnaire (IPAQ) (Craig et al., 2003). Basal metabolic rate was calculated according to the Mifflin–St Jeor equation (Mifflin et al., 1990).

Menstrual cycle status was assessed via self‐report of the first day of the last menstrual period. Based on this, participants were categorized into follicular (days 1–14) or luteal (days 15–28) phase. Additionally, the use of hormonal contraception and menstrual irregularities were documented.

Dietary intake was estimated from the average of two interviewer‐guided 24‐h recalls (Thompson & Subar, 2017), one representing the day before the blood draw and another on a random day 1–4 weeks thereafter. Nutritive values were calculated using nation‐specific software nut.s science v1.33.11 (dato Denkwerkzeuge, Vienna, Austria).

### 2.5 Laboratory parameters

Standard blood values were determined. Total cholesterol, high‐density lipoprotein (HDL) cholesterol, and triglycerides were measured using enzymatic reagents on a Cobas 8000 analyser (Roche Diagnostics, Mannheim, Germany). Low‐density lipoprotein (LDL) cholesterol concentrations were calculated using Friedewald's formula (Fukuyama et al., 2008). C‐reactive protein (CRP) was measured by a particle‐enhanced turbidimetric assay on a Cobas 6000 chemical routine analyser (Roche Diagnostics). Lecithin–cholesterol acyltransferase (LCAT) activity of serum was determined using a fluorometric assay kit (Merck, Darmstadt, Germany).

### 2.6 Metabolomics analysis

For the metabolomics analysis of plasma samples, a non‐targeted metabolomics approach utilizing ultra‐high performance liquid chromatography (UHPLC)–quadrupole time‐of‐flight (TOF) mass spectrometry was performed by Afekta Technologies Ltd, Kuopio, Finland (Klåvus et al., 2020). An aliquot of the plasma sample, 100 µL, was mixed with 400 µL of acetonitrile and mixed by pipetting. Samples were centrifuged at 18,000 *g* for 10 min at 4°C to filter through 0.2‐µm polytetrafluoroethylene filters in a 96‐well plate. Small aliquots (2–5 µL) were taken from the plasma samples, mixed in a 1 tube and used as the quality control (QC) sample in the analysis. These pooled QC samples were included to monitor analytical stability, analytical drift and reproducibility across the measurement sequence. QC samples were first used to stabilize the column, then injected every 12th sample and also used to produce autoMSMS data.

The samples were then analysed by liquid chromatography–mass spectrometry, consisting of a 1290 Infinity Binary UPLC coupled with a 6540 UHD Accurate‐Mass Q‐TOF (Agilent Technologies, Santa Clara, CA, USA). In brief, a Zorbax Eclipse XDB‐C18 column (2.1 × 100 mm, 1.8 µm; Agilent Technologies) was used for the reversed‐phase (RP) separation and an Aqcuity UPLC BEH amide column (Waters, Milford, MA, USA) for the hydrophilic interaction liquid chromatography (HILIC) separation. After each chromatographic run, the ionization was carried out using jet stream electrospray ionization (ESI) in the positive and negative mode, yielding four data files per sample. Data obtained from RP and HILIC separations and from positive and negative ionization modes were initially processed as separate datasets. Accordingly, the reported total number of detected features reflects the sum of molecular features across all modes. The modes were merged for statistical and multivariate analyses. Merging was performed at the level of annotated metabolites. If an identified metabolite was observed in several modes, a single representative molecular feature was selected to represent that metabolite in the results and for subsequent correlation analyses. Selection was based on the molecular feature quality, including ion abundance, peak shape, retention time consistency, and tandem mass spectrometry (MS/MS) spectral quality. No single predefined criterion (e.g., highest signal intensity alone) was applied; instead, selection was based on an overall assessment of feature quality to ensure robust and representative signals for downstream analysis. The collision energies for the MS/MS analysis were selected as 10, 20 and 40 V, for compatibility with spectral databases.

Metabolite abundances are reported as relative signal intensities derived from an untargeted workflow and therefore represent semi‐quantitative measurements. The non‐targeted metabolomics approach applied in this study was designed to assess relative metabolite patterns rather than absolute concentrations. Accordingly, internal standards were not used for absolute quantification. Subsequent analyses therefore focused on normalized relative abundances, which reduces the influence of global intensity differences between samples and mitigates potential matrix effects related to differences in total lipid content across study groups.

The mass spectrometry data processing was performed using MassHunter Profinder B.06.00 (Agilent Technologies) with standard settings. The batch recursive feature extraction function was used to extract ion to molecular features exhibiting isotopic peaks, dimers and common adducts, with an intensity threshold of at least 10,000. Final alignment and QC of peak spectra were done manually. Reported features needed to be present in >70% of the QC samples, with a relative standard deviation (RSD) value <0.2 and D‐ratio <0.4 in the QC samples.

The chromatographic and mass spectrometric characteristics (retention time, exact mass, and MS/MS spectra) of the significantly differential molecular features were compared with entries in an inhouse standard library and publicly available databases as well as with published literature. Identifications against *m*/*z*, retention time and MS/MS spectra from our in‐house library are marked as level 1, and identifications against *m*/*z* and MS/MS spectra from public databases or publications are marked as level 2.

### 2.7 Statistical analysis and visualization

Background variables were analysed using IBM SPSS Statistics v.29.0 (IBM Corp., Armonk, NY, USA) with the Shapiro–Wilk test assessing normal distribution. Data are reported using median (Md) and interquartile range (IQR). The Kruskal–Wallis test and pairwise comparisons, with Bonferroni correction for multiple testing, were used for group comparisons. Pearson's χ^2^ test assessed binary variables. Group comparison of metabolomics data was done with ANOVA using rank normalized abundance values, comparing AN, athletes, overweight and obese to normal weight controls. False discovery rate (FDR) correction (*q*‐value) was used to correct for multiple testing. For group‐wise comparisons, Welch's *t*‐test and Cohen's effect size were calculated. SIMCA (version 17, Umetrics, Sartorius Stedim Data Analytics AB, Umeå, Sweden) was used for the principal component analysis (PCA). The molecular features were scaled and centred by *z*‐score normalization. Cross‐validation was done by splitting the dataset into seven subsets. Correlations between metabolites and anthropometrics used Spearman correlations with FDR correction. Euclidean clustering grouped metabolites and variables in heatmaps. Figures were created with GraphPad Prism v9.0 (GraphPad Software, Boston, MA, USA) and the R package ComplexHeatmap.

## 3 RESULTS

### 3.1 Study population

One hundred and seven white females were allocated to groups AN (*n *= 18), athletes (*n *= 20), normal weight (*n *= 27), overweight (*n *= 22) and obese (*n *= 20) (Table 1).

**TABLE 1:** Study population characteristics.

	Anorexia nervosa	Normal weight	Overweight	Obese	Athletes	*P*
*n*	18	27	22	20	20	
**Demographic characteristics**						
Age (years)	22 (6)	24 (5)	24 (6)	26 (10)	21 (3)	0.00193^**^
Education (years)	13 (3)	17 (3)	18 (4)	16 (3)	15 (3)	<0.001^**^
**Anthropometry**						
BMI (kg/m^2^)	15.5 (2)	21.8 (3.4)	27 (1.3)	33 (4.4)	21.6 (2.5)	<0.001^**^
Waist (cm)	60 (5.3)	70 (7)	78.5 (6.3)	95.3 (17.5)	71 (6.7)	<0.001^**^
Hip (cm)	78.8 (4.8)	93 (8)	107 (7.9)	121.5 (13)	99 (5.6)	<0.001^**^
Upper‐arm (cm)	19.9 (2)	26.5 (2)	31.2 (2.6)	35.5 (4.1)	28 (2.3)	<0.001^**^
Triceps‐skinfold (mm)	7 (4.3)	19 (3)	26.5 (9.4)	34.5 (5)	14 (5.6)	<0.001^**^
**Body fat**						
**Ultrasound**						
*D*_INCL_ (mm)	30.2 (36.1)	83.6 (33.6)	140.8 (43.7)	196.9 (42.8)	58.4 (43.6)	<0.001^**^
Brachio radialis (mm)	1.1 (2.3)	4.2 (1.8)	6.7 (2.4)	7.6 (1.8)	3 (2.1)	<0.001^**^
Upper abdomen (mm)	4 (3.5)	12.2 (7.5)	24.4 (13.1)	44.9 (20)	7 (7.8)	<0.001^**^
Lower abdomen (mm)	5.7 (8.5)	17.3 (7.8)	33.4 (13.6)	52.7 (16.2)	15 (10)	<0.001^**^
External oblique (mm)	1.7 (2.7)	6.6 (5.6)	14.6 (6.8)	22.2 (11)	4.7 (6.9)	<0.001^**^
Errector spinae (mm)	2.9 (3.4)	7 (2.7)	13.1 (4.7)	23.1 (8.5)	5.5 (6.8)	<0.001^**^
Distal triceps (mm)	3.9 (5.2)	10.2 (5.6)	14.8 (5.8)	17.9 (6.1)	7.6 (3.9)	<0.001^**^
Front thigh (mm)	5 (5.4)	12.8 (4.3)	18.1 (5.2)	22.8 (10.8)	9.9 (4.6)	<0.001^**^
Medial calf (mm)	2.9 (4.6)	8 (4.5)	12.3 (6.2)	15.7 (6)	5.7 (2.9)	<0.001^**^
**Bioelectrical impedance analysis**						
Total body fat (kg)	4.1 (4.9)	17.2 (7.3)	28.2 (3.9)	41.1 (8.5)	16.8 (4.9)	<0.001^**^
Body cell mass (kg)	17.5 (2.8)	21.9 (2.4)	23.7 (2.4)	28.4 (4.4)	24.9 (5.1)	<0.001^**^
**Lifestyle measures**						
Smoking (%)	50	19	36.4	30	0	0.00577^**^
Fagerström score	4 (5)	0 (2)	1 (1)	3 (4)		0.0136^*^
IPAQ (MET‐min/week)	2200 (5008)	3764 (5260)	2486 (3897)	3100 (5792)	6013 (4139)	0.0183^*^
Basal metabolic rate (kcal/d)	1186 (108)	1395 (161)	1502 (147)	1700 (155)	1479 (111)	<0.001^**^
**Menstrual cycle status**						
Follicular/luteal phase (%)	44/17	52/37	59/27	20/55	55/45	0.101
Cycle irregularities (%)	39	11	14	25	0	0.0170^*^
Use of hormonal contraception (%)	44	22	36	20	20	0.299
**Nutritive assessment – macronutrient composition**						
Energy (kcal)	1919 (1472)	1894 (896)	1749 (328)	2065 (616)	2011 (585)	0.187
Carbohydrates (g)	233 (215)	201 (107)	189 (61)	210 (71)	233 (104)	0.240
Fat (g)	79 (37)	80 (49)	67 (21)	79 (33)	76 (35)	0.460
Saturated fat (g)	25 (18)	31 (21)	30 (12)	37 (9)	31 (16)	0.0331^*^
Protein (g)	79 (52)	62 (33)	61 (20)	74 (32)	74 (42)	0.072
Fibre (g)	19 (13)	21 (8)	17 (7)	17 (7)	24 (15)	0.0498^*^
Alcohol (g)	0.1 (0.6)	0.5 (9.2)	0.2 (7.5)	0 (0.1)	0.1 (0.7)	0.00945^**^
**Blood values**						
Cholesterol (mg/dL)	169 (57)	170 (57)	173 (45)	190.5 (61)	176.5 (49)	0.598
HDL‐cholesterol (mg/dL)	73 (15)	80 (20)	75 (23)	58 (24)	81.5 (20)	<0.001^**^
LDL‐cholesterol (mg/dL)	78 (43)	80 (41)	82 (44)	106.5 (47)	81 (19)	0.113
Triglycerides (mgl/dL)	78.5 (54)	66 (39)	78.5 (61)	105 (75)	75.5 (50)	0.0343^*^
CRP (mg/L)	0.6 (1)	1.3 (1.9)	1.45 (3.6)	5.3 (6.2)	0.65 (1.4)	<0.001^**^
LCAT activity (% substrate turnover)	28.4 (4.7)	26.8 (3)	27 (3.6)	30.7 (4.6)	28.6 (3.4)	<0.001^**^
Hb (g/dl)	13.4 (1.4)	13.9 (0.8)	13.8 (1.3)	14.0 (1.3)	13.3 (1.0)	0.106
Ferritin (µg/l)	38 (44)	34 (37)	49 (70)	35 (64)	22 (32)	0.313
Transferrin (mg/dl)	286 (64)	279 (79)	288 (56)	299 (37)	335 (64)	0.0278^*^
Iron (µg/dl)	84 (70)	94 (53)	94 (51)	85 (53)	84 (62)	0.822
Transferrin saturation (%)	20 (19)	23 (8)	24 (14)	21 (12)	20 (13)	0.477

Data are reported as median (IQR). Data are presented as median and interquartile range. ^*^ *P *< 0.05, ^**^ *P *< 0.01.

Abbreviations: BDI, Beck depression inventory; BIA, Bioelectrical impedance analysis; BMI, body mass index; CRP, C‐reactive protein; HAMD, Hamilton rating scale for depression; Hb, haemoglobin; IPAQ, International Physical Activity Questionnaire; LCAT, lecithin–cholesterol acyltransferase.

#### 3.1.1 Clinical information on AN patients

Mean ± SD age at disease onset was 21.8 ± 3.6 years and mean illness duration was 3.1 ± 3.5 years. Blood samples were acquired 11 ± 13 days after hospital admission. AN patients were under dietary treatment to regain weight. Five patients received high‐caloric supplements 7–19 days (mean ± SD 15 ± 6 days) before the investigation (Mörkl et al., 2017).

#### 3.1.2 Dietary intake

Although energy expenditure and the basal metabolic rate varied, study groups showed no significant difference in the reported energy and macronutrient intake, except for lower saturated fatty acids (FA) in AN vs. obese (*P* = 0.0124), higher fibre in athletes vs. overweight (*P* = 0.0417), and higher alcohol intake in normal weight vs. obese (*P* = 0.0128) (Table 1). Dietary amino acid, fatty acid and micronutrient profiles were analysed (Supporting information, Table S1). Significant differences in 11 proteinogenic amino acids (cysteine, histidine, isoleucine, leucine, methionine, phenylalanine, serine, threonine, tryptophan, tyrosine and valine) were observed between the groups, but none remained significant after Bonferroni correction. AN patients had great variation in amino acid and micronutrients intake, related to nutrient‐rich dietary sip feeds for refeeding.

#### 3.1.3 Body composition and SAT patterning

**FIGURE 1:**
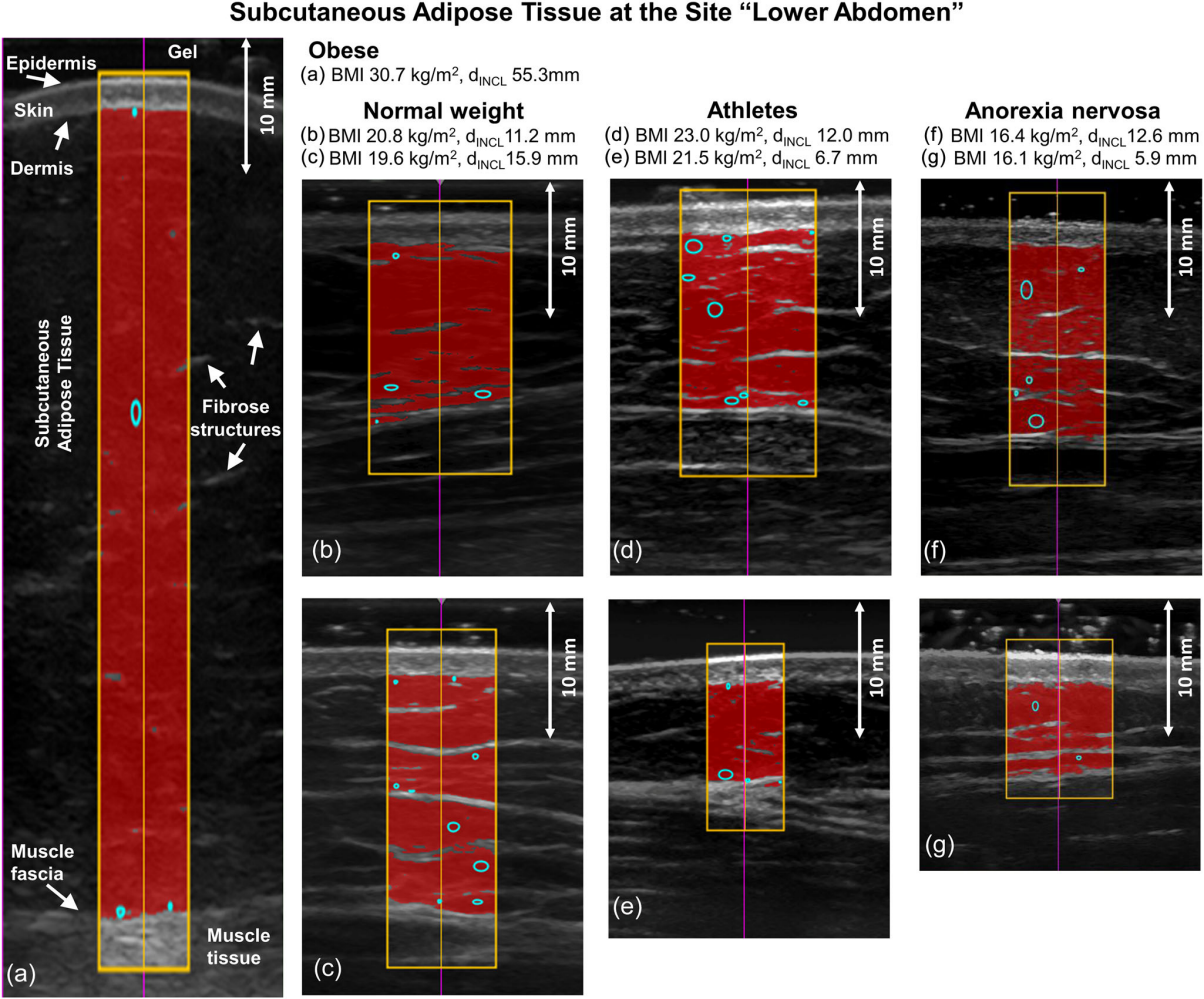
Differences in subcutaneous adipose tissue (SAT) thicknesses of the study cohort. The figure shows a comparison of SAT layers derived by the direct and highly accurate ultrasound method in different participants at the measurement site lower abdomen. The black area at the top of the ultrasound image represents a thick ultrasound gel layer applied to prevent tissue compression. The skin (epidermis and dermis) is clearly visible (two bright lines on top of the image), followed by the SAT layer, including diverse fibrous structures (fasciae) in the tissue (bright lines within this layer). SAT is highlighted in red; the lower border is the muscle fascia. (a) Obese participant: BMI 30.7 kg/m^2^; *d*_INCL_ 55.3 mm, *D*_INCL_ 216.4 mm; (b) normal weight control participant: BMI 20.8 kg/m^2^; *d*_INCL_11.2 mm, *D*_INCL_ 61.9 mm; (c) normal weight control participant: BMI 19.6 kg/m^2^; *d*_INCL_15.9 mm, *D*_INCL_ 53.7 mm; (d) athlete (non‐weight‐sensitive sport): BMI 23.0 kg/m^2^; *d*_INCL_ 12.0 mm, *D*_INCL_ 48.6 mm; (e) athlete (non‐weight‐sensitive sport): BMI 21.5 kg/m^2^; *d*_INCL_ 6.7 mm, *D*_INCL_ 36.9 mm; (f) anorexia nervosa patient: BMI 16.4 kg/m^2^; *d*_INCL_ 12.6 mm, *D*_INCL_ 58.2 mm; (g) anorexia nervosa patient: BMI 16.1 kg/m^2^; *d*_INCL_ 5.9 mm, *D*_INCL_ 31.9 mm. The images clearly show structural differences in SAT thicknesses and composition. For example, SAT of the two normal weight examples (b, c) differ in the amount of fibrous structures while (c) contained more that contribute to higher SAT thickness. AN patients differ completely in their body fat content despite the same BMI and show differences in fat patterning. (g) presented a larger amount of fibrose structures despite lower SAT thicknesses. *d*_INCL_: average thickness of SAT at the highlighted red region from dermis to muscle facia at the single site; *D*_INCL_: calculated sum of the SAT thicknesses at the eight body sites: brachio radialis, upper abdomen, lower abdomen, external oblique, front thigh, medial calf, distal triceps and erector spinae; SAT, subcutaneous adipose tissue.

SAT thickness sums (*D*_INCL_) and individual site measurements significantly varied across groups (Table 1, Figure 1). Notably, individuals with the same BMI showed vast differences in SAT layers, remarkably within the AN group. We previously reported the SAT variations in AN (Lackner et al., 2019); however, here we add the data from the other four groups and the correlations with the metabolite profiles. Some AN had fat layers comparable to normal weight females and athletes (Figure 1b, d, f), while others had substantially reduced fat layers (Figure 1g), suggesting reductions in muscle, organ and bone mass to maintain low body weight despite normal fat mass.

### 3.2 Metabolic profile of energy status groups – group comparison

**FIGURE 2:**
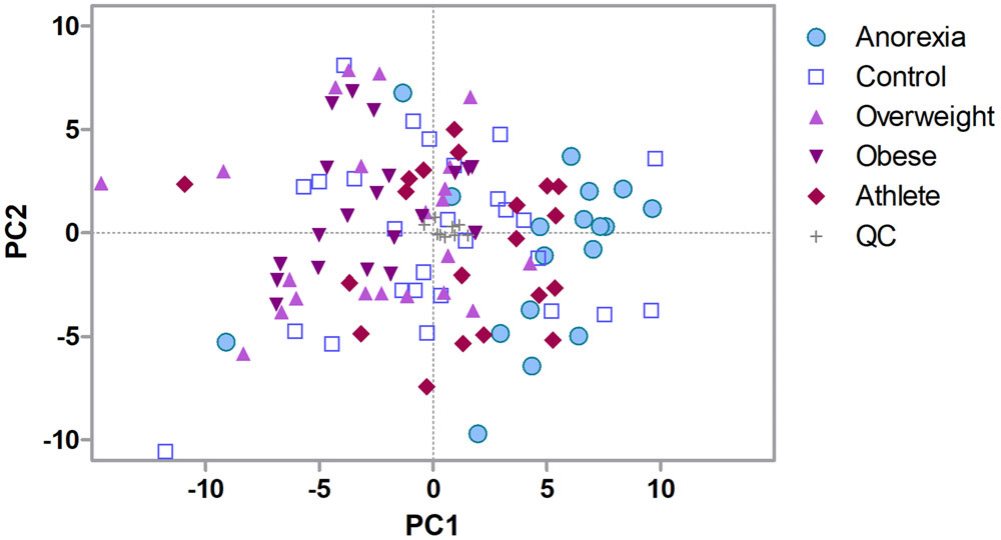
Principal component analysis. Score plot of first two principal components (PC) from principal components analysis. PC1 explains 14% of the variance in the data and separates most of the persons with anorexia nervosa to the right side of the graph. Similarly, athletes are clustered mostly on the right side and obese accumulated on the left side. PC2 explains 8% of the variance in the data but does not show clear separation of any group. Of note, controls were included in this visualization and were distributed equally in all quadrants. PC analysis shows no clear analytical drift in the QC samples injected every 12th injection during the metabolomics analysis. QC, quality control.

Untargeted metabolomics analysis resulted in a data matrix of 8948 molecular features detected across all chromatographic and ionization modes. After FDR correction for multiple testing, 1340 of these were significantly altered in the ANOVA comparison. Out of these, 60 individual metabolites related to lipid, phospholipid and amino acid metabolism were identified, which were significantly different between the investigated groups. PCA revealed a separation of AN, athletes and obese groups (Figure 2).

**FIGURE 3:**
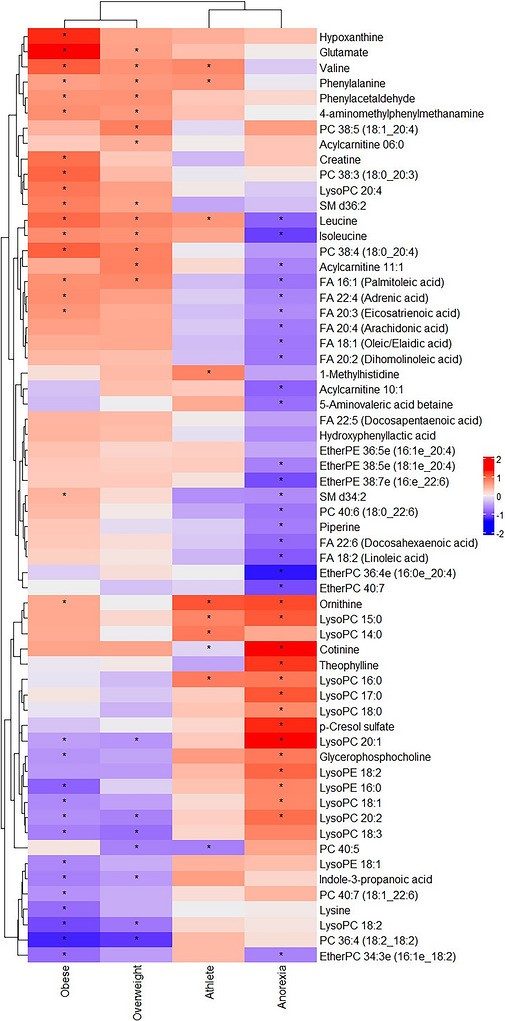
Metabolic differences between the study groups. Identified metabolites with FDR‐corrected *q*‐value from ANOVA comparison below 0.05 are shown. Cohen's *d* was used to assess the effect sizes: negative effect sizes are highlighted in blue, indicating lower values than normal weight controls; positive effect sizes are depicted in red and show higher values than controls. The *q*‐values compared to normal weight control groups revealed by Welch's *t*‐test are indicated with an asterisk (*q < 0.05). Both metabolites and groups are clustered with Euclidean clustering.

A distinct metabolic profile was obvious among the study groups. FA, medium chain acylcarnitines (AC), phospholipids and branched chain amino acids (BCAAs) displayed a clear pattern, showing significantly lower relative abundances in AN and higher relative abundances in overweight and obese compared to normal weight (Figure 3). Conversely, lysophospholipids (LysoPLs) showed significantly higher relative abundance in AN and lower relative abundance in overweight and obese. Athletes showed a more heterogeneous metabolic pattern, with similarities to AN in some metabolites like FA and LysoPLs, while others like AC and phospholipids were more comparable to normal weight (Figure 3).

#### 3.2.1 FA and AC

A total of nine FA and three AC were significantly divergent between the study groups. Thereof, the abundance of eight FA and two unsaturated, medium chain AC were significantly lower in AN compared to normal weight. In contrast to AN, the relative abundances of FA and AC were higher in overweight and obese compared to normal weight (Figure 3).

**FIGURE 4:**
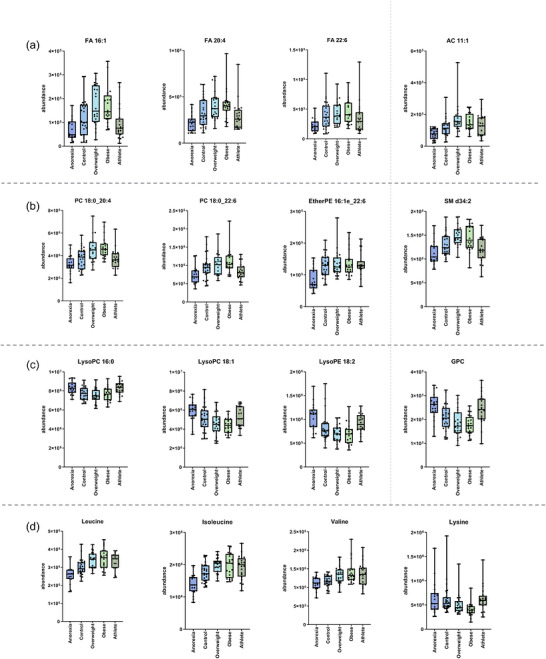
Plasma metabolite pattern related to energy metabolism across energy status groups. Overview of relative plasma metabolite abundances across study groups. (a) Free fatty acids and acylcarnitines: long‐chain free fatty acids (LCFFAs) and acylcarnitines (AC), which are involved in fatty acids transport and  β‐oxidation, showed lower relative abundance in anorexia nervosa (AN) patients during early refeeding compared to other groups. Relative abundance increased with higher BMI and were associated with greater body fat mass. (b) Phospholipids: several phospholipids, including phosphatidylcholines (PCs), phosphatidylethanolamines (PE), etherphosphatidylethanolamines (EtherPEs) and shingolipids (SM), showed lower relative abundance in AN. Athletes showed a similar pattern, except for shingolipids. (c) Lysophospholipids and glycerophosphocholine. Lysophosphatidylcholins (LysoPCs), lysophosphatidylethanolamines (LysoPEs), and glycerophosphocholine (GPC), which are products of phospholipid metabolism, showed higher relative abundance in AN and athletes, compared to other groups. (d) Amino acids: the branched chain amino acids leucin, isoleucine and valine, known for their contribution in energy metabolism, showed lower relative abundance in AN, whereas higher relative abundance was observed in overweight, obese and athletes. Lysine, which is an important mediator of muscle protein synthesis, showed lower relative abundance in overweight and obese individuals compared to other groups. Data are presented as relative metabolite abundances derived from non‐targeted metabolomics analysis.

**FIGURE A1:**
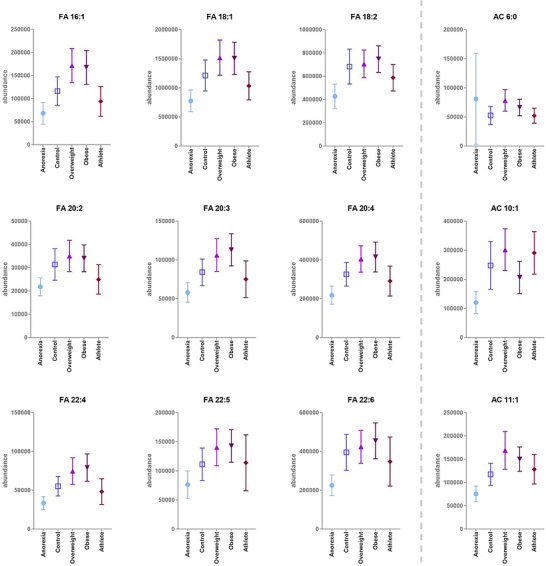
Fatty acids and acylcarnitines. In AN, fatty acids were lower compared to the other groups. The abundance of fatty acids increased continually from AN patients to obese participants, while the levels of the athlete group were intermediate between those of normal‐weight and AN groups. Regarding acylcarnitines, AN showed significantly lower levels, except for AC6:0, which exhibited considerable variation within the AN group. AC, acylcarnitine; AN, anorexia nervosa.

The relative abundance of FA and AC in plasma followed a typical pattern among the groups with a continuous increase from AN to obese, while the athletes’ values were in‐between those of AN and normal weight (Figure 4a, Appendix Figure A1). Besides these significant altered AC, several further AC showed a non‐significantly lower relative abundances in AN (Supporting information, Table S2).

#### 3.2.2 Phospholipids

**FIGURE A2:**
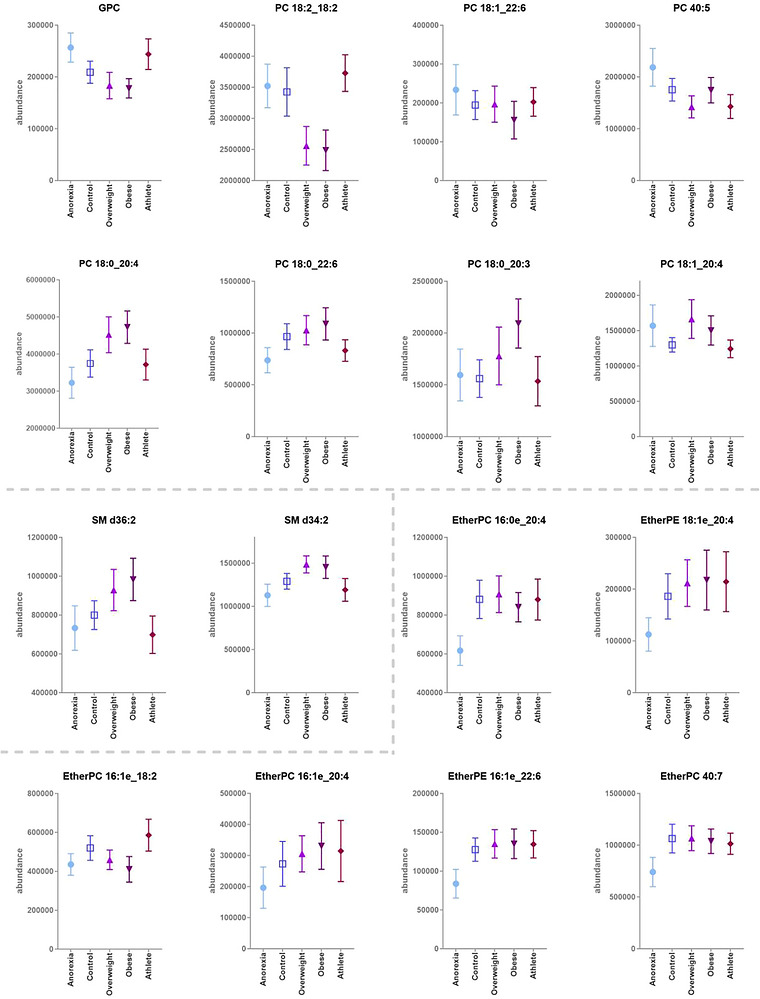
Phospholipids. Distribution of PC and PE among the studied groups. PC, phosphatidylcholines; PE, phosphatidylethanolamines.

Group comparison revealed significant differences in some phosphatidylcholines (PCs). 18:0‐containing PCs showed higher relative abundances in obese but slightly lower relative abundances in AN. Similarly, sphingolipids – crucial components of cell membranes and the nerve myelin layer – showed lower relative abundances in AN and significantly higher relative abundances in obese compared to normal weight (Figure 4b, Appendix Figure A2). Notably, AN exhibited lower relative abundances of several etherPCs and etherphosphatidylethanolamines (etherPEs).

**FIGURE A3:**
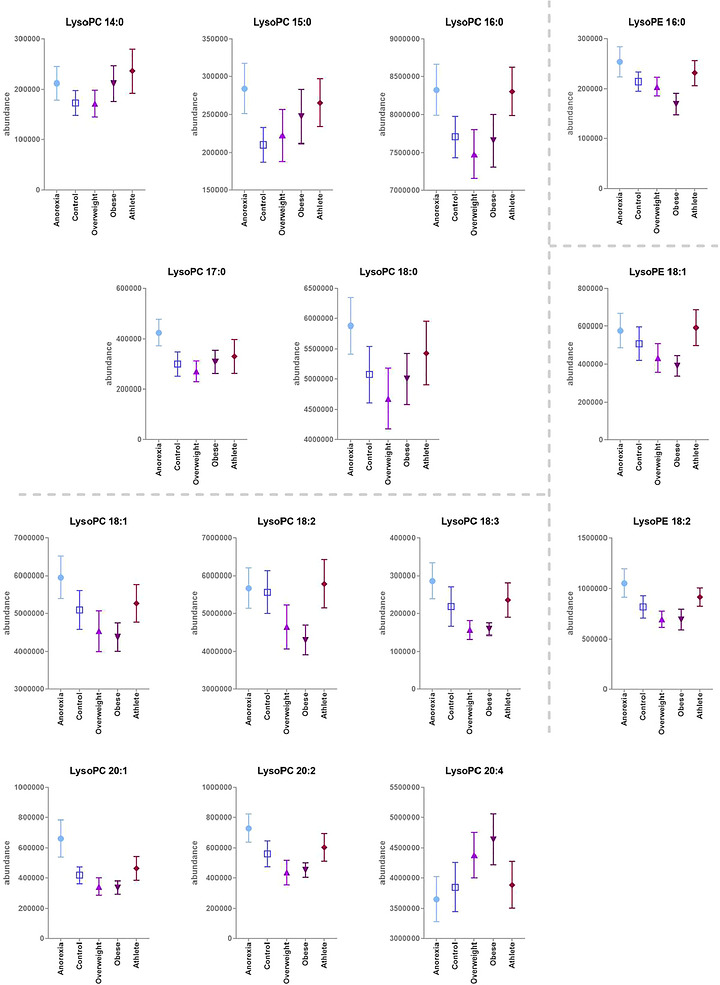
Lysophospholipids. Distribution of LysoPC and LysoPE among the studied groups. LysoPC, lysophosphatidylcholines; LysoPE, lysophosphatidylethanolamines.

By contrast, the relative abundance of glycerophosphocholine (GPC) was significantly higher in AN and lower in obese. In contrast to lower abundance of PCs and PEs in AN, seven LysoPCs and two LysoPEs showed significantly higher relative abundance in AN compared to normal weight, overweight and obese. Also in athletes, a similar pattern was observed (Figure 4c, Appendix Figure A3).

#### 3.2.3 Amino acids and other metabolites

The BCAAs leucine, isoleucine and valine showed lower relative abundances in AN, while they showed higher relative abundance in overweight and obese, and in athletes (Figure 4d). BCAAs are involved in energy metabolism. In contrast, lysine – an important mediator of muscle protein synthesis – exhibited a similar range between AN, normal weight and athletes. Conversely, lysine levels were relatively lower in overweight and obese.

Cotinine – a key metabolite of nicotine consumption – showed significantly higher relative abundance in AN and lower relative abundance in athletes, confirming the reported smoking behaviour and nicotine dependency measure. Moreover, 5‐aminovaleric acid betaine levels, a microbiota‐made metabolite associated with β‐oxidation of FA (Kärkkäinen et al., 2018), were less abundant in AN. Other significant variations in plasma metabolites among the study groups were observed (Figure 3) but will not be elaborated on in this paper.

### 3.3 Correlations of plasma metabolites, adipose tissue and SAT‐patterning, and dietary data

**FIGURE 5:**
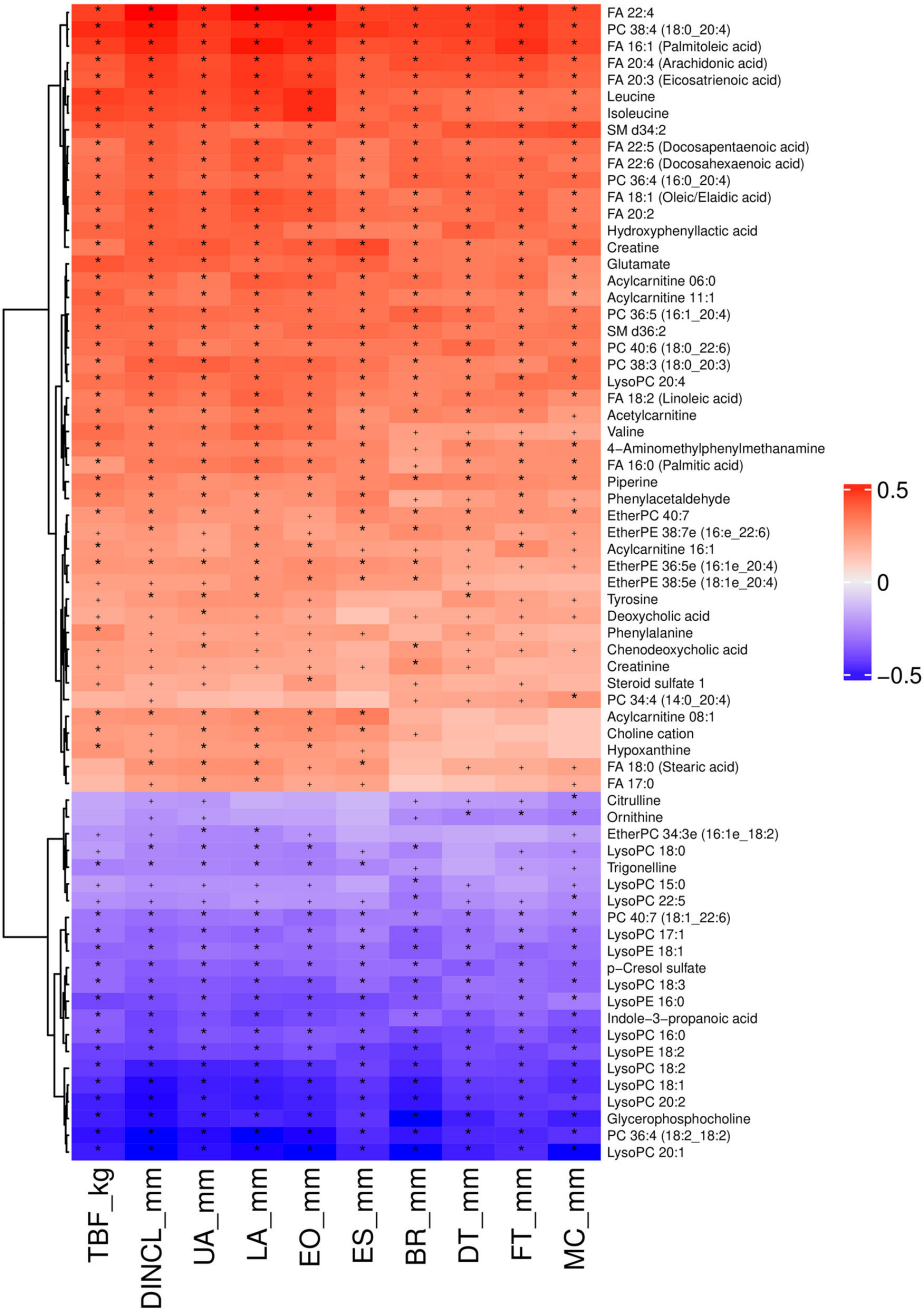
Association of body fat measures, body fat patterning and metabolic characteristics. Hierarchical clustering based on Euclidean distance was used to assess associations between body fat measures, fat distribution and metabolite abundances. Positive correlations were observed between body fat measures and long‐chain fatty acids, acylcarnitines, phospholipids, sphingolipids, and the branched‐chain amino acids leucine, isoleucine and valine. In contrast, inverse correlations were observed between body fat measures and lysophospholipids as well as glycerophosphocholine (GPC). Similar correlation patterns were observed for measures of total body fat (TBF [kg], *D*_INCL_ [mm]) and fat patterning (all eight ultrasound sites: UA, LA; EO, ES, BR, DT, FT, MC [mm]). Red depicts positive correlations, blue shows negative correlation of all identified metabolites with FDR corrected *q*‐value below 0.05 in at least one correlation. * denotes FDR corrected *q*‐value < 0.05; + denotes *P* < 0.05. *D*_INCL_, sum of subcutaneous adipose tissue thicknesses of the eight measurement sites including fibrous structures; BR, brachioradialis; DT, distal triceps; EO, external oblique; ES, erector spinae; FT, front thigh; LA, lower abdomen; MC, medial calf; TBF, total body fat; UA, upper abdomen.

Significant correlations were seen between the metabolites and TBF and SAT patterning (Figure 5). On whole, most LysoPCs and LysoPEs were negatively associated with body fat. In contrast, most FAs, PCs and other phospholipids were positively correlated with body fat.

**FIGURE A4:**
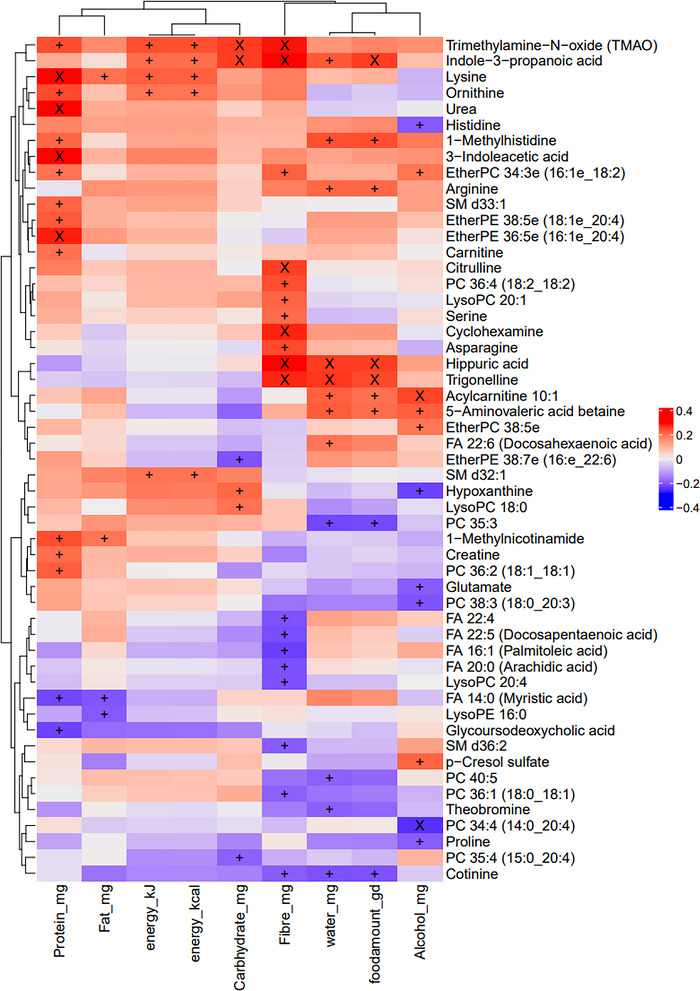
Correlation of dietary components and plasma metabolites. The heatmap shows the correlation between dietary variables and plasma metabolites. All identified metabolites with *P*‐value <0.05 in at least one correlation are shown. Euclidean clustering was performed to cluster metabolites and variables. Red indicates a positive correlation, whereas blue indicates a negative correlation. + denotes *P* < 0.05; × denotes FDR‐corrected *q*‐value < 0.05.

Several plasma metabolites and dietary intake values remained significant after FDR correction (Appendix Figure A4). The strongest positive correlations were found for total protein intake and lysine, urea, 3‐indoleacetic acid and etherPE36:5e (16:1e_20:4), as well as for total fibre intake and trimethylamine‐*N*‐oxide (TMAO), indole‐3‐propanoic acid, citrulline, cyclohexamine, hippuric acid and trigonelline.

## 4 DISCUSSION

This cross‐sectional study identified distinct plasma metabolite patterns associated with body composition and states of energy balance in young adult females. The main finding is that states of altered energy balance, particularly in individuals with AN, reflecting prolonged negative energy balance, and to a lesser extent in athletes, potentially reflecting LEA, are associated with distinct relative metabolomic patterns consistent with alterations in lipid metabolism. AN patients showed lower relative abundances of long‐chain free FA, medium‐chain AC, ether‐phospholipids and BCAAs, alongside higher relative abundances of LysoPLs and GPC. Athletes demonstrated a similar relative lipid‐related metabolomic profile but with preserved BCAA levels. In contrast, overweight and obese individuals displayed metabolomic profiles consistent with a state of positive energy balance, characterized by higher relative abundances of free FA, AC, phospholipids and amino acids, as well as lower relative abundances of LysoPLs and GPC.

These results suggest an association between body composition and lipid metabolism that may relate to differences in energy balance. The observation that low body fat is associated with increased LysoPLs is consistent with the hypothesis that energy may, in part, be derived from cellular membrane components in energy deficient states. Conversely, the metabolite profiles in overweight and obese participants may reflect reduced reliance on such mechanisms due to sustained energy stores.

### 4.1 Membrane phospholipid remodelling and its potential role in energy metabolism under conditions of energy deficiency

Phospholipids, the major components of cell membranes, play a crucial role in cell physiology. They consist of a glycerol backbone, a phosphate group bonded to the name‐giving group (e.g., choline for PC and ethanolamine for PE), and two fatty acid chains (Morita & Ikeda, 2022). When one fatty acid is linked with an ether‐bond instead of glycerol the molecule is referred to as an ether‐phospholipid, essential for membrane structure in neural, cardiac and immune tissues (Dean & Lodhi, 2018). LysoPLs are mainly formed by enzymatic cleavage of one fatty acid (Wang & Tontonoz, 2019), while GPC is yielded from cleavage of both FA (Nilsson & Duan, 2019). Both metabolites can exert bioactive effects in cellular signalling and membrane remodelling (Wymann & Schneiter, 2008); however, elevated levels of LysoPCs are associated with adverse health outcomes, including cardiovascular and neurodegenerative diseases (Kano et al., 2022; Law et al., 2019).

Importantly, Ray et al. (2016) highlighted the role of cell membrane lipids in energy homeostasis, emphasizing the plasma membrane's reservoir role in metabolism and stress adaptation. Thereby, plasma membranes act as a dynamic reservoir of bioactive lipids that can be utilized for metabolic adaptation and energy production. Under conditions of energy deficiency, membrane phospholipids may serve as a potential auxiliary energy source.

Following this concept, the higher relative abundance of LysoPLs and lower relative abundance of FFAs in the AN cohort and athletes are consistent with the possibility that FA may be mobilized from membrane phospholipids and subsequently utilized for energy production, which may be associated with their lower relative abundance in circulation. Moreover, in AN, higher relative abundance of GPC alongside lower relative abundance of circulating free FA (FFAs) may reflect increased membrane phospholipid turnover and subsequent utilization of released FA via β‐oxidation.

This interpretation is further supported by low concentrations of 5‐aminovaleric acid betaine (5‐AVAB), a microbiota‐derived inhibitor of β‐oxidation (Haikonen et al., 2022; Kärkkäinen et al., 2018), which may be compatible with increased mitochondrial fatty acid oxidation in AN. Similarly, higher relative abundance of GPC in athletes may indicate alterations in membrane phospholipid turnover as a potential adaptive response to increased energy demands. While α‐GPC is known as a supplement for improvement of cognitive and physical performance (Kansakar et al., 2023), none of our participants reported the use of such supplements. GPC has also been associated with increased hepatic fat oxidation (Kawamura et al., 2012), further linking it to energy metabolism. However, unlike in AN, 5‐AVAB levels were not lower in athletes but slightly more abundant compared to normal‐weight controls, which may indicate that β‐oxidation was not maximally upregulated in this group. However, as this study did not investigate β‐oxidation flux or membrane turnover, this interpretation remains speculative.

Even though the literature on performance and recovery in athletes describes adaptive processes in lipid metabolism (Fritzen et al., 2020; Muscella et al., 2020), these adaptations are strongly dependent on dietary intake and are typically associated with elevated circulating FFAs. Most known adaptations refer to triglyceride‐based lipolysis, involving the mobilization of lipids stored in adipocytes as classical energy reserves. In contrast, our observations may be consistent with an additional contribution of membrane‐derived lipids to energy metabolism. This deviates from classical endurance training responses and may reflect subtle energy deficiency, including LEA, and adaptive membrane remodelling.

Ether‐phospholipids were only relatively lower in AN, which may indicate alterations in membrane composition and potential impairment of antioxidative and neuroprotective lipid functions (Goodenowe & Senanayake, 2022), with the exception of the linolenic acid‐containing etherPC34:3e (16:1e_18:2), which was also lower in obese and overweight groups, suggesting a potential link to metabolic alterations and early membrane remodelling in states of overnutrition (Graessler et al., 2009).

Of note, there are alternative metabolic pathways related to LysoPC physiology. LysoPC can be reacylated to PC if sufficient acyl‐CoA is available (Law et al., 2019; Wang & Tontonoz, 2019). As these processes were not directly assessed in the present study, their contribution cannot be excluded. Mechanisms that could account for elevated plasma LysoPCs involve increased enzymatic cleavage by lipoprotein‐associated phospholipase A2 (Lp‐PLA2) or LCAT, as well as hypoxia‐induced glycolysis leading to LysoPC overproduction (Law et al., 2019). However, LCAT activity was not significantly elevated in AN (Stadler et al., 2022) or athletes (Table 1), and no signs of hypoxia were observed in the cohort, although these pathways cannot be definitively ruled out as pulmonary complications with reduced aerobic capacity are likely in AN (Puckett et al., 2021).

Taken together, these observations are consistent with previous literature on membrane lipid remodelling and lipid‐derived energy metabolism, but cannot be confirmed mechanistically within the present cross‐sectional study design and should therefore be interpreted as hypothesis‐generating.

### 4.2 Alteration of intracellular energy metabolism and amino acid utilization

ACs are critical for cellular energy metabolism, facilitating the transportation of FA into mitochondria for β‐oxidation (Li, S. et al., 2019), with circulating ACs representing intermediates of β‐oxidation (Dambrova et al., 2022). In our study, distinct AC profiles were observed across the groups, suggesting differences in energy metabolism. In AN, significantly lower relative abundance of medium‐chain ACs was observed, which may reflect limited availability of FA for β‐oxidation or efficient mitochondrial utilization of available lipids, leaving fewer intermediate products detectable in circulation.

Interestingly, AC6:0 was slightly higher (n.s.) in AN. As short‐chain AC can be considered a terminal product of repeated β‐oxidation cycles, its accumulation may be consistent with extensive fatty acid breakdown (Brejchova et al., 2024). Athletes showed a different profile: circulating AC concentrations were comparable or slightly higher (n.s.) compared to normal weight, which may indicate efficient lipid flux without accumulation of intermediates.

In overweight individuals, higher relative abundance of ACs may reflect an oversupply of FA and potential incomplete β‐oxidation. In obesity, this pattern was less distinct, with only slight changes, which may be consistent with early alterations in mitochondrial function. These contrasts are consistent with the interpretation that lower abundance of medium‐chain ACs in AN reflects substrate limitation and efficient lipid utilization, whereas increased levels in overweight/obese may indicate oversupply and reduced metabolic flexibility (Guerra et al., 2022). Consistent with previous literature, the athlete profile may reflect metabolic adaptation with high turnover and preserved efficiency (Hernández‐Saavedra et al., 2024).

BCAAs are key regulators of tissue protein metabolism. During prolonged starvation, they serve as energy substrates in skeletal muscle and act as signalling molecules in adipocytes (Nie et al., 2018; Zhang et al., 2017). The AN cohort in this study reflects a state of early nutritional rehabilitation following prolonged energy deficiency rather than untreated starvation, yet BCAA levels were relatively lower despite high dietary intake via sip feeds, which may indicate a persistent catabolic state with enhanced amino acid turnover and impaired retention in circulation.

Elevated BCAA levels have been linked to obesity and insulin resistance in metabolomics studies (Cheng et al., 2012; Lynch & Adams, 2014; Newgard et al., 2009; Ottosson et al., 2018). In our cohort, overweight, obese and athletes had higher plasma BCAAs compared to normal weight and AN patients (Figure 4d), consistent with prior findings (Ottosson et al., 2018).

Despite previous reports of reduced BCAAs in physically active individuals – due to increased oxidation during exercise, increased gluconeogenesis and lipolysis, and enhanced metabolic flexibility (Floegel et al., 2014; Kelly et al., 2020; San‐Millán, 2019) – our athletes showed no reduction. This may indicate a balance between elevated turnover and adaptive mechanisms maintaining BCAA availability for anabolic and regulatory functions under high physiological demand (Li et al., 2024).

Taking the metabolomic profile together, our findings in AN are supported by previous metabolomics studies (Hussain et al., 2023; Mayo‐Martínez et al., 2021; Tomášová et al., 2022). Föcker et al. (2012, 2020) reported increased ACs, PCs, sphingomyelins (SMs) and LysoPCs (Föcker, Manuel et al., 2020) in various stages of AN, consistent with our results. Similar trends in ACs and BCAAs, as well as LysoPCs and LysoPEs, were described by Miyata et al. (2021) and Tomášová et al. (2022), respectively. Notably, Tomášová et al. observed only slight metabolic normalization after treatment and highlighted persistent metabolic changes due to starvation (Tomášová et al., 2022).

Hussain et al. (2023) reported that during weight recovery in AN, LysoPLs (LysoPCs and LysoPEs) decrease with increasing energy intake but remain elevated compared to healthy controls, while phospholipid levels remained relatively stable. This suggests a gradual metabolic adaption rather than immediate normalization during refeeding. In our study, blood sampling was performed during early nutritional rehabilitation, on average 11 days after hospital admission. This time point reflects an early refeeding phase rather than a state of untreated starvation. Importantly, despite this early nutritional intervention, key metabolite patterns remained clearly distinct from healthy controls and were consistent with previously reported alterations in AN. This suggests that substantial components of the metabolic phenotype persist during early refeeding and are not immediately reversed by short‐term nutritional intake. At the same time, it cannot be excluded that some aspects of the metabolomic profile are influenced by recent nutritional intake or early metabolic adaptation. Therefore, the observed metabolite patterns likely reflect the metabolic state characteristic of early refeeding in AN, integrating both persistent effects of prior energy deficiency and initial adaptive responses to nutritional rehabilitation. This should be considered when interpreting the findings, particularly with regard to distinguishing between starvation‐related and recovery‐related metabolic processes.

In contrast, the overweight and obese groups in our cohort showed higher relative abundance of free FA, AC, phospholipids and amino acids, along with lower abundance of GPC and LysoPLs, pattern also reported by others (Cirulli et al., 2019; del Bas et al., 2016), which may reflect a chronic oversupply of dietary energy.

### 4.3 Similarities in plasma metabolite profile between AN and athletes

Our prior findings revealed that half of our AN cohort had SAT layers comparable to healthy females, despite their low body weight (Lackner et al., 2019) (Figure 1). This discrepancy may reflect loss of lean body mass, including muscle and organ tissue (Lackner et al., 2019). The observed higher relative abundance of LysoPLs and lower relative abundance of BCAAs in AN may be consistent with structural and metabolic alterations, associated with prior energy deficiency. Notably, these patterns were observed despite ongoing nutritional rehabilitation, suggesting that such alterations may persist during early refeeding and are not immediately reversed. However, given the cross‐sectional design, it cannot be determined whether these alterations reflect ongoing processes or residual effects of previous energy deficiency.

Interestingly, athletes displayed a partly similar metabolic pattern: lower free FA and higher abundance of LysoPLs and GPC, yet higher levels of BCAAs. This may indicate early metabolic adaptations to LEA without evidence of overt structural alterations.

These findings are particularly relevant in the context of REDs, a condition recognized for its adverse effects on athletes' health and performance, characterized by impaired physiological function due to insufficient energy intake relative to expenditure (Mountjoy et al., 2023). REDs has been associated with endocrine, cardiovascular and endothelial disturbances (Areta et al., 2021).

In our study athletes reported energy intake comparable to normal weights but exhibited higher energy expenditure (Table 1), which may be consistent with LEA in athletes, although no overt clinical signs of REDs such as amenorrhoea were observed. The observed metabolic patterns may be consistent with early metabolic adaptations to LEA. However, longitudinal and mechanistic studies are required to determine their temporal relationship to clinically apparent symptoms or structural changes, as described in early stages of REDs (Bachner‐Melman et al., 2006; Mountjoy et al., 2023).

### 4.4 Clinical implications

Established AN treatment approaches emphasize weight gain through high‐caloric diet and restricted exercise (Crone et al., 2023). While effective for initial weight restoration, such approaches may overlook the low muscle mass in patients who may have sufficient SAT (Lackner et al., 2019). Rapid weight gain without addressing body composition might oppose long‐term weight maintenance and elevate abdominal body fat accumulation (Hübel et al., 2019), potentially compromising compliance due to worsening body image (Gutierrez & Carrera, 2016). Sustainable recovery requires more than weight restoration alone. Previous studies suggest that implementing physical activity (Cook et al., 2016), particularly low‐repetition strength training during recovery, may aid in rebuilding muscle without excessive fat gain, key determinants of therapy success (Achamrah et al., 2016). Accurate body composition assessments may be useful to tailor therapy to each patient's needs.

Notably, the observed metabolic similarities between AN patients and athletes might be a hint to a shared vulnerability to states of impaired energy‐balance. Early detection of REDs is crucial but remains difficult due to a lack of sensitive biomarkers and screening tools (Mountjoy et al., 2023). Clinical symptoms such as amenorrhea and bone loss often appear only after prolonged LEA, making early detection challenging (Dipla et al., 2021). Conventional biomarkers (like leptin, insulin‐like growth factor 1 (IGF‐1) and thyroid hormones) have shown limited sensitivity for identifying short‐term or subclinical REDs (Heikura et al., 2022; Mathisen et al., 2023). LysoPLs may represent candidate markers associated with early metabolic adaptations to LEA. Further studies are needed to validate this potential as timely marker for REDs detection.

### 4.5 Strengths and limitations

This is the first metabolomics study to compare extreme body weight groups in a well‐characterized cohort. A major strength lies in the integration of standardized phenotyping with targeted nutritional assessments and non‐targeted plasma metabolomics. All participants were young adult women aged 18–40 years, resulting in a homogeneous cohort that reduces age‐ and sex‐related metabolic variability. The cohort was extensively phenotyped, allowing comparison across groups with differing body composition and likely differences in energy balance, including the difficult‐to‐recruit population of individuals with AN.

However, the cross‐sectional design limits conclusions regarding causality, treatment responses and long‐term metabolic adaptations. Despite the total sample size of *n* = 107, the individual group sizes remain relatively small and warrant validation in larger, longitudinal cohorts. Accordingly, the present findings should be interpreted as exploratory and hypothesis‐generating rather than confirmatory.

Nutritional intake was collected via 24‐h dietary recall interviews, which are subject to recall and reporting bias – particularly in individuals with AN, where food perception may be distorted. Nevertheless, the interviewer‐based approach enhances data quality compared to self‐administered questionnaires.

Several clinical parameters were assessed, including thyroid‐stimulating hormone, iron status, inflammatory markers and leptin. However, other established functional REDs biomarkers such as IGF‐1, oestradiol or triiodothyronine (T3) were not measured, and REDs status could not be formally assessed.

Finally, the study included only female participants. While this limits generalizability to sex‐specific differences in hormonal regulation and body composition, it also contributes to the growing field of gender‐specific metabolic research.

Given the untargeted nature of the metabolomics approach, the present findings should be interpreted as hypothesis‐generating and based on relative differences in metabolite abundances rather than absolute concentrations. Accordingly, the reported alterations reflect relative changes in signal intensities and should not be interpreted as absolute quantitative differences.

In addition, the absence of class‐specific internal standards does not allow complete exclusion of matrix‐dependent ion suppression or ion enhancement effects, which are recognized limitations of ESI mass spectrometry. As the investigated groups differed in body composition, metabolic status and circulating lipid profiles, differential matrix effects may have contributed to some of the observed lipid‐related differences between groups. While all samples were analysed under identical analytical conditions, the influence of group‐specific matrix composition on signal intensities cannot be fully excluded. Therefore, lipid‐related findings should be interpreted with appropriate caution and regarded as exploratory observations requiring confirmation in future targeted lipidomics studies employing class‐representative internal standards and dedicated quantitative workflows.

Future studies using targeted metabolomics and lipidomics approaches with appropriate internal standards, together with longitudinal study designs, are required to validate and quantify the observed alterations and to further investigate their biological significance.

### 4.6 Conclusion

This cross‐sectional study demonstrated distinct plasma metabolic patterns across female participants with widely varying energy and body composition statuses – ranging from AN and athletes to normal‐weight, and overweight and obese individuals.

Notably, AN patients undergoing rehabilitative treatment exhibited a metabolic pattern consistent with alterations in metabolic patterns that may reflect structural tissue mobilization. Athletes displayed similar patterns, which may be consistent with early adaptive responses to LEA, whereas obese participants showed metabolically opposing patterns, likely reflecting energy excess.

These findings may have implications for clinical considerations: the potential role of resistance and muscle‐strengthening exercise in AN treatment to counteract muscle and organ mass loss during refeeding; and the importance of adequate energy intake in athletes’ training regimens to prevent LEA and its associated health consequences.

Understanding the metabolic effects of energy deficiency is critical for the prevention of long‐term health risks, especially in individuals following restrictive diets or engaging in intense physical activity. This study suggests that energy status even in treated AN patients is associated with metabolic patterns that may be consistent with structural tissue alterations. The observed metabolite pattern in athletes may reflect early adaptive responses to LEA, although their clinical relevance requires further investigation. Future longitudinal and targeted studies are warranted to evaluate the effectiveness of targeted interventions in AN and to better characterize the metabolic consequences of energy imbalance in physically active populations.

## AUTHOR CONTRIBUTIONS

Conceptualization: Sandra Johanna Holasek, Kati Hanhineva; Data curation: Sonja Lackner, Olli Kärkkäinen, Sabrina Mörkl, Alfred Fürhapter‐Rieger, Formal Analysis; Sonja Lackner, Olli Kärkkäinen, Sabrina Mörkl; Funding acquisition: Sandra Johanna Holasek; Investigation: Sonja Lackner, Sabrina Mörkl, Alfred Fürhapter‐Rieger, Wolfram Müller; Methodology: Olli Kärkkäinen, Kati Hanhineva, Wolfram Müller, Alfred Fürhapter‐Rieger; Project administration: Sonja Lackner, Sabrina Mörkl, Sandra Johanna Holasek; Resources: Sandra Johanna Holasek, Kati Hanhineva, Olli Kärkkäinen; Software: Olli Kärkkäinen, Wolfram Müller, Alfred Fürhapter‐Rieger; Supervision: Sandra Johanna Holasek, Kati Hanhineva; Visualization: Sonja Lackner, Olli Kärkkäinen; Writing – original draft: Sonja Lackner; Writing – review & editing: all authors. All authors have read and approved the final version of this manuscript and agree to be accountable for all aspects of the work in ensuring that questions related to the accuracy or integrity of any part of the work are appropriately investigated and resolved. All persons designated as authors qualify for authorship, and all those who qualify for authorship are listed.

## CONFLICT OF INTEREST

O.K. and K.H. are founders of Afekta Technologies Ltd, a metabolomics analysis service company. W.M. and A.F.‐R. contributed to developing the commercially available image evaluation software used here and participate in the returns. All other authors do not have declared a conflict of interest.

## FUNDING INFORMATION

This research did not receive any specific grant from funding agencies in the public, commercial, or not‐for‐profit sectors.

## GENERATIVE AI STATEMENT

OpenAI ChatGPT (GPT 5.5) was used to assist with language editing and text refinement during manuscript revision. All scientific content, interpretations and conclusions were reviewed and approved by the authors, who take full responsibility for the manuscript.

## Supporting information



Supporting Materials

Table S1. Dietary intake data.

Table S2. Metabolomics analysis results.

## Data Availability

The data supporting this study's findings are not publicly available due to their sensitive nature and the potential risk of compromising participant privacy. In line with ethical guidelines and informed consent agreements, the data will remain confidential and not be shared outside the research team. However, anonymized or aggregated data may be available upon reasonable request, subject to ethics committee approval and compliance with data protection regulations.
