# Supplementary material for: Metabolic pattern across energy imbalance: An exploratory metabolomics study of female body weight extremes including anorexia nervosa and athletes
Source: Exp Physiol. 2026 Jul 14:10.1113/EP092957. Online ahead of print. doi: 10.1113/EP092957 (PMC13394067; doi:10.1113/EP092957)
Supplement: Supplementary file 1 — Supporting Materials [file EPH-9999-0-s002.pdf]

Supplemental Figures 1-4

Fatty Acids and Acylcarnitines

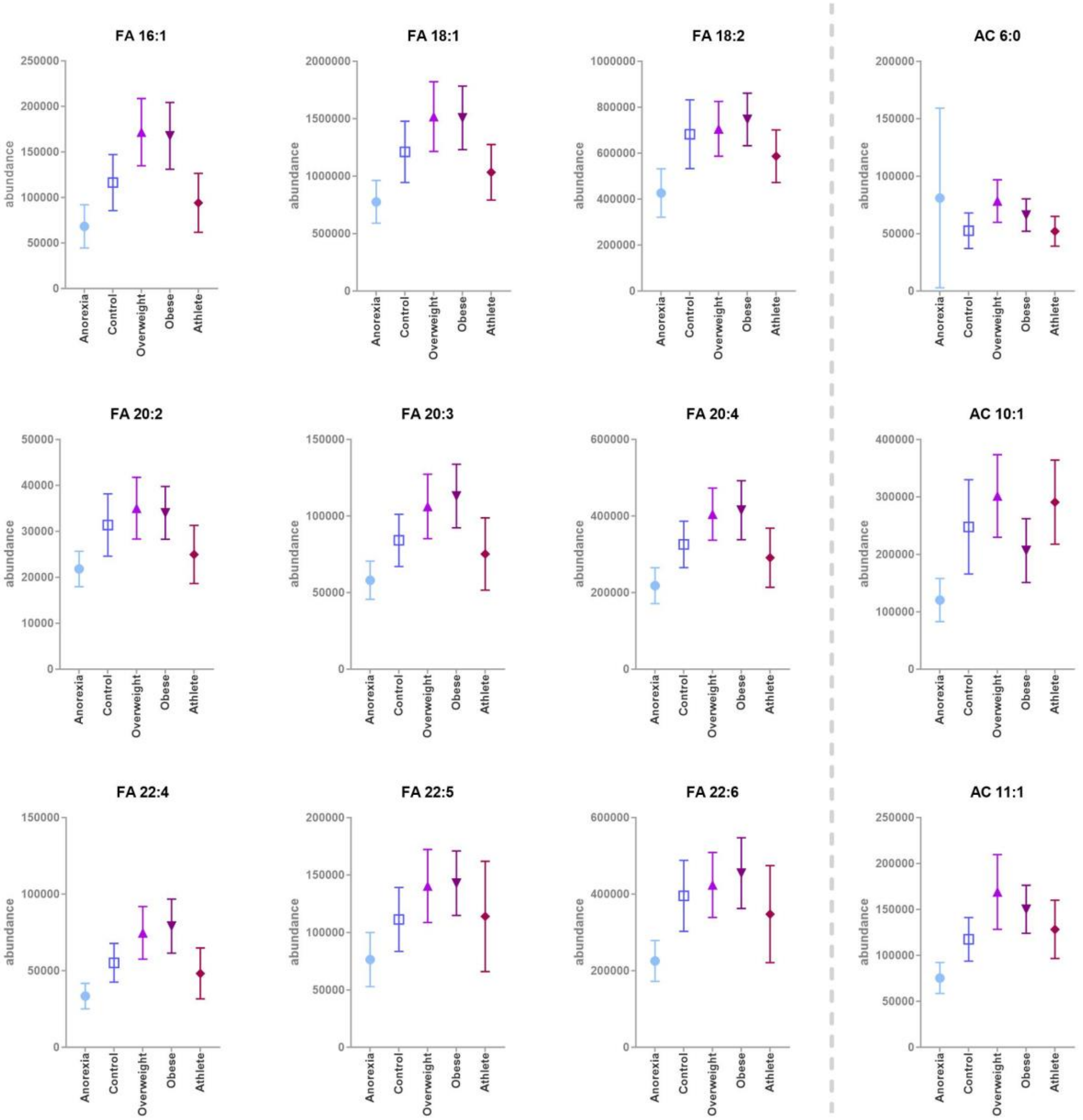

Supplemental Figure 1: Distribution of fatty acids and acylcarnites among the study groups.
In AN fatty acids were lower compared to the other groups. The abundance of fatty acids increased continually from AN patients to obese participants, while the levels of the athletes group were between normal weight and AN. Regarding acylcarnitines, AN had significantly lower levels except for AC6:0 which showed a great variation within the AN group.
AC: acylcarnitine, AN: anorexia nervosa

Phospholipids

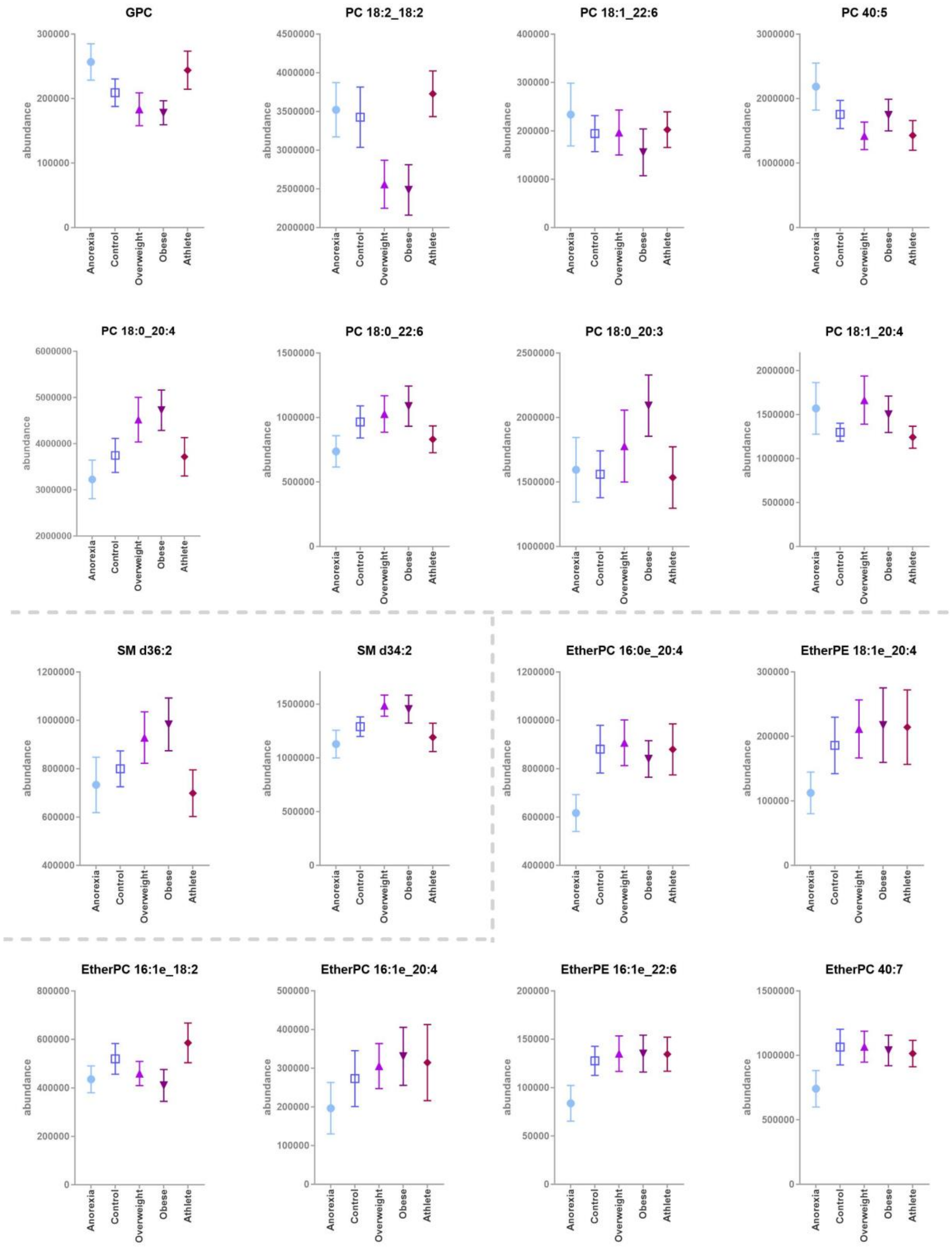

Supplemental Figure 2: Phospholipids: Phosphatidylcholines (PC) and Phosphatidylethanolamine (PE)

Lysophosphatidylcholins (LysoPC) and Lysophosphatidylethanolamines (LysoPE)

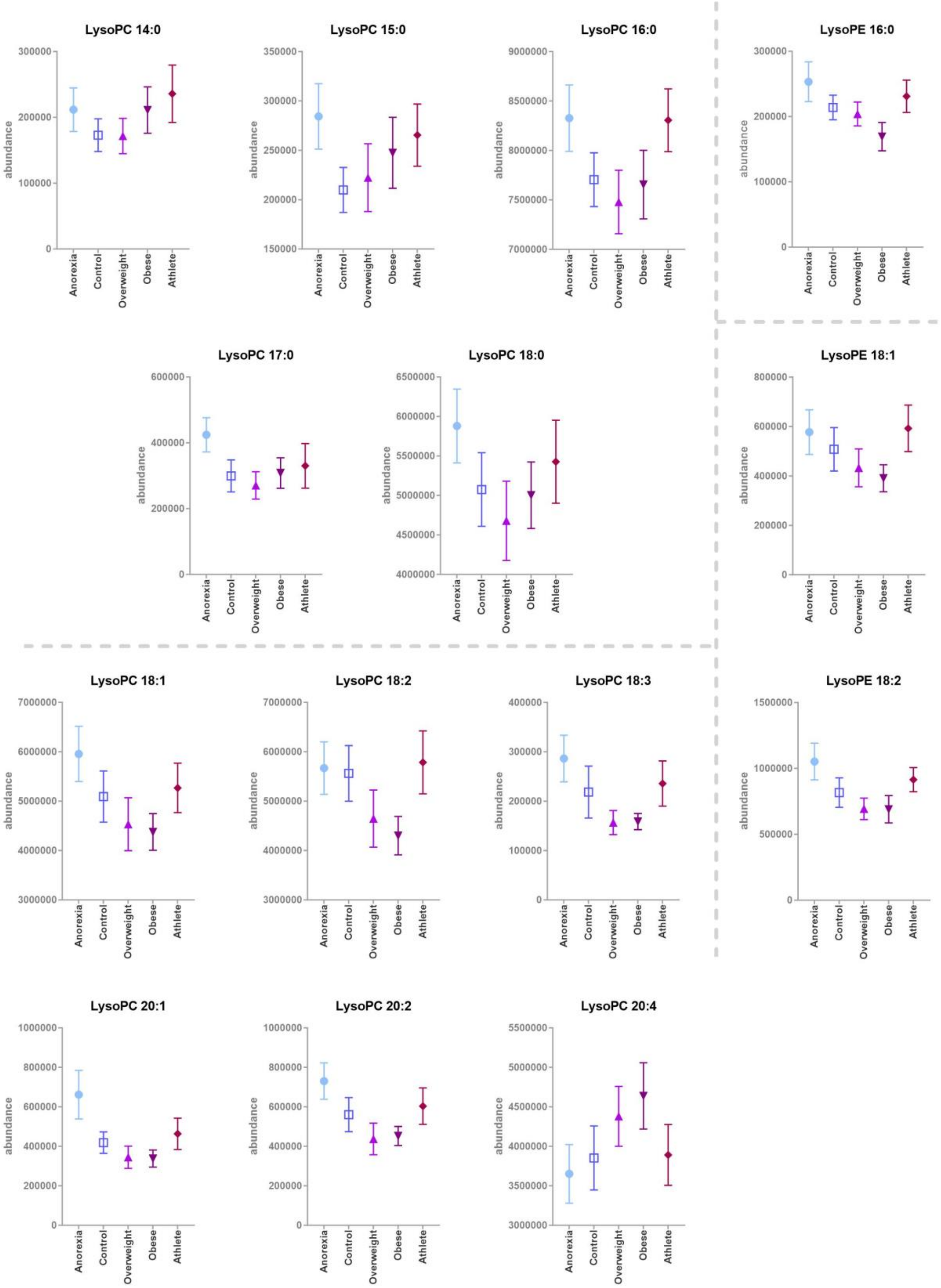

Supplemental Figure 3: Lysophosphatidylcholins (LysoPC) and Lysophosphatidylethanolamines (LysoPE)

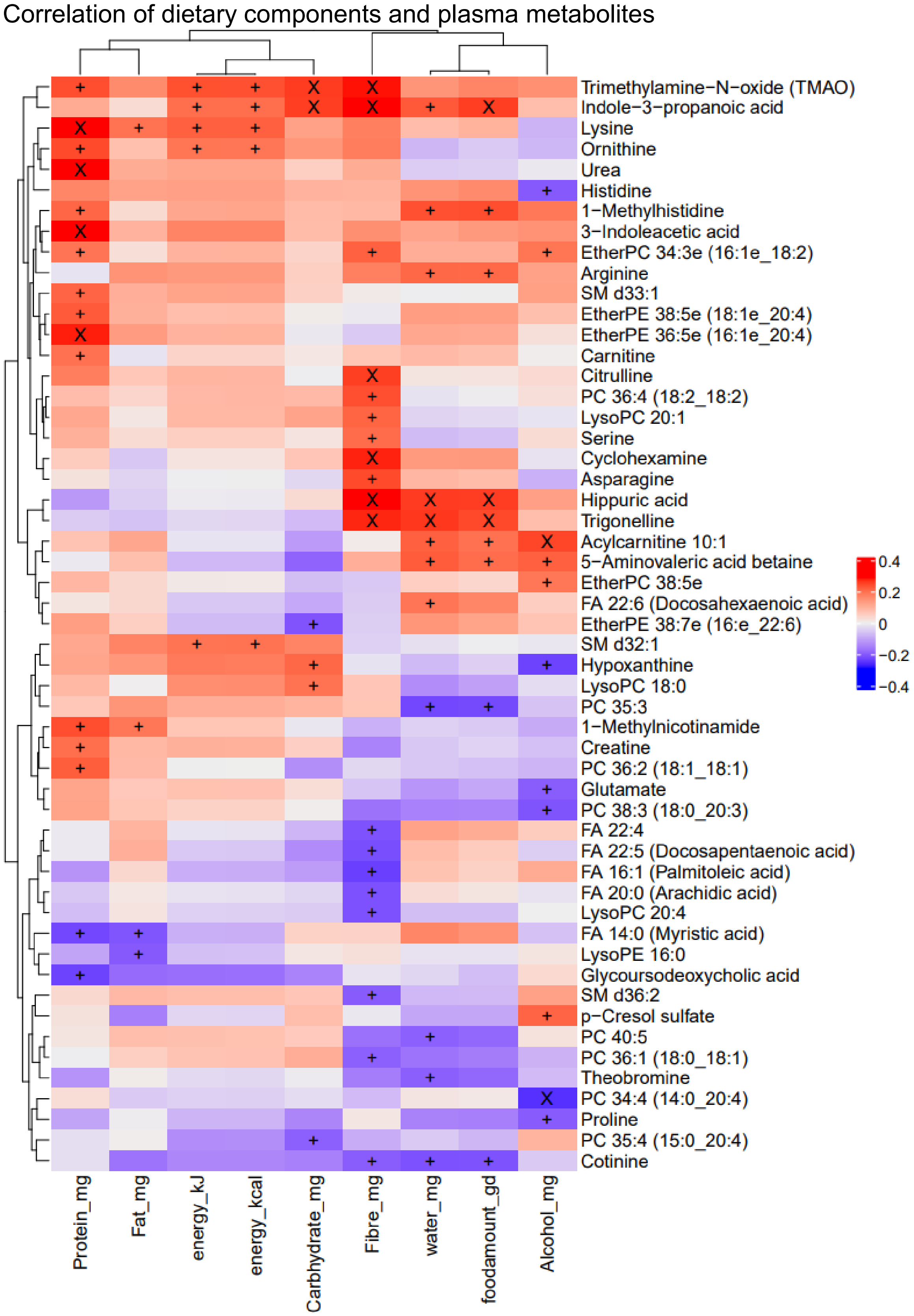

Supplemental Figure 4: Heatmap of dietary items and plasma metabolites
All identified metabolites with p-value below 0.05 in at least one correlation are shown. Euclidean clusterins was done to cluster metabolites and variables. Red is positive correlation, blue is negative correlation. + means p-value < 0.05 and X means FDR corrected q-value < 0.05.
