# Supplementary material for: Metabolic pattern across energy imbalance: An exploratory metabolomics study of female body weight extremes including anorexia nervosa and athletes
Source: Exp Physiol. 2026 Jul 14:10.1113/EP092957. Online ahead of print. doi: 10.1113/EP092957 (PMC13394067; doi:10.1113/EP092957)
Supplement: Supplementary file 2 — Table S1. Dietary intake data. [file EPH-9999-0-s001.pdf]

Supplemental Table 1 **Dietary Intake Data**

	Anorexia nervosa		Normal weight		Overweight		Obese		Athletes		p-value
n=	18		27		22		20		20		
	Md	IQR	Md	IQR	Md	IQR	Md	IQR	Md	IQR	
Nutritive Assessment – Macronutrient composition											
Energy (kcal)	1919	1472	1894	896	1749	328	2065	616	2011	585	0.187
Carbohydrates (g)	233	215	201	107	189	61	210	71	233	104	0.240
Fat (g)	79	37	80	49	67	21	79	33	76	35	0.460
Saturated fat (g)	25	18	31	21	30	12	37	9	31	16	0.033*
Protein (g)	79	52	62	33	61	20	74	32	74	42	0.072
Fiber (g)	19	13	21	8	17	7	17	7	24	15	0.050*
Alcohol (g)	0.1	0.6	0.5	9,2	0.2	7,5	0	0.1	0.1	0.7	0.009**
Amino Acid Composition											
Alanine [mg]	3335	1954	2809	1512	2817	1148	3632	1879	3380	1785	0.083
Arginine [mg]	3996	2202	3244	1390	3093	1338	3898	1954	3972	2926	0.171
Aspartic Acid [mg]	6911	4740	5338	3033	4771	2063	6461	2941	6502	4429	0.067
Cystine [mg]	1002	576	803	404	790	186	1045	411	935	589	0.040*
Glutamic Acid [mg]	15152	7159	13341	7420	13165	2740	16725	6611	15502	8030	0.182
Glycine [mg]	2795	1811	2388	1181	2293	979	3029	1590	2958	1795	0.117
Uric acid [mg]	316	264	311	141	304	112	301	200	389	258	0.195
Histidine [mg]	2085	1479	1584	993	1734	575	2188	824	2026	1484	0.048*
Isoleucine [mg]	3907	2584	3048	1783	2942	1082	3648	1448	3585	1709	0.047*
Leucine [mg]	6660	4341	5119	3040	4871	1436	5887	2640	6033	3066	0.034*
Lysine [mg]	5129	3853	3761	2708	3977	1380	4976	2328	4669	2960	0.069
Methionine [mg]	1690	1232	1337	873	1339	612	1646	800	1504	927	0.039*
Purine [mg]	106	88	103	52	102	37	100	65	130	85	0.219
Phenylalanine [mg]	3711	2211	2974	1595	2806	703	3386	1468	3404	1906	0.047*
Proline [mg]	6638	4215	4866	2431	4651	1925	5523	2224	5044	2982	0.068
Serine [mg]	4108	2297	3297	1902	3138	893	3794	1848	3830	1759	0.039*
Threonine [mg]	3158	2146	2464	1485	2426	1078	3007	1378	2986	1559	0.041*
Tryptophane [mg]	923	619	756	415	716	259	877	386	877	516	0.040*
Tyrosine [mg]	3023	2366	2291	1245	2268	697	2756	1262	2690	1447	0.036*
Valine [mg]	4552	2978	3557	2180	3379	1092	4062	1884	4160	1936	0.031*
Fatty Acid Composition											
Caprinc Acid C10:0 [mg]	717	635	959	845	1002	711	1080	365	1045	796	0.231
Lauric Acid C12:0 [mg]	1259	1412	2383	2094	1596	2268	2201	1634	1890	2053	0.165
Myristic Acid C14:0 [mg]	3214	2086	3290	3522	3721	2185	4237	1562	3808	2745	0.160
Tetradecenoic Acid C14:1 n5 [mg]	311	261	311	262	316	237	452	249	380	312	0.226
Pentadecylic Acid [mg]	285	215	271	244	296	200	391	197	326	304	0.201
Pentadecenoic Acid C15:0 [mg]	144	138	122	90	133	94	182	88	149	133	0.205
Palmitic Acid C16:0 [mg]	12001	8011	14390	9158	14122	4978	16398	6180	15359	8104	0.032*
Palmitoleic Acid C16:1 [mg]	895	623	1057	610	1160	371	1293	817	1126	785	0.038*
Margaric Acid C17:0 [mg]	222	146	225	165	221	85	302	117	259	198	0.074
Heptadecenoic Acid C17:1 [mg]	179	211	207	175	183	132	286	131	223	218	0.236
Stearic Acid C18:0 [mg]	4677	3150	6645	3482	5782	3313	7635	3148	5841	3878	0.011*
Oleic Acid C18:1 n9 [mg]	25360	22912	23295	15671	22029	8853	26881	15436	23069	9799	0.363
Linolic Acid C18:2 n6 [mg]	10044	7917	13756	5999	8809	6995	9847	11073	14453	14845	0.158
Linolenic acid C18:3 n3 [mg]	1936	1384	1224	1250	1263	715	1402	1073	1162	1982	0.731
Arachindic Acid C20:0 [mg]	251	226	302	126	263	82	276	171	342	205	0.187
Eicosenic Acid C20:1 [mg]	424	937	232	154	287	103	305	212	230	187	0.123
Eicosadienoic Acid C20:2 [mg]	8	5	8	14	17	26	19	22	8	24	0.003*
Eicosatrienoic Acid C20:3 [mg]	2	12	4	8	13	28	8	17	9	18	0.002*
Arachidonic Acid C20:4 n6 [mg]	80	92	55	64	89	91	107	88	100	88	0.141
icosapentaenoic Acid (EPA) C20:5 n3 [mg]	4	25	10	16	23	70	19	23	10	44	0.124
Behenic Acid C22:0 [mg]	86	152	70	54	69	54	68	70	64	95	0.621
Erucic Acid C22:1 n9 [mg]	62	73	47	62	67	87	57	89	36	85	0.704
ocosapentaenoic Acid (DPA) C22:5 n3 [mg]	10	28	7	17	21	47	12	23	18	26	0.282
ocosahexaenoic Acid (DHA) C22:6 n3 [mg]	101	79	43	57	99	164	73	102	83	108	0.287
Lignoceric Acid C24:0 [mg]	33	114	27	22	21	13	24	22	19	25	0.161
Selacholeic Acid C24:1 n9 [mg]	14	20	3	6	5	10	4	11	4	19	0.167
Butyrate C3:0 [mg]	847	740	806	789	917	736	1187	648	1107	1091	0.247
Capronic Acid C6:0 [mg]	501	440	507	394	551	452	724	324	712	696	0.250
Caprylic Acid C8:0 [mg]	423	428	612	513	509	599	661	411	659	503	0.191
Cholesterol [mg]	235	223	205	214	244	148	354	303	240	266	0.037*
Micronutrients											
Minerals											
Calcium [mg]	1162	752	787	429	868	371	978	325	936	595	0.280
Chlorine [mg]	4075	2125	4263	1750	4591	2451	5151	1752	4262	3013	0.390
Copper [µg]	2703	1532	1803	644	1628	717	1503	669	1588	1789	0.007**
Fluorine [µg]	1267	1130	1043	870	897	942	929	454	1192	744	0.178
Iron [µg]	16962	15370	11273	3456	10459	4345	9709	7185	11726	8420	0.045*
Iodine [µg]	126	182	94	73	112	70	93	45	97	98	0.384
Potassium [mg]	3088	1594	2583	1024	2447	704	2548	1251	2574	1590	0.394
Magnesium [mg]	373	232	319	139	303	109	304	144	323	255	0.319
Manganese [µg]	5268	4241	4202	1471	3524	1951	3572	2307	5265	4140	0.048*
Sodium [mg]	2308	1175	2413	1226	2728	1226	3000	1146	2553	995	0.159
Phosphor [mg]	1267	650	1124	510	1034	337	1227	614	1318	645	0.179
Sulfur [mg]	732	326	677	343	676	218	829	375	825	402	0.081
Zinc [µg]	10741	8050	8968	4303	7580	1402	9703	5121	10194	5028	0.059
Vitamins											
Vitamin A Retinol-equivalent [µg]	1290	1152	1028	794	882	948	1114	786	1242	859	0.281
Vitamin B1 [µg]	1604	1194	1000	445	1067	372	1007	544	1151	898	0.037*
Vitamin B12 [µg]	4,3	2,7	3,0	2,5	3,0	1,6	4,3	2,6	3,6	1,9	0.010*
Vitamin B2 [µg]	2125	1461	1319	834	1181	386	1469	1006	1422	834	0.003**
Niacinequivalents [µg]	35824	28603	23282	11138	23511	10304	31076	12819	27046	21496	0.086
Pantothenic acid B5 [µg]	6476	6205	3958	2572	3776	1709	4364	2956	4737	2919	0.005*
Vitamin B6 [µg]	1980	1917	1186	661	1193	417	1342	744	1479	1291	0.024*
Biotine B7 [µg]	69	30	51	24	44	16	44	30	49	45	0.006**
Folic acid B9 [µg]	411	259	245	143	248	98	288	248	298	197	0.004**
Vitamin C [µg]	125719	134735	80460	74610	67963	42155	87166	150864	133223	115027	0.013*
Vitamin D [µg]	5,1	11,4	1,8	1,2	1,8	2,2	2,5	2,7	2,3	2,9	0.026*
Vitamin E Tocopherol-equivalents [µg]	13060	19106	13524	5449	10461	6516	12172	10091	14033	11852	0.323
Vitamin K [µg]	137	112	101	77	74	58	66	98	101	136	0.134
