# Supplementary material for: Metabolic pattern across energy imbalance: An exploratory metabolomics study of female body weight extremes including anorexia nervosa and athletes
Source: Exp Physiol. 2026 Jul 14:10.1113/EP092957. Online ahead of print. doi: 10.1113/EP092957 (PMC13394067; doi:10.1113/EP092957)
Supplement: Supplementary file 3 — Table S2. Metabolomics analysis results. [file EPH-9999-0-s003.pdf]

Supplementary table 2: Metabolomics analysis results

								ANOVA	Anorexia vs control		overweight vs control		obese vs control		athletes vs control	
Compound	Mass	Retention Time	Ionization mode	column	Compound_ID	MSMS_spectra	ID_level	P-FDR	P	Estimate	P	Estimate	P	Estimate	P	Estimate
54.0101@13.24397	54.0101	13.24	POS	RP				5.96E-01	9.79E-01	-0.01	1.95E-01	0.37	4.32E-01	-0.19	5.89E-01	-0.18
54.0102@0.39798874	54.0102	0.40	POS	hilic				6.39E-01	4.66E-01	0.19	2.40E-01	0.33	4.50E-01	-0.21	5.99E-01	0.15
56.0621@9.190699	56.0621	9.19	POS	RP				9.15E-01	5.82E-01	-0.16	4.88E-01	0.19	7.06E-01	0.13	9.89E-01	0.00
56.0623@10.908552	56.0623	10.91	POS	RP				7.08E-01	9.07E-01	0.03	4.15E-01	-0.25	6.72E-01	0.14	2.99E-01	0.29
57.0575@5.281956	57.0575	5.28	POS	hilic				1.33E-01	1.23E-01	-0.28	9.86E-02	0.44	1.41E-01	0.35	8.49E-01	0.04
58.0528@1.2487531	58.0528	1.25	POS	hilic				2.91E-01	3.25E-02	-0.52	4.45E-01	0.21	7.47E-01	0.08	4.98E-01	-0.19
58.9829@1.5472499	58.9829	1.55	NEG	hilic				8.87E-01	2.89E-01	0.28	5.23E-01	0.16	7.19E-01	0.09	9.38E-01	-0.02
58.983@0.5368571	58.9830	0.54	NEG	hilic				9.34E-01	7.98E-01	0.08	4.51E-01	0.19	5.32E-01	0.18	8.53E-01	-0.05
59.0733@1.4420639	59.0733	1.44	POS	hilic				2.71E-01	1.90E-02	-0.72	5.95E-02	-0.48	1.60E-02	-0.66	2.14E-01	-0.42
59.0734@10.909165	59.0734	10.91	POS	RP				6.56E-01	1.06E-01	0.41	9.01E-01	0.04	5.96E-01	-0.17	4.08E-01	0.22
59.0736@15.303731	59.0736	15.30	POS	RP				2.23E-01	2.34E-02	0.74	4.19E-02	0.58	3.02E-01	0.31	1.90E-02	0.66
60.0212@5.2141185	60.0212	5.21	NEG	hilic				5.47E-02	3.32E-01	-0.30	2.86E-03	0.71	6.78E-01	0.11	5.06E-01	-0.19
60.032@0.9704358	60.0320	0.97	POS	hilic	Urea	10 eV: 44.0122 (100), 61.0391 (76)	2	5.66E-02	4.34E-03	1.02	1.82E-02	0.62	4.51E-02	0.55	6.64E-03	0.75
63.0085@4.334565	63.0085	4.33	POS	hilic				3.45E-01	1.57E-02	0.69	1.00E-01	0.55	1.07E-01	0.46	4.06E-02	0.58
66.0461@0.42174998	66.0461	0.42	POS	hilic				6.08E-01	8.84E-02	-0.24	9.15E-01	0.02	3.10E-01	-0.16	7.11E-01	0.08
68.0622@0.47438616	68.0622	0.47	POS	hilic				9.79E-01	7.81E-01	0.08	8.32E-01	-0.06	6.38E-01	0.16	7.87E-01	0.08
68.0624@8.337067	68.0624	8.34	POS	RP				9.74E-01	8.78E-01	-0.04	5.82E-01	0.13	7.07E-01	-0.11	8.96E-01	-0.04
68.0625@9.648219	68.0625	9.65	POS	RP				4.41E-01	8.15E-01	0.07	9.80E-01	0.01	6.58E-02	0.61	9.73E-01	0.01
68.0626@4.5942817	68.0626	4.59	POS	RP				9.19E-01	9.67E-01	0.01	2.83E-01	0.27	8.81E-01	-0.05	7.81E-01	0.07
69.0575@4.778957	69.0575	4.78	POS	hilic				3.99E-01	6.72E-01	-0.14	7.42E-01	0.09	1.36E-01	0.39	1.18E-01	0.54
70.003@15.362604	70.0030	15.36	NEG	RP				5.27E-01	8.23E-02	-0.50	4.19E-01	-0.25	6.82E-01	-0.13	6.02E-02	-0.56
70.0781@10.908804	70.0781	10.91	POS	RP				6.71E-01	5.46E-01	0.16	7.68E-01	0.09	5.98E-01	0.18	6.61E-02	0.54
71.0735@4.7646327	71.0735	4.76	POS	hilic	fragment of valine			4.53E-04	1.72E-01	-0.36	4.75E-04	0.87	5.55E-04	0.98	8.00E-02	0.50
72.0208@5.2312226	72.0208	5.23	NEG	hilic				5.35E-01	2.43E-01	-0.25	2.02E-01	0.34	1.00E+00	0.00	4.81E-01	0.19
72.0209@5.2173905	72.0209	5.22	NEG	hilic				3.94E-01	3.19E-01	-0.24	1.36E-01	0.42	1.33E-01	0.38	7.20E-01	0.11
75.0683@1.4923913	75.0683	1.49	POS	hilic				7.84E-01	1.55E-01	0.41	8.87E-01	0.04	8.40E-01	-0.06	9.39E-01	0.02
76.031@1.8775036	76.0310	1.88	POS	RP				2.31E-01	4.16E-01	-0.25	2.17E-02	0.58	1.37E-01	0.36	4.31E-01	0.24
76.031@3.1452339	76.0310	3.15	POS	RP				7.09E-01	3.52E-01	-0.26	4.46E-01	-0.21	1.62E-01	-0.39	8.45E-01	0.06
76.0313@3.8292542	76.0313	3.83	POS	hilic				1.17E-01	9.49E-01	0.02	5.82E-03	0.74	1.77E-02	0.65	1.64E-01	0.39
80.0623@0.44752082	80.0623	0.45	POS	hilic				8.86E-01	7.66E-01	-0.07	4.00E-01	0.21	8.24E-01	-0.05	7.78E-01	0.07
80.0624@10.45901	80.0624	10.46	POS	RP				6.97E-01	1.49E-01	0.50	9.83E-01	-0.01	8.49E-01	0.05	8.44E-01	0.06
82.0778@10.61733	82.0778	10.62	POS	RP				5.93E-01	3.24E-02	-0.54	2.93E-01	-0.28	1.90E-01	-0.37	4.46E-01	-0.22
82.078@8.538976	82.0780	8.54	POS	RP				1.70E-01	5.33E-02	-0.31	9.47E-01	-0.01	1.17E-01	0.44	9.67E-01	-0.01
82.0782@8.626396	82.0782	8.63	POS	RP				2.90E-01	1.58E-02	-0.44	7.11E-01	-0.08	4.12E-01	0.22	4.61E-01	-0.17
82.0787@0.42858064	82.0787	0.43	POS	hilic				8.41E-01	2.03E-01	-0.32	5.48E-01	-0.16	5.38E-01	-0.16	8.97E-01	0.03
83.037@6.0245767	83.0370	6.02	POS	hilic				8.04E-01	4.32E-01	0.27	9.53E-01	-0.02	3.79E-01	-0.24	9.30E-01	-0.03
83.0729@3.8106663	83.0729	3.81	POS	hilic				1.74E-01	7.71E-01	-0.08	1.16E-01	-0.35	3.43E-03	-0.57	8.21E-01	0.06
83.9524@15.303496	83.9524	15.30	POS	RP				6.31E-01	5.27E-01	-0.17	7.26E-01	-0.10	2.70E-01	-0.39	4.61E-02	-0.51
84.0208@2.9006925	84.0208	2.90	POS	hilic				6.50E-03	3.00E-02	-0.75	3.01E-02	0.58	6.33E-01	0.12	2.98E-01	-0.27
85.0161@5.2360077	85.0161	5.24	NEG	hilic				4.29E-01	1.73E-01	-0.39	3.21E-01	0.29	4.09E-01	0.22	4.64E-01	0.21
85.0526@0.87146103	85.0526	0.87	POS	hilic				7.07E-02	2.54E-01	-0.37	2.62E-01	-0.27	1.43E-03	-0.80	5.82E-01	0.17
85.0527@0.87010586	85.0527	0.87	POS	hilic				1.74E-01	8.12E-01	-0.07	2.76E-01	-0.28	5.23E-03	-0.73	7.36E-01	0.11
85.0641@1.2518727	85.0641	1.25	POS	hilic				2.91E-01	6.62E-01	-0.13	1.81E-01	0.38	6.82E-01	-0.12	1.60E-01	-0.43
85.0891@1.3028976	85.0891	1.30	POS	RP	fragment of leucine/norleucine			6.68E-04	3.74E-03	-0.68	2.87E-03	0.67	2.78E-02	0.71	2.18E-01	0.35
85.0891@1.3054274	85.0891	1.31	POS	RP	fragment of leucine/norleucine			1.62E-04	1.23E-03	-0.85	3.07E-03	0.63	2.77E-02	0.67	4.22E-01	0.21
85.0891@3.9141726	85.0891	3.91	POS	hilic				1.20E-05	1.15E-03	-0.87	5.01E-03	0.63	3.33E-03	0.84	7.16E-02	0.43
85.0891@4.161393	85.0891	4.16	POS	hilic				4.10E-05	3.65E-04	-0.94	5.70E-03	0.56	2.10E-02	0.65	8.81E-02	0.46
87.998@13.245579	87.9980	13.25	POS	RP				1.61E-01	7.99E-01	-0.07	2.70E-01	0.31	2.48E-02	-0.63	5.78E-01	-0.17
88.0159@1.8612394	88.0159	1.86	NEG	hilic				3.13E-03	3.13E-01	-0.33	3.12E-02	0.65	3.79E-03	0.85	6.16E-01	-0.13
88.0159@8.349359	88.0159	8.35	POS	RP				9.39E-01	9.10E-01	0.03	6.80E-01	0.12	7.17E-01	-0.12	5.61E-01	-0.17
89.0475@5.542285	89.0475	5.54	POS	hilic				6.33E-01	7.02E-01	-0.11	9.19E-01	-0.03	9.73E-02	0.46	8.06E-01	0.08
89.9695@4.34817	89.9695	4.35	POS	hilic				6.21E-01	9.90E-02	0.44	1.55E-01	0.44	9.09E-01	0.03	7.68E-01	0.09
90.0312@5.4136534	90.0312	5.41	NEG	hilic				6.26E-01	1.65E-01	-0.37	8.92E-01	0.04	6.92E-01	0.11	2.22E-01	-0.33
90.0314@1.4097521	90.0314	1.41	NEG	hilic				3.96E-02	8.64E-02	0.43	4.69E-02	0.57	4.36E-04	1.07	3.76E-02	0.68
90.0314@2.4673927	90.0314	2.47	NEG	hilic				4.61E-01	8.72E-01	-0.04	4.15E-01	0.19	1.59E-01	0.38	7.96E-02	0.45
90.0315@1.1170832	90.0315	1.12	NEG	RP				1.23E-02	5.10E-03	-0.80	1.96E-01	0.36	2.47E-01	0.30	4.85E-01	-0.20
90.0315@1.5247691	90.0315	1.52	NEG	hilic				1.05E-02	2.79E-01	0.29	1.85E-01	0.35	6.86E-05	1.22	1.22E-01	0.48
90.0315@5.209071:1	90.0315	5.21	NEG	hilic				2.32E-01	8.63E-02	-0.47	1.01E-01	0.44	6.86E-01	0.11	8.45E-01	0.06
90.0315@5.209071:2	90.0315	5.21	NEG	hilic				2.31E-01	7.54E-02	-0.48	1.14E-01	0.43	7.48E-01	0.09	8.96E-01	0.04
90.0315@5.4121437	90.0315	5.41	NEG	hilic				4.58E-01	1.72E-01	-0.31	5.54E-01	0.16	8.29E-01	-0.06	7.40E-02	-0.41
91.0348@5.248118	91.0348	5.25	NEG	hilic				9.55E-01	6.28E-01	-0.11	5.73E-01	0.16	8.90E-01	0.04	8.08E-01	0.06
92.006@15.411795	92.0060	15.41	POS	RP				5.08E-01	3.59E-02	-0.60	7.74E-01	-0.08	4.24E-01	-0.25	2.41E-01	-0.31
92.0622@0.4237083	92.0622	0.42	POS	hilic				7.07E-01	5.07E-02	-0.29	8.88E-01	-0.03	7.79E-01	-0.05	7.89E-01	0.06
93.0573@1.226375	93.0573	1.23	POS	hilic				6.75E-02	4.41E-02	0.57	2.97E-01	0.25	6.90E-01	0.10	6.31E-02	-0.33
94.0779@10.6544895	94.0779	10.65	POS	RP				8.97E-01	2.47E-01	-0.31	4.67E-01	-0.22	9.03E-01	-0.03	5.83E-01	-0.17
94.078@10.607089	94.0780	10.61	POS	RP				7.81E-01	1.17E-01	-0.41	4.53E-01	-0.21	1.91E-01	-0.35	3.72E-01	-0.28
94.078@9.910607	94.0780	9.91	POS	RP				8.71E-01	8.39E-01	0.04	9.86E-01	0.00	3.42E-01	0.20	8.50E-01	-0.03
94.0781@0.42406762	94.0781	0.42	POS	hilic				8.34E-01	1.40E-01	-0.39	7.05E-01	-0.11	6.54E-01	-0.12	5.60E-01	-0.16
94.0781@8.561951	94.0781	8.56	POS	RP				7.31E-01	7.67E-01	-0.06	7.55E-01	-0.07	2.56E-01	0.34	9.31E-01	0.02
94.0781@8.626288	94.0781	8.63	POS	RP				5.54E-01	1.82E-01	-0.31	3.99E-01	-0.21	4.35E-01	0.24	9.14E-01	0.03
94.0782@10.459575	94.0782	10.46	POS	RP				8.02E-01	2.70E-01	0.44	4.05E-01	0.23	8.41E-01	0.05	4.88E-01	0.21
95.037@0.8210751	95.0370	0.82	POS	hilic				4.76E-02	4.61E-02	0.55	6.49E-01	-0.12	8.05E-01	-0.07	2.32E-02	-0.52
95.0733@0.5408422	95.0733	0.54	POS	hilic				7.98E-01	3.78E-01	-0.20	8.24E-01	0.06	8.02E-01	0.07	3.63E-01	0.22

Supplementary table 2: Metabolomics analysis results

Compound	Mass	Retention Time	Ionization mode	column	Compound_ID	MSMS_spectra	ID_level	ANOVA	Anorexia vs control		overweight vs control		obese vs control		athletes vs control	
								P-FDR	P	Estimate	P	Estimate	P	Estimate	P	Estimate
96.0934@0.42239133	96.0934	0.42	POS	hilic				3.64E-01	9.86E-01	0.00	2.33E-01	0.23	5.21E-01	0.09	4.20E-02	0.40
96.0935@10.615963	96.0935	10.62	POS	RP				7.46E-01	4.35E-01	-0.19	9.47E-01	0.02	2.26E-01	-0.30	7.54E-01	0.09
96.0944@10.638777	96.0944	10.64	POS	RP				2.63E-01	1.87E-01	-0.37	3.18E-01	0.27	1.16E-01	-0.40	6.59E-01	0.13
96.9623@15.327222	96.9623	15.33	POS	RP				4.86E-01	4.17E-01	0.25	9.46E-02	0.52	4.70E-01	0.19	3.55E-02	0.57
97.0888@4.035088	97.0888	4.04	POS	hilic				9.72E-01	9.73E-01	-0.01	6.52E-01	0.12	7.21E-01	-0.08	8.98E-01	-0.03
97.9678@7.729391	97.9678	7.73	NEG	hilic				3.36E-01	4.33E-02	0.72	6.61E-02	0.52	1.91E-01	0.33	6.70E-02	0.52
98.0367@5.3718557	98.0367	5.37	POS	RP				9.51E-01	8.94E-01	-0.04	5.65E-01	-0.16	8.53E-01	0.05	6.75E-01	0.14
98.0368@0.42760652	98.0368	0.43	POS	hilic				3.97E-02	6.03E-03	0.86	6.85E-01	0.12	7.24E-01	0.10	2.50E-01	-0.32
98.0369@0.42760655	98.0369	0.43	POS	hilic				5.12E-02	6.13E-03	0.82	7.60E-01	0.09	8.13E-01	0.07	2.28E-01	-0.33
98.0369@5.956538	98.0369	5.96	POS	RP				5.99E-02	7.71E-04	1.05	3.78E-02	0.53	4.10E-02	0.58	3.09E-01	0.33
98.0369@6.833608	98.0369	6.83	POS	RP				1.49E-01	2.22E-02	0.76	1.25E-02	0.65	1.65E-01	0.39	8.27E-01	0.06
99.043@1.7589222	99.0430	1.76	POS	hilic				2.82E-01	4.21E-01	-0.28	7.72E-02	0.55	4.30E-01	0.21	4.35E-01	0.23
99.0679@0.5986434	99.0679	0.60	POS	hilic				7.75E-01	8.81E-01	0.05	8.30E-01	-0.05	2.45E-01	0.39	8.93E-01	-0.04
99.0682@1.0626372	99.0682	1.06	POS	hilic	Cyclohexamine	10 eV: 83.0843 (100), 100.0741 (90), 55.0548 (8)	2	4.73E-01	7.15E-01	-0.11	7.11E-01	-0.11	2.99E-02	-0.62	7.13E-01	-0.10
99.0682@1.9596666	99.0682	1.96	POS	RP				6.96E-01	4.57E-01	0.20	5.87E-01	-0.12	2.03E-01	0.29	4.78E-01	0.18
99.0683@1.6962239	99.0683	1.70	POS	RP				9.19E-01	6.06E-01	0.16	7.91E-01	0.07	2.89E-01	0.33	8.08E-01	0.08
99.1046@1.069037	99.1046	1.07	POS	hilic				4.43E-01	6.37E-01	-0.14	2.03E-01	0.35	5.70E-01	-0.15	3.04E-01	-0.27
100.0639@6.024513	100.0639	6.02	POS	hilic				4.10E-01	2.34E-01	-0.36	8.04E-01	0.06	2.91E-02	-0.54	3.24E-01	-0.28
100.0996@15.328711	100.0996	15.33	POS	RP				9.02E-01	8.00E-01	0.07	4.25E-01	-0.19	4.92E-01	-0.18	5.37E-01	-0.14
100.9562@15.25567	100.9562	15.26	POS	RP				8.30E-02	3.42E-01	-0.29	2.68E-02	0.62	6.63E-01	-0.13	3.63E-01	-0.27
101.0473@3.4913027	101.0473	3.49	POS	hilic				2.84E-02	7.78E-02	0.46	6.35E-01	0.08	8.77E-01	-0.02	4.56E-03	0.71
101.0478@6.0230007	101.0478	6.02	POS	hilic				5.98E-01	9.97E-02	0.52	7.12E-01	0.11	8.43E-01	-0.05	8.68E-01	-0.05
101.0838@1.3591145	101.0838	1.36	POS	hilic				9.78E-01	9.06E-01	-0.03	7.78E-01	-0.06	4.47E-01	-0.16	7.21E-01	-0.08
101.0839@0.79506874	101.0839	0.80	POS	hilic				9.79E-01	8.48E-01	0.05	7.55E-01	-0.09	6.58E-01	0.13	8.20E-01	0.08
101.0839@4.7493334	101.0839	4.75	POS	hilic				2.08E-01	3.77E-01	-0.28	1.91E-01	0.35	8.35E-02	0.50	5.15E-01	-0.16
101.1206@0.83960915	101.1206	0.84	POS	hilic				3.84E-01	9.61E-01	0.01	5.17E-01	-0.20	7.21E-02	0.54	8.12E-01	-0.07
101.9994@15.2546425	101.9994	15.25	POS	RP				4.20E-01	8.68E-01	0.03	2.64E-01	0.25	1.10E-01	0.37	4.57E-02	0.49
102.0314@5.2220716	102.0314	5.22	NEG	hilic				1.81E-01	1.42E-01	-0.37	6.10E-02	0.53	3.06E-01	0.29	7.04E-01	0.11
102.032@4.7502646	102.0320	4.75	POS	hilic				5.84E-01	2.60E-01	-0.39	4.53E-01	0.20	4.96E-01	0.21	8.27E-01	0.06
102.0467@1.1521207	102.0467	1.15	POS	RP				1.14E-01	4.23E-01	-0.26	3.19E-02	0.59	3.10E-02	0.56	2.23E-01	0.34
102.0468@3.8305035	102.0468	3.83	POS	hilic	Phenylacetaldehyde	10 eV: 77.0393 (100), 103.0550 (95)	2	3.49E-02	8.12E-01	-0.07	6.52E-04	0.87	1.70E-02	0.67	1.99E-01	0.37
102.0468@1.877448	102.0468	1.88	POS	RP				4.12E-02	2.21E-01	-0.39	1.49E-02	0.62	3.93E-02	0.52	1.54E-01	0.41
103.0632@5.283374	103.0632	5.28	POS	hilic				1.31E-04	1.75E-04	-1.05	1.71E-02	0.61	2.70E-01	0.29	9.91E-01	0.00
103.0635@4.626375	103.0635	4.63	POS	hilic	Dimethylglycine	10 eV: 58.0646 (100), 104.0688 (10)	2	4.02E-01	4.46E-02	-0.56	8.95E-01	0.04	6.59E-01	0.13	5.99E-01	0.14
103.0635@5.110222	103.0635	5.11	POS	hilic				1.96E-01	2.94E-01	-0.32	1.65E-01	0.36	1.42E-01	-0.48	4.26E-01	-0.24
103.0636@0.7205319	103.0636	0.72	POS	hilic				7.42E-01	4.62E-01	-0.18	5.76E-01	-0.14	1.19E-01	-0.37	9.93E-01	0.00
103.0743@2.5040388	103.0743	2.50	POS	hilic				6.72E-01	8.84E-01	-0.04	1.39E-01	0.37	4.35E-01	0.21	2.22E-01	0.33
103.0996@1.4754527	103.0996	1.48	POS	hilic	Choline cation	10 eV: 104.1071 (100), 60.0807 (25), 45.0330 (5)	2	6.67E-01	5.18E-01	-0.19	5.86E-02	-0.52	3.84E-01	-0.24	4.59E-01	-0.22
103.0997@10.169591	103.0997	10.17	POS	RP				7.71E-01	2.62E-01	0.34	6.94E-01	0.11	1.82E-01	0.33	1.95E-01	0.39
103.0997@10.529837	103.0997	10.53	POS	RP				1.21E-01	8.61E-03	0.70	4.93E-01	-0.22	8.56E-01	0.05	1.69E-01	0.42
103.0997@9.623161	103.0997	9.62	POS	RP				7.31E-01	1.36E-01	0.41	1.98E-01	0.26	2.06E-01	0.35	2.61E-01	0.29
103.0997@9.958594	103.0997	9.96	POS	RP				7.61E-01	3.58E-01	-0.31	4.37E-01	0.20	7.04E-01	0.10	9.43E-01	0.02
103.0998@10.195333	103.0998	10.20	POS	RP				2.59E-01	1.48E-01	0.48	1.89E-01	0.34	7.09E-01	-0.11	2.77E-02	0.61
104.0264@3.1456459	104.0264	3.15	POS	RP				4.67E-01	2.79E-01	-0.36	9.39E-01	-0.02	1.39E-01	-0.45	5.60E-01	0.17
104.0266@8.340258	104.0266	8.34	POS	RP				6.98E-01	2.81E-01	-0.25	8.64E-01	0.04	4.66E-01	0.20	4.84E-01	0.18
104.047@0.9882882	104.0470	0.99	NEG	hilic				3.98E-02	3.09E-01	-0.31	3.85E-02	0.60	3.58E-02	0.61	9.97E-01	0.00
104.9495@15.3319845	104.9495	15.33	POS	RP				9.28E-01	8.27E-01	-0.07	4.72E-01	0.20	8.62E-01	0.06	4.78E-01	0.21
105.0426@6.069145	105.0426	6.07	POS	hilic	Serine	20 eV: 42.0330 (100), 60.0438 (95)	2	8.84E-01	8.34E-01	0.07	8.91E-01	-0.04	6.11E-01	-0.15	3.50E-01	0.25
105.0788@1.8593242	105.0788	1.86	POS	hilic				5.91E-01	3.85E-01	0.31	8.27E-01	0.06	4.68E-02	0.50	1.54E-01	0.43
105.0788@3.407232	105.0788	3.41	POS	hilic				8.37E-05	2.76E-02	-0.64	3.15E-04	-0.84	2.18E-05	-0.98	2.93E-08	-1.24
106.0262@5.12798	106.0262	5.13	NEG	hilic				8.62E-01	6.12E-01	-0.15	9.88E-01	0.00	2.95E-01	-0.33	4.49E-01	-0.22
106.0413@1.8773245	106.0413	1.88	POS	RP				4.48E-02	6.01E-01	-0.16	6.17E-02	0.48	7.88E-02	0.50	2.59E-03	0.83
106.0416@3.8304102	106.0416	3.83	POS	hilic				1.18E-01	6.23E-01	-0.15	6.94E-02	0.51	4.63E-02	0.57	1.38E-02	0.62
106.0417@8.872395	106.0417	8.87	POS	RP				6.16E-01	5.12E-01	-0.19	5.97E-01	-0.12	3.01E-01	0.29	4.10E-01	0.22
106.042@9.534905	106.0420	9.53	POS	RP				7.29E-01	9.23E-01	-0.03	2.40E-01	-0.36	6.48E-01	-0.14	5.93E-01	0.16
106.0777@8.533233	106.0777	8.53	POS	RP				2.31E-01	3.47E-01	-0.15	5.17E-01	-0.11	1.52E-01	0.38	4.63E-01	-0.13
106.0778@8.625897	106.0778	8.63	POS	RP				2.45E-01	3.87E-02	-0.33	8.01E-01	-0.05	3.42E-01	0.25	1.88E-01	-0.24
108.0416@5.243594	108.0416	5.24	NEG	hilic				7.40E-02	7.26E-03	-0.65	6.09E-01	0.14	8.70E-01	-0.04	2.87E-02	-0.54
108.0935@10.655853	108.0935	10.66	POS	RP				8.66E-01	1.63E-01	-0.38	4.75E-01	-0.21	3.80E-01	-0.25	4.30E-01	-0.25
108.0936@10.61224	108.0936	10.61	POS	RP				7.05E-01	2.26E-01	-0.32	3.00E-01	-0.31	8.82E-02	-0.49	6.62E-01	-0.15
108.0937@0.44836518	108.0937	0.45	POS	hilic				9.51E-01	9.43E-01	-0.02	7.78E-01	0.08	4.99E-01	-0.21	9.08E-01	0.04
108.0937@8.62511	108.0937	8.63	POS	RP				3.60E-01	2.18E-01	-0.23	9.39E-01	-0.02	1.40E-01	0.41	8.65E-01	0.04
108.0938@5.069295	108.0938	5.07	POS	RP				8.01E-01	7.20E-01	0.11	1.43E-01	0.42	7.54E-01	0.11	3.18E-01	0.28
108.0938@5.2085123	108.0938	5.21	POS	RP				7.65E-01	2.66E-01	0.28	1.30E-01	0.39	9.24E-01	-0.03	7.29E-01	0.10
108.0938@8.542045	108.0938	8.54	POS	RP				3.33E-01	2.97E-01	-0.19	9.57E-01	0.01	1.05E-01	0.44	9.79E-01	0.01
108.0939@7.2457542	108.0939	7.25	POS	RP				8.13E-01	5.37E-01	0.17	4.17E-01	0.22	5.36E-01	-0.21	6.88E-01	0.13
108.094@9.909809	108.0940	9.91	POS	RP				9.89E-01	8.66E-01	-0.03	5.14E-01	-0.11	8.23E-01	-0.04	8.37E-01	-0.04
109.0012@7.315267	109.0012	7.32	POS	hilic				8.36E-01	2.36E-01	0.33	5.72E-01	0.13	9.70E-01	0.01	7.64E-01	0.07
109.0638@6.6858034	109.0638	6.69	POS	hilic				7.26E-01	8.29E-01	0.08	2.89E-01	0.30	3.88E-01	0.25	9.73E-02	0.48
110.1093@0.4796189	110.1093	0.48	POS	hilic				9.29E-01	8.25E-01	0.07	9.86E-01	0.00	3.62E-01	0.27	5.49E-01	0.17
110.1094@8.334665	110.1094	8.33	POS	RP				9.45E-01	3.14E-01	0.30	8.59E-01	0.05	7.10E-01	0.12	7.24E-01	0.11
110.1095@4.5944595	110.1095	4.59	POS	RP				9.64E-01	6.65E-01	0.12	4.58E-01	0.18	9.95E-01	0.00	4.66E-01	0.21
110.1095@9.6488905	110.1095	9.65	POS	RP				5.44E-01	4.81E-01	0.20	8.75E-01	0.04	9.95E-02	0.60	3.64E-01	0.22

Supplementary table 2: Metabolomics analysis results

Compound	Mass	Retention Time	Ionization mode	column	Compound_ID	MSMS_spectra	ID_level	ANOVA	Anorexia vs control		overweight vs control		obese vs control		athletes vs control	
								P-FDR	P	Estimate	P	Estimate	P	Estimate	P	Estimate
110.1096@5.9435534	110.1096	5.94	POS	RP				7.69E-01	1.42E-01	0.39	7.36E-01	0.09	9.09E-01	0.04	2.42E-01	0.31
110.1096@8.275993	110.1096	8.28	POS	RP				9.11E-01	6.40E-01	-0.13	5.17E-01	0.17	7.06E-01	-0.13	7.05E-01	0.10
110.9991@15.254765	110.9991	15.25	POS	RP				4.32E-01	4.56E-02	-0.67	1.60E-01	-0.46	2.44E-01	-0.32	7.75E-02	-0.52
111.0428@1.9253248	111.0428	1.93	POS	hilic				6.43E-01	7.16E-01	0.10	2.16E-01	-0.27	2.05E-01	-0.27	8.83E-01	-0.03
111.0429@1.5100774	111.0429	1.51	POS	hilic				4.54E-01	8.99E-01	-0.04	3.50E-01	-0.26	3.50E-02	-0.62	1.10E-01	-0.41
111.983@15.359863	111.9830	15.36	NEG	RP				9.02E-01	3.76E-01	0.27	2.58E-01	0.33	6.62E-01	0.13	5.11E-01	0.21
112.0633@6.268417	112.0633	6.27	POS	hilic				4.27E-01	4.11E-01	0.24	5.33E-01	0.15	2.06E-01	-0.27	2.24E-01	0.33
112.0904@0.43699995	112.0904	0.44	POS	hilic				6.56E-01	2.31E-02	-0.32	2.89E-01	-0.19	7.88E-01	-0.05	5.12E-01	-0.12
112.9572@15.41105	112.9572	15.41	POS	RP				6.28E-01	4.04E-01	0.24	1.66E-01	0.41	2.79E-01	0.34	6.94E-02	0.53
113.0296@10.276819	113.0296	10.28	POS	RP				3.65E-01	3.65E-01	-0.24	9.10E-02	0.48	4.53E-01	0.19	7.67E-01	0.08
113.059@5.4748683	113.0590	5.47	POS	hilic				3.86E-03	7.20E-01	0.10	3.32E-01	0.24	1.99E-03	0.74	2.39E-02	-0.63
113.0591@1.2515038	113.0591	1.25	POS	hilic	Creatinine	10 eV: 44.0491 (100), 114.0661 (95), 86.0705 (10)	2	6.50E-01	6.40E-01	-0.10	2.29E-01	0.31	2.14E-01	0.33	4.02E-01	0.24
113.0841@2.9336677	113.0841	2.93	POS	RP	epsilon-Caprolactam	10 eV: 114.0915 (100), 69.0692 (10), 96.0807 (5), 43.0540 (5)	2	8.68E-01	9.18E-01	0.03	3.47E-01	0.27	8.32E-01	-0.05	5.18E-01	0.20
113.0841@0.53946155	113.0841	0.54	POS	hilic				7.63E-01	7.29E-01	-0.10	3.39E-01	0.27	5.65E-01	-0.15	5.48E-01	0.18
113.1205@3.8748043	113.1205	3.87	POS	RP				1.53E-01	9.97E-02	-0.44	8.77E-01	0.04	3.07E-01	-0.32	9.00E-02	0.44
113.9929@0.4510273	113.9929	0.45	NEG	hilic				4.97E-01	1.16E-01	-0.52	9.72E-01	0.01	5.42E-01	0.18	7.44E-01	-0.09
113.9929@15.360983	113.9929	15.36	NEG	RP				6.64E-01	4.87E-02	-0.57	5.80E-01	-0.15	6.50E-01	-0.13	4.30E-01	-0.24
114.0136@13.243971	114.0136	13.24	POS	RP				2.89E-01	9.22E-01	0.03	1.52E-01	0.38	1.21E-01	-0.38	3.81E-01	-0.29
114.031@5.2374144	114.0310	5.24	NEG	hilic				9.20E-01	3.63E-01	-0.22	7.17E-01	0.10	8.13E-01	-0.06	9.88E-01	0.00
114.0475@3.9578252	114.0475	3.96	POS	hilic				5.03E-01	5.33E-02	-0.51	6.81E-01	0.12	9.65E-01	-0.01	7.57E-01	-0.08
114.068@2.2173367	114.0680	2.22	POS	RP				6.15E-01	3.98E-01	0.23	8.42E-02	0.50	9.02E-01	0.04	8.81E-01	0.04
114.0789@3.9838793	114.0789	3.98	POS	hilic				5.47E-02	1.27E-02	0.86	7.11E-01	-0.08	2.46E-01	0.33	3.91E-02	0.51
114.1037@7.159485	114.1037	7.16	POS	RP				8.62E-01	6.53E-01	0.11	8.09E-01	-0.05	4.49E-01	0.20	3.89E-01	0.25
115.0093@10.32954	115.0093	10.33	POS	RP				9.66E-01	9.00E-01	0.03	4.05E-01	0.20	9.62E-01	0.01	6.68E-01	0.12
115.0631@6.2775245	115.0631	6.28	POS	hilic				7.12E-02	2.23E-01	0.32	4.03E-01	0.22	8.13E-02	0.39	1.45E-03	0.93
115.0633@4.778641	115.0633	4.78	POS	hilic	Proline	10 eV: 70.0655 (100), 116.0708 (30)	2	9.69E-01	3.44E-01	0.22	5.51E-01	0.18	4.70E-01	0.20	7.80E-01	0.09
115.0634@0.8341875	115.0634	0.83	POS	hilic				2.63E-01	3.29E-01	0.27	2.52E-01	0.30	2.86E-02	0.48	7.80E-03	0.68
115.0634@7.079857	115.0634	7.08	POS	hilic				5.87E-03	1.96E-03	0.95	6.78E-01	-0.10	1.76E-01	0.38	2.52E-03	0.87
115.0635@6.869046	115.0635	6.87	POS	hilic				7.63E-02	7.06E-05	-0.84	2.21E-01	-0.29	1.15E-01	-0.39	2.92E-01	-0.29
115.0997@0.8798664	115.0997	0.88	POS	hilic				7.66E-03	5.33E-02	-0.58	1.31E-01	0.35	1.73E-02	0.71	5.39E-01	-0.13
115.978@15.30112	115.9780	15.30	POS	RP				8.53E-01	5.49E-01	-0.17	5.45E-01	-0.20	2.61E-01	-0.35	2.44E-01	-0.35
115.996@15.250398	115.9960	15.25	NEG	RP				4.90E-01	5.92E-01	-0.16	1.62E-01	-0.36	5.00E-02	-0.52	7.25E-01	-0.09
116.0278@1.4153885	116.0278	1.42	NEG	hilic				1.68E-01	3.79E-01	0.26	1.80E-01	0.36	2.32E-03	0.90	1.85E-01	0.39
116.0471@8.952731	116.0471	8.95	POS	RP				7.44E-01	3.31E-01	-0.31	4.53E-01	0.19	5.62E-01	-0.19	9.19E-01	-0.03
116.0474@1.0678977	116.0474	1.07	NEG	hilic				8.08E-04	5.84E-01	-0.15	1.26E-03	0.89	9.18E-04	0.99	9.14E-01	-0.03
116.0474@1.8097012	116.0474	1.81	NEG	RP				2.63E-02	5.99E-01	-0.14	9.86E-03	0.70	2.91E-02	0.67	6.27E-01	-0.14
117.0539@5.611424	117.0539	5.61	POS	hilic				4.64E-01	8.98E-01	-0.03	2.35E-01	-0.39	4.91E-02	-0.59	1.77E-01	-0.38
117.0579@3.9577475	117.0579	3.96	POS	hilic				7.34E-01	2.27E-01	-0.35	7.72E-01	-0.08	4.52E-01	0.21	9.71E-01	0.01
117.0787@3.677879	117.0787	3.68	POS	hilic	Glycine betaine	10 eV: 118.0859 (100), 59.0727 (15)	2	9.22E-01	3.76E-01	-0.26	9.14E-01	-0.03	5.68E-01	-0.18	4.16E-01	-0.22
117.079@4.7647963	117.0790	4.76	POS	hilic	Valine	10 eV: 72.0805 (100), 55.0536 (24), 118.0855 (2)	2	4.11E-03	2.58E-01	-0.28	4.38E-03	0.69	1.25E-03	0.89	4.47E-02	0.63
117.0802@15.366269	117.0802	15.37	POS	RP				5.70E-01	4.36E-01	-0.19	5.82E-01	0.14	9.47E-01	0.02	1.52E-01	0.40
117.9357@10.755115	117.9357	10.76	NEG	RP				9.01E-01	2.88E-01	0.35	6.24E-01	0.14	9.55E-01	0.02	6.51E-01	0.15
117.9358@10.7058325	117.9358	10.71	NEG	RP				1.46E-01	2.22E-01	-0.38	1.56E-01	0.40	4.35E-02	0.53	8.86E-01	0.05
117.9359@11.034285	117.9359	11.03	NEG	RP				5.07E-01	8.78E-01	-0.04	3.38E-01	-0.29	1.26E-01	0.39	7.09E-01	0.12
118.0085@4.839818	118.0085	4.84	POS	hilic				2.96E-01	7.92E-02	-0.36	4.37E-01	-0.17	2.99E-01	-0.22	3.18E-01	0.25
118.0263@1.1837574	118.0263	1.18	NEG	hilic				5.04E-01	3.92E-01	-0.26	8.25E-01	0.07	1.35E-01	-0.46	6.10E-01	0.13
118.063@0.7588034	118.0630	0.76	NEG	hilic				6.73E-03	8.44E-03	-0.86	1.48E-01	0.39	7.93E-01	-0.06	2.45E-01	0.35
118.063@2.628428	118.0630	2.63	NEG	RP				3.19E-03	3.74E-03	-0.97	2.14E-01	0.33	8.48E-01	-0.05	2.48E-01	0.33
118.0632@2.628428	118.0632	2.63	NEG	RP				3.94E-03	3.99E-03	-0.95	2.48E-01	0.31	8.30E-01	-0.05	2.28E-01	0.35
119.0734@3.828991	119.0734	3.83	POS	hilic	4-aminomethylphenylmethanamine	10 eV: 120.0801 (100), 93.0691 (25), 103.0538 (15), 77.0377 (15)	2	1.53E-02	2.56E-01	-0.38	5.54E-03	0.70	6.51E-03	0.66	1.85E-01	0.36
119.0734@1.8760849	119.0734	1.88	POS	RP				3.03E-03	1.91E-01	-0.43	3.52E-03	0.76	3.41E-02	0.54	3.19E-03	0.72
119.0735@1.1519742	119.0735	1.15	POS	RP				9.74E-02	3.74E-01	-0.29	2.36E-02	0.63	5.14E-02	0.51	2.05E-01	0.33
120.0931@8.536384	120.0931	8.54	POS	RP				5.31E-01	1.92E-01	-0.20	7.95E-01	0.05	2.27E-01	0.30	9.44E-01	-0.01
120.0935@8.620334	120.0935	8.62	POS	RP				3.64E-01	1.12E-01	-0.24	7.34E-01	-0.06	1.96E-01	0.32	9.73E-01	0.01
121.9172@3.0699918	121.9172	3.07	POS	hilic				6.82E-01	9.60E-01	-0.02	9.77E-01	0.01	8.31E-02	0.46	4.09E-01	0.24
122.0037@9.757599	122.0037	9.76	NEG	RP				5.37E-01	4.34E-01	-0.25	2.67E-01	0.31	5.03E-01	-0.17	5.45E-01	0.18
122.0368@5.014296	122.0368	5.01	POS	hilic				1.68E-01	1.19E-01	-0.43	1.40E-01	0.40	2.28E-01	0.33	7.80E-01	-0.07
122.1088@10.586321	122.1088	10.59	POS	RP				7.44E-01	7.26E-01	-0.09	3.24E-01	-0.25	9.14E-02	-0.38	7.23E-01	-0.09
122.1091@0.42566666	122.1091	0.43	POS	hilic				8.46E-01	2.79E-01	-0.25	2.04E-01	-0.27	4.97E-01	-0.16	4.06E-01	-0.21
123.0795@2.8870518	123.0795	2.89	POS	RP				7.34E-01	1.98E-01	0.35	2.18E-01	0.37	1.18E-01	0.46	2.69E-01	0.33
123.0796@6.171	123.0796	6.17	POS	hilic				3.32E-02	4.92E-02	-0.60	2.42E-01	0.32	6.22E-01	0.12	7.15E-02	0.56
123.0798@2.8909662	123.0798	2.89	POS	RP				7.34E-01	5.03E-01	0.17	1.48E-01	0.45	1.93E-01	0.38	2.79E-01	0.30
123.9157@3.070491	123.9157	3.07	POS	hilic				8.58E-01	8.23E-01	0.06	8.91E-01	-0.03	4.84E-01	-0.19	3.08E-01	-0.25
123.9925@1.2835585	123.9925	1.28	POS	hilic				2.68E-01	6.54E-01	0.13	6.06E-01	-0.11	2.77E-02	-0.59	2.65E-01	-0.30
124.032@15.409439	124.0320	15.41	POS	RP				4.69E-01	2.00E-01	0.36	1.07E-01	0.49	1.39E-01	0.44	1.89E-02	0.60
124.9787@7.87247	124.9787	7.87	POS	hilic				8.72E-01	4.12E-01	0.28	3.96E-01	0.26	2.89E-01	0.30	2.46E-01	0.34
124.9795@15.30481	124.9795	15.30	POS	RP				5.89E-01	5.48E-01	-0.18	9.98E-01	0.00	1.63E-01	-0.47	1.36E-01	-0.43
125.0143@5.3760877	125.0143	5.38	NEG	hilic				7.61E-01	4.00E-01	0.31	3.71E-01	0.23	6.13E-02	0.49	2.96E-01	0.26
125.0146@5.2790084	125.0146	5.28	POS	hilic				7.30E-01	4.33E-01	0.32	5.77E-01	0.13	5.46E-02	0.50	3.06E-01	0.24
125.0841@0.6572281	125.0841	0.66	POS	hilic				7.14E-01	5.78E-01	0.16	8.73E-01	0.04	7.62E-01	0.07	1.96E-01	-0.30
125.9645@1.0501376	125.9645	1.05	POS	RP				8.96E-01	2.39E-01	-0.32	3.35E-01	-0.28	4.88E-01	-0.17	4.30E-01	-0.27
125.9982@3.6451204	125.9982	3.65	NEG	hilic				1.47E-01	7.36E-01	0.11	8.81E-01	0.03	8.77E-03	-0.69	1.36E-01	-0.45
126.0679@7.794576	126.0679	7.79	POS	RP				8.94E-01	9.32E-01	-0.03	3.24E-01	0.26	7.34E-01	0.11	3.54E-01	0.25

Supplementary table 2: Metabolomics analysis results

								ANOVA	Anorexia vs control		overweight vs control		obese vs control		athletes vs control	
Compound	Mass	Retention Time	Ionization mode	column	Compound_ID	MSMS_spectra	ID_level	P-FDR	P	Estimate	P	Estimate	P	Estimate	P	Estimate
126.1045@5.2092724	126.1045	5.21	POS	RP				9.60E-01	4.93E-01	0.18	6.27E-01	0.13	7.89E-01	-0.09	8.93E-01	0.04
126.1046@7.2459927	126.1046	7.25	POS	RP				7.50E-01	1.44E-01	0.44	2.55E-01	0.31	9.46E-01	0.02	5.58E-01	0.19
127.9435@1.0505097	127.9435	1.05	POS	RP				8.31E-01	1.56E-01	0.38	5.81E-01	0.16	9.38E-01	-0.02	8.48E-01	0.06
127.9437@15.431387	127.9437	15.43	POS	RP				1.16E-01	2.15E-01	0.34	2.75E-02	0.65	9.88E-01	0.00	1.38E-02	0.75
127.9795@15.4076395	127.9795	15.41	POS	RP				9.28E-01	5.08E-01	0.17	5.01E-01	0.17	9.40E-01	-0.02	4.38E-01	0.20
128.023@15.2647915	128.0230	15.26	POS	RP				4.02E-01	1.84E-01	-0.33	4.96E-01	0.20	1.37E-01	-0.40	3.81E-01	-0.21
128.0585@5.6001887	128.0585	5.60	POS	hilic				2.80E-02	1.14E-01	0.52	4.77E-01	0.19	2.02E-02	-0.66	4.00E-01	0.21
128.0586@6.0783277	128.0586	6.08	NEG	hilic				4.67E-01	7.94E-01	-0.08	4.81E-01	-0.19	2.02E-02	-0.62	7.28E-01	-0.09
128.095@0.83362955	128.0950	0.83	POS	hilic				5.87E-03	1.53E-01	0.42	3.68E-02	-0.38	3.33E-02	-0.39	2.60E-02	-0.40
128.12@8.273703	128.1200	8.27	POS	RP				9.76E-01	8.99E-01	0.03	9.20E-01	0.02	6.57E-01	-0.16	7.80E-01	0.08
128.1201@0.42636493	128.1201	0.43	POS	hilic				3.25E-04	9.39E-05	-1.08	1.67E-01	0.34	7.09E-01	-0.10	1.36E-01	0.44
129.0412@3.270612	129.0412	3.27	POS	RP				1.12E-01	1.38E-02	0.72	9.86E-01	0.00	7.13E-01	0.08	1.13E-01	0.44
129.0425@1.9136741	129.0425	1.91	POS	hilic				6.42E-02	2.07E-02	0.79	9.45E-01	0.02	9.11E-01	0.03	4.04E-02	0.56
129.0427@6.025734	129.0427	6.03	POS	hilic	Pyroglutamic acid	10 eV: 84.0444 (100), 130.0495 (20), 56.0477 (8)	2	7.89E-01	9.42E-01	0.02	8.06E-01	-0.08	8.71E-02	-0.42	7.58E-01	-0.09
129.0427@4.414352	129.0427	4.41	NEG	hilic				3.69E-01	2.93E-01	0.35	3.32E-01	-0.23	6.84E-02	-0.44	9.85E-01	0.01
129.0429@4.235587	129.0429	4.24	POS	hilic				3.02E-01	2.95E-01	0.33	3.37E-01	-0.23	2.01E-01	-0.31	3.78E-01	0.25
129.0538@2.0566142	129.0538	2.06	POS	hilic				7.96E-01	7.91E-01	0.08	3.23E-01	0.32	8.06E-01	-0.06	3.16E-01	0.28
129.0576@4.8607855	129.0576	4.86	POS	RP				7.46E-01	8.26E-01	0.07	2.73E-01	-0.27	4.69E-01	-0.23	5.84E-01	0.17
129.0577@5.6154857	129.0577	5.62	POS	RP				7.04E-03	7.65E-01	0.09	5.17E-02	-0.49	5.74E-03	-0.72	1.02E-01	0.47
129.0578@2.4436226	129.0578	2.44	POS	RP				5.63E-02	1.92E-01	-0.22	1.83E-02	0.64	2.03E-01	0.28	2.33E-01	0.27
129.0578@3.9576833	129.0578	3.96	POS	hilic				6.32E-01	2.08E-01	-0.33	6.46E-01	-0.13	1.08E-01	-0.42	9.07E-02	-0.44
129.0788@4.031155	129.0788	4.03	POS	hilic				6.73E-03	1.75E-01	0.38	3.46E-01	-0.21	1.50E-03	-0.86	3.38E-01	0.28
129.0788@4.886183	129.0788	4.89	POS	hilic				3.95E-01	7.07E-01	-0.10	3.62E-01	-0.26	1.60E-01	-0.42	3.51E-01	0.30
129.079@3.7241032	129.0790	3.72	POS	hilic	Pipecolic acid	20 eV: 84.0813 (100)	2	4.61E-01	5.01E-01	0.20	5.21E-01	-0.17	2.45E-01	-0.29	2.99E-01	0.36
129.0791@3.8098817	129.0791	3.81	POS	hilic	Lysine	10 eV: 84.0795 (100), 130.0860 (60)	2	2.07E-02	6.47E-01	-0.15	5.02E-02	-0.48	1.89E-04	-1.04	9.12E-01	-0.03
129.0791@7.0236163	129.0791	7.02	POS	hilic				7.61E-02	2.58E-02	0.68	2.71E-02	0.65	2.09E-04	0.96	1.41E-01	0.44
129.1516@2.8131456	129.1516	2.81	POS	RP				9.32E-01	7.69E-01	0.07	6.85E-01	0.13	4.74E-01	0.22	6.95E-01	-0.10
129.152@0.6080689	129.1520	0.61	POS	hilic				7.65E-01	3.74E-01	0.26	7.77E-01	-0.08	4.03E-01	-0.26	8.39E-01	0.06
129.9668@3.1967528	129.9668	3.20	NEG	hilic				5.87E-01	6.90E-02	0.57	4.67E-01	0.21	2.66E-01	0.33	1.56E-01	0.46
130.0414@3.829871	130.0414	3.83	POS	hilic				6.79E-02	4.31E-01	-0.26	1.88E-02	0.61	1.38E-02	0.63	1.07E-01	0.42
130.0418@1.8777694	130.0418	1.88	POS	RP				6.72E-03	4.35E-01	-0.25	7.65E-04	0.84	1.58E-02	0.68	7.15E-03	0.67
130.0629@0.8811372	130.0629	0.88	NEG	hilic				4.73E-02	1.70E-01	-0.41	1.61E-02	0.65	2.11E-01	0.38	8.64E-01	-0.04
130.0631@3.3287094	130.0631	3.33	NEG	RP				5.33E-02	1.19E-01	-0.49	3.90E-02	0.54	1.97E-01	0.40	7.50E-01	-0.08
130.0634@3.0825312	130.0634	3.08	NEG	RP				2.90E-02	7.73E-02	-0.56	2.99E-02	0.55	1.56E-01	0.43	8.57E-01	-0.04
130.1106@3.3405387	130.1106	3.34	POS	hilic				4.85E-01	5.02E-01	0.21	9.92E-01	0.00	7.54E-01	-0.09	6.77E-02	0.55
130.9242@15.346692	130.9242	15.35	POS	RP				4.46E-01	3.80E-01	0.30	1.82E-01	0.45	3.06E-01	0.29	1.69E-02	0.68
130.9434@15.438294	130.9434	15.44	POS	RP				1.30E-01	8.48E-01	-0.05	4.03E-01	0.25	5.57E-02	0.63	1.25E-02	0.73
131.0579@1.680561	131.0579	1.68	NEG	hilic				4.57E-01	3.40E-01	0.28	3.73E-01	0.23	2.24E-01	-0.30	5.87E-01	0.15
131.0696@5.476163	131.0696	5.48	POS	hilic	Creatine	10 eV: 90.0549 (100), 132.0765 (75), 44.0491 (70)	1	7.21E-03	4.43E-01	0.22	4.59E-01	0.20	8.61E-04	0.83	6.69E-02	-0.52
131.0734@3.9558392	131.0734	3.96	POS	hilic				7.24E-01	6.90E-01	-0.13	2.15E-01	0.37	6.72E-01	0.12	4.54E-01	0.23
131.0736@2.4401617	131.0736	2.44	POS	RP				8.57E-01	9.71E-01	0.01	3.36E-01	0.29	9.12E-01	-0.03	7.86E-01	-0.07
131.0946@3.9148564	131.0946	3.91	POS	hilic	Leucine	10 eV: 86.0962 (100), 43.0538 (7), 30.0342 (5), 44.0498 (4)	2	1.57E-05	2.20E-03	-0.82	2.42E-03	0.68	4.25E-03	0.84	3.19E-02	0.52
131.0946@3.4264941	131.0946	3.43	POS	hilic				9.32E-01	5.90E-01	-0.14	4.65E-01	-0.20	5.24E-01	-0.17	8.68E-01	0.04
131.0949@1.3033249	131.0949	1.30	POS	RP	Leucine/Norleucine	10 eV: 86.0965 (100), 44.0500 (15), 30.0336 (10), 69.0695 (5)	1	8.96E-05	2.82E-03	-0.82	2.29E-03	0.72	2.85E-02	0.63	7.77E-02	0.47
131.0949@1.3161452	131.0949	1.32	NEG	RP				8.78E-06	6.26E-04	-0.87	1.13E-02	0.50	9.00E-03	0.84	3.50E-03	0.66
131.095@4.161487	131.0950	4.16	POS	hilic	Isoleucine	10 eV: 86.0965 (100), 69.0695 (10), 44.0487 (4), 132.0999 (2)	2	5.08E-05	5.49E-04	-0.92	4.88E-03	0.59	2.21E-02	0.64	8.70E-02	0.47
131.095@1.3003676	131.0950	1.30	POS	RP	Leucine/Norleucine	10 eV: 86.0965 (100), 44.0500 (15), 30.0336 (10), 69.0695 (5)	1	2.57E-03	1.46E-02	-0.56	9.60E-04	0.81	1.12E-01	0.50	2.95E-01	0.30
131.9797@1.7967633	131.9797	1.80	POS	hilic				1.10E-02	1.95E-01	-0.36	2.63E-01	0.34	2.64E-03	0.74	2.69E-01	-0.27
131.9998@15.329927	131.9998	15.33	NEG	RP				1.04E-01	1.13E-01	-0.47	5.85E-01	-0.16	5.14E-01	-0.18	7.10E-03	-0.91
132.0244@4.5294046	132.0244	4.53	POS	hilic				4.71E-01	5.93E-01	-0.14	5.81E-01	-0.13	8.80E-02	-0.34	3.29E-01	0.23
132.0252@13.248303	132.0252	13.25	POS	RP				1.83E-01	8.31E-01	-0.06	1.60E-01	0.39	7.01E-02	-0.53	5.83E-01	-0.15
132.0415@5.2340302	132.0415	5.23	NEG	hilic				5.44E-01	8.92E-01	-0.03	7.60E-02	0.52	4.23E-01	0.22	3.94E-01	0.25
132.0532@6.1868196	132.0532	6.19	NEG	hilic				1.82E-01	5.62E-01	-0.16	6.41E-01	-0.14	5.29E-02	-0.51	1.20E-01	0.43
132.0534@6.1388288	132.0534	6.14	POS	hilic	Asparagine	10 eV: 74.0241 (100), 87.0538 (78), 116.0361 (20)	2	1.22E-01	6.51E-01	0.13	9.40E-01	-0.02	1.24E-01	-0.45	2.17E-02	0.57
132.0782@0.6555235	132.0782	0.66	NEG	hilic				1.73E-02	1.45E-02	-0.73	1.63E-01	0.37	6.31E-01	-0.12	2.16E-01	0.38
132.0789@4.147735	132.0789	4.15	NEG	RP				8.23E-01	5.16E-01	-0.14	5.61E-01	0.14	3.71E-01	0.23	7.12E-01	0.09
132.0897@7.079165	132.0897	7.08	POS	hilic	Ornithine	10 eV: 116.0707 (100), 70.0646 (99)	2.00E+00	2.66E-03	1.16E-03	1.02	8.22E-01	-0.06	3.44E-02	0.56	1.75E-03	0.92
132.0902@6.305458	132.0902	6.31	POS	hilic				2.16E-08	2.42E-02	0.45			4.52E-02	0.25	2.95E-06	1.29
133.0115@5.5463676	133.0115	5.55	POS	hilic				8.44E-01	1.27E-01	-0.37	4.87E-01	-0.19	9.14E-01	-0.03	6.99E-01	-0.12
133.0533@0.5896268	133.0533	0.59	POS	hilic				1.08E-01	4.21E-02	0.66	4.67E-01	-0.18	3.99E-01	-0.20	9.46E-01	0.02
133.1097@1.6707184	133.1097	1.67	POS	hilic				9.94E-01	8.46E-01	0.07	7.73E-01	0.09	9.87E-01	0.01	6.43E-01	0.14
134.0368@3.7556875	134.0368	3.76	POS	RP				4.22E-01	5.25E-01	0.19	9.85E-01	0.00	7.24E-02	-0.55	8.64E-01	-0.05
134.094@2.3870485	134.0940	2.39	POS	RP				9.26E-01	6.25E-01	0.15	6.40E-01	-0.12	6.72E-01	0.15	7.58E-01	-0.07
134.0945@2.5807095	134.0945	2.58	POS	RP				1.78E-01	2.84E-01	0.37	6.68E-01	0.11	1.32E-02	0.73	6.37E-01	-0.12
134.1094@8.539423	134.1094	8.54	POS	RP				4.84E-01	7.22E-01	0.08	6.55E-01	-0.13	1.18E-01	0.51	6.10E-01	0.14
134.1094@8.618093	134.1094	8.62	POS	RP				4.93E-01	8.97E-01	-0.03	4.72E-01	-0.21	1.80E-01	0.42	6.00E-01	0.14
135.0287@15.391096	135.0287	15.39	POS	RP				7.69E-01	7.49E-01	0.09	7.57E-01	0.10	4.26E-01	-0.28	3.90E-01	-0.26
135.0332@0.7721816	135.0332	0.77	POS	hilic				3.02E-02	3.08E-02	0.75	8.78E-02	0.39	3.54E-03	0.73	7.40E-01	-0.08
135.041@5.4746556	135.0410	5.47	POS	hilic				4.37E-03	8.21E-01	-0.06	2.77E-01	0.26	5.54E-03	0.72	1.69E-02	-0.59
135.066@2.9358373	135.0660	2.94	POS	RP				9.35E-01	8.41E-01	-0.06	7.85E-01	0.08	4.07E-01	0.27	8.00E-01	0.08
135.068@1.014932	135.0680	1.01	NEG	hilic				6.15E-01	9.11E-01	-0.03	8.57E-01	-0.04	2.06E-01	-0.29	3.57E-01	0.26
135.068@5.0145206	135.0680	5.01	POS	hilic				9.14E-03	4.01E-02	-0.67	3.58E-02	0.53	3.03E-02	0.50	9.05E-01	0.03
135.0683@1.0884997	135.0683	1.09	POS	RP				4.55E-01	6.65E-01	-0.12	1.80E-01	0.36	8.93E-02	0.48	6.71E-01	0.11

Supplementary table 2: Metabolomics analysis results

								ANOVA	Anorexia vs control		overweight vs control		obese vs control		athletes vs control	
Compound	Mass	Retention Time	Ionization mode	column	Compound_ID	MSMS_spectra	ID_level	P-FDR	P	Estimate	P	Estimate	P	Estimate	P	Estimate
136.037@5.606868	136.0370	5.61	NEG	hilic				6.53E-01	4.93E-01	0.22	6.04E-01	0.14	2.38E-01	-0.29	5.00E-01	-0.22
136.0373@5.608196	136.0373	5.61	NEG	hilic				4.68E-01	6.70E-01	0.14	7.63E-01	0.07	1.59E-01	-0.38	1.68E-01	-0.41
136.0378@1.454819	136.0378	1.45	NEG	hilic				1.66E-02	8.19E-02	0.46	8.27E-02	0.49	9.56E-06	1.17	1.13E-01	0.46
136.0387@1.4240268	136.0387	1.42	POS	hilic	Hypoxanthine	10 eV: 137.0452 (100), 119.0349 (9), 110.0321 (4)	2	1.06E-02	7.41E-02	0.48	4.23E-02	0.60	1.29E-05	1.24	1.11E-01	0.48
136.0635@2.177495	136.0635	2.18	POS	hilic	1-Methylnicotinamide	10 eV: 137.0706 (100), 92.0486 (5), 78.0330 (5, 94.0613 (2)	2	3.67E-01	1.64E-01	0.52	2.15E-01	0.34	8.54E-01	0.05	4.57E-01	-0.21
136.9093@13.252808	136.9093	13.25	NEG	RP				6.88E-01	3.70E-01	-0.22	4.01E-01	0.23	5.25E-01	-0.14	6.27E-01	-0.11
137.048@4.1457515	137.0480	4.15	POS	hilic	Trigonelline	10 eV: 138.0547 (100), 92.0499 (5)	2	8.31E-01	8.69E-01	0.06	5.21E-01	-0.19	2.48E-01	-0.36	5.86E-01	-0.13
137.0589@3.3880005	137.0589	3.39	POS	RP				1.94E-01	1.18E-01	0.53	8.48E-01	-0.05	8.57E-01	-0.05	1.43E-01	-0.36
137.0591@0.5057419	137.0591	0.51	POS	hilic				1.40E-02	3.64E-02	0.59	7.59E-01	0.06	4.16E-01	-0.17	3.74E-02	-0.36
137.8755@15.296538	137.8755	15.30	NEG	RP				3.64E-01	6.81E-01	0.10	1.20E-01	0.52	1.17E-02	0.65	1.77E-01	0.36
137.9901@4.635194	137.9901	4.64	NEG	hilic				7.17E-01	5.75E-01	0.16	4.02E-01	-0.22	3.21E-01	0.28	7.10E-01	0.12
138.0313@0.641	138.0313	0.64	NEG	hilic				6.81E-01	5.41E-01	0.12	1.40E-01	0.28	6.37E-01	0.08	7.97E-01	-0.03
138.0315@5.152815	138.0315	5.15	NEG	RP				9.11E-01	9.07E-01	-0.02	4.81E-01	0.14	9.62E-01	0.01	5.55E-01	-0.10
138.0428@1.0756472	138.0428	1.08	POS	hilic				1.66E-01	1.32E-01	-0.44	2.29E-01	0.30	1.46E-01	0.46	6.88E-01	-0.13
138.1158@15.300009	138.1158	15.30	POS	RP				8.70E-01	5.16E-01	0.18	7.75E-01	0.09	9.35E-01	0.02	2.74E-01	0.36
139.061@4.4025455	139.0610	4.40	POS	hilic				9.21E-01	5.87E-01	-0.16	7.97E-01	0.07	8.23E-01	0.07	5.41E-01	-0.20
139.061@5.3543687	139.0610	5.35	POS	hilic				7.44E-01	9.26E-01	-0.03	2.94E-01	0.31	8.69E-01	-0.04	2.78E-01	0.29
139.0611@4.389388	139.0611	4.39	POS	hilic				8.31E-01	9.73E-01	-0.01	9.87E-01	0.00	1.58E-01	-0.32	7.97E-01	-0.07
139.136@2.3465304	139.1360	2.35	POS	RP				8.39E-01	3.70E-01	0.27	9.30E-01	0.02	9.03E-01	0.04	2.92E-01	0.34
139.1361@0.74637556	139.1361	0.75	POS	hilic				4.94E-01	1.31E-01	0.46	5.14E-01	-0.22	3.41E-01	0.26	6.79E-01	0.12
140.0327@2.1529884	140.0327	2.15	NEG	hilic				1.67E-01	2.67E-02	0.59	2.38E-01	-0.31	5.88E-01	0.18	5.03E-01	0.16
140.0583@5.1961374	140.0583	5.20	POS	hilic				6.39E-01	9.05E-01	-0.03	2.38E-01	0.29	3.30E-01	0.27	1.10E-01	0.42
140.1063@2.5103953	140.1063	2.51	POS	hilic	Methenamine	10 eV: 141.1124 (100), 112.0859 (78), 98.0727 (25), 85.0732 (15), 71.0605 (13)	2	9.11E-01	8.43E-01	-0.07	4.15E-01	0.22	4.34E-01	0.22	8.61E-01	0.05
140.9521@15.326193	140.9521	15.33	POS	RP				5.12E-01	6.81E-02	0.62	1.45E-01	0.46	1.02E-01	0.45	2.25E-01	0.36
141.0176@1.8531269	141.0176	1.85	NEG	hilic				7.71E-01	5.52E-01	0.17	3.02E-01	-0.23	7.88E-01	0.08	5.38E-01	-0.15
141.0792@1.3285648	141.0792	1.33	POS	hilic				3.28E-01	4.04E-01	-0.21	7.80E-01	0.08	3.74E-02	0.53	8.96E-01	-0.04
141.0796@2.9660592	141.0796	2.97	POS	hilic				8.28E-01	5.70E-01	-0.14	9.78E-01	-0.01	2.88E-01	-0.25	2.93E-01	-0.27
141.1148@1.0038096	141.1148	1.00	POS	hilic				3.87E-01	7.31E-02	-0.45	8.61E-01	0.05	4.25E-01	0.22	7.52E-01	0.08
141.1517@8.725725	141.1517	8.73	POS	RP				8.67E-01	4.33E-01	-0.24	7.86E-01	0.07	3.51E-01	-0.27	6.92E-01	-0.13
142.0083@13.243363	142.0083	13.24	POS	RP				2.24E-01	9.71E-01	0.01	4.68E-02	0.53	2.48E-01	-0.29	5.46E-01	-0.20
142.0087@0.39691305	142.0087	0.40	POS	hilic				2.77E-01	5.39E-01	-0.18	1.85E-01	0.36	1.79E-01	-0.37	2.02E-01	-0.36
142.0741@5.2478094	142.0741	5.25	POS	hilic				7.00E-02	1.42E-01	-0.25	9.34E-01	0.02	4.75E-01	0.16	1.57E-02	0.61
142.1104@1.5290929	142.1104	1.53	POS	hilic				6.64E-05	2.16E-03	0.93	3.17E-01	-0.24	2.25E-04	-0.82	9.65E-01	0.01
142.8752@15.342186	142.8752	15.34	NEG	RP				6.48E-01	6.65E-01	0.13	5.69E-01	-0.15	5.25E-01	-0.18	2.74E-01	0.30
142.9523@15.256681	142.9523	15.26	POS	RP				5.16E-02	1.75E-04	-0.88	7.02E-01	-0.10	5.27E-01	0.18	8.01E-01	0.08
143.0327@1.8520774	143.0327	1.85	NEG	hilic				6.07E-01	2.28E-01	0.35	3.05E-01	-0.26	9.50E-01	-0.02	7.81E-01	0.08
143.0729@3.9574876	143.0729	3.96	POS	hilic				4.15E-01	9.02E-01	-0.03	4.35E-02	0.60	3.17E-01	0.30	6.63E-01	0.13
143.0732@2.440973	143.0732	2.44	POS	RP				7.44E-01	2.64E-01	-0.32	8.29E-01	0.07	4.15E-01	0.22	9.46E-01	-0.02
143.0941@5.0568953	143.0941	5.06	POS	hilic				5.78E-01	8.27E-01	-0.06	1.80E-01	0.38	8.41E-01	0.06	1.77E-01	0.42
143.0944@2.8997605	143.0944	2.90	POS	hilic				2.37E-03	1.87E-02	-0.80	1.79E-02	0.63	4.35E-01	0.20	3.35E-01	-0.24
143.0946@3.4850678	143.0946	3.49	POS	hilic				8.98E-01	7.10E-01	-0.12	6.12E-01	-0.15	9.47E-01	-0.02	2.12E-01	-0.33
143.0946@4.034845	143.0946	4.03	POS	hilic				8.12E-01	5.60E-01	-0.20	6.38E-01	0.15	9.76E-01	-0.01	3.64E-01	0.26
143.0948@0.58951133	143.0948	0.59	POS	hilic				4.73E-01	7.75E-02	0.52	8.58E-01	0.05	8.99E-01	0.03	5.53E-01	-0.15
143.1669@0.57944465	143.1669	0.58	POS	hilic				9.16E-01	6.87E-01	-0.13	5.22E-01	-0.18	5.89E-01	0.16	8.74E-01	-0.05
143.9744@1.0505669	143.9744	1.05	POS	RP				5.28E-01	9.78E-02	0.49	9.63E-02	0.46	6.09E-01	0.14	8.42E-01	0.06
144.0419@5.188255	144.0419	5.19	NEG	hilic				4.17E-01	6.26E-01	-0.10	9.19E-02	0.46	9.43E-01	0.02	9.78E-01	0.01
144.0421@2.8990338	144.0421	2.90	POS	hilic				3.98E-03	4.00E-02	-0.71	1.19E-02	0.68	4.15E-01	0.21	5.08E-01	-0.17
144.079@8.347538	144.0790	8.35	POS	RP				6.18E-01	1.48E-01	-0.46	5.34E-01	0.15	8.87E-01	0.04	9.87E-01	0.00
144.8727@15.352193	144.8727	15.35	NEG	RP				6.24E-01	6.92E-01	-0.11	8.03E-01	-0.07	4.17E-02	-0.48	6.97E-01	-0.10
144.9476@15.255891	144.9476	15.26	POS	RP				9.51E-01	6.49E-01	0.14	9.74E-01	0.01	6.31E-01	0.13	4.14E-01	0.25
145.0201@1.4413334	145.0201	1.44	POS	hilic				8.06E-02	1.80E-01	-0.24	9.21E-01	-0.02	6.83E-02	0.44	1.38E-01	-0.24
145.0526@1.4134004	145.0526	1.41	POS	hilic				3.86E-01	8.46E-02	-0.44	8.35E-02	-0.41	1.66E-02	-0.58	3.05E-01	-0.30
145.0526@3.9568305	145.0526	3.96	POS	hilic				4.24E-01	3.12E-01	-0.28	3.34E-01	0.30	1.17E-01	0.40	8.01E-01	-0.08
145.0528@2.4409657	145.0528	2.44	POS	RP	1H-Indole-4-carboxaldehyde	10 eV: 118.0645 (100), 146.0607 (45)	2	6.02E-01	4.63E-02	-0.52	7.64E-01	0.10	7.62E-01	-0.08	8.37E-01	-0.06
145.0529@2.0410607	145.0529	2.04	POS	RP				3.66E-01	3.82E-01	-0.26	8.58E-02	0.52	3.97E-01	0.21	6.56E-01	0.14
145.0529@4.5103416	145.0529	4.51	POS	RP				8.90E-02	1.38E-01	0.41	8.01E-01	0.07	2.32E-01	-0.24	1.79E-02	0.63
145.0558@0.52271885	145.0558	0.52	POS	hilic				9.19E-01	5.02E-01	0.16	3.20E-01	0.19	3.87E-01	0.18	2.80E-01	0.20
145.0735@0.7896463	145.0735	0.79	POS	hilic				2.33E-01	3.43E-01	0.28	2.40E-01	0.30	4.34E-01	0.20	1.39E-02	0.84
145.0738@0.79303485	145.0738	0.79	POS	hilic				2.95E-01	3.91E-01	0.25	5.75E-01	0.15	7.37E-01	0.09	2.12E-02	0.75
145.0739@4.8155937	145.0739	4.82	POS	hilic				3.37E-02	6.09E-04	-0.82	1.27E-01	-0.40	2.32E-03	-0.72	4.14E-01	-0.22
145.1101@3.2600265	145.1101	3.26	POS	hilic				3.13E-01	4.26E-01	-0.28	1.87E-01	0.36	1.12E-01	0.47	1.00E+00	0.00
145.1103@3.1860774	145.1103	3.19	POS	hilic				3.65E-01	1.66E-01	-0.46	2.72E-01	0.34	6.54E-01	0.12	7.28E-01	0.09
145.9111@15.266749	145.9111	15.27	NEG	RP				7.88E-01	7.61E-01	0.11	7.56E-01	0.09	7.78E-01	0.08	2.50E-01	-0.32
145.9738@15.328926	145.9738	15.33	POS	RP				8.87E-01	1.00E+00	0.00	3.09E-01	0.33	7.39E-01	0.11	9.44E-01	0.02
145.9744@1.8688331	145.9744	1.87	NEG	hilic				3.75E-02	4.13E-02	-0.49	7.88E-02	0.50	1.66E-01	0.40	7.19E-01	0.08
145.9993@15.346149	145.9993	15.35	NEG	RP				5.48E-01	4.35E-01	-0.23	4.81E-01	0.23	2.57E-01	-0.35	3.52E-01	-0.28
146.069@6.0790167	146.0690	6.08	NEG	hilic				8.13E-01	6.95E-01	0.13	9.83E-01	-0.01	1.68E-01	-0.34	7.20E-01	-0.11
146.0693@6.025324	146.0693	6.03	POS	hilic	Glutamine	10 eV: 130.0496 (100), 84.0443 (78), 147.0756 (8), 101.0705 (7)	1	7.98E-01	8.70E-01	0.05	8.43E-01	-0.06	1.15E-01	-0.39	5.53E-01	-0.18
146.0943@8.809741	146.0943	8.81	POS	RP				8.25E-01	6.83E-01	-0.13	2.57E-01	0.30	6.07E-01	0.15	5.47E-01	0.17
146.1052@6.1161766	146.1052	6.12	POS	hilic				2.36E-01	9.79E-01	-0.01	9.58E-01	-0.01	6.36E-01	-0.12	8.92E-03	-0.74
146.1056@5.593138	146.1056	5.59	POS	hilic				4.60E-02	7.85E-01	-0.10	2.27E-02	-0.54	1.03E-02	-0.78	1.54E-04	-0.88
146.1057@7.0247946	146.1057	7.02	POS	hilic	Lysine	10 eV: 84.0805 (100), 130.0851 (80), 105.9561 (15), 147.1119 (10)	2	3.19E-01	1.49E-02	0.67	1.13E-01	0.45	1.92E-02	0.66	1.50E-01	0.42
147.0328@3.4643612	147.0328	3.46	POS	RP				8.16E-01	1.59E-01	0.43	3.31E-01	0.26	3.37E-01	0.23	4.97E-01	0.21

Supplementary table 2: Metabolomics analysis results

								ANOVA	Anorexia vs control		overweight vs control		obese vs control		athletes vs control	
Compound	Mass	Retention Time	Ionization mode	column	Compound_ID	MSMS_spectra	ID_level	P-FDR	P	Estimate	P	Estimate	P	Estimate	P	Estimate
147.0533@6.2765384	147.0533	6.28	POS	hilic	Glutamate			9.29E-04	7.80E-01	0.07	4.31E-02	0.54	5.11E-06	1.32	7.26E-02	0.44
147.0895@4.7697954	147.0895	4.77	POS	hilic				8.18E-01	3.00E-01	0.40	7.10E-01	0.10	8.66E-01	-0.04	8.38E-01	0.05
147.0896@4.1091585	147.0896	4.11	POS	hilic				6.31E-01	6.84E-01	-0.14	9.44E-01	-0.02	5.90E-01	-0.13	6.52E-02	-0.54
147.0896@4.8976693	147.0896	4.90	NEG	hilic				5.51E-01	1.59E-01	0.45	2.59E-01	0.32	9.44E-02	0.44	9.55E-01	0.01
147.908@15.262468	147.9080	15.26	NEG	RP				6.67E-01	3.27E-01	-0.26	9.18E-01	0.03	2.50E-01	0.36	7.42E-01	0.08
147.9449@15.261137	147.9449	15.26	NEG	RP				7.90E-01	7.75E-01	-0.08	9.43E-01	0.02	4.13E-01	0.21	2.43E-01	0.36
148.0152@5.496655	148.0152	5.50	POS	RP				9.94E-01	8.57E-01	0.04	9.05E-01	-0.02	8.18E-01	0.05	7.11E-01	0.08
148.0158@7.179677	148.0158	7.18	POS	RP				9.64E-01	9.87E-01	0.00	9.16E-01	-0.03	9.89E-01	0.00	4.97E-01	0.22
148.016@9.267434	148.0160	9.27	POS	RP				8.67E-01	7.20E-01	-0.11	2.91E-01	-0.37	7.80E-01	-0.08	4.59E-01	-0.21
148.0161@10.908634	148.0161	10.91	POS	RP				6.71E-01	7.16E-01	0.09	5.71E-01	-0.17	9.21E-01	-0.03	1.44E-01	0.40
148.0161@9.190386	148.0161	9.19	POS	RP				8.09E-01	3.31E-01	-0.31	9.60E-01	0.01	6.17E-01	0.16	7.13E-01	0.11
148.0162@10.304535	148.0162	10.30	POS	RP				7.31E-01	7.62E-01	0.08	3.88E-01	-0.23	7.44E-01	0.10	3.22E-01	0.27
148.0167@0.4061363	148.0167	0.41	POS	hilic				8.34E-01	6.09E-01	-0.13	2.83E-01	-0.27	7.84E-01	-0.08	7.03E-01	0.10
148.0522@9.535206	148.0522	9.54	POS	RP				9.77E-01	6.72E-01	-0.13	9.90E-01	0.00	6.78E-01	0.12	8.45E-01	-0.07
148.053@3.831673	148.0530	3.83	POS	hilic				1.07E-01	6.89E-01	-0.12	1.53E-02	0.60	4.85E-02	0.58	8.19E-01	-0.05
148.1252@8.5359	148.1252	8.54	POS	RP				3.80E-01	2.11E-01	-0.22	9.70E-01	-0.01	1.57E-01	0.38	9.00E-01	-0.03
148.1252@8.623873	148.1252	8.62	POS	RP				4.06E-01	1.90E-01	-0.24	8.14E-01	-0.05	1.91E-01	0.34	8.08E-01	-0.05
148.9455@15.259295	148.9455	15.26	POS	RP				8.62E-01	4.69E-01	0.20	7.96E-01	-0.08	4.41E-01	-0.24	7.92E-01	-0.08
149.0064@6.0759697	149.0064	6.08	POS	hilic				6.42E-01	1.88E-01	-0.40	2.15E-01	-0.32	9.16E-02	-0.52	2.48E-01	-0.33
149.0507@4.52954	149.0507	4.53	POS	hilic	Methionine	10 eV: 104.0522 (100), 56.0488 (75), 133.0305 (65)	2	4.91E-01	1.89E-01	-0.45	8.47E-01	-0.05	1.01E-01	-0.38	5.78E-01	0.16
149.0509@0.87475014	149.0509	0.87	POS	hilic				1.99E-01	7.79E-01	0.09	2.25E-01	-0.31	2.71E-02	-0.62	5.63E-01	0.20
149.1052@2.8043842	149.1052	2.80	POS	hilic	Triethanolamine	10 eV: 150.1118 (100), 132.1021 (40), 45.0330 (9), 88.0905 (8), 70.0632 (8)		8.41E-01	6.21E-01	-0.11	2.00E-01	-0.28	5.65E-01	-0.14	2.88E-01	-0.25
150.0193@8.349511	150.0193	8.35	POS	RP				8.59E-01	8.13E-01	-0.06	5.67E-01	0.13	8.77E-01	0.04	3.10E-01	-0.21
150.028@7.3150797	150.0280	7.32	POS	hilic				3.24E-01	5.49E-01	-0.19	6.32E-01	-0.12	1.71E-01	-0.38	1.64E-01	0.37
150.0284@15.255544	150.0284	15.26	POS	RP				9.94E-01	6.48E-01	-0.12	8.03E-01	-0.08	8.46E-01	-0.06	6.49E-01	-0.12
150.0523@5.2293787	150.0523	5.23	NEG	hilic				3.12E-01	1.44E-01	-0.39	8.99E-02	0.44	9.17E-01	0.03	5.80E-01	0.16
150.0526@3.998744	150.0526	4.00	NEG	hilic				1.80E-02	6.48E-02	0.50	1.25E-01	-0.34	1.86E-02	-0.59	8.81E-01	0.04
150.0687@6.864846	150.0687	6.86	POS	RP				5.38E-01	7.32E-01	0.09	1.71E-01	-0.34	3.65E-01	-0.27	5.69E-01	0.15
150.0896@0.6752906	150.0896	0.68	POS	hilic				1.14E-01	3.02E-01	0.30	6.01E-01	-0.14	1.73E-01	0.48	1.08E-01	-0.46
150.1368@1.4923913	150.1368	1.49	POS	hilic	Trimethylamine-N-oxide (TMAO)	10 eV: 76.0757 (100), 151.1387 (5)	2	7.84E-01	1.57E-01	0.41	8.92E-01	0.04	8.36E-01	-0.06	9.49E-01	0.02
150.1407@8.625113	150.1407	8.63	POS	RP				5.96E-01	2.75E-01	-0.24	4.22E-01	-0.19	3.76E-01	0.26	8.91E-01	0.03
150.1409@8.548317	150.1409	8.55	POS	RP				5.31E-01	4.69E-01	-0.15	9.34E-01	-0.02	1.81E-01	0.39	8.09E-01	-0.06
150.9429@15.265569	150.9429	15.27	POS	RP				8.77E-01	2.44E-01	0.28	4.45E-01	0.18	3.53E-01	0.24	7.24E-01	0.07
151.9903@15.436277	151.9903	15.44	NEG	RP				2.51E-01	2.65E-02	0.73	3.59E-01	0.25	7.39E-01	-0.09	7.85E-01	0.09
152.0329@1.8145479	152.0329	1.81	NEG	hilic				2.24E-02	4.41E-01	-0.22	1.11E-01	0.43	2.45E-02	0.69	2.27E-01	-0.36
152.0473@4.6293197	152.0473	4.63	NEG	RP				4.22E-01	8.09E-01	0.07	2.65E-01	-0.25	1.94E-01	-0.30	4.08E-02	-0.44
152.0583@1.4230007	152.0583	1.42	POS	RP				1.88E-02	1.24E-02	0.94	4.82E-02	0.53	9.24E-04	0.99	3.05E-01	0.25
152.059@0.7614279	152.0590	0.76	POS	hilic				3.90E-02	2.49E-02	0.83	9.56E-02	0.42	2.14E-03	0.90	6.04E-01	0.13
152.12@8.625319	152.1200	8.63	POS	RP				6.30E-01	9.28E-01	-0.02	6.81E-01	-0.12	1.95E-01	0.43	6.20E-01	0.14
152.1203@8.536046	152.1203	8.54	POS	RP				5.42E-01	9.09E-01	0.03	7.77E-01	-0.08	1.32E-01	0.51	4.79E-01	0.20
153.0191@4.783297	153.0191	4.78	POS	hilic				6.97E-01	7.71E-01	-0.09	8.79E-01	-0.04	9.02E-01	0.03	2.27E-01	0.43
153.0541@1.4132106	153.0541	1.41	POS	hilic				1.70E-01	1.36E-01	0.41	9.94E-02	0.40	8.94E-01	0.03	2.30E-01	-0.26
153.986@15.431656	153.9860	15.43	NEG	RP				1.65E-01	2.36E-04	0.85	6.90E-02	0.55	1.53E-01	0.34	6.32E-01	0.15
154.0263@0.5088129	154.0263	0.51	NEG	hilic				1.19E-02	6.01E-01	0.14	2.26E-02	-0.61	3.31E-03	-0.82	6.59E-01	0.14
154.0357@6.1301384	154.0357	6.13	POS	hilic				2.27E-01	2.12E-01	-0.34	4.26E-01	-0.25	3.69E-02	-0.62	4.41E-01	0.22
154.0373@3.7510555	154.0373	3.75	NEG	hilic				1.70E-01	8.21E-01	0.07	9.02E-02	0.48	6.10E-01	-0.14	1.56E-01	-0.34
154.0993@0.43564713	154.0993	0.44	POS	hilic				8.34E-01	9.79E-01	-0.01	3.84E-01	0.19	4.02E-01	0.18	7.86E-01	-0.06
154.1244@15.306698	154.1244	15.31	POS	RP				9.77E-01	4.14E-01	0.21	7.20E-01	0.10	4.98E-01	0.19	8.26E-01	0.07
154.136@8.638343	154.1360	8.64	POS	RP				1.51E-01	1.90E-02	-0.71	9.10E-01	0.03	6.56E-01	-0.12	5.25E-01	0.19
155.0346@3.0282238	155.0346	3.03	POS	hilic				8.83E-01	9.31E-01	-0.03	8.88E-01	0.04	2.74E-01	-0.32	6.34E-01	-0.14
155.0346@3.115896	155.0346	3.12	POS	hilic				9.65E-01	7.96E-01	-0.08	5.76E-01	0.17	8.47E-01	-0.05	9.61E-01	0.02
155.0348@3.6788375	155.0348	3.68	POS	hilic				7.15E-01	3.34E-01	-0.26	3.56E-01	-0.28	5.62E-01	0.19	9.00E-01	0.04
155.0692@6.737642	155.0692	6.74	NEG	hilic				5.99E-01	7.21E-01	0.13	5.65E-02	0.52	2.97E-01	0.29	1.65E-01	0.44
155.0696@6.6856413	155.0696	6.69	POS	hilic	Histidine	10 eV: 110.0713 (100), 156.0767 (45), 95.0602 (20)	2	7.69E-01	8.16E-01	0.08	2.71E-01	0.30	4.22E-01	0.24	1.40E-01	0.45
155.1676@9.204164	155.1676	9.20	POS	RP				9.11E-01	7.32E-01	-0.11	9.43E-01	0.02	3.32E-01	-0.29	5.01E-01	-0.20
155.94@15.259696	155.9400	15.26	NEG	RP				4.64E-01	3.15E-01	-0.35	1.14E-01	-0.45	6.47E-01	0.13	1.95E-01	-0.35
156.0029@1.87099	156.0029	1.87	NEG	hilic				5.58E-02	5.58E-02	-0.56	4.54E-01	0.22	1.15E-01	0.50	4.00E-01	-0.21
156.0784@6.439703	156.0784	6.44	NEG	RP				7.31E-01	1.85E-01	-0.41	9.04E-01	0.03	6.20E-01	-0.13	8.80E-01	0.04
156.0788@6.433657	156.0788	6.43	NEG	RP				6.90E-01	1.84E-01	-0.42	9.23E-01	0.03	8.50E-01	0.05	7.94E-01	0.07
157.0597@4.119857	157.0597	4.12	NEG	hilic				4.02E-01	7.98E-01	0.06	3.61E-02	-0.42	6.02E-01	0.15	7.27E-01	-0.08
157.0602@3.3079891	157.0602	3.31	POS	hilic				7.76E-01	8.76E-01	0.04	4.04E-01	-0.23	4.60E-01	0.25	8.28E-01	-0.06
157.0603@4.7238383	157.0603	4.72	POS	hilic				6.98E-01	5.87E-01	0.15	3.07E-01	-0.29	6.06E-01	0.19	5.42E-01	-0.15
157.0965@1.244119	157.0965	1.24	POS	hilic				7.28E-02	2.15E-01	-0.36	3.56E-02	0.64	1.66E-01	0.36	9.52E-01	0.02
157.1103@3.0136266	157.1103	3.01	POS	hilic				2.89E-01	5.97E-01	0.19	2.93E-02	-0.54	1.06E-01	-0.38	5.25E-01	-0.17
157.1467@1.2455338	157.1467	1.25	POS	hilic				7.06E-01	4.41E-01	0.25	4.85E-01	0.20	7.90E-02	0.51	8.28E-01	0.07
157.1469@0.4368976	157.1469	0.44	POS	hilic				7.34E-01	8.48E-01	0.06	1.99E-01	-0.43	6.41E-01	-0.13	7.84E-01	-0.07
157.1471@7.3530774	157.1471	7.35	POS	RP				6.85E-01	3.55E-01	-0.27	9.38E-01	-0.02	3.13E-01	0.29	5.69E-01	0.19
157.9541@1.0524567	157.9541	1.05	POS	RP				7.34E-01	3.75E-01	0.26	3.21E-01	0.28	5.17E-01	-0.18	5.82E-01	0.18
157.9569@4.348701	157.9569	4.35	POS	hilic				8.17E-01	5.93E-01	0.14	9.18E-01	0.03	5.68E-01	-0.17	3.56E-01	-0.30
157.9906@15.408698	157.9906	15.41	POS	RP				1.61E-01	1.49E-01	0.36	3.36E-02	0.56	7.95E-01	-0.06	3.98E-02	0.63
158.0037@15.353507	158.0037	15.35	NEG	RP				7.96E-01	1.98E-01	-0.34	5.56E-01	-0.15	7.29E-01	-0.10	2.17E-01	-0.34
158.0191@1.5541959	158.0191	1.55	NEG	hilic				4.68E-01	1.17E-01	0.51	1.58E-01	0.37	1.04E-01	0.49	2.59E-02	0.60
158.0193@1.4500896	158.0193	1.45	NEG	hilic				8.96E-01	8.83E-01	-0.05	4.57E-01	0.19	8.76E-01	0.04	2.89E-01	0.26

Supplementary table 2: Metabolomics analysis results

Compound	Mass	Retention Time	Ionization mode	column	Compound_ID	MSMS_spectra	ID_level	ANOVA P-FDR	Anorexia vs control P	Anorexia vs control Estimate	overweight vs control P	overweight vs control Estimate	obese vs control P	obese vs control Estimate	athletes vs control P	athletes vs control Estimate
158.0435@2.0051708	158.0435	2.01	NEG	hilic				9.70E-01	7.05E-01	0.13	6.71E-01	-0.11	7.64E-01	-0.10	9.25E-01	0.02
158.0578@4.510972	158.0578	4.51	POS	RP				5.75E-01	3.07E-01	-0.29	3.74E-01	-0.28	4.49E-01	0.24	6.84E-01	0.12
158.0689@6.2694435	158.0689	6.27	POS	hilic				1.41E-01	1.37E-01	0.48	5.91E-01	0.17	7.92E-01	-0.07	7.46E-03	0.77
158.0844@2.4411542	158.0844	2.44	POS	RP				6.24E-01	3.07E-01	-0.30	3.32E-01	0.30	5.73E-01	0.16	6.49E-01	0.15
158.0844@3.9589329	158.0844	3.96	POS	hilic				7.14E-01	6.66E-01	-0.13	2.44E-01	0.37	3.03E-01	0.26	5.97E-01	0.17
158.0845@2.0415134	158.0845	2.04	POS	RP				8.98E-01	2.79E-01	-0.33	9.24E-01	-0.03	9.96E-01	0.00	8.83E-01	-0.04
158.0945@6.4211426	158.0945	6.42	NEG	RP				2.58E-01	7.29E-02	-0.47	3.87E-01	-0.24	1.81E-01	-0.39	2.94E-01	0.29
158.8705@15.340465	158.8705	15.34	NEG	RP				8.35E-01	6.98E-01	-0.10	1.73E-01	-0.40	8.45E-01	-0.05	7.78E-01	-0.09
158.9062@15.321702	158.9062	15.32	NEG	RP				6.09E-01	7.89E-01	-0.08	2.29E-01	-0.34	6.18E-02	-0.50	6.36E-01	-0.13
159.0271@4.7761617	159.0271	4.78	POS	hilic				6.07E-01	6.10E-01	-0.17	5.67E-01	0.15	1.63E-01	0.38	3.22E-01	0.34
159.035@10.2764	159.0350	10.28	POS	RP				1.56E-01	5.92E-02	-0.56	2.53E-01	0.30	5.96E-01	0.13	3.10E-01	-0.32
159.0894@0.82033324	159.0894	0.82	POS	hilic				7.05E-01	6.09E-01	-0.18	5.83E-01	-0.15	1.37E-01	-0.40	1.27E-01	-0.45
159.0894@4.565365	159.0894	4.57	POS	hilic				4.24E-01	9.97E-01	0.00	5.63E-01	0.13	9.35E-01	-0.02	3.99E-02	0.55
159.0895@4.525885	159.0895	4.53	POS	hilic				8.14E-02	8.63E-01	0.06	8.36E-01	0.05	7.97E-01	-0.05	2.99E-03	0.80
159.1258@2.3197322	159.1258	2.32	POS	hilic	5-Aminovaleric acid betaine	10 eV: 160.1337 (100), 60.0804 (15), 101.0603 (12)	1	4.40E-02	7.02E-03	-0.63	9.52E-01	-0.02	3.48E-01	-0.25	1.23E-01	0.39
159.126@2.017698	159.1260	2.02	POS	hilic	Valine/norvaline betaine			1.80E-01	5.05E-01	0.24	3.88E-01	0.23	6.08E-01	0.14	2.33E-02	-0.58
159.1261@3.875059	159.1261	3.88	POS	RP				7.04E-02	9.82E-02	-0.46	8.82E-01	-0.04	1.41E-01	-0.45	6.07E-02	0.51
159.1262@2.0310857	159.1262	2.03	POS	hilic	Valine/norvaline betaine			5.75E-01	4.32E-01	0.29	9.33E-01	0.03	5.78E-01	0.14	1.79E-01	-0.36
159.9854@7.367169	159.9854	7.37	NEG	hilic				9.63E-01	9.18E-01	-0.03	5.01E-01	-0.17	4.40E-01	-0.23	6.64E-01	-0.14
159.986@15.251381	159.9860	15.25	NEG	RP				9.22E-01	5.75E-01	-0.17	2.88E-01	-0.28	4.12E-01	-0.21	6.76E-01	-0.12
160.1204@4.4486704	160.1204	4.45	POS	hilic				4.67E-01	9.95E-01	0.00	1.49E-01	-0.43	1.53E-01	-0.41	5.53E-02	-0.50
160.121@6.718236	160.1210	6.72	POS	hilic				5.15E-01	4.74E-02	-0.58	8.85E-01	-0.04	3.56E-01	-0.25	2.97E-01	-0.34
161.068@3.5324166	161.0680	3.53	NEG	hilic				5.89E-02	4.25E-01	0.21	4.45E-01	0.19	3.80E-03	-0.67	7.71E-01	-0.09
161.105@4.7506323	161.1050	4.75	POS	hilic	Carnitine	10 eV: 162.1125 (100), 103.0387 (16), 60.0803 (15)	2	5.89E-01	2.96E-02	-0.52	7.05E-01	-0.10	2.53E-01	-0.35	3.35E-01	-0.25
161.1164@3.680508	161.1164	3.68	POS	hilic				6.72E-01	2.54E-01	-0.32	1.58E-01	-0.43	9.44E-01	0.02	7.34E-01	-0.10
161.9837@15.251117	161.9837	15.25	NEG	RP				7.51E-01	2.27E-01	0.31	6.00E-01	0.11	9.60E-01	-0.01	2.89E-01	0.29
162.0328@1.4115651	162.0328	1.41	NEG	hilic				3.37E-01	4.48E-02	0.53	6.03E-01	0.12	4.75E-02	0.62	4.15E-01	0.21
162.0525@5.1936617	162.0525	5.19	NEG	hilic				9.09E-01	3.28E-01	-0.21	6.82E-01	0.11	8.86E-01	0.04	8.61E-01	-0.05
162.0525@5.2154894	162.0525	5.22	NEG	hilic				2.64E-01	9.70E-02	-0.46	1.20E-01	0.42	7.69E-01	0.08	8.81E-01	0.05
162.0525@5.2163053	162.0525	5.22	NEG	hilic				2.54E-01	5.34E-02	-0.54	1.72E-01	0.36	9.20E-01	0.03	9.68E-01	-0.01
162.0669@9.106301	162.0669	9.11	POS	RP				8.75E-01	7.75E-01	-0.07	7.58E-01	0.09	3.34E-01	-0.29	9.76E-01	0.01
162.0674@8.209848	162.0674	8.21	POS	RP				3.30E-01	7.44E-01	-0.06	8.28E-01	-0.04	1.21E-01	0.40	5.01E-01	-0.13
162.0679@5.7847853	162.0679	5.78	POS	RP				9.35E-01	7.92E-01	0.07	5.77E-01	-0.15	4.93E-01	-0.24	8.68E-01	-0.05
162.0681@8.136055	162.0681	8.14	POS	RP				1.63E-01	8.70E-01	0.03	6.93E-01	0.08	2.69E-02	0.58	8.37E-01	-0.04
162.116@1.3207369	162.1160	1.32	POS	hilic				4.74E-04	1.18E-03	0.92	4.40E-02	0.26	8.61E-02	0.28		
162.1256@5.453019	162.1256	5.45	POS	RP				9.92E-01	7.64E-01	0.10	8.96E-01	0.04	6.28E-01	0.14	6.21E-01	0.14
162.9703@15.416721	162.9703	15.42	POS	RP				7.13E-01	8.66E-01	0.06	8.38E-01	-0.06	1.54E-01	0.44	4.98E-01	0.20
163.0221@5.687787	163.0221	5.69	POS	hilic				2.41E-01	3.84E-01	-0.27	5.60E-01	-0.15	4.57E-01	-0.23	5.79E-02	0.52
163.0662@3.415267	163.0662	3.42	POS	hilic				5.42E-01	2.77E-01	0.32	3.54E-02	0.45	6.74E-01	0.08	2.79E-01	0.23
163.0666@0.73309094	163.0666	0.73	POS	hilic				1.02E-01	4.50E-01	0.18	8.12E-01	-0.05	5.55E-02	-0.30	7.70E-02	0.46
164.0239@0.46723056	164.0239	0.47	POS	hilic				9.92E-01	7.08E-01	-0.12	8.12E-01	0.07	9.50E-01	-0.02	9.41E-01	-0.02
164.0472@5.015419	164.0472	5.02	POS	hilic	p-Hydroxyphenyllactic acid	10 eV: 123.0437 (100), 95.0488 (50), 119.0479 (42), 147.0445 (30), 165.0540 (25)	2	2.75E-02	4.29E-02	-0.67	1.51E-01	0.37	6.38E-02	0.47	9.59E-01	-0.01
164.0473@1.0875579	164.0473	1.09	POS	RP				1.51E-01	9.86E-01	0.01	7.31E-02	0.52	6.29E-03	0.78	2.99E-01	0.28
164.0838@10.757396	164.0838	10.76	POS	RP				3.39E-01	6.90E-01	0.12	6.30E-01	0.14	1.56E-02	0.72	5.02E-01	0.16
164.9851@12.383488	164.9851	12.38	NEG	RP				9.71E-01	8.03E-01	-0.07	9.20E-01	0.03	5.50E-01	-0.17	7.90E-01	0.08
165.0455@5.9928284	165.0455	5.99	POS	hilic				4.06E-02	2.95E-01	0.29	8.17E-02	-0.39	3.37E-03	-0.59	7.09E-01	0.10
165.0554@1.2919719	165.0554	1.29	POS	hilic				2.44E-02	4.05E-02	0.61	5.94E-02	-0.52	3.07E-01	-0.27	5.45E-01	0.18
165.0653@1.4960177	165.0653	1.50	POS	hilic				3.34E-01	8.04E-01	0.08	4.95E-01	-0.20	1.82E-02	-0.59	1.11E-01	-0.49
165.0658@1.6287202	165.0658	1.63	POS	hilic				1.80E-01	2.25E-01	0.31	6.66E-01	-0.11	8.45E-01	0.05	2.91E-02	0.68
165.079@1.2024221	165.0790	1.20	NEG	RP				1.28E-01	2.44E-01	-0.38	8.19E-02	0.46	8.42E-02	0.44	1.21E-01	0.42
165.079@4.003874	165.0790	4.00	NEG	hilic				1.86E-01	3.88E-01	0.29	4.57E-03	0.78	2.86E-02	0.66	9.27E-02	0.50
165.0791@1.8905048	165.0791	1.89	NEG	RP				2.48E-02	2.32E-01	-0.39	8.08E-02	0.47	3.76E-02	0.54	5.08E-03	0.67
165.0793@3.828564	165.0793	3.83	POS	hilic	Phenylalanine	10 eV: 120.0812 (100), 166.0856 (10), 103.0538 (7), 131.0512 (7)	2	1.23E-02	3.13E-01	-0.33	3.52E-03	0.74	3.32E-03	0.71	1.60E-01	0.37
165.0794@1.8761706	165.0794	1.88	POS	RP	Phenylalanine	10 eV: 120.0804 (100), 166.0847 (8)	1	7.41E-03	3.16E-01	-0.34	6.26E-03	0.73	1.75E-02	0.61	3.71E-03	0.71
165.0795@1.1509142	165.0795	1.15	POS	RP				9.87E-02	5.83E-01	-0.17	1.62E-02	0.66	4.81E-02	0.52	5.85E-02	0.52
165.1516@0.5802214	165.1516	0.58	POS	hilic				9.64E-01	7.06E-01	-0.12	6.40E-01	-0.14	7.72E-01	0.08	6.50E-01	-0.15
165.9512@7.3180904	165.9512	7.32	POS	hilic				3.24E-01	2.52E-02	-0.77	2.56E-01	-0.34	4.05E-01	-0.23	4.96E-02	-0.45
166.005@15.303636	166.0050	15.30	POS	RP				7.46E-01	3.99E-01	-0.27	9.73E-01	-0.01	3.65E-01	-0.29	1.17E-01	-0.42
166.0053@7.8736677	166.0053	7.87	POS	hilic				7.98E-01	2.52E-01	0.36	6.01E-01	0.16	3.54E-01	0.27	1.56E-01	0.41
166.0264@10.9096	166.0264	10.91	POS	RP				9.08E-01	7.81E-01	0.07	8.54E-01	-0.06	7.31E-01	-0.12	3.78E-01	0.23
166.0488@0.889652	166.0488	0.89	NEG	hilic				2.80E-01	7.05E-01	0.11	4.88E-01	-0.18	2.77E-01	-0.25	2.35E-02	-0.59
166.0489@0.86557806	166.0489	0.87	NEG	hilic				2.93E-02	1.78E-03	0.81	6.87E-01	0.10	5.21E-01	0.15	3.40E-01	-0.23
166.049@0.89484197	166.0490	0.89	NEG	hilic				7.82E-01	3.91E-01	0.24	9.71E-01	0.01	9.90E-01	0.00	3.93E-01	-0.20
166.0624@8.872859	166.0624	8.87	POS	RP				8.94E-01	6.61E-01	0.14	8.35E-01	-0.06	4.01E-01	0.26	8.00E-01	-0.08
166.0625@4.316701	166.0625	4.32	NEG	RP				1.85E-01	2.22E-01	-0.35	6.84E-03	-0.65	1.13E-02	-0.63	1.21E-01	-0.35
166.0627@0.7214524	166.0627	0.72	NEG	hilic				3.11E-01	4.07E-01	-0.23	8.66E-01	-0.04	2.11E-02	-0.49	5.83E-01	0.13
166.0629@4.251595	166.0629	4.25	NEG	RP				4.99E-01	1.08E-01	-0.42	1.35E-01	-0.34	3.52E-02	-0.44	4.67E-01	-0.18
167.0695@6.3098884	167.0695	6.31	POS	hilic				6.97E-01	6.91E-01	-0.11	2.30E-01	-0.32	1.29E-01	-0.39	7.97E-01	-0.07
167.1164@0.6766347	167.1164	0.68	POS	hilic				1.78E-01	6.74E-01	-0.11	9.95E-02	-0.43	4.04E-01	0.26	6.78E-02	-0.54
167.131@6.35973	167.1310	6.36	POS	RP				1.57E-01	2.66E-02	-0.61	8.56E-01	-0.05	4.74E-01	0.20	9.25E-02	-0.52
167.8425@3.605553	167.8425	3.61	NEG	hilic				7.66E-01	3.60E-01	0.28	8.53E-01	0.05	7.85E-01	-0.08	2.43E-01	0.33
167.9766@15.338996	167.9766	15.34	POS	RP				7.16E-02	5.04E-02	0.51	4.51E-01	0.21	3.87E-03	0.94	3.30E-02	0.64
168.028@4.821554	168.0280	4.82	NEG	hilic				1.50E-01	3.19E-02	0.57	2.31E-01	-0.36	4.65E-01	0.25	2.88E-01	0.29

Supplementary table 2: Metabolomics analysis results

								ANOVA	Anorexia vs control		overweight vs control		obese vs control		athletes vs control	
Compound	Mass	Retention Time	Ionization mode	column	Compound_ID	MSMS_spectra	ID_level	P-FDR	P	Estimate	P	Estimate	P	Estimate	P	Estimate
168.0507@6.0849175	168.0507	6.08	NEG	hilic				5.56E-01	9.88E-02	-0.48	1.29E-01	-0.40	1.62E-01	-0.38	6.27E-01	-0.13
168.9355@13.25054	168.9355	13.25	NEG	RP				4.66E-01	1.96E-01	0.35	1.94E-01	0.37	3.50E-01	-0.26	7.81E-01	0.09
168.9785@5.280482	168.9785	5.28	POS	hilic				7.66E-01	3.08E-01	0.39	1.03E-01	0.40	2.13E-01	0.34	1.98E-01	0.34
169.0253@5.473402	169.0253	5.47	POS	hilic				3.32E-02	8.21E-01	0.06	3.26E-01	0.22	1.32E-03	0.76	3.78E-01	-0.18
169.0853@6.774595	169.0853	6.77	POS	hilic	3-Methyl-histidine	10 eV: 170.0927 (100), 109.0766 (25)	2	1.53E-01	3.03E-01	-0.33	4.26E-01	0.21	7.16E-02	0.51	6.27E-02	0.51
169.0854@6.170744	169.0854	6.17	POS	hilic	1-Methyl-histidine	10 eV: 124.0869 (100), 170.0935 (64), 109.0762 (18)	2	1.76E-02	7.17E-02	-0.55	1.45E-01	0.38	5.75E-01	0.14	3.11E-02	0.69
169.8942@0.93217087	169.8942	0.93	NEG	hilic				7.70E-01	3.08E-01	0.28	5.25E-01	0.14	5.94E-01	-0.13	4.60E-01	0.22
170.0686@5.5382533	170.0686	5.54	POS	hilic				1.38E-01	4.56E-01	-0.14	1.94E-01	0.31	2.16E-01	0.30	1.04E-02	0.62
171.0689@5.61557	171.0689	5.62	POS	RP				1.77E-02	6.77E-01	0.11	5.12E-02	-0.39	5.26E-03	-0.55	1.85E-01	0.37
171.1264@9.930923	171.1264	9.93	POS	RP				4.14E-01	2.40E-01	-0.21	5.64E-01	-0.11	1.42E-01	-0.24	4.18E-01	0.20
171.1266@10.035733	171.1266	10.04	POS	RP				7.29E-01	1.40E-01	-0.30	7.42E-01	0.08	6.62E-01	0.10	9.93E-01	0.00
171.1626@8.204835	171.1626	8.20	POS	RP				1.08E-01	8.06E-02	-0.51	2.85E-01	0.32	1.45E-01	-0.42	1.10E-01	-0.40
171.8407@15.316304	171.8407	15.32	NEG	RP				6.30E-01	3.21E-01	-0.31	2.45E-01	-0.35	4.94E-01	0.19	5.68E-01	-0.18
171.9698@1.0500425	171.9698	1.05	POS	RP				9.75E-01	8.63E-01	-0.05	6.49E-01	0.13	7.29E-01	0.10	5.51E-01	0.17
172.0225@5.600395	172.0225	5.60	POS	hilic				2.17E-01	1.91E-01	0.40	4.79E-01	0.18	5.77E-02	-0.39	8.64E-01	0.04
172.0346@0.9889255	172.0346	0.99	NEG	hilic				9.49E-02	2.98E-01	-0.31	1.04E-01	0.46	1.14E-01	0.48	5.01E-01	-0.16
172.0655@6.07898	172.0655	6.08	NEG	hilic				3.78E-01	9.19E-01	0.03	5.50E-02	-0.53	9.35E-02	-0.42	1.24E-01	-0.43
172.0847@5.14789	172.0847	5.15	POS	hilic				2.54E-02	2.40E-01	0.46	1.04E-01	0.40	4.40E-05	1.03	2.93E-04	0.87
172.085@3.1735208	172.0850	3.17	POS	hilic				1.71E-02	7.41E-02	0.70	1.84E-01	0.31	1.58E-03	0.89	7.64E-06	1.02
172.121@5.2167425	172.1210	5.22	POS	hilic				3.69E-01	2.95E-01	0.28	3.53E-01	-0.17	2.08E-01	0.28	2.48E-01	0.24
172.1252@0.4209822	172.1252	0.42	POS	hilic				6.37E-01	1.88E-01	-0.33	6.37E-01	-0.12	8.16E-01	-0.06	4.48E-01	0.21
172.1579@0.9195846	172.1579	0.92	POS	hilic				5.57E-01	7.57E-01	0.08	2.49E-01	0.29	5.41E-01	-0.15	2.09E-01	0.37
172.9542@15.194367	172.9542	15.19	POS	RP				7.12E-01	1.16E-01	0.40	6.02E-01	0.14	6.23E-01	0.12	2.15E-01	0.41
173.0297@6.1250453	173.0297	6.13	NEG	hilic				8.89E-01	3.64E-01	-0.25	2.44E-01	-0.33	7.25E-01	-0.12	7.35E-01	-0.09
173.1416@0.7632178	173.1416	0.76	POS	hilic				8.77E-01	3.75E-01	0.28	6.64E-01	0.13	8.14E-01	-0.07	3.89E-01	0.23
173.1418@0.7620261	173.1418	0.76	POS	hilic				9.95E-01	6.35E-01	0.15	8.14E-01	0.08	8.53E-01	0.05	9.08E-01	0.03
173.8379@15.315518	173.8379	15.32	NEG	RP				1.32E-01	4.79E-01	0.23	1.99E-01	0.34	1.27E-02	0.57	2.30E-01	-0.37
173.9503@15.342137	173.9503	15.34	POS	RP				7.59E-01	9.54E-01	-0.01	8.51E-01	0.05	7.08E-01	0.10	1.49E-01	0.37
173.9942@1.4150689	173.9942	1.42	POS	hilic				1.53E-02	5.45E-01	0.17	9.30E-02	0.44	7.09E-05	1.11	2.66E-01	0.30
173.9981@0.51808566	173.9981	0.52	NEG	hilic				8.70E-01	7.21E-01	-0.12	1.31E-01	-0.37	4.56E-01	-0.25	4.41E-01	-0.20
173.9989@2.2275658	173.9989	2.23	NEG	RP				6.88E-01	5.32E-01	0.21	1.90E-01	-0.32	4.32E-01	-0.26	5.80E-01	-0.15
173.9989@2.2276413	173.9989	2.23	NEG	RP				6.81E-01	6.13E-01	0.17	1.40E-01	-0.35	3.82E-01	-0.29	4.93E-01	-0.19
174.0061@1.0734944	174.0061	1.07	NEG	hilic				3.94E-02	9.56E-02	-0.43	3.41E-01	0.25	9.53E-03	0.60	4.24E-01	-0.18
174.0272@0.8604317	174.0272	0.86	POS	hilic				2.04E-01	4.87E-02	-0.42	7.23E-01	0.09	9.86E-01	0.00	1.60E-02	-0.47
174.0638@3.80197	174.0638	3.80	POS	hilic				6.53E-03	9.15E-02	-0.53	4.96E-01	-0.16	2.38E-02	-0.55	2.64E-02	0.61
174.1005@5.720793	174.1005	5.72	POS	hilic				4.59E-03	6.65E-01	0.13	3.33E-01	-0.27	7.27E-04	-0.88	1.47E-01	0.42
174.101@3.5132551	174.1010	3.51	POS	hilic				7.58E-04	9.61E-03	0.77	5.49E-01	-0.06	4.72E-03	0.68	7.73E-05	0.78
174.1119@6.869654	174.1119	6.87	POS	hilic	Arginine	10 eV: 175.1195 (100), 116.0710 (70), 70.0647 (65)	2	1.71E-01	7.55E-03	-0.89	4.76E-01	-0.18	7.14E-02	-0.48	3.11E-01	-0.30
174.1371@6.5887427	174.1371	6.59	POS	hilic				9.30E-01	6.04E-01	0.17	5.92E-01	0.16	8.75E-01	-0.05	4.39E-01	0.24
174.1402@10.617984	174.1402	10.62	POS	RP				8.08E-01	2.27E-01	-0.33	2.49E-01	-0.34	4.78E-01	-0.20	2.30E-01	-0.38
174.1406@10.628785	174.1406	10.63	POS	RP				8.35E-01	1.91E-01	-0.32	5.03E-01	-0.19	2.15E-01	-0.34	3.02E-01	-0.29
174.9779@15.254664	174.9779	15.25	POS	RP				8.71E-01	8.63E-01	0.05	4.32E-01	-0.24	3.79E-01	-0.24	8.44E-01	0.05
174.9934@0.6101034	174.9934	0.61	NEG	hilic				4.48E-02	9.56E-03	0.67	2.10E-01	0.22	1.16E-01	0.29	8.69E-01	-0.02
175.0334@5.4733176	175.0334	5.47	POS	hilic				9.59E-02	8.79E-01	0.04	6.27E-02	0.42	1.02E-02	0.64	5.94E-01	-0.11
175.0635@4.8587937	175.0635	4.86	POS	RP	3-Indoleacetic acid	10 eV: 130.0647 (100), 176.0682 (13)	1	7.29E-01	6.98E-01	0.13	3.38E-01	-0.24	5.58E-01	-0.19	4.69E-01	0.23
175.0954@6.319921	175.0954	6.32	NEG	hilic				1.92E-01	2.15E-01	0.43	4.44E-01	0.22	9.04E-01	-0.03	6.45E-03	0.76
175.0958@6.270665	175.0958	6.27	POS	hilic	Citrulline	10 eV: 159.0765 (100), 70.0644 (27), 113.0703 (25)	2	1.73E-01	2.46E-01	0.40	5.89E-01	0.16	5.03E-01	-0.19	1.26E-02	0.68
175.0967@5.5447845	175.0967	5.54	POS	hilic				6.29E-01	4.68E-01	0.22	7.91E-01	0.06	1.05E-01	0.47	1.67E-01	0.38
175.1224@3.9469788	175.1224	3.95	POS	hilic				7.99E-02	3.19E-01	0.26	1.70E-02	0.59	1.54E-02	0.75	9.17E-01	-0.02
175.132@3.679623	175.1320	3.68	POS	hilic				6.68E-01	2.19E-01	-0.33	1.31E-01	-0.50	7.94E-01	-0.07	4.44E-01	-0.23
175.9633@7.9218473	175.9633	7.92	NEG	hilic				4.46E-01	5.23E-02	0.63	2.03E-01	0.38	7.07E-02	0.56	1.36E-01	0.45
175.9637@15.289888	175.9637	15.29	NEG	RP				7.07E-01	4.92E-01	-0.22	1.62E-01	-0.43	2.45E-01	-0.31	1.46E-01	-0.47
176.0173@6.12783	176.0173	6.13	POS	hilic				2.24E-01	1.02E-01	-0.44	5.81E-01	-0.16	4.33E-02	-0.59	4.45E-01	0.20
176.0475@7.1802077	176.0475	7.18	POS	RP				8.58E-01	8.24E-01	0.07	9.38E-01	0.02	2.84E-01	0.33	4.44E-01	0.28
176.0491@0.9882306	176.0491	0.99	NEG	hilic				4.28E-01	9.46E-01	0.01	8.08E-02	0.45	9.99E-02	0.41	1.87E-01	0.31
176.0951@1.2544872	176.0951	1.25	POS	RP				5.83E-03	5.86E-03	0.77	1.23E-01	0.30	3.06E-01	0.21	1.17E-02	-0.24
176.0956@0.57723993	176.0956	0.58	POS	hilic	Cotinine	10 eV: 177.1030 (100), 98.0601 (8), 80.0482 (7)	2	4.50E-02	2.39E-02	0.64	6.11E-01	0.11	9.75E-01	0.01	3.42E-02	-0.30
177.0162@5.7478476	177.0162	5.75	NEG	hilic				1.13E-01	7.94E-01	0.08	9.98E-01	0.00	8.85E-01	0.04	3.12E-03	0.81
177.0787@4.6632566	177.0787	4.66	POS	hilic				2.44E-01	3.77E-01	0.26	9.26E-01	-0.02	2.45E-01	-0.28	6.79E-02	-0.48
177.0789@3.5886335	177.0789	3.59	POS	hilic				3.77E-01	3.89E-01	-0.33	9.28E-01	-0.03	3.81E-01	-0.21	1.89E-02	-0.68
178.048@8.951854	178.0480	8.95	POS	RP				8.77E-01	8.42E-01	-0.06	3.22E-01	0.25	6.68E-01	-0.14	9.30E-01	0.03
178.9643@12.382802	178.9643	12.38	NEG	RP				6.18E-01	1.51E-01	0.43	9.27E-02	0.54	2.24E-01	0.39	2.62E-01	0.30
179.0423@4.729779	179.0423	4.73	POS	hilic				7.11E-01	6.91E-01	0.10	2.34E-01	-0.34	6.29E-01	0.17	9.78E-01	0.01
179.0579@1.0168455	179.0579	1.02	NEG	hilic				4.71E-01	6.12E-01	-0.17	7.30E-01	0.09	2.16E-01	-0.37	2.63E-01	0.33
179.0583@3.1593685	179.0583	3.16	NEG	RP				5.12E-01	3.68E-01	-0.29	8.14E-01	-0.06	1.43E-01	-0.46	5.44E-01	0.18
179.0584@3.1457353	179.0584	3.15	POS	RP	Hippuric acid	10 eV: 105.0335 (100), 180.0669 (8)	1	4.92E-01	3.03E-01	-0.34	9.61E-01	0.01	1.63E-01	-0.43	5.32E-01	0.18
179.0585@0.99687135	179.0585	1.00	POS	hilic				6.62E-01	2.02E-01	-0.36	6.16E-01	-0.13	1.91E-01	-0.37	8.08E-01	0.07
179.0709@3.6586254	179.0709	3.66	NEG	hilic				1.37E-04	4.40E-02	0.48	3.27E-01	-0.05	3.27E-01	-0.05	8.83E-04	0.80
179.0792@5.5410457	179.0792	5.54	POS	hilic				1.57E-01	2.30E-02	-0.67	3.03E-01	0.28	9.66E-01	-0.01	5.37E-01	-0.17
179.0798@5.816551	179.0798	5.82	POS	hilic				3.77E-01	8.46E-02	0.51	6.23E-01	0.14	3.33E-01	-0.24	7.97E-01	-0.08
179.08@6.204264	179.0800	6.20	POS	hilic				3.96E-01	9.05E-02	0.52	4.21E-02	0.61	1.59E-01	0.40	5.35E-02	0.57
179.1288@7.3524694	179.1288	7.35	POS	RP				2.93E-01	2.52E-01	-0.36	5.11E-01	-0.17	2.36E-01	0.40	3.26E-01	0.28
179.872@15.263101	179.8720	15.26	NEG	RP				7.06E-02	3.46E-01	-0.34	3.99E-02	-0.62	1.73E-01	0.38	8.13E-01	0.06

Supplementary table 2: Metabolomics analysis results

Compound	Mass	Retention Time	Ionization mode	column	Compound_ID	MSMS_spectra	ID_level	ANOVA	Anorexia vs control		overweight vs control		obese vs control		athletes vs control	
								P-FDR	P	Estimate	P	Estimate	P	Estimate	P	Estimate
179.8873@0.55454046	179.8873	0.55	NEG	hilic				9.35E-01	8.53E-01	0.06	5.13E-01	0.19	5.74E-01	0.16	7.10E-01	-0.10
179.9851@15.435857	179.9851	15.44	NEG	RP				2.13E-01	6.34E-03	0.86	7.37E-02	0.52	1.01E-01	0.45	4.51E-02	0.54
180.0188@0.5182992	180.0188	0.52	POS	hilic				4.46E-01	5.47E-02	-0.59	8.34E-01	-0.06	5.28E-01	-0.20	6.86E-02	-0.49
180.0458@1.5834101	180.0458	1.58	POS	hilic				4.41E-01	6.85E-01	0.14	2.98E-01	0.26	1.49E-01	-0.38	4.15E-01	0.28
180.0631@4.8985496	180.0631	4.90	NEG	hilic				5.71E-04	1.69E-01	-0.24	2.95E-04	0.84	1.43E-02	0.75	6.52E-01	-0.09
180.0631@5.209071	180.0631	5.21	NEG	hilic				2.56E-01	9.24E-02	-0.46	1.11E-01	0.43	7.05E-01	0.10	8.62E-01	0.05
180.0643@0.60995615	180.0643	0.61	NEG	hilic				1.82E-02	3.01E-02	0.74	6.15E-01	-0.13	9.69E-01	-0.01	5.14E-02	-0.45
180.0643@2.7958977	180.0643	2.80	NEG	RP				9.60E-03	1.69E-02	0.76	7.26E-01	0.07	1.56E-01	-0.23	6.70E-01	-0.08
180.0646@2.2599664	180.0646	2.26	POS	RP	Theobromine	10 eV: 181.0725 (100), 138.0675 (25), 95.9724 (5)	2	3.53E-01	8.09E-01	-0.08	9.84E-01	0.01	3.26E-01	0.26	9.61E-02	-0.54
180.0646@2.7774756	180.0646	2.78	POS	RP	Theophylline	10 eV: 181.0730 (100), 124.0500 (45), 95.9733 (40)	2	2.80E-02	2.38E-02	0.81	6.13E-01	0.14	5.92E-01	0.13	1.31E-01	-0.38
180.0646@1.7410524	180.0646	1.74	POS	RP				3.62E-01	8.97E-01	-0.04	9.40E-01	0.02	2.71E-01	0.29	1.17E-01	-0.51
180.0647@2.6598654	180.0647	2.66	POS	RP				7.57E-03	1.60E-02	0.84	8.21E-01	-0.06	2.62E-01	0.28	5.57E-02	-0.49
180.0652@0.62902915	180.0652	0.63	POS	hilic				5.37E-02	1.18E-01	0.54	5.67E-01	-0.15	7.96E-01	0.07	2.51E-02	-0.58
180.0761@1.4218104	180.0761	1.42	POS	hilic				7.21E-03	7.66E-02	0.43	2.06E-02	0.64	1.92E-05	1.27	1.46E-01	0.46
180.0783@8.131284	180.0783	8.13	POS	RP				6.82E-01	3.29E-01	0.29	8.98E-01	-0.04	7.44E-01	0.11	1.25E-01	0.45
180.0789@7.463869	180.0789	7.46	POS	RP				7.10E-01	2.25E-01	-0.39	6.19E-01	0.14	9.18E-01	-0.03	8.95E-01	-0.03
180.0789@9.534136	180.0789	9.53	POS	RP				9.51E-01	6.94E-01	-0.12	6.60E-01	-0.13	8.07E-01	0.07	7.06E-01	0.12
181.0502@0.46710506	181.0502	0.47	POS	hilic				8.04E-01	2.35E-01	-0.38	8.81E-01	-0.04	6.16E-01	-0.15	2.20E-01	-0.33
181.0505@3.485131	181.0505	3.49	POS	hilic				2.83E-02	4.11E-01	0.23	6.67E-01	0.10	9.14E-01	-0.02	7.26E-04	0.90
181.0726@5.0148783	181.0726	5.01	POS	hilic	Tyrosine	10 eV: 136.0758 (100), 165.0542 (55), 147.0439 (28)	2	4.34E-01	8.11E-01	-0.05	1.16E-01	0.38	1.01E-01	0.40	5.49E-01	0.12
181.0733@1.0955688	181.0733	1.10	NEG	RP				1.17E-01	1.16E-01	-0.52	2.51E-01	0.33	5.67E-02	0.45	6.43E-01	0.12
181.0739@1.086461	181.0739	1.09	POS	RP				2.13E-01	3.60E-01	-0.29	1.62E-01	0.40	2.28E-02	0.56	4.82E-01	0.20
181.8689@15.264459	181.8689	15.26	NEG	RP				7.40E-01	8.09E-01	-0.07	4.36E-01	-0.23	3.77E-01	0.27	6.22E-01	0.15
181.8851@0.55731523	181.8851	0.56	NEG	hilic				9.52E-01	9.20E-01	0.03	9.94E-01	0.00	6.63E-01	0.13	5.18E-01	-0.18
181.9067@15.286648	181.9067	15.29	NEG	RP				2.44E-01	4.44E-01	0.26	3.36E-02	0.66	6.67E-03	0.72	9.19E-02	0.44
181.9791@15.279082	181.9791	15.28	POS	RP				3.41E-01	1.41E-01	0.45	1.87E-02	0.70	1.51E-01	0.39	6.73E-02	0.55
181.9802@5.4112535	181.9802	5.41	NEG	hilic				3.84E-01	1.20E-01	-0.45	2.20E-02	-0.66	8.82E-02	-0.52	4.25E-01	-0.23
181.9803@4.4906745	181.9803	4.49	NEG	hilic				6.42E-01	1.69E-01	0.45	2.21E-01	0.37	2.88E-01	0.28	9.38E-01	-0.03
181.9805@4.5935683	181.9805	4.59	NEG	hilic				4.85E-01	3.48E-01	0.28	3.60E-01	0.21	1.99E-01	-0.26	8.47E-01	-0.04
181.9816@15.405258	181.9816	15.41	NEG	RP				5.10E-01	5.00E-01	0.22	5.19E-01	-0.17	7.59E-01	-0.08	1.41E-01	0.44
181.9828@15.351211	181.9828	15.35	NEG	RP				9.15E-02	4.75E-02	-0.52	1.39E-02	-0.65	4.18E-02	-0.59	8.16E-01	0.07
182.0435@2.0526273	182.0435	2.05	NEG	hilic				2.22E-03	4.76E-03	0.89	9.67E-01	-0.01	6.73E-01	0.10	2.89E-02	-0.49
182.0435@2.534063	182.0435	2.53	NEG	hilic				9.61E-01	6.84E-01	0.13	6.53E-01	0.11	5.16E-01	0.16	8.35E-01	-0.05
182.0576@1.1447266	182.0576	1.14	NEG	hilic				1.59E-03	1.32E-03	-0.96	2.27E-01	0.34	5.12E-01	0.15	2.53E-01	0.33
182.0579@2.6289093	182.0579	2.63	NEG	RP				1.18E-02	3.41E-04	-1.11	7.29E-01	-0.09	1.11E-01	-0.36	9.84E-01	0.01
182.073@7.7832932	182.0730	7.78	POS	RP				9.97E-01	9.04E-01	0.03	8.63E-01	-0.05	8.12E-01	0.08	9.15E-01	0.03
182.1057@0.7843577	182.1057	0.78	POS	hilic				5.84E-02	7.28E-01	0.10	4.05E-01	-0.24	6.90E-04	-0.86	5.79E-01	-0.15
182.1668@9.444373	182.1668	9.44	POS	RP				6.68E-04	3.04E-03	-0.89	1.03E-01	0.40	6.75E-01	0.09	4.75E-02	0.62
183.0391@2.153299	183.0391	2.15	NEG	hilic				2.40E-01	4.04E-02	0.57	2.99E-01	-0.32	6.04E-01	0.18	5.26E-01	0.17
183.0527@0.86584127	183.0527	0.87	NEG	hilic				2.12E-02	7.49E-03	0.92	4.59E-01	-0.16	6.92E-01	-0.10	5.34E-01	0.16
183.0563@0.85337055	183.0563	0.85	POS	hilic				6.95E-04	3.84E-03	0.88	1.58E-02	-0.40	9.54E-01	-0.01	8.81E-01	0.03
183.0656@0.8999699	183.0656	0.90	POS	hilic				1.45E-01	1.68E-01	-0.38	7.44E-02	0.48	2.31E-01	0.34	7.75E-01	-0.09
183.0658@0.64410263	183.0658	0.64	POS	hilic				6.41E-01	9.39E-01	-0.02	1.40E-01	0.42	8.32E-01	0.05	2.75E-01	0.38
183.066@1.2893248	183.0660	1.29	POS	hilic				1.80E-02	6.53E-02	0.50	6.42E-02	-0.53	1.37E-01	-0.38	2.65E-01	0.37
183.0661@1.153385	183.0661	1.15	POS	hilic				2.10E-02	1.08E-01	0.47	3.70E-02	-0.59	4.50E-02	-0.51	6.91E-01	0.13
183.0661@10.171174	183.0661	10.17	POS	RP				1.97E-01	5.48E-02	0.54	4.72E-01	-0.21	5.52E-01	-0.18	2.37E-01	0.39
183.0661@10.489666	183.0661	10.49	POS	RP				1.72E-01	3.13E-01	0.29	7.88E-02	-0.53	7.15E-01	0.09	1.77E-01	-0.38
183.0662@10.002155	183.0662	10.00	POS	RP				1.69E-02	7.62E-02	0.49	4.34E-02	-0.60	3.26E-01	-0.29	2.16E-01	0.37
183.0663@10.1237335	183.0663	10.12	POS	RP				4.68E-01	3.64E-01	0.31	6.40E-01	-0.13	5.84E-01	-0.16	1.81E-01	0.41
183.0663@10.511725	183.0663	10.51	POS	RP				1.68E-02	2.12E-02	0.62	4.43E-02	-0.56	8.53E-01	0.04	2.43E-01	0.38
183.0663@9.848332	183.0663	9.85	POS	RP				4.21E-02	6.34E-01	0.14	3.83E-02	-0.65	1.77E-02	-0.64	6.31E-01	0.15
183.1622@0.71705544	183.1622	0.72	POS	hilic				9.70E-01	7.39E-01	-0.10	4.73E-01	-0.20	9.61E-01	0.01	9.28E-01	-0.02
183.1624@1.2764487	183.1624	1.28	POS	RP				9.65E-01	6.21E-01	-0.16	8.81E-01	0.04	7.31E-01	-0.11	7.83E-01	0.09
183.1625@0.7940339	183.1625	0.79	POS	hilic				9.97E-01	8.10E-01	-0.07	7.83E-01	-0.08	9.31E-01	0.03	8.61E-01	-0.06
183.1625@3.7361457	183.1625	3.74	POS	RP				9.81E-01	7.70E-01	-0.08	7.38E-01	-0.08	9.48E-01	0.02	7.59E-01	0.08
183.1626@0.7220631	183.1626	0.72	POS	hilic				8.53E-01	8.27E-01	-0.05	2.72E-01	0.30	8.17E-01	0.05	6.94E-01	0.12
183.1626@1.9253331	183.1626	1.93	POS	RP				8.96E-01	8.32E-01	-0.06	8.38E-01	-0.06	9.87E-01	-0.01	3.74E-01	0.28
183.8667@15.264759	183.8667	15.26	NEG	RP				7.22E-01	3.33E-01	-0.32	8.74E-01	-0.05	4.56E-01	0.24	6.17E-01	-0.16
183.9779@1.0500286	183.9779	1.05	POS	RP				9.29E-01	7.44E-01	-0.10	4.32E-01	0.22	9.99E-01	0.00	8.91E-01	-0.04
183.9788@15.255793	183.9788	15.26	POS	RP				8.25E-01	9.04E-01	0.04	9.05E-01	0.03	6.49E-01	-0.12	2.64E-01	0.33
184.0228@2.4897318	184.0228	2.49	NEG	hilic				1.59E-01	4.03E-02	0.59	2.19E-01	-0.31	7.32E-01	0.11	3.33E-01	0.24
184.0252@6.0241966	184.0252	6.02	POS	hilic				8.31E-01	7.73E-01	0.09	9.02E-01	0.04	2.44E-01	-0.30	7.41E-01	0.11
184.0348@1.072421	184.0348	1.07	NEG	hilic				1.30E-02	3.31E-02	-0.59	1.72E-01	0.39	4.79E-02	0.61	6.22E-01	-0.12
184.1097@9.270881	184.1097	9.27	POS	RP				5.75E-01	6.18E-01	-0.14	8.24E-01	0.06	4.76E-01	0.23	1.06E-01	0.48
184.1196@2.1097457	184.1196	2.11	POS	RP				8.31E-01	3.68E-01	0.24	5.72E-01	0.14	6.32E-01	0.12	5.93E-01	-0.13
184.1212@2.4557095	184.1212	2.46	POS	RP				8.62E-01	8.33E-01	0.07	3.30E-01	0.27	6.99E-01	0.11	6.25E-01	-0.15
184.1218@0.6607348	184.1218	0.66	POS	hilic				6.80E-01	6.14E-01	0.16	5.90E-01	0.15	6.08E-01	0.14	2.48E-01	-0.34
184.8554@0.5802703	184.8554	0.58	NEG	hilic				9.94E-01	9.92E-01	0.00	8.12E-01	0.07	7.89E-01	0.08	5.85E-01	0.14
185.1051@3.9019828	185.1051	3.90	POS	hilic				1.19E-02	4.54E-01	-0.24	1.97E-02	0.62	2.93E-02	0.61	2.19E-01	-0.36
186.0386@1.8590093	186.0386	1.86	NEG	hilic				2.37E-01	2.08E-01	0.35	5.81E-02	-0.50	9.40E-01	0.03	5.06E-01	0.17
186.0387@2.6953115	186.0387	2.70	NEG	hilic				2.99E-01	2.80E-01	0.32	4.45E-02	-0.50	9.42E-01	-0.02	7.86E-01	0.07
186.0502@2.3806665	186.0502	2.38	POS	hilic				1.25E-01	1.44E-01	-0.25	6.30E-02	0.46	2.96E-01	0.24	1.00E-01	0.42
186.1257@7.6509814	186.1257	7.65	NEG	RP				1.60E-01	2.25E-02	-0.52	3.26E-01	-0.24	5.47E-03	-0.61	9.40E-01	-0.02

Supplementary table 2: Metabolomics analysis results

Compound	Mass	Retention Time	Ionization mode	column	Compound_ID	MSMS_spectra	ID_level	ANOVA P-FDR	Anorexia vs control P	Anorexia vs control Estimate	overweight vs control P	overweight vs control Estimate	obese vs control P	obese vs control Estimate	athletes vs control P	athletes vs control Estimate
187.0009@4.4154444	187.0009	4.42	NEG	hilic				7.09E-01	5.38E-01	0.18	9.52E-01	-0.01	1.49E-01	-0.37	8.42E-01	0.06
187.0453@5.7476244	187.0453	5.75	NEG	hilic				4.89E-02	7.68E-01	0.08	6.61E-01	-0.12	8.75E-01	-0.05	2.75E-03	0.87
187.0634@1.4137594	187.0634	1.41	POS	hilic		10 eV: 188.0705 (100), 146.0601 (45). 118.0652 (13)		3.37E-01	5.53E-02	-0.61	2.14E-01	-0.32	1.91E-02	-0.67	4.48E-01	-0.24
187.0637@2.038879	187.0637	2.04	POS	RP				5.52E-01	1.50E-01	-0.40	6.80E-01	0.13	3.57E-01	0.23	6.11E-01	-0.16
187.0637@3.9582503	187.0637	3.96	POS	hilic				5.86E-01	3.37E-01	-0.28	2.71E-01	0.34	3.51E-01	0.24	8.56E-01	0.06
187.0639@2.4425468	187.0639	2.44	POS	RP				5.24E-01	3.47E-01	-0.26	3.10E-01	0.33	3.10E-01	0.26	3.42E-01	0.30
187.1208@3.8929832	187.1208	3.89	POS	hilic				4.54E-01	5.06E-01	0.19	6.87E-01	0.11	5.14E-01	0.22	1.35E-01	-0.43
187.1212@10.027937	187.1212	10.03	POS	RP				7.05E-01	9.37E-01	0.01	2.32E-01	0.27	1.98E-01	0.29	6.88E-01	0.07
187.1214@10.043389	187.1214	10.04	POS	RP				7.24E-01	6.88E-02	-0.33	9.80E-01	-0.01	8.91E-01	0.03	5.33E-01	-0.13
187.132@3.486	187.1320	3.49	POS	hilic				9.27E-04	2.19E-02	0.55	2.01E-01	0.18	7.61E-01	0.02	3.17E-04	0.87
187.1685@6.0785604	187.1685	6.08	POS	hilic				6.41E-01	9.40E-01	-0.03	1.77E-01	0.34	2.78E-01	-0.27	9.64E-01	0.01
188.0143@0.506393	188.0143	0.51	NEG	hilic				6.93E-02	5.78E-02	0.76	3.61E-01	0.19	2.49E-01	-0.33	2.45E-01	0.28
188.0147@3.4464011	188.0147	3.45	NEG	RP	p-Cresol sulfate	10 eV: 107.0503 (100), 187.0074 (50), 79.9581 (20)	2	4.78E-02	5.20E-02	0.77	6.28E-01	0.10	1.72E-01	-0.40	3.64E-01	0.22
188.0208@0.8819211	188.0208	0.88	NEG	hilic				9.61E-01	9.67E-01	-0.01	7.07E-01	-0.11	5.72E-01	0.16	7.94E-01	0.07
188.0451@3.6299658	188.0451	3.63	NEG	hilic				3.68E-02	1.54E-01	0.52	4.76E-01	-0.20	4.79E-02	-0.47	6.82E-02	0.46
188.0491@1.4045727	188.0491	1.40	NEG	hilic				3.50E-02	3.18E-02	0.55	1.41E-01	0.39	5.17E-04	1.10	6.77E-02	0.57
188.0499@3.181942	188.0499	3.18	POS	hilic				6.96E-01	7.58E-01	-0.07	3.13E-01	0.27	4.41E-01	0.19	5.85E-01	-0.13
188.1044@5.607509	188.1044	5.61	NEG	RP				9.78E-01	5.96E-01	-0.12	8.02E-01	-0.06	9.28E-01	0.02	5.45E-01	-0.15
188.1157@5.559622	188.1157	5.56	POS	hilic				6.00E-01	2.23E-01	0.38	3.66E-01	0.27	9.16E-01	0.03	1.12E-01	0.52
188.1162@5.368249	188.1162	5.37	POS	hilic				1.39E-07	2.98E-02	0.43			1.11E-03	0.84	4.58E-08	1.24
188.1274@6.7451797	188.1274	6.75	POS	hilic				4.21E-02	9.48E-01	0.02	1.22E-01	0.44	3.65E-03	0.83	6.30E-03	0.79
188.1409@8.151123	188.1409	8.15	NEG	RP				4.28E-01	1.08E-01	-0.44	8.43E-01	0.06	1.64E-01	-0.34	5.71E-01	0.18
188.1412@7.79607	188.1412	7.80	NEG	RP				4.64E-01	1.26E-01	-0.38	3.30E-01	0.27	9.57E-01	-0.01	9.63E-01	0.01
188.1523@6.669867	188.1523	6.67	POS	hilic				2.40E-01	8.58E-02	-0.53	3.00E-01	0.26	6.61E-01	0.12	3.69E-01	0.28
188.8679@0.9414784	188.8679	0.94	NEG	hilic				9.56E-01	7.76E-01	0.08	9.97E-01	0.00	4.01E-01	0.23	8.43E-01	0.06
188.8706@15.33468	188.8706	15.33	NEG	RP				1.98E-01	2.70E-01	0.34	1.82E-01	-0.36	2.01E-01	-0.38	1.17E-01	-0.47
188.9485@6.078283	188.9485	6.08	NEG	hilic				4.25E-01	2.93E-01	0.32	3.12E-01	0.25	8.36E-01	-0.04	5.02E-02	0.52
188.9487@6.2253523	188.9487	6.23	NEG	hilic				5.38E-01	5.52E-02	0.55	2.69E-01	0.32	6.45E-01	0.13	1.00E-01	0.46
188.9489@6.2333217	188.9489	6.23	NEG	hilic				6.83E-01	5.77E-01	0.15	3.43E-01	-0.19	3.30E-01	0.23	4.48E-01	0.21
189.079@5.615351	189.0790	5.62	POS	RP	Indole-3-propanoic acid	10 eV: 130.0651 (100), 190.0866 (25), 172.0773 (25), 55.0174 (10), 84.9594 (10)	2	8.64E-03	8.53E-01	0.06	4.65E-02	-0.50	5.35E-03	-0.70	9.24E-02	0.48
189.1115@6.041323	189.1115	6.04	POS	hilic				1.58E-01	4.11E-01	0.25	9.01E-01	0.03	9.29E-03	-0.58	8.41E-01	-0.05
189.1365@0.8038297	189.1365	0.80	POS	hilic				1.91E-01	7.72E-01	-0.08	7.29E-01	0.07	3.46E-01	0.29	5.74E-03	0.70
189.1371@0.8462797	189.1371	0.85	POS	hilic				3.29E-01	1.03E-02	0.70	2.43E-01	0.32	9.44E-01	0.02	6.31E-01	0.14
189.9011@15.262188	189.9011	15.26	NEG	RP				7.42E-01	8.94E-01	0.04	3.80E-01	-0.29	9.62E-01	-0.01	1.84E-01	-0.38
189.9936@1.8992503	189.9936	1.90	NEG	RP				3.44E-02	5.05E-01	0.18	3.12E-02	-0.60	2.13E-03	-0.84	5.28E-01	-0.21
189.9937@1.8992653	189.9937	1.90	NEG	RP				3.89E-02	5.01E-01	0.18	3.30E-02	-0.58	2.19E-03	-0.84	5.06E-01	-0.23
189.9943@0.4888459	189.9943	0.49	NEG	hilic				1.33E-01	9.03E-01	-0.04	4.72E-02	-0.56	2.29E-03	-0.81	5.20E-01	-0.21
190.0331@6.0259314	190.0331	6.03	POS	hilic				6.45E-02	1.98E-02	-0.75	3.08E-01	-0.30	9.27E-03	-0.67	2.36E-03	-0.91
190.0476@3.342414	190.0476	3.34	NEG	hilic				5.12E-01	6.85E-01	0.13	3.59E-02	0.55	4.05E-01	0.26	8.23E-01	-0.05
190.0781@9.187453	190.0781	9.19	POS	RP				6.14E-01	6.49E-01	0.11	7.28E-02	0.48	9.07E-01	-0.04	9.32E-01	-0.03
190.1062@6.0245214	190.1062	6.02	POS	hilic				6.17E-01	5.61E-01	0.20	9.89E-01	0.00	2.10E-01	-0.39	5.34E-01	0.16
190.1354@9.427631	190.1354	9.43	POS	RP				7.72E-01	3.55E-01	0.26	3.62E-01	0.25	5.82E-01	0.14	1.69E-01	0.45
190.1571@7.1598516	190.1571	7.16	POS	RP				8.73E-01	2.30E-01	0.34	6.34E-01	0.14	4.48E-01	0.22	2.53E-01	0.33
190.1722@8.534382	190.1722	8.53	POS	RP				6.91E-01	9.74E-01	-0.01	7.80E-01	-0.08	2.43E-01	0.41	3.93E-01	0.23
190.1722@8.624215	190.1722	8.62	POS	RP				7.01E-01	9.55E-01	0.02	7.50E-01	-0.10	2.42E-01	0.42	4.84E-01	0.19
191.0585@4.980439	191.0585	4.98	POS	RP				1.10E-01	2.89E-03	-0.86	2.78E-01	-0.30	5.32E-01	-0.18	2.54E-02	-0.70
191.059@3.9686472	191.0590	3.97	POS	hilic				7.23E-01	3.72E-01	-0.26	4.62E-01	0.18	6.07E-01	0.15	7.33E-01	-0.09
191.1522@1.054615	191.1522	1.05	POS	hilic				8.80E-01	6.16E-01	0.15	7.01E-01	-0.11	5.18E-01	0.20	6.68E-01	-0.14
191.8976@15.26358	191.8976	15.26	NEG	RP				6.03E-01	5.08E-01	-0.19	7.60E-02	-0.54	2.35E-01	-0.32	1.71E-01	-0.44
191.9357@15.266379	191.9357	15.27	NEG	RP				6.96E-01	3.60E-01	-0.25	3.19E-01	0.26	5.17E-01	0.20	8.43E-01	-0.05
192.0579@2.9314442	192.0579	2.93	POS	RP				9.35E-01	8.70E-01	0.05	6.55E-01	-0.14	7.28E-01	0.12	6.00E-01	0.17
192.0902@0.7047316	192.0902	0.70	POS	hilic				2.46E-03	1.05E-02	0.77	3.36E-01	0.19	8.90E-01	0.03	3.54E-03	-0.34
192.1506@10.662227	192.1506	10.66	POS	RP				8.37E-01	2.18E-01	-0.32	2.62E-01	-0.31	2.49E-01	-0.30	5.57E-01	-0.17
192.151@10.6044855	192.1510	10.60	POS	RP				8.52E-01	2.35E-01	-0.30	4.16E-01	-0.23	1.98E-01	-0.35	4.21E-01	-0.24
192.1511@7.4389906	192.1511	7.44	POS	RP				7.44E-01	4.03E-01	-0.23	3.07E-01	-0.28	7.32E-02	-0.47	5.41E-01	-0.19
193.0016@5.378582	193.0016	5.38	NEG	hilic				8.61E-01	2.28E-01	0.36	5.90E-01	0.12	5.13E-01	0.15	5.39E-01	0.13
193.0151@4.5304523	193.0151	4.53	POS	hilic				3.03E-01	1.05E-01	-0.56	7.74E-01	-0.08	6.42E-02	-0.46	6.56E-01	0.12
193.0361@4.1185203	193.0361	4.12	NEG	hilic				6.85E-01	6.78E-02	0.47	9.27E-01	-0.02	6.38E-01	0.15	3.58E-01	0.27
193.0364@4.8417706	193.0364	4.84	NEG	hilic				5.08E-01	3.02E-01	0.28	1.51E-01	-0.34	8.22E-01	0.07	6.90E-01	0.10
193.0366@3.584512	193.0366	3.58	NEG	hilic				7.45E-01	8.07E-01	0.05	2.23E-01	-0.30	7.41E-01	0.11	4.01E-01	-0.23
193.0369@3.59345	193.0369	3.59	NEG	hilic				9.70E-01	8.07E-01	-0.07	8.15E-01	0.06	6.20E-01	0.16	7.15E-01	0.09
194.0805@3.391562	194.0805	3.39	POS	RP	Caffeine	10 eV: 195.0870 (100), 138.0645 (25)	2	2.26E-01	1.59E-01	0.51	7.57E-01	-0.09	9.28E-01	0.02	1.21E-01	-0.42
194.0808@3.391562	194.0808	3.39	POS	RP	Caffeine	10 eV: 195.0870 (100), 138.0645 (25)	2	2.25E-01	1.59E-01	0.51	7.60E-01	-0.09	9.28E-01	0.02	1.19E-01	-0.42
194.0813@0.5053739	194.0813	0.51	POS	hilic				1.97E-01	1.48E-01	0.50	6.41E-01	-0.14	9.62E-01	0.01	1.08E-01	-0.43
194.0943@7.3390164	194.0943	7.34	NEG	RP				3.53E-01	1.92E-02	-0.71	4.20E-01	-0.24	1.25E-01	-0.47	6.98E-01	-0.11
194.1156@0.6605638	194.1156	0.66	POS	hilic				7.70E-01	5.62E-01	-0.19	9.47E-01	-0.02	6.05E-01	0.15	3.14E-01	-0.33
194.1156@1.8260089	194.1156	1.83	POS	RP				7.81E-01	9.40E-01	-0.02	7.77E-01	-0.08	1.81E-01	-0.39	3.41E-01	-0.31
194.1156@2.3178728	194.1156	2.32	POS	RP				9.94E-01	6.25E-01	-0.14	7.62E-01	-0.09	7.01E-01	-0.10	9.43E-01	-0.02
195.0155@4.7556663	195.0155	4.76	POS	hilic				3.66E-01	4.48E-01	-0.17	2.50E-01	-0.25	2.70E-01	0.30	3.61E-01	-0.19
195.0159@3.8468044	195.0159	3.85	POS	hilic				4.46E-01	6.33E-01	-0.13	7.95E-02	-0.54	7.45E-01	0.11	9.36E-01	0.02
195.016@3.853294	195.0160	3.85	POS	hilic				5.08E-01	8.87E-01	-0.04	9.59E-02	-0.50	7.46E-01	0.11	8.74E-01	0.04
195.0161@4.1627717	195.0161	4.16	POS	hilic				2.27E-01	1.05E-01	-0.42	3.55E-01	-0.24	2.45E-01	0.38	6.30E-01	0.11
195.066@3.0326452	195.0660	3.03	POS	hilic				1.57E-01	4.08E-01	0.28	3.09E-02	-0.50	6.57E-02	-0.41	5.42E-01	-0.15

Supplementary table 2: Metabolomics analysis results

Compound	Mass	Retention Time	Ionization mode	column	Compound_ID	MSMS_spectra	ID_level	ANOVA P-FDR	Anorexia vs control P	Anorexia vs control Estimate	overweight vs control P	overweight vs control Estimate	obese vs control P	obese vs control Estimate	athletes vs control P	athletes vs control Estimate
195.1625@8.564618	195.1625	8.56	POS	RP				4.95E-01	1.31E-01	-0.45	7.69E-01	-0.08	5.35E-01	0.19	5.25E-01	0.17
195.9959@15.3317585	195.9959	15.33	NEG	RP				9.52E-01	6.23E-01	-0.13	6.47E-01	-0.14	3.99E-01	-0.29	6.97E-01	-0.11
196.0596@1.0282269	196.0596	1.03	NEG	hilic				1.69E-02	1.43E-02	0.84	8.65E-01	-0.04	6.20E-01	0.12	1.25E-01	-0.37
196.1214@0.51339996	196.1214	0.51	POS	hilic				5.24E-01	8.31E-01	0.06	8.17E-01	-0.06	8.10E-01	0.06	6.29E-02	-0.50
196.1214@3.3239913	196.1214	3.32	POS	RP				1.84E-01	7.50E-01	-0.10	2.95E-01	0.32	8.65E-02	0.49	1.98E-01	-0.37
196.818@1.1231455	196.8180	1.12	NEG	RP				4.76E-01	5.45E-01	-0.19	1.86E-01	-0.39	1.53E-01	-0.37	1.68E-02	-0.66
196.8185@15.361493	196.8185	15.36	NEG	RP				6.09E-01	2.50E-01	0.38	9.61E-01	-0.01	3.47E-01	-0.29	9.85E-01	0.00
196.9127@0.93447596	196.9127	0.93	NEG	hilic				1.48E-01	5.39E-01	0.16	1.39E-02	-0.60	1.01E-01	-0.47	6.57E-01	-0.12
197.0301@4.4249115	197.0301	4.42	NEG	hilic				2.40E-01	1.23E-01	0.45	3.88E-01	-0.22	1.93E-01	-0.36	6.36E-01	0.14
197.0369@2.9340088	197.0369	2.93	POS	RP				9.88E-01	7.16E-01	0.11	7.68E-01	-0.09	8.86E-01	0.05	8.79E-01	0.05
197.0454@4.5247498	197.0454	4.52	POS	hilic				3.60E-02	1.90E-01	0.30	5.73E-01	0.10	8.82E-01	-0.02	3.22E-03	0.71
197.0455@0.5182696	197.0455	0.52	POS	hilic				3.64E-01	5.93E-02	-0.61	9.93E-01	0.00	2.46E-01	-0.36	7.47E-02	-0.48
197.0901@5.119281	197.0901	5.12	POS	hilic				1.33E-02	8.69E-03	-0.74	1.85E-02	0.55	5.92E-01	-0.14	5.00E-01	-0.20
197.1418@6.115468	197.1418	6.12	POS	RP				3.78E-01	1.14E-01	-0.43	2.83E-02	-0.57	2.61E-01	-0.32	5.56E-02	-0.54
197.1419@6.1271796	197.1419	6.13	POS	RP				9.91E-01	6.69E-01	-0.14	7.42E-01	-0.09	9.82E-01	-0.01	9.21E-01	0.03
197.1439@0.45061404	197.1439	0.45	POS	hilic				9.73E-01	6.85E-01	-0.11	4.37E-01	-0.18	8.63E-01	-0.04	9.24E-01	-0.03
197.178@10.646352	197.1780	10.65	POS	RP				7.31E-01	4.87E-01	-0.18	9.62E-02	-0.47	6.38E-01	-0.13	5.70E-01	-0.17
197.1782@10.673931	197.1782	10.67	POS	RP				7.37E-01	8.08E-01	-0.06	5.83E-01	-0.14	9.84E-02	-0.40	8.93E-01	-0.04
198.0136@1.862034	198.0136	1.86	NEG	hilic				1.88E-03	1.50E-01	-0.46	3.45E-02	0.62	4.29E-03	0.79	4.83E-01	-0.18
198.0502@0.880564	198.0502	0.88	NEG	hilic				2.36E-01	2.95E-01	-0.32	3.86E-02	0.52	2.69E-01	0.33	7.98E-01	0.07
198.0751@1.0216384	198.0751	1.02	NEG	hilic				2.38E-01	9.28E-02	0.57	2.51E-01	0.30	1.43E-01	0.37	4.80E-01	-0.19
198.0751@1.0343	198.0751	1.03	NEG	hilic				4.46E-01	4.22E-01	0.23	2.27E-01	0.29	1.29E-02	0.58	4.22E-01	0.18
198.0751@1.81775	198.0751	1.82	NEG	hilic				8.09E-03	2.55E-03	0.91	4.46E-01	0.17	2.81E-01	0.26	1.63E-01	-0.28
198.162@8.332896	198.1620	8.33	POS	RP				7.10E-01	5.91E-01	0.16	1.69E-01	0.36	9.53E-01	-0.02	1.56E-01	0.40
198.1622@8.327501	198.1622	8.33	POS	RP				5.57E-01	5.07E-02	0.61	1.93E-01	0.40	4.51E-01	0.23	5.92E-01	0.15
198.1626@9.648872	198.1626	9.65	POS	RP				5.58E-01	4.16E-01	0.23	8.06E-01	0.07	1.07E-01	0.60	2.87E-01	0.26
198.8148@4.384145	198.8148	4.38	NEG	hilic				9.84E-01	8.99E-01	-0.04	7.32E-01	-0.09	7.24E-01	-0.09	7.71E-01	0.08
198.8586@15.322038	198.8586	15.32	NEG	RP				7.29E-01	8.17E-01	0.06	4.03E-01	-0.23	7.72E-01	-0.08	1.79E-01	-0.39
199.0184@15.418259	199.0184	15.42	POS	RP				8.98E-01	2.72E-01	0.33	7.37E-01	0.11	7.21E-01	0.09	3.53E-01	0.28
199.0334@6.6866994	199.0334	6.69	POS	hilic				1.37E-01	1.85E-02	-0.71	4.99E-01	0.21	1.66E-01	-0.34	2.35E-01	-0.34
199.1574@0.45157894	199.1574	0.45	POS	hilic				7.55E-01	2.96E-01	-0.32	1.32E-01	-0.46	4.08E-01	-0.26	6.57E-01	-0.13
199.1574@3.7177517	199.1574	3.72	POS	RP				9.75E-01	8.88E-01	0.04	6.29E-01	0.15	6.37E-01	0.13	8.61E-01	-0.06
199.1574@6.149734	199.1574	6.15	POS	RP				3.89E-01	9.30E-02	-0.41	7.45E-02	-0.42	1.11E-01	-0.40	8.28E-01	0.06
199.1577@10.367973	199.1577	10.37	POS	RP				1.15E-01	8.71E-03	-0.73	1.17E-02	-0.66	7.59E-03	-0.63	4.87E-02	-0.57
199.1577@4.0609913	199.1577	4.06	POS	RP				7.91E-01	1.47E-01	-0.43	9.59E-01	-0.01	7.73E-01	-0.09	9.97E-01	0.00
199.1578@10.30089	199.1578	10.30	POS	RP				2.50E-01	9.59E-04	-0.70	7.46E-02	-0.43	2.37E-01	-0.28	5.25E-01	-0.19
199.1938@9.168131	199.1938	9.17	POS	RP				9.95E-01	9.49E-01	-0.02	9.56E-01	0.02	9.08E-01	-0.04	7.36E-01	0.11
199.961@3.3659997	199.9610	3.37	NEG	hilic				9.53E-01	4.25E-01	0.22	8.92E-01	0.04	8.26E-01	0.06	4.83E-01	0.20
199.9621@3.418951	199.9621	3.42	NEG	hilic				7.31E-01	1.62E-01	-0.39	9.50E-01	0.02	2.15E-01	-0.34	4.49E-01	-0.19
200.014@0.47828263	200.0140	0.48	NEG	hilic				2.78E-01	1.17E-01	0.43	7.09E-01	-0.08	2.38E-01	-0.25	8.93E-01	-0.03
200.014@4.196414	200.0140	4.20	NEG	RP				1.08E-01	2.45E-01	0.35	1.86E-01	-0.31	2.18E-02	-0.53	5.08E-01	-0.18
200.045@2.4676409	200.0450	2.47	NEG	hilic				2.70E-02	1.21E-01	-0.46	8.55E-03	0.70	1.94E-01	0.31	2.35E-01	0.36
200.0479@8.102424	200.0479	8.10	POS	RP				3.79E-01	1.49E-02	-0.48	2.54E-01	-0.25	7.68E-01	0.08	8.33E-01	-0.05
200.1049@5.721582	200.1049	5.72	POS	RP				7.97E-01	7.04E-01	-0.11	9.16E-01	0.03	3.29E-01	0.31	3.95E-01	0.25
200.1414@7.7956014	200.1414	7.80	POS	RP				9.65E-01	9.73E-01	0.01	3.82E-01	0.22	7.30E-01	0.11	9.48E-01	0.02
201.0614@5.541793	201.0614	5.54	POS	hilic				8.70E-02	2.47E-03	-0.72	5.66E-01	0.15	2.89E-01	-0.27	7.72E-01	-0.07
201.0794@0.5321563	201.0794	0.53	POS	hilic				5.27E-01	3.96E-01	0.23	3.27E-01	0.26	3.54E-01	-0.22	6.40E-01	-0.12
201.111@3.9129822	201.1110	3.91	POS	hilic				5.03E-02	2.98E-02	0.64	5.92E-01	0.15	2.64E-03	0.97	2.01E-01	0.32
201.1112@4.099697	201.1112	4.10	POS	hilic				6.84E-03	9.38E-01	-0.02	5.86E-01	0.11	1.12E-04	1.05	5.78E-01	0.15
201.148@3.4897892	201.1480	3.49	POS	hilic				1.13E-02	2.47E-01	0.33	8.98E-01	0.03	4.39E-01	-0.13	9.50E-04	0.83
201.1731@0.8183514	201.1731	0.82	POS	hilic				6.95E-01	3.35E-01	0.31	1.31E-01	0.38	8.70E-01	0.05	7.73E-01	-0.08
201.1733@0.8136776	201.1733	0.81	POS	hilic				9.80E-01	4.97E-01	-0.18	5.84E-01	-0.16	5.97E-01	-0.15	8.04E-01	-0.07
201.1733@1.3565121	201.1733	1.36	POS	hilic				9.00E-01	8.43E-01	-0.07	8.49E-01	-0.06	3.12E-01	0.27	7.93E-01	0.08
201.8655@10.722008	201.8655	10.72	NEG	RP				9.16E-01	3.58E-01	-0.28	7.20E-01	-0.09	9.52E-01	0.02	4.44E-01	-0.20
201.8659@10.430251	201.8659	10.43	NEG	RP				3.34E-01	1.31E-01	-0.38	2.14E-01	0.37	9.76E-01	-0.01	4.60E-01	-0.21
202.0153@2.60972	202.0153	2.61	POS	RP				6.79E-02	6.04E-03	-0.74	9.79E-01	-0.01	2.37E-01	-0.30	3.64E-01	0.25
202.0294@0.48553842	202.0294	0.49	NEG	hilic				2.97E-02	1.09E-03	0.75	1.51E-01	0.34	9.56E-01	-0.01	7.52E-01	0.06
202.0295@4.7026978	202.0295	4.70	NEG	RP				1.29E-02	8.33E-05	0.96	1.26E-01	0.41	9.28E-01	-0.02	3.32E-01	0.23
202.0448@1.4423965	202.0448	1.44	NEG	hilic				6.10E-02	6.63E-01	-0.12	1.57E-01	0.42	1.78E-04	0.89	3.48E-01	0.30
202.045@1.526839	202.0450	1.53	NEG	hilic				1.50E-01	2.71E-01	0.32	1.73E-01	0.35	1.65E-03	0.89	6.85E-02	0.59
202.046@5.1926155	202.0460	5.19	POS	hilic				8.67E-01	5.29E-01	0.17	9.75E-01	0.01	3.63E-01	-0.24	6.89E-01	-0.13
202.132@4.580699	202.1320	4.58	POS	hilic				2.33E-02	2.82E-01	0.33	4.35E-01	-0.19	3.13E-03	-0.86	4.72E-01	-0.18
202.1432@6.3047647	202.1432	6.30	POS	hilic				1.24E-01	4.15E-01	-0.25	3.83E-01	0.24	1.42E-02	-0.72	7.76E-01	-0.08
202.1432@6.3367705	202.1432	6.34	POS	hilic				6.07E-01	8.10E-02	-0.52	1.59E-01	-0.41	1.15E-01	-0.49	2.11E-01	-0.39
202.1569@8.810083	202.1569	8.81	POS	RP				7.64E-01	9.37E-01	0.03	1.68E-01	0.36	3.22E-01	0.29	2.36E-01	0.36
202.8963@15.328296	202.8963	15.33	NEG	RP				5.39E-01	7.65E-02	0.47	2.18E-02	0.57	2.97E-01	0.36	1.64E-01	0.40
203.035@3.8302386	203.0350	3.83	POS	hilic				1.90E-03	7.87E-03	-0.86	4.64E-01	0.16	1.09E-02	0.64	5.32E-01	-0.17
203.1159@2.89935	203.1159	2.90	POS	hilic	Acetylcarnitine	10 eV: 204.1238 (100), 85.0292 (95), 145.0517 (5)	2	8.31E-01	5.96E-01	-0.14	9.45E-01	0.02	7.00E-01	0.10	3.74E-01	0.28
204.0086@0.4754259	204.0086	0.48	NEG	hilic				7.01E-01	5.00E-01	-0.17	6.83E-01	-0.09	5.53E-01	0.16	3.18E-01	0.26
204.0087@0.4778751	204.0087	0.48	NEG	hilic				5.25E-01	1.28E-01	0.42	3.15E-01	-0.24	8.78E-01	0.05	6.90E-01	0.11
204.0087@3.05	204.0087	3.05	NEG	RP				9.19E-01	7.87E-01	0.08	4.63E-01	-0.17	4.78E-01	-0.23	8.58E-01	-0.05
204.0271@6.0789866	204.0271	6.08	NEG	hilic				2.25E-01	2.14E-02	0.73	3.05E-01	0.26	6.02E-01	0.11	8.91E-02	0.45
204.0517@5.16136	204.0517	5.16	POS	hilic				3.84E-01	4.46E-02	-0.50	6.61E-01	0.14	1.25E-01	-0.41	3.80E-01	-0.22

Supplementary table 2: Metabolomics analysis results

								ANOVA	Anorexia vs control		overweight vs control		obese vs control		athletes vs control	
Compound	Mass	Retention Time	Ionization mode	column	Compound_ID	MSMS_spectra	ID_level	P-FDR	P	Estimate	P	Estimate	P	Estimate	P	Estimate
204.0608@4.8377023	204.0608	4.84	POS	hilic				2.04E-01	7.14E-01	0.11	1.73E-01	-0.32	8.17E-02	-0.44	2.79E-02	-0.51
204.0788@9.19206	204.0788	9.19	POS	RP				9.40E-01	7.15E-01	-0.12	8.27E-01	-0.07	5.56E-01	0.17	7.18E-01	-0.11
204.0791@9.267768	204.0791	9.27	POS	RP				9.68E-01	8.45E-01	0.06	7.37E-01	-0.10	9.83E-01	0.01	5.79E-01	0.16
204.0893@4.115113	204.0893	4.12	NEG	hilic				5.16E-01	1.48E-01	-0.42	5.56E-01	0.18	3.24E-01	0.24	7.77E-01	0.09
204.0897@2.4622643	204.0897	2.46	NEG	RP				8.27E-01	9.13E-01	-0.03	2.81E-01	0.35	5.35E-01	0.16	4.57E-01	0.24
204.0898@2.0822325	204.0898	2.08	NEG	RP				3.30E-01	3.23E-02	-0.65	9.12E-01	-0.03	6.31E-01	0.13	3.85E-01	-0.25
204.0904@3.9583356	204.0904	3.96	POS	hilic	Tryptophan	10 eV: 188.0710 (100), 146.0602 (38)	2	6.33E-01	4.13E-01	-0.23	2.98E-01	0.32	2.74E-01	0.28	8.82E-01	0.05
204.0906@2.4428887	204.0906	2.44	POS	RP	Tryptophan	20 eV: 146.0602 (100), 118.0648 (65), 188.0665 (15)	1	5.79E-01	5.37E-01	-0.18	2.82E-01	0.35	2.80E-01	0.29	2.84E-01	0.34
204.0907@2.0389564	204.0907	2.04	POS	RP				8.91E-01	5.24E-01	-0.17	4.79E-01	0.22	8.00E-01	0.06	8.82E-01	-0.05
204.0924@0.49116123	204.0924	0.49	POS	hilic				7.82E-01	6.09E-01	-0.14	5.46E-01	-0.16	9.67E-02	-0.40	7.18E-01	-0.10
205.0409@0.50695455	205.0409	0.51	POS	hilic				3.45E-03	2.51E-03	0.98	8.28E-01	-0.04	5.17E-01	-0.14	3.89E-01	0.19
205.0736@0.73800015	205.0736	0.74	NEG	hilic				5.45E-01	3.65E-01	-0.27	9.05E-01	-0.03	1.92E-01	-0.35	4.52E-01	0.22
205.0736@4.3926497	205.0736	4.39	NEG	RP				4.67E-01	3.96E-01	-0.29	6.66E-01	-0.13	3.48E-01	-0.26	2.42E-01	0.37
205.0744@4.3720164	205.0744	4.37	POS	RP				3.69E-01	1.17E-01	-0.48	7.27E-01	0.10	9.99E-01	0.00	2.86E-01	0.34
205.1313@0.69056356	205.1313	0.69	POS	hilic				5.11E-01	3.97E-01	0.25	2.26E-01	-0.26	8.63E-01	0.04	3.97E-01	0.21
205.8787@3.0694878	205.8787	3.07	POS	hilic				9.91E-01	6.40E-01	0.17	9.86E-01	0.00	7.79E-01	0.08	7.83E-01	0.08
205.9878@1.6004286	205.9878	1.60	NEG	RP				6.90E-01	6.02E-01	-0.14	9.66E-01	0.01	1.14E-01	-0.32	1.97E-01	-0.28
205.9883@0.47816885	205.9883	0.48	NEG	hilic				7.00E-01	2.71E-01	-0.32	9.66E-01	-0.01	1.28E-01	-0.39	3.40E-01	-0.24
206.0594@5.213191	206.0594	5.21	NEG	hilic				1.18E-03	9.59E-06	-1.29	6.12E-01	-0.15	3.12E-01	-0.27	2.84E-04	-0.82
206.0694@0.76992935	206.0694	0.77	POS	hilic				5.77E-01	6.49E-01	-0.14	2.69E-01	-0.32	3.71E-02	-0.54	2.52E-01	-0.32
206.1517@0.46093336	206.1517	0.46	POS	hilic				7.83E-01	2.92E-01	0.31	7.56E-01	-0.07	4.45E-01	0.22	4.08E-01	0.24
206.152@5.845367	206.1520	5.85	POS	RP				9.55E-01	5.77E-01	0.17	8.12E-01	-0.07	5.86E-01	0.16	9.67E-01	-0.01
206.1664@9.434797	206.1664	9.43	NEG	RP				8.41E-01	2.27E-01	-0.32	4.86E-01	-0.18	9.65E-01	-0.01	9.38E-01	-0.02
206.8471@15.345901	206.8471	15.35	NEG	RP				2.50E-01	4.09E-03	0.83	2.39E-01	0.38	3.63E-01	0.24	7.80E-02	0.51
206.9783@7.3172903	206.9783	7.32	POS	hilic				5.08E-01	4.59E-01	0.24	5.26E-01	0.19	1.44E-01	-0.39	5.87E-01	-0.13
207.1106@4.900833	207.1106	4.90	NEG	hilic				7.77E-02	2.79E-01	0.19	2.54E-01	0.21	6.55E-03	0.73	3.01E-01	0.18
207.1259@1.3446486	207.1259	1.34	POS	hilic				8.69E-01	2.25E-01	-0.20	9.47E-01	0.01	7.00E-01	0.08	7.54E-01	0.08
207.8616@0.5650428	207.8616	0.57	NEG	hilic				3.41E-01	3.65E-01	0.31	7.24E-02	0.50	7.60E-02	0.54	7.55E-01	-0.08
207.8774@3.0703616	207.8774	3.07	POS	hilic				5.01E-01	3.64E-01	-0.28	1.04E-01	-0.47	3.09E-01	-0.29	6.61E-01	0.13
207.9795@7.9216857	207.9795	7.92	NEG	hilic				9.72E-01	5.44E-01	0.20	4.43E-01	0.19	7.76E-01	0.08	7.63E-01	0.08
208.0849@1.6506155	208.0849	1.65	POS	RP				3.30E-01	1.47E-01	-0.48	4.94E-01	0.18	2.21E-01	0.33	7.58E-01	0.10
208.0852@3.9666748	208.0852	3.97	POS	hilic				1.98E-01	1.60E-01	-0.45	4.20E-01	0.21	1.12E-01	0.44	6.10E-01	-0.16
208.0952@2.4147947	208.0952	2.41	POS	hilic				9.17E-01	3.18E-01	0.32	5.73E-01	0.17	6.78E-01	0.13	3.99E-01	0.27
208.1102@8.534527	208.1102	8.53	POS	RP				2.04E-01	1.77E-02	-0.70	9.79E-01	0.01	8.24E-01	-0.06	1.03E-01	-0.52
208.1465@8.739932	208.1465	8.74	POS	RP				3.50E-01	2.60E-01	-0.36	2.04E-01	0.35	9.23E-01	-0.03	2.46E-01	0.34
208.147@10.610396	208.1470	10.61	POS	RP				7.09E-01	4.32E-01	-0.21	7.36E-01	-0.08	4.91E-02	-0.41	9.64E-01	-0.01
208.1488@0.4221201	208.1488	0.42	POS	hilic				3.07E-01	1.28E-01	-0.37	6.66E-02	-0.42	4.94E-02	-0.46	8.66E-01	0.05
208.1496@10.614676	208.1496	10.61	POS	RP				9.34E-01	8.74E-01	0.04	8.32E-01	-0.05	3.96E-01	-0.18	5.64E-01	-0.13
208.1829@8.61951	208.1829	8.62	POS	RP				6.31E-01	5.84E-01	-0.12	5.74E-01	-0.14	2.53E-01	0.34	8.52E-01	-0.05
208.1831@8.534173	208.1831	8.53	POS	RP				5.28E-01	7.96E-01	0.06	8.74E-01	-0.04	1.02E-01	0.52	5.63E-01	0.16
208.927@15.270849	208.9270	15.27	NEG	RP				6.18E-01	8.08E-02	-0.53	9.64E-02	-0.43	2.98E-01	-0.30	3.03E-01	-0.30
209.0365@3.0744796	209.0365	3.07	POS	RP				1.48E-01	3.14E-01	-0.30	5.30E-02	0.53	3.03E-01	0.30	4.02E-01	-0.23
209.0366@3.3182056	209.0366	3.32	POS	RP				4.62E-01	5.84E-01	-0.17	6.58E-02	0.50	4.74E-01	0.22	9.71E-01	-0.01
209.0431@3.8306742	209.0431	3.83	POS	hilic				1.01E-02	2.97E-01	-0.35	1.14E-03	0.70	1.77E-02	0.66	7.24E-01	-0.09
209.1779@7.6997156	209.1779	7.70	POS	RP				7.31E-01	2.94E-01	0.37	8.06E-01	-0.07	4.62E-01	0.20	7.40E-01	-0.10
209.1786@9.045297	209.1786	9.05	POS	RP				8.84E-01	4.18E-01	-0.30	3.28E-01	-0.25	9.59E-01	0.02	6.08E-01	-0.16
209.8588@0.5629434	209.8588	0.56	NEG	hilic				7.47E-01	4.65E-01	0.24	1.62E-01	0.40	3.77E-01	0.27	9.71E-01	-0.01
209.9416@3.335978	209.9416	3.34	NEG	hilic				7.58E-01	2.23E-01	0.31	8.42E-01	0.04	6.45E-01	0.10	2.24E-01	0.32
209.9416@3.4830983	209.9416	3.48	NEG	hilic				2.22E-01	3.44E-02	0.68	9.25E-01	-0.03	6.28E-01	0.13	6.60E-02	0.53
209.9971@0.50769806	209.9971	0.51	POS	hilic				2.96E-01	1.52E-01	0.50	4.43E-01	0.20	2.71E-01	-0.33	9.44E-01	0.02
210.0736@2.4672139	210.0736	2.47	NEG	hilic				9.36E-03	1.73E-02	-0.69	1.58E-02	0.60	2.43E-01	0.26	3.47E-01	0.29
210.0895@6.0877876	210.0895	6.09	POS	RP				2.35E-01	6.35E-01	0.13	3.57E-02	-0.52	2.16E-01	-0.35	5.64E-01	0.16
210.137@4.298421	210.1370	4.30	POS	RP				3.53E-01	2.09E-01	0.43	9.87E-01	-0.01	2.30E-01	0.31	2.19E-01	-0.32
210.137@4.4407744	210.1370	4.44	POS	RP				5.56E-01	2.85E-01	0.34	4.08E-01	-0.24	4.77E-01	-0.21	5.32E-01	-0.16
210.1371@4.301035	210.1371	4.30	POS	RP				1.51E-01	1.50E-01	0.49	9.22E-01	-0.03	1.90E-01	0.34	9.30E-02	-0.43
210.1377@0.4959245	210.1377	0.50	POS	hilic				4.91E-01	2.12E-01	0.38	9.73E-01	-0.01	4.80E-01	-0.17	4.14E-01	-0.18
210.1626@10.604657	210.1626	10.60	POS	RP				5.76E-01	5.95E-02	-0.56	3.71E-01	-0.26	2.44E-01	-0.31	9.62E-01	-0.01
210.1627@10.65407	210.1627	10.65	POS	RP				7.72E-01	2.26E-01	-0.33	1.69E-01	-0.42	4.78E-01	-0.18	6.89E-01	-0.12
211.013@0.45337963	211.0130	0.45	POS	hilic				1.09E-01	6.96E-01	-0.10	4.46E-01	0.23	7.11E-01	-0.11	1.56E-02	-0.75
211.013@6.649564	211.0130	6.65	POS	RP				1.10E-01	4.55E-01	-0.18	5.74E-01	0.17	4.38E-01	-0.25	9.52E-03	-0.79
211.1425@0.6607388	211.1425	0.66	POS	hilic				4.53E-01	5.44E-01	0.18	6.86E-01	0.11	3.82E-01	0.26	1.76E-01	-0.42
211.8905@15.286368	211.8905	15.29	NEG	RP				9.54E-01	3.76E-01	-0.26	4.92E-01	-0.18	9.34E-01	-0.03	7.81E-01	-0.09
211.9384@3.4869785	211.9384	3.49	NEG	hilic				8.41E-01	8.21E-01	-0.06	3.05E-01	0.30	9.05E-01	-0.03	5.65E-01	0.16
211.9697@7.731865	211.9697	7.73	NEG	hilic				9.51E-01	8.20E-01	0.06	6.00E-01	0.12	9.87E-01	0.00	4.35E-01	0.21
211.9928@4.6944184	211.9928	4.69	POS	hilic				5.45E-01	1.64E-01	0.40	5.61E-01	-0.18	4.57E-01	0.26	2.76E-01	0.31
212.1415@7.833681	212.1415	7.83	POS	RP				9.79E-01	7.82E-01	-0.09	4.64E-01	-0.21	9.61E-01	-0.01	8.57E-01	-0.06
213.0091@0.59974355	213.0091	0.60	NEG	hilic				2.91E-01	9.39E-02	0.64	8.47E-01	-0.05	5.45E-01	-0.15	4.32E-01	0.22
213.0098@2.374222	213.0098	2.37	NEG	RP				3.80E-01	1.33E-01	0.56	7.38E-01	-0.09	4.34E-01	-0.20	6.81E-01	0.11
213.0281@6.7400002	213.0281	6.74	NEG	hilic				8.43E-01	7.38E-01	-0.08	4.28E-01	0.19	7.14E-01	-0.09	5.85E-01	0.14
213.0828@6.64583	213.0828	6.65	POS	RP				4.09E-01	8.94E-02	0.58	8.38E-01	0.05	2.60E-01	0.32	9.80E-02	0.53
213.1726@6.847028	213.1726	6.85	POS	RP				8.09E-01	6.86E-01	-0.12	4.61E-01	-0.22	8.05E-01	-0.07	4.57E-01	0.22
213.1729@10.374838	213.1729	10.37	POS	RP				7.10E-01	6.06E-01	-0.12	8.59E-01	-0.04	6.31E-02	-0.37	8.35E-01	-0.05
213.1731@0.42365015	213.1731	0.42	POS	hilic				9.98E-01	8.32E-01	0.05	8.94E-01	0.04	9.25E-01	-0.02	8.53E-01	0.05

Supplementary table 2: Metabolomics analysis results

								ANOVA	Anorexia vs control		overweight vs control		obese vs control		athletes vs control	
Compound	Mass	Retention Time	Ionization mode	column	Compound_ID	MSMS_spectra	ID_level	P-FDR	P	Estimate	P	Estimate	P	Estimate	P	Estimate
213.1732@10.68387	213.1732	10.68	POS	RP				8.82E-01	2.35E-01	-0.29	3.29E-01	-0.25	3.86E-01	-0.21	5.48E-01	-0.16
213.9104@7.874809	213.9104	7.87	POS	hilic				2.13E-01	5.29E-02	0.71	4.24E-02	0.53	2.21E-02	0.62	1.06E-02	0.72
213.9107@15.275828	213.9107	15.28	POS	RP				1.97E-01	9.04E-02	-0.53	1.12E-01	-0.49	1.27E-01	-0.46	2.86E-03	-0.86
213.928@3.1975944	213.9280	3.20	NEG	hilic				9.14E-01	7.82E-01	0.09	9.26E-01	-0.03	9.76E-01	-0.01	3.16E-01	0.29
214.0295@0.47573212	214.0295	0.48	NEG	hilic				9.58E-04	1.57E-02	0.61	3.55E-01	-0.27	3.73E-04	-0.89	4.73E-01	-0.20
214.0299@5.318545	214.0299	5.32	NEG	RP				7.54E-04	1.53E-02	0.62	2.55E-01	-0.33	5.58E-04	-0.93	2.29E-01	-0.34
214.0301@5.131558	214.0301	5.13	NEG	RP				1.78E-03	1.41E-01	0.41	1.61E-02	-0.57	1.21E-03	-0.74	9.88E-01	0.00
214.056@6.079522	214.0560	6.08	NEG	hilic				8.66E-01	3.74E-01	0.31	8.35E-01	0.07	6.62E-01	-0.11	9.03E-01	-0.04
214.1209@6.5456386	214.1209	6.55	POS	RP				8.15E-01	4.41E-01	-0.21	7.67E-01	0.08	4.39E-01	0.24	6.95E-01	0.11
214.1567@8.64003	214.1567	8.64	POS	RP				1.74E-01	4.69E-02	-0.59	3.39E-01	0.26	8.80E-01	-0.04	4.54E-01	0.23
215.1524@0.53830296	215.1524	0.54	POS	hilic				3.04E-01	2.55E-01	0.28	9.63E-01	-0.01	7.29E-02	0.40	4.39E-02	0.41
215.1525@10.3757715	215.1525	10.38	POS	RP				7.70E-01	4.47E-01	-0.17	2.45E-01	-0.26	2.06E-01	-0.26	9.48E-01	-0.02
215.1528@0.5114667	215.1528	0.51	POS	hilic				8.69E-01	6.20E-01	0.12	8.51E-01	-0.03	6.33E-01	0.10	2.83E-01	0.22
215.189@6.9020944	215.1890	6.90	POS	RP				8.42E-01	7.28E-01	0.08	8.61E-01	-0.03	2.39E-01	-0.20	8.37E-01	0.04
215.8869@0.9300865	215.8869	0.93	NEG	hilic				3.11E-01	1.63E-01	0.38	2.69E-01	-0.27	2.82E-01	-0.32	4.84E-01	0.23
215.9155@4.5710444	215.9155	4.57	POS	hilic				8.42E-01	4.89E-01	-0.20	4.99E-01	-0.20	5.41E-01	-0.15	6.11E-01	0.15
216.0091@3.8289502	216.0091	3.83	NEG	RP				5.46E-03	8.56E-03	0.74	7.54E-01	0.05	2.47E-01	0.21	1.01E-01	-0.17
216.0093@0.45852783	216.0093	0.46	NEG	hilic				4.99E-03	3.38E-03	0.85	1.25E-01	0.26	1.81E-01	0.27	3.62E-01	-0.10
216.0399@4.3152885	216.0399	4.32	NEG	hilic				2.86E-02	1.40E-03	1.08	5.09E-01	0.16	3.05E-02	0.53	2.69E-01	0.32
216.0399@5.1244497	216.0399	5.12	NEG	hilic				1.58E-01	6.15E-02	-0.54	9.27E-02	0.44	6.73E-01	0.11	9.23E-01	0.03
216.0401@5.45767	216.0401	5.46	NEG	hilic				9.41E-01	7.96E-01	0.08	3.60E-01	0.26	5.30E-01	0.15	9.71E-01	0.01
216.0401@6.2369375	216.0401	6.24	NEG	hilic				3.57E-01	6.04E-01	0.14	1.42E-01	-0.31	8.91E-02	-0.39	3.25E-01	-0.22
216.0746@6.456321	216.0746	6.46	POS	hilic				5.28E-03	2.56E-02	0.85	1.89E-02	0.55	2.31E-03	0.94	2.20E-06	1.20
216.0805@0.9873499	216.0805	0.99	NEG	hilic				7.66E-03	2.54E-01	-0.36	1.21E-02	0.72	1.75E-02	0.70	9.39E-01	-0.02
216.0996@4.364951	216.0996	4.36	POS	RP				8.38E-01	8.75E-01	-0.04	8.30E-01	0.05	2.75E-01	0.31	6.05E-01	0.14
216.1223@5.560302	216.1223	5.56	POS	hilic				7.82E-01	8.79E-01	-0.04	8.39E-01	0.06	1.74E-01	0.40	9.36E-01	0.02
216.1723@8.855814	216.1723	8.86	NEG	RP				6.64E-01	2.02E-01	-0.27	4.39E-01	0.20	6.25E-01	0.11	9.59E-01	0.01
216.1726@8.33587	216.1726	8.34	POS	RP				9.95E-01	9.03E-01	0.03	6.54E-01	-0.12	9.71E-01	-0.01	8.86E-01	-0.04
216.8758@15.336867	216.8758	15.34	NEG	RP				7.71E-01	7.99E-02	0.46	5.43E-01	0.18	9.70E-01	0.01	6.54E-01	0.14
216.9861@4.7771087	216.9861	4.78	POS	hilic				3.21E-01	4.33E-01	-0.24	1.95E-01	0.32	4.18E-01	0.24	1.00E-01	0.53
217.0858@3.961594	217.0858	3.96	POS	hilic				9.18E-01	4.14E-01	-0.25	7.27E-01	0.10	7.92E-01	-0.08	8.05E-01	-0.06
217.1316@1.2558547	217.1316	1.26	POS	RP				1.69E-02	9.40E-01	0.02	1.00E-02	0.78	2.48E-04	0.96	2.11E-01	0.32
217.1318@2.0368545	217.1318	2.04	POS	hilic				1.07E-02	6.68E-01	-0.13	1.49E-02	0.70	2.93E-04	0.95	4.00E-01	0.21
217.1318@5.7212896	217.1318	5.72	POS	RP				7.76E-01	6.63E-01	-0.13	6.66E-01	0.12	3.52E-01	0.32	3.58E-01	0.27
217.8604@10.712858	217.8604	10.71	NEG	RP				8.81E-01	9.66E-01	0.01	5.87E-01	0.13	4.81E-01	-0.16	6.26E-01	0.13
217.8608@11.035775	217.8608	11.04	NEG	RP				9.13E-01	3.38E-01	-0.25	9.69E-01	0.01	5.12E-01	-0.17	9.93E-01	0.00
218.0197@5.1919217	218.0197	5.19	POS	hilic				9.30E-02	8.17E-02	-0.54	4.01E-02	0.51	6.38E-01	0.13	4.95E-01	0.20
218.0246@0.45345905	218.0246	0.45	NEG	hilic				1.25E-02	2.30E-03	0.92	1.53E-01	0.32	1.51E-01	0.33	5.65E-01	-0.10
218.0373@5.128968	218.0373	5.13	NEG	hilic				5.50E-02	1.59E-01	-0.37	2.02E-02	0.65	5.80E-01	0.14	1.60E-01	0.37
218.0796@4.5111775	218.0796	4.51	POS	RP				6.36E-01	1.70E-01	-0.32	3.80E-01	-0.22	6.12E-01	0.14	8.60E-01	0.05
218.0899@6.289621	218.0899	6.29	POS	hilic				1.12E-03	1.08E-02	0.90	2.68E-01	0.22	7.35E-04	0.85	2.80E-07	1.09
218.1316@7.548444	218.1316	7.55	POS	RP				6.40E-01	1.91E-01	0.35	6.15E-01	0.13	2.01E-01	0.38	1.22E-01	0.52
218.1665@9.34025	218.1665	9.34	POS	RP				3.61E-01	8.28E-01	0.07	3.63E-01	-0.23	4.76E-01	0.18	8.57E-02	0.52
218.167@8.93753	218.1670	8.94	POS	RP				7.40E-01	5.68E-01	0.16	6.91E-01	0.12	8.20E-01	0.06	2.60E-01	-0.32
218.1672@11.612746	218.1672	11.61	POS	RP				2.85E-01	9.26E-02	-0.47	8.41E-01	-0.06	1.46E-01	-0.41	3.07E-02	-0.69
218.169@11.087286	218.1690	11.09	POS	RP				5.21E-01	2.21E-02	0.55	7.46E-01	0.09	8.57E-01	-0.05	9.53E-01	-0.02
218.188@8.736352	218.1880	8.74	POS	RP				3.19E-01	4.65E-01	-0.18	4.84E-01	-0.19	1.11E-01	0.48	9.72E-01	0.01
219.0409@5.152028	219.0409	5.15	NEG	hilic				7.86E-03	2.89E-03	-0.82	4.82E-02	0.52	8.74E-01	0.04	8.25E-01	0.06
219.1104@1.0735776	219.1104	1.07	NEG	hilic				3.49E-02	1.34E-02	0.80	4.66E-02	0.41	4.51E-01	0.14	9.22E-01	-0.02
219.1111@1.047401	219.1111	1.05	POS	hilic				2.95E-02	2.28E-02	0.86	1.04E-01	0.41	7.17E-02	0.43	3.59E-01	-0.26
219.1395@0.5299815	219.1395	0.53	POS	hilic				8.57E-01	2.51E-01	0.29	9.58E-01	0.01	3.47E-01	0.20	5.50E-01	0.16
220.0039@0.46631747	220.0039	0.47	NEG	hilic				4.09E-01	3.87E-02	-0.50	4.60E-01	-0.20	2.43E-02	-0.54	3.30E-01	-0.25
220.0195@5.1700373	220.0195	5.17	NEG	hilic				5.37E-01	2.46E-01	-0.34	3.07E-01	0.26	3.72E-01	-0.27	9.22E-01	0.03
220.1105@8.229339	220.1105	8.23	POS	RP				6.72E-01	7.63E-01	-0.07	2.54E-01	0.27	2.38E-01	0.35	8.40E-01	0.05
220.8522@3.9273748	220.8522	3.93	NEG	hilic				6.05E-01	2.41E-01	0.33	6.61E-02	0.52	4.28E-01	0.23	1.14E-01	0.38
220.8527@1.090906	220.8527	1.09	NEG	RP				4.86E-01	5.13E-01	-0.19	6.17E-01	0.15	7.08E-02	-0.51	4.16E-01	-0.25
222.019@1.4256324	222.0190	1.43	NEG	hilic				1.48E-02	7.28E-02	0.48	3.38E-02	0.61	1.54E-04	1.20	5.64E-02	0.58
222.0191@1.4263246	222.0191	1.43	NEG	hilic				1.24E-02	5.81E-02	0.51	2.86E-02	0.63	1.19E-04	1.22	4.65E-02	0.60
222.0673@0.5134999	222.0673	0.51	POS	hilic				9.94E-03	2.90E-02	0.63	8.26E-02	-0.31	2.92E-01	-0.21	6.75E-01	-0.09
222.0893@7.4646482	222.0893	7.46	POS	RP				7.14E-01	1.89E-01	-0.43	6.95E-01	0.12	7.83E-01	-0.09	7.20E-01	-0.10
222.0897@7.1801906	222.0897	7.18	POS	RP				8.69E-01	3.40E-01	0.30	2.62E-01	0.27	7.65E-01	0.09	3.29E-01	0.32
222.1096@0.49761802	222.1096	0.50	POS	hilic				3.10E-01	9.95E-02	-0.37	5.63E-01	-0.14	2.25E-01	0.33	7.94E-01	0.06
222.1262@7.2539663	222.1262	7.25	NEG	RP				7.64E-01	3.03E-01	0.30	3.17E-01	0.25	6.72E-01	0.11	7.20E-01	-0.08
222.1621@0.42719573	222.1621	0.43	NEG	hilic				5.75E-02	2.03E-02	-0.40	4.15E-02	0.51	4.42E-01	0.20	6.19E-01	0.11
222.1984@8.625105	222.1984	8.63	POS	RP				1.38E-01	8.59E-02	-0.21	8.59E-02	-0.21	2.42E-01	0.27	5.81E-01	-0.09
223.0567@6.739915	223.0567	6.74	NEG	hilic				4.69E-01	9.60E-02	-0.58	8.06E-01	0.07	5.20E-01	-0.16	8.59E-01	0.06
223.1215@5.354837	223.1215	5.35	POS	hilic	4-Hydroxybenzoylcholine	10 eV: 224.1272 (100), 165.0525 (20)	2	7.29E-01	6.85E-01	0.13	9.31E-01	0.03	3.02E-01	-0.30	4.78E-01	0.24
223.1788@5.844018	223.1788	5.84	POS	RP				8.77E-01	2.39E-01	0.29	3.26E-01	0.28	4.78E-01	0.18	2.95E-01	0.33
223.1939@9.391043	223.1939	9.39	POS	RP				9.91E-01	9.09E-01	0.03	7.21E-01	-0.10	6.54E-01	-0.13	7.74E-01	-0.10
223.8372@15.359009	223.8372	15.36	NEG	RP				8.70E-02	6.35E-03	-0.75	2.22E-02	-0.67	8.34E-01	0.06	1.82E-01	-0.39
223.9907@1.8742659	223.9907	1.87	NEG	hilic				1.39E-03	1.75E-03	-0.84	1.27E-01	0.44	8.61E-02	0.50	3.47E-01	-0.22
224.0269@3.2428603	224.0269	3.24	POS	hilic				8.19E-01	6.99E-01	0.12	1.67E-01	0.43	4.69E-01	0.17	5.10E-01	0.21
224.0273@3.268991	224.0273	3.27	POS	hilic				7.99E-01	5.78E-01	-0.19	3.24E-01	0.28	7.64E-01	-0.07	7.97E-01	-0.08

Supplementary table 2: Metabolomics analysis results

Compound	Mass	Retention Time	Ionization mode	column	Compound_ID	MSMS_spectra	ID_level	ANOVA	Anorexia vs control		overweight vs control		obese vs control		athletes vs control	
								P-FDR	P	Estimate	P	Estimate	P	Estimate	P	Estimate
224.057@0.5225678	224.0570	0.52	POS	hilic				3.10E-01	4.50E-02	-0.67	7.46E-01	0.08	8.25E-01	-0.07	2.37E-01	-0.31
224.1403@9.1213665	224.1403	9.12	POS	RP				1.91E-02	1.97E-03	-0.69	9.99E-01	0.00	1.30E-01	0.42	4.32E-01	0.22
224.1411@7.559844	224.1411	7.56	POS	RP				3.11E-01	6.13E-01	0.13	2.25E-01	0.37	2.57E-01	0.33	3.39E-03	0.76
224.1886@7.693085	224.1886	7.69	POS	RP				2.12E-01	1.45E-01	0.42	9.28E-01	0.03	6.49E-01	0.15	2.91E-03	0.76
225.0165@3.8287756	225.0165	3.83	POS	hilic				5.33E-01	2.22E-01	-0.32	3.84E-01	-0.23	3.68E-01	0.28	6.92E-01	0.10
225.0379@5.0145373	225.0379	5.01	POS	hilic				3.94E-01	4.64E-01	-0.24	2.11E-01	0.38	7.96E-02	0.46	4.80E-01	0.20
225.0758@1.6935135	225.0758	1.69	POS	hilic				7.60E-01	7.99E-01	-0.08	3.48E-01	0.23	5.60E-01	0.17	3.57E-01	-0.23
225.1731@10.626313	225.1731	10.63	POS	RP				7.89E-01	2.81E-01	-0.26	3.65E-01	-0.25	1.19E-01	-0.44	6.06E-01	-0.17
225.1732@7.3216753	225.1732	7.32	POS	RP				7.36E-01	4.86E-01	-0.17	2.11E-01	-0.39	1.18E-01	-0.46	3.72E-01	-0.27
225.1733@10.619044	225.1733	10.62	POS	RP				8.24E-01	3.09E-01	-0.26	2.16E-01	-0.36	2.23E-01	-0.33	5.69E-01	-0.17
225.1735@0.42187846	225.1735	0.42	POS	hilic				7.50E-01	6.57E-01	-0.11	8.45E-02	-0.43	4.75E-01	-0.20	4.65E-01	-0.20
225.1741@0.4165615	225.1741	0.42	POS	hilic				8.01E-01	2.38E-01	-0.33	2.05E-01	-0.35	2.22E-01	-0.33	2.59E-01	-0.38
225.9444@4.34829	225.9444	4.35	POS	hilic				7.50E-01	1.09E-01	0.45	2.97E-01	0.30	7.88E-01	0.07	8.23E-01	0.07
225.9446@5.354539	225.9446	5.35	POS	hilic				7.02E-01	6.87E-01	-0.13	5.36E-01	0.17	1.72E-01	-0.37	9.86E-01	0.00
225.9873@4.823372	225.9873	4.82	NEG	hilic				9.70E-02	2.81E-01	0.30	3.73E-02	-0.50	2.90E-01	-0.28	3.72E-01	0.26
226.0059@1.5601476	226.0059	1.56	NEG	hilic				2.40E-01	9.26E-01	-0.02	3.25E-01	0.30	5.26E-03	0.76	3.60E-01	0.29
226.0239@0.5184696	226.0239	0.52	NEG	hilic				9.54E-01	9.11E-01	-0.04	4.00E-01	-0.20	6.79E-01	-0.11	3.68E-01	-0.20
226.0682@5.413812	226.0682	5.41	NEG	hilic				7.16E-01	1.25E-01	0.39	9.01E-01	0.03	9.96E-01	0.00	9.17E-01	0.03
226.0684@5.210883	226.0684	5.21	NEG	hilic				2.67E-01	1.92E-01	-0.36	6.82E-02	0.51	5.42E-01	0.17	6.72E-01	0.12
226.0685@5.2111278	226.0685	5.21	NEG	hilic				2.64E-01	1.93E-01	-0.36	6.71E-02	0.51	5.18E-01	0.18	6.67E-01	0.12
226.07@0.80024004	226.0700	0.80	NEG	hilic				4.15E-02	1.14E-02	0.76	6.72E-01	0.10	3.83E-01	0.21	1.79E-01	-0.28
226.1181@1.2514691	226.1181	1.25	POS	hilic				7.77E-02	8.76E-02	-0.53	7.38E-02	0.56	7.26E-01	0.10	8.35E-01	-0.06
226.1208@7.9325833	226.1208	7.93	POS	RP				1.87E-01	2.89E-02	-0.64	1.32E-01	-0.47	9.07E-01	-0.03	5.98E-01	0.15
226.8057@10.78634	226.8057	10.79	NEG	RP				7.97E-01	7.11E-01	0.13	6.28E-01	0.14	1.57E-01	0.40	2.61E-01	0.35
226.9045@13.251697	226.9045	13.25	NEG	RP				3.54E-01	2.06E-01	0.33	2.96E-02	0.67	8.93E-01	0.03	3.25E-01	0.35
227.0715@5.237075	227.0715	5.24	NEG	hilic				6.76E-01	4.20E-01	-0.20	2.35E-01	0.34	8.39E-01	-0.06	6.96E-01	0.11
227.1888@10.307817	227.1888	10.31	POS	RP				6.27E-01	9.48E-01	-0.02	7.00E-01	-0.10	8.05E-02	-0.42	2.72E-01	-0.31
227.1888@7.437212	227.1888	7.44	POS	RP				7.65E-01	4.95E-01	-0.18	2.28E-01	-0.37	1.93E-01	-0.38	8.71E-01	-0.05
227.1889@0.42257273	227.1889	0.42	POS	hilic				7.71E-01	6.27E-01	-0.12	1.65E-01	-0.41	2.22E-01	-0.34	3.46E-01	-0.32
227.1889@10.299089	227.1889	10.30	POS	RP				5.07E-01	1.81E-02	-0.52	4.40E-01	-0.19	2.17E-01	-0.30	5.16E-01	-0.17
227.1891@8.441606	227.1891	8.44	POS	RP				9.88E-01	6.87E-01	0.12	7.88E-01	-0.08	9.15E-01	-0.04	8.37E-01	-0.05
227.1891@9.933624	227.1891	9.93	POS	RP				4.23E-01	4.40E-02	-0.30	3.47E-01	-0.18	2.03E-01	-0.21	4.40E-02	-0.30
227.1892@10.67298	227.1892	10.67	POS	RP				8.95E-01	7.17E-01	-0.10	2.95E-01	-0.31	2.88E-01	-0.30	5.16E-01	-0.21
227.1894@10.602895	227.1894	10.60	POS	RP	Myristic acid	10 eV: 175.1475 (100), 95.0851 (75), 193.1589 (70), 228.1959 (25)	1	9.27E-01	6.18E-01	-0.14	3.74E-01	-0.26	3.19E-01	-0.28	4.89E-01	-0.22
227.1901@10.336824	227.1901	10.34	POS	RP				9.18E-01	5.22E-01	0.17	5.20E-01	0.17	8.64E-01	-0.04	4.78E-01	0.21
227.2252@9.84253	227.2252	9.84	POS	RP				7.31E-01	9.95E-01	0.00	8.51E-01	-0.05	6.71E-01	0.11	2.29E-01	-0.41
228.0145@8.949769	228.0145	8.95	NEG	RP				8.41E-01	3.39E-01	-0.32	8.68E-01	-0.05	7.79E-01	0.08	4.25E-01	-0.22
228.0992@0.47977075	228.0992	0.48	POS	hilic				3.79E-01	8.69E-03	-0.61	2.81E-01	-0.29	2.05E-01	-0.31	1.13E-01	-0.39
228.1113@6.02565	228.1113	6.03	POS	hilic				5.40E-01	4.67E-01	0.23	4.64E-01	-0.19	2.97E-01	-0.31	4.24E-01	0.25
228.1373@7.1039686	228.1373	7.10	POS	RP				9.05E-01	3.35E-01	-0.26	5.01E-01	-0.19	8.47E-01	0.06	7.10E-01	-0.11
228.1477@4.0302577	228.1477	4.03	POS	hilic				1.99E-02	3.91E-01	-0.30	3.33E-03	0.81	7.46E-01	-0.08	1.28E-01	0.41
228.1477@4.83667	228.1477	4.84	POS	hilic				1.88E-02	1.80E-02	-0.69	1.43E-01	0.41	1.49E-01	-0.38	3.84E-01	0.25
228.2088@10.318144	228.2088	10.32	NEG	RP				8.83E-03	6.57E-03	-0.77	1.67E-01	0.42	1.50E-01	0.36	3.91E-01	-0.23
229.0445@1.8608526	229.0445	1.86	NEG	hilic				2.50E-01	4.96E-01	0.24	6.18E-02	-0.49	6.19E-01	0.14	3.10E-01	0.23
229.0445@2.6971626	229.0445	2.70	NEG	hilic				4.10E-01	6.00E-02	0.47	3.92E-01	-0.26	5.19E-01	0.23	4.76E-01	0.19
229.0888@5.338535	229.0888	5.34	POS	hilic				5.73E-01	7.80E-01	-0.06	4.59E-01	0.18	1.79E-01	0.33	1.25E-01	0.37
229.1686@0.46017075	229.1686	0.46	POS	hilic				8.00E-01	8.48E-01	-0.05	5.01E-01	0.17	5.57E-01	-0.13	4.57E-01	-0.16
229.1688@6.73462	229.1688	6.73	POS	RP				6.60E-01	5.10E-02	-0.28	9.65E-01	-0.01	2.63E-01	-0.19	8.85E-01	-0.03
229.1692@0.4548182	229.1692	0.45	POS	hilic				6.40E-01	8.30E-01	0.06	5.32E-01	-0.13	6.26E-02	-0.33	3.64E-01	-0.19
229.2401@0.51523954	229.2401	0.52	POS	hilic				8.81E-01	5.37E-01	-0.17	8.24E-01	0.07	8.25E-01	0.06	4.07E-01	-0.24
229.8829@15.281984	229.8829	15.28	POS	RP				2.00E-01	9.02E-02	-0.55	4.33E-01	-0.25	3.58E-01	-0.28	5.85E-03	-0.83
230.0242@0.4479999	230.0242	0.45	NEG	hilic				9.84E-01	7.20E-01	0.10	9.39E-01	-0.02	7.11E-01	-0.09	8.82E-01	-0.04
230.0604@0.45645612	230.0604	0.46	NEG	hilic				1.73E-01	9.80E-02	-0.43	7.80E-02	-0.38	4.53E-02	-0.48	5.93E-01	0.15
230.061@6.3709526	230.0610	6.37	NEG	RP				2.84E-01	1.24E-01	-0.41	5.65E-02	-0.44	4.47E-02	-0.48	9.10E-01	0.03
230.0762@0.9897299	230.0762	0.99	NEG	hilic				5.43E-01	2.66E-01	-0.24	7.44E-01	0.08	3.06E-01	0.26	5.15E-01	-0.14
230.0767@2.4171083	230.0767	2.42	POS	hilic				9.58E-01	7.12E-01	0.11	5.96E-01	-0.14	1.00E+00	0.00	7.78E-01	0.07
230.1318@15.315142	230.1318	15.32	POS	RP				9.01E-01	4.04E-01	0.25	4.17E-01	0.25	6.74E-01	0.13	2.56E-01	0.33
230.868@0.5464957	230.8680	0.55	NEG	hilic				1.73E-01	4.50E-01	0.26	2.98E-01	0.28	8.90E-02	0.49	8.81E-02	-0.40
231.1472@1.5561537	231.1472	1.56	POS	hilic	Isobutyryl carnitine	10 eV: 232.1551 (100), 85.0278 (70), 173.0800 (30)	2	1.65E-01	8.60E-02	0.55	3.85E-03	0.86	3.70E-02	0.50	6.55E-02	0.58
231.1473@1.9131881	231.1473	1.91	POS	RP				1.73E-01	7.41E-02	0.55	4.28E-03	0.80	2.85E-01	0.27	6.07E-02	0.62
231.1473@6.54606	231.1473	6.55	POS	RP				8.70E-01	5.47E-01	-0.18	9.36E-01	0.02	5.60E-01	0.19	4.96E-01	0.20
231.1475@1.4404123	231.1475	1.44	POS	RP				2.78E-01	3.95E-02	0.61	8.49E-03	0.73	1.88E-01	0.34	3.90E-01	0.29
231.9785@0.50949997	231.9785	0.51	POS	hilic				8.64E-01	9.20E-01	-0.03	3.76E-01	0.24	6.16E-01	-0.16	9.93E-01	0.00
231.9931@15.303086	231.9931	15.30	NEG	RP				7.10E-01	5.16E-01	-0.20	2.62E-01	0.29	5.56E-01	-0.17	8.15E-01	0.07
231.9932@5.497239	231.9932	5.50	NEG	RP				7.31E-01	2.69E-01	-0.37	8.44E-01	0.06	9.20E-01	0.03	3.55E-01	-0.29
232.0039@3.4948418	232.0039	3.49	NEG	RP				6.04E-01	4.95E-01	0.19	5.11E-01	-0.14	2.18E-01	-0.26	4.42E-01	-0.17
232.0041@0.47700003	232.0041	0.48	NEG	hilic				6.10E-01	7.09E-01	0.11	2.67E-01	-0.23	1.65E-01	-0.31	3.20E-01	-0.22
232.038@0.4446	232.0380	0.44	NEG	hilic				4.78E-01	5.20E-01	0.14	2.31E-01	0.30	3.94E-01	-0.17	5.82E-01	-0.11
232.0534@3.3176758	232.0534	3.32	POS	RP				1.30E-01	2.95E-01	-0.32	1.57E-02	0.63	4.36E-01	0.24	8.31E-01	-0.06
232.1407@15.297229	232.1407	15.30	POS	RP				9.04E-01	7.07E-01	0.12	8.60E-01	-0.06	2.96E-01	0.29	9.21E-01	0.02
232.1827@11.692184	232.1827	11.69	POS	RP				7.36E-01	1.40E-01	0.46	3.74E-01	0.22	6.02E-01	0.12	5.40E-01	0.15
232.9386@6.0780616	232.9386	6.08	NEG	hilic				5.89E-01	2.16E-01	0.35	7.34E-01	-0.10	9.10E-01	0.04	1.46E-01	0.42
232.9386@6.225939	232.9386	6.23	NEG	hilic				2.78E-01	2.38E-02	0.63	1.18E-01	0.47	5.82E-02	0.54	2.68E-02	0.73

Supplementary table 2: Metabolomics analysis results

Compound	Mass	Retention Time	Ionization mode	column	Compound_ID	MSMS_spectra	ID_level	ANOVA	Anorexia vs control		overweight vs control		obese vs control		athletes vs control	
								P-FDR	P	Estimate	P	Estimate	P	Estimate	P	Estimate
232.939@6.2254906	232.9390	6.23	NEG	hilic				3.56E-01	1.19E-01	0.45	3.03E-01	0.32	1.91E-01	0.36	1.04E-02	0.75
234.1619@8.869179	234.1619	8.87	NEG	RP				7.34E-01	2.64E-01	0.41	1.11E-01	0.38	2.74E-01	0.32	1.64E-01	0.43
234.1624@8.840513	234.1624	8.84	POS	RP				3.82E-01	1.80E-01	-0.43	9.45E-01	0.02	1.67E-01	0.38	6.67E-01	0.14
234.8696@15.333877	234.8696	15.33	NEG	RP				5.97E-01	4.67E-01	0.22	8.99E-01	-0.03	9.43E-02	0.49	9.15E-01	0.03
235.1061@4.511271	235.1061	4.51	POS	RP				5.38E-01	2.98E-01	-0.30	4.12E-01	-0.26	4.21E-01	0.26	5.89E-01	0.16
235.155@10.684142	235.1550	10.68	POS	RP				7.91E-01	4.61E-01	-0.17	6.73E-01	-0.10	1.63E-01	-0.30	8.89E-01	0.03
236.0155@4.8217654	236.0155	4.82	NEG	hilic				8.31E-01	7.28E-01	0.09	3.23E-01	-0.28	8.32E-01	0.07	6.30E-01	-0.14
236.0689@5.503587	236.0689	5.50	POS	RP				6.85E-01	4.35E-01	-0.17	8.30E-01	-0.05	3.12E-01	0.26	5.75E-01	0.15
236.1168@6.2942266	236.1168	6.29	POS	RP				5.58E-01	5.54E-01	-0.16	7.31E-01	-0.09	5.32E-02	-0.47	8.95E-01	0.03
236.1169@0.55278933	236.1169	0.55	POS	hilic				5.54E-01	6.09E-01	-0.15	8.96E-01	-0.04	2.62E-02	-0.53	6.70E-01	-0.12
236.1404@9.232895	236.1404	9.23	POS	RP				3.16E-01	1.79E-01	-0.38	4.46E-01	0.22	3.19E-01	0.24	3.24E-01	-0.26
236.1413@8.132318	236.1413	8.13	POS	RP				5.37E-02	1.63E-02	0.67	5.64E-01	-0.16	4.36E-01	-0.23	6.10E-02	0.52
236.1416@9.533872	236.1416	9.53	POS	RP				9.78E-01	4.71E-01	-0.22	5.87E-01	-0.15	6.76E-01	-0.13	6.14E-01	-0.17
236.1419@8.2545395	236.1419	8.25	POS	RP				5.05E-01	7.16E-01	-0.12	8.44E-02	0.47	6.25E-01	0.12	7.66E-01	-0.09
237.016@1.0268463	237.0160	1.03	NEG	hilic				3.78E-01	1.64E-01	-0.36	6.33E-02	-0.42	2.81E-02	-0.50	6.09E-01	-0.13
237.2096@8.805989	237.2096	8.81	POS	RP				6.88E-01	6.04E-01	0.15	3.22E-01	-0.27	8.80E-01	-0.05	3.49E-01	0.27
237.21@9.710166	237.2100	9.71	POS	RP				6.51E-01	7.83E-01	-0.08	5.38E-01	-0.18	6.18E-02	-0.55	4.72E-01	-0.21
237.8773@15.258869	237.8773	15.26	POS	RP				3.41E-01	9.19E-02	-0.50	9.52E-02	-0.57	3.67E-02	-0.59	2.57E-02	-0.65
238.0835@5.1731925	238.0835	5.17	POS	RP				5.52E-01	5.64E-01	-0.19	7.82E-01	0.07	4.87E-02	-0.45	9.78E-01	0.01
238.1548@8.33746	238.1548	8.34	POS	RP				9.61E-01	8.84E-01	-0.04	7.31E-01	-0.09	6.96E-01	-0.14	3.32E-01	-0.26
238.1549@8.274805	238.1549	8.27	POS	RP				9.62E-01	4.45E-01	-0.21	6.59E-01	-0.11	5.00E-01	-0.25	6.09E-01	-0.16
238.1554@0.4486	238.1554	0.45	POS	hilic				6.67E-01	9.07E-01	-0.02	9.08E-01	0.03	8.96E-01	0.03	1.64E-01	0.38
238.1573@9.388292	238.1573	9.39	POS	RP				3.52E-01	1.63E-01	-0.38	9.45E-02	0.43	7.74E-01	-0.07	9.92E-01	0.00
239.0951@1.7257519	239.0951	1.73	POS	hilic				2.58E-01	1.87E-01	-0.42	9.21E-01	-0.02	2.25E-01	0.36	1.96E-01	-0.37
239.1393@0.49770007	239.1393	0.50	POS	hilic				7.37E-01	2.63E-01	-0.37	9.35E-01	-0.02	6.93E-01	0.11	6.97E-01	0.10
240.0616@4.51118	240.0616	4.51	POS	RP				6.57E-01	1.66E-01	-0.32	3.66E-01	-0.23	6.25E-01	0.14	9.65E-01	0.01
240.0862@0.87476575	240.0862	0.87	POS	hilic				2.26E-01	1.30E-02	-0.69	4.95E-02	-0.53	6.81E-02	-0.50	1.64E-01	-0.38
240.0992@7.476539	240.0992	7.48	NEG	RP	3-Carboxy-4-methyl-5-propyl-2-furanpropionic acid (CMPF)	10 eV: 195.1032 (100), 239.0925 (45), 151.1134 (40)	2	7.01E-01	1.65E-01	-0.44	7.58E-01	0.09	7.75E-01	-0.09	6.74E-01	-0.10
240.1005@7.4636617	240.1005	7.46	POS	RP				7.90E-01	2.25E-01	-0.35	8.58E-01	0.05	9.11E-01	-0.03	4.97E-01	-0.17
240.1356@0.43331257	240.1356	0.43	NEG	hilic				8.55E-01	6.32E-01	0.13	8.28E-01	-0.04	3.57E-01	0.23	4.05E-01	0.20
240.1363@7.933704	240.1363	7.93	NEG	RP				8.25E-01	9.79E-01	0.01	5.58E-01	-0.15	4.59E-01	0.23	4.33E-01	0.23
240.948@3.1363237	240.9480	3.14	NEG	hilic				2.59E-01	3.52E-03	0.79	6.03E-01	0.17	2.72E-01	0.28	6.09E-02	0.52
240.948@3.2618628	240.9480	3.26	NEG	hilic				9.32E-01	8.54E-01	-0.06	7.31E-01	0.11	7.35E-01	0.09	3.80E-01	0.27
241.0173@6.12406	241.0173	6.12	NEG	hilic				7.15E-01	5.89E-01	-0.17	1.17E-01	-0.43	1.80E-01	-0.44	4.67E-01	-0.21
241.055@3.3163671	241.0550	3.32	POS	RP				3.36E-01	4.88E-01	-0.22	3.87E-02	0.53	5.28E-01	0.20	7.30E-01	-0.09
241.0717@3.0147908	241.0717	3.01	POS	hilic				2.18E-01	3.28E-01	-0.21	1.01E-01	0.43	5.82E-01	0.13	4.20E-01	-0.17
241.1675@0.45468757	241.1675	0.45	POS	hilic				5.24E-01	1.41E-01	-0.37	9.16E-02	-0.44	3.52E-01	-0.24	1.07E-01	-0.42
241.1678@0.46860433	241.1678	0.47	POS	hilic				7.64E-01	1.74E-01	-0.40	6.53E-01	-0.14	9.83E-01	0.01	9.25E-01	0.03
241.204@0.43668422	241.2040	0.44	POS	hilic				6.38E-01	2.00E-01	-0.22	5.79E-01	-0.11	4.79E-02	-0.30	4.76E-01	-0.13
241.2044@7.950726	241.2044	7.95	POS	RP				6.18E-01	8.99E-01	-0.03	6.69E-01	0.11	3.45E-01	-0.22	2.74E-01	0.30
241.2045@10.670545	241.2045	10.67	POS	RP				8.60E-01	3.03E-01	-0.26	3.21E-01	-0.29	2.13E-01	-0.35	3.41E-01	-0.32
241.2046@10.603074	241.2046	10.60	POS	RP				7.66E-01	1.97E-01	-0.32	2.06E-01	-0.37	1.52E-01	-0.41	2.65E-01	-0.37
241.2775@7.2938824	241.2775	7.29	POS	RP				3.52E-01	8.24E-02	0.57	7.54E-02	0.46	8.68E-01	-0.05	1.31E-01	0.42
242.0462@3.958951	242.0462	3.96	POS	hilic				6.91E-01	2.18E-01	-0.35	7.06E-01	0.12	3.86E-01	0.22	8.61E-01	-0.06
242.1268@1.7296154	242.1268	1.73	POS	hilic				4.14E-01	2.11E-01	0.36	3.41E-02	0.45	2.23E-01	0.25	8.82E-01	0.03
242.176@1.8719615	242.1760	1.87	POS	hilic				2.44E-01	1.92E-02	0.69	5.47E-01	0.13	5.73E-01	0.12	3.06E-01	0.25
242.1882@9.318319	242.1882	9.32	NEG	RP				7.08E-01	2.40E-01	-0.26	4.34E-01	0.20	8.14E-01	0.06	7.41E-01	0.09
242.1885@9.445284	242.1885	9.45	POS	RP				1.68E-02	8.26E-02	-0.47	2.92E-02	0.56	5.74E-01	0.14	5.59E-02	0.63
242.1927@0.4397778	242.1927	0.44	POS	hilic				8.00E-01	3.92E-01	0.16	8.81E-01	0.03	3.82E-01	-0.11	5.31E-01	0.13
242.2242@10.526534	242.2242	10.53	NEG	RP				9.91E-02	2.44E-02	-0.61	7.37E-01	0.10	4.22E-01	-0.21	8.93E-03	-0.65
242.8005@10.712629	242.8005	10.71	NEG	RP				1.09E-01	7.17E-02	-0.59	5.96E-01	-0.14	1.56E-01	0.38	1.41E-01	-0.39
242.9058@0.935215	242.9058	0.94	NEG	hilic				3.27E-01	3.93E-01	0.24	1.56E-01	-0.37	1.30E-01	-0.47	8.95E-01	0.04
243.0585@5.1654344	243.0585	5.17	NEG	hilic				1.42E-01	6.12E-03	-0.55	9.85E-01	0.00	9.90E-01	0.00	1.53E-02	-0.51
243.147@1.4366338	243.1470	1.44	POS	hilic				9.12E-02	7.00E-01	-0.12	2.11E-01	0.33	6.24E-01	0.14	1.82E-03	0.81
243.183@10.691823	243.1830	10.69	NEG	RP				8.89E-01	2.82E-01	-0.25	4.30E-01	-0.21	3.73E-01	-0.22	8.93E-01	-0.04
243.1835@0.47563246	243.1835	0.48	POS	hilic				8.81E-01	6.69E-01	-0.12	2.55E-01	-0.36	3.89E-01	-0.22	8.01E-01	-0.08
243.1836@0.41858983	243.1836	0.42	POS	hilic				6.27E-01	3.04E-01	-0.26	8.34E-02	-0.53	1.39E-01	-0.43	2.16E-01	-0.39
243.1837@10.673595	243.1837	10.67	POS	RP				7.66E-01	2.73E-01	-0.27	1.54E-01	-0.41	1.74E-01	-0.39	2.94E-01	-0.35
243.1837@7.321949	243.1837	7.32	POS	RP				6.96E-01	3.44E-01	-0.23	1.71E-01	-0.43	8.68E-02	-0.48	2.91E-01	-0.32
243.1837@7.3333635	243.1837	7.33	NEG	RP				6.74E-01	3.15E-01	-0.24	1.98E-01	-0.37	6.47E-02	-0.48	3.70E-01	-0.25
243.184@0.7358001	243.1840	0.74	POS	hilic				7.50E-01	1.85E-01	-0.30	1.89E-01	-0.31	5.52E-01	-0.14	5.52E-01	-0.14
243.184@10.646839	243.1840	10.65	POS	RP				4.44E-01	5.56E-02	-0.60	1.03E-01	-0.46	2.92E-01	-0.32	6.83E-02	-0.58
243.2199@8.6264715	243.2199	8.63	POS	RP				6.64E-01	9.23E-01	0.02	6.92E-01	-0.12	1.96E-01	0.42	6.28E-01	0.13
243.22@8.539775	243.2200	8.54	POS	RP				6.58E-01	9.89E-01	0.00	7.10E-01	-0.10	1.99E-01	0.42	6.68E-01	0.11
243.2565@7.9032207	243.2565	7.90	POS	RP				4.87E-04	9.16E-02	-0.46	3.55E-04	-0.77	8.94E-04	-0.79	1.48E-06	-1.04
243.2568@0.5868302	243.2568	0.59	POS	hilic				8.78E-06	2.63E-02	-0.61	2.22E-05	-0.90	6.63E-05	-0.99	8.68E-10	-1.36
243.9938@2.470572	243.9938	2.47	NEG	hilic				9.18E-01	6.02E-01	-0.15	4.76E-01	0.18	9.45E-01	0.02	8.67E-01	0.04
244.0691@1.5444874	244.0691	1.54	NEG	hilic				4.86E-01	6.23E-01	0.15	1.78E-01	0.35	8.01E-03	0.64	6.21E-01	0.15
244.0692@3.7498028	244.0692	3.75	NEG	hilic				5.89E-02	7.95E-01	-0.08	4.79E-01	0.19	8.64E-02	-0.47	9.49E-03	-0.78
244.0695@1.1377474	244.0695	1.14	NEG	RP				6.39E-01	6.56E-01	-0.14	9.23E-01	0.03	6.37E-02	0.42	8.49E-01	-0.05
244.2194@10.305549	244.2194	10.31	POS	RP				5.45E-02	2.00E-01	-0.24	3.15E-01	0.24	1.07E-02	0.67	7.48E-01	0.07
244.9067@0.93443364	244.9067	0.93	NEG	hilic				2.23E-01	8.83E-01	0.05	2.01E-02	-0.62	2.56E-01	-0.32	6.38E-01	0.13
245.1019@3.8386056	245.1019	3.84	POS	hilic				2.30E-01	9.31E-01	-0.02	4.16E-01	-0.17	3.88E-01	-0.17	9.21E-02	0.43

Supplementary table 2: Metabolomics analysis results

Compound	Mass	Retention Time	Ionization mode	column	Compound_ID	MSMS_spectra	ID_level	ANOVA	Anorexia vs control		overweight vs control		obese vs control		athletes vs control	
								P-FDR	P	Estimate	P	Estimate	P	Estimate	P	Estimate
245.1628@2.7117484	245.1628	2.71	POS	RP				1.86E-02	4.72E-01	-0.19	8.17E-03	0.64	6.67E-03	0.87	2.26E-01	0.36
245.163@1.2638462	245.1630	1.26	POS	hilic				3.25E-03	5.84E-02	-0.59	1.78E-02	0.57	1.68E-02	0.74	4.71E-01	0.18
245.1632@7.105544	245.1632	7.11	POS	RP				7.63E-01	2.19E-01	-0.33	4.75E-01	-0.18	6.90E-01	0.11	9.10E-01	-0.03
245.1633@2.7107701	245.1633	2.71	POS	RP				7.43E-02	2.52E-01	-0.29	1.54E-02	0.55	5.75E-02	0.64	5.68E-01	0.17
245.1989@1.2457827	245.1989	1.25	POS	hilic				9.01E-01	7.23E-01	-0.09	5.22E-01	-0.15	5.74E-01	0.14	9.87E-01	0.00
245.856@15.281119	245.8560	15.28	POS	RP				2.05E-01	1.25E-01	0.45	1.38E-01	0.49	1.00E-02	0.65	4.41E-03	0.81
245.9153@0.42377034	245.9153	0.42	NEG	hilic				6.42E-01	7.66E-02	0.59	3.12E-01	0.29	3.31E-01	0.28	4.91E-01	0.21
245.9925@15.324702	245.9925	15.32	NEG	RP				6.87E-01	1.91E-01	-0.41	2.01E-01	-0.35	7.24E-01	-0.11	1.18E-01	-0.45
246.0194@0.4497714	246.0194	0.45	NEG	hilic				8.60E-01	7.57E-01	-0.08	4.66E-01	-0.17	2.10E-01	-0.26	2.78E-01	-0.23
246.0851@1.6519413	246.0851	1.65	NEG	hilic				3.43E-01	7.30E-01	0.10	2.40E-01	0.31	2.07E-01	-0.27	2.83E-01	-0.24
246.1369@2.616434	246.1369	2.62	POS	RP				2.44E-01	4.27E-02	-0.66	1.57E-01	-0.38	1.21E-02	-0.74	3.88E-01	-0.27
246.1371@2.611744	246.1371	2.61	POS	RP				2.08E-01	3.07E-02	-0.71	1.78E-01	-0.36	9.27E-03	-0.74	4.00E-01	-0.27
246.1374@1.4142648	246.1374	1.41	POS	hilic				2.57E-01	4.83E-02	-0.66	1.93E-01	-0.33	1.38E-02	-0.72	4.33E-01	-0.25
246.9023@0.9345088	246.9023	0.93	NEG	hilic				5.01E-01	6.60E-01	0.14	1.32E-01	-0.41	2.32E-01	-0.35	8.10E-01	0.07
247.0456@1.0184429	247.0456	1.02	NEG	hilic				4.37E-01	8.48E-01	-0.05	3.87E-01	-0.21	1.26E-01	-0.40	3.93E-01	0.25
247.142@4.116106	247.1420	4.12	POS	hilic				2.25E-02	3.85E-01	-0.27	3.25E-03	0.78	5.04E-02	0.53	9.64E-01	-0.01
247.2158@0.73381823	247.2158	0.73	POS	hilic				6.50E-01	4.10E-01	0.18	5.41E-01	-0.10	5.10E-01	-0.10	4.79E-01	0.15
247.912@0.42342666	247.9120	0.42	NEG	hilic				8.76E-01	2.62E-01	0.34	3.61E-01	0.25	5.12E-01	0.17	5.70E-01	0.16
247.97@4.7025647	247.9700	4.70	POS	hilic				7.68E-01	3.39E-01	0.29	8.38E-01	0.06	9.62E-01	-0.01	1.72E-01	0.39
248.0541@3.9566755	248.0541	3.96	POS	hilic				7.94E-01	4.44E-01	-0.24	7.40E-01	0.10	5.96E-01	0.15	4.51E-01	0.24
248.1006@1.250344	248.1006	1.25	POS	hilic				1.39E-01	4.53E-02	-0.45	1.39E-01	0.39	6.63E-01	0.10	6.70E-01	-0.11
248.1263@0.47776094	248.1263	0.48	POS	hilic				3.88E-01	6.23E-01	-0.14	8.88E-01	0.04	8.83E-01	0.04	7.95E-02	-0.62
248.1266@4.227633	248.1266	4.23	POS	RP				8.08E-01	5.55E-01	0.17	3.72E-01	0.24	8.43E-01	0.06	5.22E-01	-0.21
248.1761@8.620031	248.1761	8.62	POS	RP				7.35E-01	6.52E-01	-0.10	6.06E-01	-0.13	3.22E-01	0.30	8.71E-01	-0.04
248.1779@8.942768	248.1779	8.94	POS	RP				9.79E-01	8.00E-01	0.08	9.99E-01	0.00	4.83E-01	0.21	8.51E-01	0.07
248.1989@0.43565452	248.1989	0.44	POS	hilic				2.34E-02	2.30E-01	-0.26	6.55E-01	-0.11	4.78E-01	-0.15	9.59E-03	0.69
249.0615@5.11063	249.0615	5.11	NEG	hilic				2.14E-01	2.40E-01	-0.34	1.46E-01	0.39	6.71E-02	0.44	5.66E-01	0.16
249.0979@1.4182043	249.0979	1.42	POS	hilic				2.77E-01	8.04E-02	0.46	2.25E-02	0.64	2.77E-02	0.69	4.25E-01	0.26
249.1716@10.682968	249.1716	10.68	POS	RP				6.08E-01	2.24E-01	-0.28	1.30E-01	-0.40	6.32E-02	-0.45	4.66E-01	-0.20
249.9673@4.4895372	249.9673	4.49	NEG	hilic				7.09E-01	3.23E-01	0.26	1.80E-01	0.44	1.92E-01	0.37	1.54E-01	0.44
249.9674@13.253086	249.9674	13.25	NEG	RP				7.33E-01	3.14E-01	-0.28	6.33E-01	0.15	4.14E-01	-0.21	3.28E-01	-0.29
249.968@0.44332087	249.9680	0.44	NEG	hilic				5.75E-02	6.24E-01	-0.14	2.94E-03	-0.83	5.57E-02	-0.64	1.11E-02	-0.72
250.1211@8.534963	250.1211	8.53	POS	RP				2.07E-01	2.13E-02	-0.70	9.05E-01	0.03	5.16E-01	-0.18	7.22E-02	-0.54
250.1214@7.8980603	250.1214	7.90	POS	RP				9.19E-01	9.15E-01	0.03	9.37E-01	0.02	3.84E-01	-0.27	7.09E-01	-0.11
250.7964@15.346859	250.7964	15.35	NEG	RP				8.26E-01	4.06E-01	0.28	7.31E-01	0.10	7.00E-01	0.10	1.50E-01	0.40
251.0264@1.829198	251.0264	1.83	NEG	hilic				5.11E-01	5.75E-01	0.16	9.63E-02	-0.45	9.12E-01	-0.04	7.72E-01	0.08
251.1519@9.38935	251.1519	9.39	NEG	RP				2.57E-01	1.24E-01	-0.48	4.21E-01	0.21	1.49E-01	0.38	6.57E-01	0.14
251.1596@0.54222226	251.1596	0.54	POS	hilic				6.28E-01	1.75E-01	0.38	5.48E-01	-0.13	9.47E-01	0.02	6.98E-01	-0.10
252.0227@1.073909	252.0227	1.07	NEG	hilic				2.63E-01	1.56E-01	-0.32	8.08E-01	0.06	7.57E-01	0.08	1.92E-02	-0.48
252.1003@7.407949	252.1003	7.41	NEG	RP				7.34E-01	7.23E-01	-0.11	2.25E-01	-0.39	3.96E-01	-0.25	7.80E-01	0.08
252.2088@9.52801	252.2088	9.53	POS	RP				6.06E-01	1.79E-01	-0.40	4.78E-01	0.21	6.66E-01	0.12	9.99E-01	0.00
252.2092@9.446498	252.2092	9.45	POS	RP				8.55E-01	3.78E-01	0.23	9.15E-01	-0.02	8.96E-01	0.03	5.84E-01	-0.12
252.2093@9.526174	252.2093	9.53	POS	RP				2.56E-01	5.13E-02	-0.52	2.56E-01	0.27	9.04E-01	0.03	5.47E-01	0.17
252.2096@9.287863	252.2096	9.29	POS	RP				9.84E-01	6.82E-01	-0.13	8.87E-01	0.04	8.65E-01	0.04	7.76E-01	0.09
252.2097@9.31872	252.2097	9.32	POS	RP				8.56E-01	5.62E-01	-0.14	8.20E-01	0.06	5.38E-01	-0.15	5.15E-01	0.17
252.2101@9.78118	252.2101	9.78	POS	RP				3.01E-01	4.90E-01	0.19	4.10E-01	0.23	1.65E-02	0.68	7.72E-01	-0.09
252.7937@15.347099	252.7937	15.35	NEG	RP				1.89E-01	2.39E-01	0.35	1.63E-01	0.42	4.67E-02	0.60	1.59E-03	0.85
252.9626@4.7794876	252.9626	4.78	POS	hilic				9.13E-01	5.98E-01	-0.13	8.15E-01	-0.06	7.56E-01	0.08	5.56E-01	0.19
253.0956@5.5042744	253.0956	5.50	POS	RP				8.35E-01	7.64E-01	-0.07	7.63E-01	-0.07	3.59E-01	0.25	7.40E-01	0.09
253.1685@8.13196	253.1685	8.13	POS	RP				8.72E-01	3.16E-01	0.27	6.01E-01	0.16	8.19E-01	0.07	3.01E-01	0.35
253.2043@0.41945946	253.2043	0.42	POS	hilic				7.64E-01	1.16E-01	-0.30	8.46E-01	-0.05	8.67E-01	0.04	8.80E-01	0.04
254.0765@1.0683707	254.0765	1.07	NEG	hilic				2.37E-03	4.09E-02	-0.49	4.61E-02	0.54	2.65E-02	0.60	2.06E-01	-0.27
254.1081@0.50645167	254.1081	0.51	POS	hilic				2.84E-01	2.44E-01	0.41	1.19E-01	0.40	3.15E-01	-0.28	6.97E-01	0.09
254.154@0.448147	254.1540	0.45	POS	hilic				8.01E-01	5.18E-01	-0.16	5.62E-01	-0.14	1.71E-01	-0.30	2.09E-01	-0.28
254.2036@8.027161	254.2036	8.03	POS	RP				5.96E-01	1.91E-01	-0.31	7.03E-01	-0.09	9.12E-01	0.03	3.19E-01	0.27
254.2056@10.460656	254.2056	10.46	POS	RP				8.96E-01	2.57E-01	0.38	6.71E-01	0.15	7.13E-01	0.10	6.32E-01	0.14
254.2236@10.430495	254.2236	10.43	POS	RP				1.53E-03	5.86E-01	-0.14	1.12E-03	0.91	1.53E-03	0.84	9.05E-01	0.03
254.2243@10.431939	254.2243	10.43	NEG	RP	FA 16:1	10 eV: 253.3178 (100)	1	2.16E-04	8.49E-03	-0.75	1.71E-02	0.64	1.46E-02	0.66	3.56E-01	-0.24
254.2247@9.352461	254.2247	9.35	POS	RP				6.51E-01	4.93E-01	-0.20	6.40E-01	-0.13	2.28E-01	-0.33	4.26E-01	0.25
254.2249@9.791817	254.2249	9.79	POS	RP				8.93E-01	1.91E-01	0.33	5.35E-01	0.17	4.29E-01	0.24	6.64E-01	0.11
254.225@9.479365	254.2250	9.48	POS	RP				5.27E-02	6.26E-01	0.15	3.93E-02	0.52	1.14E-01	0.39	1.07E-01	-0.39
254.225@9.650946	254.2250	9.65	POS	RP				3.15E-01	2.02E-02	-0.60	7.61E-01	0.08	3.52E-01	-0.25	1.80E-01	-0.41
254.935@15.278869	254.9350	15.28	POS	RP				4.27E-01	3.68E-01	-0.30	1.00E-01	-0.52	5.66E-01	-0.17	5.28E-02	-0.63
254.9367@7.874403	254.9367	7.87	POS	hilic				5.67E-01	1.52E-01	0.47	8.12E-01	0.06	3.59E-01	0.27	8.54E-02	0.50
255.0329@5.7466836	255.0329	5.75	NEG	hilic				1.12E-01	6.35E-01	-0.14	2.70E-01	-0.28	6.86E-01	-0.13	9.87E-03	0.65
255.2197@10.892775	255.2197	10.89	POS	RP				9.48E-01	5.58E-01	-0.14	4.84E-01	-0.17	4.60E-01	-0.18	9.19E-01	-0.03
255.22@10.599878	255.2200	10.60	POS	RP				7.64E-01	9.10E-02	-0.40	4.53E-01	-0.20	6.33E-01	-0.11	8.66E-01	-0.04
255.2201@0.42388883	255.2201	0.42	POS	hilic				9.57E-01	8.94E-01	0.03	8.64E-01	-0.04	7.60E-01	0.07	4.88E-01	0.18
255.2202@8.398077	255.2202	8.40	POS	RP				9.97E-01	8.63E-01	-0.03	9.24E-01	-0.02	6.60E-01	-0.08	9.37E-01	-0.02
255.2212@10.642144	255.2212	10.64	POS	RP				9.00E-01	2.25E-01	-0.29	5.60E-01	-0.15	6.86E-01	-0.10	9.73E-01	-0.01
255.256@0.48280847	255.2560	0.48	POS	hilic				9.11E-01	8.66E-01	0.04	6.58E-01	-0.10	7.78E-01	-0.07	3.43E-01	-0.23
255.2568@7.466759	255.2568	7.47	POS	RP				8.86E-01	3.11E-01	0.31	5.24E-01	0.17	9.49E-01	-0.02	8.08E-01	0.07
255.2569@10.3352585	255.2569	10.34	POS	RP				6.41E-01	5.36E-01	-0.18	3.20E-01	0.30	1.24E-01	0.37	6.80E-01	0.15

Supplementary table 2: Metabolomics analysis results

								ANOVA	Anorexia vs control		overweight vs control		obese vs control		athletes vs control	
Compound	Mass	Retention Time	Ionization mode	column	Compound_ID	MSMS_spectra	ID_level	P-FDR	P	Estimate	P	Estimate	P	Estimate	P	Estimate
255.2929@7.359746	255.2929	7.36	POS	RP				8.80E-02	5.09E-03	0.91	8.32E-01	-0.06	3.87E-01	0.24	3.07E-01	0.32
255.9359@3.3291702	255.9359	3.33	NEG	RP				1.92E-01	2.63E-01	-0.34	4.72E-02	0.53	2.54E-01	0.35	9.11E-01	0.03
255.9363@3.0826385	255.9363	3.08	NEG	RP				1.53E-01	1.76E-01	-0.44	7.71E-02	0.47	2.31E-01	0.36	6.22E-01	0.13
256.0355@4.5118957	256.0355	4.51	POS	RP				7.70E-01	1.24E-01	-0.31	6.19E-01	-0.11	8.30E-01	0.05	5.69E-01	-0.13
256.1406@3.6797073	256.1406	3.68	POS	hilic				8.89E-01	3.18E-01	-0.28	4.72E-01	-0.21	9.65E-01	0.01	9.32E-01	0.02
256.1425@1.500968	256.1425	1.50	POS	hilic				7.33E-01	5.10E-01	0.21	2.51E-01	0.28	4.64E-01	0.17	5.62E-01	-0.13
256.2191@8.463317	256.2191	8.46	POS	RP				7.65E-01	1.93E-01	0.40	2.64E-01	0.29	2.33E-01	0.36	5.77E-01	0.15
256.2194@9.737324	256.2194	9.74	POS	RP				5.11E-01	8.98E-02	-0.49	6.79E-01	0.09	5.88E-01	0.16	6.65E-01	-0.14
256.24@0.42532408	256.2400	0.43	NEG	hilic				2.32E-01	8.75E-01	-0.05	2.74E-02	0.65	4.95E-02	0.52	2.37E-01	0.35
256.2401@10.116852	256.2401	10.12	NEG	RP				6.51E-03	8.65E-01	-0.03	3.10E-03	0.75	2.19E-01	0.24	5.94E-01	-0.07
256.2405@10.7062	256.2405	10.71	NEG	RP	FA 16:0	10 eV: 255.2329 (100)	2	6.29E-02	1.44E-02	-0.66	5.82E-01	0.18	1.82E-01	0.30	1.35E-01	-0.41
256.8292@3.5901287	256.8292	3.59	NEG	hilic				4.80E-01	3.82E-01	-0.17	9.05E-01	-0.03	8.76E-01	-0.04	1.11E-01	0.38
256.8292@4.2304125	256.8292	4.23	NEG	hilic				2.68E-01	2.42E-01	0.39	6.97E-01	0.10	9.47E-01	-0.02	1.60E-02	0.70
256.8293@1.0991539	256.8293	1.10	NEG	RP				5.50E-01	4.90E-01	0.20	3.08E-01	-0.31	1.44E-01	-0.41	8.09E-01	-0.07
256.8293@3.7781415	256.8293	3.78	NEG	hilic				8.00E-01	1.04E-01	-0.44	3.67E-01	-0.25	4.56E-01	-0.20	3.16E-01	-0.24
256.8293@3.9301465	256.8293	3.93	NEG	hilic				8.81E-01	9.68E-01	0.01	6.46E-01	0.15	5.76E-01	-0.16	3.69E-01	0.22
256.8293@4.376941	256.8293	4.38	NEG	hilic				7.14E-02	2.27E-03	0.90	4.70E-01	0.21	8.68E-01	0.05	3.68E-02	0.61
257.0662@4.053942	257.0662	4.05	NEG	hilic				1.99E-02	9.91E-01	0.00	9.43E-03	0.74	2.62E-03	0.80	9.96E-01	0.00
257.1015@1.5088973	257.1015	1.51	POS	hilic				4.77E-01	4.20E-01	0.27	4.86E-01	-0.20	1.15E-01	-0.43	3.86E-01	-0.25
257.1016@3.8338828	257.1016	3.83	POS	hilic				2.11E-01	6.81E-01	0.12	3.91E-01	0.22	1.45E-01	-0.45	1.26E-01	-0.44
257.1033@6.131676	257.1033	6.13	POS	hilic	Glycerophosphocholine	10 eV: 258.1110 (100), 104.1066 (80), 86.0942 (14), 184.0752 (5)	1	8.95E-04	7.16E-03	0.74	1.03E-01	-0.44	3.03E-02	-0.49	6.36E-02	0.54
257.1038@1.2932615	257.1038	1.29	POS	hilic				1.75E-01	2.02E-01	0.37	9.57E-02	-0.44	2.87E-01	-0.30	5.79E-01	0.18
257.1621@1.1938992	257.1621	1.19	POS	hilic				7.18E-01	7.59E-01	0.10	9.72E-02	0.42	1.74E-01	0.37	3.79E-01	0.22
257.9626@1.0610754	257.9626	1.06	NEG	RP				5.89E-01	8.89E-01	-0.03	1.36E-01	0.40	2.04E-01	0.32	2.01E-01	0.30
257.9981@4.6946764	257.9981	4.69	POS	hilic				5.24E-01	6.93E-01	0.11	6.69E-01	-0.12	4.22E-01	0.26	7.66E-02	0.50
258.0194@0.43864995	258.0194	0.44	NEG	hilic				1.01E-02	3.64E-03	0.78	2.42E-01	0.19	1.15E-01	0.30	7.74E-01	-0.03
258.1834@9.270051	258.1834	9.27	POS	RP				4.28E-01	9.19E-01	-0.03	5.36E-01	-0.19	3.40E-02	0.52	6.09E-01	0.16
258.22@10.048557	258.2200	10.05	POS	RP				2.76E-02	1.37E-02	-0.62	5.45E-02	0.53	3.55E-01	0.22	4.79E-01	-0.24
258.8259@3.5819201	258.8259	3.58	NEG	hilic				4.25E-02	3.67E-01	0.23	1.22E-01	-0.23	6.56E-01	-0.08	3.25E-02	0.51
258.826@3.9269526	258.8260	3.93	NEG	hilic				9.96E-01	7.96E-01	0.08	7.72E-01	0.09	9.86E-01	0.00	9.36E-01	-0.02
258.826@4.23235	258.8260	4.23	NEG	hilic				6.45E-01	6.34E-01	0.14	5.67E-01	-0.15	6.30E-01	-0.17	1.75E-01	0.35
258.8261@3.7735457	258.8261	3.77	NEG	hilic				7.31E-01	7.90E-01	-0.07	3.52E-01	0.27	1.64E-01	0.36	5.13E-01	0.17
258.8263@4.378955	258.8263	4.38	NEG	hilic				7.59E-01	3.80E-01	0.27	6.09E-01	0.15	6.33E-01	0.14	7.50E-02	0.48
258.9499@7.318409	258.9499	7.32	POS	hilic				8.94E-01	9.33E-01	-0.02	4.67E-01	0.20	7.87E-01	0.09	2.03E-01	0.31
259.046@3.8419771	259.0460	3.84	POS	hilic				2.17E-01	5.43E-01	0.16	1.86E-02	0.69	7.25E-02	0.56	3.14E-01	0.26
259.0809@4.1065807	259.0809	4.11	NEG	hilic				2.02E-01	5.86E-02	0.53	4.01E-01	0.18	4.20E-01	-0.14	9.65E-01	0.01
259.1784@1.057572	259.1784	1.06	POS	hilic				1.28E-02	3.01E-01	-0.39	2.94E-03	0.77	1.85E-02	0.57	6.74E-01	0.11
259.1786@3.8053994	259.1786	3.81	POS	RP	Acylcarnitine 06:0	10 eV: 260.1874 (100), 85.029 (60)	1	7.42E-03	2.58E-01	-0.42	2.36E-03	0.79	1.36E-02	0.59	6.23E-01	0.13
259.9888@5.137761	259.9888	5.14	NEG	hilic				1.10E-02	2.66E-02	-0.56	2.63E-02	0.60	7.66E-02	0.48	1.91E-01	0.36
260.0207@3.0836906	260.0207	3.08	NEG	RP				7.36E-04	1.08E-02	-0.73	2.73E-03	0.75	3.60E-01	0.26	2.02E-01	-0.28
260.0207@3.3274963	260.0207	3.33	NEG	RP				1.31E-03	4.17E-03	-0.84	2.33E-02	0.53	3.92E-01	0.24	6.19E-02	-0.46
260.0978@5.747482	260.0978	5.75	NEG	hilic				1.41E-01	9.57E-01	-0.02	4.40E-01	-0.20	6.55E-01	-0.13	1.50E-02	0.69
260.1407@10.909361	260.1407	10.91	POS	RP				6.64E-01	7.07E-01	0.11	7.76E-01	-0.08	2.40E-01	0.36	1.98E-01	0.38
260.9887@5.376917	260.9887	5.38	NEG	hilic				7.38E-01	1.57E-01	0.44	5.18E-01	0.15	2.02E-01	0.31	2.75E-01	0.25
261.158@3.391885	261.1580	3.39	POS	hilic				7.09E-01	8.34E-01	0.07	3.14E-01	0.24	1.61E-01	0.42	7.26E-02	0.42
262.0484@4.1156416	262.0484	4.12	NEG	hilic				6.79E-01	5.13E-01	-0.18	2.28E-01	0.35	6.39E-01	0.14	7.22E-01	-0.11
262.0958@0.4614723	262.0958	0.46	POS	hilic				1.46E-01	2.47E-01	-0.34	6.99E-02	-0.46	4.67E-04	-0.81	2.62E-01	-0.30
262.1431@8.348947	262.1431	8.35	POS	RP				3.88E-01	1.87E-01	-0.37	2.99E-01	-0.30	1.41E-01	-0.51	9.95E-03	-0.70
262.2294@10.305355	262.2294	10.31	POS	RP				1.12E-01	2.38E-01	-0.27	1.91E-01	0.37	3.36E-02	0.67	6.55E-01	0.13
262.2295@0.4223334	262.2295	0.42	POS	hilic				1.51E-01	2.00E-01	-0.26	1.07E-01	0.41	5.66E-01	-0.14	2.19E-01	0.32
263.1507@10.685097	263.1507	10.69	POS	RP				5.55E-01	2.98E-02	-0.41	9.00E-01	-0.03	3.21E-01	-0.21	9.41E-01	-0.02
264.0285@3.9606414	264.0285	3.96	POS	hilic				7.83E-01	7.18E-01	-0.12	6.07E-01	-0.16	2.80E-01	0.30	8.29E-01	0.07
264.0421@3.3287945	264.0421	3.33	NEG	RP				1.10E-01	2.13E-01	-0.39	2.04E-02	0.58	2.53E-01	0.36	9.15E-01	0.03
264.0425@3.083704	264.0425	3.08	NEG	RP				8.28E-02	2.37E-01	-0.34	1.39E-02	0.64	2.08E-01	0.41	6.72E-01	0.10
264.1108@3.2796497	264.1108	3.28	NEG	RP				1.32E-01	2.17E-02	0.84	6.20E-01	0.12	8.15E-01	0.07	7.85E-02	0.52
264.1109@2.040223	264.1109	2.04	NEG	hilic				1.00E-01	1.80E-02	0.84	4.83E-01	0.16	1.00E+00	0.00	6.01E-02	0.57
264.1111@1.9144356	264.1111	1.91	POS	hilic				5.90E-02	1.21E-02	0.90	7.26E-01	0.08	9.82E-01	-0.01	6.77E-02	0.53
264.1116@3.2693682	264.1116	3.27	POS	RP				4.80E-01	1.10E-01	0.57	5.19E-01	0.14	9.22E-01	0.03	1.59E-01	0.45
264.1364@6.8644876	264.1364	6.86	POS	RP				5.93E-01	5.47E-01	0.18	3.53E-01	-0.28	6.32E-01	-0.16	3.15E-01	0.28
264.173@8.249256	264.1730	8.25	POS	RP				1.61E-01	8.78E-03	-0.71	6.40E-01	0.15	9.75E-01	0.01	5.58E-01	0.17
264.2087@0.40691757	264.2087	0.41	POS	hilic				1.77E-02	1.67E-01	-0.35	2.96E-01	0.26	5.98E-01	-0.12	8.53E-03	0.76
265.0171@4.4243817	265.0171	4.42	NEG	hilic				6.35E-01	6.61E-01	0.14	2.09E-01	-0.28	1.12E-01	-0.42	5.21E-01	-0.21
265.0797@5.2106605	265.0797	5.21	NEG	hilic				6.31E-01	4.46E-01	-0.20	1.42E-01	0.40	6.69E-01	0.12	7.62E-01	0.09
265.107@1.6719261	265.1070	1.67	POS	hilic				2.48E-01	1.80E-01	-0.36	3.45E-01	-0.24	4.93E-03	-0.70	6.39E-01	-0.13
265.1316@1.3859783	265.1316	1.39	POS	hilic				8.77E-01	8.38E-01	0.07	8.85E-01	0.04	9.14E-01	-0.03	2.22E-01	0.32
265.1529@3.7678554	265.1529	3.77	POS	RP				5.74E-01	6.72E-02	0.56	1.65E-01	0.39	5.90E-01	0.15	8.41E-01	0.06
265.1531@0.4884222	265.1531	0.49	POS	hilic				4.12E-01	1.03E-01	0.51	9.68E-02	0.45	1.90E-01	0.35	7.88E-01	-0.08
265.1531@4.228881	265.1531	4.23	POS	RP				1.14E-01	1.31E-02	0.82	1.68E-01	0.34	3.16E-01	0.29	6.29E-01	-0.15
265.1657@10.684438	265.1657	10.68	POS	RP				6.06E-01	4.78E-02	-0.47	1.45E-01	-0.39	1.80E-01	-0.33	4.64E-01	-0.20
265.8825@7.884908	265.8825	7.88	POS	hilic				2.20E-01	1.52E-02	0.64	9.04E-02	0.41	1.37E-01	0.39	1.13E-02	0.69
265.9415@3.105909	265.9415	3.11	NEG	hilic				7.38E-01	6.01E-01	-0.14	4.60E-01	-0.22	7.62E-01	-0.10	2.79E-01	0.28
266.0068@6.4464345	266.0068	6.45	NEG	RP				5.97E-01	3.54E-01	0.31	4.38E-01	-0.24	5.14E-01	-0.18	5.76E-01	0.19
266.0255@1.81356	266.0255	1.81	NEG	hilic				2.97E-02	4.30E-01	-0.23	4.88E-01	0.18	2.81E-02	0.64	1.13E-01	-0.44

Supplementary table 2: Metabolomics analysis results

								ANOVA	Anorexia vs control		overweight vs control		obese vs control		athletes vs control	
Compound	Mass	Retention Time	Ionization mode	column	Compound_ID	MSMS_spectra	ID_level	P-FDR	P	Estimate	P	Estimate	P	Estimate	P	Estimate
266.0374@0.88431066	266.0374	0.88	NEG	hilic				6.95E-03	4.09E-02	-0.60	4.85E-02	0.55	2.55E-02	0.58	6.70E-01	-0.10
266.1375@0.5535794	266.1375	0.55	POS	hilic				6.86E-01	8.92E-01	0.04	3.41E-01	-0.26	1.72E-01	-0.39	9.89E-01	0.00
266.1376@0.7928825	266.1376	0.79	POS	hilic				7.56E-01	7.79E-01	0.09	8.26E-01	0.06	7.20E-01	0.10	1.17E-01	0.47
266.1549@0.430268	266.1549	0.43	NEG	hilic				9.35E-01	4.79E-01	0.24	7.39E-01	0.08	3.60E-01	0.26	7.11E-01	0.10
266.1549@10.052398	266.1549	10.05	NEG	RP				5.05E-01	6.80E-02	0.55	9.73E-01	0.01	7.01E-01	0.10	5.41E-01	0.15
266.1551@0.42581722	266.1551	0.43	NEG	hilic				7.31E-01	9.81E-01	0.01	7.96E-01	-0.06	5.86E-01	0.15	1.64E-01	-0.37
266.1576@10.313367	266.1576	10.31	POS	RP				4.84E-01	2.21E-01	-0.32	2.55E-01	0.35	8.25E-01	0.06	7.15E-01	-0.10
266.1661@9.129134	266.1661	9.13	POS	RP				8.20E-01	3.12E-01	0.34	3.49E-01	0.25	7.66E-01	0.09	8.53E-01	-0.05
266.1676@9.124958	266.1676	9.12	POS	RP				9.65E-01	5.40E-01	0.20	7.43E-01	0.08	4.37E-01	0.25	6.90E-01	0.13
266.9885@3.4503677	266.9885	3.45	POS	RP				2.44E-02	2.97E-02	0.83	3.80E-01	0.19	1.52E-01	-0.39	3.62E-01	0.21
267.1105@8.687391	267.1105	8.69	NEG	RP				8.43E-01	5.53E-01	-0.18	5.13E-01	0.18	5.31E-01	-0.19	9.91E-01	0.00
268.028@6.181889	268.0280	6.18	NEG	hilic				2.38E-01	6.04E-01	-0.14	3.43E-01	-0.23	2.50E-03	-0.58	6.45E-01	0.12
268.1308@0.49862862	268.1308	0.50	NEG	hilic				1.01E-01	1.72E-01	-0.39	2.64E-01	0.27	2.68E-01	0.30	1.07E-01	-0.38
268.131@8.544612	268.1310	8.54	NEG	RP				7.36E-02	5.30E-03	-0.83	9.93E-01	0.00	5.17E-01	-0.18	2.87E-02	-0.66
268.1318@8.534809	268.1318	8.53	POS	RP				3.00E-01	1.90E-02	-0.68	8.42E-01	-0.06	4.11E-01	-0.23	9.09E-02	-0.54
268.1946@6.5241046	268.1946	6.52	POS	RP				7.78E-01	6.16E-01	0.11	2.67E-01	0.29	7.61E-01	-0.07	8.64E-01	-0.04
268.2161@0.4165556	268.2161	0.42	POS	hilic				5.99E-01	2.69E-01	0.37	7.43E-01	-0.07	6.48E-01	-0.10	3.83E-01	0.20
268.22@10.459926	268.2200	10.46	POS	RP				5.76E-01	1.06E-01	0.61	7.20E-01	0.11	6.84E-01	0.11	3.60E-01	0.26
268.2396@10.618942	268.2396	10.62	NEG	RP				3.28E-02	2.16E-02	-0.48	2.24E-01	0.33	1.99E-01	0.32	2.01E-01	-0.30
269.2358@10.877436	269.2358	10.88	POS	RP				9.46E-01	5.08E-01	-0.17	7.22E-01	-0.09	9.42E-01	0.02	7.54E-01	0.09
269.2726@10.307983	269.2726	10.31	POS	RP				3.09E-02	3.92E-01	-0.28	7.70E-03	0.73	7.71E-02	0.50	7.85E-01	-0.08
269.9511@4.7017255	269.9511	4.70	POS	hilic				3.85E-01	5.89E-02	0.55	6.71E-01	-0.13	8.02E-01	0.08	1.66E-01	0.40
270.0612@3.5655496	270.0612	3.57	NEG	hilic				1.84E-02	1.78E-01	-0.40	3.00E-01	-0.27	2.83E-01	-0.32	7.41E-03	0.69
270.0858@1.7354447	270.0858	1.74	POS	hilic				3.94E-01	8.28E-01	-0.07	1.10E-01	0.41	8.66E-02	0.53	9.35E-01	-0.02
270.0947@5.1943626	270.0947	5.19	NEG	hilic				7.10E-01	9.73E-01	-0.01	5.47E-01	0.19	9.17E-02	0.48	6.63E-01	0.12
270.1382@8.919009	270.1382	8.92	NEG	RP				7.97E-01	9.06E-01	0.04	5.71E-01	-0.15	2.83E-01	0.28	4.57E-01	0.22
270.1383@0.6303111	270.1383	0.63	NEG	hilic				7.59E-01	2.69E-01	0.28	9.23E-01	0.02	3.60E-01	0.21	2.13E-01	0.29
270.1397@9.064045	270.1397	9.06	POS	RP				6.31E-01	6.50E-01	0.12	9.47E-01	-0.01	2.06E-01	0.29	1.22E-01	0.37
270.1626@8.93494	270.1626	8.93	POS	RP				8.63E-01	8.92E-01	0.05	1.95E-01	0.37	4.29E-01	0.22	5.59E-01	0.17
270.1629@8.934078	270.1629	8.93	POS	RP				9.70E-01	7.83E-01	-0.10	7.41E-01	0.10	5.42E-01	0.16	8.53E-01	0.05
270.1981@0.53984463	270.1981	0.54	POS	hilic				5.84E-01	7.14E-01	0.10	1.01E-01	0.45	1.85E-01	0.41	9.53E-01	0.02
270.1985@10.456389	270.1985	10.46	POS	RP				8.20E-01	2.79E-01	0.35	9.29E-01	-0.03	3.18E-01	0.24	7.91E-01	0.08
270.1986@7.2422986	270.1986	7.24	POS	RP				3.08E-01	3.62E-01	-0.29	8.21E-02	0.48	3.28E-01	0.30	9.01E-01	-0.03
270.1998@10.339704	270.1998	10.34	POS	RP				6.71E-01	5.93E-01	0.17	1.36E-01	0.38	1.03E-01	0.50	7.17E-01	0.10
270.2181@9.781129	270.2181	9.78	POS	RP				6.19E-01	8.69E-01	0.05	1.41E-01	0.43	2.45E-01	0.32	8.58E-01	-0.05
270.2188@9.793461	270.2188	9.79	NEG	RP				5.70E-01	3.89E-01	-0.23	4.36E-01	0.22	1.67E-01	0.34	9.06E-01	0.03
270.219@9.53047	270.2190	9.53	POS	RP				9.65E-01	8.98E-01	0.04	9.46E-01	0.02	4.65E-01	0.24	7.55E-01	0.10
270.2193@0.43085113	270.2193	0.43	NEG	hilic				2.63E-03	6.87E-03	-0.67	1.52E-02	0.68	2.95E-01	0.27	1.53E-01	0.32
270.2193@9.290676	270.2193	9.29	POS	RP				2.40E-01	8.46E-02	0.51	1.09E-01	0.47	8.12E-01	-0.07	7.01E-01	-0.09
270.2193@9.454246	270.2193	9.45	POS	RP				4.73E-01	9.58E-02	0.44	4.03E-02	0.63	1.06E-01	0.46	3.48E-01	0.29
270.2194@9.441451	270.2194	9.44	POS	RP				9.33E-01	8.64E-01	0.04	8.60E-01	0.05	4.30E-01	0.26	5.09E-01	0.19
270.2347@8.964571	270.2347	8.96	POS	RP				8.41E-01	2.41E-01	-0.31	5.59E-01	-0.14	2.14E-01	-0.29	6.58E-01	-0.12
270.2557@10.866607	270.2557	10.87	NEG	RP	FA 17:0	10 eV: 269.2499 (100)	1	5.08E-01	2.73E-01	-0.32	8.00E-01	0.09	1.62E-01	0.34	4.47E-01	-0.20
270.2697@10.4474945	270.2697	10.45	POS	RP				9.83E-02	8.28E-01	0.06	5.56E-02	0.54	4.24E-03	0.87	5.41E-01	0.18
270.9096@15.280178	270.9096	15.28	POS	RP				3.21E-01	5.40E-02	-0.61	3.37E-01	-0.28	4.87E-01	-0.21	3.12E-02	-0.68
271.0528@4.84255	271.0528	4.84	NEG	hilic				2.14E-01	5.76E-02	0.50	2.00E-01	-0.40	5.38E-01	-0.19	9.67E-01	0.01
271.1786@0.6591111	271.1786	0.66	POS	hilic				9.77E-01	9.95E-01	0.00	6.09E-01	-0.12	8.62E-01	0.04	7.24E-01	-0.08
271.1788@1.0397545	271.1788	1.04	POS	hilic				8.98E-01	3.60E-01	0.25	9.79E-01	-0.01	5.30E-01	0.14	9.87E-01	0.00
271.1788@1.2065	271.1788	1.21	POS	hilic				6.72E-03	5.96E-01	-0.06	5.50E-02	0.36	3.51E-03	0.75	7.22E-01	0.05
271.1788@3.1340373	271.1788	3.13	POS	RP				9.65E-01	8.93E-01	0.04	8.56E-01	-0.04	5.96E-01	0.13	6.19E-01	0.14
271.2148@8.282387	271.2148	8.28	POS	RP				8.23E-01	4.10E-01	-0.16	8.70E-01	0.04	2.73E-01	-0.21	8.85E-01	0.03
271.2184@0.41023642	271.2184	0.41	POS	hilic				6.16E-01	8.03E-01	0.06	4.50E-01	-0.20	6.66E-02	-0.43	5.80E-01	-0.15
271.9772@15.410888	271.9772	15.41	NEG	RP				6.17E-01	8.51E-02	0.46	4.75E-01	0.16	7.65E-01	0.06	3.05E-01	0.24
272.0119@5.609913	272.0119	5.61	NEG	hilic				4.92E-01	5.43E-01	0.19	7.68E-01	0.08	6.87E-02	-0.45	5.04E-01	-0.22
272.0407@3.3859272	272.0407	3.39	POS	hilic				8.58E-02	1.05E-01	-0.48	3.76E-01	-0.21	6.52E-01	-0.13	7.95E-02	0.52
272.0769@4.116964	272.0769	4.12	NEG	hilic				6.28E-01	3.18E-01	-0.29	8.69E-01	0.05	2.35E-01	0.34	8.17E-01	-0.06
272.1145@3.6789367	272.1145	3.68	POS	hilic				7.10E-01	2.82E-01	-0.29	4.00E-01	-0.24	5.23E-01	0.19	7.78E-01	-0.08
272.1999@9.614064	272.1999	9.61	POS	RP				8.85E-01	2.30E-01	-0.31	4.17E-01	-0.21	4.95E-01	-0.20	2.95E-01	-0.26
272.214@8.029341	272.2140	8.03	POS	RP				7.96E-01	3.61E-01	-0.24	9.45E-01	-0.02	4.48E-01	0.22	7.08E-01	0.11
272.2143@6.776245	272.2143	6.78	POS	RP				2.32E-02	1.16E-01	-0.47	2.84E-01	0.29	1.00E-01	0.52	4.10E-02	-0.47
272.2342@10.1335745	272.2342	10.13	NEG	RP				7.73E-01	5.43E-01	-0.16	4.50E-01	0.23	3.44E-01	0.23	8.11E-01	-0.08
272.2345@9.364982	272.2345	9.36	NEG	RP				8.27E-01	5.56E-01	-0.16	5.07E-01	0.21	5.47E-01	-0.20	8.45E-01	-0.05
272.2347@0.47455972	272.2347	0.47	NEG	hilic				4.28E-01	6.32E-01	0.14	3.56E-02	0.64	1.45E-01	0.41	4.52E-01	0.23
272.2347@9.793543	272.2347	9.79	NEG	RP				2.42E-01	7.67E-01	-0.08	5.06E-02	0.54	7.08E-02	0.47	1.38E-01	0.46
272.2348@10.28087	272.2348	10.28	NEG	RP				4.93E-01	4.53E-01	0.22	9.68E-01	0.01	5.21E-01	-0.19	1.20E-01	-0.48
273.0773@0.43417308	273.0773	0.43	POS	hilic				6.18E-01	4.07E-01	-0.21	7.52E-01	-0.07	3.26E-01	0.26	4.54E-01	0.18
273.0934@6.114017	273.0934	6.11	NEG	hilic				8.58E-01	6.83E-01	-0.11	9.97E-01	0.00	1.93E-01	-0.31	6.10E-01	-0.13
273.1769@7.103265	273.1769	7.10	POS	RP				1.54E-03	8.22E-02	-0.43	1.85E-03	0.82	2.03E-02	0.58	9.21E-01	0.03
273.1783@0.8766665	273.1783	0.88	POS	hilic				2.93E-02	1.35E-02	-0.53	4.85E-02	0.54	6.88E-01	0.10	7.55E-01	0.08
273.194@0.9369904	273.1940	0.94	POS	hilic				1.08E-01	9.20E-01	0.03	9.20E-03	0.65	1.99E-03	0.76	3.29E-01	0.31
274.1974@10.484048	274.1974	10.48	POS	RP				4.09E-02	9.71E-01	0.01	1.31E-03	0.95	3.32E-01	0.22	5.07E-01	0.20
274.8342@15.35582	274.8342	15.36	NEG	RP				7.41E-01	8.58E-01	0.06	1.93E-01	0.36	5.42E-01	0.19	1.21E-01	0.43
275.1368@5.011302	275.1368	5.01	POS	hilic				6.91E-02	5.45E-01	0.19	4.02E-02	0.50	2.89E-01	-0.20	5.66E-03	0.60

Supplementary table 2: Metabolomics analysis results

								ANOVA	Anorexia vs control		overweight vs control		obese vs control		athletes vs control	
Compound	Mass	Retention Time	Ionization mode	column	Compound_ID	MSMS_spectra	ID_level	P-FDR	P	Estimate	P	Estimate	P	Estimate	P	Estimate
275.1744@2.3022196	275.1744	2.30	POS	hilic				2.91E-01	6.84E-01	-0.12	3.72E-02	0.58	3.24E-01	0.25	7.84E-01	0.07
275.1913@0.8505757	275.1913	0.85	POS	hilic				1.38E-01	9.18E-02	-0.34	5.31E-02	0.51	4.57E-01	0.16	4.48E-01	0.20
275.1924@7.462454	275.1924	7.46	POS	RP				2.00E-03	9.40E-03	-0.63	7.04E-03	0.72	5.30E-01	0.15	3.11E-01	0.30
276.0902@0.50758415	276.0902	0.51	POS	hilic				1.72E-01	6.08E-02	0.63	2.57E-02	0.58	9.21E-01	-0.03	1.75E-01	0.34
276.1728@8.925592	276.1728	8.93	POS	RP				6.53E-01	2.71E-01	0.33	4.23E-01	-0.21	4.34E-01	0.22	5.28E-01	0.20
276.173@8.820375	276.1730	8.82	POS	RP				4.06E-01	1.93E-01	-0.39	6.26E-02	-0.61	8.10E-01	-0.06	8.55E-01	-0.05
276.2087@0.43572927	276.2087	0.44	POS	hilic				7.15E-01	3.55E-01	-0.24	4.37E-01	0.23	7.98E-01	-0.07	5.17E-01	0.21
276.8316@15.355039	276.8316	15.36	NEG	RP				4.83E-01	3.19E-01	-0.28	1.05E-01	0.41	5.45E-01	0.16	4.85E-01	0.23
277.1791@0.66255563	277.1791	0.66	POS	hilic				5.64E-01	3.24E-01	0.25	6.00E-01	-0.10	3.14E-01	-0.18	6.14E-01	-0.10
277.9016@15.281045	277.9016	15.28	NEG	RP				7.34E-01	8.77E-01	-0.04	3.56E-01	-0.21	3.76E-01	0.25	6.87E-01	-0.09
277.985@3.4008045	277.9850	3.40	NEG	hilic				7.88E-01	8.44E-01	0.06	8.82E-01	-0.04	3.88E-01	0.24	3.94E-01	-0.22
277.985@3.4549823	277.9850	3.45	NEG	hilic				9.64E-01	7.04E-01	-0.12	8.17E-01	0.07	5.92E-01	0.17	8.69E-01	0.05
278.0216@4.116463	278.0216	4.12	NEG	hilic				5.61E-02	4.84E-02	-0.50	7.69E-02	0.49	2.40E-01	0.38	6.41E-01	-0.14
278.061@2.4687092	278.0610	2.47	NEG	hilic				6.01E-03	3.80E-02	-0.61	2.91E-03	0.71	1.43E-01	0.35	2.38E-01	0.37
278.0801@5.1163416	278.0801	5.12	NEG	hilic				4.16E-01	7.23E-01	-0.07	4.08E-01	-0.18	1.66E-01	0.39	8.38E-01	-0.04
278.0808@5.264237	278.0808	5.26	NEG	hilic				2.78E-01	4.26E-02	-0.50	6.01E-01	0.14	6.22E-01	-0.14	1.30E-01	-0.36
278.1524@10.909249	278.1524	10.91	POS	RP				5.63E-01	6.88E-01	0.11	9.70E-01	-0.01	9.50E-01	-0.02	7.26E-02	0.54
278.1524@9.267607	278.1524	9.27	POS	RP				8.46E-01	2.60E-01	-0.38	8.13E-01	-0.07	4.40E-01	-0.25	9.17E-01	-0.03
278.1525@9.189624	278.1525	9.19	POS	RP				8.35E-01	8.85E-01	0.05	9.64E-01	-0.01	1.89E-01	0.37	5.39E-01	0.19
278.1528@0.42327273	278.1528	0.42	POS	hilic				8.11E-01	4.44E-01	0.19	2.13E-01	0.32	2.05E-01	0.30	2.00E-01	0.27
278.1884@8.879961	278.1884	8.88	NEG	RP				6.31E-01	7.58E-01	-0.09	2.79E-01	-0.32	2.79E-01	0.29	9.58E-01	-0.02
278.2236@0.42446664	278.2236	0.42	POS	hilic				6.27E-02	6.31E-02	-0.36	6.86E-01	0.10	8.81E-01	-0.03	4.20E-02	0.58
278.2239@10.737236	278.2239	10.74	POS	RP				4.65E-03	6.01E-03	-0.84	1.46E-01	0.36	1.15E-01	-0.44	3.29E-01	0.27
278.2242@10.354721	278.2242	10.35	NEG	RP				1.54E-01	1.82E-01	-0.42	3.30E-01	0.30	7.80E-02	0.52	8.93E-01	-0.04
278.2242@10.785433	278.2242	10.79	POS	RP				2.58E-01	2.31E-02	-0.49	6.28E-01	0.11	1.25E-01	-0.32	9.67E-01	0.01
278.2242@11.484842	278.2242	11.48	POS	RP				3.09E-02	4.52E-02	-0.58	1.85E-01	0.32	2.70E-01	0.24	8.78E-02	0.55
278.2245@9.470816	278.2245	9.47	POS	RP				4.29E-01	5.86E-02	-0.55	8.18E-02	-0.41	2.30E-01	-0.32	8.71E-01	-0.04
278.2249@9.658054	278.2249	9.66	POS	RP				6.94E-01	7.86E-01	0.07	4.50E-01	0.24	6.14E-02	0.51	7.21E-01	0.11
278.225@11.016239	278.2250	11.02	POS	RP				1.37E-01	2.82E-01	-0.20	9.68E-01	-0.01	7.44E-01	-0.07	6.06E-02	0.56
278.2254@10.34741	278.2254	10.35	POS	RP				3.27E-01	4.55E-01	-0.21	2.24E-01	0.39	9.02E-02	0.48	9.30E-01	-0.03
278.2254@9.675613	278.2254	9.68	POS	RP				1.79E-01	1.93E-02	0.57	2.19E-01	0.35	1.29E-02	0.72	8.54E-01	0.05
279.0853@6.129445	279.0853	6.13	POS	hilic				2.41E-03	1.27E-01	0.39	8.18E-02	-0.50	2.32E-03	-0.72	1.20E-01	0.46
279.1477@1.2734721	279.1477	1.27	POS	hilic				4.93E-01	1.43E-01	0.49	1.96E-01	0.38	2.02E-02	0.61	2.26E-01	0.33
279.1689@8.349966	279.1689	8.35	POS	RP				9.39E-01	9.98E-01	0.00	6.74E-01	0.11	6.42E-01	-0.17	5.93E-01	-0.15
279.862@15.269362	279.8620	15.27	NEG	RP				7.39E-01	3.57E-01	-0.24	5.42E-01	-0.16	1.68E-01	-0.36	1.67E-01	-0.38
280.0414@0.8466364	280.0414	0.85	NEG	hilic				6.04E-04	4.48E-03	0.81	6.72E-01	-0.07	8.98E-01	0.02	5.04E-02	-0.26
280.0458@1.5533589	280.0458	1.55	NEG	hilic				6.17E-01	3.33E-01	0.31	1.28E-01	0.40	1.22E-01	0.39	8.63E-01	-0.05
280.0665@4.4148893	280.0665	4.41	NEG	hilic				4.61E-01	2.73E-01	0.35	6.15E-01	-0.12	1.33E-01	-0.37	9.75E-01	0.01
280.2044@0.43690646	280.2044	0.44	POS	hilic				1.52E-03	2.79E-02	-0.58	4.51E-03	0.77	3.16E-01	0.26	3.11E-02	0.55
280.2398@0.41727027	280.2398	0.42	NEG	hilic				2.79E-01	1.24E-01	-0.51	3.76E-01	0.22	9.14E-01	-0.03	2.41E-01	0.31
280.2398@9.969644	280.2398	9.97	NEG	RP				1.24E-02	8.06E-02	0.52	9.29E-02	-0.41	2.86E-02	-0.53	5.81E-01	0.15
280.2402@10.567332	280.2402	10.57	NEG	RP	FA 18:2	10 eV: 279.2325 (100)	1	1.53E-02	5.79E-03	-0.87	5.10E-01	0.19	2.34E-01	0.33	4.21E-01	-0.23
280.2402@0.4255533	280.2402	0.43	POS	hilic				2.24E-04	8.87E-04	-0.90	9.41E-03	0.67	7.72E-02	0.41	3.65E-01	0.25
280.2402@10.921834	280.2402	10.92	POS	RP				6.36E-02	5.87E-02	-0.42	7.10E-02	0.47	3.07E-01	0.24	2.26E-01	0.33
280.2403@11.027382	280.2403	11.03	POS	RP				3.48E-02	7.57E-02	-0.53	1.06E-01	0.44	2.85E-01	0.27	7.48E-02	0.53
280.2407@10.072515	280.2407	10.07	POS	RP				2.25E-01	8.96E-01	-0.03	5.83E-02	0.58	3.92E-02	0.58	7.80E-01	0.08
280.2407@9.838494	280.2407	9.84	POS	RP				4.34E-02	1.05E-01	-0.48	3.54E-02	0.58	5.13E-01	0.16	3.54E-01	-0.29
280.2407@9.905324	280.2407	9.91	POS	RP				5.97E-01	1.70E-01	-0.38	6.21E-01	0.16	8.77E-01	0.04	3.65E-01	-0.30
280.2408@10.561055	280.2408	10.56	POS	RP				6.94E-01	4.02E-02	-0.44	9.58E-01	0.02	7.07E-01	-0.10	4.41E-01	-0.20
280.2417@9.635463	280.2417	9.64	POS	RP				9.45E-01	9.65E-01	-0.01	7.20E-01	0.11	4.95E-01	-0.18	8.34E-01	-0.06
281.1129@4.32394	281.1129	4.32	POS	hilic				5.57E-01	3.45E-01	0.31	1.34E-01	0.40	9.81E-01	0.01	5.79E-01	-0.18
281.1966@7.439314	281.1966	7.44	POS	RP				7.88E-01	2.34E-01	-0.35	2.25E-01	-0.36	1.94E-01	-0.30	2.47E-01	-0.39
281.2711@0.4432951	281.2711	0.44	POS	hilic				9.53E-01	6.24E-01	-0.14	8.32E-01	0.05	8.99E-01	0.03	5.98E-01	-0.14
281.2723@10.439795	281.2723	10.44	POS	RP				8.36E-01	4.24E-01	-0.15	1.93E-01	-0.23	9.42E-01	0.02	8.99E-01	-0.03
281.8976@7.8812118	281.8976	7.88	POS	hilic				9.06E-01	6.49E-01	-0.15	6.33E-01	-0.14	5.44E-01	0.19	8.92E-01	-0.04
281.9904@6.479113	281.9904	6.48	NEG	RP				8.86E-01	7.36E-01	-0.11	2.91E-01	-0.31	6.34E-01	-0.13	3.03E-01	-0.32
282.0207@4.826001	282.0207	4.83	NEG	hilic				1.94E-01	5.09E-02	0.49	1.69E-01	-0.40	5.27E-01	0.21	4.45E-01	0.22
282.0254@3.5679061	282.0254	3.57	POS	hilic				8.39E-01	8.65E-01	0.06	5.31E-01	-0.19	9.02E-01	0.03	2.76E-01	-0.32
282.0434@6.0835724	282.0434	6.08	NEG	hilic				5.10E-01	9.83E-02	-0.52	2.00E-01	-0.41	1.62E-01	-0.41	4.74E-02	-0.59
282.0609@1.6533159	282.0609	1.65	NEG	hilic				3.65E-01	5.81E-01	0.18	4.93E-02	0.57	7.43E-01	-0.09	7.64E-01	-0.08
282.1075@0.88192326	282.1075	0.88	NEG	hilic				1.86E-02	5.56E-02	-0.59	5.27E-02	0.51	1.59E-01	0.40	3.16E-01	-0.23
282.1484@8.535082	282.1484	8.54	POS	RP				7.76E-01	2.31E-01	-0.35	8.80E-01	0.05	5.78E-01	-0.15	3.37E-01	-0.29
282.235@7.9259067	282.2350	7.93	POS	RP				4.65E-02	4.88E-01	-0.17	2.37E-02	0.65	3.70E-01	0.28	2.07E-01	-0.38
282.2553@10.232252	282.2553	10.23	NEG	RP				1.28E-02	2.71E-01	0.35	4.75E-03	-0.72	5.68E-02	-0.50	9.30E-01	0.02
282.2554@0.41859776	282.2554	0.42	NEG	hilic				2.98E-02	7.94E-02	-0.47	5.02E-02	0.56	1.04E-01	0.44	9.31E-01	-0.02
282.256@10.783302	282.2560	10.78	NEG	RP	FA 18:1	10 eV: 281.2483 (100)	1	4.14E-03	1.78E-02	-0.69	9.38E-02	0.48	4.44E-02	0.55	4.16E-01	-0.23
282.2567@10.775377	282.2567	10.78	POS	RP				1.65E-01	2.31E-01	-0.38	2.13E-01	0.38	7.55E-02	0.49	9.67E-01	0.01
282.2571@10.327184	282.2571	10.33	POS	RP				5.89E-02	1.03E-01	-0.51	1.67E-01	0.44	1.39E-01	0.47	7.54E-01	-0.08
282.2572@10.5414715	282.2572	10.54	POS	RP				5.62E-03	9.06E-04	-0.69	3.72E-02	0.56	3.61E-01	0.22	6.98E-01	-0.10
282.2578@9.8668375	282.2578	9.87	POS	RP				1.38E-01	4.06E-01	0.21	1.16E-01	0.50	3.97E-03	0.78	9.72E-01	-0.01
282.2581@3.2843335	282.2581	3.28	POS	hilic				1.38E-01	5.62E-01	-0.09	2.27E-02	0.50	5.89E-02	0.50	1.79E-01	0.30
283.1199@0.42660496	283.1199	0.43	POS	hilic				4.49E-01	2.16E-02	-0.68	7.75E-01	-0.08	6.67E-01	-0.12	6.47E-01	-0.14
283.1623@1.520162	283.1623	1.52	NEG	hilic				1.14E-03	8.23E-01	0.04	5.24E-01	0.10	2.66E-04	0.93	1.19E-02	0.62

Supplementary table 2: Metabolomics analysis results

								ANOVA	Anorexia vs control		overweight vs control		obese vs control		athletes vs control	
Compound	Mass	Retention Time	Ionization mode	column	Compound_ID	MSMS_spectra	ID_level	P-FDR	P	Estimate	P	Estimate	P	Estimate	P	Estimate
283.164@0.5779403	283.1640	0.58	POS	hilic				6.60E-01	3.76E-01	0.26	3.66E-01	0.27	4.69E-01	-0.21	7.13E-01	-0.11
283.1644@0.7928201	283.1644	0.79	POS	hilic				9.29E-01	6.82E-01	0.13	9.73E-01	0.01	7.56E-01	-0.10	4.51E-01	0.23
283.1786@1.0396063	283.1786	1.04	POS	hilic				8.45E-01	4.35E-01	0.24	7.88E-01	-0.06	3.46E-01	0.23	7.39E-01	0.08
283.2881@10.704753	283.2881	10.70	POS	RP				8.55E-01	8.41E-01	-0.06	2.42E-01	0.32	4.72E-01	0.19	9.24E-01	0.04
283.3242@8.79094	283.3242	8.79	POS	RP				5.95E-01	2.19E-01	0.40	5.53E-01	0.17	4.29E-01	-0.21	4.13E-01	0.25
284.037@5.248319	284.0370	5.25	NEG	hilic				2.93E-02	7.11E-04	-0.85	4.24E-01	-0.24	3.88E-01	-0.24	2.30E-04	-0.88
284.0891@3.5855002	284.0891	3.59	NEG	hilic				2.64E-01	1.15E-01	0.54	8.43E-01	-0.05	2.82E-01	-0.27	9.66E-01	-0.01
284.1156@11.224781	284.1156	11.22	NEG	RP				2.04E-01	1.81E-01	-0.36	8.95E-01	-0.03	3.70E-03	-0.63	4.95E-01	-0.16
284.1164@11.214509	284.1164	11.21	POS	RP				5.79E-01	3.16E-01	-0.27	6.70E-01	-0.11	6.55E-02	-0.47	1.32E-01	-0.36
284.1246@8.348857	284.1246	8.35	POS	RP				5.62E-01	8.66E-01	-0.05	8.71E-01	-0.04	3.95E-01	-0.30	6.74E-02	-0.54
284.2704@10.509749	284.2704	10.51	NEG	RP				3.35E-01	4.47E-01	-0.19	3.35E-01	-0.19	8.07E-01	0.05	1.43E-01	0.39
284.271@0.41687185	284.2710	0.42	NEG	hilic				7.50E-01	2.19E-01	0.38	7.65E-02	0.43	2.21E-01	0.35	2.70E-01	0.33
284.2715@11.037408	284.2715	11.04	NEG	RP	dublicate of FA 18:0			3.04E-01	1.34E-01	-0.44	9.56E-01	0.02	1.39E-01	0.35	2.85E-01	-0.31
284.2717@11.037408	284.2717	11.04	NEG	RP	FA 18:0	10 eV: 283.2647 (100)	1	3.01E-01	1.31E-01	-0.45	9.60E-01	0.02	1.40E-01	0.35	2.83E-01	-0.31
285.0254@4.6511736	285.0254	4.65	NEG	hilic				1.24E-01	7.94E-02	-0.55	9.70E-01	-0.01	9.91E-02	-0.41	2.13E-01	0.34
285.096@1.4112161	285.0960	1.41	POS	hilic				8.56E-01	7.88E-01	0.09	6.69E-01	0.13	9.92E-01	0.00	3.37E-01	-0.27
285.1222@3.3733811	285.1222	3.37	POS	hilic				2.22E-02	3.47E-02	0.65	1.56E-01	-0.30	1.56E-01	-0.30	7.19E-01	-0.08
285.1369@8.106468	285.1369	8.11	POS	RP	Piperine	10 eV: 286.1447 (100), 201.0561 (63), 69.0693 (10)	2	3.30E-02	6.23E-03	-0.83	9.16E-01	0.03	2.13E-01	0.36	9.20E-01	0.03
285.1372@0.4304796	285.1372	0.43	POS	hilic				3.49E-02	2.73E-03	-0.86	6.17E-01	-0.13	3.30E-01	0.26	7.43E-01	-0.10
285.1373@8.099503	285.1373	8.10	POS	RP				6.67E-02	1.02E-02	-0.76	8.33E-01	-0.06	2.02E-01	0.37	9.87E-01	0.01
285.1942@0.9454013	285.1942	0.95	POS	hilic				9.83E-02	8.22E-01	-0.08	2.22E-01	0.31	6.40E-03	0.81	8.53E-01	-0.05
285.1944@4.8190756	285.1944	4.82	POS	RP	Acylcarnitine 08:1	10 eV: 286.2013 (100), 85.0278 (15)	2	7.87E-02	9.49E-01	-0.02	2.81E-01	0.28	2.60E-03	0.87	9.53E-01	-0.02
285.1944@0.9450953	285.1944	0.95	POS	hilic				1.92E-01	6.56E-01	-0.15	2.20E-01	0.30	3.50E-02	0.67	7.59E-01	-0.08
285.1945@4.914461	285.1945	4.91	POS	RP				2.13E-01	2.76E-01	-0.33	1.29E-01	0.48	1.83E-01	0.32	6.38E-01	-0.13
285.1948@5.1824403	285.1948	5.18	POS	RP				3.11E-02	8.12E-02	-0.49	1.06E-01	0.46	3.53E-01	-0.23	1.64E-01	0.39
285.8782@0.5814999	285.8782	0.58	NEG	hilic				4.63E-01	2.21E-01	0.34	3.17E-01	0.24	5.20E-01	-0.14	1.84E-01	0.35
285.8989@15.266647	285.8989	15.27	NEG	RP				4.33E-01	4.78E-02	-0.52	2.06E-01	-0.32	1.66E-01	-0.40	5.93E-02	-0.67
286.0895@5.1777773	286.0895	5.18	NEG	hilic				4.88E-01	8.25E-02	-0.46	6.31E-01	0.13	2.97E-01	-0.29	2.94E-01	-0.28
286.1418@0.45912838	286.1418	0.46	POS	hilic				3.84E-01	3.17E-02	-0.63	1.96E-01	-0.38	8.59E-01	0.05	6.82E-01	-0.13
286.1897@0.4323878	286.1897	0.43	POS	hilic				6.87E-01	2.59E-01	0.30	4.40E-01	-0.18	7.90E-01	-0.07	9.51E-01	-0.02
286.2151@9.648907	286.2151	9.65	POS	RP				5.42E-01	4.70E-01	0.20	9.27E-01	0.03	1.08E-01	0.59	4.45E-01	0.19
286.2281@10.460352	286.2281	10.46	POS	RP				8.16E-01	2.11E-01	0.46	5.16E-01	0.21	5.39E-01	0.16	4.05E-01	0.23
286.8827@15.280678	286.8827	15.28	POS	RP				1.50E-01	1.91E-01	0.35	1.11E-01	0.52	3.56E-01	0.27	2.07E-03	0.90
287.0044@1.833017	287.0044	1.83	NEG	hilic				7.85E-02	1.08E-01	0.40	1.79E-02	-0.48	9.25E-01	-0.03	3.54E-01	0.23
287.2104@5.631093	287.2104	5.63	POS	RP	Acylcarnitine 08:0	10 eV: 288.2157 (100), 85.0290 (30), 229.1421 (25)	1	1.54E-01	4.47E-01	-0.29	1.44E-02	0.64	3.55E-01	0.23	2.64E-01	0.31
287.211@0.84693193	287.2110	0.85	POS	hilic				4.21E-01	7.78E-01	-0.10	4.01E-02	0.56	1.70E-01	0.35	6.07E-01	0.15
287.2254@7.241788	287.2254	7.24	POS	RP				5.59E-01	5.77E-01	-0.14	2.04E-01	0.33	2.33E-01	0.33	9.79E-01	0.01
287.2263@0.5374814	287.2263	0.54	POS	hilic				3.97E-01	6.11E-01	0.13	3.35E-01	-0.19	6.66E-01	0.10	5.69E-02	-0.33
287.2466@8.766212	287.2466	8.77	POS	RP				1.58E-01	9.22E-03	0.64	4.51E-02	0.36	1.80E-02	0.58	2.57E-02	0.46
287.2829@0.5876102	287.2829	0.59	POS	hilic				2.16E-06	4.09E-03	-0.79	1.61E-04	-0.81	4.10E-06	-1.12	6.24E-11	-1.45
287.283@7.6257596	287.2830	7.63	POS	RP				5.81E-06	7.62E-03	-0.74	1.88E-04	-0.80	8.33E-06	-1.07	6.26E-11	-1.42
287.2831@7.803516	287.2831	7.80	POS	RP				3.83E-06	2.13E-03	-0.81	1.77E-04	-0.81	2.09E-05	-1.08	8.38E-11	-1.43
287.8914@15.267842	287.8914	15.27	NEG	RP				3.41E-01	9.07E-03	-0.70	1.64E-01	-0.42	3.10E-01	-0.29	7.67E-02	-0.62
287.9576@4.926044	287.9576	4.93	NEG	hilic				7.61E-01	3.91E-01	0.27	2.57E-01	0.31	8.34E-01	-0.05	3.70E-01	0.27
288.0101@6.0310907	288.0101	6.03	POS	hilic				3.24E-01	1.30E-01	-0.44	6.84E-01	0.12	6.55E-02	-0.43	1.13E-01	-0.48
288.0297@2.9338334	288.0297	2.93	NEG	RP				5.35E-01	6.90E-01	-0.10	6.63E-01	-0.10	2.57E-01	-0.27	3.23E-02	-0.45
288.0303@0.4913888	288.0303	0.49	NEG	hilic				7.80E-01	5.83E-01	-0.14	7.95E-01	-0.06	4.34E-01	-0.19	1.02E-01	-0.34
288.0397@1.2474953	288.0397	1.25	POS	hilic				8.03E-02	1.34E-01	-0.46	8.43E-02	0.52	1.93E-01	0.35	7.08E-01	-0.12
288.0467@7.0237393	288.0467	7.02	POS	hilic				5.59E-02	2.95E-02	0.57	2.74E-02	0.58	3.40E-03	0.81	4.77E-01	0.14
288.1118@0.4978841	288.1118	0.50	POS	hilic				2.43E-01	1.49E-01	-0.48	5.70E-01	-0.15	9.16E-03	-0.77	4.19E-01	-0.24
288.843@0.5446383	288.8430	0.54	NEG	hilic				1.68E-01	7.20E-01	0.11	7.87E-01	0.07	2.70E-01	0.29	1.92E-02	-0.57
289.2411@8.025526	289.2411	8.03	POS	RP				2.94E-01	5.60E-02	-0.36	3.23E-01	-0.21	7.02E-01	0.09	3.25E-01	0.25
289.8404@3.0704625	289.8404	3.07	POS	hilic				9.64E-01	6.62E-01	0.15	4.55E-01	0.18	4.00E-01	0.25	5.71E-01	0.18
289.8836@4.414106	289.8836	4.41	NEG	hilic				3.85E-01	1.59E-01	0.46	8.59E-01	-0.04	2.38E-01	-0.27	7.91E-01	0.07
290.0744@1.5430768	290.0744	1.54	NEG	hilic				5.25E-01	6.79E-01	0.14	3.00E-01	0.25	6.82E-03	0.64	3.78E-01	0.26
290.1746@8.9525385	290.1746	8.95	POS	RP				8.27E-01	7.79E-01	-0.08	4.44E-01	0.19	4.50E-01	-0.26	8.04E-01	-0.07
290.1886@8.805338	290.1886	8.81	POS	RP				9.32E-01	7.53E-01	-0.12	4.63E-01	-0.18	6.84E-01	0.12	8.82E-01	0.05
290.9513@1.890464	290.9513	1.89	NEG	RP				3.36E-04	1.58E-01	-0.42	1.47E-03	0.78	3.03E-04	1.00	5.20E-02	0.51
291.044@6.737565	291.0440	6.74	NEG	hilic				7.54E-01	5.68E-01	-0.20	2.99E-01	0.27	7.79E-01	0.08	4.45E-01	0.25
291.9414@15.276582	291.9414	15.28	NEG	RP				9.97E-01	7.80E-01	-0.10	8.43E-01	-0.06	7.62E-01	-0.08	9.83E-01	0.01
292.0907@7.2404613	292.0907	7.24	POS	hilic				3.95E-01	2.19E-01	0.42	9.25E-01	0.03	1.08E-01	0.45	2.77E-02	0.56
292.091@7.437775	292.0910	7.44	POS	hilic				4.98E-01	1.64E-01	0.47	8.13E-01	0.07	1.88E-01	0.40	5.37E-02	0.52
292.0911@7.4378233	292.0911	7.44	POS	hilic				5.93E-01	4.06E-01	0.26	8.90E-01	0.04	2.20E-01	0.39	5.99E-02	0.50
292.0911@7.6997	292.0911	7.70	POS	hilic				5.00E-01	9.83E-02	0.56	6.13E-01	0.15	1.81E-01	0.42	5.87E-02	0.50
292.0912@6.195631	292.0912	6.20	POS	hilic				3.60E-01	2.30E-02	0.68	5.76E-01	0.18	6.77E-01	0.11	5.28E-02	0.51
292.1673@8.42509	292.1673	8.43	POS	RP				7.34E-01	6.42E-01	0.15	1.17E-01	0.48	6.05E-01	0.16	3.14E-01	0.32
292.1718@10.425628	292.1718	10.43	POS	RP				6.77E-04	1.36E-02	-0.58	4.71E-03	0.77	4.75E-02	0.47	6.97E-01	-0.10
292.1842@1.0586128	292.1842	1.06	POS	hilic				7.87E-01	7.72E-01	0.09	9.97E-01	0.00	3.44E-01	0.25	1.77E-01	0.37
292.204@9.534099	292.2040	9.53	POS	RP				5.62E-01	1.17E-01	-0.50	5.49E-01	-0.18	7.22E-01	0.11	8.75E-01	0.05
292.2041@0.43784413	292.2041	0.44	POS	hilic				9.58E-01	9.31E-01	-0.02	7.19E-01	0.10	7.98E-01	-0.08	5.81E-01	0.19
293.1034@0.533198	293.1034	0.53	POS	hilic				2.79E-01	9.45E-03	-0.75	6.12E-02	-0.59	3.52E-01	-0.27	3.03E-01	-0.26
293.932@4.350726	293.9320	4.35	POS	hilic				8.35E-01	3.08E-01	0.28	2.23E-01	0.36	7.77E-01	0.08	3.66E-01	0.29
293.9936@5.2778707	293.9936	5.28	POS	hilic				8.31E-01	3.67E-01	0.34	2.81E-01	0.26	1.51E-01	0.38	5.04E-01	0.17

Supplementary table 2: Metabolomics analysis results

Compound	Mass	Retention Time	Ionization mode	column	Compound_ID	MSMS_spectra	ID_level	ANOVA	Anorexia vs control		overweight vs control		obese vs control		athletes vs control	
								P-FDR	P	Estimate	P	Estimate	P	Estimate	P	Estimate
294.0406@1.4076029	294.0406	1.41	NEG	hilic				1.95E-01	1.08E-01	0.38	1.96E-01	0.32	1.05E-02	0.76	5.01E-02	0.45
294.0558@4.900211	294.0558	4.90	NEG	hilic				3.03E-03	1.76E-01	-0.18	2.15E-03	0.73	2.87E-02	0.57	8.83E-01	0.03
294.0617@1.2270635	294.0617	1.23	NEG	hilic				7.38E-01	7.91E-01	-0.07	3.65E-01	0.20	3.78E-01	-0.18	5.83E-01	0.14
294.0698@6.870387	294.0698	6.87	POS	hilic				2.08E-01	1.77E-02	-0.61	4.70E-01	0.19	5.54E-01	-0.16	6.78E-01	-0.11
294.1795@4.8985	294.1795	4.90	NEG	hilic				2.26E-01	3.73E-01	0.25	8.08E-03	0.69	4.90E-01	0.18	1.31E-02	0.52
294.1824@0.9876628	294.1824	0.99	NEG	hilic				2.63E-02	2.27E-03	0.88	1.40E-01	0.37	1.13E-01	0.42	6.46E-04	0.93
294.1825@0.42453837	294.1825	0.42	NEG	hilic				3.48E-01	6.13E-01	-0.17	9.64E-02	0.44	1.87E-01	0.39	6.55E-01	-0.13
294.1828@8.113557	294.1828	8.11	NEG	RP				4.60E-01	1.56E-01	0.47	7.12E-01	-0.09	9.49E-01	0.02	4.28E-01	-0.26
294.1839@8.10299	294.1839	8.10	POS	RP				9.58E-01	7.29E-01	-0.10	7.17E-01	0.11	5.68E-01	0.16	7.09E-01	0.11
294.1877@10.703872	294.1877	10.70	POS	RP				6.85E-01	3.64E-01	0.24	9.66E-01	-0.02	8.29E-01	0.07	2.03E-01	-0.36
294.2193@10.771666	294.2193	10.77	POS	RP				1.29E-01	1.27E-02	-0.45	3.63E-01	0.22	3.90E-01	0.19	2.02E-01	0.35
294.2196@0.43416536	294.2196	0.43	POS	hilic				5.64E-02	3.46E-02	-0.62	2.63E-01	0.34	9.21E-01	0.03	1.86E-01	0.44
294.8769@15.2575	294.8769	15.26	POS	RP				8.39E-01	4.15E-01	-0.24	3.35E-01	-0.29	7.77E-01	0.09	8.19E-01	-0.07
295.1243@9.78987	295.1243	9.79	POS	RP				1.99E-01	2.77E-01	-0.32	5.69E-02	0.52	9.89E-01	0.00	2.24E-01	0.34
295.1429@2.0621147	295.1429	2.06	POS	hilic				1.73E-01	7.20E-01	-0.09	2.93E-01	0.26	1.33E-02	-0.51	7.51E-01	0.09
295.2154@8.871355	295.2154	8.87	POS	RP				7.34E-01	6.65E-01	0.14	8.12E-01	0.06	1.93E-01	0.43	2.12E-01	0.37
295.8892@15.260258	295.8892	15.26	NEG	RP				7.23E-01	7.21E-01	-0.10	9.72E-01	-0.01	1.24E-01	0.40	7.49E-01	0.09
295.9893@15.326149	295.9893	15.33	NEG	RP				1.72E-01	2.06E-02	-0.61	9.47E-01	0.02	4.54E-01	-0.23	1.64E-02	-0.65
295.9893@5.498142	295.9893	5.50	NEG	RP				8.52E-01	2.73E-01	-0.35	6.05E-01	-0.15	2.58E-01	-0.33	3.01E-01	-0.30
296.0365@2.0536983	296.0365	2.05	NEG	hilic				2.26E-03	2.55E-03	0.90	8.29E-01	0.05	6.27E-01	0.12	4.39E-02	-0.42
296.0685@4.982719	296.0685	4.98	NEG	hilic				4.23E-02	1.78E-02	0.64	5.74E-01	-0.09	9.96E-01	0.00	7.00E-01	-0.07
296.2343@9.478251	296.2343	9.48	NEG	RP				3.07E-01	1.31E-02	-0.60	1.82E-01	-0.32	6.40E-02	-0.47	7.23E-01	-0.10
296.2348@9.627304	296.2348	9.63	NEG	RP				3.43E-01	5.82E-01	0.13	2.26E-01	0.37	2.46E-02	0.66	8.12E-01	0.07
296.2351@0.4268736	296.2351	0.43	NEG	hilic				3.53E-02	2.16E-02	-0.62	6.29E-01	0.14	8.50E-01	-0.05	7.21E-02	0.56
296.2353@9.623126	296.2353	9.62	POS	RP				5.89E-01	6.97E-01	-0.10	1.62E-01	0.42	9.93E-01	0.00	6.21E-01	-0.16
296.2355@0.4269142	296.2355	0.43	POS	hilic				4.53E-04	8.56E-04	-1.01	1.18E-01	0.39	3.69E-01	0.21	1.34E-01	0.45
296.2355@9.625125	296.2355	9.63	POS	RP				5.05E-01	7.35E-01	-0.09	1.76E-01	0.40	9.71E-01	0.01	3.98E-01	-0.29
296.2357@10.77779	296.2357	10.78	POS	RP				5.45E-03	8.21E-02	-0.42	2.82E-02	0.57	1.17E-01	0.35	1.70E-02	0.76
296.2711@10.936769	296.2711	10.94	NEG	RP				2.58E-02	2.35E-02	-0.42	1.99E-01	0.33	1.55E-01	0.35	1.86E-01	-0.28
296.2712@10.3687105	296.2712	10.37	POS	RP				1.70E-01	4.25E-02	-0.50	6.40E-01	0.14	3.42E-01	0.26	1.30E-01	-0.45
296.2715@10.730783	296.2715	10.73	POS	RP				9.11E-01	4.87E-01	-0.23	9.07E-01	0.03	8.45E-01	0.06	5.03E-01	-0.20
296.9111@15.44535	296.9111	15.45	NEG	RP				1.53E-01	2.48E-03	0.91	1.24E-01	0.45	1.68E-01	0.37	4.17E-01	0.21
297.0099@6.6892853	297.0099	6.69	POS	hilic				7.40E-01	3.01E-01	-0.29	5.76E-01	0.15	4.69E-01	-0.22	9.63E-01	0.01
297.1079@1.9704024	297.1079	1.97	POS	hilic				6.82E-01	1.56E-01	-0.48	6.85E-01	-0.11	4.43E-01	-0.20	1.82E-01	-0.43
297.1934@0.5209269	297.1934	0.52	POS	hilic				9.32E-01	5.32E-01	-0.15	8.14E-01	-0.05	8.83E-01	-0.03	6.61E-01	0.12
297.3024@10.808884	297.3024	10.81	POS	RP				8.82E-01	5.93E-01	-0.16	6.87E-01	-0.10	3.76E-01	-0.27	7.31E-01	0.11
297.8892@3.1991267	297.8892	3.20	NEG	hilic				9.59E-01	5.65E-01	0.20	7.60E-01	-0.08	9.95E-01	0.00	7.89E-01	0.08
297.8893@3.1973069	297.8893	3.20	NEG	hilic				9.76E-01	6.32E-01	0.18	9.38E-01	-0.02	7.15E-01	0.11	6.06E-01	0.15
297.8894@3.1963253	297.8894	3.20	NEG	hilic				8.28E-01	2.56E-01	0.40	2.89E-01	0.29	2.71E-01	0.33	4.73E-01	0.23
298.1108@9.048948	298.1108	9.05	POS	RP				7.40E-01	2.53E-01	0.37	3.23E-01	0.31	2.35E-01	0.33	1.10E-01	0.47
298.1318@11.213074	298.1318	11.21	POS	RP				7.88E-01	3.15E-01	-0.33	8.55E-01	-0.06	1.69E-01	-0.36	8.54E-01	-0.05
298.1322@2.3926585	298.1322	2.39	POS	hilic				4.80E-01	6.42E-01	-0.12	6.28E-01	-0.12	1.94E-02	-0.50	8.05E-01	-0.06
298.1322@5.179577	298.1322	5.18	POS	RP				8.14E-02	3.29E-01	-0.30	3.75E-01	-0.22	5.30E-04	-0.91	3.55E-01	-0.26
298.1324@6.4504232	298.1324	6.45	POS	RP				7.98E-01	8.42E-01	-0.05	5.88E-01	-0.16	1.36E-01	-0.41	4.76E-01	-0.20
298.1326@5.353987	298.1326	5.35	POS	RP				5.24E-01	6.93E-01	-0.12	5.48E-01	-0.17	2.84E-02	-0.58	5.86E-01	-0.16
298.1326@6.526667	298.1326	6.53	POS	RP				5.36E-01	8.77E-02	-0.54	1.17E-01	-0.46	7.24E-02	-0.49	2.07E-01	-0.38
298.1327@6.5412188	298.1327	6.54	POS	RP				6.74E-01	8.30E-01	-0.06	4.61E-01	-0.24	6.68E-02	-0.50	2.68E-01	-0.34
298.1329@7.6015058	298.1329	7.60	POS	RP				8.42E-01	4.21E-01	-0.24	7.00E-01	-0.10	3.69E-01	-0.26	2.19E-01	-0.37
298.133@11.107923	298.1330	11.11	POS	RP				1.76E-01	1.73E-01	-0.38	8.17E-01	-0.06	2.77E-03	-0.66	7.56E-02	-0.43
298.1334@0.4224296	298.1334	0.42	POS	hilic				5.57E-01	4.86E-01	-0.25	7.93E-01	-0.08	7.67E-02	-0.50	6.81E-01	0.11
298.1599@10.166212	298.1599	10.17	NEG	RP				6.05E-01	2.75E-01	0.27	9.30E-01	-0.02	9.59E-01	-0.01	1.72E-01	0.37
298.1599@10.341072	298.1599	10.34	NEG	RP				4.74E-01	2.35E-01	-0.31	5.62E-02	-0.44	6.40E-01	0.13	4.25E-01	-0.23
298.1601@10.344581	298.1601	10.34	NEG	RP				4.89E-01	3.34E-01	-0.27	1.04E-01	-0.41	4.76E-01	0.19	5.04E-01	-0.18
298.1946@9.422096	298.1946	9.42	POS	RP				9.97E-01	9.81E-01	0.01	9.22E-01	-0.02	7.01E-01	-0.10	8.34E-01	-0.06
298.1949@9.707261	298.1949	9.71	POS	RP				3.92E-01	7.11E-01	0.12	2.65E-02	0.67	7.75E-02	0.43	3.88E-01	0.27
298.2492@10.9218	298.2492	10.92	POS	RP				8.64E-03	1.38E-02	-0.72	4.27E-02	0.56	5.65E-01	0.14	4.36E-01	0.21
298.2503@10.272017	298.2503	10.27	NEG	RP				1.12E-01	1.80E-01	-0.41	8.85E-02	0.52	8.28E-02	0.45	7.59E-01	0.10
298.2505@9.651842	298.2505	9.65	NEG	RP				2.73E-01	1.79E-01	-0.41	2.02E-01	0.38	3.50E-01	0.26	7.31E-01	-0.10
298.2505@9.897776	298.2505	9.90	NEG	RP				5.08E-01	7.99E-01	0.07	1.06E-01	0.44	1.03E-01	0.55	2.97E-01	0.29
298.2506@0.42670065	298.2506	0.43	NEG	hilic				4.10E-05	9.39E-04	-0.94	3.27E-03	0.77	7.21E-02	0.45	1.66E-01	0.33
298.2506@10.400566	298.2506	10.40	NEG	RP				4.04E-01	7.91E-01	0.07	6.83E-02	0.57	6.96E-02	0.48	8.34E-01	0.07
298.2508@9.856786	298.2508	9.86	NEG	RP				6.50E-01	7.92E-01	-0.07	5.21E-01	0.19	1.26E-01	0.47	9.72E-01	0.01
298.2509@11.021789	298.2509	11.02	POS	RP				8.14E-03	9.09E-03	-0.85	1.61E-01	0.40	6.58E-01	0.11	2.65E-01	0.30
298.251@9.903797	298.2510	9.90	NEG	RP				9.35E-01	9.01E-01	0.03	8.33E-01	0.06	5.80E-01	0.17	5.74E-01	-0.15
298.2513@10.072761	298.2513	10.07	POS	RP				2.45E-02	9.54E-01	-0.02	2.01E-03	0.95	4.03E-02	0.58	6.06E-01	0.15
298.2513@10.163925	298.2513	10.16	POS	RP				7.60E-03	1.18E-01	-0.46	8.21E-03	0.79	3.67E-02	0.51	8.22E-01	0.07
298.2513@9.846223	298.2513	9.85	POS	RP				5.50E-02	6.83E-01	-0.12	1.34E-01	0.44	1.65E-01	0.36	7.50E-02	-0.55
298.2513@9.909349	298.2513	9.91	POS	RP				3.87E-01	7.60E-01	-0.09	5.94E-02	0.52	1.91E-01	0.35	8.29E-01	-0.06
298.2514@10.785875	298.2514	10.79	NEG	RP				8.33E-02	3.64E-01	-0.22	5.46E-02	0.53	4.12E-02	0.49	8.23E-01	-0.06
298.2516@10.382618	298.2516	10.38	POS	RP				9.42E-01	8.71E-01	-0.04	5.82E-01	0.14	6.81E-01	-0.09	7.90E-01	-0.06
298.2517@0.42674327	298.2517	0.43	POS	hilic				6.24E-05	5.46E-04	-0.99	1.34E-02	0.62	5.55E-02	0.49	1.61E-01	0.35
298.2865@11.2218895	298.2865	11.22	NEG	RP				4.83E-01	4.12E-01	-0.24	6.77E-01	-0.13	2.16E-01	0.31	1.87E-01	-0.35
299.193@7.5310283	299.1930	7.53	POS	RP				2.49E-03	2.46E-04	-1.07	8.57E-01	0.04	4.51E-01	-0.20	3.29E-01	0.31

Supplementary table 2: Metabolomics analysis results

Compound	Mass	Retention Time	Ionization mode	column	Compound_ID	MSMS_spectra	ID_level	ANOVA P-FDR	Anorexia vs control P	Anorexia vs control Estimate	overweight vs control P	overweight vs control Estimate	obese vs control P	obese vs control Estimate	athletes vs control P	athletes vs control Estimate
299.2802@0.4373367	299.2802	0.44	POS	hilic				5.68E-01	7.01E-02	0.53	2.72E-01	0.27	9.77E-02	0.52	3.14E-01	0.32
299.2815@10.748162	299.2815	10.75	POS	RP				7.09E-01	9.42E-01	-0.02	1.26E-01	0.41	3.18E-01	0.28	6.24E-01	0.14
299.282@10.294	299.2820	10.29	POS	RP				4.80E-01	7.94E-01	-0.08	3.53E-01	0.26	1.70E-02	0.55	9.99E-01	0.00
299.2824@10.660548	299.2824	10.66	POS	RP				7.34E-01	5.41E-01	0.20	9.78E-01	0.01	9.11E-01	0.03	2.67E-01	-0.35
299.8872@3.1977568	299.8872	3.20	NEG	hilic				4.34E-01	6.41E-01	0.15	7.28E-01	0.09	3.64E-02	0.58	8.28E-02	0.50
300.1117@0.74374026	300.1117	0.74	POS	hilic				8.70E-01	7.82E-01	-0.08	6.25E-01	-0.13	4.69E-01	-0.19	5.77E-01	0.16
300.1119@10.455796	300.1119	10.46	POS	RP				6.51E-02	1.23E-01	-0.48	7.57E-01	-0.09	9.45E-04	-0.91	5.05E-01	-0.18
300.1121@0.4962137	300.1121	0.50	POS	hilic				6.52E-01	3.66E-01	-0.32	5.06E-01	-0.19	4.63E-02	-0.51	8.61E-01	-0.05
300.1708@8.873135	300.1708	8.87	POS	RP				8.42E-01	6.16E-01	0.17	9.41E-01	-0.02	2.33E-01	0.37	7.20E-01	0.11
300.2092@9.283719	300.2092	9.28	POS	RP				6.94E-01	4.40E-01	0.28	1.00E-01	0.50	5.81E-01	0.15	2.37E-01	0.35
300.2105@9.675068	300.2105	9.68	POS	RP				9.71E-01	7.54E-01	0.10	6.67E-01	0.12	7.42E-01	0.11	4.13E-01	0.25
300.2127@13.360285	300.2127	13.36	POS	RP				8.05E-01	1.87E-01	-0.36	4.84E-01	-0.22	9.18E-01	0.03	3.86E-01	-0.27
300.2458@7.9248824	300.2458	7.92	POS	RP				1.47E-02	1.24E-01	-0.41	2.55E-02	0.61	3.18E-01	0.29	1.32E-01	-0.43
300.2653@9.8743925	300.2653	9.87	NEG	RP				4.62E-01	3.19E-01	-0.23	2.50E-01	0.34	2.29E-01	0.36	2.99E-01	0.29
300.2658@0.4396153	300.2658	0.44	NEG	hilic				2.97E-01	8.59E-01	0.05	6.03E-02	0.52	2.47E-02	0.62	6.82E-01	0.13
300.2658@9.868834	300.2658	9.87	NEG	RP				1.58E-01	6.51E-03	-0.64	9.73E-01	-0.01	3.67E-01	0.25	4.86E-01	0.18
300.266@10.365685	300.2660	10.37	NEG	RP				9.19E-01	6.43E-01	-0.15	5.76E-01	0.15	4.88E-01	0.18	9.69E-01	-0.01
300.2661@10.275174	300.2661	10.28	NEG	RP				5.14E-01	2.12E-01	-0.36	1.65E-01	0.33	6.90E-01	0.13	9.97E-01	0.00
300.2661@10.528547	300.2661	10.53	NEG	RP				3.80E-01	5.43E-01	-0.18	5.69E-02	0.52	1.63E-01	0.39	5.56E-01	0.20
300.2662@10.6500435	300.2662	10.65	NEG	RP				4.70E-01	7.66E-02	0.47	1.27E-01	0.44	7.13E-02	0.53	8.48E-01	0.07
301.0115@6.2292295	301.0115	6.23	NEG	hilic				5.36E-01	6.15E-02	0.49	2.43E-01	0.28	1.73E-01	0.30	8.97E-02	0.47
301.0734@9.058429	301.0734	9.06	NEG	RP				7.58E-01	2.73E-01	0.32	1.62E-01	0.32	3.98E-01	0.18	1.76E-01	0.33
301.116@3.296352	301.1160	3.30	POS	hilic				5.48E-02	5.06E-02	0.59	7.45E-01	-0.07	1.16E-01	-0.34	7.63E-01	-0.07
301.1893@1.5425649	301.1893	1.54	POS	RP				7.96E-01	2.73E-01	0.31	9.96E-01	0.00	4.01E-01	0.25	2.92E-01	0.33
301.1894@1.558089	301.1894	1.56	POS	hilic				9.13E-01	8.19E-01	0.08	7.37E-01	0.10	5.65E-01	-0.17	5.38E-01	0.18
301.1895@1.2495177	301.1895	1.25	POS	hilic				9.03E-01	9.16E-01	-0.04	9.68E-01	0.01	3.32E-01	0.25	4.53E-01	0.22
301.1895@2.1128733	301.1895	2.11	POS	RP				7.70E-01	5.49E-01	0.18	6.72E-01	-0.12	6.42E-01	0.13	2.89E-01	0.35
301.2085@7.9143114	301.2085	7.91	POS	RP				2.57E-03	5.23E-03	-0.82	3.41E-02	0.52	3.95E-01	0.21	1.97E-01	0.41
301.2234@0.77945596	301.2234	0.78	POS	hilic				7.70E-01	1.94E-01	-0.34	6.93E-01	0.11	5.35E-01	-0.16	8.56E-01	0.06
301.2258@5.9964952	301.2258	6.00	POS	RP	Acylcarnitine 09:0	20 eV: 85.0276 (100), 302.2281 (20)	2	2.56E-01	1.57E-02	-0.73	8.06E-01	0.07	7.75E-01	-0.07	8.44E-01	-0.07
302.0673@1.9187362	302.0673	1.92	POS	hilic				1.84E-02	4.61E-03	0.96	8.57E-01	-0.04	8.58E-01	-0.05	7.00E-02	0.54
302.1371@5.956813	302.1371	5.96	POS	RP				1.91E-01	7.94E-03	0.83	4.39E-02	0.56	5.66E-02	0.52	4.60E-01	0.22
302.1399@0.43124983	302.1399	0.43	POS	hilic				4.30E-01	7.40E-02	-0.43	3.05E-02	-0.62	3.38E-01	-0.29	1.06E-01	-0.44
302.2225@0.43232203	302.2225	0.43	POS	hilic				6.07E-01	2.68E-01	-0.24	2.57E-01	0.28	6.95E-01	-0.10	8.33E-01	0.05
302.2242@10.334537	302.2242	10.33	NEG	RP				3.39E-02	1.85E-01	-0.39	1.73E-01	0.38	6.06E-02	0.54	2.15E-01	-0.38
302.245@10.707047	302.2450	10.71	NEG	RP				1.69E-01	8.48E-02	-0.46	3.05E-01	0.32	9.69E-02	0.39	6.05E-01	-0.16
303.0524@1.5475639	303.0524	1.55	NEG	hilic				4.76E-01	7.01E-02	0.50	9.75E-01	0.01	5.07E-01	-0.18	6.58E-01	-0.14
303.0945@4.329349	303.0945	4.33	POS	hilic				4.35E-01	6.13E-02	0.59	2.33E-01	0.35	8.61E-01	0.04	8.71E-01	-0.05
303.2049@1.5133165	303.2049	1.51	POS	hilic				1.68E-02	4.82E-02	-0.70	1.12E-01	0.45	3.41E-01	-0.25	2.42E-01	0.33
303.2051@4.1964235	303.2051	4.20	POS	RP				6.73E-03	5.08E-02	-0.64	5.40E-02	0.54	4.27E-01	-0.20	8.84E-02	0.48
303.2054@4.4480777	303.2054	4.45	POS	RP				2.33E-01	1.18E-01	-0.42	2.67E-01	0.32	7.96E-01	-0.07	3.35E-01	0.27
303.2237@8.182859	303.2237	8.18	POS	RP				1.54E-03	3.80E-03	-0.72	1.11E-02	0.59	8.70E-01	0.04	1.49E-01	0.46
303.2416@9.648539	303.2416	9.65	POS	RP				5.28E-01	3.77E-01	0.25	8.42E-01	0.06	9.72E-02	0.61	3.38E-01	0.24
303.8804@15.264363	303.8804	15.26	NEG	RP				9.44E-01	8.91E-01	-0.03	7.77E-01	-0.06	7.89E-01	0.06	5.43E-01	0.18
304.0025@4.821586	304.0025	4.82	NEG	hilic				8.41E-02	1.76E-02	0.68	2.61E-01	-0.31	9.98E-01	0.00	1.98E-01	0.33
304.2356@10.780301	304.2356	10.78	POS	RP				9.18E-01	6.18E-01	-0.13	6.89E-01	0.11	5.35E-01	-0.16	8.36E-01	0.06
304.2398@10.539195	304.2398	10.54	NEG	RP	FA 20:4	10 eV: 303.2331 (100)	2	6.66E-04	5.66E-03	-0.84	5.48E-02	0.50	3.42E-02	0.56	3.72E-01	-0.25
304.2404@10.532927	304.2404	10.53	POS	RP				2.68E-03	5.20E-03	-0.75	1.45E-02	0.67	4.19E-01	0.23	9.47E-01	0.02
305.0849@9.425764	305.0849	9.43	POS	RP				9.16E-01	3.10E-01	0.31	3.60E-01	0.26	6.65E-01	0.14	4.96E-01	0.23
305.2695@10.705384	305.2695	10.71	POS	RP				8.34E-01	9.33E-01	-0.02	6.61E-01	-0.13	3.21E-01	0.26	6.69E-01	-0.14
305.8819@15.270163	305.8819	15.27	NEG	RP				8.42E-01	3.63E-01	-0.25	5.80E-01	0.16	6.98E-01	-0.11	9.64E-01	-0.01
305.9194@15.282548	305.9194	15.28	NEG	RP				9.32E-01	8.24E-01	0.08	6.57E-01	-0.13	9.64E-01	-0.01	3.93E-01	-0.24
305.9207@7.9218974	305.9207	7.92	NEG	hilic				5.67E-01	5.11E-02	0.59	6.77E-01	0.11	7.20E-01	0.11	3.25E-01	0.31
306.2554@10.700768	306.2554	10.70	NEG	RP	FA 20:3	10 eV: 305.2493 (100)	2	1.87E-03	3.08E-02	-0.59	5.41E-02	0.52	1.02E-02	0.68	3.19E-01	-0.30
306.8782@15.2721815	306.8782	15.27	NEG	RP				5.34E-01	7.95E-01	0.07	5.32E-01	-0.17	2.39E-01	-0.41	9.34E-02	-0.49
306.9088@7.877441	306.9088	7.88	POS	hilic				6.47E-01	1.41E-01	0.46	2.27E-01	0.33	7.70E-01	0.10	1.12E-01	0.45
307.1827@10.312595	307.1827	10.31	POS	RP				3.67E-01	1.03E-01	-0.41	4.25E-01	0.24	8.32E-01	0.06	2.85E-01	-0.30
307.2003@8.95271	307.2003	8.95	POS	RP				7.79E-01	9.60E-01	0.02	2.38E-01	0.29	5.60E-01	-0.20	9.14E-01	-0.03
308.0749@1.9141203	308.0749	1.91	POS	hilic				5.96E-01	1.03E-01	0.47	5.91E-01	0.13	8.94E-01	0.03	2.12E-01	0.35
308.0906@5.2774954	308.0906	5.28	NEG	hilic				2.96E-01	1.47E-01	-0.41	1.21E-01	0.43	5.26E-01	0.17	8.14E-01	0.08
308.0916@5.2781253	308.0916	5.28	NEG	hilic				3.61E-01	5.92E-02	-0.52	3.14E-01	0.27	9.74E-01	0.01	9.40E-01	0.02
308.1457@10.424083	308.1457	10.42	POS	RP				8.18E-03	9.70E-03	-0.49	4.59E-02	0.53	6.72E-02	0.45	8.64E-01	-0.04
308.1487@1.9139824	308.1487	1.91	POS	hilic				3.69E-02	1.01E-02	0.93	8.34E-01	0.05	9.73E-01	-0.01	5.07E-02	0.57
308.1656@10.084469	308.1656	10.08	NEG	RP				4.10E-01	5.13E-02	0.50	5.14E-01	0.13	7.94E-01	0.06	9.37E-01	-0.02
308.2712@10.878491	308.2712	10.88	NEG	RP	FA 20:2	10 eV: 307.2631 (100)	2	1.27E-02	1.45E-02	-0.69	2.30E-01	0.35	1.10E-01	0.43	3.42E-01	-0.28
308.2725@10.308216	308.2725	10.31	POS	RP				1.64E-01	6.09E-01	-0.12	3.05E-01	0.25	7.44E-02	0.54	2.98E-01	-0.29
308.8635@15.308069	308.8635	15.31	NEG	RP				9.83E-01	8.01E-01	0.06	6.90E-01	0.10	7.18E-01	-0.08	9.40E-01	0.02
309.1945@5.237095	309.1945	5.24	POS	RP				1.95E-01	8.57E-01	-0.05	1.92E-02	0.56	2.16E-02	0.63	6.91E-01	0.12
309.1945@8.596343	309.1945	8.60	POS	RP				8.89E-01	2.05E-01	-0.33	5.43E-01	-0.18	9.26E-01	-0.02	7.55E-01	-0.08
309.1946@0.85320294	309.1946	0.85	POS	hilic				6.96E-01	7.45E-01	0.11	5.44E-01	0.15	9.10E-02	0.49	9.96E-01	0.00
309.1946@5.5598807	309.1946	5.56	POS	RP				6.81E-01	7.51E-01	0.11	9.18E-01	0.03	1.01E-01	0.48	9.09E-01	-0.04
309.2316@9.533342	309.2316	9.53	POS	RP				9.79E-01	8.31E-01	0.06	9.07E-01	0.03	8.61E-01	-0.06	5.93E-01	0.18
309.3037@10.766326	309.3037	10.77	POS	RP				4.12E-01	1.72E-01	-0.45	8.45E-01	0.06	8.15E-01	0.08	1.31E-01	-0.47

Supplementary table 2: Metabolomics analysis results

Compound	Mass	Retention Time	Ionization mode	column	Compound_ID	MSMS_spectra	ID_level	ANOVA	Anorexia vs control		overweight vs control		obese vs control		athletes vs control	
								P-FDR	P	Estimate	P	Estimate	P	Estimate	P	Estimate
309.9913@6.035566	309.9913	6.04	POS	hilic				1.59E-01	2.24E-02	-0.67	8.19E-01	0.07	4.81E-02	-0.51	1.05E-01	-0.48
310.0525@1.0361643	310.0525	1.04	NEG	hilic				3.02E-04	4.21E-03	0.86	3.41E-01	-0.21	8.53E-01	0.04	2.05E-03	-0.56
310.1632@10.702385	310.1632	10.70	POS	RP				3.24E-01	2.27E-02	0.68	4.39E-02	0.56	1.06E-02	0.61	2.60E-01	0.38
310.1776@7.985957	310.1776	7.99	NEG	RP				8.61E-01	4.49E-01	-0.23	5.29E-01	-0.18	7.35E-01	0.11	4.49E-01	-0.23
310.1786@7.9748273	310.1786	7.97	POS	RP				8.60E-01	5.45E-01	-0.17	8.23E-01	-0.07	5.23E-01	-0.21	5.55E-01	0.18
310.18@0.42744544	310.1800	0.43	NEG	hilic				7.44E-02	6.46E-02	-0.59	6.72E-01	-0.11	7.93E-01	-0.08	6.06E-03	-0.83
310.1803@11.037721	310.1803	11.04	NEG	RP				1.69E-01	1.01E-01	0.40	5.26E-01	-0.10	3.82E-01	0.20	3.95E-02	0.51
310.2143@0.4415125	310.2143	0.44	POS	hilic				3.59E-01	1.69E-01	-0.32	1.80E-01	0.39	6.06E-01	-0.15	8.86E-01	0.04
310.2869@11.101286	310.2869	11.10	NEG	RP				2.98E-02	4.27E-02	-0.55	1.12E-01	0.45	9.86E-02	0.45	6.49E-01	-0.13
310.2892@10.623282	310.2892	10.62	POS	RP				4.11E-01	1.85E-01	-0.34	4.13E-01	0.25	2.39E-01	0.34	6.27E-01	-0.15
310.2994@10.30832	310.2994	10.31	POS	RP				6.93E-02	8.14E-03	0.82	2.40E-01	0.33	3.17E-02	0.58	6.44E-03	0.89
310.2995@0.41463518	310.2995	0.41	POS	hilic				1.29E-02	1.34E-02	0.74	3.99E-01	0.20	7.72E-02	0.41	2.22E-04	0.98
311.1257@1.6977937	311.1257	1.70	POS	hilic				8.25E-01	5.90E-01	-0.15	7.35E-01	-0.10	5.45E-01	-0.16	4.43E-01	0.24
311.1528@0.42553246	311.1528	0.43	POS	hilic				3.37E-01	3.24E-02	-0.57	7.70E-01	-0.08	5.34E-01	0.17	8.68E-01	-0.05
311.1534@8.698426	311.1534	8.70	POS	RP				5.93E-01	3.40E-02	-0.47	8.25E-01	-0.05	9.96E-01	0.00	6.97E-01	-0.10
311.1538@8.690155	311.1538	8.69	POS	RP				4.84E-01	2.32E-02	-0.55	8.60E-01	-0.04	9.84E-01	-0.01	6.64E-01	-0.11
311.2097@0.8160796	311.2097	0.82	POS	hilic				5.87E-01	4.59E-01	-0.24	2.42E-01	0.30	6.34E-01	-0.14	4.60E-01	0.23
311.2098@0.8150695	311.2098	0.82	POS	hilic				8.45E-01	7.73E-01	0.09	2.69E-01	0.32	8.56E-01	0.05	3.29E-01	0.32
311.2102@5.831571	311.2102	5.83	POS	RP				6.21E-01	4.82E-01	-0.23	1.74E-01	0.35	9.67E-01	0.01	5.94E-01	0.16
311.2102@5.928282	311.2102	5.93	POS	RP				1.20E-01	5.88E-02	-0.53	2.78E-01	0.29	7.32E-01	-0.09	2.45E-01	0.37
311.3189@11.020178	311.3189	11.02	POS	RP				6.02E-01	1.08E-01	-0.51	8.35E-01	0.06	9.77E-01	-0.01	5.14E-01	-0.21
311.3195@11.051726	311.3195	11.05	POS	RP				9.76E-01	4.55E-01	-0.20	6.83E-01	-0.14	7.21E-01	-0.11	4.66E-01	-0.21
311.356@9.224542	311.3560	9.22	POS	RP				5.96E-01	4.53E-01	0.24	1.30E-01	-0.37	9.01E-01	0.04	5.23E-01	-0.19
311.8239@15.290377	311.8239	15.29	NEG	RP				4.07E-01	1.93E-01	-0.38	1.79E-01	0.37	4.98E-01	0.20	9.64E-01	-0.01
311.8654@15.29907	311.8654	15.30	NEG	RP				9.65E-01	9.97E-01	0.00	4.32E-01	0.22	9.18E-01	-0.03	8.24E-01	0.06
312.0671@1.042477	312.0671	1.04	NEG	hilic				1.13E-01	1.48E-01	0.42	2.38E-01	0.25	6.52E-03	0.58	5.08E-01	-0.11
312.0676@1.8185312	312.0676	1.82	NEG	hilic				1.53E-03	3.68E-04	1.04	6.21E-02	0.34	4.82E-02	0.39	5.98E-01	0.07
312.1369@9.218614	312.1369	9.22	POS	RP				7.72E-01	3.14E-01	-0.31	7.76E-01	-0.07	7.37E-01	-0.11	4.64E-01	0.21
312.1608@10.702241	312.1608	10.70	POS	RP				5.30E-01	8.69E-01	0.04	1.55E-01	0.45	1.13E-01	0.45	9.91E-01	0.00
312.1754@10.713162	312.1754	10.71	NEG	RP				9.35E-01	7.30E-01	0.09	5.29E-01	-0.15	8.76E-01	0.04	7.00E-01	0.10
312.1756@10.71319	312.1756	10.71	NEG	RP				6.06E-01	3.58E-01	0.24	4.24E-01	-0.14	2.56E-01	0.29	5.18E-01	0.14
312.176@11.037251	312.1760	11.04	NEG	RP				9.80E-01	5.19E-01	0.20	7.10E-01	0.10	8.86E-01	0.04	8.07E-01	0.06
312.218@10.374552	312.2180	10.37	POS	RP				9.32E-01	3.31E-01	-0.20	8.94E-01	0.03	9.17E-01	-0.02	8.81E-01	0.04
312.2286@0.4324118	312.2286	0.43	NEG	hilic				4.34E-01	3.83E-01	-0.23	1.21E-01	0.42	9.14E-01	0.03	4.40E-01	0.25
312.2309@0.4365658	312.2309	0.44	POS	hilic				7.40E-01	5.17E-01	0.17	1.21E-01	0.43	2.74E-01	0.31	6.38E-01	0.14
312.2655@10.460011	312.2655	10.46	POS	RP				9.17E-01	9.93E-01	0.00	3.21E-01	0.29	7.84E-01	0.09	9.23E-01	0.03
312.2659@10.450726	312.2659	10.45	POS	RP				4.19E-01	6.48E-01	-0.13	6.12E-01	0.16	5.39E-02	0.60	4.11E-01	0.22
312.2666@10.449067	312.2666	10.45	POS	RP				8.72E-01	8.67E-01	-0.05	5.66E-01	-0.19	7.52E-01	0.10	3.04E-01	-0.27
312.2667@9.86295	312.2667	9.86	POS	RP				1.42E-01	8.53E-01	0.05	9.58E-03	0.72	9.65E-01	-0.01	5.91E-01	-0.14
312.2668@11.028429	312.2668	11.03	POS	RP				1.13E-01	1.08E-02	-0.56	3.77E-01	0.21	7.47E-01	0.09	2.63E-01	0.32
312.2668@9.842572	312.2668	9.84	POS	RP				5.18E-01	9.12E-01	-0.03	6.77E-01	0.13	7.95E-02	0.51	5.98E-01	-0.13
312.2675@1.2979094	312.2675	1.30	POS	hilic				2.87E-01	2.78E-01	0.30	1.02E-01	-0.32	7.74E-01	0.08	2.21E-01	0.30
312.3023@11.428651	312.3023	11.43	NEG	RP	FA 20:0	10 eV: 311.2949 (100)	1	4.60E-01	3.27E-01	-0.29	9.63E-01	0.01	9.34E-02	0.41	5.04E-01	-0.19
312.3025@11.095747	312.3025	11.10	NEG	RP				6.67E-02	6.18E-01	0.15	5.22E-01	0.17	6.47E-02	0.47	4.19E-02	-0.53
313.2256@6.4196672	313.2256	6.42	POS	RP	Acylcarnitine 10:1	10 eV: 314.2337 (100), 85.0276 (37)	2	2.73E-03	7.24E-03	-0.84	7.48E-02	0.49	7.26E-01	-0.10	1.65E-01	0.42
313.2258@6.422121	313.2258	6.42	POS	RP				1.87E-01	1.57E-01	-0.53	2.56E-01	0.31	9.44E-01	-0.02	2.37E-01	0.34
313.2258@6.5804048	313.2258	6.58	POS	RP				1.18E-03	3.17E-03	-0.95	2.53E-01	0.31	2.49E-01	-0.29	9.54E-02	0.47
313.2262@0.77271837	313.2262	0.77	POS	hilic				1.97E-01	9.99E-02	-0.58	2.18E-01	0.33	6.84E-01	-0.11	6.32E-01	0.15
313.745@15.344595	313.7450	15.34	NEG	RP				8.38E-01	8.22E-01	0.09	2.96E-01	-0.29	3.54E-01	-0.25	7.19E-01	-0.11
313.8219@15.298482	313.8219	15.30	NEG	RP				1.99E-01	3.10E-01	0.27	1.56E-01	0.42	6.19E-03	0.84	3.47E-01	0.25
313.861@15.294777	313.8610	15.29	NEG	RP				1.05E-01	6.35E-03	0.85	3.95E-01	0.22	4.58E-01	0.24	1.16E-02	0.71
313.8868@3.4432192	313.8868	3.44	NEG	RP				8.39E-04	1.92E-03	0.97	5.88E-01	-0.09	4.12E-01	-0.14	4.45E-01	0.16
313.9094@15.263311	313.9094	15.26	NEG	RP				3.48E-01	3.33E-01	-0.27	2.46E-01	-0.33	2.09E-01	0.39	9.52E-01	0.02
313.915@3.4605126	313.9150	3.46	NEG	hilic				5.56E-01	5.30E-01	0.19	7.18E-01	-0.09	7.00E-01	0.12	7.51E-02	0.52
313.9151@3.442244	313.9151	3.44	NEG	hilic				7.04E-01	8.71E-01	0.05	9.42E-01	0.02	3.28E-01	-0.29	3.54E-01	0.26
314.0247@3.0841522	314.0247	3.08	NEG	RP				1.76E-02	9.60E-02	-0.51	7.46E-03	0.69	2.68E-01	0.34	9.37E-01	-0.02
314.0254@3.3293931	314.0254	3.33	NEG	RP				6.60E-02	2.19E-01	-0.37	1.65E-02	0.65	2.59E-01	0.34	8.12E-01	-0.06
314.0729@7.23771	314.0729	7.24	POS	hilic				5.95E-01	4.56E-01	0.23	9.33E-01	0.02	2.69E-01	0.33	5.65E-02	0.50
314.0731@6.1991644	314.0731	6.20	POS	hilic				4.83E-01	8.20E-02	0.59	2.75E-01	0.31	9.05E-01	0.03	9.34E-02	0.42
314.1198@6.078931	314.1198	6.08	NEG	hilic				6.19E-01	6.71E-01	-0.14	5.51E-01	-0.19	4.02E-02	-0.52	1.60E-01	-0.43
314.1262@0.43742734	314.1262	0.44	NEG	hilic				7.10E-01	2.27E-01	-0.42	6.08E-01	-0.14	8.37E-02	-0.45	5.99E-01	-0.16
314.1271@6.6970177	314.1271	6.70	POS	RP				5.04E-01	3.47E-01	-0.32	3.32E-01	-0.27	2.06E-02	-0.63	7.16E-01	-0.11
314.1271@6.8937206	314.1271	6.89	POS	RP				2.50E-01	3.76E-01	-0.30	4.64E-01	-0.20	3.14E-03	-0.81	5.13E-01	-0.20
314.1273@0.4451855	314.1273	0.45	POS	hilic				2.10E-01	4.16E-01	-0.26	5.10E-01	-0.18	1.58E-03	-0.81	4.37E-01	-0.23
314.1283@11.211065	314.1283	11.21	POS	RP				6.02E-01	3.67E-01	-0.25	2.86E-01	-0.29	3.86E-02	-0.48	5.31E-01	-0.15
314.187@9.535761	314.1870	9.54	POS	RP				6.27E-01	9.97E-01	0.00	1.72E-01	-0.43	7.92E-01	0.08	7.97E-01	0.08
314.1875@0.43740463	314.1875	0.44	POS	hilic				9.40E-01	6.25E-01	0.14	5.05E-01	0.17	9.14E-01	0.03	4.06E-01	0.26
314.1894@8.751238	314.1894	8.75	POS	RP				5.10E-01	1.58E-01	0.42	1.38E-01	0.40	8.84E-01	0.04	1.18E-01	0.53
314.2247@8.733323	314.2247	8.73	POS	RP				5.38E-01	1.19E-01	-0.23	7.29E-01	0.07	2.94E-01	0.26	9.83E-01	0.00
314.2448@0.4423482	314.2448	0.44	NEG	hilic				1.49E-01	7.09E-01	-0.10	4.22E-02	0.63	4.50E-02	0.46	9.50E-01	0.02
314.2452@10.352432	314.2452	10.35	NEG	RP				7.49E-01	6.67E-01	0.12	2.75E-01	0.31	6.92E-01	0.09	5.39E-01	-0.15
315.1145@11.508958	315.1145	11.51	POS	RP				8.57E-01	8.01E-01	0.07	9.64E-01	-0.01	3.03E-01	0.28	3.78E-01	0.24
315.1155@0.4229428	315.1155	0.42	POS	hilic				5.42E-01	8.95E-01	0.03	8.01E-01	-0.04	4.05E-01	0.19	7.26E-02	0.38

Supplementary table 2: Metabolomics analysis results

Compound	Mass	Retention Time	Ionization mode	column	Compound_ID	MSMS_spectra	ID_level	ANOVA	Anorexia vs control		overweight vs control		obese vs control		athletes vs control	
								P-FDR	P	Estimate	P	Estimate	P	Estimate	P	Estimate
315.2044@2.1912603	315.2044	2.19	POS	RP				9.65E-01	7.80E-01	-0.08	6.45E-01	-0.11	7.50E-01	0.08	8.57E-01	0.05
315.2046@0.6440233	315.2046	0.64	POS	hilic				9.25E-01	5.87E-01	-0.15	3.09E-01	-0.30	7.33E-01	-0.10	5.90E-01	-0.16
315.205@2.4513009	315.2050	2.45	POS	RP				8.96E-01	8.27E-01	-0.06	4.64E-01	-0.18	6.91E-01	0.11	7.51E-01	0.09
315.205@2.8197064	315.2050	2.82	POS	RP				6.72E-01	2.80E-01	-0.31	2.35E-01	-0.34	8.55E-01	0.05	9.08E-01	0.03
315.2228@0.42990276	315.2228	0.43	POS	hilic				3.98E-02	4.01E-01	-0.19	2.57E-02	0.63	4.97E-03	0.61	3.55E-01	0.24
315.2407@9.253602	315.2407	9.25	POS	RP				1.29E-01	1.82E-01	-0.39	1.54E-01	-0.41	2.11E-03	-0.79	8.94E-03	-0.68
315.242@6.899444	315.2420	6.90	POS	RP	Acylcarnitine 10:0	10 eV: 316.2483 (100), 85.0280 (26), 257.1766 (5)	1	1.63E-01	5.06E-01	-0.26	1.26E-02	0.64	4.96E-01	0.17	1.85E-01	0.37
315.242@0.7454953	315.2420	0.75	POS	hilic	Decanoylcarnitine	20 eV: 85.0285 (100), 316.2484 (10)	2	9.39E-01	7.71E-01	-0.10	6.71E-01	0.12	5.54E-01	0.16	5.39E-01	0.19
315.2776@9.914906	315.2776	9.91	POS	RP				4.02E-02	6.09E-01	0.16	6.44E-03	0.86	3.09E-03	0.81	4.55E-01	0.22
315.3141@8.299374	315.3141	8.30	POS	RP				9.53E-06	6.35E-03	-0.75	4.98E-04	-0.77	1.19E-05	-1.03	1.94E-10	-1.37
315.3143@0.5690689	315.3143	0.57	POS	hilic				8.78E-06	5.70E-03	-0.75	1.21E-03	-0.73	9.00E-06	-1.05	1.58E-10	-1.40
315.3143@8.437501	315.3143	8.44	POS	RP				8.78E-06	3.26E-03	-0.80	6.55E-04	-0.76	1.23E-05	-1.04	1.36E-10	-1.38
315.7418@15.344876	315.7418	15.34	NEG	RP				7.49E-01	3.91E-01	0.33	7.89E-01	-0.07	6.77E-01	-0.11	5.06E-01	-0.19
315.7725@15.315867	315.7725	15.32	NEG	RP				4.53E-01	6.39E-01	0.14	3.08E-01	0.32	7.73E-01	-0.08	5.20E-02	0.57
316.0483@5.602551	316.0483	5.60	NEG	hilic				6.33E-01	6.06E-02	-0.56	3.02E-01	-0.30	6.34E-01	-0.14	2.12E-01	-0.38
316.0483@5.6049013	316.0483	5.60	NEG	hilic				9.83E-01	8.43E-01	0.06	9.85E-01	-0.01	7.63E-01	0.11	6.43E-01	-0.12
316.0483@5.865211	316.0483	5.87	NEG	hilic				4.71E-01	2.85E-01	-0.33	3.03E-02	-0.56	2.25E-01	-0.36	8.17E-01	-0.06
316.0484@6.225009	316.0484	6.23	NEG	hilic				9.93E-01	8.78E-01	-0.05	7.06E-01	0.11	9.75E-01	0.01	7.85E-01	0.08
316.0484@7.3745413	316.0484	7.37	NEG	hilic				9.75E-01	5.32E-01	0.19	9.68E-01	-0.01	9.09E-01	-0.03	9.88E-01	0.00
316.0492@5.4066143	316.0492	5.41	POS	hilic				4.41E-01	6.92E-01	-0.14	7.36E-02	-0.56	6.26E-01	-0.15	5.80E-02	-0.54
316.173@10.347381	316.1730	10.35	POS	RP				3.30E-01	1.68E-01	-0.39	2.62E-01	0.32	4.90E-01	0.20	5.22E-01	-0.17
316.2409@10.460045	316.2409	10.46	POS	RP				5.22E-01	1.03E-01	0.59	9.91E-01	0.00	4.71E-01	0.18	2.25E-01	0.32
316.2608@0.437234	316.2608	0.44	POS	hilic				4.60E-01	3.51E-02	-0.43	5.02E-01	0.18	7.59E-01	-0.07	8.97E-01	-0.03
317.0483@5.11383	317.0483	5.11	NEG	hilic				3.32E-01	6.99E-01	0.08	6.41E-02	0.47	2.37E-02	0.51	1.24E-01	0.33
317.142@9.789287	317.1420	9.79	POS	RP				1.08E-01	2.10E-01	-0.35	3.32E-02	0.58	3.90E-01	0.20	3.93E-01	0.24
317.2565@9.650058	317.2565	9.65	POS	RP				5.22E-01	4.57E-01	0.20	8.73E-01	0.04	8.77E-02	0.59	8.33E-01	0.06
317.7703@15.316394	317.7703	15.32	NEG	RP				8.57E-01	9.07E-01	-0.04	6.35E-01	0.15	7.15E-01	-0.09	2.87E-01	0.29
317.9546@5.4135237	317.9546	5.41	NEG	hilic				3.79E-01	6.69E-01	0.14	7.36E-01	0.10	6.14E-02	-0.51	6.18E-01	0.16
317.9548@4.4918914	317.9548	4.49	NEG	hilic				9.94E-01	9.23E-01	-0.03	6.59E-01	-0.13	9.05E-01	-0.03	6.96E-01	-0.11
317.9548@4.495837	317.9548	4.50	NEG	hilic				9.87E-01	8.64E-01	-0.05	6.65E-01	-0.13	4.75E-01	-0.19	7.46E-01	-0.09
317.9719@3.4484766	317.9719	3.45	NEG	RP				1.32E-01	2.65E-02	0.70	5.44E-01	0.14	3.60E-01	-0.22	6.16E-01	0.14
318.1211@5.568117	318.1211	5.57	NEG	RP				2.95E-01	7.17E-01	-0.10	3.55E-01	-0.22	2.40E-03	-0.60	6.86E-01	-0.10
318.1213@5.51485	318.1213	5.51	NEG	RP				1.50E-01	2.73E-01	-0.31	1.58E-01	-0.35	9.35E-04	-0.76	5.65E-01	-0.15
318.1218@0.5322122	318.1218	0.53	POS	hilic				2.99E-01	3.73E-01	-0.25	1.57E-01	-0.35	6.13E-03	-0.64	7.86E-01	-0.07
318.122@5.4982557	318.1220	5.50	POS	RP				3.51E-01	3.42E-01	-0.32	4.15E-01	-0.22	5.72E-03	-0.74	5.82E-01	-0.16
318.1221@5.61381	318.1221	5.61	POS	RP				5.11E-01	5.57E-01	-0.18	4.45E-01	-0.21	2.27E-02	-0.61	7.15E-01	-0.11
318.1222@5.3124175	318.1222	5.31	POS	RP				5.22E-02	2.17E-01	-0.38	2.35E-01	-0.29	9.61E-05	-0.96	2.57E-01	-0.31
318.1223@0.7647361	318.1223	0.76	POS	hilic				1.73E-01	4.27E-01	-0.24	1.53E-01	-0.41	1.42E-03	-0.84	1.70E-01	-0.40
318.1586@1.3828269	318.1586	1.38	POS	hilic				9.17E-01	4.94E-01	-0.21	6.26E-01	0.14	7.93E-01	-0.07	9.36E-01	0.03
318.1881@10.562933	318.1881	10.56	POS	RP				2.50E-03	9.97E-04	-0.95	2.93E-01	0.31	1.68E-01	0.37	2.91E-01	-0.29
318.2774@0.42678565	318.2774	0.43	POS	hilic				5.58E-01	1.96E-01	0.37	1.48E-01	0.34	8.06E-01	0.06	7.49E-01	-0.08
318.2916@10.8578	318.2916	10.86	POS	RP				6.61E-01	1.03E-01	-0.40	1.95E-01	-0.32	4.82E-01	-0.18	2.22E-01	-0.30
319.0134@2.1542645	319.0134	2.15	NEG	hilic				5.50E-01	1.87E-01	0.37	5.26E-01	-0.17	3.46E-01	0.33	4.99E-01	0.18
319.1639@0.43850425	319.1639	0.44	POS	hilic				9.94E-01	7.90E-01	0.10	7.05E-01	0.10	9.41E-01	-0.02	9.88E-01	0.00
319.1639@5.9575987	319.1639	5.96	POS	RP				1.67E-01	4.01E-03	0.88	1.33E-01	0.37	9.70E-02	0.49	6.41E-01	0.14
319.1643@0.44128382	319.1643	0.44	POS	hilic				8.41E-01	4.67E-01	0.24	5.91E-01	-0.12	4.76E-01	0.18	8.85E-01	0.04
319.8578@15.261955	319.8578	15.26	NEG	RP				2.01E-01	8.79E-02	-0.53	7.15E-02	-0.52	7.20E-01	-0.10	1.80E-02	-0.75
320.1262@9.154517	320.1262	9.15	POS	RP				6.17E-01	5.38E-01	-0.16	4.14E-01	-0.18	4.93E-02	-0.38	1.55E-01	-0.30
320.2054@10.778076	320.2054	10.78	POS	RP				5.16E-02	7.73E-02	-0.51	1.21E-01	0.49	6.54E-02	0.48	9.35E-01	-0.02
320.2131@0.5715556	320.2131	0.57	POS	hilic				5.80E-01	1.72E-01	-0.25	1.28E-01	-0.27	7.33E-01	-0.07	1.58E-01	-0.26
320.2335@9.856947	320.2335	9.86	POS	RP				3.11E-01	5.13E-01	-0.20	7.63E-02	0.55	2.36E-01	0.31	9.28E-01	-0.03
320.3081@11.073271	320.3081	11.07	POS	RP				7.58E-01	2.93E-01	-0.34	8.22E-01	0.07	6.72E-01	0.14	5.82E-01	-0.17
321.1773@0.47475275	321.1773	0.47	POS	hilic				4.56E-01	1.00E+00	0.00	1.90E-01	0.33	9.33E-02	0.53	9.49E-01	-0.02
321.2681@10.533131	321.2681	10.53	POS	RP				1.57E-01	1.24E-01	-0.29	2.59E-01	0.30	5.12E-02	0.49	6.59E-01	0.11
321.8139@15.250915	321.8139	15.25	NEG	RP				5.42E-01	2.34E-01	-0.34	4.42E-01	-0.22	2.23E-01	-0.36	3.73E-02	-0.62
321.8533@15.263185	321.8533	15.26	NEG	RP				4.63E-01	2.67E-02	-0.65	1.29E-01	-0.42	5.72E-01	-0.16	2.19E-01	-0.43
321.8935@15.275522	321.8935	15.28	NEG	RP				8.14E-01	6.22E-01	0.17	8.64E-01	-0.05	4.19E-01	0.23	4.92E-01	-0.20
322.0691@2.0426083	322.0691	2.04	NEG	hilic				1.40E-01	1.74E-02	0.70	8.24E-01	0.05	3.94E-01	0.18	8.61E-02	0.44
322.0906@4.75833	322.0906	4.76	POS	hilic				1.29E-01	2.04E-02	-0.55	1.05E-01	0.41	6.71E-01	0.13	9.03E-01	-0.03
322.212@10.432822	322.2120	10.43	NEG	RP				1.92E-02	6.62E-04	-0.64	2.91E-01	0.26	6.99E-01	0.10	1.01E-01	-0.36
322.219@11.028181	322.2190	11.03	POS	RP				7.26E-01	9.99E-02	0.40	7.35E-01	0.11	6.15E-01	-0.16	6.80E-01	0.12
323.1282@0.58620787	323.1282	0.59	POS	hilic				1.91E-01	8.43E-01	-0.06	5.94E-01	-0.13	2.82E-03	-0.71	9.94E-01	0.00
323.1553@10.312486	323.1553	10.31	POS	RP				1.75E-04	5.73E-02	-0.53	1.51E-03	0.92	1.42E-03	0.78	8.64E-01	0.05
323.1951@5.478404	323.1951	5.48	POS	RP				6.02E-01	2.93E-01	0.38	9.17E-02	0.51	1.38E-01	0.42	6.53E-01	0.13
323.1953@0.44197312	323.1953	0.44	POS	hilic				3.98E-01	1.12E-02	0.67	4.44E-01	0.21	1.70E-01	0.45	7.22E-01	0.09
323.2466@9.53399	323.2466	9.53	POS	RP				8.45E-01	9.25E-01	-0.03	6.72E-01	-0.12	8.73E-01	0.05	3.47E-01	0.28
323.3196@10.9151125	323.3196	10.92	POS	RP				6.30E-01	1.15E-01	-0.40	3.80E-01	-0.23	8.00E-01	-0.07	1.40E-01	-0.36
323.7676@15.281125	323.7676	15.28	NEG	RP				9.27E-01	8.95E-01	0.05	6.47E-01	0.13	7.43E-01	-0.09	3.73E-01	0.24
323.81@15.285357	323.8100	15.29	NEG	RP				9.72E-01	6.43E-01	0.14	7.22E-01	-0.11	9.34E-01	0.02	8.47E-01	-0.06
323.8496@15.265295	323.8496	15.27	NEG	RP				1.49E-01	3.99E-01	-0.27	7.69E-03	-0.67	4.43E-01	0.22	2.90E-01	-0.33
323.9891@0.51215315	323.9891	0.51	NEG	hilic				4.35E-01	4.15E-01	-0.23	7.15E-02	0.41	9.20E-01	0.03	2.42E-01	0.33
323.9951@3.7521484	323.9951	3.75	NEG	hilic				1.27E-02	2.71E-01	-0.29	3.12E-02	0.54	3.62E-01	-0.20	3.51E-02	-0.57
323.9954@1.5433587	323.9954	1.54	NEG	hilic				9.40E-01	9.18E-01	-0.04	6.48E-01	0.10	5.75E-01	-0.12	5.24E-01	-0.21

Supplementary table 2: Metabolomics analysis results

Compound	Mass	Retention Time	Ionization mode	column	Compound_ID	MSMS_spectra	ID_level	ANOVA	Anorexia vs control		overweight vs control		obese vs control		athletes vs control	
								P-FDR	P	Estimate	P	Estimate	P	Estimate	P	Estimate
324.2275@10.708695	324.2275	10.71	NEG	RP				1.95E-01	9.14E-03	-0.72	2.70E-01	-0.38	7.23E-01	-0.09	1.68E-02	-0.66
325.334@11.193427	325.3340	11.19	POS	RP				6.46E-01	3.05E-01	-0.25	7.82E-01	-0.07	8.86E-01	0.04	1.36E-01	-0.38
325.7644@15.25061	325.7644	15.25	NEG	RP				9.16E-01	3.40E-01	0.32	6.36E-01	0.15	9.64E-01	0.01	5.39E-01	0.18
325.7648@15.281214	325.7648	15.28	NEG	RP				6.82E-01	3.67E-01	0.31	8.51E-01	0.06	1.97E-01	-0.29	8.10E-01	0.06
325.8021@15.2907	325.8021	15.29	NEG	RP				7.69E-01	8.10E-01	0.09	2.05E-01	-0.35	7.71E-01	0.08	9.48E-01	-0.02
325.9851@4.696825	325.9851	4.70	POS	hilic				8.65E-01	9.16E-01	-0.03	4.61E-01	-0.20	5.65E-01	0.19	8.51E-01	0.05
325.9932@1.5432433	325.9932	1.54	NEG	hilic				9.23E-01	5.03E-01	-0.23	8.79E-01	0.03	3.55E-01	-0.22	6.52E-01	-0.14
326.0014@7.843	326.0014	7.84	POS	RP				9.51E-01	2.93E-01	-0.29	6.95E-01	-0.11	6.03E-01	-0.18	6.23E-01	-0.15
326.0711@8.789572	326.0711	8.79	POS	RP				8.80E-01	2.36E-01	-0.37	4.37E-01	-0.24	4.44E-01	-0.25	6.84E-01	-0.13
326.1427@9.788642	326.1427	9.79	POS	RP				6.80E-02	3.01E-01	-0.33	2.66E-02	0.67	1.50E-01	0.36	1.76E-01	0.43
326.1926@13.587582	326.1926	13.59	POS	RP				4.20E-01	8.17E-01	0.06	1.90E-01	0.36	2.04E-01	-0.36	6.00E-01	-0.16
326.2814@10.314744	326.2814	10.31	NEG	RP				1.92E-01	5.10E-02	-0.50	5.48E-01	0.15	4.77E-01	0.20	2.50E-01	-0.33
327.201@0.57684	327.2010	0.58	POS	hilic				9.19E-01	8.09E-01	-0.05	9.01E-01	0.02	6.87E-01	-0.08	5.21E-01	0.15
327.223@10.276871	327.2230	10.28	POS	RP				1.98E-02	9.97E-03	-0.74	1.60E-01	0.38	3.20E-01	0.24	3.13E-01	-0.32
327.2413@6.8765984	327.2413	6.88	POS	RP	Acylcarnitine 11:1	10 eV: 328.2501 (100), 269.1758 (20), 85.0266 (13)	2	7.05E-04	5.99E-03	-0.75	7.10E-03	0.72	3.98E-02	0.55	5.60E-01	0.17
327.2415@0.74148196	327.2415	0.74	POS	hilic				8.33E-03	3.62E-02	-0.55	7.29E-03	0.71	7.62E-02	0.46	6.99E-01	0.13
327.278@9.914614	327.2780	9.91	POS	RP				2.29E-02	2.38E-01	0.31	4.98E-03	0.96	8.69E-04	0.85	4.28E-01	0.24
327.2781@10.052482	327.2781	10.05	POS	RP				8.18E-02	4.63E-02	-0.56	4.59E-01	0.22	2.96E-01	0.28	1.23E-01	-0.47
327.3137@10.664772	327.3137	10.66	POS	RP				2.25E-01	7.05E-01	0.11	2.88E-01	0.33	4.77E-03	0.80	5.27E-01	0.20
327.7627@15.246036	327.7627	15.25	NEG	RP				8.18E-01	9.90E-01	0.00	7.42E-01	-0.11	2.95E-01	-0.31	5.91E-01	0.15
327.8383@15.262514	327.8383	15.26	NEG	RP				7.77E-02	3.92E-03	-0.82	1.27E-01	-0.40	5.20E-01	0.18	7.85E-01	-0.09
327.8841@15.262	327.8841	15.26	NEG	RP				6.42E-01	6.64E-02	-0.53	1.41E-01	-0.41	4.71E-01	-0.22	2.26E-01	-0.36
327.9958@6.127795	327.9958	6.13	NEG	RP				5.24E-01	9.55E-01	-0.02	4.49E-01	-0.22	1.93E-01	0.35	3.65E-01	-0.29
328.1338@10.318391	328.1338	10.32	NEG	RP				2.26E-02	8.98E-01	-0.04	2.01E-03	0.97	2.81E-02	0.58	1.01E-01	0.49
328.2074@0.39637935	328.2074	0.40	POS	hilic				4.07E-01	4.07E-01	0.24	4.22E-01	0.22	1.27E-01	-0.40	4.75E-01	-0.23
328.2078@13.243678	328.2078	13.24	POS	RP				4.19E-01	4.45E-01	0.22	6.09E-02	0.49	5.18E-01	-0.17	7.68E-01	-0.10
328.2244@0.4508571	328.2244	0.45	POS	hilic				7.15E-04	7.41E-02	-0.23	8.85E-02	0.36	5.10E-04	0.93	8.26E-01	0.04
328.2401@10.51773	328.2401	10.52	NEG	RP	FA 22:6 (DHA)	10 eV: 327.2317 (100)	2	1.19E-02	5.10E-03	-0.82	3.48E-01	0.26	1.69E-01	0.39	4.03E-01	-0.27
328.2404@0.41654173	328.2404	0.42	NEG	hilic				1.72E-01	7.76E-03	-0.54	8.31E-01	-0.05	4.13E-01	0.21	3.00E-01	-0.27
328.2426@10.5178	328.2426	10.52	POS	RP				8.25E-03	3.80E-03	-0.77	7.81E-02	0.53	2.17E-01	0.30	8.62E-01	-0.05
328.261@10.333139	328.2610	10.33	NEG	RP				4.05E-02	3.65E-03	-0.84	6.59E-01	-0.12	1.86E-01	0.33	4.96E-01	-0.20
328.2631@10.164119	328.2631	10.16	POS	RP				9.05E-03	8.18E-01	-0.04	3.65E-01	0.22	3.08E-04	0.93	3.86E-01	0.19
328.2631@10.328408	328.2631	10.33	POS	RP				1.07E-02	4.46E-03	-0.81	4.65E-01	0.21	1.83E-01	0.33	1.20E-01	-0.39
328.2953@10.763298	328.2953	10.76	NEG	RP				4.93E-01	1.68E-01	-0.37	3.92E-01	0.23	8.08E-01	0.07	4.37E-01	-0.23
329.0857@1.5341775	329.0857	1.53	NEG	hilic				4.28E-01	9.88E-01	0.00	5.56E-01	-0.15	2.73E-01	0.28	8.02E-02	0.47
329.1049@9.797778	329.1049	9.80	NEG	RP				2.48E-01	6.92E-01	-0.12	3.06E-02	0.62	2.69E-01	0.28	1.57E-01	0.43
329.1497@11.213552	329.1497	11.21	POS	RP				2.79E-01	1.88E-01	-0.36	9.14E-01	-0.03	9.13E-03	-0.59	2.21E-01	-0.31
329.2196@0.5302941	329.2196	0.53	POS	hilic				8.31E-01	4.93E-01	-0.14	1.64E-01	-0.24	4.11E-01	-0.16	4.97E-01	-0.14
329.2413@8.42064	329.2413	8.42	POS	RP				7.89E-03	2.64E-03	-0.78	1.30E-01	0.37	8.00E-01	-0.07	1.58E-01	0.41
329.2546@0.69744587	329.2546	0.70	POS	hilic				6.82E-01	8.81E-01	-0.04	2.68E-01	0.31	4.70E-01	-0.17	5.58E-01	0.15
329.2553@0.43525594	329.2553	0.44	POS	hilic				5.70E-01	2.06E-01	0.35	7.68E-02	0.39	7.82E-01	-0.07	9.73E-01	0.01
329.2556@9.276838	329.2556	9.28	POS	RP				4.41E-01	5.29E-02	-0.47	6.30E-01	-0.15	2.40E-02	-0.61	4.02E-01	-0.23
329.256@7.3409257	329.2560	7.34	POS	RP				6.39E-01	6.15E-01	-0.14	3.22E-01	0.23	3.12E-01	-0.21	9.76E-01	-0.01
329.2569@7.2324996	329.2569	7.23	POS	RP				6.35E-03	3.30E-02	-0.70	1.92E-02	0.61	8.04E-01	-0.06	2.37E-01	0.34
329.7601@15.279377	329.7601	15.28	NEG	RP				7.01E-01	6.64E-01	-0.14	3.31E-01	-0.31	2.30E-01	-0.33	6.39E-01	0.14
329.7948@15.290085	329.7948	15.29	NEG	RP				9.62E-01	9.81E-01	0.01	9.91E-01	0.00	4.07E-01	0.24	9.80E-01	0.01
329.8434@15.260341	329.8434	15.26	NEG	RP				6.77E-01	7.82E-02	-0.50	2.58E-01	-0.35	7.97E-01	-0.07	3.55E-01	-0.29
329.8886@15.261208	329.8886	15.26	NEG	RP				3.15E-01	3.01E-02	-0.63	6.10E-02	-0.62	5.37E-01	-0.17	1.01E-01	-0.46
329.9628@2.4622648	329.9628	2.46	NEG	RP				4.52E-01	4.06E-02	-0.58	8.30E-01	0.07	8.51E-01	0.05	8.93E-01	0.04
330.0352@4.118633	330.0352	4.12	NEG	hilic				7.10E-01	9.78E-01	0.01	1.81E-01	0.39	2.39E-01	0.29	8.01E-01	0.07
330.1597@3.67599	330.1597	3.68	NEG	hilic				2.60E-01	1.24E-01	0.55	2.96E-01	0.27	7.02E-01	0.09	6.69E-03	0.70
330.2292@0.88478136	330.2292	0.88	POS	hilic				3.43E-01	3.12E-01	0.27	2.89E-01	-0.17	8.53E-02	0.37	7.61E-01	0.07
330.2551@10.700088	330.2551	10.70	NEG	RP	FA 22:5	10 eV: 329.2483 (100)	2	2.91E-02	8.34E-02	-0.54	7.16E-02	0.49	5.31E-02	0.53	9.10E-01	-0.04
330.2557@10.637848	330.2557	10.64	NEG	RP				2.73E-01	7.09E-01	-0.11	9.81E-02	0.42	3.39E-02	0.56	8.19E-01	-0.08
330.2581@10.62621	330.2581	10.63	POS	RP				8.78E-02	1.90E-01	-0.23	2.82E-01	0.27	9.38E-02	0.40	1.85E-01	-0.25
330.2767@11.0361805	330.2767	11.04	NEG	RP				2.23E-01	1.97E-01	-0.39	6.81E-01	0.13	4.88E-02	0.46	3.50E-01	-0.26
330.2771@10.442886	330.2771	10.44	POS	RP				3.90E-02	4.54E-02	-0.54	3.99E-01	0.24	4.69E-02	0.57	4.89E-01	-0.23
330.2771@3.2868826	330.2771	3.29	POS	hilic				9.55E-01	7.48E-01	0.09	4.61E-01	0.19	4.08E-01	0.25	5.56E-01	0.16
331.2359@5.928253	331.2359	5.93	POS	RP				1.50E-02	6.31E-02	-0.61	8.78E-02	0.47	5.61E-01	-0.14	8.72E-02	0.48
331.2364@1.1731907	331.2364	1.17	POS	hilic				1.41E-02	4.43E-02	-0.68	6.41E-02	0.52	6.14E-01	-0.13	2.19E-01	0.35
331.2366@6.0963683	331.2366	6.10	POS	RP				2.70E-02	3.90E-02	-0.62	1.64E-01	0.39	3.72E-01	-0.22	2.07E-01	0.35
331.2542@0.72825706	331.2542	0.73	POS	hilic				8.85E-02	8.40E-03	-0.61	8.13E-01	0.06	3.57E-01	-0.23	3.11E-01	0.30
331.2548@8.729207	331.2548	8.73	POS	RP				1.88E-03	2.15E-04	-0.98	3.91E-01	0.19	2.68E-01	-0.27	2.26E-01	0.37
331.2727@9.650244	331.2727	9.65	POS	RP				4.42E-01	8.36E-01	0.06	4.72E-01	-0.21	1.29E-01	0.51	6.67E-01	0.10
331.8424@15.265005	331.8424	15.27	NEG	RP				6.33E-01	6.86E-02	-0.50	7.59E-01	-0.10	7.13E-01	0.10	5.34E-01	-0.18
331.883@15.262085	331.8830	15.26	NEG	RP				2.54E-01	4.09E-02	-0.60	5.61E-02	-0.60	4.58E-01	-0.20	2.99E-02	-0.66
331.926@3.4648557	331.9260	3.46	NEG	hilic				5.20E-01	2.89E-01	-0.38	3.67E-01	-0.24	5.22E-01	0.18	1.38E-01	-0.38
331.9262@4.077751	331.9262	4.08	NEG	hilic				2.24E-01	6.13E-02	-0.51	7.97E-01	-0.07	6.20E-01	0.14	1.22E-01	0.35
331.9873@7.2200165	331.9873	7.22	NEG	RP				8.76E-01	5.80E-01	0.16	8.69E-01	-0.05	6.41E-01	-0.12	4.17E-01	-0.26
331.9885@7.544211	331.9885	7.54	NEG	RP				4.21E-01	5.39E-01	0.22	6.30E-01	-0.12	3.72E-01	-0.27	7.50E-02	-0.51
332.0501@4.777776	332.0501	4.78	POS	hilic				6.22E-01	9.73E-01	-0.01	7.03E-01	0.09	3.41E-01	0.24	1.18E-01	0.45
332.0974@2.043037	332.0974	2.04	NEG	hilic				1.13E-01	1.75E-02	0.71	9.65E-01	0.01	6.71E-01	0.09	1.36E-01	0.36
332.1205@6.737871	332.1205	6.74	NEG	hilic				4.20E-01	5.65E-01	-0.21	1.81E-01	0.37	8.10E-01	0.06	1.32E-01	0.45

Supplementary table 2: Metabolomics analysis results

								ANOVA	Anorexia vs control		overweight vs control		obese vs control		athletes vs control	
Compound	Mass	Retention Time	Ionization mode	column	Compound_ID	MSMS_spectra	ID_level	P-FDR	P	Estimate	P	Estimate	P	Estimate	P	Estimate
332.1367@0.59417963	332.1367	0.59	NEG	hilic				3.21E-01	8.07E-02	-0.50	2.51E-01	-0.32	1.62E-02	-0.61	7.26E-01	-0.09
332.1373@6.2819285	332.1373	6.28	POS	RP				6.02E-01	3.48E-01	-0.34	7.09E-01	-0.10	3.55E-02	-0.56	6.66E-01	-0.14
332.1375@5.7628884	332.1375	5.76	POS	RP				7.75E-01	3.48E-01	-0.33	7.77E-01	-0.08	1.22E-01	-0.42	7.49E-01	-0.10
332.1376@0.5808462	332.1376	0.58	POS	hilic				7.45E-01	3.81E-01	-0.31	6.56E-01	-0.12	7.55E-02	-0.48	5.79E-01	-0.18
332.1378@5.980673	332.1378	5.98	POS	RP				7.75E-01	3.21E-01	-0.34	8.96E-01	-0.04	1.48E-01	-0.39	7.70E-01	-0.10
332.1379@6.4410944	332.1379	6.44	POS	RP				6.21E-01	3.28E-01	-0.33	5.36E-01	-0.18	2.81E-02	-0.56	5.79E-01	-0.17
332.1451@10.3476	332.1451	10.35	POS	RP				1.82E-01	1.96E-01	-0.38	2.00E-01	0.38	1.15E-01	0.44	8.96E-01	-0.04
332.1831@7.757088	332.1831	7.76	NEG	RP				2.20E-01	5.54E-01	0.17	6.76E-02	-0.40	1.17E-01	-0.37	6.01E-01	0.13
332.2357@10.459287	332.2357	10.46	POS	RP				9.62E-01	8.10E-01	0.08	8.35E-01	-0.06	5.07E-01	-0.17	7.74E-01	0.08
332.2713@10.804096	332.2713	10.80	NEG	RP	FA 22:4	10 eV: 331.2636 (100)	2	1.68E-04	6.21E-03	-0.80	3.02E-02	0.56	6.64E-03	0.71	4.10E-01	-0.22
333.1031@1.0751429	333.1031	1.08	NEG	hilic				9.58E-03	5.58E-03	0.84	7.27E-02	0.33	3.18E-01	0.15	9.10E-01	-0.01
333.1988@10.424679	333.1988	10.42	POS	RP				1.58E-02	3.03E-02	-0.39	3.10E-02	0.58	1.37E-01	0.36	5.93E-01	-0.12
333.2972@0.4208947	333.2972	0.42	POS	hilic				4.41E-01	1.13E-01	-0.31	8.92E-02	-0.33	3.89E-01	-0.18	8.44E-02	-0.33
333.3037@10.696058	333.3037	10.70	POS	RP				3.69E-01	7.38E-02	-0.52	3.58E-01	-0.27	5.36E-01	0.17	2.95E-01	-0.30
333.7964@15.258537	333.7964	15.26	NEG	RP				3.34E-01	2.47E-03	-0.77	1.48E-01	-0.51	1.68E-01	-0.31	2.93E-01	-0.31
333.8392@15.265901	333.8392	15.27	NEG	RP				4.46E-01	1.33E-02	-0.65	4.67E-01	-0.24	4.87E-01	-0.17	9.68E-02	-0.49
333.8698@7.913965	333.8698	7.91	POS	hilic				8.47E-01	4.35E-01	-0.24	9.37E-01	0.02	5.12E-01	-0.20	3.17E-01	-0.29
334.047@2.0840166	334.0470	2.08	NEG	RP				2.31E-01	7.19E-02	-0.35	4.36E-01	0.20	1.15E-01	0.42	7.13E-01	0.09
334.047@2.4626832	334.0470	2.46	NEG	RP				8.62E-01	3.74E-01	-0.26	6.21E-01	0.16	7.23E-01	-0.11	7.21E-01	-0.12
334.1011@1.5386753	334.1011	1.54	NEG	hilic				6.73E-02	7.98E-01	0.08	1.97E-01	0.32	1.31E-04	0.98	2.33E-01	0.35
334.1599@10.562248	334.1599	10.56	POS	RP				1.18E-02	1.71E-02	-0.71	1.15E-01	0.48	1.01E-01	0.44	9.41E-01	-0.02
334.2137@0.43675	334.2137	0.44	POS	hilic				7.98E-01	4.93E-01	-0.18	5.02E-01	-0.18	8.97E-01	0.04	1.88E-01	-0.33
334.2155@10.300465	334.2155	10.30	POS	RP				4.97E-01	5.66E-01	0.15	6.67E-01	-0.11	2.75E-01	0.36	1.02E-01	0.45
335.2142@10.702274	335.2142	10.70	POS	RP				7.07E-01	6.47E-01	0.12	4.99E-01	0.22	9.20E-01	-0.03	2.83E-01	-0.32
335.3179@0.42610076	335.3179	0.43	POS	hilic				9.23E-01	4.94E-01	-0.21	8.34E-01	-0.06	5.85E-01	-0.17	3.49E-01	-0.29
335.318@10.423494	335.3180	10.42	POS	RP				8.38E-01	2.23E-01	-0.31	2.92E-01	-0.29	2.68E-01	-0.29	3.80E-01	-0.24
335.3195@10.073605	335.3195	10.07	POS	RP				4.73E-01	3.54E-01	-0.29	1.84E-01	0.40	8.31E-01	0.07	6.70E-01	-0.14
335.3195@10.857564	335.3195	10.86	POS	RP				7.55E-01	3.56E-01	-0.30	6.51E-01	0.13	6.84E-01	0.13	5.77E-01	-0.18
335.7938@15.257545	335.7938	15.26	NEG	RP				5.25E-01	1.30E-02	-0.60	1.73E-01	-0.44	2.01E-01	-0.31	1.48E-01	-0.48
335.8321@15.268266	335.8321	15.27	NEG	RP				7.27E-01	1.72E-01	-0.38	7.63E-01	-0.10	6.51E-01	0.12	3.65E-01	-0.27
336.0564@4.8213863	336.0564	4.82	NEG	hilic				1.89E-01	2.13E-01	0.36	5.99E-02	-0.51	8.40E-01	0.07	3.68E-01	0.23
336.1027@6.024018	336.1027	6.02	POS	hilic				7.27E-02	3.24E-02	-0.66	1.41E-01	-0.44	3.90E-03	-0.74	2.98E-03	-0.94
336.1351@3.8641005	336.1351	3.86	POS	hilic				9.72E-01	7.49E-01	-0.07	6.97E-01	0.11	6.92E-01	-0.10	8.47E-01	-0.05
336.1366@3.9081597	336.1366	3.91	POS	hilic				6.54E-01	5.16E-01	0.18	5.02E-01	0.22	4.65E-01	-0.21	2.26E-01	0.34
336.1368@4.161385	336.1368	4.16	POS	hilic				4.63E-01	6.57E-01	-0.14	5.33E-02	0.54	5.27E-01	0.18	7.15E-01	0.09
336.1513@9.788982	336.1513	9.79	POS	RP				2.58E-01	4.80E-01	-0.22	3.37E-02	0.61	3.61E-01	0.24	3.55E-01	0.29
336.1605@10.562249	336.1605	10.56	POS	RP				2.08E-02	1.55E-02	-0.73	2.16E-01	0.38	1.51E-01	0.39	8.05E-01	-0.07
336.1758@10.776845	336.1758	10.78	POS	RP				1.10E-02	2.80E-01	-0.31	1.00E-02	0.77	7.98E-03	0.71	6.53E-01	0.14
336.2533@10.778776	336.2533	10.78	POS	RP				3.98E-01	3.65E-01	0.21	3.51E-02	0.58	4.63E-01	0.16	5.75E-01	0.13
336.2656@9.852684	336.2656	9.85	POS	RP				8.34E-03	7.34E-03	0.70	6.14E-01	-0.10	3.40E-01	-0.19	6.39E-02	0.43
336.2672@10.309284	336.2672	10.31	POS	RP				6.20E-01	5.87E-02	-0.44	7.47E-01	-0.09	6.47E-01	0.13	9.33E-01	0.02
337.1746@0.46180186	337.1746	0.46	POS	hilic				6.66E-01	1.03E-01	0.47	8.57E-01	0.05	2.61E-01	0.37	3.25E-01	0.34
337.1746@0.47389665	337.1746	0.47	POS	hilic				7.99E-01	2.65E-01	0.36	4.75E-01	0.22	9.12E-01	-0.03	3.92E-01	0.28
337.2105@6.1370354	337.2105	6.14	POS	RP				7.34E-01	8.29E-02	0.46	6.35E-01	0.13	2.22E-01	0.36	2.74E-01	0.33
337.2112@0.43671814	337.2112	0.44	POS	hilic				1.46E-01	2.05E-03	0.84	3.37E-01	0.29	6.54E-01	0.13	8.05E-01	-0.07
337.226@0.7612269	337.2260	0.76	POS	hilic				4.03E-01	3.38E-01	-0.30	1.97E-01	0.37	1.94E-01	0.33	9.95E-01	0.00
337.3344@10.294451	337.3344	10.29	POS	RP				6.18E-01	5.18E-02	-0.51	2.31E-01	-0.41	6.35E-01	-0.11	2.20E-01	-0.38
337.3348@10.623496	337.3348	10.62	POS	RP				5.44E-01	4.11E-01	-0.24	8.31E-01	0.06	1.60E-01	0.40	7.35E-01	-0.10
337.3348@3.284727	337.3348	3.28	POS	hilic				6.43E-01	9.69E-01	-0.01	8.55E-02	0.50	3.77E-01	0.26	5.87E-01	0.14
337.335@11.071354	337.3350	11.07	POS	RP				9.19E-01	7.52E-01	-0.09	6.68E-01	0.12	4.01E-01	0.22	6.39E-01	0.15
337.3351@3.1778803	337.3351	3.18	POS	hilic				6.50E-01	6.71E-01	0.14	1.04E-01	0.44	7.15E-01	-0.11	3.78E-01	0.24
337.3355@0.41648728	337.3355	0.42	POS	hilic				7.40E-01	4.02E-01	-0.28	9.41E-01	0.02	5.98E-01	0.17	3.72E-01	-0.27
337.8275@15.267043	337.8275	15.27	NEG	RP				8.81E-01	3.61E-01	0.26	7.77E-01	-0.07	5.57E-01	0.16	9.28E-01	0.02
337.8686@15.270019	337.8686	15.27	NEG	RP				8.23E-01	5.64E-01	0.18	9.47E-01	-0.02	2.48E-01	0.37	4.28E-01	0.18
337.8816@7.3003592	337.8816	7.30	POS	hilic				4.41E-01	9.52E-01	0.02	1.42E-01	0.42	8.10E-02	0.55	1.50E-01	0.42
338.031@5.408007	338.0310	5.41	POS	hilic				7.34E-01	1.77E-01	-0.44	3.19E-01	-0.27	1.80E-01	-0.43	5.02E-01	-0.17
338.0628@5.232337	338.0628	5.23	NEG	hilic				9.17E-01	4.83E-01	0.18	9.66E-01	0.01	7.20E-01	0.10	5.90E-01	-0.14
338.1513@9.788437	338.1513	9.79	POS	RP				7.43E-02	2.04E-01	-0.43	3.28E-02	0.61	2.29E-01	0.29	2.89E-01	0.32
338.1567@10.562156	338.1567	10.56	POS	RP				2.07E-02	1.76E-02	-0.72	1.74E-01	0.42	1.68E-01	0.37	8.28E-01	-0.07
338.1913@11.030038	338.1913	11.03	POS	RP				7.24E-01	3.15E-01	0.30	1.60E-01	0.45	1.45E-01	0.43	3.06E-01	0.32
338.2113@0.43049374	338.2113	0.43	NEG	hilic				3.79E-02	1.75E-02	-0.52	1.76E-01	0.33	2.22E-01	0.33	1.18E-01	0.45
338.2423@10.867736	338.2423	10.87	NEG	RP				5.95E-01	2.04E-01	-0.35	9.44E-01	-0.02	7.07E-01	0.09	1.09E-01	-0.40
338.2811@10.537986	338.2811	10.54	POS	RP				1.04E-01	6.46E-02	-0.37	5.56E-02	0.49	2.52E-01	0.31	9.99E-01	0.00
338.2819@14.622584	338.2819	14.62	POS	RP				5.18E-02	3.04E-01	0.27	5.15E-03	0.69	2.81E-03	0.93	5.91E-01	0.17
339.1629@10.424654	339.1629	10.42	POS	RP				1.61E-04	4.63E-02	-0.56	1.57E-03	0.88	2.81E-03	0.72	8.66E-01	-0.05
339.2412@7.067244	339.2412	7.07	POS	RP				9.59E-04	2.58E-03	-0.91	2.94E-01	0.28	2.91E-01	-0.26	4.62E-02	0.54
339.2425@0.6950002	339.2425	0.70	POS	hilic				8.35E-01	6.59E-01	0.11	6.58E-01	0.12	3.10E-01	-0.23	9.52E-01	-0.02
339.2506@0.69285226	339.2506	0.69	POS	hilic				4.66E-02	1.31E-01	-0.52	2.09E-02	0.61	8.74E-01	-0.04	4.59E-01	0.20
339.2522@4.0315	339.2522	4.03	POS	RP				8.18E-01	5.12E-01	0.17	7.81E-01	0.06	4.18E-01	-0.18	5.86E-01	0.14
339.3156@10.679354	339.3156	10.68	POS	RP				9.28E-01	7.31E-01	-0.09	8.09E-01	-0.07	4.20E-01	-0.21	7.63E-01	0.09
339.3505@11.391494	339.3505	11.39	POS	RP				9.01E-01	5.63E-01	-0.19	5.88E-01	0.17	6.77E-01	0.13	9.46E-01	-0.02
340.1895@8.394234	340.1895	8.39	POS	RP				3.56E-01	2.88E-01	-0.27	1.46E-01	-0.33	8.82E-02	-0.42	1.82E-02	-0.55
340.2957@10.7263365	340.2957	10.73	POS	RP				7.29E-01	5.23E-01	0.19	1.62E-01	0.43	6.14E-01	0.16	9.01E-01	-0.04

Supplementary table 2: Metabolomics analysis results

Compound	Mass	Retention Time	Ionization mode	column	Compound_ID	MSMS_spectra	ID_level	ANOVA	Anorexia vs control		overweight vs control		obese vs control		athletes vs control	
								P-FDR	P	Estimate	P	Estimate	P	Estimate	P	Estimate
340.299@10.725847	340.2990	10.73	POS	RP				9.75E-01	8.93E-01	-0.03	5.95E-01	0.15	8.63E-01	-0.05	9.53E-01	0.01
340.3339@11.9235525	340.3339	11.92	NEG	RP				1.08E-01	6.20E-01	-0.15	3.06E-01	0.30	2.20E-03	0.76	7.93E-01	-0.07
340.3441@10.761819	340.3441	10.76	POS	RP				2.16E-01	1.45E-02	-0.61	5.50E-02	-0.48	2.02E-01	-0.35	3.11E-02	-0.52
341.1775@10.702992	341.1775	10.70	POS	RP				7.93E-01	4.02E-01	0.24	3.23E-01	0.30	1.70E-01	0.42	2.45E-01	0.37
341.2569@7.5702405	341.2569	7.57	POS	RP				3.86E-03	5.79E-02	-0.61	9.17E-03	0.72	6.40E-01	0.11	8.10E-02	0.49
341.257@7.3855104	341.2570	7.39	POS	RP				7.48E-02	3.78E-01	-0.34	8.73E-03	0.71	2.59E-01	0.27	3.32E-01	0.27
341.2571@7.3849893	341.2571	7.38	POS	RP	Acylcarnitine 12:1	10 eV: 342.2639 (100), 85.0276 (15)		8.26E-02	4.05E-01	-0.32	8.06E-03	0.71	2.50E-01	0.28	4.22E-01	0.22
341.2572@0.6927524	341.2572	0.69	POS	hilic				5.98E-02	2.42E-01	-0.42	1.22E-02	0.69	4.99E-01	0.16	6.00E-01	0.15
341.2572@7.4988337	341.2572	7.50	POS	RP				5.20E-03	2.79E-02	-0.69	2.39E-02	0.63	8.50E-01	0.05	1.35E-01	0.42
341.7867@15.261696	341.7867	15.26	NEG	RP				7.18E-01	3.75E-01	-0.25	5.77E-01	-0.15	3.32E-01	0.28	8.33E-01	-0.06
341.8677@15.263167	341.8677	15.26	NEG	RP				6.03E-01	2.21E-01	-0.31	7.88E-01	0.08	1.97E-01	-0.32	3.25E-01	-0.25
342.0185@3.0838568	342.0185	3.08	NEG	RP				8.75E-03	1.22E-01	-0.46	2.79E-03	0.73	1.15E-01	0.49	9.73E-01	0.01
342.0192@3.3288379	342.0192	3.33	NEG	RP				2.02E-02	1.42E-01	-0.43	9.56E-03	0.71	1.54E-01	0.43	8.22E-01	-0.06
342.2788@10.7267475	342.2788	10.73	POS	RP				5.31E-01	7.22E-01	0.11	8.23E-01	-0.05	8.32E-01	-0.06	6.23E-02	-0.53
342.2874@0.8966825	342.2874	0.90	POS	hilic				9.14E-01	6.38E-01	0.12	7.37E-01	-0.09	6.08E-01	0.14	6.74E-01	-0.11
342.2881@8.6581335	342.2881	8.66	POS	RP				5.65E-01	3.17E-01	0.32	9.76E-01	0.01	4.81E-01	0.21	1.05E-01	0.55
343.0063@6.227981	343.0063	6.23	NEG	hilic				2.24E-01	3.66E-02	0.67	7.66E-02	0.52	1.86E-01	0.37	1.20E-02	0.77
343.037@1.8676053	343.0370	1.87	NEG	hilic				6.67E-01	9.42E-01	0.02	9.31E-02	-0.42	3.73E-01	-0.26	3.64E-01	-0.23
343.0373@2.6972566	343.0373	2.70	NEG	hilic				1.20E-01	2.83E-02	0.56	1.18E-01	-0.47	7.93E-01	0.09	7.41E-01	0.08
343.2727@0.681034	343.2727	0.68	POS	hilic				1.48E-01	5.80E-01	-0.22	7.16E-03	0.69	5.92E-01	0.13	1.91E-01	0.36
343.2728@7.805326	343.2728	7.81	POS	RP	Acylcarnitine 12:0	10 eV: 344.2790 (100), 85.0274 (50)	2	9.35E-02	7.27E-01	-0.14	3.78E-03	0.73	5.16E-01	0.15	4.32E-02	0.55
343.8258@15.2628765	343.8258	15.26	NEG	RP				2.38E-01	1.06E-03	-0.82	1.81E-01	-0.47	2.36E-01	-0.28	6.32E-02	-0.55
343.8681@15.2640085	343.8681	15.26	NEG	RP				6.20E-01	7.12E-02	-0.53	3.03E-01	-0.28	7.09E-01	-0.11	2.19E-01	-0.34
343.8684@7.873378	343.8684	7.87	POS	hilic				4.04E-01	1.30E-01	0.50	8.74E-01	0.04	1.27E-01	0.43	5.08E-02	0.51
344.0772@3.3301835	344.0772	3.33	NEG	RP				3.76E-03	1.07E-01	-0.35	5.62E-03	0.70	1.86E-01	0.38	1.10E-01	-0.30
344.2379@10.4845495	344.2379	10.48	POS	RP				5.59E-01	9.64E-01	0.01	2.02E-01	0.33	3.88E-01	0.18	8.09E-02	0.40
344.251@10.276965	344.2510	10.28	POS	RP				2.84E-02	1.27E-02	-0.70	1.44E-01	0.39	3.35E-01	0.24	3.43E-01	-0.31
344.2749@11.493174	344.2749	11.49	POS	RP				4.94E-01	7.91E-01	-0.07	2.12E-01	0.32	2.08E-01	-0.28	7.06E-01	-0.09
345.0015@6.224049	345.0015	6.22	NEG	hilic				1.39E-01	1.71E-02	0.75	8.52E-02	0.52	9.26E-02	0.48	4.08E-03	0.85
345.1735@10.312922	345.1735	10.31	POS	RP				1.28E-02	1.91E-01	-0.42	1.84E-02	0.70	1.02E-02	0.64	5.11E-01	0.18
345.2156@0.79448503	345.2156	0.79	POS	hilic				6.31E-01	2.15E-01	0.40	9.06E-01	0.03	9.16E-02	0.46	4.51E-01	0.23
345.2692@10.509112	345.2692	10.51	POS	RP				3.12E-02	5.59E-02	-0.55	5.69E-02	0.55	1.60E-01	0.38	7.91E-01	-0.09
345.2881@10.689951	345.2881	10.69	POS	RP				6.83E-01	2.47E-01	-0.37	5.69E-01	0.16	8.65E-01	0.05	5.54E-01	-0.19
345.2882@10.329438	345.2882	10.33	POS	RP				5.40E-01	4.22E-01	-0.24	3.70E-01	0.26	3.48E-01	0.25	4.88E-01	-0.23
345.2896@3.2825868	345.2896	3.28	POS	hilic				8.08E-02	8.69E-02	0.45	4.23E-02	-0.55	1.48E-01	-0.37	6.19E-01	-0.15
345.822@15.262954	345.8220	15.26	NEG	RP				3.25E-01	2.30E-03	-0.75	1.76E-01	-0.45	3.79E-01	-0.22	1.05E-01	-0.51
345.8596@15.265125	345.8596	15.27	NEG	RP				6.68E-01	1.24E-01	-0.41	3.25E-01	-0.32	9.58E-01	0.01	1.78E-01	-0.38
345.9867@6.478865	345.9867	6.48	NEG	RP				9.36E-01	9.99E-01	0.00	3.33E-01	-0.25	8.94E-01	0.04	7.92E-01	-0.09
346.0097@6.183897	346.0097	6.18	POS	hilic				8.28E-01	5.16E-01	0.21	7.59E-01	-0.09	4.79E-01	0.22	2.92E-01	0.29
346.0565@6.0249414	346.0565	6.02	POS	hilic				6.53E-01	2.47E-01	0.36	5.54E-01	0.19	5.04E-01	-0.17	2.57E-01	0.31
346.1636@6.832993	346.1636	6.83	POS	RP				2.05E-01	1.18E-02	0.78	7.91E-03	0.71	1.19E-01	0.46	1.55E-01	0.42
346.2146@7.2033305	346.2146	7.20	POS	RP				1.53E-01	6.63E-01	-0.12	7.85E-02	0.48	4.70E-01	-0.19	6.90E-02	0.52
346.2182@13.242969	346.2182	13.24	POS	RP				3.36E-01	7.93E-01	0.07	1.26E-01	0.42	1.69E-01	-0.39	9.29E-01	-0.03
346.2199@10.296893	346.2199	10.30	POS	RP				8.65E-01	7.30E-01	0.08	9.26E-01	0.03	4.19E-01	0.23	5.06E-01	-0.19
347.1077@10.318759	347.1077	10.32	NEG	RP				2.65E-02	7.45E-01	0.09	2.27E-03	0.98	1.30E-02	0.69	7.31E-02	0.54
347.1241@3.5792842	347.1241	3.58	NEG	hilic				3.53E-01	7.42E-01	0.10	5.50E-01	0.18	1.38E-02	0.70	7.56E-01	0.09
347.1836@0.41115004	347.1836	0.41	NEG	hilic				3.34E-01	2.14E-02	0.58	1.42E-01	0.34	2.74E-02	0.53	4.72E-01	0.19
347.2833@10.64479	347.2833	10.64	POS	RP				9.02E-02	3.90E-01	-0.25	1.99E-01	0.33	9.38E-02	0.49	2.00E-01	-0.38
347.2836@10.636907	347.2836	10.64	POS	RP				6.86E-03	4.85E-02	-0.53	3.56E-01	0.24	3.85E-02	0.60	9.99E-02	-0.44
347.3027@10.444552	347.3027	10.44	POS	RP				4.07E-01	1.70E-01	-0.33	9.87E-01	0.00	1.82E-01	0.40	6.73E-01	-0.12
348.0018@7.294421	348.0018	7.29	NEG	RP				9.42E-01	5.61E-01	-0.19	4.52E-01	-0.20	8.83E-01	-0.04	4.32E-01	-0.24
348.0098@7.4694076	348.0098	7.47	NEG	hilic				6.74E-01	1.46E-01	0.50	2.60E-01	0.31	1.93E-01	0.35	1.43E-01	0.41
348.0109@7.4394355	348.0109	7.44	POS	hilic				4.45E-01	2.24E-01	0.40	5.39E-01	-0.17	2.78E-01	0.33	1.50E-01	0.39
348.1072@9.798564	348.1072	9.80	NEG	RP				3.38E-01	6.23E-01	-0.11	1.37E-01	0.37	4.18E-01	-0.15	3.16E-01	0.25
348.2273@10.567826	348.2273	10.57	NEG	RP				1.07E-02	3.11E-03	-0.88	6.89E-01	0.12	2.21E-01	0.34	2.04E-01	-0.36
348.9839@0.6017671	348.9839	0.60	NEG	hilic				7.76E-01	6.31E-01	0.13	2.86E-01	-0.27	7.14E-01	0.10	8.19E-01	-0.06
349.0085@7.3602047	349.0085	7.36	POS	hilic				4.97E-01	9.03E-01	-0.04	8.40E-01	-0.06	1.17E-01	0.48	5.80E-01	-0.18
349.1053@3.9630375	349.1053	3.96	POS	hilic				2.43E-02	1.05E-01	0.56	9.66E-03	0.70	1.25E-01	-0.34	2.73E-01	0.29
349.1713@10.426002	349.1713	10.43	POS	RP				2.35E-04	2.62E-02	-0.63	4.14E-03	0.80	4.72E-03	0.69	7.24E-01	-0.10
349.1985@0.5381351	349.1985	0.54	POS	hilic				5.04E-01	4.27E-01	0.24	5.06E-01	-0.14	3.10E-01	0.27	1.30E-01	0.39
349.2993@10.800955	349.2993	10.80	POS	RP				3.68E-02	4.31E-01	-0.09	5.12E-02	0.44	7.31E-03	0.57	8.22E-01	0.04
349.3191@0.42294	349.3191	0.42	POS	hilic				8.95E-01	5.74E-01	-0.14	8.35E-01	0.06	6.41E-01	0.12	5.47E-01	-0.15
349.819@15.262239	349.8190	15.26	NEG	RP				3.17E-01	6.92E-03	-0.67	1.17E-01	-0.42	7.39E-01	-0.08	2.01E-01	-0.38
349.8369@7.8793635	349.8369	7.88	POS	hilic				9.51E-01	7.14E-01	0.11	9.86E-01	0.00	6.72E-01	-0.11	5.81E-01	0.16
350.02@2.4620783	350.0200	2.46	NEG	RP				6.25E-01	4.31E-01	-0.23	3.61E-01	0.30	2.72E-01	0.31	9.40E-01	0.02
350.0305@6.0858626	350.0305	6.09	NEG	hilic				6.03E-01	5.16E-02	0.53	2.44E-01	0.30	4.72E-01	0.17	6.04E-01	0.12
350.0944@0.8816279	350.0944	0.88	NEG	hilic				2.66E-01	9.98E-02	-0.47	7.02E-01	0.10	4.27E-01	0.23	1.32E-01	-0.34
350.2244@10.508508	350.2244	10.51	POS	RP				1.12E-01	1.92E-02	-0.46	2.35E-01	0.33	4.17E-01	0.19	4.29E-01	-0.18
350.2374@1.1085165	350.2374	1.11	POS	hilic				9.04E-01	7.38E-01	-0.10	2.38E-01	-0.29	5.17E-01	-0.16	5.98E-01	-0.14
350.2428@10.7828665	350.2428	10.78	NEG	RP				5.71E-03	7.00E-03	-0.81	3.73E-01	0.26	1.14E-01	0.44	1.50E-01	-0.40
350.2451@10.327907	350.2451	10.33	POS	RP				1.29E-01	5.11E-02	-0.52	4.91E-01	0.19	6.20E-01	0.15	7.09E-02	-0.47
350.2489@11.422104	350.2489	11.42	POS	RP				7.09E-01	7.20E-01	0.09	6.56E-01	0.10	2.22E-01	0.29	1.61E-01	0.42
350.2797@10.884828	350.2797	10.88	POS	RP				8.34E-01	4.42E-01	-0.17	4.20E-01	0.20	9.62E-01	-0.01	8.51E-01	-0.05

Supplementary table 2: Metabolomics analysis results

Compound	Mass	Retention Time	Ionization mode	column	Compound_ID	MSMS_spectra	ID_level	ANOVA	Anorexia vs control		overweight vs control		obese vs control		athletes vs control	
								P-FDR	P	Estimate	P	Estimate	P	Estimate	P	Estimate
350.8717@15.251321	350.8717	15.25	NEG	RP				7.64E-01	7.26E-01	-0.10	5.36E-01	-0.16	5.84E-01	0.17	3.16E-01	-0.29
351.1583@1.8148016	351.1583	1.81	POS	hilic				7.18E-01	3.68E-01	-0.29	3.76E-01	0.25	6.43E-01	-0.14	9.04E-01	-0.03
351.1589@4.2497053	351.1589	4.25	POS	RP				8.19E-02	3.54E-02	-0.62	1.39E-01	0.43	7.18E-01	-0.09	6.11E-01	0.14
351.1716@10.425722	351.1716	10.43	POS	RP				3.27E-04	2.93E-02	-0.60	5.08E-03	0.79	5.47E-03	0.67	6.38E-01	-0.13
351.1865@10.702309	351.1865	10.70	POS	RP				7.43E-01	6.70E-01	0.15	2.09E-01	0.39	2.77E-01	0.28	8.61E-01	-0.05
351.3135@0.42442593	351.3135	0.42	POS	hilic				7.27E-01	2.16E-01	-0.32	1.73E-01	-0.33	6.99E-01	-0.11	1.84E-01	-0.32
352.0714@6.025227	352.0714	6.03	POS	hilic				5.47E-01	9.54E-01	0.02	7.71E-01	-0.08	3.71E-02	-0.50	7.35E-01	0.10
352.1849@10.708419	352.1849	10.71	NEG	RP				2.97E-01	8.55E-02	-0.41	2.09E-01	-0.32	2.67E-02	-0.50	2.27E-02	-0.55
352.2586@11.036837	352.2586	11.04	NEG	RP				3.66E-01	2.39E-02	-0.59	4.14E-01	-0.30	5.76E-01	-0.13	2.44E-02	-0.63
353.1681@10.424401	353.1681	10.42	POS	RP				2.94E-02	1.55E-02	-0.31	2.73E-02	0.54	1.55E-01	0.31	7.67E-01	0.06
353.3291@0.42396727	353.3291	0.42	POS	hilic				6.90E-01	2.44E-01	-0.34	7.74E-01	0.08	4.10E-01	-0.25	2.87E-01	-0.32
353.3296@10.322839	353.3296	10.32	POS	RP				8.00E-01	2.74E-01	-0.31	3.86E-01	-0.24	4.26E-01	-0.23	1.67E-01	-0.38
353.33@10.421187	353.3300	10.42	POS	RP				6.07E-01	1.36E-01	-0.46	6.10E-01	0.15	7.82E-01	-0.08	5.68E-01	-0.17
353.3647@11.614763	353.3647	11.61	POS	RP				9.66E-01	3.97E-01	-0.21	7.51E-01	-0.09	9.38E-01	-0.02	6.61E-01	-0.12
354.0041@7.302124	354.0041	7.30	POS	hilic				3.11E-01	4.34E-01	-0.21	1.02E-01	-0.42	3.42E-01	0.30	3.49E-01	-0.26
354.0404@5.417417	354.0404	5.42	NEG	RP				4.04E-01	4.06E-01	0.14	6.54E-01	0.07	9.19E-01	-0.01	6.81E-02	0.40
354.1017@6.685859	354.1017	6.69	POS	hilic				7.57E-01	3.93E-01	-0.21	5.59E-01	0.15	3.83E-01	-0.19	7.70E-01	-0.07
354.1497@10.430554	354.1497	10.43	NEG	RP				1.11E-03	3.43E-02	-0.54	1.71E-02	0.64	6.26E-03	0.61	4.51E-01	-0.21
354.1716@10.707375	354.1716	10.71	NEG	RP				1.31E-01	9.89E-01	0.00	1.03E-01	0.48	3.00E-03	0.81	1.06E-01	0.49
354.1737@10.312512	354.1737	10.31	POS	RP				8.40E-04	2.63E-01	-0.33	3.04E-03	0.91	7.26E-04	0.88	5.34E-01	0.17
354.2449@11.029693	354.2449	11.03	POS	RP				7.35E-01	1.04E-01	0.41	4.19E-01	0.25	6.10E-01	0.17	7.76E-01	-0.08
354.256@8.975509	354.2560	8.98	POS	RP				1.67E-01	5.69E-01	-0.12	7.85E-01	-0.06	5.30E-01	0.14	4.74E-02	0.60
354.2565@8.268907	354.2565	8.27	POS	RP				3.54E-01	2.15E-01	0.32	2.35E-01	-0.23	2.94E-01	-0.22	9.23E-01	0.02
354.2774@10.3071165	354.2774	10.31	POS	RP				5.67E-02	7.23E-02	-0.40	4.01E-01	0.23	2.95E-02	0.63	6.37E-01	-0.14
354.3131@10.611206	354.3131	10.61	POS	RP				7.13E-01	9.64E-01	0.01	4.58E-01	0.16	1.00E-01	0.38	6.40E-01	0.12
355.2563@8.593706	355.2563	8.59	POS	RP				1.47E-02	5.53E-03	-0.50	5.15E-02	-0.40	4.44E-02	-0.41	2.50E-01	0.31
355.2726@7.7739205	355.2726	7.77	POS	RP				1.01E-02	2.47E-02	-0.68	2.25E-02	0.61	2.71E-01	0.28	4.25E-01	0.25
355.2729@0.6784324	355.2729	0.68	POS	hilic				5.85E-02	6.43E-02	-0.48	2.22E-02	0.61	5.04E-01	0.19	8.43E-01	-0.06
355.2732@9.382835	355.2732	9.38	POS	RP				9.46E-01	3.36E-01	-0.26	9.13E-01	0.03	6.43E-01	-0.13	8.60E-01	-0.06
355.3084@10.32797	355.3084	10.33	POS	RP				3.88E-01	2.45E-01	-0.26	3.14E-01	0.25	5.23E-01	-0.14	3.29E-01	-0.20
355.3437@10.298871	355.3437	10.30	POS	RP				2.65E-01	8.42E-03	-0.72	9.61E-01	-0.02	1.92E-01	-0.33	9.44E-02	-0.48
356.1652@10.706609	356.1652	10.71	NEG	RP				1.45E-01	8.88E-01	0.04	1.02E-02	0.78	3.04E-02	0.58	2.81E-01	0.33
356.2023@0.44673514	356.2023	0.45	POS	hilic				8.58E-01	7.24E-01	-0.10	2.06E-01	-0.32	9.70E-01	-0.01	7.09E-01	-0.11
356.2714@0.49534494	356.2714	0.50	POS	hilic				5.93E-01	8.90E-01	0.04	4.49E-01	0.17	8.27E-01	-0.06	1.33E-01	0.48
356.2717@8.9562845	356.2717	8.96	POS	RP				7.08E-01	6.76E-01	-0.10	2.29E-01	0.31	2.46E-01	0.30	5.43E-01	0.19
356.2718@9.518665	356.2718	9.52	POS	RP				5.70E-01	8.02E-01	0.08	9.10E-02	0.48	3.71E-01	0.25	1.50E-01	0.50
356.2718@9.571075	356.2718	9.57	POS	RP				6.88E-01	9.33E-01	-0.03	1.65E-01	0.38	2.49E-01	0.31	2.85E-01	0.38
356.272@8.501867	356.2720	8.50	POS	RP				2.37E-01	3.89E-01	0.20	1.23E-01	0.39	3.09E-02	0.58	8.66E-01	-0.04
356.2722@10.754447	356.2722	10.75	NEG	RP				2.74E-01	2.17E-02	-0.43	3.97E-01	0.19	4.01E-01	0.23	8.58E-01	0.04
356.2747@10.32449	356.2747	10.32	POS	RP				6.07E-01	4.62E-01	0.20	5.96E-01	-0.14	2.32E-01	-0.30	3.82E-01	-0.22
356.2934@10.527708	356.2934	10.53	POS	RP				7.48E-02	9.22E-01	-0.02	2.31E-02	0.62	1.28E-02	0.75	9.90E-01	0.00
356.2936@10.530397	356.2936	10.53	POS	RP				4.35E-01	3.96E-01	-0.21	3.30E-01	0.30	1.23E-01	0.45	8.92E-01	-0.04
357.1364@10.317854	357.1364	10.32	NEG	RP				6.64E-01	7.84E-01	0.09	9.25E-02	0.38	6.30E-01	-0.11	5.28E-01	0.18
357.2515@1.0184945	357.2515	1.02	POS	hilic				5.42E-02	5.40E-02	-0.56	1.15E-01	0.47	8.41E-01	0.05	2.78E-01	0.31
357.2515@6.625752	357.2515	6.63	POS	RP				2.20E-03	1.33E-03	-0.91	7.98E-01	0.07	2.81E-01	-0.26	7.44E-02	0.50
357.2518@0.56206673	357.2518	0.56	POS	hilic				9.77E-01	6.89E-01	-0.10	5.54E-01	-0.14	9.94E-01	0.00	9.82E-01	0.01
357.2524@0.8333435	357.2524	0.83	POS	hilic				5.99E-03	3.38E-03	-0.82	9.70E-01	0.01	1.11E-01	-0.41	1.05E-01	0.46
357.2713@8.869646	357.2713	8.87	POS	RP				6.53E-03	2.13E-03	-0.93	4.51E-01	0.17	2.15E-01	-0.36	2.57E-01	0.32
357.285@9.430078	357.2850	9.43	POS	RP				1.86E-01	9.81E-02	-0.40	7.06E-02	0.46	7.33E-01	-0.09	8.92E-01	-0.03
357.2879@0.66469127	357.2879	0.66	POS	hilic				4.27E-01	5.93E-02	-0.49	4.96E-01	0.19	8.26E-01	0.06	7.48E-01	-0.09
357.2884@8.062746	357.2884	8.06	POS	RP				3.64E-02	7.31E-03	-0.72	8.16E-02	0.46	6.40E-01	0.13	9.75E-01	-0.01
358.0381@4.820603	358.0381	4.82	NEG	hilic				2.78E-01	4.14E-02	0.53	2.91E-01	-0.29	6.86E-01	0.14	5.31E-01	0.17
358.062@1.5417095	358.0620	1.54	NEG	hilic				7.70E-01	9.38E-01	0.03	3.48E-01	0.24	7.15E-02	0.44	5.38E-01	0.19
358.0623@3.7544193	358.0623	3.75	NEG	hilic				6.00E-02	6.77E-01	-0.13	5.37E-01	0.17	7.76E-02	-0.51	8.00E-03	-0.78
358.0845@6.0242314	358.0845	6.02	POS	hilic				5.82E-02	1.17E-02	-0.70	7.16E-02	-0.53	1.83E-03	-0.83	3.59E-03	-0.93
358.1598@10.533739	358.1598	10.53	POS	RP				3.59E-01	1.71E-01	-0.26	1.34E-01	0.40	5.04E-01	0.16	5.65E-01	0.14
358.1628@10.319465	358.1628	10.32	NEG	RP				7.36E-01	9.96E-01	0.00	8.90E-01	0.03	1.50E-01	0.36	7.52E-01	-0.07
358.2476@0.43889472	358.2476	0.44	POS	hilic				8.98E-01	4.23E-01	-0.15	3.27E-01	-0.17	4.31E-01	-0.15	9.71E-01	-0.01
358.2556@10.7624855	358.2556	10.76	POS	RP				5.38E-01	7.39E-01	0.10	9.78E-02	0.45	5.17E-01	-0.21	5.33E-01	0.17
358.2663@10.277998	358.2663	10.28	POS	RP				4.81E-02	4.85E-03	-0.77	7.61E-01	0.08	3.78E-01	0.22	2.25E-01	-0.36
358.2873@10.915978	358.2873	10.92	NEG	RP				4.97E-01	3.44E-02	-0.46	7.96E-01	0.06	9.52E-01	0.02	8.29E-01	0.06
358.3079@3.2954464	358.3079	3.30	POS	hilic				8.00E-01	1.69E-01	-0.42	4.98E-01	-0.19	6.20E-01	-0.15	9.61E-01	-0.01
358.3088@10.774291	358.3088	10.77	POS	RP				2.87E-01	6.70E-01	-0.12	3.50E-01	-0.31	1.15E-01	0.47	5.82E-01	-0.16
359.1716@10.711588	359.1716	10.71	NEG	RP				9.79E-01	8.13E-01	0.07	9.66E-01	0.01	6.06E-01	-0.16	8.59E-01	-0.06
359.2677@7.2326035	359.2677	7.23	POS	RP				3.86E-02	1.27E-01	-0.35	2.05E-02	0.63	6.63E-01	0.11	1.82E-01	0.37
359.2678@0.9992916	359.2678	1.00	POS	hilic				2.97E-02	1.27E-01	-0.46	2.65E-02	0.61	7.85E-01	-0.07	1.66E-01	0.39
359.3064@10.723048	359.3064	10.72	POS	RP				7.22E-01	8.19E-01	0.06	2.40E-01	-0.33	5.68E-01	-0.15	7.62E-01	0.08
359.3163@5.814403	359.3163	5.81	POS	hilic				7.20E-01	5.58E-01	0.14	7.79E-01	0.07	9.88E-01	0.00	1.96E-01	-0.30
359.3169@11.061351	359.3169	11.06	POS	RP				4.54E-01	2.34E-01	-0.39	4.54E-01	0.23	3.99E-01	0.27	6.54E-01	-0.13
360.0124@5.4026413	360.0124	5.40	POS	hilic				7.82E-01	2.58E-01	-0.36	3.49E-01	-0.25	2.54E-01	-0.36	8.91E-01	-0.04
360.0775@1.6525128	360.0775	1.65	NEG	hilic				3.15E-01	6.85E-01	0.14	3.32E-01	0.27	1.02E-01	-0.48	3.75E-01	-0.23
360.1607@10.535293	360.1607	10.54	POS	RP				3.93E-04	2.00E-02	-0.63	3.43E-03	0.78	1.38E-02	0.64	7.71E-01	-0.09
360.1794@13.81722	360.1794	13.82	POS	RP				6.30E-01	9.47E-01	-0.02	7.29E-01	0.10	1.42E-01	-0.40	2.85E-01	-0.34

Supplementary table 2: Metabolomics analysis results

								ANOVA	Anorexia vs control		overweight vs control		obese vs control		athletes vs control	
Compound	Mass	Retention Time	Ionization mode	column	Compound_ID	MSMS_spectra	ID_level	P-FDR	P	Estimate	P	Estimate	P	Estimate	P	Estimate
360.1932@6.39558	360.1932	6.40	NEG	RP				6.68E-01	1.36E-01	-0.40	7.34E-01	-0.08	2.99E-01	-0.28	8.44E-01	0.06
360.1941@6.384384	360.1941	6.38	POS	RP	Cortisone	10 eV: 361.2017 (100), 163.1093 (15)	2	4.68E-01	6.72E-01	-0.13	9.48E-01	-0.02	1.63E-01	-0.39	2.88E-01	0.34
360.2321@10.632709	360.2321	10.63	POS	RP				7.30E-01	5.10E-01	-0.19	5.50E-01	0.19	7.16E-01	0.10	3.31E-01	-0.28
361.1371@5.11267	361.1371	5.11	NEG	hilic				6.97E-03	4.27E-02	-0.59	1.56E-02	0.64	2.81E-02	0.52	9.71E-01	-0.01
361.2656@10.484222	361.2656	10.48	POS	RP				7.40E-02	6.90E-01	-0.11	6.29E-03	0.85	3.81E-01	0.20	2.77E-01	0.33
361.2965@10.680915	361.2965	10.68	POS	RP				8.75E-01	2.63E-01	0.27	7.58E-01	-0.08	9.65E-01	0.01	8.37E-01	0.06
361.9196@4.3505974	361.9196	4.35	POS	hilic				5.06E-01	8.40E-02	0.55	2.86E-01	0.31	8.90E-01	-0.04	9.87E-01	0.01
362.1774@10.685377	362.1774	10.69	POS	RP				1.17E-01	8.87E-02	-0.30	4.77E-01	0.16	1.03E-01	0.41	2.41E-01	-0.25
362.21@6.620646	362.2100	6.62	POS	RP	Cortisol	10 eV: 363.2162 (100), 121.0650 (15), 327.1975 (10), 157.1032 (8)	2	2.04E-01	5.03E-01	0.20	9.35E-02	0.46	5.15E-01	-0.20	2.97E-02	0.60
362.2102@0.5084137	362.2102	0.51	POS	hilic				2.29E-01	6.73E-01	0.12	7.32E-02	0.53	6.06E-01	-0.16	4.46E-02	0.54
362.2108@9.756028	362.2108	9.76	NEG	RP				6.82E-01	2.46E-01	-0.36	4.63E-01	0.24	8.77E-01	0.05	9.79E-01	-0.01
362.2133@9.749478	362.2133	9.75	POS	RP				3.11E-01	3.59E-01	0.29	5.59E-01	0.18	2.61E-02	0.75	9.99E-02	0.46
362.2748@10.726282	362.2748	10.73	POS	RP				7.94E-01	7.39E-01	-0.10	2.86E-01	0.32	5.97E-01	0.14	8.55E-01	-0.06
362.3033@0.4273418	362.3033	0.43	POS	hilic				8.75E-01	2.18E-01	-0.32	7.37E-01	-0.09	8.51E-01	-0.06	3.31E-01	-0.25
363.1897@6.156989	363.1897	6.16	POS	RP				8.29E-02	1.64E-03	0.99	4.36E-02	0.53	1.29E-01	0.43	3.61E-01	0.25
363.1904@0.42770064	363.1904	0.43	POS	hilic				6.77E-02	1.56E-03	0.96	6.19E-01	0.14	1.50E-01	0.43	8.68E-01	0.04
363.1906@2.4998024	363.1906	2.50	POS	hilic				9.53E-01	8.14E-01	0.06	8.32E-01	0.06	7.55E-01	0.10	3.80E-01	0.27
363.2457@11.028824	363.2457	11.03	POS	RP				8.42E-01	2.93E-01	0.25	2.36E-01	0.38	6.76E-01	0.14	8.21E-01	0.07
364.1819@10.312922	364.1819	10.31	POS	RP				6.68E-04	6.23E-02	-0.50	2.89E-03	0.84	1.72E-03	0.73	9.40E-01	-0.02
364.2613@10.726758	364.2613	10.73	POS	RP				7.64E-01	5.59E-01	-0.20	1.35E-01	-0.39	3.04E-01	-0.29	1.75E-01	-0.43
364.2913@10.778	364.2913	10.78	POS	RP				8.20E-01	8.15E-01	-0.06	3.75E-01	0.22	3.88E-01	0.23	8.59E-01	-0.05
365.0187@1.8270313	365.0187	1.83	NEG	hilic				5.08E-01	3.51E-01	0.25	3.23E-01	-0.20	4.78E-01	0.21	1.82E-01	0.32
365.179@10.562676	365.1790	10.56	POS	RP				1.49E-02	1.70E-02	-0.72	1.72E-01	0.43	1.32E-01	0.41	7.76E-01	-0.09
365.2014@10.861785	365.2014	10.86	POS	RP				3.86E-01	1.70E-02	0.58	7.99E-01	0.06	1.88E-01	0.38	5.27E-01	0.14
365.2335@2.989445	365.2335	2.99	POS	hilic				6.85E-01	7.07E-01	-0.11	6.52E-01	0.13	8.33E-01	-0.06	1.29E-01	-0.41
365.2563@0.6877172	365.2563	0.69	POS	hilic				3.46E-01	2.66E-01	-0.38	1.62E-01	0.38	7.98E-01	-0.07	4.88E-01	0.20
365.2572@7.36716	365.2572	7.37	POS	RP				6.40E-01	7.28E-01	0.10	9.06E-02	0.44	6.56E-01	0.11	2.15E-01	0.37
365.3663@0.41639695	365.3663	0.42	POS	hilic				3.05E-01	2.00E-01	-0.34	9.25E-01	0.02	9.98E-01	0.00	3.00E-02	-0.54
365.3664@11.448628	365.3664	11.45	POS	RP				6.62E-01	2.72E-01	-0.36	6.11E-01	0.15	7.54E-01	0.11	4.69E-01	-0.22
366.1833@10.312359	366.1833	10.31	POS	RP				1.06E-03	2.54E-02	-0.61	1.65E-02	0.71	4.94E-03	0.69	9.63E-01	0.01
366.3126@11.028995	366.3126	11.03	POS	RP				8.59E-01	4.05E-01	0.22	2.37E-01	0.37	3.09E-01	0.27	3.87E-01	0.26
367.1949@10.776922	367.1949	10.78	POS	RP				3.11E-02	3.41E-01	-0.29	2.94E-02	0.67	1.26E-02	0.68	6.38E-01	0.14
367.2723@8.031188	367.2723	8.03	POS	RP				2.14E-01	1.06E-01	-0.43	2.21E-01	0.35	3.75E-01	-0.22	6.74E-01	0.11
367.2725@7.7704635	367.2725	7.77	POS	RP	Acylcarnitine 14:2	10 eV: 368.2798 (100), 85.0271 (18)	2	1.78E-01	3.21E-01	-0.37	1.23E-01	0.43	9.35E-01	-0.02	1.13E-01	0.46
367.2729@0.6708447	367.2729	0.67	POS	hilic				2.08E-01	2.08E-01	-0.47	1.43E-01	0.41	8.13E-01	-0.06	3.36E-01	0.26
367.2729@7.87373	367.2729	7.87	POS	RP				2.28E-01	8.19E-01	-0.08	1.13E-01	0.47	8.73E-01	-0.04	4.11E-02	0.60
367.3816@11.862906	367.3816	11.86	POS	RP				8.47E-01	6.43E-01	-0.15	3.27E-01	0.29	8.41E-01	0.07	8.20E-01	0.07
367.9913@6.2287602	367.9913	6.23	NEG	hilic				3.67E-01	3.07E-02	0.72	3.32E-01	0.28	1.27E-01	0.42	5.78E-02	0.54
368.1126@5.295709	368.1126	5.30	NEG	hilic				9.78E-02	4.66E-01	-0.14	6.14E-02	0.53	8.89E-01	0.03	1.98E-01	-0.25
368.1126@5.3010435	368.1126	5.30	NEG	hilic				5.97E-04	7.18E-04	-0.87	3.73E-03	0.69	3.78E-01	0.23	4.99E-01	-0.19
368.1362@1.4574374	368.1362	1.46	NEG	hilic				2.22E-01	1.61E-01	0.35	5.83E-01	-0.06	7.58E-01	0.05	1.61E-01	-0.13
368.137@6.59775	368.1370	6.60	NEG	RP				3.96E-01	1.45E-01	0.38	7.21E-01	-0.05	9.96E-01	0.00	8.25E-01	-0.03
368.1376@1.3547499	368.1376	1.35	POS	hilic				9.34E-01	8.26E-01	0.05	4.80E-01	-0.13	7.09E-01	0.09	9.60E-01	-0.01
368.1378@6.5872197	368.1378	6.59	POS	RP				9.29E-01	5.45E-01	0.15	8.23E-01	-0.05	5.59E-01	0.14	9.65E-01	-0.01
368.1651@8.025178	368.1651	8.03	NEG	RP				1.38E-01	3.17E-02	-0.47	1.00E-01	-0.42	2.50E-02	-0.52	7.58E-01	0.08
368.1654@0.5493331	368.1654	0.55	NEG	hilic				3.62E-01	2.40E-01	-0.36	1.56E-01	0.38	3.43E-01	0.30	9.55E-01	0.02
368.1659@7.585913	368.1659	7.59	NEG	RP				1.37E-01	8.23E-01	-0.06	8.90E-02	-0.38	5.03E-02	-0.42	3.68E-01	0.24
368.166@7.189057	368.1660	7.19	NEG	RP	Steroid sulfate 1	10 eV: 367.1580 (100), 96.9597 (10)	3	2.66E-01	1.93E-01	-0.41	1.53E-01	0.37	4.35E-01	0.25	4.79E-01	-0.18
368.184@10.312077	368.1840	10.31	POS	RP				1.88E-02	1.75E-01	-0.37	1.28E-02	0.69	2.64E-02	0.57	8.48E-01	0.06
368.1908@10.496449	368.1908	10.50	POS	RP				3.99E-01	7.38E-01	-0.09	7.79E-02	0.54	9.52E-01	0.02	2.95E-01	0.27
368.2458@11.0367365	368.2458	11.04	NEG	RP				4.57E-01	5.32E-01	-0.18	6.19E-01	0.16	5.48E-02	0.48	6.93E-01	-0.11
368.3433@11.24437	368.3433	11.24	POS	RP				6.24E-01	6.46E-02	0.52	8.65E-01	0.05	6.23E-01	0.14	1.74E-01	0.38
368.3447@12.369326	368.3447	12.37	POS	RP	Cholesterol	10 eV: 369.3495 (100), 81.0696 (20), 161.1344 (10), 147.1159 (9), 257.2249 (8)	2	9.11E-01	9.03E-01	-0.03	3.28E-01	0.24	8.87E-01	0.04	7.40E-01	-0.11
368.3448@0.40759772	368.3448	0.41	POS	hilic				2.88E-01	6.58E-02	0.57	7.19E-01	-0.10	4.10E-01	-0.22	9.04E-01	-0.04
368.3649@12.575676	368.3649	12.58	NEG	RP				5.47E-01	6.45E-01	-0.15	9.38E-01	-0.02	4.43E-02	0.50	7.49E-01	0.10
368.3769@11.073618	368.3769	11.07	POS	RP				7.90E-01	3.79E-01	-0.29	6.64E-01	0.13	7.15E-01	0.12	6.19E-01	-0.15
369.1159@5.298404	369.1159	5.30	NEG	hilic				1.36E-02	2.40E-03	-0.77	6.11E-02	0.49	5.18E-01	0.19	7.77E-01	-0.08
369.2006@0.5175061	369.2006	0.52	POS	hilic				9.93E-01	6.75E-01	0.10	8.57E-01	0.05	8.45E-01	-0.06	8.28E-01	0.06
369.21@11.02992	369.2100	11.03	POS	RP				8.50E-01	3.16E-01	0.30	3.57E-01	0.30	2.86E-01	0.36	6.64E-01	0.14
369.2422@0.97069997	369.2422	0.97	POS	hilic				7.40E-01	6.81E-01	0.11	7.78E-01	0.07	4.07E-01	-0.16	2.15E-01	-0.24
369.2882@8.152095	369.2882	8.15	POS	RP				1.42E-01	2.92E-01	-0.35	3.39E-02	0.64	6.92E-01	0.10	7.77E-01	0.08
369.2882@8.297218	369.2882	8.30	POS	RP				3.10E-01	8.51E-01	-0.07	3.37E-02	0.60	8.90E-01	0.04	1.77E-01	0.40
369.2884@8.156967	369.2884	8.16	POS	RP	Acylcarnitine 14:1	10 eV: 370.2957 (100), 85.0282 (25)	2	2.10E-01	7.79E-01	-0.11	7.71E-03	0.71	3.11E-01	0.25	2.11E-01	0.35
369.2887@0.6655858	369.2887	0.67	POS	hilic				1.89E-01	5.57E-01	-0.23	1.20E-02	0.67	3.83E-01	0.22	5.01E-01	0.18
370.0947@10.177166	370.0947	10.18	POS	RP				2.91E-01	3.38E-03	-0.75	7.06E-01	-0.11	9.98E-01	0.00	5.46E-01	-0.18
370.1175@5.2878976	370.1175	5.29	NEG	hilic				1.05E-02	4.48E-03	-0.72	3.16E-02	0.55	6.21E-01	0.13	3.63E-01	-0.25
370.1786@7.093647	370.1786	7.09	NEG	RP				5.36E-01	1.84E-01	-0.30	6.33E-01	0.12	4.36E-01	0.23	4.03E-01	-0.20
370.1797@0.5595001	370.1797	0.56	NEG	hilic				5.99E-01	1.00E-01	-0.47	8.29E-01	-0.06	9.59E-01	0.02	5.62E-01	0.17
370.1799@0.5598818	370.1799	0.56	NEG	hilic				5.31E-01	6.66E-02	-0.51	5.55E-01	-0.16	8.49E-01	-0.06	5.39E-01	0.17
370.1808@10.860653	370.1808	10.86	NEG	RP				8.62E-01	7.65E-01	0.08	7.92E-01	-0.08	3.10E-01	0.25	3.94E-01	0.22
370.181@6.7367606	370.1810	6.74	NEG	RP				2.13E-02	2.79E-01	-0.32	2.04E-01	0.33	4.02E-02	0.65	8.29E-02	-0.42
370.181@8.532862	370.1810	8.53	NEG	RP				4.68E-01	1.90E-01	-0.37	2.74E-01	0.29	3.99E-01	0.27	9.55E-01	-0.02
370.1811@7.437743	370.1811	7.44	NEG	RP	Steroid sulfate 2	10 eV: 369.1740 (100), 96.9590 (5)	3	7.30E-01	8.51E-02	-0.50	7.23E-01	-0.09	8.10E-01	-0.08	8.79E-01	-0.05

Supplementary table 2: Metabolomics analysis results

								ANOVA	Anorexia vs control		overweight vs control		obese vs control		athletes vs control	
Compound	Mass	Retention Time	Ionization mode	column	Compound_ID	MSMS_spectra	ID_level	P-FDR	P	Estimate	P	Estimate	P	Estimate	P	Estimate
370.1811@6.7368364	370.1811	6.74	NEG	RP				2.48E-02	1.05E-01	-0.46	4.91E-01	0.19	5.78E-02	0.61	7.16E-02	-0.41
370.1813@7.8936067	370.1813	7.89	NEG	RP				5.42E-01	8.52E-01	-0.05	8.31E-01	0.05	1.32E-01	0.48	2.59E-01	0.38
370.1814@7.9363613	370.1814	7.94	NEG	RP				2.08E-01	5.43E-01	0.17	3.60E-01	0.26	3.33E-02	0.68	3.01E-02	0.72
370.1817@10.866513	370.1817	10.87	NEG	RP				7.93E-01	5.28E-01	0.16	9.37E-01	0.02	1.36E-01	0.39	6.65E-01	0.10
370.2154@10.709983	370.2154	10.71	NEG	RP				1.82E-01	3.80E-01	0.19	1.16E-01	0.42	7.92E-01	-0.06	1.30E-02	0.61
370.3078@10.780159	370.3078	10.78	POS	RP				6.86E-01	4.88E-01	-0.19	3.38E-01	0.23	3.62E-01	0.24	4.21E-01	0.21
370.3088@10.942528	370.3088	10.94	POS	RP				8.93E-01	3.12E-01	0.27	6.17E-01	0.16	9.89E-01	0.00	2.65E-01	0.28
370.3089@10.728195	370.3089	10.73	POS	RP				3.07E-01	7.81E-01	0.10	2.12E-02	0.70	8.40E-01	0.06	4.21E-01	0.21
371.1512@10.524907	371.1512	10.52	NEG	RP				6.14E-01	4.71E-02	-0.52	7.76E-01	0.08	8.31E-01	-0.06	8.33E-01	-0.07
371.1514@10.525376	371.1514	10.53	NEG	RP				2.24E-01	2.49E-02	0.70	8.25E-01	-0.06	9.42E-01	-0.02	7.29E-01	-0.08
371.1854@10.866957	371.1854	10.87	NEG	RP				8.38E-01	9.77E-01	-0.01	5.46E-01	0.17	1.86E-01	0.33	4.51E-01	0.19
371.2188@0.58386946	371.2188	0.58	POS	hilic				8.62E-01	3.73E-01	-0.22	3.18E-01	-0.24	9.67E-01	-0.01	4.20E-01	-0.20
371.2669@9.320468	371.2669	9.32	POS	RP				7.10E-01	3.74E-01	-0.23	7.16E-01	-0.09	5.75E-01	0.14	4.42E-01	0.22
371.3028@9.570887	371.3028	9.57	POS	RP				1.38E-01	2.70E-02	-0.56	1.56E-01	0.38	9.25E-01	0.02	4.78E-01	-0.20
371.3036@8.481426	371.3036	8.48	POS	RP				8.92E-02	9.63E-01	0.02	8.24E-04	0.86	3.54E-02	0.50	1.14E-01	0.45
371.3039@10.306377	371.3039	10.31	POS	RP				1.68E-01	3.92E-01	-0.20	3.69E-01	0.26	2.25E-02	0.68	4.27E-01	0.22
371.3041@0.66186184	371.3041	0.66	POS	hilic				1.64E-01	8.14E-01	-0.09	4.52E-03	0.75	1.37E-01	0.37	5.74E-01	0.15
372.0777@1.2300516	372.0777	1.23	NEG	hilic				8.57E-02	5.28E-01	-0.19	3.63E-01	-0.27	8.86E-04	-0.83	5.93E-01	0.17
372.1119@4.1141195	372.1119	4.11	NEG	hilic				9.06E-01	6.30E-01	-0.14	8.66E-01	0.05	5.73E-01	0.18	6.06E-01	-0.14
372.1124@3.5131288	372.1124	3.51	NEG	hilic				4.92E-01	4.20E-01	0.26	1.59E-01	0.37	4.55E-02	0.64	9.24E-02	0.40
372.1966@7.619105	372.1966	7.62	NEG	RP				4.98E-01	8.07E-02	-0.44	4.72E-01	-0.20	4.57E-01	0.21	7.11E-01	-0.09
372.2267@10.542151	372.2267	10.54	NEG	RP				1.12E-03	6.26E-03	-0.69	5.35E-02	0.51	4.33E-02	0.52	2.61E-01	-0.32
372.2671@8.973268	372.2671	8.97	POS	RP				1.94E-01	7.02E-01	-0.08	6.07E-01	-0.12	8.58E-01	-0.05	5.58E-02	0.60
373.0869@9.433814	373.0869	9.43	NEG	RP				9.59E-01	4.42E-01	-0.23	9.16E-01	0.03	6.70E-01	-0.11	7.89E-01	-0.09
373.1245@10.430966	373.1245	10.43	NEG	RP				1.33E-03	7.25E-02	-0.46	9.10E-03	0.74	5.84E-03	0.68	8.38E-01	-0.06
373.2046@10.70283	373.2046	10.70	POS	RP				3.96E-01	6.98E-02	0.59	1.39E-01	0.47	4.64E-02	0.64	3.41E-01	0.28
373.3191@10.539161	373.3191	10.54	POS	RP				7.37E-02	4.92E-01	0.15	2.30E-02	0.67	4.61E-03	0.87	3.52E-01	0.27
373.8018@3.0693681	373.8018	3.07	POS	hilic				9.47E-01	6.64E-01	0.13	6.90E-01	-0.11	8.60E-01	0.05	6.23E-01	-0.15
373.9039@15.25319	373.9039	15.25	NEG	RP				9.31E-01	8.55E-01	0.04	6.42E-01	0.10	9.69E-01	-0.01	4.66E-01	-0.12
373.9085@7.925502	373.9085	7.93	NEG	hilic				5.08E-01	2.65E-01	0.42	5.51E-01	0.15	3.27E-02	0.57	7.49E-01	0.09
374.0915@3.9641027	374.0915	3.96	POS	hilic				8.48E-01	7.03E-01	0.12	8.11E-01	-0.07	1.00E+00	0.00	3.09E-01	0.33
374.1391@10.319055	374.1391	10.32	NEG	RP				3.11E-02	3.97E-01	-0.19	1.41E-01	0.45	4.55E-01	0.19	1.63E-02	-0.60
374.2092@10.706554	374.2092	10.71	POS	RP				2.16E-01	3.24E-01	-0.32	8.54E-01	-0.05	1.12E-01	0.42	1.31E-01	-0.39
374.2823@9.569856	374.2823	9.57	POS	RP				3.11E-01	6.96E-01	-0.09	5.68E-01	-0.12	5.85E-01	0.12	8.92E-02	0.50
375.1391@10.715907	375.1391	10.72	NEG	RP				2.38E-01	7.53E-03	0.81	1.30E-01	0.48	1.73E-01	0.36	4.80E-02	0.60
375.2902@11.061693	375.2902	11.06	POS	RP				6.89E-01	2.86E-01	-0.37	5.67E-01	0.17	7.65E-01	0.09	6.57E-01	0.13
375.9756@7.5415173	375.9756	7.54	NEG	RP				7.00E-01	3.78E-01	-0.25	3.59E-01	0.27	5.29E-01	0.17	6.42E-01	0.14
376.1399@10.318633	376.1399	10.32	NEG	RP				8.21E-03	3.07E-01	-0.28	2.53E-02	0.71	1.61E-02	0.62	3.54E-01	-0.22
376.2593@10.307699	376.2593	10.31	POS	RP				4.72E-02	7.94E-02	-0.40	2.56E-01	0.31	2.35E-02	0.65	6.70E-01	-0.13
376.2595@0.42023718	376.2595	0.42	POS	hilic				2.15E-01	3.49E-02	-0.71	6.60E-01	0.12	4.03E-01	-0.22	3.05E-01	-0.25
376.3193@0.42679033	376.3193	0.43	POS	hilic				7.81E-01	3.12E-01	0.24	3.60E-01	-0.20	9.12E-01	-0.03	8.46E-01	0.05
377.2024@10.776213	377.2024	10.78	POS	RP				1.93E-01	5.84E-01	-0.13	1.57E-01	0.46	1.73E-02	0.62	7.94E-01	0.07
377.2616@10.632451	377.2616	10.63	POS	RP				7.05E-01	7.66E-01	-0.07	6.78E-01	0.12	3.79E-01	-0.27	2.02E-01	-0.36
377.9917@6.978595	377.9917	6.98	NEG	RP				8.37E-01	3.63E-01	-0.21	1.92E-01	-0.35	9.24E-01	0.03	6.15E-01	-0.18
378.1031@2.0407298	378.1031	2.04	NEG	hilic				2.72E-02	1.13E-02	0.84	8.95E-01	0.03	9.52E-01	0.01	2.37E-02	0.61
378.1395@10.327021	378.1395	10.33	NEG	RP				6.36E-01	9.90E-02	-0.48	9.03E-01	-0.04	7.80E-01	-0.08	7.51E-01	0.08
378.1396@10.711652	378.1396	10.71	NEG	RP				1.19E-01	2.90E-02	0.59	6.81E-02	0.61	1.56E-01	0.36	2.32E-03	0.90
378.1981@10.5079975	378.1981	10.51	POS	RP				6.69E-01	6.74E-01	-0.09	2.03E-01	0.39	8.65E-01	0.04	7.52E-01	0.07
378.2753@10.55046	378.2753	10.55	POS	RP				3.13E-02	3.72E-01	-0.22	4.00E-02	0.59	9.39E-03	0.74	9.53E-01	-0.02
379.1766@0.86827993	379.1766	0.87	POS	hilic				3.42E-01	8.27E-02	0.43	7.75E-01	-0.03	2.55E-01	0.19	3.63E-01	0.16
379.2029@10.776324	379.2029	10.78	POS	RP				1.39E-02	2.29E-01	-0.35	1.62E-02	0.73	1.53E-02	0.66	7.38E-01	0.10
379.2178@11.029952	379.2178	11.03	POS	RP				3.36E-01	1.16E-01	0.48	5.45E-02	0.57	2.52E-02	0.68	7.52E-02	0.56
379.2706@10.37553	379.2706	10.38	POS	RP				5.38E-01	3.17E-01	-0.23	7.44E-02	-0.43	7.36E-02	-0.41	5.90E-01	-0.14
379.2707@10.345206	379.2707	10.35	POS	RP				6.44E-01	3.33E-02	-0.42	3.83E-01	-0.20	6.55E-01	-0.10	7.80E-01	-0.07
379.2939@11.029589	379.2939	11.03	POS	RP				7.38E-01	1.93E-01	0.38	2.19E-01	0.38	2.11E-01	0.35	7.84E-01	0.09
379.3297@0.4282271	379.3297	0.43	POS	hilic				7.26E-01	1.75E-01	0.43	8.30E-01	-0.06	8.34E-01	-0.06	7.67E-01	0.09
379.3305@10.174636	379.3305	10.17	POS	RP				9.40E-01	4.76E-01	0.21	4.17E-01	0.22	3.58E-01	0.27	7.73E-01	0.08
380.0206@4.6951613	380.0206	4.70	POS	hilic				7.36E-01	6.66E-01	0.12	3.56E-01	-0.27	5.72E-01	0.20	5.49E-01	0.15
380.0982@1.0199825	380.0982	1.02	NEG	hilic				7.03E-01	3.23E-01	-0.26	4.65E-01	-0.17	1.21E-01	-0.37	9.31E-01	0.02
380.1654@10.567665	380.1654	10.57	NEG	RP				1.64E-01	7.97E-02	-0.53	3.08E-01	0.33	1.73E-01	0.37	8.34E-01	0.06
380.1858@10.782589	380.1858	10.78	NEG	RP				4.56E-02	1.80E-01	-0.34	3.32E-02	0.61	7.99E-02	0.47	9.60E-01	0.01
380.1981@10.512837	380.1981	10.51	POS	RP				1.92E-03	3.35E-01	-0.21	1.47E-04	0.95	2.67E-02	0.52	4.07E-01	0.21
380.2338@0.4219231	380.2338	0.42	POS	hilic				1.08E-01	1.53E-01	0.36	2.69E-03	0.64	3.28E-01	0.15	4.69E-01	0.11
380.2684@11.037838	380.2684	11.04	NEG	RP				2.78E-01	5.76E-01	-0.14	6.46E-01	0.11	6.27E-02	0.53	1.65E-01	0.40
380.2895@11.429011	380.2895	11.43	NEG	RP				2.31E-01	3.80E-02	-0.55	8.10E-01	-0.07	2.51E-01	0.25	1.97E-01	-0.32
380.2913@10.775982	380.2913	10.78	POS	RP				3.24E-02	1.43E-02	-0.79	9.50E-01	0.02	6.06E-01	0.15	5.08E-02	-0.64
380.3761@10.858366	380.3761	10.86	POS	RP				7.84E-01	2.12E-01	-0.32	6.90E-01	-0.10	4.86E-01	-0.20	1.90E-01	-0.34
380.9787@6.207477	380.9787	6.21	POS	hilic				8.47E-01	7.25E-01	0.12	8.90E-01	0.04	5.10E-01	0.18	1.54E-01	0.39
381.3722@3.35812	381.3722	3.36	POS	hilic				5.16E-02	9.62E-01	-0.02	5.50E-01	0.15	5.26E-01	0.13	3.05E-03	-0.82
381.8502@3.1958985	381.8502	3.20	NEG	hilic				5.31E-01	1.49E-01	0.47	9.98E-01	0.00	2.61E-01	0.32	1.19E-01	0.47
381.8503@3.1978567	381.8503	3.20	NEG	hilic				6.20E-01	3.26E-01	0.32	6.86E-01	-0.12	5.93E-01	0.15	1.29E-01	0.42
381.8507@3.1958985	381.8507	3.20	NEG	hilic				5.55E-01	1.66E-01	0.45	8.73E-01	-0.05	3.24E-01	0.28	1.56E-01	0.43
381.9837@7.850308	381.9837	7.85	NEG	RP				4.92E-01	5.71E-01	-0.16	4.36E-01	0.20	8.74E-02	-0.49	7.00E-01	-0.13

Supplementary table 2: Metabolomics analysis results

								ANOVA	Anorexia vs control		overweight vs control		obese vs control		athletes vs control	
Compound	Mass	Retention Time	Ionization mode	column	Compound_ID	MSMS_spectra	ID_level	P-FDR	P	Estimate	P	Estimate	P	Estimate	P	Estimate
381.9837@8.20162	381.9837	8.20	NEG	RP				9.48E-01	6.91E-01	-0.13	9.27E-01	-0.02	9.62E-01	0.01	5.53E-01	0.19
382.1071@6.0783577	382.1071	6.08	NEG	hilic				6.94E-01	8.67E-01	-0.05	7.75E-01	-0.09	5.49E-02	-0.46	2.14E-01	-0.36
382.1094@5.088783	382.1094	5.09	POS	hilic				1.01E-02	4.68E-03	-0.77	1.77E-02	0.56	5.76E-01	-0.15	6.23E-01	-0.14
382.1608@10.509623	382.1608	10.51	POS	RP				1.41E-02	1.04E-02	-0.67	1.16E-01	0.44	1.34E-01	0.38	5.39E-01	-0.20
382.181@10.7833	382.1810	10.78	NEG	RP				1.21E-02	7.62E-02	-0.51	2.67E-02	0.64	4.08E-02	0.52	9.96E-01	0.00
382.2014@11.037304	382.2014	11.04	NEG	RP				2.37E-01	3.86E-01	0.26	3.23E-02	0.63	1.01E-02	0.71	4.02E-02	0.58
382.2045@10.703341	382.2045	10.70	POS	RP				2.05E-01	1.66E-01	0.40	2.44E-02	0.70	1.50E-02	0.74	4.00E-01	0.27
382.3244@10.236972	382.3244	10.24	POS	RP				9.36E-03	3.51E-01	0.22	3.55E-02	0.56	5.82E-02	-0.35	2.55E-01	-0.22
382.3928@11.075798	382.3928	11.08	POS	RP				8.42E-01	3.68E-01	-0.30	7.74E-01	0.09	8.62E-01	0.06	5.98E-01	-0.16
383.1153@9.4339285	383.1153	9.43	NEG	RP				9.68E-01	8.87E-01	0.04	7.41E-01	0.08	4.99E-01	0.17	5.13E-01	0.19
383.153@10.429922	383.1530	10.43	NEG	RP				6.67E-01	7.38E-01	0.08	1.64E-01	0.43	2.48E-01	0.33	8.33E-01	0.05
383.2703@0.9244865	383.2703	0.92	POS	hilic				1.26E-01	1.40E-01	-0.36	1.30E-01	0.41	1.66E-01	-0.34	8.74E-01	0.04
383.3405@10.623351	383.3405	10.62	POS	RP				1.67E-01	6.18E-01	-0.13	8.06E-01	0.08	9.25E-02	0.55	1.60E-01	-0.40
383.8492@3.198136	383.8492	3.20	NEG	hilic				8.69E-01	8.68E-01	0.05	8.21E-01	-0.07	4.57E-01	0.22	3.36E-01	0.28
383.9954@0.5165	383.9954	0.52	NEG	hilic				4.51E-01	3.14E-01	-0.34	5.38E-01	-0.15	4.18E-02	-0.62	8.82E-01	-0.04
384.1151@5.101631	384.1151	5.10	POS	hilic				1.13E-01	1.07E-02	-0.68	2.11E-01	0.34	8.60E-01	0.05	9.72E-01	-0.01
384.1294@5.111266	384.1294	5.11	NEG	hilic				6.99E-02	2.58E-01	-0.33	4.67E-02	0.53	1.96E-02	0.60	4.92E-01	0.19
384.16@6.0763426	384.1600	6.08	NEG	RP				2.60E-02	7.52E-01	-0.11	5.84E-04	0.88	6.17E-01	0.14	5.49E-01	-0.14
384.1964@11.034529	384.1964	11.03	NEG	RP				5.07E-01	2.41E-01	0.36	4.27E-02	0.57	6.14E-02	0.55	1.96E-01	0.38
384.2132@11.029407	384.2132	11.03	POS	RP				6.94E-01	3.24E-01	-0.26	4.09E-01	0.21	4.29E-01	0.23	9.20E-01	-0.03
384.4078@11.393392	384.4078	11.39	POS	RP				7.33E-01	6.46E-02	-0.37	5.57E-01	-0.14	6.09E-01	-0.11	7.07E-01	-0.09
385.0997@10.319334	385.0997	10.32	NEG	RP				3.15E-02	1.02E-01	-0.49	5.56E-02	0.57	5.89E-02	0.47	8.98E-01	0.03
385.168@10.724508	385.1680	10.72	NEG	RP				1.53E-01	5.94E-02	0.62	6.68E-01	0.12	3.04E-01	-0.29	1.54E-01	0.37
385.1685@10.911501	385.1685	10.91	NEG	RP				6.07E-01	3.67E-01	0.21	4.98E-01	0.15	8.68E-01	-0.03	9.51E-02	0.39
385.2115@10.701848	385.2115	10.70	POS	RP				7.81E-01	3.27E-01	0.33	3.70E-01	0.24	5.19E-01	0.20	7.41E-01	-0.09
385.2138@11.030126	385.2138	11.03	POS	RP				8.43E-01	2.37E-01	0.40	7.28E-01	0.11	3.55E-01	0.30	4.98E-01	0.19
385.2829@0.9019354	385.2829	0.90	POS	hilic				5.79E-02	8.53E-02	-0.53	4.08E-02	0.57	8.67E-01	-0.04	5.84E-01	0.17
385.283@7.6416035	385.2830	7.64	POS	RP				5.53E-02	1.92E-02	-0.74	1.93E-01	0.37	8.29E-01	-0.05	9.03E-01	0.03
385.2832@0.7497236	385.2832	0.75	POS	hilic				1.18E-03	6.92E-04	-0.65	9.50E-02	0.41	4.10E-01	-0.19	3.36E-02	0.60
385.2832@7.6605	385.2832	7.66	POS	RP				1.33E-02	1.25E-02	-0.78	1.13E-01	0.45	4.90E-01	-0.18	5.05E-01	0.19
385.3201@0.41868094	385.3201	0.42	POS	hilic				9.09E-01	7.00E-01	-0.09	4.09E-01	-0.24	4.69E-01	-0.20	3.46E-01	-0.30
385.9418@5.41463	385.9418	5.41	NEG	hilic				9.29E-01	3.71E-01	-0.27	4.68E-01	-0.20	3.23E-01	-0.28	6.95E-01	-0.12
385.942@4.4890447	385.9420	4.49	NEG	hilic				6.93E-01	6.11E-01	-0.14	2.21E-01	0.41	5.88E-01	0.15	8.63E-01	0.05
385.942@4.4912424	385.9420	4.49	NEG	hilic				7.74E-01	9.28E-01	-0.03	2.20E-01	0.42	5.61E-01	0.16	6.10E-01	0.14
386.0236@7.024734	386.0236	7.02	POS	hilic				6.56E-01	3.54E-01	0.24	1.05E-01	0.42	2.32E-01	0.29	5.87E-01	0.11
386.1227@5.1815004	386.1227	5.18	NEG	hilic				1.10E-03	5.67E-06	-1.12	4.70E-01	0.20	8.24E-01	-0.06	1.03E-02	-0.66
386.1753@6.2626595	386.1753	6.26	NEG	RP				8.15E-01	8.40E-01	0.05	2.21E-01	0.31	9.52E-01	-0.01	9.44E-01	0.02
386.1755@5.450059	386.1755	5.45	NEG	RP				2.08E-01	5.83E-01	-0.18	4.03E-02	0.55	6.66E-01	-0.12	3.19E-01	-0.27
386.1757@0.83618665	386.1757	0.84	NEG	hilic				8.17E-01	9.37E-01	-0.02	2.57E-01	0.32	5.68E-01	0.15	9.38E-01	-0.02
386.1759@5.4672036	386.1759	5.47	NEG	RP				4.33E-01	8.69E-01	-0.05	6.77E-02	0.51	9.04E-01	-0.03	5.55E-01	-0.17
386.176@7.0528364	386.1760	7.05	NEG	RP				8.92E-01	5.80E-01	-0.15	9.15E-01	0.03	6.75E-01	-0.11	5.47E-01	0.18
386.1762@0.8386521	386.1762	0.84	NEG	hilic				3.24E-01	5.79E-01	0.18	3.06E-02	0.58	8.62E-01	0.05	6.60E-01	-0.11
386.1861@10.709295	386.1861	10.71	NEG	RP				7.36E-01	2.57E-01	0.34	8.06E-01	0.06	5.85E-01	-0.13	8.35E-01	-0.05
386.2461@8.142707	386.2461	8.14	POS	RP				1.93E-02	2.20E-01	-0.39	6.58E-01	0.12	3.08E-03	0.84	6.17E-01	0.12
387.2137@11.031229	387.2137	11.03	POS	RP				3.20E-01	2.81E-02	0.67	3.79E-02	0.59	1.63E-01	0.45	7.19E-02	0.59
387.2208@10.861688	387.2208	10.86	POS	RP				2.40E-01	1.13E-02	0.64	6.76E-01	-0.12	2.17E-01	0.36	5.84E-01	0.13
387.2519@2.1612282	387.2519	2.16	POS	hilic				6.34E-01	2.04E-01	0.33	8.66E-01	0.04	3.95E-01	0.22	9.07E-02	0.44
387.298@0.8955538	387.2980	0.90	POS	hilic				4.65E-02	1.21E-01	-0.45	4.08E-02	0.61	2.87E-01	0.28	3.02E-01	0.30
387.2994@8.056891	387.2994	8.06	POS	RP				1.01E-02	1.07E-02	-0.69	3.48E-02	0.53	6.20E-01	0.13	4.35E-01	0.23
388.1914@6.675987	388.1914	6.68	NEG	RP				7.42E-01	6.26E-01	-0.13	9.24E-01	-0.03	3.13E-01	-0.26	4.53E-01	0.21
388.2622@8.699546	388.2622	8.70	POS	RP				5.63E-01	1.17E-01	-0.48	6.71E-01	0.12	6.92E-01	0.11	8.69E-01	0.05
388.2947@11.029506	388.2947	11.03	POS	RP				9.56E-01	3.80E-01	0.24	5.71E-01	0.15	8.59E-01	0.05	5.20E-01	0.20
388.3306@10.377259	388.3306	10.38	POS	RP				7.91E-01	1.03E-01	-0.31	6.80E-01	-0.09	3.48E-01	-0.19	5.36E-01	-0.13
389.0491@10.322059	389.0491	10.32	NEG	RP				1.22E-02	5.50E-02	-0.58	2.44E-02	0.60	1.26E-01	0.40	9.68E-01	-0.01
389.1545@10.867128	389.1545	10.87	NEG	RP				3.77E-01	5.89E-01	0.18	3.60E-02	0.60	3.86E-02	0.59	4.46E-01	0.22
389.1787@10.533352	389.1787	10.53	POS	RP				9.17E-04	4.93E-03	-0.66	5.43E-03	0.70	9.33E-02	0.43	6.14E-01	-0.14
389.2084@10.328001	389.2084	10.33	POS	RP				9.57E-01	9.93E-01	0.00	3.70E-01	0.20	7.71E-01	0.08	5.88E-01	0.15
389.8813@15.260575	389.8813	15.26	NEG	RP				1.03E-01	3.76E-01	0.24	1.83E-02	0.70	9.82E-01	-0.01	2.87E-02	0.68
389.9723@6.226841	389.9723	6.23	POS	hilic				4.80E-01	1.42E-01	-0.41	2.03E-01	-0.39	9.95E-01	0.00	1.02E-01	-0.51
390.095@5.192646	390.0950	5.19	POS	hilic				1.25E-02	8.14E-05	-1.02	7.70E-01	-0.09	6.53E-01	-0.11	9.48E-03	-0.70
390.0952@5.1923666	390.0952	5.19	POS	hilic				1.30E-02	8.31E-05	-1.03	7.61E-01	-0.10	6.47E-01	-0.11	1.02E-02	-0.69
390.1062@10.707045	390.1062	10.71	NEG	RP				1.18E-01	3.14E-01	0.27	1.06E-02	0.86	1.84E-02	0.68	1.69E-01	0.39
390.1527@10.526259	390.1527	10.53	NEG	RP				1.07E-02	8.70E-05	0.98	9.12E-01	0.03	4.71E-01	-0.20	8.51E-01	-0.05
390.2767@8.940665	390.2767	8.94	POS	RP				8.14E-02	9.24E-01	-0.02	9.62E-01	-0.01	2.30E-01	-0.24	4.19E-02	0.60
390.2778@10.908455	390.2778	10.91	POS	RP				6.38E-01	4.27E-01	0.21	7.62E-01	-0.09	8.81E-01	0.05	7.59E-02	0.47
390.3071@10.718597	390.3071	10.72	POS	RP				3.54E-01	8.15E-01	-0.06	1.58E-01	0.38	3.01E-02	0.56	3.63E-01	0.24
391.1005@5.0206604	391.1005	5.02	POS	hilic				5.05E-01	8.92E-02	-0.37	4.40E-01	0.21	7.70E-01	0.07	9.11E-01	0.03
391.1331@10.712528	391.1331	10.71	NEG	RP				5.01E-01	1.30E-01	0.50	7.90E-02	0.53	1.37E-01	0.42	6.20E-02	0.54
391.1848@6.294166	391.1848	6.29	POS	RP				2.36E-01	1.47E-02	0.84	4.81E-02	0.47	1.49E-01	0.40	4.98E-01	0.21
391.1853@0.42648688	391.1853	0.43	POS	hilic				4.37E-02	2.38E-03	0.94	4.41E-01	0.19	6.10E-01	0.14	5.70E-01	-0.17
391.2731@7.812083	391.2731	7.81	POS	RP				2.41E-02	1.87E-02	-0.59	3.18E-02	0.53	5.66E-01	0.16	4.83E-01	0.20
391.2735@0.66869235	391.2735	0.67	POS	hilic				5.95E-01	1.08E-01	-0.32	4.08E-01	0.21	9.53E-01	0.01	8.18E-01	0.05
392.0018@3.329452	392.0018	3.33	NEG	RP				3.25E-03	2.31E-02	-0.67	1.08E-02	0.64	1.84E-01	0.37	2.67E-01	-0.27

Supplementary table 2: Metabolomics analysis results

								ANOVA	Anorexia vs control		overweight vs control		obese vs control		athletes vs control	
Compound	Mass	Retention Time	Ionization mode	column	Compound_ID	MSMS_spectra	ID_level	P-FDR	P	Estimate	P	Estimate	P	Estimate	P	Estimate
392.0019@3.0846002	392.0019	3.08	NEG	RP				1.47E-02	1.74E-02	-0.55	1.86E-02	0.61	4.66E-01	0.21	6.33E-01	-0.11
392.1037@10.707918	392.1037	10.71	NEG	RP				6.43E-02	6.07E-01	0.14	8.62E-03	0.85	7.34E-03	0.76	2.27E-01	0.36
392.1397@10.716858	392.1397	10.72	NEG	RP				6.46E-01	7.92E-01	0.10	1.77E-01	-0.34	1.56E-01	-0.35	9.86E-01	0.00
392.2136@10.703002	392.2136	10.70	POS	RP				2.37E-01	3.09E-02	0.74	4.21E-02	0.60	2.75E-01	0.31	3.49E-02	0.62
392.2166@10.711629	392.2166	10.71	NEG	RP				4.92E-01	4.92E-02	-0.55	4.94E-01	-0.23	9.52E-01	-0.02	7.17E-02	-0.45
392.2919@9.581674	392.2919	9.58	NEG	RP	dublicate of chenodeoxycholic acid			7.77E-01	5.74E-01	-0.17	5.07E-01	0.17	2.67E-01	0.28	5.46E-01	0.22
392.2919@10.724863	392.2919	10.72	POS	RP				5.22E-01	5.79E-01	0.15	7.02E-02	0.51	8.89E-01	0.04	1.37E-01	0.33
392.2919@8.967485	392.2919	8.97	NEG	RP				7.49E-01	3.14E-01	-0.25	3.84E-01	0.21	6.30E-01	0.12	9.72E-01	0.01
392.2922@9.568017	392.2922	9.57	NEG	RP	Chenodeoxycholic acid	10 eV: 391.2847 (100)	1	8.00E-01	7.84E-01	-0.09	4.07E-01	0.23	2.64E-01	0.32	9.66E-01	-0.01
393.2006@9.638894	393.2006	9.64	NEG	RP				3.17E-03	3.30E-03	-0.66	3.04E-01	0.27	1.48E-02	0.53	1.67E-01	-0.38
393.2007@0.94819987	393.2007	0.95	POS	hilic				1.14E-01	1.59E-01	-0.35	1.89E-01	0.36	3.89E-02	0.56	9.10E-01	-0.03
393.2015@9.644169	393.2015	9.64	POS	RP				8.95E-04	8.28E-03	-0.67	1.37E-01	0.38	1.63E-03	0.70	1.69E-01	-0.43
393.2539@11.076495	393.2539	11.08	POS	RP				6.96E-01	1.86E-01	-0.41	5.36E-01	-0.18	7.34E-01	0.10	4.33E-01	-0.23
393.2881@8.161538	393.2881	8.16	POS	RP				2.82E-02	2.17E-02	-0.69	9.25E-02	0.47	3.65E-01	0.23	5.79E-01	0.16
393.2884@0.6621312	393.2884	0.66	POS	hilic				5.73E-01	5.96E-01	-0.15	1.19E-01	0.39	8.47E-01	0.05	6.90E-01	0.09
393.3466@0.4288055	393.3466	0.43	POS	hilic				6.70E-01	9.24E-01	0.03	2.48E-01	0.36	6.30E-01	0.16	1.31E-01	0.47
394.14@10.717411	394.1400	10.72	NEG	RP				2.72E-01	5.26E-02	0.55	2.21E-01	-0.32	8.19E-01	-0.06	8.23E-01	0.07
394.2138@10.703197	394.2138	10.70	POS	RP				1.31E-01	1.30E-01	0.44	7.34E-03	0.78	4.30E-03	0.72	5.48E-01	0.20
394.2147@10.860955	394.2147	10.86	POS	RP				4.43E-01	5.96E-02	0.60	7.31E-02	0.49	1.52E-01	0.45	6.48E-01	0.13
394.2331@9.4895115	394.2331	9.49	POS	RP				8.01E-01	6.52E-01	0.16	9.57E-01	0.02	7.53E-01	0.08	2.02E-01	0.42
394.2462@11.071921	394.2462	11.07	NEG	RP				6.30E-01	2.43E-01	0.36	7.91E-02	0.51	7.18E-01	0.09	3.86E-01	0.24
395.1283@10.320584	395.1283	10.32	NEG	RP				1.13E-01	5.09E-01	0.18	1.88E-02	0.75	2.69E-03	0.78	2.29E-01	0.36
395.2151@0.4980001	395.2151	0.50	POS	hilic				7.90E-01	9.04E-01	0.03	3.61E-01	0.25	2.00E-01	0.36	3.33E-01	0.26
395.3039@8.358348	395.3039	8.36	POS	RP				4.03E-01	4.54E-01	-0.27	1.27E-01	0.38	9.22E-01	-0.03	2.40E-01	0.33
395.3048@0.65674984	395.3048	0.66	POS	hilic				2.50E-01	5.13E-02	0.57	6.90E-01	-0.10	3.25E-01	0.25	5.61E-01	-0.13
395.9829@7.219608	395.9829	7.22	NEG	RP				7.61E-01	5.00E-01	-0.20	9.25E-01	-0.03	2.35E-01	0.30	5.66E-01	0.17
395.983@4.4154305	395.9830	4.42	NEG	hilic				5.96E-01	9.37E-01	-0.02	3.69E-01	0.23	7.38E-01	0.08	1.13E-01	0.44
396.1698@6.3955717	396.1698	6.40	NEG	RP				4.68E-01	1.02E-01	-0.42	3.45E-01	-0.23	1.64E-01	-0.36	7.06E-01	0.11
396.1967@0.52507347	396.1967	0.53	NEG	hilic				4.61E-01	2.79E-02	-0.56	6.05E-01	-0.14	9.22E-01	0.03	3.63E-01	-0.22
396.1969@8.213946	396.1969	8.21	NEG	RP	Steroid sulfate 3	10 eV: 395.1894 (100), 96.9610 (24)	3	2.05E-01	3.74E-01	-0.22	1.19E-02	0.65	9.13E-01	0.04	9.41E-01	0.02
396.2102@10.702146	396.2102	10.70	POS	RP				2.40E-01	2.69E-01	0.32	1.76E-02	0.72	1.62E-02	0.62	5.84E-01	0.18
396.2192@10.860416	396.2192	10.86	POS	RP				2.55E-01	2.74E-02	0.71	6.84E-02	0.53	2.31E-02	0.69	6.17E-02	0.55
396.2195@10.860766	396.2195	10.86	POS	RP				9.54E-02	1.12E-02	0.89	6.72E-02	0.48	6.24E-03	0.81	1.20E-01	0.43
396.2267@10.516776	396.2267	10.52	NEG	RP				4.72E-03	1.67E-03	-0.90	2.77E-01	0.29	1.97E-01	0.35	4.60E-01	-0.23
396.2302@9.489563	396.2302	9.49	POS	RP				7.10E-01	3.00E-01	0.36	5.37E-02	0.47	3.17E-01	0.25	1.95E-01	0.39
396.3736@11.554121	396.3736	11.55	POS	RP				4.37E-02	1.65E-01	0.43	8.73E-02	-0.38	2.12E-01	-0.29	1.78E-01	0.37
396.4066@11.449861	396.4066	11.45	POS	RP				8.31E-01	2.65E-01	-0.32	9.76E-01	-0.01	9.51E-01	0.02	4.57E-01	-0.20
397.0254@5.278744	397.0254	5.28	POS	hilic				2.77E-01	4.78E-02	0.59	1.17E-01	0.30	2.58E-02	0.56	7.02E-02	0.34
397.1238@10.350054	397.1238	10.35	NEG	RP				3.12E-01	2.29E-01	-0.33	1.53E-01	0.41	2.82E-01	0.30	8.68E-01	0.05
397.2259@9.865583	397.2259	9.87	POS	RP				5.04E-01	9.96E-01	0.00	7.02E-02	0.45	6.89E-02	0.53	5.46E-01	0.21
397.3191@8.67308	397.3191	8.67	POS	RP	Acylcarnitine 16:1	10 eV: 398.3261 (100), 85.0278 (38)	2	9.28E-02	9.96E-01	0.00	2.68E-03	0.79	1.76E-02	0.60	5.97E-01	0.15
397.3205@0.65744793	397.3205	0.66	POS	hilic				4.97E-02	7.77E-01	0.09	2.57E-03	0.80	1.39E-02	0.61	9.57E-01	0.01
397.7559@12.270452	397.7559	12.27	POS	RP				6.98E-01	3.35E-01	0.26	3.26E-01	0.30	6.57E-01	-0.12	7.01E-01	-0.12
397.998@7.5764008	397.9980	7.58	NEG	RP				1.41E-01	7.02E-03	-0.90	8.24E-01	-0.07	3.95E-01	-0.27	5.05E-01	-0.18
398.0098@0.50295	398.0098	0.50	NEG	hilic				2.22E-01	8.52E-02	0.66	1.82E-01	0.30	5.00E-01	-0.18	6.26E-01	0.12
398.01@3.4465885	398.0100	3.45	NEG	RP				2.51E-02	1.24E-02	0.89	7.86E-01	0.06	4.49E-01	-0.20	2.51E-01	0.28
398.1851@6.63513	398.1851	6.64	NEG	RP				3.02E-01	6.70E-01	0.13	1.92E-01	0.37	3.20E-01	-0.30	1.29E-01	0.44
398.1881@3.5227287	398.1881	3.52	NEG	hilic				3.89E-01	4.75E-01	-0.23	4.38E-02	-0.62	4.09E-01	-0.23	2.77E-02	-0.52
398.2119@8.466343	398.2119	8.47	NEG	RP				3.82E-01	2.21E-01	-0.19	1.95E-01	0.31	2.49E-01	0.28	9.73E-01	0.01
398.2121@7.852924	398.2121	7.85	NEG	RP				3.31E-02	2.73E-01	-0.28	2.27E-02	0.64	3.82E-01	0.26	1.86E-01	-0.39
398.2123@0.5770433	398.2123	0.58	NEG	hilic				2.85E-01	4.04E-01	-0.20	7.49E-02	0.52	7.70E-01	0.10	4.08E-01	-0.23
398.2123@7.4171634	398.2123	7.42	NEG	RP				4.04E-01	1.30E-01	-0.33	4.82E-01	0.17	7.08E-01	-0.10	9.95E-02	-0.34
398.2126@8.785706	398.2126	8.79	NEG	RP				1.08E-01	1.02E-01	-0.28	1.53E-01	0.34	9.35E-02	0.44	8.64E-01	-0.04
398.2298@9.119494	398.2298	9.12	NEG	RP				9.53E-01	7.23E-01	0.10	5.17E-01	-0.16	8.04E-01	-0.06	7.62E-01	-0.08
398.2429@10.700084	398.2429	10.70	NEG	RP				6.51E-02	8.73E-03	-0.62	2.82E-01	0.27	2.55E-01	0.31	7.08E-01	-0.11
398.2435@0.4190418	398.2435	0.42	POS	hilic				6.42E-01	1.79E-01	-0.40	8.92E-01	-0.04	1.84E-01	-0.34	1.39E-01	-0.35
398.2443@9.445611	398.2443	9.45	POS	RP				8.61E-01	7.31E-01	-0.10	6.96E-01	0.12	8.08E-01	-0.07	3.18E-01	-0.28
398.2478@11.041294	398.2478	11.04	NEG	RP				6.36E-02	3.33E-04	0.86	2.37E-02	0.66	5.08E-01	0.16	8.69E-02	0.45
398.3391@11.076452	398.3391	11.08	NEG	RP				7.64E-01	2.73E-01	-0.33	4.98E-01	-0.17	1.71E-01	-0.39	9.26E-01	-0.03
398.3399@11.026076	398.3399	11.03	POS	RP				2.43E-01	5.75E-02	0.66	9.29E-01	0.02	5.06E-01	-0.16	6.35E-01	0.13
398.3407@11.121802	398.3407	11.12	POS	RP				9.35E-01	8.77E-01	-0.05	5.15E-01	0.22	8.37E-01	-0.06	8.40E-01	-0.06
398.7626@12.707377	398.7626	12.71	POS	RP				2.91E-01	1.08E-01	0.47	5.93E-01	-0.16	1.77E-01	-0.38	7.98E-01	0.08
399.1138@3.5897415	399.1138	3.59	NEG	hilic				7.98E-01	7.42E-01	0.08	5.73E-01	0.17	7.72E-01	-0.09	3.26E-01	-0.26
399.1392@10.568119	399.1392	10.57	NEG	RP				6.79E-02	9.94E-02	-0.50	1.06E-01	0.50	1.10E-01	0.43	6.81E-01	0.12
399.1835@10.868436	399.1835	10.87	NEG	RP				7.79E-01	1.54E-01	0.42	2.03E-01	0.36	3.88E-01	0.24	2.81E-01	0.33
399.3356@8.996438	399.3356	9.00	POS	RP	Acylcarnitine 16:0	10 eV: 400.3432 (100), 85.0303 (10)	2	2.48E-01	8.58E-01	0.07	2.02E-02	0.63	2.08E-02	0.60	6.27E-01	0.13
399.3358@0.65139365	399.3358	0.65	POS	hilic				2.38E-01	6.60E-01	0.16	4.97E-02	0.57	1.75E-02	0.65	9.86E-01	0.00
399.3546@10.466066	399.3546	10.47	POS	RP				7.99E-02	2.47E-01	0.30	1.80E-01	-0.35	4.38E-02	0.59	9.22E-01	0.02
399.3546@10.639213	399.3546	10.64	POS	RP				5.54E-01	2.13E-01	-0.38	3.50E-01	0.28	7.02E-01	0.12	8.57E-01	-0.05
399.3547@10.452336	399.3547	10.45	POS	RP				4.95E-02	7.17E-01	0.07	7.43E-02	-0.37	9.24E-02	0.53	8.25E-02	0.44
400.0856@2.0440834	400.0856	2.04	NEG	hilic				1.21E-01	2.27E-02	0.67	7.98E-01	-0.05	9.52E-01	0.01	1.64E-01	0.35
400.1551@10.432836	400.1551	10.43	NEG	RP				1.18E-03	1.40E-01	-0.38	3.11E-03	0.86	4.95E-03	0.73	7.29E-01	0.09
400.1922@11.047415	400.1922	11.05	NEG	RP				7.50E-01	1.55E-01	0.42	3.27E-01	0.27	8.62E-01	0.05	2.61E-01	0.31

Supplementary table 2: Metabolomics analysis results

Compound	Mass	Retention Time	Ionization mode	column	Compound_ID	MSMS_spectra	ID_level	ANOVA P-FDR	Anorexia vs control P	Anorexia vs control Estimate	overweight vs control P	overweight vs control Estimate	obese vs control P	obese vs control Estimate	athletes vs control P	athletes vs control Estimate
400.226@0.5844118	400.2260	0.58	NEG	hilic				1.93E-01	1.41E-01	-0.30	3.00E-01	0.27	2.58E-01	0.30	2.57E-01	-0.24
400.2272@8.08729	400.2272	8.09	NEG	RP				1.17E-01	2.22E-02	-0.60	7.67E-01	0.08	4.77E-01	0.21	6.75E-02	-0.47
400.2276@8.731572	400.2276	8.73	NEG	RP				5.69E-02	3.02E-02	-0.54	4.34E-01	0.21	3.05E-01	0.31	7.50E-02	-0.47
400.2575@10.80492	400.2575	10.80	NEG	RP				6.77E-02	8.44E-03	-0.38	9.05E-01	0.03	9.65E-02	0.41	4.64E-01	-0.14
400.2619@0.42287803	400.2619	0.42	POS	hilic				8.52E-01	2.25E-01	-0.35	7.60E-01	-0.08	5.69E-01	-0.16	9.79E-01	0.01
400.2621@9.657208	400.2621	9.66	POS	RP				7.93E-01	1.02E-01	0.44	3.12E-01	0.27	6.06E-01	0.15	8.63E-01	0.06
400.3361@10.900044	400.3361	10.90	POS	RP				8.41E-01	6.85E-01	-0.13	7.90E-01	-0.07	3.82E-01	0.23	5.73E-01	-0.15
401.1555@10.783429	401.1555	10.78	NEG	RP				4.10E-01	9.60E-01	-0.01	1.95E-01	0.39	2.26E-02	0.57	2.83E-01	0.30
401.1628@10.321154	401.1628	10.32	NEG	RP				3.10E-02	4.09E-02	0.56	6.26E-02	-0.26	4.76E-01	-0.13	5.24E-01	0.13
401.1641@10.709885	401.1641	10.71	NEG	RP				4.97E-01	7.76E-02	0.49	1.09E-01	0.48	6.39E-02	0.48	7.44E-02	0.55
401.1962@10.712071	401.1962	10.71	NEG	RP				3.01E-01	7.45E-01	0.08	3.68E-02	0.60	2.64E-01	0.25	6.66E-02	0.45
401.205@1.0038731	401.2050	1.00	POS	hilic				9.33E-01	9.10E-01	0.03	5.30E-01	-0.19	6.30E-01	-0.12	8.11E-01	0.06
401.2368@11.028873	401.2368	11.03	POS	RP				5.84E-01	1.15E-01	0.48	2.57E-01	0.36	1.48E-01	0.45	1.00E-01	0.53
401.2376@10.777383	401.2376	10.78	POS	RP				7.62E-01	8.31E-01	-0.06	5.72E-01	0.15	9.26E-01	-0.02	2.37E-01	-0.31
401.3534@8.85294	401.3534	8.85	POS	RP				2.83E-01	3.69E-01	0.28	2.15E-01	-0.34	8.78E-01	0.04	1.74E-01	0.46
401.9896@7.8510857	401.9896	7.85	NEG	RP				9.84E-01	7.66E-01	0.08	7.01E-01	0.12	5.82E-01	0.16	5.36E-01	0.20
402.0021@4.6952615	402.0021	4.70	POS	hilic				6.47E-01	2.20E-01	0.32	6.51E-01	-0.12	3.90E-01	0.30	2.58E-01	0.28
402.155@10.432704	402.1550	10.43	NEG	RP				7.42E-04	3.62E-03	-0.71	3.92E-02	0.55	3.21E-02	0.58	4.36E-01	-0.21
402.1695@10.709597	402.1695	10.71	NEG	RP				3.04E-01	8.17E-03	0.72	2.61E-01	0.35	9.99E-01	0.00	5.96E-01	0.15
402.2268@9.681847	402.2268	9.68	POS	RP				5.82E-01	2.68E-01	0.38	1.58E-01	0.40	5.06E-02	0.59	2.17E-01	0.39
402.2388@11.028251	402.2388	11.03	POS	RP				5.96E-01	1.20E-01	0.40	3.73E-01	0.27	6.71E-01	0.14	5.08E-02	0.54
402.346@10.378629	402.3460	10.38	POS	RP				1.49E-01	3.66E-01	-0.21	6.42E-03	-0.55	1.10E-02	-0.53	4.53E-01	-0.20
403.1703@11.060112	403.1703	11.06	NEG	RP				4.76E-01	1.55E-01	0.47	7.68E-02	0.57	1.06E-01	0.44	4.55E-02	0.55
403.1983@0.4592143	403.1983	0.46	POS	hilic				6.68E-01	4.67E-01	-0.16	7.38E-01	-0.08	5.28E-01	-0.14	2.99E-01	0.26
403.2961@10.696589	403.2961	10.70	POS	RP				9.23E-01	9.93E-01	0.00	9.64E-01	-0.01	3.53E-01	-0.25	5.01E-01	-0.17
403.3656@9.3223505	403.3656	9.32	NEG	RP				9.84E-01	9.03E-01	0.04	8.43E-01	-0.05	6.72E-01	0.13	8.17E-01	-0.07
404.1534@10.433543	404.1534	10.43	NEG	RP				1.51E-02	2.28E-02	-0.50	3.42E-02	0.56	4.11E-01	0.22	4.25E-01	-0.20
404.164@10.539699	404.1640	10.54	NEG	RP				4.66E-01	1.10E-01	-0.33	3.59E-01	0.24	8.35E-01	0.05	4.89E-01	-0.17
404.1702@10.713857	404.1702	10.71	NEG	RP				7.36E-01	9.79E-02	0.50	3.21E-01	0.33	4.91E-01	0.20	2.69E-01	0.32
404.3648@0.42150941	404.3648	0.42	POS	hilic				1.52E-03	3.65E-04	-1.00	1.47E-01	0.36	4.91E-01	0.16	4.98E-01	0.21
405.097@5.5407825	405.0970	5.54	POS	hilic				6.10E-01	9.12E-01	-0.03	3.65E-01	-0.23	2.39E-01	0.36	9.64E-01	-0.01
405.1562@10.318137	405.1562	10.32	NEG	RP				2.76E-02	3.09E-01	-0.33	1.13E-02	0.73	2.05E-02	0.59	4.14E-01	0.25
405.2047@0.46535003	405.2047	0.47	POS	hilic				8.01E-01	8.56E-01	-0.05	9.19E-01	0.03	1.85E-01	0.34	7.06E-01	0.09
405.3226@10.6244955	405.3226	10.62	POS	RP				2.19E-01	8.00E-01	0.06	1.96E-01	0.40	1.10E-01	0.48	2.98E-01	-0.32
405.8518@15.283245	405.8518	15.28	NEG	RP				4.51E-01	8.29E-02	0.59	9.93E-01	0.00	4.30E-01	0.24	8.50E-01	-0.06
406.0497@0.51769626	406.0497	0.52	NEG	hilic				7.96E-01	8.08E-01	-0.07	4.77E-01	-0.16	1.55E-01	-0.33	8.91E-01	0.03
406.069@5.0305486	406.0690	5.03	POS	hilic				1.53E-02	2.67E-04	-1.04	1.72E-01	-0.43	9.15E-01	-0.03	1.76E-02	-0.65
406.0692@5.0295095	406.0692	5.03	POS	hilic				1.41E-02	5.71E-05	-1.13	1.64E-01	-0.43	5.07E-01	-0.18	1.95E-02	-0.62
406.167@10.71324	406.1670	10.71	NEG	RP				4.48E-01	1.62E-01	0.51	9.60E-01	0.01	3.83E-01	-0.23	5.10E-01	0.19
406.1698@11.055955	406.1698	11.06	NEG	RP				2.40E-01	1.08E-02	0.68	3.04E-01	0.25	7.00E-01	0.09	9.99E-02	0.45
406.198@6.3958473	406.1980	6.40	NEG	RP				4.46E-01	3.75E-01	-0.24	3.78E-01	-0.23	6.09E-02	-0.51	6.49E-01	0.15
406.1983@0.516085	406.1983	0.52	NEG	hilic				2.58E-01	2.30E-02	-0.45	5.22E-01	-0.15	3.60E-01	-0.21	3.67E-01	0.26
406.2289@10.860066	406.2289	10.86	POS	RP				3.03E-01	1.31E-02	0.69	8.27E-02	0.41	2.33E-01	0.29	7.73E-02	0.37
406.2289@10.860747	406.2289	10.86	POS	RP				4.14E-01	4.80E-02	0.59	4.66E-01	0.19	5.66E-02	0.55	1.68E-01	0.34
406.2416@11.030253	406.2416	11.03	POS	RP				3.92E-01	1.35E-01	0.39	4.27E-01	-0.19	5.61E-01	-0.15	4.43E-01	-0.20
407.1524@10.356826	407.1524	10.36	NEG	RP				6.74E-01	5.94E-01	0.12	8.31E-02	0.44	4.72E-01	0.17	5.07E-01	0.14
407.1731@10.574903	407.1731	10.57	NEG	RP				3.65E-01	2.29E-01	-0.32	1.41E-01	0.44	7.26E-01	0.09	6.27E-01	0.14
407.2203@0.42902598	407.2203	0.43	POS	hilic				1.50E-01	2.76E-01	0.30	1.10E-01	0.44	7.20E-01	-0.09	1.37E-01	-0.36
407.2494@11.420949	407.2494	11.42	POS	RP				4.00E-01	5.23E-01	0.20	4.14E-02	0.53	3.14E-02	0.62	1.23E-01	0.49
407.3013@0.41824257	407.3013	0.42	POS	hilic				6.21E-01	2.15E-02	-0.54	3.45E-01	-0.25	4.27E-01	-0.22	4.51E-01	-0.21
407.302@10.684309	407.3020	10.68	POS	RP				7.31E-01	2.06E-01	-0.32	1.31E-01	-0.44	1.66E-01	-0.40	2.48E-01	-0.38
407.3021@10.602895	407.3021	10.60	POS	RP				6.96E-01	6.60E-02	-0.47	2.05E-01	-0.37	1.99E-01	-0.36	1.90E-01	-0.43
407.303@9.30612	407.3030	9.31	POS	RP				9.77E-01	5.87E-01	0.15	9.86E-01	0.00	7.73E-01	-0.07	9.17E-01	-0.03
407.3572@10.59547	407.3572	10.60	POS	RP				3.14E-01	4.14E-02	0.55	1.69E-02	0.66	2.27E-01	0.28	1.60E-01	0.39
407.3617@0.42267638	407.3617	0.42	POS	hilic				8.72E-01	5.90E-01	0.18	9.99E-01	0.00	2.79E-01	0.27	3.41E-01	0.28
408.1539@0.42640916	408.1539	0.43	NEG	hilic				1.06E-03	1.17E-03	0.90	6.77E-01	-0.03	3.31E-01	0.14	8.21E-02	0.36
408.1795@2.4409876	408.1795	2.44	POS	RP				6.35E-01	1.35E-01	-0.38	8.04E-01	0.07	6.74E-01	-0.10	5.32E-01	0.16
408.2145@0.5176993	408.2145	0.52	NEG	hilic				1.26E-01	7.24E-01	-0.09	6.40E-02	0.57	7.98E-01	-0.08	2.74E-02	0.60
408.2145@6.636454	408.2145	6.64	NEG	RP				1.98E-01	6.39E-01	0.14	5.81E-02	0.54	4.77E-01	-0.21	5.90E-02	0.52
408.2286@10.860228	408.2286	10.86	POS	RP				4.54E-01	6.96E-02	0.48	2.34E-01	0.35	2.36E-02	0.66	4.46E-01	0.24
408.229@10.860929	408.2290	10.86	POS	RP				2.82E-01	9.14E-02	0.50	6.57E-02	0.52	9.29E-03	0.72	5.43E-01	0.18
408.2868@8.989321	408.2868	8.99	NEG	RP				4.76E-02	4.03E-01	-0.17	3.98E-01	-0.18	6.80E-01	0.10	2.41E-02	0.65
409.1673@10.567768	409.1673	10.57	NEG	RP				4.83E-01	9.74E-01	0.01	2.10E-01	0.38	1.98E-01	0.34	6.60E-02	0.53
409.3107@11.208275	409.3107	11.21	POS	RP				4.30E-01	4.82E-01	-0.20	6.30E-01	-0.11	4.61E-01	-0.18	1.88E-01	0.37
409.3193@9.574346	409.3193	9.57	POS	RP	Deoxycholic acid			7.58E-01	5.06E-01	-0.20	8.83E-01	0.04	3.30E-01	0.24	4.46E-01	0.27
409.3197@0.5008796	409.3197	0.50	POS	hilic				8.86E-01	3.52E-01	0.26	8.13E-01	0.06	9.55E-01	0.01	3.92E-01	0.26
409.6322@3.3354788	409.6322	3.34	NEG	hilic				8.55E-01	4.18E-01	-0.25	6.64E-01	0.10	7.72E-01	0.08	6.43E-01	-0.11
410.2274@10.861113	410.2274	10.86	POS	RP				6.36E-01	1.91E-01	0.47	8.21E-02	0.43	8.92E-02	0.47	2.76E-01	0.30
410.2365@11.029098	410.2365	11.03	POS	RP				3.60E-01	4.74E-02	0.63	1.07E-01	0.49	5.54E-02	0.61	6.84E-02	0.56
410.4235@11.450401	410.4235	11.45	POS	RP				7.26E-01	3.61E-01	-0.31	5.51E-01	0.18	7.70E-01	0.10	5.33E-01	-0.18
410.7618@12.442799	410.7618	12.44	POS	RP				4.60E-01	3.27E-01	0.31	2.20E-01	-0.36	4.33E-01	-0.21	5.09E-01	-0.19
411.1156@10.431452	411.1156	10.43	NEG	RP				8.99E-04	1.76E-02	-0.55	2.80E-02	0.61	4.96E-03	0.64	5.47E-01	-0.15
411.1838@10.788256	411.1838	10.79	NEG	RP				5.56E-01	4.78E-01	-0.18	1.87E-01	0.39	2.82E-01	0.28	9.51E-01	0.02

Supplementary table 2: Metabolomics analysis results

Compound	Mass	Retention Time	Ionization mode	column	Compound_ID	MSMS_spectra	ID_level	ANOVA P-FDR	Anorexia vs control P	Anorexia vs control Estimate	overweight vs control P	overweight vs control Estimate	obese vs control P	obese vs control Estimate	athletes vs control P	athletes vs control Estimate
411.2987@0.8617916	411.2987	0.86	POS	hilic				1.26E-01	1.88E-02	-0.40	1.08E-01	0.39	3.04E-01	0.25	9.36E-01	0.02
411.7707@12.96317	411.7707	12.96	POS	RP				2.73E-01	5.51E-01	0.18	9.50E-03	-0.62	7.90E-01	-0.07	5.45E-01	-0.19
411.7707@12.970002	411.7707	12.97	POS	RP				3.11E-01	1.13E-01	0.39	8.00E-02	-0.46	9.35E-01	-0.02	8.01E-01	-0.08
411.8556@7.8778577	411.8556	7.88	POS	hilic				2.38E-01	5.84E-01	0.16	3.13E-02	-0.56	6.62E-01	0.14	9.68E-01	0.01
412.077@5.19045	412.0770	5.19	POS	hilic				1.88E-01	3.93E-02	-0.49	7.38E-01	0.10	2.86E-01	0.29	1.79E-01	-0.39
412.0772@5.1915684	412.0772	5.19	POS	hilic				2.52E-01	2.35E-02	-0.52	6.62E-01	0.12	1.04E-01	-0.43	5.14E-01	-0.15
412.1091@1.4797033	412.1091	1.48	NEG	hilic				4.32E-01	4.52E-01	0.22	1.99E-01	-0.32	1.85E-01	-0.36	8.84E-01	0.04
412.1297@0.5448113	412.1297	0.54	POS	hilic				6.65E-01	9.89E-02	-0.39	9.30E-01	0.02	9.90E-01	0.00	3.73E-01	-0.23
412.1913@6.541105	412.1913	6.54	NEG	RP				6.07E-01	6.38E-01	-0.14	4.96E-01	0.19	9.44E-01	-0.02	1.55E-01	-0.40
412.1913@7.416	412.1913	7.42	NEG	RP				1.55E-01	1.11E-01	-0.31	9.45E-02	0.41	2.70E-01	0.30	9.17E-01	-0.02
412.2265@11.42965	412.2265	11.43	NEG	RP				4.72E-01	4.57E-01	0.25	9.37E-02	0.43	2.16E-02	0.64	1.61E-01	0.44
412.3537@11.266987	412.3537	11.27	NEG	RP				9.34E-01	4.79E-01	0.21	9.05E-01	-0.03	5.07E-01	0.18	9.06E-01	0.03
412.3556@11.262979	412.3556	11.26	POS	RP				8.78E-02	4.37E-02	-0.53	7.63E-01	-0.08	2.89E-01	0.32	5.56E-02	-0.49
412.9878@6.2443337	412.9878	6.24	NEG	hilic				6.19E-01	5.02E-01	0.21	5.77E-01	0.15	5.78E-01	0.13	2.54E-02	0.53
413.1304@10.712383	413.1304	10.71	NEG	RP				4.39E-01	3.94E-01	0.28	6.09E-02	0.54	4.40E-02	0.61	5.44E-01	0.18
413.1785@10.506416	413.1785	10.51	POS	RP				1.79E-01	1.82E-02	-0.42	2.27E-01	0.30	6.69E-01	0.09	5.95E-01	-0.13
413.1993@11.0735235	413.1993	11.07	NEG	RP				8.75E-01	7.22E-01	-0.11	8.55E-01	0.05	1.74E-01	0.29	6.86E-01	0.12
413.2009@11.071366	413.2009	11.07	NEG	RP				9.29E-01	8.14E-01	0.07	7.51E-01	-0.08	3.67E-01	0.22	7.46E-01	0.09
413.2323@11.428259	413.2323	11.43	NEG	RP				7.43E-01	4.45E-01	0.18	7.21E-01	0.07	1.38E-01	0.41	5.15E-01	0.15
413.2463@11.029749	413.2463	11.03	POS	RP				7.01E-01	4.67E-01	0.20	1.44E-01	0.42	2.38E-01	0.34	1.47E-01	0.42
413.2929@8.924243	413.2929	8.92	POS	RP				8.44E-01	2.85E-01	0.35	2.91E-01	0.31	7.94E-01	0.07	6.12E-01	0.16
413.2932@7.936396	413.2932	7.94	POS	RP				3.10E-01	9.65E-01	-0.01	3.53E-02	0.57	9.98E-02	0.45	4.08E-01	0.19
413.2935@1.0046699	413.2935	1.00	POS	hilic				5.68E-01	1.06E-01	0.52	1.15E-01	0.48	6.28E-01	0.13	4.73E-01	0.22
413.3152@0.8408729	413.3152	0.84	POS	hilic				6.57E-03	5.73E-02	-0.46	1.22E-02	0.70	7.03E-02	0.49	5.64E-01	-0.17
413.983@3.4428005	413.9830	3.44	NEG	RP				1.52E-03	3.65E-03	0.90	3.49E-01	-0.15	3.53E-01	-0.17	6.21E-01	0.10
413.9844@0.5018667	413.9844	0.50	NEG	hilic				1.81E-01	7.55E-02	0.53	7.80E-01	-0.05	2.71E-01	-0.22	9.58E-01	0.01
414.1054@10.567186	414.1054	10.57	NEG	RP				1.74E-01	7.06E-02	-0.40	4.50E-01	0.21	7.67E-01	0.07	8.47E-02	-0.41
414.1449@0.5607273	414.1449	0.56	POS	hilic				4.19E-02	3.99E-04	-0.84	4.51E-03	-0.69	1.52E-01	-0.37	5.85E-02	-0.50
414.2049@8.057128	414.2049	8.06	POS	RP				1.62E-04	4.87E-03	-0.71	5.78E-03	-0.63	3.58E-04	-0.88	2.76E-09	-1.21
414.205@8.37122	414.2050	8.37	POS	RP				6.24E-05	1.66E-03	-0.81	4.03E-02	-0.49	3.14E-04	-0.86	1.33E-10	-1.32
414.2055@0.43710867	414.2055	0.44	POS	hilic				1.14E-04	2.69E-03	-0.74	1.21E-02	-0.60	1.27E-04	-0.90	5.76E-11	-1.25
414.2071@0.6650999	414.2071	0.67	NEG	hilic				1.84E-01	2.13E-01	-0.23	1.00E-01	0.39	5.19E-01	0.16	3.18E-01	-0.17
414.2071@7.236684	414.2071	7.24	NEG	RP				1.69E-02	7.49E-01	-0.08	2.12E-03	0.85	4.54E-02	0.60	7.46E-01	-0.09
414.2754@9.57326	414.2754	9.57	POS	RP				4.01E-01	8.58E-01	0.05	5.08E-01	0.16	1.25E-01	0.37	6.40E-02	0.59
414.3124@9.522218	414.3124	9.52	POS	RP				2.43E-02	9.11E-01	-0.02	2.47E-01	0.22	6.09E-03	0.71	8.00E-01	0.04
414.3135@8.99794	414.3135	9.00	POS	RP				4.67E-01	5.13E-01	-0.19	7.23E-01	0.10	2.82E-01	0.33	2.16E-01	-0.35
414.3136@10.374443	414.3136	10.37	POS	RP				5.01E-01	1.55E-02	-0.54	7.48E-01	-0.09	9.88E-01	0.00	9.99E-01	0.00
415.0666@10.435166	415.0666	10.44	NEG	RP				7.15E-04	1.40E-02	-0.57	1.44E-02	0.67	1.44E-02	0.56	5.44E-01	-0.16
415.1418@10.318431	415.1418	10.32	NEG	RP				4.41E-01	1.99E-01	0.38	3.02E-02	0.62	5.68E-01	0.16	1.20E-01	0.43
415.2123@10.711389	415.2123	10.71	NEG	RP				7.09E-01	9.07E-01	0.03	3.09E-01	0.31	3.38E-01	0.27	6.21E-01	-0.13
415.2209@3.2988398	415.2209	3.30	POS	RP				4.43E-01	7.88E-01	0.08	7.14E-01	-0.08	1.13E-01	0.43	1.32E-01	0.40
415.2225@0.5418387	415.2225	0.54	POS	hilic				7.19E-01	4.68E-01	0.22	9.11E-01	0.03	1.02E-01	0.44	3.99E-01	0.25
415.2377@0.57885367	415.2377	0.58	POS	hilic				2.68E-01	3.33E-02	-0.48	5.89E-01	0.14	4.33E-01	-0.20	1.57E-01	-0.34
415.2579@9.118819	415.2579	9.12	POS	RP				8.31E-01	4.61E-01	0.22	5.25E-01	-0.17	4.98E-01	-0.17	9.78E-01	0.01
415.3089@9.178929	415.3089	9.18	POS	RP				4.16E-01	8.64E-01	-0.06	9.04E-01	-0.03	4.54E-02	-0.53	5.50E-01	0.16
415.3113@9.4846945	415.3113	9.48	POS	RP				6.46E-01	8.93E-01	0.04	5.06E-01	-0.17	8.18E-02	-0.45	4.81E-01	-0.17
415.3303@0.8314501	415.3303	0.83	POS	hilic				4.44E-02	4.10E-01	-0.23	4.84E-02	0.60	3.42E-02	0.55	6.23E-01	-0.14
415.3573@11.930732	415.3573	11.93	POS	RP				2.81E-01	2.06E-01	-0.44	2.28E-01	0.33	1.89E-01	0.33	9.13E-01	-0.03
416.103@10.569416	416.1030	10.57	NEG	RP				4.56E-02	1.10E-01	-0.26	2.83E-02	0.59	2.88E-01	0.22	2.41E-01	0.25
416.1223@10.781991	416.1223	10.78	NEG	RP				1.31E-02	3.30E-01	-0.26	5.18E-03	0.81	2.07E-02	0.60	5.34E-01	0.17
416.1411@10.570193	416.1411	10.57	NEG	RP				5.36E-01	3.26E-01	-0.27	4.10E-01	0.25	8.61E-01	0.05	2.31E-01	0.34
416.2062@7.262893	416.2062	7.26	NEG	RP				5.97E-01	6.63E-01	0.10	1.66E-01	-0.31	5.91E-01	-0.14	5.57E-01	0.16
416.2134@10.570901	416.2134	10.57	NEG	RP				1.68E-01	1.45E-01	-0.36	1.11E-01	0.46	3.32E-01	0.27	7.31E-01	-0.10
416.2232@8.336346	416.2232	8.34	NEG	RP				9.87E-02	9.82E-01	0.00	1.76E-02	0.48	7.72E-02	0.44	6.81E-01	-0.08
416.3249@10.333461	416.3249	10.33	NEG	RP				9.04E-01	4.98E-01	-0.18	7.42E-01	-0.09	3.21E-01	-0.26	2.67E-01	-0.25
416.3289@9.735581	416.3289	9.74	POS	RP				7.64E-01	3.87E-01	0.31	8.81E-01	-0.04	1.65E-01	0.37	5.48E-01	0.19
416.3613@10.609633	416.3613	10.61	POS	RP				6.53E-01	1.56E-01	-0.35	1.15E-01	-0.47	1.11E-01	-0.44	1.79E-01	-0.44
416.3613@10.651625	416.3613	10.65	POS	RP				6.02E-01	1.75E-01	-0.31	8.85E-02	-0.52	7.83E-02	-0.48	2.13E-01	-0.41
417.0813@10.723675	417.0813	10.72	NEG	RP				8.05E-01	8.04E-01	-0.09	4.66E-01	-0.20	3.22E-01	0.26	8.18E-01	-0.07
417.149@10.784229	417.1490	10.78	NEG	RP				9.70E-02	4.57E-01	-0.22	2.85E-02	0.64	2.45E-02	0.55	4.79E-01	0.22
417.1928@10.432183	417.1928	10.43	NEG	RP				5.37E-02	5.58E-03	-0.49	2.06E-01	0.31	3.13E-01	0.24	4.53E-01	-0.18
417.2@0.9557334	417.2000	0.96	POS	hilic				9.92E-01	9.81E-01	0.01	9.38E-01	0.02	6.01E-01	0.12	8.62E-01	-0.06
418.1192@10.78233	418.1192	10.78	NEG	RP				3.15E-02	2.39E-01	-0.30	1.43E-02	0.70	6.60E-02	0.48	6.92E-01	0.10
418.1381@11.045997	418.1381	11.05	NEG	RP				3.78E-01	1.02E-02	0.71	8.27E-02	0.54	1.41E-01	0.43	2.80E-01	0.32
418.1422@10.57013	418.1422	10.57	NEG	RP				5.15E-01	2.29E-01	-0.36	5.20E-01	0.20	3.44E-01	0.26	8.47E-01	0.06
418.1536@10.78389	418.1536	10.78	NEG	RP				3.24E-01	6.67E-01	-0.10	1.23E-01	0.39	1.77E-01	0.35	5.66E-01	-0.14
418.185@10.867359	418.1850	10.87	NEG	RP				5.93E-01	7.97E-02	0.55	4.19E-01	0.22	1.36E-01	0.48	4.44E-01	0.20
418.2288@10.782525	418.2288	10.78	NEG	RP				3.99E-02	6.26E-03	-0.73	9.23E-01	0.03	2.02E-01	0.36	2.08E-01	-0.35
418.3089@11.233485	418.3089	11.23	POS	RP				9.12E-01	8.21E-01	0.06	5.64E-01	0.19	6.07E-01	-0.15	6.08E-01	0.15
419.1358@10.322367	419.1358	10.32	NEG	RP				7.76E-01	6.64E-01	-0.10	2.54E-01	0.29	4.70E-01	0.16	7.61E-01	0.08
419.1645@11.053592	419.1645	11.05	NEG	RP				6.09E-01	2.01E-01	0.43	3.80E-01	0.28	9.33E-02	0.47	7.17E-02	0.52
419.1725@10.528052	419.1725	10.53	NEG	RP				8.48E-01	9.09E-01	-0.03	4.20E-01	0.20	3.36E-01	0.25	3.07E-01	0.23
419.2078@10.707346	419.2078	10.71	NEG	RP				7.36E-02	2.97E-02	-0.50	7.41E-01	0.08	6.15E-01	0.15	1.53E-02	-0.54

Supplementary table 2: Metabolomics analysis results

								ANOVA	Anorexia vs control		overweight vs control		obese vs control		athletes vs control	
Compound	Mass	Retention Time	Ionization mode	column	Compound_ID	MSMS_spectra	ID_level	P-FDR	P	Estimate	P	Estimate	P	Estimate	P	Estimate
419.326@10.247469	419.3260	10.25	POS	RP				1.27E-01	1.10E-02	-0.56	1.64E-01	0.37	3.47E-01	0.29	7.90E-01	-0.08
419.8771@4.4963274	419.8771	4.50	POS	hilic				9.17E-01	6.63E-01	0.14	7.91E-01	-0.07	4.01E-01	0.25	6.27E-01	0.13
419.8997@15.281598	419.8997	15.28	NEG	RP				3.68E-01	5.62E-01	0.18	1.28E-01	-0.46	7.03E-01	0.10	2.41E-01	-0.36
420.0662@0.5029694	420.0662	0.50	NEG	hilic				1.47E-01	4.92E-02	0.74	2.80E-01	0.25	5.35E-01	-0.17	3.50E-01	0.24
420.1357@11.044926	420.1357	11.04	NEG	RP				5.96E-01	5.06E-02	0.54	1.53E-01	0.44	3.46E-01	0.29	1.01E-01	0.46
420.1683@11.054812	420.1683	11.05	NEG	RP				9.32E-01	8.46E-01	0.06	7.81E-01	0.08	4.73E-01	-0.19	6.49E-01	0.13
420.2446@11.030022	420.2446	11.03	POS	RP				3.56E-01	5.69E-02	0.59	3.48E-02	0.63	6.34E-02	0.55	9.98E-02	0.53
420.2459@11.03677	420.2459	11.04	NEG	RP				2.86E-01	1.92E-02	-0.58	3.20E-01	-0.36	9.48E-01	-0.02	1.91E-02	-0.63
420.3227@11.019043	420.3227	11.02	POS	RP				6.67E-01	7.88E-01	0.06	1.13E-01	0.39	9.37E-01	-0.02	6.52E-01	0.11
421.0855@3.439357	421.0855	3.44	NEG	RP				7.37E-05	1.79E-03	0.91	3.27E-01	-0.05	7.60E-01	0.03	2.08E-01	0.18
421.144@10.433112	421.1440	10.43	NEG	RP				2.00E-03	7.34E-02	-0.46	1.01E-02	0.71	6.50E-03	0.67	8.60E-01	-0.05
421.1915@0.52928245	421.1915	0.53	NEG	hilic				5.49E-01	9.43E-01	0.02	9.67E-02	0.48	8.00E-01	0.08	1.43E-01	0.37
421.3189@8.5996475	421.3189	8.60	POS	RP				4.81E-01	1.58E-01	0.48	9.14E-01	-0.03	7.69E-01	0.08	1.31E-01	0.46
422.1688@11.052761	422.1688	11.05	NEG	RP				7.44E-01	4.96E-01	0.20	4.80E-01	-0.19	5.75E-01	-0.15	2.66E-01	-0.27
422.2454@11.029937	422.2454	11.03	POS	RP				4.57E-01	8.69E-02	0.53	5.50E-02	0.56	9.29E-02	0.49	1.16E-01	0.53
422.3194@10.903581	422.3194	10.90	POS	RP				9.76E-01	8.55E-01	-0.06	4.74E-01	-0.19	7.95E-01	-0.07	4.79E-01	-0.18
423.1593@10.707839	423.1593	10.71	NEG	RP				3.22E-01	3.65E-02	0.66	2.44E-02	0.65	7.18E-02	0.53	1.92E-01	0.39
423.275@10.672728	423.2750	10.67	POS	RP				8.52E-01	9.87E-01	0.00	3.33E-01	-0.28	3.17E-01	-0.28	4.23E-01	-0.27
423.2756@10.606431	423.2756	10.61	POS	RP				8.09E-01	2.32E-01	-0.31	2.59E-01	-0.32	2.33E-01	-0.35	2.44E-01	-0.38
423.2992@0.520206	423.2992	0.52	POS	hilic				7.98E-01	5.71E-01	-0.13	1.69E-01	-0.27	8.93E-01	0.03	8.50E-01	-0.05
423.3354@8.852931	423.3354	8.85	POS	RP	Acylcarnitine 18:2	10 eV: 424.3422 (100), 85.0287 (10)	2	4.07E-01	6.04E-01	0.17	3.81E-01	-0.24	9.17E-01	0.03	1.33E-01	0.50
423.3373@0.64914584	423.3373	0.65	POS	hilic				6.43E-01	4.50E-01	0.26	5.22E-01	-0.17	8.01E-01	0.07	2.44E-01	0.38
423.3565@10.180918	423.3565	10.18	POS	RP				6.13E-01	9.13E-01	-0.03	9.78E-01	0.01	6.36E-02	0.51	9.65E-01	0.01
423.9676@6.478499	423.9676	6.48	NEG	hilic				4.92E-01	1.46E-01	0.42	1.71E-01	0.34	8.97E-01	-0.03	1.34E-01	0.40
424.1538@10.354418	424.1538	10.35	NEG	RP				3.65E-01	2.87E-01	0.20	6.43E-02	0.43	2.79E-02	0.49	4.98E-02	0.45
424.1661@11.044981	424.1661	11.04	NEG	RP				5.89E-01	2.11E-01	0.42	7.40E-01	0.10	4.90E-01	-0.19	2.84E-01	0.27
424.2414@11.029056	424.2414	11.03	POS	RP				6.03E-01	1.41E-01	0.44	9.39E-02	0.53	1.55E-01	0.43	2.15E-01	0.40
424.7659@0.4605001	424.7659	0.46	POS	hilic				9.39E-01	9.82E-01	0.01	3.61E-01	-0.24	7.27E-01	-0.10	6.50E-01	-0.13
425.1642@10.587944	425.1642	10.59	NEG	RP				6.45E-01	8.08E-01	0.08	1.30E-01	0.42	7.89E-01	0.07	7.13E-01	-0.10
425.227@0.4602895	425.2270	0.46	POS	hilic				8.37E-01	3.60E-01	-0.24	9.33E-01	-0.02	7.12E-01	0.11	5.95E-01	0.15
425.3142@0.52089185	425.3142	0.52	POS	hilic				7.92E-01	6.38E-01	-0.11	3.82E-01	-0.19	6.66E-01	0.10	6.66E-01	0.11
425.3144@8.9748745	425.3144	8.97	POS	RP				6.14E-02	8.66E-01	-0.04	8.99E-01	-0.03	3.02E-01	0.26	1.04E-02	0.80
425.3511@9.119193	425.3511	9.12	POS	RP	Acylcarnitine 18:1	20 eV: 85.0281 (100), 426.3604 (87)	2	6.94E-01	4.63E-01	0.28	1.27E-01	0.42	7.73E-02	0.50	3.37E-01	0.29
425.3516@0.6373844	425.3516	0.64	POS	hilic				7.98E-01	4.09E-01	0.32	2.47E-01	0.31	1.22E-01	0.43	4.66E-01	0.21
425.8489@15.278794	425.8489	15.28	NEG	RP				2.36E-01	2.44E-01	-0.43	4.86E-01	-0.18	1.38E-01	0.42	6.48E-01	0.12
425.9725@8.199162	425.9725	8.20	NEG	RP				8.12E-01	1.47E-01	-0.45	7.91E-01	-0.08	8.33E-01	-0.07	7.20E-01	-0.12
426.0708@5.256354	426.0708	5.26	NEG	hilic				5.48E-02	2.95E-03	-0.78	3.66E-01	0.27	5.39E-01	-0.18	2.03E-01	-0.30
426.1238@7.1932673	426.1238	7.19	NEG	RP				1.40E-01	1.99E-03	-0.76	9.55E-01	0.02	9.23E-01	0.03	2.08E-01	-0.35
426.1243@0.5656071	426.1243	0.57	NEG	hilic				5.63E-01	3.78E-01	-0.22	6.59E-01	-0.12	4.25E-01	-0.18	2.36E-01	0.29
426.1551@10.353953	426.1551	10.35	NEG	RP				8.96E-01	6.09E-01	-0.13	6.53E-01	0.12	4.78E-01	0.21	8.16E-01	0.06
426.1696@10.572593	426.1696	10.57	NEG	RP				4.01E-01	9.01E-03	-0.63	9.36E-01	-0.02	7.33E-01	-0.10	8.84E-01	0.04
426.278@10.859667	426.2780	10.86	POS	RP				7.50E-01	5.60E-01	0.13	9.30E-01	0.02	5.78E-01	-0.10	3.05E-01	0.26
426.3499@10.481858	426.3499	10.48	POS	RP				1.99E-01	2.83E-01	0.23	3.80E-02	0.55	3.78E-03	0.65	2.64E-01	0.25
426.3507@10.756963	426.3507	10.76	POS	RP				1.39E-01	3.22E-01	0.29	7.91E-02	0.48	6.93E-03	0.87	6.26E-01	0.14
426.37@11.514261	426.3700	11.51	POS	RP				4.76E-01	4.35E-01	-0.21	5.25E-01	0.16	3.40E-01	0.27	2.58E-01	-0.26
426.4068@13.455421	426.4068	13.46	POS	RP				4.67E-01	6.32E-01	0.13	1.86E-01	0.38	4.06E-01	-0.22	4.81E-01	-0.23
427.11@10.712	427.1100	10.71	NEG	RP				4.46E-01	8.98E-02	0.52	3.89E-02	0.60	2.38E-01	0.37	7.33E-02	0.54
427.1486@10.866916	427.1486	10.87	NEG	RP				8.25E-01	3.51E-01	0.27	6.34E-01	-0.12	6.70E-01	0.13	4.17E-01	0.22
427.1771@10.43294	427.1771	10.43	NEG	RP				7.07E-02	1.37E-01	-0.27	1.03E-01	0.39	4.41E-02	0.54	8.33E-01	0.05
427.18@10.78315	427.1800	10.78	NEG	RP				6.10E-01	7.77E-01	-0.06	3.45E-01	-0.21	6.28E-01	0.12	2.81E-01	0.27
427.2433@0.42414513	427.2433	0.42	POS	hilic				9.84E-01	6.53E-01	-0.12	7.33E-01	0.08	9.28E-01	0.02	9.62E-01	0.01
427.2692@11.422182	427.2692	11.42	POS	RP				7.45E-01	7.99E-01	0.06	3.42E-01	0.22	1.41E-01	0.38	2.80E-01	0.28
427.3657@0.63066345	427.3657	0.63	POS	hilic				6.63E-01	1.39E-01	0.47	5.28E-01	0.18	3.15E-01	0.25	8.65E-01	-0.04
427.3665@9.392909	427.3665	9.39	POS	RP	Acylcarnitine 18:0	10 eV: 428.3750 (100), 85.0285 (20)	1	8.35E-01	6.22E-01	0.16	2.29E-01	0.33	9.01E-01	0.04	8.13E-01	-0.07
427.9747@8.396924	427.9747	8.40	NEG	RP				7.01E-01	9.66E-01	-0.01	1.52E-01	-0.32	4.84E-01	0.20	6.67E-01	0.14
427.9749@0.49432778	427.9749	0.49	NEG	hilic				1.60E-02	1.56E-01	-0.44	1.16E-01	0.41	1.13E-02	0.74	6.85E-01	-0.11
427.9886@7.644209	427.9886	7.64	NEG	RP				4.96E-01	1.99E-01	0.35	2.25E-01	0.35	4.84E-01	-0.21	2.97E-01	0.30
428.0517@5.038278	428.0517	5.04	POS	hilic				5.23E-01	8.67E-01	-0.04	2.96E-01	0.25	8.03E-02	0.51	4.38E-01	0.21
428.0519@5.186969	428.0519	5.19	POS	hilic				4.05E-01	4.37E-01	0.20	1.81E-01	0.34	5.59E-01	-0.12	8.82E-02	0.46
428.1518@10.354544	428.1518	10.35	NEG	RP				4.54E-01	1.96E-01	-0.30	5.19E-01	0.17	2.85E-01	0.28	4.36E-01	0.19
428.1699@10.574655	428.1699	10.57	NEG	RP				4.67E-01	1.34E-01	-0.39	8.04E-01	0.08	2.64E-01	0.33	8.52E-01	-0.05
428.186@10.791649	428.1860	10.79	NEG	RP				3.68E-02	2.04E-01	-0.35	2.52E-01	0.36	1.63E-03	0.75	9.12E-01	-0.03
428.2222@7.3779035	428.2222	7.38	NEG	RP				8.43E-05	1.35E-01	-0.29	1.65E-01	0.30	4.73E-05	1.14	9.60E-01	-0.01
428.2352@10.924196	428.2352	10.92	POS	RP				9.35E-01	4.77E-01	0.21	7.75E-01	-0.10	7.68E-01	-0.09	9.19E-01	-0.03
428.2418@11.030376	428.2418	11.03	POS	RP				3.96E-01	6.75E-02	0.54	1.71E-01	0.41	1.14E-02	0.61	8.85E-02	0.42
428.3293@11.032287	428.3293	11.03	NEG	RP				9.06E-01	7.31E-01	-0.09	5.09E-01	-0.19	1.84E-01	-0.32	6.68E-01	-0.12
428.3653@12.920095	428.3653	12.92	POS	RP				5.87E-01	2.43E-01	0.37	7.84E-01	-0.09	3.31E-01	-0.30	8.05E-01	-0.07
428.3654@10.755881	428.3654	10.76	POS	RP				5.04E-01	3.75E-01	0.26	4.95E-01	0.20	4.49E-02	0.63	7.57E-01	0.08
428.3659@11.914921	428.3659	11.91	POS	RP				9.82E-01	9.86E-01	0.00	5.76E-01	0.14	6.03E-01	0.12	9.22E-01	-0.03
428.366@11.609345	428.3660	11.61	POS	RP				6.42E-01	1.94E-01	0.43	6.90E-01	0.10	5.07E-01	-0.20	7.58E-01	0.10
428.3666@11.215149	428.3666	11.22	POS	RP				8.19E-01	9.51E-01	0.02	2.97E-01	-0.24	2.86E-01	-0.24	8.88E-01	-0.04
428.397@10.626045	428.3970	10.63	POS	RP				9.06E-01	2.58E-01	0.32	6.04E-01	0.16	4.12E-01	0.23	9.27E-01	0.03
429.1937@10.708373	429.1937	10.71	NEG	RP				3.09E-01	5.95E-01	0.17	3.73E-02	0.57	6.35E-03	0.68	3.22E-01	0.29

Supplementary table 2: Metabolomics analysis results

Compound	Mass	Retention Time	Ionization mode	column	Compound_ID	MSMS_spectra	ID_level	ANOVA P-FDR	Anorexia vs control P	Anorexia vs control Estimate	overweight vs control P	overweight vs control Estimate	obese vs control P	obese vs control Estimate	athletes vs control P	athletes vs control Estimate
429.1955@11.043001	429.1955	11.04	NEG	RP				6.95E-01	9.21E-02	0.45	3.62E-01	0.27	4.91E-01	0.20	1.22E-01	0.46
429.2368@3.928277	429.2368	3.93	POS	RP				9.40E-01	6.98E-01	-0.10	4.04E-01	-0.20	9.15E-01	0.03	9.70E-01	-0.01
429.2375@0.44636354	429.2375	0.45	POS	hilic				9.70E-01	8.68E-01	-0.04	4.53E-01	-0.19	9.95E-01	0.00	8.64E-01	-0.05
429.2683@11.421691	429.2683	11.42	POS	RP				6.39E-01	3.04E-01	0.32	1.32E-01	0.44	1.41E-01	0.45	1.22E-01	0.47
429.2882@8.425708	429.2882	8.43	POS	RP				4.48E-01	1.18E-01	0.49	2.21E-01	0.33	9.51E-01	0.01	8.68E-02	0.43
429.3714@0.41098025	429.3714	0.41	POS	hilic				6.90E-01	2.94E-01	-0.32	1.80E-01	-0.39	2.71E-01	-0.31	9.70E-01	0.01
429.3719@12.272297	429.3719	12.27	POS	RP				8.81E-01	3.08E-01	0.29	3.32E-01	0.23	3.32E-01	0.23	5.25E-01	0.14
429.3823@9.254697	429.3823	9.25	POS	RP				2.02E-01	3.97E-01	0.23	1.57E-01	-0.39	6.47E-01	-0.13	5.08E-02	-0.63
429.9069@4.3499994	429.9069	4.35	POS	hilic				5.79E-01	6.66E-02	0.48	9.47E-02	0.47	7.16E-01	0.11	8.11E-01	0.07
430.1681@10.575163	430.1681	10.58	NEG	RP				8.42E-01	2.08E-01	-0.30	8.17E-01	-0.06	7.16E-01	-0.11	2.79E-01	-0.32
430.1725@0.40624768	430.1725	0.41	POS	hilic				6.46E-01	2.01E-01	0.35	9.03E-02	0.46	1.40E-01	0.42	7.54E-01	0.09
430.1725@11.673395	430.1725	11.67	POS	RP				2.89E-01	2.11E-01	0.35	1.31E-02	0.70	9.80E-01	-0.01	4.75E-01	0.21
430.1876@10.799001	430.1876	10.80	NEG	RP				1.68E-01	4.74E-01	-0.21	4.99E-01	0.21	3.59E-03	0.71	3.49E-01	0.26
430.2002@11.049487	430.2002	11.05	NEG	RP				4.06E-01	2.59E-02	0.66	1.47E-01	0.47	9.08E-02	0.48	4.73E-02	0.55
430.3081@9.633554	430.3081	9.63	NEG	RP				1.00E-01	2.88E-02	-0.75	9.99E-01	0.00	5.79E-01	0.15	4.74E-02	-0.48
430.3082@0.4540887	430.3082	0.45	POS	hilic				8.34E-01	8.99E-01	0.04	8.98E-01	-0.04	2.14E-01	0.36	6.18E-01	0.14
430.3084@9.62946	430.3084	9.63	POS	RP				2.50E-01	2.00E-01	-0.43	5.95E-01	0.16	1.63E-01	0.37	2.49E-01	-0.29
430.3089@9.62858	430.3089	9.63	POS	RP				2.36E-01	3.03E-02	-0.70	8.96E-01	-0.04	7.24E-01	-0.10	4.85E-02	-0.52
430.3456@11.932347	430.3456	11.93	POS	RP				1.85E-01	7.97E-02	-0.58	5.54E-01	0.17	2.86E-01	0.30	4.33E-01	-0.22
430.3766@10.664798	430.3766	10.66	POS	RP				5.84E-01	2.61E-01	-0.26	9.42E-02	-0.47	5.35E-02	-0.55	2.63E-01	-0.38
430.3772@10.603586	430.3772	10.60	POS	RP				7.66E-01	2.71E-01	-0.28	2.21E-01	-0.36	1.27E-01	-0.45	3.24E-01	-0.33
430.3781@10.705739	430.3781	10.71	POS	RP				5.89E-01	5.06E-01	0.17	2.54E-01	-0.39	6.18E-01	-0.14	6.37E-01	0.15
430.3803@12.270477	430.3803	12.27	POS	RP				5.51E-01	6.28E-01	-0.14	7.05E-01	-0.12	9.94E-01	0.00	3.40E-02	-0.58
431.0972@10.867054	431.0972	10.87	NEG	RP				6.92E-01	2.02E-01	0.40	1.08E-01	0.41	2.71E-01	0.34	8.24E-01	0.06
431.1728@10.432329	431.1728	10.43	NEG	RP				5.73E-02	2.65E-01	-0.29	1.82E-02	0.70	4.93E-01	0.19	9.78E-01	-0.01
431.2314@8.369916	431.2314	8.37	POS	RP				7.36E-04	1.24E-03	-0.80	9.29E-02	-0.40	2.61E-03	-0.69	1.64E-08	-1.08
431.3034@1.0102588	431.3034	1.01	POS	hilic				5.80E-01	1.10E-01	0.52	1.26E-01	0.46	7.35E-01	0.09	4.25E-01	0.25
431.304@7.936475	431.3040	7.94	POS	RP				3.64E-01	8.81E-01	0.04	3.76E-02	0.57	1.04E-01	0.45	4.65E-01	0.17
431.304@9.076146	431.3040	9.08	POS	RP				8.86E-01	4.70E-01	0.25	4.73E-01	0.20	7.24E-01	-0.09	9.03E-01	0.03
431.3041@8.92546	431.3041	8.93	POS	RP				7.43E-01	1.86E-01	0.42	2.61E-01	0.33	8.86E-01	0.04	4.14E-01	0.26
431.3257@10.99174	431.3257	10.99	POS	RP				4.71E-01	8.93E-03	0.68	4.28E-01	0.21	1.47E-01	0.37	4.61E-01	0.24
431.9805@8.398797	431.9805	8.40	NEG	RP				2.49E-01	1.84E-02	-0.72	8.17E-02	-0.61	2.27E-01	-0.34	2.70E-02	-0.63
432.2011@11.068623	432.2011	11.07	NEG	RP				2.36E-01	1.73E-02	0.73	3.51E-02	0.64	7.36E-02	0.54	3.51E-02	0.64
432.3223@0.4670217	432.3223	0.47	POS	hilic				4.69E-01	1.35E-01	0.41	1.31E-01	0.36	5.71E-01	0.14	7.96E-01	-0.05
432.323@9.529796	432.3230	9.53	NEG	RP				1.61E-02	2.14E-02	-0.64	8.64E-01	0.04	1.15E-01	0.45	5.76E-02	-0.46
432.3234@0.4527712	432.3234	0.45	NEG	hilic				7.36E-04	2.70E-02	-0.49	3.54E-02	0.54	7.89E-03	0.76	3.79E-01	-0.21
432.3247@11.332106	432.3247	11.33	POS	RP				7.76E-01	4.09E-01	-0.27	5.54E-01	0.18	9.02E-01	0.03	4.84E-01	-0.21
433.0666@6.0787888	433.0666	6.08	NEG	hilic				6.95E-01	6.17E-01	0.15	8.43E-01	0.06	1.26E-01	-0.39	9.98E-01	0.00
433.1879@10.707071	433.1879	10.71	NEG	RP				5.22E-01	9.66E-02	0.52	1.01E-01	0.54	9.67E-02	0.49	2.38E-01	0.36
433.1894@12.381913	433.1894	12.38	NEG	RP				7.47E-01	2.00E-01	-0.40	8.97E-01	-0.04	9.78E-01	0.01	6.37E-01	0.14
434.1985@11.066879	434.1985	11.07	NEG	RP				5.01E-01	4.74E-02	0.57	2.73E-01	0.34	2.86E-01	0.32	7.18E-02	0.58
434.2277@9.551441	434.2277	9.55	POS	RP				8.56E-03	7.36E-02	-0.55	3.52E-01	0.23	5.64E-03	0.78	6.66E-01	-0.12
434.228@0.79709995	434.2280	0.80	POS	hilic				2.32E-02	2.93E-02	-0.67	8.07E-01	0.06	4.45E-02	0.57	5.28E-01	-0.17
434.2611@11.212786	434.2611	11.21	POS	RP				6.40E-01	3.01E-01	-0.30	3.43E-01	-0.25	4.67E-01	0.20	6.16E-01	-0.14
435.1065@5.6871734	435.1065	5.69	POS	hilic				7.97E-01	6.16E-01	0.14	4.52E-01	-0.18	5.21E-01	0.16	5.25E-01	0.18
435.1173@10.3516035	435.1173	10.35	NEG	RP				5.29E-01	2.58E-01	-0.24	2.10E-01	0.31	5.30E-01	0.16	9.63E-01	-0.01
435.3352@10.908793	435.3352	10.91	POS	RP				5.33E-01	5.52E-01	0.15	9.36E-01	-0.03	8.99E-01	-0.04	4.84E-02	0.54
435.8758@7.9220934	435.8758	7.92	NEG	hilic				9.07E-01	6.77E-01	0.11	7.46E-01	0.08	2.67E-01	0.30	7.61E-01	0.08
435.8761@15.283128	435.8761	15.28	NEG	RP				8.66E-01	6.42E-01	0.16	6.32E-01	-0.13	8.83E-01	-0.04	3.38E-01	-0.26
435.979@6.247496	435.9790	6.25	NEG	hilic				8.09E-01	1.96E-01	0.38	7.88E-01	0.08	2.29E-01	0.36	5.24E-01	0.18
436.0994@5.271544	436.0994	5.27	NEG	hilic				1.02E-03	2.46E-06	-1.07	2.21E-01	0.33	8.94E-01	0.04	4.15E-02	-0.51
436.0994@5.271982	436.0994	5.27	NEG	hilic				6.90E-05	3.51E-06	-1.13	7.17E-02	0.46	4.92E-01	0.17	2.96E-02	-0.58
436.1519@0.56549054	436.1519	0.57	NEG	hilic				4.58E-01	1.57E-02	-0.50	8.65E-01	0.04	5.37E-01	-0.16	9.64E-01	0.01
436.152@7.1909385	436.1520	7.19	NEG	RP				1.36E-01	3.28E-02	-0.62	2.37E-01	0.33	6.85E-01	0.12	4.01E-01	-0.23
436.4034@11.073354	436.4034	11.07	POS	RP				7.64E-01	2.78E-01	-0.36	7.93E-01	0.08	7.77E-01	0.09	5.69E-01	-0.17
437.1317@10.569	437.1317	10.57	NEG	RP				2.26E-02	1.72E-02	-0.72	2.26E-01	0.37	1.23E-01	0.40	8.13E-01	-0.07
437.179@10.869975	437.1790	10.87	NEG	RP				6.51E-01	7.64E-01	0.08	2.43E-01	-0.31	6.96E-01	0.12	3.32E-01	-0.25
437.2899@10.300301	437.2899	10.30	NEG	RP				1.02E-01	5.86E-02	0.56	1.30E-01	-0.44	4.76E-01	-0.17	5.04E-01	0.21
437.2919@10.297492	437.2919	10.30	POS	RP				2.08E-01	7.55E-02	0.50	1.69E-01	-0.39	7.74E-01	-0.07	5.08E-01	0.23
437.2924@1.6286448	437.2924	1.63	POS	hilic				8.34E-01	5.42E-01	0.18	3.94E-01	-0.22	9.62E-01	-0.01	7.30E-01	0.12
438.0611@6.422768	438.0611	6.42	NEG	RP				1.86E-01	9.73E-01	-0.01	1.38E-01	-0.35	7.51E-03	-0.62	1.02E-01	-0.39
438.0619@0.44755808	438.0619	0.45	NEG	hilic				2.64E-01	7.84E-01	-0.07	7.96E-02	-0.42	2.56E-02	-0.54	1.10E-01	-0.39
438.1683@7.9479666	438.1683	7.95	NEG	RP				5.25E-01	3.85E-02	-0.51	4.26E-01	-0.21	8.07E-01	-0.07	7.02E-01	0.11
438.2675@11.421735	438.2675	11.42	POS	RP				3.91E-01	2.04E-01	0.42	4.80E-02	0.53	2.00E-02	0.69	1.50E-01	0.44
438.2965@8.966178	438.2965	8.97	NEG	RP				2.53E-01	9.55E-01	0.01	1.78E-01	0.30	2.24E-02	0.58	5.75E-01	0.11
438.2974@9.510269	438.2974	9.51	NEG	RP				4.96E-02	6.45E-01	-0.10	1.29E-01	0.39	5.69E-03	0.67	2.77E-02	0.67
438.2975@8.633897	438.2975	8.63	NEG	RP				2.31E-01	8.60E-01	-0.03	1.56E-01	0.33	2.17E-02	0.54	4.52E-01	0.15
438.3703@11.393594	438.3703	11.39	NEG	RP				5.83E-01	6.11E-01	-0.12	2.27E-01	0.32	4.54E-01	-0.15	8.15E-01	0.06
438.3711@11.385284	438.3711	11.39	POS	RP				7.57E-01	4.05E-01	0.23	3.68E-01	0.28	7.11E-01	-0.09	3.35E-01	0.33
439.1468@10.783208	439.1468	10.78	NEG	RP				3.68E-02	2.73E-01	-0.33	3.14E-02	0.64	2.61E-02	0.61	8.81E-01	0.05
439.2353@11.429077	439.2353	11.43	NEG	RP				1.20E-01	3.77E-02	0.62	8.82E-01	0.03	3.38E-01	-0.21	9.98E-01	0.00
439.2708@1.5415184	439.2708	1.54	POS	hilic				3.55E-01	3.96E-02	0.55	3.15E-01	0.24	2.67E-01	0.28	5.43E-02	0.61
439.2711@8.9241705	439.2711	8.92	POS	RP				2.46E-01	5.11E-02	0.57	1.34E-01	0.35	1.50E-01	0.37	2.27E-02	0.73

Supplementary table 2: Metabolomics analysis results

Compound	Mass	Retention Time	Ionization mode	column	Compound_ID	MSMS_spectra	ID_level	ANOVA	Anorexia vs control		overweight vs control		obese vs control		athletes vs control	
								P-FDR	P	Estimate	P	Estimate	P	Estimate	P	Estimate
439.4027@0.48686957	439.4027	0.49	POS	hilic				8.81E-01	8.02E-01	-0.07	9.67E-01	-0.01	3.10E-01	0.28	7.60E-01	0.09
439.4027@9.154641	439.4027	9.15	POS	RP				7.70E-01	8.64E-01	0.06	2.33E-01	0.30	1.82E-01	0.42	3.69E-01	0.29
440.3264@1.7007676	440.3264	1.70	POS	hilic				9.70E-01	5.95E-01	0.15	9.44E-01	0.02	5.19E-01	0.17	6.32E-01	0.14
440.3519@0.43560004	440.3519	0.44	POS	hilic				6.19E-02	4.81E-03	-0.46	5.43E-01	0.14	4.11E-01	-0.16	1.57E-01	0.39
440.3849@11.681712	440.3849	11.68	NEG	RP				6.77E-01	2.35E-01	0.31	9.03E-02	0.46	2.88E-01	0.20	2.82E-01	0.28
440.3865@11.687472	440.3865	11.69	POS	RP				7.84E-01	5.13E-01	0.18	1.78E-01	0.44	3.82E-01	0.24	2.68E-01	0.32
440.9899@5.2785063	440.9899	5.28	POS	hilic				5.51E-01	1.52E-01	0.45	2.24E-01	0.28	3.62E-02	0.52	3.16E-01	0.23
441.0818@10.570444	441.0818	10.57	NEG	RP				1.28E-02	6.96E-03	-0.85	3.93E-01	0.25	1.51E-01	0.37	6.22E-01	-0.15
441.1545@0.54228055	441.1545	0.54	POS	hilic				1.66E-01	6.78E-01	-0.12	7.44E-02	-0.49	5.72E-03	-0.71	7.94E-01	-0.07
441.1575@10.431723	441.1575	10.43	NEG	RP				1.80E-03	1.09E-01	-0.43	4.04E-03	0.79	8.63E-03	0.66	8.97E-01	0.04
441.1621@11.0569935	441.1621	11.06	NEG	RP				5.36E-01	1.30E-01	0.53	1.20E-01	0.47	5.93E-02	0.52	1.28E-01	0.44
441.2309@11.429619	441.2309	11.43	NEG	RP				5.28E-01	7.13E-01	0.10	1.91E-01	0.37	8.29E-02	0.50	8.13E-02	0.48
441.3457@0.79226834	441.3457	0.79	POS	hilic				1.70E-01	1.83E-01	0.26	1.92E-02	0.60	5.10E-01	0.12	2.87E-02	0.50
441.8235@15.288387	441.8235	15.29	NEG	RP				3.38E-01	1.67E-01	0.47	2.83E-01	-0.28	7.06E-01	-0.10	4.54E-01	-0.23
441.8952@7.932422	441.8952	7.93	NEG	hilic				9.81E-01	7.13E-01	-0.10	7.35E-01	-0.09	8.26E-01	0.07	6.82E-01	-0.12
442.1159@0.4066991	442.1159	0.41	NEG	hilic				6.26E-03	5.74E-04	0.99	2.87E-01	-0.25	6.70E-01	0.11	1.23E-01	0.47
442.1271@10.320501	442.1271	10.32	NEG	RP				6.65E-01	7.08E-01	0.09	4.75E-01	0.19	1.04E-01	0.45	7.81E-01	0.06
442.2396@0.5831042	442.2396	0.58	POS	hilic				7.60E-01	5.88E-01	0.14	3.31E-01	-0.20	8.56E-01	0.05	4.86E-01	0.18
442.2416@4.5234394	442.2416	4.52	POS	RP				6.08E-01	2.60E-01	-0.32	1.26E-01	-0.46	9.93E-02	-0.52	1.38E-01	-0.43
442.3846@14.426626	442.3846	14.43	POS	RP				2.09E-01	8.39E-01	0.06	1.68E-01	0.39	1.13E-01	-0.45	3.46E-01	-0.29
442.8333@7.8803587	442.8333	7.88	POS	hilic				7.75E-01	1.10E-01	0.38	4.28E-01	0.16	3.50E-01	0.24	3.70E-01	0.21
443.097@10.784952	443.0970	10.78	NEG	RP				2.18E-02	6.71E-02	-0.56	8.86E-02	0.48	3.45E-02	0.57	9.78E-01	0.01
443.1738@10.708633	443.1738	10.71	NEG	RP				2.43E-01	5.53E-02	0.59	3.02E-01	0.33	4.97E-02	0.59	3.46E-03	0.75
443.2078@10.567674	443.2078	10.57	NEG	RP				2.94E-01	7.63E-01	-0.08	5.52E-02	0.56	1.22E-01	0.47	2.96E-01	0.29
443.2455@11.046662	443.2455	11.05	NEG	RP				7.42E-01	7.90E-01	-0.07	2.14E-01	0.37	7.82E-01	0.09	8.38E-01	-0.06
443.3612@0.7826945	443.3612	0.78	POS	hilic				1.55E-01	5.86E-02	-0.50	1.99E-01	0.37	2.71E-01	0.29	7.07E-01	0.11
443.8697@15.26891	443.8697	15.27	NEG	RP				2.85E-01	3.69E-01	-0.29	4.73E-02	-0.57	3.31E-01	-0.26	7.91E-03	-0.70
443.9008@4.6731462	443.9008	4.67	NEG	hilic				5.63E-01	4.72E-01	-0.26	2.64E-01	0.30	2.89E-01	-0.28	9.97E-01	0.00
444.1131@11.173603	444.1131	11.17	POS	RP				1.79E-01	4.25E-02	-0.61	6.12E-01	-0.16	2.64E-01	-0.34	1.79E-02	-0.80
444.1613@3.5937116	444.1613	3.59	NEG	hilic				5.24E-01	7.16E-01	-0.08	3.24E-01	0.23	1.16E-01	0.42	8.90E-01	0.03
444.1751@10.708141	444.1751	10.71	NEG	RP				6.38E-01	4.60E-02	0.52	7.59E-01	0.10	6.93E-01	0.11	2.04E-01	0.36
444.216@11.221674	444.2160	11.22	NEG	RP				8.27E-01	3.36E-01	0.26	5.72E-01	-0.11	9.85E-01	0.01	6.15E-01	0.10
444.2883@9.422585	444.2883	9.42	POS	RP				4.59E-02	9.95E-01	0.00	9.80E-02	-0.30	5.32E-02	-0.36	1.14E-01	0.42
444.3232@0.42725584	444.3232	0.43	NEG	hilic				6.47E-02	3.79E-02	-0.35	3.69E-02	0.53	3.69E-01	0.23	5.31E-01	0.14
444.3596@11.684813	444.3596	11.68	POS	RP				7.72E-01	9.59E-01	-0.02	6.81E-01	0.11	3.20E-01	-0.29	3.95E-01	-0.27
444.3603@12.275967	444.3603	12.28	POS	RP				3.27E-01	2.06E-01	-0.39	5.03E-01	-0.22	9.95E-01	0.00	9.16E-03	-0.69
444.3614@11.256999	444.3614	11.26	POS	RP				7.01E-01	4.49E-01	0.23	8.37E-01	-0.06	3.63E-01	-0.27	3.52E-01	-0.25
444.3614@11.308798	444.3614	11.31	POS	RP				2.79E-01	3.38E-01	0.29	2.71E-01	-0.34	8.61E-02	-0.47	1.82E-01	-0.37
444.3927@10.626369	444.3927	10.63	POS	RP				5.03E-01	1.03E-01	-0.45	4.79E-01	0.21	3.75E-01	-0.26	8.61E-01	-0.06
444.3929@10.625895	444.3929	10.63	POS	RP				5.85E-01	2.06E-01	-0.37	9.35E-01	0.02	9.39E-02	-0.47	8.56E-01	-0.06
444.966@1.0357	444.9660	1.04	POS	hilic				6.60E-01	8.29E-01	-0.06	6.32E-02	-0.35	2.97E-01	-0.22	6.70E-01	-0.10
445.1121@11.066325	445.1121	11.07	NEG	RP				1.25E-01	4.10E-02	-0.53	4.63E-01	-0.20	8.17E-02	0.46	3.81E-01	-0.27
445.1277@3.9635048	445.1277	3.96	POS	hilic				7.45E-01	9.61E-01	0.01	2.44E-01	0.35	3.10E-01	0.26	8.33E-01	-0.06
445.1666@10.568394	445.1666	10.57	NEG	RP				8.29E-01	1.29E-01	-0.36	7.47E-01	-0.09	3.57E-01	-0.24	7.80E-01	-0.08
445.2274@0.7275532	445.2274	0.73	POS	hilic				7.76E-01	9.18E-01	0.02	5.57E-01	0.13	2.34E-01	0.31	2.39E-01	0.28
445.3673@10.760414	445.3673	10.76	POS	RP				2.11E-01	4.46E-01	0.19	2.40E-01	0.34	1.02E-02	0.81	3.93E-01	0.20
445.9796@7.8501368	445.9796	7.85	NEG	RP				2.96E-01	3.76E-01	0.30	2.50E-01	0.31	1.54E-01	0.41	3.90E-03	0.80
445.9874@7.440351	445.9874	7.44	POS	hilic				3.24E-01	1.18E-01	0.52	5.22E-01	-0.18	2.39E-01	0.36	1.72E-01	0.38
446.0598@0.70224994	446.0598	0.70	NEG	hilic				8.23E-01	5.44E-01	0.18	5.48E-01	0.15	2.61E-01	0.37	8.72E-01	-0.05
446.111@8.434702	446.1110	8.43	NEG	RP				8.37E-01	4.81E-01	-0.22	1.32E-01	-0.41	4.32E-01	-0.20	3.88E-01	-0.29
446.1289@0.60980946	446.1289	0.61	NEG	hilic				2.28E-01	3.67E-02	-0.56	1.58E-01	-0.37	1.13E-02	-0.62	5.91E-01	-0.14
446.3036@9.891231	446.3036	9.89	POS	RP				5.71E-02	2.65E-01	0.27	5.03E-01	-0.13	1.29E-01	-0.29	7.48E-02	0.48
446.3052@0.50109994	446.3052	0.50	POS	hilic				7.27E-01	9.10E-01	0.03	8.32E-01	0.05	3.05E-01	-0.21	3.58E-01	0.24
446.3392@0.42697275	446.3392	0.43	NEG	hilic				2.32E-02	1.13E-02	-0.69	1.17E-01	0.45	5.37E-01	0.18	1.66E-01	0.35
446.3394@10.699521	446.3394	10.70	NEG	RP				7.68E-04	5.14E-04	-0.87	1.97E-02	0.57	6.16E-01	0.15	6.11E-02	0.47
446.3404@11.523129	446.3404	11.52	POS	RP				7.78E-01	3.31E-01	0.29	1.62E-01	0.44	4.67E-01	0.19	7.24E-01	0.10
446.3406@11.526952	446.3406	11.53	POS	RP				8.35E-02	3.23E-02	0.59	1.46E-03	0.96	1.62E-01	0.40	2.30E-01	0.35
446.3414@10.693482	446.3414	10.69	POS	RP				1.50E-03	1.91E-03	-0.79	1.65E-02	0.60	2.04E-01	0.39	1.74E-01	0.36
447.1099@11.066043	447.1099	11.07	NEG	RP				1.75E-01	2.13E-02	-0.60	3.17E-01	-0.28	2.87E-01	0.29	2.08E-01	-0.38
447.1593@10.567934	447.1593	10.57	NEG	RP				3.96E-02	7.23E-02	-0.56	1.21E-01	0.48	7.49E-02	0.48	7.04E-01	0.11
447.2034@10.867602	447.2034	10.87	NEG	RP				6.15E-01	4.34E-02	0.60	5.19E-01	0.17	3.98E-01	0.26	3.20E-01	0.30
447.2043@10.867536	447.2043	10.87	NEG	RP				7.55E-01	1.13E-01	0.51	4.54E-01	0.20	5.63E-01	0.19	3.33E-01	0.28
447.24@11.036676	447.2400	11.04	NEG	RP				4.16E-01	5.17E-01	0.21	6.13E-01	0.13	3.10E-02	0.67	8.96E-01	0.04
447.2987@7.538539	447.2987	7.54	POS	RP				2.00E-01	1.46E-01	0.40	4.46E-01	-0.15	2.74E-01	-0.21	4.45E-01	-0.16
447.2987@8.360515	447.2987	8.36	POS	RP				4.77E-01	1.68E-01	0.46	5.94E-01	0.15	6.16E-01	-0.12	1.84E-01	0.38
447.2987@8.400559	447.2987	8.40	POS	RP				8.34E-01	3.50E-01	0.31	4.06E-01	0.23	8.41E-01	-0.05	9.00E-01	0.04
447.2996@0.8478097	447.2996	0.85	POS	hilic				5.00E-01	3.51E-01	0.31	9.93E-01	0.00	1.85E-01	-0.34	5.48E-01	-0.16
447.3351@8.869195	447.3351	8.87	POS	RP				6.02E-01	1.23E-01	0.51	3.57E-01	0.22	5.11E-02	0.49	2.75E-01	0.37
447.9926@7.9419265	447.9926	7.94	NEG	RP				2.23E-01	4.31E-01	-0.26	5.90E-02	0.49	3.04E-01	-0.30	8.47E-01	-0.06
448.0002@0.5996766	448.0002	0.60	NEG	hilic				5.45E-01	2.33E-01	0.43	7.37E-01	-0.09	4.35E-01	-0.20	6.26E-01	0.13
448.1417@8.611973	448.1417	8.61	NEG	RP				6.33E-01	3.76E-01	0.25	4.42E-01	-0.20	4.30E-01	0.26	5.91E-01	-0.16
448.2759@10.692654	448.2759	10.69	POS	RP				6.63E-01	5.36E-01	0.18	4.00E-01	0.24	8.35E-01	-0.06	3.11E-01	-0.28
448.2759@11.42154	448.2759	11.42	POS	RP				3.25E-01	2.78E-01	0.34	2.10E-02	0.59	1.69E-02	0.70	1.10E-01	0.50

Supplementary table 2: Metabolomics analysis results

Compound	Mass	Retention Time	Ionization mode	column	Compound_ID	MSMS_spectra	ID_level	ANOVA P-FDR	Anorexia vs control P	Anorexia vs control Estimate	overweight vs control P	overweight vs control Estimate	obese vs control P	obese vs control Estimate	athletes vs control P	athletes vs control Estimate
448.3535@10.784709	448.3535	10.78	NEG	RP				2.53E-01	5.89E-01	-0.11	1.01E-01	0.45	4.12E-01	0.19	5.97E-02	0.49
448.3537@0.42669544	448.3537	0.43	NEG	hilic				7.24E-02	4.41E-02	-0.52	1.45E-01	0.42	8.15E-01	-0.07	2.29E-01	0.32
448.3601@0.4294167	448.3601	0.43	POS	hilic				7.08E-01	7.80E-01	-0.06	1.57E-01	0.34	5.67E-01	0.13	5.93E-01	0.14
449.1078@11.065359	449.1078	11.07	NEG	RP				2.29E-01	4.88E-02	-0.50	8.92E-01	-0.04	1.36E-01	0.43	5.88E-01	-0.16
449.1298@0.5479467	449.1298	0.55	NEG	hilic				9.66E-02	4.88E-01	-0.21	2.62E-01	0.29	1.87E-01	0.40	2.28E-02	-0.52
449.1746@10.782754	449.1746	10.78	NEG	RP				8.25E-02	4.03E-01	-0.25	3.60E-02	0.63	2.39E-02	0.58	6.63E-01	0.13
449.2271@0.42804167	449.2271	0.43	POS	hilic				6.90E-01	2.46E-01	0.31	8.10E-01	-0.04	2.01E-01	0.30	5.60E-01	0.15
449.3132@8.933625	449.3132	8.93	NEG	RP	Glycoursodeoxycholic acid	10 eV: 448.3073 (100), 74.0255 (10)	2	7.61E-01	2.17E-01	0.40	2.55E-01	0.34	9.78E-01	0.01	5.64E-01	0.19
449.3132@1.0704684	449.3132	1.07	NEG	hilic				7.06E-01	2.82E-01	0.35	1.36E-01	0.41	9.96E-01	0.00	6.94E-01	0.12
449.3132@9.067623	449.3132	9.07	NEG	RP				6.81E-01	2.04E-01	0.43	5.30E-01	0.19	7.11E-01	-0.10	3.57E-01	0.27
449.3137@7.9459987	449.3137	7.95	NEG	RP				3.63E-01	9.81E-01	-0.01	4.02E-02	0.54	1.69E-01	0.37	7.41E-01	0.07
449.3142@1.024783	449.3142	1.02	POS	hilic				7.61E-01	3.14E-01	0.35	4.22E-01	0.23	6.58E-01	-0.12	6.39E-01	0.14
449.3146@9.076888	449.3146	9.08	POS	RP				9.22E-01	4.84E-01	0.24	9.05E-01	0.03	6.38E-01	-0.12	9.64E-01	0.01
449.3498@9.040304	449.3498	9.04	POS	RP				3.85E-01	7.17E-02	0.60	9.61E-01	-0.01	8.95E-02	0.47	2.91E-01	0.30
449.3511@10.908891	449.3511	10.91	POS	RP				6.38E-01	4.83E-01	0.19	7.75E-01	0.09	6.95E-01	0.13	2.97E-02	0.56
449.4222@0.43776593	449.4222	0.44	POS	hilic				5.36E-01	9.70E-01	-0.01	4.96E-01	0.14	3.36E-01	0.24	8.74E-02	0.46
450.0946@6.0781274	450.0946	6.08	NEG	hilic				3.61E-01	6.23E-01	0.16	5.88E-01	-0.17	5.11E-02	-0.53	8.27E-02	-0.44
450.2228@8.796863	450.2228	8.80	POS	RP				1.86E-01	3.84E-01	-0.25	2.34E-01	0.31	1.24E-02	0.62	8.67E-01	-0.05
450.2607@1.4499857	450.2607	1.45	NEG	hilic				3.28E-01	1.15E-01	-0.33	4.02E-01	0.18	1.62E-01	0.39	9.64E-01	-0.01
450.2616@9.736852	450.2616	9.74	NEG	RP				3.02E-01	4.23E-02	-0.49	3.79E-01	0.20	3.74E-01	0.25	9.67E-01	-0.01
451.1093@10.570157	451.1093	10.57	NEG	RP				2.77E-01	6.08E-02	-0.35	2.89E-01	0.28	5.93E-01	0.12	4.54E-01	-0.18
451.1446@0.5590715	451.1446	0.56	NEG	hilic				8.34E-01	4.53E-01	-0.21	6.38E-01	-0.13	6.15E-01	0.15	4.18E-01	-0.20
451.1904@11.05271	451.1904	11.05	NEG	RP				3.60E-01	3.24E-02	0.68	7.24E-02	0.53	2.41E-01	0.34	4.62E-02	0.58
451.2091@0.49057424	451.2091	0.49	POS	hilic				7.12E-01	3.19E-01	-0.24	9.97E-01	0.00	2.21E-01	-0.28	6.60E-01	0.12
451.2431@0.4311149	451.2431	0.43	POS	hilic				7.07E-01	2.01E-01	0.38	7.21E-01	-0.09	3.74E-01	0.27	6.91E-01	0.11
451.2675@9.782114	451.2675	9.78	NEG	RP				7.28E-01	3.52E-01	0.27	6.55E-01	-0.10	5.17E-01	-0.17	7.88E-01	0.06
451.2701@2.0498838	451.2701	2.05	POS	hilic				5.33E-01	8.96E-02	0.51	9.92E-01	0.00	4.21E-01	0.22	2.94E-01	0.26
451.2708@9.780753	451.2708	9.78	POS	RP				5.04E-01	1.27E-01	0.55	7.09E-01	0.10	2.78E-01	0.35	7.33E-02	0.49
451.3858@10.594439	451.3858	10.59	POS	RP				7.00E-01	4.62E-01	0.17	6.24E-01	-0.15	2.06E-01	0.31	7.08E-01	-0.12
451.8504@15.282061	451.8504	15.28	NEG	RP				8.80E-01	9.80E-01	0.01	5.68E-01	-0.18	2.66E-01	-0.27	3.31E-01	-0.29
452.2977@11.038767	452.2977	11.04	NEG	RP				7.46E-01	8.00E-01	-0.08	7.55E-01	-0.10	8.91E-01	0.04	2.36E-01	0.38
452.2986@10.7081995	452.2986	10.71	NEG	RP				5.43E-01	5.06E-01	0.22	3.46E-01	0.26	5.89E-02	0.51	9.08E-01	-0.03
452.3365@4.851964	452.3365	4.85	POS	RP				8.96E-01	3.72E-01	0.23	7.93E-01	0.06	7.16E-01	-0.09	6.97E-01	0.10
452.3369@0.8155452	452.3369	0.82	POS	hilic				7.53E-01	8.32E-01	0.06	4.93E-01	0.15	2.69E-01	-0.30	6.80E-01	-0.13
453.1271@10.783891	453.1271	10.78	NEG	RP				1.21E-01	3.61E-01	-0.26	3.04E-02	0.63	1.07E-01	0.42	4.18E-01	0.24
453.1423@4.040143	453.1423	4.04	POS	hilic				5.20E-03	4.36E-02	0.49						
453.1881@11.048554	453.1881	11.05	NEG	RP				6.53E-01	2.87E-01	0.34	1.36E-01	0.45	3.27E-01	0.27	7.90E-02	0.51
453.1919@10.56746	453.1919	10.57	NEG	RP				8.25E-01	8.30E-01	0.05	7.28E-01	-0.08	5.85E-01	0.15	2.90E-01	0.27
453.2845@2.2097335	453.2845	2.21	NEG	hilic				1.76E-04	2.74E-03	0.87	9.37E-02	-0.36	1.10E-02	-0.67	1.44E-01	0.41
453.285@10.121674	453.2850	10.12	NEG	RP	LysoPE 16:0	20 eV: 255.2325 (100), 452.2751 (20)	2	8.57E-05	1.02E-02	0.77	7.16E-02	-0.39	3.99E-03	-0.82	8.58E-02	0.43
453.2856@10.011109	453.2856	10.01	NEG	RP				3.36E-04	2.58E-02	0.61	9.29E-02	-0.38	3.56E-03	-0.88	1.99E-01	0.36
453.2859@10.1173525	453.2859	10.12	POS	RP	LysoPE 16:0	10 eV: 454.2987 (100), 44.0491 (30), 313.2825 (25), 393.2383 (25)	1	6.23E-04	3.29E-02	0.64	3.99E-01	-0.19	1.70E-03	-0.89	2.67E-01	0.29
453.286@1.9970428	453.2860	2.00	POS	hilic				8.80E-05	2.97E-03	0.88	3.72E-02	-0.45	7.96E-03	-0.70	1.95E-01	0.36
453.2868@10.005556	453.2868	10.01	POS	RP				8.62E-04	1.60E-02	0.75	6.40E-02	-0.40	1.55E-02	-0.68	2.42E-01	0.31
453.2869@9.614759	453.2869	9.61	NEG	RP				4.89E-01	1.62E-01	0.39	7.14E-01	0.09	1.91E-01	0.35	6.43E-02	0.54
453.2871@1.741598	453.2871	1.74	POS	hilic				4.88E-05	4.91E-03	0.86	2.73E-02	-0.49	5.56E-03	-0.70	1.52E-01	0.37
453.3815@9.471057	453.3815	9.47	POS	RP				9.92E-01	7.83E-01	-0.11	8.50E-01	0.05	9.95E-01	0.00	7.74E-01	-0.09
453.9293@4.539945	453.9293	4.54	NEG	hilic				9.19E-01	5.26E-01	0.22	8.01E-01	0.07	6.24E-01	-0.14	6.42E-01	0.12
453.9293@5.413889	453.9293	5.41	NEG	hilic				1.52E-01	8.68E-02	-0.61	2.66E-02	-0.56	1.83E-02	-0.60	8.48E-01	0.06
453.9296@4.4878626	453.9296	4.49	NEG	hilic				2.70E-01	2.53E-02	0.71	9.24E-01	-0.03	7.76E-01	-0.08	6.14E-01	0.12
454.9335@4.4942183	454.9335	4.49	NEG	hilic				6.85E-01	3.32E-01	0.31	6.79E-01	0.14	6.67E-01	-0.11	9.76E-02	0.39
455.1418@11.053496	455.1418	11.05	NEG	RP				4.62E-01	9.56E-02	0.54	1.86E-01	0.42	1.38E-01	0.38	2.99E-02	0.63
455.1728@10.354239	455.1728	10.35	NEG	RP				4.97E-01	4.00E-01	-0.24	4.01E-01	0.24	1.50E-01	0.39	9.10E-01	-0.03
455.3133@0.5731249	455.3133	0.57	POS	hilic				8.96E-01	3.93E-01	-0.21	3.69E-01	-0.20	9.86E-01	0.00	8.75E-01	-0.04
455.8455@15.279589	455.8455	15.28	NEG	RP				7.24E-01	1.64E-01	-0.41	4.59E-01	-0.19	7.24E-01	-0.10	1.48E-01	-0.39
456.0599@6.0265384	456.0599	6.03	POS	hilic				6.98E-01	4.10E-01	-0.25	7.74E-01	-0.09	8.62E-02	-0.47	1.77E-01	-0.37
457.1695@10.514244	457.1695	10.51	NEG	RP				5.24E-01	3.55E-02	-0.47	9.45E-01	0.02	6.87E-01	-0.11	7.46E-01	0.09
457.1874@10.568966	457.1874	10.57	NEG	RP				2.13E-01	1.28E-01	-0.45	6.95E-01	0.13	8.17E-02	0.46	4.45E-01	0.22
457.2084@10.783192	457.2084	10.78	NEG	RP				6.29E-01	4.78E-01	-0.17	1.82E-01	0.35	5.82E-01	0.15	6.79E-01	0.10
457.2275@11.036755	457.2275	11.04	NEG	RP				7.34E-01	1.30E-01	-0.43	9.59E-01	-0.02	7.90E-01	0.07	5.47E-01	-0.17
457.2385@10.988429	457.2385	10.99	NEG	RP				7.88E-01	4.87E-01	0.18	6.09E-01	-0.09	4.21E-01	-0.13	8.84E-01	-0.03
457.2419@10.649082	457.2419	10.65	NEG	RP				1.05E-02	1.26E-02	0.80	3.05E-01	-0.26	2.00E-01	-0.30	3.27E-01	0.25
457.2421@10.654362	457.2421	10.65	NEG	RP				1.07E-02	5.71E-04	1.05	6.84E-01	-0.12	8.72E-01	0.04	1.19E-01	0.43
457.2601@0.4291698	457.2601	0.43	POS	hilic				3.24E-01	1.76E-01	0.38	3.89E-02	0.52	1.17E-01	0.39	9.30E-01	0.02
457.7625@3.069305	457.7625	3.07	POS	hilic				9.53E-01	9.75E-01	-0.01	3.85E-01	0.24	9.09E-01	0.03	6.30E-01	0.13
457.7629@3.069305	457.7629	3.07	POS	hilic				9.31E-01	7.14E-01	-0.13	4.48E-01	0.21	9.60E-01	-0.01	8.59E-01	0.05
457.797@15.295657	457.7970	15.30	NEG	RP				4.07E-01	4.77E-02	0.59	8.73E-01	-0.04	6.31E-01	0.12	2.45E-01	0.33
457.8408@7.2850227	457.8408	7.29	POS	hilic				8.41E-01	2.28E-01	0.35	8.69E-01	0.05	8.27E-01	-0.06	6.27E-01	0.15
458.234@11.428668	458.2340	11.43	NEG	RP				2.86E-01	1.31E-01	0.45	7.17E-03	0.68	6.29E-02	0.57	2.50E-01	0.29
458.2959@10.989232	458.2959	10.99	POS	RP				5.03E-01	4.31E-02	0.62	5.47E-02	0.51	2.96E-01	0.32	2.15E-01	0.39
458.3027@0.41153193	458.3027	0.41	NEG	hilic				8.93E-01	6.34E-01	-0.13	3.56E-01	-0.22	7.86E-01	0.07	9.02E-01	-0.03
458.4081@10.9244585	458.4081	10.92	POS	RP				9.92E-01	9.36E-01	-0.02	7.50E-01	-0.07	9.75E-01	-0.01	7.86E-01	0.07
459.2034@10.782709	459.2034	10.78	NEG	RP				9.83E-02	4.73E-01	-0.23	2.84E-02	0.65	2.71E-02	0.57	4.48E-01	0.23

Supplementary table 2: Metabolomics analysis results

Compound	Mass	Retention Time	Ionization mode	column	Compound_ID	MSMS_spectra	ID_level	ANOVA	Anorexia vs control		overweight vs control		obese vs control		athletes vs control	
								P-FDR	P	Estimate	P	Estimate	P	Estimate	P	Estimate
459.2451@0.4782051	459.2451	0.48	POS	hilic				4.41E-01	4.01E-01	-0.22	7.82E-02	-0.38	1.69E-01	-0.31	7.52E-01	0.07
459.2681@0.5820772	459.2681	0.58	POS	hilic				7.40E-01	9.55E-01	0.02	9.24E-02	-0.42	4.78E-01	-0.22	3.25E-01	-0.27
459.2684@4.5228043	459.2684	4.52	POS	RP				8.02E-01	4.40E-01	-0.22	2.76E-01	-0.32	2.47E-01	-0.37	1.76E-01	-0.39
459.7604@3.0711741	459.7604	3.07	POS	hilic				6.66E-01	5.76E-01	-0.16	2.21E-01	0.38	3.47E-01	0.26	7.86E-01	0.07
459.8446@15.267651	459.8446	15.27	NEG	RP				7.57E-01	9.49E-01	0.02	6.76E-01	0.14	9.95E-01	0.00	1.95E-01	-0.36
459.9618@4.696795	459.9618	4.70	POS	hilic				7.10E-01	1.03E-01	0.42	8.07E-01	-0.07	9.41E-01	-0.02	6.75E-01	0.12
460.2328@11.428686	460.2328	11.43	NEG	RP				3.27E-01	3.19E-01	0.29	9.20E-02	0.45	8.40E-03	0.77	3.35E-01	0.32
460.2795@9.571047	460.2795	9.57	NEG	RP				2.86E-01	3.51E-01	-0.19	9.23E-01	-0.02	9.53E-01	-0.01	9.24E-02	0.48
460.3556@0.4286383	460.3556	0.43	NEG	hilic				4.94E-01	1.62E-02	-0.51	1.07E-01	-0.39	4.36E-01	-0.19	2.09E-01	-0.32
461.1346@10.55832	461.1346	10.56	NEG	RP				5.96E-02	2.19E-01	-0.24	6.12E-02	0.48	1.06E-01	0.37	4.39E-01	-0.17
461.1397@11.173424	461.1397	11.17	POS	RP				3.01E-01	4.71E-01	-0.20	8.33E-01	0.06	4.34E-01	-0.21	4.56E-02	-0.69
461.219@12.383448	461.2190	12.38	NEG	RP				6.26E-01	8.61E-01	-0.05	9.47E-01	0.02	8.23E-02	-0.49	9.92E-01	0.00
461.2191@11.036623	461.2191	11.04	NEG	RP				4.22E-01	8.02E-02	0.57	5.46E-02	0.57	9.40E-02	0.48	5.27E-02	0.55
461.3505@7.241634	461.3505	7.24	POS	RP				7.83E-01	7.14E-01	-0.09	1.98E-01	-0.26	7.68E-01	0.07	8.42E-01	0.05
461.3506@0.50329787	461.3506	0.50	POS	hilic				8.22E-01	8.88E-01	-0.04	3.27E-01	-0.21	5.75E-01	0.14	7.32E-01	-0.09
462.1@3.9574277	462.1000	3.96	POS	hilic				9.72E-01	8.84E-01	-0.04	6.60E-01	0.13	6.27E-01	0.11	5.81E-01	0.16
462.2227@11.0367	462.2227	11.04	NEG	RP				3.79E-01	7.99E-02	0.58	5.11E-02	0.59	4.03E-02	0.55	5.91E-02	0.54
462.2286@11.429884	462.2286	11.43	NEG	RP				3.87E-01	9.59E-02	0.47	2.45E-02	0.65	6.14E-02	0.57	2.60E-01	0.35
462.3328@0.4373422	462.3328	0.44	POS	hilic				5.94E-02	5.62E-03	-0.45	5.08E-01	0.16	4.84E-01	-0.14	1.26E-01	0.40
462.3364@0.42896557	462.3364	0.43	NEG	hilic				5.90E-02	5.39E-02	-0.46	1.56E-01	-0.37	3.78E-01	-0.20	7.60E-01	-0.08
462.3713@11.307519	462.3713	11.31	POS	RP				7.02E-01	9.81E-01	0.01	8.41E-01	-0.06	1.10E-01	-0.42	7.03E-01	0.10
463.1836@3.7323487	463.1836	3.73	NEG	hilic				2.10E-01	5.48E-03	-0.53	6.75E-01	-0.10	3.97E-01	0.22	3.62E-01	-0.22
463.1847@6.1523995	463.1847	6.15	POS	RP				1.62E-01	1.28E-02	-0.56	8.75E-01	-0.04	2.01E-01	0.33	7.28E-01	-0.10
463.185@3.4388027	463.1850	3.44	POS	hilic				2.11E-01	4.23E-02	-0.48	1.00E+00	0.00	1.50E-01	0.39	7.34E-01	-0.10
463.2068@0.4209098	463.2068	0.42	POS	hilic				2.56E-01	8.29E-03	0.81	2.39E-01	0.31	4.89E-01	0.20	7.11E-01	0.11
463.2326@11.428588	463.2326	11.43	NEG	RP				6.91E-01	9.66E-01	0.01	4.95E-01	0.16	1.45E-01	0.40	3.15E-01	0.24
463.2758@9.317048	463.2758	9.32	POS	RP				7.11E-01	5.72E-01	0.18	1.41E-01	0.38	7.46E-01	-0.08	6.71E-01	0.11
463.2765@1.4284693	463.2765	1.43	POS	hilic				5.78E-01	2.56E-01	0.33	1.56E-01	0.36	8.54E-01	-0.04	9.62E-01	-0.01
463.3056@10.38829	463.3056	10.39	NEG	RP				2.02E-01	2.58E-01	0.33	4.85E-01	-0.17	3.18E-01	0.25	4.06E-02	0.64
463.3068@1.5375578	463.3068	1.54	POS	hilic				3.30E-01	3.78E-01	0.25	3.88E-01	-0.18	4.26E-01	-0.16	1.74E-01	0.39
463.308@10.385712	463.3080	10.39	POS	RP				2.94E-01	9.74E-02	0.49	2.03E-01	-0.34	7.82E-01	0.08	7.28E-01	0.11
463.3681@11.240206	463.3681	11.24	POS	RP				6.79E-01	9.96E-01	0.00	1.06E-01	0.48	7.35E-01	0.09	4.86E-01	0.21
463.3701@10.6947775	463.3701	10.69	POS	RP				5.57E-01	2.34E-01	-0.39	3.20E-01	0.26	7.26E-01	-0.10	7.71E-01	-0.08
463.3789@0.67116374	463.3789	0.67	POS	hilic				6.50E-01	8.00E-01	-0.07	1.62E-01	-0.32	1.69E-01	-0.35	1.05E-01	-0.35
463.8404@15.27172	463.8404	15.27	NEG	RP				6.94E-01	4.93E-01	0.19	6.86E-01	0.11	8.97E-02	0.44	1.82E-01	0.35
464.3509@0.4285	464.3509	0.43	NEG	hilic				4.53E-01	4.67E-02	-0.36	7.82E-01	0.07	4.28E-01	0.18	7.09E-01	0.09
464.3516@0.43349034	464.3516	0.43	POS	hilic				3.55E-01	1.38E-01	-0.28	2.55E-01	0.29	4.32E-01	-0.18	4.28E-01	0.21
465.1575@10.355834	465.1575	10.36	NEG	RP				7.67E-01	5.16E-01	-0.16	8.98E-01	-0.03	4.52E-01	0.19	4.27E-01	-0.21
465.1703@11.048272	465.1703	11.05	NEG	RP				8.32E-01	1.62E-01	0.41	4.06E-01	0.27	5.31E-01	0.17	2.72E-01	0.31
465.286@1.4442878	465.2860	1.44	POS	hilic				5.18E-01	5.38E-02	0.54	5.16E-01	0.15	3.30E-01	0.25	1.38E-01	0.38
465.2928@2.097838	465.2928	2.10	POS	hilic				2.79E-01	9.99E-01	0.00	1.51E-01	0.40	1.39E-01	-0.34	9.78E-01	-0.01
465.3083@8.435421	465.3083	8.44	NEG	RP				4.41E-01	9.98E-02	0.54	1.90E-01	0.39	7.44E-01	0.08	8.30E-02	0.48
465.3084@8.425499	465.3084	8.43	POS	RP				5.88E-01	1.92E-01	0.43	4.23E-01	0.24	7.60E-01	-0.07	1.79E-01	0.37
465.309@2.244387	465.3090	2.24	POS	hilic				5.45E-01	2.66E-01	0.34	5.15E-01	0.17	4.00E-01	-0.18	2.55E-01	0.27
465.3207@10.634195	465.3207	10.63	NEG	RP				5.43E-02	3.13E-01	0.26	5.40E-02	-0.39	1.55E-02	-0.48	6.49E-01	0.13
465.3224@1.5213271	465.3224	1.52	POS	hilic				7.40E-01	4.72E-01	-0.20	2.22E-01	-0.28	6.73E-02	-0.41	3.46E-01	-0.26
465.3231@10.634218	465.3231	10.63	POS	RP				3.92E-02	9.67E-02	0.44	5.36E-02	-0.52	6.61E-02	-0.47	6.20E-01	0.16
465.8114@3.1993065	465.8114	3.20	NEG	hilic				9.46E-01	5.82E-01	0.18	9.73E-01	-0.01	4.58E-01	0.22	5.18E-01	0.19
465.8117@3.1987438	465.8117	3.20	NEG	hilic				8.87E-01	3.67E-01	0.29	8.22E-01	0.07	2.46E-01	0.32	6.16E-01	0.15
466.1099@0.40283903	466.1099	0.40	NEG	hilic				3.67E-01	6.09E-01	-0.13	1.97E-01	0.36	4.64E-01	0.19	6.84E-02	0.54
466.2284@11.429427	466.2284	11.43	NEG	RP				9.38E-02	6.85E-02	0.43	1.32E-02	0.62	5.49E-03	0.80	1.96E-01	0.28
466.2558@8.147117	466.2558	8.15	NEG	RP				6.64E-02	6.21E-03	-0.69	7.81E-01	0.06	1.76E-01	0.40	5.42E-01	-0.19
466.2561@8.037977	466.2561	8.04	NEG	RP				9.20E-01	8.26E-01	0.06	3.73E-01	0.23	6.87E-01	0.14	7.59E-01	-0.08
466.3105@0.4676323	466.3105	0.47	NEG	hilic				3.83E-01	1.99E-01	0.35	1.19E-01	0.42	7.40E-01	-0.10	8.82E-02	0.53
466.3152@10.700105	466.3152	10.70	NEG	RP				6.09E-02	2.98E-01	-0.27	1.12E-02	0.61	1.27E-01	0.41	7.05E-02	0.54
466.3412@0.96910155	466.3412	0.97	POS	hilic				9.94E-01	9.34E-01	-0.02	6.80E-01	-0.10	9.24E-01	0.03	9.85E-01	0.01
467.1724@10.567046	467.1724	10.57	NEG	RP				3.50E-01	9.74E-01	-0.01	2.83E-02	0.65	2.35E-01	0.35	2.79E-01	0.34
467.1841@10.783026	467.1841	10.78	NEG	RP				5.71E-01	9.31E-01	0.02	4.17E-01	-0.17	5.41E-01	0.15	1.29E-01	-0.32
467.3012@1.2537347	467.3012	1.25	POS	hilic				5.89E-02	8.34E-02	0.52	8.25E-01	0.06	2.18E-02	0.66	5.87E-03	0.87
467.3018@9.466462	467.3018	9.47	POS	RP	LysoPC 14:0	10 eV: 184.0734 (100), 468.3094 (80)	2	4.68E-02	2.79E-02	0.62	8.54E-01	-0.05	4.24E-02	0.56	5.62E-03	0.82
467.3024@1.3940765	467.3024	1.39	POS	hilic				1.97E-01	3.89E-02	0.60	7.24E-01	0.10	3.50E-02	0.59	4.40E-02	0.67
467.3024@10.317916	467.3024	10.32	POS	RP				4.32E-02	9.85E-03	0.78	9.10E-01	0.03	3.34E-01	-0.23	9.04E-01	0.03
467.3024@9.61647	467.3024	9.62	POS	RP				8.25E-02	3.60E-02	0.62	8.69E-01	0.05	6.01E-02	0.56	8.61E-03	0.82
467.3033@10.321999	467.3033	10.32	NEG	RP				2.00E-03	4.98E-04	1.06	5.19E-01	0.11	5.72E-01	0.10	2.28E-02	0.53
467.382@0.43765455	467.3820	0.44	POS	hilic				6.66E-01	2.00E-01	0.33	4.93E-01	-0.17	7.63E-01	0.08	9.78E-01	-0.01
467.382@10.185162	467.3820	10.19	POS	RP				3.37E-01	2.39E-01	0.38	6.10E-02	0.56	7.57E-02	0.55	9.84E-01	0.01
467.434@0.47883713	467.4340	0.48	POS	hilic				9.03E-01	4.47E-01	0.21	3.74E-01	0.21	3.60E-01	0.28	3.38E-01	0.29
467.4343@9.543164	467.4343	9.54	POS	RP				8.79E-01	5.14E-01	0.20	2.98E-01	0.26	3.30E-01	0.32	2.80E-01	0.34
467.81@3.2038007	467.8100	3.20	NEG	hilic				4.91E-01	4.65E-01	0.19	2.13E-02	0.61	1.36E-01	0.47	2.64E-01	0.31
467.8241@15.275421	467.8241	15.28	NEG	RP				6.00E-01	4.72E-01	0.25	6.86E-01	-0.11	6.53E-01	-0.11	1.68E-01	-0.41
467.9688@7.4403377	467.9688	7.44	POS	hilic				7.81E-01	8.68E-01	-0.05	2.41E-01	-0.32	8.75E-01	0.05	6.14E-01	0.15
468.0878@7.190406	468.0878	7.19	NEG	RP				2.80E-01	1.22E-01	-0.39	3.65E-01	0.26	2.34E-01	0.30	6.35E-01	-0.12
468.1394@10.434726	468.1394	10.43	NEG	RP				2.97E-01	7.91E-01	-0.05	4.41E-02	0.55	2.60E-01	0.26	3.27E-01	0.22

Supplementary table 2: Metabolomics analysis results

Compound	Mass	Retention Time	Ionization mode	column	Compound_ID	MSMS_spectra	ID_level	ANOVA P-FDR	Anorexia vs control P	Anorexia vs control Estimate	overweight vs control P	overweight vs control Estimate	obese vs control P	obese vs control Estimate	athletes vs control P	athletes vs control Estimate
468.3206@10.6965685	468.3206	10.70	NEG	RP				8.32E-03	4.33E-02	-0.45	2.08E-03	0.76	1.40E-01	0.42	1.84E-01	0.38
468.3221@10.691046	468.3221	10.69	POS	RP				1.59E-02	2.93E-04	-0.84	1.88E-01	0.34	4.82E-01	0.20	7.82E-01	0.08
468.3559@11.608636	468.3559	11.61	POS	RP				8.64E-01	3.03E-01	0.37	6.16E-01	0.13	9.39E-01	-0.02	6.75E-01	0.13
468.3579@10.764137	468.3579	10.76	POS	RP				1.03E-01	5.27E-01	-0.15	7.90E-01	0.07	1.19E-02	0.75	2.35E-01	0.30
468.3798@10.662566	468.3798	10.66	NEG	RP				5.89E-03	3.08E-03	-0.59	3.97E-02	0.56	9.08E-01	0.03	5.79E-02	0.46
468.3801@10.653772	468.3801	10.65	POS	RP				2.41E-02	9.06E-02	-0.27	7.57E-01	0.07	7.31E-01	-0.07	1.53E-02	0.64
468.383@0.4341364	468.3830	0.43	POS	hilic				5.08E-03	4.60E-03	-0.60	3.67E-01	0.25	3.38E-01	-0.24	1.14E-02	0.64
468.8859@15.26041	468.8859	15.26	NEG	RP				3.42E-01	1.69E-01	0.36	3.18E-02	0.60	5.04E-01	0.17	6.89E-02	0.48
469.0978@10.433143	469.0978	10.43	NEG	RP				1.03E-01	4.63E-02	-0.32	1.19E-01	0.39	1.97E-01	0.30	6.53E-01	-0.10
469.1159@10.727151	469.1159	10.73	NEG	RP				5.64E-02	1.32E-02	0.68	4.17E-01	-0.24	5.48E-01	0.17	2.53E-02	0.62
469.1518@10.326793	469.1518	10.33	NEG	RP				5.71E-01	7.26E-01	0.10	6.43E-01	0.14	3.27E-02	0.56	5.84E-01	0.15
469.1886@10.783532	469.1886	10.78	NEG	RP				6.36E-02	5.27E-01	-0.19	3.30E-02	0.65	1.26E-02	0.70	3.92E-01	0.27
469.2534@0.4673203	469.2534	0.47	POS	hilic				3.63E-01	2.10E-01	0.35	3.17E-02	0.64	3.35E-02	0.60	2.35E-01	0.34
469.2583@11.925836	469.2583	11.93	NEG	RP				7.02E-01	6.67E-01	-0.14	2.72E-01	-0.31	4.42E-01	0.24	9.42E-01	-0.02
470.0555@0.59984624	470.0555	0.60	NEG	hilic				5.20E-01	1.28E-01	0.51	9.14E-01	-0.03	8.84E-01	-0.03	4.93E-01	0.19
470.1053@7.9492297	470.1053	7.95	NEG	RP				1.62E-01	1.85E-03	-0.64	1.49E-01	-0.35	9.91E-01	0.00	8.23E-01	0.06
470.1431@10.43515	470.1431	10.44	NEG	RP				5.45E-04	1.44E-02	-0.47	8.71E-03	0.68	9.34E-03	0.62	5.04E-01	-0.16
470.3489@0.42672923	470.3489	0.43	NEG	hilic				1.40E-01	1.83E-03	-0.62	5.10E-01	0.17	8.41E-01	-0.05	8.66E-01	0.04
470.3707@0.44668424	470.3707	0.45	POS	hilic				7.42E-01	2.22E-01	-0.22	2.33E-01	-0.21	1.81E-01	-0.23	5.27E-01	-0.13
470.3718@9.878678	470.3718	9.88	POS	RP				9.10E-01	7.63E-01	0.06	7.79E-01	0.05	5.99E-01	0.10	3.30E-01	0.24
471.1134@10.72698	471.1134	10.73	NEG	RP				1.79E-01	1.10E-01	0.45	3.78E-01	-0.28	7.53E-01	-0.09	9.62E-02	0.49
471.1664@10.565389	471.1664	10.57	NEG	RP				4.35E-01	3.62E-01	-0.25	1.58E-01	0.41	6.71E-01	-0.12	7.32E-01	0.09
471.2043@11.036462	471.2043	11.04	NEG	RP				4.10E-01	1.05E-01	0.51	1.01E-01	0.46	2.99E-02	0.64	7.52E-02	0.54
471.2965@8.9259205	471.2965	8.93	POS	RP				6.40E-01	1.64E-01	0.43	2.33E-01	0.32	9.14E-01	0.03	2.37E-01	0.34
471.297@1.019769	471.2970	1.02	POS	hilic				8.42E-01	4.01E-01	0.28	4.55E-01	0.21	6.96E-01	-0.11	7.65E-01	0.09
471.8226@15.276755	471.8226	15.28	NEG	RP				2.36E-01	2.38E-02	-0.61	1.84E-02	-0.66	3.62E-01	-0.25	1.21E-01	-0.47
472.1161@10.721322	472.1161	10.72	NEG	RP				8.94E-01	9.72E-01	0.01	4.96E-01	-0.18	5.56E-01	0.17	7.63E-01	0.09
472.1292@3.955053	472.1292	3.96	POS	hilic				8.69E-01	3.39E-01	-0.26	7.67E-01	0.08	6.52E-01	0.13	7.71E-01	0.09
472.1554@10.711927	472.1554	10.71	NEG	RP				9.35E-01	6.34E-01	0.15	8.65E-01	0.05	6.76E-01	-0.10	6.35E-01	-0.13
472.2064@11.03639	472.2064	11.04	NEG	RP				4.39E-01	7.99E-02	0.53	3.51E-02	0.64	2.34E-01	0.37	1.31E-01	0.41
472.2443@9.736884	472.2443	9.74	POS	RP				5.45E-01	1.17E-01	-0.38	8.91E-01	-0.03	3.34E-01	0.26	7.06E-01	-0.12
472.2489@8.666469	472.2489	8.67	NEG	RP				5.78E-01	2.90E-01	-0.26	3.26E-01	0.22	7.24E-01	0.09	3.43E-01	0.27
472.3108@10.952119	472.3108	10.95	POS	RP				7.93E-01	8.17E-01	0.07	1.99E-01	0.36	1.96E-01	0.36	6.76E-01	0.12
473.1413@10.319869	473.1413	10.32	NEG	RP				8.41E-02	9.95E-01	0.00	1.87E-01	0.29	1.63E-03	0.77	3.49E-01	0.25
473.1446@11.429096	473.1446	11.43	NEG	RP				5.62E-01	8.72E-01	-0.05	3.21E-01	0.26	6.37E-02	0.53	3.01E-01	0.30
473.184@10.781948	473.1840	10.78	NEG	RP				6.52E-01	6.66E-01	0.11	1.47E-01	0.44	1.14E-01	0.40	3.81E-01	0.23
473.3064@10.562251	473.3064	10.56	POS	RP				9.58E-01	4.86E-01	-0.18	4.83E-01	-0.17	9.07E-01	-0.03	8.48E-01	-0.05
473.3087@0.40632352	473.3087	0.41	POS	hilic				7.69E-01	1.24E-01	-0.33	2.77E-01	-0.24	6.82E-01	-0.10	5.06E-01	-0.16
473.8204@15.265489	473.8204	15.27	NEG	RP				4.98E-01	5.81E-02	-0.57	9.31E-01	-0.02	2.56E-01	-0.30	1.72E-01	-0.41
474.1251@3.9551804	474.1251	3.96	POS	hilic				8.86E-01	2.65E-01	-0.29	9.53E-01	0.02	6.96E-01	0.11	9.36E-01	-0.03
474.289@0.42930767	474.2890	0.43	NEG	hilic				4.47E-01	6.37E-01	-0.17	5.75E-01	0.13	5.61E-02	0.53	3.21E-01	0.31
474.2969@0.43037504	474.2969	0.43	POS	hilic				7.56E-02	1.21E-01	0.36	6.75E-01	0.07	9.43E-03	0.68	8.23E-01	0.04
474.355@10.7960005	474.3550	10.80	NEG	RP				9.46E-01	5.82E-01	0.13	9.50E-01	-0.01	7.09E-01	-0.08	6.62E-01	0.09
474.3699@10.860984	474.3699	10.86	NEG	RP				3.63E-01	3.72E-02	-0.56	8.47E-01	-0.05	6.47E-01	-0.12	6.33E-01	0.14
474.3703@0.4264208	474.3703	0.43	NEG	hilic				8.80E-02	7.03E-02	-0.50	6.07E-02	0.50	5.23E-01	-0.19	4.18E-01	0.23
475.1998@11.036258	475.1998	11.04	NEG	RP				4.41E-01	3.32E-02	0.64	1.53E-01	0.45	1.06E-01	0.48	6.29E-02	0.53
475.2346@11.222376	475.2346	11.22	NEG	RP				7.40E-01	1.73E-01	0.46	9.52E-01	0.02	7.26E-01	0.09	4.11E-01	0.25
475.2377@10.599613	475.2377	10.60	NEG	RP				5.89E-01	4.92E-01	0.19	1.06E-01	0.46	4.48E-01	0.20	7.75E-01	-0.08
475.2689@9.715475	475.2689	9.72	NEG	RP				2.71E-03	8.58E-03	0.80	1.01E-01	-0.40	2.06E-01	-0.35	1.78E-01	0.39
475.2711@9.71037	475.2711	9.71	POS	RP				2.87E-04	3.66E-04	0.98	5.27E-02	-0.51	2.87E-01	-0.28	1.36E-01	0.43
475.2717@11.428949	475.2717	11.43	NEG	RP				7.58E-01	6.50E-01	0.13	2.73E-01	0.27	1.37E-01	0.44	3.51E-01	0.24
475.3217@10.988891	475.3217	10.99	POS	RP				6.09E-01	6.34E-01	0.11	9.49E-01	0.02	2.09E-01	-0.38	4.46E-01	0.24
475.8203@15.266517	475.8203	15.27	NEG	RP				6.66E-01	1.40E-01	-0.46	1.52E-01	-0.44	4.53E-01	-0.23	1.15E-01	-0.43
476.2016@0.51806825	476.2016	0.52	NEG	hilic				3.00E-01	1.64E-01	-0.34	2.15E-01	0.35	7.99E-01	0.07	1.71E-01	0.36
476.2017@6.635505	476.2017	6.64	NEG	RP				2.12E-01	4.60E-01	0.22	1.21E-01	0.45	5.69E-01	-0.16	2.72E-02	0.58
476.2282@0.5058571	476.2282	0.51	POS	hilic				1.31E-01	5.86E-02	-0.46	1.47E-02	-0.54	6.92E-01	-0.11	6.11E-03	-0.59
476.3124@9.633829	476.3124	9.63	NEG	RP				9.20E-02	3.56E-02	-0.71	8.28E-01	0.06	4.34E-01	0.20	7.64E-02	-0.41
476.3842@0.42501435	476.3842	0.43	NEG	hilic				5.60E-02	1.56E-01	-0.31	4.38E-02	0.52	2.90E-01	0.29	2.77E-02	0.53
476.3846@11.029632	476.3846	11.03	NEG	RP				5.56E-01	9.15E-01	0.03	2.96E-01	0.27	8.37E-01	0.05	1.91E-01	-0.31
477.0565@6.1417694	477.0565	6.14	POS	hilic				1.12E-01	9.42E-01	-0.02	7.94E-01	-0.08	4.23E-02	-0.55	6.38E-02	0.49
477.1904@0.54628944	477.1904	0.55	POS	hilic				5.25E-01	6.93E-01	-0.12	5.83E-01	-0.14	1.38E-02	-0.57	5.04E-01	-0.18
477.2269@11.430125	477.2269	11.43	NEG	RP				3.95E-01	1.67E-01	0.36	1.88E-02	0.63	4.16E-02	0.54	1.97E-01	0.39
477.2847@2.157991	477.2847	2.16	NEG	hilic				5.05E-04	1.28E-02	0.69	1.67E-02	-0.63	1.40E-02	-0.65	3.71E-01	0.27
477.285@9.9696455	477.2850	9.97	NEG	RP	LysoPE 18:2	10 eV: 279.2313 (100), 476.2770 (80)	2	1.39E-04	1.35E-02	0.70	1.62E-02	-0.65	1.49E-02	-0.68	1.48E-01	0.37
477.285@1.8676724	477.2850	1.87	NEG	hilic				3.43E-04	1.05E-02	0.70	2.29E-02	-0.58	1.24E-02	-0.69	3.48E-01	0.27
477.285@9.859716	477.2850	9.86	NEG	RP				1.57E-04	8.28E-02	0.49	3.10E-03	-0.83	4.62E-03	-0.80	3.48E-01	0.24
477.2857@9.853358	477.2857	9.85	POS	RP				2.26E-04	1.06E-02	0.74	3.01E-02	-0.58	2.14E-02	-0.64	1.32E-01	0.38
477.286@1.7020333	477.2860	1.70	POS	hilic				8.58E-04	1.63E-02	0.68	1.58E-02	-0.63	1.97E-02	-0.63	4.94E-01	0.20
477.2865@1.952171	477.2865	1.95	POS	hilic				6.23E-04	1.79E-02	0.67	1.09E-02	-0.68	1.75E-02	-0.63	4.84E-01	0.21
477.2871@9.965579	477.2871	9.97	POS	RP	LysoPE 18:2	10 eV: 337.2734 (100), 478.2931 (12)	2	5.24E-04	3.72E-03	0.84	8.64E-02	-0.46	8.32E-02	-0.52	7.91E-02	0.44
477.3173@10.032336	477.3173	10.03	POS	RP				8.94E-01	4.87E-01	-0.19	8.26E-01	-0.06	7.49E-01	0.10	4.01E-01	-0.22
477.3223@1.1564573	477.3223	1.16	POS	hilic				1.61E-01	1.25E-01	0.46	8.76E-02	-0.46	8.38E-01	-0.06	4.20E-01	0.25
477.3224@1.2968115	477.3224	1.30	POS	hilic				4.14E-01	1.85E-01	0.46	1.76E-01	-0.27	8.11E-01	0.06	8.96E-01	-0.04

Supplementary table 2: Metabolomics analysis results

Compound	Mass	Retention Time	Ionization mode	column	Compound_ID	MSMS_spectra	ID_level	ANOVA	Anorexia vs control		overweight vs control		obese vs control		athletes vs control	
								P-FDR	P	Estimate	P	Estimate	P	Estimate	P	Estimate
477.3224@12.204577	477.3224	12.20	POS	RP				1.85E-01	3.92E-02	-0.71	5.57E-01	0.17	7.62E-01	0.07	7.78E-01	-0.08
477.9857@8.215192	477.9857	8.22	NEG	RP				3.22E-01	6.17E-02	-0.51	6.63E-01	-0.13	6.70E-01	-0.12	3.53E-02	-0.67
478.0417@6.0301485	478.0417	6.03	POS	hilic				4.47E-01	2.83E-01	-0.37	4.99E-01	-0.18	1.89E-02	-0.65	9.68E-02	-0.44
478.1688@0.9982528	478.1688	1.00	NEG	hilic				2.48E-01	2.08E-01	0.37	2.20E-03	0.80	3.10E-01	0.29	2.31E-01	0.33
478.1693@0.99837893	478.1693	1.00	NEG	hilic				2.11E-01	3.88E-01	0.28	9.35E-04	0.83	9.05E-02	0.45	2.96E-01	0.30
478.1848@10.6352005	478.1848	10.64	NEG	RP				2.44E-01	2.66E-01	-0.24	4.96E-01	-0.16	3.62E-01	0.25	3.65E-02	-0.42
479.1402@10.313998	479.1402	10.31	NEG	RP				3.37E-01	4.06E-01	-0.14	2.35E-01	0.27	7.99E-02	0.45	5.50E-01	0.14
479.1668@7.2434306	479.1668	7.24	POS	RP				3.04E-01	2.30E-01	-0.35	2.14E-01	0.34	9.75E-01	0.01	1.25E-01	-0.39
479.2215@11.429559	479.2215	11.43	NEG	RP				6.32E-01	1.51E-01	0.43	2.93E-01	0.32	5.31E-02	0.55	2.77E-01	0.32
479.2399@0.42763296	479.2399	0.43	POS	hilic				6.53E-01	9.67E-01	0.01	9.68E-02	0.45	8.74E-01	0.04	4.61E-01	0.20
479.2988@9.784055	479.2988	9.78	NEG	RP				5.27E-01	2.77E-01	0.38	4.79E-01	-0.19	3.61E-01	0.26	1.98E-01	0.32
479.3003@2.085845	479.3003	2.09	NEG	hilic				1.20E-03	3.91E-02	0.55	4.19E-02	-0.57	8.84E-03	-0.72	3.06E-01	0.31
479.3004@10.130736	479.3004	10.13	NEG	RP	LysoPE 18:1	10 eV: 478.2931 (100), 281.2473 (50), 196.0365 (5)	2	1.11E-03	7.20E-02	0.46	1.02E-01	-0.49	1.30E-02	-0.68	4.74E-02	0.54
479.3005@10.2309475	479.3005	10.23	NEG	RP	LysoPE 18:1	10 eV: 478.2931 (100), 281.2473 (50), 196.0365 (5)	2	1.53E-03	8.57E-02	0.46	2.68E-02	-0.60	6.42E-03	-0.77	3.57E-01	0.28
479.3005@1.8008212	479.3005	1.80	NEG	hilic				1.33E-02	3.34E-01	0.29	5.94E-02	-0.45	5.54E-02	-0.45	1.25E-01	0.41
479.3021@1.8755898	479.3021	1.88	POS	hilic				5.19E-02	2.57E-01	0.34	4.15E-02	-0.56	8.13E-02	-0.51	5.71E-01	0.18
479.3021@10.221932	479.3021	10.22	POS	RP				1.11E-02	2.39E-03	0.90	5.34E-01	0.17	6.61E-01	-0.11	1.04E-02	0.80
479.3022@10.225419	479.3022	10.23	POS	RP	LysoPE 18:1	10 eV: 480.3075 (100), 339.2896 (80), 44.0481 (13)	1	1.13E-02	1.07E-01	0.45	2.08E-01	-0.37	4.95E-02	-0.57	1.07E-01	0.47
479.3024@1.6300776	479.3024	1.63	POS	hilic				7.89E-03	3.51E-02	0.59	1.18E-01	-0.43	4.38E-02	-0.60	3.75E-01	0.27
479.3032@1.3617406	479.3032	1.36	POS	hilic				2.82E-01	6.17E-02	0.52	3.35E-01	-0.20	7.34E-01	0.08	8.07E-01	0.07
479.3378@1.0988618	479.3378	1.10	POS	hilic				5.19E-01	6.70E-01	-0.12	1.98E-01	-0.41	3.36E-02	-0.51	9.33E-01	0.03
479.3401@10.315655	479.3401	10.32	POS	RP				2.97E-01	9.36E-01	-0.02	1.66E-01	-0.46	3.98E-02	-0.48	5.56E-01	0.18
479.7917@7.8759413	479.7917	7.88	POS	hilic				4.66E-01	1.32E-01	0.48	9.27E-01	0.02	5.38E-01	-0.15	8.98E-01	-0.03
479.8148@15.26669	479.8148	15.27	NEG	RP				7.34E-01	4.73E-01	0.17	5.29E-01	0.20	9.42E-01	-0.02	3.38E-01	-0.29
479.83@10.028275	479.8300	10.03	POS	RP				3.23E-01	2.14E-01	-0.35	4.64E-01	0.24	3.82E-01	0.29	2.27E-01	-0.32
480.1975@10.7992115	480.1975	10.80	NEG	RP				6.98E-01	7.34E-01	0.09	4.89E-01	0.17	2.52E-01	-0.25	6.22E-01	-0.12
480.9755@6.2489405	480.9755	6.25	NEG	hilic				3.59E-01	5.46E-02	0.61	6.40E-01	0.14	8.76E-01	-0.04	1.06E-01	0.43
481.1876@11.0735	481.1876	11.07	NEG	RP				6.98E-01	2.07E-01	0.31	2.82E-01	0.27	7.80E-01	-0.05	4.92E-01	0.14
481.2187@11.429703	481.2187	11.43	NEG	RP				7.46E-01	5.24E-01	0.18	8.84E-01	-0.03	2.00E-01	0.37	3.74E-01	0.24
481.3152@2.0603967	481.3152	2.06	NEG	hilic				1.88E-03	7.57E-03	0.78	7.89E-02	-0.44	3.66E-02	-0.58	4.66E-01	0.21
481.3158@10.112241	481.3158	10.11	NEG	RP				2.93E-02	8.54E-02	0.55	2.11E-01	-0.32	4.62E-01	-0.20	2.15E-02	0.62
481.316@10.403984	481.3160	10.40	NEG	RP				6.77E-04	2.43E-02	0.63	2.44E-02	-0.56	6.60E-03	-0.81	6.33E-01	0.13
481.316@10.497587	481.3160	10.50	NEG	RP				2.39E-03	1.11E-02	0.77	1.98E-01	-0.32	5.88E-02	-0.55	1.53E-01	0.39
481.3164@1.4099486	481.3164	1.41	NEG	hilic				6.68E-04	2.51E-04	1.03	8.87E-01	-0.03	8.22E-01	0.07	1.02E-04	1.03
481.3165@10.002692	481.3165	10.00	NEG	RP				7.88E-03	3.10E-03	0.90	2.35E-01	-0.30	5.59E-01	0.16	3.37E-02	0.62
481.3168@1.8618116	481.3168	1.86	POS	hilic				1.17E-03	2.80E-03	0.90	9.26E-02	-0.43	9.73E-02	-0.46	3.47E-01	0.26
481.317@9.77865	481.3170	9.78	POS	RP				5.47E-01	9.74E-01	-0.01	1.06E-01	-0.56	7.54E-01	-0.09	4.70E-01	-0.22
481.3171@9.769395	481.3171	9.77	POS	RP				2.29E-01	8.90E-01	0.04	2.12E-01	-0.37	1.20E-01	0.41	2.75E-01	0.35
481.3175@1.2003074	481.3175	1.20	POS	hilic				2.77E-01	1.06E-02	0.72	7.75E-01	0.09	8.04E-02	0.47	9.37E-02	0.48
481.3177@9.888199	481.3177	9.89	POS	RP	LysoPC 15:0	20 eV: 184.0739 (100), 104.1071 (98), 482.3237 (70)	1	1.07E-01	4.67E-03	0.80	9.23E-01	0.03	1.26E-01	0.42	1.43E-02	0.66
481.3185@1.623217	481.3185	1.62	POS	hilic				1.28E-02	6.00E-01	0.16	1.03E-02	-0.51	8.92E-05	-0.77	2.08E-01	-0.30
481.319@1.3459315	481.3190	1.35	POS	hilic				1.82E-02	8.23E-04	1.07	6.23E-02	0.53	1.37E-03	0.93	1.27E-02	0.74
481.3196@10.491303	481.3196	10.49	POS	RP				3.19E-03	1.09E-02	0.74	3.01E-01	-0.27	4.32E-02	-0.61	2.25E-01	0.34
481.3481@10.483487	481.3481	10.48	POS	RP				1.47E-01	7.50E-01	0.08	9.28E-01	0.02	6.64E-01	0.11	1.09E-02	0.75
481.3507@1.3541994	481.3507	1.35	POS	hilic				3.90E-01	6.23E-01	-0.16	2.92E-02	-0.61	5.13E-01	-0.16	7.98E-01	0.08
481.3532@10.41524	481.3532	10.42	POS	RP				4.41E-01	5.66E-01	-0.16	2.11E-01	-0.43	5.63E-01	-0.15	3.62E-01	0.30
481.9767@8.894436	481.9767	8.89	NEG	RP				6.25E-01	3.41E-01	0.27	5.53E-01	-0.17	2.50E-01	0.36	4.56E-01	0.22
482.2873@8.966752	482.2873	8.97	NEG	RP				6.44E-01	9.99E-02	-0.44	2.77E-01	-0.27	9.27E-02	-0.43	2.48E-01	-0.32
482.8642@15.256722	482.8642	15.26	NEG	RP				1.00E+00	8.26E-01	-0.06	9.66E-01	-0.01	9.85E-01	-0.01	9.31E-01	-0.03
483.2034@10.722054	483.2034	10.72	NEG	RP				6.28E-01	3.96E-01	0.23	3.15E-01	0.24	8.23E-02	0.53	2.68E-01	0.27
483.2833@8.01353	483.2833	8.01	POS	RP				9.63E-01	6.29E-01	0.14	9.07E-01	0.03	8.29E-01	-0.07	6.24E-01	-0.14
483.5646@3.3320153	483.5646	3.33	NEG	hilic				3.85E-01	8.68E-01	0.05	7.88E-01	0.07	8.88E-01	0.04	1.38E-02	0.59
483.8084@15.265401	483.8084	15.27	NEG	RP				3.37E-01	2.86E-01	0.28	7.68E-01	0.07	2.20E-02	0.68	3.71E-01	0.24
484.1037@0.40736964	484.1037	0.41	NEG	hilic				5.47E-01	3.40E-01	0.29	2.62E-01	-0.29	5.37E-01	-0.16	6.15E-01	0.15
484.3517@1.8704811	484.3517	1.87	POS	hilic				4.20E-01	4.36E-02	0.62	4.04E-01	0.19	2.86E-01	0.26	1.19E-01	0.45
484.3523@1.681235	484.3523	1.68	POS	hilic				8.42E-01	5.58E-01	0.20	6.43E-01	-0.14	3.87E-01	0.22	5.68E-01	0.18
484.3526@1.5157495	484.3526	1.52	POS	hilic				9.08E-01	7.62E-01	0.10	5.28E-01	-0.19	5.58E-01	0.16	9.16E-01	0.03
484.3528@2.2344613	484.3528	2.23	POS	RP				6.50E-01	9.73E-01	-0.01	2.64E-01	-0.33	5.25E-01	0.17	5.49E-01	0.18
484.3529@1.7904218	484.3529	1.79	POS	RP				8.35E-01	9.26E-01	0.03	5.15E-01	-0.19	3.61E-01	0.25	9.88E-01	0.00
484.3533@11.308876	484.3533	11.31	POS	RP				1.73E-01	2.65E-01	0.34	3.42E-01	-0.29	1.11E-01	-0.41	6.67E-02	-0.53
484.3823@0.43936366	484.3823	0.44	POS	hilic				4.63E-01	7.66E-01	0.05	1.38E-01	0.32	3.36E-01	-0.12	8.03E-01	0.05
484.8607@15.255816	484.8607	15.26	NEG	RP				3.49E-01	1.15E-01	0.50	7.45E-01	-0.08	1.02E-01	0.43	8.61E-01	0.04
485.1306@10.867656	485.1306	10.87	NEG	RP				2.72E-01	7.85E-01	-0.08	1.36E-01	0.40	3.47E-02	0.62	3.06E-01	0.28
485.1954@10.440012	485.1954	10.44	NEG	RP				4.62E-01	2.55E-01	0.28	4.14E-01	0.22	2.92E-01	0.31	9.86E-03	0.64
485.2075@10.720492	485.2075	10.72	NEG	RP				2.47E-01	6.32E-01	-0.16	9.98E-03	-0.73	9.99E-01	0.00	2.50E-01	-0.29
485.254@11.429036	485.2540	11.43	NEG	RP				4.41E-01	4.84E-01	-0.20	5.14E-01	0.17	7.11E-02	0.54	7.26E-01	0.10
485.8067@15.262953	485.8067	15.26	NEG	RP				4.46E-01	2.91E-02	-0.71	2.37E-01	-0.36	2.26E-01	-0.33	1.73E-01	-0.40
486.1035@10.433219	486.1035	10.43	NEG	RP				1.55E-01	4.49E-02	-0.44	2.88E-01	0.30	1.73E-01	0.36	9.30E-01	-0.02
486.1722@3.6015844	486.1722	3.60	NEG	hilic				6.76E-01	7.49E-01	0.11	3.18E-01	0.28	2.12E-01	0.33	5.57E-01	-0.17
486.2627@11.925027	486.2627	11.93	NEG	RP				3.86E-01	3.19E-01	0.30	5.15E-02	0.53	4.07E-02	0.66	1.14E-01	0.40
486.272@10.556133	486.2720	10.56	POS	RP				7.31E-01	9.97E-01	0.00	9.01E-01	0.04	1.39E-01	0.42	5.89E-01	0.16
486.3336@0.43502703	486.3336	0.44	POS	hilic				4.91E-01	4.81E-01	-0.15	4.84E-01	-0.17	5.72E-01	0.14	5.27E-02	-0.39
486.3746@12.425986	486.3746	12.43	POS	RP				6.67E-01	5.91E-01	-0.16	7.14E-01	0.11	4.71E-01	-0.17	1.75E-01	-0.45

Supplementary table 2: Metabolomics analysis results

								ANOVA	Anorexia vs control		overweight vs control		obese vs control		athletes vs control	
Compound	Mass	Retention Time	Ionization mode	column	Compound_ID	MSMS_spectra	ID_level	P-FDR	P	Estimate	P	Estimate	P	Estimate	P	Estimate
487.1979@10.718429	487.1979	10.72	NEG	RP				2.13E-01	7.14E-01	0.12	1.49E-01	0.41	9.59E-03	0.81	5.91E-01	0.16
487.2527@11.429366	487.2527	11.43	NEG	RP				1.59E-01	8.02E-02	0.54	7.86E-02	0.48	1.09E-02	0.73	4.22E-03	0.75
487.2609@8.925028	487.2609	8.93	POS	RP				7.61E-01	2.93E-01	0.31	3.93E-01	0.23	6.97E-01	-0.10	5.38E-01	0.17
487.2687@1.0137498	487.2687	1.01	POS	hilic				8.50E-01	3.56E-01	0.27	7.48E-01	0.09	6.32E-01	-0.11	6.78E-01	0.10
487.2855@12.380536	487.2855	12.38	NEG	RP				3.94E-01	2.28E-01	-0.34	5.09E-01	0.19	8.56E-01	-0.05	1.38E-01	-0.51
487.8019@15.261649	487.8019	15.26	NEG	RP				2.64E-01	4.12E-02	-0.62	2.96E-01	-0.33	6.81E-01	0.11	1.49E-01	-0.38
487.8651@4.495804	487.8651	4.50	POS	hilic				7.70E-01	2.44E-01	0.38	9.25E-01	0.03	8.05E-01	0.06	6.18E-01	-0.14
488.1208@7.7791743	488.1208	7.78	NEG	RP				2.15E-01	1.15E-03	-0.85	5.57E-01	-0.16	3.60E-01	-0.28	5.57E-01	-0.19
488.1211@0.4066037	488.1211	0.41	NEG	hilic				1.51E-01	3.62E-01	0.31	5.78E-02	-0.52	6.68E-02	-0.49	8.47E-01	-0.05
488.1661@7.242787	488.1661	7.24	POS	RP				3.91E-01	2.28E-01	-0.36	3.36E-01	0.26	9.24E-01	-0.03	9.12E-02	-0.41
488.2011@10.723714	488.2011	10.72	NEG	RP				2.97E-01	1.47E-01	0.37	1.26E-02	0.58	5.08E-01	0.13	1.66E-01	0.26
488.2265@0.50155807	488.2265	0.50	POS	hilic				9.05E-01	2.56E-01	0.27	8.73E-01	0.04	6.30E-01	0.11	4.99E-01	0.17
488.2309@11.042643	488.2309	11.04	NEG	RP				6.12E-02	5.41E-03	-0.72	8.75E-01	-0.05	9.84E-01	-0.01	1.03E-02	-0.68
488.2643@11.925111	488.2643	11.93	NEG	RP				3.58E-01	2.05E-01	0.38	3.63E-02	0.55	2.35E-02	0.72	2.08E-01	0.37
488.2898@11.48826	488.2898	11.49	POS	RP				3.40E-01	4.32E-02	0.57	1.92E-02	0.65	9.16E-02	0.52	7.55E-02	0.57
488.3357@9.51366	488.3357	9.51	NEG	RP				3.44E-01	3.55E-02	-0.47	8.76E-01	-0.04	6.60E-01	0.12	3.97E-01	0.24
488.3814@0.42837784	488.3814	0.43	NEG	hilic				3.26E-01	9.66E-03	-0.50	7.03E-01	0.09	3.88E-01	-0.19	8.25E-01	0.06
489.2214@0.85431594	489.2214	0.85	POS	hilic				9.18E-01	7.52E-01	-0.08	3.53E-01	-0.26	5.21E-01	-0.18	4.07E-01	-0.24
489.2509@11.429691	489.2509	11.43	NEG	RP				3.77E-01	1.57E-01	0.45	5.34E-02	0.52	2.05E-02	0.68	8.99E-02	0.53
489.3454@0.7586665	489.3454	0.76	POS	hilic				1.14E-01	1.11E-02	-0.47	2.91E-01	0.26	2.30E-01	0.31	7.21E-01	-0.09
489.9512@7.441774	489.9512	7.44	POS	hilic				7.58E-01	2.99E-01	-0.31	1.77E-01	-0.38	8.85E-01	-0.05	9.96E-01	0.00
490.0994@3.9581046	490.0994	3.96	POS	hilic				7.57E-01	2.64E-01	-0.32	6.91E-01	-0.12	5.73E-01	0.16	7.02E-01	0.13
490.1819@11.043524	490.1819	11.04	NEG	RP				5.37E-01	9.52E-01	0.01	6.02E-02	0.47	4.32E-01	0.18	5.60E-01	0.12
490.1819@8.028754	490.1819	8.03	POS	RP				3.82E-01	4.48E-03	-0.67	5.40E-01	-0.16	6.73E-01	-0.14	9.69E-01	-0.01
490.2533@11.430735	490.2533	11.43	NEG	RP				9.32E-01	6.57E-01	0.12	9.18E-01	-0.02	3.91E-01	0.24	7.39E-01	0.08
490.2865@0.41635048	490.2865	0.42	NEG	hilic				6.14E-01	9.99E-01	0.00	7.61E-02	-0.47	6.90E-01	0.10	7.81E-01	-0.09
490.3633@0.4352581	490.3633	0.44	POS	hilic				2.18E-01	8.79E-01	0.02	1.48E-01	0.29	1.93E-02	0.50	6.31E-02	0.48
490.3663@0.42761362	490.3663	0.43	NEG	hilic				1.37E-01	1.22E-02	-0.59	9.00E-01	0.04	9.52E-01	-0.01	1.84E-01	0.37
491.1014@10.331936	491.1014	10.33	NEG	RP				5.24E-01	6.54E-01	-0.14	1.54E-01	0.41	2.34E-01	0.32	9.98E-01	0.00
491.1189@6.024732	491.1189	6.02	POS	hilic				7.18E-01	1.29E-01	0.48	6.84E-01	0.10	4.38E-01	0.18	4.02E-01	0.22
491.3005@1.3640491	491.3005	1.36	POS	hilic				5.14E-02	4.57E-02	0.66	1.53E-01	-0.36	1.54E-01	0.36	2.42E-01	0.31
491.3944@10.847252	491.3944	10.85	POS	RP				2.40E-01	1.12E-01	-0.39	9.12E-01	-0.03	2.29E-01	-0.28	2.60E-01	0.31
492.114@0.40630364	492.1140	0.41	NEG	hilic				4.83E-01	6.85E-01	0.13	9.87E-01	0.00	8.83E-01	0.03	8.83E-02	0.59
492.3801@0.42691943	492.3801	0.43	NEG	hilic				5.99E-02	1.21E-02	-0.50	2.36E-01	0.29	4.01E-01	-0.20	1.77E-01	0.34
492.3819@0.42974365	492.3819	0.43	POS	hilic				2.18E-01	3.11E-02	-0.39	5.35E-01	0.15	4.77E-01	-0.14	2.42E-01	0.30
493.221@11.212263	493.2210	11.21	POS	RP				2.74E-01	3.47E-02	-0.62	6.41E-01	-0.13	4.73E-02	-0.55	5.55E-01	-0.14
493.3174@1.1858466	493.3174	1.19	POS	hilic				4.85E-01	1.75E-01	0.45	7.85E-01	-0.08	9.77E-02	0.48	4.73E-01	0.21
493.3176@1.332598	493.3176	1.33	POS	hilic				3.92E-01	1.64E-01	0.46	9.72E-01	0.01	3.44E-02	0.61	4.58E-01	0.21
493.3177@9.649972	493.3177	9.65	POS	RP				3.94E-01	1.27E-01	0.49	5.61E-01	-0.17	2.09E-01	0.37	2.87E-01	0.30
493.318@9.786307	493.3180	9.79	POS	RP	LysoPC 16:1	20 eV: 184.0727 (100), 104.1071 (100), 494.3240 (50)	1	3.82E-01	1.26E-01	0.49	6.02E-01	-0.16	1.80E-01	0.39	2.21E-01	0.34
493.3537@1.0425581	493.3537	1.04	POS	hilic				4.32E-01	1.05E-01	0.45	2.19E-01	0.31	8.57E-01	-0.04	9.76E-01	-0.01
494.1637@1.7363939	494.1637	1.74	NEG	hilic				3.74E-01	4.65E-01	0.18	2.13E-02	0.52	5.00E-01	0.14	8.99E-01	0.03
494.2508@8.676929	494.2508	8.68	NEG	RP				3.50E-01	8.90E-01	0.03	4.19E-01	0.17	2.68E-02	-0.28	3.02E-01	0.23
494.3968@0.42668346	494.3968	0.43	NEG	hilic				1.33E-03	2.21E-02	-0.57	1.79E-03	0.80	1.80E-01	0.37	7.09E-02	0.45
494.397@10.782781	494.3970	10.78	NEG	RP				2.42E-01	4.82E-02	-0.37	2.34E-01	0.32	5.15E-01	-0.13	5.55E-01	0.15
495.1126@10.57265	495.1126	10.57	NEG	RP				1.19E-02	7.38E-03	-0.72	1.42E-01	0.41	1.27E-01	0.39	7.86E-01	-0.08
495.2349@10.965001	495.2349	10.97	NEG	RP				1.95E-02	1.89E-03	0.88	3.41E-01	-0.29	5.11E-01	-0.15	7.97E-01	-0.07
495.3302@10.325333	495.3302	10.33	NEG	RP				3.33E-03	5.10E-03	0.79	4.34E-02	-0.39	2.68E-01	-0.24	4.67E-01	0.19
495.333@1.1584961	495.3330	1.16	POS	hilic				6.22E-01	4.28E-01	-0.22	1.03E-01	-0.46	3.37E-01	-0.29	9.62E-01	0.01
495.3332@10.110489	495.3332	10.11	POS	RP	LysoPC 16:0	20 eV: 184.0736 (100), 496.3433 (5)	2	7.20E-01	1.65E-01	-0.43	3.16E-01	-0.30	3.01E-01	-0.31	1.49E-01	-0.45
495.3332@1.2960688	495.3332	1.30	POS	hilic				9.98E-01	9.77E-01	-0.01	9.35E-01	-0.02	7.05E-01	-0.10	8.99E-01	-0.04
495.3333@10.003035	495.3333	10.00	POS	RP				9.92E-01	7.86E-01	0.09	9.22E-01	0.02	7.19E-01	0.09	8.13E-01	-0.05
495.3342@11.029595	495.3342	11.03	POS	RP				1.66E-01	5.22E-03	0.80	8.90E-01	0.04	2.25E-01	0.34	7.05E-02	0.55
495.4126@10.5932665	495.4126	10.59	POS	RP				2.23E-01	1.24E-01	0.46	1.50E-01	0.42	8.60E-03	0.84	3.96E-01	0.27
495.4131@0.43035948	495.4131	0.43	POS	hilic				6.90E-01	6.96E-01	-0.11	2.98E-01	0.27	9.25E-01	-0.03	2.73E-01	0.33
495.9759@8.398429	495.9759	8.40	NEG	RP				1.16E-01	6.34E-03	-0.81	3.11E-02	-0.73	1.49E-01	-0.40	8.79E-03	-0.73
496.158@10.580973	496.1580	10.58	NEG	RP				2.96E-01	7.33E-03	-0.63	7.79E-01	0.08	9.31E-01	-0.03	6.16E-01	-0.13
496.2524@5.791831	496.2524	5.79	POS	RP				8.29E-01	3.31E-01	-0.27	5.64E-01	-0.17	3.56E-01	-0.26	1.69E-01	-0.42
496.2561@0.46880007	496.2561	0.47	POS	hilic				7.97E-01	8.64E-02	-0.32	9.16E-01	-0.02	4.14E-01	-0.18	8.96E-01	-0.03
496.2849@9.399901	496.2849	9.40	NEG	RP				6.35E-03	1.10E-02	0.69	9.80E-01	0.00	6.35E-03	-0.34	3.20E-01	0.23
496.3028@8.685936	496.3028	8.69	NEG	RP				4.15E-01	3.93E-01	-0.21	4.14E-01	0.22	4.64E-01	0.21	1.75E-01	-0.33
496.3243@0.42204544	496.3243	0.42	NEG	hilic				9.72E-01	9.74E-01	-0.01	8.98E-01	-0.03	4.78E-01	-0.16	9.14E-01	0.03
496.3298@12.38231	496.3298	12.38	NEG	RP				7.61E-01	2.25E-01	0.33	2.42E-01	0.30	7.48E-01	-0.10	7.90E-01	0.09
496.3423@10.792999	496.3423	10.79	POS	RP				8.94E-01	5.29E-01	-0.14	3.76E-01	-0.19	2.50E-01	-0.25	5.54E-01	-0.13
496.4109@0.4231463	496.4109	0.42	NEG	hilic				2.87E-02	3.90E-01	-0.12	5.43E-03	0.72	1.23E-01	0.38	3.83E-02	0.39
497.0682@3.9618933	497.0682	3.96	POS	hilic				3.96E-01	1.29E-01	-0.41	4.32E-01	0.23	1.57E-01	-0.36	7.42E-01	-0.10
497.1289@10.788618	497.1289	10.79	NEG	RP				2.16E-01	3.58E-01	-0.28	4.61E-02	0.59	2.88E-01	0.32	6.39E-01	0.13
497.2318@10.961205	497.2318	10.96	NEG	RP				2.70E-02	1.19E-02	0.73	1.79E-01	-0.39	1.95E-01	-0.33	8.81E-01	-0.04
497.2511@2.0043626	497.2511	2.00	POS	hilic				6.04E-04	2.39E-03	0.89	4.98E-02	-0.47	4.06E-02	-0.55	5.67E-01	0.17
497.7942@0.45245096	497.7942	0.45	POS	hilic				8.15E-01	6.76E-01	-0.11	1.50E-01	-0.34	7.18E-01	-0.09	7.97E-01	-0.07
497.8943@4.351487	497.8943	4.35	POS	hilic				7.98E-01	1.31E-01	0.38	3.00E-01	0.25	4.10E-01	0.27	9.89E-01	0.00
498.1227@7.190673	498.1227	7.19	NEG	RP				5.69E-01	7.71E-01	-0.10	9.61E-02	0.43	4.79E-01	0.22	6.33E-01	-0.12
498.4287@12.694001	498.4287	12.69	POS	RP				4.02E-01	6.99E-01	0.11	1.81E-01	0.37	2.27E-01	-0.34	5.23E-01	-0.20

Supplementary table 2: Metabolomics analysis results

Compound	Mass	Retention Time	Ionization mode	column	Compound_ID	MSMS_spectra	ID_level	ANOVA P-FDR	Anorexia vs control P	Estimate	overweight vs control P	Estimate	obese vs control P	Estimate	athletes vs control P	Estimate
498.4288@0.41145316	498.4288	0.41	POS	hilic				2.39E-03	3.88E-03	-0.71	1.13E-02	0.71	8.04E-01	0.06	1.75E-01	0.39
499.0383@6.140127	499.0383	6.14	POS	hilic				1.30E-01	2.79E-01	-0.31	2.51E-01	-0.29	4.25E-03	-0.68	6.45E-01	0.13
499.1453@11.061896	499.1453	11.06	NEG	RP				2.24E-01	1.98E-02	0.74	9.06E-01	0.03	4.98E-01	0.18	8.63E-02	0.48
499.183@10.6591425	499.1830	10.66	NEG	RP				4.36E-02	5.87E-02	0.54	1.16E-01	-0.36	1.69E-01	-0.32	8.23E-01	0.06
499.1852@10.964037	499.1852	10.96	NEG	RP				7.89E-03	1.53E-03	0.86	2.29E-01	-0.33	1.87E-01	-0.36	9.37E-01	-0.02
499.1857@10.395767	499.1857	10.40	NEG	RP				3.66E-01	1.37E-01	0.47	6.28E-01	-0.15	4.93E-01	-0.21	3.25E-01	0.29
499.2326@11.429641	499.2326	11.43	NEG	RP				4.22E-01	2.23E-01	0.38	8.93E-02	0.47	3.81E-02	0.65	9.53E-02	0.54
499.2692@9.717826	499.2692	9.72	NEG	RP				1.12E-01	1.18E-02	0.83	6.93E-01	-0.11	4.52E-01	0.22	9.87E-01	0.00
499.2708@9.711667	499.2708	9.71	POS	RP				7.36E-02	5.21E-03	0.90	8.35E-01	-0.05	7.89E-01	0.09	9.52E-01	0.01
499.2955@9.393087	499.2955	9.39	NEG	RP				8.49E-01	2.26E-01	0.35	3.81E-01	0.25	7.01E-01	0.10	8.19E-01	0.06
499.296@9.163962	499.2960	9.16	NEG	RP				6.94E-01	2.32E-01	0.39	1.45E-01	0.43	8.33E-01	0.06	5.13E-01	0.19
499.2961@1.5354035	499.2961	1.54	NEG	hilic				5.16E-01	2.35E-01	0.32	2.15E-01	0.32	5.06E-01	-0.16	9.70E-01	0.01
499.2974@1.4311862	499.2974	1.43	POS	hilic				5.87E-01	2.38E-01	0.32	2.54E-01	0.29	6.93E-01	-0.10	8.53E-01	-0.04
499.9362@0.44097096	499.9362	0.44	NEG	hilic				6.43E-02	4.13E-01	-0.22	3.04E-03	-0.82	2.54E-02	-0.74	3.01E-02	-0.64
499.9366@8.9673605	499.9366	8.97	NEG	RP				1.62E-01	5.09E-01	-0.19	8.33E-01	0.05	6.80E-03	-0.73	7.25E-01	-0.11
500.0946@8.631375	500.0946	8.63	NEG	RP				7.87E-01	3.13E-01	0.36	9.75E-01	-0.01	3.36E-01	0.26	8.90E-01	-0.04
500.1387@7.946218	500.1387	7.95	NEG	RP				8.19E-01	3.31E-01	-0.27	7.20E-01	-0.09	9.88E-01	0.00	4.96E-01	0.19
500.193@5.1790476	500.1930	5.18	POS	hilic				3.30E-01	1.70E-01	-0.34	4.76E-01	0.19	1.14E-01	0.40	3.69E-01	0.26
500.3894@0.4223403	500.3894	0.42	POS	hilic				3.38E-02	1.05E-01	-0.40	4.43E-02	0.57	8.50E-01	0.05	7.03E-02	0.53
500.39@12.804012	500.3900	12.80	POS	RP				6.34E-01	5.02E-01	0.19	8.40E-01	0.06	1.59E-01	-0.39	5.48E-01	-0.19
501.0788@6.684963	501.0788	6.68	POS	hilic				9.22E-01	9.83E-01	0.01	6.36E-01	-0.12	4.71E-01	0.20	8.96E-01	0.04
501.1793@10.712744	501.1793	10.71	NEG	RP				7.13E-01	1.99E-01	0.40	1.74E-01	0.40	1.83E-01	0.42	4.53E-01	0.20
501.2841@1.741198	501.2841	1.74	NEG	hilic				7.99E-01	3.07E-01	0.33	9.09E-01	0.03	8.83E-01	-0.05	3.18E-01	0.28
501.2845@9.870385	501.2845	9.87	NEG	RP				8.62E-01	5.31E-01	0.20	8.13E-01	0.07	6.29E-01	-0.15	4.46E-01	0.23
501.2846@9.967106	501.2846	9.97	NEG	RP	LysoPE 20:4	10 eV: 500.2758 (100), 303.2326 (50), 196.0373 (15)	2	8.80E-01	3.97E-01	0.30	4.57E-01	0.21	9.26E-01	0.03	2.83E-01	0.30
501.2848@2.0101106	501.2848	2.01	NEG	hilic				7.64E-01	2.16E-01	0.40	8.41E-01	0.06	8.44E-01	-0.06	4.01E-01	0.24
501.2853@1.8190436	501.2853	1.82	POS	hilic				4.54E-01	4.04E-01	0.24	2.95E-01	-0.31	2.83E-01	-0.35	5.85E-01	0.16
501.2857@1.5915769	501.2857	1.59	POS	hilic				2.56E-01	1.23E-01	0.46	4.46E-01	-0.22	2.66E-01	-0.36	5.34E-01	0.18
501.2866@9.8664	501.2866	9.87	POS	RP	LysoPE 20:4	10 eV: 502.2953 (100), 44.0494 (62), 361.2748 (28)	1	3.55E-01	6.33E-02	0.57	7.55E-01	-0.09	7.64E-01	0.09	9.94E-02	0.45
501.2874@9.960119	501.2874	9.96	POS	RP				2.92E-01	2.49E-01	0.41	1.14E-02	0.74	7.93E-02	0.56	2.65E-01	0.31
501.323@1.2703173	501.3230	1.27	POS	hilic				2.00E-01	1.69E-01	-0.43	4.33E-02	-0.53	3.93E-03	-0.74	5.01E-01	-0.21
501.3259@10.416216	501.3259	10.42	POS	RP				8.91E-01	1.90E-01	-0.37	5.55E-01	-0.19	5.62E-01	-0.15	5.55E-01	-0.17
502.1045@0.3973845	502.1045	0.40	NEG	hilic				6.35E-01	7.19E-02	0.54	5.84E-01	0.17	9.34E-01	-0.02	4.79E-01	0.23
502.1424@7.186139	502.1424	7.19	NEG	RP				4.33E-01	8.18E-01	0.06	6.16E-02	0.45	8.75E-02	0.46	7.38E-01	0.08
502.3169@0.5416364	502.3169	0.54	POS	hilic				6.96E-01	1.30E-01	0.45	9.21E-01	-0.02	7.09E-01	0.10	7.61E-01	0.08
502.4007@11.034007	502.4007	11.03	NEG	RP				1.86E-02	1.07E-02	-0.80	2.20E-01	0.29	2.88E-01	-0.27	3.12E-01	0.31
502.4012@0.42167497	502.4012	0.42	NEG	hilic				1.55E-01	1.23E-01	-0.49	1.37E-01	0.41	5.18E-01	-0.20	4.19E-01	0.24
502.402@0.42938635	502.4020	0.43	POS	hilic				1.55E-01	4.73E-01	-0.13	1.66E-01	0.35	8.19E-01	0.05	1.96E-02	0.56
503.2311@11.429681	503.2311	11.43	NEG	RP				6.44E-01	2.00E-01	0.34	6.70E-02	0.46	3.95E-01	0.23	6.37E-01	0.12
503.2767@1.5305806	503.2767	1.53	NEG	hilic				1.61E-01	4.56E-02	0.56	9.16E-01	0.02	1.68E-01	0.37	1.31E-02	0.66
503.2767@9.613526	503.2767	9.61	NEG	RP				2.76E-01	3.04E-01	0.31	8.45E-01	0.05	1.33E-01	0.41	1.41E-02	0.69
503.2981@9.893502	503.2981	9.89	POS	RP				8.71E-03	1.34E-04	1.07	9.17E-02	0.40	6.90E-01	0.11	6.34E-03	0.69
503.2984@2.0406315	503.2984	2.04	NEG	hilic				3.34E-01	1.20E-01	0.47	2.67E-01	-0.31	8.04E-01	-0.07	6.40E-01	0.12
503.2998@10.13366	503.2998	10.13	NEG	RP				5.43E-02	9.03E-03	0.92	3.07E-01	0.28	9.87E-01	0.00	1.60E-02	0.60
503.3@10.140387	503.3000	10.14	NEG	RP				3.54E-01	4.49E-02	0.64	6.79E-01	-0.11	9.04E-01	0.04	8.69E-01	0.05
503.3003@1.3529238	503.3003	1.35	POS	hilic				8.69E-01	3.50E-01	-0.24	8.71E-01	0.04	8.10E-01	0.07	6.34E-01	0.12
503.3018@1.7958957	503.3018	1.80	POS	hilic				8.25E-01	6.28E-01	-0.15	7.30E-01	-0.09	4.80E-01	-0.24	4.38E-01	0.20
503.3031@10.14172	503.3031	10.14	POS	RP				3.92E-01	6.52E-02	0.65	7.95E-01	-0.08	7.99E-01	0.07	5.57E-01	0.16
503.3363@10.4179735	503.3363	10.42	POS	RP				9.01E-01	3.11E-01	-0.28	5.38E-01	-0.22	9.04E-01	0.03	7.32E-01	-0.10
503.3367@1.25786	503.3367	1.26	POS	hilic				6.35E-01	7.33E-02	-0.44	5.01E-01	-0.15	6.37E-01	-0.12	2.06E-01	-0.34
503.337@10.234958	503.3370	10.23	POS	RP				1.62E-01	2.22E-01	0.37	5.49E-01	-0.16	8.08E-02	-0.42	2.62E-01	0.34
503.3374@1.1034445	503.3374	1.10	POS	hilic				2.85E-01	4.94E-01	0.18	1.54E-01	-0.30	6.24E-02	-0.43	5.84E-01	-0.12
503.3553@10.988136	503.3553	10.99	POS	RP				7.76E-01	8.01E-01	-0.07	3.30E-01	-0.28	3.69E-01	-0.24	2.00E-01	-0.44
503.8657@7.9222193	503.8657	7.92	NEG	hilic				6.48E-01	8.93E-02	0.54	4.37E-01	0.21	6.01E-01	0.14	3.01E-01	0.29
503.9653@6.2568445	503.9653	6.26	NEG	hilic				8.04E-01	6.15E-01	-0.14	7.47E-01	-0.11	8.00E-01	-0.07	8.98E-02	-0.43
504.0864@5.245273	504.0864	5.25	NEG	hilic				7.65E-01	3.92E-01	-0.18	6.14E-01	0.12	7.96E-01	0.07	3.26E-01	0.23
504.0868@5.2650876	504.0868	5.27	NEG	hilic				1.53E-01	2.66E-03	-0.76	8.44E-01	-0.05	6.69E-01	-0.12	4.96E-02	-0.56
504.0936@4.115691	504.0936	4.12	NEG	hilic				7.15E-01	2.83E-01	-0.29	4.82E-01	0.23	8.99E-01	0.03	5.83E-01	-0.19
504.1146@0.4064235	504.1146	0.41	NEG	hilic				5.21E-01	5.30E-01	0.19	9.04E-01	0.04	1.48E-01	-0.40	3.30E-01	0.24
504.14@0.56429464	504.1400	0.56	NEG	hilic				9.60E-03	2.15E-04	-1.07	7.88E-01	-0.06	5.18E-01	0.18	6.04E-01	-0.16
504.4161@0.41997233	504.4161	0.42	NEG	hilic				2.37E-01	3.94E-01	-0.26	2.32E-02	0.60	7.87E-01	0.08	5.85E-01	0.15
504.4171@11.132166	504.4171	11.13	NEG	RP				8.81E-02	5.57E-01	-0.13	2.64E-02	0.60	7.07E-01	-0.09	4.55E-01	-0.19
504.4173@0.4272242	504.4173	0.43	POS	hilic				1.06E-01	7.47E-01	0.06	5.09E-03	0.74	7.46E-02	0.43	1.37E-01	0.35
505.1889@10.514865	505.1889	10.51	NEG	RP				3.06E-01	1.67E-01	-0.33	1.64E-01	0.36	2.69E-01	0.29	8.73E-01	0.05
505.2525@9.616598	505.2525	9.62	POS	RP				4.05E-01	3.01E-01	0.34	6.44E-01	-0.12	1.55E-01	0.38	1.09E-01	0.50
505.2557@0.4675455	505.2557	0.47	POS	hilic				3.02E-01	2.16E-02	-0.45	9.58E-01	-0.01	5.92E-01	0.14	1.77E-01	-0.30
505.2579@1.3963789	505.2579	1.40	POS	hilic				2.81E-01	7.16E-02	0.55	8.13E-01	-0.06	9.67E-02	0.46	1.17E-01	0.52
505.2638@10.962829	505.2638	10.96	NEG	RP				1.01E-02	7.37E-04	0.94	3.82E-01	-0.27	3.52E-01	-0.22	8.78E-01	0.05
505.3147@9.852008	505.3147	9.85	NEG	RP				1.57E-01	5.88E-01	-0.14	3.46E-01	-0.31	5.19E-03	-0.83	9.54E-01	-0.02
505.3156@1.1717429	505.3156	1.17	POS	hilic				4.22E-01	5.23E-01	0.21	7.90E-02	-0.44	7.05E-01	-0.10	5.68E-01	0.18
505.3157@9.960537	505.3157	9.96	NEG	RP				5.62E-03	8.71E-01	0.04	2.18E-02	-0.70	5.05E-04	-0.92	6.01E-01	0.16
505.3474@1.3369411	505.3474	1.34	POS	hilic				8.66E-01	4.64E-01	0.23	7.33E-01	-0.10	7.19E-01	-0.08	6.35E-01	-0.14
505.3515@1.0451207	505.3515	1.05	POS	hilic				5.70E-01	2.18E-01	-0.37	2.36E-01	-0.31	8.40E-02	-0.44	8.31E-01	-0.06

Supplementary table 2: Metabolomics analysis results

								ANOVA	Anorexia vs control		overweight vs control		obese vs control		athletes vs control	
Compound	Mass	Retention Time	Ionization mode	column	Compound_ID	MSMS_spectra	ID_level	P-FDR	P	Estimate	P	Estimate	P	Estimate	P	Estimate
505.352@1.2087353	505.3520	1.21	POS	hilic				2.63E-01	1.43E-01	0.50	3.09E-01	-0.27	9.29E-01	-0.02	2.90E-01	0.33
505.3533@10.416085	505.3533	10.42	POS	RP				3.41E-01	6.20E-01	-0.15	2.15E-01	-0.36	2.58E-02	-0.52	5.34E-01	0.17
505.3539@10.510229	505.3539	10.51	POS	RP				8.85E-01	3.19E-01	0.30	8.86E-01	0.04	7.68E-01	-0.07	7.43E-01	0.11
506.0819@6.0294876	506.0819	6.03	POS	hilic				4.41E-01	1.23E-01	-0.42	3.71E-02	-0.50	5.75E-01	-0.14	3.57E-01	-0.23
506.1285@0.4064175	506.1285	0.41	NEG	hilic				9.63E-01	5.17E-01	0.22	6.29E-01	0.14	8.98E-01	-0.03	7.62E-01	0.08
506.1295@8.159844	506.1295	8.16	NEG	RP				4.85E-01	7.93E-01	-0.06	2.48E-01	-0.25	2.90E-01	0.27	5.14E-01	-0.14
506.1555@0.56887347	506.1555	0.57	NEG	hilic				1.86E-01	3.03E-02	-0.51	3.27E-01	-0.24	5.39E-01	0.18	3.26E-01	0.25
506.185@11.050831	506.1850	11.05	NEG	RP				4.60E-01	1.80E-02	0.61	3.05E-01	0.25	4.75E-01	0.20	4.80E-01	0.16
507.2063@10.641598	507.2063	10.64	NEG	RP				6.97E-01	2.80E-01	0.34	1.74E-01	0.32	8.40E-01	-0.04	4.54E-01	0.18
507.3313@1.3290833	507.3313	1.33	NEG	hilic				7.65E-02	6.44E-02	0.56	1.31E-01	-0.34	4.01E-01	-0.20	9.45E-01	-0.02
507.3315@10.228367	507.3315	10.23	NEG	RP				5.45E-03	2.35E-02	0.69	1.00E-01	-0.51	4.57E-02	-0.52	5.56E-01	0.16
507.3318@10.567456	507.3318	10.57	NEG	RP				1.54E-01	6.31E-01	0.13	2.92E-01	-0.20	2.46E-02	-0.38	2.13E-01	0.31
507.3323@9.9504385	507.3323	9.95	POS	RP				3.61E-01	9.12E-01	0.04	4.24E-01	0.24	2.52E-01	-0.31	7.30E-02	-0.47
507.3326@1.1501664	507.3326	1.15	POS	hilic				7.61E-01	4.51E-01	-0.22	1.67E-01	-0.36	1.36E-01	-0.39	4.91E-01	-0.20
507.3334@10.021551	507.3334	10.02	POS	RP	LysoPC 17:1	20 eV: 184.0719 (100), 508.3376 (48), 104.1066 (43)	2	5.64E-02	8.68E-03	0.82	3.89E-01	-0.27	6.64E-01	0.11	2.96E-01	0.28
507.3337@1.2703112	507.3337	1.27	POS	hilic				4.29E-02	3.22E-01	0.29	6.90E-02	-0.56	2.16E-02	-0.62	6.98E-01	0.13
507.3686@1.0308573	507.3686	1.03	POS	hilic				1.55E-01	9.36E-01	0.02	4.67E-01	-0.19	2.38E-03	-0.78	5.75E-01	-0.18
507.3691@10.496908	507.3691	10.50	POS	RP				2.83E-01	1.57E-01	0.50	2.77E-01	-0.35	6.19E-01	-0.13	6.62E-01	0.12
507.3693@10.700334	507.3693	10.70	POS	RP				4.43E-01	8.68E-01	-0.04	8.80E-01	0.03	1.88E-01	0.27	9.09E-02	0.46
508.302@11.069611	508.3020	11.07	POS	RP				7.86E-01	3.37E-01	-0.26	8.03E-01	0.07	5.47E-01	0.17	6.60E-01	-0.12
508.3744@0.4360417	508.3744	0.44	NEG	hilic				4.48E-02	4.63E-02	-0.34	2.50E-01	0.28	6.09E-01	0.11	2.18E-02	0.59
508.3753@0.43976188	508.3753	0.44	POS	hilic				2.02E-01	6.86E-03	-0.46	8.47E-01	0.05	2.69E-01	-0.23	4.23E-01	0.20
509.107@10.734969	509.1070	10.73	NEG	RP				3.72E-01	8.14E-01	0.05	1.33E-01	0.33	2.97E-02	0.50	6.50E-01	0.10
509.2136@10.96288	509.2136	10.96	NEG	RP				2.42E-01	1.13E-02	0.75	9.51E-01	-0.02	9.09E-01	-0.03	6.11E-01	0.14
509.2475@0.5003965	509.2475	0.50	POS	hilic				8.14E-01	3.45E-01	-0.24	3.75E-01	-0.23	9.78E-01	-0.01	2.18E-01	-0.29
509.3472@10.502092	509.3472	10.50	NEG	RP				9.32E-02	1.81E-01	0.34	1.88E-01	-0.43	8.41E-02	-0.50	3.99E-01	0.22
509.3475@10.453053	509.3475	10.45	NEG	RP				6.05E-01	4.95E-01	0.19	4.81E-01	0.21	3.58E-01	-0.29	3.68E-01	0.26
509.3484@1.1215001	509.3484	1.12	POS	hilic				2.76E-02	7.02E-02	0.53	8.19E-02	-0.52	4.46E-02	-0.51	8.63E-01	0.06
509.3485@1.252198	509.3485	1.25	POS	hilic				1.97E-02	9.69E-02	0.44	4.17E-02	-0.61	1.44E-02	-0.60	9.91E-01	0.00
509.3488@10.260061	509.3488	10.26	POS	RP				4.05E-01	3.31E-01	0.35	4.46E-01	-0.20	1.69E-01	0.41	2.40E-01	0.38
509.349@10.33326	509.3490	10.33	POS	RP				4.33E-01	3.08E-02	0.72	5.93E-01	0.16	2.94E-01	0.27	2.01E-01	0.36
509.3493@10.30853	509.3493	10.31	POS	RP	LysoPC 17:0	10 eV: 510.3556 (100), 184.0736 (13)	2	2.10E-01	1.28E-01	0.50	8.89E-01	0.04	6.56E-01	0.11	1.08E-01	-0.45
509.3828@10.788109	509.3828	10.79	POS	RP				7.67E-01	3.01E-01	-0.31	2.21E-01	-0.33	1.50E-01	-0.38	5.28E-01	-0.18
510.1231@0.40556425	510.1231	0.41	NEG	hilic				1.49E-01	1.48E-01	0.40	2.30E-01	-0.37	4.98E-02	-0.54	6.31E-01	-0.15
510.1231@8.631547	510.1231	8.63	NEG	RP				9.74E-01	6.44E-01	-0.15	8.53E-01	0.05	6.79E-01	-0.12	9.02E-01	0.04
510.1232@8.631547	510.1232	8.63	NEG	RP				9.84E-01	6.56E-01	-0.15	8.64E-01	0.05	9.16E-01	0.03	8.61E-01	0.06
510.1237@8.929864	510.1237	8.93	NEG	RP				5.38E-01	6.95E-01	-0.12	2.94E-01	0.28	3.27E-01	-0.26	4.09E-01	0.23
510.2899@11.069833	510.2899	11.07	POS	RP				7.35E-01	4.03E-01	-0.25	6.42E-01	0.14	4.97E-01	0.22	5.80E-01	-0.17
510.3371@0.42422387	510.3371	0.42	NEG	hilic				4.37E-01	5.85E-02	0.51	4.00E-02	0.59	3.04E-01	0.29	7.95E-01	0.08
511.1587@10.7455	511.1587	10.75	NEG	RP				9.03E-01	3.29E-01	0.24	8.39E-01	-0.05	8.69E-01	-0.04	7.79E-01	0.07
511.2434@2.2133749	511.2434	2.21	NEG	hilic				9.93E-05	2.05E-03	0.85	1.72E-01	-0.12	1.72E-01	-0.12	2.31E-01	0.23
511.2594@1.2125313	511.2594	1.21	NEG	hilic				7.42E-01	8.86E-01	-0.04	1.85E-01	0.36	3.12E-01	0.25	6.46E-01	0.11
511.2602@8.2357435	511.2602	8.24	NEG	RP				8.75E-01	9.69E-01	0.01	7.82E-01	0.07	2.14E-01	0.33	5.98E-01	0.14
511.2655@0.4761112	511.2655	0.48	POS	hilic				9.69E-01	8.50E-01	-0.06	9.65E-01	-0.01	8.26E-01	0.07	4.74E-01	-0.19
511.2787@8.670666	511.2787	8.67	POS	RP				3.35E-01	7.54E-01	0.06	1.73E-01	0.27	9.31E-02	-0.18	2.22E-01	0.26
511.326@1.6016072	511.3260	1.60	POS	hilic				5.97E-01	7.51E-02	0.52	8.57E-01	0.05	9.71E-01	0.01	5.73E-01	0.16
511.3272@8.418071	511.3272	8.42	POS	RP				1.05E-01	3.91E-02	0.64	3.10E-01	-0.28	3.03E-01	-0.30	7.49E-01	0.09
511.3274@9.738915	511.3274	9.74	POS	RP				9.86E-02	1.15E-02	0.74	9.28E-01	0.03	2.97E-01	-0.28	2.90E-01	0.31
511.3278@1.8106581	511.3278	1.81	POS	hilic				1.53E-01	1.80E-02	0.65	4.22E-01	-0.25	3.90E-01	-0.23	9.19E-01	-0.03
511.3279@8.576522	511.3279	8.58	POS	RP				1.27E-01	4.70E-02	0.60	2.75E-01	-0.30	3.27E-01	-0.29	6.14E-01	0.14
511.3418@0.42501375	511.3418	0.43	NEG	hilic				8.60E-01	1.63E-01	0.38	5.85E-01	0.14	6.55E-01	0.12	6.49E-01	0.12
511.3553@10.338031	511.3553	10.34	POS	RP				9.28E-02	3.82E-02	0.63	2.37E-01	-0.32	4.25E-01	-0.19	4.48E-01	0.21
511.4073@10.187343	511.4073	10.19	POS	RP				7.35E-01	5.64E-01	0.19	4.07E-01	0.26	9.64E-02	0.50	7.15E-01	0.10
511.4081@0.44056702	511.4081	0.44	POS	hilic				7.96E-01	6.80E-01	0.12	5.81E-01	0.15	2.93E-01	0.30	2.10E-01	0.41
511.8872@4.654289	511.8872	4.65	NEG	hilic				1.55E-01	2.18E-01	0.39	3.03E-01	0.29	3.01E-01	-0.27	1.83E-02	0.62
512.1191@10.580745	512.1191	10.58	NEG	RP				7.22E-01	1.95E-01	-0.35	6.60E-01	-0.12	6.13E-01	0.15	5.12E-01	-0.19
512.2974@8.470846	512.2974	8.47	NEG	RP				6.67E-01	3.88E-01	0.24	9.51E-01	-0.02	2.71E-01	0.32	4.84E-01	-0.21
513.2096@10.788284	513.2096	10.79	NEG	RP				6.79E-01	1.67E-01	-0.36	6.48E-01	0.11	7.32E-01	0.09	6.23E-01	-0.14
513.2752@9.044064	513.2752	9.04	NEG	RP				4.41E-01	8.61E-01	0.06	7.38E-01	0.09	5.93E-02	-0.53	7.39E-01	0.09
513.2791@5.792165	513.2791	5.79	POS	RP				7.61E-01	3.09E-01	-0.29	7.34E-01	-0.10	4.04E-01	-0.23	1.30E-01	-0.47
513.2797@0.46888006	513.2797	0.47	POS	hilic				7.27E-01	7.59E-01	0.09	7.20E-01	-0.10	4.64E-01	-0.19	1.68E-01	-0.43
513.2869@11.923896	513.2869	11.92	NEG	RP				1.20E-01	3.27E-01	0.31	3.71E-02	0.58	1.01E-03	0.89	3.18E-01	0.26
513.3055@1.5237414	513.3055	1.52	NEG	hilic				2.68E-01	7.46E-02	0.54	8.05E-01	0.07	1.05E-01	0.45	4.09E-02	0.67
513.3058@9.46764	513.3058	9.47	NEG	RP				1.04E-01	6.13E-02	0.58	8.99E-01	0.04	6.17E-02	0.53	9.41E-03	0.81
513.3059@9.614343	513.3059	9.61	NEG	RP	LysoPC 14:0 (M+FA)	10 eV: 452.2771 (100), 512.2957 (28), 227.2016 (25)	2	1.11E-01	7.21E-02	0.57	9.02E-01	0.03	5.66E-02	0.55	1.18E-02	0.79
513.3301@8.678905	513.3301	8.68	POS	RP				6.80E-01	9.37E-01	-0.02	4.98E-01	0.17	3.63E-01	0.25	4.07E-01	-0.19
514.0959@7.1889105	514.0959	7.19	NEG	RP				1.76E-01	1.12E-01	-0.50	1.88E-01	0.37	3.13E-01	0.29	7.29E-01	-0.09
514.295@12.575625	514.2950	12.58	NEG	RP				2.97E-01	7.93E-02	0.57	6.81E-02	0.49	2.18E-02	0.70	9.04E-02	0.41
514.406@13.2428	514.4060	13.24	POS	RP				9.69E-01	7.76E-01	0.08	5.05E-01	0.21	9.47E-01	-0.02	9.01E-01	0.03
514.4064@0.39619663	514.4064	0.40	POS	hilic				4.85E-01	1.73E-01	-0.41	1.06E-01	-0.48	9.01E-01	-0.04	9.33E-01	0.02
515.1887@10.8689165	515.1887	10.87	NEG	RP				8.09E-01	2.47E-01	0.31	8.95E-01	0.03	3.66E-01	0.22	2.94E-01	0.26
515.227@11.037439	515.2270	11.04	NEG	RP				4.18E-01	5.21E-01	0.17	7.20E-01	0.10	1.95E-01	0.44	2.96E-01	-0.31
515.2434@1.9520612	515.2434	1.95	POS	hilic				4.83E-02	2.81E-01	0.35	4.48E-02	-0.49	7.21E-02	-0.44	4.97E-01	0.19

Supplementary table 2: Metabolomics analysis results

								ANOVA	Anorexia vs control		overweight vs control		obese vs control		athletes vs control	
Compound	Mass	Retention Time	Ionization mode	column	Compound_ID	MSMS_spectra	ID_level	P-FDR	P	Estimate	P	Estimate	P	Estimate	P	Estimate
515.2821@12.382061	515.2821	12.38	NEG	RP				9.42E-01	9.17E-01	-0.03	5.06E-01	0.16	6.34E-01	-0.15	8.16E-01	-0.08
515.2899@8.439904	515.2899	8.44	NEG	RP				6.34E-01	2.69E-01	0.31	3.81E-01	0.21	5.14E-01	-0.15	8.15E-01	-0.06
515.2989@1.3422267	515.2989	1.34	POS	hilic				5.55E-01	9.73E-01	-0.01	6.13E-01	0.14	9.24E-02	0.52	7.77E-01	-0.08
515.2992@9.790794	515.2992	9.79	POS	RP				2.78E-01	3.01E-02	0.66	9.53E-01	-0.02	2.11E-01	0.39	1.03E-01	0.49
515.2995@9.789861	515.2995	9.79	POS	RP				1.79E-01	5.01E-02	0.65	6.93E-01	-0.12	6.71E-02	0.52	1.58E-01	0.37
515.3037@1.1774918	515.3037	1.18	POS	hilic				2.78E-01	1.04E-01	-0.38	9.76E-01	0.01	2.53E-01	0.32	3.18E-01	-0.25
515.4149@11.68168	515.4149	11.68	POS	RP				4.29E-01	4.48E-01	0.22	7.09E-01	-0.08	9.41E-01	0.02	6.12E-02	0.50
515.4552@12.694266	515.4552	12.69	POS	RP				3.40E-01	6.61E-01	0.12	2.13E-01	0.35	1.58E-01	-0.40	4.72E-01	-0.23
516.1108@7.9449983	516.1108	7.94	NEG	RP				4.02E-01	7.15E-02	-0.41	4.73E-01	-0.18	8.56E-01	0.05	3.70E-01	0.25
516.2946@12.575702	516.2946	12.58	NEG	RP				7.52E-01	3.07E-01	0.28	2.14E-01	0.26	1.08E-01	0.39	3.03E-01	0.25
516.4156@0.42684737	516.4156	0.43	NEG	hilic				3.77E-02	2.65E-02	-0.58	3.10E-01	0.29	3.19E-01	-0.25	1.05E-01	0.46
517.1201@10.436195	517.1201	10.44	NEG	RP				1.11E-03	2.07E-01	-0.24	4.23E-03	0.80	5.46E-03	0.59	7.03E-01	-0.08
517.2805@11.924706	517.2805	11.92	NEG	RP				4.21E-01	1.79E-01	0.41	4.01E-02	0.58	3.70E-02	0.62	1.16E-01	0.45
517.2925@9.889457	517.2925	9.89	NEG	RP				8.14E-02	6.88E-03	0.74	9.84E-01	-0.01	3.81E-02	0.59	1.16E-02	0.65
517.3004@8.944276	517.3004	8.94	NEG	RP				6.41E-01	2.87E-01	0.30	1.94E-01	0.32	6.78E-01	-0.10	5.60E-01	0.15
517.302@1.0771276	517.3020	1.08	NEG	hilic				7.15E-01	2.07E-01	0.34	1.70E-01	0.33	8.76E-01	0.04	4.50E-01	0.18
517.315@10.304795	517.3150	10.30	POS	RP				1.50E-03	2.43E-01	0.32	1.59E-03	-0.83	8.91E-01	0.04	2.64E-02	0.53
517.3155@10.173991	517.3155	10.17	POS	RP				7.36E-01	5.32E-01	-0.18	2.54E-01	0.30	4.44E-01	0.24	5.36E-01	0.19
517.3157@10.171738	517.3157	10.17	POS	RP				6.16E-01	3.17E-01	0.23	6.44E-02	0.50	4.48E-01	0.19	1.80E-01	0.32
517.3158@1.3295382	517.3158	1.33	POS	hilic				1.17E-01	4.34E-02	0.62	1.64E-01	-0.41	6.95E-01	-0.12	8.19E-01	0.08
517.3159@10.750074	517.3159	10.75	POS	RP				7.29E-01	1.84E-01	0.31	4.89E-01	0.18	9.96E-01	0.00	5.93E-01	-0.13
517.3168@9.718786	517.3168	9.72	POS	RP	LysoPC 18:3	20 eV: 184.0734 (100), 518.3254 (75), 104.1072 (74)	2	4.15E-02	6.80E-02	0.61	7.50E-02	-0.50	5.25E-01	-0.16	4.90E-01	0.23
517.3169@10.561231	517.3169	10.56	POS	RP				8.65E-01	2.74E-01	-0.26	7.04E-01	0.09	8.80E-01	0.04	9.25E-01	0.03
517.3176@9.593461	517.3176	9.59	POS	RP				1.37E-04	1.36E-01	0.39	4.85E-03	-0.75	2.08E-03	-0.82	6.13E-01	0.14
517.3183@9.962052	517.3183	9.96	POS	RP				5.76E-01	1.47E-01	0.45	4.73E-01	-0.21	7.53E-01	0.10	9.25E-01	0.03
517.3281@10.381587	517.3281	10.38	NEG	RP				7.98E-01	9.66E-01	0.01	4.49E-01	-0.22	3.68E-01	-0.23	2.48E-01	-0.35
517.3705@10.9870825	517.3705	10.99	POS	RP				6.27E-01	1.21E-01	0.41	5.23E-01	-0.17	7.81E-01	0.07	8.27E-01	0.06
517.4164@12.80333	517.4164	12.80	POS	RP				5.32E-01	4.94E-01	0.19	7.28E-01	0.10	1.34E-01	-0.42	4.73E-01	-0.23
518.2444@0.46975872	518.2444	0.47	POS	hilic				6.14E-01	9.15E-02	-0.41	9.82E-01	-0.01	9.53E-01	0.01	7.29E-01	0.09
518.3967@0.4268316	518.3967	0.43	NEG	hilic				3.67E-03	4.62E-03	-0.81	1.46E-01	0.38	4.21E-01	-0.21	8.77E-02	0.48
518.3971@10.698992	518.3971	10.70	POS	RP				3.54E-01	2.63E-02	-0.53	6.33E-01	0.14	6.38E-01	0.11	5.77E-01	0.17
518.3983@0.43369398	518.3983	0.43	POS	hilic				5.83E-03	1.26E-04	-0.95	2.86E-01	0.29	5.34E-01	-0.15	3.85E-01	0.26
519.1349@10.733354	519.1349	10.73	NEG	RP				3.79E-01	2.66E-01	0.33	1.03E-01	-0.41	4.71E-01	-0.22	8.38E-01	0.05
519.3328@1.2703345	519.3328	1.27	POS	hilic				9.01E-01	3.62E-01	-0.28	8.43E-01	0.06	9.34E-01	0.02	9.05E-01	-0.03
519.333@9.847101	519.3330	9.85	POS	RP				7.68E-01	1.98E-01	-0.35	4.89E-01	-0.18	2.33E-01	-0.33	9.24E-01	0.03
519.3332@9.959968	519.3332	9.96	POS	RP	LysoPC 18:2	10 eV: 520.3417 (100), 184.0743 (76)	1	2.88E-01	4.56E-01	-0.21	9.98E-01	0.00	5.89E-02	-0.57	5.98E-02	-0.59
519.3333@1.1350509	519.3333	1.14	POS	hilic				5.92E-02	5.29E-01	0.18	1.16E-01	-0.47	1.31E-02	-0.66	4.97E-01	0.23
519.3961@11.4338455	519.3961	11.43	POS	RP				4.14E-01	8.90E-01	0.04	5.54E-02	0.52	6.27E-01	-0.13	6.94E-01	-0.12
519.8361@15.256808	519.8361	15.26	NEG	RP				7.85E-01	2.25E-01	-0.31	6.48E-01	-0.12	2.40E-01	-0.28	9.54E-01	-0.01
520.4116@0.42671794	520.4116	0.43	NEG	hilic				6.52E-03	1.96E-02	-0.65	2.32E-02	0.61	9.01E-01	-0.03	8.45E-02	0.44
520.4121@0.43529657	520.4121	0.44	POS	hilic				9.50E-04	3.31E-03	-0.86	3.54E-02	0.57	6.39E-01	0.11	7.66E-02	0.48
520.4126@10.601782	520.4126	10.60	POS	RP				4.48E-02	2.17E-02	-0.71	1.73E-01	0.37	4.05E-01	0.24	7.55E-01	0.09
520.4126@10.699265	520.4126	10.70	POS	RP				3.89E-01	2.01E-02	-0.56	8.54E-01	-0.05	6.89E-01	-0.11	6.17E-01	0.13
520.4132@10.778543	520.4132	10.78	NEG	RP				4.20E-02	7.22E-03	-0.73	4.36E-01	0.21	3.82E-01	-0.23	2.36E-01	0.33
520.4491@12.50818	520.4491	12.51	POS	RP				3.55E-01	5.88E-02	0.52	4.45E-01	0.16	2.37E-02	0.56	2.44E-01	0.32
521.1038@6.269749	521.1038	6.27	POS	hilic				2.42E-01	4.51E-01	0.25	7.16E-01	-0.11	1.82E-01	-0.37	1.27E-01	0.45
521.251@1.7025756	521.2510	1.70	POS	hilic				9.36E-03	2.96E-02	0.62	1.72E-01	-0.25	2.03E-01	-0.25	2.47E-01	0.27
521.2517@1.9551469	521.2517	1.96	POS	hilic				3.78E-01	6.03E-01	0.17	9.09E-02	-0.40	1.09E-01	-0.40	7.78E-01	-0.08
521.2724@2.2118053	521.2724	2.21	NEG	hilic				1.11E-03	7.44E-02	0.57	4.77E-02	-0.43	7.38E-03	-0.60	1.44E-01	0.34
521.2812@1.4322102	521.2812	1.43	POS	hilic				5.10E-01	7.67E-01	0.08	2.68E-01	0.27	3.85E-01	-0.21	3.35E-01	-0.22
521.3472@1.2218463	521.3472	1.22	POS	hilic				7.68E-01	1.63E-01	-0.34	9.22E-01	0.03	5.76E-01	0.17	9.21E-01	-0.03
521.3484@10.12519	521.3484	10.13	POS	RP				3.60E-01	3.33E-01	0.34	2.40E-01	-0.32	2.91E-01	-0.30	4.99E-01	0.20
521.3488@10.233932	521.3488	10.23	POS	RP	LysoPC 18:1	10 eV: 522.3549 (100), 184.0733 (78), 104.1061 (5)	1	7.99E-01	2.62E-01	-0.37	9.39E-01	-0.02	2.36E-01	-0.33	6.69E-01	-0.13
521.3489@1.105992	521.3489	1.11	POS	hilic				2.62E-02	1.28E-01	0.46	6.76E-02	-0.55	1.98E-02	-0.62	7.87E-01	-0.08
521.4165@0.43446586	521.4165	0.43	POS	hilic				5.47E-03	1.81E-03	-0.56	1.36E-02	0.63	3.69E-01	0.23	1.22E-01	0.40
521.5905@10.603144	521.5905	10.60	POS	hilic				4.28E-01	8.51E-03	0.73	1.76E-01	0.38	2.24E-01	0.30	3.16E-01	0.32
521.7878@1.3292918	521.7878	1.33	POS	hilic				4.82E-01	9.17E-01	-0.03	8.58E-02	0.48	1.30E-01	0.48	2.88E-01	0.27
521.7899@10.139275	521.7899	10.14	POS	RP				1.53E-01	1.24E-01	0.48	2.89E-02	0.75	3.66E-02	0.56	5.96E-03	0.80
521.9164@5.412966	521.9164	5.41	NEG	hilic				3.95E-01	6.02E-02	-0.57	1.94E-02	-0.60	2.34E-02	-0.57	1.56E-01	-0.48
521.9165@4.5401034	521.9165	4.54	NEG	hilic				9.01E-01	8.59E-01	0.06	5.97E-01	0.18	8.81E-01	-0.04	4.67E-01	-0.20
521.9166@4.5410347	521.9166	4.54	NEG	hilic				4.44E-01	4.69E-01	0.25	4.86E-01	0.20	1.90E-01	-0.36	3.34E-01	-0.26
522.12@0.40665305	522.1200	0.41	NEG	hilic				2.97E-01	7.72E-01	0.06	2.91E-02	0.52	4.07E-02	0.47	4.26E-01	0.19
522.329@12.383544	522.3290	12.38	NEG	RP				3.56E-01	1.13E-02	0.63	1.50E-01	0.41	6.29E-01	0.11	2.02E-01	0.32
522.4272@0.42868575	522.4272	0.43	POS	hilic				1.28E-02	2.36E-01	-0.22	4.34E-03	0.78	7.28E-02	0.45	4.43E-02	0.50
522.4274@0.42617112	522.4274	0.43	NEG	hilic				2.44E-03	4.40E-02	-0.53	3.42E-03	0.80	2.22E-01	0.34	4.07E-02	0.49
522.4657@13.118946	522.4657	13.12	POS	RP				5.30E-01	1.36E-01	0.46	5.77E-01	0.14	3.34E-02	0.56	4.18E-01	0.26
522.9203@4.528642	522.9203	4.53	NEG	hilic				5.56E-01	7.25E-02	0.54	9.05E-01	-0.04	6.17E-01	0.15	2.70E-01	0.32
523.1968@10.815056	523.1968	10.82	NEG	RP				8.97E-01	7.17E-01	-0.11	4.98E-01	-0.17	2.15E-01	-0.30	7.49E-01	-0.08
523.2614@0.49786878	523.2614	0.50	POS	hilic				7.35E-01	9.34E-01	-0.02	2.60E-01	0.29	1.79E-01	0.33	8.24E-01	0.06
523.2629@6.723674	523.2629	6.72	POS	RP				3.85E-01	5.00E-01	0.23	5.86E-02	0.45	5.84E-02	0.54	7.60E-01	-0.08
523.2639@0.4266313	523.2639	0.43	POS	hilic				2.40E-01	1.28E-02	0.85	2.45E-01	0.28	1.24E-01	0.47	8.69E-02	0.43
523.2674@1.8222507	523.2674	1.82	POS	hilic				9.82E-01	7.51E-01	0.10	7.38E-01	-0.10	7.93E-01	-0.09	8.52E-01	0.05
523.2677@1.5909727	523.2677	1.59	POS	hilic				7.49E-01	4.67E-01	0.23	9.39E-01	-0.02	4.44E-01	-0.25	4.52E-01	0.21

Supplementary table 2: Metabolomics analysis results

Compound	Mass	Retention Time	Ionization mode	column	Compound_ID	MSMS_spectra	ID_level	ANOVA P-FDR	Anorexia vs control P	Anorexia vs control Estimate	overweight vs control P	overweight vs control Estimate	obese vs control P	obese vs control Estimate	athletes vs control P	athletes vs control Estimate
523.2948@10.104432	523.2948	10.10	POS	RP				6.24E-01	1.39E-01	-0.37	1.20E-01	-0.38	3.61E-01	-0.23	9.80E-01	0.01
523.3637@1.2099396	523.3637	1.21	POS	hilic				7.82E-01	1.22E-01	-0.36	7.46E-01	-0.08	6.91E-01	-0.11	6.96E-01	0.12
523.3641@10.419394	523.3641	10.42	POS	RP				2.00E-01	2.18E-01	0.41	7.62E-02	-0.52	6.95E-01	-0.09	9.96E-01	0.00
523.3643@1.0862727	523.3643	1.09	POS	hilic				1.15E-01	8.65E-02	0.53	9.70E-02	-0.50	5.00E-01	-0.18	9.95E-01	0.00
523.3644@10.514791	523.3644	10.51	POS	RP	LysoPC 18:0	10 eV: 524.3713 (100), 184.0745 (62)	1	5.04E-01	4.62E-01	-0.24	8.72E-02	-0.45	5.09E-02	-0.52	8.22E-01	-0.07
523.3647@10.522354	523.3647	10.52	POS	RP				9.43E-01	5.03E-01	0.20	5.48E-01	0.17	3.18E-01	0.29	5.63E-01	0.16
523.3648@10.692446	523.3648	10.69	POS	RP				5.29E-01	4.05E-01	0.21	9.21E-01	-0.03	9.57E-02	-0.35	3.75E-01	-0.21
524.1468@2.4632897	524.1468	2.46	NEG	RP				3.89E-01	1.36E-01	-0.36	3.77E-01	0.25	9.13E-01	0.03	2.98E-01	0.26
524.4431@0.42485186	524.4431	0.42	NEG	hilic				1.47E-02	6.54E-01	-0.04	1.61E-03	0.72	7.69E-02	0.41	1.49E-02	0.45
524.7948@10.127208	524.7948	10.13	POS	RP				9.40E-01	8.00E-01	0.07	4.57E-01	0.19	7.59E-01	-0.07	6.53E-01	0.12
525.1764@10.582892	525.1764	10.58	NEG	RP				1.26E-01	4.98E-03	-0.77	8.96E-01	-0.04	6.65E-01	0.12	7.36E-01	-0.09
525.1802@10.796762	525.1802	10.80	NEG	RP				8.00E-01	3.62E-01	-0.21	4.84E-01	-0.15	5.16E-01	0.17	9.30E-01	-0.02
525.216@11.093113	525.2160	11.09	NEG	RP				3.37E-01	3.81E-02	-0.65	9.08E-02	-0.52	3.72E-02	-0.56	3.74E-02	-0.61
525.2583@8.9391365	525.2583	8.94	POS	RP				1.72E-01	2.34E-02	-0.51	9.14E-01	0.02	4.73E-02	-0.46	8.79E-01	0.04
525.2843@1.5678881	525.2843	1.57	POS	hilic				9.42E-01	2.69E-01	-0.32	6.51E-01	-0.12	7.68E-01	-0.10	7.80E-01	-0.08
525.2845@1.9746065	525.2845	1.97	NEG	hilic				7.76E-01	1.47E-01	-0.42	9.17E-01	0.03	9.26E-01	-0.03	9.25E-01	0.03
525.2846@1.7166935	525.2846	1.72	NEG	hilic				9.51E-01	6.06E-01	-0.13	6.29E-01	-0.11	9.68E-01	-0.01	6.52E-01	0.11
525.2847@9.956956	525.2847	9.96	NEG	RP	LysoPE 22:6	10 eV: 524.2778 (100), 327.2317 (25), 196.0392 (5)	2	5.78E-01	7.84E-02	-0.53	9.62E-01	0.01	9.53E-01	0.02	8.58E-01	0.05
525.285@9.866127	525.2850	9.87	NEG	RP				5.08E-01	4.87E-02	-0.59	7.32E-01	-0.09	8.62E-01	0.06	8.80E-01	-0.04
525.2855@1.7903764	525.2855	1.79	POS	hilic				8.86E-01	1.81E-01	-0.37	6.32E-01	-0.13	7.71E-01	-0.10	9.22E-01	-0.03
525.2868@9.92798	525.2868	9.93	POS	RP				4.64E-01	3.46E-01	-0.27	2.61E-01	0.32	2.48E-01	0.37	9.90E-01	0.00
525.287@9.858511	525.2870	9.86	POS	RP				7.64E-01	2.13E-01	-0.35	5.47E-01	-0.16	5.18E-01	-0.23	6.39E-01	0.13
525.3425@1.1841998	525.3425	1.18	NEG	hilic				4.42E-01	7.99E-01	0.07	2.82E-01	-0.33	8.44E-02	-0.40	5.12E-01	0.22
525.3433@10.306142	525.3433	10.31	NEG	RP				5.55E-01	3.00E-01	0.30	7.05E-01	0.11	7.15E-01	-0.09	1.21E-01	0.46
525.8561@1.1080004	525.8561	1.11	POS	hilic				8.48E-01	7.64E-01	-0.09	1.50E-01	-0.37	6.60E-01	-0.12	5.67E-01	-0.17
526.1181@0.4069293	526.1181	0.41	NEG	hilic				6.47E-01	3.38E-01	0.30	2.65E-01	-0.27	8.31E-01	0.06	5.80E-01	-0.18
526.1355@6.0242996	526.1355	6.02	POS	hilic				1.05E-01	5.25E-03	-0.84	1.32E-01	-0.46	7.94E-03	-0.77	1.43E-02	-0.70
526.2184@11.429375	526.2184	11.43	NEG	RP				3.38E-01	8.61E-01	0.05	9.69E-02	0.43	6.82E-02	0.56	8.87E-01	-0.04
526.2763@6.7875	526.2763	6.79	NEG	RP				2.81E-01	3.50E-01	-0.22	2.84E-01	0.28	2.36E-01	0.30	3.12E-01	-0.24
527.1937@10.783374	527.1937	10.78	NEG	RP				8.55E-01	8.90E-01	0.03	2.14E-01	0.32	7.38E-01	0.09	4.54E-01	0.18
527.212@11.039041	527.2120	11.04	NEG	RP				7.23E-01	5.20E-01	-0.18	3.29E-01	0.25	8.48E-01	0.05	4.59E-01	-0.19
527.2667@11.924533	527.2667	11.92	NEG	RP				1.89E-01	1.91E-01	0.39	1.51E-02	0.59	8.01E-03	0.78	7.26E-02	0.46
527.2981@1.9700848	527.2981	1.97	NEG	hilic				3.04E-01	3.68E-02	0.64	7.75E-01	0.06	9.40E-01	0.02	4.13E-01	0.20
527.3003@10.173398	527.3003	10.17	NEG	RP				7.73E-01	8.90E-01	0.05	8.54E-01	-0.05	7.32E-01	-0.11	2.53E-01	0.36
527.3005@10.085997	527.3005	10.09	NEG	RP				2.35E-01	2.26E-02	0.81	8.75E-01	0.04	4.81E-01	0.19	6.94E-01	0.10
527.3007@1.796112	527.3007	1.80	POS	hilic				5.16E-01	1.75E-01	0.43	6.24E-01	-0.13	6.04E-01	-0.15	3.97E-01	0.23
527.3013@10.173611	527.3013	10.17	POS	RP				9.23E-01	9.83E-01	0.01	9.71E-01	0.01	6.11E-01	-0.13	4.99E-01	0.21
527.3015@10.081007	527.3015	10.08	POS	RP				3.14E-01	4.71E-02	0.73	3.22E-01	0.26	6.23E-01	0.13	9.96E-01	0.00
527.3208@9.764566	527.3208	9.76	NEG	RP				4.39E-01	1.24E-01	0.45	8.17E-01	-0.08	8.04E-02	0.50	2.80E-01	0.29
527.321@9.779209	527.3210	9.78	NEG	RP				8.96E-01	4.55E-01	0.19	8.80E-01	0.05	5.31E-01	0.19	2.75E-01	0.33
527.3215@9.888121	527.3215	9.89	NEG	RP	LysoPC 15:0 (M+FA)	10 eV: 466.2942 (100), 526.3146 (25), 241.2165 (12)	2	1.92E-02	1.50E-04	1.04	6.48E-01	0.14	5.24E-02	0.55	3.33E-03	0.77
527.3215@1.4572226	527.3215	1.46	NEG	hilic				2.60E-01	6.14E-03	0.77	2.22E-01	0.31	1.76E-01	0.32	1.33E-01	0.43
527.3573@10.383289	527.3573	10.38	NEG	RP				5.22E-01	9.35E-01	-0.02	1.67E-01	-0.46	4.03E-01	-0.23	5.81E-01	0.18
528.2024@0.5503069	528.2024	0.55	POS	hilic				4.66E-01	3.32E-01	-0.31	4.14E-01	-0.21	1.97E-02	-0.66	4.57E-01	-0.22
528.3793@1.4379413	528.3793	1.44	POS	hilic				8.10E-01	7.00E-01	0.10	4.32E-01	-0.14	9.83E-01	0.00	4.31E-01	0.18
528.4176@0.41864094	528.4176	0.42	NEG	hilic				2.97E-02	2.12E-02	-0.68	2.18E-01	0.29	3.44E-01	-0.28	1.92E-01	0.41
528.4184@13.622424	528.4184	13.62	POS	RP				3.61E-01	6.00E-01	-0.14	2.19E-01	0.32	6.21E-01	-0.13	1.63E-01	-0.45
528.4185@0.40667605	528.4185	0.41	POS	hilic				1.41E-02	1.11E-02	-0.56	2.20E-01	0.32	1.91E-01	-0.29	1.15E-01	0.45
529.2068@11.038532	529.2068	11.04	NEG	RP				5.35E-01	8.98E-02	0.52	1.40E-01	0.47	1.65E-01	0.40	4.97E-02	0.54
529.2699@8.5103655	529.2699	8.51	NEG	RP				3.75E-01	4.80E-01	0.25	1.78E-01	0.35	1.01E-01	-0.37	6.70E-01	0.12
529.27@3.3830922	529.2700	3.38	NEG	hilic				2.77E-01	3.96E-01	0.26	1.59E-01	0.37	1.07E-01	-0.33	7.69E-01	0.07
529.2701@8.354378	529.2701	8.35	NEG	RP				5.22E-01	6.83E-01	0.12	3.15E-02	0.57	6.41E-01	0.12	3.63E-01	0.27
529.2702@3.394304	529.2702	3.39	NEG	hilic				4.67E-01	5.22E-01	0.20	2.01E-01	0.34	1.96E-01	-0.27	6.22E-01	0.12
529.2736@0.53987634	529.2736	0.54	POS	hilic				7.11E-01	5.59E-01	-0.19	2.71E-01	-0.31	8.62E-01	0.05	5.65E-01	0.16
529.275@0.49525288	529.2750	0.50	POS	hilic				3.67E-01	8.30E-01	-0.06	2.22E-01	0.37	3.38E-02	0.54	9.56E-01	0.01
529.2933@9.784111	529.2933	9.78	NEG	RP				4.97E-01	5.19E-01	0.21	4.32E-01	-0.24	1.29E-01	0.44	6.55E-01	0.12
529.3032@0.41539073	529.3032	0.42	POS	hilic				7.61E-01	2.59E-01	0.29	1.16E-01	0.40	6.27E-01	0.13	4.48E-01	0.19
529.3155@1.7608755	529.3155	1.76	POS	hilic				4.54E-01	7.95E-02	0.49	8.27E-01	0.06	9.48E-01	0.02	1.13E-01	0.41
529.3168@10.294953	529.3168	10.29	NEG	RP				7.22E-01	2.07E-01	0.38	9.85E-01	-0.01	5.44E-01	0.17	2.11E-01	0.36
529.317@9.9456	529.3170	9.95	NEG	RP				8.72E-01	3.21E-01	0.21	3.36E-01	0.23	9.87E-01	0.00	6.09E-01	0.12
529.3524@10.663259	529.3524	10.66	POS	RP				8.99E-01	9.47E-01	-0.02	5.70E-01	0.15	6.83E-01	-0.10	5.47E-01	-0.15
529.3526@10.528017	529.3526	10.53	POS	RP				3.65E-01	7.21E-01	0.10	2.34E-02	0.63	1.61E-01	0.42	9.80E-02	0.52
530.188@1.4702119	530.1880	1.47	NEG	hilic				5.07E-01	5.97E-02	-0.51	8.68E-01	0.04	7.30E-01	0.10	4.84E-01	-0.23
530.2101@11.0431385	530.2101	11.04	NEG	RP				7.17E-01	2.32E-01	0.35	1.68E-01	0.34	3.29E-01	0.26	9.78E-02	0.42
530.4013@11.717642	530.4013	11.72	POS	RP				2.40E-01	4.51E-02	0.47	6.48E-02	0.54	4.55E-01	-0.21	6.62E-01	0.14
530.4036@11.162964	530.4036	11.16	POS	RP				9.35E-01	5.54E-01	0.17	6.75E-01	0.12	2.40E-01	0.31	5.88E-01	0.17
530.4328@0.4183394	530.4328	0.42	NEG	hilic				1.32E-01	4.99E-02	-0.54	3.07E-01	0.26	5.64E-01	-0.17	2.24E-01	0.38
530.433@11.260513	530.4330	11.26	NEG	RP				6.67E-01	1.48E-01	-0.29	6.40E-01	-0.11	4.14E-01	0.19	9.16E-01	-0.03
530.4333@11.232657	530.4333	11.23	NEG	RP				1.36E-02	4.81E-03	-0.77	2.29E-01	0.28	9.90E-02	-0.46	4.22E-01	0.26
531.2664@9.78703	531.2664	9.79	POS	RP				6.39E-01	3.60E-01	0.28	4.04E-01	-0.22	8.53E-01	0.05	3.73E-01	-0.26
531.275@1.3338362	531.2750	1.33	POS	hilic				3.87E-01	2.17E-01	0.44	3.61E-01	-0.24	2.20E-01	0.32	8.34E-01	-0.06
531.2764@12.380982	531.2764	12.38	NEG	RP				8.76E-01	2.39E-01	0.32	2.83E-01	0.31	7.85E-01	0.09	7.21E-01	0.11
531.2886@0.5456286	531.2886	0.55	POS	hilic				9.46E-01	4.08E-01	0.24	9.66E-01	-0.01	6.26E-01	0.16	6.49E-01	0.13

Supplementary table 2: Metabolomics analysis results

								ANOVA	Anorexia vs control		overweight vs control		obese vs control		athletes vs control	
Compound	Mass	Retention Time	Ionization mode	column	Compound_ID	MSMS_spectra	ID_level	P-FDR	P	Estimate	P	Estimate	P	Estimate	P	Estimate
531.3077@1.5722665	531.3077	1.57	NEG	hilic				3.50E-02	2.79E-01	0.30	9.82E-02	-0.28	6.11E-02	-0.30	1.19E-01	0.37
531.3081@10.007631	531.3081	10.01	NEG	RP				1.31E-01	5.04E-02	0.68	3.92E-01	0.21	1.29E-02	0.79	2.89E-02	0.62
531.3084@1.2519573	531.3084	1.25	NEG	hilic				1.51E-02	1.95E-03	0.97	8.44E-01	0.05	1.24E-01	0.45	4.79E-03	0.84
531.3084@10.141334	531.3084	10.14	NEG	RP				2.51E-01	9.42E-01	-0.03	1.64E-01	0.42	1.10E-01	0.44	1.21E-02	0.69
531.3086@1.4369742	531.3086	1.44	NEG	hilic				8.21E-02	6.85E-01	0.13	8.21E-02	0.55	2.32E-03	0.78	7.43E-01	-0.09
531.3087@10.142461	531.3087	10.14	NEG	RP				3.48E-01	8.01E-01	0.09	1.84E-01	0.40	7.26E-02	0.45	1.11E-02	0.69
531.3307@10.331458	531.3307	10.33	POS	RP				1.08E-01	1.49E-02	0.69	3.19E-01	-0.31	6.20E-01	-0.12	5.25E-01	0.18
531.3328@1.2387776	531.3328	1.24	POS	hilic				6.66E-01	4.05E-01	0.23	2.73E-01	-0.34	8.88E-01	-0.04	4.98E-01	-0.21
531.4327@13.242819	531.4327	13.24	POS	RP				8.45E-01	4.62E-01	0.24	5.01E-01	0.17	6.88E-01	-0.12	4.69E-01	0.18
531.4335@0.3961112	531.4335	0.40	POS	hilic				9.40E-01	3.75E-01	-0.28	4.61E-01	-0.24	5.66E-01	-0.18	5.19E-01	-0.19
532.2736@9.593668	532.2736	9.59	POS	RP				5.70E-01	1.52E-01	-0.28	5.97E-01	-0.11	5.00E-01	0.17	3.26E-01	-0.22
532.3802@10.56543	532.3802	10.57	POS	RP				4.94E-01	2.02E-01	-0.23	1.46E-01	-0.25	4.59E-02	-0.32	5.14E-01	-0.13
532.4476@0.41821617	532.4476	0.42	NEG	hilic				4.17E-01	4.07E-01	-0.25	8.92E-02	0.46	7.61E-01	0.09	3.12E-01	0.29
533.1061@10.57713	533.1061	10.58	NEG	RP				9.86E-02	1.73E-02	-0.57	6.34E-01	0.13	4.01E-01	0.24	2.39E-01	-0.30
533.2815@10.112881	533.2815	10.11	POS	RP				8.87E-01	5.20E-01	0.17	5.13E-01	0.21	6.35E-01	-0.15	7.66E-01	0.08
533.2851@10.007807	533.2851	10.01	POS	RP				6.31E-01	5.54E-02	0.58	7.22E-01	0.12	4.32E-01	0.26	2.86E-01	0.31
533.2889@10.17315	533.2889	10.17	POS	RP				2.64E-01	1.86E-01	0.38	1.81E-02	0.78	6.71E-02	0.50	8.03E-02	0.53
533.2891@1.3348346	533.2891	1.33	POS	hilic				2.50E-01	3.71E-01	-0.28	3.31E-02	-0.65	2.02E-02	-0.66	6.60E-01	-0.14
533.2892@1.155522	533.2892	1.16	POS	hilic				7.01E-01	8.31E-01	-0.07	5.20E-01	0.18	1.08E-01	0.46	8.02E-01	0.08
533.2895@10.116765	533.2895	10.12	POS	RP				4.82E-01	9.28E-01	-0.03	1.27E-01	0.44	1.74E-01	0.39	9.19E-01	-0.03
533.3063@1.4345134	533.3063	1.43	NEG	hilic				5.37E-01	9.35E-01	0.02	1.48E-01	0.43	8.22E-02	0.51	6.84E-01	0.11
533.3489@1.2245163	533.3489	1.22	POS	hilic				3.20E-01	2.67E-01	0.29	5.06E-01	-0.14	1.16E-01	-0.31	5.89E-01	0.14
533.7882@1.2837856	533.7882	1.28	POS	hilic				7.88E-01	9.61E-01	0.01	8.10E-01	0.05	5.29E-01	0.14	2.23E-01	-0.21
534.1235@0.40937385	534.1235	0.41	NEG	hilic				2.49E-01	3.86E-01	0.23	1.79E-01	0.40	4.93E-03	0.66	2.96E-02	0.68
534.2923@1.3356582	534.2923	1.34	POS	hilic				3.75E-01	7.92E-02	0.63	7.16E-01	-0.09	9.41E-01	0.02	4.44E-01	0.25
534.3907@0.43305728	534.3907	0.43	NEG	hilic				2.44E-02	5.45E-02	-0.39	1.94E-01	0.33	5.26E-01	-0.14	2.82E-02	0.60
534.4624@10.708414	534.4624	10.71	NEG	RP				2.94E-01	1.72E-02	-0.64	3.84E-01	-0.30	5.94E-01	-0.13	1.55E-02	-0.65
534.4631@0.41738892	534.4631	0.42	NEG	hilic				5.07E-01	6.33E-01	0.10	3.62E-02	0.49	4.14E-01	0.21	2.12E-01	0.24
534.8002@10.207139	534.8002	10.21	POS	RP				2.01E-01	6.85E-03	0.63	7.89E-01	0.09	2.95E-01	-0.26	3.34E-01	0.26
535.158@10.58187	535.1580	10.58	NEG	RP				9.65E-01	7.51E-01	0.08	6.34E-01	0.12	9.49E-01	0.01	4.27E-01	0.18
535.1586@11.538477	535.1586	11.54	POS	RP				1.26E-01	2.52E-02	-0.65	2.70E-01	-0.35	5.84E-01	-0.16	7.97E-03	-0.85
535.2558@9.037016	535.2558	9.04	NEG	RP				4.44E-01	7.85E-01	0.08	7.04E-01	-0.09	3.73E-02	-0.50	9.07E-01	-0.03
535.2682@0.47720012	535.2682	0.48	POS	hilic				1.71E-01	5.55E-01	-0.13	1.17E-01	0.41	5.12E-01	-0.17	1.00E-01	0.47
535.2919@9.999149	535.2919	10.00	POS	RP				3.20E-02	3.16E-01	0.31	1.27E-01	-0.39	2.90E-02	-0.53	1.63E-01	0.40
535.3648@1.1903613	535.3648	1.19	POS	hilic				1.92E-03	1.33E-03	0.98	5.10E-01	-0.16	1.30E-01	-0.33	9.64E-01	-0.01
535.3656@10.429869	535.3656	10.43	POS	RP				6.94E-01	9.51E-01	0.02	1.61E-01	-0.33	6.48E-01	-0.10	6.65E-01	0.13
535.8082@15.270196	535.8082	15.27	NEG	RP				7.29E-01	4.95E-01	0.20	8.71E-01	0.04	5.09E-01	0.19	2.85E-01	-0.25
536.0887@6.0238376	536.0887	6.02	POS	hilic				5.53E-01	4.21E-01	0.21	5.61E-01	0.16	1.11E-01	-0.33	8.38E-01	0.05
536.3025@10.211093	536.3025	10.21	POS	RP				1.47E-02	5.88E-04	0.92	5.53E-01	0.18	6.18E-01	0.13	2.36E-03	0.91
536.388@0.40034804	536.3880	0.40	POS	hilic				5.55E-01	8.72E-01	-0.05	5.73E-01	0.16	8.14E-01	-0.07	1.06E-01	-0.48
536.407@0.43519044	536.4070	0.44	NEG	hilic				7.44E-02	6.00E-02	-0.32	2.57E-01	0.27	4.73E-01	-0.14	7.21E-02	0.46
536.5278@12.945899	536.5278	12.95	POS	RP				9.52E-01	4.14E-01	-0.22	3.91E-01	-0.24	4.12E-01	-0.23	6.83E-01	-0.15
537.137@11.057603	537.1370	11.06	NEG	RP				3.29E-01	1.25E-01	0.41	9.32E-01	-0.02	1.14E-01	0.49	7.12E-01	-0.10
537.1373@11.059795	537.1373	11.06	NEG	RP				2.42E-01	2.08E-01	0.33	9.92E-01	0.00	2.93E-02	0.68	6.82E-01	0.11
537.1747@10.807076	537.1747	10.81	NEG	RP				9.75E-01	7.33E-01	0.11	8.91E-01	0.04	8.72E-01	0.04	6.65E-01	-0.13
537.2568@2.0872886	537.2568	2.09	NEG	hilic				3.13E-02	9.05E-01	-0.04	1.13E-02	-0.65	4.97E-03	-0.67	7.83E-01	0.08
537.3435@1.6958958	537.3435	1.70	POS	hilic				1.33E-01	6.15E-02	0.56	4.70E-01	-0.15	3.82E-01	-0.18	7.76E-01	-0.07
537.3806@1.0561036	537.3806	1.06	POS	hilic				3.75E-04	2.43E-02	0.66	5.82E-02	-0.33	7.78E-04	-0.49	4.38E-01	-0.15
537.3818@1.178574	537.3818	1.18	POS	hilic				1.56E-04	4.76E-04	1.00	9.21E-02	-0.41	8.46E-03	-0.60	8.12E-01	0.07
537.3825@10.720072	537.3825	10.72	POS	RP				5.83E-03	2.49E-02	0.77	5.55E-02	-0.42	1.52E-01	-0.30	9.56E-01	-0.01
537.4763@0.5899169	537.4763	0.59	POS	hilic				9.43E-01	7.83E-01	-0.08	6.45E-01	-0.13	9.39E-01	0.02	6.29E-01	0.16
538.1174@0.41219613	538.1174	0.41	NEG	hilic				1.73E-01	1.16E-01	0.32	3.02E-02	0.51	1.35E-02	0.58	1.32E-02	0.65
538.1536@0.40553755	538.1536	0.41	NEG	hilic				7.70E-01	1.89E-01	-0.35	4.46E-01	-0.21	3.62E-01	-0.25	1.29E-01	-0.41
538.154@9.173871	538.1540	9.17	NEG	RP				7.86E-01	5.78E-01	-0.17	3.37E-01	0.24	6.65E-01	0.12	3.95E-01	0.28
538.1572@11.53724	538.1572	11.54	POS	RP				2.86E-01	2.70E-02	-0.66	6.51E-02	-0.56	3.77E-01	-0.25	4.63E-02	-0.64
538.422@0.43485984	538.4220	0.43	NEG	hilic				5.12E-04	7.03E-03	-0.77	5.52E-03	0.75	4.45E-01	0.20	4.95E-02	0.48
538.4231@10.609428	538.4231	10.61	NEG	RP				1.25E-04	1.58E-04	-1.05	5.50E-02	0.50	9.69E-01	0.01	1.02E-01	0.42
539.1564@11.536863	539.1564	11.54	POS	RP				8.63E-01	3.72E-01	-0.28	3.14E-01	-0.31	2.87E-01	-0.28	2.61E-01	-0.34
539.1909@11.087321	539.1909	11.09	NEG	RP				6.04E-01	4.59E-01	0.19	5.73E-02	0.51	2.61E-01	0.34	1.66E-01	0.37
539.242@1.8217434	539.2420	1.82	POS	hilic				3.35E-01	9.64E-01	0.02	1.36E-01	-0.38	9.65E-02	-0.51	5.42E-01	0.16
539.3212@9.652087	539.3212	9.65	NEG	RP				4.49E-01	1.72E-01	0.45	6.98E-01	-0.12	1.13E-01	0.47	4.07E-01	0.23
539.3214@1.4469138	539.3214	1.45	NEG	hilic				6.95E-01	3.75E-01	0.28	9.54E-01	-0.02	1.23E-01	0.45	8.46E-01	0.06
539.3215@9.782795	539.3215	9.78	NEG	RP				3.80E-01	1.52E-01	0.47	6.57E-01	-0.13	9.41E-02	0.49	3.17E-01	0.27
539.3408@10.31743	539.3408	10.32	NEG	RP				7.27E-01	8.53E-01	0.05	2.80E-01	0.32	1.18E-01	0.42	4.59E-01	0.21
539.3583@10.1999655	539.3583	10.20	POS	RP				8.14E-02	2.38E-03	0.86	8.57E-01	-0.04	5.94E-01	0.14	3.32E-01	0.28
539.3587@9.208345	539.3587	9.21	POS	RP				1.98E-01	1.03E-01	0.50	2.38E-01	-0.36	2.50E-01	-0.31	8.99E-01	-0.04
539.3592@1.6931977	539.3592	1.69	POS	hilic				2.54E-02	2.97E-04	1.04	7.80E-01	-0.07	5.87E-01	0.15	4.06E-01	0.29
539.3594@1.5966933	539.3594	1.60	POS	hilic				4.08E-01	1.21E-01	0.47	3.78E-01	-0.25	5.08E-01	-0.19	9.09E-01	-0.03
539.4189@9.654241	539.4189	9.65	POS	RP				9.06E-01	5.78E-01	-0.16	6.19E-01	-0.13	6.77E-01	0.11	7.76E-01	0.08
539.4219@0.4477948	539.4219	0.45	POS	hilic				8.96E-01	4.22E-01	-0.20	2.47E-01	-0.26	5.35E-01	-0.15	5.49E-01	-0.15
539.4318@10.590336	539.4318	10.59	POS	RP				7.99E-01	8.25E-01	-0.05	1.62E-01	-0.38	6.96E-01	-0.12	8.49E-01	-0.06
540.1438@10.712588	540.1438	10.71	NEG	RP				4.10E-01	6.91E-01	0.12	2.77E-02	-0.47	6.70E-01	-0.11	2.73E-01	-0.25
540.2255@0.47278833	540.2255	0.47	POS	hilic				3.82E-01	1.89E-01	-0.45	2.14E-01	-0.34	5.24E-03	-0.69	5.10E-01	-0.19

Supplementary table 2: Metabolomics analysis results

								ANOVA	Anorexia vs control		overweight vs control		obese vs control		athletes vs control	
Compound	Mass	Retention Time	Ionization mode	column	Compound_ID	MSMS_spectra	ID_level	P-FDR	P	Estimate	P	Estimate	P	Estimate	P	Estimate
540.2555@6.7192636	540.2555	6.72	NEG	RP				4.92E-01	6.57E-01	0.14	9.24E-01	-0.02	1.76E-01	0.36	2.59E-01	-0.32
540.2565@7.0241737	540.2565	7.02	NEG	RP				1.01E-01	2.86E-02	-0.38	3.93E-01	0.18	1.70E-01	0.40	3.82E-01	-0.16
540.4364@10.698514	540.4364	10.70	NEG	RP				3.36E-04	7.68E-04	-0.94	2.21E-02	0.58	3.84E-01	0.21	2.18E-01	0.31
540.4371@10.714588	540.4371	10.71	NEG	RP				1.01E-02	3.44E-02	-0.57	3.58E-02	0.57	1.16E-01	0.39	1.77E-01	0.37
540.4378@0.4302263	540.4378	0.43	NEG	hilic				2.50E-04	6.01E-03	-0.74	2.52E-03	0.83	1.97E-01	0.33	7.45E-02	0.40
540.8215@10.242106	540.8215	10.24	POS	RP				7.69E-01	1.82E-01	-0.35	8.55E-01	0.05	6.49E-01	-0.11	5.17E-01	-0.17
541.3148@9.948709	541.3148	9.95	POS	RP				3.09E-01	2.56E-01	-0.34	6.49E-02	0.49	8.31E-01	-0.06	8.94E-01	0.05
541.3155@1.2330005	541.3155	1.23	POS	hilic				5.60E-01	4.86E-01	0.20	6.42E-01	-0.14	4.36E-01	0.19	2.12E-01	-0.38
541.3156@1.1278788	541.3156	1.13	POS	hilic				2.56E-01	2.91E-01	-0.30	3.85E-02	-0.66	8.48E-03	-0.72	3.22E-01	-0.33
541.3169@9.709091	541.3169	9.71	POS	RP	LysoPC 20:5	10 eV: 542.3239 (100), 184.0724 (15)	2	5.81E-01	1.73E-01	0.42	6.97E-01	-0.12	3.11E-01	0.27	7.15E-01	-0.11
541.317@9.603494	541.3170	9.60	POS	RP				3.87E-01	2.77E-02	0.63	9.29E-01	0.03	6.14E-01	0.13	7.22E-01	-0.11
541.3372@10.110867	541.3372	10.11	NEG	RP	LysoPC 16:0 (M+FA)	10 eV: 480.3100 (100), 540.3288 (48), 255.2323 (16)	2	2.22E-03	3.46E-03	0.83	2.16E-01	-0.33	8.05E-01	-0.07	4.79E-03	0.77
541.3373@1.2513417	541.3373	1.25	NEG	hilic				2.00E-03	1.81E-03	0.92	9.33E-02	-0.41	8.24E-01	-0.06	4.60E-02	0.58
541.3376@1.4064443	541.3376	1.41	NEG	hilic				2.87E-03	4.46E-03	0.81	6.58E-02	-0.47	4.23E-01	-0.21	1.05E-01	0.49
541.3379@10.000221	541.3379	10.00	NEG	RP				3.33E-03	1.63E-03	0.94	2.30E-01	-0.29	6.26E-01	0.15	6.97E-03	0.70
541.7234@3.0549307	541.7234	3.05	POS	hilic				9.52E-01	4.55E-01	0.26	9.75E-01	0.01	9.32E-01	-0.02	8.24E-01	0.06
542.2713@7.029448	542.2713	7.03	NEG	RP				1.36E-02	7.81E-01	0.05	8.23E-01	0.04	9.98E-03	0.72	2.76E-01	-0.17
542.2716@6.5388207	542.2716	6.54	NEG	RP				1.07E-01	6.34E-01	0.12	9.24E-01	0.02	2.66E-02	0.65	2.24E-01	-0.34
542.3407@1.2513239	542.3407	1.25	NEG	hilic				1.54E-03	1.88E-02	0.76	2.86E-02	-0.51	4.40E-01	-0.20	2.52E-02	0.59
542.4368@14.374182	542.4368	14.37	POS	RP				2.69E-01	5.90E-01	0.15	9.12E-02	0.46	2.05E-01	-0.37	7.14E-01	-0.11
542.4397@0.39712724	542.4397	0.40	POS	hilic				5.83E-01	4.73E-01	0.18	7.92E-01	-0.07	6.59E-01	-0.11	7.10E-02	-0.38
542.8409@10.524272	542.8409	10.52	POS	RP				4.09E-01	1.38E-01	0.42	2.38E-01	-0.37	8.03E-01	-0.07	9.74E-01	-0.01
543.0868@6.269795	543.0868	6.27	POS	hilic				1.08E-01	6.14E-01	0.17	8.22E-01	-0.06	8.03E-02	-0.49	4.12E-02	0.51
543.1356@10.576507	543.1356	10.58	NEG	RP				1.84E-01	7.01E-02	-0.42	2.89E-01	0.29	6.40E-01	0.13	1.38E-01	0.36
543.2566@11.430176	543.2566	11.43	NEG	RP				7.29E-01	2.68E-01	-0.32	9.09E-01	-0.03	4.17E-01	0.21	6.63E-01	-0.12
543.3308@10.420765	543.3308	10.42	POS	RP				7.63E-01	6.93E-01	0.11	2.98E-01	-0.33	8.17E-01	0.06	5.24E-01	-0.17
543.3313@10.271045	543.3313	10.27	POS	RP				2.79E-01	3.10E-02	0.61	9.05E-01	0.04	5.59E-01	-0.15	1.70E-01	0.41
543.3322@1.079924	543.3322	1.08	POS	hilic				9.19E-01	9.03E-01	0.04	4.36E-01	-0.26	8.45E-01	-0.06	8.60E-01	0.05
543.3331@9.852595	543.3331	9.85	POS	RP				9.47E-01	6.34E-01	-0.15	5.56E-01	-0.19	8.34E-01	-0.06	7.62E-01	0.09
543.3332@1.1974442	543.3332	1.20	POS	hilic				2.81E-01	5.80E-01	0.16	6.11E-02	-0.50	5.29E-01	0.20	3.92E-01	-0.24
543.3334@9.944305	543.3334	9.94	POS	RP	LysoPC 20:4	20 eV: 184.0733 (100), 544.3387 (10)	1	1.41E-02	4.44E-01	-0.23	2.68E-02	0.61	3.95E-03	0.87	8.30E-01	0.06
543.3335@9.944903	543.3335	9.94	POS	RP	dublicate of LysoPC 20:4			5.47E-02	4.50E-01	-0.23	8.44E-02	0.50	1.16E-02	0.77	8.53E-01	0.06
543.4328@13.243438	543.4328	13.24	POS	RP				7.12E-02	4.72E-02	0.52	3.93E-03	0.84	8.36E-01	-0.05	3.50E-01	0.28
543.7222@3.072391	543.7222	3.07	POS	hilic				9.23E-01	3.82E-01	0.27	9.97E-01	0.00	5.02E-01	0.20	5.32E-01	0.19
543.7227@3.0593243	543.7227	3.06	POS	hilic				9.01E-01	2.62E-01	-0.35	6.23E-01	-0.13	3.91E-01	-0.25	5.77E-01	-0.18
543.8066@15.268047	543.8066	15.27	NEG	RP				9.94E-01	9.43E-01	-0.02	6.69E-01	-0.12	6.83E-01	-0.11	7.94E-01	-0.07
544.2538@0.49493328	544.2538	0.49	POS	hilic				6.55E-01	3.95E-01	-0.20	4.99E-01	0.17	4.80E-01	-0.17	4.57E-01	0.20
544.2845@12.381133	544.2845	12.38	NEG	RP				9.29E-01	5.63E-01	0.18	4.31E-01	0.20	7.84E-01	-0.08	6.78E-01	0.13
544.4133@0.4266715	544.4133	0.43	NEG	hilic				3.80E-02	3.10E-03	-0.67	9.53E-01	0.02	2.25E-02	-0.54	5.41E-01	0.17
545.1506@10.7903185	545.1506	10.79	NEG	RP				7.12E-01	4.43E-01	0.24	6.09E-02	0.44	2.87E-01	0.30	6.54E-01	0.10
545.2495@1.8246578	545.2495	1.82	POS	hilic				9.27E-01	7.65E-01	-0.10	5.17E-01	0.18	6.64E-01	-0.14	8.84E-01	0.04
545.2718@9.979092	545.2718	9.98	NEG	RP				3.68E-02	5.76E-02	0.60	1.15E-01	-0.43	3.49E-01	-0.26	2.33E-01	0.30
545.2727@2.159889	545.2727	2.16	NEG	hilic				1.31E-03	4.57E-02	0.57	2.72E-02	-0.59	6.52E-03	-0.73	4.83E-01	0.21
545.3013@0.42063648	545.3013	0.42	POS	hilic				8.66E-01	7.05E-01	0.09	1.47E-01	0.31	5.37E-01	0.16	4.53E-01	0.19
545.3137@12.576624	545.3137	12.58	NEG	RP				3.37E-01	8.52E-02	0.57	6.70E-02	0.49	2.78E-02	0.63	4.26E-02	0.62
545.3233@10.336494	545.3233	10.34	NEG	RP				4.71E-01	2.50E-01	0.35	2.83E-01	-0.27	3.71E-01	-0.21	5.44E-01	-0.17
545.3463@10.573923	545.3463	10.57	POS	RP				3.96E-01	3.40E-01	0.31	3.89E-01	0.21	9.01E-03	0.70	1.26E-01	0.47
545.3465@10.626859	545.3465	10.63	POS	RP				1.73E-01	3.50E-03	0.71	4.11E-02	0.41	1.50E-01	0.34	3.11E-02	0.54
545.3466@10.56319	545.3466	10.56	POS	RP				3.16E-01	1.89E-02	0.65	9.94E-01	0.00	1.20E-01	0.45	1.68E-01	0.41
545.3493@10.157528	545.3493	10.16	POS	RP				8.62E-01	6.04E-01	0.20	9.15E-01	0.03	2.30E-01	0.31	8.22E-01	-0.06
545.3494@10.157357	545.3494	10.16	POS	RP	LysoPC 20:3	10 eV: 546.3542 (100), 184.0731 (100)	2	8.81E-01	6.21E-01	0.19	9.55E-01	0.02	2.38E-01	0.31	8.80E-01	-0.04
545.3499@10.031565	545.3499	10.03	POS	RP				9.92E-01	9.39E-01	0.02	9.11E-01	0.03	8.24E-01	-0.07	6.99E-01	-0.13
545.4479@13.622846	545.4479	13.62	POS	RP				7.43E-01	9.38E-01	0.02	2.37E-01	0.33	5.89E-01	-0.15	7.14E-01	-0.12
545.4479@13.762422	545.4479	13.76	POS	RP				4.04E-01	6.78E-01	0.11	1.34E-01	0.42	2.44E-01	-0.34	9.15E-01	-0.03
545.4484@13.245296	545.4484	13.25	POS	RP				4.39E-01	2.78E-01	0.30	2.10E-01	0.35	3.28E-01	-0.23	5.38E-01	-0.20
545.7892@9.958297	545.7892	9.96	POS	RP				2.55E-02	2.63E-01	0.32	3.37E-01	-0.28	1.03E-02	-0.69	1.84E-01	0.37
545.9729@8.895891	545.9729	8.90	NEG	RP				4.11E-01	1.26E-01	-0.40	5.31E-01	-0.21	2.80E-02	-0.68	9.53E-02	-0.47
546.2141@0.48502004	546.2141	0.49	POS	hilic				4.23E-01	4.75E-01	-0.22	2.01E-01	-0.34	7.01E-03	-0.69	3.54E-01	-0.29
546.3784@13.818294	546.3784	13.82	POS	RP				4.70E-01	8.84E-01	-0.04	5.15E-01	0.17	1.08E-01	-0.46	2.84E-01	-0.33
546.3886@10.945064	546.3886	10.95	POS	RP				5.93E-01	4.72E-01	0.19	4.67E-01	-0.19	7.17E-01	0.09	2.07E-01	0.37
546.3925@12.607502	546.3925	12.61	POS	RP				9.58E-01	5.41E-01	-0.18	6.18E-01	-0.16	5.10E-01	-0.17	3.70E-01	-0.26
546.4275@10.768173	546.4275	10.77	POS	RP				7.78E-01	5.13E-01	-0.11	4.45E-01	0.16	7.52E-01	-0.06	4.92E-01	0.15
546.4281@0.42632988	546.4281	0.43	NEG	hilic				2.10E-03	4.39E-03	-0.79	7.31E-02	0.46	5.12E-01	-0.17	8.51E-02	0.49
546.43@0.42749226	546.4300	0.43	POS	hilic				6.72E-02	3.51E-02	-0.46	2.13E-01	0.33	5.54E-01	-0.14	1.32E-01	0.40
547.1649@11.064585	547.1649	11.06	NEG	RP				3.22E-01	1.67E-01	0.49	3.62E-01	-0.23	1.51E-01	0.38	9.13E-01	0.03
547.1653@11.044519	547.1653	11.04	NEG	RP				1.33E-01	2.28E-01	0.33	4.51E-01	0.20	8.45E-02	-0.53	2.96E-01	0.28
547.2264@1.5027347	547.2264	1.50	NEG	hilic				5.30E-01	2.10E-01	0.36	8.89E-01	0.04	1.61E-01	0.43	1.27E-01	0.51
547.2883@2.080896	547.2883	2.08	NEG	hilic				2.20E-03	1.93E-02	0.59	5.59E-02	-0.52	9.39E-03	-0.65	6.71E-01	0.13
547.2884@10.235462	547.2884	10.24	NEG	RP				1.83E-01	7.55E-01	0.09	1.90E-01	-0.38	1.07E-01	-0.42	2.62E-01	0.34
547.2928@9.958488	547.2928	9.96	POS	RP				2.01E-02	6.91E-01	0.11	1.42E-01	-0.44	2.73E-03	-0.85	4.84E-01	0.21
547.3627@10.260615	547.3627	10.26	POS	RP				1.13E-01	3.64E-01	0.25	5.98E-02	-0.39	3.38E-02	-0.41	5.99E-01	0.15
547.3641@10.387241	547.3641	10.39	POS	RP	LysoPC 20:2	20 eV: 548.3708 (100), 104.1064 (76), 184.0733 (70)	2	1.02E-03	9.29E-03	0.73	2.78E-02	-0.67	8.04E-02	-0.44	4.07E-01	0.23
547.8046@10.245417	547.8046	10.25	POS	RP				1.29E-02	4.55E-03	0.88	5.22E-01	-0.19	3.24E-01	-0.24	1.37E-01	0.42

Supplementary table 2: Metabolomics analysis results

								ANOVA	Anorexia vs control		overweight vs control		obese vs control		athletes vs control	
Compound	Mass	Retention Time	Ionization mode	column	Compound_ID	MSMS_spectra	ID_level	P-FDR	P	Estimate	P	Estimate	P	Estimate	P	Estimate
547.8049@1.220812	547.8049	1.22	POS	hilic				7.15E-01	6.69E-01	-0.13	5.65E-01	0.16	1.76E-01	-0.33	9.53E-01	0.02
548.1262@0.4065001	548.1262	0.41	NEG	hilic				4.06E-01	3.68E-01	0.25	6.32E-01	-0.14	9.23E-01	-0.02	7.13E-02	0.54
548.1758@5.254259	548.1758	5.25	NEG	hilic	dublicate			1.18E-03	1.62E-07	-1.25	9.22E-01	0.03	5.96E-01	-0.13	1.53E-02	-0.60
548.1758@5.250646	548.1758	5.25	NEG	hilic	Unknown	10 eV: 367.1053 (100), 165.4250 (5), 547.2883 (4)	4	1.06E-02	2.81E-06	-1.04	7.38E-01	0.10	8.94E-02	-0.48	5.79E-02	-0.45
548.2081@0.5084445	548.2081	0.51	NEG	hilic				7.36E-02	1.83E-02	0.68	1.85E-02	0.54	8.99E-01	0.02	3.55E-02	0.47
548.3994@10.876784	548.3994	10.88	POS	RP				1.85E-01	4.36E-01	-0.24	8.36E-02	-0.40	1.60E-02	-0.62	6.32E-01	0.14
548.3995@10.9416	548.3995	10.94	POS	RP				2.17E-01	9.42E-01	-0.02	8.11E-02	-0.47	1.25E-01	-0.44	4.43E-01	0.23
548.4356@0.4282882	548.4356	0.43	POS	hilic				5.35E-01	7.69E-02	-0.40	6.37E-01	0.12	5.41E-01	0.14	9.52E-01	-0.02
548.4422@10.701979	548.4422	10.70	POS	RP				5.45E-01	6.51E-02	-0.38	6.86E-01	0.10	2.30E-01	-0.28	9.65E-01	-0.01
548.4424@11.036409	548.4424	11.04	NEG	RP				3.19E-03	3.42E-03	-0.83	8.73E-02	0.45	7.08E-01	0.09	1.73E-01	0.40
548.4428@0.42455313	548.4428	0.42	NEG	hilic				1.20E-02	3.94E-03	-0.80	1.55E-01	0.38	9.85E-01	0.00	2.47E-01	0.32
548.4791@13.312599	548.4791	13.31	POS	RP				1.11E-01	8.38E-02	0.53	6.76E-02	0.44	1.67E-03	0.91	6.75E-02	0.51
548.8094@1.2118357	548.8094	1.21	POS	hilic				4.03E-01	5.58E-02	-0.48	5.20E-01	0.17	7.29E-01	-0.09	8.75E-01	0.05
548.8221@10.526331	548.8221	10.53	POS	RP				6.59E-02	1.28E-02	0.69	2.12E-01	-0.39	7.88E-01	0.07	3.32E-01	0.31
549.1793@5.2031074	549.1793	5.20	NEG	hilic				4.72E-01	9.40E-02	-0.41	4.56E-01	0.22	9.98E-01	0.00	5.98E-01	-0.13
549.1795@5.2671566	549.1795	5.27	NEG	hilic				8.78E-02	3.21E-03	-0.60	2.68E-01	0.32	2.03E-01	-0.35	3.81E-01	-0.21
549.238@8.933246	549.2380	8.93	NEG	RP				7.45E-01	4.17E-01	0.25	3.71E-01	0.23	5.25E-01	-0.16	5.61E-01	0.16
549.2381@9.086209	549.2381	9.09	NEG	RP				5.38E-01	3.62E-01	0.26	9.96E-01	0.00	1.33E-01	-0.30	8.58E-01	0.04
549.2537@10.112556	549.2537	10.11	POS	RP				1.85E-01	4.13E-02	0.65	1.05E-01	0.50	1.07E-01	0.48	4.45E-03	0.84
549.283@1.8009635	549.2830	1.80	POS	hilic				3.97E-01	2.84E-01	0.34	3.78E-01	-0.25	3.00E-01	-0.32	4.39E-01	0.20
549.3027@10.491329	549.3027	10.49	NEG	RP				2.81E-02	1.58E-01	0.39	3.23E-01	-0.25	8.63E-03	-0.61	4.43E-01	0.19
549.3028@2.0641334	549.3028	2.06	NEG	hilic				6.01E-03	2.87E-02	0.60	1.85E-01	-0.25	2.93E-02	-0.37	6.51E-01	0.11
549.3089@10.239683	549.3089	10.24	POS	RP				1.13E-02	1.38E-02	0.77	2.16E-01	-0.37	9.53E-02	-0.42	5.39E-01	0.17
549.3786@10.655205	549.3786	10.66	POS	RP				8.34E-03	4.09E-03	0.94	5.62E-01	0.11	7.64E-01	-0.06	5.80E-01	0.12
549.3791@1.0454361	549.3791	1.05	POS	hilic				1.12E-03	1.87E-02	0.70	9.52E-02	-0.52	4.93E-03	-0.75	9.22E-01	-0.03
549.3795@10.6185875	549.3795	10.62	POS	RP	LysoPC 20:1	10 eV: 550.3878 (100), 184.0735 (13)	2	8.57E-05	3.70E-04	1.06	4.16E-02	-0.54	5.79E-02	-0.41	4.10E-01	0.22
549.3795@10.529713	549.3795	10.53	POS	RP				4.77E-01	5.20E-01	-0.15	3.74E-02	-0.58	9.29E-01	-0.03	6.83E-01	-0.11
549.3799@1.1611799	549.3799	1.16	POS	hilic				6.77E-04	8.45E-03	0.82	2.10E-02	-0.63	3.07E-02	-0.52	9.38E-01	0.02
549.6221@10.4581175	549.6221	10.46	POS	RP				4.97E-01	6.03E-01	0.18	5.56E-02	0.52	1.96E-01	0.34	4.80E-02	0.57
549.7726@3.2229018	549.7726	3.22	NEG	hilic				7.34E-01	1.73E-01	0.46	4.92E-01	0.19	7.54E-01	0.08	2.25E-01	0.36
549.7727@3.2229018	549.7727	3.22	NEG	hilic				8.80E-01	4.88E-01	0.24	5.52E-01	0.16	7.57E-01	0.08	2.24E-01	0.37
549.7729@3.2229018	549.7729	3.22	NEG	hilic				7.07E-01	1.55E-01	0.48	4.84E-01	0.19	6.91E-01	0.11	1.94E-01	0.39
549.8107@10.238966	549.8107	10.24	POS	RP				1.22E-02	2.12E-02	0.74	2.43E-01	-0.35	6.40E-02	-0.46	4.84E-01	0.20
549.8203@10.52439	549.8203	10.52	POS	RP				4.83E-02	8.25E-03	0.71	2.10E-01	-0.38	8.55E-01	0.05	2.66E-01	0.35
549.8554@15.281879	549.8554	15.28	NEG	RP				2.77E-01	1.35E-02	-0.73	1.76E-01	-0.38	6.89E-01	-0.11	6.05E-01	-0.15
550.1581@6.078523	550.1581	6.08	NEG	hilic				8.62E-01	8.52E-01	-0.06	5.37E-01	-0.19	1.47E-01	-0.38	5.32E-01	-0.19
550.263@6.8338733	550.2630	6.83	POS	RP				1.74E-01	9.41E-03	0.83	2.87E-02	0.56	7.09E-02	0.55	4.77E-01	0.20
550.2638@0.42657232	550.2638	0.43	POS	hilic				2.66E-02	4.95E-01	0.21	2.51E-02	0.56	1.33E-03	0.96	8.28E-01	-0.06
550.3006@9.948069	550.3006	9.95	POS	RP				9.01E-01	9.73E-01	0.01	7.37E-01	0.09	7.58E-01	0.08	3.92E-01	-0.22
550.4176@0.41502792	550.4176	0.42	POS	hilic				6.16E-01	8.66E-02	0.45	9.57E-01	-0.01	9.74E-01	-0.01	4.64E-01	0.22
550.4181@10.876335	550.4181	10.88	POS	RP				4.30E-01	1.42E-01	-0.39	7.24E-02	-0.40	8.17E-02	-0.42	8.88E-01	-0.04
550.4275@10.701412	550.4275	10.70	POS	RP				8.17E-01	4.66E-01	0.17	9.52E-01	-0.02	9.40E-01	0.02	3.27E-01	-0.31
550.4576@0.42700002	550.4576	0.43	POS	hilic				7.92E-03	4.45E-02	-0.34	3.33E-03	0.74	3.23E-01	0.22	3.88E-01	0.21
550.4588@0.4217497	550.4588	0.42	NEG	hilic				5.45E-03	1.43E-02	-0.70	2.92E-02	0.55	2.94E-01	0.29	1.15E-01	0.43
551.2812@12.381632	551.2812	12.38	NEG	RP				6.02E-01	1.49E-01	-0.45	4.09E-01	-0.25	5.04E-01	-0.19	1.00E-01	-0.55
551.3257@10.520078	551.3257	10.52	POS	RP				4.13E-01	6.25E-01	0.17	1.34E-01	-0.46	1.25E-01	-0.38	9.49E-01	0.02
551.3929@1.1452643	551.3929	1.15	POS	hilic				1.82E-03	4.59E-02	0.70	1.69E-02	-0.55	2.63E-03	-0.65	7.44E-01	-0.09
551.4245@0.41018552	551.4245	0.41	POS	hilic				9.96E-02	4.46E-01	-0.21	4.53E-01	-0.18	1.46E-02	-0.66	3.04E-01	0.32
551.7715@3.2202613	551.7715	3.22	NEG	hilic				6.74E-01	1.02E-01	0.55	1.98E-01	0.36	5.55E-01	0.18	2.39E-01	0.28
551.8262@10.521109	551.8262	10.52	POS	RP				4.08E-02	3.68E-02	0.62	8.15E-02	-0.53	4.13E-01	-0.21	8.36E-01	0.06
552.1323@8.388849	552.1323	8.39	NEG	RP				9.85E-01	5.91E-01	-0.18	9.41E-01	0.02	8.85E-01	-0.04	9.52E-01	-0.02
552.1331@8.507726	552.1331	8.51	NEG	RP				8.94E-01	9.17E-01	-0.03	5.18E-01	0.18	3.00E-01	0.28	5.32E-01	0.20
552.1334@8.152846	552.1334	8.15	NEG	RP				7.05E-01	7.29E-01	0.12	8.70E-01	0.04	4.72E-01	0.19	1.22E-01	0.50
552.1339@8.051275	552.1339	8.05	NEG	RP				4.51E-01	2.60E-01	-0.36	9.48E-01	0.02	2.81E-01	-0.26	7.23E-02	-0.59
552.1346@0.40659845	552.1346	0.41	NEG	hilic				3.25E-01	1.00E-02	0.69	6.41E-01	0.14	7.45E-01	-0.09	4.37E-01	0.24
552.2411@9.615607	552.2411	9.62	POS	RP				7.39E-02	2.91E-02	0.68	9.87E-01	0.00	6.22E-02	0.53	9.53E-03	0.78
552.3626@0.403517	552.3626	0.40	POS	hilic				7.01E-01	3.59E-01	0.23	3.09E-01	0.29	8.46E-01	-0.06	5.30E-01	-0.20
552.3634@13.242202	552.3634	13.24	POS	RP				2.64E-01	1.14E-01	0.42	3.38E-02	0.62	9.63E-01	0.01	8.48E-01	-0.06
552.3663@10.845515	552.3663	10.85	NEG	RP				8.96E-01	7.78E-01	-0.08	2.19E-01	-0.25	4.11E-01	-0.19	7.25E-01	-0.08
552.4736@0.42040786	552.4736	0.42	NEG	hilic				2.97E-02	4.93E-01	-0.14	6.75E-03	0.66	1.00E-02	0.68	1.25E-01	0.40
552.824@10.518	552.8240	10.52	POS	RP				6.32E-01	5.26E-01	-0.19	5.68E-02	-0.45	4.64E-01	-0.17	2.08E-01	-0.35
553.138@0.40651363	553.1380	0.41	NEG	hilic				3.24E-01	1.28E-01	0.42	5.36E-01	-0.19	1.50E-01	-0.39	5.03E-01	-0.18
553.2474@11.429984	553.2474	11.43	NEG	RP				9.84E-01	7.08E-01	0.10	5.27E-01	0.16	7.69E-01	0.07	9.91E-01	0.00
553.2737@12.381632	553.2737	12.38	NEG	RP				6.40E-01	1.38E-01	-0.42	5.44E-01	-0.18	6.17E-01	-0.16	1.18E-01	-0.52
553.2929@9.714437	553.2929	9.71	NEG	RP				7.66E-03	3.15E-02	0.64	7.11E-02	-0.48	7.44E-02	-0.46	6.12E-01	0.16
553.302@0.42812198	553.3020	0.43	POS	hilic				9.37E-01	8.07E-01	-0.06	3.64E-01	-0.20	6.66E-01	-0.11	8.81E-01	0.03
553.3368@10.026984	553.3368	10.03	NEG	RP				4.62E-02	1.93E-03	0.96	7.06E-01	-0.12	3.49E-01	0.24	3.23E-01	0.27
553.337@10.020475	553.3370	10.02	NEG	RP				1.83E-02	1.61E-01	-0.11	5.14E-01	0.09	1.16E-02	0.64	3.21E-01	0.17
553.3377@10.201342	553.3377	10.20	NEG	RP				6.24E-01	1.43E-01	0.44	9.27E-01	0.02	5.98E-01	-0.12	9.59E-01	-0.01
553.3733@10.471539	553.3733	10.47	NEG	RP				1.81E-01	2.42E-01	0.41	1.06E-01	-0.51	3.12E-01	-0.26	9.34E-01	0.02
553.374@1.3574399	553.3740	1.36	NEG	hilic				6.09E-01	1.92E-01	0.37	4.79E-01	-0.15	8.46E-01	0.04	9.83E-01	0.01
553.3751@10.696439	553.3751	10.70	NEG	RP				5.11E-01	2.96E-01	0.27	4.94E-02	0.47	4.64E-01	0.16	1.17E-01	0.42
553.4327@10.016903	553.4327	10.02	POS	RP				8.58E-01	7.45E-01	0.07	9.58E-01	-0.01	4.01E-01	0.20	3.07E-01	0.22

Supplementary table 2: Metabolomics analysis results

Compound	Mass	Retention Time	Ionization mode	column	Compound_ID	MSMS_spectra	ID_level	ANOVA P-FDR	Anorexia vs control P	Anorexia vs control Estimate	overweight vs control P	overweight vs control Estimate	obese vs control P	obese vs control Estimate	athletes vs control P	athletes vs control Estimate
554.1936@9.614546	554.1936	9.61	POS	RP				1.55E-02	4.83E-01	0.16	2.39E-01	-0.23	1.46E-02	0.60	1.54E-02	0.66
554.3566@0.6624182	554.3566	0.66	POS	hilic				8.81E-01	5.44E-01	0.18	4.87E-01	-0.16	9.37E-01	0.02	9.40E-01	0.02
554.4332@0.4183971	554.4332	0.42	NEG	hilic				5.57E-01	1.11E-01	-0.38	4.68E-01	-0.19	2.03E-01	-0.33	7.02E-01	0.11
555.271@12.381927	555.2710	12.38	NEG	RP				6.84E-01	9.90E-01	0.00	4.26E-01	-0.22	5.43E-01	0.17	2.65E-01	-0.32
555.2879@0.42952517	555.2879	0.43	POS	hilic				3.39E-01	3.22E-02	0.63	4.68E-01	0.20	2.09E-02	0.57	5.45E-01	0.18
555.3084@9.845973	555.3084	9.85	NEG	RP				5.45E-03	7.18E-01	-0.10	1.16E-02	-0.79	3.26E-04	-0.95	7.88E-01	0.08
555.3086@1.3458031	555.3086	1.35	NEG	hilic				8.25E-01	7.54E-01	-0.09	8.68E-01	-0.05	1.73E-01	-0.41	8.22E-01	-0.06
555.3094@9.911018	555.3094	9.91	NEG	RP				8.75E-01	7.11E-01	-0.12	8.35E-01	-0.06	8.77E-01	-0.04	3.84E-01	0.27
555.3102@1.2175545	555.3102	1.22	NEG	hilic				1.31E-02	5.14E-01	0.20	1.93E-02	-0.65	2.02E-03	-0.79	7.63E-01	0.09
555.3459@1.3375001	555.3459	1.34	NEG	hilic				4.39E-01	8.41E-02	0.44	4.27E-01	-0.14	5.47E-01	0.15	8.57E-01	0.04
555.3524@10.324308	555.3524	10.32	NEG	RP	LysoPC 17:0 (M+FA)	10 eV: 494.324 (100), 554.3471 (48), 269.2476 (5)	2	1.24E-02	8.02E-04	1.00	3.19E-01	-0.25	7.31E-01	0.09	4.54E-01	0.24
555.3525@10.273165	555.3525	10.27	NEG	RP				9.93E-01	9.38E-01	0.02	9.23E-01	0.02	9.32E-01	0.02	7.06E-01	-0.10
555.3528@10.313402	555.3528	10.31	NEG	RP				6.97E-01	8.33E-01	0.07	1.16E-01	-0.39	5.00E-01	-0.20	7.69E-01	0.09
555.3885@10.769482	555.3885	10.77	NEG	RP				5.03E-01	6.00E-01	-0.16	7.48E-02	-0.53	3.81E-02	-0.55	3.19E-01	-0.35
555.4349@10.19097	555.4349	10.19	POS	RP				4.91E-01	5.92E-01	0.15	5.45E-01	-0.17	3.57E-01	0.28	2.25E-01	-0.37
555.851@4.4905915	555.8510	4.49	POS	hilic				3.25E-01	5.41E-02	-0.58	5.90E-02	-0.52	6.33E-02	-0.60	3.01E-02	-0.65
556.126@0.40521556	556.1260	0.41	NEG	hilic				4.46E-01	8.74E-02	0.49	6.31E-01	-0.15	3.31E-01	0.24	2.51E-01	0.36
556.1795@0.5461429	556.1795	0.55	NEG	hilic				2.49E-01	3.51E-02	0.57	5.60E-01	0.11	6.79E-01	-0.08	4.12E-01	0.20
556.19@9.615514	556.1900	9.62	POS	RP				4.53E-02	1.99E-02	0.63	8.64E-01	0.05	9.97E-02	0.52	1.69E-03	0.92
556.4481@10.7063675	556.4481	10.71	POS	RP				7.74E-03	1.33E-03	-0.72	7.33E-02	0.41	2.16E-01	-0.30	4.53E-01	0.23
556.4484@0.41722223	556.4484	0.42	NEG	hilic				1.26E-02	1.71E-02	-0.71	1.87E-01	0.31	6.99E-01	-0.11	9.43E-02	0.54
556.4491@10.967391	556.4491	10.97	POS	RP				1.84E-02	2.14E-01	-0.19	6.24E-01	0.10	4.41E-01	-0.13	1.74E-02	0.65
557.2242@12.385114	557.2242	12.39	NEG	RP				9.35E-01	3.54E-01	0.24	4.58E-01	0.24	9.26E-01	0.03	5.85E-01	0.15
557.2384@11.429964	557.2384	11.43	NEG	RP				6.39E-01	3.65E-01	0.30	2.85E-01	0.26	5.27E-02	0.55	2.07E-01	0.39
557.2815@9.965831	557.2815	9.97	POS	RP				9.67E-01	9.87E-01	0.00	6.01E-01	-0.14	4.92E-01	-0.18	5.91E-01	-0.15
557.2846@9.881413	557.2846	9.88	POS	RP				2.84E-01	1.51E-01	-0.39	7.32E-01	-0.10	3.09E-02	-0.66	5.40E-02	-0.55
557.2883@1.2293563	557.2883	1.23	POS	hilic				4.64E-01	5.97E-01	0.19	8.44E-01	-0.05	5.92E-02	-0.51	9.55E-01	-0.01
557.289@1.118431	557.2890	1.12	POS	hilic				1.06E-01	8.77E-01	-0.05	3.25E-02	-0.66	7.89E-03	-0.70	9.53E-01	-0.02
557.322@1.3278098	557.3220	1.33	NEG	hilic				3.66E-01	4.91E-02	0.60	4.05E-01	0.24	1.26E-01	0.45	8.35E-01	-0.07
557.3223@1.184685	557.3223	1.18	NEG	hilic				1.25E-01	2.74E-01	0.38	1.31E-01	-0.40	1.92E-01	-0.32	2.70E-01	0.34
557.3266@10.236538	557.3266	10.24	NEG	RP				1.08E-01	4.72E-02	0.55	3.67E-01	-0.29	1.21E-01	-0.38	4.89E-01	0.21
557.3305@1.5247997	557.3305	1.52	NEG	hilic				2.35E-01	6.01E-02	0.57	5.84E-01	0.13	4.24E-02	0.57	4.54E-02	0.65
557.3313@2.0072663	557.3313	2.01	NEG	hilic				2.72E-02	9.09E-03	0.73	6.05E-01	-0.14	7.39E-02	-0.45	7.15E-01	0.11
557.3325@8.582389	557.3325	8.58	NEG	RP				2.85E-01	1.66E-01	0.43	2.50E-01	-0.35	3.26E-01	-0.28	6.96E-01	0.11
557.3325@9.738369	557.3325	9.74	NEG	RP				1.88E-01	3.13E-02	0.60	6.28E-01	-0.15	2.61E-01	-0.31	5.94E-01	0.15
557.4984@3.333543	557.4984	3.33	NEG	hilic				4.46E-01	5.83E-01	-0.17	6.26E-02	-0.48	5.70E-01	0.16	8.88E-01	-0.04
557.7874@9.940683	557.7874	9.94	POS	RP				2.46E-02	7.74E-01	0.08	7.81E-03	0.86	1.13E-03	0.87	2.57E-01	0.32
558.1054@0.4042735	558.1054	0.40	NEG	hilic				4.63E-01	4.27E-02	0.61	8.01E-01	0.07	1.54E-01	0.46	4.89E-01	0.21
558.3398@10.3169365	558.3398	10.32	NEG	RP				3.40E-03	4.34E-03	-0.50	4.60E-02	0.55	6.68E-03	0.59	9.72E-01	-0.01
558.3905@0.43772468	558.3905	0.44	POS	hilic				1.07E-01	3.70E-02	-0.46	3.80E-01	0.24	9.17E-01	-0.02	1.24E-01	0.44
558.4639@10.821075	558.4639	10.82	POS	RP				1.40E-02	6.39E-03	-0.65	1.24E-01	0.39	9.56E-01	0.01	1.93E-01	0.40
558.464@11.491802	558.4640	11.49	NEG	RP				8.57E-04	1.47E-03	-0.95	1.02E-01	0.36	8.98E-01	-0.03	8.22E-02	0.53
558.4641@0.41735137	558.4641	0.42	NEG	hilic				1.41E-02	6.52E-03	-0.77	5.68E-01	0.14	4.55E-01	-0.21	9.52E-02	0.51
558.4652@11.486458	558.4652	11.49	POS	RP				1.74E-02	1.10E-02	-0.70	4.09E-01	0.19	3.82E-01	-0.21	1.34E-01	0.49
559.245@2.009239	559.2450	2.01	NEG	hilic				6.44E-01	1.94E-01	-0.42	1.18E-01	-0.40	8.25E-02	-0.46	3.33E-01	-0.26
559.2982@10.248369	559.2982	10.25	POS	RP				5.63E-01	1.42E-01	0.43	8.51E-01	-0.06	4.17E-01	-0.23	6.74E-01	0.12
559.3016@10.247227	559.3016	10.25	POS	RP				1.68E-01	3.73E-02	0.60	7.14E-01	-0.11	2.52E-01	-0.32	3.71E-01	0.25
559.3041@1.0939909	559.3041	1.09	POS	hilic				1.30E-01	5.92E-01	0.16	3.43E-02	-0.67	4.62E-02	-0.56	4.69E-01	-0.22
559.3046@1.2055752	559.3046	1.21	POS	hilic				3.71E-01	7.83E-02	-0.47	3.84E-01	0.23	8.57E-01	-0.05	2.95E-01	-0.32
559.3362@1.1703303	559.3362	1.17	NEG	hilic				6.65E-02	8.08E-03	0.84	4.94E-01	-0.19	5.10E-01	0.17	2.37E-01	0.37
559.3374@1.3077692	559.3374	1.31	NEG	hilic				1.16E-01	5.00E-02	0.61	1.81E-01	0.37	3.81E-03	0.91	4.24E-01	0.24
559.3389@10.412071	559.3389	10.41	NEG	RP				2.73E-01	1.49E-01	0.43	1.74E-01	-0.44	9.49E-01	0.02	7.47E-01	0.10
559.3395@10.513581	559.3395	10.51	NEG	RP				1.34E-01	7.82E-03	0.69	3.98E-01	-0.25	4.34E-01	0.21	2.29E-01	0.36
559.4632@14.373761	559.4632	14.37	POS	RP				1.67E-01	3.87E-01	0.23	2.32E-02	0.62	3.16E-01	-0.29	9.47E-01	0.02
559.4648@13.249466	559.4648	13.25	POS	RP				6.45E-01	2.12E-01	0.31	2.18E-01	0.36	7.58E-01	-0.08	8.07E-01	-0.08
559.8062@1.1945535	559.8062	1.19	POS	hilic				8.83E-01	5.16E-01	-0.16	8.87E-01	-0.04	8.08E-01	-0.06	4.99E-01	0.18
560.281@0.48935488	560.2810	0.49	POS	hilic				4.75E-02	4.71E-02	-0.39	6.09E-02	-0.38	2.75E-01	0.30	9.44E-02	-0.35
560.4051@0.43620008	560.4051	0.44	NEG	hilic				1.19E-01	2.75E-02	-0.41	2.36E-01	0.30	5.61E-01	-0.13	2.20E-01	0.30
560.4084@0.4371385	560.4084	0.44	POS	hilic				3.68E-04	1.80E-04	-0.85	5.98E-03	0.70	3.31E-01	0.25	6.80E-01	0.11
560.4781@0.41718018	560.4781	0.42	NEG	hilic				2.97E-01	1.70E-01	-0.45	3.10E-01	0.26	9.38E-01	-0.02	1.85E-01	0.35
560.4783@11.702765	560.4783	11.70	NEG	RP				2.13E-01	1.70E-01	-0.44	6.46E-02	0.43	8.09E-01	0.07	3.13E-01	0.32
560.4792@0.4190909	560.4792	0.42	POS	hilic				7.42E-04	1.05E-04	-0.91	9.14E-02	0.44	2.53E-01	0.28	1.82E-01	0.37
560.7938@9.956808	560.7938	9.96	POS	RP				8.53E-01	1.95E-01	0.32	3.95E-01	0.27	9.72E-01	-0.01	4.89E-01	0.21
560.8137@10.539402	560.8137	10.54	POS	RP				2.51E-01	3.47E-02	0.61	5.55E-01	-0.18	3.07E-01	0.29	2.49E-01	0.38
561.2407@1.9918408	561.2407	1.99	NEG	hilic				3.16E-01	1.44E-01	0.40	7.23E-02	0.41	8.84E-01	-0.03	7.68E-01	-0.06
561.2744@1.4823035	561.2744	1.48	NEG	hilic				6.83E-01	9.95E-01	0.00	2.87E-01	-0.28	1.58E-01	-0.35	2.42E-01	-0.30
561.3073@12.381216	561.3073	12.38	NEG	RP				2.73E-01	3.97E-01	0.22	6.18E-01	0.15	4.85E-01	-0.18	5.65E-02	-0.49
561.3132@10.537691	561.3132	10.54	POS	RP				4.57E-01	3.27E-02	0.59	9.56E-01	-0.02	6.39E-01	0.13	2.14E-01	0.39
561.3173@1.0936226	561.3173	1.09	POS	hilic				4.21E-01	3.86E-01	0.24	3.67E-01	-0.29	1.23E-01	-0.41	2.44E-01	-0.35
561.3183@1.1987454	561.3183	1.20	POS	hilic				9.12E-02	2.53E-02	-0.70	8.07E-01	0.07	3.22E-01	0.31	2.79E-01	-0.32
561.3209@10.532896	561.3209	10.53	POS	RP				3.01E-01	2.99E-02	0.59	6.05E-01	-0.17	7.91E-01	0.07	2.17E-01	0.38
561.3367@1.3015682	561.3367	1.30	NEG	hilic				4.87E-02	6.10E-01	-0.13	2.08E-01	0.29	4.12E-03	0.85	3.03E-01	0.27
561.4156@0.94150007	561.4156	0.94	POS	hilic				4.52E-01	4.95E-01	0.19	9.91E-01	0.00	5.31E-02	-0.43	7.78E-01	0.08

Supplementary table 2: Metabolomics analysis results

								ANOVA	Anorexia vs control		overweight vs control		obese vs control		athletes vs control	
Compound	Mass	Retention Time	Ionization mode	column	Compound_ID	MSMS_spectra	ID_level	P-FDR	P	Estimate	P	Estimate	P	Estimate	P	Estimate
561.4165@1.171258	561.4165	1.17	POS	hilic				3.61E-01	5.67E-01	-0.16	1.63E-02	-0.56	3.24E-02	-0.50	3.56E-01	-0.27
561.4823@0.416982	561.4823	0.42	NEG	hilic				3.96E-01	2.76E-01	-0.36	2.66E-01	0.28	9.41E-01	0.02	1.58E-01	0.37
562.2071@0.46508572	562.2071	0.47	POS	hilic				2.02E-01	4.78E-01	-0.20	3.60E-01	-0.23	2.53E-03	-0.73	7.99E-01	-0.07
562.2245@11.824465	562.2245	11.82	POS	RP				6.11E-01	4.33E-01	-0.22	4.31E-01	-0.26	4.21E-01	0.22	4.78E-01	0.22
562.3235@1.200549	562.3235	1.20	POS	hilic				7.42E-01	9.37E-01	0.02	3.34E-01	0.25	5.59E-01	-0.16	4.68E-01	0.17
562.3855@11.217516	562.3855	11.22	NEG	RP				6.53E-01	2.01E-01	0.34	1.08E-01	0.50	4.00E-01	0.21	3.11E-01	0.22
562.4222@0.42768338	562.4222	0.43	NEG	hilic				5.43E-02	1.11E-01	-0.32	4.09E-01	0.19	6.57E-01	-0.10	2.92E-02	0.61
562.4931@0.4168438	562.4931	0.42	NEG	hilic				8.00E-01	7.36E-01	-0.10	1.65E-01	0.33	6.13E-01	0.14	6.29E-01	0.14
563.2406@1.7934651	563.2406	1.79	POS	hilic				8.40E-01	3.47E-01	0.28	8.61E-01	-0.04	6.64E-01	0.12	3.40E-01	0.24
563.3022@12.3808975	563.3022	12.38	NEG	RP				6.89E-01	1.32E-01	-0.41	5.59E-01	-0.16	5.21E-01	-0.20	9.58E-02	-0.50
563.3067@1.0662534	563.3067	1.07	NEG	hilic				5.36E-01	1.15E-01	0.44	9.63E-02	0.44	7.99E-01	0.06	2.82E-01	0.26
563.3068@0.42251998	563.3068	0.42	POS	hilic				4.38E-01	4.86E-01	-0.16	1.18E-01	0.41	2.55E-01	0.26	4.07E-01	0.19
563.3211@9.582872	563.3211	9.58	NEG	RP				9.68E-03	1.82E-02	0.74	1.50E-01	-0.42	1.86E-01	-0.36	3.94E-01	0.27
563.3215@9.713085	563.3215	9.71	NEG	RP	LysoPC 18:3	10 eV: 502.2927 (100), 562.3161 (40), 277.2168 (8)	2	3.33E-03	1.89E-02	0.71	4.91E-02	-0.59	1.34E-01	-0.38	4.04E-01	0.26
563.3222@1.4240967	563.3222	1.42	NEG	hilic				1.56E-01	3.29E-01	0.26	3.68E-01	-0.21	2.40E-02	-0.46	8.74E-01	-0.04
563.4047@13.817686	563.4047	13.82	POS	RP				4.41E-01	7.58E-01	0.08	3.43E-01	0.25	1.34E-01	-0.42	4.39E-01	-0.24
563.4228@11.680196	563.4228	11.68	POS	RP				2.96E-01	5.11E-02	-0.55	7.69E-01	0.08	5.99E-01	0.13	4.07E-01	-0.23
563.4314@1.1600444	563.4314	1.16	POS	hilic				2.25E-01	8.17E-01	0.07	7.95E-02	-0.48	1.27E-02	-0.68	3.41E-01	-0.30
564.1062@9.049095	564.1062	9.05	NEG	RP				7.61E-01	5.27E-01	0.19	4.92E-01	0.20	6.45E-01	0.13	3.76E-01	-0.25
564.1076@8.785008	564.1076	8.79	NEG	RP				7.12E-01	1.52E-01	0.44	7.46E-01	-0.10	7.60E-01	0.08	9.71E-01	-0.01
564.1076@9.164432	564.1076	9.16	NEG	RP				7.64E-01	2.84E-01	0.31	2.55E-01	0.34	9.42E-01	-0.02	9.96E-01	0.00
564.3051@12.380813	564.3051	12.38	NEG	RP				6.89E-01	1.44E-01	-0.41	4.82E-01	-0.19	4.29E-01	-0.24	9.00E-02	-0.51
564.3959@10.962181	564.3959	10.96	POS	RP				6.07E-01	8.70E-01	-0.05	7.60E-01	-0.07	8.71E-02	-0.39	5.46E-01	0.19
564.3972@11.507637	564.3972	11.51	POS	RP				3.88E-01	2.83E-01	0.32	1.77E-01	0.37	1.35E-01	0.37	1.75E-02	0.73
564.4389@0.42706814	564.4389	0.43	NEG	hilic				8.60E-03	2.87E-03	-0.78	3.69E-01	0.24	3.20E-01	-0.25	1.37E-01	0.39
564.5596@13.923225	564.5596	13.92	POS	RP				4.10E-01	4.20E-01	0.27	8.10E-02	0.50	6.28E-02	0.52	1.02E-02	0.64
565.2586@12.381351	565.2586	12.38	NEG	RP				8.76E-01	6.37E-01	0.12	9.57E-01	-0.01	3.48E-01	-0.25	9.62E-01	-0.01
565.2774@0.4718462	565.2774	0.47	POS	hilic				8.34E-01	1.76E-01	-0.40	8.45E-01	-0.06	7.62E-01	-0.08	5.67E-01	-0.16
565.3142@1.0669391	565.3142	1.07	POS	hilic				4.64E-01	2.56E-02	-0.66	6.55E-01	-0.13	9.26E-01	-0.03	5.97E-01	-0.16
565.315@1.1864616	565.3150	1.19	POS	hilic				4.28E-05	2.76E-06	-1.21	5.95E-02	0.41	3.65E-01	0.27	1.40E-01	-0.40
565.3152@9.934439	565.3152	9.93	POS	RP				6.30E-04	7.86E-01	0.07	7.83E-04	0.93	1.14E-04	0.99	5.38E-01	-0.17
565.3373@9.959135	565.3373	9.96	NEG	RP	LysoPC 18:2 (M+FA)	10 eV: 504.3088 (100), 564.3299 (60), 279.2332 (12)	2	6.75E-03	7.84E-01	0.08	2.59E-02	-0.68	8.48E-04	-0.88	5.65E-01	0.17
565.3373@1.2182992	565.3373	1.22	NEG	hilic				1.23E-02	3.39E-01	0.27	3.04E-02	-0.64	2.47E-03	-0.79	8.70E-01	0.05
565.3374@9.846836	565.3374	9.85	NEG	RP				5.45E-03	8.76E-01	0.04	1.68E-02	-0.73	7.04E-04	-0.91	6.47E-01	0.14
565.3383@1.3680853	565.3383	1.37	NEG	hilic				2.46E-02	8.54E-01	-0.05	1.92E-02	-0.68	4.73E-04	-0.93	8.14E-01	-0.08
565.3503@11.085673	565.3503	11.09	NEG	RP				7.34E-01	3.68E-01	0.24	2.18E-01	0.34	1.94E-01	0.39	9.87E-01	-0.01
565.351@0.41110277	565.3510	0.41	NEG	hilic				1.55E-01	1.26E-02	0.65	7.61E-02	0.48	3.36E-03	0.73	8.64E-01	0.06
565.4066@0.4203429	565.4066	0.42	POS	hilic				9.88E-01	8.11E-01	-0.05	8.01E-01	0.05	7.21E-01	0.07	7.45E-01	0.08
565.4232@0.95259637	565.4232	0.95	POS	hilic				9.22E-01	6.29E-01	0.13	8.68E-01	0.04	4.86E-01	-0.18	9.42E-01	0.02
565.4446@1.142202	565.4446	1.14	POS	hilic				2.61E-01	9.85E-01	0.01	6.02E-02	-0.54	2.57E-02	-0.56	1.34E-01	-0.53
565.8318@15.283844	565.8318	15.28	NEG	RP				6.92E-01	8.99E-01	-0.04	2.66E-01	-0.32	5.72E-01	0.16	3.32E-01	-0.30
565.8818@4.3538294	565.8818	4.35	POS	hilic				7.31E-01	9.62E-02	0.47	2.14E-01	0.34	7.93E-01	0.07	6.11E-01	0.17
566.1118@7.19163	566.1118	7.19	NEG	RP				4.99E-01	2.81E-01	-0.24	4.34E-01	0.20	6.90E-01	0.11	1.52E-01	-0.30
566.1369@0.40661108	566.1369	0.41	NEG	hilic				6.67E-01	1.65E-01	0.40	3.47E-01	0.24	1.66E-01	0.35	1.12E-01	0.44
566.1782@1.9576552	566.1782	1.96	POS	hilic				8.14E-02	3.02E-02	0.58	6.40E-01	-0.08	5.33E-01	-0.11	3.26E-01	0.24
566.3567@0.5755917	566.3567	0.58	POS	hilic				9.23E-01	9.98E-01	0.00	5.65E-01	-0.13	6.30E-01	0.12	7.28E-01	0.09
566.4106@10.947769	566.4106	10.95	POS	RP				9.53E-01	8.09E-01	-0.07	7.14E-01	0.08	5.85E-01	-0.12	7.77E-01	0.07
566.4537@0.42692205	566.4537	0.43	NEG	hilic				2.41E-03	9.21E-03	-0.71	2.29E-02	0.62	6.89E-01	0.10	7.86E-02	0.45
566.4553@0.42872506	566.4553	0.43	POS	hilic				8.28E-02	8.34E-02	-0.34	7.78E-01	0.07	9.11E-02	-0.33	1.32E-01	0.39
566.4553@10.72936	566.4553	10.73	POS	RP				5.12E-02	1.77E-02	-0.53	1.47E-01	0.38	5.30E-01	-0.15	2.95E-01	0.31
567.1853@10.614233	567.1853	10.61	POS	RP				1.40E-02	4.83E-01	-0.22	3.36E-03	0.83	1.73E-02	0.70	9.60E-02	0.48
567.2242@11.429832	567.2242	11.43	NEG	RP				1.80E-01	1.86E-02	0.59	6.54E-03	0.78	3.90E-02	0.54	6.62E-02	0.54
567.253@12.381181	567.2530	12.38	NEG	RP				8.72E-01	1.98E-01	0.35	3.13E-01	0.30	7.30E-01	0.11	7.30E-01	0.11
567.2895@6.8337703	567.2895	6.83	POS	RP				2.11E-01	1.14E-02	0.83	3.20E-02	0.57	1.25E-01	0.47	3.46E-01	0.26
567.2907@0.42665783	567.2907	0.43	POS	hilic				5.57E-01	9.85E-01	0.00	6.73E-01	-0.09	3.62E-01	0.25	1.76E-01	0.40
567.3318@10.177521	567.3318	10.18	POS	RP				1.17E-01	2.33E-01	-0.43	2.50E-01	0.30	7.60E-02	0.44	2.70E-01	-0.29
567.3321@9.851543	567.3321	9.85	POS	RP				7.22E-01	1.19E-01	-0.46	2.04E-01	-0.40	3.09E-01	-0.31	5.68E-01	-0.16
567.3324@1.176846	567.3324	1.18	POS	hilic				7.12E-01	1.16E-01	-0.47	2.16E-01	-0.37	2.45E-01	-0.35	1.81E-01	-0.41
567.3327@1.057146	567.3327	1.06	POS	hilic				8.41E-01	4.34E-01	-0.23	2.13E-01	-0.38	3.65E-01	-0.27	2.95E-01	-0.32
567.3335@9.937043	567.3335	9.94	POS	RP	LysoPC 22:6	10 eV: 568.3403 (100), 104.1068 (15), 184.0728 (8)	2	2.00E-01	1.69E-02	-0.74	8.75E-01	-0.04	7.06E-01	0.12	2.79E-01	-0.31
567.3526@1.1815382	567.3526	1.18	NEG	hilic				1.13E-02	2.09E-02	0.69	8.35E-02	-0.51	9.85E-02	-0.42	8.45E-01	0.06
567.3526@10.125068	567.3526	10.13	NEG	RP				2.13E-02	7.99E-02	0.56	1.99E-01	-0.40	1.44E-02	-0.63	9.59E-01	0.01
567.3527@1.3276325	567.3527	1.33	NEG	hilic				9.47E-03	2.12E-02	0.68	6.22E-02	-0.55	7.58E-02	-0.45	9.92E-01	0.00
567.3532@10.228359	567.3532	10.23	NEG	RP	LysoPC 18:1 (M+FA)	10 eV: 506.3252 (100), 566.3455 (95), 281.2507 (12)	2	6.37E-03	2.18E-02	0.70	1.34E-01	-0.46	3.75E-02	-0.53	5.47E-01	0.17
567.4175@0.42212778	567.4175	0.42	POS	hilic				3.68E-03	3.39E-01	0.30	5.93E-02	-0.44	3.50E-03	-0.69	1.36E-01	0.40
567.4195@11.224579	567.4195	11.22	POS	RP				1.55E-01	8.15E-01	0.07	1.15E-01	-0.44	2.58E-02	-0.63	6.65E-01	0.14
567.4197@11.008578	567.4197	11.01	POS	RP				3.33E-03	5.70E-01	0.17	2.13E-01	-0.31	1.22E-03	-0.93	1.02E-01	0.39
567.4203@11.343333	567.4203	11.34	POS	RP				1.88E-02	8.96E-01	0.04	1.11E-01	-0.43	1.38E-03	-1.01	8.88E-01	-0.04
567.4208@11.347024	567.4208	11.35	POS	RP				4.29E-02	7.77E-01	0.10	4.40E-02	-0.56	2.97E-03	-0.86	7.06E-01	-0.11
567.4214@10.87477	567.4214	10.87	POS	RP				4.20E-03	9.78E-01	-0.01	3.37E-02	-0.50	1.52E-03	-0.98	3.00E-01	0.29
567.4496@10.055083	567.4496	10.06	POS	RP				8.01E-01	6.93E-01	-0.11	6.60E-01	-0.12	4.36E-01	0.22	5.75E-01	0.16
568.1283@0.40624332	568.1283	0.41	NEG	hilic				1.95E-01	7.41E-02	0.57	7.75E-01	0.08	1.93E-01	-0.33	2.19E-01	0.34
568.3557@2.989641	568.3557	2.99	POS	hilic				6.97E-01	8.76E-01	0.05	9.56E-01	-0.02	5.14E-01	-0.22	1.80E-01	-0.45

Supplementary table 2: Metabolomics analysis results

Compound	Mass	Retention Time	Ionization mode	column	Compound_ID	MSMS_spectra	ID_level	ANOVA	Anorexia vs control		overweight vs control		obese vs control		athletes vs control	
								P-FDR	P	Estimate	P	Estimate	P	Estimate	P	Estimate
568.3562@3.1326954	568.3562	3.13	POS	hilic				9.66E-01	9.31E-01	-0.03	3.81E-01	-0.22	5.79E-01	-0.18	7.69E-01	-0.08
568.3589@11.717371	568.3589	11.72	POS	RP				5.10E-01	1.21E-01	0.37	6.95E-02	0.57	8.06E-01	0.06	6.91E-01	0.12
568.3603@13.820732	568.3603	13.82	POS	RP				6.08E-01	6.83E-01	-0.11	8.24E-01	0.06	1.12E-01	-0.43	2.17E-01	-0.38
568.4211@0.42488232	568.4211	0.42	POS	hilic				5.62E-01	2.32E-01	-0.27	9.20E-01	0.02	4.77E-01	-0.16	3.56E-01	0.28
568.4271@10.7513075	568.4271	10.75	POS	RP				8.99E-01	5.13E-01	-0.17	5.11E-01	-0.16	7.85E-01	0.07	8.81E-01	0.04
568.4679@10.78485	568.4679	10.78	NEG	RP				7.41E-03	3.34E-03	-0.69	5.97E-02	0.52	4.47E-01	0.18	3.02E-01	0.27
568.469@0.42689985	568.4690	0.43	NEG	hilic				6.30E-03	1.37E-02	-0.59	2.26E-02	0.64	2.32E-01	0.30	1.77E-01	0.34
568.778@15.289054	568.7780	15.29	NEG	RP				3.75E-01	6.12E-01	-0.14	2.02E-01	-0.32	2.91E-01	0.28	2.52E-01	-0.33
569.2493@1.787285	569.2493	1.79	POS	hilic				1.41E-01	2.59E-03	-0.82	7.87E-01	0.07	9.80E-01	-0.01	5.62E-01	-0.16
569.2517@12.380958	569.2517	12.38	NEG	RP				7.99E-01	1.21E-01	0.44	4.02E-01	0.26	7.40E-01	0.11	7.59E-01	0.09
569.2691@9.960337	569.2691	9.96	NEG	RP				2.47E-01	5.04E-01	0.19	1.48E-02	0.65	2.28E-01	0.30	9.32E-01	-0.02
569.2729@2.0112522	569.2729	2.01	NEG	hilic				8.69E-01	5.92E-01	0.18	5.86E-01	0.15	5.39E-01	-0.20	7.58E-01	0.09
569.3015@0.67111754	569.3015	0.67	POS	hilic				5.56E-01	1.82E-01	-0.15	8.92E-01	0.02	3.67E-01	0.17	3.06E-01	0.22
569.3028@6.4810905	569.3028	6.48	POS	RP				2.92E-01	7.65E-02	-0.29	4.42E-01	-0.14	2.68E-01	0.27	5.31E-01	0.15
569.3464@1.1774207	569.3464	1.18	POS	hilic				3.45E-01	4.47E-01	0.26	3.50E-01	-0.26	5.38E-02	-0.49	3.04E-01	-0.29
569.3471@10.395499	569.3471	10.40	POS	RP				1.53E-02	4.40E-02	0.58	8.12E-02	-0.50	5.42E-02	-0.53	7.99E-01	0.07
569.3482@10.183327	569.3482	10.18	POS	RP				9.13E-01	5.59E-01	-0.21	5.52E-01	-0.17	4.91E-01	-0.19	7.78E-01	0.09
569.3483@10.080083	569.3483	10.08	POS	RP	dublicate of LysoPC 22:5			3.18E-01	1.46E-01	0.50	4.34E-01	-0.23	4.67E-01	-0.17	4.31E-01	-0.24
569.3485@10.080083	569.3485	10.08	POS	RP	LysoPC 22:5	10 eV: 570.3552 (100), 184.0703 (10)	2	3.18E-01	1.48E-01	0.50	4.29E-01	-0.23	4.67E-01	-0.17	4.24E-01	-0.24
569.3683@1.1689658	569.3683	1.17	NEG	hilic				6.36E-02	2.65E-02	0.68	1.35E-01	-0.43	8.26E-01	-0.06	6.07E-01	0.16
569.3684@1.3129102	569.3684	1.31	NEG	hilic				9.89E-02	1.11E-01	0.51	6.13E-02	-0.50	6.72E-01	-0.11	5.25E-01	0.19
569.3684@10.411224	569.3684	10.41	NEG	RP				7.70E-02	7.47E-02	0.55	9.30E-02	-0.53	5.68E-01	-0.15	7.51E-01	0.09
569.3686@10.504905	569.3686	10.50	NEG	RP	LysoPC 18:0 (M+FA)	10 eV: 508.3399 (100), 568.3605 (75), 283.2627 (5)	2	4.25E-02	2.77E-02	0.64	1.25E-01	-0.47	5.53E-01	-0.15	4.28E-01	0.24
569.4327@0.4067755	569.4327	0.41	POS	hilic				7.36E-01	8.06E-01	0.06	8.08E-01	0.06	1.97E-01	-0.30	5.20E-01	-0.17
569.4366@11.548334	569.4366	11.55	POS	RP				1.37E-01	2.78E-02	-0.68	4.33E-01	-0.20	2.36E-02	-0.66	8.10E-01	-0.07
570.0316@4.695193	570.0316	4.70	POS	hilic				7.98E-01	4.97E-01	0.18	4.28E-01	-0.22	6.38E-01	0.16	7.45E-01	0.08
570.1415@0.4060001	570.1415	0.41	NEG	hilic				2.14E-01	2.05E-02	0.63	7.14E-01	0.11	4.77E-01	-0.18	1.42E-01	0.45
570.2067@9.891967	570.2067	9.89	POS	RP				2.05E-02	3.93E-04	0.97	7.43E-01	0.09	5.70E-02	0.61	1.85E-03	0.79
570.3154@10.703818	570.3154	10.70	POS	RP				1.08E-01	8.56E-01	-0.05	1.38E-01	-0.38	5.57E-04	-0.74	4.64E-01	-0.19
570.3718@1.1696547	570.3718	1.17	NEG	hilic				1.39E-01	9.22E-02	0.54	7.54E-02	-0.44	9.52E-01	0.02	5.21E-01	0.19
570.4255@0.42449066	570.4255	0.42	NEG	hilic				8.11E-01	3.23E-01	-0.24	8.64E-01	0.04	6.17E-01	-0.12	6.08E-01	0.14
570.438@0.42460394	570.4380	0.42	POS	hilic				4.46E-02	1.75E-04	-0.96	1.13E-01	-0.39	2.27E-02	-0.64	6.34E-01	-0.15
571.3622@10.197063	571.3622	10.20	POS	RP				7.34E-01	9.20E-01	0.03	1.91E-01	-0.41	7.56E-01	-0.08	9.25E-01	0.03
571.3627@1.1557413	571.3627	1.16	POS	hilic				7.92E-01	9.46E-01	0.02	5.93E-01	-0.15	1.65E-01	-0.41	8.36E-01	-0.06
571.3646@10.31176	571.3646	10.31	POS	RP				5.10E-01	3.65E-01	0.31	2.87E-01	-0.34	8.93E-01	-0.04	4.83E-01	0.21
571.7803@15.287823	571.7803	15.29	NEG	RP				6.76E-01	8.99E-01	-0.04	4.80E-01	0.18	4.65E-01	-0.20	2.86E-01	0.30
571.8527@7.9274273	571.8527	7.93	NEG	hilic				2.26E-01	1.29E-01	0.42	8.09E-01	-0.05	3.16E-02	0.58	9.49E-01	0.02
571.9534@6.2704167	571.9534	6.27	NEG	hilic				6.97E-01	1.88E-01	-0.33	7.51E-01	-0.10	2.13E-01	-0.34	1.62E-01	-0.40
572.1215@0.39844432	572.1215	0.40	NEG	hilic				7.01E-01	3.29E-01	0.26	1.98E-01	0.40	7.25E-01	0.10	1.53E-01	0.46
572.3212@0.42764795	572.3212	0.43	POS	hilic				4.47E-01	3.15E-01	0.32	9.48E-01	0.02	1.25E-01	-0.35	4.75E-01	-0.18
572.3677@1.1524496	572.3677	1.15	POS	hilic				5.19E-01	9.83E-01	-0.01	7.44E-01	-0.09	4.80E-02	-0.57	9.04E-01	-0.03
572.4436@0.42461085	572.4436	0.42	NEG	hilic				7.44E-02	5.77E-03	-0.78	3.18E-01	0.25	6.63E-01	-0.11	9.72E-01	0.01
572.4449@0.42496487	572.4449	0.42	POS	hilic				1.36E-02	1.06E-02	-0.74	2.76E-01	0.28	9.44E-01	0.02	1.25E-01	0.53
573.2424@1.4748715	573.2424	1.47	NEG	hilic				1.10E-01	2.71E-01	0.39	7.91E-02	-0.40	4.67E-01	-0.20	9.93E-02	0.46
573.2505@9.897803	573.2505	9.90	POS	RP				5.55E-01	1.98E-01	0.33	3.97E-01	-0.26	7.70E-01	-0.07	4.53E-01	0.23
573.2536@9.958854	573.2536	9.96	POS	RP				1.29E-02	4.88E-01	0.19	2.27E-01	-0.34	1.51E-03	-0.83	2.68E-01	0.33
573.3047@0.40607545	573.3047	0.41	POS	hilic				9.41E-01	3.89E-01	-0.19	9.47E-01	-0.02	8.16E-01	0.06	6.55E-01	-0.10
573.3047@10.7293	573.3047	10.73	POS	RP				9.01E-01	4.84E-01	-0.21	5.86E-01	0.17	7.61E-01	-0.09	8.96E-01	-0.03
573.3097@1.3296021	573.3097	1.33	POS	hilic				2.36E-01	2.01E-01	-0.34	8.98E-01	-0.03	1.08E-01	-0.48	1.71E-02	-0.68
573.3135@1.1564348	573.3135	1.16	POS	hilic				4.14E-01	2.47E-01	-0.37	2.59E-01	0.34	8.93E-01	-0.04	4.33E-01	-0.25
573.4769@14.375052	573.4769	14.38	POS	RP				2.66E-01	9.96E-01	0.00	6.32E-02	0.46	4.24E-01	-0.15	7.52E-01	-0.07
573.4781@13.759596	573.4781	13.76	POS	RP				2.79E-01	2.88E-01	0.29	2.60E-02	0.60	6.93E-01	-0.10	9.15E-01	0.03
574.1356@9.1739645	574.1356	9.17	NEG	RP				9.53E-01	9.04E-01	0.04	9.39E-01	0.02	2.82E-01	0.27	8.26E-01	0.08
574.1361@8.785538	574.1361	8.79	NEG	RP				7.96E-01	2.54E-01	-0.39	6.07E-01	-0.14	7.68E-01	0.08	8.96E-01	-0.04
574.1361@9.049565	574.1361	9.05	NEG	RP				7.38E-01	2.74E-01	-0.36	7.36E-01	0.09	6.15E-01	0.13	7.30E-01	0.11
574.1361@9.442353	574.1361	9.44	NEG	RP				5.80E-01	9.26E-01	0.02	7.77E-02	0.50	2.29E-01	0.38	5.93E-01	0.17
574.1367@0.4041795	574.1367	0.40	NEG	hilic				4.72E-01	6.73E-02	0.50	7.96E-01	-0.08	4.65E-01	-0.19	6.10E-01	0.16
574.3072@10.462471	574.3072	10.46	NEG	RP				3.26E-02	2.14E-02	-0.69	3.72E-01	-0.25	8.36E-05	-0.98	2.30E-01	-0.31
574.3112@10.453795	574.3112	10.45	POS	RP				2.24E-01	3.41E-01	-0.27	3.12E-01	-0.30	1.57E-03	-0.80	6.24E-01	-0.14
574.4578@10.707111	574.4578	10.71	NEG	RP				2.40E-01	2.13E-01	0.28	8.60E-02	-0.36	2.49E-01	-0.25	8.49E-01	-0.05
574.4581@11.023413	574.4581	11.02	POS	RP				2.89E-02	5.10E-03	-0.67	3.45E-01	0.23	9.19E-01	-0.02	1.63E-01	0.45
574.4587@10.961017	574.4587	10.96	NEG	RP				4.13E-02	6.41E-03	-0.89	7.26E-01	-0.08	7.29E-01	-0.09	4.27E-01	0.25
574.4588@10.948726	574.4588	10.95	POS	RP				4.98E-02	2.76E-02	-0.54	5.29E-01	0.15	9.30E-01	-0.02	9.52E-02	0.54
574.4589@11.025638	574.4589	11.03	NEG	RP				1.57E-01	1.16E-03	-0.71	5.87E-01	-0.13	1.31E-01	-0.34	9.49E-01	0.02
574.4594@10.709322	574.4594	10.71	POS	RP				1.24E-02	4.21E-03	-0.71	2.37E-01	0.29	3.52E-01	-0.24	1.61E-01	0.44
574.4599@0.42388868	574.4599	0.42	NEG	hilic				2.37E-03	2.65E-03	-0.92	2.31E-01	0.29	7.46E-01	-0.08	1.20E-01	0.49
574.46@10.739691	574.4600	10.74	POS	RP				3.19E-03	1.32E-03	-0.53	8.52E-01	-0.04	1.27E-01	-0.30	4.33E-02	0.57
574.4601@0.42648688	574.4601	0.43	POS	hilic				2.62E-03	2.23E-03	-0.93	2.45E-01	0.29	8.01E-01	0.06	1.52E-01	0.46
574.4947@13.551058	574.4947	13.55	POS	RP				1.08E-01	5.88E-02	0.54	1.98E-01	0.34	5.32E-03	0.84	8.78E-01	0.04
574.4954@13.504178	574.4954	13.50	POS	RP				1.21E-02	1.16E-01	0.42	9.78E-03	0.69	5.43E-04	1.06	4.21E-01	0.20
574.4967@2.9066863	574.4967	2.91	POS	hilic				6.55E-01	6.55E-01	0.13	9.20E-01	-0.03	2.17E-01	-0.33	4.15E-01	0.24
575.2567@1.4748715	575.2567	1.47	NEG	hilic				1.03E-01	2.61E-01	0.40	7.88E-02	-0.40	5.10E-01	-0.18	8.95E-02	0.46
575.2692@10.240261	575.2692	10.24	POS	RP				1.61E-01	1.05E-02	0.69	7.36E-01	-0.10	4.43E-01	-0.22	4.14E-01	0.22

Supplementary table 2: Metabolomics analysis results

								ANOVA	Anorexia vs control		overweight vs control		obese vs control		athletes vs control	
Compound	Mass	Retention Time	Ionization mode	column	Compound_ID	MSMS_spectra	ID_level	P-FDR	P	Estimate	P	Estimate	P	Estimate	P	Estimate
575.2707@1.155123	575.2707	1.16	POS	hilic				5.56E-01	2.55E-01	-0.30	3.02E-01	0.29	8.93E-01	-0.04	6.54E-01	-0.11
575.272@1.3581828	575.2720	1.36	POS	hilic				7.17E-02	9.42E-03	-0.62	9.15E-01	0.03	1.55E-01	-0.37	7.33E-03	-0.76
575.2864@12.3812275	575.2864	12.38	NEG	RP				9.49E-01	6.20E-01	-0.14	8.69E-01	-0.04	3.61E-01	-0.25	6.99E-01	-0.11
575.3943@1.1167018	575.3943	1.12	POS	hilic				6.22E-03	7.23E-03	0.80	1.45E-01	-0.39	1.41E-01	-0.37	5.40E-01	0.20
575.4597@11.717805	575.4597	11.72	POS	RP				3.15E-01	8.88E-03	0.64	1.12E-01	0.51	9.65E-01	-0.01	3.24E-01	0.29
575.4908@11.486486	575.4908	11.49	POS	RP				3.93E-04	8.47E-04	-0.98	1.20E-01	0.34	7.18E-01	-0.09	5.83E-02	0.60
575.491@11.486486	575.4910	11.49	POS	RP				3.98E-04	7.95E-04	-0.98	1.27E-01	0.33	7.71E-01	-0.07	6.08E-02	0.59
576.4543@10.706784	576.4543	10.71	NEG	RP				1.45E-01	9.88E-02	0.39	7.28E-02	0.54	4.19E-03	0.69	9.56E-01	-0.02
576.4633@13.2424755	576.4633	13.24	POS	RP				2.26E-02	7.19E-02	0.50	3.47E-04	0.97	8.29E-01	-0.05	1.49E-01	0.47
576.4732@11.028647	576.4732	11.03	POS	RP				6.53E-03	1.35E-02	-0.56	1.89E-02	0.61	6.65E-02	0.40	2.35E-01	0.35
576.4743@0.4224015	576.4743	0.42	NEG	hilic				1.24E-03	2.66E-03	-0.92	7.45E-02	0.44	6.90E-01	0.10	1.27E-01	0.46
576.4746@10.700024	576.4746	10.70	POS	RP				5.23E-02	1.33E-02	-0.67	3.26E-01	0.27	9.72E-01	0.01	2.88E-01	0.33
576.4753@0.42665783	576.4753	0.43	POS	hilic				8.97E-04	1.59E-03	-0.96	1.02E-01	0.40	4.65E-01	0.18	1.50E-01	0.44
576.4755@10.82579	576.4755	10.83	POS	RP				4.87E-04	6.48E-04	-1.07	1.76E-01	0.35	9.11E-01	0.03	1.65E-01	0.41
576.5117@14.389858	576.5117	14.39	POS	RP				1.28E-02	1.35E-01	0.47	3.38E-03	0.76	3.81E-05	1.19	6.40E-02	0.54
577.2092@9.936389	577.2092	9.94	NEG	RP				6.33E-01	4.17E-01	0.25	3.57E-01	-0.26	9.61E-01	0.01	3.26E-01	-0.30
577.2092@9.9671755	577.2092	9.97	NEG	RP				6.35E-03	2.67E-02	0.67	5.34E-02	-0.50	4.65E-02	-0.53	6.22E-01	0.13
577.2563@1.4747859	577.2563	1.47	NEG	hilic				1.08E-01	2.55E-01	0.40	9.49E-02	-0.39	4.39E-01	-0.21	1.00E-01	0.45
577.271@10.237518	577.2710	10.24	POS	RP				1.28E-01	1.41E-01	0.44	6.07E-01	-0.15	4.36E-02	-0.60	9.21E-01	-0.03
577.2816@12.381274	577.2816	12.38	NEG	RP				9.44E-01	6.26E-01	0.14	3.80E-01	0.21	8.61E-01	-0.06	9.71E-01	-0.01
577.293@9.706414	577.2930	9.71	NEG	RP				7.64E-01	2.84E-01	0.29	9.91E-01	0.00	2.78E-01	0.26	7.01E-01	-0.11
577.4092@1.10799	577.4092	1.11	POS	hilic				2.31E-01	3.70E-01	0.29	1.07E-01	-0.41	6.40E-02	-0.46	8.90E-01	-0.05
577.4198@13.819773	577.4198	13.82	POS	RP				5.31E-01	3.45E-01	0.23	1.05E-01	0.45	5.21E-01	-0.19	7.57E-01	0.10
578.1103@8.632	578.1103	8.63	NEG	RP				6.55E-01	8.26E-02	-0.48	5.75E-01	-0.17	9.71E-01	-0.01	2.61E-01	-0.34
578.1432@11.093098	578.1432	11.09	NEG	RP				2.97E-01	6.56E-02	0.52	4.71E-02	0.46	7.01E-01	0.07	8.27E-02	0.44
578.161@0.54005694	578.1610	0.54	NEG	hilic				7.36E-02	2.27E-02	0.77	4.73E-01	0.16	3.79E-01	-0.23	4.46E-01	0.20
578.2848@12.381633	578.2848	12.38	NEG	RP				8.82E-01	4.52E-01	0.22	2.94E-01	0.25	7.62E-01	-0.10	8.65E-01	0.06
578.3804@10.906893	578.3804	10.91	NEG	RP				5.01E-01	2.34E-01	-0.33	2.00E-01	-0.30	2.15E-01	-0.29	5.70E-01	0.15
578.3806@10.917374	578.3806	10.92	NEG	RP				3.34E-01	1.73E-01	-0.37	4.96E-02	-0.44	1.44E-01	-0.34	7.17E-01	0.10
578.4098@10.9511795	578.4098	10.95	POS	RP				4.42E-01	2.30E-01	0.38	8.32E-01	0.05	1.95E-01	-0.29	6.39E-01	0.12
578.4325@0.41690293	578.4325	0.42	NEG	hilic				6.69E-02	4.06E-02	-0.56	8.34E-01	0.05	6.05E-01	0.14	7.80E-02	0.54
578.4885@0.42555743	578.4885	0.43	POS	hilic				1.08E-03	2.76E-03	-0.78	1.65E-02	0.64	7.00E-02	0.43	6.32E-01	0.14
578.4892@0.4210261	578.4892	0.42	NEG	hilic				5.45E-04	2.00E-03	-0.88	1.84E-02	0.61	2.62E-01	0.29	1.31E-01	0.41
578.4897@10.819233	578.4897	10.82	POS	RP				4.81E-03	1.13E-03	-0.79	7.42E-02	0.46	5.50E-01	0.14	2.64E-01	0.32
578.4897@11.031726	578.4897	11.03	POS	RP				1.01E-01	6.78E-02	-0.49	1.08E-01	0.41	6.98E-01	0.10	2.46E-01	0.35
579.225@10.233375	579.2250	10.23	NEG	RP				5.42E-01	8.00E-01	0.08	4.59E-01	-0.22	2.79E-01	-0.32	3.86E-01	0.29
579.3092@1.1731131	579.3092	1.17	NEG	hilic				4.66E-01	3.99E-02	-0.49	8.79E-01	0.04	7.66E-01	0.08	8.04E-01	-0.07
579.3092@9.878733	579.3092	9.88	NEG	RP				3.37E-01	1.88E-01	0.45	5.96E-01	0.14	1.26E-01	0.36	1.80E-02	0.71
579.31@9.935649	579.3100	9.94	NEG	RP				8.55E-03	1.42E-02	0.72	1.14E-03	0.87	1.45E-05	1.12	3.52E-01	0.30
579.401@1.2096665	579.4010	1.21	POS	hilic				4.67E-01	4.98E-01	0.18	3.18E-01	-0.28	1.58E-01	-0.41	9.89E-01	0.00
579.4269@1.0938158	579.4269	1.09	POS	hilic				4.06E-03	4.68E-02	0.56	2.70E-02	-0.48	4.98E-03	-0.61	7.97E-01	-0.07
579.4946@0.4233797	579.4946	0.42	POS	hilic				1.05E-02	5.00E-04	-0.66	1.17E-01	0.39	1.08E-01	0.38	3.34E-01	0.28
579.8757@4.6341085	579.8757	4.63	NEG	hilic				8.81E-01	5.36E-01	0.20	4.68E-01	0.21	5.95E-01	-0.14	7.74E-01	0.09
580.1743@0.55536	580.1743	0.56	NEG	hilic				1.66E-01	3.00E-02	0.53	8.83E-01	-0.02	6.62E-01	-0.08	3.18E-01	0.24
580.2319@11.095757	580.2319	11.10	POS	RP				6.11E-01	2.63E-01	-0.29	7.75E-01	0.08	1.25E-01	-0.37	7.92E-01	-0.07
580.2325@11.21247	580.2325	11.21	POS	RP				6.65E-01	5.07E-01	-0.21	9.57E-01	-0.02	1.14E-01	-0.42	8.20E-01	0.06
580.2325@11.306661	580.2325	11.31	POS	RP				7.31E-01	6.91E-01	-0.11	5.00E-01	-0.19	1.30E-01	-0.36	7.75E-01	0.07
580.2364@0.42589998	580.2364	0.43	POS	hilic				6.03E-01	2.82E-01	-0.31	3.72E-01	-0.24	2.70E-02	-0.51	2.85E-01	-0.28
580.2729@10.13267	580.2729	10.13	POS	RP				6.44E-01	3.58E-01	-0.26	3.32E-01	0.28	5.16E-01	-0.17	9.69E-01	0.01
580.2732@10.082532	580.2732	10.08	POS	RP				4.46E-01	2.47E-02	0.67	1.31E-01	0.49	3.66E-01	0.28	1.25E-01	0.43
580.2732@10.176384	580.2732	10.18	POS	RP				9.99E-01	8.69E-01	0.05	8.84E-01	0.04	9.22E-01	-0.03	9.27E-01	0.03
580.3306@1.1817015	580.3306	1.18	NEG	hilic				5.48E-02	5.79E-02	0.55	3.21E-01	-0.21	1.46E-01	-0.32	2.58E-01	0.32
580.4459@11.491451	580.4459	11.49	NEG	RP				4.53E-04	2.70E-04	-1.12	3.27E-01	0.21	5.39E-01	-0.14	1.75E-01	0.43
580.4469@0.4168673	580.4469	0.42	NEG	hilic				7.46E-02	8.54E-02	-0.50	2.89E-01	0.27	6.69E-01	0.12	6.63E-02	0.58
580.4537@10.708452	580.4537	10.71	NEG	RP				7.29E-01	1.83E-01	0.39	1.45E-01	0.43	2.52E-01	0.28	2.83E-01	0.30
580.5029@0.41903773	580.5029	0.42	POS	hilic				3.26E-01	3.75E-01	-0.16	9.05E-02	0.42	4.92E-01	0.18	1.14E-01	0.38
580.5029@0.4211045	580.5029	0.42	NEG	hilic				1.12E-03	1.53E-02	-0.62	1.76E-03	0.74	6.32E-02	0.50	1.12E-01	0.46
580.5032@11.035302	580.5032	11.04	NEG	RP				1.06E-02	1.32E-02	-0.50	1.64E-02	0.59	7.05E-01	0.10	1.01E-01	0.46
581.1778@10.672083	581.1778	10.67	NEG	RP				4.35E-01	7.70E-01	-0.09	3.17E-02	-0.50	6.50E-02	-0.45	2.48E-01	-0.31
581.1795@10.657466	581.1795	10.66	NEG	RP				6.81E-01	2.79E-01	0.31	8.02E-01	-0.08	6.31E-01	-0.13	3.92E-01	-0.28
581.18@10.955387	581.1800	10.96	NEG	RP				2.26E-04	3.66E-04	0.97	1.36E-02	-0.67	2.76E-01	-0.26	7.94E-01	0.07
581.1801@10.407738	581.1801	10.41	NEG	RP				5.81E-01	5.77E-02	-0.49	7.97E-01	0.07	5.65E-01	-0.15	8.30E-01	-0.07
581.1829@10.450109	581.1829	10.45	NEG	RP				9.79E-01	9.48E-01	0.02	6.49E-01	-0.13	7.98E-01	-0.07	8.03E-01	0.07
581.241@10.489346	581.2410	10.49	NEG	RP				1.65E-03	8.30E-03	0.77	7.96E-01	-0.07	8.42E-02	-0.49	1.13E-02	0.70
581.2766@9.935193	581.2766	9.94	POS	RP				4.26E-03	1.28E-01	-0.33	1.02E-01	0.42	3.16E-02	0.55	3.10E-02	-0.44
581.2875@1.1159142	581.2875	1.12	POS	hilic				6.67E-01	3.96E-01	-0.25	8.30E-01	-0.06	2.52E-01	0.34	9.47E-01	0.02
581.2905@1.146211	581.2905	1.15	POS	hilic				3.00E-02	3.85E-02	-0.55	1.05E-01	0.47	1.77E-01	0.39	3.75E-01	-0.28
581.2927@9.614342	581.2927	9.61	NEG	RP				1.45E-01	1.96E-01	0.39	7.38E-01	-0.09	8.71E-02	0.49	1.91E-02	0.72
581.2939@1.5293225	581.2939	1.53	NEG	hilic				2.03E-01	1.13E-01	0.47	7.24E-01	-0.08	2.70E-01	0.30	2.53E-02	0.63
581.3248@10.162768	581.3248	10.16	NEG	RP				7.65E-01	8.22E-01	0.08	8.63E-01	0.05	9.78E-02	0.43	9.97E-01	0.00
581.3651@10.430064	581.3651	10.43	NEG	RP				9.39E-01	8.90E-01	-0.04	5.86E-01	0.13	5.01E-01	-0.15	8.12E-01	-0.06
581.4645@10.373991	581.4645	10.37	POS	RP				4.51E-01	9.13E-02	0.49	2.09E-01	0.32	3.63E-02	0.61	1.21E-01	0.47
581.5074@0.4189167	581.5074	0.42	NEG	hilic				6.18E-01	8.32E-01	-0.04	2.76E-01	0.25	3.22E-01	0.23	1.37E-01	0.39

Supplementary table 2: Metabolomics analysis results

								ANOVA	Anorexia vs control		overweight vs control		obese vs control		athletes vs control	
Compound	Mass	Retention Time	Ionization mode	column	Compound_ID	MSMS_spectra	ID_level	P-FDR	P	Estimate	P	Estimate	P	Estimate	P	Estimate
581.806@15.281546	581.8060	15.28	NEG	RP				6.85E-01	9.01E-01	-0.05	7.10E-02	-0.47	3.27E-01	-0.26	2.37E-01	-0.35
582.0543@2.4624171	582.0543	2.46	NEG	RP				7.24E-01	9.22E-01	-0.03	1.98E-01	0.36	9.96E-01	0.00	3.87E-01	0.22
582.2072@9.786071	582.2072	9.79	POS	RP				1.89E-01	1.80E-01	0.48	4.86E-01	0.19	6.40E-03	0.82	8.57E-02	0.48
582.246@11.247405	582.2460	11.25	NEG	RP				4.80E-01	1.94E-01	-0.35	9.67E-01	0.01	4.86E-02	-0.46	4.26E-01	-0.20
582.2472@11.222339	582.2472	11.22	POS	RP				5.53E-01	4.24E-01	-0.28	4.01E-01	0.26	2.82E-01	-0.30	8.00E-01	0.07
582.2473@11.250761	582.2473	11.25	POS	RP				3.34E-01	4.61E-01	-0.25	4.61E-01	0.22	7.74E-02	-0.54	9.21E-01	0.03
582.2477@11.104297	582.2477	11.10	POS	RP				8.25E-01	2.18E-01	-0.40	6.23E-01	-0.14	2.61E-01	-0.29	5.58E-01	-0.17
582.2477@8.07732	582.2477	8.08	POS	RP				4.41E-01	1.38E-01	-0.48	9.33E-01	-0.02	5.54E-02	-0.53	3.20E-01	-0.29
582.2486@0.5588704	582.2486	0.56	POS	hilic				3.85E-01	2.41E-01	-0.36	5.14E-01	-0.18	1.07E-02	-0.66	1.95E-01	-0.37
582.249@0.5741351	582.2490	0.57	POS	hilic				6.04E-01	3.45E-01	-0.25	2.69E-01	-0.26	7.01E-02	-0.40	1.15E-01	-0.38
582.325@9.957074	582.3250	9.96	NEG	RP				2.92E-01	3.73E-01	-0.26	2.28E-01	-0.38	6.54E-03	-0.77	6.11E-01	-0.15
582.3283@9.848338	582.3283	9.85	NEG	RP				7.01E-02	5.07E-02	-0.48	6.75E-02	-0.47	2.65E-03	-0.70	9.99E-01	0.00
582.341@1.2833878	582.3410	1.28	NEG	hilic				9.16E-01	5.14E-01	0.19	8.25E-01	0.06	3.78E-01	0.26	9.89E-01	0.00
582.3815@13.246447	582.3815	13.25	POS	RP				2.07E-01	6.00E-01	0.12	3.33E-01	0.28	7.25E-02	-0.48	2.04E-01	-0.39
582.4065@10.450435	582.4065	10.45	POS	RP				8.81E-01	2.44E-01	-0.26	3.76E-01	-0.19	6.32E-01	-0.11	5.70E-01	-0.14
582.5324@12.956764	582.5324	12.96	NEG	RP				9.65E-01	9.02E-01	-0.04	7.26E-01	-0.10	7.28E-01	-0.11	4.12E-01	-0.26
583.175@10.882502	583.1750	10.88	NEG	RP				2.56E-01	1.73E-01	0.37	4.71E-01	-0.20	1.07E-01	-0.42	8.55E-01	0.04
583.245@1.9740909	583.2450	1.97	NEG	hilic				6.99E-01	3.00E-01	-0.31	4.54E-01	0.20	8.57E-01	-0.06	5.62E-01	0.16
583.2557@0.5772386	583.2557	0.58	POS	hilic				2.77E-01	1.24E-01	-0.49	4.56E-01	0.21	1.53E-01	-0.36	3.42E-01	-0.27
583.2587@0.5701363	583.2587	0.57	POS	hilic				6.94E-01	9.52E-01	0.02	9.74E-01	-0.01	9.60E-02	-0.42	6.85E-01	-0.11
583.3471@1.4465826	583.3471	1.45	NEG	hilic				3.48E-02	1.59E-01	0.43	1.47E-01	0.43	2.49E-04	1.11	2.66E-01	0.32
583.3838@10.70642	583.3838	10.71	NEG	RP				1.97E-04	6.25E-04	0.98	5.86E-02	-0.50	1.84E-02	-0.54	9.31E-01	0.02
583.4143@10.8696785	583.4143	10.87	POS	RP				1.96E-02	5.41E-01	-0.17	2.21E-02	-0.58	7.10E-04	-0.88	1.07E-02	-0.69
584.1443@0.40644702	584.1443	0.41	NEG	hilic				6.96E-01	8.02E-01	-0.07	5.38E-01	-0.18	7.84E-02	-0.45	2.52E-01	-0.35
584.2211@1.3529545	584.2211	1.35	POS	hilic				7.36E-01	7.84E-01	0.08	1.36E-01	0.42	3.78E-01	0.23	2.34E-01	0.39
584.2228@10.188266	584.2228	10.19	POS	RP				1.02E-02	1.75E-01	-0.41	1.62E-02	0.74	1.88E-02	0.64	6.47E-01	0.13
584.2233@10.055864	584.2233	10.06	POS	RP				6.95E-01	7.57E-01	0.08	1.32E-01	0.48	3.02E-01	0.34	6.12E-01	0.14
584.2234@1.1527714	584.2234	1.15	POS	hilic				1.95E-01	5.11E-01	-0.19	9.16E-02	0.52	7.79E-02	0.45	8.39E-01	-0.06
584.2237@10.054112	584.2237	10.05	POS	RP				6.32E-01	6.20E-01	0.13	8.72E-02	0.53	2.71E-01	0.37	4.95E-01	0.19
584.2568@11.216768	584.2568	11.22	NEG	RP				4.06E-01	1.48E-01	-0.45	9.30E-01	0.03	4.60E-02	-0.52	4.57E-01	-0.20
584.2611@0.58589995	584.2611	0.59	POS	hilic				7.02E-01	9.86E-01	0.00	6.61E-01	0.12	1.96E-01	-0.29	5.96E-01	0.14
584.2625@6.8002014	584.2625	6.80	NEG	RP				6.36E-01	1.44E-01	-0.40	3.98E-01	-0.23	4.91E-02	-0.45	2.71E-01	-0.28
584.2627@0.42284593	584.2627	0.42	NEG	hilic				7.29E-01	5.24E-01	-0.23	8.87E-01	0.05	2.08E-01	-0.35	6.89E-01	0.12
584.2627@11.213474	584.2627	11.21	POS	RP				6.83E-01	3.30E-01	-0.34	7.88E-01	0.08	1.93E-01	-0.38	8.67E-01	-0.05
584.2631@0.6767587	584.2631	0.68	POS	hilic				8.90E-01	7.86E-01	0.08	3.97E-01	0.24	6.43E-01	-0.12	7.99E-01	0.08
584.2638@1.2528268	584.2638	1.25	POS	hilic				9.52E-01	7.80E-01	-0.07	9.36E-01	0.02	4.36E-01	-0.17	5.02E-01	-0.15
584.2638@5.5132914	584.2638	5.51	POS	RP				9.07E-01	3.94E-01	-0.31	9.30E-01	-0.03	5.16E-01	-0.17	9.86E-01	-0.01
584.264@6.7754126	584.2640	6.78	POS	RP				8.37E-01	2.94E-01	-0.39	8.75E-01	-0.05	3.19E-01	-0.27	7.13E-01	-0.12
584.2653@0.42051262	584.2653	0.42	POS	hilic				7.59E-01	6.23E-01	-0.18	9.38E-01	0.02	2.17E-01	-0.35	6.77E-01	0.13
584.3364@13.820547	584.3364	13.82	POS	RP				3.77E-01	5.47E-01	0.15	4.68E-01	0.20	1.10E-01	-0.43	2.84E-01	-0.33
584.3675@1.0962912	584.3675	1.10	POS	hilic				8.27E-01	8.61E-01	0.06	5.42E-01	-0.17	4.17E-01	0.20	4.73E-01	0.21
584.3679@1.0223262	584.3679	1.02	POS	hilic				6.33E-01	6.90E-01	0.13	7.94E-01	-0.07	2.91E-01	0.28	1.18E-01	0.44
584.3681@1.1067356	584.3681	1.11	POS	hilic				9.51E-01	7.66E-01	0.09	5.35E-01	-0.15	9.57E-01	0.01	7.92E-01	0.07
584.4224@0.40729728	584.4224	0.41	POS	hilic				7.64E-01	6.53E-01	0.11	9.36E-01	-0.02	2.20E-01	-0.25	7.21E-01	0.09
585.131@10.4368925	585.1310	10.44	NEG	RP				7.62E-01	5.55E-01	-0.16	1.98E-01	-0.38	2.82E-01	-0.33	1.99E-01	-0.37
585.1314@10.405583	585.1314	10.41	NEG	RP				5.95E-01	9.59E-02	-0.50	6.14E-01	-0.15	8.49E-01	0.06	2.50E-01	-0.34
585.134@10.961649	585.1340	10.96	NEG	RP				2.48E-01	8.86E-01	0.04	4.14E-02	-0.62	8.73E-01	0.04	1.91E-01	-0.39
585.1342@10.659759	585.1342	10.66	NEG	RP				3.02E-01	3.06E-01	0.29	1.24E-01	-0.49	8.65E-01	0.04	3.87E-01	-0.26
585.2371@9.614856	585.2371	9.61	NEG	RP				3.50E-01	6.18E-02	0.52	9.05E-01	0.03	9.91E-01	0.00	7.41E-02	0.55
585.2639@0.6867242	585.2639	0.69	POS	hilic				9.11E-01	4.04E-01	-0.25	8.10E-01	0.07	5.86E-01	-0.14	7.78E-01	-0.08
585.2985@0.45955116	585.2985	0.46	POS	hilic				8.64E-01	9.88E-01	0.00	5.88E-01	0.17	2.44E-01	0.29	8.01E-01	-0.06
585.2999@6.0673575	585.2999	6.07	POS	RP				5.95E-01	1.67E-01	-0.38	1.24E-01	-0.43	4.91E-01	-0.20	7.65E-02	-0.56
585.3575@10.261088	585.3575	10.26	NEG	RP				7.70E-01	2.74E-01	0.32	1.93E-01	0.31	3.30E-01	0.19	7.31E-01	0.07
585.3637@1.4115494	585.3637	1.41	NEG	hilic				7.96E-02	3.01E-02	0.63	9.18E-01	0.03	4.55E-02	0.64	9.34E-03	0.75
585.3638@1.4114441	585.3638	1.41	NEG	hilic				3.00E-02	3.80E-03	0.78	4.23E-01	0.20	5.58E-03	0.91	1.44E-02	0.76
585.3639@9.208999	585.3639	9.21	NEG	RP				5.19E-01	3.27E-01	0.30	6.30E-01	-0.13	1.90E-01	-0.33	5.47E-01	-0.17
585.4312@11.873043	585.4312	11.87	POS	RP				9.40E-01	3.97E-01	-0.22	4.81E-01	-0.16	9.87E-01	0.00	9.33E-01	-0.02
586.3591@10.517939	586.3591	10.52	NEG	RP				7.64E-01	4.11E-01	0.26	4.36E-01	-0.24	6.42E-01	0.13	9.64E-01	-0.02
586.4258@12.582056	586.4258	12.58	POS	RP				6.02E-01	5.40E-01	0.17	3.20E-01	0.28	5.37E-01	-0.18	4.29E-01	-0.27
586.4266@0.40343526	586.4266	0.40	POS	hilic				3.87E-03	3.44E-04	-0.89	4.54E-01	0.20	8.91E-02	-0.44	2.01E-01	0.36
586.4381@10.754102	586.4381	10.75	NEG	RP				8.23E-01	2.11E-01	-0.27	3.29E-01	-0.20	6.46E-01	-0.10	9.41E-01	-0.02
586.4921@10.783224	586.4921	10.78	NEG	RP				3.74E-03	3.52E-03	-0.78	3.32E-01	0.28	1.67E-01	0.37	8.28E-02	-0.48
587.168@8.257265	587.1680	8.26	POS	RP				9.23E-01	9.33E-01	0.02	4.22E-01	0.24	3.38E-01	0.27	6.86E-01	0.12
587.1687@8.5557575	587.1687	8.56	POS	RP				6.94E-01	3.56E-01	0.27	7.56E-02	0.50	8.49E-01	0.06	5.88E-01	0.16
587.3052@9.931603	587.3052	9.93	POS	RP				9.17E-04	4.31E-03	-0.85	1.75E-01	0.35	2.07E-01	0.36	3.28E-02	-0.60
587.3212@9.706562	587.3212	9.71	NEG	RP				6.34E-01	2.72E-01	0.33	7.59E-01	-0.09	3.19E-01	0.26	5.12E-01	-0.20
587.3221@9.605331	587.3221	9.61	NEG	RP				9.50E-01	9.00E-01	-0.03	4.82E-01	-0.19	7.06E-01	0.10	7.37E-01	-0.12
587.3224@1.2919769	587.3224	1.29	NEG	hilic				3.63E-01	1.15E-01	0.35	4.85E-01	0.17	4.12E-02	0.48	7.69E-01	-0.07
587.3336@1.1699053	587.3336	1.17	POS	hilic				7.98E-01	8.59E-01	0.04	6.80E-01	0.12	3.39E-01	-0.26	7.57E-01	0.08
587.3609@10.3225565	587.3609	10.32	NEG	RP				3.29E-01	1.70E-02	0.75	7.97E-01	0.08	7.26E-01	0.08	5.21E-01	0.21
587.4946@13.245662	587.4946	13.25	POS	RP				7.02E-01	2.65E-01	-0.32	8.59E-01	0.05	8.98E-01	0.03	2.58E-01	-0.36
587.4947@14.373436	587.4947	14.37	POS	RP				1.49E-01	2.56E-01	0.30	3.31E-02	0.60	2.43E-01	-0.31	9.94E-01	0.00
587.7591@15.295152	587.7591	15.30	NEG	RP				4.32E-01	8.50E-02	0.49	8.43E-01	0.04	8.87E-02	0.43	3.05E-01	0.23

Supplementary table 2: Metabolomics analysis results

Compound	Mass	Retention Time	Ionization mode	column	Compound_ID	MSMS_spectra	ID_level	ANOVA	Anorexia vs control		overweight vs control		obese vs control		athletes vs control	
								P-FDR	P	Estimate	P	Estimate	P	Estimate	P	Estimate
587.7936@15.272738	587.7936	15.27	NEG	RP				6.66E-01	5.26E-01	-0.21	3.64E-01	-0.25	5.76E-01	0.17	2.86E-01	-0.31
587.9276@7.4409866	587.9276	7.44	POS	hilic				8.63E-01	9.92E-01	0.00	2.91E-01	-0.25	7.18E-01	0.10	6.11E-01	-0.14
588.1504@0.4060856	588.1504	0.41	NEG	hilic				9.35E-01	9.90E-01	0.00	4.75E-01	-0.21	8.88E-01	-0.03	7.25E-01	0.12
588.2137@6.832898	588.2137	6.83	POS	RP				6.94E-01	2.52E-01	0.29	4.07E-01	0.24	9.39E-01	-0.03	4.20E-01	-0.22
588.2243@0.42672056	588.2243	0.43	POS	hilic				5.14E-01	4.03E-01	-0.19	2.40E-01	0.33	6.27E-01	-0.12	4.25E-01	0.21
588.3839@10.718836	588.3839	10.72	POS	RP				4.24E-01	9.97E-02	0.50	6.80E-01	-0.13	5.69E-01	-0.14	5.10E-01	-0.18
588.4369@0.42670155	588.4369	0.43	POS	hilic				1.36E-02	1.46E-03	-0.67	6.33E-01	0.12	5.58E-01	-0.14	9.47E-02	0.47
588.4375@0.42670003	588.4375	0.43	NEG	hilic				1.15E-01	5.12E-01	-0.14	3.72E-02	-0.52	4.43E-02	-0.48	5.72E-01	0.16
589.2607@2.2109852	589.2607	2.21	NEG	hilic				3.75E-04	7.55E-03	0.82	4.70E-02	-0.40	3.43E-02	-0.42	2.44E-01	0.27
589.315@9.932289	589.3150	9.93	POS	RP				1.23E-02	2.06E-02	-0.67	5.90E-01	0.16	7.95E-02	0.49	7.51E-02	-0.49
589.3153@1.1061618	589.3153	1.11	POS	hilic				9.61E-01	5.25E-01	-0.17	8.91E-01	-0.04	8.21E-01	0.07	6.01E-01	-0.14
589.337@1.2942733	589.3370	1.29	NEG	hilic				8.52E-01	4.63E-01	-0.23	9.83E-01	0.01	4.05E-01	0.23	8.96E-01	0.04
589.3371@9.854981	589.3371	9.85	NEG	RP				9.39E-01	7.31E-01	-0.12	7.85E-01	-0.08	4.74E-01	0.20	9.52E-01	-0.02
589.3372@1.1557676	589.3372	1.16	NEG	hilic				9.64E-01	9.24E-01	-0.03	7.15E-01	-0.11	6.28E-01	0.14	7.49E-01	0.10
589.3375@9.946435	589.3375	9.95	NEG	RP	LysoPC 20:4 (M+FA)	10 eV: 528.3089 (100), 588.3282 (87), 303.2305 (15)	2	6.86E-02	6.88E-01	-0.12	1.14E-01	0.47	5.99E-03	0.81	9.24E-01	0.03
589.4455@0.89992446	589.4455	0.90	POS	hilic				1.73E-01	1.41E-01	0.43	5.71E-01	0.14	6.60E-02	-0.38	7.04E-01	-0.09
589.4466@1.1210176	589.4466	1.12	POS	hilic				3.24E-01	9.14E-01	0.03	1.04E-01	-0.44	2.93E-02	-0.62	1.99E-01	-0.40
589.4741@11.718182	589.4741	11.72	POS	RP				1.81E-01	9.17E-01	-0.03	6.56E-01	0.13	2.52E-03	-0.70	2.68E-01	-0.35
589.7929@15.270072	589.7929	15.27	NEG	RP				6.86E-01	6.04E-01	-0.16	2.61E-01	-0.36	5.32E-01	-0.17	1.14E-01	-0.50
589.9042@4.582406	589.9042	4.58	NEG	hilic				5.67E-01	3.86E-01	0.25	5.29E-01	0.20	2.45E-01	-0.33	4.01E-01	0.20
589.9042@5.4130006	589.9042	5.41	NEG	hilic				5.24E-01	6.33E-02	-0.62	1.46E-01	-0.41	1.44E-01	-0.40	9.53E-02	-0.47
590.1305@0.40650454	590.1305	0.41	NEG	hilic				9.32E-01	8.52E-01	-0.06	7.56E-01	0.08	4.30E-01	0.22	5.37E-01	0.21
590.256@10.18157	590.2560	10.18	POS	RP				4.34E-01	5.02E-01	0.16	5.83E-02	0.58	1.20E-01	0.50	3.03E-01	0.23
590.2797@10.1108055	590.2797	10.11	POS	RP				1.40E-01	3.20E-02	0.63	5.15E-01	0.20	5.56E-01	0.17	3.00E-03	0.82
590.451@10.709981	590.4510	10.71	POS	RP				8.63E-01	3.05E-01	-0.20	9.75E-01	-0.01	8.98E-01	0.03	5.29E-01	0.17
590.4513@10.749233	590.4513	10.75	POS	RP				7.37E-02	4.19E-01	-0.22	9.64E-02	0.45	7.16E-02	0.42	3.02E-02	0.71
590.4536@10.720308	590.4536	10.72	NEG	RP				8.15E-03	2.11E-02	-0.64	4.41E-01	0.21	9.07E-01	-0.03	2.44E-02	0.72
590.454@0.42687157	590.4540	0.43	NEG	hilic				8.83E-03	9.71E-03	-0.80	2.74E-01	0.28	8.58E-01	-0.05	1.17E-01	0.51
590.4552@0.42690447	590.4552	0.43	POS	hilic				4.60E-02	7.38E-03	-0.69	5.98E-01	0.14	9.97E-01	0.00	1.86E-01	0.43
590.5239@11.036236	590.5239	11.04	NEG	RP				2.64E-01	1.13E-02	-0.70	3.46E-01	-0.32	5.66E-01	-0.15	3.23E-02	-0.63
590.9074@4.5693874	590.9074	4.57	NEG	hilic				9.31E-01	4.03E-01	0.28	6.89E-01	0.11	5.76E-01	0.15	9.02E-01	-0.03
591.126@8.785034	591.1260	8.79	NEG	RP				7.99E-01	5.27E-01	0.19	6.33E-01	-0.13	9.49E-01	0.02	3.55E-01	0.31
591.1783@11.037843	591.1783	11.04	NEG	RP				7.42E-01	7.57E-01	0.09	1.16E-01	-0.33	8.44E-01	-0.05	6.55E-01	-0.11
591.2079@10.960496	591.2079	10.96	NEG	RP				3.01E-03	3.64E-04	1.02	2.20E-01	-0.35	5.01E-01	-0.15	8.63E-01	0.05
591.2088@10.656838	591.2088	10.66	NEG	RP				2.98E-01	6.56E-02	0.54	3.68E-01	-0.25	5.02E-01	0.19	2.94E-01	0.30
591.3512@10.035679	591.3512	10.04	NEG	RP				7.68E-01	5.09E-01	0.22	5.20E-01	-0.21	3.73E-01	0.24	8.63E-01	0.05
591.3513@1.1715674	591.3513	1.17	NEG	hilic				4.92E-01	1.51E-01	-0.43	3.86E-02	-0.53	3.05E-01	-0.26	6.75E-01	-0.11
591.3522@1.1720546	591.3522	1.17	NEG	hilic				4.79E-01	3.49E-01	-0.28	5.06E-02	-0.50	2.11E-01	-0.32	9.26E-01	0.02
591.3526@10.145286	591.3526	10.15	NEG	RP				8.98E-01	5.61E-01	0.20	6.53E-01	-0.14	6.28E-01	0.13	8.04E-01	-0.07
591.4357@13.81926	591.4357	13.82	POS	RP				1.97E-01	4.26E-02	0.48	6.96E-02	0.49	3.00E-01	-0.28	8.38E-01	0.06
591.4624@1.1159657	591.4624	1.12	POS	hilic				4.62E-01	6.84E-01	0.13	2.03E-01	-0.37	9.58E-02	-0.46	2.39E-01	-0.38
591.7941@15.270167	591.7941	15.27	NEG	RP				7.83E-01	2.40E-01	-0.35	1.55E-01	-0.44	3.28E-01	-0.25	4.23E-01	-0.23
592.2345@1.3420701	592.2345	1.34	POS	hilic				4.53E-01	3.46E-01	0.28	8.64E-01	0.05	4.95E-02	0.56	9.59E-01	-0.01
592.3556@1.3033334	592.3556	1.30	NEG	hilic				4.92E-01	2.91E-01	0.37	2.37E-01	-0.34	9.06E-01	-0.03	7.87E-01	0.08
592.3963@11.075812	592.3963	11.08	NEG	RP				5.94E-01	2.12E-01	-0.41	4.11E-01	-0.25	2.80E-01	-0.32	6.39E-01	0.15
592.4689@10.7402	592.4689	10.74	NEG	RP				3.63E-02	2.29E-02	-0.57	9.19E-01	0.03	9.31E-01	0.02	7.24E-02	0.60
592.4693@0.42670065	592.4693	0.43	NEG	hilic				5.05E-04	1.79E-03	-0.94	3.24E-02	0.54	4.87E-01	0.17	1.35E-01	0.44
592.4694@10.701928	592.4694	10.70	POS	RP				5.45E-01	1.01E-01	-0.33	8.53E-01	-0.04	9.23E-01	-0.02	3.89E-01	0.26
592.4695@10.703662	592.4695	10.70	NEG	RP				1.36E-02	3.21E-03	-0.84	5.64E-01	0.14	3.02E-01	-0.26	2.46E-01	0.35
592.4706@10.797178	592.4706	10.80	POS	RP				2.68E-03	6.37E-03	-0.75	9.15E-02	0.45	8.96E-01	-0.03	4.75E-02	0.57
592.4712@0.42674327	592.4712	0.43	POS	hilic				4.74E-04	8.41E-04	-1.01	1.28E-01	0.38	3.86E-01	0.20	1.32E-01	0.45
592.4747@11.031402	592.4747	11.03	POS	RP				2.77E-01	4.83E-01	-0.13	8.66E-02	0.42	6.54E-01	0.11	1.09E-01	0.42
592.507@13.53199	592.5070	13.53	POS	RP				9.22E-01	3.17E-01	0.30	5.19E-01	0.16	4.27E-01	0.25	4.15E-01	0.25
593.1532@3.301197	593.1532	3.30	POS	hilic				8.17E-01	4.05E-01	-0.27	9.36E-01	0.02	4.68E-01	-0.19	5.19E-01	0.16
593.1533@9.327683	593.1533	9.33	POS	RP				4.55E-01	5.69E-02	0.70	1.22E-01	0.39	2.68E-01	0.31	5.56E-01	0.18
593.2721@1.9773031	593.2721	1.98	NEG	hilic				7.61E-01	2.91E-01	-0.30	8.86E-01	-0.03	3.87E-01	-0.27	7.17E-01	0.10
593.3304@10.307803	593.3304	10.31	NEG	RP				7.27E-01	1.23E-01	-0.44	4.71E-01	-0.22	1.20E-01	-0.41	6.58E-01	-0.14
593.3577@0.41731113	593.3577	0.42	POS	hilic				7.97E-01	8.50E-01	-0.06	7.52E-01	-0.07	2.70E-01	-0.23	4.94E-01	0.17
593.3595@10.525048	593.3595	10.53	POS	RP				4.29E-01	5.78E-01	0.13	2.92E-01	0.31	5.85E-01	-0.14	2.04E-01	-0.33
593.3653@1.2901428	593.3653	1.29	NEG	hilic				1.64E-02	1.10E-01	0.51	1.30E-01	-0.30	2.75E-02	-0.42	1.86E-01	0.33
593.3677@10.3680935	593.3677	10.37	NEG	RP				1.10E-03	5.43E-03	0.80	8.30E-02	-0.52	1.27E-01	-0.41	1.64E-01	0.38
593.475@1.1002704	593.4750	1.10	POS	hilic				5.24E-01	2.64E-01	-0.33	2.77E-01	-0.28	2.67E-02	-0.57	5.19E-01	-0.17
594.2488@1.3361307	594.2488	1.34	POS	hilic				8.18E-01	7.16E-01	0.13	1.18E-01	0.43	4.97E-01	0.19	5.52E-01	0.16
594.2498@1.1560106	594.2498	1.16	POS	hilic				3.80E-01	1.40E-01	0.44	7.45E-02	0.48	2.28E-02	0.62	5.67E-02	0.55
594.3023@0.42675	594.3023	0.43	POS	hilic				8.41E-01	4.39E-01	0.22	2.90E-01	0.24	1.67E-01	0.37	6.08E-01	0.14
594.4118@11.25033	594.4118	11.25	NEG	RP				9.60E-01	6.20E-01	0.17	4.19E-01	0.20	6.19E-01	0.15	3.37E-01	0.24
594.4198@0.42358336	594.4198	0.42	POS	hilic				2.95E-01	7.25E-03	-0.55	4.24E-01	-0.19	1.55E-01	-0.31	7.93E-01	0.08
594.4855@0.42670065	594.4855	0.43	NEG	hilic				3.93E-04	1.85E-03	-0.92	1.91E-02	0.60	3.49E-01	0.22	1.45E-01	0.42
594.4856@10.833314	594.4856	10.83	NEG	RP				5.12E-04	6.46E-04	-1.05	1.36E-01	0.38	6.59E-01	0.10	2.01E-01	0.38
594.4858@10.791101	594.4858	10.79	POS	RP				3.06E-01	3.47E-03	-0.64	7.11E-01	-0.11	5.38E-01	0.15	3.34E-01	-0.29
594.4859@11.026366	594.4859	11.03	POS	RP				2.37E-03	1.75E-02	-0.60	5.01E-02	0.52	3.87E-01	0.22	1.38E-02	0.68
594.4866@10.806113	594.4866	10.81	POS	RP				1.54E-03	1.43E-03	-0.91	1.01E-01	0.43	5.93E-01	0.13	1.90E-01	0.38
594.4872@0.42674327	594.4872	0.43	POS	hilic				3.86E-04	5.91E-04	-1.06	1.46E-01	0.37	4.40E-01	0.18	1.86E-01	0.39

Supplementary table 2: Metabolomics analysis results

Compound	Mass	Retention Time	Ionization mode	column	Compound_ID	MSMS_spectra	ID_level	ANOVA	Anorexia vs control		overweight vs control		obese vs control		athletes vs control	
								P-FDR	P	Estimate	P	Estimate	P	Estimate	P	Estimate
594.5225@14.382851	594.5225	14.38	POS	RP				1.51E-02	1.80E-01	0.42	9.57E-03	0.69	4.78E-05	1.18	6.48E-02	0.52
595.1583@10.65743	595.1583	10.66	NEG	RP				2.62E-02	2.90E-03	0.92	4.82E-01	-0.18	8.21E-01	0.06	2.38E-01	0.33
595.1605@10.398458	595.1605	10.40	NEG	RP				9.92E-01	7.53E-01	-0.12	9.00E-01	-0.04	7.06E-01	-0.11	9.05E-01	0.03
595.1609@10.9618635	595.1609	10.96	NEG	RP				1.06E-02	1.11E-03	0.95	4.12E-01	-0.24	5.72E-01	-0.16	2.99E-01	0.27
595.1612@10.9596	595.1612	10.96	NEG	RP				6.10E-02	4.66E-03	0.90	8.84E-01	0.04	9.69E-01	-0.01	3.45E-01	0.26
595.2566@10.06101	595.2566	10.06	POS	RP				4.02E-01	4.43E-01	-0.25	2.63E-01	0.31	5.04E-01	-0.18	2.09E-01	0.33
595.2582@10.154474	595.2582	10.15	POS	RP				8.06E-01	8.38E-01	-0.06	2.11E-01	0.34	5.33E-01	0.15	4.70E-01	0.24
595.3099@9.889879	595.3099	9.89	NEG	RP				2.34E-01	2.01E-02	0.72	4.10E-01	0.22	1.78E-02	0.65	5.10E-01	0.19
595.3482@10.381706	595.3482	10.38	NEG	RP				4.82E-01	1.76E-01	-0.37	3.57E-02	-0.51	1.31E-01	-0.37	1.67E-01	-0.39
595.3626@1.1254638	595.3626	1.13	POS	hilic				2.14E-01	3.14E-01	0.32	8.75E-02	-0.47	1.47E-01	-0.43	9.05E-01	-0.04
595.3834@1.2642667	595.3834	1.26	NEG	hilic				8.78E-06	2.36E-03	0.86	3.09E-03	-0.52	5.13E-03	-0.49	7.77E-01	0.06
595.3836@10.595637	595.3836	10.60	NEG	RP				8.96E-05	1.35E-02	0.69	5.92E-04	-0.89	4.44E-03	-0.65	5.19E-01	-0.18
595.3839@10.595637	595.3839	10.60	NEG	RP				8.96E-05	1.35E-02	0.69	5.62E-04	-0.89	4.17E-03	-0.65	5.07E-01	-0.18
595.4023@10.706532	595.4023	10.71	NEG	RP				2.93E-01	3.17E-01	0.29	1.74E-02	0.74	4.18E-02	0.57	2.09E-01	0.40
595.9691@9.339223	595.9691	9.34	NEG	RP				6.93E-01	7.26E-01	0.09	1.89E-01	-0.42	8.81E-01	-0.05	9.29E-01	0.03
596.4058@10.706882	596.4058	10.71	NEG	RP				3.16E-01	3.03E-01	0.30	1.78E-02	0.73	4.58E-02	0.54	2.13E-01	0.38
596.4254@10.95442	596.4254	10.95	POS	RP				8.37E-01	9.08E-01	0.04	4.89E-01	-0.14	2.14E-01	-0.31	5.75E-01	-0.14
596.4399@10.958338	596.4399	10.96	NEG	RP				9.27E-02	7.01E-03	-0.61	6.71E-01	-0.10	3.62E-01	-0.20	3.03E-01	0.34
596.4415@0.42259786	596.4415	0.42	NEG	hilic				1.16E-01	5.50E-04	-0.80	2.72E-01	-0.26	1.55E-01	-0.36	7.62E-01	0.10
596.4416@11.018423	596.4416	11.02	POS	RP				1.53E-01	5.25E-02	-0.37	7.51E-01	0.08	4.82E-01	0.15	1.23E-01	0.45
596.4423@0.42266336	596.4423	0.42	POS	hilic				5.82E-04	1.41E-05	-1.15	5.75E-01	0.12	3.30E-01	-0.25	2.97E-01	0.33
596.4985@10.951963	596.4985	10.95	NEG	RP				4.87E-04	1.00E-03	-0.92	1.48E-02	0.60	1.43E-01	0.33	4.11E-01	0.25
596.5005@11.014522	596.5005	11.01	NEG	RP				2.20E-03	5.85E-02	-0.54	2.60E-03	0.88	1.40E-01	0.39	2.24E-01	0.35
596.5005@11.030551	596.5005	11.03	NEG	RP				8.83E-04	2.40E-02	-0.65	2.11E-03	0.84	1.70E-01	0.35	1.00E-01	0.46
596.5006@0.42661518	596.5006	0.43	NEG	hilic				4.28E-05	1.01E-03	-0.93	3.33E-03	0.77	7.51E-02	0.45	1.53E-01	0.34
596.5007@10.978871	596.5007	10.98	NEG	RP				4.07E-01	4.86E-02	-0.39	6.34E-01	-0.11	9.92E-01	0.00	3.17E-01	0.26
597.257@9.614813	597.2570	9.61	NEG	RP				1.22E-01	2.51E-01	0.33	5.28E-01	-0.18	5.08E-02	0.55	4.17E-02	0.65
597.2589@10.157352	597.2589	10.16	POS	RP				7.35E-01	2.97E-01	-0.29	9.33E-01	-0.02	5.84E-01	0.14	4.87E-01	0.21
597.2599@9.887959	597.2599	9.89	NEG	RP				3.36E-01	3.36E-01	0.22	2.47E-01	0.24	5.06E-01	0.15	2.05E-02	0.63
597.2993@12.2803545	597.2993	12.28	NEG	RP				7.30E-01	8.84E-01	0.04	6.22E-01	0.11	3.02E-01	0.26	3.91E-01	-0.18
597.3079@1.1215659	597.3079	1.12	POS	hilic				5.75E-01	8.42E-01	-0.06	7.18E-01	-0.10	4.32E-02	-0.57	4.67E-01	-0.23
597.3768@1.1252168	597.3768	1.13	POS	hilic				1.53E-02	9.10E-02	0.51	1.56E-02	-0.62	1.25E-01	-0.38	5.56E-01	0.17
597.4001@10.873322	597.4001	10.87	NEG	RP				2.79E-05	1.86E-01	0.38	2.20E-05	-0.95	2.29E-04	-0.81	9.80E-04	-0.86
597.7812@15.27788	597.7812	15.28	NEG	RP				8.35E-01	9.88E-01	0.01	6.31E-01	-0.15	6.99E-01	0.11	2.50E-01	-0.31
598.2278@5.018924	598.2278	5.02	POS	hilic				2.92E-01	1.49E-02	-0.72	9.74E-01	-0.01	8.60E-01	0.05	6.08E-01	-0.17
598.2479@0.5127869	598.2479	0.51	POS	hilic				7.14E-02	1.73E-01	-0.40	9.43E-01	0.02	9.36E-04	-0.77	3.00E-01	-0.26
598.3581@13.254149	598.3581	13.25	POS	RP				4.62E-01	9.14E-01	0.03	2.70E-01	-0.32	1.19E-01	-0.43	6.51E-02	-0.55
598.384@0.6320249	598.3840	0.63	POS	hilic				9.19E-01	7.68E-01	0.09	4.37E-01	-0.21	6.73E-01	-0.12	6.75E-01	-0.12
598.385@3.1074598	598.3850	3.11	POS	RP				9.25E-01	8.46E-01	-0.05	7.31E-01	-0.10	5.71E-01	0.16	6.55E-01	0.13
598.427@10.562019	598.4270	10.56	POS	RP				2.50E-02	1.10E-02	-0.51	2.26E-01	0.33	2.15E-01	0.31	1.61E-01	-0.32
598.4325@10.136846	598.4325	10.14	POS	RP				2.28E-01	4.45E-02	0.63	9.62E-02	0.51	1.15E-01	0.41	1.38E-02	0.79
598.4344@10.013909	598.4344	10.01	POS	RP				4.94E-01	8.03E-02	0.45	7.74E-01	0.09	9.97E-01	0.00	9.93E-02	0.51
598.4391@10.876743	598.4391	10.88	POS	RP				1.43E-01	5.07E-01	0.18	8.30E-02	-0.44	5.26E-02	-0.48	4.45E-01	0.26
598.4408@11.343906	598.4408	11.34	POS	RP				9.26E-04	6.73E-03	0.77	1.67E-01	-0.29	1.36E-02	-0.49	4.05E-01	0.21
598.453@11.026704	598.4530	11.03	POS	RP				9.96E-02	1.97E-01	-0.23	1.70E-02	0.56	2.07E-01	0.27	4.12E-01	0.22
598.4568@0.4225553	598.4568	0.42	NEG	hilic				6.01E-03	4.64E-03	-0.74	2.25E-01	0.32	2.65E-01	0.29	9.87E-02	0.50
598.4954@2.6627016	598.4954	2.66	POS	hilic				7.37E-01	4.06E-01	0.21	1.87E-01	0.34	2.82E-01	0.29	1.15E-01	0.43
598.4966@13.127029	598.4966	13.13	POS	RP				7.94E-01	1.88E-01	-0.28	9.29E-01	0.02	4.50E-01	-0.17	3.68E-01	-0.20
599.2561@9.889361	599.2561	9.89	NEG	RP				3.60E-01	1.97E-02	0.69	4.22E-01	0.22	2.83E-01	0.28	5.89E-02	0.53
599.2562@9.614459	599.2562	9.61	NEG	RP				1.54E-01	6.99E-01	0.12	4.78E-01	0.17	2.16E-02	0.63	2.31E-02	0.72
599.258@1.4784483	599.2580	1.48	NEG	hilic				8.94E-03	6.77E-01	0.13	2.05E-02	-0.67	2.49E-03	-0.80	5.74E-01	0.16
599.2873@10.379752	599.2873	10.38	NEG	RP				5.73E-01	2.32E-01	-0.29	2.75E-01	-0.27	9.16E-02	-0.39	9.18E-01	0.03
599.2929@10.149245	599.2929	10.15	NEG	RP				7.09E-01	6.02E-01	0.12	1.01E-01	0.40	8.11E-01	0.05	5.54E-01	0.13
599.2943@1.4430754	599.2943	1.44	NEG	hilic				2.26E-01	2.46E-01	-0.22	1.85E-01	0.34	7.38E-02	0.45	9.84E-01	-0.01
599.2948@10.147146	599.2948	10.15	NEG	RP				5.31E-01	4.68E-01	-0.17	2.06E-01	-0.31	4.74E-01	0.20	8.31E-01	0.05
599.2955@1.2532451	599.2955	1.25	NEG	hilic				8.89E-01	4.15E-01	-0.21	9.55E-01	-0.01	3.00E-01	-0.25	8.85E-01	-0.03
599.3149@0.41769645	599.3149	0.42	POS	hilic				6.63E-01	1.38E-01	0.44	6.45E-01	0.12	8.78E-02	0.47	5.84E-01	0.16
599.3229@1.0992872	599.3229	1.10	POS	hilic				6.05E-01	3.28E-01	0.30	5.09E-01	-0.19	2.94E-01	-0.28	6.25E-01	-0.14
599.4959@13.2467375	599.4959	13.25	POS	RP				8.91E-01	3.71E-01	-0.24	9.42E-01	-0.02	4.21E-01	-0.22	3.81E-01	-0.30
600.2534@9.615212	600.2534	9.62	NEG	RP				3.96E-01	1.38E-01	0.39	9.57E-01	-0.01	2.93E-01	0.30	5.96E-02	0.60
600.2579@1.8286669	600.2579	1.83	POS	hilic				1.13E-01	3.79E-01	0.28	8.26E-01	-0.06	6.16E-02	-0.49	1.26E-01	0.45
600.2583@0.781827	600.2583	0.78	POS	hilic				3.59E-01	3.89E-01	-0.27	3.08E-01	-0.31	3.51E-03	-0.75	4.47E-01	-0.23
600.2584@5.072584	600.2584	5.07	POS	RP				2.23E-01	8.64E-01	-0.06	8.80E-01	-0.04	8.19E-02	-0.50	1.45E-01	0.41
600.4711@0.42103925	600.4711	0.42	POS	hilic				3.86E-02	6.19E-03	-0.54	2.89E-01	0.27	1.09E-01	0.42	8.94E-01	0.03
600.4721@0.42192864	600.4721	0.42	NEG	hilic				2.55E-02	3.19E-01	-0.18	1.64E-01	0.32	4.28E-03	0.76	7.54E-02	0.43
600.512@13.802752	600.5120	13.80	POS	RP				4.03E-01	7.59E-01	0.08	1.83E-01	0.35	3.60E-02	0.68	6.25E-01	0.17
600.5125@2.782812	600.5125	2.78	POS	hilic				3.20E-01	9.21E-01	-0.03	1.92E-01	-0.32	2.85E-03	-0.65	8.88E-01	-0.04
600.7381@10.483087	600.7381	10.48	POS	RP				8.01E-01	7.87E-01	-0.08	5.56E-01	0.16	3.24E-01	-0.26	7.08E-01	-0.10
601.21@9.96369	601.2100	9.96	NEG	RP				7.95E-01	7.44E-01	-0.09	6.78E-01	0.11	3.15E-01	0.31	7.87E-01	-0.07
601.2106@9.965898	601.2106	9.97	NEG	RP				6.82E-01	3.00E-01	0.32	1.47E-01	0.39	3.60E-01	0.24	9.60E-01	-0.01
601.2871@1.0866048	601.2871	1.09	POS	hilic				8.13E-01	1.79E-01	0.37	6.32E-01	0.10	5.58E-01	0.14	5.41E-01	0.13
601.2916@6.283659	601.2916	6.28	NEG	RP				6.75E-01	4.09E-01	-0.17	2.79E-01	0.30	5.54E-01	0.14	9.02E-01	0.03
601.3255@0.41489133	601.3255	0.41	POS	hilic				3.27E-01	7.44E-02	-0.42	3.15E-01	-0.24	4.32E-02	-0.47	8.03E-01	0.07

Supplementary table 2: Metabolomics analysis results

Compound	Mass	Retention Time	Ionization mode	column	Compound_ID	MSMS_spectra	ID_level	ANOVA P-FDR	Anorexia vs control P	Anorexia vs control Estimate	overweight vs control P	overweight vs control Estimate	obese vs control P	obese vs control Estimate	athletes vs control P	athletes vs control Estimate
601.3404@1.177091	601.3404	1.18	POS	hilic				8.26E-01	4.49E-01	-0.19	6.30E-01	0.14	8.02E-01	0.06	5.18E-01	-0.19
601.7742@15.272954	601.7742	15.27	NEG	RP				7.86E-01	5.95E-01	-0.19	4.97E-01	-0.19	6.50E-01	-0.12	3.42E-02	-0.47
602.166@0.4039914	602.1660	0.40	NEG	hilic				6.50E-01	1.78E-01	0.36	4.78E-01	0.24	5.27E-01	-0.17	3.91E-01	0.27
602.1666@9.259821	602.1666	9.26	NEG	RP				6.23E-01	1.39E-01	-0.47	5.19E-01	0.15	9.17E-01	-0.03	7.52E-01	-0.11
602.1669@9.144251	602.1669	9.14	NEG	RP				2.83E-01	9.74E-03	-0.76	6.59E-01	-0.12	9.95E-01	0.00	4.50E-01	-0.23
602.1763@11.037542	602.1763	11.04	NEG	RP				5.60E-01	1.71E-01	0.37	6.97E-01	-0.07	9.03E-01	0.03	2.70E-01	0.29
602.3747@10.716739	602.3747	10.72	NEG	RP				8.12E-01	2.27E-01	-0.35	5.43E-01	-0.16	2.28E-01	-0.32	4.23E-01	-0.22
602.4182@11.020488	602.4182	11.02	NEG	RP				6.96E-01	9.04E-01	-0.03	6.76E-01	0.11	1.17E-01	-0.32	9.30E-01	-0.02
602.4481@10.711362	602.4481	10.71	NEG	RP				7.36E-02	1.73E-02	-0.64	8.98E-01	-0.04	7.30E-01	0.08	1.51E-02	-0.71
602.456@10.775434	602.4560	10.78	POS	RP				6.68E-01	7.16E-01	-0.09	5.67E-01	0.16	3.94E-01	0.24	3.98E-01	-0.23
602.5277@14.655688	602.5277	14.66	POS	RP				7.35E-02	1.56E-01	0.38	3.40E-03	0.73	3.83E-03	0.90	4.66E-01	0.23
602.5282@2.7841814	602.5282	2.78	POS	hilic				1.98E-01	9.75E-01	-0.01	3.04E-01	-0.27	3.43E-03	-0.62	5.99E-01	0.13
603.3027@1.0785404	603.3027	1.08	POS	hilic				7.53E-01	2.97E-01	0.30	9.86E-01	0.00	3.04E-01	0.27	2.66E-01	0.32
603.3084@1.2741935	603.3084	1.27	NEG	hilic				4.09E-01	1.58E-02	-0.54	8.94E-01	-0.04	8.80E-01	0.04	3.14E-01	-0.24
603.3086@9.933599	603.3086	9.93	NEG	RP				1.18E-01	8.37E-03	-0.62	9.27E-01	0.02	3.83E-01	0.25	2.77E-01	-0.29
603.3401@0.41487283	603.3401	0.41	POS	hilic				4.66E-01	9.36E-01	0.02	1.05E-01	0.41	4.73E-01	-0.16	4.23E-01	0.19
603.4532@0.3961112	603.4532	0.40	POS	hilic				1.99E-01	6.43E-01	0.13	4.22E-02	0.56	2.35E-01	-0.33	8.76E-01	-0.04
603.4536@12.5804405	603.4536	12.58	POS	RP				6.42E-01	6.64E-01	0.12	4.86E-01	0.20	4.31E-01	-0.21	3.41E-01	-0.33
603.4644@10.758425	603.4644	10.76	POS	RP				7.09E-01	2.72E-01	-0.27	2.85E-01	-0.25	7.29E-01	0.09	9.88E-01	0.00
603.769@15.269771	603.7690	15.27	NEG	RP				8.89E-01	3.37E-01	-0.30	5.08E-01	-0.19	8.85E-01	-0.04	2.83E-01	-0.28
603.7759@15.271893	603.7759	15.27	NEG	RP				8.38E-01	8.77E-01	-0.05	3.28E-01	-0.29	5.11E-01	-0.19	1.91E-01	-0.37
604.2262@11.242351	604.2262	11.24	NEG	RP				2.41E-01	7.02E-02	-0.49	6.50E-01	-0.12	6.16E-03	-0.64	1.74E-01	-0.35
604.271@9.953061	604.2710	9.95	POS	RP				3.84E-01	2.05E-01	0.40	3.10E-01	0.30	7.74E-01	-0.08	4.87E-02	0.59
604.2717@9.961832	604.2717	9.96	POS	RP				4.06E-01	6.18E-01	0.17	6.11E-01	0.15	5.46E-01	-0.16	4.63E-02	0.58
604.2718@9.849989	604.2718	9.85	POS	RP				2.57E-02	7.42E-01	-0.10	1.21E-02	-0.72	3.91E-03	-0.82	7.44E-01	0.09
604.3748@10.71455	604.3748	10.71	NEG	RP				6.29E-01	9.64E-01	0.01	6.09E-01	0.16	9.08E-01	-0.03	1.01E-01	0.50
604.4406@0.42700002	604.4406	0.43	NEG	hilic				2.05E-01	4.31E-02	-0.50	7.86E-01	0.07	5.57E-01	-0.14	2.61E-01	0.33
605.1879@10.950579	605.1879	10.95	NEG	RP				8.17E-02	1.36E-02	0.77	8.72E-01	-0.04	3.11E-01	-0.25	4.64E-01	0.22
605.2903@1.1723633	605.2903	1.17	POS	hilic				1.46E-01	5.77E-02	-0.57	5.51E-01	0.16	4.87E-01	0.21	9.45E-02	-0.44
605.3268@10.923236	605.3268	10.92	POS	RP				7.99E-01	6.54E-01	0.10	8.22E-01	-0.05	4.44E-01	0.21	2.73E-01	0.31
605.3282@0.40638295	605.3282	0.41	POS	hilic				5.25E-01	5.43E-01	-0.13	8.18E-01	-0.05	2.30E-01	0.29	2.53E-01	0.30
605.3328@12.27822	605.3328	12.28	NEG	RP				8.35E-01	6.49E-01	0.15	9.83E-01	0.01	5.25E-01	0.17	4.20E-01	-0.24
605.4443@1.0554507	605.4443	1.06	POS	hilic				4.26E-02	1.10E-01	0.54	1.02E-01	-0.42	4.55E-02	-0.49	5.82E-01	-0.15
605.7717@15.271137	605.7717	15.27	NEG	RP				7.36E-01	4.72E-01	-0.22	9.30E-01	-0.03	8.51E-01	0.05	1.48E-01	-0.42
606.2072@9.713806	606.2072	9.71	POS	RP				7.37E-02	2.39E-01	0.34	2.42E-02	-0.43	3.31E-01	-0.20	5.13E-01	0.17
606.2448@0.4215981	606.2448	0.42	NEG	hilic				8.68E-01	6.50E-01	-0.16	7.47E-01	-0.10	1.52E-01	-0.39	7.34E-01	-0.11
606.2902@10.260038	606.2902	10.26	POS	RP				9.65E-01	8.17E-01	0.07	3.86E-01	0.19	9.25E-01	-0.02	6.46E-01	0.13
606.293@0.52142864	606.2930	0.52	POS	hilic				3.17E-01	1.68E-02	-0.46	1.25E-01	-0.32	9.18E-01	0.03	3.16E-01	-0.23
606.3319@0.40642852	606.3319	0.41	POS	hilic				6.93E-01	3.52E-01	-0.18	6.06E-01	-0.09	7.22E-01	0.08	3.61E-01	0.22
606.3854@12.722732	606.3854	12.72	POS	RP				9.70E-01	9.12E-01	-0.03	8.71E-01	0.05	9.66E-01	-0.01	4.12E-01	0.21
606.4122@11.188036	606.4122	11.19	NEG	RP				1.93E-01	9.18E-02	0.50	7.79E-01	-0.09	1.54E-01	-0.43	4.05E-01	0.21
606.452@0.4269764	606.4520	0.43	NEG	hilic				1.15E-01	3.50E-02	-0.52	8.63E-01	0.05	2.99E-01	-0.26	1.70E-01	0.41
606.4553@0.4269564	606.4553	0.43	NEG	hilic				5.34E-02	3.24E-02	-0.55	4.77E-01	0.20	4.07E-01	-0.21	1.18E-01	0.47
606.49@11.029226	606.4900	11.03	POS	RP				8.91E-01	4.68E-01	0.18	4.85E-01	0.23	8.52E-01	-0.06	3.81E-01	0.24
607.3085@9.769429	607.3085	9.77	NEG	RP				9.08E-01	3.42E-01	0.32	3.09E-01	0.28	5.68E-01	0.17	6.05E-01	0.14
607.3089@9.749386	607.3089	9.75	NEG	RP				8.09E-01	8.80E-01	-0.05	3.25E-01	-0.25	9.87E-01	0.01	4.29E-01	0.22
607.31@1.432675	607.3100	1.43	NEG	hilic				9.11E-01	3.87E-01	-0.24	8.19E-01	-0.06	7.61E-01	0.08	8.78E-01	0.04
607.3283@12.277944	607.3283	12.28	NEG	RP				6.33E-01	9.13E-01	0.03	7.14E-01	-0.11	9.91E-01	0.00	1.16E-01	-0.50
607.4557@1.0446881	607.4557	1.04	POS	hilic				3.76E-03	3.70E-02	0.56	3.60E-01	-0.22	8.83E-04	-0.75	9.50E-01	0.02
607.5186@12.870194	607.5186	12.87	POS	RP				1.98E-02	4.03E-01	0.24	9.36E-02	0.43	4.09E-04	1.14	3.57E-01	0.28
608.0067@0.50359523	608.0067	0.50	NEG	hilic				3.11E-01	7.06E-02	0.61	2.42E-01	0.30	6.96E-01	-0.11	6.00E-01	0.13
608.1868@6.024115	608.1868	6.02	POS	hilic				7.46E-01	5.42E-01	0.19	3.64E-01	-0.23	5.50E-01	0.16	7.34E-01	0.09
608.2217@9.846538	608.2217	9.85	POS	RP				1.99E-02	1.90E-01	0.39	7.45E-02	-0.50	8.08E-02	-0.48	1.40E-01	0.42
608.2218@9.873768	608.2218	9.87	POS	RP				3.02E-01	8.08E-01	0.08	1.40E-01	-0.38	1.39E-01	-0.36	3.34E-01	0.29
608.2219@1.1139737	608.2219	1.11	POS	hilic				8.00E-01	1.17E-01	0.43	3.74E-01	0.30	3.15E-01	0.29	2.28E-01	0.32
608.2223@10.041753	608.2223	10.04	POS	RP				4.21E-01	9.60E-01	0.02	3.68E-02	0.62	4.28E-01	0.23	1.63E-01	0.39
608.3021@10.451266	608.3021	10.45	POS	RP				1.05E-02	2.83E-01	0.32	1.14E-03	-0.88	7.00E-02	-0.45	2.71E-01	-0.31
608.3032@10.514192	608.3032	10.51	POS	RP				1.78E-01	2.35E-02	0.69	6.48E-01	-0.13	7.74E-01	-0.08	2.47E-01	0.35
608.3033@10.512593	608.3033	10.51	POS	RP				1.47E-02	1.93E-03	0.83	2.73E-01	-0.30	3.36E-01	-0.27	3.60E-01	0.29
608.3315@12.277561	608.3315	12.28	NEG	RP				6.53E-01	6.29E-01	0.14	6.56E-01	-0.13	9.45E-01	0.02	1.48E-01	-0.42
608.3731@10.432013	608.3731	10.43	NEG	RP				2.00E-03	3.40E-02	-0.45	2.40E-02	0.59	3.10E-02	0.53	3.01E-01	-0.25
608.4087@0.39491832	608.4087	0.39	POS	hilic				7.35E-01	2.54E-01	-0.31	7.82E-01	0.08	2.85E-01	-0.29	3.35E-01	-0.28
608.4211@10.7572975	608.4211	10.76	POS	RP				7.55E-01	3.31E-01	-0.24	4.09E-01	-0.20	6.35E-01	0.12	7.42E-01	-0.08
608.4676@0.42697614	608.4676	0.43	NEG	hilic				1.92E-01	3.77E-02	-0.52	9.17E-01	-0.03	1.92E-01	-0.33	3.10E-01	0.30
609.1773@11.925488	609.1773	11.93	POS	RP				3.69E-01	4.70E-01	-0.21	2.98E-01	0.31	7.97E-01	0.08	1.99E-01	-0.43
609.2583@9.7847805	609.2583	9.78	NEG	RP				9.18E-01	9.64E-01	0.01	2.99E-01	-0.24	9.07E-01	0.03	8.50E-01	-0.05
609.3243@10.007306	609.3243	10.01	NEG	RP				9.75E-02	3.44E-02	0.71	9.67E-01	0.01	2.84E-02	0.70	5.37E-02	0.55
609.3244@10.140144	609.3244	10.14	NEG	RP				8.53E-02	4.67E-01	0.25	1.90E-01	0.37	9.51E-03	0.64	1.46E-03	0.94
609.3246@1.2512308	609.3246	1.25	NEG	hilic				1.40E-02	6.94E-03	0.80	2.53E-01	-0.27	3.16E-01	0.31	1.91E-02	0.70
609.3246@10.141101	609.3246	10.14	NEG	RP				1.98E-01	4.74E-01	0.24	8.82E-02	0.50	3.42E-02	0.60	7.34E-03	0.80
609.3253@1.4431932	609.3253	1.44	NEG	hilic				7.28E-01	9.10E-01	-0.03	3.80E-01	0.24	3.71E-01	-0.25	5.48E-01	-0.15
609.3256@1.4333644	609.3256	1.43	NEG	hilic				7.92E-01	8.07E-01	-0.07	5.17E-01	0.18	4.60E-01	-0.22	4.06E-01	-0.22
609.3613@1.384141	609.3613	1.38	NEG	hilic				3.07E-01	7.48E-01	0.10	3.36E-01	-0.26	1.75E-01	-0.35	1.87E-01	0.41

Supplementary table 2: Metabolomics analysis results

Compound	Mass	Retention Time	Ionization mode	column	Compound_ID	MSMS_spectra	ID_level	ANOVA P-FDR	Anorexia vs control P	Anorexia vs control Estimate	overweight vs control P	overweight vs control Estimate	obese vs control P	obese vs control Estimate	athletes vs control P	athletes vs control Estimate
609.4249@11.102829	609.4249	11.10	POS	RP				6.40E-01	2.26E-01	-0.37	4.18E-01	-0.26	2.34E-01	-0.36	7.37E-01	0.11
609.5335@13.573595	609.5335	13.57	POS	RP				2.42E-01	3.80E-01	0.30	5.78E-01	0.16	7.28E-03	0.76	6.55E-02	0.54
609.5338@13.521248	609.5338	13.52	POS	RP				4.31E-01	9.07E-01	0.04	9.84E-01	-0.01	3.02E-02	0.62	3.17E-01	0.27
609.769@15.271504	609.7690	15.27	NEG	RP				8.62E-01	3.03E-01	-0.33	5.03E-01	-0.20	7.29E-01	-0.10	2.93E-01	-0.30
610.1191@0.40333718	610.1191	0.40	NEG	hilic				3.54E-01	3.86E-01	0.26	4.92E-03	0.67	9.08E-02	0.45	3.67E-01	0.25
610.234@1.1000087	610.2340	1.10	POS	hilic				7.21E-01	4.12E-01	0.23	4.75E-01	-0.23	8.47E-01	-0.06	4.18E-01	0.25
610.2382@10.257284	610.2382	10.26	POS	RP				3.65E-01	3.59E-01	-0.22	1.28E-01	0.43	3.15E-01	0.27	7.87E-01	-0.06
610.2404@10.256488	610.2404	10.26	POS	RP				2.86E-01	3.00E-01	-0.27	1.84E-01	0.37	8.87E-02	0.42	3.05E-01	0.27
610.2478@10.415728	610.2478	10.42	POS	RP				4.28E-01	1.19E-01	0.50	8.54E-01	-0.05	9.17E-02	0.37	2.02E-01	0.33
610.2586@10.561475	610.2586	10.56	POS	RP				9.34E-01	4.41E-01	0.21	8.23E-01	0.06	7.65E-01	-0.07	9.43E-01	-0.02
610.3275@10.14112	610.3275	10.14	NEG	RP				3.18E-01	8.04E-01	0.08	9.89E-01	0.00	1.32E-01	0.45	2.28E-02	0.64
610.3291@1.4330376	610.3291	1.43	NEG	hilic				4.67E-01	3.56E-01	-0.27	3.60E-01	0.28	4.95E-01	0.20	3.07E-01	-0.27
610.3724@10.431839	610.3724	10.43	NEG	RP				9.58E-04	1.35E-02	-0.61	2.07E-02	0.62	2.77E-02	0.55	4.72E-01	-0.19
610.3842@0.5590769	610.3842	0.56	POS	hilic				9.42E-01	4.92E-01	-0.16	3.71E-01	-0.20	8.28E-01	-0.05	7.44E-01	-0.08
610.4793@10.812963	610.4793	10.81	NEG	RP				2.08E-01	1.35E-01	-0.36	1.19E-01	0.45	3.31E-01	0.22	4.18E-01	0.22
610.48@0.42756072	610.4800	0.43	NEG	hilic				3.25E-01	1.36E-01	-0.34	2.47E-01	0.31	3.07E-01	-0.25	7.77E-01	0.08
610.563@13.940609	610.5630	13.94	NEG	RP				9.97E-01	8.22E-01	-0.07	6.64E-01	-0.10	9.59E-01	-0.02	7.80E-01	-0.08
611.2542@9.785246	611.2542	9.79	NEG	RP				5.23E-01	5.08E-01	0.23	3.72E-01	-0.25	1.35E-01	0.39	7.11E-01	0.10
611.2709@10.1478	611.2709	10.15	NEG	RP				9.68E-02	7.43E-01	-0.11	6.46E-02	0.51	1.19E-02	0.69	5.19E-02	0.59
611.2721@10.022608	611.2721	10.02	NEG	RP				9.08E-01	4.59E-01	0.26	9.03E-01	-0.03	7.28E-01	0.10	4.24E-01	0.24
611.2888@10.306661	611.2888	10.31	NEG	RP				1.96E-01	7.10E-02	-0.42	1.48E-01	-0.39	7.03E-04	-0.74	3.73E-01	-0.25
611.3158@1.6436846	611.3158	1.64	POS	hilic				9.94E-01	6.81E-01	-0.12	7.57E-01	-0.08	9.07E-01	-0.04	9.10E-01	0.04
611.3172@4.1951323	611.3172	4.20	POS	RP				9.58E-01	4.64E-01	-0.21	9.58E-01	-0.01	7.82E-01	0.08	9.28E-01	0.03
611.3766@1.3734224	611.3766	1.37	NEG	hilic				2.32E-01	2.41E-02	0.56	2.87E-01	0.20	9.78E-03	0.57	1.25E-01	0.39
611.3998@10.707944	611.3998	10.71	NEG	RP				7.69E-01	7.07E-01	0.10	5.88E-01	0.16	6.41E-02	0.47	6.19E-01	0.16
611.5491@14.3663225	611.5491	14.37	POS	RP				6.94E-03	1.15E-01	0.49	4.35E-03	0.76	1.82E-05	1.26	3.80E-02	0.59
611.7648@15.272208	611.7648	15.27	NEG	RP				1.20E-01	6.42E-02	-0.53	1.10E-01	-0.42	7.38E-01	0.10	2.14E-02	-0.67
612.1446@0.40650016	612.1446	0.41	NEG	hilic				5.87E-01	9.71E-01	0.01	5.47E-02	0.50	8.03E-01	0.06	3.18E-01	0.32
612.176@11.924446	612.1760	11.92	POS	RP				6.68E-01	2.68E-01	-0.30	5.23E-01	0.20	8.47E-01	0.06	4.33E-01	-0.27
612.2485@1.0877383	612.2485	1.09	POS	hilic				9.70E-01	4.52E-01	0.22	9.23E-01	0.03	8.04E-01	0.07	5.43E-01	0.16
612.2494@1.1850443	612.2494	1.19	POS	hilic				4.71E-02	2.00E-01	-0.28	4.20E-02	0.52	1.82E-02	0.59	4.80E-01	0.19
612.252@10.42866	612.2520	10.43	POS	RP				4.41E-01	2.08E-01	0.40	6.04E-01	-0.15	6.77E-01	-0.10	2.16E-01	0.39
612.2533@10.517565	612.2533	10.52	POS	RP				1.22E-01	6.33E-01	0.12	8.81E-01	0.04	8.22E-03	0.73	4.60E-02	0.58
612.2535@10.561831	612.2535	10.56	POS	RP				4.23E-01	7.76E-01	0.10	1.56E-01	0.44	2.04E-02	0.65	5.55E-01	0.18
612.2541@9.783667	612.2541	9.78	NEG	RP				7.02E-01	9.38E-01	-0.02	1.39E-01	0.37	7.16E-01	0.07	4.30E-01	0.17
612.257@11.103657	612.2570	11.10	POS	RP				4.41E-01	3.53E-01	-0.28	9.91E-01	0.00	2.39E-02	-0.54	7.71E-01	-0.07
612.2576@12.001833	612.2576	12.00	POS	RP				2.08E-01	1.59E-01	-0.43	1.75E-02	-0.70	3.95E-02	-0.57	7.94E-01	-0.08
612.2581@11.2132845	612.2581	11.21	POS	RP				6.42E-01	2.16E-01	-0.44	8.76E-01	0.05	2.09E-01	-0.34	8.82E-01	-0.04
612.2585@11.262604	612.2585	11.26	POS	RP				9.03E-01	3.77E-01	-0.26	8.27E-01	-0.06	6.22E-01	-0.13	3.16E-01	-0.28
612.3813@1.3733283	612.3813	1.37	NEG	hilic				4.02E-01	2.05E-01	0.31	9.48E-01	-0.02	4.06E-02	0.55	4.33E-01	0.20
612.4039@10.708523	612.4039	10.71	NEG	RP				1.52E-01	2.68E-01	0.33	1.01E-02	0.83	1.40E-02	0.69	1.05E-01	0.49
612.4284@0.4275469	612.4284	0.43	POS	hilic				1.45E-01	8.00E-03	-0.60	2.96E-01	-0.27	2.31E-02	-0.51	7.41E-01	0.10
612.4325@0.4276875	612.4325	0.43	NEG	hilic				2.12E-02	1.42E-03	-0.70	8.94E-01	0.03	6.14E-01	-0.12	1.12E-01	0.46
612.4972@0.43319568	612.4972	0.43	NEG	hilic				8.80E-02	3.73E-03	-0.59	7.02E-01	-0.10	1.39E-01	-0.33	3.84E-01	0.22
613.2307@9.616088	613.2307	9.62	NEG	RP				5.79E-02	3.95E-02	0.52	9.35E-01	0.02	6.46E-01	0.11	5.83E-03	0.84
613.2555@1.0853913	613.2555	1.09	POS	hilic				7.93E-01	3.02E-01	0.30	3.82E-01	0.21	8.16E-01	-0.05	5.12E-01	0.15
613.2592@10.556927	613.2592	10.56	POS	RP				4.46E-02	1.58E-01	-0.38	1.68E-01	0.40	6.93E-03	0.67	5.87E-01	0.15
613.2605@2.15813	613.2605	2.16	NEG	hilic				4.53E-04	4.16E-02	0.56	9.69E-03	-0.66	3.15E-03	-0.80	4.72E-01	0.21
613.2669@10.008426	613.2669	10.01	NEG	RP				2.98E-01	2.72E-01	0.36	8.55E-01	-0.05	1.35E-01	0.51	4.98E-02	0.59
613.2676@9.889591	613.2676	9.89	NEG	RP				6.18E-01	8.72E-02	0.55	7.90E-01	0.08	2.76E-01	0.32	2.91E-01	0.32
613.2677@10.143785	613.2677	10.14	NEG	RP				1.25E-01	9.62E-01	-0.01	6.54E-02	0.54	1.26E-02	0.71	4.02E-02	0.63
613.3021@10.381046	613.3021	10.38	NEG	RP				7.12E-01	3.14E-01	-0.29	1.05E-01	-0.48	2.46E-01	-0.31	3.71E-01	-0.28
613.3367@9.852357	613.3367	9.85	NEG	RP				5.08E-01	2.91E-02	-0.68	2.81E-01	-0.32	5.35E-01	-0.19	3.09E-01	-0.29
613.3373@9.937655	613.3373	9.94	NEG	RP	LysoPC 22:6 (M+FA)	10 eV: 552.3099 (100), 612.3329 (72), 327.2362 (25)	2	2.08E-01	1.17E-02	-0.78	6.94E-01	-0.11	9.29E-01	0.03	1.97E-01	-0.37
613.338@1.2746413	613.3380	1.27	NEG	hilic				8.34E-01	2.12E-01	-0.38	3.25E-01	-0.29	3.43E-01	-0.29	2.70E-01	-0.35
613.3383@1.1395937	613.3383	1.14	NEG	hilic				9.27E-01	9.66E-01	-0.01	9.25E-01	-0.03	3.38E-01	-0.24	5.07E-01	-0.17
613.3924@1.3701428	613.3924	1.37	NEG	hilic				5.22E-01	5.72E-02	0.40	5.07E-01	0.14	8.60E-02	0.44	1.54E-01	0.33
613.7566@15.271772	613.7566	15.27	NEG	RP				3.88E-01	3.12E-02	-0.55	4.49E-01	-0.23	7.83E-01	0.09	1.01E-01	-0.47
614.1361@0.40642354	614.1361	0.41	NEG	hilic				5.45E-01	1.62E-01	0.43	7.61E-01	0.09	4.86E-01	-0.18	2.65E-01	0.32
614.2392@0.55249983	614.2392	0.55	POS	hilic				7.96E-01	6.78E-01	-0.14	8.65E-01	-0.05	2.07E-01	-0.37	8.12E-01	0.07
614.2403@0.5518424	614.2403	0.55	POS	hilic				9.51E-01	6.44E-01	-0.16	9.99E-01	0.00	7.54E-01	-0.09	6.37E-01	0.14
614.2551@12.003115	614.2551	12.00	POS	RP				6.51E-02	5.97E-01	-0.15	1.47E-02	-0.74	1.56E-02	-0.70	8.84E-01	0.04
614.2691@10.042802	614.2691	10.04	NEG	RP				4.37E-01	1.39E-01	0.44	1.20E-01	0.49	4.82E-02	0.62	5.24E-01	0.20
614.3306@13.24285	614.3306	13.24	POS	RP				6.52E-01	9.03E-01	-0.03	3.75E-01	0.25	2.67E-01	-0.30	5.06E-01	-0.22
614.365@8.503767	614.3650	8.50	NEG	RP				1.48E-01	9.41E-01	0.02	4.83E-02	0.63	6.81E-03	0.68	4.00E-01	0.22
614.3807@11.103698	614.3807	11.10	POS	RP				4.68E-02	1.43E-03	-0.77	9.51E-03	-0.65	1.78E-02	-0.59	4.30E-01	-0.22
614.405@10.707917	614.4050	10.71	NEG	RP				1.72E-01	8.60E-01	0.05	1.21E-01	0.42	6.85E-03	0.74	8.70E-01	-0.05
614.4487@10.776243	614.4487	10.78	NEG	RP				1.48E-01	1.69E-02	-0.51	6.23E-01	0.12	9.79E-01	-0.01	2.56E-01	0.34
614.4516@0.4271148	614.4516	0.43	NEG	hilic				1.32E-04	8.66E-04	-0.96	2.83E-02	0.54	1.14E-01	0.36	8.25E-02	0.52
614.4516@10.750979	614.4516	10.75	POS	RP				4.79E-02	1.29E-01	-0.28	9.37E-01	0.02	2.49E-01	-0.23	4.56E-02	0.57
614.489@13.54954	614.4890	13.55	POS	RP				6.76E-01	8.19E-01	-0.08	6.25E-01	-0.11	2.46E-01	0.32	2.46E-01	0.31
614.4893@13.5644655	614.4893	13.56	POS	RP				4.21E-01	4.31E-01	0.26	7.04E-01	-0.10	5.74E-02	0.52	1.38E-01	0.42
614.5043@10.700606	614.5043	10.70	NEG	RP				4.02E-01	9.03E-02	0.44	5.74E-01	0.10	5.16E-01	-0.11	5.83E-01	0.12

Supplementary table 2: Metabolomics analysis results

Compound	Mass	Retention Time	Ionization mode	column	Compound_ID	MSMS_spectra	ID_level	ANOVA	Anorexia vs control		overweight vs control		obese vs control		athletes vs control	
								P-FDR	P	Estimate	P	Estimate	P	Estimate	P	Estimate
614.5091@0.43142858	614.5091	0.43	NEG	hilic				4.18E-01	4.13E-02	0.50	4.79E-01	0.12	6.06E-01	0.09	3.68E-01	0.18
614.5108@10.738027	614.5108	10.74	POS	RP				5.16E-01	4.20E-02	0.53	4.16E-01	0.16	5.89E-01	0.12	5.29E-01	0.15
614.512@10.706768	614.5120	10.71	POS	RP				1.34E-01	1.88E-02	0.62	6.38E-01	0.08	7.22E-01	-0.06	4.66E-01	0.15
615.2275@9.783252	615.2275	9.78	POS	RP				7.77E-01	8.07E-01	-0.06	4.94E-01	-0.15	4.28E-01	0.23	5.34E-01	0.15
615.275@2.0815945	615.2750	2.08	NEG	hilic				5.86E-04	5.72E-02	0.52	2.40E-02	-0.63	4.11E-03	-0.77	3.14E-01	0.29
615.3311@0.42571428	615.3311	0.43	POS	hilic				1.45E-01	1.22E-01	-0.44	5.99E-02	0.43	6.72E-01	0.10	8.35E-01	-0.05
615.351@1.2723373	615.3510	1.27	NEG	hilic				1.90E-01	3.57E-01	0.31	1.23E-01	-0.41	4.91E-02	-0.47	2.66E-01	-0.32
615.3527@10.07829	615.3527	10.08	NEG	RP				2.57E-01	1.40E-01	0.50	3.87E-01	-0.26	4.64E-01	-0.18	3.10E-01	-0.31
615.3543@10.1767645	615.3543	10.18	NEG	RP				5.96E-01	6.01E-01	-0.18	2.33E-01	-0.34	2.39E-01	-0.34	5.28E-01	0.18
615.4109@10.479499	615.4109	10.48	POS	RP				8.50E-01	6.85E-01	0.13	2.10E-01	0.34	1.72E-01	0.34	7.12E-01	0.12
615.4579@3.51948	615.4579	3.52	NEG	hilic				8.01E-01	5.04E-01	-0.18	1.54E-01	-0.34	9.38E-01	-0.02	5.13E-01	-0.15
615.7442@10.478535	615.7442	10.48	POS	RP				7.10E-01	1.98E-01	-0.35	4.48E-01	-0.20	9.60E-01	-0.01	6.46E-01	0.15
616.146@8.677215	616.1460	8.68	NEG	RP				1.84E-02	9.45E-03	-0.72	5.09E-01	0.17	2.40E-02	0.55	7.58E-01	-0.09
616.1463@8.562572	616.1463	8.56	NEG	RP				2.44E-01	7.00E-01	0.13	1.79E-03	0.79	2.58E-01	0.28	4.11E-01	0.27
616.1469@8.265965	616.1469	8.27	NEG	RP				6.47E-01	2.86E-01	-0.34	6.20E-01	0.12	2.18E-01	0.26	8.66E-01	-0.06
616.147@8.3560295	616.1470	8.36	NEG	RP				9.61E-01	5.49E-01	-0.20	3.63E-01	-0.23	5.60E-01	-0.15	4.50E-01	-0.22
616.1475@0.4064275	616.1475	0.41	NEG	hilic				4.43E-01	5.44E-02	0.57	3.81E-01	0.27	6.69E-01	-0.11	3.01E-01	0.32
616.1824@9.430604	616.1824	9.43	NEG	RP				6.36E-01	1.83E-01	-0.38	5.55E-01	0.17	6.92E-01	-0.11	6.63E-01	0.15
616.4023@10.7089405	616.4023	10.71	NEG	RP				2.50E-01	6.13E-01	0.15	3.83E-02	0.61	1.13E-02	0.63	8.16E-01	0.07
616.4665@0.42693782	616.4665	0.43	NEG	hilic				4.28E-05	5.11E-04	-0.98	1.20E-02	0.64	4.78E-02	0.46	1.20E-01	0.42
616.4668@10.805515	616.4668	10.81	POS	RP				2.57E-03	2.43E-03	-0.83	5.00E-02	0.50	3.21E-01	0.24	2.93E-01	0.32
616.4669@10.834981	616.4669	10.83	NEG	RP				3.36E-04	3.14E-04	-1.09	1.47E-01	0.38	7.39E-01	0.07	2.06E-01	0.36
616.4671@11.019968	616.4671	11.02	POS	RP				3.64E-02	1.43E-02	-0.65	8.65E-02	0.43	4.77E-01	0.19	6.05E-01	0.15
616.4681@0.42864335	616.4681	0.43	POS	hilic				1.37E-04	1.63E-05	-1.19	2.22E-01	0.30	9.70E-01	-0.01	2.12E-01	0.36
616.5045@14.383278	616.5045	14.38	POS	RP				1.47E-02	2.04E-01	0.40	1.00E-02	0.68	3.60E-05	1.18	8.82E-02	0.48
616.5065@13.124152	616.5065	13.12	POS	RP				6.88E-01	6.01E-01	-0.14	3.80E-01	-0.26	3.46E-01	0.30	9.00E-01	-0.04
617.1509@0.40642354	617.1509	0.41	NEG	hilic				4.73E-01	9.22E-02	0.48	6.81E-01	0.13	5.93E-01	-0.14	1.58E-01	0.39
617.2404@10.190051	617.2404	10.19	POS	RP				9.32E-01	9.11E-01	-0.03	4.05E-01	0.26	7.28E-01	0.11	7.45E-01	0.09
617.3281@0.42561176	617.3281	0.43	POS	hilic				2.26E-01	3.56E-02	0.78	2.62E-02	0.51	3.24E-01	0.28	7.65E-01	0.08
617.3674@10.302474	617.3674	10.30	NEG	RP				7.56E-01	5.20E-01	0.22	3.26E-01	-0.30	5.77E-01	-0.15	9.74E-01	-0.01
617.4077@10.716726	617.4077	10.72	POS	RP				2.17E-01	7.16E-01	-0.08	8.64E-01	-0.04	2.31E-02	0.60	7.35E-01	0.08
617.473@10.806308	617.4730	10.81	POS	RP				2.33E-02	1.66E-03	-0.65	9.28E-02	0.43	9.18E-01	-0.02	6.42E-01	0.13
617.7552@15.268807	617.7552	15.27	NEG	RP				7.59E-01	3.95E-01	-0.28	3.15E-01	-0.29	6.29E-01	0.15	7.08E-01	-0.11
618.145@6.0792656	618.1450	6.08	NEG	hilic				5.65E-01	3.83E-01	-0.27	5.29E-01	-0.20	9.80E-02	-0.44	4.03E-02	-0.58
618.2556@1.1203393	618.2556	1.12	POS	hilic				1.54E-01	5.90E-01	0.15	4.83E-02	0.50	2.71E-01	-0.24	7.01E-01	-0.09
618.2853@10.52208	618.2853	10.52	POS	RP				1.43E-02	2.09E-03	0.85	1.55E-01	-0.42	9.90E-01	0.00	5.57E-01	0.16
618.2867@12.990344	618.2867	12.99	POS	RP				1.22E-02	6.62E-02	-0.47	7.53E-01	-0.09	7.09E-03	0.68	7.53E-02	-0.44
618.3121@10.51586	618.3121	10.52	POS	RP				2.37E-03	1.18E-02	0.72	2.31E-02	-0.62	2.64E-01	-0.26	3.74E-01	0.24
618.4311@10.777884	618.4311	10.78	POS	RP				1.92E-01	4.50E-01	-0.21	7.89E-02	0.49	1.22E-01	0.39	6.09E-01	-0.16
618.4745@1.0405514	618.4745	1.04	POS	hilic				4.79E-02	8.17E-01	0.06	3.35E-02	0.52	6.35E-05	0.87	1.97E-01	0.36
618.481@10.931491	618.4810	10.93	NEG	RP				1.68E-03	5.17E-04	-0.93	5.97E-02	0.49	5.23E-01	0.16	6.58E-01	0.12
618.482@0.4268429	618.4820	0.43	NEG	hilic				1.52E-05	7.12E-04	-0.80	1.94E-03	0.78	7.67E-03	0.72	1.12E-01	0.37
618.482@10.927497	618.4820	10.93	NEG	RP				1.45E-04	7.22E-04	-0.92	1.75E-02	0.65	7.78E-02	0.43	5.31E-01	0.16
618.4825@10.991477	618.4825	10.99	POS	RP				2.28E-03	2.69E-03	-0.93	8.47E-02	0.46	5.01E-01	0.17	4.06E-01	0.23
618.4834@11.016873	618.4834	11.02	POS	RP				5.44E-04	1.19E-03	-0.94	3.10E-02	0.55	2.64E-01	0.29	3.54E-01	0.26
618.5224@13.794175	618.5224	13.79	POS	RP				4.94E-01	7.80E-01	0.08	2.81E-01	0.28	5.17E-02	0.63	7.51E-01	0.11
619.2213@1.4479704	619.2213	1.45	POS	hilic				5.56E-01	8.79E-01	-0.05	1.09E-01	-0.45	9.96E-02	-0.48	3.33E-01	-0.30
619.4009@10.714699	619.4009	10.71	POS	RP				3.68E-01	4.87E-01	0.21	1.49E-01	0.45	2.49E-02	0.73	2.34E-01	0.34
619.4859@0.4265511	619.4859	0.43	NEG	hilic				3.71E-03	9.53E-03	-0.39	9.57E-02	0.42	3.59E-03	0.68	7.62E-01	-0.06
619.4867@11.019833	619.4867	11.02	POS	RP				5.13E-03	7.35E-03	-0.72	1.39E-02	0.60	6.72E-01	0.11	2.60E-01	0.30
619.7571@15.268474	619.7571	15.27	NEG	RP				9.00E-01	2.41E-01	-0.33	6.27E-01	-0.13	8.60E-01	-0.05	9.91E-01	0.00
620.1218@8.148859	620.1218	8.15	NEG	RP				7.00E-01	4.91E-01	-0.19	4.10E-01	0.22	8.67E-01	0.05	3.79E-01	-0.23
620.1376@0.4034482	620.1376	0.40	NEG	hilic				9.19E-01	9.46E-01	-0.02	7.87E-01	0.08	3.02E-01	-0.27	8.32E-01	-0.07
620.2653@1.2068771	620.2653	1.21	POS	hilic				1.06E-01	2.22E-01	0.30	7.37E-02	0.55	9.75E-02	0.49	2.16E-01	-0.28
620.2682@1.0906774	620.2682	1.09	POS	hilic				6.53E-01	4.41E-01	0.23	3.67E-01	-0.21	3.70E-01	-0.23	8.77E-01	0.04
620.2954@12.408949	620.2954	12.41	NEG	RP				2.56E-01	6.98E-01	-0.07	7.73E-02	0.44	9.66E-01	0.01	8.86E-02	0.39
620.3035@10.929221	620.3035	10.93	POS	RP				8.17E-02	1.22E-01	0.45	3.34E-01	0.28	1.31E-03	0.95	6.74E-01	0.13
620.3995@10.710262	620.3995	10.71	NEG	RP				6.55E-01	6.99E-01	-0.10	2.42E-01	-0.24	4.60E-01	0.19	7.45E-01	0.07
620.5382@14.645405	620.5382	14.65	POS	RP				7.40E-02	2.14E-01	0.33	4.53E-03	0.71	4.30E-03	0.89	5.37E-01	0.19
621.2997@7.259009	621.2997	7.26	POS	RP				1.55E-01	8.62E-03	0.71	4.49E-03	0.80	3.84E-02	0.64	7.08E-02	0.52
621.3@7.3677354	621.3000	7.37	POS	RP				3.91E-01	2.95E-02	0.70	2.03E-01	0.35	1.12E-01	0.43	6.80E-01	0.12
621.3085@12.277687	621.3085	12.28	NEG	RP				3.96E-01	4.45E-01	0.19	4.74E-02	0.54	7.14E-02	0.44	7.44E-01	0.08
621.4182@10.768597	621.4182	10.77	NEG	RP				3.37E-01	6.77E-01	0.13	8.53E-02	0.49	5.58E-03	0.67	4.24E-01	0.25
621.4337@0.67851716	621.4337	0.68	POS	hilic				4.10E-01	2.77E-01	0.21	7.83E-01	0.05	7.53E-01	-0.05	1.04E-01	0.40
621.7513@15.270277	621.7513	15.27	NEG	RP				8.69E-01	9.67E-01	0.01	5.32E-01	-0.18	3.23E-01	-0.29	3.53E-01	-0.27
622.118@0.4009656	622.1180	0.40	NEG	hilic				8.64E-01	6.39E-01	0.15	6.42E-01	0.12	4.05E-01	-0.22	7.22E-01	-0.12
622.2187@0.42034876	622.2187	0.42	NEG	hilic				4.55E-01	3.57E-01	-0.25	8.37E-01	-0.05	1.44E-02	-0.53	4.80E-01	-0.18
622.2199@0.42805	622.2199	0.43	POS	hilic				4.05E-01	7.13E-01	-0.11	5.69E-01	-0.14	6.84E-03	-0.60	6.73E-01	-0.11
622.2805@1.199644	622.2805	1.20	POS	hilic				7.56E-01	2.42E-01	-0.28	5.47E-01	0.16	9.90E-01	0.00	5.71E-01	-0.13
622.3249@13.245442	622.3249	13.25	POS	RP				3.56E-01	9.77E-01	0.01	4.15E-01	0.23	4.76E-02	-0.52	4.62E-01	-0.25
622.4326@9.958175	622.4326	9.96	POS	RP				1.22E-01	7.01E-01	0.11	1.73E-01	-0.42	3.19E-02	-0.56	3.01E-01	0.30
622.433@1.2299908	622.4330	1.23	POS	hilic				1.00E-01	1.18E-02	0.68	3.85E-01	-0.24	6.95E-01	-0.11	1.67E-01	0.37
622.4333@10.232187	622.4333	10.23	POS	RP				6.12E-02	1.29E-01	0.45	2.50E-01	-0.27	5.95E-02	-0.41	3.95E-01	0.23

Supplementary table 2: Metabolomics analysis results

								ANOVA	Anorexia vs control		overweight vs control		obese vs control		athletes vs control	
Compound	Mass	Retention Time	Ionization mode	column	Compound_ID	MSMS_spectra	ID_level	P-FDR	P	Estimate	P	Estimate	P	Estimate	P	Estimate
622.4427@11.565322	622.4427	11.57	NEG	RP				5.80E-03	2.38E-01	0.32	1.61E-02	-0.40	1.98E-02	-0.39	1.53E-01	0.35
622.5175@0.41740912	622.5175	0.42	POS	hilic				3.87E-01	1.08E-01	-0.32	6.51E-01	0.11	2.00E-01	0.31	5.49E-01	0.15
623.2871@10.433641	623.2871	10.43	POS	RP				6.27E-01	1.03E-01	0.51	6.84E-01	0.11	3.41E-01	0.25	9.33E-01	-0.02
623.2875@10.507659	623.2875	10.51	POS	RP				6.23E-01	8.45E-02	0.56	8.25E-01	0.07	9.36E-01	0.02	7.98E-01	0.08
623.2887@10.511566	623.2887	10.51	POS	RP				2.23E-01	1.76E-02	0.75	9.16E-01	-0.03	8.25E-01	-0.05	5.75E-01	0.16
623.296@1.224886	623.2960	1.22	NEG	hilic				6.90E-01	8.80E-01	0.05	4.04E-01	-0.19	7.19E-01	-0.09	3.27E-01	0.30
623.2963@10.018556	623.2963	10.02	NEG	RP				9.03E-01	8.12E-01	0.07	9.20E-01	0.02	3.65E-01	-0.25	9.62E-01	-0.01
623.3137@0.6710963	623.3137	0.67	POS	hilic				9.70E-01	9.80E-01	0.01	5.68E-01	-0.16	1.00E+00	0.00	5.80E-01	-0.18
623.3403@10.333071	623.3403	10.33	NEG	RP				3.75E-02	8.72E-03	0.79	1.93E-01	-0.38	8.63E-01	0.04	6.20E-01	0.15
623.4242@10.708717	623.4242	10.71	NEG	RP				5.65E-01	2.41E-01	-0.29	5.88E-01	0.14	4.83E-01	0.19	3.20E-01	0.27
623.4401@11.200996	623.4401	11.20	POS	RP				1.39E-01	2.04E-02	0.74	7.52E-01	0.10	4.87E-01	-0.21	1.66E-01	0.37
623.7073@3.2949154	623.7073	3.29	NEG	hilic				2.13E-01	7.51E-01	0.07	6.52E-01	0.08	1.65E-02	0.60	2.04E-01	0.26
623.7487@15.267792	623.7487	15.27	NEG	RP				6.31E-01	8.45E-01	-0.06	7.54E-01	-0.08	1.85E-01	0.36	3.79E-01	-0.23
623.8389@4.48565	623.8389	4.49	POS	hilic				6.14E-01	1.22E-01	0.47	2.95E-01	0.24	6.41E-01	-0.14	6.16E-01	0.16
624.4479@10.513351	624.4479	10.51	POS	RP				2.38E-01	2.11E-01	0.35	3.09E-01	-0.20	2.81E-01	-0.22	2.70E-01	-0.24
624.448@10.233119	624.4480	10.23	POS	RP				5.08E-03	1.43E-02	0.74	1.55E-01	-0.40	4.88E-02	-0.50	4.60E-01	0.21
624.4485@1.2165225	624.4485	1.22	POS	hilic				8.25E-02	4.21E-03	0.90	3.89E-01	0.23	8.73E-01	-0.04	1.11E-01	0.42
625.2108@9.961778	625.2108	9.96	NEG	RP				3.10E-01	3.53E-01	-0.26	2.20E-01	0.36	1.49E-01	0.43	7.03E-01	-0.11
625.2707@9.783403	625.2707	9.78	NEG	RP				6.66E-01	1.65E-01	0.40	4.87E-01	0.16	1.61E-01	0.35	8.00E-01	0.06
625.2897@10.010434	625.2897	10.01	NEG	RP				8.70E-02	6.26E-01	0.16	5.18E-01	0.19	2.67E-03	0.88	8.27E-01	-0.06
625.2923@10.149177	625.2923	10.15	NEG	RP				8.21E-01	1.96E-01	-0.29	7.87E-01	-0.08	2.35E-01	-0.36	4.62E-01	-0.20
625.2945@1.0311555	625.2945	1.03	POS	hilic				3.01E-03	8.77E-01	0.04	8.52E-03	-0.72	5.65E-04	-1.01	1.38E-03	-0.88
625.2951@1.7341535	625.2951	1.73	POS	hilic				5.45E-03	8.32E-01	-0.06	2.80E-03	-0.78	9.05E-04	-0.95	8.47E-04	-0.87
625.2959@4.646104	625.2959	4.65	POS	RP				3.26E-03	9.66E-01	-0.01	3.39E-03	-0.77	4.36E-04	-1.04	1.84E-03	-0.87
625.3068@1.2209177	625.3068	1.22	NEG	hilic				7.59E-01	3.24E-01	0.28	9.87E-01	0.00	5.30E-01	-0.18	5.20E-01	0.20
625.4516@10.232307	625.4516	10.23	POS	RP				2.95E-03	2.21E-02	0.70	9.80E-02	-0.42	2.71E-02	-0.54	5.84E-01	0.15
625.6847@3.034547	625.6847	3.03	POS	hilic				5.47E-01	6.01E-01	0.18	7.10E-01	0.11	4.78E-01	-0.19	1.11E-01	0.47
625.7438@15.267816	625.7438	15.27	NEG	RP				9.77E-01	8.21E-01	-0.07	7.15E-01	0.10	6.92E-01	-0.12	7.77E-01	-0.09
626.271@9.784714	626.2710	9.78	NEG	RP				9.15E-01	5.13E-01	-0.20	4.83E-01	-0.17	3.03E-01	-0.29	4.10E-01	-0.22
626.4634@10.514425	626.4634	10.51	POS	RP				1.93E-01	1.90E-01	0.43	7.72E-02	-0.44	5.93E-01	-0.12	8.43E-01	0.05
626.5265@2.5544412	626.5265	2.55	POS	hilic				7.29E-01	2.21E-01	-0.38	6.21E-01	0.14	7.81E-01	-0.08	5.04E-01	-0.19
627.1902@10.428241	627.1902	10.43	POS	RP				9.41E-01	9.21E-01	0.03	6.84E-01	0.12	3.18E-01	0.25	4.48E-01	0.21
627.2833@10.147219	627.2833	10.15	NEG	RP				4.65E-02	6.66E-02	-0.55	3.21E-01	0.27	3.05E-02	0.61	7.12E-01	0.11
627.286@10.329167	627.2860	10.33	NEG	RP				4.38E-02	2.71E-02	0.61	9.89E-02	-0.38	2.98E-01	-0.29	8.60E-01	-0.04
627.3094@0.41035292	627.3094	0.41	POS	hilic				2.38E-01	5.75E-01	-0.10	2.42E-01	-0.18	4.60E-02	-0.27	3.48E-01	0.23
627.31@2.6223326	627.3100	2.62	POS	hilic				4.79E-01	6.70E-01	-0.09	2.16E-01	-0.23	4.01E-01	0.20	4.26E-01	0.20
627.3238@1.3029753	627.3238	1.30	NEG	hilic				4.15E-01	6.69E-01	-0.11	6.69E-01	0.11	8.66E-02	0.48	5.71E-01	-0.14
627.324@10.447562	627.3240	10.45	NEG	RP				8.34E-01	5.77E-01	0.17	8.31E-01	0.06	6.55E-01	-0.12	3.87E-01	-0.23
627.3242@1.1884522	627.3242	1.19	NEG	hilic				1.38E-01	3.32E-02	0.75	9.28E-01	-0.03	1.56E-01	0.41	7.25E-01	-0.09
627.3248@10.53936	627.3248	10.54	NEG	RP				5.89E-01	8.10E-02	0.49	9.41E-01	-0.02	4.39E-01	0.23	7.50E-01	-0.09
627.5299@2.5550632	627.5299	2.56	POS	hilic				2.21E-01	4.99E-03	-0.70	9.45E-01	-0.02	6.67E-01	-0.11	1.32E-01	-0.38
627.6842@3.0468423	627.6842	3.05	POS	hilic				7.45E-01	6.19E-01	0.18	4.77E-01	0.20	5.62E-01	-0.16	2.24E-01	0.33
627.6844@3.0477295	627.6844	3.05	POS	hilic				8.94E-01	6.16E-01	0.17	5.79E-01	0.16	7.41E-01	-0.09	3.59E-01	0.26
628.1193@9.143994	628.1193	9.14	NEG	RP				9.43E-01	3.41E-01	0.28	9.41E-01	0.02	5.27E-01	0.17	7.76E-01	0.09
628.1193@9.254796	628.1193	9.25	NEG	RP				9.06E-01	2.58E-01	0.29	3.28E-01	0.29	4.95E-01	0.22	3.34E-01	0.28
628.1198@8.9076395	628.1198	8.91	NEG	RP				7.76E-01	1.26E-01	0.44	9.88E-01	0.00	5.60E-01	0.13	4.73E-01	0.22
628.1198@9.494957	628.1198	9.49	NEG	RP				2.37E-01	4.41E-02	0.49	3.87E-02	0.63	6.38E-02	0.64	7.18E-01	0.09
628.2853@10.007307	628.2853	10.01	NEG	RP				3.72E-01	4.12E-01	0.26	5.08E-01	-0.18	8.66E-02	0.53	2.80E-01	0.32
628.286@10.149624	628.2860	10.15	NEG	RP				2.66E-03	2.08E-02	-0.65	1.67E-01	0.37	1.61E-03	0.79	4.43E-01	0.24
628.3442@4.195545	628.3442	4.20	POS	RP				9.39E-01	4.45E-01	-0.23	9.89E-01	0.00	8.53E-01	0.05	7.79E-01	0.10
628.3448@1.648514	628.3448	1.65	POS	hilic				9.84E-01	7.69E-01	-0.09	9.09E-01	-0.03	8.25E-01	-0.07	7.17E-01	0.12
628.3815@8.810408	628.3815	8.81	NEG	RP				5.31E-01	4.09E-01	0.26	7.68E-01	0.09	2.49E-01	0.32	2.24E-01	-0.30
628.3942@0.7430546	628.3942	0.74	POS	hilic				5.89E-01	2.50E-01	0.34	4.19E-01	-0.17	6.71E-01	0.10	4.78E-01	0.17
628.3976@11.201402	628.3976	11.20	POS	RP				8.12E-01	5.53E-01	0.17	7.84E-01	-0.08	3.32E-01	-0.26	5.47E-01	-0.17
628.3982@1.1527876	628.3982	1.15	POS	hilic				9.19E-01	5.74E-01	0.15	5.74E-01	-0.11	9.94E-01	0.00	8.32E-01	-0.05
628.4215@10.866695	628.4215	10.87	NEG	RP				6.38E-01	3.62E-01	0.28	3.97E-01	-0.25	9.73E-01	-0.01	3.90E-01	-0.23
628.423@0.430137	628.4230	0.43	POS	hilic				2.05E-01	2.18E-02	-0.55	6.00E-01	0.15	5.32E-01	0.15	5.48E-01	0.17
628.4788@10.782968	628.4788	10.78	NEG	RP				4.64E-02	3.55E-01	-0.19	1.76E-02	0.64	1.45E-01	0.36	7.23E-01	-0.09
629.2019@0.51227784	629.2019	0.51	POS	hilic				4.43E-01	5.33E-02	-0.49	7.36E-01	-0.09	7.51E-02	-0.44	2.35E-01	-0.30
629.3247@10.542453	629.3247	10.54	NEG	RP				3.82E-01	4.99E-01	-0.20	7.56E-01	-0.09	6.39E-01	-0.11	7.32E-02	0.49
629.4424@10.706847	629.4424	10.71	NEG	RP				3.74E-03	2.55E-02	-0.62	8.78E-02	0.50	2.51E-02	0.51	1.55E-01	-0.40
629.4559@10.87631	629.4559	10.88	POS	RP				1.33E-02	9.10E-01	0.03	3.53E-02	-0.51	4.02E-03	-0.78	2.64E-01	0.34
629.4561@11.34697	629.4561	11.35	POS	RP				1.19E-02	5.01E-01	0.20	9.99E-03	-0.55	6.25E-03	-0.63	4.09E-01	0.23
629.7508@15.26816	629.7508	15.27	NEG	RP				9.84E-01	6.92E-01	-0.09	7.11E-01	0.11	9.44E-01	0.02	9.82E-01	0.01
630.0858@10.475512	630.0858	10.48	POS	RP				7.10E-01	1.52E-01	0.47	1.85E-01	0.43	1.99E-01	0.34	2.35E-01	0.34
630.1612@0.40460685	630.1612	0.40	NEG	hilic				7.34E-01	1.06E-01	0.47	6.46E-01	0.13	4.94E-01	0.17	2.78E-01	0.37
630.2337@0.58600014	630.2337	0.59	POS	hilic				1.25E-01	1.85E-01	-0.37	5.42E-01	-0.15	7.82E-04	-0.82	3.29E-01	-0.29
630.4203@10.474826	630.4203	10.47	POS	RP				5.44E-01	8.06E-01	0.07	3.26E-01	-0.28	2.10E-01	-0.36	1.32E-01	-0.42
630.4408@0.43032694	630.4408	0.43	POS	hilic				1.26E-02	7.94E-03	-0.42	4.07E-02	0.55	6.56E-02	0.41	8.22E-01	0.05
630.4448@10.7073345	630.4448	10.71	NEG	RP				7.31E-01	2.19E-01	-0.29	5.72E-01	0.15	5.61E-01	0.16	9.63E-01	-0.01
631.3091@9.712752	631.3091	9.71	NEG	RP				3.65E-01	2.19E-01	0.37	2.28E-01	-0.28	8.25E-01	0.05	6.55E-01	-0.12
631.3366@0.40658823	631.3366	0.41	POS	hilic				6.91E-01	5.39E-01	0.17	7.55E-01	0.07	3.55E-01	-0.19	3.38E-01	0.26
631.3706@13.2432375	631.3706	13.24	POS	RP				6.16E-01	3.65E-01	0.24	2.92E-01	0.30	4.19E-01	-0.21	6.49E-01	-0.15

Supplementary table 2: Metabolomics analysis results

								ANOVA	Anorexia vs control		overweight vs control		obese vs control		athletes vs control	
Compound	Mass	Retention Time	Ionization mode	column	Compound_ID	MSMS_spectra	ID_level	P-FDR	P	Estimate	P	Estimate	P	Estimate	P	Estimate
631.4008@10.708772	631.4008	10.71	NEG	RP				2.08E-01	4.12E-01	0.27	1.30E-02	0.68	3.34E-02	0.61	8.62E-01	0.05
631.4302@3.3314307	631.4302	3.33	NEG	hilic				5.58E-01	1.50E-01	0.46	1.00E+00	0.00	4.96E-01	0.16	1.41E-01	0.45
631.4733@11.548575	631.4733	11.55	POS	RP				2.15E-01	3.73E-02	-0.63	9.39E-01	-0.02	5.26E-02	-0.58	7.55E-01	-0.09
631.4899@12.582271	631.4899	12.58	POS	RP				6.47E-01	9.02E-01	-0.04	6.57E-01	0.12	2.81E-01	-0.27	1.99E-01	-0.40
632.1437@0.40422434	632.1437	0.40	NEG	hilic				7.10E-01	4.30E-01	0.23	8.13E-01	-0.07	7.88E-01	-0.07	2.20E-01	0.38
632.247@8.178384	632.2470	8.18	NEG	RP				4.91E-01	1.23E-01	-0.54	4.80E-01	-0.20	3.14E-02	-0.58	4.17E-01	-0.25
632.2474@0.7964055	632.2474	0.80	NEG	hilic				3.96E-01	1.83E-01	-0.39	2.90E-01	-0.28	1.58E-02	-0.62	6.42E-01	-0.13
632.2481@8.017048	632.2481	8.02	POS	RP				4.59E-01	5.08E-01	-0.20	3.12E-01	-0.29	1.50E-02	-0.62	7.12E-01	-0.10
632.2481@8.313646	632.2481	8.31	POS	RP				4.07E-01	5.17E-01	-0.19	3.69E-01	-0.24	1.09E-02	-0.65	4.33E-01	-0.21
632.2482@8.168874	632.2482	8.17	POS	RP				2.40E-01	8.19E-01	0.05	1.61E-01	0.37	3.01E-02	0.52	7.33E-01	-0.07
632.2483@0.7595521	632.2483	0.76	POS	hilic				4.58E-01	1.48E-01	-0.51	3.47E-01	-0.25	1.41E-02	-0.65	3.05E-01	-0.33
632.2484@8.168714	632.2484	8.17	POS	RP				6.05E-01	2.58E-01	-0.41	5.11E-01	-0.18	3.32E-02	-0.56	5.76E-01	-0.18
632.2485@0.5231447	632.2485	0.52	POS	hilic				2.84E-01	4.21E-01	-0.24	6.58E-01	-0.11	6.35E-03	-0.69	6.66E-01	-0.12
632.4511@0.43003708	632.4511	0.43	POS	hilic				1.29E-02	5.14E-01	-0.13	7.44E-02	0.42	7.10E-04	0.80	8.20E-01	0.05
632.5127@11.0364685	632.5127	11.04	NEG	RP				3.60E-01	1.34E-01	0.44	2.77E-02	0.64	7.90E-02	0.51	4.20E-02	0.62
633.3241@9.846212	633.3241	9.85	NEG	RP				7.03E-03	9.15E-01	-0.03	1.61E-02	-0.75	4.58E-04	-0.93	7.81E-01	0.08
633.3243@9.956833	633.3243	9.96	NEG	RP				2.16E-02	4.23E-01	-0.21	4.47E-02	-0.63	1.08E-03	-0.95	8.39E-01	0.06
633.3246@1.3535897	633.3246	1.35	NEG	hilic				1.43E-02	3.68E-01	-0.24	5.75E-02	-0.58	1.46E-04	-1.17	1.71E-01	-0.39
633.3253@1.2139742	633.3253	1.21	NEG	hilic				1.58E-02	4.00E-01	0.24	2.83E-02	-0.65	2.54E-03	-0.79	9.88E-01	0.00
633.4123@3.1652696	633.4123	3.17	POS	hilic				8.18E-01	3.55E-01	-0.31	7.21E-01	-0.11	9.71E-01	-0.01	5.79E-01	0.17
633.533@13.119336	633.5330	13.12	POS	RP				7.84E-01	6.44E-01	-0.13	5.75E-01	-0.17	3.57E-01	0.29	9.89E-01	0.00
633.7345@3.2231596	633.7345	3.22	NEG	hilic				6.10E-01	1.13E-01	0.49	9.84E-01	-0.01	4.30E-01	0.23	2.22E-01	0.38
633.7346@3.2233863	633.7346	3.22	NEG	hilic				7.15E-01	3.12E-01	0.34	6.56E-01	0.13	9.22E-01	0.03	1.12E-01	0.45
633.7348@3.2229466	633.7348	3.22	NEG	hilic				7.83E-01	2.14E-01	0.38	4.80E-01	0.19	3.44E-01	0.32	1.42E-01	0.41
633.8687@4.3545566	633.8687	4.35	POS	hilic				7.98E-01	3.58E-01	0.35	3.86E-01	0.23	8.49E-01	-0.05	9.55E-01	-0.02
634.1522@11.2137	634.1522	11.21	POS	RP				7.12E-01	2.83E-01	-0.32	7.53E-01	-0.09	1.07E-01	-0.40	2.27E-01	-0.32
634.1531@8.5094	634.1531	8.51	NEG	RP				9.65E-01	6.41E-01	-0.14	5.31E-01	-0.19	6.86E-01	-0.11	4.04E-01	-0.24
634.1532@0.40494022	634.1532	0.40	NEG	hilic				6.93E-01	6.84E-02	0.50	4.30E-01	0.23	5.09E-01	0.16	2.30E-01	0.41
634.2034@10.706968	634.2034	10.71	NEG	RP				2.66E-01	6.69E-01	0.14	1.19E-02	0.67	2.44E-02	0.56	8.59E-02	0.54
634.4629@0.43023288	634.4629	0.43	POS	hilic				5.12E-02	3.41E-02	-0.40	7.16E-02	0.51	1.06E-01	0.40	9.04E-01	0.03
635.2688@9.903693	635.2688	9.90	NEG	RP				6.04E-01	6.42E-01	-0.13	1.54E-01	-0.44	1.25E-01	-0.45	9.07E-01	-0.04
635.2706@9.952656	635.2706	9.95	NEG	RP				3.73E-02	1.66E-01	-0.39	5.12E-03	-0.84	1.64E-03	-0.87	5.20E-01	-0.19
635.3205@0.47591108	635.3205	0.48	POS	hilic				8.68E-01	6.25E-01	0.13	5.67E-01	-0.15	4.38E-01	-0.19	7.26E-01	-0.09
635.3396@1.1805041	635.3396	1.18	NEG	hilic				2.65E-02	3.79E-02	0.64	8.49E-02	-0.51	1.71E-01	-0.35	9.17E-01	0.03
635.3403@10.236128	635.3403	10.24	NEG	RP				1.44E-02	2.47E-02	0.68	2.15E-01	-0.39	4.52E-02	-0.49	5.25E-01	0.18
635.3405@1.3266919	635.3405	1.33	NEG	hilic				3.50E-02	1.49E-03	0.92	4.29E-01	-0.20	2.03E-01	0.33	6.92E-01	0.11
635.3909@10.707985	635.3909	10.71	NEG	RP				7.76E-01	2.85E-01	0.40	6.49E-01	0.13	2.69E-01	0.30	9.33E-01	-0.02
635.4333@10.814349	635.4333	10.81	NEG	RP				2.05E-02	2.19E-01	-0.32	2.17E-02	0.66	1.13E-02	0.63	6.51E-01	0.13
635.5489@13.785558	635.5489	13.79	POS	RP				4.70E-01	7.55E-01	0.08	1.60E-01	0.36	5.10E-02	0.63	6.11E-01	0.17
635.733@3.232179	635.7330	3.23	NEG	hilic				6.74E-01	1.38E-01	0.49	3.68E-01	0.26	1.80E-01	0.40	1.75E-01	0.44
635.7332@3.232179	635.7332	3.23	NEG	hilic				4.49E-01	6.07E-02	0.62	2.31E-01	0.36	6.86E-02	0.55	1.10E-01	0.50
636.1335@0.39876908	636.1335	0.40	NEG	hilic				5.56E-01	2.56E-01	0.32	1.30E-01	0.47	5.55E-01	0.16	1.04E-01	0.55
636.2977@10.23688	636.2977	10.24	POS	RP				1.17E-02	9.38E-02	-0.50	8.96E-02	0.51	2.23E-02	0.64	5.78E-01	-0.15
636.2987@0.4067714	636.2987	0.41	POS	hilic				7.59E-01	4.52E-01	0.19	8.14E-01	0.04	4.32E-01	0.20	4.24E-01	-0.15
636.5005@0.4358182	636.5005	0.44	POS	hilic				9.59E-02	7.71E-02	-0.34	9.56E-01	0.01	3.63E-01	-0.18	9.13E-02	0.48
636.516@11.038271	636.5160	11.04	NEG	RP				1.21E-01	5.07E-04	0.97	7.10E-02	0.57	8.96E-02	0.47	9.42E-02	0.51
637.2598@2.010743	637.2598	2.01	NEG	hilic				3.78E-01	6.48E-01	0.14	6.16E-01	-0.14	4.97E-02	-0.59	8.64E-01	-0.04
637.2678@9.93082	637.2678	9.93	NEG	RP				5.19E-01	6.92E-01	-0.13	4.51E-01	-0.23	2.18E-01	-0.35	3.03E-01	0.30
637.2678@9.958007	637.2678	9.96	NEG	RP				2.34E-02	6.71E-01	0.11	1.89E-01	-0.36	5.71E-03	-0.78	2.56E-01	0.34
637.2864@10.238457	637.2864	10.24	NEG	RP				1.88E-02	6.19E-02	0.60	4.00E-01	-0.23	1.02E-02	-0.65	8.37E-01	0.05
637.3313@0.41305748	637.3313	0.41	POS	hilic				8.25E-01	9.21E-02	-0.42	6.54E-01	-0.11	7.93E-01	-0.08	6.51E-01	-0.12
637.3536@10.705215	637.3536	10.71	NEG	RP				7.35E-01	1.80E-01	0.39	1.50E-01	0.38	1.44E-01	0.40	4.76E-01	0.22
637.3553@1.1675541	637.3553	1.17	NEG	hilic				1.31E-01	1.40E-01	0.50	7.36E-02	-0.47	5.03E-01	-0.17	6.10E-01	0.15
637.3555@10.50587	637.3555	10.51	NEG	RP				1.89E-01	4.11E-02	0.56	2.43E-01	-0.37	8.47E-01	0.05	3.97E-01	0.23
637.3556@10.438759	637.3556	10.44	NEG	RP				3.59E-01	4.17E-01	0.21	7.59E-01	-0.10	8.90E-02	0.52	4.36E-01	-0.21
637.3561@1.3076154	637.3561	1.31	NEG	hilic				2.13E-01	1.31E-01	0.43	9.16E-01	-0.03	2.10E-02	0.72	7.54E-01	0.09
637.5645@14.622815	637.5645	14.62	POS	RP				8.36E-02	1.80E-01	0.36	3.36E-03	0.73	4.05E-03	0.89	4.21E-01	0.26
637.8332@4.6906757	637.8332	4.69	NEG	hilic				7.06E-01	9.89E-01	0.00	2.88E-01	0.31	9.94E-01	0.00	1.34E-01	0.40
638.148@9.256257	638.1480	9.26	NEG	RP				5.24E-01	2.17E-01	-0.38	2.31E-01	0.29	7.48E-01	0.08	6.83E-01	-0.13
638.1483@9.144165	638.1483	9.14	NEG	RP				5.49E-01	4.27E-02	-0.60	9.78E-01	-0.01	6.51E-01	-0.13	8.32E-01	-0.07
638.1484@8.908502	638.1484	8.91	NEG	RP				8.41E-01	2.26E-01	-0.39	6.47E-01	-0.12	8.86E-01	-0.04	9.45E-01	0.02
638.1486@9.4990425	638.1486	9.50	NEG	RP				7.33E-01	3.19E-01	-0.27	2.47E-01	0.27	7.28E-01	0.11	7.27E-01	0.12
638.1491@0.40140146	638.1491	0.40	NEG	hilic				5.23E-01	4.01E-02	0.55	5.85E-01	0.17	7.71E-01	-0.08	3.38E-01	0.30
638.1839@0.4189106	638.1839	0.42	POS	hilic				9.16E-01	9.67E-01	-0.01	9.43E-01	-0.02	2.81E-01	-0.28	8.57E-01	0.06
638.2698@9.9456	638.2698	9.95	NEG	RP				6.29E-01	7.27E-01	-0.10	9.25E-02	-0.35	1.38E-01	-0.32	8.68E-01	0.05
638.3574@1.2970222	638.3574	1.30	NEG	hilic				3.96E-02	5.44E-03	0.73	3.59E-01	-0.16	6.74E-01	0.11	8.22E-01	-0.05
638.522@0.4352135	638.5220	0.44	POS	hilic				1.38E-03	5.45E-04	-0.84	5.84E-02	0.48	6.17E-01	0.13	1.91E-01	0.39
639.2461@9.858571	639.2461	9.86	POS	RP				3.97E-01	5.91E-01	0.17	1.68E-01	-0.31	8.24E-02	-0.38	8.96E-01	0.03
639.2835@10.240089	639.2835	10.24	NEG	RP				5.93E-01	8.40E-01	-0.07	1.65E-01	-0.35	2.65E-01	-0.29	5.73E-01	0.15
639.3105@1.3565338	639.3105	1.36	POS	hilic				5.19E-02	1.82E-02	-0.56	9.41E-01	0.02	9.92E-01	0.00	1.11E-02	-0.84
639.311@6.997118	639.3110	7.00	POS	RP				1.89E-01	8.94E-03	0.84	2.50E-02	0.58	5.84E-02	0.56	3.10E-01	0.30
639.3115@0.42570907	639.3115	0.43	POS	hilic				3.88E-02	3.06E-03	0.99	5.13E-02	0.46	1.56E-01	0.40	8.72E-01	-0.05
639.3186@10.50855	639.3186	10.51	NEG	RP				5.29E-01	7.50E-01	0.09	9.91E-02	-0.39	9.27E-01	-0.02	5.34E-01	0.17

Supplementary table 2: Metabolomics analysis results

								ANOVA	Anorexia vs control		overweight vs control		obese vs control		athletes vs control	
Compound	Mass	Retention Time	Ionization mode	column	Compound_ID	MSMS_spectra	ID_level	P-FDR	P	Estimate	P	Estimate	P	Estimate	P	Estimate
639.3591@13.247238	639.3591	13.25	POS	RP				9.40E-01	3.82E-01	-0.26	6.98E-01	-0.11	5.44E-01	-0.16	3.81E-01	-0.27
639.3903@10.430359	639.3903	10.43	NEG	RP				6.38E-03	2.96E-02	-0.42	3.67E-02	0.55	7.16E-02	0.43	3.56E-01	-0.21
640.1153@8.785613	640.1153	8.79	NEG	RP				9.57E-01	9.17E-01	-0.03	5.19E-01	0.21	8.78E-01	-0.05	8.03E-01	0.08
640.2857@10.255858	640.2857	10.26	NEG	RP				5.18E-01	2.99E-01	-0.28	2.58E-01	0.32	5.82E-01	-0.12	8.82E-01	0.04
640.315@0.42593014	640.3150	0.43	POS	hilic				5.44E-01	1.19E-01	0.56	2.35E-01	0.30	6.96E-01	0.11	8.92E-01	-0.04
640.4153@10.780029	640.4153	10.78	POS	RP				4.11E-02	4.80E-01	-0.19	1.61E-01	0.40	1.34E-03	0.84	4.34E-01	0.23
640.4327@0.572	640.4327	0.57	POS	hilic				7.80E-01	1.29E-01	-0.31	3.11E-01	-0.21	5.26E-01	-0.15	3.76E-01	-0.20
640.5042@13.793928	640.5042	13.79	POS	RP				4.49E-01	8.05E-01	0.07	2.76E-01	0.28	4.41E-02	0.65	7.68E-01	0.10
640.5411@0.4275053	640.5411	0.43	POS	hilic				6.73E-03	9.65E-02	-0.39	4.52E-03	0.75	4.76E-02	0.55	1.41E-01	0.40
641.2037@10.815926	641.2037	10.82	POS	RP				3.33E-01	3.89E-01	0.28	4.58E-02	0.59	5.77E-02	0.56	3.92E-02	0.64
641.2391@9.859623	641.2391	9.86	POS	RP				5.92E-02	3.08E-01	0.32	2.03E-02	-0.69	1.31E-01	-0.45	9.16E-01	-0.03
641.2621@10.021839	641.2621	10.02	NEG	RP				6.05E-01	6.84E-01	-0.11	2.08E-01	0.39	2.19E-01	0.33	4.04E-01	0.28
641.2631@10.140765	641.2631	10.14	NEG	RP				6.38E-01	1.22E-01	0.48	1.47E-01	0.47	2.69E-01	0.30	1.45E-01	0.41
641.2641@10.1533	641.2641	10.15	NEG	RP				8.92E-01	5.43E-01	0.18	2.67E-01	0.36	2.61E-01	0.25	5.48E-01	0.20
641.2959@10.343931	641.2959	10.34	NEG	RP				2.30E-01	3.56E-03	0.80	9.22E-01	0.03	4.80E-01	0.22	7.08E-01	0.11
641.299@10.513334	641.2990	10.51	NEG	RP				1.23E-01	2.72E-03	0.88	7.27E-01	0.10	3.29E-01	0.25	4.86E-02	0.57
641.2991@10.408411	641.2991	10.41	NEG	RP				4.79E-01	4.25E-02	0.62	2.57E-01	0.37	8.99E-01	0.04	5.85E-01	0.15
641.3547@13.245929	641.3547	13.25	POS	RP				8.34E-01	3.08E-01	-0.27	9.67E-01	-0.01	5.04E-01	-0.16	2.73E-01	-0.36
641.4062@10.566488	641.4062	10.57	NEG	RP				2.13E-01	5.90E-02	-0.45	1.89E-01	0.37	5.11E-01	0.17	8.07E-01	0.07
641.4281@10.707613	641.4281	10.71	NEG	RP				1.88E-01	4.77E-01	0.19	1.30E-02	0.78	6.37E-02	0.54	4.91E-01	0.21
642.1231@9.17527	642.1231	9.18	NEG	RP				8.89E-01	4.36E-01	-0.20	5.83E-01	-0.14	8.18E-01	0.07	4.12E-01	-0.24
642.1233@8.785359	642.1233	8.79	NEG	RP				6.24E-01	9.75E-02	-0.52	4.60E-01	-0.22	5.04E-01	-0.18	1.26E-01	-0.48
642.1244@9.058834	642.1244	9.06	NEG	RP				4.52E-01	6.67E-01	0.11	5.58E-02	0.48	7.58E-01	-0.08	2.77E-01	0.29
642.1263@0.3967778	642.1263	0.40	NEG	hilic				6.26E-01	8.46E-01	-0.05	1.41E-01	-0.34	7.80E-02	-0.40	6.90E-01	-0.10
642.4344@10.866651	642.4344	10.87	NEG	RP				2.04E-01	1.20E-01	-0.35	8.28E-02	-0.39	2.37E-01	-0.28	4.20E-01	0.22
642.52@14.64619	642.5200	14.65	POS	RP				6.64E-02	2.46E-01	0.31	4.02E-03	0.72	3.43E-03	0.90	5.32E-01	0.20
642.5216@13.638435	642.5216	13.64	POS	RP				4.83E-02	8.24E-01	0.05	7.54E-03	0.69	1.47E-03	0.90	1.98E-01	0.46
643.1273@8.783971	643.1273	8.78	NEG	RP				1.38E-01	5.42E-01	-0.15	7.20E-02	-0.41	2.91E-01	0.29	2.26E-01	0.34
643.3388@0.48544434	643.3388	0.49	POS	hilic				8.41E-01	4.92E-01	-0.22	3.99E-01	0.22	8.28E-01	0.05	8.06E-01	0.07
643.4015@10.566025	643.4015	10.57	NEG	RP				4.57E-01	1.13E-01	-0.36	2.82E-01	0.30	7.14E-01	0.10	9.58E-01	0.01
643.4225@10.707302	643.4225	10.71	NEG	RP				1.99E-01	8.03E-01	0.07	2.22E-02	0.67	1.87E-02	0.65	5.35E-01	0.20
644.2516@9.614455	644.2516	9.61	NEG	RP				6.99E-02	4.88E-01	0.20	5.94E-01	-0.12	6.32E-02	0.49	9.70E-03	0.72
644.2658@0.4261363	644.2658	0.43	POS	hilic				4.57E-01	5.48E-02	0.54	1.39E-01	0.37	8.92E-02	0.45	5.32E-01	0.15
644.2819@11.112999	644.2819	11.11	NEG	RP				2.36E-01	3.49E-01	-0.27	6.80E-01	-0.11	3.17E-03	-0.70	4.83E-01	-0.18
644.2826@11.212621	644.2826	11.21	POS	RP				5.45E-01	2.94E-01	-0.38	5.76E-01	0.17	1.79E-01	-0.36	9.06E-01	-0.04
644.2829@11.223826	644.2829	11.22	NEG	RP				5.95E-01	2.16E-01	-0.46	8.58E-01	0.06	1.86E-01	-0.36	8.41E-01	-0.06
644.2836@11.261853	644.2836	11.26	POS	RP				4.03E-01	2.88E-01	-0.27	3.88E-01	0.24	1.44E-01	-0.37	7.62E-01	-0.08
644.2838@11.099681	644.2838	11.10	POS	RP				5.36E-01	3.19E-01	-0.34	8.08E-01	0.07	9.42E-02	-0.45	9.77E-01	0.01
644.4096@10.567027	644.4096	10.57	NEG	RP				3.34E-01	6.81E-02	-0.43	3.04E-01	0.29	5.35E-01	0.16	9.56E-01	0.02
644.4261@10.705968	644.4261	10.71	NEG	RP				7.80E-01	2.78E-01	0.24	1.62E-01	0.37	8.68E-01	0.05	5.26E-01	0.16
644.4475@11.022852	644.4475	11.02	POS	RP				1.02E-01	7.60E-02	0.52	9.61E-02	-0.40	3.20E-01	-0.26	8.98E-01	0.04
644.4897@0.98421526	644.4897	0.98	POS	hilic				2.13E-01	7.45E-01	0.11	1.26E-02	0.62	8.84E-03	0.70	5.33E-01	0.20
645.2712@10.529026	645.2712	10.53	POS	RP				9.18E-01	7.99E-01	-0.08	7.43E-01	0.10	2.41E-01	0.26	6.23E-01	0.14
645.2716@10.548197	645.2716	10.55	POS	RP				9.15E-01	5.10E-01	0.18	3.83E-01	0.28	8.27E-01	0.06	3.63E-01	0.26
645.3498@13.243634	645.3498	13.24	POS	RP				8.00E-02	1.16E-02	0.65	1.54E-02	0.76	8.32E-01	-0.05	1.90E-01	0.41
645.4381@10.783559	645.4381	10.78	NEG	RP				1.53E-02	1.71E-01	-0.41	1.87E-02	0.70	2.50E-02	0.58	8.03E-01	0.07
645.9661@9.7480345	645.9661	9.75	NEG	RP				8.87E-01	9.26E-01	0.04	4.96E-01	-0.21	7.27E-01	0.10	5.63E-01	0.16
646.09@6.0262036	646.0900	6.03	POS	hilic				3.99E-02	3.15E-01	-0.29	1.38E-01	-0.40	2.55E-04	-1.03	4.41E-01	-0.22
646.2275@0.5509363	646.2275	0.55	POS	hilic				2.16E-01	2.88E-01	-0.35	4.31E-01	-0.20	2.78E-03	-0.82	5.72E-01	-0.17
646.452@0.403846	646.4520	0.40	POS	hilic				2.88E-01	5.71E-03	0.82	4.61E-01	0.20	4.89E-01	0.22	5.33E-01	0.20
646.4531@11.557721	646.4531	11.56	POS	RP				9.12E-01	1.27E-01	-0.35	8.37E-01	-0.06	8.00E-01	-0.07	8.72E-01	-0.05
646.504@11.23439	646.5040	11.23	POS	RP				7.97E-01	5.37E-01	-0.16	9.39E-01	0.02	4.83E-01	0.17	3.51E-01	-0.24
646.5054@0.9837263	646.5054	0.98	POS	hilic				2.24E-01	7.14E-01	0.12	1.09E-02	0.63	9.62E-03	0.69	5.13E-01	0.21
647.2946@9.933104	647.2946	9.93	NEG	RP				7.24E-01	8.19E-01	-0.06	3.14E-01	-0.23	2.83E-01	0.25	9.67E-01	-0.01
647.2967@1.283721	647.2967	1.28	NEG	hilic				4.21E-02	9.50E-03	-0.66	7.79E-01	0.07	3.00E-01	0.32	3.03E-01	0.26
647.4515@0.67030007	647.4515	0.67	POS	hilic				5.67E-01	2.06E-01	0.31	4.48E-01	0.15	4.65E-01	-0.14	3.65E-01	0.24
647.5083@0.98377186	647.5083	0.98	POS	hilic				4.42E-01	7.88E-01	0.09	2.23E-02	0.52	4.57E-02	0.57	4.50E-01	0.22
647.8622@5.4186735	647.8622	5.42	NEG	hilic				3.11E-01	1.73E-01	-0.31	4.32E-01	-0.18	2.97E-02	-0.44	5.41E-01	0.17
647.8625@4.6285577	647.8625	4.63	NEG	hilic				9.19E-01	7.68E-01	0.09	5.72E-01	0.16	8.47E-01	-0.06	5.43E-01	-0.18
648.2404@0.59195083	648.2404	0.59	POS	hilic				4.95E-01	5.58E-01	-0.17	1.37E-01	-0.37	2.12E-02	-0.54	4.65E-01	-0.20
648.4373@10.783466	648.4373	10.78	NEG	RP				3.10E-02	2.38E-01	-0.34	2.92E-02	0.67	2.41E-02	0.59	7.18E-01	0.10
649.2897@9.851786	649.2897	9.85	NEG	RP				3.66E-01	7.02E-01	0.10	6.77E-02	-0.56	2.49E-01	-0.38	9.14E-01	-0.03
649.2907@9.957864	649.2907	9.96	NEG	RP				5.82E-01	1.48E-01	-0.41	1.72E-01	-0.44	8.46E-02	-0.51	6.28E-01	-0.13
649.4319@10.4077215	649.4319	10.41	POS	RP				4.15E-01	2.11E-01	0.34	8.01E-01	-0.05	1.27E-01	-0.24	9.00E-01	0.03
649.4334@0.71266663	649.4334	0.71	POS	hilic				9.11E-01	4.39E-01	-0.19	7.95E-01	0.06	5.31E-01	-0.13	8.00E-01	-0.06
649.4688@0.6704406	649.4688	0.67	POS	hilic				1.28E-01	6.40E-03	0.68	8.81E-02	0.39	5.28E-01	0.14	3.06E-02	0.60
649.4698@11.037399	649.4698	11.04	NEG	RP				4.79E-01	1.28E-01	0.48	4.00E-02	0.59	6.71E-02	0.49	1.20E-01	0.48
649.7893@15.267851	649.7893	15.27	NEG	RP				1.08E-01	2.90E-01	0.30	4.43E-01	-0.19	3.69E-01	-0.20	1.48E-02	-0.52
650.1468@0.40587175	650.1468	0.41	NEG	hilic				3.89E-01	8.28E-01	0.05	7.57E-01	-0.06	9.56E-01	-0.01	8.39E-02	0.48
650.4364@10.417477	650.4364	10.42	POS	RP				6.90E-01	1.93E-01	0.45	5.16E-01	0.18	7.77E-01	-0.08	9.21E-01	0.03
651.1808@8.698133	651.1808	8.70	POS	RP				8.66E-01	3.33E-01	0.31	9.87E-01	-0.01	6.92E-01	-0.12	9.74E-01	0.01
651.1812@8.43	651.1812	8.43	POS	RP				2.28E-01	1.47E-01	0.47	1.31E-02	0.70	9.79E-01	-0.01	1.32E-01	0.38
651.2831@9.9347105	651.2831	9.93	NEG	RP				3.77E-01	6.96E-01	-0.10	6.23E-02	-0.58	1.12E-01	-0.50	8.94E-01	-0.04

Supplementary table 2: Metabolomics analysis results

								ANOVA	Anorexia vs control		overweight vs control		obese vs control		athletes vs control	
Compound	Mass	Retention Time	Ionization mode	column	Compound_ID	MSMS_spectra	ID_level	P-FDR	P	Estimate	P	Estimate	P	Estimate	P	Estimate
651.3046@10.236971	651.3046	10.24	NEG	RP				1.25E-01	1.01E-01	0.52	1.87E-01	-0.38	1.94E-01	-0.36	9.04E-01	0.04
651.3351@1.4346544	651.3351	1.43	NEG	hilic				5.37E-01	2.18E-01	-0.29	5.07E-01	-0.16	3.30E-01	0.25	4.98E-01	-0.18
651.4@10.708555	651.4000	10.71	NEG	RP				7.64E-01	7.12E-01	-0.09	4.39E-01	0.20	2.57E-01	0.29	9.91E-01	0.00
652.1627@0.40545222	652.1627	0.41	NEG	hilic				2.77E-01	4.36E-02	0.50	2.23E-01	0.35	4.13E-01	-0.22	1.65E-01	0.43
652.285@9.877058	652.2850	9.88	NEG	RP				1.13E-01	5.09E-02	0.59	2.57E-01	-0.27	5.27E-01	-0.20	2.03E-01	0.39
652.2857@9.960956	652.2857	9.96	NEG	RP				2.25E-02	1.49E-01	-0.38	2.43E-02	-0.74	6.87E-04	-1.06	4.29E-01	-0.23
652.4336@10.818622	652.4336	10.82	NEG	RP				5.27E-01	5.13E-01	-0.11	3.31E-01	0.24	2.16E-01	0.29	1.40E-01	0.33
652.4464@10.8711815	652.4464	10.87	POS	RP				3.33E-03	3.65E-04	0.83	7.57E-01	-0.06	4.88E-01	-0.14	7.42E-01	-0.08
652.4682@11.037325	652.4682	11.04	NEG	RP				4.15E-01	7.70E-02	0.52	4.95E-02	0.60	6.30E-02	0.55	8.00E-02	0.52
653.2987@10.237691	653.2987	10.24	NEG	RP				3.22E-02	1.11E-01	0.48	1.23E-01	-0.52	2.36E-02	-0.59	8.61E-01	-0.05
653.3123@10.434719	653.3123	10.43	NEG	RP				7.27E-01	2.49E-01	0.33	8.94E-01	-0.04	3.29E-01	-0.24	9.64E-01	0.01
653.3221@10.514563	653.3221	10.51	NEG	RP				8.96E-02	2.96E-02	0.62	2.07E-01	-0.38	7.63E-01	-0.08	3.19E-01	0.30
653.3246@10.236622	653.3246	10.24	POS	RP				6.22E-03	8.22E-02	-0.51	9.12E-02	0.51	9.02E-03	0.74	7.79E-01	-0.08
653.3267@0.4064928	653.3267	0.41	POS	hilic				6.63E-01	3.39E-01	0.25	8.65E-01	-0.04	3.59E-01	0.26	4.48E-01	-0.17
653.3501@1.4341725	653.3501	1.43	NEG	hilic				3.38E-01	3.41E-01	-0.30	3.04E-01	0.26	1.47E-01	0.42	5.92E-01	-0.17
653.4072@10.709271	653.4072	10.71	NEG	RP				8.09E-01	9.76E-01	0.01	9.63E-01	0.01	2.12E-01	-0.35	6.07E-01	-0.17
653.4808@11.029541	653.4808	11.03	POS	RP				4.38E-01	5.93E-02	0.63	8.54E-01	0.06	1.84E-01	0.44	3.79E-01	0.26
654.1457@0.406174	654.1457	0.41	NEG	hilic				5.87E-01	5.60E-01	0.16	3.53E-01	0.25	3.52E-01	-0.26	3.14E-01	0.32
654.3013@10.236792	654.3013	10.24	NEG	RP				7.86E-02	7.70E-01	0.09	4.94E-02	-0.54	5.11E-03	-0.71	6.74E-01	-0.11
654.3074@13.690916	654.3074	13.69	NEG	RP				2.78E-01	6.40E-01	0.10	2.43E-02	0.57	7.01E-01	0.07	2.23E-01	0.25
654.3122@0.48920757	654.3122	0.49	POS	hilic				4.45E-01	4.60E-01	0.20	1.71E-01	-0.32	4.02E-01	0.25	5.03E-01	0.18
654.3155@0.48782125	654.3155	0.49	POS	hilic				3.54E-01	5.03E-01	0.16	7.90E-01	0.07	1.91E-02	0.66	4.31E-01	0.22
654.4098@10.708309	654.4098	10.71	NEG	RP				8.83E-01	3.80E-01	0.21	4.72E-01	0.22	8.05E-01	-0.06	5.16E-01	0.20
654.4365@10.818002	654.4365	10.82	NEG	RP				9.66E-02	4.82E-01	-0.16	1.41E-02	0.70	1.59E-01	0.37	6.51E-01	0.12
654.4772@10.781531	654.4772	10.78	NEG	RP				4.65E-02	1.40E-02	-0.60	5.90E-01	0.15	5.86E-01	0.16	3.12E-02	-0.53
655.1384@8.907828	655.1384	8.91	NEG	RP				9.79E-01	8.91E-01	-0.04	6.94E-01	-0.13	4.55E-01	-0.22	7.40E-01	-0.10
655.1388@9.145299	655.1388	9.15	NEG	RP				6.04E-01	3.50E-01	-0.29	7.16E-02	-0.50	1.24E-01	-0.48	1.57E-01	-0.42
655.1392@9.235265	655.1392	9.24	NEG	RP				5.61E-01	2.83E-01	-0.31	1.56E-01	-0.41	5.99E-02	-0.52	1.29E-01	-0.44
655.3051@1.9164888	655.3051	1.92	POS	hilic				5.64E-01	7.84E-01	0.07	5.44E-01	-0.15	6.89E-01	-0.10	4.79E-02	-0.43
655.3097@9.706014	655.3097	9.71	NEG	RP				7.50E-01	1.39E-01	0.36	8.12E-01	0.06	7.77E-01	0.08	6.70E-01	-0.11
655.3143@10.41183	655.3143	10.41	NEG	RP				3.60E-01	5.37E-01	0.19	8.11E-02	-0.58	4.95E-01	-0.18	8.36E-01	-0.06
655.3145@10.514564	655.3145	10.51	NEG	RP				1.52E-01	6.28E-02	0.54	2.03E-01	-0.40	6.89E-01	-0.11	4.38E-01	0.24
655.4436@0.40758333	655.4436	0.41	POS	hilic				3.00E-01	4.55E-02	-0.33	4.55E-02	-0.33	2.14E-01	-0.23	1.81E-01	-0.24
656.3165@10.433486	656.3165	10.43	NEG	RP				4.10E-01	4.26E-01	-0.22	4.88E-02	-0.65	9.66E-02	-0.49	4.29E-01	-0.20
656.3167@10.515667	656.3167	10.52	NEG	RP				1.82E-01	7.96E-02	0.47	1.55E-01	-0.43	6.74E-01	-0.12	5.01E-01	0.22
656.4294@10.224024	656.4294	10.22	NEG	RP				7.72E-01	4.40E-01	-0.19	4.76E-01	-0.16	5.06E-01	0.16	9.30E-01	-0.02
656.4789@13.791185	656.4789	13.79	POS	RP				3.84E-01	7.18E-01	0.10	1.75E-01	0.37	4.05E-02	0.68	7.46E-01	0.11
657.3245@1.2899228	657.3245	1.29	NEG	hilic				2.22E-03	8.84E-03	-0.77	9.10E-02	0.43	3.97E-02	0.57	4.83E-01	-0.22
657.325@1.1536549	657.3250	1.15	NEG	hilic				9.23E-01	4.69E-01	-0.22	4.93E-01	-0.21	8.55E-01	0.05	9.68E-01	-0.01
657.3252@9.883582	657.3252	9.88	NEG	RP				9.46E-01	7.49E-01	-0.11	4.66E-01	-0.19	9.43E-01	-0.02	7.37E-01	0.10
657.3252@9.927113	657.3252	9.93	NEG	RP				5.17E-01	4.63E-01	0.21	7.37E-01	-0.09	8.04E-02	0.54	6.98E-01	0.11
657.4018@10.708011	657.4018	10.71	NEG	RP				3.89E-01	5.55E-01	0.18	3.84E-02	0.67	9.79E-02	0.46	1.43E-01	0.45
657.5337@12.994911	657.5337	12.99	POS	RP				2.40E-01	1.88E-01	-0.35	5.68E-01	0.15	6.08E-02	0.56	9.55E-01	-0.02
657.8907@5.4124317	657.8907	5.41	NEG	hilic				3.69E-01	4.93E-01	-0.24	4.28E-01	0.22	2.91E-01	-0.31	1.70E-01	0.36
657.8915@4.57755	657.8915	4.58	NEG	hilic				8.09E-01	8.98E-01	0.04	3.44E-01	0.30	9.83E-01	0.01	2.40E-01	0.34
658.0868@8.785792	658.0868	8.79	NEG	RP				2.21E-01	2.46E-02	-0.77	4.05E-02	-0.57	4.73E-02	-0.49	1.20E-01	-0.40
658.1486@0.40542033	658.1486	0.41	NEG	hilic				8.61E-01	9.18E-01	-0.03	3.89E-01	-0.24	4.39E-01	-0.18	6.93E-01	0.12
658.2296@9.616758	658.2296	9.62	NEG	RP				3.98E-01	1.95E-01	0.31	8.82E-01	0.03	1.72E-01	0.32	3.62E-02	0.47
658.2682@9.890265	658.2682	9.89	NEG	RP				3.02E-01	4.17E-02	0.48	1.95E-01	0.30	7.84E-02	0.48	1.52E-02	0.64
658.387@11.491936	658.3870	11.49	NEG	RP				1.72E-02	1.73E-02	-0.62	9.24E-02	0.36	4.87E-01	-0.17	1.52E-01	0.46
658.44@11.059805	658.4400	11.06	NEG	RP				2.71E-01	7.90E-01	-0.07	7.36E-01	0.09	5.53E-01	0.17	2.90E-02	-0.57
658.5042@0.95211506	658.5042	0.95	POS	hilic				8.73E-01	6.35E-01	0.16	2.69E-01	0.29	2.48E-01	0.33	8.38E-01	0.06
658.5118@11.035907	658.5118	11.04	NEG	RP				3.27E-01	1.81E-02	-0.65	2.32E-01	-0.43	6.11E-01	-0.13	3.09E-02	-0.59
658.895@4.570645	658.8950	4.57	NEG	hilic				9.76E-01	4.96E-01	0.20	6.80E-01	0.12	9.56E-01	-0.02	9.25E-01	0.03
659.3391@10.160961	659.3391	10.16	NEG	RP				4.53E-01	8.02E-01	-0.09	7.16E-01	0.10	4.98E-02	0.47	4.00E-01	-0.23
659.3599@0.42390695	659.3599	0.42	POS	hilic				3.25E-01	1.21E-01	0.32	9.75E-03	0.60	3.36E-01	0.21	1.43E-01	0.36
659.3653@9.31144	659.3653	9.31	NEG	RP				2.44E-01	6.32E-01	-0.09	9.40E-01	0.02	4.92E-01	-0.14	5.26E-02	0.44
659.3668@9.316555	659.3668	9.32	POS	RP				2.91E-02	1.06E-01	-0.38	2.90E-01	-0.27	1.23E-02	-0.59	1.20E-01	0.36
659.5486@13.63302	659.5486	13.63	POS	RP				8.58E-02	9.37E-01	-0.02	1.91E-02	0.58	3.66E-03	0.84	2.84E-01	0.38
659.553@11.058814	659.5530	11.06	POS	RP				4.67E-01	7.07E-01	-0.12	9.58E-02	0.48	4.52E-01	0.23	6.99E-01	-0.12
660.0815@8.7859745	660.0815	8.79	NEG	RP				5.44E-01	3.73E-01	-0.32	2.26E-01	-0.35	2.42E-01	-0.29	3.32E-02	-0.64
660.0823@9.176888	660.0823	9.18	NEG	RP				3.11E-01	6.43E-02	-0.65	5.14E-01	-0.17	1.45E-02	-0.57	8.06E-02	-0.55
660.1508@0.40460864	660.1508	0.40	NEG	hilic				9.32E-01	4.89E-01	-0.21	7.53E-01	0.08	9.34E-01	0.02	7.70E-01	0.09
660.2177@10.783061	660.2177	10.78	NEG	RP				1.01E-01	5.56E-01	-0.13	1.06E-02	0.70	7.62E-01	0.07	3.89E-01	0.23
660.3834@11.075125	660.3834	11.08	NEG	RP				3.59E-01	3.44E-02	-0.53	9.22E-02	-0.43	3.06E-02	-0.52	5.44E-01	-0.18
660.4038@10.567808	660.4038	10.57	NEG	RP				5.49E-02	4.83E-02	-0.60	2.13E-01	0.38	1.31E-01	0.43	8.89E-01	0.04
660.4375@11.052922	660.4375	11.05	NEG	RP				6.51E-01	6.34E-02	-0.40	1.20E-01	-0.44	8.25E-01	-0.07	4.87E-01	-0.22
660.5205@0.9516725	660.5205	0.95	POS	hilic				7.29E-01	3.32E-01	0.32	2.54E-01	0.31	1.40E-01	0.39	9.95E-01	0.00
661.2396@9.61485	661.2396	9.61	NEG	RP				4.62E-01	4.52E-01	0.19	9.93E-01	0.00	3.35E-02	0.51	7.50E-01	0.08
661.2598@1.9727417	661.2598	1.97	NEG	hilic				6.51E-01	6.96E-02	-0.50	9.20E-01	-0.03	7.31E-01	-0.11	9.39E-01	-0.02
661.2667@9.937786	661.2667	9.94	NEG	RP				3.01E-03	8.89E-01	-0.04	4.93E-03	0.75	6.49E-04	0.91	7.51E-01	-0.08
661.3588@10.369861	661.3588	10.37	NEG	RP				5.18E-02	1.49E-01	0.40	5.58E-02	-0.37	1.43E-01	-0.29	6.12E-01	0.13
661.5238@0.95257324	661.5238	0.95	POS	hilic				6.67E-01	3.94E-01	0.27	1.25E-01	0.37	1.71E-01	0.36	9.55E-01	0.01

Supplementary table 2: Metabolomics analysis results

Compound	Mass	Retention Time	Ionization mode	column	Compound_ID	MSMS_spectra	ID_level	ANOVA	Anorexia vs control		overweight vs control		obese vs control		athletes vs control	
								P-FDR	P	Estimate	P	Estimate	P	Estimate	P	Estimate
662.2339@11.036477	662.2339	11.04	NEG	RP				5.22E-01	2.01E-01	0.40	1.03E-01	0.50	2.71E-02	0.53	1.01E-01	0.52
662.4043@10.568277	662.4043	10.57	NEG	RP				2.39E-02	1.19E-02	-0.76	3.60E-01	0.26	1.56E-01	0.40	8.30E-01	-0.06
662.4464@10.585058	662.4464	10.59	POS	hilic				3.46E-01	1.23E-02	0.77	5.54E-01	0.18	6.02E-01	0.15	4.84E-01	0.18
662.4472@12.722874	662.4472	12.72	POS	RP				4.92E-01	8.98E-01	0.04	2.58E-01	0.33	1.97E-01	-0.35	6.25E-01	-0.14
662.4474@0.3960257	662.4474	0.40	POS	hilic				2.24E-01	2.42E-01	-0.36	8.00E-01	-0.06	7.40E-02	-0.54	2.08E-02	-0.72
663.4314@10.783568	663.4314	10.78	NEG	RP				1.32E-01	1.82E-01	-0.33	1.65E-01	0.41	6.08E-02	0.47	8.69E-01	-0.05
663.5803@13.787866	663.5803	13.79	POS	RP				3.89E-01	3.50E-01	0.25	1.33E-01	0.38	2.91E-02	0.73	5.06E-01	0.23
663.7643@15.259081	663.7643	15.26	NEG	RP				7.56E-01	8.26E-01	0.06	2.85E-01	-0.23	6.49E-01	0.12	4.41E-01	-0.19
664.2379@0.5507265	664.2379	0.55	POS	hilic				5.11E-01	2.48E-01	-0.40	5.27E-01	-0.17	1.94E-02	-0.63	4.88E-01	-0.22
664.2383@0.5508174	664.2383	0.55	POS	hilic				2.33E-01	1.45E-01	-0.51	4.81E-01	-0.18	4.51E-03	-0.78	5.05E-01	-0.20
664.2383@7.237051	664.2383	7.24	POS	RP				5.03E-01	3.44E-01	-0.31	4.00E-01	-0.24	1.58E-02	-0.66	6.26E-01	-0.16
664.2387@8.1755705	664.2387	8.18	POS	RP				5.27E-01	2.52E-01	-0.40	4.41E-01	-0.22	1.67E-02	-0.63	5.35E-01	-0.20
664.4026@10.567613	664.4026	10.57	NEG	RP				3.21E-02	1.64E-02	-0.73	2.59E-01	0.33	2.38E-01	0.32	8.02E-01	-0.07
664.4157@10.701028	664.4157	10.70	NEG	RP				2.51E-01	6.57E-01	-0.12	1.31E-01	0.45	8.17E-02	0.47	6.30E-01	-0.14
664.4253@0.41852507	664.4253	0.42	NEG	hilic				5.03E-02	1.09E-01	-0.26	4.93E-01	0.14	3.08E-01	0.20	3.37E-02	0.61
664.435@10.7829685	664.4350	10.78	NEG	RP				1.86E-02	1.33E-01	-0.46	3.72E-02	0.61	2.67E-02	0.59	9.44E-01	0.02
664.4774@10.885353	664.4774	10.89	NEG	RP				5.22E-01	4.08E-01	-0.19	6.74E-01	0.10	1.27E-01	0.39	7.30E-01	-0.09
665.1823@3.9587357	665.1823	3.96	POS	hilic				5.87E-01	3.64E-01	-0.23	2.00E-01	0.41	7.20E-01	0.10	7.68E-01	0.09
665.2581@9.872152	665.2581	9.87	NEG	RP				3.69E-01	9.50E-02	0.41	5.11E-01	0.20	3.97E-01	-0.23	1.14E-01	0.44
665.2622@9.955725	665.2622	9.96	NEG	RP				2.95E-01	6.13E-02	0.47	6.42E-01	0.14	5.44E-01	-0.17	5.65E-02	0.54
665.3239@5.389529	665.3239	5.39	NEG	RP				8.84E-01	7.69E-01	-0.08	7.10E-01	0.10	3.23E-01	0.27	5.41E-01	0.22
665.3263@0.8663161	665.3263	0.87	POS	hilic				9.87E-01	6.70E-01	-0.13	9.51E-01	-0.02	7.13E-01	0.09	9.35E-01	-0.03
665.3274@5.383325	665.3274	5.38	POS	RP				8.63E-01	9.58E-01	0.02	4.35E-01	0.21	1.65E-01	0.35	5.38E-01	0.22
665.3326@0.49258512	665.3326	0.49	POS	hilic				5.62E-01	9.23E-01	-0.03	1.20E-01	0.43	1.87E-01	0.37	2.15E-01	0.35
665.3583@10.930022	665.3583	10.93	POS	RP				4.20E-01	2.93E-02	0.53	1.41E-01	0.43	2.31E-01	0.36	9.01E-01	-0.03
665.428@10.327789	665.4280	10.33	POS	RP				9.87E-01	8.13E-01	0.07	9.91E-01	0.00	8.24E-01	0.06	5.44E-01	0.16
665.5965@14.642636	665.5965	14.64	POS	RP				1.39E-01	1.01E-01	0.42	5.12E-03	0.70	6.60E-03	0.85	2.74E-01	0.35
665.7657@15.274641	665.7657	15.27	NEG	RP				9.17E-01	5.54E-01	0.18	9.38E-01	0.02	3.63E-01	0.23	3.76E-01	0.23
666.1782@9.337401	666.1782	9.34	NEG	RP				9.80E-01	5.19E-01	0.18	9.73E-01	-0.01	9.91E-01	0.00	6.45E-01	0.13
666.1792@0.40113655	666.1792	0.40	NEG	hilic				4.20E-01	7.44E-02	0.55	1.27E-01	0.47	9.04E-01	-0.03	2.87E-01	0.33
666.1795@9.235908	666.1795	9.24	NEG	RP				8.06E-01	4.24E-01	-0.21	6.56E-01	0.12	4.11E-01	0.24	8.92E-01	0.04
666.18@9.434411	666.1800	9.43	NEG	RP				9.88E-01	8.01E-01	0.08	9.35E-01	-0.02	9.03E-01	-0.04	6.57E-01	-0.13
666.1802@9.532645	666.1802	9.53	NEG	RP				9.32E-01	5.12E-01	0.17	7.60E-01	0.08	5.98E-01	0.16	3.08E-01	0.28
666.4366@10.782787	666.4366	10.78	NEG	RP				1.36E-02	9.55E-02	-0.51	3.53E-02	0.61	3.19E-02	0.57	9.74E-01	0.01
667.2777@10.24001	667.2777	10.24	NEG	RP				2.38E-01	3.13E-02	0.66	5.85E-01	0.16	8.83E-01	0.04	5.55E-02	0.60
667.2832@1.2512255	667.2832	1.25	NEG	hilic				1.15E-01	9.30E-01	-0.02	1.60E-01	0.41	6.04E-02	0.58	3.33E-01	-0.29
667.3025@10.510783	667.3025	10.51	NEG	RP				1.95E-01	6.55E-02	0.52	2.28E-03	0.63	9.77E-03	0.52	5.69E-02	0.47
667.372@3.1755552	667.3720	3.18	POS	hilic				8.96E-01	4.07E-01	0.25	7.26E-01	0.09	4.61E-01	0.22	2.76E-01	0.33
667.402@10.5590315	667.4020	10.56	NEG	RP				5.11E-01	2.56E-01	-0.25	3.06E-01	0.29	4.08E-01	0.21	7.51E-01	-0.08
667.424@10.7	667.4240	10.70	NEG	RP				3.87E-01	4.20E-01	-0.15	1.36E-01	0.36	1.33E-01	0.35	9.31E-01	0.02
667.462@11.038231	667.4620	11.04	NEG	RP				2.25E-01	2.44E-02	0.69	4.34E-02	0.57	5.29E-02	0.55	7.24E-01	0.11
667.4784@10.7184105	667.4784	10.72	NEG	RP				2.42E-01	1.98E-02	0.68	7.98E-01	0.07	8.19E-02	0.38	8.21E-01	-0.06
668.2804@10.2424135	668.2804	10.24	NEG	RP				3.77E-01	1.85E-01	0.35	1.43E-01	0.36	5.80E-01	-0.12	1.31E-01	0.41
668.4339@10.781024	668.4339	10.78	NEG	RP				5.46E-02	1.99E-01	-0.30	4.58E-02	0.57	4.89E-02	0.49	9.96E-01	0.00
668.465@11.036282	668.4650	11.04	NEG	RP				4.17E-01	1.27E-01	0.49	5.23E-02	0.56	2.57E-02	0.64	1.45E-01	0.42
669.2944@10.501732	669.2944	10.50	NEG	RP				3.03E-03	1.03E-04	1.02	3.85E-01	-0.24	3.20E-01	-0.26	2.27E-01	0.31
669.2955@10.462172	669.2955	10.46	NEG	RP				3.59E-01	8.43E-01	0.05	2.44E-01	-0.23	1.72E-01	0.29	2.05E-01	0.34
669.3188@1.3017077	669.3188	1.30	POS	hilic				8.48E-01	9.58E-01	0.01	4.93E-01	0.17	6.88E-01	-0.12	3.88E-01	0.22
669.4371@10.775815	669.4371	10.78	NEG	RP				4.99E-03	7.23E-02	-0.46	5.41E-02	0.55	4.23E-03	0.75	8.45E-01	0.05
670.1303@8.465066	670.1303	8.47	NEG	RP				1.47E-01	7.53E-01	-0.06	1.14E-02	0.61	3.45E-01	0.19	1.30E-01	0.39
670.1307@8.812292	670.1307	8.81	NEG	RP				9.41E-01	7.08E-01	0.11	9.85E-01	0.01	6.51E-01	-0.13	5.62E-01	0.17
670.2698@9.7840805	670.2698	9.78	NEG	RP				7.12E-01	6.24E-01	0.16	3.92E-01	0.21	7.25E-01	0.08	8.15E-02	0.48
671.2836@1.2267718	671.2836	1.23	POS	hilic				7.04E-02	4.04E-02	-0.59	8.97E-01	0.03	1.05E-01	0.45	4.70E-01	-0.17
671.4019@3.442549	671.4019	3.44	NEG	hilic				4.25E-01	2.61E-01	0.30	5.95E-01	0.12	3.17E-02	0.60	4.01E-01	0.19
671.4328@10.777884	671.4328	10.78	POS	RP				3.20E-02	6.04E-01	-0.14	9.07E-03	0.70	9.99E-03	0.71	8.53E-01	0.06
671.4419@10.7155905	671.4419	10.72	NEG	RP				2.64E-01	2.73E-01	-0.31	8.59E-02	-0.44	7.55E-03	-0.68	6.35E-01	-0.13
671.4997@0.4348333	671.4997	0.43	POS	hilic				9.97E-01	9.76E-01	-0.01	8.73E-01	0.04	7.69E-01	-0.07	9.23E-01	-0.02
672.2814@10.013332	672.2814	10.01	NEG	RP				4.72E-01	4.97E-01	0.24	7.05E-01	0.10	2.89E-02	0.63	7.99E-01	0.08
672.2817@10.141691	672.2817	10.14	NEG	RP				1.71E-01	2.54E-01	-0.38	5.85E-02	0.50	1.15E-01	0.38	9.40E-01	0.02
672.2817@10.150111	672.2817	10.15	NEG	RP				5.80E-01	2.61E-01	0.39	5.80E-01	0.16	1.94E-01	0.35	3.16E-02	0.58
672.3975@0.4356667	672.3975	0.44	NEG	hilic				3.30E-02	5.17E-02	-0.44	1.06E-01	0.38	4.21E-01	-0.18	1.35E-01	0.46
672.5209@0.9294782	672.5209	0.93	POS	hilic				5.75E-01	3.24E-01	0.30	5.15E-02	0.52	1.06E-01	0.46	7.58E-01	0.11
673.3583@0.42847505	673.3583	0.43	POS	hilic				8.38E-01	8.08E-01	0.07	7.22E-01	0.09	4.24E-01	0.20	4.82E-01	-0.20
673.4597@0.40881822	673.4597	0.41	POS	hilic				1.70E-01	1.33E-02	-0.46	2.26E-01	-0.25	1.33E-02	-0.46	3.47E-01	-0.21
673.4672@11.036233	673.4672	11.04	NEG	RP				4.14E-01	7.63E-02	0.57	1.82E-01	0.34	4.17E-02	0.63	9.57E-02	0.47
674.132@0.4008878	674.1320	0.40	NEG	hilic				8.71E-01	2.72E-01	0.34	2.79E-01	0.33	3.39E-01	0.27	5.07E-01	0.20
674.1838@0.40645882	674.1838	0.41	NEG	hilic				9.41E-01	8.56E-01	-0.04	6.21E-01	0.13	6.78E-01	0.11	4.18E-01	0.23
674.2319@0.4184166	674.2319	0.42	NEG	hilic				6.86E-01	7.93E-01	-0.08	2.69E-01	-0.32	6.20E-02	-0.45	2.51E-01	-0.35
674.3978@11.187836	674.3978	11.19	NEG	RP				5.38E-01	3.53E-01	0.25	9.29E-01	-0.02	3.32E-01	-0.25	2.99E-01	0.30
674.4076@0.43560615	674.4076	0.44	NEG	hilic				2.90E-02	1.29E-02	-0.55	3.12E-01	0.25	9.85E-01	0.00	8.93E-02	0.51
674.5367@11.643483	674.5367	11.64	POS	RP	SM d32:1	10 eV: 675.5428 (100), 184.0739 (60)	2	4.98E-01	9.29E-01	0.02	3.53E-01	-0.25	9.92E-02	0.42	8.23E-01	-0.07
674.5371@0.9286237	674.5371	0.93	POS	hilic				5.45E-01	3.54E-01	0.29	3.94E-02	0.54	9.95E-02	0.46	7.46E-01	0.11
675.1499@1.4773177	675.1499	1.48	NEG	hilic				9.18E-01	8.01E-01	0.10	3.93E-01	-0.21	7.30E-01	-0.10	7.67E-01	0.07

Supplementary table 2: Metabolomics analysis results

								ANOVA	Anorexia vs control		overweight vs control		obese vs control		athletes vs control	
Compound	Mass	Retention Time	Ionization mode	column	Compound_ID	MSMS_spectra	ID_level	P-FDR	P	Estimate	P	Estimate	P	Estimate	P	Estimate
675.2779@1.0839901	675.2779	1.08	POS	hilic				3.75E-01	5.70E-01	0.17	4.94E-01	0.17	2.75E-01	0.31	1.47E-01	-0.40
675.2801@9.85441	675.2801	9.85	NEG	RP				2.69E-01	1.58E-01	0.36	1.63E-01	-0.45	3.23E-01	-0.29	8.58E-01	0.05
675.2828@9.916058	675.2828	9.92	NEG	RP				1.09E-01	6.36E-01	0.11	2.32E-01	0.35	7.96E-03	0.84	8.80E-01	-0.04
675.3623@3.1760733	675.3623	3.18	POS	hilic				5.56E-01	1.48E-01	-0.43	3.44E-01	-0.27	5.33E-01	0.18	5.12E-01	-0.21
675.3706@0.43723297	675.3706	0.44	POS	hilic				9.74E-01	8.55E-01	0.06	6.78E-01	0.11	8.48E-01	-0.05	6.99E-01	-0.13
675.4629@11.030872	675.4629	11.03	POS	RP				6.67E-01	1.00E-01	0.50	9.28E-01	0.03	3.20E-01	0.33	4.74E-01	0.24
675.4864@0.6614284	675.4864	0.66	POS	hilic				8.01E-01	1.71E-01	0.40	5.74E-01	0.14	4.18E-01	0.21	2.86E-01	0.33
675.525@11.066376	675.5250	11.07	POS	RP				4.28E-01	3.55E-01	-0.30	2.19E-01	0.36	2.77E-01	0.35	9.75E-01	0.01
675.5401@0.92811096	675.5401	0.93	POS	hilic				4.92E-01	3.95E-01	0.25	3.01E-02	0.58	1.01E-01	0.47	7.57E-01	0.11
676.159@0.40626737	676.1590	0.41	NEG	hilic				9.21E-01	7.78E-01	-0.08	6.12E-01	-0.14	5.74E-01	-0.16	2.45E-01	-0.32
676.3592@10.435028	676.3592	10.44	NEG	RP				7.05E-03	2.52E-02	-0.32	2.47E-02	0.55	5.80E-02	0.47	5.00E-01	-0.12
676.4175@0.4346251	676.4175	0.43	NEG	hilic				1.32E-01	2.64E-02	-0.48	2.67E-01	0.30	3.27E-01	-0.22	4.71E-01	0.20
676.4618@11.037047	676.4618	11.04	NEG	RP				4.43E-01	5.26E-02	0.50	7.33E-01	0.10	5.79E-02	0.59	4.60E-01	0.22
677.1636@1.477327	677.1636	1.48	NEG	hilic				8.45E-01	3.99E-01	0.34	6.89E-01	-0.09	9.29E-01	0.03	6.85E-01	0.10
677.3059@10.041478	677.3059	10.04	NEG	RP				4.37E-02	1.82E-02	-0.53	2.26E-01	-0.36	2.40E-01	0.34	1.90E-02	-0.61
677.3093@10.16459	677.3093	10.16	NEG	RP				2.30E-02	5.72E-01	0.17	1.36E-02	0.67	5.73E-04	0.89	7.89E-01	-0.08
677.3111@1.2512157	677.3111	1.25	NEG	hilic				1.46E-01	8.44E-01	0.06	7.47E-01	0.09	7.27E-03	0.83	8.63E-01	0.05
677.3316@0.45367077	677.3316	0.45	POS	hilic				6.42E-01	1.15E-01	-0.42	9.49E-01	0.02	8.19E-01	0.06	6.90E-01	0.11
677.3476@1.3734063	677.3476	1.37	NEG	hilic				4.00E-01	4.49E-01	-0.11	2.75E-01	0.23	5.38E-02	0.41	4.93E-01	0.16
677.4705@10.021121	677.4705	10.02	POS	RP				2.33E-02	5.02E-02	-0.57	2.42E-01	-0.35	1.08E-01	-0.43	2.08E-05	-1.10
677.5002@11.628445	677.5002	11.63	POS	RP				8.58E-01	3.72E-01	0.26	7.03E-01	-0.10	6.18E-01	0.12	5.08E-01	0.20
677.5016@0.66111326	677.5016	0.66	POS	hilic				7.19E-01	1.32E-01	0.37	7.89E-01	0.05	8.99E-01	0.03	3.25E-01	0.26
678.1477@0.4064275	678.1477	0.41	NEG	hilic				5.04E-01	2.83E-02	0.59	9.92E-02	0.52	1.33E-01	0.37	1.38E-01	0.46
678.2167@3.9564419	678.2167	3.96	POS	hilic				3.58E-01	1.83E-01	-0.33	9.08E-01	0.03	3.89E-01	0.22	1.29E-01	-0.43
678.3056@10.166476	678.3056	10.17	NEG	RP				4.38E-01	4.63E-01	0.23	2.99E-02	0.60	7.09E-02	0.47	1.68E-01	0.30
678.4243@14.199526	678.4243	14.20	POS	RP				2.81E-01	3.17E-02	0.60	7.71E-02	0.50	5.79E-01	0.15	7.30E-01	-0.11
678.4856@0.7168197	678.4856	0.72	POS	hilic				3.20E-01	1.81E-01	-0.29	3.63E-01	0.25	5.59E-01	0.13	1.28E-01	0.39
678.5002@0.70299065	678.5002	0.70	POS	hilic				4.21E-01	4.03E-01	0.26	1.45E-01	0.38	2.82E-01	0.27	3.44E-02	0.70
678.5552@0.5303571	678.5552	0.53	POS	hilic				2.18E-01	4.59E-02	-0.32	4.59E-02	-0.32	4.59E-02	-0.32	5.13E-01	-0.13
678.5588@10.665355	678.5588	10.67	POS	hilic				4.68E-01	4.17E-01	0.26	3.09E-01	-0.28	2.67E-01	0.31	5.83E-01	-0.15
679.2411@10.174685	679.2411	10.17	NEG	RP				2.52E-01	5.85E-02	-0.41	2.80E-01	0.29	3.72E-01	0.23	9.13E-01	-0.03
679.3114@10.365407	679.3114	10.37	NEG	RP				1.68E-02	4.98E-02	0.57	1.15E-01	-0.38	4.68E-02	-0.43	9.53E-01	0.02
679.3176@9.880251	679.3176	9.88	NEG	RP				5.00E-01	4.18E-01	0.22	1.44E-01	-0.37	6.82E-01	0.12	7.66E-01	0.08
679.3182@9.909645	679.3182	9.91	NEG	RP				5.95E-01	3.14E-01	-0.21	2.62E-01	0.28	9.72E-01	0.01	2.40E-01	0.27
679.3295@0.45341668	679.3295	0.45	POS	hilic				6.58E-01	9.79E-01	-0.01	3.46E-01	-0.26	7.79E-01	-0.08	2.97E-01	0.29
679.3773@10.930199	679.3773	10.93	POS	RP				4.44E-01	5.25E-01	0.18	9.39E-02	0.49	5.77E-02	0.61	5.92E-01	0.16
679.4029@10.567477	679.4029	10.57	NEG	RP				9.71E-02	2.79E-02	-0.54	2.81E-01	0.31	1.88E-01	0.35	8.82E-01	-0.04
680.1588@8.708483	680.1588	8.71	NEG	RP				4.06E-01	2.56E-01	-0.31	2.96E-01	0.29	5.45E-01	-0.18	2.78E-01	0.30
680.1588@8.808536	680.1588	8.81	NEG	RP				3.10E-01	9.00E-01	0.04	4.90E-02	0.54	5.16E-02	0.64	4.50E-01	0.22
680.1594@8.51421	680.1594	8.51	NEG	RP				9.77E-01	7.62E-01	-0.09	9.91E-01	0.00	6.41E-01	0.13	7.56E-01	-0.09
680.1596@8.441036	680.1596	8.44	NEG	RP				7.83E-01	3.84E-01	-0.27	6.17E-01	0.13	6.21E-01	0.15	5.97E-01	-0.17
680.1609@0.4064275	680.1609	0.41	NEG	hilic				4.60E-01	4.25E-02	0.57	7.45E-02	0.56	1.70E-01	0.33	9.62E-02	0.52
680.1961@9.48787	680.1961	9.49	NEG	RP				7.49E-01	4.11E-01	0.19	4.32E-01	0.21	6.61E-02	0.40	2.09E-01	0.31
680.3923@10.709576	680.3923	10.71	NEG	RP				1.96E-01	5.24E-01	-0.16	4.28E-01	0.22	3.47E-02	0.62	5.68E-01	-0.16
681.1513@6.0240707	681.1513	6.02	POS	hilic				9.02E-01	4.50E-01	0.22	8.30E-01	0.05	2.81E-01	0.26	4.71E-01	0.17
681.164@0.40642354	681.1640	0.41	NEG	hilic				4.46E-01	7.85E-02	0.54	4.32E-01	0.24	9.67E-01	-0.01	7.27E-02	0.49
681.3192@9.933073	681.3192	9.93	NEG	RP				2.04E-01	2.05E-02	-0.58	3.87E-01	-0.24	6.33E-01	0.14	5.78E-02	-0.46
681.321@1.2614831	681.3210	1.26	POS	hilic				6.33E-01	2.40E-01	-0.42	7.74E-01	-0.07	4.37E-01	0.21	6.14E-01	-0.17
681.3246@1.2709358	681.3246	1.27	NEG	hilic				7.21E-02	1.22E-03	-1.00	3.10E-01	-0.28	4.35E-01	-0.23	6.89E-02	-0.51
681.3364@0.44007146	681.3364	0.44	POS	hilic				6.49E-01	5.44E-01	-0.15	4.88E-01	-0.18	6.59E-01	0.12	2.97E-01	0.29
681.357@0.75054294	681.3570	0.75	POS	hilic				2.92E-02	7.34E-03	-0.76	1.30E-01	0.40	4.63E-01	0.22	8.22E-01	-0.06
681.3898@10.319753	681.3898	10.32	NEG	RP				8.22E-01	8.78E-01	0.04	1.89E-01	0.35	6.00E-01	0.14	9.09E-01	0.03
681.3928@10.567511	681.3928	10.57	NEG	RP				2.98E-02	9.20E-03	-0.67	3.12E-01	0.29	1.31E-01	0.38	6.51E-01	-0.13
682.2632@10.162742	682.2632	10.16	NEG	RP				5.12E-01	2.02E-01	0.43	6.31E-01	0.14	5.86E-01	0.16	3.26E-01	-0.25
682.3057@10.241075	682.3057	10.24	NEG	RP				2.56E-01	2.98E-01	-0.28	1.83E-01	-0.37	7.98E-01	0.08	7.89E-03	-0.64
682.3071@2.1545186	682.3071	2.15	NEG	hilic				2.22E-03	2.37E-03	0.74	6.99E-02	-0.41	3.23E-01	-0.25	3.10E-01	0.28
682.355@0.86444795	682.3550	0.86	POS	hilic				9.84E-01	5.29E-01	-0.19	7.00E-01	-0.11	9.99E-01	0.00	8.14E-01	-0.08
682.3553@5.381385	682.3553	5.38	POS	RP				8.32E-01	8.05E-01	0.08	3.23E-01	0.28	1.24E-01	0.39	5.02E-01	0.24
682.4397@0.4277001	682.4397	0.43	NEG	hilic				7.88E-03	3.22E-02	-0.49	9.29E-02	0.44	7.20E-03	0.64	5.56E-01	0.14
683.1953@12.315504	683.1953	12.32	POS	RP				3.36E-01	1.83E-01	-0.37	4.03E-01	-0.23	2.84E-01	0.32	1.77E-01	-0.39
683.3474@0.42751563	683.3474	0.43	POS	hilic				9.13E-01	6.90E-01	0.11	3.92E-01	-0.20	9.12E-01	-0.03	7.38E-01	-0.08
683.4466@10.782895	683.4466	10.78	NEG	RP				7.00E-01	2.65E-01	-0.25	7.61E-01	0.08	4.42E-01	0.19	5.19E-01	-0.16
683.5015@11.036001	683.5015	11.04	NEG	RP				4.97E-01	4.69E-01	-0.22	2.19E-01	0.34	1.98E-01	0.38	8.75E-01	0.04
683.5488@13.452458	683.5488	13.45	POS	RP				1.24E-02	5.58E-02	-0.57	7.31E-02	0.45	1.83E-02	0.67	9.46E-01	0.02
684.1331@8.26375	684.1331	8.26	NEG	RP				9.70E-01	9.17E-01	0.04	5.04E-01	0.18	9.47E-01	-0.02	6.00E-01	0.16
684.1463@0.40055132	684.1463	0.40	NEG	hilic				7.84E-01	9.51E-01	-0.02	9.00E-01	0.03	2.59E-01	0.30	2.41E-01	0.30
684.3876@0.8461111	684.3876	0.85	POS	hilic				5.67E-01	9.34E-01	-0.02	8.30E-01	-0.05	1.90E-02	-0.41	9.34E-01	-0.02
684.3948@10.708265	684.3948	10.71	NEG	RP				8.34E-01	8.84E-02	0.36	8.18E-01	0.06	9.65E-01	0.01	6.36E-01	0.12
684.4215@11.560037	684.4215	11.56	POS	RP				2.83E-01	1.68E-01	0.38	6.97E-01	0.13	7.81E-02	-0.45	4.31E-01	-0.21
684.4546@0.4284118	684.4546	0.43	NEG	hilic				3.01E-03	2.26E-02	-0.51	1.38E-01	0.41	5.44E-03	0.65	6.04E-01	-0.12
684.5258@0.91815376	684.5258	0.92	POS	hilic				3.45E-01	3.48E-01	-0.20	6.72E-02	0.48	4.01E-01	0.23	5.24E-01	0.16
684.5759@0.89641035	684.5759	0.90	POS	hilic				4.55E-01	2.47E-01	0.28	6.46E-01	-0.09	1.43E-01	0.38	8.34E-01	0.04
685.4252@10.783087	685.4252	10.78	NEG	RP				1.53E-02	1.26E-01	-0.44	3.19E-02	0.61	2.49E-02	0.58	9.10E-01	-0.03

Supplementary table 2: Metabolomics analysis results

								ANOVA	Anorexia vs control		overweight vs control		obese vs control		athletes vs control	
Compound	Mass	Retention Time	Ionization mode	column	Compound_ID	MSMS_spectra	ID_level	P-FDR	P	Estimate	P	Estimate	P	Estimate	P	Estimate
685.4545@0.40509525	685.4545	0.41	POS	hilic				2.00E-01	1.24E-02	-0.48	4.34E-02	-0.40	1.10E-01	-0.33	2.74E-01	-0.24
685.4568@10.64014	685.4568	10.64	POS	RP				7.99E-01	5.26E-01	-0.16	5.20E-01	-0.16	4.94E-01	0.17	8.28E-01	0.06
685.5049@11.036828	685.5049	11.04	NEG	RP				3.00E-01	9.33E-01	0.02	2.36E-01	0.35	2.16E-02	0.61	6.88E-01	-0.12
686.1298@0.39883766	686.1298	0.40	NEG	hilic				9.29E-01	5.39E-01	0.19	7.54E-01	0.09	8.13E-01	0.07	2.56E-01	0.31
686.2204@0.5768037	686.2204	0.58	POS	hilic				3.39E-01	1.19E-01	-0.55	3.89E-01	-0.23	7.90E-03	-0.70	3.20E-01	-0.30
686.2603@10.01203	686.2603	10.01	NEG	RP				1.16E-01	2.62E-01	0.34	3.56E-01	0.25	9.13E-03	0.64	6.69E-03	0.87
686.2621@10.122863	686.2621	10.12	NEG	RP				2.52E-01	8.16E-01	-0.07	2.14E-01	0.41	4.28E-01	0.18	6.35E-03	0.68
686.2624@10.148791	686.2624	10.15	NEG	RP				4.52E-01	5.18E-01	0.21	2.70E-02	0.68	1.93E-01	0.28	3.70E-01	0.27
686.5075@11.036698	686.5075	11.04	NEG	RP				7.62E-01	8.20E-01	0.06	7.47E-01	-0.09	3.76E-01	0.27	3.90E-01	-0.23
686.534@11.661658	686.5340	11.66	NEG	RP				6.73E-03	3.72E-02	-0.58	9.80E-02	0.44	2.26E-02	0.62	3.84E-01	-0.24
686.5364@0.91332483	686.5364	0.91	POS	hilic				9.40E-01	9.04E-01	0.04	2.38E-01	0.28	7.49E-01	0.10	8.79E-01	0.05
687.1334@0.3995816	687.1334	0.40	NEG	hilic				9.01E-01	9.04E-01	-0.03	6.21E-01	-0.15	3.01E-01	-0.32	9.86E-01	0.00
687.2543@9.780646	687.2543	9.78	NEG	RP				8.94E-01	3.96E-01	0.32	9.78E-01	0.01	3.97E-01	0.20	7.77E-01	0.08
687.2653@10.009509	687.2653	10.01	NEG	RP				9.83E-02	1.00E-01	0.54	7.69E-02	0.48	5.34E-04	0.98	1.19E-01	0.46
687.3111@5.3822913	687.3111	5.38	POS	RP				8.96E-01	9.96E-01	0.00	4.88E-01	0.19	2.02E-01	0.32	6.58E-01	0.16
687.4229@10.56464	687.4229	10.56	NEG	RP				1.89E-01	1.71E-02	-0.58	3.25E-01	0.27	7.97E-01	0.07	5.20E-01	-0.18
687.4638@11.05089	687.4638	11.05	NEG	RP				6.45E-01	1.08E-01	0.47	4.60E-01	0.24	7.58E-02	0.50	1.54E-01	0.37
687.4669@10.321131	687.4669	10.32	NEG	RP				8.09E-01	3.01E-01	0.30	2.75E-01	0.30	6.00E-01	0.13	1.82E-01	0.34
687.5195@0.66034204	687.5195	0.66	POS	hilic				7.47E-01	1.61E-01	0.36	7.22E-01	0.08	9.30E-01	0.02	9.29E-01	0.02
687.5407@0.9233602	687.5407	0.92	POS	hilic				2.00E-01	7.08E-01	-0.10	1.22E-02	0.62	1.38E-01	0.46	9.33E-01	-0.03
687.7385@7.873653	687.7385	7.87	POS	hilic				8.61E-01	3.84E-01	0.30	6.59E-01	0.11	2.09E-01	0.36	5.39E-01	0.17
688.4058@10.709335	688.4058	10.71	NEG	RP				1.17E-01	6.47E-01	0.11	4.75E-01	0.13	3.66E-03	0.70	4.14E-01	0.17
688.5507@11.942485	688.5507	11.94	NEG	RP				7.22E-01	8.84E-01	-0.05	2.65E-01	0.31	9.03E-01	0.04	4.16E-01	-0.24
688.5518@11.835567	688.5518	11.84	POS	RP				6.90E-01	5.85E-02	0.52	3.43E-01	0.25	5.55E-01	0.17	3.27E-01	0.31
688.5523@11.823808	688.5523	11.82	POS	RP	SM d33:1	10 eV: 689.5582 (100), 184.0733 (35)	2	7.64E-01	4.86E-01	0.22	4.14E-01	0.20	1.88E-01	0.39	8.51E-01	-0.06
688.5524@0.91121376	688.5524	0.91	POS	hilic				8.47E-01	5.32E-01	0.19	2.06E-01	0.32	4.23E-01	0.24	9.30E-01	-0.03
689.2678@10.010862	689.2678	10.01	NEG	RP				5.16E-02	1.64E-01	0.45	1.17E-02	0.67	8.00E-04	1.01	3.10E-01	0.29
689.2686@10.136971	689.2686	10.14	NEG	RP				3.95E-01	2.99E-01	-0.32	2.64E-01	0.31	1.06E-01	0.39	6.63E-01	0.13
689.2694@10.151482	689.2694	10.15	NEG	RP				2.98E-01	7.63E-01	-0.10	1.89E-01	0.39	1.60E-02	0.59	1.18E-01	0.47
689.4072@11.493676	689.4072	11.49	NEG	RP				5.62E-03	2.28E-03	-0.73	2.66E-01	0.25	8.99E-01	-0.03	6.26E-02	0.57
689.5344@0.6575401	689.5344	0.66	POS	hilic				4.57E-01	1.23E-02	-0.57	7.75E-01	-0.07	6.63E-01	-0.11	4.76E-01	-0.20
689.5554@0.90707016	689.5554	0.91	POS	hilic				7.70E-01	8.36E-01	-0.06	8.90E-02	0.39	6.84E-01	0.12	9.09E-01	0.04
690.007@6.1878715	690.0070	6.19	POS	hilic				8.31E-01	4.04E-01	0.25	2.82E-01	0.29	8.64E-01	-0.04	7.17E-01	0.10
690.3487@0.5021645	690.3487	0.50	POS	hilic				2.85E-01	4.59E-01	-0.20	2.79E-02	-0.59	2.33E-01	-0.32	8.27E-01	0.07
690.3687@0.6658787	690.3687	0.67	POS	hilic				9.97E-01	9.86E-01	0.00	9.23E-01	-0.02	9.51E-01	0.01	7.12E-01	-0.07
690.4921@10.95965	690.4921	10.96	NEG	RP				1.06E-01	2.15E-01	-0.24	3.77E-01	0.21	3.55E-02	0.54	5.33E-01	-0.15
691.0111@6.2298284	691.0111	6.23	NEG	hilic				6.02E-01	1.24E-01	0.48	8.22E-02	0.44	1.42E-01	0.37	1.32E-01	0.47
691.0116@6.183796	691.0116	6.18	POS	hilic				3.40E-01	3.48E-02	0.64	3.71E-01	0.29	2.02E-01	0.38	8.28E-03	0.68
691.2813@1.2096871	691.2813	1.21	NEG	hilic				2.93E-01	4.83E-01	-0.20	8.79E-01	0.04	4.26E-02	0.56	8.93E-01	0.04
691.4148@10.320896	691.4148	10.32	NEG	RP				3.24E-01	1.91E-01	0.36	4.79E-01	-0.19	4.19E-01	-0.19	1.78E-01	-0.31
691.4222@10.567507	691.4222	10.57	NEG	RP				7.21E-02	6.25E-02	-0.52	1.84E-01	0.41	9.36E-02	0.44	9.18E-01	-0.03
691.4557@11.050183	691.4557	11.05	NEG	RP				4.06E-01	2.76E-02	0.63	3.38E-01	0.29	6.45E-02	0.51	7.16E-01	0.10
691.4585@10.783524	691.4585	10.78	NEG	RP				6.46E-02	1.13E-01	-0.43	1.38E-01	0.43	8.62E-02	0.47	5.24E-01	-0.19
691.5177@0.72099984	691.5177	0.72	POS	hilic				1.49E-01	3.39E-02	0.57	8.03E-02	0.43	6.55E-01	-0.09	8.53E-02	0.48
691.8269@4.4850693	691.8269	4.49	POS	hilic				9.69E-01	8.57E-01	0.06	9.25E-01	-0.03	7.50E-01	0.09	5.62E-01	-0.17
692.0171@6.2286916	692.0171	6.23	NEG	hilic				2.61E-01	3.25E-02	0.66	7.48E-02	0.50	2.37E-01	0.30	2.05E-02	0.68
692.1314@9.006947	692.1314	9.01	NEG	RP				5.93E-01	6.30E-02	0.52	7.55E-01	-0.10	8.52E-01	0.05	7.01E-01	0.11
692.1322@9.321498	692.1322	9.32	NEG	RP				5.66E-01	2.34E-02	0.63	2.20E-01	0.36	5.57E-01	0.18	5.02E-01	0.22
692.1327@9.223378	692.1327	9.22	NEG	RP				5.31E-01	8.48E-02	0.54	1.23E-01	0.49	1.19E-01	0.46	5.12E-01	0.20
692.1343@9.53726	692.1343	9.54	NEG	RP				6.20E-01	3.99E-01	-0.23	6.21E-01	-0.15	2.77E-01	0.31	5.39E-01	0.20
692.1371@0.39769086	692.1371	0.40	NEG	hilic				3.27E-01	5.66E-01	0.14	2.13E-01	0.29	6.41E-01	0.10	2.23E-02	0.63
692.4058@0.41069233	692.4058	0.41	NEG	hilic				6.45E-02	1.21E-01	-0.30	2.38E-02	-0.40	2.38E-02	-0.40	2.38E-02	-0.40
692.5089@11.202299	692.5089	11.20	NEG	RP				3.23E-01	9.49E-01	0.02	5.52E-02	0.46	3.51E-02	0.58	9.31E-01	-0.03
693.2966@1.1839882	693.2966	1.18	NEG	hilic				7.53E-01	2.02E-01	0.35	3.59E-01	0.26	2.46E-01	0.33	9.46E-02	0.41
693.4083@11.042494	693.4083	11.04	NEG	RP				8.81E-01	9.11E-01	0.02	7.11E-01	-0.11	6.78E-01	-0.10	4.57E-01	0.23
693.4615@10.782981	693.4615	10.78	NEG	RP				1.31E-01	8.57E-01	-0.05	1.38E-01	0.47	4.40E-03	0.74	7.59E-01	0.09
693.4882@12.724747	693.4882	12.72	POS	RP				9.45E-01	7.41E-01	0.10	8.44E-01	-0.06	7.13E-01	0.11	3.85E-01	0.23
693.492@11.03634	693.4920	11.04	NEG	RP				5.13E-01	7.19E-01	0.10	1.34E-01	-0.34	2.26E-01	-0.32	1.81E-01	-0.30
694.1755@0.4063048	694.1755	0.41	NEG	hilic				4.67E-01	8.57E-01	0.05	5.33E-01	-0.16	6.06E-01	0.12	6.08E-02	0.49
694.5024@0.9404118	694.5024	0.94	POS	hilic				9.71E-01	7.48E-01	-0.10	9.81E-01	0.01	8.98E-01	0.04	5.55E-01	-0.19
695.2716@10.170332	695.2716	10.17	NEG	RP				4.11E-01	1.41E-01	-0.31	2.73E-01	0.28	8.73E-01	-0.04	3.13E-01	0.24
695.2933@9.845777	695.2933	9.85	NEG	RP				1.85E-02	7.07E-03	0.78	1.50E-01	0.24	6.31E-01	-0.06	1.82E-01	0.28
695.454@10.783278	695.4540	10.78	NEG	RP				2.24E-02	2.22E-01	-0.36	2.66E-02	0.65	1.59E-02	0.64	9.70E-01	0.01
695.7849@15.280128	695.7849	15.28	NEG	RP				8.96E-01	8.14E-01	0.08	6.98E-01	-0.10	6.64E-01	-0.12	2.74E-01	-0.27
695.963@10.11846	695.9630	10.12	NEG	RP				1.83E-01	1.38E-02	0.67	2.86E-02	0.63	1.19E-02	0.68	3.26E-02	0.50
696.2808@9.950999	696.2808	9.95	NEG	RP				9.52E-02	8.90E-01	0.03	5.59E-02	-0.59	5.79E-03	-0.67	8.04E-01	0.08
696.2824@9.8457	696.2824	9.85	NEG	RP				6.11E-03	8.74E-01	0.04	1.30E-02	-0.78	3.42E-04	-0.91	9.19E-01	0.03
696.3326@1.9557414	696.3326	1.96	POS	hilic				2.45E-01	3.67E-01	-0.26	9.76E-01	0.01	1.63E-01	-0.33	2.21E-02	-0.75
696.457@10.7835245	696.4570	10.78	NEG	RP				2.08E-02	1.88E-01	-0.38	3.46E-02	0.62	1.86E-02	0.61	9.11E-01	-0.03
696.5188@11.636822	696.5188	11.64	POS	RP				8.23E-01	8.12E-01	-0.06	7.87E-01	-0.07	4.16E-01	0.23	4.46E-01	-0.20
697.3541@0.43723953	697.3541	0.44	POS	hilic				8.63E-01	4.36E-01	0.23	4.68E-01	0.20	5.71E-01	-0.15	9.11E-01	0.04
697.4312@10.709033	697.4312	10.71	NEG	RP				2.38E-01	1.85E-02	-0.72	7.37E-02	-0.47	5.29E-01	-0.17	6.09E-02	-0.55
697.4912@11.036961	697.4912	11.04	NEG	RP				5.89E-01	8.21E-02	0.47	3.36E-01	0.27	6.87E-02	0.52	1.89E-01	0.36

Supplementary table 2: Metabolomics analysis results

								ANOVA	Anorexia vs control		overweight vs control		obese vs control		athletes vs control	
Compound	Mass	Retention Time	Ionization mode	column	Compound_ID	MSMS_spectra	ID_level	P-FDR	P	Estimate	P	Estimate	P	Estimate	P	Estimate
698.1651@0.4022648	698.1651	0.40	NEG	hilic				6.21E-01	3.36E-01	0.28	3.17E-01	0.29	8.74E-01	0.04	8.14E-02	0.54
698.2971@10.238956	698.2971	10.24	NEG	RP				8.87E-01	7.34E-01	0.12	7.53E-01	0.08	8.98E-01	-0.03	4.20E-01	-0.24
698.3499@5.2993655	698.3499	5.30	POS	RP				8.79E-01	5.86E-01	0.16	8.93E-01	-0.03	3.19E-01	0.27	5.13E-01	0.19
698.3562@0.43772882	698.3562	0.44	POS	hilic				4.11E-01	6.30E-02	0.48	6.87E-02	0.48	7.08E-01	0.08	3.66E-01	0.21
698.3983@0.53869885	698.3983	0.54	POS	hilic				7.76E-01	3.03E-01	0.32	7.82E-01	-0.06	2.98E-01	0.29	6.81E-01	0.12
698.535@0.89558566	698.5350	0.90	POS	hilic				3.27E-05	1.50E-03	-0.80	2.20E-03	0.72	1.74E-02	0.64	1.73E-01	-0.36
699.2819@9.933741	699.2819	9.93	NEG	RP				1.20E-01	3.34E-01	-0.33	3.56E-02	0.49	2.27E-02	0.56	4.14E-01	0.27
699.5188@12.466068	699.5188	12.47	NEG	RP				4.76E-01	6.16E-01	-0.16	9.25E-01	-0.02	8.40E-01	0.05	8.13E-02	0.53
699.5213@0.68244046	699.5213	0.68	POS	hilic				7.24E-01	5.41E-01	0.16	5.75E-01	0.14	8.35E-01	-0.05	1.87E-01	0.41
699.669@7.8728456	699.6690	7.87	POS	hilic				5.95E-01	8.43E-01	0.05	5.98E-01	-0.11	1.62E-01	0.35	7.94E-01	-0.06
700.1449@9.163961	700.1449	9.16	NEG	RP				8.62E-01	4.50E-01	0.22	6.95E-01	0.12	4.51E-01	0.21	1.76E-01	0.39
700.1463@0.399146	700.1463	0.40	NEG	hilic				9.50E-01	5.99E-01	-0.16	9.09E-01	0.03	7.39E-01	-0.08	6.55E-01	0.12
700.1983@3.955515	700.1983	3.96	POS	hilic				6.43E-01	6.12E-02	-0.50	5.23E-01	-0.16	4.89E-01	-0.18	2.32E-01	-0.33
700.3113@10.435403	700.3113	10.44	NEG	RP				2.97E-02	9.66E-02	0.50	9.36E-02	-0.46	2.50E-01	-0.32	1.20E-01	0.43
700.3123@10.5027075	700.3123	10.50	NEG	RP				2.10E-03	4.98E-04	0.97	3.63E-01	-0.25	5.38E-01	-0.16	1.51E-02	0.61
700.4014@3.1368814	700.4014	3.14	POS	hilic				3.58E-01	2.17E-01	-0.43	1.71E-01	-0.37	4.70E-02	-0.55	4.41E-03	-0.70
700.4017@2.9867518	700.4017	2.99	POS	hilic				7.36E-01	7.16E-01	0.11	9.28E-01	-0.03	5.20E-02	0.46	6.79E-01	0.13
700.4884@11.036273	700.4884	11.04	NEG	RP				4.86E-01	1.48E-01	0.47	7.86E-02	0.50	3.66E-02	0.60	1.44E-01	0.43
700.5534@0.8917688	700.5534	0.89	POS	hilic				1.72E-01	3.79E-03	0.83	1.24E-01	0.41	7.37E-01	0.09	3.27E-01	0.27
700.5542@11.704946	700.5542	11.70	POS	RP	SM d34:2	20 eV: 184.0735 (100), 701.5602 (25)	2	3.96E-01	9.69E-01	0.01	2.17E-01	0.32	4.76E-02	0.52	7.88E-01	0.06
701.2065@10.6261215	701.2065	10.63	POS	RP				1.87E-01	7.17E-01	-0.11	3.90E-02	0.58	2.48E-01	0.31	4.63E-01	-0.23
701.304@10.073302	701.3040	10.07	NEG	RP				7.01E-01	3.61E-01	0.24	7.86E-01	-0.07	3.03E-01	-0.24	7.60E-01	-0.07
701.3097@9.846397	701.3097	9.85	NEG	RP				9.35E-02	3.25E-01	-0.25	4.67E-02	-0.52	1.16E-02	-0.66	5.78E-01	0.18
701.3118@1.2206551	701.3118	1.22	NEG	hilic				1.73E-01	6.42E-01	-0.13	8.20E-01	-0.07	6.28E-03	-0.84	4.01E-01	-0.26
701.3121@1.3388296	701.3121	1.34	NEG	hilic				2.14E-01	2.18E-02	-0.65	1.53E-01	-0.39	7.65E-01	0.09	1.68E-01	-0.39
701.4065@10.56636	701.4065	10.57	NEG	RP				2.37E-01	2.22E-02	-0.53	3.84E-01	0.24	6.75E-01	0.11	9.41E-01	-0.02
701.4469@10.3179035	701.4469	10.32	NEG	RP				8.61E-01	2.66E-01	0.29	3.89E-01	0.23	2.13E-01	0.28	4.93E-01	0.16
701.5024@0.6605493	701.5024	0.66	POS	hilic				5.08E-01	9.01E-02	0.48	7.66E-01	0.09	3.47E-01	0.27	5.78E-02	0.55
701.7371@15.278608	701.7371	15.28	NEG	RP				3.05E-01	2.03E-02	-0.64	2.15E-01	-0.35	8.46E-01	-0.05	1.07E-01	-0.49
701.8563@4.355514	701.8563	4.36	POS	hilic				9.79E-01	7.92E-01	0.08	5.25E-01	0.16	9.20E-01	-0.03	9.07E-01	-0.04
702.1604@9.224395	702.1604	9.22	NEG	RP				7.07E-01	8.03E-01	-0.08	8.92E-01	0.04	4.85E-01	0.21	1.92E-01	0.44
702.1606@9.006325	702.1606	9.01	NEG	RP				6.76E-01	6.73E-02	-0.57	4.64E-01	-0.21	6.47E-01	-0.13	5.84E-01	-0.17
702.1606@9.323214	702.1606	9.32	NEG	RP				1.21E-01	1.04E-02	-0.89	6.22E-01	-0.15	7.64E-02	-0.50	5.56E-02	-0.55
702.1617@0.39904258	702.1617	0.40	NEG	hilic				5.36E-01	4.32E-02	0.56	2.65E-01	0.35	7.32E-01	0.09	1.78E-01	0.44
702.5685@0.89037347	702.5685	0.89	POS	hilic				7.14E-01	7.59E-01	-0.09	1.18E-01	0.37	8.40E-01	-0.06	5.07E-01	0.21
702.5688@11.985874	702.5688	11.99	POS	RP	SM d34:1	10 eV: 703.5759 (100), 184.0733 (42)	2	6.64E-01	4.33E-01	0.22	1.53E-01	0.38	4.22E-01	0.24	6.12E-01	-0.15
703.1647@0.39927915	703.1647	0.40	NEG	hilic				7.05E-01	2.07E-01	0.37	7.68E-01	0.09	9.26E-01	-0.02	1.86E-01	0.39
703.2368@9.853079	703.2368	9.85	NEG	RP				5.67E-01	7.82E-01	0.08	4.09E-01	-0.25	1.87E-01	-0.36	4.71E-01	0.19
703.3222@10.260999	703.3222	10.26	NEG	RP				9.92E-01	6.97E-01	0.11	8.07E-01	0.06	6.23E-01	0.13	9.16E-01	0.03
703.3272@1.1835794	703.3272	1.18	NEG	hilic				2.97E-02	9.32E-02	0.57	6.17E-02	-0.54	1.65E-01	-0.37	4.44E-01	-0.21
703.3277@1.3165617	703.3277	1.32	NEG	hilic				3.75E-01	1.06E-01	-0.47	4.65E-01	0.20	4.16E-01	-0.23	2.63E-01	-0.30
703.3652@3.333261	703.3652	3.33	NEG	hilic				9.96E-01	6.69E-01	-0.12	7.06E-01	-0.09	8.72E-01	-0.04	8.95E-01	-0.03
703.4266@10.782193	703.4266	10.78	NEG	RP				1.25E-02	4.70E-01	-0.14	4.36E-02	0.55	1.11E-03	0.77	4.63E-01	0.16
703.4644@10.721558	703.4644	10.72	NEG	RP				5.92E-01	8.66E-01	0.05	1.17E-01	-0.41	9.17E-01	-0.02	1.99E-01	-0.34
703.5144@0.6608155	703.5144	0.66	POS	hilic				5.85E-01	8.25E-02	0.42	1.51E-01	0.34	9.07E-01	0.03	2.33E-01	0.32
703.5191@11.707302	703.5191	11.71	POS	RP				7.46E-01	1.52E-01	0.38	9.11E-01	0.03	3.06E-01	0.29	2.58E-01	0.34
703.5728@0.889786	703.5728	0.89	POS	hilic				8.17E-01	8.60E-01	0.05	9.28E-02	0.41	7.37E-01	0.11	5.81E-01	0.18
704.1273@8.908159	704.1273	8.91	NEG	RP				7.87E-01	6.08E-01	0.17	3.79E-01	0.24	6.65E-01	0.11	1.79E-01	0.46
704.143@6.024713	704.1430	6.02	POS	hilic				6.16E-01	8.44E-01	-0.05	6.00E-01	-0.12	3.91E-02	-0.47	8.97E-01	-0.04
704.5@11.042622	704.5000	11.04	NEG	RP				1.41E-01	7.19E-02	0.50	2.61E-02	0.63	7.57E-03	0.80	2.99E-01	0.27
704.5476@11.067519	704.5476	11.07	POS	RP				5.36E-01	1.67E-01	-0.36	6.79E-01	0.11	3.60E-01	0.27	6.73E-01	-0.11
705.2598@10.259572	705.2598	10.26	NEG	RP				1.99E-02	1.28E-03	-0.78	6.94E-01	-0.10	2.52E-03	-0.71	6.31E-02	-0.52
705.3438@10.417655	705.3438	10.42	NEG	RP				7.98E-01	4.88E-01	-0.18	2.61E-01	-0.33	5.49E-01	-0.17	1.24E-01	-0.37
705.3444@10.520234	705.3444	10.52	NEG	RP				4.09E-01	2.57E-02	0.65	2.58E-01	0.33	9.32E-01	0.03	2.36E-01	0.39
705.3463@3.1664443	705.3463	3.17	POS	hilic				4.68E-01	4.60E-01	-0.22	4.17E-01	0.22	5.54E-01	0.16	1.33E-01	0.46
705.3633@3.330697	705.3633	3.33	NEG	hilic				7.66E-01	6.44E-01	0.15	8.51E-01	0.05	1.81E-01	0.40	2.26E-01	0.32
705.3982@10.566157	705.3982	10.57	NEG	RP				1.25E-01	5.39E-02	-0.50	2.59E-01	0.34	1.49E-01	0.36	9.82E-01	-0.01
705.4386@10.783272	705.4386	10.78	NEG	RP				1.72E-02	2.31E-01	-0.30	2.19E-02	0.66	2.42E-02	0.59	8.56E-01	-0.05
705.468@10.723123	705.4680	10.72	NEG	RP				1.35E-01	8.25E-02	0.53	1.51E-01	-0.37	6.33E-01	0.14	3.54E-01	-0.26
705.5315@0.6580434	705.5315	0.66	POS	hilic				7.83E-01	8.52E-01	0.05	7.74E-01	0.08	6.22E-01	0.15	1.60E-01	0.45
705.533@12.012951	705.5330	12.01	POS	RP	PC 30:0	20 eV: 184.0734 (100), 706.5399 (45)	2	7.73E-01	3.61E-01	0.30	3.51E-01	0.26	3.36E-01	0.28	1.29E-01	0.47
705.5862@0.8850695	705.5862	0.89	POS	hilic				4.64E-01	5.06E-01	-0.19	4.13E-02	0.50	4.40E-01	0.24	7.89E-01	0.09
705.8225@4.681271	705.8225	4.68	NEG	hilic				9.97E-01	8.41E-01	0.07	6.97E-01	0.11	7.86E-01	0.08	9.22E-01	0.03
706.135@9.255317	706.1350	9.26	NEG	RP				6.16E-01	9.87E-01	0.00	9.22E-01	0.03	6.78E-02	-0.51	6.26E-01	-0.16
706.1355@8.907905	706.1355	8.91	NEG	RP				9.92E-01	9.80E-01	-0.01	9.02E-01	0.03	9.39E-01	0.02	6.32E-01	0.16
706.1358@9.146951	706.1358	9.15	NEG	RP				9.47E-01	8.53E-01	0.06	7.32E-01	0.10	7.37E-01	0.10	5.79E-01	-0.17
706.136@0.3967957	706.1360	0.40	NEG	hilic				8.84E-01	9.76E-01	0.01	9.45E-01	-0.02	2.71E-01	-0.32	7.68E-01	-0.10
706.1365@0.39666656	706.1365	0.40	NEG	hilic				6.29E-01	4.77E-01	-0.18	4.63E-02	-0.56	2.39E-01	-0.33	3.83E-01	-0.27
706.2631@9.901144	706.2631	9.90	NEG	RP				2.66E-02	2.45E-03	-0.65	2.43E-03	-0.61	8.16E-03	-0.58	7.37E-01	-0.09
706.3369@11.1872425	706.3369	11.19	NEG	RP				3.95E-01	4.36E-01	0.22	6.73E-01	0.12	1.69E-01	-0.34	1.81E-01	0.34
706.4829@0.39480177	706.4829	0.39	POS	hilic				6.82E-01	6.12E-01	0.14	5.30E-01	-0.18	3.27E-01	0.29	2.54E-01	0.31
706.4831@10.5775175	706.4831	10.58	POS	hilic				7.65E-01	7.61E-02	0.51	4.90E-01	0.23	3.73E-01	0.24	4.14E-01	0.24
706.5338@0.6603476	706.5338	0.66	POS	hilic				8.41E-01	4.69E-01	0.23	4.72E-01	0.19	7.67E-01	0.09	1.83E-01	0.41

Supplementary table 2: Metabolomics analysis results

Compound	Mass	Retention Time	Ionization mode	column	Compound_ID	MSMS_spectra	ID_level	ANOVA	Anorexia vs control		overweight vs control		obese vs control		athletes vs control	
								P-FDR	P	Estimate	P	Estimate	P	Estimate	P	Estimate
706.5437@11.066983	706.5437	11.07	POS	RP				4.13E-01	6.57E-01	-0.15	1.29E-01	0.43	1.26E-01	0.49	6.84E-01	0.11
706.58@0.51287496	706.5800	0.51	POS	hilic				4.03E-01	6.80E-02	-0.35	5.77E-01	-0.12	3.76E-01	0.22	6.65E-01	0.11
707.1389@8.907321	707.1389	8.91	NEG	RP				7.92E-01	5.66E-01	-0.18	4.46E-01	-0.20	8.74E-01	0.04	4.67E-01	0.24
707.3418@3.166067	707.3418	3.17	POS	hilic				6.60E-01	7.81E-01	-0.09	3.48E-01	0.26	5.99E-01	0.15	1.37E-01	0.44
707.3604@3.3328521	707.3604	3.33	NEG	hilic				9.38E-01	4.96E-01	-0.20	7.77E-01	0.07	7.49E-01	0.10	9.06E-01	-0.03
707.3937@10.434608	707.3937	10.43	NEG	RP				3.73E-03	4.71E-02	-0.41	4.87E-02	0.53	9.05E-03	0.60	3.81E-01	-0.22
707.3941@0.5587025	707.3941	0.56	POS	hilic				8.45E-01	4.18E-01	-0.24	3.24E-01	-0.25	8.94E-01	0.04	3.52E-01	-0.28
707.4186@10.714977	707.4186	10.71	NEG	RP				6.31E-01	6.03E-01	-0.13	1.34E-01	-0.36	1.87E-01	-0.34	1.32E-01	-0.37
707.4632@10.721701	707.4632	10.72	NEG	RP				2.78E-01	3.70E-01	0.26	5.92E-01	-0.14	2.37E-02	-0.46	8.11E-01	-0.06
707.4691@11.036188	707.4691	11.04	NEG	RP				6.04E-01	2.72E-01	0.35	4.26E-01	0.23	4.44E-02	0.58	2.13E-01	0.35
707.4888@0.39517492	707.4888	0.40	POS	hilic				6.91E-01	4.45E-01	0.20	8.59E-01	-0.04	2.34E-01	0.32	1.84E-01	0.35
707.5036@12.723822	707.5036	12.72	POS	RP				9.19E-01	6.77E-01	0.13	9.15E-01	0.03	8.16E-01	0.07	2.32E-01	0.32
707.5284@11.429047	707.5284	11.43	NEG	RP				4.46E-01	1.23E-01	0.37	1.72E-01	0.27	4.52E-02	0.51	6.90E-02	0.43
707.5462@11.065852	707.5462	11.07	POS	RP				5.96E-01	4.93E-01	-0.21	4.34E-01	0.22	2.05E-01	0.39	9.15E-01	-0.03
707.668@3.2933693	707.6680	3.29	NEG	hilic				9.88E-01	7.42E-01	0.10	8.73E-01	-0.04	6.69E-01	0.11	8.49E-01	0.05
708.0795@8.907228	708.0795	8.91	NEG	RP				4.04E-01	1.52E-01	-0.51	5.81E-02	-0.51	4.30E-01	-0.20	3.54E-02	-0.62
708.1836@0.40818423	708.1836	0.41	NEG	hilic				4.60E-01	2.55E-02	0.53	4.74E-01	0.15	6.54E-01	0.09	3.54E-01	0.22
708.277@0.57643753	708.2770	0.58	POS	hilic				2.97E-01	3.03E-01	-0.28	2.54E-01	-0.28	2.58E-03	-0.66	3.65E-01	-0.24
708.4953@1.0349617	708.4953	1.03	NEG	hilic				7.77E-01	2.16E-01	-0.33	7.99E-01	-0.06	5.86E-01	0.15	8.21E-01	-0.06
709.3523@0.50498646	709.3523	0.50	POS	hilic				5.71E-01	3.53E-01	-0.24	2.65E-01	0.30	5.84E-01	-0.13	6.49E-01	0.12
709.358@3.3433096	709.3580	3.34	NEG	hilic				8.01E-01	3.33E-01	0.25	3.73E-01	0.21	6.49E-01	0.11	1.65E-01	0.34
709.4208@10.729635	709.4208	10.73	NEG	RP				1.49E-01	2.24E-01	0.29	1.87E-01	-0.37	2.89E-02	-0.57	4.88E-01	-0.20
709.6459@3.0285556	709.6459	3.03	POS	hilic				9.18E-01	5.97E-01	0.19	6.18E-01	0.13	9.41E-01	-0.02	3.10E-01	0.28
710.0778@8.907239	710.0778	8.91	NEG	RP				7.69E-01	4.95E-01	-0.24	2.89E-01	-0.29	6.72E-01	0.11	4.00E-01	-0.26
710.0798@9.254806	710.0798	9.25	NEG	RP				9.01E-01	8.99E-01	0.04	5.78E-01	-0.14	4.24E-01	0.23	8.85E-01	-0.05
710.1759@0.40793058	710.1759	0.41	NEG	hilic				9.94E-01	7.03E-01	0.11	7.72E-01	0.08	9.77E-01	-0.01	9.99E-01	0.00
710.2573@9.904585	710.2573	9.90	NEG	RP				8.18E-01	7.27E-01	0.11	8.68E-01	-0.05	5.74E-01	-0.14	3.15E-01	0.29
710.2589@9.850267	710.2589	9.85	NEG	RP				8.42E-01	2.71E-01	-0.29	2.24E-01	-0.30	7.30E-01	-0.10	4.16E-01	-0.22
710.2929@10.503572	710.2929	10.50	NEG	RP				5.22E-01	5.54E-01	0.17	3.93E-01	-0.20	2.70E-01	-0.24	4.42E-01	0.19
710.3934@0.45568427	710.3934	0.46	POS	hilic				7.88E-01	2.18E-01	-0.22	4.26E-01	-0.15	1.77E-01	-0.24	4.91E-01	-0.14
710.4062@10.5411625	710.4062	10.54	NEG	RP				1.57E-01	8.23E-02	-0.35	1.44E-01	0.38	2.36E-01	0.31	3.50E-01	0.24
710.4727@11.036197	710.4727	11.04	NEG	RP				3.97E-01	1.57E-01	0.45	6.03E-02	0.54	3.06E-02	0.66	9.40E-02	0.50
710.5116@1.0343394	710.5116	1.03	NEG	hilic				4.37E-01	2.28E-01	0.39	6.37E-02	0.49	2.97E-02	0.64	5.76E-01	0.19
710.5119@11.577948	710.5119	11.58	NEG	RP				6.02E-01	5.01E-01	0.17	9.55E-01	-0.01	1.00E-01	0.45	2.63E-01	0.31
710.5334@0.911886	710.5334	0.91	POS	hilic				7.96E-01	3.73E-01	-0.27	5.61E-01	0.15	5.42E-01	0.19	9.64E-01	-0.02
711.264@9.846068	711.2640	9.85	NEG	RP				3.43E-03	7.68E-01	0.09	1.41E-02	-0.71	3.92E-04	-0.97	7.25E-01	0.10
711.3356@0.42005646	711.3356	0.42	POS	hilic				4.12E-02	3.20E-02	0.63	1.34E-01	0.43	1.56E-01	0.38	1.79E-01	-0.39
711.4132@10.712087	711.4132	10.71	NEG	RP				8.80E-01	5.69E-01	-0.18	7.29E-01	0.10	4.38E-01	0.23	7.85E-01	0.07
711.6457@3.0435653	711.6457	3.04	POS	hilic				9.86E-01	5.63E-01	0.19	7.39E-01	0.10	9.11E-01	0.03	9.42E-01	0.02
711.7628@15.280039	711.7628	15.28	NEG	RP				9.03E-01	6.87E-01	-0.12	7.75E-01	-0.10	7.13E-01	-0.09	2.30E-01	-0.35
712.2738@10.242375	712.2738	10.24	NEG	RP				6.36E-01	1.96E-01	-0.31	8.96E-01	-0.03	6.64E-01	-0.11	4.10E-01	0.24
712.4137@10.558982	712.4137	10.56	NEG	RP				6.41E-02	1.53E-01	-0.31	1.32E-01	0.40	4.32E-02	0.50	7.87E-01	-0.07
712.4153@0.4477418	712.4153	0.45	POS	hilic				9.01E-01	5.19E-01	-0.17	2.24E-01	-0.30	5.91E-01	-0.14	5.22E-01	-0.18
712.4159@3.9055212	712.4159	3.91	POS	RP				9.22E-01	7.35E-01	-0.09	5.56E-01	-0.14	6.21E-01	0.13	9.50E-01	0.02
712.4169@10.713195	712.4169	10.71	NEG	RP				6.38E-01	4.84E-01	0.22	8.47E-01	-0.05	7.55E-01	-0.08	1.41E-01	-0.37
712.4928@0.9416693	712.4928	0.94	POS	hilic				5.95E-01	2.98E-01	0.34	2.99E-01	0.28	1.17E-01	0.39	7.04E-01	-0.13
712.55@0.8865787	712.5500	0.89	POS	hilic				3.96E-01	3.21E-01	-0.30	7.43E-02	0.43	5.18E-01	0.20	7.63E-01	-0.10
712.9925@6.228395	712.9925	6.23	NEG	hilic				4.43E-01	6.73E-02	0.50	5.40E-02	0.48	6.82E-01	0.10	1.88E-01	0.37
712.9929@6.1864004	712.9929	6.19	POS	hilic				7.57E-01	5.08E-01	0.22	9.19E-01	0.03	4.36E-01	0.24	1.04E-01	0.45
713.2673@9.846059	713.2673	9.85	NEG	RP				3.01E-03	8.91E-01	-0.04	7.86E-03	-0.83	3.40E-04	-0.97	8.03E-01	0.07
713.2673@9.947022	713.2673	9.95	NEG	RP				1.28E-01	8.06E-01	-0.07	1.02E-01	-0.51	1.20E-02	-0.76	9.58E-01	0.02
713.3602@1.9466174	713.3602	1.95	POS	hilic				2.22E-01	2.00E-01	-0.39	4.88E-01	-0.20	3.11E-02	-0.52	8.29E-03	-0.81
713.3604@5.246413	713.3604	5.25	POS	RP				3.19E-01	6.37E-01	0.14	5.54E-01	-0.18	3.72E-02	-0.52	9.62E-02	-0.52
713.3667@0.42138898	713.3667	0.42	POS	hilic				7.12E-01	8.15E-01	0.06	3.03E-01	0.28	6.07E-01	-0.11	5.56E-01	-0.15
713.4647@11.035497	713.4647	11.04	NEG	RP				4.43E-01	1.02E-01	0.52	5.57E-02	0.58	7.64E-02	0.52	6.49E-02	0.52
713.4832@10.434146	713.4832	10.43	NEG	RP				7.37E-03	6.07E-02	-0.40	2.10E-02	0.61	2.91E-02	0.50	7.53E-01	-0.08
713.5035@0.7275374	713.5035	0.73	POS	hilic				1.45E-01	1.46E-02	0.66	5.43E-01	0.17	9.11E-01	0.03	2.23E-02	0.60
713.999@6.2309084	713.9990	6.23	NEG	hilic				4.39E-01	1.06E-01	0.50	3.35E-01	0.29	2.56E-01	0.31	2.38E-02	0.67
714.2903@10.47278	714.2903	10.47	NEG	RP				7.56E-01	4.77E-01	-0.22	4.30E-01	0.20	5.71E-01	-0.16	6.17E-01	0.16
714.2906@10.488705	714.2906	10.49	NEG	RP				3.83E-02	1.48E-03	0.87	8.41E-01	0.05	9.80E-01	-0.01	5.33E-01	0.14
714.5677@0.8802504	714.5677	0.88	POS	hilic				3.37E-01	6.46E-01	-0.13	6.19E-02	0.47	1.88E-01	0.40	7.09E-01	-0.13
715.1929@8.572504	715.1929	8.57	POS	RP				9.34E-01	3.73E-01	0.29	7.75E-01	0.09	5.92E-01	0.16	4.46E-01	0.25
715.1933@8.816255	715.1933	8.82	POS	RP				6.81E-01	8.25E-01	-0.07	8.92E-01	-0.04	1.65E-01	0.38	3.69E-01	0.31
715.1935@8.912316	715.1935	8.91	POS	RP				2.31E-01	4.58E-02	0.60	1.23E-01	0.46	2.36E-02	0.59	9.18E-01	-0.03
715.2217@10.999735	715.2217	11.00	POS	RP				6.01E-02	1.56E-01	0.42	4.02E-01	-0.28	9.81E-02	-0.45	1.12E-01	0.44
715.2836@10.239378	715.2836	10.24	NEG	RP				8.41E-03	8.15E-03	0.81	2.67E-01	-0.34	7.98E-02	-0.42	5.03E-01	0.18
715.2908@10.406219	715.2908	10.41	NEG	RP				6.40E-02	8.45E-01	0.06	6.20E-03	-0.61	2.24E-02	-0.52	8.25E-02	-0.46
715.3417@0.74457717	715.3417	0.74	POS	hilic				7.73E-01	6.90E-01	-0.14	5.73E-01	0.15	3.29E-01	0.26	5.62E-01	-0.18
715.4235@10.552946	715.4235	10.55	NEG	RP				3.90E-02	3.24E-02	-0.43	2.29E-01	0.31	5.84E-02	0.46	5.60E-01	-0.14
715.4972@10.731709	715.4972	10.73	NEG	RP				4.57E-01	2.62E-01	0.38	2.04E-01	-0.35	8.60E-01	-0.05	6.84E-01	0.13
715.5135@12.188637	715.5135	12.19	NEG	RP				1.08E-01	1.24E-02	0.70	3.64E-01	0.29	9.98E-01	0.00	7.41E-03	0.75
715.5139@0.7563616	715.5139	0.76	NEG	hilic				5.18E-02	1.06E-03	0.82	2.47E-01	0.32	7.75E-01	0.06	1.23E-02	0.68
715.5145@0.7569884	715.5145	0.76	NEG	hilic				2.86E-02	9.76E-04	0.83	2.50E-01	0.32	8.06E-01	0.06	3.70E-03	0.77

Supplementary table 2: Metabolomics analysis results

Compound	Mass	Retention Time	Ionization mode	column	Compound_ID	MSMS_spectra	ID_level	ANOVA	Anorexia vs control		overweight vs control		obese vs control		athletes vs control	
								P-FDR	P	Estimate	P	Estimate	P	Estimate	P	Estimate
715.517@0.7040495	715.5170	0.70	POS	hilic				7.27E-02	4.70E-03	0.77	4.02E-01	0.24	7.77E-01	0.07	8.78E-03	0.75
715.5178@12.182767	715.5178	12.18	POS	RP				7.21E-03	2.58E-04	1.04	1.33E-01	0.44	8.41E-01	0.06	6.67E-04	0.89
715.5499@0.6499216	715.5499	0.65	POS	hilic				7.25E-01	2.82E-01	-0.27	4.72E-01	-0.17	8.25E-01	0.06	2.04E-01	-0.29
715.5533@12.361029	715.5533	12.36	POS	RP				8.03E-02	1.37E-03	-0.68	7.03E-01	-0.10	1.04E-02	-0.54	7.75E-02	-0.38
715.8509@4.623684	715.8509	4.62	NEG	hilic				5.04E-01	5.30E-01	0.18	6.89E-02	0.59	1.22E-01	0.48	2.58E-01	0.32
716.1736@0.40195674	716.1736	0.40	NEG	hilic				7.96E-01	2.44E-01	0.34	8.14E-01	0.07	6.86E-01	-0.11	4.62E-01	0.23
716.4461@10.793046	716.4461	10.79	NEG	RP				2.76E-02	5.70E-01	0.13	7.57E-02	0.47	4.45E-04	0.84	9.83E-01	-0.01
716.5015@10.727284	716.5015	10.73	NEG	RP				6.81E-01	6.46E-01	0.15	5.66E-01	-0.13	6.99E-01	-0.09	2.15E-01	0.33
716.5475@1.1129651	716.5475	1.11	POS	hilic				4.39E-01	8.71E-01	-0.05	7.30E-02	0.47	9.99E-01	0.00	3.77E-01	-0.24
716.583@0.87745655	716.5830	0.88	POS	hilic				5.60E-01	4.49E-01	-0.22	1.02E-01	0.40	6.03E-01	0.16	8.60E-01	-0.06
717.2987@10.425816	717.2987	10.43	NEG	RP				7.77E-01	6.11E-02	0.45	3.60E-01	0.29	6.48E-01	0.13	8.15E-01	0.08
717.2993@10.499043	717.2993	10.50	NEG	RP				1.74E-03	1.45E-03	0.95	1.06E-01	-0.47	6.05E-01	-0.13	1.82E-01	0.37
717.3806@0.45102745	717.3806	0.45	POS	hilic				7.10E-01	6.92E-01	0.11	2.31E-01	-0.28	5.14E-01	0.18	7.21E-01	-0.10
717.4311@10.43417	717.4311	10.43	NEG	RP				6.20E-02	5.98E-01	-0.13	2.42E-02	0.65	2.69E-02	0.59	7.33E-02	0.45
717.4428@10.700816	717.4428	10.70	NEG	RP				2.22E-01	7.98E-02	-0.37	3.60E-01	0.24	1.78E-01	0.32	5.72E-01	-0.14
717.5286@12.617399	717.5286	12.62	NEG	RP				1.07E-01	2.41E-02	0.58	4.67E-02	0.53	3.63E-01	0.20	4.36E-03	0.75
717.5343@11.907377	717.5343	11.91	POS	RP				3.50E-01	2.02E-02	0.64	7.50E-02	0.46	2.08E-01	0.32	4.83E-01	0.17
717.5421@0.6837778	717.5421	0.68	POS	hilic				4.97E-01	1.59E-01	0.35	7.67E-02	0.49	7.83E-01	0.07	1.54E-01	0.38
717.5589@0.6572715	717.5589	0.66	POS	hilic				3.04E-01	3.40E-02	0.57	3.17E-02	0.57	4.80E-01	0.18	1.72E-01	0.31
717.5658@12.545474	717.5658	12.55	POS	RP				6.00E-01	1.19E-01	-0.42	7.79E-01	-0.08	2.73E-01	-0.27	9.81E-02	-0.45
717.5853@0.8768935	717.5853	0.88	POS	hilic				8.34E-01	6.33E-01	-0.13	4.20E-01	0.23	3.99E-01	0.25	8.90E-01	0.05
717.696@3.2277086	717.6960	3.23	NEG	hilic				7.02E-01	1.20E-01	0.49	9.52E-01	-0.02	5.44E-01	0.17	5.29E-01	0.22
717.6961@3.231207	717.6961	3.23	NEG	hilic				8.34E-01	4.65E-01	0.23	6.31E-01	-0.15	9.37E-01	-0.02	5.13E-01	0.21
718.1527@0.5481818	718.1527	0.55	POS	hilic				3.36E-01	3.65E-01	-0.26	2.24E-01	-0.28	2.98E-03	-0.62	3.50E-01	-0.24
718.158@0.40446362	718.1580	0.40	NEG	hilic				6.94E-01	2.59E-01	0.37	6.55E-01	0.13	8.78E-01	-0.04	1.16E-01	0.40
718.2095@6.0787506	718.2095	6.08	NEG	hilic				4.02E-01	2.57E-01	-0.29	6.24E-01	-0.13	2.97E-02	-0.52	7.61E-02	-0.43
718.5263@1.0127329	718.5263	1.01	NEG	hilic				3.54E-01	5.86E-02	0.62	3.95E-02	0.54	4.11E-02	0.60	4.57E-01	0.24
719.1506@9.224552	719.1506	9.22	NEG	RP				5.67E-01	8.01E-01	0.08	3.69E-02	-0.48	4.31E-01	-0.24	2.72E-01	-0.34
719.1515@9.303879	719.1515	9.30	NEG	RP				4.35E-01	7.88E-01	-0.07	2.51E-02	-0.66	2.74E-01	-0.28	4.35E-01	-0.26
719.152@9.006787	719.1520	9.01	NEG	RP				1.31E-01	8.09E-01	0.07	1.88E-01	-0.37	6.67E-02	-0.45	1.49E-01	0.41
719.2726@9.848675	719.2726	9.85	NEG	RP				6.86E-03	6.24E-01	0.15	6.38E-04	-0.85	4.09E-03	-0.76	1.32E-01	-0.41
719.3424@0.7649889	719.3424	0.76	POS	hilic				9.11E-01	6.38E-01	-0.14	5.64E-01	-0.15	5.95E-01	-0.14	6.63E-01	0.13
719.4478@10.720597	719.4478	10.72	NEG	RP				5.11E-01	8.98E-02	0.49	9.60E-01	0.01	8.38E-01	0.04	7.56E-01	-0.08
719.5518@0.6595219	719.5518	0.66	POS	hilic				8.97E-01	5.73E-01	0.18	2.81E-01	0.29	3.69E-01	0.27	2.81E-01	0.31
719.6951@3.2265406	719.6951	3.23	NEG	hilic				8.96E-01	5.67E-01	0.21	6.66E-01	-0.12	5.89E-01	-0.16	9.08E-01	0.03
719.747@15.272234	719.7470	15.27	NEG	RP				9.79E-01	5.50E-01	-0.17	5.26E-01	-0.16	8.31E-01	-0.05	7.25E-01	-0.09
720.2775@9.938124	720.2775	9.94	NEG	RP				3.75E-04	5.68E-01	-0.13	1.14E-03	0.82	5.00E-04	1.00	6.00E-01	0.12
720.2959@10.502242	720.2959	10.50	NEG	RP				1.88E-01	1.21E-01	0.54	8.65E-01	0.04	2.64E-01	-0.24	3.34E-01	-0.25
720.4981@0.39574414	720.4981	0.40	POS	hilic				6.16E-01	5.57E-01	0.16	5.49E-01	0.16	1.53E-01	0.43	5.40E-02	0.47
720.5406@11.574748	720.5406	11.57	NEG	RP	SM d32:1 (M+FA)	40 eV: 659.5118 (100), 168.0422 (52), 78.9586 (24)	2	5.04E-01	4.30E-01	0.25	8.89E-02	0.44	1.11E-01	0.44	7.98E-01	-0.09
720.5413@1.007595	720.5413	1.01	NEG	hilic				4.21E-01	3.65E-02	0.68	5.01E-02	0.52	2.00E-01	0.37	4.43E-01	0.25
720.5561@0.6592629	720.5561	0.66	POS	hilic				8.76E-01	5.81E-01	0.17	3.19E-01	0.27	5.73E-01	0.17	2.00E-01	0.37
721.287@10.252738	721.2870	10.25	NEG	RP				9.81E-01	6.29E-01	-0.14	8.03E-01	0.06	7.47E-01	-0.09	9.19E-01	-0.03
721.4@10.719423	721.4000	10.72	NEG	RP				9.83E-02	1.14E-01	0.40	5.04E-02	-0.44	2.53E-01	-0.25	9.82E-01	0.01
721.4968@10.022949	721.4968	10.02	POS	RP				1.95E-01	1.72E-01	-0.46	5.21E-01	0.21	6.88E-01	0.11	7.22E-02	-0.53
721.501@0.43682504	721.5010	0.44	POS	hilic				1.28E-01	4.42E-01	-0.21	2.57E-01	-0.27	3.48E-01	-0.22	1.18E-04	-0.74
721.5034@0.6997681	721.5034	0.70	NEG	hilic				8.87E-01	7.26E-01	-0.09	4.44E-01	0.18	9.54E-01	0.02	4.26E-01	0.22
721.505@12.0400505	721.5050	12.04	NEG	RP				7.36E-01	8.54E-01	-0.05	4.49E-01	0.21	1.56E-01	0.41	8.05E-01	0.07
721.5076@0.69323397	721.5076	0.69	POS	hilic				6.46E-01	1.03E-01	-0.34	7.61E-01	-0.07	5.29E-01	0.17	9.21E-01	0.03
721.5205@12.723054	721.5205	12.72	POS	RP				9.65E-01	9.43E-01	0.02	9.51E-01	-0.02	6.39E-01	0.13	4.25E-01	0.21
721.5449@1.0096002	721.5449	1.01	NEG	hilic				8.10E-01	2.58E-01	0.39	2.63E-01	0.29	1.89E-01	0.37	3.98E-01	0.26
721.6914@3.2317367	721.6914	3.23	NEG	hilic				9.00E-01	7.62E-01	0.10	6.47E-01	-0.15	4.26E-01	-0.21	7.41E-01	0.09
721.7449@15.2738495	721.7449	15.27	NEG	RP				8.42E-01	9.23E-01	-0.03	9.23E-01	-0.03	7.98E-01	0.07	2.17E-01	-0.30
722.0973@9.257403	722.0973	9.26	NEG	RP				5.06E-01	2.35E-02	-0.59	4.64E-01	-0.23	8.97E-01	-0.03	1.82E-01	-0.41
722.1001@8.907799	722.1001	8.91	NEG	RP				8.37E-01	5.65E-01	-0.19	3.38E-01	-0.29	7.79E-01	-0.08	2.25E-01	-0.38
722.1687@0.4057976	722.1687	0.41	NEG	hilic				8.14E-01	4.36E-01	0.23	1.76E-01	0.36	7.72E-01	0.07	2.78E-01	0.27
722.3932@10.704113	722.3932	10.70	POS	RP				1.26E-01	1.73E-01	0.40	9.89E-02	0.50	3.57E-04	0.92	2.28E-01	0.30
722.5322@11.970144	722.5322	11.97	POS	RP				9.84E-01	7.20E-01	0.09	9.76E-01	-0.01	6.14E-01	0.13	9.09E-01	-0.03
722.5347@11.7045	722.5347	11.70	POS	RP				3.97E-02	1.81E-02	-0.53	6.74E-01	-0.11	4.00E-02	0.53	3.53E-01	-0.21
722.5823@0.48657894	722.5823	0.49	POS	hilic				9.71E-01	6.01E-01	0.13	9.52E-01	0.01	4.93E-01	0.16	7.96E-01	0.06
723.2168@0.42915136	723.2168	0.43	POS	hilic				2.58E-01	4.25E-03	0.83	2.90E-01	0.27	2.23E-01	0.36	2.80E-01	0.32
723.3017@10.519268	723.3017	10.52	NEG	RP				7.72E-01	2.85E-01	0.37	1.63E-01	0.34	9.80E-01	0.01	6.71E-01	0.13
723.3022@10.482787	723.3022	10.48	NEG	RP				5.91E-01	1.31E-01	0.45	1.53E-01	0.37	9.35E-01	0.03	8.96E-01	-0.04
723.3632@0.50242615	723.3632	0.50	POS	hilic				7.93E-01	9.34E-01	0.03	3.77E-01	0.25	3.53E-01	0.28	1.51E-01	0.39
723.434@10.8670635	723.4340	10.87	NEG	RP				7.46E-01	6.49E-01	0.11	1.67E-01	0.39	4.38E-01	0.21	1.94E-01	0.36
723.5@3.8009617	723.5000	3.80	POS	hilic				1.03E-01	1.82E-02	-0.46	1.16E-01	0.42	8.42E-01	-0.05	9.60E-01	0.01
723.5037@1.357279	723.5037	1.36	POS	hilic				9.76E-01	9.64E-01	0.01	5.46E-01	-0.15	8.52E-01	-0.05	8.71E-01	0.05
723.5074@11.579311	723.5074	11.58	NEG	RP				7.58E-01	4.57E-01	0.18	8.15E-01	0.05	6.40E-01	-0.10	2.77E-01	0.29
723.5174@0.70126337	723.5174	0.70	NEG	hilic				4.36E-01	2.41E-01	-0.31	5.68E-01	-0.16	1.35E-01	0.41	8.08E-01	0.08
723.518@0.70067596	723.5180	0.70	NEG	hilic				3.33E-01	8.63E-02	-0.46	4.77E-01	0.18	2.83E-01	0.32	6.30E-01	0.16
723.519@12.381276	723.5190	12.38	NEG	RP	EtherPE 36:5e (16:1e_20:4)	20 eV: 722.5126 (100), 303.2323 (55), 436.2810 (10)	2	4.87E-02	5.83E-02	-0.61	1.81E-01	0.34	7.83E-02	0.46	4.74E-01	0.22
723.5196@0.6834714	723.5196	0.68	POS	hilic				4.72E-02	3.40E-02	-0.50	2.75E-01	0.27	1.14E-01	0.45	1.65E-01	0.40
723.52@0.71781975	723.5200	0.72	POS	hilic				2.46E-01	3.81E-02	-0.46	5.91E-01	0.14	3.24E-01	0.27	4.26E-01	0.20

Supplementary table 2: Metabolomics analysis results

Compound	Mass	Retention Time	Ionization mode	column	Compound_ID	MSMS_spectra	ID_level	ANOVA	Anorexia vs control		overweight vs control		obese vs control		athletes vs control	
								P-FDR	P	Estimate	P	Estimate	P	Estimate	P	Estimate
723.5211@12.37675	723.5211	12.38	POS	RP				1.67E-01	7.72E-02	-0.49	9.05E-01	0.03	1.09E-01	0.46	5.35E-01	0.18
723.5514@0.98470575	723.5514	0.98	NEG	hilic				9.15E-01	5.79E-01	0.16	2.44E-01	0.29	7.80E-01	0.07	7.62E-01	0.08
724.0939@9.4949665	724.0939	9.49	NEG	RP				6.00E-01	9.10E-01	-0.03	4.02E-01	-0.19	2.15E-01	0.34	8.34E-01	0.06
724.0942@8.907905	724.0942	8.91	NEG	RP				6.85E-01	3.33E-01	-0.31	1.42E-01	-0.42	4.94E-01	-0.18	1.23E-01	-0.48
724.0942@9.257403	724.0942	9.26	NEG	RP				5.19E-01	2.49E-02	-0.65	2.68E-01	-0.31	2.89E-01	-0.29	1.54E-01	-0.44
724.0943@9.147199	724.0943	9.15	NEG	RP				6.87E-01	6.83E-01	-0.13	2.25E-01	-0.36	6.49E-01	-0.12	8.54E-02	-0.49
724.5233@0.70080835	724.5233	0.70	NEG	hilic				1.89E-01	2.02E-01	-0.37	6.45E-01	0.12	1.48E-01	0.39	1.29E-01	0.44
724.5234@0.6781856	724.5234	0.68	POS	hilic				5.13E-01	3.10E-01	-0.29	5.64E-01	0.16	6.78E-01	0.12	1.51E-01	0.38
724.5301@11.428603	724.5301	11.43	NEG	RP				5.60E-01	4.08E-01	0.24	3.95E-02	0.58	1.32E-01	0.44	2.96E-01	0.32
724.5499@11.967591	724.5499	11.97	POS	RP				6.89E-01	8.11E-01	0.06	7.32E-01	0.10	2.96E-01	0.27	2.96E-01	-0.28
724.55@0.8858467	724.5500	0.89	POS	hilic				1.57E-01	1.62E-01	-0.39	4.00E-02	0.51	3.37E-01	0.31	7.78E-01	-0.09
724.5504@11.970891	724.5504	11.97	POS	RP				5.58E-01	2.96E-01	-0.32	7.59E-01	0.08	5.88E-01	0.13	1.50E-01	-0.36
725.0957@9.2587185	725.0957	9.26	NEG	RP				5.42E-01	8.95E-01	0.05	8.35E-02	-0.46	7.27E-02	-0.42	7.24E-01	-0.10
725.0969@8.907556	725.0969	8.91	NEG	RP				5.11E-01	2.19E-01	-0.42	6.40E-02	-0.53	2.44E-01	-0.31	7.05E-02	-0.57
725.0974@9.164538	725.0974	9.16	NEG	RP				6.13E-01	1.34E-01	0.43	7.65E-01	0.08	8.58E-01	0.06	4.14E-01	-0.22
725.1512@0.54924005	725.1512	0.55	POS	hilic				4.12E-01	1.58E-01	-0.38	5.39E-02	-0.44	2.34E-02	-0.53	3.12E-01	-0.27
725.3885@10.713534	725.3885	10.71	NEG	RP				9.62E-02	1.30E-02	0.68	2.88E-03	0.81	1.37E-02	0.62	3.30E-02	0.57
725.5328@0.70556176	725.5328	0.71	NEG	hilic				3.70E-01	7.57E-01	0.07	3.69E-01	0.24	3.33E-01	0.26	2.60E-02	0.67
725.5347@12.583478	725.5347	12.58	NEG	RP				2.83E-01	8.33E-01	-0.06	6.82E-01	0.12	4.87E-01	0.20	1.75E-02	0.71
725.5379@12.588198	725.5379	12.59	POS	RP				2.47E-01	3.30E-01	-0.31	7.47E-01	-0.10	4.79E-01	0.19	6.25E-02	0.55
725.7314@15.278011	725.7314	15.28	NEG	RP				4.92E-01	1.48E-01	-0.44	1.92E-01	-0.34	4.78E-02	-0.52	8.45E-01	-0.06
725.8789@4.5735407	725.8789	4.57	NEG	hilic				8.89E-01	3.26E-01	0.31	4.75E-01	0.22	9.58E-01	0.02	4.48E-01	0.23
725.8795@4.5761085	725.8795	4.58	NEG	hilic				8.35E-01	2.42E-01	0.38	4.98E-01	0.21	9.82E-01	-0.01	4.79E-01	0.21
726.3874@10.70423	726.3874	10.70	POS	RP				7.18E-02	4.42E-01	0.24	2.64E-01	0.33	3.21E-03	0.92	9.06E-01	-0.04
726.4445@11.03524	726.4445	11.04	NEG	RP				9.03E-01	7.17E-01	0.09	4.05E-01	-0.19	5.30E-01	-0.15	7.92E-01	-0.07
726.4979@11.042425	726.4979	11.04	NEG	RP				7.54E-01	8.52E-02	-0.42	8.46E-01	-0.06	6.57E-01	-0.11	4.79E-01	-0.17
726.5671@0.8656922	726.5671	0.87	POS	hilic				8.08E-04	1.04E-01	-0.45	1.42E-03	0.80	1.63E-02	0.66	3.47E-01	-0.26
726.8825@4.5715528	726.8825	4.57	NEG	hilic				8.69E-01	9.09E-01	0.04	6.84E-01	0.13	3.72E-01	-0.23	5.82E-01	0.13
727.2084@10.171963	727.2084	10.17	NEG	RP				6.27E-02	1.64E-03	-0.77	8.65E-01	0.05	2.85E-01	0.30	3.93E-01	-0.20
727.2108@10.049682	727.2108	10.05	NEG	RP				6.50E-01	1.75E-01	-0.33	3.93E-01	0.24	8.92E-01	0.04	7.19E-01	0.10
727.2112@10.176826	727.2112	10.18	NEG	RP				1.85E-01	7.49E-02	-0.47	7.50E-01	0.09	1.08E-01	0.49	6.92E-01	-0.12
727.3742@0.42574	727.3742	0.43	POS	hilic				3.11E-01	1.96E-02	-0.54	8.12E-01	-0.06	1.46E-01	-0.34	8.65E-01	0.04
727.4346@10.440541	727.4346	10.44	POS	RP				6.95E-02	9.70E-01	-0.01	6.10E-03	0.78	8.24E-01	0.06	7.42E-01	0.08
727.4607@10.425954	727.4607	10.43	NEG	RP				1.76E-02	6.87E-01	-0.10	1.18E-02	0.75	2.58E-03	0.72	7.60E-01	0.08
727.4791@10.735102	727.4791	10.74	NEG	RP				9.15E-01	8.07E-01	-0.07	7.29E-01	-0.09	4.83E-01	-0.17	6.00E-01	0.16
727.518@0.6550265	727.5180	0.66	POS	hilic				7.64E-01	7.24E-01	0.10	9.89E-01	0.00	3.20E-01	0.28	2.70E-01	0.41
727.5501@13.2170315	727.5501	13.22	NEG	RP				5.27E-01	4.47E-01	-0.21	9.64E-01	0.01	4.52E-01	-0.20	2.16E-01	0.41
727.7345@15.278554	727.7345	15.28	NEG	RP				6.05E-01	1.03E-01	-0.55	6.10E-01	-0.16	4.71E-01	-0.20	1.52E-01	-0.44
728.1747@0.39995453	728.1747	0.40	NEG	hilic				2.79E-01	7.37E-02	0.44	3.01E-01	0.29	2.47E-01	-0.28	2.75E-01	0.31
728.3918@10.569749	728.3918	10.57	NEG	RP				7.36E-02	9.49E-03	-0.65	8.05E-01	0.07	3.24E-01	0.26	1.58E-01	-0.36
728.5829@12.06618	728.5829	12.07	POS	RP	SM d36:2	10 eV: 729.5915 (100), 184.0731 (25)	2	2.57E-03	2.43E-01	-0.32	3.72E-02	0.52	8.00E-03	0.72	8.42E-02	-0.48
728.5837@0.86517084	728.5837	0.87	POS	hilic				1.53E-03	1.02E-01	-0.47	2.26E-03	0.77	2.71E-02	0.62	4.02E-01	-0.24
729.369@10.729654	729.3690	10.73	NEG	RP				9.18E-01	2.90E-01	0.25	6.51E-01	0.12	5.00E-01	0.18	3.57E-01	0.29
729.4458@10.440254	729.4458	10.44	POS	RP				3.21E-01	6.21E-01	-0.16	5.89E-02	0.59	2.05E-01	0.31	7.78E-01	0.08
729.4699@10.595203	729.4699	10.60	NEG	RP				9.61E-01	6.00E-01	-0.13	6.22E-01	0.14	9.48E-01	0.02	8.73E-01	0.04
729.5322@11.813907	729.5322	11.81	POS	RP	PC 32:2 (14:0_18:2)	10 eV: 730.5387 (100), 184.0725 (25)	2	6.10E-01	7.11E-01	0.10	9.19E-01	-0.03	3.07E-01	0.28	1.45E-01	0.50
729.5328@0.6535224	729.5328	0.65	POS	hilic				8.94E-01	7.22E-01	0.12	4.84E-01	0.20	2.96E-01	0.32	3.03E-01	0.30
729.5873@0.8645219	729.5873	0.86	POS	hilic				2.36E-03	2.62E-02	-0.60	3.47E-03	0.72	1.93E-01	0.37	3.38E-01	-0.27
730.1916@0.4000682	730.1916	0.40	NEG	hilic				7.30E-01	4.10E-01	0.22	4.70E-01	0.23	3.82E-01	-0.20	4.23E-01	0.26
730.1921@9.337606	730.1921	9.34	NEG	RP				4.69E-01	2.04E-01	-0.40	3.43E-01	-0.28	2.97E-01	-0.29	2.36E-02	-0.65
730.1933@9.577958	730.1933	9.58	NEG	RP				8.28E-01	6.32E-01	-0.13	4.92E-01	0.19	3.26E-01	0.28	7.17E-01	0.10
730.392@10.570297	730.3920	10.57	NEG	RP				2.44E-02	1.75E-02	-0.64	5.39E-01	0.18	1.18E-01	0.41	1.86E-01	-0.37
730.5346@0.6555351	730.5346	0.66	POS	hilic				7.31E-01	8.41E-01	0.06	9.14E-01	0.03	5.38E-01	0.18	1.61E-01	0.47
730.5974@0.86289716	730.5974	0.86	POS	hilic				1.01E-02	1.35E-01	-0.45	8.39E-03	0.70	6.55E-02	0.50	5.83E-01	-0.16
730.598@12.570404	730.5980	12.57	POS	RP				6.45E-02	5.81E-02	-0.49	2.47E-01	0.30	1.29E-01	0.40	3.08E-01	-0.29
731.1946@0.40155843	731.1946	0.40	NEG	hilic				7.34E-01	5.30E-01	0.19	8.20E-01	0.07	4.22E-01	-0.20	3.06E-01	0.32
731.3369@1.0928149	731.3369	1.09	POS	hilic				3.82E-01	3.14E-01	-0.32	3.48E-01	0.27	1.02E-01	0.42	8.78E-01	-0.04
731.3716@3.5975459	731.3716	3.60	NEG	hilic				2.20E-01	5.81E-01	-0.18	8.96E-01	0.03	2.61E-01	0.29	4.55E-02	-0.58
731.5479@12.09236	731.5479	12.09	POS	RP	PC 32:1	20 eV: 184.0735 (100), 732.5559 (80)	2	5.19E-01	1.63E-01	0.53	2.12E-01	0.34	4.13E-02	0.59	2.12E-01	0.35
731.5484@0.6506245	731.5484	0.65	POS	hilic				7.14E-01	1.44E-01	0.45	6.06E-01	0.15	1.68E-01	0.41	7.67E-01	0.10
731.5997@0.86438596	731.5997	0.86	POS	hilic				1.72E-02	9.20E-02	-0.50	7.36E-03	0.72	3.56E-01	0.25	7.69E-01	-0.08
731.731@15.271984	731.7310	15.27	NEG	RP				1.56E-01	7.00E-02	-0.54	2.33E-01	-0.32	9.40E-01	0.02	9.43E-03	-0.73
732.5515@0.6523276	732.5515	0.65	POS	hilic				7.12E-01	7.46E-01	0.11	1.18E-01	0.41	1.56E-01	0.42	3.20E-01	0.29
733.396@10.777041	733.3960	10.78	POS	RP				7.99E-02	3.68E-01	-0.23	1.23E-01	0.46	1.05E-02	0.68	7.37E-01	0.09
733.4193@10.572038	733.4193	10.57	NEG	RP				1.72E-01	7.69E-02	-0.44	9.48E-02	0.48	4.41E-01	0.25	9.84E-01	-0.01
733.4971@11.076	733.4971	11.08	NEG	RP				9.65E-01	3.96E-01	0.20	1.00E+00	0.00	9.90E-01	0.00	8.07E-01	0.07
733.5001@11.076035	733.5001	11.08	NEG	RP				6.36E-01	4.88E-02	-0.53	3.81E-01	-0.21	2.04E-01	-0.37	2.81E-01	-0.31
733.5637@12.591005	733.5637	12.59	POS	RP	PC 32:0 (16:0/16:0)	20 eV: 184.0736 (100), 734.5718 (90)	1	9.18E-01	8.26E-01	0.06	9.77E-01	0.01	4.74E-01	-0.20	5.87E-01	0.16
733.7284@15.271353	733.7284	15.27	NEG	RP				3.70E-02	2.71E-02	-0.64	4.47E-02	-0.56	3.15E-02	-0.53	1.23E-04	-0.96
734.1425@8.922346	734.1425	8.92	NEG	RP				7.76E-01	9.79E-01	0.01	8.69E-01	-0.05	2.61E-01	-0.30	1.70E-01	-0.38
734.5563@0.98932177	734.5563	0.99	NEG	hilic				6.65E-01	7.79E-02	0.56	5.08E-01	0.17	3.51E-01	0.28	7.65E-01	0.10
734.5567@11.757483	734.5567	11.76	NEG	RP				3.82E-01	2.68E-01	0.29	9.53E-02	-0.42	7.05E-01	-0.10	8.06E-01	-0.07
734.975@6.188642	734.9750	6.19	POS	hilic				7.09E-01	5.34E-01	-0.17	2.56E-01	-0.35	5.31E-01	0.19	6.85E-01	-0.12

Supplementary table 2: Metabolomics analysis results

Compound	Mass	Retention Time	Ionization mode	column	Compound_ID	MSMS_spectra	ID_level	ANOVA	Anorexia vs control		overweight vs control		obese vs control		athletes vs control	
								P-FDR	P	Estimate	P	Estimate	P	Estimate	P	Estimate
734.9751@6.22925	734.9751	6.23	NEG	hilic				5.24E-01	9.73E-02	0.49	8.94E-01	-0.03	7.60E-01	-0.07	9.80E-01	0.01
735.396@10.776895	735.3960	10.78	POS	RP				3.26E-02	2.59E-01	-0.31	3.89E-02	0.60	1.73E-02	0.65	9.07E-01	0.04
735.435@10.786286	735.4350	10.79	NEG	RP				7.48E-03	6.20E-02	-0.55	3.89E-02	0.65	2.08E-02	0.58	8.04E-01	0.07
735.5391@12.723449	735.5391	12.72	POS	RP				9.12E-01	8.63E-01	0.04	3.48E-01	-0.26	8.39E-01	-0.06	9.08E-01	0.03
735.7292@15.27124	735.7292	15.27	NEG	RP				8.30E-01	3.27E-01	-0.32	4.88E-01	-0.19	3.08E-01	-0.31	1.54E-01	-0.38
735.9811@6.194678	735.9811	6.19	POS	hilic				7.99E-01	5.19E-01	-0.18	2.72E-01	-0.32	8.18E-01	0.07	3.65E-01	-0.26
735.9827@6.230343	735.9827	6.23	NEG	hilic				6.07E-01	1.15E-01	0.54	4.97E-01	0.19	3.32E-01	0.27	8.31E-02	0.47
736.4541@11.041107	736.4541	11.04	NEG	RP				2.86E-01	4.59E-01	0.24	7.24E-02	0.46	1.07E-02	0.77	8.69E-02	0.50
736.5307@1.0043603	736.5307	1.00	NEG	hilic				9.70E-01	9.37E-01	0.02	4.40E-01	0.22	6.26E-01	0.15	8.47E-01	0.06
736.5309@1.005522	736.5309	1.01	NEG	hilic				6.39E-01	5.38E-01	-0.20	1.70E-01	0.38	4.55E-01	0.23	8.54E-01	0.06
736.5479@0.87252814	736.5479	0.87	POS	hilic				1.62E-01	9.47E-02	-0.42	3.62E-01	0.23	2.20E-01	0.37	3.51E-01	-0.27
737.2808@10.499505	737.2808	10.50	NEG	RP				2.71E-02	2.29E-02	0.72	9.76E-01	0.01	9.35E-01	0.02	4.48E-03	0.84
737.3478@0.8144393	737.3478	0.81	POS	hilic				9.84E-01	6.38E-01	-0.15	8.21E-01	-0.06	8.84E-01	-0.04	8.21E-01	0.08
737.4318@11.029509	737.4318	11.03	POS	RP				3.99E-01	7.65E-02	0.55	1.40E-01	0.46	3.72E-02	0.66	1.12E-01	0.48
737.499@0.73240584	737.4990	0.73	NEG	hilic				5.58E-02	6.96E-04	0.83	9.25E-01	-0.03	7.75E-01	0.07	3.17E-02	0.60
737.4993@0.6982949	737.4993	0.70	POS	hilic				1.57E-01	3.35E-02	0.55	1.39E-01	0.43	6.52E-01	-0.12	3.09E-02	0.58
737.7256@15.272423	737.7256	15.27	NEG	RP				9.82E-01	5.02E-01	-0.20	6.80E-01	-0.12	4.92E-01	-0.17	7.40E-01	-0.11
738.1428@0.3993792	738.1428	0.40	NEG	hilic				7.53E-01	3.66E-01	0.25	5.01E-01	0.21	3.62E-01	-0.23	7.82E-01	0.09
738.1961@0.40584117	738.1961	0.41	NEG	hilic				4.97E-01	6.63E-01	-0.15	2.29E-01	0.32	2.32E-01	0.30	1.63E-01	0.45
738.3446@0.55717695	738.3446	0.56	NEG	hilic				6.80E-01	1.25E-01	-0.40	7.29E-01	-0.09	7.60E-01	0.09	6.65E-01	0.12
738.4546@11.039299	738.4546	11.04	NEG	RP				1.72E-01	4.33E-01	0.25	2.48E-01	0.31	2.41E-03	0.87	6.57E-01	0.13
738.509@0.8932251	738.5090	0.89	POS	hilic				1.76E-02	1.73E-01	-0.40	1.53E-02	0.60	3.30E-02	0.62	9.36E-01	0.02
738.5251@11.650188	738.5251	11.65	NEG	RP				2.85E-01	3.71E-02	-0.44	1.31E-01	-0.32	9.43E-01	0.02	3.25E-02	-0.46
738.5422@11.934449	738.5422	11.93	NEG	RP				9.66E-02	2.60E-01	-0.31	3.58E-01	0.25	9.16E-02	0.41	1.01E-01	-0.43
738.5427@0.9980183	738.5427	1.00	NEG	hilic				9.92E-01	9.02E-01	0.04	8.37E-01	0.06	9.05E-01	-0.04	6.54E-01	0.13
739.279@10.493734	739.2790	10.49	NEG	RP				3.39E-01	5.07E-02	0.65	3.14E-01	0.24	1.79E-01	0.31	8.20E-01	0.06
739.3488@0.55870837	739.3488	0.56	NEG	hilic				7.50E-01	2.25E-01	-0.30	7.09E-01	0.10	3.38E-01	-0.25	7.58E-01	-0.08
739.497@10.58471	739.4970	10.58	NEG	RP				3.96E-01	1.39E-01	-0.42	7.66E-01	0.08	6.27E-01	0.13	2.51E-01	0.36
739.5142@12.122419	739.5142	12.12	NEG	RP	PE 36:4 (16:0_20:4)	20 eV: 738.5076 (100), 303.2334 (85), 255.2325 (18)	2	5.18E-02	8.98E-04	0.81	2.94E-02	0.66	8.28E-01	0.07	7.71E-03	0.74
739.5147@0.73406917	739.5147	0.73	NEG	hilic				1.65E-01	1.11E-02	0.67	3.53E-01	0.28	7.65E-01	-0.08	3.31E-02	0.61
739.5151@0.73406917	739.5151	0.73	NEG	hilic				1.73E-01	1.11E-02	0.67	3.51E-01	0.28	7.66E-01	-0.08	3.72E-02	0.60
739.5166@0.70778066	739.5166	0.71	POS	hilic				2.42E-01	1.04E-01	0.46	5.33E-01	0.14	9.55E-01	-0.01	3.69E-02	0.62
739.5178@12.118881	739.5178	12.12	POS	RP				8.78E-02	4.06E-03	0.77	7.56E-03	0.69	3.92E-01	0.20	9.43E-02	0.45
739.518@0.7081741	739.5180	0.71	POS	hilic				1.58E-01	1.80E-02	0.67	2.84E-01	0.24	6.56E-01	0.11	2.39E-02	0.69
739.5181@12.322731	739.5181	12.32	NEG	RP				2.03E-02	1.29E-02	0.71	8.02E-01	-0.07	2.08E-01	-0.34	6.42E-02	0.55
739.5446@0.9959114	739.5446	1.00	NEG	hilic				6.42E-01	6.05E-01	-0.15	6.73E-02	-0.49	3.41E-01	-0.26	2.18E-01	-0.33
739.5493@0.62955546	739.5493	0.63	POS	hilic				9.66E-02	3.59E-03	-0.65	1.84E-01	-0.32	1.58E-02	-0.53	9.88E-01	0.00
739.7213@15.2739105	739.7213	15.27	NEG	RP				8.81E-01	5.54E-01	-0.18	9.89E-01	0.00	4.57E-01	0.22	8.61E-01	0.05
740.1726@0.40472284	740.1726	0.40	NEG	hilic				9.95E-01	7.21E-01	0.11	6.88E-01	0.10	7.96E-01	0.07	9.34E-01	0.02
740.2695@10.1717205	740.2695	10.17	NEG	RP				5.00E-02	1.95E-02	-0.61	3.05E-01	0.29	3.69E-01	0.26	1.54E-01	-0.36
740.5187@0.7352427	740.5187	0.74	NEG	hilic				8.17E-02	3.64E-02	0.60	5.23E-01	0.18	5.26E-01	-0.17	8.63E-03	0.69
741.3646@0.55902284	741.3646	0.56	NEG	hilic				6.69E-01	2.25E-01	-0.25	7.39E-01	-0.08	3.74E-01	0.22	7.98E-01	0.06
741.4273@11.029603	741.4273	11.03	POS	RP				4.30E-01	5.98E-02	0.57	1.50E-01	0.45	2.95E-02	0.63	1.56E-01	0.45
741.4521@11.038523	741.4521	11.04	NEG	RP				2.33E-02	2.70E-02	0.60	1.04E-01	-0.26	2.44E-01	0.28	4.58E-02	0.48
741.5137@10.804115	741.5137	10.80	NEG	RP				2.18E-01	4.71E-01	-0.22	5.09E-01	0.17	1.07E-02	0.65	3.94E-01	0.26
741.5281@0.740679	741.5281	0.74	NEG	hilic				3.99E-02	4.60E-02	0.59	6.13E-01	-0.15	5.81E-01	-0.13	9.15E-03	0.69
741.5281@0.7407798	741.5281	0.74	NEG	hilic				6.50E-03	5.60E-03	0.74	4.97E-01	-0.19	3.45E-01	-0.23	7.34E-03	0.68
741.5291@12.309319	741.5291	12.31	NEG	RP				5.91E-02	3.33E-02	0.69	5.65E-01	-0.12	4.93E-01	-0.15	2.25E-01	0.28
741.5296@12.326116	741.5296	12.33	NEG	RP				2.14E-01	1.45E-01	0.43	6.77E-01	-0.11	3.00E-01	-0.27	1.47E-01	0.45
741.5639@0.63	741.5639	0.63	POS	hilic				3.61E-01	5.74E-02	-0.44	1.31E-01	-0.37	1.25E-01	-0.38	8.22E-01	0.06
741.569@12.527634	741.5690	12.53	POS	RP	EtherPC 34:3e	10 eV: 742.5769 (100), 184.0754 (8)	3	2.35E-01	3.57E-01	-0.22	3.49E-01	-0.25	2.98E-02	-0.62	4.99E-01	0.21
741.716@15.275233	741.7160	15.28	NEG	RP				2.27E-01	1.01E-02	-0.79	1.60E-01	-0.41	6.76E-01	-0.12	1.79E-01	-0.37
742.1639@0.4064183	742.1639	0.41	NEG	hilic				2.07E-01	2.42E-02	0.61	1.40E-01	0.44	2.30E-01	0.28	1.19E-02	0.81
742.4297@0.5893249	742.4297	0.59	POS	hilic				8.14E-01	5.29E-01	-0.16	2.85E-01	-0.24	7.33E-01	0.08	7.32E-01	-0.09
743.4459@10.580423	743.4459	10.58	NEG	RP				5.94E-01	9.97E-01	0.00	6.26E-01	0.15	5.83E-02	0.53	7.89E-01	0.07
743.527@11.046764	743.5270	11.05	NEG	RP				9.00E-01	5.27E-01	0.20	8.19E-01	0.06	6.15E-01	0.12	2.85E-01	0.33
743.5282@11.08529	743.5282	11.09	NEG	RP				2.70E-01	1.47E-01	0.43	4.86E-01	0.20	4.65E-01	-0.21	7.24E-02	0.53
743.5439@0.73359793	743.5439	0.73	NEG	hilic				1.41E-01	7.04E-02	0.56	8.79E-01	0.04	9.34E-01	0.02	9.03E-03	0.68
743.545@12.208323	743.5450	12.21	NEG	RP				1.65E-01	6.82E-01	0.13	9.09E-01	-0.03	1.12E-01	-0.42	5.58E-02	0.55
743.5453@0.73775566	743.5453	0.74	NEG	hilic				1.55E-01	2.74E-01	0.33	8.75E-01	0.04	7.24E-01	-0.08	5.44E-03	0.72
743.5455@12.843265	743.5455	12.84	NEG	RP				1.96E-01	2.02E-01	0.38	4.01E-01	0.26	8.14E-01	-0.06	4.52E-03	0.76
743.5474@12.005032	743.5474	12.01	POS	RP	PC 33:2 (15:0_18:2)	20 eV: 184.0737 (100), 744. 5532 (75)	2	5.22E-01	5.85E-01	0.16	9.50E-02	0.45	6.37E-02	0.57	2.36E-01	0.42
743.5552@0.6505418	743.5552	0.65	POS	hilic				7.90E-01	1.39E-01	0.37	2.60E-01	0.31	4.36E-01	0.21	2.69E-01	0.37
743.5818@12.687743	743.5818	12.69	POS	RP				1.97E-01	7.56E-02	-0.46	2.48E-01	-0.32	3.15E-01	-0.26	2.36E-01	0.34
743.5828@13.046265	743.5828	13.05	POS	RP	EtherPC 34:2e	10 eV: 744.5900 (100), 184.0709 (20)	2	5.93E-01	7.56E-02	-0.47	1.45E-01	-0.42	1.10E-01	-0.41	1.26E-01	-0.42
743.7151@15.27446	743.7151	15.27	NEG	RP				4.85E-01	8.61E-02	-0.52	1.91E-01	-0.39	6.76E-02	-0.56	3.59E-01	-0.23
744.1706@8.616302	744.1706	8.62	NEG	RP				5.04E-01	2.40E-01	-0.37	1.49E-01	-0.46	2.18E-01	-0.36	4.12E-02	-0.64
744.1714@8.60193	744.1714	8.60	NEG	RP				1.40E-01	1.51E-01	0.41	2.13E-01	0.36	7.96E-03	0.73	6.62E-01	-0.15
744.1721@8.919743	744.1721	8.92	NEG	RP				6.76E-01	6.25E-01	-0.15	8.15E-01	0.06	9.58E-01	-0.01	1.83E-01	0.42
744.1738@8.869357	744.1738	8.87	NEG	RP				5.68E-01	1.54E-01	-0.50	9.82E-01	-0.01	6.49E-01	-0.10	6.11E-01	0.15
744.1739@0.4064275	744.1739	0.41	NEG	hilic				1.27E-01	1.17E-02	0.71	9.33E-03	0.73	7.96E-02	0.42	2.89E-02	0.79
744.4525@11.038945	744.4525	11.04	NEG	RP				7.98E-01	1.15E-01	0.44	5.97E-01	0.16	2.62E-01	0.32	3.91E-01	0.25
744.5268@0.52605563	744.5268	0.53	POS	hilic				8.90E-01	8.69E-01	0.03	2.29E-01	-0.17	8.58E-01	0.04	8.50E-01	-0.04

Supplementary table 2: Metabolomics analysis results

Compound	Mass	Retention Time	Ionization mode	column	Compound_ID	MSMS_spectra	ID_level	ANOVA P-FDR	Anorexia vs control P	Anorexia vs control Estimate	overweight vs control P	overweight vs control Estimate	obese vs control P	obese vs control Estimate	athletes vs control P	athletes vs control Estimate
745.1768@0.40623435	745.1768	0.41	NEG	hilic				5.08E-01	1.63E-01	0.43	1.01E-01	0.48	5.03E-01	0.17	5.77E-02	0.56
745.4645@10.794859	745.4645	10.79	NEG	RP				3.49E-01	7.33E-01	-0.08	5.66E-02	0.53	1.36E-01	0.39	4.56E-01	0.20
745.5117@0.40622985	745.5117	0.41	POS	hilic				4.46E-01	3.61E-01	-0.23	9.22E-02	-0.40	8.13E-01	0.07	7.44E-02	-0.53
745.56@12.669731	745.5600	12.67	NEG	RP				6.45E-01	1.75E-01	0.47	4.78E-01	0.21	8.58E-01	-0.05	2.18E-01	0.34
745.5625@12.366111	745.5625	12.37	POS	RP	PC 33:1	20 eV: 184.0739 (100), 746.5674 (95)	2	3.50E-01	1.89E-02	0.72	1.79E-01	0.43	4.92E-02	0.58	1.86E-01	0.37
745.5737@0.6420304	745.5737	0.64	POS	hilic				1.17E-01	2.19E-03	0.70	9.89E-01	0.00	4.66E-01	0.15	4.97E-01	0.17
745.5983@13.369236	745.5983	13.37	POS	RP				9.43E-01	7.16E-01	0.11	5.94E-01	0.16	9.80E-01	0.01	3.61E-01	0.26
745.7128@15.271255	745.7128	15.27	NEG	RP				6.29E-01	3.30E-01	-0.30	9.92E-01	0.00	2.28E-01	-0.34	5.75E-01	0.15
745.9599@10.502685	745.9599	10.50	NEG	RP				8.82E-01	5.14E-01	0.16	6.06E-01	0.14	6.33E-01	-0.13	5.89E-01	0.14
746.3606@10.57316	746.3606	10.57	NEG	RP				5.43E-01	1.18E-01	-0.31	4.86E-01	0.18	4.26E-01	0.21	8.28E-01	0.05
746.5563@0.98406243	746.5563	0.98	NEG	hilic				1.61E-04	3.50E-01	-0.24	2.84E-03	0.69	8.34E-04	0.83	1.16E-01	-0.30
746.5566@11.645046	746.5566	11.65	NEG	RP	SM d34:2 (M+FA)	40 eV: 685.5278 (100), 168.0425 (48), 78.9607 (8)	2	3.74E-03	2.42E-02	-0.59	1.18E-01	0.43	3.57E-02	0.55	8.70E-02	-0.45
746.5655@12.364126	746.5655	12.36	POS	RP				1.46E-01	3.64E-02	0.62	8.08E-01	-0.07	7.70E-02	0.46	5.36E-01	-0.17
746.5742@0.6509726	746.5742	0.65	POS	hilic				3.79E-01	2.22E-01	0.36	4.32E-02	0.49	5.49E-01	0.16	6.65E-01	-0.10
747.35@3.552718	747.3500	3.55	NEG	hilic				2.53E-01	5.65E-01	-0.20	9.63E-01	-0.01	6.07E-01	0.14	1.08E-02	-0.66
747.4797@11.077011	747.4797	11.08	NEG	RP				2.61E-01	8.69E-01	0.05	6.26E-01	0.13	1.91E-01	0.32	1.31E-02	0.71
747.4828@11.085852	747.4828	11.09	NEG	RP				6.16E-01	1.09E-01	0.44	7.17E-01	-0.09	7.99E-01	-0.07	8.58E-01	-0.05
747.5179@0.71292263	747.5179	0.71	NEG	hilic				6.98E-01	3.11E-01	-0.29	4.30E-01	0.21	5.47E-01	0.16	5.22E-01	0.21
747.5189@0.6882899	747.5189	0.69	NEG	hilic				8.64E-03	1.51E-02	-0.69	1.51E-01	0.38	4.67E-02	0.48	1.73E-01	0.43
747.5192@12.27833	747.5192	12.28	NEG	RP	EtherPE 38:7e (16:e_22:6)	20 eV: 746.5112 (100), 327.2309 (35), 283.2437 (8), 436.2861 (5)	2	6.49E-03	1.05E-03	-0.99	4.40E-01	0.20	4.52E-01	0.21	7.32E-01	0.10
747.5197@12.278273	747.5197	12.28	NEG	RP				9.16E-02	6.95E-03	-0.71	2.39E-01	0.29	8.06E-01	0.07	9.45E-01	0.02
747.5208@12.28294	747.5208	12.28	POS	RP				3.36E-04	7.48E-05	-1.25	6.05E-01	0.13	4.94E-01	0.19	7.24E-01	0.09
747.5248@0.6760544	747.5248	0.68	POS	hilic				4.84E-01	3.39E-01	0.30	1.28E-02	0.55	1.92E-01	0.39	8.18E-01	0.06
747.5277@0.66875476	747.5277	0.67	POS	hilic				8.14E-02	6.78E-02	-0.48	6.03E-02	0.46	7.83E-01	-0.08	2.37E-01	0.35
747.5377@12.722863	747.5377	12.72	POS	RP				9.51E-01	4.59E-01	0.19	5.21E-01	0.15	7.18E-01	0.09	3.48E-01	0.22
747.7157@15.274845	747.7157	15.27	NEG	RP				8.05E-01	4.57E-01	-0.20	5.18E-01	-0.20	1.22E-01	-0.42	3.24E-01	-0.29
748.1564@0.3996494	748.1564	0.40	NEG	hilic				5.96E-01	3.42E-01	0.27	3.99E-01	0.25	2.48E-01	-0.31	9.56E-01	-0.02
748.5484@0.86321026	748.5484	0.86	POS	hilic				8.78E-06	1.49E-03	-0.77	2.74E-04	0.83	2.89E-02	0.63	1.08E-01	-0.38
748.5722@11.937529	748.5722	11.94	NEG	RP				7.35E-01	5.38E-01	0.18	9.69E-02	0.41	4.15E-01	0.25	8.82E-01	-0.05
749.3462@3.554579	749.3462	3.55	NEG	hilic				7.04E-01	6.79E-01	-0.14	9.99E-01	0.00	9.53E-01	-0.02	5.42E-02	-0.47
749.3956@0.39268228	749.3956	0.39	POS	hilic				5.69E-01	1.25E-01	0.45	7.45E-01	-0.08	2.64E-01	0.29	3.59E-01	0.22
749.5229@11.662042	749.5229	11.66	NEG	RP				7.96E-01	2.05E-01	0.35	6.56E-01	0.12	4.39E-01	0.23	1.80E-01	0.39
749.535@0.6989913	749.5350	0.70	NEG	hilic				4.80E-01	3.18E-02	-0.56	9.22E-01	-0.03	3.97E-01	-0.24	7.26E-01	0.11
749.5352@12.498271	749.5352	12.50	NEG	RP				9.87E-02	6.15E-02	-0.54	7.50E-01	0.09	1.03E-01	0.42	2.07E-01	0.37
749.5359@12.500529	749.5359	12.50	NEG	RP				5.83E-01	2.61E-01	-0.28	6.88E-01	-0.12	3.96E-01	0.23	4.12E-01	0.24
749.536@12.502086	749.5360	12.50	POS	RP				1.15E-01	1.48E-01	-0.43	4.83E-01	0.21	8.95E-02	0.50	1.40E-01	0.45
749.537@12.650344	749.5370	12.65	POS	RP				2.85E-01	8.20E-02	-0.35	9.43E-01	-0.02	1.33E-01	0.40	5.22E-01	0.17
749.538@0.6744856	749.5380	0.67	POS	hilic				2.92E-02	3.40E-02	-0.55	6.12E-02	0.42	7.10E-02	0.52	3.39E-01	0.30
749.538@0.85774076	749.5380	0.86	POS	hilic				5.95E-01	3.24E-01	0.22	5.32E-01	0.14	1.17E-01	0.44	9.35E-01	-0.02
749.5764@0.9844046	749.5764	0.98	NEG	hilic				5.51E-01	7.01E-02	0.56	1.55E-01	0.36	1.00E+00	0.00	4.06E-01	0.27
749.706@15.273979	749.7060	15.27	NEG	RP				8.01E-01	1.99E-01	-0.43	2.84E-01	-0.32	4.86E-01	-0.20	3.58E-01	-0.24
750.377@10.712494	750.3770	10.71	NEG	RP				3.96E-01	1.20E-01	-0.34	1.00E-01	-0.39	1.96E-01	-0.32	6.03E-01	0.16
750.5011@0.9611395	750.5011	0.96	POS	hilic				7.82E-02	6.13E-01	-0.11	1.24E-01	0.38	5.13E-01	0.16	1.00E-02	-0.42
750.5647@0.8618827	750.5647	0.86	POS	hilic				2.87E-04	3.03E-02	-0.54	3.68E-04	0.88	6.50E-02	0.59	3.96E-01	-0.20
750.6138@0.44762504	750.6138	0.45	POS	hilic				2.19E-01	1.21E-02	-0.43	2.57E-01	-0.22	3.55E-01	-0.21	1.21E-02	-0.43
751.214@10.034437	751.2140	10.03	NEG	RP				6.51E-01	4.05E-01	-0.20	6.53E-01	0.13	2.75E-01	-0.31	2.24E-01	-0.28
751.3676@0.47239602	751.3676	0.47	POS	hilic				6.62E-01	6.19E-02	-0.51	7.38E-01	-0.09	4.26E-01	-0.22	9.23E-01	-0.03
751.4914@11.037637	751.4914	11.04	NEG	RP				4.05E-01	9.32E-01	0.02	2.22E-01	0.29	5.25E-02	0.57	6.54E-01	0.11
751.5136@11.7595625	751.5136	11.76	POS	RP				5.76E-01	7.11E-01	-0.10	5.00E-01	0.19	1.11E-01	0.46	2.81E-01	0.33
751.5177@11.548001	751.5177	11.55	POS	RP				1.69E-02	7.79E-01	0.06	2.31E-02	-0.38	2.03E-02	0.60	9.96E-01	0.00
751.5365@11.983017	751.5365	11.98	NEG	RP				7.66E-01	4.13E-01	0.27	3.94E-01	0.25	4.24E-01	0.22	9.76E-02	0.49
751.5469@0.6847019	751.5469	0.68	NEG	hilic				1.00E-01	5.97E-02	-0.50	3.81E-01	0.23	3.32E-01	0.28	9.85E-02	0.49
751.5488@12.655541	751.5488	12.66	NEG	RP				4.38E-01	2.70E-01	-0.30	4.44E-01	0.24	3.19E-01	0.30	2.40E-01	0.36
751.5507@13.097205	751.5507	13.10	NEG	RP	EtherPE 38:5e (18:1e_20:4)	20 eV: 750.5439 (100), 303.2319 (40)	2	2.89E-02	1.22E-02	-0.76	1.92E-01	0.34	4.29E-01	0.22	4.90E-01	0.22
751.5515@0.6406151	751.5515	0.64	POS	hilic				7.01E-01	7.64E-01	0.08	6.18E-01	0.14	1.49E-01	0.48	2.62E-01	0.32
751.5516@13.105653	751.5516	13.11	POS	RP	EtherPE 38:5e (18:1e_20:4)	10 eV: 752.5575 (100), 361.2743 (22), 611.5342 (21)	2	1.06E-02	3.30E-03	-0.89	3.03E-01	0.27	3.88E-01	0.24	5.05E-01	0.21
751.7072@15.274458	751.7072	15.27	NEG	RP				9.83E-01	5.72E-01	-0.18	8.16E-01	-0.06	5.69E-01	-0.16	7.79E-01	-0.08
752.3088@1.876169	752.3088	1.88	POS	hilic				2.37E-01	7.90E-01	-0.07	3.30E-02	-0.60	5.41E-01	-0.18	5.92E-01	0.14
752.5498@0.688541	752.5498	0.69	NEG	hilic				1.76E-02	1.65E-02	-0.65	2.22E-01	0.32	3.09E-02	0.47	3.17E-01	0.31
752.5807@0.8621479	752.5807	0.86	POS	hilic				3.05E-01	5.41E-02	-0.46	4.20E-01	0.23	4.16E-01	0.22	7.09E-01	-0.09
753.2245@10.263487	753.2245	10.26	NEG	RP				5.82E-01	4.83E-01	-0.20	2.41E-01	-0.32	8.46E-01	0.06	9.44E-02	-0.44
753.3775@0.4906237	753.3775	0.49	POS	hilic				7.36E-01	9.11E-01	0.03	1.31E-01	0.41	3.96E-01	0.25	2.75E-01	0.26
753.4775@10.587288	753.4775	10.59	NEG	RP				3.49E-01	3.85E-01	-0.24	1.66E-01	0.41	1.25E-01	0.40	2.74E-01	0.32
753.4915@11.038394	753.4915	11.04	NEG	RP				1.87E-01	4.16E-01	0.27	6.02E-01	0.15	9.45E-03	0.73	6.00E-01	-0.14
753.5296@12.090765	753.5296	12.09	POS	RP				4.14E-01	7.15E-02	0.66	5.14E-01	0.18	6.38E-02	0.52	3.10E-01	0.29
753.5312@11.764119	753.5312	11.76	POS	RP	PC 34:4 (14:0_20:4)	20 eV: 184.0736 (100), 754.5364 (80)	2	4.54E-01	9.95E-01	0.00	3.19E-01	0.26	1.37E-01	0.41	6.12E-02	0.49
753.5324@0.6503223	753.5324	0.65	POS	hilic				6.36E-01	1.42E-01	0.44	5.08E-01	-0.18	7.77E-01	0.08	9.24E-01	0.03
753.5494@12.381915	753.5494	12.38	NEG	RP				7.12E-01	2.76E-01	-0.30	7.26E-01	0.10	3.42E-01	0.25	7.41E-01	0.11
753.703@15.271324	753.7030	15.27	NEG	RP				4.59E-01	2.42E-02	-0.67	4.42E-01	-0.20	6.06E-01	-0.14	4.87E-01	-0.20
754.3526@0.8039056	754.3526	0.80	POS	hilic				5.24E-01	8.25E-01	-0.07	5.48E-01	0.19	3.09E-01	-0.27	1.57E-01	-0.44
754.3762@0.80753744	754.3762	0.81	POS	hilic				5.79E-01	4.78E-01	0.18	1.91E-01	0.31	7.72E-02	0.47	1.12E-01	0.47
754.5972@0.85635054	754.5972	0.86	POS	hilic				2.44E-02	7.19E-02	-0.53	1.47E-02	0.56	2.50E-01	0.37	3.99E-01	-0.21
755.2393@10.4869995	755.2393	10.49	NEG	RP				6.46E-01	1.73E-01	0.50	3.81E-01	0.23	8.46E-01	-0.05	3.95E-01	0.22

Supplementary table 2: Metabolomics analysis results

Compound	Mass	Retention Time	Ionization mode	column	Compound_ID	MSMS_spectra	ID_level	ANOVA P-FDR	Anorexia vs control P	Anorexia vs control Estimate	overweight vs control P	overweight vs control Estimate	obese vs control P	obese vs control Estimate	athletes vs control P	athletes vs control Estimate
755.2398@10.464672	755.2398	10.46	NEG	RP				4.98E-01	1.37E-01	-0.43	4.98E-02	-0.55	6.00E-01	-0.15	1.27E-01	-0.41
755.4926@10.807008	755.4926	10.81	NEG	RP				1.38E-01	4.98E-02	0.49	2.26E-02	0.67	2.25E-03	0.86	7.51E-02	0.55
755.5474@11.4288225	755.5474	11.43	NEG	RP				4.05E-01	4.21E-01	0.22	7.38E-02	0.43	2.20E-02	0.64	1.54E-01	0.42
755.5481@0.6490701	755.5481	0.65	POS	hilic				5.93E-01	1.37E-01	0.46	5.59E-01	-0.14	4.10E-01	0.24	9.78E-01	0.01
755.5482@11.904878	755.5482	11.90	POS	RP	PC 34:3 (16:1_18:2)	20 eV: 184.0736 (100), 756.5541 (95)	2	4.25E-01	1.77E-02	-0.61	9.39E-01	-0.02	2.98E-01	-0.36	1.63E-01	-0.35
755.7045@15.272904	755.7045	15.27	NEG	RP				5.95E-01	3.93E-02	-0.59	4.67E-01	-0.22	6.45E-01	-0.14	6.55E-01	-0.13
756.1445@9.375	756.1445	9.38	NEG	RP				7.50E-01	1.64E-01	0.37	9.20E-01	0.03	1.26E-01	0.40	6.53E-01	0.15
756.1446@9.088899	756.1446	9.09	NEG	RP				9.14E-01	2.85E-01	0.35	7.58E-01	0.10	4.44E-01	0.19	6.27E-01	0.14
756.1453@9.573821	756.1453	9.57	NEG	RP				3.86E-01	6.61E-01	0.11	9.77E-01	-0.01	3.10E-02	0.67	5.71E-01	0.15
756.1456@9.289862	756.1456	9.29	NEG	RP				3.80E-01	6.96E-01	0.12	2.45E-01	0.31	3.41E-01	-0.26	2.06E-01	-0.38
756.1481@0.3964616	756.1481	0.40	NEG	hilic				2.60E-01	8.92E-02	-0.47	8.46E-01	-0.05	4.48E-02	-0.51	6.55E-02	-0.46
756.5519@0.65033096	756.5519	0.65	POS	hilic				2.08E-01	9.56E-01	-0.02	1.36E-01	-0.37	7.62E-02	-0.50	3.08E-01	0.32
756.6162@0.8551694	756.6162	0.86	POS	hilic				5.70E-02	3.35E-01	-0.21	4.78E-02	0.46	7.72E-02	0.50	4.59E-01	-0.15
756.9556@6.1995935	756.9556	6.20	POS	hilic				3.61E-01	8.36E-02	-0.49	4.63E-02	-0.62	8.62E-01	-0.05	2.04E-01	-0.39
757.2412@10.500572	757.2412	10.50	NEG	RP				4.42E-01	8.09E-02	0.57	9.41E-01	-0.02	2.39E-01	0.26	5.25E-01	0.17
757.4372@11.053328	757.4372	11.05	NEG	RP				7.24E-01	1.37E-01	0.35	9.85E-01	0.00	2.86E-01	0.28	3.58E-01	0.22
757.5073@11.083558	757.5073	11.08	NEG	RP				4.97E-01	2.37E-01	0.35	2.65E-01	-0.29	9.14E-01	0.03	4.37E-01	0.22
757.51@11.090922	757.5100	11.09	NEG	RP				6.03E-01	6.67E-02	0.53	8.38E-01	0.05	9.74E-01	0.01	4.94E-01	0.20
757.5629@0.6499322	757.5629	0.65	POS	hilic				2.31E-01	1.28E-01	-0.42	6.30E-01	0.14	5.75E-02	-0.60	2.23E-01	-0.34
757.5632@12.208438	757.5632	12.21	POS	RP	PC 34:2 (16:0_18:2)	20 eV: 184.0736 (100), 758.5721 (95)	1	3.49E-01	1.91E-01	-0.31	2.17E-01	-0.30	3.10E-01	0.28	4.36E-01	-0.20
757.5633@12.22199	757.5633	12.22	POS	RP				8.86E-01	4.60E-01	0.21	9.20E-01	-0.03	3.56E-01	0.27	5.26E-01	0.17
757.7@15.271093	757.7000	15.27	NEG	RP				3.96E-01	1.31E-01	-0.36	4.73E-01	0.18	4.28E-01	0.22	5.44E-01	-0.15
757.9651@6.233871	757.9651	6.23	NEG	hilic				2.74E-01	5.65E-02	0.63	5.15E-01	0.17	1.34E-01	0.38	1.86E-02	0.69
758.1847@0.4051897	758.1847	0.41	NEG	hilic				3.96E-01	9.03E-03	0.73	1.99E-01	0.38	6.24E-01	0.13	3.83E-01	0.29
758.2319@10.175396	758.2319	10.18	NEG	RP				9.82E-02	3.27E-01	0.22	3.22E-01	0.27	4.06E-02	-0.39	1.53E-01	-0.31
758.3129@0.54987043	758.3129	0.55	NEG	hilic				1.76E-01	7.66E-02	-0.54	2.12E-01	0.32	3.10E-01	0.32	9.75E-01	-0.01
758.3132@7.1890793	758.3132	7.19	NEG	RP				1.21E-01	5.71E-02	-0.55	2.09E-01	0.34	3.88E-01	0.25	3.90E-01	-0.24
758.5665@0.6478526	758.5665	0.65	POS	hilic				9.82E-01	9.68E-01	-0.01	7.79E-01	-0.07	6.43E-01	0.13	9.44E-01	0.02
759.4104@10.570584	759.4104	10.57	NEG	RP				2.63E-02	1.50E-02	-0.63	2.20E-01	0.35	2.12E-01	0.33	2.96E-01	-0.29
759.5789@12.699037	759.5789	12.70	POS	RP	PC 34:1	20 eV: 760.5849 (100), 184.0735 (98)	2	7.69E-01	2.54E-01	0.30	8.50E-01	-0.06	2.61E-01	0.34	6.90E-01	0.09
759.6994@15.273224	759.6994	15.27	NEG	RP				6.21E-01	2.97E-01	-0.28	5.92E-01	0.14	6.58E-01	0.13	3.74E-01	-0.23
759.8136@4.4803867	759.8136	4.48	POS	hilic				8.34E-01	5.32E-01	-0.16	7.07E-01	0.12	3.25E-01	0.29	8.37E-01	0.06
759.8731@4.5643973	759.8731	4.56	NEG	hilic				8.31E-01	3.78E-01	0.28	2.25E-01	0.37	7.27E-01	0.10	7.75E-01	0.07
760.3267@0.5589232	760.3267	0.56	NEG	hilic				5.29E-01	5.35E-02	-0.51	7.31E-01	-0.09	8.36E-01	0.07	6.40E-01	0.12
760.5679@11.849488	760.5679	11.85	NEG	RP				6.17E-01	4.61E-01	0.16	2.04E-01	0.30	4.14E-01	0.23	4.79E-01	-0.13
761.3269@3.5059571	761.3269	3.51	NEG	hilic				7.77E-01	2.75E-01	0.34	5.16E-01	0.16	3.79E-01	0.23	1.03E-01	0.39
761.3307@0.5593617	761.3307	0.56	NEG	hilic				5.61E-01	9.65E-02	-0.41	4.52E-01	-0.20	8.54E-01	0.06	4.87E-01	0.19
761.4047@0.46741003	761.4047	0.47	POS	hilic				8.18E-01	3.65E-01	0.28	8.90E-01	0.04	8.33E-01	-0.06	5.49E-01	-0.21
761.406@6.5478797	761.4060	6.55	POS	RP				8.96E-01	8.66E-01	-0.05	6.82E-01	-0.12	4.98E-01	-0.18	2.82E-01	-0.35
761.4861@10.108897	761.4861	10.11	NEG	RP				2.89E-01	4.80E-01	0.19	8.99E-02	-0.29	1.73E-01	0.33	7.37E-01	0.07
761.4997@0.7280356	761.4997	0.73	POS	hilic				6.78E-01	2.35E-01	0.32	1.09E-01	0.48	4.95E-01	0.18	2.33E-01	0.28
761.502@11.689636	761.5020	11.69	NEG	RP				9.77E-01	6.26E-01	0.10	6.64E-01	0.10	8.12E-01	-0.05	8.75E-01	0.04
761.6917@15.272764	761.6917	15.27	NEG	RP				5.75E-01	8.30E-02	-0.44	7.58E-01	0.08	8.53E-01	0.05	5.31E-01	-0.17
762.1783@0.39900884	762.1783	0.40	NEG	hilic				7.46E-01	6.99E-01	0.10	2.18E-01	0.38	3.63E-01	0.25	1.52E-01	0.42
762.344@7.946183	762.3440	7.95	NEG	RP				2.94E-01	1.16E-02	-0.57	3.08E-01	-0.25	9.49E-01	-0.02	6.45E-01	0.12
762.3724@12.721918	762.3724	12.72	POS	RP				9.02E-01	9.66E-01	-0.01	9.46E-01	-0.02	9.46E-01	0.02	2.85E-01	0.26
763.3253@3.5049474	763.3253	3.50	NEG	hilic				8.29E-01	3.37E-01	0.32	9.86E-01	0.00	3.08E-01	0.30	3.93E-01	0.21
763.3462@0.55948395	763.3462	0.56	NEG	hilic				2.03E-01	1.11E-01	-0.30	1.04E-01	-0.31	8.63E-01	0.04	2.58E-01	0.28
763.4393@10.782496	763.4393	10.78	NEG	RP				2.91E-02	4.60E-01	-0.23	2.55E-02	0.66	7.97E-03	0.72	9.57E-01	0.02
763.5145@12.039863	763.5145	12.04	NEG	RP				5.15E-01	2.63E-01	0.30	9.90E-02	0.53	8.64E-01	0.05	1.17E-01	0.45
763.5147@0.72030973	763.5147	0.72	NEG	hilic				1.50E-01	7.84E-03	0.65	7.47E-03	0.78	5.70E-01	0.17	7.78E-02	0.50
763.5148@0.7231197	763.5148	0.72	NEG	hilic				7.20E-01	3.32E-01	0.31	7.45E-02	0.49	4.36E-01	0.23	6.95E-01	0.11
763.5165@12.373845	763.5165	12.37	NEG	RP				5.73E-01	8.63E-02	0.47	1.62E-01	0.41	4.57E-01	0.20	5.52E-02	0.48
763.5171@0.7407468	763.5171	0.74	POS	hilic				8.37E-01	7.85E-01	0.07	3.08E-01	0.31	8.70E-01	-0.04	4.63E-01	0.18
763.5183@0.69065887	763.5183	0.69	POS	hilic				4.06E-01	3.13E-02	0.55	1.74E-01	0.38	9.67E-01	-0.01	2.06E-01	0.35
763.5533@0.60990644	763.5533	0.61	POS	hilic				9.13E-02	1.55E-02	-0.68	3.05E-01	0.27	7.50E-01	0.09	3.79E-01	0.26
763.5712@12.49999	763.5712	12.50	NEG	RP				8.89E-01	8.39E-01	-0.06	4.63E-01	0.21	6.39E-01	-0.15	7.40E-01	0.10
763.5726@12.854275	763.5726	12.85	NEG	RP				7.24E-01	7.30E-01	0.10	3.67E-01	0.23	4.67E-01	-0.19	3.43E-01	0.25
764.1566@9.288408	764.1566	9.29	NEG	RP				9.79E-01	4.84E-01	-0.20	6.26E-01	-0.13	6.37E-01	-0.13	5.81E-01	-0.19
764.1573@9.4239235	764.1573	9.42	NEG	RP				8.52E-01	4.35E-01	-0.26	9.00E-01	0.03	5.55E-01	0.16	6.77E-01	-0.14
764.1587@0.39732346	764.1587	0.40	NEG	hilic				5.93E-01	8.94E-01	-0.04	1.85E-01	0.40	4.47E-01	-0.19	6.59E-01	0.12
764.269@10.018933	764.2690	10.02	NEG	RP				2.96E-01	9.87E-01	0.00	3.18E-01	0.25	3.84E-02	0.58	7.18E-02	0.42
764.2694@9.853794	764.2694	9.85	NEG	RP				2.94E-02	9.37E-01	-0.02	1.39E-01	-0.39	9.01E-04	-0.73	4.08E-01	0.22
764.3718@12.72206	764.3718	12.72	POS	RP				8.95E-01	8.82E-01	0.04	8.73E-01	-0.05	3.71E-01	0.26	4.02E-01	0.22
764.4429@10.782158	764.4429	10.78	NEG	RP				5.91E-01	3.66E-01	-0.21	6.83E-01	0.11	1.65E-01	0.34	9.36E-01	-0.02
765.3218@3.5055158	765.3218	3.51	NEG	hilic				9.91E-01	8.42E-01	-0.07	9.88E-01	0.00	6.80E-01	0.12	8.55E-01	0.04
765.3799@0.48720947	765.3799	0.49	POS	hilic				3.82E-01	9.42E-01	0.02	2.72E-01	-0.30	2.31E-02	-0.65	4.95E-01	-0.21
765.4765@11.036511	765.4765	11.04	NEG	RP				6.62E-01	1.74E-01	0.48	4.11E-01	0.24	9.95E-02	0.49	4.11E-01	0.25
765.4854@11.430633	765.4854	11.43	NEG	RP				8.62E-01	4.71E-01	0.20	8.35E-01	0.05	4.81E-01	0.20	2.41E-01	0.35
765.5224@10.023305	765.5224	10.02	POS	RP				7.36E-02	6.01E-02	-0.61	5.71E-01	-0.17	2.39E-01	-0.33	8.17E-04	-0.94
765.5294@0.71746135	765.5294	0.72	NEG	hilic				4.62E-01	8.18E-02	0.51	7.67E-02	0.55	1.47E-01	0.41	2.95E-02	0.54
765.5304@12.023301	765.5304	12.02	POS	RP				8.39E-01	1.22E-01	0.43	4.67E-01	0.21	6.46E-01	0.14	6.48E-01	0.15
765.5304@12.378306	765.5304	12.38	NEG	RP				3.87E-01	1.26E-02	0.67	9.60E-02	0.50	5.60E-01	0.18	8.24E-02	0.48

Supplementary table 2: Metabolomics analysis results

								ANOVA	Anorexia vs control		overweight vs control		obese vs control		athletes vs control	
Compound	Mass	Retention Time	Ionization mode	column	Compound_ID	MSMS_spectra	ID_level	P-FDR	P	Estimate	P	Estimate	P	Estimate	P	Estimate
765.5307@12.241305	765.5307	12.24	NEG	RP				5.03E-02	7.04E-04	0.94	1.34E-01	0.42	8.55E-01	0.05	2.27E-02	0.62
765.5627@0.9509365	765.5627	0.95	NEG	hilic				9.34E-01	5.79E-01	0.16	7.53E-01	-0.08	7.52E-01	-0.10	6.33E-01	0.14
765.5695@0.61032206	765.5695	0.61	POS	hilic				6.86E-03	5.70E-04	-0.95	3.60E-01	0.24	7.36E-01	-0.10	3.08E-01	0.31
766.1041@8.812535	766.1041	8.81	NEG	RP				1.57E-01	1.69E-01	-0.41	1.84E-01	-0.33	4.36E-01	-0.21	8.16E-04	-0.79
766.1056@8.812082	766.1056	8.81	NEG	RP				8.42E-01	2.48E-01	-0.41	8.10E-01	-0.07	8.33E-01	-0.06	6.09E-01	-0.15
766.1729@9.421742	766.1729	9.42	NEG	RP				7.96E-01	5.88E-01	-0.18	7.03E-01	0.10	3.24E-01	0.29	5.44E-01	0.20
766.173@9.381445	766.1730	9.38	NEG	RP				2.71E-01	2.10E-02	-0.74	2.48E-01	-0.32	8.85E-01	0.04	2.64E-01	-0.32
766.173@9.5729065	766.1730	9.57	NEG	RP				9.82E-01	9.03E-01	0.04	9.45E-01	0.02	7.79E-01	0.09	6.93E-01	-0.14
766.1732@9.089112	766.1732	9.09	NEG	RP				7.28E-01	1.73E-01	-0.44	8.46E-01	-0.05	7.28E-01	0.09	6.61E-01	-0.14
766.1734@9.28946	766.1734	9.29	NEG	RP				8.77E-01	9.15E-01	0.03	3.77E-01	0.27	4.18E-01	0.22	8.40E-01	-0.07
766.1745@0.39745936	766.1745	0.40	NEG	hilic				6.02E-01	1.29E-01	0.43	3.87E-01	0.28	8.13E-01	-0.06	1.79E-01	0.39
766.2866@10.254543	766.2866	10.25	NEG	RP				9.63E-01	4.86E-01	0.20	4.25E-01	0.18	5.91E-01	0.12	5.03E-01	0.17
766.3719@12.721726	766.3719	12.72	POS	RP				8.74E-01	9.04E-01	-0.04	6.24E-01	0.13	5.86E-01	0.17	1.94E-01	0.34
766.3765@0.5705666	766.3765	0.57	NEG	hilic				4.67E-01	6.51E-01	-0.08	4.00E-01	0.18	8.01E-02	0.43	6.32E-01	0.11
766.5339@12.378358	766.5339	12.38	NEG	RP				6.51E-01	5.46E-02	0.45	1.58E-01	0.35	1.71E-01	0.30	3.00E-01	0.26
766.5443@0.91987103	766.5443	0.92	POS	hilic				5.87E-01	7.52E-01	0.06	3.27E-01	-0.16	2.33E-01	0.29	6.02E-01	0.12
766.5665@0.95087075	766.5665	0.95	NEG	hilic				9.20E-01	3.16E-01	0.28	9.24E-01	0.03	8.62E-01	-0.06	7.06E-01	0.12
767.1762@9.409087	767.1762	9.41	NEG	RP				4.77E-01	1.12E-01	0.41	2.70E-02	0.62	5.90E-01	0.16	5.85E-01	0.17
767.1776@0.39748704	767.1776	0.40	NEG	hilic				8.66E-01	3.53E-01	0.29	2.76E-01	0.34	6.70E-01	0.11	3.89E-01	0.28
767.3185@3.5051572	767.3185	3.51	NEG	hilic				1.08E-01	2.93E-01	0.33	6.55E-02	-0.42	1.40E-01	0.40	8.67E-01	0.04
767.4723@11.035649	767.4723	11.04	NEG	RP				5.24E-01	2.50E-01	0.38	2.17E-01	0.35	3.01E-02	0.64	4.39E-01	0.23
767.5314@1.2376896	767.5314	1.24	POS	hilic				9.48E-01	8.26E-01	0.07	5.68E-01	-0.16	6.56E-01	0.13	8.80E-01	-0.05
767.5314@1.2379284	767.5314	1.24	POS	hilic				9.95E-01	9.09E-01	0.04	7.51E-01	-0.09	8.81E-01	0.04	9.38E-01	-0.02
767.5428@0.7105045	767.5428	0.71	NEG	hilic				4.60E-01	1.14E-01	0.43	5.02E-02	0.60	2.62E-01	0.32	5.49E-02	0.53
767.5428@12.030979	767.5428	12.03	NEG	RP				6.73E-02	1.24E-01	-0.34	1.20E-02	-0.54	2.70E-02	-0.45	4.88E-01	0.19
767.5435@12.131033	767.5435	12.13	NEG	RP				6.39E-01	1.32E-01	0.46	1.37E-01	0.39	7.49E-01	0.09	2.43E-01	0.33
767.5438@0.7108897	767.5438	0.71	NEG	hilic				2.14E-01	1.66E-02	0.59	1.94E-02	0.72	2.56E-01	0.34	1.93E-02	0.64
767.5454@12.754854	767.5454	12.75	NEG	RP	PE 38:4 (18:0_20:4)	20 eV: 766.5407 (100), 303.2302 (40), 283.2651 (15)	2	3.48E-01	1.55E-01	0.37	2.52E-02	0.71	1.70E-01	0.41	6.49E-02	0.55
767.5488@11.930871	767.5488	11.93	POS	RP				3.04E-01	8.03E-01	0.08	3.31E-01	0.28	7.63E-03	0.75	4.92E-01	0.19
767.574@12.395777	767.5740	12.40	POS	RP				2.24E-01	2.12E-03	-0.71	9.30E-01	0.02	8.32E-01	-0.06	6.25E-01	-0.15
767.5827@0.61016273	767.5827	0.61	POS	hilic				6.95E-02	3.79E-03	-0.82	5.41E-01	0.18	7.46E-01	-0.09	7.00E-01	0.13
767.5841@12.615473	767.5841	12.62	POS	RP				7.98E-01	1.58E-01	-0.40	4.08E-01	-0.21	9.21E-01	0.04	7.34E-01	-0.09
768.136@9.006672	768.1360	9.01	NEG	RP				5.97E-01	1.80E-01	-0.43	5.33E-01	-0.17	6.64E-01	-0.12	5.20E-01	0.21
768.3@10.505618	768.3000	10.51	NEG	RP				8.49E-03	5.23E-03	0.89	3.24E-01	-0.28	6.82E-01	0.10	2.22E-02	0.62
768.3365@10.719278	768.3365	10.72	NEG	RP				9.39E-01	8.77E-01	-0.04	4.75E-01	-0.17	4.44E-01	-0.17	3.82E-01	-0.21
768.4762@11.035222	768.4762	11.04	NEG	RP				5.25E-01	2.85E-01	0.38	3.50E-01	0.26	3.57E-02	0.64	5.26E-01	0.19
768.5462@0.7107441	768.5462	0.71	NEG	hilic				6.28E-01	4.35E-01	0.16	1.44E-01	0.36	4.12E-01	0.15	9.25E-02	0.39
769.3804@0.43910348	769.3804	0.44	POS	hilic				7.64E-01	7.53E-01	-0.07	4.71E-01	0.18	4.35E-01	-0.19	4.89E-01	-0.19
769.4565@1.3162223	769.4565	1.32	POS	hilic				3.84E-01	2.23E-01	0.35	1.79E-01	0.36	3.05E-02	0.73	1.62E-01	0.36
769.5606@12.098888	769.5606	12.10	POS	RP	PC 35:3	20 eV: 184.0742 (100), 770.5663 (99)	2	2.85E-01	8.40E-02	0.59	3.69E-01	-0.20	4.81E-01	0.20	3.63E-01	0.22
769.5611@12.389474	769.5611	12.39	NEG	RP				4.06E-01	2.63E-01	0.32	2.33E-01	-0.35	2.64E-01	-0.26	5.87E-01	0.16
769.5965@12.826443	769.5965	12.83	POS	RP				2.30E-01	4.20E-02	-0.56	4.18E-02	-0.59	2.73E-01	-0.31	8.94E-01	0.04
769.8433@4.35607	769.8433	4.36	POS	hilic				5.23E-01	1.90E-01	0.36	1.54E-02	0.60	5.89E-01	0.16	6.15E-01	0.15
769.8435@4.3564267	769.8435	4.36	POS	hilic				5.23E-01	1.90E-01	0.36	1.54E-02	0.60	5.89E-01	0.16	6.15E-01	0.15
770.1484@9.323849	770.1484	9.32	NEG	RP				7.71E-02	4.09E-01	-0.24	1.49E-01	0.37	8.73E-01	-0.05	3.02E-02	0.74
770.1485@9.224854	770.1485	9.22	NEG	RP				9.42E-01	6.58E-01	-0.13	7.45E-01	0.09	4.53E-01	-0.20	7.18E-01	-0.11
770.149@9.006614	770.1490	9.01	NEG	RP				7.72E-01	2.89E-01	-0.34	6.53E-01	-0.12	6.64E-01	-0.12	6.03E-01	0.18
770.1493@0.39727017	770.1493	0.40	NEG	hilic				5.89E-01	4.71E-01	-0.21	1.13E-01	-0.45	1.65E-01	-0.38	8.47E-01	0.06
770.1495@0.39654043	770.1495	0.40	NEG	hilic				4.71E-01	4.70E-01	-0.21	1.02E-01	-0.47	1.52E-01	-0.40	6.87E-01	0.12
770.1506@9.540001	770.1506	9.54	NEG	RP				8.60E-01	6.37E-01	-0.14	3.56E-01	-0.26	8.80E-01	0.04	3.89E-01	-0.29
770.3354@10.717365	770.3354	10.72	NEG	RP				8.00E-01	2.97E-01	0.32	2.21E-01	0.26	7.44E-01	0.07	2.67E-01	0.27
770.6236@0.84906447	770.6236	0.85	POS	hilic				7.58E-01	8.87E-01	-0.03	4.01E-01	0.21	5.62E-01	0.13	4.50E-01	-0.14
770.6389@10.6736355	770.6389	10.67	POS	RP				8.00E-01	3.08E-01	-0.21	8.65E-01	-0.04	4.62E-01	-0.15	6.16E-01	0.12
771.1533@9.007387	771.1533	9.01	NEG	RP				1.46E-01	6.18E-02	-0.53	2.02E-01	-0.30	7.32E-02	-0.42	3.62E-01	0.27
771.3729@10.709956	771.3729	10.71	NEG	RP				5.45E-01	6.89E-01	-0.11	7.12E-01	-0.10	2.51E-01	0.30	2.23E-01	-0.29
771.3809@6.346981	771.3809	6.35	POS	RP				6.64E-01	8.23E-01	0.06	2.95E-01	0.33	6.14E-01	0.13	4.34E-01	-0.25
771.5608@11.430889	771.5608	11.43	NEG	RP				6.44E-01	7.80E-01	-0.09	8.14E-01	0.07	1.06E-01	0.47	4.38E-01	0.25
771.5764@12.904053	771.5764	12.90	NEG	RP				7.28E-01	2.66E-01	0.30	7.55E-01	-0.09	6.40E-01	-0.15	4.56E-01	0.25
771.5782@12.578603	771.5782	12.58	POS	RP				9.65E-01	9.78E-01	-0.01	4.58E-01	-0.20	6.48E-01	-0.14	9.32E-01	0.03
771.5793@12.558074	771.5793	12.56	POS	RP	PC 35:2 (17:0_18:2)	10 eV: 772.5860 (100), 184.0715 (25)	2	9.57E-01	3.70E-01	-0.24	5.62E-01	-0.14	6.14E-01	-0.13	5.38E-01	-0.18
771.5802@0.64061517	771.5802	0.64	POS	hilic				4.41E-01	6.94E-02	0.50	6.66E-01	-0.11	5.23E-01	-0.19	5.55E-01	0.21
772.5706@0.9724723	772.5706	0.97	NEG	hilic				7.20E-01	8.18E-01	-0.05	6.53E-01	0.12	4.28E-01	-0.17	1.78E-01	-0.26
772.572@11.755873	772.5720	11.76	NEG	RP				3.28E-02	5.03E-02	-0.41	4.98E-01	-0.19	3.11E-02	0.61	2.64E-01	-0.28
772.5775@12.491886	772.5775	12.49	NEG	RP				4.54E-01	1.37E-01	-0.31	3.29E-01	-0.20	7.70E-01	-0.08	3.12E-01	0.26
772.5804@12.5718775	772.5804	12.57	POS	RP				6.96E-01	3.16E-01	0.27	2.99E-01	-0.27	8.80E-01	-0.04	7.49E-01	0.10
773.4239@10.78697	773.4239	10.79	NEG	RP				6.72E-02	1.91E-02	-0.45	1.24E-01	0.43	1.48E-01	0.37	4.32E-01	0.18
773.5208@11.556375	773.5208	11.56	NEG	RP				9.31E-01	8.92E-01	-0.03	4.89E-01	-0.16	5.06E-01	-0.15	7.97E-01	0.07
773.5347@0.667905	773.5347	0.67	NEG	hilic				6.66E-01	6.95E-01	0.12	3.43E-01	0.27	7.65E-02	0.49	1.91E-01	0.42
773.5347@12.384668	773.5347	12.38	NEG	RP				1.52E-01	2.64E-02	-0.77	7.64E-01	0.08	7.28E-01	-0.10	7.13E-01	0.11
773.5386@12.39057	773.5386	12.39	POS	RP				9.27E-01	4.72E-01	-0.20	7.70E-01	0.08	9.83E-01	0.01	7.49E-01	0.09
773.5556@11.642328	773.5556	11.64	POS	RP				3.63E-01	1.39E-02	-0.59	4.33E-01	-0.23	6.39E-01	0.13	5.29E-01	-0.16
773.5925@13.0883045	773.5925	13.09	POS	RP				7.79E-01	3.46E-01	0.29	9.45E-01	0.02	5.88E-01	0.12	5.69E-01	-0.13
773.8102@4.671885	773.8102	4.67	NEG	hilic				8.34E-01	9.69E-01	0.01	4.22E-01	0.23	2.04E-01	0.33	8.90E-01	0.04

Supplementary table 2: Metabolomics analysis results

								ANOVA	Anorexia vs control		overweight vs control		obese vs control		athletes vs control	
Compound	Mass	Retention Time	Ionization mode	column	Compound_ID	MSMS_spectra	ID_level	P-FDR	P	Estimate	P	Estimate	P	Estimate	P	Estimate
774.0902@9.005657	774.0902	9.01	NEG	RP				9.14E-01	4.15E-01	-0.28	4.88E-01	-0.21	5.37E-01	-0.18	3.05E-01	-0.32
774.0917@9.304286	774.0917	9.30	NEG	RP				8.04E-01	4.94E-01	0.23	2.49E-01	0.32	1.28E-01	0.42	5.90E-01	0.14
774.0918@9.230078	774.0918	9.23	NEG	RP				6.07E-01	3.60E-01	0.32	6.27E-02	0.48	9.31E-02	0.47	6.80E-01	0.12
774.2941@1.8303846	774.2941	1.83	POS	hilic				5.00E-01	5.68E-01	-0.14	3.96E-02	-0.54	8.64E-01	-0.05	6.75E-01	-0.11
774.5676@0.8445659	774.5676	0.84	POS	hilic				2.82E-01	2.07E-01	-0.28	1.80E-01	0.37	1.75E-01	0.39	5.28E-01	0.16
774.5862@0.9718948	774.5862	0.97	NEG	hilic				9.22E-04	3.05E-01	-0.26	2.62E-04	0.95	4.29E-03	0.81	9.31E-01	-0.03
774.587@0.97236615	774.5870	0.97	NEG	hilic				8.29E-03	1.20E-01	-0.40	7.39E-03	0.72	2.72E-02	0.66	9.56E-01	0.01
774.5873@12.033049	774.5873	12.03	NEG	RP	SM d36:2 (M+FA)	20 eV: 713.5576 (100), 773.5814 (13), 168.0405 (5)	2	1.53E-02	1.52E-01	-0.38	1.05E-01	0.46	3.12E-02	0.58	1.48E-01	-0.40
775.2259@10.786145	775.2259	10.79	POS	RP				9.32E-01	6.09E-01	0.15	7.88E-01	0.09	4.92E-01	-0.18	9.54E-01	0.02
775.304@3.3326392	775.3040	3.33	NEG	hilic				4.10E-01	1.05E-01	0.53	9.41E-01	0.02	7.99E-02	0.56	4.76E-01	0.19
775.5328@0.5397778	775.5328	0.54	POS	hilic				3.44E-01	1.59E-01	0.35	7.92E-01	-0.05	5.81E-02	0.48	3.30E-01	0.21
775.5353@11.772399	775.5353	11.77	NEG	RP	PC 32:2 (14:0_18:2, M+FA)	20 eV: 714.5091 (100), 279.2304 (18), 774.5306 (10), 227.2004 (7)	2	4.20E-01	8.67E-01	0.05	9.32E-01	-0.03	4.16E-01	0.24	6.30E-02	0.62
775.5403@0.66603357	775.5403	0.67	NEG	hilic				3.99E-01	4.72E-01	-0.20	6.29E-01	0.12	4.48E-01	0.22	5.61E-02	0.52
775.5497@12.956013	775.5497	12.96	NEG	RP				2.38E-02	9.30E-04	-1.02	9.26E-01	-0.03	6.11E-01	-0.15	7.70E-01	0.08
775.5509@12.553295	775.5509	12.55	NEG	RP				3.25E-02	3.76E-01	0.21	2.81E-01	0.29	7.83E-01	0.07	1.26E-03	0.96
775.5521@12.962715	775.5521	12.96	POS	RP				1.46E-01	4.05E-02	-0.59	2.61E-01	0.30	8.94E-01	-0.04	5.11E-01	0.19
775.5671@11.898558	775.5671	11.90	POS	RP				2.96E-01	4.51E-01	0.19	5.19E-01	0.17	1.48E-01	-0.34	1.45E-01	-0.31
775.5912@0.9713477	775.5912	0.97	NEG	hilic				4.40E-01	7.78E-01	-0.07	6.36E-02	0.55	3.86E-01	0.26	2.41E-01	0.29
776.1706@0.40856844	776.1706	0.41	NEG	hilic				9.50E-01	4.34E-01	0.20	9.23E-01	-0.03	9.60E-01	-0.01	7.26E-01	-0.10
776.58@0.84898275	776.5800	0.85	POS	hilic				1.62E-01	3.12E-01	-0.22	1.66E-01	0.34	9.47E-02	0.46	6.36E-01	-0.11
776.6007@12.521658	776.6007	12.52	NEG	RP				3.64E-02	8.63E-02	-0.52	4.50E-02	0.52	1.75E-01	0.38	4.81E-01	-0.21
776.6018@0.97477967	776.6018	0.97	NEG	hilic				2.23E-01	2.99E-01	-0.19	7.41E-02	0.45	1.19E-01	0.43	3.13E-01	0.23
776.6856@7.3946056	776.6856	7.39	POS	RP				7.21E-01	1.55E-01	-0.37	9.31E-01	0.03	5.59E-01	0.17	7.88E-01	-0.08
777.0392@12.250002	777.0392	12.25	POS	RP				6.86E-01	4.55E-01	-0.21	8.53E-02	-0.50	1.66E-01	-0.34	6.33E-01	-0.15
777.3@3.3313498	777.3000	3.33	NEG	hilic				9.19E-01	5.33E-01	0.22	6.75E-01	-0.12	8.74E-01	0.05	6.02E-01	0.15
777.4564@11.041467	777.4564	11.04	NEG	RP				1.66E-01	1.19E-01	0.39	4.88E-01	0.19	6.11E-03	0.84	2.82E-01	0.29
777.5109@4.781464	777.5109	4.78	POS	hilic				2.18E-01	9.33E-01	0.03	4.79E-02	0.53	8.01E-02	0.47	3.79E-02	0.70
777.5299@11.897956	777.5299	11.90	POS	RP	PC 36:6	20 eV: 778.5314 (100), 184.0703 (13)	2	9.52E-01	7.37E-01	0.09	3.63E-01	0.24	9.68E-01	0.01	8.22E-01	0.06
777.5299@11.898999	777.5299	11.90	POS	RP				8.72E-01	3.10E-01	0.29	4.54E-01	0.22	9.17E-01	0.03	3.76E-01	0.26
777.5319@0.6303043	777.5319	0.63	POS	hilic				2.42E-01	1.99E-01	0.37	8.11E-01	-0.07	7.25E-02	0.47	2.84E-01	-0.31
777.551@12.074178	777.5510	12.07	NEG	RP				5.31E-01	2.13E-01	0.48	2.62E-01	0.30	3.40E-02	0.61	2.10E-01	0.36
777.5515@0.6640267	777.5515	0.66	NEG	hilic				8.64E-01	3.75E-01	0.30	3.80E-01	0.25	2.35E-01	0.35	2.87E-01	0.31
777.5643@13.238078	777.5643	13.24	NEG	RP				4.79E-01	2.14E-02	-0.61	8.57E-01	-0.04	5.91E-01	-0.17	9.76E-01	0.01
778.4337@0.4676987	778.4337	0.47	POS	hilic				7.88E-01	5.26E-01	0.19	4.69E-01	-0.20	7.75E-01	0.08	5.45E-01	-0.17
778.4341@6.548324	778.4341	6.55	POS	RP				9.74E-01	8.48E-01	0.05	4.79E-01	0.20	8.96E-01	-0.04	8.82E-01	0.05
778.4554@11.029226	778.4554	11.03	POS	RP				3.26E-01	1.92E-01	0.42	2.75E-02	0.66	3.32E-02	0.64	1.14E-01	0.49
778.5555@0.66154796	778.5555	0.66	NEG	hilic				7.58E-01	5.53E-01	0.19	5.08E-01	0.19	3.70E-01	0.26	1.23E-01	0.50
779.0519@12.698994	779.0519	12.70	POS	RP				6.36E-02	3.75E-01	0.27	4.64E-01	-0.19	1.13E-02	-0.61	3.08E-02	-0.56
779.152@0.54032695	779.1520	0.54	NEG	hilic				5.38E-01	8.18E-01	0.07	7.79E-01	-0.07	8.05E-01	0.06	5.38E-02	-0.42
779.2049@9.001473	779.2049	9.00	POS	RP				6.36E-01	4.91E-02	0.58	6.33E-01	0.15	3.21E-01	0.30	6.05E-01	0.16
779.205@8.918334	779.2050	8.92	POS	RP				9.69E-01	6.40E-01	0.14	5.78E-01	0.17	7.31E-01	0.09	8.65E-01	-0.06
779.2816@3.1625264	779.2816	3.16	POS	hilic				7.50E-01	8.81E-01	-0.04	2.62E-01	-0.27	4.46E-01	-0.22	5.74E-01	0.15
779.2969@3.3323593	779.2969	3.33	NEG	hilic				9.16E-01	5.40E-01	0.20	6.53E-01	-0.13	8.49E-01	0.05	6.69E-01	-0.12
779.2987@3.483881	779.2987	3.48	NEG	hilic				8.94E-01	6.22E-01	0.17	4.27E-01	-0.20	9.53E-01	0.02	7.53E-01	0.08
779.3628@13.808585	779.3628	13.81	NEG	RP				5.03E-01	6.87E-01	-0.10	4.02E-01	0.20	8.98E-02	0.50	6.12E-01	0.14
779.3951@10.710895	779.3951	10.71	NEG	RP				4.16E-01	1.79E-01	0.37	4.29E-02	0.56	1.05E-01	0.41	9.03E-01	0.04
779.5495@0.6302695	779.5495	0.63	POS	hilic				5.58E-01	5.16E-01	0.20	6.72E-01	0.10	2.33E-01	0.36	3.34E-01	-0.28
779.5509@11.89187	779.5509	11.89	POS	RP				3.75E-01	2.23E-02	-0.59	6.18E-01	-0.13	4.75E-02	-0.48	4.16E-01	-0.23
779.5661@12.555788	779.5661	12.56	NEG	RP				7.93E-01	5.10E-01	-0.18	9.62E-01	0.01	5.05E-01	-0.20	4.06E-01	0.25
780.1834@0.3990089	780.1834	0.40	NEG	hilic				8.37E-01	8.03E-02	0.42	8.52E-01	0.06	7.31E-01	0.09	6.22E-01	0.15
780.3677@0.553609	780.3677	0.55	NEG	hilic				3.40E-01	1.99E-01	-0.34	1.93E-01	0.33	2.53E-01	0.34	6.54E-01	0.12
780.3909@4.747407	780.3909	4.75	POS	hilic				9.37E-01	8.59E-01	0.06	7.44E-01	0.09	6.41E-01	0.14	3.45E-01	0.30
780.4534@11.029246	780.4534	11.03	POS	RP				7.09E-01	1.61E-01	0.43	1.44E-01	0.37	4.54E-01	0.22	1.46E-01	0.37
780.5502@0.63017577	780.5502	0.63	POS	hilic				1.73E-01	1.23E-03	0.78	5.21E-02	0.48	5.64E-02	0.56	1.67E-01	0.32
780.5692@12.558718	780.5692	12.56	NEG	RP				8.84E-01	5.57E-01	-0.17	6.58E-01	0.12	5.50E-01	-0.17	6.73E-01	0.13
781.2456@9.8463125	781.2456	9.85	NEG	RP				8.65E-01	4.52E-01	0.22	7.69E-01	-0.07	9.91E-01	0.00	4.27E-01	0.21
781.2738@3.1614652	781.2738	3.16	POS	hilic				9.15E-01	6.46E-01	-0.12	3.49E-01	-0.24	3.40E-01	-0.26	8.01E-01	-0.07
781.2917@3.3326316	781.2917	3.33	NEG	hilic				9.15E-01	3.91E-01	0.23	8.16E-01	-0.07	5.46E-01	0.21	8.92E-01	0.04
781.2972@3.483583	781.2972	3.48	NEG	hilic				5.22E-01	8.28E-01	0.07	8.63E-01	-0.04	1.15E-01	0.39	1.03E-01	0.41
781.3792@14.659959	781.3792	14.66	NEG	RP				6.69E-02	2.54E-01	0.29	2.74E-03	0.74	5.96E-03	0.86	1.40E-01	0.39
781.3848@0.51577634	781.3848	0.52	POS	hilic				7.38E-01	6.31E-01	-0.12	5.39E-01	0.16	1.82E-01	0.35	7.97E-01	0.07
781.3983@10.787223	781.3983	10.79	NEG	RP				6.11E-01	2.26E-01	0.27	8.90E-01	-0.03	9.62E-01	0.01	1.75E-01	0.34
781.4521@11.041739	781.4521	11.04	NEG	RP				5.15E-01	4.84E-02	0.52	4.44E-01	0.25	2.46E-01	0.32	2.94E-02	0.60
781.5624@12.004386	781.5624	12.00	POS	RP	PC 36:4 (18:2_18:2)	10 eV: 782.5702 (100), 184.0734 (13)	2	9.72E-01	8.81E-01	-0.04	8.59E-01	0.05	7.53E-01	0.10	4.45E-01	0.19
781.564@12.14552	781.5640	12.15	POS	RP	PC 36:4 (16:0_20:4)	20 eV: 782.5682 (100), 184.0726 (82)	2	8.18E-01	9.65E-01	-0.02	3.90E-01	0.25	9.75E-01	-0.01	2.71E-01	0.32
781.5667@0.6302136	781.5667	0.63	POS	hilic				7.35E-01	2.11E-01	-0.36	1.00E+00	0.00	4.22E-01	-0.21	7.45E-01	0.10
781.959@10.0014715	781.9590	10.00	POS	RP				7.32E-04	1.87E-03	0.96	2.39E-01	-0.29	5.18E-01	-0.17	4.34E-03	0.74
782.3837@0.5587544	782.3837	0.56	NEG	hilic				4.79E-01	3.18E-01	-0.22	4.70E-01	0.17	2.22E-01	0.33	3.03E-01	0.26
782.4492@11.029388	782.4492	11.03	POS	RP				3.33E-01	2.82E-01	0.37	1.05E-01	0.45	1.38E-02	0.77	2.56E-01	0.33
782.6277@0.84443545	782.6277	0.84	POS	hilic				8.34E-01	4.70E-01	-0.18	9.33E-01	-0.03	3.56E-01	-0.24	5.67E-01	0.15
783.1629@9.368181	783.1629	9.37	NEG	RP				9.46E-01	6.86E-01	-0.14	9.67E-01	-0.01	6.80E-01	0.11	6.25E-01	0.16
783.1638@9.088399	783.1638	9.09	NEG	RP				7.29E-01	1.43E-01	0.50	3.67E-01	0.25	4.38E-01	0.25	1.75E-01	0.36
783.1638@9.591468	783.1638	9.59	NEG	RP				3.01E-01	8.53E-02	0.53	2.36E-02	0.58	3.06E-02	0.61	3.70E-02	0.67

Supplementary table 2: Metabolomics analysis results

								ANOVA	Anorexia vs control		overweight vs control		obese vs control		athletes vs control	
Compound	Mass	Retention Time	Ionization mode	column	Compound_ID	MSMS_spectra	ID_level	P-FDR	P	Estimate	P	Estimate	P	Estimate	P	Estimate
783.1654@9.303253	783.1654	9.30	NEG	RP				7.88E-01	9.24E-01	0.03	3.03E-01	0.31	4.49E-01	0.20	2.08E-01	0.39
783.2916@3.3379662	783.2916	3.34	NEG	hilic				7.21E-01	1.82E-01	0.35	7.90E-01	-0.07	3.16E-01	0.28	9.80E-01	0.01
783.4503@11.029292	783.4503	11.03	POS	RP				7.30E-01	4.63E-01	0.22	1.34E-01	0.36	1.54E-01	0.39	7.25E-01	0.09
783.5785@0.63000864	783.5785	0.63	POS	hilic				9.12E-01	5.63E-01	0.18	9.80E-01	0.01	2.68E-01	0.26	9.40E-01	0.02
783.5791@12.397228	783.5791	12.40	POS	RP	PC 36:3	20 eV: 784.5848 (100), 184.0733 (88)	2	7.10E-01	2.50E-01	0.33	6.13E-01	-0.15	3.06E-01	0.27	8.63E-01	0.06
783.8367@4.6197557	783.8367	4.62	NEG	hilic				6.48E-01	7.14E-01	0.12	4.54E-01	-0.24	6.04E-01	-0.15	2.43E-01	0.31
784.2438@10.261026	784.2438	10.26	NEG	RP				6.48E-01	6.86E-01	0.10	2.57E-01	0.29	4.74E-02	0.48	3.06E-01	0.30
784.6444@0.8283799	784.6444	0.83	POS	hilic				1.50E-02	3.13E-01	-0.31	4.34E-02	0.55	2.58E-01	0.29	2.13E-02	-0.59
784.6451@0.8330181	784.6451	0.83	POS	hilic				3.95E-01	8.72E-02	-0.53	7.32E-01	0.10	9.00E-01	0.04	1.51E-01	-0.38
785.0251@12.238403	785.0251	12.24	POS	RP				8.87E-01	4.89E-01	-0.20	1.62E-01	-0.35	6.67E-01	-0.11	6.65E-01	-0.13
785.3586@1.9222189	785.3586	1.92	POS	hilic				2.41E-03	4.28E-03	0.88	3.56E-01	-0.17	8.77E-01	-0.03	2.33E-02	0.55
785.4313@11.054503	785.4313	11.05	NEG	RP				3.10E-02	2.42E-02	0.71	2.97E-02	0.69	3.51E-03	0.82	2.74E-04	1.01
785.4404@13.8098345	785.4404	13.81	NEG	RP				3.91E-01	2.87E-01	-0.28	5.69E-01	0.13	1.29E-01	0.46	7.09E-01	0.11
785.4967@0.5539846	785.4967	0.55	POS	hilic				4.70E-01	4.31E-02	0.58	7.14E-01	0.08	6.05E-01	0.13	7.06E-01	0.10
785.4977@0.747194	785.4977	0.75	POS	hilic				8.47E-01	5.43E-01	0.16	2.83E-01	0.29	8.85E-01	-0.03	4.84E-01	0.17
785.5267@12.233003	785.5267	12.23	POS	RP				6.93E-01	7.28E-01	-0.10	2.48E-01	-0.30	1.21E-01	-0.47	9.41E-01	-0.02
785.5931@0.6334696	785.5931	0.63	POS	hilic				9.45E-01	5.31E-01	-0.16	6.84E-01	0.10	7.12E-01	0.09	8.94E-01	0.04
785.5941@12.931573	785.5941	12.93	POS	RP	PC 36:2 (18:1_18:1)	10 eV: 786.6013 (100), 184.0727 (15)	2	6.34E-01	4.93E-01	0.15	4.68E-01	0.21	1.43E-01	0.47	7.50E-01	-0.10
786.1079@9.224839	786.1079	9.22	NEG	RP				6.36E-01	2.27E-01	-0.39	1.43E-01	-0.44	3.01E-01	-0.27	9.75E-02	-0.52
786.1112@9.323651	786.1112	9.32	NEG	RP				4.43E-01	1.78E-01	-0.46	2.83E-01	-0.32	4.19E-01	-0.21	1.63E-02	-0.67
786.1132@9.00635	786.1132	9.01	NEG	RP				6.82E-01	3.02E-01	-0.35	2.45E-01	-0.34	5.98E-01	-0.14	9.27E-02	-0.51
786.1792@0.4048058	786.1792	0.40	NEG	hilic				4.83E-01	3.82E-01	-0.23	6.89E-01	0.12	1.31E-01	-0.43	1.39E-01	-0.40
786.2584@10.499419	786.2584	10.50	NEG	RP				5.84E-01	1.12E-01	0.55	9.06E-01	0.03	8.49E-01	0.05	4.34E-01	0.23
787.4744@10.25748	787.4744	10.26	POS	RP				5.51E-01	3.87E-01	0.26	6.92E-01	0.12	1.28E-01	-0.34	5.35E-01	0.17
787.5727@12.50593	787.5727	12.51	NEG	RP	EtherPC 34:3e (16:1e_18:2, M+FA)	20 eV: 726.5426 (100), 786.5625 (32), 279.2327 (14)	2	6.94E-03	2.82E-02	-0.62	8.57E-02	-0.45	4.81E-03	-0.85	2.19E-01	0.33
787.6091@13.671175	787.6091	13.67	POS	RP	PC 36:1 (18:0_18:1)	10 eV: 788.6174 (100), 184.0767 (12)	1	8.00E-01	5.44E-01	0.19	7.08E-01	-0.11	2.98E-01	0.25	6.77E-01	-0.11
788.1065@9.323385	788.1065	9.32	NEG	RP				4.27E-01	2.19E-01	-0.41	2.91E-01	-0.31	4.49E-01	-0.20	1.27E-02	-0.70
788.1068@9.006443	788.1068	9.01	NEG	RP				7.15E-01	2.66E-01	-0.36	1.98E-01	-0.37	4.41E-01	-0.20	1.40E-01	-0.48
788.1071@9.535311	788.1071	9.54	NEG	RP				5.57E-01	1.70E-01	-0.42	2.25E-01	-0.32	7.51E-01	0.10	1.85E-01	-0.38
788.1076@9.228061	788.1076	9.23	NEG	RP				7.18E-01	1.67E-01	-0.40	2.02E-01	-0.41	7.46E-01	-0.08	2.55E-01	-0.33
788.1815@0.40339798	788.1815	0.40	NEG	hilic				8.31E-01	5.05E-01	-0.19	2.89E-01	-0.32	2.10E-01	-0.34	2.47E-01	-0.33
788.5304@1.0224177	788.5304	1.02	NEG	hilic				1.08E-01	4.41E-03	0.83	4.10E-01	0.21	7.07E-01	-0.11	6.85E-01	0.12
788.5309@11.576099	788.5309	11.58	NEG	RP				7.72E-01	4.18E-01	0.23	4.35E-01	-0.20	8.02E-01	0.07	6.25E-01	-0.13
789.1086@9.322257	789.1086	9.32	NEG	RP				5.65E-01	3.71E-01	-0.28	4.85E-01	-0.23	6.74E-01	-0.10	2.64E-02	-0.61
789.1091@9.536206	789.1091	9.54	NEG	RP				1.01E-01	1.50E-01	-0.43	2.68E-01	-0.30	3.53E-01	0.26	1.58E-02	-0.69
789.1095@9.006264	789.1095	9.01	NEG	RP				8.27E-01	3.89E-01	-0.30	2.39E-01	-0.32	4.73E-01	-0.18	2.12E-01	-0.39
789.1095@9.225284	789.1095	9.23	NEG	RP				2.93E-01	4.69E-02	-0.60	6.93E-02	-0.55	3.68E-01	-0.25	2.70E-02	-0.68
789.2421@11.207466	789.2421	11.21	POS	RP				4.46E-01	2.10E-01	0.39	6.25E-01	-0.15	2.89E-01	-0.31	5.27E-01	0.19
789.2729@10.267561	789.2729	10.27	NEG	RP				9.64E-01	7.16E-01	0.10	5.26E-01	0.17	9.03E-01	-0.03	8.74E-01	-0.04
789.3911@13.8083	789.3911	13.81	NEG	RP				5.67E-01	8.08E-01	0.07	1.97E-01	0.33	7.93E-02	0.57	5.79E-01	0.18
789.4441@0.9136225	789.4441	0.91	POS	hilic				3.77E-01	6.29E-01	-0.10	4.66E-01	0.18	1.10E-01	-0.29	2.45E-01	0.32
789.5502@11.979068	789.5502	11.98	NEG	RP				7.31E-01	4.17E-01	0.24	2.68E-01	0.31	2.24E-01	0.38	1.80E-01	0.48
789.5569@0.69684786	789.5569	0.70	NEG	hilic				5.87E-01	4.09E-01	-0.18	1.87E-01	0.33	8.76E-01	0.03	5.30E-01	0.16
789.5625@0.9322633	789.5625	0.93	NEG	hilic				1.80E-01	2.15E-01	-0.38	1.20E-01	0.40	3.07E-01	0.32	3.37E-01	-0.25
789.563@12.61293	789.5630	12.61	POS	RP				5.46E-03	1.78E-06	-1.21	5.14E-01	-0.20	3.40E-01	-0.23	8.04E-01	-0.07
789.564@12.336839	789.5640	12.34	POS	RP				3.62E-01	2.68E-02	-0.65	9.41E-01	0.02	9.56E-01	0.02	7.84E-01	-0.07
789.5694@0.6096178	789.5694	0.61	POS	hilic				6.26E-02	3.69E-03	-0.85	5.54E-01	0.17	4.04E-01	-0.26	8.53E-01	0.05
789.5836@12.655977	789.5836	12.66	NEG	RP				5.47E-01	1.08E-01	-0.37	8.06E-01	0.06	8.57E-01	0.04	5.21E-01	0.17
789.5844@12.656885	789.5844	12.66	NEG	RP				2.61E-01	5.75E-02	-0.47	3.80E-01	0.22	4.32E-01	0.19	8.14E-01	-0.07
789.5869@13.038843	789.5869	13.04	NEG	RP				2.59E-01	1.98E-01	0.33	6.83E-01	0.11	7.44E-01	-0.07	2.85E-02	0.58
790.5679@12.333958	790.5679	12.33	POS	RP				6.45E-03	5.59E-05	-1.15	5.70E-01	-0.15	9.34E-01	-0.02	3.44E-01	-0.23
791.2891@10.513236	791.2891	10.51	NEG	RP				2.22E-01	1.88E-01	0.41	9.67E-02	-0.39	4.86E-01	0.19	4.68E-01	0.21
791.4063@14.65951	791.4063	14.66	NEG	RP				1.04E-01	1.55E-01	0.35	3.41E-03	0.72	1.04E-02	0.80	1.62E-01	0.37
791.4547@0.9147631	791.4547	0.91	POS	hilic				4.62E-01	4.62E-01	0.19	1.01E-01	0.42	6.21E-01	-0.12	2.12E-01	0.37
791.5241@10.410375	791.5241	10.41	NEG	RP				5.12E-02	1.64E-01	0.42	1.68E-01	-0.37	2.50E-02	-0.56	7.97E-01	0.07
791.5248@10.659996	791.5248	10.66	NEG	RP				5.45E-03	1.64E-03	0.91	2.79E-01	-0.32	1.44E-01	-0.35	6.61E-01	0.12
791.525@11.337763	791.5250	11.34	NEG	RP				4.21E-01	2.19E-02	0.59	9.08E-01	-0.03	2.02E-01	0.35	9.75E-01	0.01
791.5251@10.35127	791.5251	10.35	NEG	RP				9.87E-02	5.83E-01	0.16	2.55E-02	-0.56	2.32E-01	-0.28	3.16E-02	-0.53
791.5252@10.959309	791.5252	10.96	NEG	RP				2.66E-03	2.43E-03	0.92	6.58E-02	-0.46	2.45E-01	-0.26	6.30E-01	-0.14
791.546@0.69949996	791.5460	0.70	NEG	hilic				4.60E-01	2.76E-01	0.23	5.94E-02	0.44	2.16E-02	0.46	1.98E-01	0.32
791.5465@12.636901	791.5465	12.64	NEG	RP				7.43E-02	8.39E-01	-0.04	1.64E-02	0.65	1.87E-02	0.54	1.63E-01	0.32
791.5491@11.870867	791.5491	11.87	POS	RP				6.36E-01	7.22E-02	-0.45	8.73E-01	-0.04	7.72E-01	0.08	6.13E-01	-0.14
791.564@12.345137	791.5640	12.35	NEG	RP				1.06E-01	5.45E-03	0.83	8.34E-03	0.78	2.85E-02	0.59	2.51E-01	0.34
791.5779@0.9307582	791.5779	0.93	NEG	hilic				1.76E-02	3.63E-01	-0.28	6.51E-03	0.68	7.33E-02	0.54	3.84E-01	-0.25
791.5834@0.6097022	791.5834	0.61	POS	hilic				6.65E-02	2.46E-03	-0.82	4.78E-01	0.19	7.80E-01	-0.08	9.94E-01	0.00
791.6013@13.27963	791.6013	13.28	NEG	RP				6.48E-01	5.06E-01	-0.18	6.87E-01	-0.12	7.27E-02	-0.54	2.36E-01	-0.34
791.62@0.39717028	791.6200	0.40	POS	hilic				7.91E-01	9.02E-01	0.03	7.70E-01	-0.06	4.39E-01	0.18	2.65E-01	0.27
791.6311@3.2929606	791.6311	3.29	NEG	hilic				5.63E-01	4.25E-01	0.27	7.47E-01	0.08	2.75E-01	0.29	3.77E-02	0.58
792.1849@0.39859077	792.1849	0.40	NEG	hilic				8.96E-01	3.50E-01	0.26	5.88E-01	0.16	8.91E-01	-0.04	4.47E-01	0.24
792.4599@0.8988846	792.4599	0.90	POS	hilic				9.76E-01	8.95E-01	0.04	7.15E-01	-0.10	9.12E-01	-0.03	6.67E-01	0.13
792.5828@0.9317041	792.5828	0.93	NEG	hilic				6.56E-01	4.06E-01	-0.22	6.69E-01	0.11	8.00E-01	0.07	1.02E-01	-0.33
793.3741@10.714494	793.3741	10.71	NEG	RP				8.87E-01	3.52E-01	-0.25	3.21E-01	-0.27	3.95E-01	-0.24	3.42E-01	-0.25
793.3789@0.50082415	793.3789	0.50	POS	hilic				7.75E-01	3.65E-01	-0.28	8.14E-01	0.06	2.82E-01	-0.27	8.57E-01	0.05

Supplementary table 2: Metabolomics analysis results

Compound	Mass	Retention Time	Ionization mode	column	Compound_ID	MSMS_spectra	ID_level	ANOVA	Anorexia vs control		overweight vs control		obese vs control		athletes vs control	
								P-FDR	P	Estimate	P	Estimate	P	Estimate	P	Estimate
793.5365@0.70027953	793.5365	0.70	NEG	hilic				3.79E-01	6.47E-01	0.14	5.98E-01	-0.15	1.95E-01	-0.34	1.89E-01	0.41
793.5389@3.9155352	793.5389	3.92	POS	hilic				1.18E-02	4.16E-01	-0.25	1.25E-02	0.64	5.28E-03	0.76	1.47E-02	0.70
793.5399@12.263858	793.5399	12.26	NEG	RP				6.85E-01	2.37E-01	0.33	8.20E-01	0.06	9.27E-02	0.46	2.74E-01	0.36
793.5418@4.1611366	793.5418	4.16	POS	hilic				1.07E-03	5.96E-03	-0.77	5.69E-02	0.51	4.14E-02	0.56	6.29E-02	0.46
793.5614@12.114059	793.5614	12.11	POS	RP				8.66E-01	3.19E-01	0.31	4.08E-01	0.28	2.09E-01	0.33	3.30E-01	0.29
793.5616@12.115961	793.5616	12.12	POS	RP				8.27E-01	5.35E-01	0.19	4.52E-01	0.24	7.88E-02	0.42	3.63E-01	0.28
793.5793@10.276025	793.5793	10.28	NEG	RP				2.42E-01	1.01E-01	0.45	8.49E-01	0.04	1.60E-01	-0.27	9.89E-01	0.00
793.5913@0.93128693	793.5913	0.93	NEG	hilic				1.94E-01	2.25E-01	-0.38	1.33E-01	0.39	3.39E-01	0.27	3.59E-01	-0.22
793.5973@12.724272	793.5973	12.72	POS	RP	EtherPC 38:5e	10 eV: 794.6065 (100), 184.0744 (8)	2	1.90E-01	1.16E-03	-0.88	4.33E-01	-0.24	2.49E-01	-0.30	4.16E-01	-0.24
793.5983@0.6095531	793.5983	0.61	POS	hilic				4.65E-02	1.99E-03	-0.92	7.62E-01	0.08	1.95E-01	-0.40	9.04E-01	-0.04
793.6075@3.0232818	793.6075	3.02	POS	hilic				2.11E-01	5.48E-03	0.82	1.37E-01	0.46	1.88E-02	0.64	2.02E-01	0.38
793.7151@10.757187	793.7151	10.76	POS	hilic				9.91E-01	7.78E-01	0.10	8.04E-01	-0.06	7.86E-01	0.08	7.65E-01	0.08
793.8663@4.5682983	793.8663	4.57	NEG	hilic				7.35E-01	1.76E-01	0.45	1.91E-01	0.39	4.50E-01	0.20	2.21E-01	0.35
793.9575@9.991833	793.9575	9.99	POS	RP				2.32E-02	5.10E-02	0.56	4.15E-01	-0.24	1.36E-01	-0.38	6.88E-02	0.56
794.2037@0.3985298	794.2037	0.40	NEG	hilic				4.96E-01	5.53E-02	0.49	1.22E-01	0.47	9.15E-01	0.03	2.14E-01	0.42
794.2042@9.429137	794.2042	9.43	NEG	RP				7.77E-01	4.29E-01	-0.24	5.67E-01	0.14	5.39E-01	0.20	6.63E-01	-0.14
794.2047@9.611614	794.2047	9.61	NEG	RP				9.84E-01	8.07E-01	0.08	9.72E-01	-0.01	6.54E-01	0.13	6.07E-01	0.16
794.5647@12.02858	794.5647	12.03	POS	RP				1.98E-01	3.15E-02	0.69	6.85E-02	0.45	2.81E-02	0.58	6.60E-01	0.11
795.2069@0.3987889	795.2069	0.40	NEG	hilic				8.41E-01	2.57E-01	0.31	2.75E-01	0.32	6.71E-01	0.12	2.89E-01	0.33
795.3923@0.50260526	795.3923	0.50	POS	hilic				7.69E-01	8.83E-01	-0.05	3.17E-01	0.28	5.31E-01	-0.18	5.47E-01	0.18
795.5105@12.23116	795.5105	12.23	POS	RP				7.46E-01	5.50E-01	-0.17	4.30E-01	-0.20	7.57E-01	0.08	1.71E-01	-0.35
795.519@12.23855	795.5190	12.24	POS	RP				8.99E-01	5.71E-01	-0.15	6.34E-01	-0.12	6.06E-01	0.13	5.56E-01	-0.15
795.5213@0.71069235	795.5213	0.71	POS	hilic				3.99E-01	4.30E-01	-0.23	1.44E-01	-0.38	1.82E-01	-0.38	4.55E-01	0.21
795.5382@12.267837	795.5382	12.27	NEG	RP				6.32E-01	6.22E-01	-0.13	1.55E-01	0.44	7.19E-01	0.09	4.64E-01	0.23
795.5446@0.70843637	795.5446	0.71	NEG	hilic				5.97E-01	2.85E-01	0.34	9.91E-01	0.00	4.54E-01	-0.21	3.19E-01	0.31
795.5544@12.67941	795.5544	12.68	NEG	RP				4.29E-02	8.84E-04	0.95	2.57E-01	0.34	7.19E-01	-0.10	1.13E-01	0.48
795.5776@12.474878	795.5776	12.47	POS	RP	PC 37:4	20 eV: 184.0728 (100), 796.5840 (95)	2	8.12E-01	8.63E-01	-0.06	6.69E-01	0.12	9.59E-01	-0.01	1.83E-01	-0.32
795.585@12.374189	795.5850	12.37	POS	RP				6.84E-01	8.60E-01	0.05	2.58E-01	-0.30	5.04E-01	0.19	7.48E-01	-0.07
795.6064@3.0352783	795.6064	3.04	POS	hilic				6.14E-01	4.71E-01	0.23	7.49E-02	0.49	2.81E-01	0.30	6.48E-02	0.51
795.614@13.506196	795.6140	13.51	POS	RP				4.81E-01	3.63E-02	-0.51	4.53E-01	-0.20	6.10E-01	0.15	6.35E-01	-0.14
795.7293@10.766242	795.7293	10.77	POS	hilic				5.30E-01	1.83E-01	0.45	5.95E-01	0.17	1.56E-01	0.39	4.69E-02	0.58
795.957@11.209511	795.9570	11.21	NEG	RP				3.99E-01	1.27E-01	0.38	4.97E-01	-0.12	2.19E-01	0.27	2.33E-01	0.32
796.5394@12.265512	796.5394	12.27	NEG	RP				2.90E-01	1.56E-01	-0.41	4.06E-01	-0.23	6.16E-01	-0.15	1.30E-01	0.41
796.5461@0.71084017	796.5461	0.71	NEG	hilic				4.65E-01	2.48E-01	0.37	1.74E-02	0.60	3.49E-01	0.21	2.03E-01	0.34
796.6396@0.8240286	796.6396	0.82	POS	hilic				4.28E-01	8.31E-01	-0.05	1.26E-01	0.43	2.30E-01	0.32	7.30E-01	-0.08
796.6404@0.82167923	796.6404	0.82	POS	hilic				2.20E-01	1.17E-01	-0.34	4.27E-01	0.22	3.60E-01	0.24	2.00E-01	-0.29
797.388@0.962567	797.3880	0.96	POS	hilic				5.36E-01	9.66E-01	0.01	1.21E-01	0.38	7.98E-02	0.42	8.19E-01	-0.07
797.5209@11.503307	797.5209	11.50	NEG	RP				4.39E-01	1.89E-01	0.36	4.66E-01	0.18	2.75E-02	0.63	5.26E-01	0.17
797.5296@12.347053	797.5296	12.35	POS	RP				8.01E-01	4.22E-01	0.23	3.72E-01	-0.22	9.40E-01	0.02	7.04E-01	0.11
797.53@12.694946	797.5300	12.69	POS	RP				6.23E-01	4.49E-01	0.26	1.84E-01	-0.33	9.53E-01	-0.02	9.15E-01	0.03
797.554@12.6774	797.5540	12.68	NEG	RP				4.29E-02	5.06E-03	0.80	5.80E-01	0.16	2.02E-01	-0.35	2.31E-01	0.32
797.5887@12.738018	797.5887	12.74	POS	RP				1.98E-01	4.10E-01	0.22	6.98E-01	-0.10	2.15E-01	0.32	4.72E-02	-0.42
798.1549@9.007982	798.1549	9.01	NEG	RP				3.59E-01	1.31E-01	0.48	1.92E-01	0.33	5.53E-01	-0.18	1.18E-01	0.43
798.5547@12.68137	798.5547	12.68	NEG	RP				1.01E-01	8.41E-03	0.80	2.61E-02	0.65	8.65E-01	0.05	7.69E-02	0.53
798.5984@0.63012	798.5984	0.63	POS	hilic				1.20E-01	1.19E-02	0.68	2.14E-01	0.32	6.32E-03	0.77	2.24E-01	0.32
798.6583@0.82351303	798.6583	0.82	POS	hilic				7.31E-01	5.44E-01	-0.14	3.08E-01	0.27	6.25E-01	0.14	6.54E-01	-0.12
799.404@0.96009105	799.4040	0.96	POS	hilic				8.33E-01	5.49E-01	0.21	3.31E-01	0.25	5.99E-01	0.14	6.51E-01	-0.14
799.4197@13.808947	799.4197	13.81	NEG	RP				4.93E-01	8.82E-01	0.04	4.80E-01	0.15	4.69E-02	0.53	3.62E-01	0.24
799.5204@11.873539	799.5204	11.87	POS	RP				2.31E-01	7.06E-01	0.09	6.28E-01	0.10	3.41E-02	0.54	6.02E-01	-0.10
799.5369@0.6609233	799.5369	0.66	NEG	hilic				3.26E-01	4.67E-02	0.56	4.08E-01	-0.25	5.13E-01	0.19	4.79E-01	0.22
799.5377@11.708419	799.5377	11.71	NEG	RP	PC 34:4 (14:0_20:4, M+FA)	20 eV: 738.5062 (100), 303.2325 (20), 798.5308 (15), 227.1998 (5)	2	5.68E-01	5.60E-01	-0.13	9.70E-01	-0.01	4.06E-01	0.22	1.46E-01	0.36
799.6636@0.82547617	799.6636	0.83	POS	hilic				2.42E-01	1.33E-01	-0.28	9.99E-02	0.42	8.00E-01	0.06	8.47E-01	-0.04
800.5451@0.40236735	800.5451	0.40	POS	hilic				1.64E-01	2.67E-03	0.77	9.39E-01	0.02	7.39E-02	0.55	6.78E-01	0.13
800.5455@11.089195	800.5455	11.09	POS	RP				3.48E-01	5.98E-03	0.79	2.54E-01	0.37	2.93E-01	0.33	1.38E-01	0.44
800.5817@0.8026526	800.5817	0.80	POS	hilic				6.63E-02	1.45E-01	-0.37	5.63E-02	0.53	1.11E-01	0.45	8.30E-01	-0.06
801.5285@11.780942	801.5285	11.78	POS	RP				3.53E-01	2.28E-01	-0.22	6.79E-01	0.09	1.13E-01	0.42	8.73E-01	0.03
801.529@11.872185	801.5290	11.87	POS	RP				1.59E-01	2.22E-01	0.35	4.43E-02	0.58	9.70E-03	0.70	9.81E-01	-0.01
801.5314@0.62718964	801.5314	0.63	POS	hilic				7.58E-01	5.87E-01	-0.14	2.39E-01	-0.35	9.97E-01	0.00	1.80E-01	-0.37
801.5513@11.894257	801.5513	11.89	NEG	RP	PC 34:3 (16:1_18:2, M+FA)	20 eV: 740.5226 (100), 800.5441 (26), 279.2324 (15)	2	4.57E-01	6.81E-02	0.51	7.66E-01	-0.09	3.77E-01	0.27	2.07E-01	0.41
801.5517@0.6608199	801.5517	0.66	NEG	hilic				9.93E-02	6.58E-01	-0.13	2.98E-01	-0.27	2.25E-02	-0.61	1.91E-01	0.41
801.5535@10.956509	801.5535	10.96	NEG	RP				8.14E-03	6.39E-04	1.01	3.43E-01	-0.26	8.10E-01	-0.05	4.64E-01	0.22
801.5898@0.6722858	801.5898	0.67	POS	hilic				5.70E-01	1.51E-01	0.40	3.28E-01	0.25	6.67E-01	-0.09	2.71E-01	0.29
801.6574@3.2282789	801.6574	3.23	NEG	hilic				7.46E-01	5.28E-01	0.20	3.39E-01	0.28	6.62E-02	0.47	2.15E-01	0.37
801.6576@3.2103875	801.6576	3.21	NEG	hilic				6.04E-01	8.59E-02	0.59	3.74E-01	0.27	6.37E-01	0.12	2.41E-01	0.34
802.1564@0.39765152	802.1564	0.40	NEG	hilic				9.54E-01	5.78E-01	0.15	9.02E-01	0.03	3.24E-01	0.23	7.33E-01	0.09
802.2076@0.4054235	802.2076	0.41	NEG	hilic				9.53E-01	5.00E-01	0.19	8.82E-01	0.05	9.30E-01	0.03	6.87E-01	-0.12
802.4102@10.781523	802.4102	10.78	NEG	RP				5.58E-01	7.98E-02	-0.38	2.83E-01	-0.25	6.28E-01	-0.12	7.04E-01	0.10
802.4719@10.307139	802.4719	10.31	POS	RP				7.22E-01	8.05E-02	0.43	6.44E-01	0.12	9.70E-01	0.01	9.74E-01	0.01
802.5363@10.908683	802.5363	10.91	POS	RP				7.24E-01	8.40E-01	-0.05	9.06E-01	-0.03	4.52E-01	0.21	1.85E-01	0.34
802.5975@0.80499995	802.5975	0.80	POS	hilic				7.36E-02	7.20E-01	-0.07	1.18E-02	0.60	7.22E-02	0.44	7.97E-01	0.05
803.5447@11.997656	803.5447	12.00	POS	RP				1.18E-03	9.52E-01	0.02	7.66E-04	-1.03	1.81E-03	-0.86	9.02E-01	0.03
803.5488@0.6202259	803.5488	0.62	POS	hilic				9.19E-01	4.68E-01	0.23	6.32E-01	0.14	9.35E-01	-0.03	4.05E-01	0.25
803.5489@11.770035	803.5489	11.77	POS	RP				4.48E-01	3.99E-01	-0.23	5.33E-01	-0.18	2.65E-01	0.32	2.09E-01	-0.33

Supplementary table 2: Metabolomics analysis results

Compound	Mass	Retention Time	Ionization mode	column	Compound_ID	MSMS_spectra	ID_level	ANOVA P-FDR	Anorexia vs control P	Anorexia vs control Estimate	overweight vs control P	overweight vs control Estimate	obese vs control P	obese vs control Estimate	athletes vs control P	athletes vs control Estimate
803.567@12.20263	803.5670	12.20	NEG	RP				9.19E-01	5.99E-01	-0.15	9.34E-01	-0.02	3.03E-01	-0.26	6.36E-01	-0.12
803.5676@0.6608378	803.5676	0.66	NEG	hilic				1.56E-01	7.60E-01	-0.09	3.91E-01	-0.22	2.83E-02	-0.59	2.58E-01	0.37
803.5997@10.560359	803.5997	10.56	NEG	RP				5.15E-01	2.80E-01	-0.34	2.98E-01	0.30	4.22E-01	0.23	6.12E-01	0.15
803.6566@3.2289393	803.6566	3.23	NEG	hilic				3.37E-01	1.52E-02	0.74	4.80E-01	0.21	6.87E-02	0.48	1.67E-01	0.47
804.4376@11.042723	804.4376	11.04	NEG	RP				4.84E-01	3.96E-01	0.25	9.31E-01	-0.02	1.91E-01	0.37	3.48E-01	-0.24
804.5162@0.9973187	804.5162	1.00	NEG	hilic				3.69E-01	6.18E-01	-0.15	1.47E-01	0.38	2.10E-01	0.37	4.73E-01	-0.21
804.5715@0.66063964	804.5715	0.66	NEG	hilic				2.54E-01	8.37E-01	0.06	6.24E-01	-0.13	7.94E-02	-0.46	1.91E-01	0.42
804.5951@0.99172056	804.5951	0.99	NEG	hilic				1.45E-01	6.40E-02	-0.44	3.08E-01	0.27	2.77E-01	0.28	3.30E-01	-0.24
804.6127@0.8069501	804.6127	0.81	POS	hilic				1.73E-01	1.74E-01	-0.35	6.67E-02	0.50	2.99E-01	0.32	9.27E-01	-0.02
805.5014@11.081897	805.5014	11.08	POS	RP				3.30E-01	2.65E-02	0.64	1.11E-01	0.48	3.04E-02	0.66	3.50E-01	0.28
805.563@12.079224	805.5630	12.08	POS	RP				5.38E-01	1.03E-01	-0.48	6.91E-01	0.11	3.32E-01	-0.25	5.37E-01	-0.17
805.5635@11.939689	805.5635	11.94	POS	RP	PC 38:6 (16:0_22:6)	20 eV: 806.5690 (100), 184.0733 (95)	2	4.48E-01	1.12E-01	-0.45	1.70E-01	-0.43	5.42E-01	-0.17	6.30E-01	0.15
805.5638@12.063821	805.5638	12.06	POS	RP	PC 38:4 (18:2_20:2)	10 eV: 806.5721 (100), 184.0733 (12)	2	8.45E-01	3.84E-01	-0.22	6.77E-01	-0.10	2.46E-01	-0.37	3.84E-01	-0.26
805.5653@0.62016225	805.5653	0.62	POS	hilic				7.53E-01	4.58E-01	-0.22	7.07E-01	0.10	9.06E-01	0.03	3.10E-01	0.27
805.5833@12.66417	805.5833	12.66	NEG	RP				9.83E-01	5.89E-01	0.15	5.63E-01	0.17	6.87E-01	0.10	5.84E-01	0.18
805.6525@3.2436564	805.6525	3.24	NEG	hilic				5.16E-01	9.00E-02	0.51	5.29E-01	0.16	1.47E-01	0.38	6.18E-02	0.51
805.9572@9.963428	805.9572	9.96	POS	RP				4.63E-01	2.31E-01	0.41	9.12E-01	0.03	5.35E-01	-0.15	2.05E-01	0.40
806.1783@0.40604514	806.1783	0.41	NEG	hilic				5.35E-01	3.25E-01	0.29	2.47E-01	0.34	8.95E-01	0.03	7.97E-02	0.57
806.439@11.039697	806.4390	11.04	NEG	RP				6.94E-01	2.55E-01	0.33	5.91E-01	0.17	9.84E-02	0.46	9.53E-01	0.02
806.4589@9.9642	806.4589	9.96	POS	RP				9.92E-01	8.02E-01	0.08	7.35E-01	0.09	9.46E-01	-0.02	7.01E-01	0.11
806.4948@11.581483	806.4948	11.58	NEG	RP				7.82E-01	3.54E-01	0.26	8.67E-01	-0.04	4.12E-01	0.23	7.37E-01	-0.08
806.5287@0.98951834	806.5287	0.99	NEG	hilic				9.92E-01	9.74E-01	-0.01	7.81E-01	-0.07	6.39E-01	-0.14	6.39E-01	-0.14
806.5701@0.6200176	806.5701	0.62	POS	hilic				9.78E-01	7.41E-01	0.10	5.02E-01	0.19	9.66E-01	-0.01	7.52E-01	0.10
806.5854@0.6604505	806.5854	0.66	NEG	hilic				7.55E-01	1.94E-01	0.43	4.90E-01	0.19	9.52E-01	-0.02	3.54E-01	0.28
806.6116@0.99210006	806.6116	0.99	NEG	hilic				2.47E-02	1.64E-01	-0.39	1.62E-02	0.62	1.20E-01	0.42	5.23E-01	-0.19
806.6255@0.8054873	806.6255	0.81	POS	hilic				1.85E-01	2.28E-01	-0.34	1.63E-01	0.36	2.66E-01	0.35	2.47E-01	-0.31
806.961@9.961624	806.9610	9.96	POS	RP				7.15E-02	7.19E-01	-0.11	6.74E-03	-0.75	6.50E-04	-0.77	4.63E-01	-0.22
807.3923@0.46605498	807.3923	0.47	POS	hilic				7.09E-01	1.20E-01	-0.46	9.29E-01	0.02	4.07E-01	-0.23	8.87E-01	-0.04
807.4612@9.9568615	807.4612	9.96	POS	RP				3.75E-02	8.44E-01	0.05	1.05E-01	-0.45	4.06E-03	-0.78	5.55E-01	0.18
807.5121@0.9230636	807.5121	0.92	POS	hilic				3.50E-02	5.15E-04	-1.02	1.40E-01	-0.43	7.79E-01	-0.07	2.55E-01	-0.36
807.5789@12.286388	807.5789	12.29	POS	RP	PC 38:5 (18:1_20:4)	20 eV: 808.5839 (100), 184.0724 (100)	2	8.22E-02	9.61E-04	-0.82	6.39E-01	-0.13	5.42E-02	-0.49	9.98E-01	0.00
807.5796@0.6199291	807.5796	0.62	POS	hilic				4.06E-01	4.62E-01	0.22	8.96E-01	-0.04	7.77E-02	-0.51	6.93E-01	0.12
807.5809@12.373184	807.5809	12.37	POS	RP				8.12E-01	6.92E-01	0.10	4.91E-01	0.16	2.88E-01	0.30	1.92E-01	0.36
807.7317@10.771945	807.7317	10.77	POS	hilic				3.88E-01	1.42E-02	-0.76	3.10E-01	-0.25	4.84E-01	-0.20	3.02E-01	-0.31
808.0914@0.50505555	808.0914	0.51	NEG	hilic				1.31E-03	1.18E-02	0.73	4.52E-02	-0.22	5.69E-01	-0.08	9.57E-01	0.01
808.183@9.009938	808.1830	9.01	NEG	RP				6.90E-01	3.23E-01	0.27	5.12E-02	0.52	4.61E-01	0.24	2.37E-01	0.35
808.1852@8.766553	808.1852	8.77	NEG	RP				3.33E-01	5.06E-02	-0.60	6.10E-01	0.16	4.27E-01	-0.20	8.42E-01	0.06
808.1867@0.40615398	808.1867	0.41	NEG	hilic				3.92E-01	1.51E-01	0.43	4.63E-02	0.57	6.30E-01	0.12	9.70E-02	0.57
808.4787@10.237472	808.4787	10.24	POS	RP				5.57E-01	3.90E-01	-0.22	2.09E-01	0.32	3.98E-01	0.19	5.84E-01	0.14
808.5166@6.0254173	808.5166	6.03	POS	hilic				5.37E-01	5.75E-01	0.17	9.20E-01	-0.03	1.30E-01	-0.40	3.93E-01	0.24
808.5759@12.101481	808.5759	12.10	POS	RP				4.08E-01	1.46E-01	-0.45	5.83E-02	-0.56	1.59E-01	-0.40	8.49E-01	-0.05
808.6455@0.8074484	808.6455	0.81	POS	hilic				8.61E-01	2.35E-01	-0.33	9.12E-01	0.03	7.67E-01	-0.09	4.34E-01	-0.18
808.6456@0.8146545	808.6456	0.81	POS	hilic				5.45E-01	5.18E-02	-0.45	8.29E-01	-0.06	1.30E-01	-0.41	4.36E-01	-0.18
808.9822@10.237158	808.9822	10.24	POS	RP				4.21E-02	2.91E-02	0.73	3.60E-01	-0.27	3.40E-01	-0.25	3.49E-01	0.28
809.1897@0.40595502	809.1897	0.41	NEG	hilic				5.11E-01	4.71E-02	0.57	1.88E-01	0.38	5.83E-01	0.16	1.05E-01	0.50
809.4048@0.46875575	809.4048	0.47	POS	hilic				6.35E-01	5.60E-02	-0.52	3.28E-01	-0.26	3.65E-01	-0.26	8.44E-01	-0.06
809.4835@10.23707	809.4835	10.24	POS	RP				2.76E-01	4.51E-02	0.70	6.72E-02	0.43	1.32E-01	0.32	4.62E-02	0.57
809.526@12.142564	809.5260	12.14	POS	RP				2.42E-01	3.29E-01	-0.24	2.90E-01	0.29	4.00E-02	0.55	7.63E-01	0.09
809.5508@10.0246725	809.5508	10.02	POS	RP				6.67E-02	5.15E-02	-0.53	7.68E-01	-0.09	4.41E-01	-0.22	1.14E-03	-0.94
809.5548@12.0506315	809.5548	12.05	NEG	RP				5.67E-01	1.24E-01	-0.46	7.13E-01	0.11	6.18E-01	0.14	6.55E-01	-0.13
809.5562@0.6304318	809.5562	0.63	NEG	hilic				2.31E-01	1.65E-02	-0.60	8.35E-01	0.05	8.94E-01	-0.04	3.61E-01	0.26
809.5778@12.107526	809.5778	12.11	POS	RP				2.74E-01	4.94E-01	-0.23	2.20E-02	-0.61	7.73E-02	-0.41	7.55E-01	0.09
809.5932@12.601767	809.5932	12.60	POS	RP	PC 38:4 (18:1_20:3)	10 eV: 810.6012 (100), 184.0738 (15)	2	2.01E-01	6.22E-02	0.53	4.72E-01	-0.23	2.56E-01	0.29	3.96E-01	-0.25
809.5945@12.830706	809.5945	12.83	POS	RP	PC 38:4 (18:0_20:4)	10 eV: 810.6012 (100), 184.0735 (13)	2	2.94E-01	1.00E-01	0.48	5.76E-01	0.16	7.51E-02	0.52	5.57E-01	-0.16
809.5955@0.6169303	809.5955	0.62	POS	hilic				9.05E-01	9.94E-01	0.00	5.84E-01	-0.15	5.71E-01	0.17	6.24E-01	-0.13
809.745@10.780698	809.7450	10.78	POS	hilic				9.29E-01	6.52E-01	-0.15	3.83E-01	-0.21	7.83E-01	0.08	6.26E-01	-0.14
810.587@12.296289	810.5870	12.30	POS	RP				4.93E-01	6.93E-01	0.12	7.73E-01	0.07	2.38E-01	-0.32	1.25E-01	-0.37
810.6605@0.802983	810.6605	0.80	POS	hilic				2.17E-01	1.47E-01	-0.45	2.63E-01	0.27	6.41E-01	0.15	7.59E-02	-0.41
810.6614@0.8116439	810.6614	0.81	POS	hilic				2.38E-01	2.95E-01	-0.33	2.31E-01	0.28	5.37E-01	0.20	7.59E-02	-0.45
811.4827@12.226587	811.4827	12.23	POS	RP				2.50E-01	9.47E-01	0.02	2.96E-01	0.27	6.54E-01	0.13	1.62E-02	0.76
811.5726@0.63063055	811.5726	0.63	NEG	hilic				3.37E-02	2.10E-03	-0.83	8.03E-01	0.07	8.62E-01	-0.05	2.42E-01	0.35
811.5728@12.384905	811.5728	12.38	NEG	RP				2.05E-01	1.74E-03	-0.74	9.03E-01	-0.04	9.34E-01	0.02	9.06E-01	-0.04
811.6096@0.6203999	811.6096	0.62	POS	hilic				2.80E-02	6.83E-01	0.13	3.15E-02	0.60	8.57E-04	0.91	7.73E-01	-0.07
811.6645@0.80535996	811.6645	0.81	POS	hilic				2.12E-01	1.54E-01	-0.43	1.50E-01	0.39	5.15E-01	0.20	3.51E-01	-0.25
811.6648@0.8057984	811.6648	0.81	POS	hilic				4.92E-01	3.19E-01	-0.27	3.08E-01	0.30	9.39E-01	0.02	2.23E-01	-0.31
812.0048@10.51998	812.0048	10.52	POS	RP				4.99E-03	4.05E-03	0.91	1.48E-01	-0.40	5.04E-01	0.18	1.20E-01	0.44
812.0053@1.4490705	812.0053	1.45	POS	hilic				8.19E-02	5.97E-01	0.15	3.72E-03	-0.57	9.85E-02	-0.40	7.07E-01	0.10
812.1667@0.3987861	812.1667	0.40	NEG	hilic				2.50E-01	1.09E-02	0.77	6.33E-02	0.52	3.20E-02	0.61	8.95E-02	0.54
812.4229@11.037379	812.4229	11.04	NEG	RP				5.79E-01	8.38E-01	-0.05	5.07E-01	-0.17	3.45E-01	-0.27	3.52E-02	-0.48
812.5478@11.0945835	812.5478	11.09	POS	RP				1.87E-01	3.02E-02	0.60	9.06E-01	-0.03	7.39E-01	-0.09	5.88E-01	-0.13
812.6136@0.61369234	812.6136	0.61	POS	hilic				2.70E-02	8.90E-01	0.04	3.50E-01	0.28	1.34E-03	0.80	1.75E-01	-0.38
812.6744@0.80995655	812.6744	0.81	POS	hilic				1.02E-01	4.34E-02	-0.59	1.94E-01	0.38	9.68E-01	-0.01	1.40E-01	-0.36
813.0505@0.50523686	813.0505	0.51	NEG	hilic				1.42E-01	5.51E-02	0.57	7.09E-01	-0.07	6.29E-01	-0.10	5.96E-01	-0.11

Supplementary table 2: Metabolomics analysis results

								ANOVA	Anorexia vs control		overweight vs control		obese vs control		athletes vs control	
Compound	Mass	Retention Time	Ionization mode	column	Compound_ID	MSMS_spectra	ID_level	P-FDR	P	Estimate	P	Estimate	P	Estimate	P	Estimate
813.0548@12.939862	813.0548	12.94	POS	RP				7.29E-01	4.83E-01	0.22	4.84E-01	-0.20	2.71E-01	0.27	7.73E-01	0.08
813.4069@10.792477	813.4069	10.79	NEG	RP				5.04E-01	4.93E-02	-0.41	6.64E-01	0.11	6.38E-01	-0.11	3.79E-01	-0.20
813.5087@10.514989	813.5087	10.51	POS	RP				9.86E-02	1.67E-01	0.44	4.49E-02	-0.55	4.67E-01	-0.19	8.95E-01	0.04
813.5497@11.92103	813.5497	11.92	NEG	RP	PC 35:4 (15:0_20:4, M+FA)	20 eV: 752.5211 (100), 812.5429 (25), 303.2275 (12)	2	2.71E-01	8.35E-01	-0.04	5.07E-02	0.47	7.95E-01	-0.06	2.75E-01	0.26
813.5864@0.63840705	813.5864	0.64	NEG	hilic				1.15E-02	6.56E-04	-0.88	3.56E-01	0.25	9.68E-01	0.01	3.11E-01	0.30
813.5874@12.587301	813.5874	12.59	NEG	RP	EtherPC 36:4e (16:0e_20:4, M+FA)	20 eV: 752.5572 (100), 812.5778 (26), 303.2313 (15)	2	1.20E-03	8.45E-05	-1.24	6.18E-01	0.14	6.82E-01	-0.11	9.19E-01	-0.03
813.6788@0.80671453	813.6788	0.81	POS	hilic				7.36E-02	5.92E-02	-0.56	7.46E-02	0.50	9.93E-01	0.00	3.93E-01	-0.21
814.5428@11.64721	814.5428	11.65	NEG	RP				2.41E-03	9.64E-03	-0.69	3.26E-01	0.26	1.31E-01	0.40	5.99E-03	-0.70
814.5438@0.98405796	814.5438	0.98	NEG	hilic				2.97E-02	2.31E-01	-0.38	2.87E-01	0.25	7.22E-02	0.47	5.80E-02	-0.47
814.561@11.096157	814.5610	11.10	POS	RP				4.41E-01	1.09E-02	0.68	6.08E-01	0.16	4.66E-01	0.21	1.76E-01	0.38
814.6847@0.8059168	814.6847	0.81	POS	hilic				1.65E-01	3.02E-01	-0.28	4.28E-02	0.59	5.41E-01	0.17	8.06E-01	-0.07
815.4155@7.313504	815.4155	7.31	POS	RP				3.17E-01	3.21E-02	0.72	2.89E-02	0.59	8.04E-02	0.51	1.83E-01	0.36
815.416@0.42665783	815.4160	0.43	POS	hilic				8.14E-02	2.29E-03	0.96	4.81E-02	0.49	6.24E-02	0.52	6.79E-01	0.13
815.565@12.089129	815.5650	12.09	NEG	RP				9.16E-01	9.64E-01	-0.01	4.01E-01	-0.20	7.89E-01	0.07	7.52E-01	0.08
815.5753@12.737174	815.5753	12.74	POS	RP				1.90E-01	3.42E-03	-0.79	5.58E-01	-0.18	5.30E-02	-0.49	2.39E-01	-0.30
815.5823@0.6040737	815.5823	0.60	POS	hilic				1.37E-01	6.01E-03	-0.88	5.38E-01	-0.18	1.60E-01	-0.43	7.52E-01	-0.09
816.3777@10.708762	816.3777	10.71	NEG	RP				3.34E-01	2.61E-02	0.48	1.41E-01	0.36	2.34E-02	0.52	5.66E-01	0.12
816.5546@11.955142	816.5546	11.96	NEG	RP				3.45E-02	3.68E-01	-0.18	3.19E-02	0.50	1.97E-02	0.57	6.97E-01	-0.09
816.7066@3.3418107	816.7066	3.34	POS	hilic				8.00E-01	5.49E-01	-0.19	2.39E-01	-0.31	7.16E-01	0.10	9.39E-01	0.02
817.5018@11.887197	817.5018	11.89	POS	RP				4.83E-02	2.24E-01	0.36	8.31E-01	-0.06	4.63E-03	0.77	3.83E-01	-0.21
817.5392@12.126354	817.5392	12.13	NEG	RP				3.85E-01	1.43E-01	0.42	6.57E-02	0.57	4.31E-02	0.66	1.06E-01	0.48
817.5408@0.7080446	817.5408	0.71	NEG	hilic				8.34E-01	3.67E-01	-0.29	8.21E-01	0.05	6.77E-01	0.11	8.03E-01	0.08
817.5459@11.986971	817.5459	11.99	NEG	RP				1.12E-03	6.79E-01	0.12	1.78E-04	-0.92	1.92E-03	-0.80	4.93E-01	-0.16
817.5632@0.98462844	817.5632	0.98	NEG	hilic				3.99E-01	4.96E-01	0.20	3.73E-01	-0.24	4.98E-02	-0.51	9.71E-01	-0.01
817.5656@12.362839	817.5656	12.36	POS	RP				1.12E-01	1.63E-03	-0.77	7.77E-01	0.08	6.97E-01	-0.12	3.17E-01	-0.25
817.5805@12.396884	817.5805	12.40	NEG	RP				4.32E-01	1.20E-01	-0.40	1.93E-01	-0.31	7.66E-01	0.08	1.04E-01	-0.39
817.5971@12.604639	817.5971	12.60	POS	RP	EtherPC 40:7	10 eV: 818.6034 (100), 184.0737 (10)	2	4.75E-02	3.50E-04	-1.01	4.26E-01	-0.24	8.43E-01	-0.06	3.64E-01	-0.26
817.5999@0.60502625	817.5999	0.61	POS	hilic				8.14E-02	4.08E-03	-0.92	7.46E-01	-0.08	7.77E-02	-0.56	4.80E-01	-0.22
817.7151@0.40915298	817.7151	0.41	POS	hilic				9.34E-01	4.44E-01	0.19	7.61E-01	-0.09	6.50E-01	-0.11	9.30E-01	-0.02
818.0018@0.5068809	818.0018	0.51	NEG	hilic				5.86E-01	1.39E-01	0.42	3.54E-01	0.22	8.97E-01	-0.03	9.20E-01	0.02
818.1497@0.3967465	818.1497	0.40	NEG	hilic				9.18E-01	2.28E-01	0.30	8.02E-01	0.06	9.34E-01	0.02	7.95E-01	0.08
818.7068@3.3433585	818.7068	3.34	POS	hilic				4.07E-01	2.68E-01	-0.41	9.76E-02	-0.42	9.82E-01	0.01	5.22E-01	0.17
819.494@11.082059	819.4940	11.08	NEG	RP				3.28E-01	7.53E-02	-0.58	4.94E-01	0.16	9.69E-01	-0.01	2.88E-01	-0.34
819.5124@12.00776	819.5124	12.01	POS	RP				4.08E-02	9.10E-01	0.03	5.49E-03	-0.67	2.03E-03	-0.77	3.17E-01	-0.31
819.5128@12.090477	819.5128	12.09	POS	RP				8.18E-01	4.36E-01	0.19	6.26E-01	-0.11	4.08E-01	-0.18	8.85E-01	-0.04
819.5181@0.7062893	819.5181	0.71	POS	hilic				3.01E-01	4.40E-02	-0.51	6.64E-01	0.12	4.06E-01	0.23	7.02E-01	0.10
819.5357@11.983824	819.5357	11.98	NEG	RP				7.09E-01	4.39E-01	0.20	2.44E-01	-0.24	8.16E-01	-0.05	6.63E-01	-0.11
819.5556@12.385884	819.5556	12.39	NEG	RP				1.32E-01	7.58E-02	0.63	2.33E-01	-0.33	6.86E-01	0.11	6.49E-01	-0.14
819.5557@0.71168816	819.5557	0.71	NEG	hilic				7.88E-01	3.85E-01	0.28	5.82E-01	-0.16	5.97E-01	-0.16	7.81E-01	0.08
819.5597@11.616355	819.5597	11.62	NEG	RP				5.13E-01	8.84E-02	-0.54	7.13E-01	0.12	7.58E-01	-0.09	8.62E-01	0.04
819.5764@12.362763	819.5764	12.36	POS	RP				5.12E-01	3.00E-02	-0.49	7.67E-01	0.08	8.27E-01	-0.06	6.52E-01	-0.11
819.5785@12.365999	819.5785	12.37	POS	RP				3.50E-02	2.82E-02	-0.61	7.84E-01	-0.07	6.42E-01	0.13	5.85E-03	-0.75
819.595@13.091549	819.5950	13.09	NEG	RP				8.89E-01	5.46E-01	0.18	7.82E-01	-0.07	5.04E-01	0.18	7.11E-01	-0.12
819.6085@12.87143	819.6085	12.87	POS	RP				2.10E-01	9.00E-03	-0.73	5.55E-02	-0.50	8.05E-02	-0.51	3.26E-02	-0.57
819.6087@0.92883784	819.6087	0.93	NEG	hilic				6.15E-01	9.14E-01	0.03	1.34E-01	0.34	4.30E-01	0.17	6.00E-01	-0.09
819.6099@0.6061657	819.6099	0.61	POS	hilic				1.34E-01	5.99E-03	-0.83	5.89E-01	-0.13	5.75E-02	-0.62	5.19E-01	-0.20
819.7304@10.7703	819.7304	10.77	POS	hilic				1.50E-01	4.37E-01	0.22	5.19E-03	0.88	2.71E-01	0.31	8.93E-02	0.48
820.1561@9.364676	820.1561	9.36	NEG	RP				3.78E-01	2.05E-01	0.36	7.24E-02	0.63	8.71E-01	0.05	1.39E-01	0.44
820.1563@9.157651	820.1563	9.16	NEG	RP				7.58E-01	2.92E-01	0.34	1.64E-01	0.41	8.08E-01	0.06	7.38E-01	0.09
820.1577@9.420353	820.1577	9.42	NEG	RP				8.11E-01	2.39E-01	0.31	2.88E-01	0.39	6.65E-01	0.11	3.01E-01	0.30
820.1577@9.604378	820.1577	9.60	NEG	RP				9.01E-01	4.88E-01	-0.20	9.39E-01	0.02	4.78E-01	0.20	9.62E-01	0.02
820.1588@0.39645702	820.1588	0.40	NEG	hilic				9.32E-01	5.43E-01	0.18	9.62E-01	0.01	5.72E-01	-0.16	8.18E-01	-0.07
820.5436@12.124943	820.5436	12.12	NEG	RP				2.31E-01	8.78E-01	-0.04	7.10E-01	0.08	2.28E-02	0.61	9.26E-01	0.02
820.5561@0.92918	820.5561	0.93	NEG	hilic				7.82E-02	4.24E-01	0.23	4.46E-01	-0.20	5.75E-02	-0.52	1.08E-01	0.47
820.5574@0.7107871	820.5574	0.71	NEG	hilic				5.92E-01	3.22E-01	0.30	3.38E-01	-0.26	7.79E-01	-0.08	4.37E-01	0.24
820.581@12.359788	820.5810	12.36	POS	RP				1.79E-01	2.68E-02	-0.52	5.89E-01	0.14	6.34E-01	0.13	2.18E-01	-0.30
821.4743@11.086253	821.4743	11.09	POS	RP				5.07E-01	1.11E-02	0.67	2.96E-01	0.33	2.29E-01	0.38	1.93E-01	0.42
821.5543@12.387265	821.5543	12.39	NEG	RP				5.97E-02	1.96E-01	0.39	1.17E-01	-0.46	7.45E-01	-0.08	4.40E-02	-0.62
821.5646@0.7127011	821.5646	0.71	NEG	hilic				5.80E-01	1.00E-01	0.48	7.32E-01	-0.09	6.95E-01	0.12	3.43E-01	0.27
821.5697@12.919981	821.5697	12.92	NEG	RP				2.00E-01	1.18E-01	0.42	8.93E-02	-0.51	9.76E-01	-0.01	8.62E-01	0.05
821.5732@11.897522	821.5732	11.90	NEG	RP				4.62E-01	5.24E-01	0.18	6.35E-01	0.11	1.14E-01	-0.34	3.87E-01	-0.18
821.587@12.605377	821.5870	12.61	POS	RP				2.73E-01	6.01E-02	0.61	5.64E-01	0.15	8.07E-01	-0.06	5.97E-01	-0.14
821.625@0.6034263	821.6250	0.60	POS	hilic				1.50E-01	5.19E-03	-0.81	7.86E-01	-0.07	1.29E-01	-0.50	8.41E-01	-0.06
821.747@10.781374	821.7470	10.78	POS	hilic				5.36E-01	3.53E-01	-0.28	7.71E-01	0.07	2.53E-01	-0.36	3.85E-01	0.23
822.1936@0.40468758	822.1936	0.40	NEG	hilic				4.67E-01	1.32E-01	0.41	3.69E-02	0.66	3.39E-01	0.26	2.95E-01	0.33
822.5559@12.385184	822.5559	12.39	NEG	RP				8.61E-01	4.88E-01	0.24	2.71E-01	0.34	2.43E-01	0.27	8.11E-01	0.06
822.6392@10.7078495	822.6392	10.71	NEG	RP				1.86E-01	6.41E-02	-0.49	6.85E-01	0.13	1.94E-01	0.31	1.89E-01	-0.40
823.2784@3.5406013	823.2784	3.54	NEG	hilic				8.00E-01	3.06E-01	0.31	6.40E-01	0.13	2.88E-01	0.28	7.67E-01	-0.07
823.5381@0.650219	823.5381	0.65	NEG	hilic				6.66E-01	2.97E-01	0.27	1.76E-01	0.31	1.21E-01	0.42	7.40E-01	0.08
823.5456@12.952928	823.5456	12.95	POS	RP				7.65E-01	8.78E-01	-0.04	2.42E-01	-0.37	7.56E-01	-0.08	2.42E-01	-0.34
823.5531@0.6947976	823.5531	0.69	POS	hilic				5.01E-01	2.38E-01	0.36	2.77E-01	0.31	9.09E-02	0.44	7.60E-01	-0.07
823.7608@10.784888	823.7608	10.78	POS	hilic				4.49E-01	1.99E-01	-0.38	7.02E-01	0.11	2.01E-01	-0.41	6.32E-01	0.15
824.554@12.905256	824.5540	12.91	POS	RP				1.49E-01	3.28E-01	-0.15	8.57E-01	-0.03	1.66E-01	0.34	4.18E-02	0.46

Supplementary table 2: Metabolomics analysis results

								ANOVA	Anorexia vs control		overweight vs control		obese vs control		athletes vs control	
Compound	Mass	Retention Time	Ionization mode	column	Compound_ID	MSMS_spectra	ID_level	P-FDR	P	Estimate	P	Estimate	P	Estimate	P	Estimate
825.409@0.5001487	825.4090	0.50	POS	hilic				9.61E-01	3.66E-01	-0.26	5.01E-01	-0.18	5.38E-01	-0.17	6.64E-01	-0.12
825.4186@0.91328186	825.4186	0.91	POS	hilic				2.83E-01	7.20E-02	0.53	4.61E-02	0.59	9.21E-01	0.02	1.17E-01	0.50
825.5244@11.929389	825.5244	11.93	POS	RP				8.01E-01	9.79E-01	-0.01	2.98E-01	-0.29	6.30E-01	0.14	8.45E-01	-0.06
825.531@11.760687	825.5310	11.76	POS	RP				5.36E-01	4.95E-01	-0.18	1.91E-01	-0.37	3.47E-01	0.25	6.76E-01	-0.12
825.5514@0.64854985	825.5514	0.65	NEG	hilic				2.67E-01	1.26E-01	-0.46	2.92E-01	0.32	5.24E-01	0.18	2.61E-01	0.31
825.5516@11.860016	825.5516	11.86	NEG	RP				3.19E-01	6.48E-01	0.11	8.22E-01	0.06	5.93E-02	0.53	3.90E-01	-0.23
825.5526@11.850762	825.5526	11.85	NEG	RP	PC 36:5 (16:1_20:4, M+FA)	20 eV: 764.5227 (100), 824.5459 (32), 303.2334 (15), 253.2137 (3)	2	6.50E-03	5.93E-01	-0.07	4.22E-01	0.14	2.73E-03	0.75	8.63E-01	0.03
825.588@0.66770935	825.5880	0.67	POS	hilic				5.98E-01	4.47E-01	0.20	9.85E-02	0.44	8.68E-01	-0.04	2.60E-01	0.32
825.6432@10.706864	825.6432	10.71	NEG	RP				3.13E-01	8.52E-02	-0.45	8.50E-01	0.06	4.23E-01	0.18	1.12E-01	-0.47
825.6522@0.5999144	825.6522	0.60	POS	hilic				3.58E-01	1.55E-02	-0.65	6.06E-01	-0.12	1.45E-01	-0.48	9.19E-01	-0.03
826.1896@0.39705113	826.1896	0.40	NEG	hilic				9.32E-01	4.72E-01	0.19	4.78E-01	0.23	6.47E-01	0.13	3.09E-01	0.30
826.5805@10.0275345	826.5805	10.03	POS	RP				2.44E-01	5.68E-01	-0.16	4.07E-01	0.24	3.96E-01	-0.27	2.83E-02	-0.59
826.6384@10.706762	826.6384	10.71	NEG	RP				4.61E-01	1.79E-01	-0.36	8.82E-01	0.05	3.82E-01	0.21	1.75E-01	-0.40
827.4201@0.48741183	827.4201	0.49	POS	hilic				5.31E-01	5.52E-02	-0.56	8.86E-01	-0.04	2.25E-01	-0.31	3.27E-01	-0.26
827.5271@3.8347776	827.5271	3.83	POS	hilic				2.46E-03	8.94E-01	-0.04	8.99E-05	1.01	3.06E-04	0.95	1.68E-01	0.37
827.5432@12.368961	827.5432	12.37	POS	RP				1.28E-01	7.36E-01	0.10	8.52E-01	0.05	6.10E-02	0.51	7.75E-02	-0.47
827.5452@12.07872	827.5452	12.08	POS	RP				5.85E-02	6.01E-03	-0.84	4.58E-01	-0.21	4.00E-01	-0.24	4.69E-03	-0.83
827.5464@0.61038965	827.5464	0.61	POS	hilic				4.96E-01	9.35E-01	0.03	6.94E-02	-0.47	9.01E-02	-0.48	3.02E-01	-0.30
827.567@0.650479	827.5670	0.65	NEG	hilic				6.88E-01	8.32E-01	-0.06	2.29E-01	0.37	3.02E-01	0.29	2.49E-01	0.35
827.5672@12.1328335	827.5672	12.13	NEG	RP	PC 36:4 (16:0_20:4, M+FA)	20 eV: 766.5370 (100), 826.5581 (29), 303.2344 (13), 255.2353 (6)	2	8.12E-01	5.21E-01	0.19	9.46E-01	-0.02	6.66E-01	0.12	2.02E-01	0.39
827.5676@11.977219	827.5676	11.98	NEG	RP	PC 36:4 (18:2_18:2, M+FA)	20 eV: 766.5381 (100), 826.5596 (25), 279.2318 (20)	2	1.74E-05	6.30E-01	0.12	8.17E-04	-0.93	3.06E-04	-1.04	1.29E-01	0.38
827.597@0.63917726	827.5970	0.64	POS	hilic				7.06E-01	7.84E-01	-0.08	9.92E-01	0.00	3.35E-01	-0.25	3.16E-01	0.27
827.6018@10.538586	827.6018	10.54	NEG	RP				8.34E-03	9.73E-02	-0.47	3.26E-02	0.59	1.47E-02	0.68	8.19E-01	-0.07
827.8019@4.4826493	827.8019	4.48	POS	hilic				8.85E-01	9.30E-01	-0.03	4.88E-01	-0.18	6.00E-01	-0.19	1.96E-01	-0.35
827.8608@4.5689096	827.8608	4.57	NEG	hilic				6.60E-01	2.09E-01	0.37	1.07E-01	0.49	6.71E-01	0.12	1.67E-01	0.36
827.8612@4.567938	827.8612	4.57	NEG	hilic				6.56E-01	4.57E-01	0.22	8.97E-02	0.50	7.02E-01	0.12	1.22E-01	0.39
828.1702@9.412938	828.1702	9.41	NEG	RP				2.43E-01	1.70E-01	-0.40	7.18E-01	0.10	9.95E-01	0.00	5.41E-02	-0.62
828.1705@9.3443	828.1705	9.34	NEG	RP				8.32E-01	5.65E-01	0.17	2.36E-01	0.34	2.54E-01	0.36	3.41E-01	0.31
828.1718@0.39663053	828.1718	0.40	NEG	hilic				6.94E-01	2.22E-01	0.35	2.47E-01	0.36	9.65E-01	0.01	1.97E-01	0.39
828.4985@10.421863	828.4985	10.42	POS	RP				6.67E-01	7.06E-01	-0.09	7.45E-01	0.08	1.25E-01	0.39	3.77E-01	0.21
828.5741@11.100172	828.5741	11.10	POS	RP				3.11E-01	2.51E-02	0.68	9.45E-01	0.02	5.85E-01	0.15	8.05E-01	0.07
828.6078@0.80491865	828.6078	0.80	POS	hilic				3.38E-02	2.03E-02	-0.54	3.55E-02	0.54	3.37E-01	0.32	7.65E-01	-0.07
828.6381@10.707682	828.6381	10.71	NEG	RP				8.83E-02	3.50E-02	-0.34	2.77E-01	-0.22	2.21E-01	0.31	1.32E-01	-0.26
828.6678@0.91315246	828.6678	0.91	POS	hilic				3.64E-02	7.71E-01	-0.08	1.28E-03	0.84	9.30E-02	0.49	5.55E-02	0.50
829.4115@10.792316	829.4115	10.79	NEG	RP				4.85E-01	8.46E-01	0.04	2.33E-01	0.28	4.34E-02	0.48	2.20E-01	0.29
829.443@11.039266	829.4430	11.04	NEG	RP				5.97E-01	6.35E-01	0.14	9.39E-01	-0.02	1.94E-01	0.45	1.34E-01	0.35
829.5618@12.372537	829.5618	12.37	POS	RP				1.49E-01	6.11E-01	0.16	3.13E-01	0.28	5.13E-02	0.52	1.13E-01	-0.38
829.5621@11.864457	829.5621	11.86	POS	RP	PC 40:8	10 eV: 830.5694 (100), 184.0727 (25)	2	6.68E-01	2.85E-01	-0.31	2.13E-01	-0.32	9.85E-02	-0.50	8.29E-01	-0.07
829.5637@0.61032206	829.5637	0.61	POS	hilic				7.50E-01	4.15E-01	-0.27	8.44E-01	-0.05	9.27E-01	-0.03	1.25E-01	-0.43
829.5813@0.6508295	829.5813	0.65	NEG	hilic				2.18E-01	1.26E-02	0.74	7.06E-01	-0.10	8.56E-01	0.05	4.63E-01	0.23
829.5832@12.38725	829.5832	12.39	NEG	RP				9.92E-01	8.85E-01	0.05	7.30E-01	0.09	8.65E-01	-0.05	7.47E-01	0.09
830.1169@8.922881	830.1169	8.92	NEG	RP				1.61E-01	3.52E-01	-0.30	2.39E-01	-0.35	7.55E-03	-0.77	7.11E-03	-0.73
830.185@9.157293	830.1850	9.16	NEG	RP				8.52E-01	2.19E-01	-0.39	5.74E-01	-0.17	5.62E-01	-0.17	9.71E-01	-0.01
830.1853@9.60322	830.1853	9.60	NEG	RP				7.62E-01	5.23E-01	0.18	7.30E-01	-0.09	2.73E-01	0.32	4.63E-01	0.19
830.1857@9.344778	830.1857	9.34	NEG	RP				9.92E-01	8.02E-01	0.08	7.26E-01	0.10	7.44E-01	0.10	5.97E-01	0.17
830.186@9.429137	830.1860	9.43	NEG	RP				4.63E-01	9.30E-02	-0.50	4.82E-01	0.20	6.72E-01	0.13	8.42E-01	-0.06
830.1874@0.39661524	830.1874	0.40	NEG	hilic				5.04E-01	1.33E-01	0.42	6.53E-02	0.58	6.27E-01	0.13	2.15E-01	0.40
830.4973@6.0313277	830.4973	6.03	POS	hilic				6.60E-01	9.54E-01	0.02	6.52E-01	0.11	3.62E-01	-0.20	1.42E-01	-0.34
830.4997@10.424333	830.4997	10.42	POS	RP				1.59E-01	3.23E-01	-0.31	5.35E-01	0.19	9.82E-03	0.65	8.49E-01	0.05
830.5629@12.368936	830.5629	12.37	POS	RP				3.04E-02	9.95E-01	0.00	4.07E-02	0.48	5.16E-03	0.84	9.92E-01	0.00
830.6272@0.80675715	830.6272	0.81	POS	hilic				3.79E-01	4.49E-03	-0.60	8.63E-01	-0.05	2.43E-01	-0.33	6.95E-01	-0.09
831.19@0.3968197	831.1900	0.40	NEG	hilic				7.92E-01	2.86E-01	0.30	6.14E-01	0.16	7.14E-01	-0.10	3.23E-01	0.29
831.4186@10.793776	831.4186	10.79	NEG	RP				7.97E-01	4.28E-01	0.17	2.08E-01	0.35	2.28E-01	0.33	8.59E-01	0.05
831.5031@0.7163394	831.5031	0.72	NEG	hilic				4.41E-01	1.62E-01	0.29	5.54E-02	0.54	9.41E-02	0.36	7.96E-02	0.41
831.5033@10.423273	831.5033	10.42	POS	RP				7.88E-02	1.08E-01	-0.33	1.19E-01	0.37	1.04E-01	0.42	5.22E-01	-0.14
831.5049@12.034041	831.5049	12.03	NEG	RP				9.15E-02	4.65E-01	0.15	1.28E-01	0.39	5.57E-01	-0.11	8.27E-03	0.64
831.5752@12.8123	831.5752	12.81	POS	RP				1.74E-01	6.07E-03	0.69	2.67E-02	0.50	4.44E-02	0.49	1.29E-01	0.29
831.5773@12.09013	831.5773	12.09	POS	RP				6.49E-01	1.65E-01	0.45	6.61E-01	0.11	6.39E-01	-0.13	5.68E-01	0.16
831.5777@12.157546	831.5777	12.16	POS	RP	PC 40:7 (18:1_22:6)	20 eV: 184.0735 (100), 832.587 (95)	2	4.30E-01	1.78E-01	0.50	5.36E-01	-0.17	5.21E-01	-0.18	6.99E-01	-0.10
831.5788@0.61034214	831.5788	0.61	POS	hilic				4.40E-01	6.21E-01	-0.15	8.75E-01	0.05	3.08E-01	0.28	1.34E-01	-0.45
831.5973@0.6508382	831.5973	0.65	NEG	hilic				8.32E-01	6.66E-01	0.14	3.38E-01	-0.25	8.89E-01	0.04	6.38E-01	0.15
831.5988@12.90066	831.5988	12.90	NEG	RP				9.29E-01	3.90E-01	-0.25	5.10E-01	-0.16	3.74E-01	-0.23	8.72E-01	-0.05
832.1461@9.2924385	832.1461	9.29	NEG	RP				4.45E-01	4.66E-02	0.65	1.37E-01	0.39	7.34E-01	0.10	2.60E-01	0.30
832.4441@7.313504	832.4441	7.31	POS	RP				1.71E-01	6.75E-03	0.87	5.93E-02	0.52	4.13E-02	0.63	1.91E-01	0.37
832.4749@10.3879175	832.4749	10.39	NEG	RP				6.98E-01	6.39E-01	-0.13	7.70E-01	0.08	2.55E-01	0.34	2.96E-01	0.30
832.5033@11.645743	832.5033	11.65	NEG	RP				2.52E-01	2.98E-01	-0.27	1.22E-01	0.42	4.50E-01	0.23	3.14E-01	-0.28
832.5173@10.698099	832.5173	10.70	POS	RP				3.49E-01	3.07E-02	0.64	2.64E-01	0.33	3.71E-01	0.22	3.08E-02	0.60
832.5281@11.926479	832.5281	11.93	NEG	RP				7.35E-02	9.52E-02	-0.36	5.21E-01	0.16	1.30E-01	0.39	7.16E-02	-0.35
832.6015@0.6508382	832.6015	0.65	NEG	hilic				8.86E-01	5.92E-01	0.17	4.41E-01	-0.21	9.81E-01	0.01	7.40E-01	0.11
832.6421@0.8053737	832.6421	0.81	POS	hilic				1.74E-01	4.02E-01	-0.19	1.63E-01	0.37	1.35E-01	0.47	3.25E-01	-0.23
833.4295@0.46380547	833.4295	0.46	POS	hilic				7.93E-01	7.86E-01	-0.06	5.79E-01	-0.15	1.26E-01	-0.36	8.85E-01	0.04
833.4359@11.038191	833.4359	11.04	NEG	RP				9.07E-02	1.51E-01	0.34	5.03E-01	0.17	8.06E-03	0.66	4.65E-01	-0.14
833.5941@12.703507	833.5941	12.70	POS	RP				7.10E-01	1.09E-01	-0.48	3.05E-01	-0.31	4.19E-01	-0.25	1.61E-01	-0.42

Supplementary table 2: Metabolomics analysis results

								ANOVA	Anorexia vs control		overweight vs control		obese vs control		athletes vs control	
Compound	Mass	Retention Time	Ionization mode	column	Compound_ID	MSMS_spectra	ID_level	P-FDR	P	Estimate	P	Estimate	P	Estimate	P	Estimate
833.7453@10.7828045	833.7453	10.78	POS	hilic				6.21E-01	2.46E-01	-0.36	1.64E-01	-0.41	8.40E-01	0.07	9.31E-01	-0.02
834.1602@9.088309	834.1602	9.09	NEG	RP				3.40E-01	5.36E-01	0.20	5.55E-01	0.17	5.37E-01	-0.17	7.58E-02	-0.52
834.1605@9.293794	834.1605	9.29	NEG	RP				5.08E-01	5.40E-02	0.60	2.09E-01	0.35	2.59E-01	0.31	9.42E-01	0.02
834.1609@9.37659	834.1609	9.38	NEG	RP				7.82E-01	6.84E-01	-0.13	4.39E-01	-0.23	3.46E-01	-0.27	1.31E-01	-0.46
834.1614@0.3965298	834.1614	0.40	NEG	hilic				9.20E-01	8.17E-01	0.06	3.98E-01	-0.26	5.74E-01	-0.16	8.24E-01	-0.07
834.1628@0.3965376	834.1628	0.40	NEG	hilic				8.67E-01	9.80E-01	0.01	4.52E-01	-0.21	5.53E-01	-0.16	6.28E-01	0.15
834.1636@9.587026	834.1636	9.59	NEG	RP				5.36E-01	1.48E-01	-0.41	3.61E-01	0.25	8.29E-01	-0.06	7.00E-01	0.11
834.5176@11.936106	834.5176	11.94	NEG	RP				8.72E-01	4.10E-01	0.28	9.86E-01	0.00	6.80E-01	0.11	6.56E-01	-0.13
834.5247@3.3322213	834.5247	3.33	NEG	hilic				6.12E-01	3.73E-01	0.25	8.68E-01	-0.05	6.96E-01	-0.10	1.51E-01	0.43
834.5989@0.610496	834.5989	0.61	POS	hilic				3.97E-01	5.64E-01	-0.17	5.77E-01	0.16	2.76E-01	0.32	1.52E-01	-0.40
835.4564@11.038561	835.4564	11.04	NEG	RP				5.01E-01	3.08E-01	0.31	9.45E-01	0.02	6.40E-02	0.58	3.59E-01	0.25
835.4835@12.145731	835.4835	12.15	POS	RP				2.05E-01	8.05E-01	-0.09	6.47E-01	0.13	4.23E-03	0.71	8.21E-01	-0.06
835.5375@0.7249501	835.5375	0.72	POS	hilic				9.92E-01	6.93E-01	-0.09	7.87E-01	-0.06	9.78E-01	-0.01	8.77E-01	0.04
835.571@12.293432	835.5710	12.29	NEG	RP				1.84E-02	2.37E-03	-1.02	5.98E-01	0.15	7.86E-01	-0.08	7.73E-01	-0.08
835.5717@0.6299222	835.5717	0.63	NEG	hilic				5.46E-02	8.74E-03	-0.77	3.28E-01	0.27	7.75E-01	-0.08	4.95E-01	0.20
835.6087@12.988363	835.6087	12.99	POS	RP	PC 40:5	20 eV: 836.6136 (100), 184.0741 (60)	2	3.76E-03	1.07E-01	0.52	1.46E-02	-0.62	7.38E-01	0.08	1.27E-02	-0.66
835.6566@0.8103266	835.6566	0.81	POS	hilic				1.26E-01	5.22E-02	-0.38	1.19E-01	0.42	4.13E-01	0.21	8.54E-01	0.04
835.7587@10.790772	835.7587	10.79	POS	hilic				6.29E-01	2.68E-01	-0.33	4.66E-01	0.20	7.42E-01	-0.10	3.25E-01	-0.29
837.4055@0.48887098	837.4055	0.49	POS	hilic				1.61E-01	9.53E-02	-0.50	2.90E-01	-0.28	4.37E-01	-0.19	1.54E-01	0.38
837.5509@11.840229	837.5509	11.84	NEG	RP				1.66E-01	5.72E-02	-0.62	4.82E-01	0.20	3.07E-01	0.29	7.65E-01	-0.09
837.5872@0.62981755	837.5872	0.63	NEG	hilic				1.76E-02	3.65E-04	-0.95	6.28E-01	0.13	5.64E-01	-0.17	6.01E-01	0.15
837.6147@12.912541	837.6147	12.91	POS	RP				1.10E-01	1.76E-02	0.72	8.03E-01	0.05	9.95E-01	0.00	7.48E-02	0.44
837.6219@0.6098786	837.6219	0.61	POS	hilic				2.44E-01	3.27E-02	0.77	7.65E-01	0.08	9.16E-01	-0.03	5.38E-01	0.16
837.6365@3.156103	837.6365	3.16	POS	hilic				5.93E-01	5.44E-02	0.49	9.16E-01	-0.03	6.55E-01	0.12	3.13E-01	0.30
837.831@4.361752	837.8310	4.36	POS	hilic				7.09E-01	1.66E-01	0.38	2.71E-01	0.29	7.28E-01	-0.11	8.94E-01	0.04
838.1021@9.32075	838.1021	9.32	NEG	RP				1.26E-01	3.87E-03	0.62	1.82E-01	-0.37	2.75E-01	0.27	5.74E-01	0.19
838.1036@9.365871	838.1036	9.37	NEG	RP				6.29E-01	6.53E-02	0.56	8.60E-01	0.05	5.55E-01	0.18	3.92E-01	0.28
838.104@9.090114	838.1040	9.09	NEG	RP				5.95E-01	1.94E-01	-0.38	8.15E-02	-0.45	1.49E-01	-0.43	1.01E-01	-0.54
838.1977@0.3975571	838.1977	0.40	NEG	hilic				2.40E-01	2.42E-01	0.25	3.36E-01	0.27	1.28E-01	0.34	7.06E-03	0.75
838.6278@0.60982096	838.6278	0.61	POS	hilic				1.69E-01	1.14E-02	0.84	8.89E-01	0.04	9.36E-01	0.02	5.80E-01	0.17
839.2527@3.4996731	839.2527	3.50	NEG	hilic				7.41E-01	2.01E-01	0.35	9.94E-01	0.00	3.15E-01	0.26	7.97E-01	0.05
839.4157@0.49343178	839.4157	0.49	POS	hilic				4.58E-01	4.25E-01	-0.23	4.41E-01	-0.23	9.29E-01	-0.02	1.40E-01	0.38
839.5666@12.010109	839.5666	12.01	NEG	RP				3.97E-01	3.40E-01	0.34	2.05E-01	0.32	2.88E-02	0.57	7.91E-01	-0.06
839.5669@12.011263	839.5669	12.01	NEG	RP				8.17E-01	4.15E-01	0.29	2.90E-01	0.26	1.89E-01	0.35	8.64E-01	0.04
839.6029@12.697409	839.6029	12.70	NEG	RP				1.21E-01	9.33E-03	-0.84	8.38E-01	0.06	2.92E-01	-0.32	9.01E-01	-0.04
840.1754@0.40714484	840.1754	0.41	NEG	hilic				6.02E-01	4.39E-01	0.20	5.70E-02	0.49	1.54E-01	0.32	1.76E-01	0.38
841.4849@10.459754	841.4849	10.46	POS	RP				4.80E-01	5.36E-01	0.16	5.53E-01	0.17	5.61E-01	-0.16	1.54E-01	-0.45
841.4877@0.40580156	841.4877	0.41	POS	hilic				8.09E-01	8.98E-01	0.04	8.27E-01	0.07	3.75E-01	-0.23	2.90E-01	-0.30
841.5387@11.910076	841.5387	11.91	NEG	RP				9.51E-01	6.37E-01	-0.14	7.42E-01	-0.10	6.56E-01	0.13	6.84E-01	-0.12
841.5396@12.064134	841.5396	12.06	NEG	RP				3.68E-01	9.25E-03	-0.72	1.28E-01	-0.47	1.14E-01	-0.45	1.61E-01	-0.39
841.5406@0.70110786	841.5406	0.70	NEG	hilic				3.58E-02	7.67E-03	-0.53	3.35E-01	0.24	9.07E-02	0.50	6.97E-01	0.09
841.5499@10.558223	841.5499	10.56	POS	RP				9.65E-01	9.57E-01	0.01	6.01E-01	0.16	4.55E-01	0.22	8.16E-01	0.07
841.5814@12.459852	841.5814	12.46	NEG	RP				8.65E-01	5.72E-01	-0.18	6.53E-01	0.11	4.79E-01	0.22	8.45E-01	-0.06
841.5817@12.359541	841.5817	12.36	NEG	RP				1.75E-01	7.42E-01	-0.07	4.55E-01	0.15	6.19E-02	0.44	2.41E-01	-0.20
841.5995@0.5997893	841.5995	0.60	POS	hilic				1.96E-01	1.06E-02	-0.80	2.72E-01	-0.26	1.01E-01	-0.54	7.48E-01	-0.10
841.7152@0.40605	841.7152	0.41	POS	hilic				9.19E-01	6.53E-01	-0.11	4.90E-01	0.19	8.05E-01	0.06	9.51E-01	0.01
842.5759@0.9308889	842.5759	0.93	NEG	hilic				8.30E-01	6.99E-01	-0.10	3.87E-01	-0.22	1.82E-01	-0.33	9.04E-01	-0.03
843.2171@9.077486	843.2171	9.08	POS	RP				5.67E-01	2.86E-01	-0.34	9.19E-01	-0.03	9.81E-01	-0.01	9.89E-02	-0.51
843.5118@12.07564	843.5118	12.08	POS	RP				6.48E-01	3.76E-01	0.25	5.69E-01	0.17	4.79E-01	0.23	2.77E-01	-0.28
843.5178@0.685793	843.5178	0.69	POS	hilic				8.77E-01	3.78E-01	0.20	8.31E-01	0.04	6.98E-01	0.08	6.35E-01	-0.10
843.5262@11.771496	843.5262	11.77	NEG	RP				5.15E-01	1.15E-01	-0.44	4.21E-01	-0.19	3.85E-01	-0.23	5.69E-01	0.17
843.5381@11.916182	843.5381	11.92	NEG	RP				3.33E-01	3.38E-03	-0.55	6.84E-01	0.10	4.85E-01	-0.17	8.96E-01	0.03
843.5536@0.70032	843.5536	0.70	NEG	hilic				6.99E-01	4.29E-01	-0.23	6.75E-01	0.11	3.32E-01	0.28	7.66E-01	-0.07
843.5566@12.296487	843.5566	12.30	NEG	RP				6.27E-02	1.06E-01	0.42	9.21E-03	-0.46	7.32E-01	0.09	9.63E-01	0.01
843.5582@11.56484	843.5582	11.56	NEG	RP				1.38E-01	9.18E-02	-0.33	4.86E-01	0.19	5.66E-02	0.54	2.14E-01	0.31
843.5941@12.748429	843.5941	12.75	NEG	RP				8.05E-01	2.35E-01	0.30	9.65E-01	0.01	9.58E-01	-0.01	4.00E-01	0.22
843.73@0.40696138	843.7300	0.41	POS	hilic				8.05E-01	1.29E-01	-0.44	7.04E-01	-0.12	7.09E-01	-0.10	3.87E-01	-0.22
844.1944@0.3969213	844.1944	0.40	NEG	hilic				2.10E-01	2.28E-01	0.35	7.99E-01	0.07	8.82E-02	0.46	8.67E-03	0.73
844.5615@0.69231033	844.5615	0.69	NEG	hilic				3.29E-01	8.37E-03	-0.52	6.98E-01	0.10	6.12E-01	0.14	9.12E-01	-0.02
844.5629@0.93064904	844.5629	0.93	NEG	hilic				6.46E-01	8.80E-01	0.04	1.84E-01	-0.33	1.97E-01	-0.33	8.53E-01	-0.05
844.6371@3.226662	844.6371	3.23	NEG	hilic				5.42E-01	5.05E-01	-0.20	1.87E-01	0.38	7.63E-01	-0.08	3.87E-01	0.24
845.5406@12.077196	845.5406	12.08	NEG	RP				5.08E-01	7.23E-02	0.61	6.01E-01	0.12	2.91E-01	0.32	2.40E-01	0.32
845.5697@12.801534	845.5697	12.80	NEG	RP				8.46E-05	2.72E-02	-0.66	3.04E-02	0.62	4.11E-04	0.88	3.33E-01	-0.23
845.5718@12.55603	845.5718	12.56	NEG	RP				2.77E-01	3.15E-01	0.24	4.05E-02	-0.57	7.47E-01	-0.10	8.26E-01	-0.07
845.6305@0.59971285	845.6305	0.60	POS	hilic				1.97E-01	7.19E-03	-0.81	3.96E-01	-0.20	9.70E-02	-0.55	6.69E-01	-0.13
845.7469@0.40816054	845.7469	0.41	POS	hilic				7.02E-01	1.57E-01	-0.42	7.97E-01	0.08	3.39E-01	-0.27	4.60E-01	-0.20
845.9535@11.927028	845.9535	11.93	NEG	RP				5.62E-01	1.70E-01	0.34	3.50E-01	-0.16	5.64E-01	0.14	6.11E-01	0.12
846.6362@0.86131704	846.6362	0.86	NEG	hilic				3.63E-01	7.07E-01	-0.07	2.30E-01	0.26	1.87E-01	0.35	3.60E-01	-0.20
847.1752@9.392529	847.1752	9.39	NEG	RP				7.45E-01	9.20E-01	0.03	9.41E-01	-0.02	1.05E-01	0.44	6.21E-01	0.14
847.1754@9.1586	847.1754	9.16	NEG	RP				7.86E-01	1.99E-01	0.41	4.87E-01	0.21	1.83E-01	0.39	3.85E-01	0.28
847.1754@9.40624	847.1754	9.41	NEG	RP				7.54E-01	4.78E-01	0.22	6.81E-01	0.12	1.16E-01	0.41	2.46E-01	0.39
847.1772@9.610366	847.1772	9.61	NEG	RP				6.74E-01	3.97E-01	0.28	3.06E-01	0.23	6.36E-02	0.53	7.35E-01	0.10
847.1806@0.39804345	847.1806	0.40	NEG	hilic				6.93E-01	1.41E-01	0.43	9.26E-01	0.02	5.25E-01	0.16	9.01E-01	-0.03

Supplementary table 2: Metabolomics analysis results

								ANOVA	Anorexia vs control		overweight vs control		obese vs control		athletes vs control	
Compound	Mass	Retention Time	Ionization mode	column	Compound_ID	MSMS_spectra	ID_level	P-FDR	P	Estimate	P	Estimate	P	Estimate	P	Estimate
847.4169@11.04614	847.4169	11.05	NEG	RP				8.66E-01	2.53E-01	0.32	7.92E-01	0.07	2.95E-01	0.28	4.88E-01	0.20
847.4377@0.42069688	847.4377	0.42	POS	hilic				8.84E-01	4.48E-01	0.19	5.31E-01	-0.17	7.98E-01	-0.07	7.69E-01	0.09
847.4585@0.72557974	847.4585	0.73	POS	hilic				7.71E-01	8.47E-01	0.05	3.96E-01	-0.22	1.86E-01	-0.32	9.94E-01	0.00
847.5682@12.562747	847.5682	12.56	NEG	RP				9.46E-01	4.97E-01	0.19	4.78E-01	0.19	4.59E-01	0.22	4.31E-01	0.24
847.5841@13.249421	847.5841	13.25	NEG	RP				9.81E-02	7.50E-01	0.09	2.09E-01	0.37	1.73E-03	0.80	6.63E-01	-0.13
847.6402@0.91985714	847.6402	0.92	NEG	hilic				8.67E-01	2.43E-01	-0.30	7.31E-01	-0.08	6.43E-01	-0.11	3.86E-01	-0.21
847.6445@0.59965795	847.6445	0.60	POS	hilic				3.10E-01	9.02E-03	-0.73	5.00E-01	-0.16	1.66E-01	-0.45	8.10E-01	-0.08
847.7632@10.785958	847.7632	10.79	POS	hilic				4.63E-01	3.50E-01	0.31	9.70E-01	-0.01	2.69E-01	0.33	3.38E-02	0.59
848.5863@13.249347	848.5863	13.25	NEG	RP				2.34E-01	1.43E-02	0.66	2.21E-02	0.54	2.74E-02	0.56	5.98E-02	0.47
848.6188@0.8134999	848.6188	0.81	POS	hilic				5.52E-02	6.45E-01	0.10	6.26E-02	0.44	1.94E-02	0.67	5.06E-01	-0.12
849.2441@10.929486	849.2441	10.93	POS	RP				4.61E-01	8.74E-01	-0.05	1.68E-01	0.45	9.97E-02	0.46	9.64E-01	0.01
849.3863@10.795597	849.3863	10.80	NEG	RP				7.34E-01	8.89E-01	-0.03	3.92E-01	-0.22	8.92E-01	0.04	1.41E-01	-0.35
849.4353@11.040533	849.4353	11.04	NEG	RP				7.71E-01	2.95E-01	0.31	1.68E-01	0.34	2.01E-01	0.36	5.83E-01	0.14
849.5018@10.381013	849.5018	10.38	POS	RP				1.07E-01	9.84E-01	0.01	1.27E-02	0.77	1.89E-02	0.60	6.29E-01	0.15
849.5352@0.46088013	849.5352	0.46	POS	hilic				8.52E-01	7.92E-01	0.07	2.77E-01	-0.29	9.23E-01	-0.03	7.09E-01	-0.11
849.5525@0.64053404	849.5525	0.64	NEG	hilic				7.80E-02	4.74E-03	-0.92	9.75E-01	-0.01	3.09E-01	-0.28	9.94E-02	-0.50
849.6614@10.708771	849.6614	10.71	NEG	RP				5.44E-01	2.23E-01	-0.30	3.50E-01	-0.27	6.63E-01	0.11	9.67E-02	-0.46
849.7757@10.796686	849.7757	10.80	POS	hilic				6.18E-01	4.39E-01	-0.23	4.94E-01	0.20	8.93E-01	-0.04	2.28E-01	0.36
850.1209@9.377232	850.1209	9.38	NEG	RP				4.42E-01	1.39E-01	-0.47	4.28E-01	-0.23	4.33E-01	-0.20	3.62E-02	-0.66
850.1226@9.3401785	850.1226	9.34	NEG	RP				7.32E-01	1.45E-01	-0.40	9.53E-01	-0.02	9.56E-01	-0.02	3.09E-01	-0.34
850.1231@9.089949	850.1231	9.09	NEG	RP				5.30E-01	1.81E-01	-0.42	2.77E-01	-0.34	4.54E-01	-0.21	4.75E-02	-0.62
850.1819@0.4020982	850.1819	0.40	NEG	hilic				7.90E-01	2.38E-01	0.36	9.66E-01	-0.01	9.36E-01	0.02	3.78E-01	0.28
850.4615@0.4642836	850.4615	0.46	POS	hilic				4.54E-01	3.07E-02	-0.53	7.49E-01	0.09	8.37E-01	-0.06	5.91E-01	-0.15
850.6651@0.5997191	850.6651	0.60	POS	hilic				1.94E-01	4.88E-03	-0.79	5.77E-01	-0.13	1.93E-01	-0.42	9.96E-01	0.00
851.4862@11.5773325	851.4862	11.58	NEG	RP				9.42E-01	5.93E-01	0.16	6.36E-01	0.14	3.60E-01	0.26	9.91E-01	0.00
851.5435@11.842406	851.5435	11.84	POS	RP				6.35E-01	4.29E-01	-0.22	2.71E-01	-0.30	7.54E-02	-0.55	3.13E-01	-0.31
851.5466@0.60292256	851.5466	0.60	POS	hilic				5.62E-01	4.17E-02	-0.58	5.59E-01	-0.12	2.52E-01	-0.39	7.19E-01	-0.11
851.567@0.6404525	851.5670	0.64	NEG	hilic				7.96E-02	4.24E-02	-0.63	3.34E-01	0.26	5.39E-01	0.18	1.59E-01	-0.43
851.567@11.911687	851.5670	11.91	NEG	RP				3.57E-01	2.26E-01	-0.37	3.71E-01	-0.27	5.71E-01	-0.17	2.20E-01	0.38
851.568@12.060532	851.5680	12.06	NEG	RP				4.07E-02	3.90E-03	-1.02	8.76E-01	-0.04	2.98E-01	-0.29	7.17E-02	-0.51
851.6034@10.515394	851.6034	10.52	NEG	RP				4.98E-02	2.05E-02	-0.67	7.23E-01	0.10	1.27E-01	0.45	4.31E-01	-0.26
851.8239@4.6169186	851.8239	4.62	NEG	hilic				9.90E-01	7.44E-01	-0.11	8.82E-01	-0.04	6.44E-01	-0.13	5.43E-01	-0.16
852.1186@9.0894375	852.1186	9.09	NEG	RP				6.29E-01	3.53E-01	-0.30	1.99E-01	-0.37	4.26E-01	-0.22	5.77E-02	-0.57
852.1193@9.289076	852.1193	9.29	NEG	RP				5.80E-01	1.16E-01	-0.46	1.67E-01	-0.40	6.59E-01	-0.12	1.03E-01	-0.50
852.1198@9.571207	852.1198	9.57	NEG	RP				5.80E-01	1.14E-01	-0.52	8.63E-01	0.05	6.59E-01	-0.12	3.00E-01	-0.31
852.1911@0.4027113	852.1911	0.40	NEG	hilic				9.85E-01	9.06E-01	0.03	9.65E-01	0.01	5.71E-01	-0.15	9.09E-01	0.04
852.6314@0.8464212	852.6314	0.85	NEG	hilic				9.68E-01	7.09E-01	-0.10	5.92E-01	-0.13	8.83E-01	-0.04	3.75E-01	-0.20
852.6777@0.6001428	852.6777	0.60	POS	hilic				6.09E-01	5.09E-01	0.17	6.59E-01	-0.10	5.16E-01	-0.15	1.69E-01	-0.30
852.9455@6.238932	852.9455	6.24	NEG	hilic				5.75E-01	7.16E-02	0.53	2.95E-01	0.29	6.55E-01	0.11	1.39E-01	0.41
853.1212@9.089526	853.1212	9.09	NEG	RP				6.18E-01	6.31E-01	-0.15	1.32E-01	-0.45	5.23E-01	-0.17	7.71E-02	-0.51
853.1218@9.3779335	853.1218	9.38	NEG	RP				4.51E-01	1.30E-01	-0.47	2.37E-01	-0.34	6.02E-01	-0.13	3.57E-02	-0.63
853.1222@9.573411	853.1222	9.57	NEG	RP				1.22E-01	1.85E-02	-0.73	3.00E-01	-0.33	9.49E-01	0.02	1.96E-02	-0.68
853.123@9.297529	853.1230	9.30	NEG	RP				5.07E-01	7.67E-01	0.09	4.14E-01	-0.24	5.77E-01	-0.15	8.04E-02	-0.56
853.2284@3.3325992	853.2284	3.33	NEG	hilic				7.20E-01	6.36E-01	-0.16	6.32E-01	-0.11	5.33E-01	-0.17	2.79E-01	0.29
853.4217@0.51387686	853.4217	0.51	POS	hilic				7.72E-01	7.62E-01	0.07	2.36E-01	0.30	8.96E-01	0.03	6.54E-01	-0.13
853.4492@0.8632698	853.4492	0.86	POS	hilic				2.10E-01	3.64E-02	-0.46	5.12E-01	0.18	4.69E-01	0.20	1.83E-01	-0.28
853.5616@11.79833	853.5616	11.80	POS	RP				7.11E-01	5.43E-02	-0.52	2.16E-01	-0.34	3.67E-01	-0.26	2.89E-01	-0.31
853.5626@0.6053743	853.5626	0.61	POS	hilic				8.30E-01	4.10E-01	-0.23	5.84E-01	-0.12	2.17E-01	-0.41	8.52E-01	-0.06
853.5766@10.025657	853.5766	10.03	POS	RP				1.28E-01	3.88E-02	-0.74	5.78E-01	-0.16	2.41E-01	-0.32	6.23E-03	-0.78
853.5812@0.64017975	853.5812	0.64	NEG	hilic				7.12E-02	7.91E-01	-0.08	1.66E-01	0.38	2.07E-02	0.72	3.48E-01	-0.24
853.5826@12.263121	853.5826	12.26	NEG	RP	PC 38:5 (18:1_20:4, M+FA)	20 eV: 792.5523 (100), 852.37 (22), 303.2330 (8), 281.2489 (6)	2	3.41E-02	1.44E-01	0.46	8.21E-03	0.76	4.96E-02	0.55	4.02E-01	-0.23
853.5846@12.383857	853.5846	12.38	NEG	RP				6.07E-01	7.51E-01	-0.09	9.31E-01	-0.02	1.85E-01	0.38	4.21E-01	-0.21
853.6573@10.707652	853.6573	10.71	NEG	RP				4.76E-02	5.75E-01	-0.15	6.08E-02	0.55	4.32E-03	0.71	6.38E-01	-0.14
854.5075@10.556853	854.5075	10.56	POS	RP				4.28E-01	5.50E-01	0.19	1.47E-01	0.45	5.12E-02	0.53	5.99E-02	0.62
855.4405@0.5120294	855.4405	0.51	POS	hilic				8.45E-02	5.46E-02	0.48	3.08E-02	-0.32	6.75E-01	0.09	8.92E-01	0.03
855.5747@12.716093	855.5747	12.72	POS	RP				2.09E-03	1.45E-04	-1.12	3.86E-01	-0.24	7.19E-01	0.09	7.67E-03	-0.70
855.5752@11.964357	855.5752	11.96	POS	RP				3.06E-02	3.45E-02	0.55	3.10E-01	-0.26	5.49E-02	-0.58	2.06E-01	-0.40
855.5758@0.60713726	855.5758	0.61	POS	hilic				4.83E-02	9.15E-02	0.47	9.69E-02	-0.39	2.42E-02	-0.64	6.77E-01	-0.13
855.5977@0.63896537	855.5977	0.64	NEG	hilic				6.77E-04	8.91E-01	-0.04	1.25E-02	0.68	1.68E-05	1.18	8.27E-01	-0.06
855.5984@12.796795	855.5984	12.80	NEG	RP	PC 38:4 (18:0_20:4, M+FA)	20 eV: 794.5709 (100), 854.5904 (25), 303.2328 (8), 283.2622 (6)	2	2.81E-04	6.98E-02	-0.52	9.33E-03	0.72	4.51E-04	0.92	8.75E-01	-0.04
856.1975@0.39709076	856.1975	0.40	NEG	hilic				4.41E-01	3.69E-01	0.24	1.56E-01	0.46	7.93E-01	0.07	3.74E-02	0.61
856.4549@11.035155	856.4549	11.04	NEG	RP				3.86E-01	4.60E-01	-0.16	5.33E-01	0.16	5.20E-01	0.17	5.76E-02	-0.39
856.5102@2.908318	856.5102	2.91	POS	hilic				3.83E-01	3.06E-01	0.27	6.30E-01	-0.14	6.31E-01	-0.12	8.96E-02	0.47
856.5146@11.624022	856.5146	11.62	NEG	RP				2.20E-01	1.95E-01	-0.27	3.48E-01	-0.20	3.74E-01	0.25	1.98E-02	-0.42
856.5164@10.5567	856.5164	10.56	POS	RP				4.44E-01	8.58E-01	0.06	1.17E-01	0.49	7.52E-02	0.48	1.03E-01	0.51
856.6013@0.6400807	856.6013	0.64	NEG	hilic				1.08E-02	4.59E-01	-0.20	1.41E-01	0.39	2.39E-03	0.93	6.14E-01	-0.13
857.0542@0.5050312	857.0542	0.51	POS	hilic				7.91E-03	1.05E-02	0.77	8.13E-01	-0.04	2.47E-01	-0.20	9.41E-01	-0.01
857.4918@12.269201	857.4918	12.27	POS	RP				4.12E-01	3.91E-01	0.24	5.61E-01	-0.17	5.01E-01	0.17	7.74E-02	0.54
857.5208@10.556659	857.5208	10.56	POS	RP				2.71E-01	9.17E-01	-0.03	1.17E-01	0.48	8.57E-02	0.46	5.03E-02	0.64
857.6102@0.64063025	857.6102	0.64	NEG	hilic				1.93E-02	8.36E-01	-0.06	2.45E-01	0.32	1.11E-03	0.96	6.94E-01	-0.09
857.6131@13.232777	857.6131	13.23	NEG	RP	PC 38:3 (18:0_20:3, M+FA)	20 eV: 796.5872 (100), 856.6065 (21), 305.2496 (15), 283.2625 (3)	2	3.90E-02	8.97E-01	0.04	1.94E-01	0.39	2.34E-04	0.91	7.38E-01	-0.10
858.2154@9.425047	858.2154	9.43	NEG	RP				7.38E-01	9.86E-01	0.00	1.06E-01	0.41	5.31E-01	0.20	3.84E-01	0.30
858.2162@0.3970597	858.2162	0.40	NEG	hilic				2.58E-01	2.70E-01	0.29	2.33E-02	0.71	6.95E-01	0.11	8.56E-02	0.54

Supplementary table 2: Metabolomics analysis results

								ANOVA	Anorexia vs control		overweight vs control		obese vs control		athletes vs control	
Compound	Mass	Retention Time	Ionization mode	column	Compound_ID	MSMS_spectra	ID_level	P-FDR	P	Estimate	P	Estimate	P	Estimate	P	Estimate
858.4598@11.048452	858.4598	11.05	NEG	RP				6.58E-01	6.55E-01	-0.14	1.21E-01	-0.34	1.95E-01	-0.35	7.76E-01	0.09
858.5253@3.0469606	858.5253	3.05	NEG	hilic				4.79E-01	3.05E-01	0.31	2.78E-01	0.31	5.71E-02	0.54	3.00E-02	0.58
858.5264@2.6620448	858.5264	2.66	POS	hilic				7.64E-01	2.26E-01	0.39	3.16E-01	0.27	1.53E-01	0.41	2.06E-01	0.36
858.6145@0.6415264	858.6145	0.64	NEG	hilic				2.44E-02	5.28E-01	0.17	1.21E-01	0.45	2.36E-04	0.98	7.99E-01	-0.07
859.2197@0.39845437	859.2197	0.40	NEG	hilic				7.01E-01	5.69E-01	0.15	1.36E-01	0.45	9.28E-01	0.02	2.94E-01	0.32
859.4895@12.260125	859.4895	12.26	POS	RP				1.11E-01	3.24E-01	0.32	6.79E-01	-0.11	1.04E-02	0.67	3.09E-02	0.59
859.5257@10.556372	859.5257	10.56	POS	RP				4.91E-01	5.61E-01	-0.17	1.62E-01	0.40	2.24E-01	0.36	2.97E-01	0.33
859.5784@12.381829	859.5784	12.38	NEG	RP				5.30E-01	2.83E-01	-0.27	1.69E-01	-0.41	7.19E-02	-0.51	6.30E-01	-0.12
859.6067@0.60747916	859.6067	0.61	POS	hilic				3.76E-03	3.99E-02	0.61	1.08E-01	-0.39	3.26E-03	-0.80	7.68E-01	-0.09
860.4938@12.263226	860.4938	12.26	POS	RP				7.27E-01	9.25E-01	0.03	2.10E-01	-0.27	7.80E-01	0.07	4.49E-01	0.21
861.4873@11.769881	861.4873	11.77	NEG	RP				4.57E-01	3.25E-01	-0.26	2.41E-01	-0.30	9.76E-01	-0.01	3.11E-01	0.32
861.4878@12.266169	861.4878	12.27	POS	RP				7.58E-01	9.35E-01	-0.02	1.53E-01	-0.31	6.37E-01	0.11	7.21E-01	-0.10
861.524@0.7235001	861.5240	0.72	NEG	hilic				5.29E-02	5.21E-01	-0.19	6.96E-03	-0.64	5.17E-01	-0.19	1.28E-01	0.45
861.5869@12.39571	861.5869	12.40	NEG	RP				5.00E-01	3.65E-02	-0.39	8.71E-01	-0.04	7.44E-01	0.08	6.23E-01	0.13
861.5888@0.62240183	861.5888	0.62	NEG	hilic				3.68E-02	6.82E-04	-1.06	5.48E-01	-0.17	1.50E-01	-0.45	5.45E-01	-0.18
861.6144@11.520714	861.6144	11.52	NEG	RP				2.11E-01	7.41E-01	-0.09	4.11E-02	0.51	9.45E-01	-0.02	1.15E-01	0.47
861.6187@11.511061	861.6187	11.51	POS	RP				2.15E-01	4.42E-02	-0.62	2.62E-01	0.31	6.39E-01	-0.14	8.07E-01	-0.08
861.6237@0.6061743	861.6237	0.61	POS	hilic				1.05E-02	9.29E-02	0.58	1.21E-01	-0.36	6.29E-03	-0.72	9.80E-01	-0.01
861.8538@4.5708737	861.8538	4.57	NEG	hilic				8.42E-01	2.50E-01	0.36	7.38E-01	0.10	7.84E-01	-0.07	7.24E-01	0.10
862.1685@9.084959	862.1685	9.08	NEG	RP				4.64E-01	6.56E-01	0.12	4.66E-01	-0.21	1.01E-01	-0.45	5.52E-01	0.18
862.1809@0.39681107	862.1809	0.40	NEG	hilic				7.31E-01	8.89E-01	0.04	3.82E-01	0.24	2.94E-01	-0.27	8.60E-01	0.05
862.6278@0.60691583	862.6278	0.61	POS	hilic				4.82E-01	2.40E-01	0.40	4.32E-01	-0.19	5.67E-01	-0.13	4.81E-01	0.20
862.8577@4.5692334	862.8577	4.57	NEG	hilic				5.24E-01	7.37E-01	0.10	4.54E-01	-0.19	7.24E-02	-0.51	4.12E-01	-0.24
863.4966@12.074282	863.4966	12.07	NEG	RP				8.05E-01	2.15E-01	0.39	9.02E-01	0.03	5.21E-01	0.18	7.09E-01	0.09
863.5288@0.721301	863.5288	0.72	NEG	hilic				2.97E-01	5.71E-01	-0.17	3.88E-02	-0.55	8.45E-01	-0.05	4.72E-01	0.21
863.5417@12.721557	863.5417	12.72	NEG	RP				5.22E-01	9.80E-01	0.01	3.01E-01	-0.24	5.02E-02	-0.44	9.12E-01	0.03
863.5998@12.58099	863.5998	12.58	NEG	RP				2.23E-01	2.46E-02	-0.74	7.15E-01	-0.11	1.26E-01	-0.50	6.86E-01	-0.11
863.6028@0.622734	863.6028	0.62	NEG	hilic				3.11E-02	4.18E-04	-1.07	2.86E-01	-0.28	1.46E-01	-0.47	7.47E-01	-0.09
863.6447@10.70763	863.6447	10.71	NEG	RP				6.67E-01	9.12E-01	-0.03	4.07E-01	0.24	6.37E-01	0.13	1.46E-01	0.49
864.6432@0.60763973	864.6432	0.61	POS	hilic				5.68E-02	2.72E-01	0.33	2.68E-01	-0.26	5.47E-03	-0.70	6.86E-01	-0.11
865.2195@9.380786	865.2195	9.38	POS	RP				8.68E-01	6.45E-01	0.14	3.39E-01	0.25	2.45E-01	0.35	9.07E-01	0.04
865.5826@12.340646	865.5826	12.34	NEG	RP				4.38E-01	1.66E-02	-0.58	5.60E-01	-0.15	7.67E-01	-0.08	2.07E-01	-0.30
865.583@12.35058	865.5830	12.35	NEG	RP				8.12E-02	4.81E-03	-0.90	6.00E-01	-0.15	9.58E-01	0.01	1.95E-01	-0.35
865.6131@0.6251896	865.6131	0.63	NEG	hilic				1.99E-01	2.65E-02	-0.61	7.87E-01	0.07	6.84E-01	-0.12	3.60E-01	0.27
866.1679@0.39712122	866.1679	0.40	NEG	hilic				2.14E-01	8.24E-01	0.06	1.46E-01	0.39	1.44E-01	-0.30	1.83E-01	0.34
866.2201@0.40338144	866.2201	0.40	NEG	hilic				7.61E-01	1.45E-01	0.42	9.36E-01	0.02	3.04E-01	0.28	5.87E-01	0.15
866.4725@11.602084	866.4725	11.60	NEG	RP				9.25E-01	4.36E-01	0.18	9.10E-01	0.03	3.84E-01	0.23	7.86E-01	0.06
866.5366@3.963172	866.5366	3.96	POS	hilic				4.78E-01	5.08E-01	0.20	1.96E-01	0.41	2.17E-02	0.65	4.50E-01	0.22
866.5855@12.348756	866.5855	12.35	NEG	RP				2.36E-01	5.19E-01	-0.15	3.03E-01	-0.21	1.20E-01	0.42	5.53E-01	-0.13
867.5458@0.9356508	867.5458	0.94	POS	hilic				7.50E-01	5.80E-01	0.18	3.04E-01	-0.30	6.56E-01	0.12	8.55E-01	0.05
867.548@0.9302542	867.5480	0.93	POS	hilic				8.97E-01	7.28E-01	0.11	4.51E-01	-0.20	9.54E-01	-0.02	6.34E-01	0.14
867.616@0.5977609	867.6160	0.60	POS	hilic				9.65E-01	8.62E-01	0.04	5.78E-01	0.13	9.46E-01	-0.02	7.32E-01	-0.09
867.6165@0.59771794	867.6165	0.60	POS	hilic				9.84E-01	8.11E-01	0.06	9.06E-01	-0.03	8.86E-01	0.04	6.56E-01	-0.11
867.6369@10.707575	867.6369	10.71	NEG	RP				1.71E-01	3.19E-01	0.29	7.38E-03	0.80	1.26E-02	0.65	3.26E-01	0.32
867.7304@0.40857995	867.7304	0.41	POS	hilic				8.58E-01	1.56E-01	-0.38	8.86E-01	-0.05	4.94E-01	-0.18	7.38E-01	-0.09
868.2009@0.40166673	868.2009	0.40	NEG	hilic				6.84E-01	5.23E-01	0.19	3.26E-01	-0.22	8.72E-01	-0.04	3.04E-01	-0.28
869.4274@0.41920862	869.4274	0.42	POS	hilic				9.70E-01	5.66E-01	-0.18	9.38E-01	-0.02	9.22E-01	-0.03	7.50E-01	0.10
869.4487@12.38073	869.4487	12.38	NEG	RP				5.79E-02	1.01E-01	-0.51	1.28E-01	0.40	7.81E-02	0.48	3.38E-01	0.29
869.5397@11.880669	869.5397	11.88	NEG	RP				5.98E-01	3.04E-01	0.32	3.87E-01	-0.25	3.76E-01	0.28	8.30E-01	0.07
869.544@0.6926791	869.5440	0.69	NEG	hilic				1.57E-01	5.09E-01	-0.19	1.71E-02	-0.59	2.12E-02	-0.65	8.03E-01	0.08
869.6284@0.5966369	869.6284	0.60	POS	hilic				9.91E-02	4.51E-03	-0.92	2.48E-01	-0.28	6.67E-02	-0.58	6.16E-01	-0.15
869.7458@0.40796295	869.7458	0.41	POS	hilic				9.03E-01	2.90E-01	-0.31	8.87E-01	-0.05	3.67E-01	-0.24	5.01E-01	-0.19
870.1892@0.40519658	870.1892	0.41	NEG	hilic				2.58E-01	8.43E-02	0.44	1.25E-02	0.77	2.54E-01	0.31	1.04E-01	0.56
870.6047@10.028415	870.6047	10.03	POS	RP				3.64E-02	4.75E-01	-0.22	1.26E-01	0.44	5.42E-01	0.17	3.06E-02	-0.66
870.9493@6.239548	870.9493	6.24	NEG	hilic				7.20E-01	5.10E-01	0.18	6.04E-01	-0.13	2.98E-01	0.30	3.17E-01	0.29
871.5545@0.69189787	871.5545	0.69	NEG	hilic				1.51E-02	6.38E-01	-0.14	2.31E-02	-0.57	3.38E-03	-0.80	2.87E-01	0.33
871.5545@12.26469	871.5545	12.26	NEG	RP				5.90E-01	3.54E-01	0.25	8.75E-01	-0.05	7.42E-01	-0.08	1.20E-01	0.46
871.5887@10.562298	871.5887	10.56	NEG	RP				1.57E-01	7.14E-02	-0.54	4.40E-01	0.23	1.74E-01	0.37	8.83E-01	-0.04
871.6451@0.59723914	871.6451	0.60	POS	hilic				2.36E-01	1.33E-02	-0.75	2.61E-01	-0.27	6.23E-02	-0.61	6.12E-01	-0.17
871.7615@0.40769628	871.7615	0.41	POS	hilic				7.41E-01	1.30E-01	-0.46	6.46E-01	-0.14	1.56E-01	-0.37	3.32E-01	-0.26
872.1957@9.08601	872.1957	9.09	NEG	RP				9.61E-01	7.28E-01	0.09	8.23E-01	0.07	3.72E-01	0.25	5.12E-01	0.19
872.1963@8.8607	872.1963	8.86	NEG	RP				7.32E-01	4.11E-01	0.25	4.08E-01	0.20	4.58E-01	-0.20	8.02E-01	-0.07
872.1993@0.4054054	872.1993	0.41	NEG	hilic				5.47E-01	1.94E-01	0.35	9.77E-02	0.51	6.49E-01	0.12	1.28E-01	0.49
872.4368@11.036392	872.4368	11.04	NEG	RP				3.69E-01	3.46E-01	0.31	3.82E-02	0.62	3.25E-02	0.59	6.90E-02	0.53
872.5589@0.69468826	872.5589	0.69	NEG	hilic				1.23E-01	6.33E-01	-0.16	1.03E-02	-0.59	1.02E-02	-0.60	6.49E-01	0.13
872.5821@0.98993754	872.5821	0.99	NEG	hilic				2.70E-01	2.59E-01	-0.25	1.88E-01	0.32	1.70E-01	0.35	7.96E-01	-0.06
873.2023@0.40486324	873.2023	0.40	NEG	hilic				2.95E-01	5.81E-02	0.54	2.75E-02	0.67	6.95E-01	0.11	1.67E-01	0.45
873.4414@11.037478	873.4414	11.04	NEG	RP				4.31E-01	4.09E-01	0.19	8.45E-02	0.45	1.15E-01	0.35	2.90E-02	0.51
873.5518@11.611102	873.5518	11.61	NEG	RP				2.71E-01	8.46E-01	-0.05	1.73E-01	-0.30	5.85E-01	0.14	1.63E-02	-0.44
873.5702@12.673383	873.5702	12.67	NEG	RP				8.78E-02	7.20E-03	0.83	5.76E-01	0.16	4.46E-01	-0.21	3.53E-01	0.29
873.6611@0.59504336	873.6611	0.60	POS	hilic				2.11E-01	1.38E-02	-0.76	3.13E-01	-0.25	7.31E-02	-0.60	7.17E-01	-0.11
873.7776@0.4096314	873.7776	0.41	POS	hilic				8.60E-01	2.11E-01	-0.37	5.81E-01	-0.18	2.53E-01	-0.28	2.93E-01	-0.30
874.6522@10.70062	874.6522	10.70	NEG	RP				6.07E-01	6.70E-02	-0.41	8.12E-01	0.06	7.92E-01	-0.07	8.20E-01	0.05

Supplementary table 2: Metabolomics analysis results

								ANOVA	Anorexia vs control		overweight vs control		obese vs control		athletes vs control	
Compound	Mass	Retention Time	Ionization mode	column	Compound_ID	MSMS_spectra	ID_level	P-FDR	P	Estimate	P	Estimate	P	Estimate	P	Estimate
875.4931@0.7524446	875.4931	0.75	NEG	hilic				3.53E-01	6.83E-02	0.54	2.51E-01	0.27	7.10E-01	0.09	3.18E-02	0.54
875.4991@11.978196	875.4991	11.98	NEG	RP				2.32E-02	2.01E-01	-0.38	1.02E-01	0.37	8.09E-02	0.52	1.62E-01	-0.37
875.5665@11.834019	875.5665	11.83	NEG	RP				1.89E-01	1.43E-01	-0.46	3.12E-02	-0.53	2.11E-02	-0.74	8.94E-01	-0.04
875.5681@0.63052976	875.5681	0.63	NEG	hilic				4.94E-01	5.71E-01	-0.17	9.23E-01	0.03	3.66E-01	0.27	1.67E-01	-0.42
875.5932@3.2918465	875.5932	3.29	NEG	hilic				9.94E-01	6.08E-01	0.14	9.61E-01	0.01	8.80E-01	0.04	8.04E-01	0.05
875.6215@0.75507706	875.6215	0.76	NEG	hilic				3.49E-02	7.78E-02	-0.31	6.82E-01	0.09	4.92E-02	-0.33	1.03E-01	0.42
875.6762@0.5953998	875.6762	0.60	POS	hilic				2.71E-01	2.02E-02	-0.70	3.77E-01	-0.22	9.84E-02	-0.55	8.63E-01	-0.05
875.793@10.7906065	875.7930	10.79	POS	hilic				7.82E-01	8.34E-01	-0.08	1.91E-01	0.34	9.47E-01	0.02	3.66E-01	0.23
876.1791@0.3981793	876.1791	0.40	NEG	hilic				6.35E-01	2.48E-01	0.36	9.69E-02	0.51	2.54E-01	0.30	1.25E-01	0.46
876.4695@1.361485	876.4695	1.36	POS	hilic				8.27E-01	8.58E-01	-0.04	4.05E-01	0.21	3.77E-01	0.22	3.57E-01	0.27
876.5558@0.9301839	876.5558	0.93	NEG	hilic				2.10E-03	1.98E-02	-0.79	3.65E-02	0.45	1.74E-01	0.38	6.65E-02	-0.44
876.6714@10.768625	876.6714	10.77	NEG	RP				2.72E-02	5.66E-02	-0.48	1.08E-01	0.47	2.01E-02	0.62	6.98E-01	0.12
877.446@12.380446	877.4460	12.38	NEG	RP				1.61E-01	1.68E-01	-0.34	1.82E-01	0.35	1.02E-01	0.44	3.63E-01	0.26
877.4661@11.883644	877.4661	11.88	POS	RP				9.75E-02	4.10E-01	-0.22	3.74E-01	-0.22	2.71E-02	0.53	2.22E-01	-0.35
877.5009@11.652018	877.5009	11.65	NEG	RP				2.64E-01	5.09E-01	0.18	4.65E-02	0.52	6.50E-01	0.12	4.02E-01	-0.18
877.5624@0.6002638	877.5624	0.60	POS	hilic				3.55E-01	5.65E-01	0.17	5.43E-02	-0.44	2.12E-01	-0.32	2.09E-01	-0.36
877.57@3.0316436	877.5700	3.03	POS	hilic				9.07E-01	4.29E-01	-0.26	4.01E-01	-0.21	4.10E-01	-0.26	9.49E-01	-0.02
877.5701@3.0212	877.5701	3.02	POS	hilic				9.32E-01	4.28E-01	-0.26	8.60E-01	-0.04	6.02E-01	-0.18	9.05E-01	0.03
877.5822@12.113346	877.5822	12.11	NEG	RP	PC 40:7 (18:1_22:6, M+FA)	20 eV: 816.5530 (100), 876.5723 (38), 327.2376 (10), 281.2444 (7)	2	3.40E-02	8.66E-02	0.52	2.15E-01	-0.35	3.50E-02	-0.57	7.02E-01	0.09
877.5829@12.148766	877.5829	12.15	NEG	RP				8.12E-01	6.39E-01	0.19	4.34E-01	-0.20	4.52E-01	-0.19	4.13E-01	-0.22
877.6906@0.59236753	877.6906	0.59	POS	hilic				3.62E-01	2.75E-02	-0.64	7.59E-01	-0.08	1.75E-01	-0.44	9.60E-01	-0.02
878.5611@10.703062	878.5611	10.70	POS	RP				2.00E-01	6.84E-01	0.12	2.74E-01	0.34	7.71E-03	0.80	8.48E-01	0.06
878.6969@0.59504396	878.6969	0.60	POS	hilic				2.31E-01	1.10E-02	-0.72	7.29E-01	-0.09	1.32E-01	-0.48	9.29E-01	-0.03
879.5656@3.0392475	879.5656	3.04	POS	hilic				9.23E-01	3.09E-01	-0.32	4.80E-01	-0.21	7.91E-01	-0.08	5.81E-01	-0.17
879.5753@0.60049176	879.5753	0.60	POS	hilic				5.89E-02	5.34E-02	0.62	2.73E-01	-0.23	2.04E-01	-0.28	6.81E-01	-0.10
879.5981@0.63053143	879.5981	0.63	NEG	hilic				2.61E-01	5.66E-02	-0.59	8.44E-01	-0.05	4.61E-01	0.22	1.92E-01	-0.36
879.5985@12.677964	879.5985	12.68	NEG	RP	PC 40:6 (18:0_22:6, M+FA)	20 eV: 818:5691 (100), 878.5883 (25), 327.2326 (9)	2	1.80E-02	7.63E-03	-0.79	7.66E-01	0.09	1.57E-01	0.38	1.18E-01	-0.42
879.6102@1.0158193	879.6102	1.02	NEG	hilic				7.61E-01	9.32E-01	0.02	4.09E-01	0.21	5.16E-01	-0.17	4.19E-01	0.23
880.1515@9.612452	880.1515	9.61	NEG	RP				1.99E-01	4.00E-01	-0.29	5.96E-03	-0.81	5.58E-01	-0.13	5.68E-01	-0.17
880.5076@2.6591601	880.5076	2.66	POS	hilic				9.02E-01	5.97E-01	0.17	9.57E-01	0.01	7.63E-01	-0.08	3.71E-01	0.25
880.5604@10.703011	880.5604	10.70	POS	RP				1.45E-01	6.53E-01	0.12	4.96E-02	0.59	5.64E-03	0.79	6.78E-01	0.14
880.6013@0.6306249	880.6013	0.63	NEG	hilic				1.63E-01	5.22E-01	-0.18	8.50E-01	0.06	9.82E-02	0.47	1.01E-01	-0.48
880.7697@11.684282	880.7697	11.68	NEG	RP				9.27E-01	3.29E-01	0.31	4.84E-01	0.19	2.69E-01	0.23	5.72E-01	0.17
881.4277@0.4913036	881.4277	0.49	POS	hilic				9.11E-01	9.54E-01	-0.02	5.47E-01	0.17	8.33E-01	-0.06	4.32E-01	0.25
881.4818@12.008508	881.4818	12.01	POS	RP				2.81E-01	9.36E-02	-0.47	6.49E-02	-0.44	1.07E-01	-0.42	8.37E-01	0.06
881.4882@12.14205	881.4882	12.14	POS	RP				4.67E-02	8.72E-01	0.05	2.38E-02	0.64	1.02E-03	0.82	9.49E-01	-0.02
881.5691@0.6176982	881.5691	0.62	POS	hilic				9.84E-01	7.14E-01	-0.10	5.84E-01	-0.14	7.21E-01	-0.09	1.00E+00	0.00
881.5918@0.6005146	881.5918	0.60	POS	hilic				2.43E-01	3.55E-01	0.31	1.91E-01	-0.31	5.55E-02	-0.45	4.82E-01	-0.18
881.6128@12.94792	881.6128	12.95	NEG	RP				1.37E-03	1.70E-05	1.38	1.78E-02	0.67	5.90E-02	0.46	5.27E-01	0.18
882.1683@0.39650586	882.1683	0.40	NEG	hilic				8.19E-01	3.22E-01	0.29	1.75E-01	0.36	6.12E-01	0.14	3.48E-01	0.28
882.4295@0.73414046	882.4295	0.73	POS	hilic				8.52E-01	4.82E-01	-0.18	8.89E-01	-0.04	1.72E-01	-0.33	7.98E-01	-0.08
882.5238@2.781183	882.5238	2.78	POS	hilic				1.36E-02	2.96E-01	0.30	2.79E-02	-0.53	1.06E-02	-0.63	3.00E-01	0.32
882.5249@2.9765666	882.5249	2.98	NEG	hilic				5.01E-01	9.51E-02	0.50	6.85E-01	0.09	7.76E-01	-0.07	8.12E-01	0.06
882.5294@11.666073	882.5294	11.67	NEG	RP				3.71E-01	8.97E-01	-0.04	6.92E-01	0.10	6.79E-01	-0.10	1.02E-02	-0.52
882.5318@0.9839458	882.5318	0.98	NEG	hilic				8.01E-01	8.44E-01	-0.06	4.32E-01	0.18	3.26E-01	0.29	3.13E-01	0.30
882.5575@10.702982	882.5575	10.70	POS	RP				5.69E-02	2.44E-02	0.61	3.20E-02	0.59	8.89E-03	0.74	7.48E-01	-0.11
883.3896@0.887	883.3896	0.89	POS	hilic				9.92E-01	9.29E-01	0.03	8.69E-01	-0.04	6.22E-01	-0.11	9.28E-01	0.02
883.4388@0.4924952	883.4388	0.49	POS	hilic				7.88E-01	7.78E-01	-0.08	6.33E-01	0.14	7.58E-01	-0.09	2.59E-01	0.33
883.4888@12.14481	883.4888	12.14	POS	RP				1.36E-02	3.87E-01	-0.27	2.63E-02	0.65	5.71E-03	0.79	9.52E-01	-0.02
883.5029@12.430425	883.5029	12.43	POS	RP				9.61E-01	6.76E-01	0.13	8.86E-01	-0.05	5.47E-01	0.17	8.63E-01	-0.05
883.6036@0.6002842	883.6036	0.60	POS	hilic				8.18E-03	3.25E-01	0.33	3.41E-02	-0.50	7.11E-04	-0.92	5.53E-01	-0.16
884.1695@9.216968	884.1695	9.22	NEG	RP				9.16E-01	2.97E-01	0.30	9.53E-01	0.02	9.50E-01	-0.02	9.28E-01	-0.03
884.17@9.623716	884.1700	9.62	NEG	RP				3.35E-01	2.23E-02	0.71	5.75E-02	0.52	1.79E-01	0.38	6.28E-01	0.15
884.1706@9.459597	884.1706	9.46	NEG	RP				9.25E-01	4.91E-01	0.18	3.05E-01	0.33	5.27E-01	0.17	6.10E-01	0.17
884.1715@0.39646417	884.1715	0.40	NEG	hilic				8.07E-01	1.18E-01	0.42	2.11E-01	0.34	3.40E-01	0.27	6.02E-01	0.18
884.5403@2.918262	884.5403	2.92	NEG	hilic				7.42E-01	2.45E-01	-0.36	7.23E-01	0.10	9.59E-01	-0.01	4.06E-01	-0.24
885.388@0.881753	885.3880	0.88	POS	hilic				5.55E-01	4.29E-01	0.24	5.66E-01	-0.14	1.08E-01	-0.37	9.54E-01	0.02
885.4508@1.3470869	885.4508	1.35	POS	hilic				3.13E-01	9.55E-02	0.44	9.94E-01	0.00	7.74E-01	0.05	9.48E-01	0.01
885.5038@12.418986	885.5038	12.42	POS	RP				6.63E-01	1.96E-01	0.37	5.13E-01	-0.21	5.75E-01	0.16	9.31E-01	-0.03
885.5041@12.414392	885.5041	12.41	POS	RP				8.48E-01	8.63E-01	-0.05	3.66E-01	-0.28	6.42E-01	0.13	9.45E-01	0.02
885.5178@12.947144	885.5178	12.95	POS	RP				3.80E-01	7.52E-01	-0.09	1.53E-02	-0.62	8.65E-01	-0.05	9.40E-01	0.02
885.5209@0.71332383	885.5209	0.71	NEG	hilic				5.82E-01	2.89E-01	-0.35	1.80E-01	-0.37	7.06E-01	0.11	8.83E-01	0.04
885.5665@12.548561	885.5665	12.55	NEG	RP				6.01E-02	3.97E-02	0.57	8.00E-01	-0.05	1.84E-01	-0.20	8.23E-01	-0.04
885.6186@3.2077196	885.6186	3.21	NEG	hilic				6.89E-01	1.82E-01	0.47	7.57E-01	-0.08	7.42E-01	0.10	6.51E-01	0.12
885.6193@3.1935036	885.6193	3.19	NEG	hilic				1.73E-01	1.84E-02	0.75	6.01E-01	-0.15	9.47E-01	0.02	8.22E-01	0.06
886.2003@0.40271097	886.2003	0.40	NEG	hilic				5.22E-01	3.34E-02	0.59	2.19E-01	0.32	1.36E-01	0.37	1.15E-01	0.45
886.5556@2.917727	886.5556	2.92	NEG	hilic				7.55E-01	2.25E-01	-0.38	7.49E-01	0.10	9.41E-01	-0.02	4.57E-01	-0.21
886.5572@2.5528421	886.5572	2.55	POS	hilic				7.27E-01	1.82E-01	-0.43	7.76E-01	0.08	5.07E-01	-0.20	5.52E-01	-0.18
887.4357@0.42370424	887.4357	0.42	POS	hilic				5.84E-01	2.40E-01	0.29	6.71E-02	0.47	1.40E-01	0.34	1.04E-01	0.45
887.4981@11.868529	887.4981	11.87	NEG	RP				7.65E-01	3.71E-01	0.27	5.31E-01	-0.16	8.62E-01	0.05	4.56E-01	0.22
887.5197@12.935147	887.5197	12.94	POS	RP				8.44E-01	4.39E-01	-0.24	1.88E-01	-0.40	5.59E-01	-0.15	4.85E-01	-0.21
887.5344@0.7131238	887.5344	0.71	NEG	hilic				4.57E-01	7.21E-01	-0.12	8.13E-02	-0.48	4.18E-01	0.22	8.95E-01	-0.04
887.561@2.9185703	887.5610	2.92	NEG	hilic				4.66E-01	1.33E-01	-0.45	3.72E-01	0.26	9.82E-01	0.01	7.61E-01	-0.08

Supplementary table 2: Metabolomics analysis results

Compound	Mass	Retention Time	Ionization mode	column	Compound_ID	MSMS_spectra	ID_level	ANOVA P-FDR	Anorexia vs control P	Anorexia vs control Estimate	overweight vs control P	overweight vs control Estimate	obese vs control P	obese vs control Estimate	athletes vs control P	athletes vs control Estimate
887.6241@0.87322015	887.6241	0.87	NEG	hilic				1.00E-01	2.49E-01	-0.33	4.89E-01	0.17	3.75E-01	-0.23	6.27E-02	0.60
887.6304@10.704789	887.6304	10.70	POS	RP				3.91E-01	3.59E-01	0.22	3.89E-02	0.57	6.91E-02	0.49	6.32E-01	0.13
888.5375@10.722477	888.5375	10.72	NEG	RP				7.07E-02	3.21E-02	-0.68	1.34E-01	0.40	5.70E-01	0.15	7.38E-01	-0.10
889.5393@10.722395	889.5393	10.72	NEG	RP				4.60E-01	2.00E-01	-0.30	2.48E-01	0.31	3.42E-01	0.25	8.77E-01	-0.04
889.5575@12.95113	889.5575	12.95	NEG	RP				4.57E-01	4.60E-01	-0.25	8.45E-02	-0.47	1.38E-02	-0.61	4.37E-01	-0.23
889.6171@3.2226305	889.6171	3.22	NEG	hilic				5.49E-02	2.82E-01	0.30	5.37E-01	0.17	6.38E-01	-0.14	4.33E-03	0.89
890.2023@0.3964443	890.2023	0.40	NEG	hilic				7.64E-01	3.10E-01	0.27	1.38E-01	0.46	4.34E-01	0.23	2.72E-01	0.33
890.4734@10.706617	890.4734	10.71	POS	RP				2.12E-01	1.22E-03	0.89	1.92E-01	0.39	2.64E-01	0.29	2.94E-01	0.35
890.5168@12.264924	890.5168	12.26	NEG	RP				8.43E-01	5.84E-01	-0.18	8.33E-01	0.06	6.66E-01	-0.11	4.12E-01	0.25
891.5175@11.647751	891.5175	11.65	NEG	RP				9.55E-01	6.15E-01	0.10	9.32E-01	-0.01	5.38E-01	0.13	5.38E-01	0.11
891.5328@12.68288	891.5328	12.68	NEG	RP				1.61E-01	1.99E-02	0.71	9.98E-01	0.00	9.04E-01	0.03	1.37E-01	0.43
891.5346@12.676232	891.5346	12.68	NEG	RP				1.64E-01	1.14E-02	0.83	1.17E-01	0.42	9.92E-01	0.00	5.17E-01	0.22
891.6058@10.705028	891.6058	10.71	POS	RP				5.89E-01	4.16E-01	0.21	1.38E-01	0.48	3.57E-01	0.28	7.99E-01	-0.08
891.7301@0.4050701	891.7301	0.41	POS	hilic				9.05E-01	3.46E-01	-0.27	5.77E-01	-0.14	3.77E-01	-0.21	8.18E-01	-0.05
892.184@0.39636025	892.1840	0.40	NEG	hilic				6.65E-01	2.39E-01	0.33	1.73E-01	0.44	7.05E-01	0.10	1.49E-01	0.44
892.5348@12.681301	892.5348	12.68	NEG	RP				2.91E-02	1.24E-03	0.99	6.41E-01	0.13	6.36E-01	-0.12	5.63E-01	0.17
893.434@0.4970533	893.4340	0.50	POS	hilic				8.45E-01	4.26E-01	-0.25	7.58E-01	-0.08	3.36E-01	-0.25	7.33E-01	0.09
893.5388@0.70444053	893.5388	0.70	NEG	hilic				5.32E-01	1.94E-01	-0.41	4.14E-01	-0.24	4.88E-01	0.20	7.93E-01	0.08
893.5403@11.863316	893.5403	11.86	NEG	RP				2.61E-01	4.42E-01	0.21	5.90E-01	0.13	9.90E-02	0.46	2.01E-01	-0.34
893.6315@10.708798	893.6315	10.71	NEG	RP				6.90E-01	1.58E-01	-0.41	5.11E-01	-0.18	6.76E-01	-0.11	1.16E-01	-0.41
893.7454@0.40595943	893.7454	0.41	POS	hilic				7.57E-01	2.23E-01	-0.37	1.99E-01	-0.39	1.63E-01	-0.37	4.07E-01	-0.21
894.1305@9.010946	894.1305	9.01	NEG	RP				6.02E-01	2.08E-01	-0.39	4.89E-01	-0.19	8.57E-01	-0.06	6.20E-02	-0.53
894.1981@9.625752	894.1981	9.63	NEG	RP				5.53E-01	3.60E-01	-0.26	6.02E-01	0.16	5.72E-01	0.16	2.45E-01	-0.35
894.1982@9.216319	894.1982	9.22	NEG	RP				7.58E-01	2.76E-01	-0.29	8.30E-01	0.07	8.84E-01	0.04	3.25E-01	-0.32
894.1983@9.449247	894.1983	9.45	NEG	RP				7.49E-01	3.42E-01	-0.29	7.22E-01	0.10	3.87E-01	0.24	7.26E-01	0.12
894.2002@0.39635882	894.2002	0.40	NEG	hilic				5.18E-01	1.90E-01	0.36	6.81E-02	0.59	6.09E-01	0.14	1.98E-01	0.42
894.4867@11.656712	894.4867	11.66	NEG	RP				1.55E-01	3.90E-01	0.20	2.52E-02	0.57	3.16E-02	0.67	8.41E-01	0.05
894.6386@10.566459	894.6386	10.57	NEG	RP				5.01E-01	1.27E-01	-0.34	4.76E-01	0.20	4.08E-01	0.21	5.62E-01	0.16
895.2026@0.39636025	895.2026	0.40	NEG	hilic				7.31E-01	3.32E-01	0.26	3.52E-01	0.30	8.56E-01	-0.05	2.10E-01	0.38
895.2902@0.41701728	895.2902	0.42	POS	hilic				4.63E-01	4.46E-02	0.65	3.57E-01	0.25	1.49E-01	0.36	9.02E-01	0.04
895.44@0.4971011	895.4400	0.50	POS	hilic				8.37E-01	8.26E-01	-0.07	6.89E-01	0.10	6.12E-01	-0.14	3.46E-01	0.26
895.4648@12.501381	895.4648	12.50	NEG	RP				1.17E-01	4.38E-02	-0.44	4.73E-01	0.19	1.71E-01	0.37	2.02E-01	0.31
895.5537@11.986244	895.5537	11.99	NEG	RP				2.24E-04	7.93E-01	0.07	1.04E-03	-0.95	8.03E-04	-0.98	4.13E-01	0.21
895.5544@0.7033931	895.5544	0.70	NEG	hilic				3.22E-01	2.01E-01	-0.39	5.80E-01	0.17	9.07E-02	0.45	9.06E-01	0.03
895.5544@12.126937	895.5544	12.13	NEG	RP				1.73E-01	4.81E-01	0.16	1.78E-01	0.40	1.41E-02	0.71	6.46E-01	-0.14
895.5545@0.7036789	895.5545	0.70	NEG	hilic				5.23E-01	1.12E-01	-0.48	9.80E-01	0.01	5.01E-01	0.19	7.21E-01	0.09
895.6231@10.71982	895.6231	10.72	POS	RP				3.57E-01	1.88E-01	0.37	9.88E-01	0.00	2.05E-01	0.37	2.68E-01	-0.28
895.6393@0.5900432	895.6393	0.59	POS	hilic				1.34E-01	6.66E-03	-0.86	2.08E-01	-0.32	5.61E-02	-0.63	6.26E-01	-0.15
895.7601@0.40688556	895.7601	0.41	POS	hilic				6.31E-01	1.02E-01	-0.52	3.68E-01	-0.27	7.14E-02	-0.47	2.44E-01	-0.31
895.7889@4.4781976	895.7889	4.48	POS	hilic				7.50E-01	2.59E-01	0.31	6.82E-01	0.11	4.42E-01	0.26	5.91E-01	-0.17
895.8477@4.565609	895.8477	4.57	NEG	hilic				7.94E-01	1.92E-01	0.39	3.59E-01	0.28	1.61E-01	0.40	4.87E-01	0.21
896.6389@10.568497	896.6389	10.57	NEG	RP				5.58E-02	1.53E-02	-0.71	3.52E-01	0.27	3.07E-01	0.28	7.18E-01	-0.11
896.7768@11.035209	896.7768	11.04	NEG	RP				1.43E-01	2.09E-02	-0.54	6.38E-01	0.14	7.28E-01	0.09	6.36E-02	-0.45
897.4917@10.312353	897.4917	10.31	NEG	RP				3.75E-01	1.79E-01	0.33	5.26E-01	-0.14	1.50E-01	-0.27	8.56E-01	-0.04
897.5671@0.7014224	897.5671	0.70	NEG	hilic				4.72E-01	3.27E-01	0.29	1.62E-01	-0.38	8.31E-01	0.07	5.26E-01	0.17
897.5677@0.70775706	897.5677	0.71	NEG	hilic				4.94E-01	4.59E-01	0.23	2.13E-01	-0.35	9.60E-01	-0.02	2.06E-01	-0.35
897.5696@12.421856	897.5696	12.42	NEG	RP				4.17E-01	8.95E-02	0.59	6.15E-01	-0.14	6.55E-01	0.13	9.06E-01	-0.04
897.5697@12.422898	897.5697	12.42	NEG	RP				2.67E-01	3.48E-02	0.70	6.83E-01	-0.11	6.27E-01	0.15	9.79E-01	-0.01
897.6214@10.720101	897.6214	10.72	POS	RP				3.49E-02	1.01E-02	0.74	2.02E-01	0.41	2.38E-03	0.91	9.51E-01	0.02
897.6427@10.567543	897.6427	10.57	NEG	RP				6.58E-02	1.97E-02	-0.68	4.06E-01	0.25	2.37E-01	0.31	8.13E-01	-0.07
897.6599@0.58978456	897.6599	0.59	POS	hilic				2.10E-01	1.14E-02	-0.78	2.65E-01	-0.29	6.72E-02	-0.61	6.28E-01	-0.16
897.7784@0.40671828	897.7784	0.41	POS	hilic				7.46E-01	1.28E-01	-0.49	5.51E-01	-0.19	2.33E-01	-0.29	2.97E-01	-0.27
898.1727@9.605222	898.1727	9.61	NEG	RP				4.39E-01	4.70E-01	-0.24	5.69E-01	0.17	1.34E-01	0.42	4.25E-01	-0.21
898.1733@9.410531	898.1733	9.41	NEG	RP				3.10E-01	9.88E-01	0.00	6.04E-02	0.52	4.63E-01	0.21	6.60E-02	0.63
898.1734@9.409122	898.1734	9.41	NEG	RP				2.15E-01	8.33E-02	-0.55	3.44E-01	0.26	7.51E-01	0.09	3.50E-01	0.30
898.1735@0.3964443	898.1735	0.40	NEG	hilic				8.31E-01	8.09E-01	0.06	2.57E-01	-0.34	9.12E-01	0.03	7.56E-01	-0.10
898.1747@9.158441	898.1747	9.16	NEG	RP				7.88E-01	8.00E-01	0.08	4.17E-01	-0.26	9.37E-01	-0.02	2.65E-01	-0.33
898.6281@1.0260413	898.6281	1.03	POS	hilic				6.89E-01	1.59E-01	0.44	4.00E-01	0.22	9.22E-01	0.02	3.05E-01	0.26
899.4975@11.9796505	899.4975	11.98	NEG	RP				7.63E-01	4.05E-01	0.25	5.56E-01	0.16	6.74E-02	0.47	5.23E-01	0.19
899.5685@11.773515	899.5685	11.77	NEG	RP				4.81E-01	2.63E-02	-0.60	8.08E-01	-0.06	8.60E-01	-0.05	8.44E-01	-0.06
899.57@0.62127507	899.5700	0.62	NEG	hilic				3.11E-01	9.88E-02	-0.39	5.41E-01	0.16	1.60E-01	-0.32	2.48E-01	-0.28
899.574@12.414777	899.5740	12.41	NEG	RP				4.40E-02	3.20E-03	0.88	9.37E-01	0.02	3.45E-01	0.21	3.43E-01	0.22
899.585@0.7084231	899.5850	0.71	NEG	hilic				9.91E-01	5.35E-01	-0.18	8.93E-01	-0.04	9.20E-01	-0.03	9.25E-01	-0.03
899.5856@12.914355	899.5856	12.91	NEG	RP				5.92E-02	2.31E-02	0.70	2.24E-01	-0.33	4.43E-01	-0.22	5.33E-01	0.20
899.676@0.589655	899.6760	0.59	POS	hilic				1.25E-01	7.44E-03	-0.85	2.33E-01	-0.29	6.69E-02	-0.60	7.50E-01	-0.10
900.4866@11.659196	900.4866	11.66	NEG	RP				2.99E-01	2.76E-01	-0.25	2.31E-01	0.28	1.39E-01	0.43	7.86E-01	0.06
900.572@11.769442	900.5720	11.77	NEG	RP				9.71E-01	7.43E-01	0.07	5.86E-01	0.14	6.38E-01	0.12	8.60E-01	-0.04
900.6871@10.783187	900.6871	10.78	NEG	RP				6.97E-03	4.30E-02	-0.58	5.65E-02	0.57	4.47E-02	0.54	6.28E-01	-0.14
901.5847@11.949261	901.5847	11.95	NEG	RP				7.79E-01	6.90E-01	0.13	9.67E-01	-0.01	1.92E-01	-0.35	8.66E-01	-0.05
901.6904@0.5900091	901.6904	0.59	POS	hilic				3.04E-01	3.19E-02	-0.67	4.75E-01	-0.18	8.87E-02	-0.58	6.44E-01	-0.14
901.8082@10.814485	901.8082	10.81	POS	hilic				5.42E-01	7.99E-01	0.08	1.94E-01	0.42	4.31E-01	-0.24	6.02E-01	0.13
902.1155@9.404231	902.1155	9.40	NEG	RP				9.81E-01	8.19E-01	-0.08	4.89E-01	-0.20	5.74E-01	-0.15	6.08E-01	-0.15
902.1159@9.401804	902.1159	9.40	NEG	RP				9.85E-01	6.54E-01	-0.15	7.82E-01	-0.08	7.76E-01	-0.08	8.72E-01	0.05

Supplementary table 2: Metabolomics analysis results

Compound	Mass	Retention Time	Ionization mode	column	Compound_ID	MSMS_spectra	ID_level	ANOVA P-FDR	Anorexia vs control P	Anorexia vs control Estimate	overweight vs control P	overweight vs control Estimate	obese vs control P	obese vs control Estimate	athletes vs control P	athletes vs control Estimate
902.1172@9.159644	902.1172	9.16	NEG	RP				6.65E-01	4.18E-01	-0.23	4.54E-01	-0.22	3.95E-01	-0.25	4.95E-02	-0.56
902.1193@9.606348	902.1193	9.61	NEG	RP				5.53E-01	5.87E-02	-0.54	6.95E-01	-0.12	9.77E-01	-0.01	1.56E-01	-0.38
902.2109@0.40574747	902.2109	0.41	NEG	hilic				1.79E-01	4.52E-03	0.80	1.83E-01	0.38	9.66E-01	0.01	4.37E-01	0.18
902.488@2.6558974	902.4880	2.66	POS	hilic				6.76E-01	2.66E-01	0.32	9.30E-01	0.02	7.03E-01	-0.09	2.43E-01	0.30
902.5161@10.509057	902.5161	10.51	POS	RP				2.19E-01	2.70E-01	-0.24	9.86E-01	0.00	5.21E-01	0.17	1.65E-02	-0.45
902.6935@0.5898918	902.6935	0.59	POS	hilic				7.61E-01	5.68E-01	0.13	5.43E-01	-0.13	5.20E-01	-0.15	5.06E-01	0.18
903.6902@10.782832	903.6902	10.78	NEG	RP				6.34E-03	3.26E-02	-0.62	8.52E-02	0.51	3.49E-02	0.57	5.81E-01	-0.16
904.5161@10.51013	904.5161	10.51	POS	RP				2.10E-02	4.75E-03	-0.82	5.64E-01	-0.16	1.49E-01	0.39	2.53E-01	-0.36
905.4875@12.079099	905.4875	12.08	POS	RP				3.33E-01	3.80E-03	-0.75	4.40E-01	-0.23	6.18E-01	-0.16	2.02E-01	-0.32
905.5651@10.720386	905.5651	10.72	POS	RP				7.97E-01	6.38E-01	0.15	2.95E-01	0.32	1.07E-01	0.43	6.47E-01	0.15
905.579@0.6229252	905.5790	0.62	NEG	hilic				3.69E-01	1.77E-02	-0.55	9.89E-01	0.00	9.29E-01	-0.02	6.84E-01	0.11
905.6056@11.262432	905.6056	11.26	NEG	RP				2.27E-01	7.89E-02	-0.35	2.25E-01	0.31	7.88E-01	0.07	3.91E-01	-0.20
905.8191@4.3803067	905.8191	4.38	POS	hilic				2.72E-01	3.09E-02	0.57	2.40E-01	0.29	6.76E-01	-0.12	5.43E-01	-0.21
906.5255@2.5534382	906.5255	2.55	POS	hilic				8.70E-01	4.13E-01	-0.28	8.33E-01	0.06	4.68E-01	-0.21	5.08E-01	-0.20
906.5678@10.719534	906.5678	10.72	POS	RP				3.58E-01	4.48E-01	-0.20	8.26E-01	0.06	1.78E-01	0.38	2.68E-01	-0.26
906.6881@10.780742	906.6881	10.78	NEG	RP				1.27E-01	6.30E-02	-0.43	3.72E-01	0.25	1.79E-01	0.36	3.88E-01	-0.22
906.7331@11.035545	906.7331	11.04	NEG	RP				2.50E-01	3.23E-01	-0.28	9.46E-01	0.02	3.31E-02	0.53	4.05E-01	-0.23
907.1779@0.39616656	907.1779	0.40	NEG	hilic				6.42E-01	7.55E-01	-0.10	1.01E-01	-0.46	8.07E-01	0.06	4.84E-01	-0.22
907.229@9.144134	907.2290	9.14	POS	RP				8.49E-01	2.46E-01	0.37	3.44E-01	0.30	5.63E-01	0.18	3.38E-01	0.31
907.5909@12.708711	907.5909	12.71	NEG	RP				6.69E-01	2.01E-01	-0.34	7.33E-01	-0.10	2.36E-01	-0.30	6.21E-01	0.13
907.5981@12.381098	907.5981	12.38	NEG	RP				8.61E-01	4.51E-01	-0.18	7.36E-01	-0.09	2.28E-01	-0.34	5.50E-01	-0.15
907.6209@11.520243	907.6209	11.52	NEG	RP				2.61E-01	1.15E-02	-0.66	7.22E-01	0.09	7.16E-01	-0.10	6.13E-01	-0.15
908.2061@0.39645314	908.2061	0.40	NEG	hilic				7.62E-01	2.47E-01	-0.27	5.22E-01	0.18	6.45E-01	-0.12	8.89E-01	-0.04
908.5387@2.5531723	908.5387	2.55	POS	hilic				7.46E-01	2.80E-01	-0.35	6.84E-01	0.12	8.15E-01	0.07	5.50E-01	-0.19
908.5922@12.709082	908.5922	12.71	NEG	RP				5.36E-01	3.23E-02	-0.52	8.59E-01	-0.05	2.99E-01	-0.30	4.49E-01	-0.19
909.5152@11.876755	909.5152	11.88	NEG	RP				5.47E-01	6.75E-02	0.48	2.61E-01	0.25	7.65E-01	0.07	6.34E-01	0.10
909.5438@2.5528998	909.5438	2.55	POS	hilic				3.02E-01	4.16E-02	-0.62	8.98E-01	-0.04	7.16E-01	0.09	2.36E-01	-0.35
909.7369@11.035831	909.7369	11.04	NEG	RP				2.77E-01	2.56E-01	-0.32	7.26E-01	-0.11	1.05E-01	0.41	1.78E-01	-0.37
910.1947@0.3967844	910.1947	0.40	NEG	hilic				8.68E-01	4.88E-01	0.18	4.34E-01	0.25	8.54E-01	0.06	2.37E-01	0.36
910.5211@10.724694	910.5211	10.72	POS	RP				1.38E-01	3.23E-01	-0.26	8.32E-01	0.06	7.51E-01	0.10	5.88E-03	-0.74
910.5227@10.563403	910.5227	10.56	POS	RP				1.32E-01	8.00E-01	-0.07	4.24E-02	0.64	1.29E-02	0.69	4.50E-01	0.25
911.1873@9.46339	911.1873	9.46	NEG	RP				9.23E-01	8.43E-01	-0.05	8.44E-01	0.06	3.38E-01	0.28	9.74E-01	0.01
911.1885@9.626825	911.1885	9.63	NEG	RP				5.70E-01	1.80E-02	0.61	3.25E-01	0.29	1.96E-01	0.41	1.52E-01	0.42
911.1942@0.39649048	911.1942	0.40	NEG	hilic				7.14E-01	2.09E-01	0.37	5.94E-01	0.16	4.99E-01	-0.18	8.19E-01	0.07
911.5029@11.863942	911.5029	11.86	NEG	RP				2.97E-01	3.39E-01	0.27	7.09E-01	-0.11	2.57E-02	0.63	8.17E-01	0.07
911.5235@12.125769	911.5235	12.13	NEG	RP				1.27E-01	3.24E-01	-0.24	1.56E-02	-0.51	8.12E-01	-0.07	2.03E-01	0.33
911.5707@0.7232571	911.5707	0.72	POS	hilic				8.93E-01	4.61E-01	-0.14	5.51E-01	0.14	7.11E-01	0.09	8.32E-01	0.04
912.7325@11.0369625	912.7325	11.04	NEG	RP				4.71E-01	9.04E-01	0.03	6.56E-01	0.15	2.94E-02	0.61	9.99E-01	0.00
913.5109@11.983408	913.5109	11.98	NEG	RP				5.80E-03	7.01E-01	0.11	3.31E-03	-0.83	1.07E-01	-0.47	1.89E-01	0.37
913.5138@12.124816	913.5138	12.12	NEG	RP				5.12E-01	9.05E-01	0.03	8.29E-02	-0.43	6.54E-01	0.14	6.89E-01	0.10
913.5367@12.406232	913.5367	12.41	NEG	RP				8.54E-01	2.03E-01	0.38	5.03E-01	0.16	6.75E-01	0.11	5.09E-01	0.19
914.1262@9.413219	914.1262	9.41	NEG	RP				2.23E-01	2.51E-01	-0.34	7.42E-02	-0.49	2.29E-01	-0.38	8.28E-03	-0.84
914.131@9.601051	914.1310	9.60	NEG	RP				8.23E-01	4.10E-01	-0.24	9.02E-01	0.04	5.41E-01	0.17	4.87E-01	-0.21
914.1334@9.40894	914.1334	9.41	NEG	RP				4.54E-01	8.06E-02	-0.55	5.63E-02	-0.49	1.27E-01	-0.46	7.04E-02	-0.59
914.1383@9.158754	914.1383	9.16	NEG	RP				5.76E-01	3.68E-01	-0.30	6.91E-02	-0.51	3.72E-01	-0.27	7.71E-02	-0.54
914.1973@0.3995699	914.1973	0.40	NEG	hilic				2.32E-01	4.38E-02	0.51	4.92E-01	-0.19	3.57E-01	-0.22	3.70E-01	0.28
914.4745@10.567242	914.4745	10.57	POS	RP				1.08E-02	1.44E-01	-0.42	2.06E-02	0.70	1.64E-02	0.65	5.00E-01	0.21
914.5152@11.984347	914.5152	11.98	NEG	RP				2.84E-01	8.56E-01	-0.05	5.32E-02	-0.44	1.02E-02	-0.56	3.80E-01	-0.23
914.5165@12.124352	914.5165	12.12	NEG	RP				7.49E-01	5.07E-01	0.21	4.63E-01	0.20	1.60E-01	0.43	8.86E-01	-0.04
914.5911@0.4113077	914.5911	0.41	NEG	hilic				8.48E-01	3.75E-01	-0.23	4.05E-01	-0.19	8.47E-01	0.05	8.92E-01	-0.03
914.6321@10.028957	914.6321	10.03	POS	RP				1.99E-02	4.91E-02	-0.59	3.02E-01	0.30	8.02E-01	0.07	6.02E-03	-0.72
915.529@12.405095	915.5290	12.41	NEG	RP				1.18E-02	5.59E-03	0.85	2.74E-01	-0.28	6.00E-01	0.13	3.54E-01	-0.28
915.6327@12.381694	915.6327	12.38	NEG	RP				9.30E-01	4.79E-01	-0.19	7.67E-01	-0.08	4.06E-01	-0.26	9.85E-01	-0.01
916.1315@9.41147	916.1315	9.41	NEG	RP				4.67E-01	3.00E-01	-0.31	6.11E-02	-0.55	2.00E-01	-0.37	5.78E-02	-0.61
916.1315@9.601248	916.1315	9.60	NEG	RP				4.10E-01	4.45E-02	-0.51	6.37E-01	0.16	5.03E-01	0.17	4.53E-01	-0.20
916.1318@9.345657	916.1318	9.35	NEG	RP				2.01E-01	1.84E-01	-0.40	2.56E-02	-0.64	1.15E-01	-0.42	1.31E-02	-0.82
916.1328@9.158419	916.1328	9.16	NEG	RP				6.89E-01	3.46E-01	-0.31	1.73E-01	-0.39	4.44E-01	-0.22	9.45E-02	-0.52
916.203@0.40062618	916.2030	0.40	NEG	hilic				8.23E-01	7.47E-01	0.09	4.07E-01	0.26	7.74E-01	-0.07	2.98E-01	0.30
916.5311@12.407917	916.5311	12.41	NEG	RP				2.27E-01	3.35E-01	0.33	1.23E-01	-0.41	9.34E-01	-0.02	1.86E-01	-0.37
916.6051@0.41153497	916.6051	0.41	NEG	hilic				8.69E-01	4.78E-01	-0.19	5.11E-01	-0.15	6.99E-01	0.10	9.65E-01	0.01
916.6058@10.925402	916.6058	10.93	NEG	RP				8.22E-01	2.71E-01	-0.32	2.04E-01	-0.34	6.84E-01	-0.11	4.18E-01	-0.24
917.1347@9.408147	917.1347	9.41	NEG	RP				8.71E-01	5.26E-01	-0.19	5.88E-01	-0.17	8.43E-01	0.06	2.33E-01	-0.30
917.1348@9.602787	917.1348	9.60	NEG	RP				6.00E-01	1.31E-01	-0.39	8.34E-02	-0.50	8.86E-01	-0.04	5.91E-01	-0.17
917.1355@9.157751	917.1355	9.16	NEG	RP				8.18E-01	5.33E-01	-0.21	3.42E-01	-0.28	5.48E-01	-0.16	1.60E-01	-0.43
917.5457@12.917249	917.5457	12.92	NEG	RP				3.44E-01	4.13E-02	0.59	7.86E-01	-0.08	9.90E-01	0.00	2.19E-01	0.40
917.61@0.41117644	917.6100	0.41	NEG	hilic				8.00E-01	2.59E-01	-0.28	2.54E-01	-0.25	8.47E-01	-0.05	6.91E-01	-0.10
917.6307@12.381776	917.6307	12.38	NEG	RP				9.39E-01	4.13E-01	-0.24	9.34E-01	-0.02	5.62E-01	-0.18	4.75E-01	-0.23
917.7463@0.40514997	917.7463	0.41	POS	hilic				6.36E-01	1.08E-01	-0.45	9.89E-01	0.00	3.33E-01	-0.23	6.78E-01	-0.10
918.5477@12.916609	918.5477	12.92	NEG	RP				5.47E-01	6.35E-01	-0.15	8.68E-02	-0.44	8.75E-01	0.04	7.23E-01	0.11
918.6342@12.381506	918.6342	12.38	NEG	RP				8.60E-01	5.56E-01	-0.17	7.47E-01	0.08	4.62E-01	-0.22	3.95E-01	-0.26
919.4622@12.384886	919.4622	12.38	NEG	RP				6.51E-01	7.66E-02	-0.49	8.42E-01	-0.05	7.43E-01	-0.09	9.77E-01	-0.01
919.4728@11.770976	919.4728	11.77	NEG	RP				1.67E-01	7.30E-02	0.41	8.84E-01	0.03	4.49E-01	0.15	2.28E-02	0.62
919.5504@12.276588	919.5504	12.28	NEG	RP				2.05E-03	2.55E-01	0.36	1.37E-04	0.97	3.82E-03	0.79	4.02E-01	-0.23

Supplementary table 2: Metabolomics analysis results

Compound	Mass	Retention Time	Ionization mode	column	Compound_ID	MSMS_spectra	ID_level	ANOVA P-FDR	Anorexia vs control P	Anorexia vs control Estimate	overweight vs control P	overweight vs control Estimate	obese vs control P	obese vs control Estimate	athletes vs control P	athletes vs control Estimate
919.5537@11.910319	919.5537	11.91	NEG	RP				2.44E-01	4.68E-02	-0.65	2.62E-01	-0.33	7.31E-01	0.10	7.40E-01	0.10
919.5542@12.062256	919.5542	12.06	NEG	RP				3.16E-02	1.45E-03	-1.08	1.38E-01	-0.41	1.84E-01	-0.39	3.05E-03	-0.77
919.5547@0.63547736	919.5547	0.64	NEG	hilic				8.47E-01	4.51E-01	-0.24	5.24E-01	0.19	8.67E-01	-0.04	7.94E-01	0.07
919.5549@0.66476345	919.5549	0.66	NEG	hilic				7.73E-01	4.12E-01	-0.26	8.76E-01	0.04	3.03E-01	-0.29	6.94E-01	0.11
919.5928@10.516024	919.5928	10.52	NEG	RP				5.86E-02	8.40E-02	-0.32	4.84E-01	0.17	8.66E-02	0.45	1.64E-01	-0.27
919.6612@10.777635	919.6612	10.78	POS	RP				6.67E-02	4.51E-01	-0.21	5.24E-02	0.56	1.58E-02	0.64	8.82E-01	0.04
919.7626@0.4057013	919.7626	0.41	POS	hilic				5.35E-01	3.53E-02	-0.58	6.91E-01	-0.12	3.05E-01	-0.25	4.47E-01	-0.19
919.8124@4.6150227	919.8124	4.62	NEG	hilic				9.36E-01	5.11E-01	0.18	4.33E-01	0.22	8.37E-01	0.05	4.33E-01	0.25
920.2117@0.3966139	920.2117	0.40	NEG	hilic				4.55E-01	1.14E-01	0.42	4.40E-02	0.61	2.13E-01	0.33	1.06E-01	0.54
920.473@11.880652	920.4730	11.88	POS	RP				1.92E-01	2.16E-01	0.34	6.56E-01	-0.10	3.11E-02	0.59	9.33E-01	-0.02
920.5473@3.0478816	920.5473	3.05	POS	hilic				8.09E-01	3.66E-01	-0.23	5.95E-01	0.14	6.04E-01	-0.13	6.82E-01	0.13
920.6405@10.557426	920.6405	10.56	NEG	RP				2.68E-02	8.60E-03	-0.49	1.12E-01	0.40	3.34E-01	0.24	4.13E-01	-0.19
921.0026@10.067156	921.0026	10.07	POS	RP				1.57E-01	7.78E-02	-0.52	2.63E-01	0.34	4.20E-01	0.21	3.39E-01	-0.29
921.4719@11.771707	921.4719	11.77	NEG	RP				1.04E-01	6.94E-01	-0.08	1.30E-01	-0.31	7.44E-01	-0.07	8.06E-02	0.47
921.5695@12.276799	921.5695	12.28	NEG	RP				5.12E-02	1.72E-01	0.36	4.59E-03	0.79	2.53E-02	0.63	9.98E-01	0.00
921.644@10.709574	921.6440	10.71	NEG	RP				2.77E-01	3.89E-01	-0.29	9.23E-01	-0.03	5.31E-02	0.51	3.77E-01	-0.23
921.6597@10.777726	921.6597	10.78	POS	RP				3.68E-02	3.33E-01	-0.30	3.27E-02	0.62	1.53E-02	0.64	9.25E-01	0.03
922.2286@0.39654946	922.2286	0.40	NEG	hilic				6.96E-01	2.92E-01	0.30	3.02E-01	0.32	3.63E-01	0.23	9.39E-02	0.54
922.2334@9.437846	922.2334	9.44	NEG	RP				4.42E-01	3.27E-01	-0.30	8.74E-01	0.04	8.38E-02	0.47	9.21E-01	-0.03
922.4973@12.009227	922.4973	12.01	POS	RP				4.43E-01	9.05E-01	-0.03	5.78E-02	-0.43	9.00E-01	-0.03	5.35E-01	0.17
922.5724@12.274813	922.5724	12.27	NEG	RP				2.32E-01	5.62E-01	0.16	8.67E-01	0.04	3.66E-02	0.64	4.42E-01	-0.20
922.6603@10.776765	922.6603	10.78	POS	RP				4.25E-02	4.21E-01	-0.23	2.32E-02	0.63	3.81E-02	0.58	7.24E-01	-0.10
922.7705@11.040226	922.7705	11.04	NEG	RP				5.04E-01	9.01E-02	0.56	7.09E-01	0.09	7.88E-01	0.06	7.69E-01	0.07
923.2315@0.3975602	923.2315	0.40	NEG	hilic				5.01E-01	6.54E-01	0.14	1.19E-01	0.46	9.93E-01	0.00	1.34E-01	0.50
923.4417@0.4983783	923.4417	0.50	POS	hilic				8.73E-01	9.55E-01	0.02	7.79E-01	0.07	5.58E-01	-0.18	4.27E-01	0.23
923.4915@12.071473	923.4915	12.07	NEG	RP				2.13E-01	5.44E-03	0.80	5.83E-01	0.15	1.47E-01	0.41	2.83E-01	0.25
923.5825@12.562371	923.5825	12.56	NEG	RP				1.73E-01	7.10E-02	0.56	2.45E-01	-0.31	9.08E-01	-0.03	4.02E-01	-0.24
923.5852@12.800651	923.5852	12.80	NEG	RP				4.33E-05	2.83E-02	-0.63	6.14E-03	0.77	5.19E-04	0.90	6.51E-01	-0.11
923.5867@0.6984998	923.5867	0.70	NEG	hilic				1.71E-01	1.99E-01	-0.24	1.73E-01	0.33	1.05E-01	0.44	9.21E-01	-0.02
924.509@12.489577	924.5090	12.49	POS	RP				4.23E-01	2.43E-01	-0.34	1.11E-02	-0.59	2.64E-01	-0.30	5.96E-01	-0.15
924.5209@10.723832	924.5209	10.72	NEG	RP				7.22E-02	6.70E-01	0.11	5.63E-01	0.13	5.53E-03	0.76	9.31E-01	-0.02
924.6527@0.8438491	924.6527	0.84	NEG	hilic				1.96E-01	8.43E-01	0.05	8.17E-02	0.40	4.95E-02	0.51	5.26E-01	-0.14
925.4515@0.50431854	925.4515	0.50	POS	hilic				8.02E-01	9.28E-01	-0.03	2.64E-01	0.32	7.69E-01	-0.08	5.19E-01	0.19
925.5876@0.7611333	925.5876	0.76	POS	hilic				4.74E-01	1.85E-01	-0.24	4.55E-02	-0.33	4.72E-01	-0.15	1.71E-01	-0.25
925.599@13.223497	925.5990	13.22	NEG	RP				1.63E-02	9.38E-01	-0.02	4.67E-01	0.21	2.40E-04	0.93	3.87E-01	-0.24
925.657@10.567827	925.6570	10.57	NEG	RP				1.61E-01	2.35E-02	-0.54	3.74E-01	0.25	3.70E-01	0.24	7.61E-01	-0.08
925.7099@11.029665	925.7099	11.03	POS	RP				7.23E-01	9.37E-02	0.45	4.25E-01	0.25	8.26E-01	0.07	9.01E-01	-0.04
925.7315@10.71499	925.7315	10.71	NEG	RP				5.16E-02	8.89E-01	0.03	4.95E-02	-0.58	2.82E-01	0.28	4.73E-02	-0.59
926.1792@9.151415	926.1792	9.15	NEG	RP				5.20E-01	1.65E-01	0.41	3.63E-01	-0.24	5.73E-01	0.16	4.61E-01	0.22
926.1922@0.3963723	926.1922	0.40	NEG	hilic				5.57E-01	3.90E-01	0.22	4.59E-02	0.58	4.69E-01	0.18	4.26E-01	0.23
926.5249@12.989493	926.5249	12.99	POS	RP				5.74E-01	5.56E-01	0.14	8.35E-01	0.05	9.05E-02	0.53	5.62E-01	0.14
926.6591@10.568025	926.6591	10.57	NEG	RP				1.44E-01	3.15E-02	-0.42	2.83E-01	0.27	3.97E-01	0.21	3.88E-01	-0.21
926.9601@0.5572904	926.9601	0.56	POS	hilic				6.94E-01	5.63E-01	0.15	1.26E-01	0.37	5.89E-01	0.14	1.62E-01	0.40
927.4647@0.5341764	927.4647	0.53	POS	hilic				7.64E-01	1.15E-01	-0.35	7.15E-01	-0.09	1.69E-01	-0.31	3.97E-01	-0.22
927.674@10.706731	927.6740	10.71	NEG	RP				4.82E-01	7.96E-01	-0.07	2.53E-01	-0.28	7.42E-01	0.08	7.05E-02	-0.46
927.708@11.030365	927.7080	11.03	POS	RP				5.08E-01	3.90E-02	0.63	5.92E-01	0.17	1.52E-01	0.45	4.67E-01	0.23
927.7091@10.783289	927.7091	10.78	NEG	RP				5.81E-01	7.76E-01	-0.07	9.95E-01	0.00	2.96E-01	0.28	2.73E-01	-0.26
928.6001@3.230037	928.6001	3.23	NEG	hilic				8.88E-01	4.62E-01	0.25	4.94E-01	0.18	1.74E-01	0.33	3.00E-01	0.30
929.2325@9.4223175	929.2325	9.42	POS	RP				6.33E-01	6.56E-01	-0.14	1.42E-01	0.42	6.47E-01	0.15	8.49E-01	-0.05
929.513@0.7388846	929.5130	0.74	NEG	hilic				3.87E-01	4.84E-01	-0.22	2.84E-02	-0.58	8.91E-01	0.04	9.67E-01	0.01
929.6819@10.71765	929.6819	10.72	NEG	RP				2.51E-01	7.90E-02	0.53	6.14E-01	-0.11	5.09E-01	0.14	5.12E-01	-0.15
929.8418@4.566488:1	929.8418	4.57	NEG	hilic				4.80E-01	6.90E-02	0.61	7.20E-02	0.54	2.87E-01	0.28	1.88E-01	0.36
929.8418@4.566488:2	929.8418	4.57	NEG	hilic				3.63E-01	4.73E-02	0.66	4.54E-02	0.60	3.16E-01	0.28	1.10E-01	0.43
930.1139@9.162462	930.1139	9.16	NEG	RP				9.14E-01	2.76E-01	-0.33	5.31E-01	-0.19	3.27E-01	-0.27	5.91E-01	-0.18
930.1823@0.3963125	930.1823	0.40	NEG	hilic				6.96E-01	1.67E-01	0.32	4.33E-01	0.17	2.83E-01	0.24	1.20E-01	0.42
930.2342@0.40377048	930.2342	0.40	NEG	hilic				5.74E-01	4.13E-01	0.22	9.16E-01	-0.03	1.04E-01	0.46	8.27E-01	0.05
930.4798@6.1704235	930.4798	6.17	POS	RP				8.04E-01	8.03E-01	0.07	4.29E-01	0.20	1.09E-01	0.41	8.62E-01	0.06
930.4814@0.83025855	930.4814	0.83	POS	hilic				9.27E-01	3.91E-01	-0.27	7.41E-01	-0.08	8.04E-01	0.06	9.58E-01	0.02
930.5206@2.5538795	930.5206	2.55	POS	hilic				8.56E-01	4.68E-01	-0.24	5.42E-01	0.17	9.03E-01	-0.04	6.94E-01	-0.11
930.5692@1.9788499	930.5692	1.98	POS	hilic				8.96E-05	1.39E-02	0.71	1.77E-02	-0.50	3.21E-02	-0.48	1.25E-01	0.37
931.5833@12.581987	931.5833	12.58	NEG	RP				2.92E-02	8.87E-04	-1.01	6.94E-01	-0.10	7.60E-02	-0.56	3.28E-01	-0.27
931.6115@10.922716	931.6115	10.92	POS	RP				8.77E-01	6.68E-01	-0.11	4.87E-01	-0.17	4.05E-01	-0.23	1.97E-01	-0.30
931.7037@10.783348	931.7037	10.78	NEG	RP				1.05E-02	7.55E-02	-0.49	4.22E-02	0.59	2.79E-02	0.57	7.28E-01	-0.10
933.5235@12.266746	933.5235	12.27	NEG	RP				5.68E-01	4.38E-01	0.23	2.72E-01	-0.28	9.96E-01	0.00	3.68E-01	0.27
933.631@10.921532	933.6310	10.92	POS	RP				7.92E-01	2.17E-01	-0.41	2.81E-01	-0.31	8.65E-01	-0.05	7.24E-01	-0.11
933.6333@0.40191987	933.6333	0.40	POS	hilic				5.02E-01	7.47E-01	-0.09	4.78E-02	-0.60	5.03E-01	-0.19	5.64E-01	-0.17
933.7583@11.03554	933.7583	11.04	NEG	RP				4.54E-01	4.61E-01	-0.23	8.80E-01	-0.04	1.93E-01	0.37	2.15E-01	-0.33
934.2015@0.40339315	934.2015	0.40	NEG	hilic				3.68E-01	2.93E-02	0.54	4.37E-02	0.61	1.34E-01	0.39	8.13E-02	0.62
934.5494@10.561501	934.5494	10.56	NEG	RP				6.22E-01	8.45E-01	-0.04	1.16E-01	0.38	3.84E-01	0.20	3.30E-01	0.26
934.6365@10.922146	934.6365	10.92	POS	RP				6.41E-01	1.38E-01	-0.54	4.22E-01	-0.22	9.74E-01	-0.01	7.59E-01	-0.09
935.5202@0.94633716	935.5202	0.95	NEG	hilic				4.57E-01	3.27E-01	0.29	1.58E-01	-0.36	4.07E-01	-0.23	9.30E-01	0.02
935.5213@12.0601635	935.5213	12.06	NEG	RP				5.61E-02	2.55E-03	-0.90	6.09E-02	-0.55	2.15E-01	-0.35	7.25E-03	-0.86
935.5241@0.81096834	935.5241	0.81	POS	hilic				2.42E-01	9.54E-01	-0.01	1.21E-01	0.42	7.33E-01	-0.08	1.19E-01	-0.33

Supplementary table 2: Metabolomics analysis results

								ANOVA	Anorexia vs control		overweight vs control		obese vs control		athletes vs control	
Compound	Mass	Retention Time	Ionization mode	column	Compound_ID	MSMS_spectra	ID_level	P-FDR	P	Estimate	P	Estimate	P	Estimate	P	Estimate
935.5377@12.682966	935.5377	12.68	NEG	RP				2.61E-01	8.01E-03	0.74	1.28E-01	0.36	8.86E-01	0.04	3.47E-01	0.30
935.6241@10.709103	935.6241	10.71	NEG	RP				2.55E-01	1.19E-01	0.43	2.21E-01	0.31	1.52E-02	0.76	3.22E-01	0.24
935.7564@11.0368805	935.7564	11.04	NEG	RP				3.71E-01	6.52E-01	0.14	1.96E-01	0.37	1.16E-02	0.70	6.64E-01	0.12
936.2092@9.150708	936.2092	9.15	NEG	RP				7.10E-01	9.05E-01	-0.04	9.93E-02	0.45	7.88E-01	0.09	8.72E-01	0.05
936.2105@9.42713	936.2105	9.43	NEG	RP				1.89E-01	6.97E-01	0.11	1.21E-02	-0.67	9.01E-01	-0.03	2.93E-01	-0.32
936.2123@0.40376577	936.2123	0.40	NEG	hilic				5.29E-01	8.12E-02	0.46	1.52E-01	0.42	2.44E-01	0.33	6.65E-02	0.60
936.5407@12.691253	936.5407	12.69	NEG	RP				4.11E-01	2.84E-01	0.32	3.82E-01	-0.18	5.34E-01	0.15	2.47E-01	-0.26
937.1986@3.6071215	937.1986	3.61	NEG	hilic				3.21E-01	8.93E-01	-0.03	1.48E-01	0.34	5.83E-01	0.13	1.25E-01	-0.26
937.2154@0.40287176	937.2154	0.40	NEG	hilic				2.91E-01	1.27E-01	0.40	7.88E-03	0.75	4.55E-01	0.20	1.48E-01	0.47
937.4523@0.47643638	937.4523	0.48	POS	hilic				7.98E-01	6.88E-01	-0.11	4.43E-01	0.21	7.20E-01	0.10	2.48E-01	0.31
937.7159@10.716923	937.7159	10.72	NEG	RP				3.85E-01	5.93E-02	0.60	2.83E-01	0.29	4.62E-01	0.20	3.53E-02	0.58
937.7517@11.036796	937.7517	11.04	NEG	RP				3.37E-01	5.23E-01	0.19	1.62E-01	0.40	9.68E-03	0.71	7.46E-01	0.09
938.5153@12.066585	938.5153	12.07	NEG	RP				2.71E-01	4.38E-02	-0.62	3.23E-01	-0.28	6.02E-01	0.15	8.41E-01	-0.06
938.5868@10.921094	938.5868	10.92	POS	RP				4.92E-01	1.57E-01	-0.47	1.25E-01	-0.48	9.49E-01	0.02	7.42E-01	-0.09
938.5889@0.3959655	938.5889	0.40	POS	hilic				8.78E-01	2.97E-01	-0.23	3.29E-01	-0.21	7.77E-01	-0.06	6.01E-01	-0.12
938.7081@0.8850975	938.7081	0.89	NEG	hilic				9.88E-01	8.09E-01	-0.05	8.00E-01	0.06	8.54E-01	0.05	6.14E-01	0.11
938.7551@11.037075	938.7551	11.04	NEG	RP				2.79E-01	4.71E-01	0.21	1.24E-01	0.43	6.88E-03	0.76	6.79E-01	0.12
939.1979@3.5987146	939.1979	3.60	NEG	hilic				7.91E-01	4.56E-01	-0.17	7.04E-01	0.09	5.42E-01	0.16	4.47E-01	0.18
939.5421@0.71344	939.5421	0.71	NEG	hilic				4.83E-01	1.97E-01	-0.47	2.91E-02	-0.57	2.34E-01	-0.34	6.69E-02	-0.55
939.6338@10.56745	939.6338	10.57	NEG	RP				1.62E-01	2.72E-02	-0.42	2.07E-01	0.31	3.76E-01	0.21	7.27E-01	-0.09
939.7104@10.714268	939.7104	10.71	NEG	RP				5.56E-01	5.91E-02	0.46	6.53E-01	0.11	7.30E-01	-0.08	3.73E-01	0.23
940.1907@0.39714515	940.1907	0.40	NEG	hilic				7.46E-01	1.84E-01	0.36	1.03E-01	0.46	4.16E-01	0.21	6.20E-01	0.17
940.7239@0.88497674	940.7239	0.88	NEG	hilic				2.66E-03	4.30E-02	-0.48	5.34E-03	0.68	8.65E-02	0.48	3.58E-01	-0.23
941.5536@12.794563	941.5536	12.79	NEG	RP				1.25E-01	2.46E-01	-0.35	1.64E-01	0.40	6.61E-02	0.52	6.38E-01	-0.12
941.6913@10.782744	941.6913	10.78	NEG	RP				2.71E-01	1.24E-01	-0.27	2.27E-01	0.30	1.80E-01	0.32	9.03E-01	0.03
942.283@3.1539788	942.2830	3.15	POS	hilic				6.02E-01	2.36E-01	0.40	5.12E-01	-0.16	6.89E-01	0.10	3.60E-01	0.25
942.5855@11.041888	942.5855	11.04	NEG	RP				6.94E-01	2.27E-01	-0.32	9.46E-01	-0.02	3.43E-01	0.26	5.80E-01	-0.17
943.5524@11.829427	943.5524	11.83	NEG	RP				1.80E-02	7.89E-02	-0.60	7.07E-04	-0.81	1.37E-03	-0.93	7.78E-01	-0.07
943.5604@13.242638	943.5604	13.24	NEG	RP				2.82E-02	8.08E-01	-0.07	5.72E-01	0.16	4.58E-04	0.86	3.89E-01	-0.24
944.1614@9.640027	944.1614	9.64	NEG	RP				5.18E-01	6.64E-01	0.13	6.57E-01	-0.15	1.95E-01	0.40	3.96E-01	-0.24
944.4875@12.069217	944.4875	12.07	POS	RP				1.82E-01	2.39E-01	-0.30	1.05E-02	-0.61	1.63E-02	-0.56	6.89E-01	-0.11
944.6004@11.042939	944.6004	11.04	NEG	RP				3.27E-02	3.29E-04	-1.00	1.01E-01	-0.50	3.33E-01	-0.25	4.56E-03	-0.83
944.6814@11.030039	944.6814	11.03	POS	RP				7.05E-01	1.74E-01	0.33	4.65E-01	0.24	9.02E-01	-0.04	5.65E-01	-0.17
945.6043@11.042272	945.6043	11.04	NEG	RP				1.74E-01	2.36E-02	-0.56	9.81E-01	-0.01	2.85E-01	0.24	1.13E-01	-0.49
945.6835@10.78327	945.6835	10.78	NEG	RP				2.28E-02	1.79E-01	-0.38	3.27E-02	0.62	2.15E-02	0.59	9.00E-01	0.04
945.948@10.552735	945.9480	10.55	NEG	hilic				2.12E-01	3.71E-02	0.59	3.75E-01	0.28	2.44E-01	-0.30	3.67E-01	0.26
946.1751@0.39612898	946.1751	0.40	NEG	hilic				9.30E-01	8.46E-01	-0.06	3.85E-01	-0.24	3.93E-01	-0.24	8.51E-01	-0.05
946.4887@12.070533	946.4887	12.07	POS	RP				1.74E-01	5.65E-03	-0.71	3.13E-02	-0.65	1.74E-02	-0.62	8.36E-02	-0.43
947.4842@11.876722	947.4842	11.88	NEG	RP				7.43E-01	5.04E-01	0.20	8.98E-01	0.04	1.14E-01	0.44	9.40E-01	0.02
947.5849@12.693162	947.5849	12.69	NEG	RP				9.22E-02	2.75E-02	-0.75	6.71E-01	-0.12	8.79E-01	0.04	1.61E-02	-0.64
947.7363@11.0352125	947.7363	11.04	NEG	RP				3.61E-01	3.68E-01	0.29	1.64E-01	0.39	1.70E-02	0.74	5.11E-01	0.19
948.1791@9.268136	948.1791	9.27	NEG	RP				9.94E-01	6.74E-01	-0.11	6.30E-01	-0.14	7.74E-01	-0.08	7.51E-01	-0.09
948.1819@9.493684	948.1819	9.49	NEG	RP				8.51E-01	1.23E-01	0.37	9.03E-01	0.04	8.14E-01	-0.06	8.43E-01	0.06
948.1822@9.647872	948.1822	9.65	NEG	RP				7.10E-01	6.43E-01	0.13	3.61E-01	0.28	9.05E-02	0.50	3.10E-01	0.33
948.1848@0.39628434	948.1848	0.40	NEG	hilic				9.89E-01	7.77E-01	0.09	6.47E-01	0.13	5.51E-01	0.16	9.57E-01	0.02
948.525@10.560566	948.5250	10.56	NEG	RP				6.65E-01	7.77E-01	-0.07	3.17E-01	0.26	3.21E-01	0.23	1.88E-01	0.37
948.6168@10.123598	948.6168	10.12	NEG	RP				1.39E-04	2.00E-02	0.71	1.22E-01	-0.33	2.17E-03	-0.86	1.25E-01	0.40
948.6182@10.116609	948.6182	10.12	POS	RP				6.68E-04	2.86E-02	0.64	6.64E-02	-0.43	2.63E-02	-0.70	7.03E-02	0.47
949.1913@0.39636362	949.1913	0.40	NEG	hilic				9.64E-01	5.18E-01	0.19	9.04E-01	0.03	8.58E-01	0.05	7.81E-01	-0.08
949.486@11.88383	949.4860	11.88	NEG	RP				5.68E-01	7.90E-02	0.41	9.58E-01	0.01	6.84E-01	0.12	1.12E-01	0.40
949.499@12.265317	949.4990	12.27	NEG	RP				3.01E-01	1.21E-01	0.46	8.44E-02	0.53	1.97E-01	0.36	1.33E-02	0.76
949.4994@12.266104	949.4994	12.27	NEG	RP				5.96E-01	1.98E-02	0.58	5.08E-01	0.23	5.68E-01	0.14	1.77E-01	0.40
949.5288@10.556688	949.5288	10.56	NEG	RP				8.95E-01	5.81E-01	0.15	6.83E-01	0.11	7.78E-01	-0.08	3.72E-01	0.27
949.7405@11.036277	949.7405	11.04	NEG	RP				5.95E-01	2.88E-01	0.33	1.42E-01	0.42	5.74E-02	0.56	1.47E-01	0.42
950.2093@0.39620593	950.2093	0.40	NEG	hilic				6.63E-01	6.09E-01	-0.11	3.71E-01	0.21	7.09E-01	-0.09	3.00E-01	-0.20
951.4678@0.5167735	951.4678	0.52	POS	hilic				6.90E-01	3.28E-01	-0.23	3.15E-01	-0.24	5.37E-01	0.16	5.43E-01	-0.15
951.4987@12.266018	951.4987	12.27	NEG	RP				7.76E-01	2.36E-01	0.30	1.69E-01	0.42	3.37E-01	0.25	2.29E-01	0.39
951.5142@12.676589	951.5142	12.68	NEG	RP				3.51E-02	1.26E-03	0.92	2.09E-01	0.37	9.90E-01	0.00	1.69E-02	0.71
951.5335@10.55448	951.5335	10.55	NEG	RP				2.81E-01	1.69E-02	0.66	2.70E-01	0.30	4.56E-01	0.22	3.62E-02	0.65
951.7306@11.036889	951.7306	11.04	NEG	RP				4.34E-01	1.17E-01	0.51	4.76E-02	0.56	4.07E-02	0.59	1.10E-01	0.48
952.4873@11.775201	952.4873	11.78	NEG	RP				2.18E-01	4.05E-01	-0.21	2.07E-02	-0.47	6.06E-01	0.13	5.93E-01	0.14
952.548@10.922222	952.5480	10.92	POS	RP				7.34E-01	6.93E-01	0.09	5.63E-01	-0.10	2.34E-01	0.25	5.79E-01	0.12
952.5522@1.9543927	952.5522	1.95	POS	hilic				9.58E-04	9.50E-02	0.51	1.14E-02	-0.62	5.56E-03	-0.74	3.64E-01	0.25
952.5819@0.40633333	952.5819	0.41	NEG	hilic				9.48E-01	5.06E-01	-0.17	6.63E-01	-0.10	9.23E-01	0.02	9.09E-01	0.03
952.6256@9.634145	952.6256	9.63	NEG	RP				3.45E-01	8.21E-03	-0.61	9.64E-01	-0.01	2.65E-01	-0.30	3.04E-01	-0.27
952.7021@10.795917	952.7021	10.80	NEG	RP				3.62E-01	6.20E-01	-0.10	6.63E-01	0.10	6.65E-02	0.48	6.63E-01	-0.10
952.7344@11.036213	952.7344	11.04	NEG	RP				5.22E-01	1.29E-01	0.49	7.34E-02	0.51	4.86E-02	0.55	1.88E-01	0.39
953.1726@3.5768852	953.1726	3.58	NEG	hilic				2.83E-01	6.23E-02	-0.61	7.06E-01	-0.09	1.02E-01	-0.38	2.54E-02	-0.54
953.498@12.258013	953.4980	12.26	NEG	RP				4.53E-01	3.33E-02	-0.55	6.61E-02	-0.50	2.51E-01	-0.31	3.07E-01	-0.28
953.5149@12.673338	953.5149	12.67	NEG	RP				2.79E-01	2.02E-02	0.71	1.34E-01	0.42	8.82E-01	0.04	1.25E-01	0.49
954.2135@0.39636025	954.2135	0.40	NEG	hilic				7.50E-01	5.08E-01	0.17	2.11E-01	0.38	7.22E-01	0.10	1.96E-01	0.42
954.518@12.681916	954.5180	12.68	NEG	RP				5.75E-02	9.88E-03	0.76	7.36E-01	0.06	4.85E-01	0.16	2.47E-02	0.61
954.5614@10.923123	954.5614	10.92	POS	RP				9.35E-01	9.29E-01	0.02	4.76E-01	-0.18	7.44E-01	0.08	8.61E-01	-0.05

Supplementary table 2: Metabolomics analysis results

Compound	Mass	Retention Time	Ionization mode	column	Compound_ID	MSMS_spectra	ID_level	ANOVA	Anorexia vs control		overweight vs control		obese vs control		athletes vs control	
								P-FDR	P	Estimate	P	Estimate	P	Estimate	P	Estimate
954.6076@10.776584	954.6076	10.78	POS	RP				8.64E-03	1.35E-01	-0.41	1.32E-02	0.70	1.69E-02	0.59	9.77E-01	0.01
955.1708@3.5787697	955.1708	3.58	NEG	hilic				5.60E-01	6.83E-01	-0.13	6.32E-01	-0.13	6.88E-01	0.11	3.34E-02	-0.50
955.4701@0.5169667	955.4701	0.52	POS	hilic				5.54E-01	2.35E-01	-0.32	3.87E-01	0.25	7.90E-01	0.08	4.31E-01	-0.21
956.1942@9.434104	956.1942	9.43	NEG	RP				8.96E-01	3.52E-01	-0.27	7.45E-01	0.09	8.35E-01	0.06	7.65E-01	-0.10
956.1954@0.39627337	956.1954	0.40	NEG	hilic				5.59E-01	4.19E-01	0.22	5.71E-02	0.60	4.85E-01	0.19	2.59E-01	0.37
956.6114@10.7763195	956.6114	10.78	POS	RP				2.97E-02	2.15E-01	-0.31	8.73E-02	0.52	6.09E-03	0.66	9.62E-01	-0.01
956.9785@3.3643281	956.9785	3.36	NEG	hilic				6.81E-01	9.45E-01	0.02	7.86E-01	0.08	1.17E-01	0.48	3.57E-01	0.29
956.9788@0.98808205	956.9788	0.99	NEG	hilic				3.92E-02	8.08E-03	0.74	3.57E-01	0.22	4.77E-01	-0.15	3.66E-02	0.61
956.9793@3.4051971	956.9793	3.41	NEG	hilic				9.19E-01	8.80E-01	-0.05	7.59E-01	0.09	3.93E-01	0.25	4.34E-01	0.21
957.1661@3.5735908	957.1661	3.57	NEG	hilic				3.01E-01	5.44E-01	-0.18	7.82E-01	-0.08	3.76E-01	0.22	1.37E-02	-0.57
957.4843@0.50864	957.4843	0.51	POS	hilic				4.43E-01	2.41E-01	-0.21	2.32E-01	-0.21	3.92E-01	0.22	4.78E-01	-0.13
957.5143@12.057872	957.5143	12.06	NEG	RP				1.38E-01	2.89E-01	0.29	6.89E-01	-0.07	9.16E-01	-0.02	2.17E-02	0.59
958.1433@9.084675	958.1433	9.08	NEG	RP				4.39E-01	8.49E-02	-0.56	8.78E-02	-0.50	1.06E-01	-0.46	2.88E-02	-0.59
958.2108@9.490444	958.2108	9.49	NEG	RP				9.22E-01	6.30E-01	-0.14	5.01E-01	0.19	6.13E-01	0.14	8.45E-01	0.06
958.2109@9.4350605	958.2109	9.44	NEG	RP				9.00E-01	2.55E-01	-0.33	9.60E-01	-0.01	9.84E-01	-0.01	9.65E-01	-0.01
958.2126@0.39618006	958.2126	0.40	NEG	hilic				7.72E-01	5.17E-01	0.18	2.41E-01	0.37	6.93E-01	0.11	1.92E-01	0.41
958.6063@10.77628	958.6063	10.78	POS	RP				1.65E-02	1.56E-01	-0.38	3.38E-02	0.62	2.06E-02	0.59	7.35E-01	-0.10
958.6105@10.712137	958.6105	10.71	NEG	RP				3.53E-02	7.26E-01	-0.08	8.69E-01	-0.04	8.47E-03	0.79	9.20E-01	-0.02
958.6574@10.028345	958.6574	10.03	POS	RP				2.11E-01	1.20E-01	-0.40	8.70E-01	0.05	8.91E-01	0.04	1.92E-02	-0.67
958.9771@3.4666493	958.9771	3.47	NEG	hilic				8.08E-01	8.83E-01	-0.05	3.66E-01	0.23	6.26E-01	0.15	5.04E-01	-0.18
959.2144@9.441213	959.2144	9.44	NEG	RP				6.68E-01	9.73E-01	-0.01	8.75E-01	-0.04	2.44E-01	0.35	4.89E-01	-0.25
959.2149@0.3961879	959.2149	0.40	NEG	hilic				7.10E-01	4.00E-01	0.23	1.54E-01	0.44	6.13E-01	0.14	2.07E-01	0.43
960.606@10.775959	960.6060	10.78	POS	RP				5.89E-02	6.63E-01	-0.11	1.45E-02	0.66	1.59E-01	0.37	5.35E-01	-0.16
960.6144@10.710813	960.6144	10.71	NEG	RP				7.70E-01	5.96E-01	0.14	4.69E-01	0.24	4.76E-01	0.21	4.60E-01	-0.19
960.6146@1.8732797	960.6146	1.87	POS	hilic				8.95E-04	2.04E-02	0.66	1.18E-01	-0.38	3.13E-02	-0.52	1.77E-01	0.34
960.6188@1.3940632	960.6188	1.39	POS	hilic				2.54E-02	3.68E-02	0.62	8.05E-01	-0.05	3.86E-01	0.19	2.75E-03	0.77
961.5284@3.0223708	961.5284	3.02	POS	hilic				9.63E-01	9.25E-01	-0.03	7.83E-01	0.08	4.22E-01	0.22	6.63E-01	0.14
961.5294@3.0233476	961.5294	3.02	POS	hilic				9.07E-01	3.90E-01	-0.29	8.86E-01	0.04	8.02E-01	-0.08	5.47E-01	-0.19
961.551@12.923665	961.5510	12.92	NEG	RP				6.76E-01	1.93E-01	0.43	8.53E-01	0.05	5.90E-01	-0.13	9.03E-01	0.04
961.618@10.710269	961.6180	10.71	NEG	RP				7.74E-01	1.63E-01	0.44	2.46E-01	0.33	3.79E-01	0.24	2.56E-01	0.27
961.63@11.040908	961.6300	11.04	POS	RP				8.28E-01	3.44E-01	0.31	4.32E-01	0.24	2.56E-01	0.36	2.83E-01	0.37
961.6632@10.923936	961.6632	10.92	POS	RP				8.16E-01	5.47E-01	-0.16	3.91E-01	-0.20	5.80E-01	0.14	9.00E-01	-0.03
962.185@9.217866	962.1850	9.22	NEG	RP				1.88E-01	5.57E-01	-0.16	7.24E-01	-0.11	9.19E-03	-0.82	5.91E-01	-0.15
962.1856@9.62464	962.1856	9.62	NEG	RP				6.89E-01	6.69E-01	-0.12	3.18E-01	0.30	2.30E-01	0.39	7.18E-01	0.12
962.1858@9.460623	962.1858	9.46	NEG	RP				5.60E-01	2.41E-01	-0.34	8.72E-01	0.05	2.74E-01	-0.32	4.76E-01	0.22
962.1866@0.3961879	962.1866	0.40	NEG	hilic				8.94E-01	3.65E-01	0.26	7.11E-01	-0.11	6.05E-01	0.15	9.95E-01	0.00
962.6361@1.357052	962.6361	1.36	POS	hilic				3.32E-02	1.21E-03	0.96	2.34E-01	0.30	4.15E-03	0.79	2.91E-02	0.59
962.6376@1.3546152	962.6376	1.35	POS	hilic				2.08E-01	7.69E-03	0.77	5.76E-01	0.14	9.05E-02	0.48	1.06E-01	0.42
962.722@11.032523	962.7220	11.03	POS	RP				7.65E-01	2.50E-01	0.30	1.34E-01	0.43	6.48E-01	0.12	4.23E-01	0.23
963.5256@3.0410173	963.5256	3.04	POS	hilic				7.68E-01	2.63E-01	-0.35	9.29E-01	0.02	7.04E-01	0.13	6.61E-01	0.13
963.5345@12.051554	963.5345	12.05	NEG	RP				2.79E-01	9.83E-02	-0.43	7.10E-02	-0.43	9.80E-03	-0.64	3.32E-01	-0.27
963.5405@0.71502024	963.5405	0.72	NEG	hilic				5.36E-01	4.65E-01	-0.22	9.54E-01	0.02	1.49E-01	0.42	6.59E-01	-0.12
963.6213@10.710022	963.6213	10.71	NEG	RP				7.55E-01	3.33E-01	0.27	1.41E-01	0.43	2.16E-01	0.34	4.71E-01	0.19
963.6425@1.3524839	963.6425	1.35	POS	hilic				3.05E-01	7.81E-02	0.58	7.96E-01	-0.07	9.81E-01	0.01	1.46E-01	0.39
963.777@4.4771543	963.7770	4.48	POS	hilic				5.56E-01	5.58E-02	0.60	5.14E-01	0.20	4.87E-01	0.20	9.75E-01	-0.01
963.8352@4.5637755	963.8352	4.56	NEG	hilic				5.03E-01	1.75E-01	0.41	8.41E-02	0.52	3.60E-01	0.27	4.08E-02	0.58
964.6288@10.519182	964.6288	10.52	NEG	RP				9.10E-01	6.94E-01	0.13	2.95E-01	0.25	4.76E-01	0.17	2.98E-01	0.29
964.6529@11.031616	964.6529	11.03	POS	RP				1.89E-01	4.70E-02	0.59	1.30E-02	0.72	1.28E-02	0.65	4.36E-02	0.70
965.4679@0.5008967	965.4679	0.50	POS	hilic				8.08E-01	6.79E-01	0.12	7.16E-01	-0.09	2.78E-01	0.27	4.37E-01	0.24
965.525@3.050759	965.5250	3.05	POS	hilic				8.92E-01	7.43E-01	0.11	7.46E-01	0.08	4.52E-01	-0.23	6.54E-01	-0.13
966.1285@9.461152	966.1285	9.46	NEG	RP				2.77E-01	8.24E-02	-0.54	3.17E-01	-0.26	9.18E-01	-0.03	1.79E-02	-0.68
966.2178@0.4029642	966.2178	0.40	NEG	hilic				5.96E-01	2.07E-02	-0.54	4.40E-01	-0.19	3.76E-01	-0.25	5.29E-01	-0.16
966.5848@11.044403	966.5848	11.04	POS	RP				8.32E-01	5.74E-01	0.18	3.38E-01	-0.27	6.61E-01	-0.12	8.71E-01	-0.05
967.4662@0.50105625	967.4662	0.50	POS	hilic				5.45E-01	1.34E-01	-0.42	5.15E-01	0.18	6.77E-01	0.12	5.13E-01	-0.17
967.563@0.76843065	967.5630	0.77	POS	hilic				8.66E-01	9.61E-01	0.01	2.94E-01	-0.28	8.70E-01	-0.04	9.25E-01	0.03
967.5686@12.960965	967.5686	12.96	NEG	RP				9.58E-01	4.25E-01	0.25	6.33E-01	0.12	6.24E-01	0.13	5.14E-01	0.18
967.5888@11.044834	967.5888	11.04	POS	RP				2.87E-01	1.67E-01	0.41	9.06E-01	-0.03	1.43E-01	-0.36	4.53E-01	0.21
968.5603@12.700528	968.5603	12.70	NEG	RP				4.95E-01	6.03E-02	0.45	3.45E-01	0.24	8.13E-01	-0.05	2.23E-01	0.30
968.6721@10.783038	968.6721	10.78	NEG	RP				5.53E-01	3.11E-01	-0.25	4.02E-01	0.24	3.20E-01	0.29	3.83E-01	0.23
969.1499@3.5345979	969.1499	3.53	NEG	hilic				7.75E-01	6.12E-01	0.18	9.72E-01	-0.01	2.70E-01	0.32	5.22E-01	-0.15
969.478@0.5025358	969.4780	0.50	POS	hilic				7.42E-01	6.08E-01	-0.17	9.23E-01	-0.02	1.06E-01	-0.45	4.17E-01	-0.26
969.5796@3.2044852	969.5796	3.20	NEG	hilic				4.10E-01	9.82E-03	0.74	9.99E-02	0.44	2.58E-01	0.30	2.76E-01	0.37
969.58@3.1884854	969.5800	3.19	NEG	hilic				5.28E-01	3.60E-02	0.66	1.11E-01	0.40	3.69E-01	0.24	3.65E-01	0.30
970.58@10.712023	970.5800	10.71	NEG	RP				8.01E-01	1.08E-01	0.43	4.82E-01	0.20	3.19E-01	0.30	6.26E-01	0.15
971.082@3.2047443	971.0820	3.20	NEG	hilic				7.40E-01	2.43E-01	0.35	8.32E-01	0.05	4.61E-01	0.19	7.56E-01	-0.07
971.1455@3.5351124	971.1455	3.54	NEG	hilic				8.09E-01	8.38E-01	-0.06	1.26E-01	-0.38	7.27E-01	-0.10	8.64E-01	-0.04
971.1892@0.39575425	971.1892	0.40	NEG	hilic				9.18E-01	8.37E-01	-0.07	3.09E-01	-0.29	8.84E-01	0.04	8.07E-01	-0.07
971.4822@11.861989	971.4822	11.86	NEG	RP				9.19E-01	2.81E-01	0.29	5.51E-01	0.16	5.53E-01	0.17	8.56E-01	0.04
971.7287@11.030568	971.7287	11.03	POS	RP				5.88E-01	3.59E-01	0.26	1.80E-01	0.41	8.65E-01	0.04	6.45E-02	0.46
972.5516@9.614532	972.5516	9.61	POS	RP				1.98E-01	7.26E-02	0.50	7.16E-01	-0.07	5.46E-02	0.44	8.47E-02	0.46
972.6169@9.9906845	972.6169	9.99	NEG	RP				8.96E-05	4.14E-03	0.80	9.22E-03	-0.68	8.94E-02	-0.48	5.50E-02	0.47
972.6193@9.990016	972.6193	9.99	POS	RP				8.95E-04	5.10E-02	0.65	5.67E-03	-0.70	1.66E-01	-0.36	8.02E-02	0.41
973.1422@3.5343463	973.1422	3.53	NEG	hilic				9.43E-01	4.06E-01	-0.29	8.43E-01	-0.05	8.93E-01	-0.04	8.46E-01	-0.05

Supplementary table 2: Metabolomics analysis results

Compound	Mass	Retention Time	Ionization mode	column	Compound_ID	MSMS_spectra	ID_level	ANOVA P-FDR	Anorexia vs control P	Anorexia vs control Estimate	overweight vs control P	overweight vs control Estimate	obese vs control P	obese vs control Estimate	athletes vs control P	athletes vs control Estimate
973.496@11.987592	973.4960	11.99	NEG	RP				7.89E-03	9.59E-01	-0.01	2.95E-02	-0.65	3.27E-03	-0.90	4.56E-01	0.20
973.4972@12.12482	973.4972	12.12	NEG	RP				1.90E-01	2.10E-03	0.77	2.80E-01	0.34	1.06E-02	0.74	4.77E-02	0.51
973.5778@3.2362447	973.5778	3.24	NEG	hilic				5.36E-01	4.50E-01	0.19	6.49E-01	-0.13	7.65E-01	0.10	5.43E-02	0.50
973.8052@4.4001203	973.8052	4.40	POS	hilic				3.77E-01	2.71E-01	0.35	2.36E-01	0.29	5.24E-01	-0.19	2.35E-01	-0.31
974.2063@0.39626074	974.2063	0.40	NEG	hilic				6.94E-01	1.11E-01	0.46	4.62E-01	0.24	7.74E-01	-0.08	6.18E-01	0.16
974.5358@1.957476	974.5358	1.96	POS	hilic				6.68E-04	1.84E-01	0.41	2.60E-02	-0.53	2.44E-03	-0.73	1.42E-01	0.36
974.5636@10.711115	974.5636	10.71	NEG	RP				3.11E-02	1.05E-02	0.75	1.29E-01	0.38	1.17E-02	0.57	5.59E-01	-0.11
974.6324@10.128193	974.6324	10.13	NEG	RP				1.08E-03	3.95E-02	0.58	3.91E-02	-0.58	7.03E-03	-0.79	6.03E-01	0.14
974.6332@10.144367	974.6332	10.14	NEG	RP				5.69E-02	4.43E-02	0.65	6.03E-01	-0.12	1.60E-01	-0.32	1.81E-01	0.35
974.6359@10.215549	974.6359	10.22	POS	RP				1.37E-01	1.27E-01	0.47	7.49E-01	-0.10	4.95E-01	-0.15	2.21E-02	0.60
974.6426@1.3327311	974.6426	1.33	POS	hilic				9.35E-02	6.75E-03	0.84	5.35E-01	0.18	1.87E-01	0.36	1.46E-02	0.73
974.6771@1.1282854	974.6771	1.13	POS	hilic				2.91E-02	7.15E-01	0.10	1.30E-01	-0.39	6.11E-04	-0.80	6.69E-01	0.13
974.7188@11.036122	974.7188	11.04	NEG	RP				3.78E-01	6.89E-01	-0.11	8.17E-01	0.07	3.41E-02	0.55	5.51E-01	-0.18
975.1407@3.5336463	975.1407	3.53	NEG	hilic				8.81E-01	4.86E-01	0.24	7.35E-01	-0.09	7.98E-01	0.08	5.59E-01	-0.12
975.2006@9.494939	975.2006	9.49	NEG	RP				8.60E-01	3.21E-01	0.28	6.58E-01	-0.13	9.71E-01	0.01	6.52E-01	0.15
975.205@0.39614528	975.2050	0.40	NEG	hilic				9.83E-01	6.09E-01	0.16	8.17E-01	-0.07	9.88E-01	0.00	8.40E-01	0.06
975.4972@12.030871	975.4972	12.03	NEG	RP				5.12E-02	1.24E-01	0.48	1.86E-01	-0.31	9.14E-02	-0.46	4.26E-01	0.22
975.5139@12.4236145	975.5139	12.42	NEG	RP				1.66E-01	1.12E-02	0.83	7.21E-01	0.10	4.92E-01	0.22	6.15E-02	0.54
975.6678@10.780962	975.6678	10.78	POS	RP				1.12E-01	6.66E-01	-0.14	9.28E-02	0.46	1.07E-02	0.69	9.09E-01	-0.04
975.7009@11.030013	975.7009	11.03	POS	RP				5.63E-01	6.04E-02	0.49	9.10E-02	0.56	2.50E-01	0.36	3.51E-01	0.28
976.5512@2.1577096	976.5512	2.16	NEG	hilic				5.87E-03	1.09E-01	0.45	5.81E-02	-0.37	4.07E-02	-0.40	2.44E-01	0.28
976.6481@10.115227	976.6481	10.12	NEG	RP				2.24E-04	8.06E-04	0.90	1.90E-01	-0.35	4.06E-01	-0.22	2.47E-03	0.85
976.6566@1.3323648	976.6566	1.33	POS	hilic				1.27E-01	3.45E-02	0.62	4.42E-01	-0.21	6.27E-01	-0.13	1.25E-01	0.43
977.1377@3.527365	977.1377	3.53	NEG	hilic				2.13E-01	3.17E-01	0.33	2.87E-01	0.26	9.75E-02	-0.39	4.38E-01	0.18
977.5168@12.43193	977.5168	12.43	NEG	RP				4.05E-01	1.13E-01	0.49	3.01E-01	-0.28	9.64E-01	-0.01	8.29E-01	0.06
977.5287@12.916096	977.5287	12.92	NEG	RP				1.57E-01	3.12E-01	0.30	2.15E-01	-0.32	1.22E-01	-0.46	3.19E-01	0.32
977.6609@1.3324469	977.6609	1.33	POS	hilic				7.23E-02	3.99E-03	0.82	8.42E-01	-0.06	6.51E-01	0.12	6.34E-02	0.59
978.1454@9.463835	978.1454	9.46	NEG	RP				8.01E-01	3.60E-01	-0.28	3.66E-01	-0.24	9.88E-01	0.00	2.42E-01	-0.37
978.1503@9.216838	978.1503	9.22	NEG	RP				8.35E-01	3.75E-01	-0.29	4.35E-01	-0.20	5.54E-01	-0.17	1.83E-01	-0.41
978.2089@0.39753497	978.2089	0.40	NEG	hilic				4.27E-01	3.16E-02	0.68	3.24E-01	0.29	6.12E-01	0.14	8.52E-01	0.05
978.5707@10.709151	978.5707	10.71	NEG	RP				2.99E-01	9.34E-03	0.67	7.94E-02	0.49	1.98E-01	0.30	7.64E-02	0.41
978.5715@9.961428	978.5715	9.96	POS	RP				5.52E-01	4.22E-02	0.63	5.19E-01	0.17	6.81E-01	0.14	2.32E-01	0.33
979.4755@0.50203675	979.4755	0.50	POS	hilic				6.34E-01	2.55E-01	-0.32	5.11E-01	-0.16	1.56E-01	-0.38	7.27E-01	0.10
979.4902@11.864777	979.4902	11.86	NEG	RP				4.34E-01	4.99E-02	0.55	9.99E-01	0.00	8.40E-01	0.05	4.13E-01	0.20
979.7014@11.028404	979.7014	11.03	POS	RP				9.14E-01	2.50E-01	0.32	8.54E-01	0.05	9.26E-01	0.03	7.55E-01	0.08
979.9849@0.54215527	979.9849	0.54	NEG	hilic				2.77E-01	5.38E-01	0.22	4.84E-01	0.21	1.75E-01	0.35	8.59E-02	-0.44
980.1442@9.394392	980.1442	9.39	NEG	RP				6.98E-01	3.90E-01	-0.29	6.20E-01	-0.14	9.01E-01	0.03	1.19E-01	-0.45
980.1444@9.216593	980.1444	9.22	NEG	RP				7.10E-01	4.61E-01	-0.24	1.84E-01	-0.34	3.07E-01	-0.29	7.69E-02	-0.52
980.1444@9.463366	980.1444	9.46	NEG	RP				9.50E-01	5.08E-01	-0.22	7.30E-01	-0.09	9.61E-01	-0.01	4.08E-01	-0.24
980.1445@9.625434	980.1445	9.63	NEG	RP				4.85E-01	2.40E-01	-0.34	7.09E-01	0.12	4.70E-01	0.19	1.92E-01	-0.37
980.5114@12.275683	980.5114	12.28	NEG	RP				2.96E-01	6.36E-01	0.10	3.08E-01	0.22	6.50E-01	0.09	2.23E-02	0.60
980.5848@1.8689643	980.5848	1.87	POS	hilic				4.59E-03	1.40E-02	0.70	1.07E-01	-0.45	8.25E-02	-0.51	3.42E-01	0.27
980.6071@9.613247	980.6071	9.61	NEG	RP				2.91E-01	1.40E-01	0.39	9.70E-01	-0.01	3.32E-01	0.25	3.25E-02	0.59
981.1471@9.4641075	981.1471	9.46	NEG	RP				5.31E-01	8.88E-01	0.05	4.74E-02	0.48	6.92E-01	0.11	5.80E-01	-0.17
981.1472@9.216796	981.1472	9.22	NEG	RP				6.37E-01	3.70E-01	-0.31	2.95E-01	-0.30	3.24E-01	-0.30	4.16E-02	-0.58
981.1473@9.626134	981.1473	9.63	NEG	RP				4.37E-01	8.67E-01	-0.05	2.80E-01	-0.31	2.54E-01	0.33	3.04E-01	-0.32
981.4715@0.5224118	981.4715	0.52	POS	hilic				9.63E-01	9.66E-01	0.01	5.45E-01	0.13	7.22E-01	-0.07	9.90E-01	0.00
981.5782@0.5395425	981.5782	0.54	POS	hilic				9.51E-01	5.70E-01	0.16	7.40E-01	-0.07	6.79E-01	0.10	7.45E-01	0.08
981.7114@10.796593	981.7114	10.80	NEG	RP				3.97E-01	7.92E-01	-0.05	3.02E-01	0.26	5.45E-02	0.44	8.38E-01	-0.04
981.7158@11.028035	981.7158	11.03	POS	RP				1.14E-01	7.68E-03	0.74	3.91E-01	-0.23	4.67E-01	0.18	8.45E-01	-0.06
981.7709@11.05895	981.7709	11.06	POS	RP				9.14E-01	5.37E-01	-0.15	8.86E-01	0.04	5.60E-01	0.16	7.23E-01	-0.08
982.2056@0.39714113	982.2056	0.40	NEG	hilic				5.93E-01	9.21E-02	0.48	1.01E-01	0.48	4.08E-01	0.25	1.54E-01	0.36
982.5322@12.682155	982.5322	12.68	NEG	RP				2.16E-01	7.71E-02	0.56	9.84E-01	0.01	1.44E-01	-0.36	5.23E-01	0.17
982.5985@1.8642151	982.5985	1.86	POS	hilic				4.11E-03	4.85E-02	0.60	1.58E-01	-0.37	3.59E-02	-0.54	1.85E-01	0.35
982.7195@11.029642	982.7195	11.03	POS	RP				1.87E-01	2.09E-02	0.73	5.59E-01	-0.16	6.53E-01	0.13	5.04E-01	0.20
983.1292@3.4977553	983.1292	3.50	NEG	hilic				7.76E-01	4.10E-01	0.26	8.46E-01	-0.05	2.54E-01	0.36	4.23E-01	0.19
983.5179@12.444709	983.5179	12.44	NEG	RP				1.85E-02	9.39E-01	0.02	4.76E-04	-0.82	2.08E-02	-0.66	3.42E-01	-0.25
983.6112@10.711	983.6112	10.71	NEG	RP				9.00E-01	3.46E-01	-0.22	7.70E-01	0.08	4.88E-01	-0.16	8.66E-01	-0.05
983.9969@0.9684963	983.9969	0.97	NEG	hilic				3.59E-01	2.93E-01	0.32	2.24E-01	-0.34	2.33E-01	-0.36	7.21E-01	0.11
983.9976@15.271886	983.9976	15.27	NEG	RP				8.95E-01	3.09E-01	-0.34	9.96E-01	0.00	9.53E-01	-0.02	8.72E-01	-0.05
983.9976@15.403788	983.9976	15.40	NEG	RP				9.42E-01	5.22E-01	0.19	5.90E-01	0.17	7.60E-01	0.10	3.36E-01	0.30
983.9993@0.9684963	983.9993	0.97	NEG	hilic				3.75E-01	3.54E-01	0.29	2.09E-01	-0.34	1.91E-01	-0.40	7.94E-01	0.08
984.2251@0.3960861	984.2251	0.40	NEG	hilic				5.55E-01	9.61E-01	0.01	7.69E-02	0.54	8.99E-01	0.03	3.97E-01	0.27
984.6169@1.3496855	984.6169	1.35	POS	hilic				7.10E-01	2.34E-01	0.34	9.42E-01	-0.02	4.97E-01	0.20	1.79E-01	0.40
985.1251@3.4973965	985.1251	3.50	NEG	hilic				8.94E-01	6.43E-01	0.17	5.58E-01	-0.14	4.94E-01	0.20	8.34E-01	0.05
985.7432@11.060056	985.7432	11.06	NEG	RP				5.04E-01	3.69E-02	0.58	1.31E-01	0.38	1.86E-01	0.33	1.05E-01	0.47
986.2411@0.39618906	986.2411	0.40	NEG	hilic				7.20E-01	9.33E-01	0.02	2.14E-01	0.38	9.36E-01	-0.02	3.01E-01	0.33
986.6159@10.880033	986.6159	10.88	NEG	RP				9.35E-01	4.20E-01	-0.18	4.59E-01	-0.16	5.86E-01	-0.12	4.05E-01	-0.18
986.6326@1.3206949	986.6326	1.32	POS	hilic				2.73E-01	4.90E-02	0.63	7.64E-01	-0.09	1.28E-01	0.45	2.34E-01	0.35
986.6326@9.791688	986.6326	9.79	POS	RP				5.53E-01	2.52E-01	0.33	3.35E-01	-0.25	5.05E-01	0.17	7.50E-01	0.08
987.1196@3.3279524	987.1196	3.33	NEG	hilic				5.89E-01	3.81E-01	0.20	2.74E-01	-0.14	2.98E-01	0.21	7.11E-01	0.07
987.1205@3.4972918	987.1205	3.50	NEG	hilic				8.86E-01	5.59E-01	0.21	4.41E-01	-0.19	7.88E-01	0.08	9.18E-01	-0.03
987.2441@0.39713782	987.2441	0.40	NEG	hilic				6.62E-01	7.99E-01	0.07	7.71E-02	0.50	5.06E-01	0.20	3.16E-01	0.35

Supplementary table 2: Metabolomics analysis results

								ANOVA	Anorexia vs control		overweight vs control		obese vs control		athletes vs control	
Compound	Mass	Retention Time	Ionization mode	column	Compound_ID	MSMS_spectra	ID_level	P-FDR	P	Estimate	P	Estimate	P	Estimate	P	Estimate
987.5391@12.052441	987.5391	12.05	NEG	RP				9.14E-01	2.50E-01	-0.32	9.80E-01	-0.01	7.13E-01	-0.11	8.49E-01	-0.05
987.6158@10.710119	987.6158	10.71	NEG	RP				6.21E-01	5.53E-01	-0.15	1.08E-01	-0.44	7.58E-01	-0.09	7.85E-01	0.07
987.6435@0.3912632	987.6435	0.39	POS	hilic				8.25E-01	6.23E-01	-0.13	1.71E-01	-0.30	2.89E-01	-0.24	5.83E-01	-0.13
987.6444@11.2259445	987.6444	11.23	POS	RP				8.18E-01	4.67E-01	-0.18	4.26E-01	-0.17	6.04E-01	0.12	9.78E-01	-0.01
987.7963@4.6137543	987.7963	4.61	NEG	hilic				1.86E-01	3.66E-02	0.55	6.10E-02	0.46	6.35E-01	-0.09	2.77E-01	0.27
988.6277@10.878	988.6277	10.88	NEG	RP				7.45E-01	2.42E-01	-0.28	3.88E-01	-0.20	8.02E-01	0.06	9.41E-01	0.02
988.6506@1.1592765	988.6506	1.16	POS	hilic				2.32E-02	1.16E-01	0.55	1.79E-01	-0.30	8.33E-01	-0.06	8.06E-03	0.70
988.6511@1.3021715	988.6511	1.30	POS	hilic				8.15E-03	2.70E-03	0.88	1.46E-01	-0.37	7.18E-01	0.11	7.00E-02	0.50
988.6517@1.321815	988.6517	1.32	POS	hilic				5.58E-01	2.17E-01	0.42	5.05E-01	-0.19	5.08E-01	0.19	3.04E-01	0.28
989.0961@3.3008914	989.0961	3.30	POS	hilic				8.62E-01	3.61E-01	0.28	6.81E-01	-0.10	9.69E-01	-0.01	7.78E-01	0.07
989.1163@3.50011	989.1163	3.50	NEG	hilic				3.41E-01	9.34E-02	0.59	9.83E-01	0.00	1.25E-01	0.42	9.31E-01	-0.03
989.4564@11.769066	989.4564	11.77	NEG	RP				3.12E-01	4.48E-01	0.15	9.19E-01	0.02	2.20E-01	0.29	3.02E-02	0.52
989.6283@10.710361	989.6283	10.71	NEG	RP				6.15E-01	6.16E-01	0.14	8.74E-01	0.05	2.82E-01	0.33	2.86E-01	-0.29
989.6587@11.225239	989.6587	11.23	POS	RP				8.53E-01	5.52E-01	-0.16	4.81E-01	-0.17	5.57E-01	0.15	8.32E-01	-0.06
990.2072@0.39644107	990.2072	0.40	NEG	hilic				8.84E-01	2.24E-01	0.30	5.54E-01	0.18	2.46E-01	0.31	7.98E-01	0.09
990.5322@11.999387	990.5322	12.00	NEG	RP				2.28E-03	3.69E-01	0.25	2.25E-03	-0.87	2.28E-03	-0.80	9.13E-01	0.03
990.5364@12.123762	990.5364	12.12	NEG	RP				4.67E-01	7.66E-02	0.51	5.33E-01	-0.16	4.63E-01	0.21	3.35E-01	0.29
990.6617@11.225953	990.6617	11.23	POS	RP				7.74E-01	3.35E-01	-0.24	4.80E-01	-0.16	6.60E-01	0.11	8.90E-01	0.03
990.6667@10.110917	990.6667	10.11	POS	RP				9.50E-01	7.01E-01	-0.10	4.88E-01	0.19	8.55E-01	0.04	7.90E-01	0.08
991.1149@3.4978733	991.1149	3.50	NEG	hilic				9.50E-01	8.01E-01	0.09	4.92E-01	-0.17	9.33E-01	0.03	6.95E-01	0.10
991.5672@12.802557	991.5672	12.80	NEG	RP				7.04E-03	1.15E-02	-0.59	7.80E-01	0.07	4.81E-02	0.55	7.58E-02	-0.43
991.6681@1.1739653	991.6681	1.17	POS	hilic				4.20E-02	1.14E-01	0.46	4.92E-02	-0.49	4.05E-01	-0.25	1.56E-01	0.41
991.7426@10.789966	991.7426	10.79	NEG	RP				9.75E-02	7.07E-01	-0.10	2.93E-02	0.60	2.32E-02	0.64	3.18E-01	0.27
991.756@11.038724	991.7560	11.04	NEG	RP				4.50E-01	9.40E-02	-0.49	3.45E-01	-0.25	5.39E-01	-0.15	3.77E-02	-0.55
992.1884@0.3964667	992.1884	0.40	NEG	hilic				8.54E-01	5.56E-01	0.14	9.40E-01	0.02	2.79E-01	0.28	3.99E-01	0.22
992.5503@12.436662	992.5503	12.44	NEG	RP				3.59E-01	9.54E-01	0.02	4.90E-02	-0.52	8.90E-01	-0.04	1.11E-01	-0.48
993.1095@3.330403	993.1095	3.33	NEG	hilic				2.43E-01	2.51E-01	0.43	2.39E-01	-0.29	4.34E-01	0.19	8.80E-02	0.46
993.2441@9.66341	993.2441	9.66	POS	RP				1.95E-01	1.52E-01	-0.39	1.29E-01	0.47	7.36E-01	0.09	4.23E-01	-0.25
993.2444@9.51544	993.2444	9.52	POS	RP				6.10E-01	1.17E-01	0.47	3.21E-01	0.30	7.20E-02	0.55	3.33E-01	0.30
993.2455@9.499768	993.2455	9.50	POS	RP				6.65E-01	1.31E-01	0.46	3.39E-01	0.27	1.15E-01	0.51	3.02E-01	0.32
993.5522@12.434679	993.5522	12.43	NEG	RP				8.96E-01	5.44E-01	-0.16	1.64E-01	-0.29	4.65E-01	-0.18	7.41E-01	-0.09
993.6445@10.568524	993.6445	10.57	NEG	RP				3.64E-02	6.27E-04	-0.66	7.90E-01	0.07	4.62E-01	0.18	1.79E-01	-0.32
994.1255@9.2173	994.1255	9.22	NEG	RP				5.11E-01	9.60E-01	0.01	8.60E-01	0.05	5.19E-01	-0.22	6.18E-02	-0.54
994.1301@9.466763	994.1301	9.47	NEG	RP				8.18E-01	9.41E-01	0.02	9.40E-01	-0.02	9.81E-01	-0.01	2.22E-01	-0.38
994.5673@12.926912	994.5673	12.93	NEG	RP				5.44E-01	9.33E-01	-0.03	2.11E-01	-0.39	4.05E-01	0.26	8.12E-01	0.07
995.105@3.330407	995.1050	3.33	NEG	hilic				4.84E-01	5.27E-01	0.24	3.10E-01	-0.23	6.37E-01	0.13	8.13E-02	0.44
995.1299@9.468271	995.1299	9.47	NEG	RP				3.06E-01	1.61E-01	0.38	4.69E-01	0.18	9.39E-01	-0.02	2.63E-02	0.57
995.569@12.936426	995.5690	12.94	NEG	RP				8.80E-01	5.13E-01	0.16	4.24E-01	0.21	5.77E-01	0.16	2.03E-01	0.34
995.5921@0.45189646	995.5921	0.45	POS	hilic				8.09E-01	7.11E-01	-0.10	1.43E-01	-0.37	5.98E-01	-0.14	5.88E-01	-0.16
995.7737@11.070147	995.7737	11.07	NEG	RP				9.73E-02	1.64E-01	0.36	5.80E-01	-0.17	1.31E-02	-0.60	3.80E-01	0.27
995.9439@10.590167	995.9439	10.59	NEG	hilic				6.12E-01	6.58E-01	0.14	2.61E-01	0.28	1.70E-01	0.36	5.19E-02	0.57
996.6167@9.942808	996.6167	9.94	NEG	RP				2.54E-01	4.07E-01	0.31	4.51E-01	-0.20	5.48E-01	-0.17	7.30E-02	0.52
996.6173@9.967106	996.6173	9.97	NEG	RP				1.04E-03	7.14E-02	0.61	2.50E-02	-0.60	2.93E-02	-0.57	1.15E-01	0.41
996.618@9.961871	996.6180	9.96	POS	RP				1.54E-03	3.47E-02	0.60	4.96E-02	-0.52	4.46E-02	-0.59	1.05E-01	0.43
996.6298@1.3366274	996.6298	1.34	POS	hilic				4.42E-01	3.71E-02	0.60	9.69E-02	0.41	2.54E-01	0.25	5.04E-01	0.19
996.6563@1.1148224	996.6563	1.11	POS	hilic				3.95E-02	6.19E-01	0.14	8.50E-02	-0.47	1.98E-03	-0.72	6.43E-01	0.14
997.1019@3.330403	997.1019	3.33	NEG	hilic				4.02E-01	3.70E-01	0.33	3.06E-01	-0.24	4.40E-01	0.22	7.87E-02	0.44
997.4929@11.912069	997.4929	11.91	NEG	RP				8.20E-01	8.26E-01	0.06	5.47E-01	0.16	5.14E-01	-0.18	4.39E-01	0.20
997.4978@12.06413	997.4978	12.06	NEG	RP				6.05E-01	6.35E-01	-0.17	2.22E-01	-0.33	8.33E-01	-0.07	5.66E-02	-0.55
997.8283@4.562704	997.8283	4.56	NEG	hilic				2.03E-01	2.11E-02	0.80	6.01E-02	0.50	4.72E-01	0.21	1.98E-02	0.59
998.2127@0.4014684	998.2127	0.40	NEG	hilic				7.34E-01	2.39E-01	0.34	1.48E-01	0.44	3.28E-01	0.28	2.11E-01	0.41
998.496@11.904627	998.4960	11.90	NEG	RP				8.89E-01	4.07E-01	-0.21	4.82E-01	-0.17	2.91E-01	-0.31	6.81E-01	-0.10
998.5353@1.9511377	998.5353	1.95	POS	hilic				7.63E-04	3.28E-02	0.61	1.50E-02	-0.64	1.55E-02	-0.67	4.44E-01	0.22
998.632@10.142634	998.6320	10.14	NEG	RP				6.58E-01	4.70E-01	0.24	2.35E-01	-0.33	9.24E-01	0.03	9.69E-01	-0.01
998.6378@1.3362813	998.6378	1.34	POS	hilic				7.83E-01	2.66E-01	0.33	8.95E-01	-0.04	6.18E-01	-0.13	9.98E-01	0.00
998.9965@0.568523	998.9965	0.57	POS	hilic				7.25E-01	4.20E-01	0.23	2.21E-01	0.31	8.70E-01	-0.04	2.57E-01	0.32
999.0968@3.3303483	999.0968	3.33	NEG	hilic				3.61E-01	3.67E-01	0.32	5.80E-01	-0.15	2.18E-01	0.33	2.26E-02	0.54
999.1034@3.4813678	999.1034	3.48	NEG	hilic				7.15E-01	5.73E-01	0.18	2.66E-01	-0.24	6.94E-01	-0.10	4.80E-01	0.17
999.5918@10.71028	999.5918	10.71	NEG	RP				5.07E-01	6.62E-02	0.59	1.11E-01	0.46	3.27E-01	0.28	1.05E-01	0.47
999.6909@10.783068	999.6909	10.78	NEG	RP				8.91E-03	6.95E-02	-0.51	4.96E-02	0.56	2.85E-02	0.58	6.59E-01	-0.13
999.7801@0.41441023	999.7801	0.41	POS	hilic				7.84E-01	2.14E-01	0.27	5.75E-01	0.12	1.76E-01	0.32	5.79E-01	0.11
1000.2205@9.432709	1000.2205	9.43	NEG	RP				5.03E-01	7.33E-01	-0.12	8.40E-01	0.05	6.53E-02	0.54	9.77E-01	-0.01
1000.2214@9.207914	1000.2214	9.21	NEG	RP				7.86E-01	7.83E-01	-0.08	4.60E-01	0.23	2.89E-01	0.30	7.70E-01	-0.09
1000.2252@0.40190595	1000.2252	0.40	NEG	hilic				3.37E-01	1.21E-01	0.43	1.45E-02	0.75	1.17E-01	0.43	2.81E-01	0.38
1000.5611@9.859467	1000.5611	9.86	POS	RP				7.85E-01	1.94E-01	0.36	5.91E-01	0.12	2.57E-01	0.33	7.71E-01	0.08
1000.6485@10.2314005	1000.6485	10.23	NEG	RP				1.07E-03	8.08E-02	0.48	1.75E-02	-0.67	1.04E-02	-0.71	3.10E-01	0.30
1000.6494@10.227175	1000.6494	10.23	POS	RP				4.00E-03	7.20E-02	0.48	4.85E-03	-0.75	1.11E-02	-0.66	8.49E-01	-0.06
1000.6504@9.916521	1000.6504	9.92	POS	RP				9.75E-01	9.13E-01	0.03	9.80E-01	0.01	6.14E-01	0.14	4.38E-01	0.20
1000.6956@10.782408	1000.6956	10.78	NEG	RP				2.08E-02	6.87E-02	-0.46	2.21E-01	0.35	2.75E-02	0.57	4.43E-01	-0.21
1001.0947@3.330403	1001.0947	3.33	NEG	hilic				4.28E-01	3.93E-01	0.31	3.36E-01	-0.22	4.57E-01	0.21	7.92E-02	0.45
1001.0997@3.4791539	1001.0997	3.48	NEG	hilic				7.37E-01	4.78E-01	0.22	8.89E-01	0.03	7.55E-01	-0.08	1.71E-01	0.34
1001.2273@0.4008287	1001.2273	0.40	NEG	hilic				2.79E-01	1.72E-01	0.41	9.51E-03	0.77	1.19E-01	0.44	9.98E-02	0.52
1001.5917@10.710433	1001.5917	10.71	NEG	RP				6.71E-01	9.09E-02	0.57	2.48E-01	0.33	2.90E-01	0.29	4.02E-01	0.25

Supplementary table 2: Metabolomics analysis results

Compound	Mass	Retention Time	Ionization mode	column	Compound_ID	MSMS_spectra	ID_level	ANOVA	Anorexia vs control		overweight vs control		obese vs control		athletes vs control	
								P-FDR	P	Estimate	P	Estimate	P	Estimate	P	Estimate
1002.1519@10.126685	1002.1519	10.13	POS	RP				7.29E-01	3.33E-01	0.26	2.64E-01	0.37	8.00E-01	-0.08	3.27E-01	0.28
1002.5705@1.8312037	1002.5705	1.83	POS	hilic				2.90E-02	3.16E-01	0.30	6.73E-02	-0.50	3.10E-02	-0.65	4.18E-01	0.21
1002.5724@1.8302835	1002.5724	1.83	POS	hilic				9.30E-02	1.27E-01	0.44	1.72E-01	-0.39	1.55E-01	-0.45	4.69E-01	0.20
1002.6441@1.20772	1002.6441	1.21	POS	hilic				1.40E-02	1.50E-03	0.81	1.96E-01	0.16	6.74E-03	0.57	9.98E-02	0.31
1002.6699@1.2020818	1002.6699	1.20	POS	hilic				4.72E-01	1.23E-01	0.41	3.74E-01	-0.19	6.30E-01	-0.11	9.56E-01	-0.01
1002.6804@10.021802	1002.6804	10.02	POS	RP				7.37E-01	1.30E-01	0.41	7.40E-01	0.10	7.03E-01	0.10	7.02E-01	-0.12
1002.6926@1.2851976	1002.6926	1.29	POS	hilic				6.06E-03	1.22E-03	1.02	2.44E-01	-0.32	5.35E-01	0.15	4.74E-01	0.20
1002.6964@10.501737	1002.6964	10.50	POS	RP				7.56E-01	2.87E-01	0.31	8.24E-01	-0.05	6.17E-01	-0.11	7.70E-01	-0.07
1002.7002@1.0905403	1002.7002	1.09	POS	hilic				2.56E-01	3.32E-01	-0.27	3.93E-02	-0.47	1.43E-02	-0.54	9.34E-01	-0.02
1003.0714@3.159727	1003.0714	3.16	POS	hilic				3.30E-01	1.69E-01	0.50	8.38E-01	0.05	8.04E-01	0.07	1.88E-02	0.62
1003.0917@3.3311422	1003.0917	3.33	NEG	hilic				4.97E-01	3.70E-01	0.34	3.42E-01	-0.23	4.08E-01	0.24	1.46E-01	0.35
1003.0925@3.4784787	1003.0925	3.48	NEG	hilic				2.18E-01	5.80E-02	0.62	8.22E-01	0.05	6.61E-02	0.53	4.19E-02	0.54
1003.5421@13.220419	1003.5421	13.22	NEG	RP				1.15E-01	8.49E-01	0.05	8.44E-01	0.05	2.73E-03	0.77	6.27E-01	0.13
1003.607@10.710718	1003.6070	10.71	NEG	RP				7.64E-01	1.88E-01	0.40	2.22E-01	0.40	2.43E-01	0.36	2.81E-01	0.31
1003.6326@10.87712	1003.6326	10.88	POS	RP				9.80E-01	8.92E-01	0.03	8.52E-01	0.04	4.74E-01	0.16	8.10E-01	0.05
1003.7351@11.035681	1003.7351	11.04	NEG	RP				1.48E-01	7.47E-01	0.09	7.71E-01	0.09	7.42E-03	0.74	4.77E-01	-0.18
1004.2035@0.3965298	1004.2035	0.40	NEG	hilic				6.02E-01	6.56E-02	0.53	1.30E-01	0.46	4.49E-01	0.20	2.49E-01	0.39
1004.5102@12.069868	1004.5102	12.07	NEG	RP				1.41E-02	1.73E-02	0.62	9.27E-01	-0.02	1.19E-01	-0.36	6.85E-02	0.49
1004.5114@3.0467677	1004.5114	3.05	POS	hilic				9.81E-01	5.41E-01	0.17	5.95E-01	0.15	8.42E-01	0.05	8.77E-01	0.04
1004.514@12.12953	1004.5140	12.13	NEG	RP				1.93E-01	1.67E-02	0.57	9.90E-02	0.34	6.69E-01	0.07	4.25E-02	0.47
1004.5829@1.8387475	1004.5829	1.84	POS	hilic				1.53E-01	8.17E-02	0.50	2.85E-01	-0.31	3.16E-01	-0.32	3.98E-01	0.23
1004.6761@10.1555	1004.6761	10.16	POS	RP				9.71E-02	6.98E-03	0.75	5.69E-01	-0.16	5.04E-01	-0.16	4.99E-01	0.20
1004.6796@10.498792	1004.6796	10.50	NEG	RP				1.12E-03	1.72E-02	0.72	1.97E-02	-0.66	6.43E-02	-0.46	3.34E-01	0.25
1004.6814@1.2050272	1004.6814	1.21	POS	hilic				3.97E-01	7.99E-02	0.55	6.02E-02	0.57	5.32E-02	0.53	4.31E-01	0.21
1004.6871@1.2766283	1004.6871	1.28	POS	hilic				1.19E-02	9.09E-01	0.03	1.60E-03	-0.87	1.57E-03	-0.82	3.78E-02	-0.67
1004.6913@10.497651	1004.6913	10.50	POS	RP				2.97E-01	8.71E-02	0.45	2.20E-01	-0.34	6.51E-01	-0.11	6.13E-01	-0.15
1005.0702@3.1599739	1005.0702	3.16	POS	hilic				7.53E-01	4.66E-01	0.27	7.31E-01	0.08	8.74E-01	0.04	1.02E-01	0.44
1005.0918@3.4810462	1005.0918	3.48	NEG	hilic				3.59E-01	2.35E-02	0.65	7.60E-01	0.07	2.84E-01	0.29	3.25E-01	0.25
1005.5091@12.050974	1005.5091	12.05	NEG	RP				7.44E-01	3.57E-01	0.27	4.85E-01	-0.17	5.95E-01	-0.12	7.50E-01	0.09
1005.6522@10.8771715	1005.6522	10.88	POS	RP				8.22E-01	3.47E-01	-0.26	2.44E-01	-0.31	9.70E-01	0.01	6.24E-01	-0.14
1005.6566@0.40407142	1005.6566	0.40	POS	hilic				8.21E-01	5.52E-01	-0.16	1.58E-01	-0.38	5.25E-01	-0.18	4.27E-01	-0.22
1005.6927@10.498459	1005.6927	10.50	POS	RP				1.74E-01	3.39E-01	0.31	5.69E-02	-0.41	1.28E-01	-0.34	6.40E-01	0.12
1005.6938@1.278522	1005.6938	1.28	POS	hilic				1.25E-01	1.70E-02	0.83	7.40E-01	-0.09	5.89E-01	0.13	2.75E-01	0.33
1005.7384@11.035667	1005.7384	11.04	NEG	RP				1.41E-01	9.70E-01	0.01	8.85E-01	-0.04	8.16E-03	0.67	2.76E-01	-0.29
1005.7606@0.52478373	1005.7606	0.52	POS	hilic				7.72E-01	6.78E-01	-0.11	1.07E-01	-0.33	9.06E-01	-0.03	6.70E-01	-0.10
1006.5293@12.440143	1006.5293	12.44	NEG	RP				2.58E-01	2.47E-02	0.72	8.52E-01	-0.05	1.43E-01	0.35	4.93E-01	0.21
1006.6565@10.8773575	1006.6565	10.88	POS	RP				7.44E-01	4.48E-01	-0.20	4.37E-01	-0.20	4.51E-01	0.19	8.98E-01	-0.03
1006.6751@1.2193366	1006.6751	1.22	POS	hilic				2.68E-02	1.79E-02	0.77	2.37E-01	-0.31	2.67E-01	-0.28	5.59E-01	0.17
1006.7416@11.035478	1006.7416	11.04	NEG	RP				1.81E-01	5.28E-01	-0.20	6.82E-01	-0.12	9.07E-02	0.44	6.82E-02	-0.50
1007.0677@3.160552	1007.0677	3.16	POS	hilic				1.34E-01	2.63E-02	0.63	3.84E-02	0.48	5.72E-01	0.14	1.23E-02	0.74
1007.0919@3.4795415	1007.0919	3.48	NEG	hilic				4.90E-01	1.30E-01	0.45	5.98E-01	-0.12	4.35E-01	0.21	2.80E-01	0.27
1008.4974@12.061051	1008.4974	12.06	NEG	RP				3.87E-01	2.49E-01	-0.27	5.08E-01	0.17	8.64E-01	-0.04	6.58E-02	-0.38
1008.5435@12.941499	1008.5435	12.94	NEG	RP				2.78E-01	8.52E-02	0.44	5.30E-01	-0.15	4.80E-01	-0.17	3.20E-01	0.24
1008.5966@10.668558	1008.5966	10.67	NEG	RP				4.61E-01	4.61E-01	0.20	1.06E-01	0.42	1.65E-01	0.40	6.50E-01	-0.13
1008.5979@10.632651	1008.5979	10.63	NEG	RP				1.01E-01	1.67E-01	-0.42	1.62E-01	0.41	1.04E-01	0.48	1.26E-01	0.41
1008.6782@10.42141	1008.6782	10.42	POS	RP				9.35E-01	7.26E-01	0.10	5.93E-01	-0.15	9.49E-01	0.02	6.71E-01	0.13
1008.7189@10.51257	1008.7189	10.51	POS	RP				4.06E-01	9.11E-02	-0.40	2.89E-01	-0.26	1.47E-01	-0.35	2.14E-02	-0.51
1009.0179@1.8603511	1009.0179	1.86	NEG	hilic				1.30E-02	6.62E-01	-0.12	8.11E-02	0.52	2.93E-03	0.84	6.52E-01	-0.11
1009.0853@3.4853802	1009.0853	3.49	NEG	hilic				5.85E-01	1.76E-01	0.40	3.99E-01	-0.19	6.84E-01	0.12	9.70E-01	0.01
1010.1861@0.3954242	1010.1861	0.40	NEG	hilic				7.76E-01	1.68E-01	-0.36	2.34E-01	-0.35	8.99E-01	-0.03	6.50E-01	-0.15
1010.61@10.875309	1010.6100	10.88	POS	RP				7.68E-01	3.98E-01	-0.21	4.71E-01	-0.18	5.13E-01	0.17	9.06E-01	-0.03
1010.6309@1.3130876	1010.6309	1.31	POS	hilic				1.06E-02	3.19E-02	0.71	5.82E-02	-0.50	2.30E-01	-0.33	4.79E-01	0.21
1010.6343@1.1585146	1010.6343	1.16	POS	hilic				2.91E-01	1.06E-01	0.54	3.93E-01	-0.19	3.42E-01	0.27	2.12E-01	0.37
1011.0342@1.4130601	1011.0342	1.41	NEG	hilic				2.04E-02	5.66E-02	0.49	8.46E-02	0.47	1.83E-04	1.18	1.14E-01	0.49
1011.4896@0.50128084	1011.4896	0.50	POS	hilic				8.73E-01	5.83E-01	-0.15	7.77E-01	0.09	6.14E-01	0.14	3.84E-01	0.26
1011.6118@10.875172	1011.6118	10.88	POS	RP				7.41E-01	4.51E-01	-0.17	5.96E-01	-0.12	4.02E-01	0.21	9.09E-01	0.03
1012.1916@0.39617229	1012.1916	0.40	NEG	hilic				9.42E-01	4.97E-01	0.18	4.37E-01	0.24	2.74E-01	0.28	7.26E-01	0.13
1012.1951@9.521615	1012.1951	9.52	NEG	RP				7.42E-01	2.20E-01	0.40	4.50E-01	0.20	7.12E-01	0.11	7.07E-01	-0.12
1012.5588@3.2109091	1012.5588	3.21	NEG	hilic				5.22E-01	6.79E-02	0.65	3.47E-01	0.26	5.87E-01	0.15	6.80E-01	0.10
1012.646@10.116054	1012.6460	10.12	POS	RP				6.94E-03	3.50E-02	0.64	1.55E-01	-0.43	2.11E-02	-0.61	5.17E-01	0.19
1012.6469@9.989724	1012.6469	9.99	POS	RP				1.82E-03	1.09E-01	0.45	3.76E-02	-0.62	1.46E-02	-0.65	1.87E-01	0.40
1012.6491@9.845209	1012.6491	9.85	POS	RP				7.86E-01	9.93E-01	0.00	5.08E-01	-0.17	2.96E-01	-0.25	6.11E-01	0.16
1012.6507@1.2844181	1012.6507	1.28	POS	hilic				1.34E-02	4.69E-01	0.20	1.86E-02	-0.70	3.22E-03	-0.77	8.25E-01	0.07
1012.8593@11.069622	1012.8593	11.07	POS	RP				4.34E-01	1.97E-01	-0.38	4.60E-01	0.21	4.56E-01	0.21	5.06E-01	-0.19
1013.1502@1.3198221	1013.1502	1.32	POS	hilic				3.79E-01	1.61E-01	-0.34	4.25E-01	0.21	2.30E-01	-0.30	4.75E-01	-0.17
1013.2041@0.39571017	1013.2041	0.40	NEG	hilic				9.84E-01	5.45E-01	0.18	7.49E-01	0.10	6.06E-01	0.14	9.35E-01	0.02
1013.5022@0.5040573	1013.5022	0.50	POS	hilic				7.76E-01	2.50E-01	-0.31	6.71E-01	0.12	5.25E-01	-0.19	8.04E-01	0.08
1013.6707@10.782924	1013.6707	10.78	NEG	RP				5.07E-01	8.43E-01	-0.05	1.12E-01	0.41	2.07E-01	0.32	9.88E-01	0.00
1014.2123@0.3947378	1014.2123	0.39	NEG	hilic				9.80E-01	8.38E-01	0.06	9.00E-01	-0.04	8.17E-01	-0.06	6.09E-01	0.15
1014.5295@11.908981	1014.5295	11.91	NEG	RP				3.48E-01	5.47E-02	-0.43	2.79E-02	-0.48	7.75E-01	-0.08	9.75E-01	0.01
1014.5299@12.062155	1014.5299	12.06	NEG	RP				4.14E-01	8.73E-01	-0.05	3.43E-01	0.29	3.21E-01	0.26	2.26E-01	-0.37
1014.6651@9.989298	1014.6651	9.99	POS	RP				2.12E-03	1.21E-01	0.44	3.57E-02	-0.62	1.65E-02	-0.64	1.89E-01	0.40
1014.6665@1.1875747	1014.6665	1.19	POS	hilic				6.29E-02	6.62E-02	0.60	7.98E-02	-0.46	5.00E-01	-0.16	4.79E-01	0.23

Supplementary table 2: Metabolomics analysis results

Compound	Mass	Retention Time	Ionization mode	column	Compound_ID	MSMS_spectra	ID_level	ANOVA P-FDR	Anorexia vs control P	Anorexia vs control Estimate	overweight vs control P	overweight vs control Estimate	obese vs control P	obese vs control Estimate	athletes vs control P	athletes vs control Estimate
1014.6665@1.2829992	1014.6665	1.28	POS	hilic				1.59E-02	5.55E-01	0.16	1.89E-02	-0.70	3.27E-03	-0.78	8.53E-01	0.06
1014.6667@10.116106	1014.6667	10.12	POS	RP				5.89E-03	2.83E-02	0.68	1.40E-01	-0.44	3.55E-02	-0.57	5.35E-01	0.18
1015.5361@12.059949	1015.5361	12.06	NEG	RP				2.89E-01	7.13E-02	-0.59	2.92E-02	-0.56	2.06E-01	-0.33	3.38E-02	-0.67
1015.6695@1.1510632	1015.6695	1.15	POS	hilic				2.93E-02	1.20E-01	0.48	8.93E-02	-0.43	1.07E-01	-0.44	2.53E-01	0.36
1015.6702@9.988853	1015.6702	9.99	POS	RP				1.02E-03	1.06E-01	0.45	1.70E-02	-0.70	1.48E-02	-0.65	1.99E-01	0.38
1016.6796@10.1224575	1016.6796	10.12	POS	RP				2.48E-02	2.76E-02	0.69	1.62E-01	-0.43	3.20E-01	-0.27	3.24E-01	0.27
1016.6801@1.1898837	1016.6801	1.19	POS	hilic				2.37E-03	2.85E-02	0.68	4.37E-02	-0.48	4.42E-02	-0.51	1.87E-01	0.37
1016.6817@10.229077	1016.6817	10.23	POS	RP				9.75E-02	3.52E-02	0.66	5.68E-01	-0.18	5.98E-01	-0.13	5.15E-02	0.49
1016.6821@10.135701	1016.6821	10.14	POS	RP				1.34E-01	1.54E-01	0.44	4.38E-01	-0.23	7.65E-02	-0.54	7.38E-01	0.10
1016.7171@10.510099	1016.7171	10.51	POS	RP				4.30E-01	4.71E-01	0.23	1.20E-01	-0.41	5.59E-01	-0.16	6.49E-01	0.13
1017.1216@1.3114657	1017.1216	1.31	POS	hilic				4.47E-01	3.12E-01	0.25	8.61E-02	0.41	5.21E-02	0.54	5.41E-01	0.13
1017.1236@10.10276	1017.1236	10.10	POS	RP				2.13E-02	1.36E-02	0.75	3.01E-01	0.31	1.49E-01	0.43	1.02E-04	1.10
1018.1256@10.104282	1018.1256	10.10	POS	RP				2.32E-02	7.50E-03	0.79	3.25E-01	0.29	1.48E-01	0.43	1.22E-04	1.06
1018.1282@1.3122023	1018.1282	1.31	POS	hilic				1.71E-01	2.29E-01	0.36	4.77E-01	0.17	2.39E-03	0.81	9.97E-02	0.47
1018.2203@9.475482	1018.2203	9.48	NEG	RP				3.78E-01	2.83E-01	0.24	1.33E-01	0.32	2.39E-02	0.59	7.67E-02	0.45
1018.2258@0.39627016	1018.2258	0.40	NEG	hilic				5.87E-01	5.73E-01	0.14	1.89E-01	0.38	2.60E-01	0.33	7.37E-02	0.57
1018.5628@12.801513	1018.5628	12.80	NEG	RP				3.02E-04	1.51E-01	-0.30	4.53E-01	0.20	4.00E-04	0.89	3.95E-02	-0.41
1018.5992@9.961244	1018.5992	9.96	POS	RP				2.03E-01	1.62E-01	0.42	4.96E-01	-0.19	1.31E-01	-0.45	4.67E-01	0.21
1018.6924@1.2580856	1018.6924	1.26	POS	hilic				2.07E-02	2.72E-01	0.31	3.17E-02	-0.64	6.68E-03	-0.70	9.88E-01	0.01
1018.6945@1.1901203	1018.6945	1.19	POS	hilic				1.50E-03	1.39E-03	0.94	6.22E-02	-0.47	6.08E-01	-0.12	1.49E-01	0.45
1018.6946@1.1880008	1018.6946	1.19	POS	hilic				1.53E-02	1.33E-03	1.00	4.64E-01	-0.19	3.67E-01	0.24	1.05E-01	0.44
1018.6974@10.512479	1018.6974	10.51	POS	RP				7.12E-01	7.67E-01	-0.09	3.00E-01	-0.21	3.14E-01	0.24	8.24E-01	0.06
1018.9708@7.7310014	1018.9708	7.73	NEG	hilic				1.28E-01	7.07E-03	0.86	3.76E-02	0.54	1.07E-01	0.37	3.03E-02	0.55
1019.5001@12.705265	1019.5001	12.71	NEG	RP				5.53E-01	8.84E-01	0.05	1.60E-01	-0.46	1.53E-01	-0.41	5.74E-01	-0.15
1019.6307@10.108895	1019.6307	10.11	POS	RP				7.02E-01	5.57E-01	-0.13	5.01E-01	0.14	1.83E-01	0.31	4.86E-01	0.20
1019.7032@1.2264177	1019.7032	1.23	POS	hilic				1.62E-02	2.19E-01	0.40	5.09E-02	-0.54	6.09E-03	-0.71	8.24E-01	0.07
1019.7181@11.036086	1019.7181	11.04	NEG	RP				4.39E-01	7.30E-02	0.60	3.18E-01	0.28	2.56E-02	0.61	3.31E-01	0.29
1020.2077@9.449587	1020.2077	9.45	NEG	RP				9.71E-01	5.61E-01	-0.13	9.04E-01	0.03	9.36E-01	-0.02	7.67E-01	0.08
1020.2084@0.3957435	1020.2084	0.40	NEG	hilic				4.88E-01	4.92E-01	0.18	4.72E-02	0.61	6.35E-01	0.13	2.43E-01	0.39
1020.6179@9.968328	1020.6179	9.97	NEG	RP				8.60E-01	3.86E-01	0.29	7.29E-01	0.10	9.50E-01	0.02	2.23E-01	0.33
1020.618@9.934441	1020.6180	9.93	NEG	RP				5.45E-01	7.54E-01	0.10	3.18E-01	0.25	3.46E-01	-0.31	3.56E-01	-0.27
1020.6186@9.959755	1020.6186	9.96	POS	RP				4.49E-01	4.10E-01	0.25	3.34E-01	-0.28	4.40E-01	-0.25	2.76E-01	0.29
1020.9639@15.301379	1020.9639	15.30	NEG	RP				9.71E-01	4.77E-01	-0.21	5.91E-01	-0.15	9.32E-01	-0.02	8.40E-01	-0.06
1021.6214@9.9622135	1021.6214	9.96	POS	RP				1.14E-01	2.30E-02	0.68	4.61E-01	-0.21	9.87E-01	0.01	7.57E-02	0.47
1022.223@9.522341	1022.2230	9.52	NEG	RP				9.52E-01	7.82E-01	-0.08	9.50E-01	0.02	5.99E-01	0.16	6.73E-01	-0.13
1022.2249@0.39562154	1022.2249	0.40	NEG	hilic				7.31E-01	6.21E-01	0.13	1.85E-01	0.41	8.40E-01	0.06	2.39E-01	0.37
1022.6328@10.117664	1022.6328	10.12	NEG	RP				2.40E-01	6.43E-02	0.55	6.23E-01	-0.09	9.63E-01	0.01	4.47E-01	0.17
1022.6335@10.19978	1022.6335	10.20	POS	RP				1.08E-01	2.51E-03	0.85	8.17E-01	0.07	9.23E-01	0.03	6.33E-02	0.48
1023.2274@0.39566657	1023.2274	0.40	NEG	hilic				4.05E-01	6.30E-01	0.13	2.45E-02	0.68	5.57E-01	0.16	4.48E-01	0.26
1023.4917@0.50732976	1023.4917	0.51	POS	hilic				8.76E-01	8.44E-01	0.06	7.42E-01	0.09	4.23E-01	-0.20	5.16E-01	0.17
1024.5518@1.8173333	1024.5518	1.82	POS	hilic				4.67E-01	8.42E-01	-0.06	2.62E-01	-0.31	1.05E-01	-0.53	7.13E-01	0.10
1024.5883@9.787274	1024.5883	9.79	POS	RP				8.02E-01	5.00E-01	0.21	9.69E-01	0.01	2.13E-01	0.32	8.51E-01	-0.05
1025.5002@0.50726986	1025.5002	0.51	POS	hilic				7.78E-01	3.03E-01	0.29	7.37E-01	0.08	4.91E-01	-0.15	9.63E-01	0.01
1025.6215@10.619709	1025.6215	10.62	POS	RP				7.29E-01	6.81E-01	-0.11	5.76E-01	-0.16	6.80E-01	0.14	2.23E-01	-0.39
1026.1981@9.4947	1026.1981	9.49	NEG	RP				7.50E-01	4.52E-01	0.26	1.48E-01	0.40	7.45E-01	0.10	8.86E-01	-0.04
1026.1993@0.39601696	1026.1993	0.40	NEG	hilic				8.37E-01	4.69E-01	0.20	4.18E-01	-0.24	5.80E-01	-0.14	7.15E-01	-0.12
1026.5688@1.8119017	1026.5688	1.81	POS	hilic				5.96E-01	7.90E-01	-0.08	3.63E-01	-0.25	1.65E-01	-0.47	6.95E-01	0.10
1026.6072@10.710503	1026.6072	10.71	NEG	RP				9.50E-01	8.99E-01	0.04	8.34E-01	0.05	3.80E-01	0.27	7.49E-01	0.08
1026.6403@10.009311	1026.6403	10.01	NEG	RP				1.17E-01	1.33E-01	0.54	2.02E-01	-0.33	2.37E-01	0.37	1.26E-01	0.42
1026.6407@1.252574	1026.6407	1.25	NEG	hilic				1.02E-02	3.12E-03	0.88	4.26E-01	-0.19	3.76E-01	0.26	9.17E-03	0.72
1026.6407@1.4287182	1026.6407	1.43	NEG	hilic				1.10E-01	6.97E-01	0.13	3.97E-02	0.57	1.89E-03	0.83	7.81E-01	0.08
1026.6414@10.114035	1026.6414	10.11	NEG	RP				3.52E-01	6.32E-01	0.18	5.85E-01	0.15	2.03E-01	0.37	1.16E-02	0.73
1026.6855@1.2561983	1026.6855	1.26	POS	hilic				2.39E-02	2.78E-02	0.64	8.88E-02	-0.50	1.17E-01	-0.36	9.50E-01	-0.02
1027.643@1.427721	1027.6430	1.43	NEG	hilic				4.63E-01	7.57E-01	0.10	9.91E-02	0.51	4.34E-02	0.54	6.38E-01	0.12
1028.5146@12.064184	1028.5146	12.06	NEG	RP				9.20E-01	5.89E-01	0.20	2.75E-01	0.30	3.80E-01	0.26	4.50E-01	0.19
1028.612@10.052205	1028.6120	10.05	POS	RP				7.11E-01	5.22E-01	-0.20	4.22E-01	-0.23	2.80E-01	-0.35	8.67E-02	-0.52
1028.6154@10.127375	1028.6154	10.13	POS	RP				4.16E-01	2.51E-01	0.35	6.86E-01	0.12	2.15E-01	-0.36	3.40E-01	0.27
1028.6198@1.3388658	1028.6198	1.34	POS	hilic				9.23E-01	5.06E-01	-0.22	9.37E-01	0.02	8.21E-01	0.07	5.28E-01	-0.17
1028.6812@1.2435714	1028.6812	1.24	POS	hilic				1.80E-02	7.85E-04	1.00	8.60E-01	-0.05	6.06E-01	-0.13	8.06E-01	0.08
1028.6838@1.1259998	1028.6838	1.13	POS	hilic				2.77E-01	1.98E-02	0.62	6.82E-01	0.11	6.51E-01	-0.11	5.67E-01	0.15
1029.1191@1.3031839	1029.1191	1.30	POS	hilic				6.53E-01	2.82E-01	0.39	9.89E-01	0.00	3.31E-01	-0.23	9.78E-01	0.01
1029.122@10.000687	1029.1220	10.00	POS	RP				1.28E-02	2.99E-02	0.72	5.84E-02	-0.42	9.25E-01	-0.02	6.65E-02	0.54
1029.5175@12.070143	1029.5175	12.07	NEG	RP				3.92E-02	5.88E-01	0.15	3.76E-02	-0.39	2.99E-02	-0.40	2.07E-01	0.33
1029.6259@10.000384	1029.6259	10.00	POS	RP				5.05E-04	1.78E-03	0.94	7.01E-02	-0.43	7.61E-01	-0.08	9.13E-03	0.70
1029.6436@10.116132	1029.6436	10.12	NEG	RP				7.57E-01	4.05E-01	-0.27	9.84E-01	-0.01	9.08E-01	-0.03	3.63E-01	0.28
1030.1425@9.494975	1030.1425	9.49	NEG	RP				2.85E-01	1.75E-02	-0.75	5.81E-01	-0.16	5.03E-01	-0.15	1.05E-01	-0.50
1030.2347@0.40508002	1030.2347	0.41	NEG	hilic				4.68E-01	8.99E-02	0.53	2.10E-01	0.36	2.44E-01	0.32	4.54E-02	0.61
1030.6309@10.130266	1030.6309	10.13	POS	RP				3.24E-02	2.75E-02	0.68	8.19E-01	-0.07	6.82E-02	-0.47	2.19E-01	0.35
1030.6938@10.25331	1030.6938	10.25	POS	RP				1.10E-02	2.73E-02	0.66	9.30E-02	-0.52	7.57E-02	-0.45	6.76E-01	0.11
1030.6956@1.2318876	1030.6956	1.23	POS	hilic				2.65E-03	2.32E-03	0.90	9.20E-02	-0.48	1.54E-01	-0.31	7.92E-01	0.07
1030.6992@1.1882558	1030.6992	1.19	POS	hilic				1.55E-01	1.07E-01	0.44	2.28E-02	0.64	8.93E-01	-0.03	4.52E-02	0.53
1030.731@10.5059595	1030.7310	10.51	POS	RP				2.65E-02	1.36E-01	0.49	1.55E-02	-0.67	1.83E-01	-0.30	8.52E-01	-0.05
1031.7635@4.4738207	1031.7635	4.47	POS	hilic				9.52E-02	3.86E-03	0.85	2.27E-01	0.31	2.65E-01	0.31	6.52E-01	-0.15

Supplementary table 2: Metabolomics analysis results

								ANOVA	Anorexia vs control		overweight vs control		obese vs control		athletes vs control	
Compound	Mass	Retention Time	Ionization mode	column	Compound_ID	MSMS_spectra	ID_level	P-FDR	P	Estimate	P	Estimate	P	Estimate	P	Estimate
1031.8228@4.563793	1031.8228	4.56	NEG	hilic				8.05E-01	2.51E-01	0.33	6.61E-01	0.14	6.50E-01	-0.13	5.40E-01	0.18
1032.4023@0.5566819	1032.4023	0.56	NEG	hilic				6.09E-01	5.88E-02	-0.42	3.44E-01	-0.23	9.20E-02	-0.39	4.76E-01	-0.19
1032.5449@12.806759	1032.5449	12.81	NEG	RP				6.82E-01	8.80E-01	0.04	1.65E-01	0.45	2.95E-01	0.32	2.11E-01	0.31
1032.638@9.781792	1032.6380	9.78	NEG	RP				5.45E-01	2.62E-01	0.36	6.96E-01	-0.11	1.39E-01	0.42	4.22E-01	0.22
1032.7137@1.2290725	1032.7137	1.23	POS	hilic				1.29E-02	8.57E-03	0.87	2.30E-01	-0.31	2.51E-01	-0.26	7.45E-01	0.09
1032.9847@15.245624	1032.9847	15.25	NEG	RP				8.41E-01	1.70E-01	-0.40	7.13E-01	-0.11	3.16E-01	-0.26	3.95E-01	-0.26
1034.6342@1.1521823	1034.6342	1.15	POS	hilic				2.36E-01	3.77E-01	-0.25	1.51E-02	-0.76	1.52E-01	-0.43	7.09E-01	-0.11
1034.6385@9.963937	1034.6385	9.96	POS	RP				5.25E-01	6.79E-02	0.49	2.12E-01	0.27	1.18E-01	0.43	6.00E-01	0.13
1034.654@1.411256	1034.6540	1.41	NEG	hilic				1.16E-02	3.19E-03	0.91	2.67E-01	-0.26	4.44E-01	0.23	3.25E-02	0.60
1035.2029@0.3922087	1035.2029	0.39	NEG	hilic				9.33E-01	3.55E-01	-0.27	3.70E-01	-0.27	6.54E-01	-0.11	6.45E-01	-0.15
1036.0142@6.1822643	1036.0142	6.18	POS	hilic				1.96E-01	3.28E-02	0.64	6.64E-01	0.13	4.69E-01	0.21	4.11E-03	0.74
1036.595@10.713979	1036.5950	10.71	NEG	RP				7.77E-01	5.03E-01	0.15	2.58E-01	0.28	3.86E-01	0.21	1.59E-01	0.37
1036.649@1.2318938	1036.6490	1.23	POS	hilic				9.39E-01	8.92E-01	-0.04	4.38E-01	0.22	9.61E-01	0.01	6.45E-01	0.15
1036.6501@9.963266	1036.6501	9.96	POS	RP				3.65E-01	5.22E-01	0.15	2.62E-01	0.28	7.80E-01	-0.06	4.03E-02	0.56
1036.6519@10.780613	1036.6519	10.78	NEG	RP				5.05E-01	2.47E-01	-0.24	3.28E-01	0.28	2.77E-01	0.27	9.99E-01	0.00
1036.669@1.2515	1036.6690	1.25	NEG	hilic				2.28E-03	2.38E-03	0.89	6.23E-02	-0.44	7.67E-01	-0.08	6.27E-02	0.54
1036.6696@1.4118716	1036.6696	1.41	NEG	hilic				1.12E-02	2.74E-03	0.92	3.13E-01	-0.24	3.97E-01	0.26	2.53E-02	0.62
1036.6711@10.064984	1036.6711	10.06	NEG	RP				2.73E-01	2.60E-01	0.35	7.50E-02	-0.46	4.38E-01	0.20	7.64E-01	0.10
1036.6711@10.110525	1036.6711	10.11	NEG	RP				2.22E-03	8.17E-03	0.78	9.87E-02	-0.43	6.94E-01	-0.11	1.04E-02	0.69
1037.0201@6.1821284	1037.0201	6.18	POS	hilic				1.84E-01	8.74E-03	0.76	1.14E-01	0.52	2.05E-01	0.36	6.97E-03	0.76
1038.2181@0.39611703	1038.2181	0.40	NEG	hilic				9.59E-01	6.62E-01	0.13	5.19E-01	0.20	3.52E-01	0.26	6.56E-01	0.13
1038.6646@1.1458132	1038.6646	1.15	POS	hilic				8.34E-01	4.51E-01	-0.22	2.03E-01	-0.33	3.93E-01	-0.23	2.97E-01	-0.32
1038.6656@1.1882104	1038.6656	1.19	POS	hilic				2.44E-01	3.79E-01	-0.29	1.26E-01	-0.45	4.83E-01	-0.19	2.55E-01	0.37
1038.6663@9.96083	1038.6663	9.96	POS	RP				9.45E-01	4.60E-01	0.21	5.97E-01	0.13	9.60E-01	-0.02	8.39E-01	-0.06
1039.211@9.523297	1039.2110	9.52	NEG	RP				9.57E-01	8.33E-01	-0.07	4.94E-01	0.20	9.83E-01	-0.01	7.30E-01	0.11
1039.2133@9.687559	1039.2133	9.69	NEG	RP				5.19E-01	2.31E-01	0.32	2.47E-01	0.27	2.20E-02	0.65	3.99E-01	0.28
1039.2166@0.39588168	1039.2166	0.40	NEG	hilic				9.94E-01	8.97E-01	0.04	7.90E-01	0.08	9.68E-01	0.01	7.83E-01	-0.08
1039.4716@11.864045	1039.4716	11.86	NEG	RP				1.31E-03	2.74E-03	0.97	9.96E-01	0.00	2.33E-03	0.77	8.00E-01	0.05
1039.6678@1.2519221	1039.6678	1.25	POS	hilic				1.73E-01	1.44E-01	-0.51	2.18E-01	-0.35	1.43E-02	-0.68	6.96E-01	0.12
1039.6707@9.933469	1039.6707	9.93	POS	RP				1.89E-01	3.61E-01	0.27	2.33E-01	-0.34	8.50E-02	-0.49	5.06E-01	0.24
1040.6812@1.2681489	1040.6812	1.27	POS	hilic				7.09E-01	8.59E-01	0.05	4.10E-01	0.21	5.28E-01	-0.21	2.81E-01	-0.28
1040.6812@10.139889	1040.6812	10.14	POS	RP				6.43E-01	4.71E-01	0.25	2.72E-01	-0.34	9.72E-01	-0.01	6.97E-01	0.11
1041.1202@9.994466	1041.1202	9.99	POS	RP				4.77E-01	1.73E-01	0.48	3.95E-01	-0.18	8.84E-01	-0.03	6.72E-01	0.13
1041.4781@12.027814	1041.4781	12.03	NEG	RP				2.81E-01	1.35E-01	0.43	1.60E-01	-0.30	8.20E-01	0.06	3.92E-01	0.26
1041.5754@1.3283695	1041.5754	1.33	POS	hilic				1.86E-01	5.06E-01	-0.14	4.71E-01	0.14	4.36E-02	0.56	1.26E-01	0.34
1041.5854@10.144513	1041.5854	10.14	POS	RP				5.47E-02	5.33E-01	-0.17	9.53E-03	0.74	3.71E-02	0.47	5.49E-01	0.16
1041.6833@10.139776	1041.6833	10.14	POS	RP				5.93E-01	3.53E-01	0.32	2.96E-01	-0.33	9.44E-01	0.02	7.99E-01	0.07
1041.7946@4.409614	1041.7946	4.41	POS	hilic				1.61E-01	1.60E-02	0.67	2.96E-01	0.25	7.72E-01	-0.09	4.11E-01	-0.24
1041.8829@11.069735	1041.8829	11.07	POS	RP				7.55E-01	1.64E-01	-0.28	7.59E-01	-0.07	8.89E-01	0.03	3.07E-01	-0.21
1042.1252@9.994037	1042.1252	9.99	POS	RP				1.02E-03	6.62E-03	0.78	3.02E-01	-0.26	1.69E-01	-0.34	1.16E-02	0.76
1042.1276@10.14602	1042.1276	10.15	POS	RP				8.34E-01	6.58E-01	0.16	7.75E-01	-0.08	2.83E-01	0.26	3.12E-01	0.28
1042.158@9.494873	1042.1580	9.49	NEG	RP				7.93E-01	2.14E-01	-0.41	6.80E-01	-0.13	4.73E-01	-0.19	2.19E-01	-0.37
1042.6952@1.1033647	1042.6952	1.10	POS	hilic				6.90E-02	3.96E-01	0.29	1.09E-01	-0.48	1.25E-02	-0.70	7.64E-01	-0.08
1042.6969@1.2198957	1042.6969	1.22	POS	hilic				5.69E-02	1.94E-01	0.43	1.05E-01	-0.46	3.09E-02	-0.56	8.32E-01	0.06
1042.6978@11.0390835	1042.6978	11.04	NEG	RP				4.44E-01	9.75E-01	0.01	4.27E-01	0.24	4.06E-02	0.57	7.76E-01	-0.08
1042.6982@10.232539	1042.6982	10.23	POS	RP				9.50E-01	8.23E-01	-0.06	9.23E-01	0.03	8.22E-01	-0.06	4.88E-01	0.21
1043.1324@10.144081	1043.1324	10.14	POS	RP				3.25E-01	3.56E-01	0.28	1.56E-01	-0.37	4.35E-01	-0.21	3.80E-01	0.22
1043.5013@12.463059	1043.5013	12.46	NEG	RP				6.85E-01	1.17E-01	0.40	5.86E-01	-0.15	4.81E-01	0.21	8.02E-01	0.08
1043.5743@1.3301138	1043.5743	1.33	POS	hilic				5.76E-01	8.74E-01	-0.05	7.20E-02	0.49	3.05E-01	0.30	2.49E-01	0.29
1043.58@10.138063	1043.5800	10.14	POS	RP				1.68E-01	7.21E-02	0.54	2.90E-02	0.72	1.03E-01	0.47	3.93E-03	0.80
1043.5802@10.117781	1043.5802	10.12	POS	RP				9.72E-01	8.59E-01	0.05	8.35E-01	-0.06	5.26E-01	0.19	8.30E-01	0.07
1043.5831@10.131032	1043.5831	10.13	POS	RP				1.83E-01	3.31E-01	0.30	3.54E-03	0.84	2.86E-01	0.30	4.79E-01	0.20
1043.701@10.232555	1043.7010	10.23	POS	RP				6.37E-03	6.25E-02	0.56	4.56E-02	-0.62	2.03E-02	-0.61	9.03E-01	0.04
1044.1569@9.49495	1044.1569	9.49	NEG	RP				7.59E-01	3.23E-01	-0.31	5.67E-01	-0.17	7.49E-01	-0.08	1.32E-01	-0.46
1044.1569@9.647301	1044.1569	9.65	NEG	RP				5.34E-01	6.75E-02	-0.56	3.53E-01	-0.31	2.97E-01	-0.25	6.14E-02	-0.55
1044.5818@1.3238047	1044.5818	1.32	POS	hilic				6.79E-01	3.13E-01	0.28	8.78E-01	-0.04	1.46E-01	0.40	5.93E-01	0.13
1044.585@10.014268	1044.5850	10.01	POS	RP				4.37E-02	7.34E-04	0.91	1.98E-01	0.36	6.14E-02	0.57	1.61E-03	0.90
1044.586@10.105012	1044.5860	10.11	POS	RP				9.35E-01	7.29E-01	0.10	3.81E-01	0.22	4.48E-01	0.18	3.51E-01	0.25
1044.6178@9.952862	1044.6178	9.95	POS	RP				7.29E-01	1.11E-01	-0.51	2.33E-01	-0.31	4.94E-01	-0.22	2.32E-01	-0.32
1044.618@9.958349	1044.6180	9.96	NEG	RP				4.85E-01	1.40E-01	-0.47	5.46E-01	0.17	5.39E-01	0.21	9.78E-01	-0.01
1044.7076@1.1392646	1044.7076	1.14	POS	hilic				4.54E-01	4.82E-02	0.48	7.91E-01	0.08	5.64E-01	-0.16	2.78E-01	0.38
1044.7083@11.03789	1044.7083	11.04	NEG	RP				4.22E-01	9.64E-01	-0.01	9.81E-01	-0.01	4.32E-02	0.54	5.86E-01	-0.15
1044.7085@10.529784	1044.7085	10.53	POS	RP				9.87E-01	7.97E-01	0.08	7.92E-01	0.06	9.65E-01	-0.01	7.39E-01	-0.08
1044.7115@1.1758779	1044.7115	1.18	POS	hilic				1.07E-01	8.40E-01	0.07	9.20E-01	-0.03	1.60E-02	-0.69	2.87E-01	0.31
1045.1595@9.494326	1045.1595	9.49	NEG	RP				7.38E-01	3.88E-01	-0.27	8.20E-01	-0.07	5.79E-01	-0.13	1.16E-01	-0.47
1045.4897@3.0276787	1045.4897	3.03	POS	hilic				7.77E-01	5.28E-01	0.20	6.61E-01	0.12	4.79E-01	-0.21	4.63E-01	-0.20
1045.4897@3.0278072	1045.4897	3.03	POS	hilic				9.17E-01	4.27E-01	0.26	5.60E-01	0.17	8.92E-01	-0.04	9.61E-01	-0.01
1045.6241@9.954066	1045.6241	9.95	POS	RP				7.22E-01	3.30E-01	-0.27	4.12E-01	-0.21	3.58E-01	-0.28	5.97E-01	0.16
1045.7094@11.0375395	1045.7094	11.04	NEG	RP				3.49E-01	9.68E-02	0.54	3.36E-02	0.63	1.16E-02	0.61	2.57E-01	0.34
1045.7144@1.1137297	1045.7144	1.11	POS	hilic				5.61E-01	7.76E-01	0.08	6.48E-01	-0.14	7.53E-02	-0.53	9.33E-01	-0.02
1046.2144@0.3764636	1046.2144	0.38	NEG	hilic				9.64E-01	6.15E-01	0.13	9.96E-01	0.00	7.41E-01	-0.10	6.72E-01	-0.14
1046.5367@1.8149648	1046.5367	1.81	POS	hilic				7.44E-01	4.44E-01	-0.24	3.47E-01	-0.26	2.09E-01	-0.43	9.96E-01	0.00
1046.5887@10.113726	1046.5887	10.11	POS	RP				6.21E-02	9.74E-02	0.52	1.89E-01	0.42	8.15E-02	0.48	1.99E-04	1.05

Supplementary table 2: Metabolomics analysis results

Compound	Mass	Retention Time	Ionization mode	column	Compound_ID	MSMS_spectra	ID_level	ANOVA P-FDR	Anorexia vs control P	Anorexia vs control Estimate	overweight vs control P	overweight vs control Estimate	obese vs control P	obese vs control Estimate	athletes vs control P	athletes vs control Estimate
1046.655@9.875398	1046.6550	9.88	NEG	RP				4.74E-02	1.80E-01	0.35	3.60E-01	-0.20	9.22E-02	-0.33	9.63E-02	0.44
1046.6934@10.296443	1046.6934	10.30	NEG	RP				1.69E-01	4.31E-01	0.20	4.97E-01	-0.19	1.00E-02	-0.59	6.49E-01	0.13
1046.7094@10.02831	1046.7094	10.03	POS	RP				1.52E-01	3.42E-01	-0.27	1.84E-01	-0.38	1.21E-02	-0.73	2.13E-02	-0.68
1046.7288@10.517538	1046.7288	10.52	POS	RP				3.90E-02	9.19E-02	0.53	3.42E-02	-0.64	4.21E-01	-0.20	9.39E-01	-0.02
1047.4781@11.857833	1047.4781	11.86	NEG	RP				6.89E-01	2.97E-01	0.27	5.35E-01	-0.15	9.34E-01	-0.02	4.21E-01	-0.21
1047.4904@3.041548	1047.4904	3.04	POS	hilic				9.72E-01	5.64E-01	0.18	4.72E-01	0.21	8.11E-01	0.07	5.45E-01	0.16
1047.7065@11.037182	1047.7065	11.04	NEG	RP				4.69E-01	1.77E-01	0.48	3.35E-02	0.61	7.76E-02	0.47	1.01E-01	0.46
1047.7133@10.028694	1047.7133	10.03	POS	RP				6.06E-01	4.99E-01	-0.21	6.25E-01	0.13	3.44E-01	-0.31	1.24E-01	-0.40
1047.731@1.1999909	1047.7310	1.20	POS	hilic				2.89E-02	1.28E-01	0.45	5.04E-02	-0.49	2.68E-02	-0.60	1.55E-01	-0.45
1047.731@10.51771	1047.7310	10.52	POS	RP				3.89E-02	9.22E-02	0.53	3.40E-02	-0.64	4.17E-01	-0.20	9.44E-01	-0.02
1047.7314@1.2094865	1047.7314	1.21	POS	hilic				1.23E-01	9.43E-02	0.58	1.03E-01	-0.45	8.24E-01	-0.06	7.56E-01	0.09
1048.1075@9.495237	1048.1075	9.50	NEG	RP				6.62E-01	8.34E-01	-0.07	9.33E-02	0.44	3.37E-01	0.26	8.22E-01	0.07
1048.232@0.39422214	1048.2320	0.39	NEG	hilic				7.31E-01	9.93E-01	0.00	3.96E-01	0.24	5.14E-01	-0.16	3.31E-01	0.31
1048.5522@1.8060799	1048.5522	1.81	POS	hilic				9.65E-01	8.39E-01	-0.06	4.83E-01	-0.20	6.59E-01	-0.15	9.12E-01	0.03
1048.6697@10.000197	1048.6697	10.00	NEG	RP				4.01E-01	1.78E-01	0.35	8.12E-01	-0.06	8.47E-02	-0.39	9.24E-01	-0.03
1049.4904@3.068104	1049.4904	3.07	POS	hilic				1.54E-01	1.45E-01	0.40	2.50E-01	0.29	5.09E-02	-0.35	7.18E-01	-0.09
1050.2535@0.3939369	1050.2535	0.39	NEG	hilic				8.55E-01	9.44E-01	0.02	5.77E-01	0.16	7.59E-01	-0.09	3.52E-01	0.31
1050.5735@9.859365	1050.5735	9.86	POS	RP				8.15E-01	3.31E-01	0.24	9.69E-01	0.01	4.27E-01	-0.15	9.55E-01	0.01
1050.6414@9.996728	1050.6414	10.00	NEG	RP				4.89E-02	9.46E-01	0.02	3.36E-02	-0.48	1.41E-01	-0.37	1.43E-01	0.43
1050.6425@1.241049	1050.6425	1.24	NEG	hilic				2.00E-03	1.09E-01	0.38	1.63E-01	-0.35	2.12E-04	-0.72	2.78E-01	0.29
1050.6436@1.3826641	1050.6436	1.38	NEG	hilic				4.39E-01	5.17E-02	-0.62	2.39E-01	-0.32	4.14E-02	-0.59	3.17E-01	-0.28
1050.6696@1.2324059	1050.6696	1.23	POS	hilic				4.94E-01	6.48E-02	0.59	6.02E-01	0.13	7.16E-01	0.08	7.76E-01	0.07
1051.066@0.8859136	1051.0660	0.89	NEG	hilic				1.38E-01	2.42E-01	-0.32	4.03E-02	0.54	1.55E-01	0.42	9.51E-01	0.02
1051.0667@3.327756	1051.0667	3.33	NEG	RP				1.68E-01	3.59E-01	-0.24	3.92E-02	0.53	1.72E-01	0.41	8.39E-01	0.05
1051.2571@0.39499995	1051.2571	0.39	NEG	hilic				9.79E-01	6.38E-01	-0.13	7.32E-01	0.10	8.67E-01	-0.05	9.30E-01	0.03
1051.6447@9.997906	1051.6447	10.00	NEG	RP				5.89E-01	7.76E-01	-0.08	1.74E-01	-0.34	4.73E-02	-0.43	9.73E-01	0.01
1052.5822@9.860057	1052.5822	9.86	POS	RP				7.90E-01	1.49E-01	-0.38	3.96E-01	-0.21	9.98E-01	0.00	4.48E-01	-0.22
1052.5872@10.081829	1052.5872	10.08	POS	RP				7.80E-01	7.77E-01	-0.08	1.56E-01	-0.31	8.95E-01	0.03	3.78E-01	-0.21
1052.5927@10.079786	1052.5927	10.08	POS	RP				8.96E-01	4.93E-01	0.23	6.11E-01	-0.13	9.54E-01	-0.01	8.65E-01	-0.04
1052.6202@1.2755209	1052.6202	1.28	POS	hilic				8.75E-02	3.06E-02	-0.71	7.66E-03	-0.69	6.65E-03	-0.76	1.20E-02	-0.74
1052.659@10.205308	1052.6590	10.21	NEG	RP				1.39E-02	3.80E-01	0.28	4.73E-03	-0.69	2.41E-01	-0.26	2.33E-01	0.33
1052.6805@1.2195557	1052.6805	1.22	POS	hilic				8.47E-01	9.86E-01	-0.01	6.73E-01	-0.10	1.24E-01	-0.34	8.89E-01	-0.04
1053.1237@9.985783	1053.1237	9.99	POS	RP				1.02E-02	8.71E-02	0.47	1.61E-01	-0.41	2.63E-02	-0.55	1.26E-01	0.47
1053.5026@0.4893452	1053.5026	0.49	POS	hilic				7.61E-01	6.21E-01	-0.16	5.20E-01	-0.18	2.19E-01	-0.33	6.25E-01	0.16
1053.5413@3.2065125	1053.5413	3.21	NEG	hilic				7.80E-01	7.55E-01	-0.10	2.66E-01	-0.31	9.87E-01	0.00	5.12E-01	0.17
1053.5413@3.2074316	1053.5413	3.21	NEG	hilic				4.28E-01	5.11E-01	-0.20	2.09E-02	-0.69	3.01E-01	-0.30	4.11E-01	-0.22
1054.2196@0.3960186	1054.2196	0.40	NEG	hilic				9.19E-01	3.91E-01	0.23	6.39E-01	0.15	9.49E-01	0.02	3.48E-01	0.28
1054.6075@10.133707	1054.6075	10.13	POS	RP				2.49E-01	2.38E-01	-0.31	9.85E-01	-0.01	8.73E-01	0.04	1.05E-01	0.51
1054.6624@11.041845	1054.6624	11.04	NEG	RP				5.32E-01	2.55E-02	0.60	1.85E-01	0.39	2.15E-01	0.31	1.64E-01	0.38
1054.6997@1.2117534	1054.6997	1.21	POS	hilic				2.74E-01	7.97E-02	0.56	9.52E-01	-0.02	3.17E-01	-0.22	6.88E-01	0.11
1055.0413@3.2082548	1055.0413	3.21	NEG	hilic				7.43E-01	8.91E-01	-0.04	3.78E-01	-0.23	4.29E-01	0.24	4.83E-01	-0.18
1055.5146@0.505135	1055.5146	0.51	POS	hilic				8.84E-01	4.52E-01	-0.22	9.83E-01	-0.01	2.65E-01	-0.30	9.99E-01	0.00
1056.1921@0.39570004	1056.1921	0.40	NEG	hilic				8.33E-01	9.46E-01	0.02	5.72E-01	-0.12	4.96E-01	-0.16	4.58E-01	0.19
1057.2559@9.544557	1057.2559	9.54	POS	RP				1.85E-01	5.06E-02	-0.52	3.66E-01	0.28	7.54E-01	0.08	1.63E-01	-0.41
1057.257@9.677363	1057.2570	9.68	POS	RP				1.92E-01	2.13E-01	-0.36	6.03E-02	0.56	6.87E-01	0.11	7.63E-01	-0.09
1057.5231@0.54840475	1057.5231	0.55	POS	hilic				1.95E-01	7.20E-01	-0.09	2.81E-03	-0.51	7.50E-01	-0.08	4.66E-01	0.18
1057.5385@3.2230105	1057.5385	3.22	NEG	hilic				4.63E-01	8.47E-01	-0.05	8.55E-01	-0.05	3.40E-01	0.28	8.20E-02	0.52
1057.6584@11.042885	1057.6584	11.04	NEG	RP				4.73E-01	5.76E-02	0.53	2.57E-01	0.32	8.18E-01	-0.05	5.17E-01	0.18
1057.9973@6.183192	1057.9973	6.18	POS	hilic				4.47E-01	8.21E-02	0.58	1.94E-01	0.38	3.73E-01	0.27	1.53E-02	0.61
1057.9976@6.229925	1057.9976	6.23	NEG	hilic				4.10E-01	3.74E-02	0.55	2.79E-01	0.27	5.80E-01	0.13	7.36E-02	0.49
1058.1385@9.495224	1058.1385	9.50	NEG	RP				7.68E-01	9.21E-01	-0.03	9.40E-01	0.02	4.29E-01	0.20	3.33E-01	-0.32
1058.1392@9.268898	1058.1392	9.27	NEG	RP				7.11E-01	3.32E-01	-0.32	8.21E-02	-0.49	5.17E-01	-0.19	7.42E-01	-0.10
1058.1414@9.696917	1058.1414	9.70	NEG	RP				7.34E-01	7.43E-01	-0.10	6.56E-01	0.15	1.63E-01	0.37	7.75E-01	-0.07
1058.2115@0.39556664	1058.2115	0.40	NEG	hilic				8.17E-01	3.34E-01	0.27	1.70E-01	0.31	2.14E-01	0.31	6.20E-01	0.13
1058.633@10.291168	1058.6330	10.29	POS	RP				4.68E-01	2.78E-01	0.31	6.38E-01	-0.11	1.60E-01	-0.29	5.17E-01	0.17
1058.6403@1.2263362	1058.6403	1.23	POS	hilic				8.31E-01	5.20E-01	0.18	4.52E-01	-0.21	4.78E-01	-0.20	6.54E-01	-0.13
1058.654@1.387234	1058.6540	1.39	NEG	hilic				1.71E-02	8.47E-01	0.06	4.33E-02	-0.60	3.22E-03	-0.78	4.79E-01	0.22
1058.654@9.837914	1058.6540	9.84	NEG	RP				7.50E-02	1.17E-01	0.45	2.59E-01	-0.32	8.09E-02	-0.42	3.47E-01	0.27
1058.6564@11.041742	1058.6564	11.04	NEG	RP				3.71E-01	3.54E-01	0.22	1.50E-02	0.61	2.23E-01	0.30	6.09E-01	0.13
1058.7336@1.195174	1058.7336	1.20	POS	hilic				8.61E-04	2.59E-03	0.89	5.13E-01	-0.08	8.90E-01	0.02	2.61E-01	0.20
1059.0013@6.182462	1059.0013	6.18	POS	hilic				5.04E-01	3.89E-01	0.30	1.04E-01	0.48	4.29E-01	0.22	1.25E-02	0.61
1059.1425@9.495694	1059.1425	9.50	NEG	RP				4.93E-01	6.31E-01	-0.15	5.22E-01	0.18	1.92E-01	0.39	1.45E-01	0.47
1060.2314@0.39565125	1060.2314	0.40	NEG	hilic				5.96E-01	2.54E-01	0.27	8.48E-02	0.41	1.32E-01	0.39	4.60E-01	0.14
1060.6451@1.2186611	1060.6451	1.22	POS	hilic				8.14E-01	9.03E-01	0.04	4.88E-01	-0.21	3.87E-01	-0.25	6.15E-01	0.15
1060.6475@1.1696659	1060.6475	1.17	POS	hilic				4.10E-01	4.60E-01	-0.21	1.14E-01	-0.47	4.66E-02	-0.57	9.70E-01	0.01
1060.6613@11.03804	1060.6613	11.04	NEG	RP				2.89E-01	6.69E-01	0.08	3.60E-02	0.53	7.44E-01	0.08	5.06E-02	0.43
1060.6692@1.3871199	1060.6692	1.39	NEG	hilic				1.28E-02	8.49E-01	0.05	2.00E-02	-0.68	6.25E-04	-0.89	8.44E-01	0.07
1060.67@1.2646537	1060.6700	1.26	NEG	hilic				9.19E-01	9.25E-01	0.02	8.23E-01	0.07	4.55E-01	-0.24	6.37E-01	-0.13
1060.67@9.990676:1	1060.6700	9.99	NEG	RP				2.49E-03	2.49E-01	0.31	1.80E-02	-0.71	6.87E-03	-0.70	3.10E-01	0.31
1060.67@9.990676:2	1060.6700	9.99	NEG	RP				2.00E-03	2.43E-01	0.31	1.67E-02	-0.72	5.70E-03	-0.71	2.86E-01	0.33
1062.2238@0.3991304	1062.2238	0.40	NEG	hilic				4.82E-01	2.25E-01	0.34	3.37E-02	0.64	1.94E-01	0.34	1.72E-01	0.46
1062.6617@10.164537	1062.6617	10.16	POS	RP				7.64E-01	3.07E-01	-0.31	8.34E-01	-0.06	7.45E-01	-0.09	1.64E-01	-0.36
1062.6624@10.156214	1062.6624	10.16	POS	RP				9.42E-01	4.45E-01	-0.27	8.75E-01	-0.05	9.37E-01	0.02	8.09E-01	-0.07

Supplementary table 2: Metabolomics analysis results

								ANOVA	Anorexia vs control		overweight vs control		obese vs control		athletes vs control	
Compound	Mass	Retention Time	Ionization mode	column	Compound_ID	MSMS_spectra	ID_level	P-FDR	P	Estimate	P	Estimate	P	Estimate	P	Estimate
1062.6635@1.209939	1062.6635	1.21	POS	hilic				8.05E-01	8.02E-01	0.07	2.82E-01	-0.35	4.25E-01	-0.22	8.43E-01	-0.06
1062.6646@11.034888	1062.6646	11.03	NEG	RP				5.27E-01	6.46E-01	0.12	7.53E-02	0.49	7.55E-01	0.08	1.59E-01	0.38
1062.6656@1.1694818	1062.6656	1.17	POS	hilic				3.96E-01	3.96E-01	-0.25	8.72E-02	-0.52	3.98E-02	-0.60	8.19E-01	-0.07
1062.6667@9.953769	1062.6667	9.95	POS	RP				7.66E-01	5.84E-01	-0.16	2.73E-01	-0.36	3.38E-01	-0.27	8.31E-01	0.07
1062.6805@1.2886386	1062.6805	1.29	NEG	hilic				5.25E-04	5.39E-03	0.86	2.65E-01	-0.22	2.37E-01	-0.24	1.18E-02	0.66
1062.685@10.122272	1062.6850	10.12	NEG	RP				9.91E-03	9.95E-02	0.50	9.45E-02	-0.52	8.66E-03	-0.70	8.55E-01	0.05
1062.685@10.21306	1062.6850	10.21	NEG	RP				4.69E-03	5.93E-03	0.79	1.61E-01	-0.40	3.52E-01	-0.24	6.82E-02	0.52
1063.5394@0.425489	1063.5394	0.43	POS	hilic				9.45E-01	3.14E-01	0.28	6.18E-01	0.13	4.80E-01	0.19	6.10E-01	0.16
1063.6696@9.954291	1063.6696	9.95	POS	RP				7.24E-01	8.16E-01	-0.07	2.86E-01	-0.35	3.52E-01	-0.26	6.73E-01	0.13
1063.684@1.3780001	1063.6840	1.38	NEG	hilic				2.64E-01	5.82E-01	0.15	2.36E-02	-0.57	2.08E-01	-0.32	5.96E-01	-0.16
1063.6895@10.121562	1063.6895	10.12	NEG	RP				1.06E-02	8.69E-02	0.53	1.07E-01	-0.51	1.05E-02	-0.68	9.03E-01	0.03
1064.2325@9.260195	1064.2325	9.26	NEG	RP				6.79E-01	2.70E-01	0.31	6.85E-01	-0.13	7.13E-01	-0.11	3.80E-01	0.29
1064.237@0.40010807	1064.2370	0.40	NEG	hilic				6.62E-01	2.94E-01	0.30	1.88E-01	0.40	4.23E-01	0.21	9.05E-02	0.53
1064.6552@10.893999	1064.6552	10.89	NEG	RP				5.11E-01	7.27E-02	-0.57	9.00E-01	-0.04	7.52E-01	0.09	9.37E-01	-0.03
1064.6624@10.916364	1064.6624	10.92	NEG	RP				1.04E-01	6.72E-02	-0.52	9.64E-03	-0.83	7.01E-01	-0.10	1.12E-01	-0.48
1064.6779@1.0888008	1064.6779	1.09	POS	hilic				2.10E-01	1.28E-01	0.45	1.75E-01	-0.42	2.84E-01	-0.28	8.74E-01	0.05
1064.679@10.14146	1064.6790	10.14	POS	RP				2.81E-01	3.29E-01	-0.26	1.38E-02	-0.54	1.57E-01	-0.35	9.34E-01	-0.02
1064.6794@1.2085379	1064.6794	1.21	POS	hilic				7.34E-01	2.52E-01	0.33	5.28E-01	-0.20	9.52E-01	-0.02	6.70E-01	0.14
1064.69@1.2870291	1064.6900	1.29	NEG	hilic				7.21E-02	2.20E-01	0.29	3.05E-01	-0.18	8.43E-01	-0.04	4.38E-02	0.56
1064.701@1.2867142	1064.7010	1.29	NEG	hilic				2.76E-02	2.92E-01	0.25	1.82E-01	-0.22	3.74E-01	-0.15	3.82E-02	0.56
1064.701@10.500197	1064.7010	10.50	NEG	RP				4.00E-01	2.25E-01	0.32	3.01E-01	-0.29	4.98E-01	-0.18	4.77E-01	0.21
1065.122@9.962634	1065.1220	9.96	POS	RP				4.99E-03	4.33E-01	0.22	4.92E-02	-0.57	1.55E-03	-0.81	2.74E-01	0.32
1065.2404@0.39929053	1065.2404	0.40	NEG	hilic				4.15E-01	5.51E-01	0.19	3.36E-02	0.62	3.66E-01	0.23	9.95E-02	0.54
1065.4818@12.057706	1065.4818	12.06	NEG	RP				6.00E-01	3.89E-01	-0.24	4.60E-01	-0.20	1.72E-01	-0.37	4.82E-01	0.14
1065.5092@0.4874838	1065.5092	0.49	POS	hilic				8.96E-01	8.43E-01	-0.05	8.61E-01	-0.04	6.13E-01	-0.11	1.82E-01	-0.26
1065.649@10.785288	1065.6490	10.79	NEG	RP				7.42E-01	7.19E-01	-0.09	8.75E-01	0.04	2.21E-01	0.33	8.37E-01	-0.06
1065.68@10.222481	1065.6800	10.22	POS	RP				1.27E-01	1.48E-01	0.46	1.94E-01	-0.39	3.88E-01	-0.22	2.12E-01	0.32
1065.8171@4.556361	1065.8171	4.56	NEG	hilic				3.91E-01	2.69E-02	0.65	1.59E-01	0.44	8.65E-01	0.05	6.19E-01	0.13
1066.5793@10.151809	1066.5793	10.15	POS	RP				6.74E-01	3.22E-01	-0.29	5.25E-01	0.21	5.63E-01	-0.15	5.44E-01	0.17
1066.58@10.043589	1066.5800	10.04	POS	RP				8.35E-01	9.01E-01	0.03	3.39E-01	0.26	5.20E-01	-0.19	9.97E-01	0.00
1066.6927@10.14388	1066.6927	10.14	POS	RP				3.14E-01	1.58E-01	0.49	3.17E-01	-0.32	4.71E-01	-0.19	9.73E-01	-0.01
1066.6938@1.1830692	1066.6938	1.18	POS	hilic				5.03E-01	7.15E-01	-0.12	3.97E-01	-0.23	1.01E-01	-0.48	5.00E-01	0.19
1067.5153@0.4865701	1067.5153	0.49	POS	hilic				8.69E-01	3.47E-01	-0.26	7.77E-01	0.08	8.32E-01	0.06	6.23E-01	0.14
1067.5898@10.0033045	1067.5898	10.00	POS	RP				5.91E-03	5.60E-03	0.86	6.57E-01	0.12	3.80E-01	0.22	1.08E-04	1.00
1067.6978@1.1563123	1067.6978	1.16	POS	hilic				8.39E-01	9.14E-01	0.03	8.90E-01	0.04	2.99E-01	-0.30	6.82E-01	0.12
1067.7017@10.1614685	1067.7017	10.16	POS	RP				8.89E-01	3.20E-01	0.26	7.36E-01	0.08	9.39E-01	0.02	4.03E-01	0.25
1067.7056@11.039429	1067.7056	11.04	NEG	RP				9.33E-01	5.43E-01	-0.17	7.42E-01	-0.10	7.31E-01	0.10	6.99E-01	0.10
1068.2147@0.3965298	1068.2147	0.40	NEG	hilic				8.93E-01	2.49E-01	0.37	3.91E-01	0.26	4.93E-01	0.20	6.20E-01	0.16
1068.5157@1.8136904	1068.5157	1.81	POS	hilic				8.25E-01	1.77E-01	-0.35	3.67E-01	-0.21	3.28E-01	-0.27	3.43E-01	-0.22
1068.5741@1.465971	1068.5741	1.47	NEG	hilic				6.22E-01	2.61E-01	0.40	5.72E-01	-0.13	3.92E-01	0.22	3.66E-01	0.28
1068.5842@1.3351885	1068.5842	1.34	POS	hilic				9.65E-01	4.36E-01	-0.20	9.74E-01	0.01	9.64E-01	-0.01	7.72E-01	-0.08
1068.6165@9.951604	1068.6165	9.95	NEG	RP				1.73E-02	1.27E-01	-0.35	7.23E-02	0.46	2.68E-02	0.68	7.53E-01	-0.08
1068.6368@10.23	1068.6368	10.23	NEG	RP				3.11E-01	7.88E-01	0.07	1.70E-01	-0.28	6.69E-02	-0.38	5.37E-01	0.16
1068.7065@10.519466	1068.7065	10.52	POS	RP				1.91E-02	4.29E-02	0.61	4.46E-02	-0.62	2.87E-01	-0.27	8.32E-01	0.06
1069.1512@10.234102	1069.1512	10.23	POS	RP				5.83E-03	3.22E-02	0.66	7.72E-02	-0.54	3.31E-02	-0.54	7.29E-01	0.10
1069.5142@12.806516	1069.5142	12.81	NEG	RP				9.74E-01	8.80E-01	0.05	6.57E-01	0.13	4.37E-01	0.20	5.68E-01	0.16
1069.596@10.214251	1069.5960	10.21	POS	RP				3.38E-01	2.09E-01	0.35	2.22E-02	0.69	1.42E-01	0.40	5.46E-01	0.16
1069.654@10.231533	1069.6540	10.23	POS	RP				5.63E-03	4.36E-02	0.58	8.85E-02	-0.44	1.19E-02	-0.59	9.32E-01	-0.02
1069.7115@1.158585	1069.7115	1.16	POS	hilic				8.77E-01	4.84E-01	-0.25	6.46E-01	-0.12	2.64E-01	-0.33	3.14E-01	-0.30
1069.7133@10.51943	1069.7133	10.52	POS	RP				4.59E-02	9.21E-02	0.51	3.87E-02	-0.62	3.21E-01	-0.26	9.06E-01	-0.04
1070.1569@1.2173071	1070.1569	1.22	POS	hilic				2.88E-02	3.05E-02	0.68	4.92E-01	-0.17	1.30E-01	-0.33	2.03E-01	0.37
1070.5323@1.8021058	1070.5323	1.80	POS	hilic				8.41E-01	2.51E-01	-0.35	6.56E-01	-0.12	4.07E-01	-0.27	9.44E-01	-0.02
1070.5875@1.4654114	1070.5875	1.47	NEG	hilic				1.16E-01	4.29E-01	0.27	1.15E-01	-0.36	6.49E-01	-0.13	4.08E-02	0.56
1070.5876@10.00089	1070.5876	10.00	POS	RP				1.62E-05	1.41E-03	0.91	6.23E-01	-0.12	1.23E-02	-0.58	2.81E-03	0.80
1070.721@1.2001952	1070.7210	1.20	POS	hilic				2.02E-01	5.62E-01	0.21	4.72E-02	-0.55	6.55E-02	-0.43	9.82E-01	-0.01
1070.7253@10.438491	1070.7253	10.44	POS	RP				2.10E-01	8.02E-01	0.06	5.62E-01	-0.11	2.58E-02	0.48	1.34E-01	0.42
1071.164@1.2142887	1071.1640	1.21	POS	hilic				9.44E-03	3.00E-03	0.89	4.08E-01	-0.22	3.28E-01	-0.22	3.19E-01	0.29
1071.5981@10.152399	1071.5981	10.15	POS	RP				7.63E-01	6.23E-01	0.14	7.53E-01	0.09	1.12E-01	0.40	2.14E-01	0.34
1071.705@11.036373	1071.7050	11.04	NEG	RP				8.74E-01	5.51E-01	0.16	6.42E-01	-0.10	6.21E-01	-0.12	6.66E-01	0.10
1071.721@1.1991541	1071.7210	1.20	POS	hilic				1.76E-03	2.04E-02	0.72	3.13E-02	-0.54	5.65E-02	-0.44	8.85E-01	0.04
1072.602@10.211358	1072.6020	10.21	POS	RP				5.06E-02	1.57E-03	0.89	6.73E-01	0.12	9.67E-01	0.01	2.35E-02	0.63
1072.9084@12.382098	1072.9084	12.38	NEG	RP				5.96E-01	2.81E-01	0.30	8.59E-01	-0.04	1.49E-01	0.42	3.06E-01	0.29
1073.1833@10.517509	1073.1833	10.52	POS	RP				7.86E-02	9.25E-02	0.53	5.89E-02	-0.54	5.94E-01	-0.14	9.51E-01	0.02
1073.591@10.151996	1073.5910	10.15	POS	RP				1.10E-02	2.39E-02	0.62	7.73E-01	0.08	8.06E-01	-0.08	7.69E-05	0.99
1073.6855@10.516963	1073.6855	10.52	POS	RP				7.59E-02	9.81E-02	0.52	5.57E-02	-0.55	5.01E-01	-0.17	9.64E-01	-0.01
1073.7286@11.036677	1073.7286	11.04	NEG	RP				1.47E-01	9.01E-01	-0.04	9.95E-01	0.00	7.49E-03	0.76	8.27E-01	-0.07
1074.1998@0.39480168	1074.1998	0.39	NEG	hilic				9.63E-01	5.99E-01	0.15	4.39E-01	0.23	6.53E-01	0.14	4.73E-01	0.22
1074.638@1.2357885	1074.6380	1.24	NEG	hilic				3.13E-02	8.32E-01	0.06	1.22E-01	-0.41	4.90E-03	-0.68	3.10E-01	0.30
1074.6404@9.85775	1074.6404	9.86	NEG	RP				2.34E-01	4.82E-01	-0.19	1.29E-01	-0.37	1.03E-02	-0.69	6.60E-01	-0.11
1074.6405@1.2300137	1074.6405	1.23	NEG	hilic				3.59E-02	3.19E-01	0.26	8.39E-02	-0.43	1.28E-02	-0.58	5.26E-01	0.19
1074.6414@9.958794	1074.6414	9.96	NEG	RP				5.88E-03	6.70E-01	0.11	3.47E-02	-0.65	6.41E-04	-0.89	5.12E-01	0.19
1074.6486@1.361	1074.6486	1.36	NEG	hilic				1.04E-01	1.61E-01	-0.33	3.39E-01	0.23	8.43E-01	-0.05	6.91E-02	0.52
1076.2072@9.548316	1076.2072	9.55	NEG	RP				1.23E-01	3.98E-03	0.91	3.48E-01	0.26	1.79E-02	0.62	8.74E-02	0.53

Supplementary table 2: Metabolomics analysis results

								ANOVA	Anorexia vs control		overweight vs control		obese vs control		athletes vs control	
Compound	Mass	Retention Time	Ionization mode	column	Compound_ID	MSMS_spectra	ID_level	P-FDR	P	Estimate	P	Estimate	P	Estimate	P	Estimate
1076.2087@0.3940256	1076.2087	0.39	NEG	hilic				8.54E-01	4.48E-01	0.21	1.93E-01	0.40	5.22E-01	0.18	4.89E-01	0.24
1076.612@9.9146	1076.6120	9.91	POS	RP				7.77E-01	6.94E-01	-0.13	1.48E-01	-0.46	5.30E-01	-0.19	2.78E-01	-0.26
1076.6136@9.949422	1076.6136	9.95	POS	RP				7.58E-01	6.23E-01	0.13	9.37E-01	-0.02	3.24E-01	-0.30	5.25E-01	0.16
1076.6187@1.244755	1076.6187	1.24	POS	hilic				4.33E-01	2.58E-01	0.29	6.29E-02	0.44	7.50E-01	-0.07	9.12E-01	0.03
1076.6567@1.2095264	1076.6567	1.21	NEG	hilic				1.53E-02	4.13E-01	0.22	4.11E-02	-0.42	2.88E-03	-0.57	5.11E-01	0.17
1076.7021@10.234329	1076.7021	10.23	NEG	RP				3.75E-04	8.98E-02	0.50	3.14E-04	-0.95	4.52E-02	-0.46	6.58E-01	0.12
1076.7358@10.490258	1076.7358	10.49	NEG	RP				4.91E-02	1.13E-01	0.54	8.54E-02	-0.43	2.02E-01	-0.30	5.63E-01	0.15
1077.2137@0.39326125	1077.2137	0.39	NEG	hilic				7.45E-01	5.58E-01	0.17	3.03E-01	0.30	7.62E-01	-0.08	2.57E-01	0.35
1077.547@1.3381431	1077.5470	1.34	POS	hilic				5.04E-02	7.92E-01	0.06	9.90E-01	0.00	4.50E-03	0.82	2.58E-01	0.26
1077.623@9.953938	1077.6230	9.95	POS	RP				2.08E-01	7.15E-01	-0.10	2.83E-01	-0.35	7.34E-03	-0.74	8.72E-01	0.05
1077.8721@10.70734	1077.8721	10.71	NEG	RP				5.27E-01	7.79E-01	0.06	1.07E-01	0.41	7.32E-02	0.40	2.77E-01	0.23
1078.2166@0.38584995	1078.2166	0.39	NEG	hilic				8.54E-01	9.18E-01	0.03	4.97E-01	0.20	8.78E-01	-0.04	2.45E-01	0.32
1078.6707@1.3332038	1078.6707	1.33	NEG	hilic				1.20E-02	3.71E-02	0.63	7.94E-02	-0.44	1.47E-01	-0.37	2.14E-01	0.37
1078.6757@10.2304945	1078.6757	10.23	NEG	RP				1.74E-01	2.86E-01	0.36	2.97E-01	-0.31	2.86E-02	-0.53	8.66E-01	0.05
1079.554@1.3365887	1079.5540	1.34	POS	hilic				4.53E-01	1.63E-01	-0.42	3.06E-01	0.32	8.22E-01	0.07	9.42E-01	0.02
1079.555@1.1563246	1079.5550	1.16	POS	hilic				3.30E-01	3.64E-01	0.29	1.20E-01	0.41	5.28E-03	0.77	2.14E-01	0.37
1079.5555@10.172367	1079.5555	10.17	POS	RP				2.02E-01	9.80E-01	-0.01	6.07E-02	0.62	4.65E-02	0.59	7.80E-02	0.53
1079.5566@10.074432	1079.5566	10.07	POS	RP				8.86E-01	6.15E-01	0.14	5.24E-01	0.16	2.86E-01	0.25	8.09E-01	-0.05
1079.6324@10.781272	1079.6324	10.78	NEG	RP				5.05E-01	4.10E-01	-0.18	3.43E-01	0.26	1.88E-01	0.31	7.39E-01	-0.09
1079.6772@10.230849	1079.6772	10.23	NEG	RP				4.42E-01	9.48E-01	0.02	1.40E-01	-0.36	2.33E-02	-0.54	5.93E-01	-0.15
1079.9775@6.184847	1079.9775	6.18	POS	hilic				6.47E-01	8.18E-02	0.57	3.08E-01	0.31	3.76E-01	0.27	1.63E-01	0.40
1079.9788@6.2279882	1079.9788	6.23	NEG	hilic				5.59E-01	3.31E-01	0.27	3.36E-01	0.27	1.07E-01	0.39	4.61E-02	0.59
1080.2408@0.39600942	1080.2408	0.40	NEG	hilic				7.96E-01	8.16E-01	-0.06	6.58E-01	0.14	5.73E-01	0.13	1.99E-01	0.39
1080.5583@1.3350716	1080.5583	1.34	POS	hilic				3.37E-01	2.57E-01	-0.26	1.22E-01	0.41	3.95E-01	0.21	4.62E-01	0.16
1080.5594@10.167939	1080.5594	10.17	POS	RP				5.17E-01	7.00E-01	-0.10	5.81E-01	0.16	4.70E-02	0.49	3.12E-01	0.28
1080.574@0.4318966	1080.5740	0.43	POS	hilic				8.41E-01	1.79E-01	0.41	5.54E-01	0.16	5.52E-01	0.17	3.52E-01	0.24
1080.6147@9.945346	1080.6147	9.95	POS	RP				9.18E-01	3.89E-01	-0.20	5.67E-01	-0.15	8.36E-01	-0.06	7.43E-01	0.08
1080.6403@10.238208	1080.6403	10.24	POS	RP				5.01E-02	3.10E-02	0.65	9.92E-02	-0.44	3.83E-01	-0.21	9.12E-01	-0.03
1080.6442@1.1775556	1080.6442	1.18	POS	hilic				9.22E-01	9.92E-01	0.00	4.77E-01	-0.19	3.84E-01	-0.23	7.44E-01	-0.09
1080.6956@1.4122992	1080.6956	1.41	NEG	hilic				6.45E-02	4.80E-03	0.78	9.35E-01	-0.02	9.59E-02	0.57	2.03E-02	0.67
1080.982@6.186461	1080.9820	6.19	POS	hilic				7.96E-01	3.10E-01	0.32	5.39E-01	0.17	6.40E-01	0.14	1.11E-01	0.44
1080.9843@6.227634	1080.9843	6.23	NEG	hilic				3.33E-01	1.35E-02	0.63	2.65E-01	0.29	2.95E-01	0.24	7.02E-02	0.54
1081.6501@10.782086	1081.6501	10.78	NEG	RP				1.73E-01	2.06E-01	-0.29	1.30E-01	0.42	4.55E-01	0.19	3.77E-01	-0.21
1081.6812@11.03749	1081.6812	11.04	NEG	RP				8.34E-01	4.28E-01	0.25	4.06E-01	0.24	1.76E-01	0.31	1.72E-01	0.37
1081.685@10.888552	1081.6850	10.89	POS	RP				2.33E-01	4.66E-01	0.19	1.87E-02	0.66	6.79E-02	0.56	9.65E-01	0.01
1081.6986@1.4121093	1081.6986	1.41	NEG	hilic				8.53E-02	2.97E-02	0.64	3.30E-01	-0.23	4.18E-01	0.26	2.43E-02	0.58
1082.2365@0.39539304	1082.2365	0.40	NEG	hilic				3.65E-01	2.23E-01	0.31	2.49E-02	0.70	2.04E-01	0.34	1.13E-01	0.53
1082.6523@9.847925	1082.6523	9.85	NEG	RP				4.77E-01	5.13E-01	0.18	6.72E-01	-0.10	1.11E-01	-0.35	5.49E-01	0.17
1082.6656@1.1525173	1082.6656	1.15	POS	hilic				8.20E-01	9.70E-01	-0.01	5.43E-01	-0.17	1.89E-01	-0.35	9.68E-01	0.01
1082.7032@10.507786	1082.7032	10.51	NEG	RP				8.18E-02	7.45E-02	0.54	8.98E-02	-0.53	7.76E-01	-0.07	6.45E-01	0.14
1082.7354@13.242737	1082.7354	13.24	POS	RP				5.14E-01	9.17E-01	0.03	1.39E-01	0.40	4.05E-01	-0.22	6.16E-01	-0.17
1082.7819@11.719395	1082.7819	11.72	POS	RP				1.41E-01	2.62E-02	0.53	3.32E-02	0.64	8.85E-01	0.04	6.32E-01	-0.15
1083.6833@11.038171	1083.6833	11.04	NEG	RP				5.89E-01	1.59E-01	0.50	9.11E-02	0.49	1.33E-01	0.41	9.81E-02	0.45
1084.2205@0.39314407	1084.2205	0.39	NEG	hilic				6.50E-01	7.51E-01	-0.09	2.14E-01	0.39	8.65E-01	-0.05	3.68E-01	0.29
1084.6443@9.949162	1084.6443	9.95	POS	RP				8.66E-01	2.37E-01	-0.34	3.55E-01	-0.31	7.54E-01	-0.10	7.13E-01	-0.11
1084.6462@1.1643752	1084.6462	1.16	POS	hilic				7.63E-01	6.15E-01	-0.13	3.15E-01	0.24	8.94E-01	0.03	5.63E-01	-0.13
1084.6682@1.1203026	1084.6682	1.12	POS	hilic				9.89E-01	7.46E-01	-0.09	8.69E-01	0.04	7.18E-01	0.09	9.94E-01	0.00
1084.6698@1.2318004	1084.6698	1.23	NEG	hilic				2.81E-01	8.23E-01	0.06	9.98E-02	-0.39	2.39E-02	-0.52	6.30E-01	-0.13
1084.67@1.3603845	1084.6700	1.36	NEG	hilic				1.55E-02	3.76E-01	0.24	2.66E-02	-0.66	1.83E-03	-0.80	8.98E-01	-0.04
1084.67@9.916292	1084.6700	9.92	NEG	RP				5.85E-01	3.89E-01	0.26	2.23E-01	-0.33	3.18E-01	-0.26	9.39E-01	0.03
1084.6707@9.959827	1084.6707	9.96	NEG	RP				4.37E-03	8.69E-01	0.04	1.49E-02	-0.74	5.29E-04	-0.92	6.40E-01	0.14
1084.6729@1.2668408	1084.6729	1.27	NEG	hilic				7.01E-01	6.85E-01	0.12	3.61E-01	-0.24	3.10E-01	-0.24	6.39E-01	0.14
1084.68@10.520132	1084.6800	10.52	POS	RP				2.91E-01	6.54E-02	0.53	3.31E-01	-0.30	6.59E-01	-0.12	6.32E-01	0.15
1085.5327@0.5301757	1085.5327	0.53	POS	hilic				9.71E-01	7.36E-01	-0.09	4.70E-01	-0.18	9.40E-01	0.02	9.67E-01	-0.01
1085.6733@9.914121	1085.6733	9.91	NEG	RP				4.02E-01	8.90E-02	0.54	5.46E-01	-0.15	7.81E-01	-0.08	4.57E-01	0.25
1086.169@9.2067795	1086.1690	9.21	NEG	RP				8.63E-01	4.65E-01	0.20	7.84E-01	0.08	3.61E-01	-0.23	8.94E-01	0.04
1086.2336@9.549868	1086.2336	9.55	NEG	RP				8.44E-01	7.64E-01	0.09	3.67E-01	0.28	6.94E-01	-0.12	8.46E-01	-0.07
1086.2367@0.39322218	1086.2367	0.39	NEG	hilic				3.96E-01	9.54E-01	0.02	4.22E-02	0.63	7.47E-01	0.09	3.29E-01	0.33
1086.6833@1.342838	1086.6833	1.34	NEG	hilic				6.58E-03	2.91E-01	0.33	1.47E-02	-0.68	2.44E-03	-0.77	8.55E-01	0.05
1086.685@10.140936	1086.6850	10.14	NEG	RP				7.69E-01	4.92E-01	0.23	3.89E-01	-0.27	7.38E-01	0.08	8.23E-01	-0.07
1087.2397@0.39278376	1087.2397	0.39	NEG	hilic				7.38E-01	9.82E-01	-0.01	2.80E-01	0.34	9.03E-01	-0.04	3.03E-01	0.33
1087.5396@0.5238393	1087.5396	0.52	POS	hilic				7.71E-01	8.26E-01	-0.06	1.63E-01	0.33	6.22E-01	0.12	7.33E-01	0.08
1087.6663@9.949854	1087.6663	9.95	POS	RP				8.86E-01	2.80E-01	-0.31	3.44E-01	-0.31	6.79E-01	-0.13	7.21E-01	-0.10
1087.7021@11.037756	1087.7021	11.04	NEG	RP				6.73E-01	1.36E-01	0.48	4.59E-01	0.23	1.71E-01	0.38	1.27E-01	0.45
1088.472@3.04199	1088.4720	3.04	POS	hilic				7.31E-01	7.52E-01	-0.10	6.31E-01	-0.12	5.02E-01	0.20	2.33E-01	-0.32
1088.6772@1.1966289	1088.6772	1.20	POS	hilic				8.64E-01	4.28E-01	-0.24	2.25E-01	-0.38	6.37E-01	-0.13	5.37E-01	-0.19
1088.6982@1.2262174	1088.6982	1.23	NEG	hilic				3.61E-01	2.97E-01	0.26	4.78E-01	-0.18	9.02E-02	-0.40	7.36E-01	-0.07
1088.6996@1.3300596	1088.6996	1.33	NEG	hilic				4.77E-03	1.18E-02	0.75	6.30E-02	-0.53	7.42E-02	-0.45	8.54E-01	0.06
1088.7018@10.227649	1088.7018	10.23	NEG	RP				7.78E-03	5.88E-02	0.56	7.01E-02	-0.57	1.81E-02	-0.62	8.55E-01	0.05
1088.7083@11.039273	1088.7083	11.04	NEG	RP				7.88E-01	5.80E-01	0.12	1.64E-01	0.28	4.02E-01	0.19	1.76E-01	0.30
1089.1198@9.945499	1089.1198	9.95	POS	RP				4.47E-01	4.21E-01	-0.20	7.97E-01	0.07	8.43E-02	0.47	9.90E-01	0.00
1089.5657@9.848049	1089.5657	9.85	POS	RP				1.06E-02	1.31E-01	0.43	5.13E-03	-0.59	7.00E-02	-0.40	7.18E-01	0.10
1089.576@9.866487	1089.5760	9.87	POS	RP				9.19E-02	1.49E-01	0.45	1.84E-01	-0.39	1.41E-01	-0.36	3.65E-01	0.23

Supplementary table 2: Metabolomics analysis results

Compound	Mass	Retention Time	Ionization mode	column	Compound_ID	MSMS_spectra	ID_level	ANOVA P-FDR	Anorexia vs control P	Anorexia vs control Estimate	overweight vs control P	overweight vs control Estimate	obese vs control P	obese vs control Estimate	athletes vs control P	athletes vs control Estimate
1089.6385@9.946111	1089.6385	9.95	POS	RP				3.87E-01	9.56E-03	-0.62	3.08E-01	-0.27	8.68E-01	-0.04	4.36E-01	-0.22
1090.2104@0.39331624	1090.2104	0.39	NEG	hilic				5.73E-01	1.77E-01	0.39	3.18E-01	-0.28	9.36E-01	0.02	8.12E-01	-0.08
1090.2133@9.523983	1090.2133	9.52	NEG	RP				3.54E-01	6.78E-01	0.12	1.93E-01	0.37	2.09E-01	-0.38	3.72E-01	0.24
1090.5872@10.130782	1090.5872	10.13	NEG	RP				7.46E-01	8.95E-01	0.04	4.03E-01	0.24	9.21E-01	-0.03	2.00E-01	0.41
1090.5905@10.150571	1090.5905	10.15	POS	RP				2.91E-01	1.33E-01	0.49	9.11E-01	-0.04	9.51E-01	0.02	4.17E-02	0.59
1090.695@1.1845098	1090.6950	1.18	POS	hilic				8.20E-01	7.36E-01	0.11	9.31E-01	-0.02	4.77E-01	-0.22	4.44E-01	0.23
1090.701@10.073004	1090.7010	10.07	POS	RP				5.67E-01	2.32E-01	0.39	5.00E-01	-0.20	4.01E-01	0.21	7.58E-01	-0.08
1090.7155@1.324263	1090.7155	1.32	NEG	hilic				5.89E-03	5.22E-03	0.85	1.55E-01	-0.40	2.03E-01	-0.35	5.99E-01	0.16
1090.7155@1.3243872	1090.7155	1.32	NEG	hilic				1.99E-02	8.32E-02	0.57	2.73E-02	-0.62	2.13E-01	-0.31	7.39E-01	0.10
1090.7166@10.409791	1090.7166	10.41	NEG	RP				1.62E-01	3.24E-01	0.30	6.64E-02	-0.44	1.35E-01	-0.37	9.71E-01	0.01
1091.5771@9.849746	1091.5771	9.85	POS	RP				1.37E-01	7.36E-01	0.08	9.42E-02	-0.47	2.65E-02	-0.64	7.35E-01	0.10
1091.5815@9.945938	1091.5815	9.95	POS	RP				7.21E-02	3.37E-01	0.28	1.34E-01	-0.45	1.19E-01	-0.35	1.11E-01	0.45
1091.6967@1.193704	1091.6967	1.19	POS	hilic				4.14E-01	7.31E-01	0.11	9.65E-02	-0.48	1.09E-01	-0.40	2.12E-01	-0.39
1092.585@9.847674	1092.5850	9.85	POS	RP				1.99E-02	8.66E-01	0.05	4.67E-02	-0.58	4.28E-03	-0.77	4.34E-01	0.24
1092.5865@9.956808	1092.5865	9.96	POS	RP				4.94E-01	2.22E-01	0.39	3.90E-01	0.24	8.97E-01	-0.03	1.22E-01	0.52
1092.7292@1.313937	1092.7292	1.31	NEG	hilic				9.75E-02	9.53E-02	0.55	5.57E-02	-0.49	7.11E-01	-0.10	6.96E-01	0.11
1092.7325@10.504742	1092.7325	10.50	NEG	RP				2.24E-02	3.20E-02	0.67	6.09E-02	-0.55	5.02E-01	-0.17	6.35E-01	0.14
1092.7336@10.438706	1092.7336	10.44	NEG	RP				9.85E-01	8.31E-01	0.06	9.77E-01	-0.01	6.04E-01	-0.15	9.90E-01	0.00
1092.7725@10.945272	1092.7725	10.95	POS	RP				3.45E-01	5.95E-01	0.14	2.48E-01	-0.24	1.18E-01	-0.32	3.89E-01	0.26
1093.1512@1.1988165	1093.1512	1.20	POS	hilic				9.01E-01	2.92E-01	0.26	5.25E-01	0.14	3.25E-01	0.24	7.28E-01	0.08
1093.6031@10.245681	1093.6031	10.25	POS	RP				1.85E-02	8.09E-03	0.85	8.41E-01	0.05	5.47E-01	-0.15	2.08E-02	0.67
1093.7115@1.191784	1093.7115	1.19	POS	hilic				6.09E-02	6.99E-02	0.55	1.35E-01	-0.42	1.60E-01	-0.35	4.66E-01	-0.22
1093.7332@1.3119619	1093.7332	1.31	NEG	hilic				9.50E-02	2.47E-02	0.69	2.18E-01	-0.34	7.66E-01	0.08	6.11E-01	0.15
1093.7365@10.461068	1093.7365	10.46	NEG	RP				9.69E-01	9.18E-01	0.04	4.49E-01	-0.20	9.64E-01	-0.01	9.72E-01	-0.01
1094.153@9.524649	1094.1530	9.52	NEG	RP				7.70E-01	4.73E-01	0.24	7.63E-01	0.08	1.19E-01	0.43	9.69E-01	0.01
1094.2404@0.404875	1094.2404	0.40	NEG	hilic				9.62E-01	8.14E-01	-0.06	5.58E-01	-0.16	7.77E-01	0.07	8.62E-01	0.04
1094.5887@9.9566965	1094.5887	9.96	POS	RP				8.57E-02	9.07E-01	0.03	2.12E-01	-0.40	3.44E-03	-0.77	6.46E-01	0.14
1094.6082@10.147375	1094.6082	10.15	NEG	RP				8.77E-01	8.90E-01	0.04	2.79E-01	0.25	6.72E-01	-0.11	9.65E-01	-0.01
1094.7195@11.03673	1094.7195	11.04	NEG	RP				8.04E-01	9.24E-01	0.03	5.76E-01	-0.13	3.09E-01	0.26	6.61E-01	0.11
1095.0013@2.2269492	1095.0013	2.23	NEG	RP				6.37E-01	5.98E-01	0.17	1.12E-01	-0.38	3.84E-01	-0.29	4.87E-01	-0.19
1095.5168@0.51162344	1095.5168	0.51	POS	hilic				7.32E-01	8.78E-01	-0.04	1.73E-01	0.36	8.46E-01	0.05	3.46E-01	0.24
1095.6086@10.24576	1095.6086	10.25	POS	RP				1.22E-02	6.34E-03	0.80	3.76E-01	-0.26	2.49E-01	-0.28	1.14E-01	0.46
1095.612@1.220279	1095.6120	1.22	POS	hilic				2.31E-01	1.07E-01	0.50	8.76E-01	-0.04	9.78E-02	-0.40	9.72E-01	-0.01
1096.5197@3.194513	1096.5197	3.19	NEG	hilic				5.89E-01	4.05E-01	0.27	5.37E-01	0.15	3.07E-01	0.27	4.00E-02	0.60
1096.5448@9.975579	1096.5448	9.98	NEG	RP				8.99E-04	5.54E-03	0.79	1.59E-02	-0.60	2.59E-01	-0.31	1.73E-01	0.35
1096.6143@10.243663	1096.6143	10.24	POS	RP				3.18E-02	1.08E-02	0.77	3.47E-01	-0.28	3.72E-01	-0.24	3.05E-01	0.29
1097.529@0.5110809	1097.5290	0.51	POS	hilic				9.15E-01	4.62E-01	0.23	4.30E-01	0.23	6.04E-01	0.15	2.32E-01	0.32
1097.6241@1.2136337	1097.6241	1.21	POS	hilic				9.85E-01	5.73E-01	-0.20	7.55E-01	-0.09	6.47E-01	-0.13	7.51E-01	-0.10
1097.643@10.524997	1097.6430	10.52	POS	RP				6.62E-02	1.25E-02	0.71	2.72E-01	-0.34	8.11E-01	0.06	2.70E-01	0.35
1097.6663@0.7239553	1097.6663	0.72	POS	hilic				3.52E-02	1.13E-01	-0.44	6.79E-02	-0.46	3.17E-05	-0.95	3.15E-01	-0.26
1098.6147@10.239606	1098.6147	10.24	POS	RP				1.70E-02	2.72E-02	0.64	2.19E-01	-0.38	5.33E-02	-0.47	3.38E-01	0.29
1098.6215@1.2038987	1098.6215	1.20	POS	hilic				9.65E-01	7.58E-01	-0.09	3.69E-01	-0.23	6.85E-01	-0.10	7.81E-01	-0.08
1098.6396@9.944693	1098.6396	9.94	NEG	RP				2.86E-01	6.70E-01	-0.13	1.94E-01	0.40	2.11E-02	0.56	8.61E-01	-0.05
1099.2137@0.3885259	1099.2137	0.39	NEG	hilic				7.91E-01	5.58E-01	0.17	3.34E-01	-0.25	7.01E-01	-0.11	4.13E-01	-0.26
1099.5236@1.3424408	1099.5236	1.34	POS	hilic				9.42E-01	8.58E-01	0.05	8.10E-01	0.06	6.82E-01	-0.10	4.99E-01	-0.17
1099.5378@0.51271707	1099.5378	0.51	POS	hilic				7.85E-01	8.80E-01	-0.05	2.03E-01	0.34	8.47E-01	-0.05	6.09E-01	0.14
1099.6407@10.524474	1099.6407	10.52	POS	RP				5.23E-02	9.42E-03	0.71	2.20E-01	-0.38	8.96E-01	0.03	2.64E-01	0.35
1099.752@4.4720016	1099.7520	4.47	POS	hilic				9.92E-01	7.54E-01	0.09	7.71E-01	-0.08	8.51E-01	-0.05	8.49E-01	-0.06
1099.8094@4.561136	1099.8094	4.56	NEG	hilic				7.24E-01	6.17E-01	0.15	5.41E-01	0.17	3.52E-01	-0.26	3.86E-01	0.24
1100.571@10.23343	1100.5710	10.23	NEG	RP				2.95E-02	4.93E-01	0.19	5.85E-02	-0.49	1.01E-01	-0.41	1.41E-01	0.41
1100.608@9.944541	1100.6080	9.94	POS	RP				8.01E-01	7.14E-01	-0.09	9.18E-01	0.03	5.74E-01	-0.17	1.59E-01	-0.38
1100.6357@1.1959431	1100.6357	1.20	POS	hilic				7.96E-01	4.71E-01	0.16	2.05E-01	0.34	9.35E-01	-0.02	8.95E-01	0.04
1100.648@10.521376	1100.6480	10.52	POS	RP				3.96E-02	1.68E-02	0.67	1.35E-01	-0.46	7.94E-01	-0.07	3.45E-01	0.30
1101.5334@1.342337	1101.5334	1.34	POS	hilic				6.58E-01	7.56E-01	-0.10	7.01E-01	-0.10	2.99E-01	-0.29	3.28E-01	0.28
1101.5446@1.1405184	1101.5446	1.14	POS	hilic				9.31E-01	5.00E-01	0.18	9.13E-01	0.03	7.99E-01	-0.06	6.89E-01	-0.11
1101.9615@6.2296653	1101.9615	6.23	NEG	hilic				3.70E-01	7.47E-02	0.55	2.39E-01	0.34	2.58E-01	0.30	2.02E-02	0.69
1101.9622@6.189129	1101.9622	6.19	POS	hilic				5.24E-01	1.40E-01	0.45	7.69E-01	-0.08	4.17E-01	0.24	1.16E-01	0.43
1102.2292@0.39406025	1102.2292	0.39	NEG	hilic				9.89E-01	7.87E-01	0.08	6.32E-01	0.14	9.94E-01	0.00	9.49E-01	-0.02
1102.5059@9.617863	1102.5059	9.62	POS	RP				1.49E-01	2.32E-01	0.38	5.74E-02	0.43	8.69E-02	0.43	7.56E-04	0.85
1102.6375@1.1944368	1102.6375	1.19	POS	hilic				4.99E-01	1.27E-01	-0.41	9.81E-01	0.01	4.60E-01	0.22	5.44E-01	-0.17
1102.6512@10.520332	1102.6512	10.52	POS	RP				5.47E-02	8.15E-02	0.51	6.52E-02	-0.56	2.15E-01	-0.30	7.34E-01	0.10
1102.6688@1.3914528	1102.6688	1.39	NEG	hilic				3.64E-02	5.55E-01	-0.16	4.25E-04	-0.80	1.49E-03	-0.71	2.12E-01	-0.37
1102.967@6.230453	1102.9670	6.23	NEG	hilic				3.00E-01	4.48E-02	0.56	2.69E-01	0.30	2.09E-01	0.29	2.01E-02	0.65
1103.2249@9.705376	1103.2249	9.71	NEG	RP				6.36E-01	4.59E-01	-0.20	1.51E-01	0.37	8.53E-01	-0.05	9.39E-01	-0.02
1103.225@9.547603	1103.2250	9.55	NEG	RP				6.89E-01	1.18E-01	0.48	8.85E-01	0.04	2.37E-01	0.33	4.79E-01	0.21
1103.2281@0.39406037	1103.2281	0.39	NEG	hilic				4.39E-01	2.25E-02	0.65	1.94E-01	0.38	1.54E-01	0.40	7.95E-01	0.08
1103.5547@1.3414034	1103.5547	1.34	POS	hilic				7.22E-01	3.92E-01	0.26	4.16E-01	-0.20	4.86E-01	0.17	7.15E-01	0.10
1103.555@1.1379911	1103.5550	1.14	POS	hilic				2.90E-02	2.16E-01	-0.36	9.12E-02	-0.48	1.18E-03	-0.86	4.77E-01	0.22
1103.559@10.015747	1103.5590	10.02	POS	RP				8.50E-01	1.84E-01	0.42	4.55E-01	0.23	5.35E-01	0.18	3.94E-01	0.25
1104.5593@1.1413078	1104.5593	1.14	POS	hilic				9.50E-01	9.91E-01	0.00	8.96E-01	0.03	4.06E-01	-0.21	8.65E-01	-0.05
1104.6569@10.011101	1104.6569	10.01	NEG	RP				6.08E-01	3.98E-01	0.26	4.90E-01	-0.22	3.84E-01	0.28	3.30E-01	0.29
1104.6573@10.117549	1104.6573	10.12	NEG	RP				1.96E-01	3.67E-01	0.33	1.85E-01	0.38	3.88E-02	0.53	1.67E-03	0.87
1104.6862@1.3901573	1104.6862	1.39	NEG	hilic				2.44E-01	4.59E-01	-0.22	4.94E-01	-0.17	2.51E-01	-0.26	1.40E-01	0.45

Supplementary table 2: Metabolomics analysis results

								ANOVA	Anorexia vs control		overweight vs control		obese vs control		athletes vs control	
Compound	Mass	Retention Time	Ionization mode	column	Compound_ID	MSMS_spectra	ID_level	P-FDR	P	Estimate	P	Estimate	P	Estimate	P	Estimate
1105.575@10.222875	1105.5750	10.22	POS	RP				4.34E-01	6.77E-01	-0.11	3.82E-01	0.24	6.27E-02	0.49	1.80E-01	0.32
1105.6934@1.3917032	1105.6934	1.39	NEG	hilic				1.51E-01	1.80E-01	0.36	1.92E-01	-0.33	1.98E-01	-0.34	3.00E-01	0.27
1108.1696@9.666856	1108.1696	9.67	NEG	RP				7.72E-01	6.64E-01	-0.15	7.70E-01	-0.09	4.65E-01	-0.16	7.71E-02	-0.47
1108.17@9.522179	1108.1700	9.52	NEG	RP				7.29E-01	1.55E-01	-0.41	2.90E-01	-0.32	6.14E-01	-0.13	1.68E-01	-0.43
1108.602@10.121102	1108.6020	10.12	NEG	RP				3.25E-01	7.41E-01	0.11	4.69E-01	0.21	2.35E-01	0.33	1.22E-02	0.75
1108.6215@11.030475	1108.6215	11.03	POS	RP				2.64E-01	1.53E-01	0.46	5.52E-02	0.53	7.90E-03	0.80	1.90E-01	0.41
1108.6451@1.1868068	1108.6451	1.19	POS	hilic				6.19E-01	3.60E-01	-0.25	8.83E-01	0.04	5.91E-01	-0.16	1.04E-01	-0.48
1108.6678@9.916512	1108.6678	9.92	NEG	RP				7.55E-01	3.77E-01	-0.23	9.61E-01	-0.01	2.34E-01	-0.42	3.21E-01	-0.30
1108.6696@1.3120092	1108.6696	1.31	NEG	hilic				7.92E-01	6.35E-01	0.13	3.06E-01	-0.30	3.66E-01	-0.24	6.51E-01	-0.15
1108.67@9.951744	1108.6700	9.95	NEG	RP				9.18E-01	4.71E-01	-0.21	5.50E-01	-0.20	9.23E-01	-0.03	7.67E-01	0.09
1108.6718@1.3121821	1108.6718	1.31	NEG	hilic				5.64E-01	9.34E-01	0.02	1.11E-01	-0.45	1.31E-01	-0.41	7.77E-01	-0.09
1108.685@11.036699	1108.6850	11.04	NEG	RP				7.30E-01	7.17E-01	0.10	3.55E-01	-0.21	5.45E-01	-0.14	1.85E-01	-0.29
1108.7365@10.702147	1108.7365	10.70	POS	RP				2.69E-01	4.51E-03	0.75	1.57E-01	0.39	4.50E-01	0.17	1.83E-01	0.38
1109.0162@0.5067105	1109.0162	0.51	NEG	hilic				2.36E-03	4.52E-03	0.86	3.68E-01	-0.15	3.65E-01	-0.16	7.32E-01	0.07
1109.0164@3.4466348	1109.0164	3.45	NEG	RP				5.19E-02	6.28E-02	0.74	4.46E-01	0.15	1.44E-01	-0.41	3.19E-01	0.25
1109.1718@9.522675	1109.1718	9.52	NEG	RP				2.87E-01	4.96E-02	-0.61	5.29E-02	-0.57	3.48E-01	-0.27	1.66E-02	-0.68
1109.5333@0.47848612	1109.5333	0.48	POS	hilic				8.76E-01	4.08E-01	0.22	9.66E-01	-0.01	2.89E-01	0.28	6.63E-01	0.12
1109.68@10.959403	1109.6800	10.96	POS	RP				4.86E-01	3.25E-01	0.29	6.66E-02	0.63	1.65E-01	0.38	5.99E-01	0.15
1109.7795@4.413982	1109.7795	4.41	POS	hilic				4.56E-01	7.12E-02	0.53	1.39E-01	0.39	7.66E-01	-0.09	6.87E-01	0.12
1110.6602@9.943972	1110.6602	9.94	POS	RP				3.34E-01	5.16E-02	-0.55	6.64E-01	-0.14	3.72E-01	0.27	3.86E-01	-0.24
1110.6613@1.1856467	1110.6613	1.19	POS	hilic				6.17E-01	4.88E-02	-0.60	5.05E-01	-0.19	3.52E-01	-0.28	2.48E-01	-0.34
1110.6844@1.3073275	1110.6844	1.31	NEG	hilic				7.79E-01	4.91E-01	0.20	3.43E-01	-0.30	8.36E-01	-0.05	8.99E-01	0.04
1110.7032@11.03709	1110.7032	11.04	NEG	RP				7.63E-01	1.88E-01	0.42	4.27E-01	0.23	4.57E-01	0.21	1.54E-01	0.42
1110.737@10.695798	1110.7370	10.70	POS	RP				2.58E-01	1.07E-01	0.56	2.61E-02	0.62	3.15E-01	0.25	1.94E-02	0.69
1110.9948@1.9134505	1110.9948	1.91	NEG	RP				2.03E-02	4.98E-01	0.18	2.87E-02	-0.58	1.04E-03	-0.85	6.01E-01	-0.16
1111.6696@9.944374	1111.6696	9.94	POS	RP				3.67E-01	9.30E-02	-0.45	8.02E-01	-0.07	4.78E-01	0.22	1.53E-01	-0.41
1111.7032@11.03676	1111.7032	11.04	NEG	RP				1.99E-01	1.60E-01	0.42	6.02E-01	0.12	1.97E-01	0.32	1.17E-01	-0.31
1112.1202@9.523087	1112.1202	9.52	NEG	RP				1.72E-01	1.96E-01	-0.36	3.95E-01	0.23	6.64E-02	0.46	3.72E-01	-0.29
1112.6755@1.1878452	1112.6755	1.19	POS	hilic				9.63E-01	8.03E-01	0.07	7.79E-01	-0.08	5.72E-01	-0.16	6.07E-01	-0.15
1112.7@1.3045363	1112.7000	1.30	NEG	hilic				6.41E-01	7.73E-01	0.09	1.44E-01	-0.43	4.72E-01	-0.19	8.77E-01	0.05
1112.7635@13.244412	1112.7635	13.24	POS	RP				2.08E-01	7.17E-01	0.10	2.17E-01	0.34	1.02E-01	-0.46	3.08E-01	-0.33
1113.6823@1.1861339	1113.6823	1.19	POS	hilic				9.84E-01	9.97E-01	0.00	5.59E-01	-0.16	8.53E-01	-0.05	7.10E-01	-0.10
1113.6971@11.041197	1113.6971	11.04	NEG	RP				7.22E-01	4.03E-01	0.26	5.21E-01	0.17	1.22E-01	0.45	1.84E-01	0.41
1113.7048@1.3049707	1113.7048	1.30	NEG	hilic				4.30E-01	4.52E-01	0.23	9.97E-02	-0.47	3.68E-01	-0.24	9.03E-01	-0.03
1114.2642@0.39177474	1114.2642	0.39	NEG	hilic				7.05E-01	2.24E-01	-0.32	6.17E-01	0.15	7.45E-01	-0.09	5.14E-01	0.22
1114.5734@9.928032	1114.5734	9.93	POS	RP				5.76E-01	2.72E-01	-0.29	9.68E-01	-0.01	1.72E-01	0.36	6.78E-01	-0.14
1114.581@9.94545	1114.5810	9.95	POS	RP				8.72E-01	9.06E-01	-0.03	6.72E-01	0.14	2.17E-01	0.35	6.58E-01	0.14
1114.596@10.001461	1114.5960	10.00	NEG	RP				1.85E-02	1.79E-01	0.42	6.22E-02	-0.45	4.80E-02	-0.47	2.03E-01	0.35
1114.6901@1.1838447	1114.6901	1.18	POS	hilic				3.57E-01	1.65E-01	0.40	5.37E-01	-0.15	2.26E-01	-0.28	9.10E-01	-0.03
1115.2675@0.39218175	1115.2675	0.39	NEG	hilic				5.76E-01	7.39E-01	0.10	8.23E-01	0.07	9.05E-01	-0.03	9.78E-02	0.54
1115.5767@9.940947	1115.5767	9.94	POS	RP				5.87E-02	8.00E-01	0.08	1.42E-02	0.78	4.93E-03	0.82	1.97E-01	0.39
1115.6934@1.1821208	1115.6934	1.18	POS	hilic				8.66E-01	5.86E-01	0.16	5.08E-01	-0.17	6.73E-01	-0.10	5.56E-01	-0.15
1116.5833@9.942623	1116.5833	9.94	POS	RP				4.02E-01	6.72E-01	0.14	6.90E-02	0.59	3.18E-02	0.58	2.46E-01	0.32
1118.2329@0.39590806	1118.2329	0.40	NEG	hilic				6.07E-01	5.21E-01	-0.19	1.50E-01	0.36	7.08E-01	0.11	8.30E-01	-0.06
1118.5844@9.944649	1118.5844	9.94	POS	RP				8.81E-01	5.52E-01	0.17	2.95E-01	0.36	3.65E-01	0.26	6.40E-01	0.12
1118.606@10.252182	1118.6060	10.25	POS	RP				2.52E-01	5.50E-02	0.63	6.51E-01	-0.12	4.26E-01	-0.19	9.70E-01	0.01
1118.7072@11.252926	1118.7072	11.25	NEG	RP				8.31E-01	9.04E-01	-0.03	3.33E-01	-0.30	6.32E-01	0.13	8.13E-01	-0.07
1120.1582@9.523436	1120.1582	9.52	NEG	RP				6.51E-01	2.17E-01	-0.35	3.92E-01	-0.27	7.67E-01	0.08	1.82E-01	-0.40
1120.5446@9.971024	1120.5446	9.97	NEG	RP				4.10E-01	4.52E-01	0.19	2.11E-01	0.29	7.06E-02	0.44	6.21E-01	-0.09
1120.622@10.132577	1120.6220	10.13	NEG	RP				8.31E-01	4.94E-01	-0.17	6.94E-01	-0.10	4.87E-01	0.20	8.41E-01	0.05
1120.7205@11.252455	1120.7205	11.25	NEG	RP				9.83E-01	5.70E-01	-0.17	7.82E-01	-0.08	8.75E-01	0.05	9.87E-01	0.01
1121.2686@9.692838	1121.2686	9.69	POS	RP				2.84E-01	2.15E-01	-0.34	8.72E-02	0.51	9.25E-01	0.03	9.55E-01	-0.02
1121.269@9.564933	1121.2690	9.56	POS	RP				6.96E-01	3.47E-01	-0.27	6.60E-01	0.13	6.98E-01	-0.11	2.17E-01	-0.38
1121.5854@9.950988	1121.5854	9.95	POS	RP				4.41E-01	1.38E-01	0.38	1.18E-01	0.55	2.78E-02	0.63	3.11E-01	0.28
1121.7242@11.252821	1121.7242	11.25	NEG	RP				6.60E-01	2.36E-01	-0.36	8.88E-01	-0.05	3.36E-01	0.26	7.55E-01	-0.09
1122.1501@9.523281	1122.1501	9.52	NEG	RP				8.75E-01	9.06E-01	-0.04	2.45E-01	-0.32	7.74E-01	-0.06	4.58E-01	-0.26
1122.1519@9.692559	1122.1519	9.69	NEG	RP				2.98E-02	8.55E-01	-0.05	6.30E-03	0.85	5.46E-02	0.53	7.85E-01	-0.07
1122.2183@0.3927464	1122.2183	0.39	NEG	hilic				7.10E-01	7.58E-01	-0.08	8.93E-01	0.04	3.90E-01	-0.21	3.45E-01	0.28
1122.6151@10.124974	1122.6151	10.12	NEG	RP				2.91E-01	8.79E-02	-0.52	6.27E-01	-0.13	2.02E-01	0.36	8.33E-01	-0.06
1122.6158@10.074675	1122.6158	10.07	NEG	RP				2.86E-01	9.40E-02	-0.47	9.32E-01	0.03	6.21E-01	0.15	1.19E-01	-0.52
1122.638@10.527324	1122.6380	10.53	POS	RP				1.15E-01	2.76E-02	0.60	2.75E-01	-0.34	9.84E-01	0.01	2.21E-01	0.39
1122.6425@10.5350485	1122.6425	10.54	POS	RP				2.57E-01	2.60E-02	0.61	6.97E-01	-0.13	4.40E-01	0.21	1.16E-01	0.49
1123.0319@4.7011056	1123.0319	4.70	NEG	RP				6.94E-01	2.25E-01	0.24	3.51E-01	0.19	6.03E-01	-0.06	4.77E-01	0.12
1123.1523@9.524486	1123.1523	9.52	NEG	RP				4.74E-01	1.68E-01	-0.49	8.17E-01	-0.07	9.02E-01	-0.03	9.89E-02	-0.51
1123.154@9.697433	1123.1540	9.70	NEG	RP				6.70E-01	4.99E-01	0.17	1.25E-01	0.51	3.26E-01	0.29	7.74E-01	0.08
1123.6154@10.2380295	1123.6154	10.24	POS	RP				7.34E-01	2.11E-01	0.35	7.94E-01	0.05	9.23E-01	0.02	3.95E-01	0.19
1123.6191@10.125642	1123.6191	10.13	NEG	RP				8.48E-01	4.02E-01	-0.26	6.48E-01	-0.14	5.37E-01	0.18	7.66E-01	-0.08
1123.6215@10.020479	1123.6215	10.02	NEG	RP				9.88E-01	7.24E-01	-0.10	8.40E-01	0.07	9.46E-01	-0.02	6.73E-01	-0.12
1123.943@6.1915717	1123.9430	6.19	POS	hilic				5.59E-01	2.59E-01	-0.33	1.06E-01	-0.46	8.37E-01	0.06	8.72E-01	-0.05
1124.6447@10.523516	1124.6447	10.52	POS	RP				2.39E-02	2.00E-01	0.37	1.50E-02	-0.75	9.56E-02	-0.41	9.06E-01	0.03
1124.8376@0.6607936	1124.8376	0.66	POS	hilic				5.42E-01	8.41E-01	0.06	1.04E-01	0.40	7.51E-01	-0.07	2.41E-01	0.30
1124.9492@6.231239	1124.9492	6.23	NEG	hilic				5.63E-01	1.08E-01	0.49	4.23E-01	0.22	2.68E-01	0.26	6.75E-02	0.49
1125.0107@3.040615	1125.0107	3.04	NEG	RP				6.67E-01	7.50E-01	0.07	1.02E-01	-0.29	9.85E-01	0.00	6.52E-01	0.11

Supplementary table 2: Metabolomics analysis results

								ANOVA	Anorexia vs control		overweight vs control		obese vs control		athletes vs control	
Compound	Mass	Retention Time	Ionization mode	column	Compound_ID	MSMS_spectra	ID_level	P-FDR	P	Estimate	P	Estimate	P	Estimate	P	Estimate
1125.5424@0.52538896	1125.5424	0.53	POS	hilic				7.24E-01	9.75E-01	0.00	1.86E-01	0.23	2.95E-01	0.18	2.92E-01	0.20
1125.6158@10.237748	1125.6158	10.24	POS	RP				6.35E-03	9.35E-02	0.53	1.08E-01	-0.43	8.07E-03	-0.69	4.71E-01	0.20
1126.2365@0.39844435	1126.2365	0.40	NEG	hilic				6.14E-01	3.24E-01	0.31	4.23E-02	0.58	3.06E-01	0.28	3.25E-01	0.32
1126.779@10.960187	1126.7790	10.96	POS	RP				2.00E-01	6.59E-01	0.11	3.54E-02	0.49	1.02E-01	0.40	5.17E-01	-0.14
1127.5522@1.1387341	1127.5522	1.14	POS	hilic				5.66E-01	4.34E-01	-0.22	5.82E-01	0.15	1.20E-01	-0.36	4.61E-01	-0.18
1127.554@9.863302	1127.5540	9.86	POS	RP				7.76E-01	4.08E-01	0.20	6.40E-01	-0.16	4.16E-01	-0.24	6.41E-01	0.13
1128.1252@6.27001	1128.1252	6.27	POS	hilic				4.99E-01	3.93E-01	-0.28	1.25E-01	-0.45	3.16E-02	-0.54	7.86E-01	-0.07
1128.2452@9.425495	1128.2452	9.43	NEG	RP				9.32E-01	5.75E-01	0.16	9.45E-01	-0.02	6.79E-01	0.12	3.67E-01	0.27
1128.2487@0.39836925	1128.2487	0.40	NEG	hilic				7.58E-01	2.65E-01	0.35	1.84E-01	0.40	5.12E-01	0.19	1.79E-01	0.40
1128.5557@9.846351	1128.5557	9.85	POS	RP				8.55E-03	8.67E-01	0.05	5.85E-02	-0.47	8.70E-04	-0.83	4.22E-01	0.24
1128.6556@1.2367016	1128.6556	1.24	NEG	hilic				3.15E-03	7.39E-01	0.08	1.57E-02	-0.57	5.63E-04	-0.79	5.20E-01	0.19
1128.6556@9.996557	1128.6556	10.00	NEG	RP				1.02E-02	2.13E-01	0.33	9.66E-02	-0.49	1.24E-02	-0.63	1.62E-01	0.42
1128.7924@10.951915	1128.7924	10.95	POS	RP				7.52E-02	9.82E-01	0.00	1.21E-02	0.67	1.35E-02	0.57	5.75E-01	0.14
1129.2515@0.39703438	1129.2515	0.40	NEG	hilic				5.97E-01	4.04E-01	0.26	6.76E-02	0.53	3.29E-01	0.27	1.40E-01	0.49
1129.4504@3.0321026	1129.4504	3.03	POS	hilic				6.87E-01	1.60E-01	0.44	9.33E-02	0.45	2.82E-01	0.34	1.73E-01	0.41
1129.571@1.1281024	1129.5710	1.13	POS	hilic				6.71E-01	2.86E-01	0.33	6.39E-01	-0.12	4.25E-01	-0.18	8.80E-01	0.04
1129.5891@10.256403	1129.5891	10.26	POS	RP				6.21E-01	3.46E-01	0.28	4.84E-01	-0.15	3.31E-01	0.25	3.60E-01	0.24
1129.6497@10.515409	1129.6497	10.52	POS	RP				2.97E-02	5.50E-02	0.60	6.98E-02	-0.55	3.99E-01	-0.22	5.55E-01	0.17
1130.675@10.208277	1130.6750	10.21	NEG	RP				8.43E-02	5.28E-01	0.19	1.47E-02	-0.71	9.41E-02	-0.42	9.05E-01	0.03
1130.6866@1.2921019	1130.6866	1.29	NEG	hilic				7.64E-01	5.15E-01	-0.17	3.75E-01	-0.22	3.07E-01	-0.26	9.93E-02	-0.39
1131.45@3.0400524	1131.4500	3.04	POS	hilic				8.34E-01	2.19E-01	0.43	5.03E-01	0.18	7.43E-01	0.10	8.20E-01	0.06
1131.5782@1.1197712	1131.5782	1.12	POS	hilic				5.82E-01	7.99E-01	0.07	7.23E-01	0.10	3.17E-01	-0.29	1.72E-01	-0.39
1131.5844@10.258283	1131.5844	10.26	POS	RP				2.85E-01	4.47E-02	0.65	4.23E-01	0.25	1.25E-01	0.42	1.07E-02	0.70
1131.591@1.2126889	1131.5910	1.21	POS	hilic				4.42E-01	4.85E-01	-0.19	1.79E-01	0.40	2.85E-01	0.30	7.46E-01	-0.08
1132.2275@0.39644334	1132.2275	0.40	NEG	hilic				8.92E-01	7.90E-01	-0.09	3.56E-01	0.25	7.50E-01	-0.09	8.38E-01	0.06
1132.5883@10.25656	1132.5883	10.26	POS	RP				3.17E-01	1.63E-02	0.75	8.92E-01	0.04	4.68E-01	0.18	3.39E-01	0.29
1132.5996@9.999736	1132.5996	10.00	NEG	RP				7.40E-02	5.17E-01	0.17	2.62E-01	-0.27	1.45E-03	-0.66	8.21E-01	-0.06
1132.669@9.935239	1132.6690	9.94	NEG	RP				2.43E-01	1.28E-01	0.44	1.88E-01	0.39	1.51E-02	0.83	1.06E-01	0.50
1132.67@9.945308	1132.6700	9.95	NEG	RP				6.24E-01	7.79E-02	-0.53	4.81E-01	-0.22	8.84E-01	0.04	5.73E-01	-0.16
1132.6794@1.2420133	1132.6794	1.24	NEG	hilic				2.81E-01	1.96E-01	-0.38	4.86E-02	-0.49	3.96E-03	-0.68	4.81E-01	-0.22
1133.5944@1.1804999	1133.5944	1.18	POS	hilic				9.11E-01	9.33E-01	0.02	7.19E-01	0.10	7.10E-01	0.11	4.59E-01	-0.20
1133.8033@4.5577216	1133.8033	4.56	NEG	hilic				2.64E-01	1.02E-02	0.75	7.75E-02	0.51	3.01E-01	0.26	6.12E-02	0.59
1134.0114@2.3786662	1134.0114	2.38	NEG	RP				3.66E-01	1.31E-01	0.56	9.83E-01	-0.01	3.81E-01	-0.23	4.96E-01	0.19
1134.6846@1.2948333	1134.6846	1.29	NEG	hilic				4.28E-01	8.65E-01	-0.05	4.01E-02	-0.42	6.50E-02	-0.41	3.57E-01	-0.23
1134.7498@10.03349	1134.7498	10.03	POS	RP				5.60E-01	4.53E-01	0.20	4.19E-01	-0.17	1.92E-01	0.30	8.99E-01	-0.03
1135.0323@5.3187413	1135.0323	5.32	NEG	RP				6.11E-03	3.41E-02	0.54	4.11E-01	-0.18	9.49E-03	-0.47	4.17E-01	-0.18
1135.5887@10.1191225	1135.5887	10.12	NEG	RP				5.13E-01	3.49E-01	0.32	3.24E-01	0.31	2.48E-01	0.29	1.84E-02	0.67
1135.5905@10.052689	1135.5905	10.05	NEG	RP				4.57E-01	4.88E-02	-0.59	8.58E-01	0.05	2.76E-01	-0.27	2.87E-01	-0.34
1135.6158@10.5315075	1135.6158	10.53	POS	RP				2.44E-02	5.77E-03	0.78	2.32E-01	-0.36	4.88E-01	0.18	1.20E-01	0.48
1135.7322@11.252899	1135.7322	11.25	POS	RP				8.81E-01	5.95E-01	-0.18	8.65E-01	-0.05	4.17E-01	0.23	8.26E-01	0.07
1135.7513@0.4001143	1135.7513	0.40	POS	hilic				7.20E-01	2.15E-01	0.28	1.68E-01	0.27	2.01E-01	0.32	6.80E-01	0.08
1136.6215@10.528989	1136.6215	10.53	POS	RP				1.66E-02	4.17E-03	0.89	8.47E-01	-0.05	5.42E-02	0.48	9.66E-03	0.79
1137.502@3.222894	1137.5020	3.22	NEG	hilic				4.53E-01	1.97E-02	0.71	4.70E-01	0.22	5.23E-01	0.17	5.83E-01	0.15
1137.5024@3.188622	1137.5024	3.19	NEG	hilic				8.62E-01	2.88E-01	0.32	9.92E-01	0.00	5.47E-01	0.15	3.48E-01	0.27
1137.7476@11.252976	1137.7476	11.25	POS	RP				8.68E-01	7.97E-01	-0.08	7.38E-01	0.10	3.13E-01	0.31	5.73E-01	0.18
1137.7502@0.39246154	1137.7502	0.39	POS	hilic				7.90E-01	4.25E-01	-0.22	1.84E-01	-0.35	3.34E-01	-0.26	2.37E-01	-0.32
1138.2126@0.39286742	1138.2126	0.39	NEG	hilic				8.96E-01	3.82E-01	0.24	6.54E-01	0.14	3.79E-01	0.25	8.79E-01	-0.04
1138.5948@9.959066	1138.5948	9.96	NEG	RP				3.22E-02	2.01E-01	0.39	2.77E-01	-0.30	2.16E-01	-0.27	1.77E-02	0.67
1138.7513@11.253319	1138.7513	11.25	POS	RP				6.89E-01	5.56E-01	-0.20	9.07E-01	0.03	1.69E-01	0.39	9.24E-01	0.03
1139.003@3.1992962	1139.0030	3.20	NEG	hilic				8.51E-01	9.90E-01	0.00	4.52E-01	-0.21	9.11E-01	0.03	4.86E-01	0.22
1139.534@1.1418573	1139.5340	1.14	POS	hilic				6.97E-01	5.82E-01	-0.12	2.77E-01	0.27	9.17E-01	-0.02	7.68E-01	-0.06
1139.5508@0.51509243	1139.5508	0.52	POS	hilic				8.41E-01	1.88E-01	-0.39	5.07E-01	-0.20	9.44E-01	-0.02	6.08E-01	-0.15
1139.58@9.935786	1139.5800	9.94	POS	RP				2.87E-02	1.33E-01	-0.42	4.39E-02	0.56	6.71E-02	0.53	6.85E-01	-0.12
1140.2188@9.571982	1140.2188	9.57	NEG	RP				9.51E-01	3.66E-01	0.28	6.99E-01	0.12	9.24E-01	0.03	8.76E-01	0.05
1140.2216@0.38932484	1140.2216	0.39	NEG	hilic				9.82E-01	4.49E-01	0.22	7.21E-01	0.11	7.75E-01	0.08	8.64E-01	0.06
1141.226@0.38757658	1141.2260	0.39	NEG	hilic				9.18E-01	6.25E-01	0.15	6.46E-01	-0.15	5.52E-01	-0.18	6.67E-01	-0.12
1141.5507@0.51390165	1141.5507	0.51	POS	hilic				8.18E-01	6.86E-01	-0.14	3.34E-01	0.29	7.94E-01	-0.08	7.36E-01	0.10
1142.612@10.240979	1142.6120	10.24	POS	RP				3.25E-03	4.45E-02	0.60	3.99E-02	-0.60	1.49E-02	-0.61	7.69E-01	0.08
1142.62@10.231205	1142.6200	10.23	NEG	RP				7.10E-01	3.37E-01	0.28	9.21E-01	-0.02	3.72E-01	-0.19	5.30E-01	0.18
1142.7028@11.25143	1142.7028	11.25	POS	RP				3.23E-01	6.50E-03	-0.78	2.30E-01	-0.36	4.57E-01	-0.22	1.11E-01	-0.48
1142.7343@0.37372452	1142.7343	0.37	NEG	hilic				9.84E-01	9.29E-01	0.03	6.70E-01	0.14	8.10E-01	-0.07	9.52E-01	-0.02
1143.5629@0.5147732	1143.5629	0.51	POS	hilic				6.46E-01	7.30E-01	-0.09	9.56E-02	-0.45	9.28E-01	-0.03	3.40E-01	-0.25
1144.5706@0.51925	1144.5706	0.52	POS	hilic				6.87E-01	9.33E-01	0.02	2.17E-01	0.28	2.08E-01	0.33	2.21E-01	0.32
1144.6357@1.3513479	1144.6357	1.35	NEG	hilic				7.41E-01	5.78E-01	-0.15	9.12E-01	0.03	1.46E-01	-0.33	3.49E-01	-0.23
1146.2476@0.39394584	1146.2476	0.39	NEG	hilic				7.48E-01	7.82E-01	0.07	2.44E-01	0.35	4.24E-01	0.23	1.48E-01	0.44
1146.6165@9.997496	1146.6165	10.00	NEG	RP				4.35E-01	7.83E-01	0.09	3.87E-01	-0.25	3.78E-02	-0.56	9.65E-01	0.01
1146.6497@10.51993	1146.6497	10.52	POS	RP				2.53E-02	3.20E-02	0.64	6.10E-02	-0.57	5.39E-01	-0.16	6.17E-01	0.15
1146.6532@10.5027685	1146.6532	10.50	NEG	RP				2.26E-02	1.13E-02	0.82	2.30E-01	-0.32	5.59E-01	-0.15	3.98E-01	0.26
1146.6545@1.3307356	1146.6545	1.33	NEG	hilic				6.09E-01	9.99E-01	0.00	7.40E-02	-0.51	7.76E-01	-0.08	5.75E-01	-0.17
1147.6173@9.9987335	1147.6173	10.00	NEG	RP				2.32E-01	9.78E-01	0.01	1.54E-01	-0.37	9.49E-02	-0.38	2.94E-01	0.34
1148.2332@0.38930637	1148.2332	0.39	NEG	hilic				8.00E-01	9.04E-01	0.03	2.96E-01	0.33	9.32E-01	0.02	3.04E-01	0.33
1148.4608@0.54935855	1148.4608	0.55	NEG	hilic				2.18E-01	1.08E-01	-0.42	1.49E-01	0.37	3.61E-01	0.28	6.89E-01	-0.10
1148.6335@10.211729	1148.6335	10.21	NEG	RP				3.74E-03	1.07E-03	1.06	6.24E-01	-0.15	5.71E-01	-0.15	7.78E-02	0.45

Supplementary table 2: Metabolomics analysis results

Compound	Mass	Retention Time	Ionization mode	column	Compound_ID	MSMS_spectra	ID_level	ANOVA P-FDR	Anorexia vs control P	Anorexia vs control Estimate	overweight vs control P	overweight vs control Estimate	obese vs control P	obese vs control Estimate	athletes vs control P	athletes vs control Estimate
1149.9467@10.711108	1149.9467	10.71	NEG	RP				4.37E-01	1.13E-01	0.53	9.75E-02	0.41	1.96E-02	0.60	4.70E-01	0.21
1150.1827@9.266306	1150.1827	9.27	NEG	RP				9.95E-01	9.50E-01	-0.02	6.26E-01	-0.14	7.70E-01	-0.08	9.23E-01	-0.02
1150.2452@9.436867	1150.2452	9.44	NEG	RP				5.19E-01	1.80E-01	-0.42	2.13E-01	-0.36	8.34E-01	-0.06	9.97E-02	-0.53
1150.2458@9.57082	1150.2458	9.57	NEG	RP				6.41E-01	4.88E-01	-0.19	3.00E-01	0.33	3.10E-01	0.28	8.60E-01	-0.05
1150.2487@0.38931632	1150.2487	0.39	NEG	hilic				5.96E-01	6.41E-01	0.12	7.57E-02	0.55	7.10E-01	0.11	3.11E-01	0.34
1150.4716@0.55718654	1150.4716	0.56	NEG	hilic				5.56E-01	5.04E-02	-0.47	8.12E-01	0.06	9.31E-01	0.03	8.67E-01	0.04
1150.6816@10.506642	1150.6816	10.51	NEG	RP				3.36E-01	2.38E-01	0.38	1.25E-01	-0.43	7.16E-01	-0.09	7.36E-01	0.11
1150.7253@0.86533326	1150.7253	0.87	POS	hilic				8.20E-01	6.07E-01	0.14	4.44E-01	-0.19	5.00E-01	0.18	9.87E-01	0.00
1151.252@0.3896667	1151.2520	0.39	NEG	hilic				7.99E-01	6.18E-01	0.14	2.72E-01	0.34	8.08E-01	0.07	2.36E-01	0.38
1151.5577@1.1676518	1151.5577	1.17	POS	hilic				2.07E-01	8.87E-03	-0.64	8.73E-01	0.05	9.78E-01	-0.01	1.62E-01	-0.34
1151.6844@10.507137	1151.6844	10.51	NEG	RP				2.18E-01	7.37E-01	0.10	2.00E-02	-0.47	6.70E-01	0.09	4.81E-01	0.20
1152.6569@9.960571	1152.6569	9.96	NEG	RP				4.63E-03	9.54E-01	-0.02	1.31E-02	-0.76	6.45E-04	-0.91	5.77E-01	0.16
1152.6584@1.3465812	1152.6584	1.35	NEG	hilic				1.78E-01	9.72E-01	0.01	1.68E-01	-0.47	4.30E-03	-0.78	2.48E-01	-0.35
1152.6584@9.896829	1152.6584	9.90	NEG	RP				9.61E-01	4.24E-01	-0.23	6.61E-01	-0.13	9.70E-01	0.01	8.61E-01	-0.05
1152.6602@1.2337362	1152.6602	1.23	NEG	hilic				5.47E-02	4.85E-01	-0.18	1.65E-02	-0.55	9.34E-03	-0.58	5.98E-01	0.15
1153.555@0.51604766	1153.5550	0.52	POS	hilic				5.90E-01	7.82E-01	0.07	6.33E-01	0.12	9.17E-01	-0.03	7.30E-02	0.50
1153.5629@1.1854665	1153.5629	1.19	POS	hilic				3.73E-03	6.41E-04	-0.81	6.56E-01	-0.12	5.92E-01	0.15	8.39E-04	-0.92
1154.2238@0.3875812	1154.2238	0.39	NEG	hilic				5.07E-01	4.05E-01	-0.24	2.83E-02	-0.58	2.33E-01	-0.33	1.25E-01	-0.52
1154.5413@10.177548	1154.5413	10.18	POS	RP				1.65E-01	1.71E-01	0.40	9.22E-01	-0.02	5.48E-02	-0.44	4.40E-01	0.21
1154.6685@1.3397715	1154.6685	1.34	NEG	hilic				1.56E-01	6.74E-01	-0.13	1.57E-02	-0.72	2.46E-02	-0.57	8.73E-01	-0.05
1155.5645@0.51673806	1155.5645	0.52	POS	hilic				7.82E-01	1.92E-01	-0.38	8.86E-01	-0.03	3.86E-01	-0.21	9.62E-01	0.02
1155.581@1.1895775	1155.5810	1.19	POS	hilic				2.97E-02	6.19E-02	-0.47	7.52E-02	0.48	1.22E-01	0.48	4.28E-01	-0.21
1156.5468@10.174321	1156.5468	10.17	POS	RP				7.09E-01	3.49E-01	0.29	1.78E-01	0.35	2.10E-01	0.35	9.01E-01	-0.04
1156.6042@9.955171	1156.6042	9.96	NEG	RP				2.09E-02	6.42E-01	0.15	1.38E-02	-0.63	1.71E-01	-0.35	9.85E-02	0.47
1156.667@9.941339	1156.6670	9.94	NEG	RP				9.34E-02	1.19E-02	-0.55	9.27E-01	-0.02	1.82E-01	0.36	4.44E-01	-0.17
1156.6862@1.3295084	1156.6862	1.33	NEG	hilic				7.66E-03	3.41E-02	0.69	3.31E-02	-0.60	1.62E-01	-0.36	4.14E-01	-0.22
1156.6884@10.23087	1156.6884	10.23	NEG	RP				1.06E-02	3.63E-02	0.66	1.31E-01	-0.46	3.15E-02	-0.53	7.83E-01	0.07
1156.7393@10.562483	1156.7393	10.56	POS	RP				2.34E-02	3.64E-02	-0.60	1.12E-01	0.47	4.70E-02	0.47	9.41E-01	-0.02
1158.1667@9.548338	1158.1667	9.55	NEG	RP				7.31E-01	6.00E-01	0.15	2.85E-01	0.33	4.34E-01	0.23	5.61E-01	-0.16
1158.5844@0.50909597	1158.5844	0.51	POS	hilic				9.51E-01	6.72E-01	0.12	8.68E-01	0.04	5.63E-01	0.14	6.86E-01	-0.10
1158.7032@1.3246144	1158.7032	1.32	NEG	hilic				2.80E-02	1.19E-01	0.52	1.07E-02	-0.67	4.21E-01	-0.22	3.49E-01	-0.26
1158.7039@1.3246144	1158.7039	1.32	NEG	hilic				1.56E-02	6.94E-02	0.60	9.58E-03	-0.65	5.43E-01	-0.17	3.37E-01	-0.26
1158.7375@10.562254	1158.7375	10.56	POS	RP				3.73E-02	7.20E-02	-0.53	9.90E-02	0.50	5.05E-02	0.50	8.39E-01	0.06
1159.564@0.5724792	1159.5640	0.57	POS	hilic				6.00E-01	8.79E-01	-0.04	3.82E-02	-0.43	4.45E-01	-0.18	9.19E-01	-0.03
1159.5875@9.998703	1159.5875	10.00	NEG	RP				1.68E-02	1.14E-02	0.62	1.41E-01	-0.47	3.94E-01	-0.21	9.78E-02	0.42
1159.7065@1.3177155	1159.7065	1.32	NEG	hilic				1.83E-01	1.41E-01	0.50	1.28E-01	-0.41	4.02E-01	0.24	7.46E-01	0.09
1159.7415@10.563012	1159.7415	10.56	POS	RP				2.90E-02	8.96E-02	-0.46	7.00E-02	0.54	2.57E-02	0.56	8.28E-01	0.06
1160.6274@10.230999	1160.6274	10.23	NEG	RP				8.98E-01	9.23E-01	0.03	9.27E-01	-0.02	7.26E-01	0.08	3.65E-01	-0.22
1160.7173@1.3102996	1160.7173	1.31	NEG	hilic				2.22E-01	1.54E-01	0.46	2.57E-01	-0.27	1.84E-01	0.40	4.99E-01	0.17
1160.7192@10.503767	1160.7192	10.50	NEG	RP				1.72E-02	1.87E-02	0.72	9.96E-02	-0.49	4.32E-01	-0.21	4.39E-01	0.23
1163.2299@0.38653842	1163.2299	0.39	NEG	hilic				6.60E-01	2.82E-01	0.31	5.22E-01	-0.19	5.71E-01	0.16	5.23E-01	-0.20
1163.5367@1.1265998	1163.5367	1.13	POS	hilic				6.67E-01	1.37E-01	-0.37	8.03E-01	0.07	3.31E-01	-0.26	6.06E-01	-0.12
1164.664@10.50637	1164.6640	10.51	NEG	RP				3.86E-01	3.75E-01	0.30	1.67E-01	-0.44	4.90E-01	-0.17	6.68E-01	0.13
1164.7413@0.6172445	1164.7413	0.62	POS	hilic				7.56E-01	5.33E-01	-0.17	2.54E-01	-0.29	1.60E-01	-0.34	2.52E-01	-0.28
1165.5521@1.11992	1165.5521	1.12	POS	hilic				6.50E-01	4.90E-01	-0.18	6.46E-01	0.12	1.33E-01	-0.31	3.89E-01	-0.20
1166.2404@0.39255455	1166.2404	0.39	NEG	hilic				6.74E-01	3.34E-01	0.30	5.42E-01	0.18	9.75E-01	-0.01	1.26E-01	0.48
1166.6255@10.114653	1166.6255	10.11	NEG	RP				1.07E-01	1.04E-03	1.00	6.80E-02	0.50	1.25E-01	0.37	1.85E-01	0.43
1167.237@9.724529	1167.2370	9.72	NEG	RP				3.24E-01	1.78E-01	0.44	6.19E-02	0.52	3.18E-02	0.52	9.62E-01	-0.01
1167.2393@9.572182	1167.2393	9.57	NEG	RP				2.36E-01	8.88E-01	-0.04	8.40E-02	-0.47	8.18E-01	-0.06	2.05E-01	0.38
1167.2408@0.39241377	1167.2408	0.39	NEG	hilic				8.24E-01	3.44E-01	0.27	2.92E-01	0.29	7.58E-01	0.09	2.87E-01	0.39
1167.5604@1.1104641	1167.5604	1.11	POS	hilic				6.60E-01	8.57E-01	-0.05	7.14E-01	0.09	7.49E-02	-0.40	8.19E-01	-0.06
1167.7396@4.469331	1167.7396	4.47	POS	hilic				9.01E-01	3.22E-01	0.30	8.12E-01	-0.07	7.25E-01	0.09	9.63E-01	0.01
1167.794@4.5572305	1167.7940	4.56	NEG	hilic				7.17E-01	5.64E-01	0.16	2.86E-01	0.32	4.04E-01	-0.21	8.00E-01	0.08
1168.6143@9.940015	1168.6143	9.94	NEG	RP				2.93E-01	6.36E-01	-0.14	2.48E-02	-0.52	4.56E-02	-0.48	9.71E-01	0.01
1169.5736@1.0960443	1169.5736	1.10	POS	hilic				8.10E-01	4.21E-01	0.27	6.18E-01	-0.14	5.64E-01	-0.14	8.02E-01	-0.07
1169.5739@0.52038664	1169.5739	0.52	POS	hilic				8.01E-01	3.08E-01	0.26	4.16E-01	0.21	1.09E-01	0.41	5.59E-01	0.18
1170.4781@1.4727856	1170.4781	1.47	NEG	hilic				2.18E-01	5.14E-02	0.56	3.25E-01	-0.19	4.57E-01	0.19	3.73E-01	0.23
1170.5833@1.0931035	1170.5833	1.09	POS	hilic				7.82E-01	7.39E-01	0.10	6.58E-01	-0.12	3.16E-01	-0.25	2.92E-01	-0.25
1170.6143@9.92094	1170.6143	9.92	NEG	RP				7.66E-01	7.36E-01	0.10	2.16E-01	-0.33	8.01E-01	-0.08	3.89E-01	-0.28
1170.6147@9.951499	1170.6147	9.95	NEG	RP				9.00E-02	1.46E-01	-0.39	1.25E-02	-0.72	1.27E-02	-0.75	8.06E-01	-0.08
1171.617@9.949767	1171.6170	9.95	NEG	RP				4.58E-01	5.15E-01	-0.16	2.25E-01	-0.37	1.67E-02	-0.60	9.92E-01	0.00
1172.1812@9.549009	1172.1812	9.55	NEG	RP				4.79E-01	3.88E-02	-0.65	2.69E-01	-0.35	2.43E-01	-0.31	9.07E-02	-0.52
1172.1816@9.688766	1172.1816	9.69	NEG	RP				6.03E-01	6.56E-01	0.13	4.59E-01	0.24	1.69E-01	0.38	4.37E-01	-0.21
1172.4294@3.0476263	1172.4294	3.05	POS	hilic				6.40E-01	2.39E-01	0.38	9.97E-01	0.00	4.00E-01	0.24	7.29E-02	0.44
1172.6486@10.233171	1172.6486	10.23	NEG	RP				5.67E-02	1.11E-01	0.46	1.27E-01	-0.51	7.49E-02	-0.47	7.96E-01	0.07
1173.1844@9.5483	1173.1844	9.55	NEG	RP				5.99E-01	6.20E-02	-0.50	1.67E-01	-0.40	7.31E-01	-0.11	1.54E-01	-0.43
1173.1855@9.6939	1173.1855	9.69	NEG	RP				5.67E-01	1.21E-01	-0.46	7.75E-01	-0.10	7.72E-01	0.08	1.70E-01	-0.38
1174.2404@0.37280732	1174.2404	0.37	NEG	hilic				8.43E-01	5.18E-01	-0.21	9.86E-01	-0.01	4.12E-01	-0.22	2.13E-01	-0.35
1174.6469@10.231761	1174.6469	10.23	NEG	RP				2.85E-02	1.23E-01	0.46	9.45E-02	-0.55	1.78E-02	-0.61	7.06E-01	-0.10
1174.7646@10.592935	1174.7646	10.59	NEG	hilic				5.09E-01	2.04E-01	-0.43	1.73E-01	-0.37	1.30E-01	-0.39	2.80E-02	-0.60
1174.7653@0.40218174	1174.7653	0.40	NEG	hilic				8.01E-01	4.94E-01	0.18	5.48E-01	-0.13	5.55E-01	0.17	6.59E-01	-0.10
1174.7668@11.786334	1174.7668	11.79	NEG	RP				7.96E-01	1.46E-01	-0.43	5.18E-01	-0.19	9.91E-01	0.00	6.82E-01	-0.13
1175.6486@10.232322	1175.6486	10.23	NEG	RP				2.38E-01	1.91E-01	0.46	6.71E-01	-0.13	1.50E-01	-0.39	4.38E-01	0.22

Supplementary table 2: Metabolomics analysis results

Compound	Mass	Retention Time	Ionization mode	column	Compound_ID	MSMS_spectra	ID_level	ANOVA	Anorexia vs control		overweight vs control		obese vs control		athletes vs control	
								P-FDR	P	Estimate	P	Estimate	P	Estimate	P	Estimate
1175.7806@4.573607	1175.7806	4.57	NEG	hilic				6.67E-02	7.14E-02	-0.44	3.37E-02	0.54	3.45E-01	0.26	9.21E-01	-0.03
1176.1313@9.549344	1176.1313	9.55	NEG	RP				4.97E-01	4.06E-01	-0.27	8.19E-01	-0.07	2.29E-01	-0.34	4.92E-02	-0.62
1176.6553@9.9450445	1176.6553	9.95	NEG	RP				3.97E-02	3.03E-01	-0.30	1.84E-01	0.42	4.91E-03	0.77	9.55E-01	0.02
1176.6628@1.3041687	1176.6628	1.30	NEG	hilic				6.60E-01	1.79E-01	-0.41	7.00E-01	-0.12	4.73E-01	0.19	8.65E-01	-0.05
1176.6805@10.50598	1176.6805	10.51	NEG	RP				4.76E-02	5.26E-02	0.58	6.09E-02	-0.56	5.13E-01	-0.18	8.86E-01	0.04
1176.7819@11.832454	1176.7819	11.83	NEG	RP				7.97E-01	1.53E-01	-0.45	4.34E-01	-0.23	7.90E-01	-0.08	8.12E-01	-0.07
1176.7823@3.2633436	1176.7823	3.26	NEG	hilic				2.87E-02	8.40E-01	0.06	1.20E-01	0.39	1.72E-01	0.39	1.99E-04	1.02
1176.783@0.4084699	1176.7830	0.41	NEG	hilic				9.52E-01	4.88E-01	-0.20	9.62E-01	-0.01	9.40E-01	0.02	7.65E-01	0.09
1177.7664@4.417825	1177.7664	4.42	POS	hilic				7.96E-01	8.38E-01	0.07	3.16E-01	0.27	4.60E-01	-0.22	8.61E-01	0.05
1178.2769@0.38914546	1178.2769	0.39	NEG	hilic				7.22E-01	4.11E-01	-0.20	3.71E-01	0.27	7.64E-01	-0.09	4.82E-01	0.22
1178.6707@1.3043147	1178.6707	1.30	NEG	hilic				5.75E-01	2.09E-01	-0.38	1.91E-01	-0.42	7.60E-01	0.08	8.53E-01	-0.05
1178.679@10.500568	1178.6790	10.50	NEG	RP				8.96E-01	9.46E-01	0.02	5.30E-01	-0.18	9.16E-01	-0.03	5.16E-01	0.21
1178.7855@10.70863	1178.7855	10.71	NEG	RP				7.48E-01	7.95E-01	0.07	7.65E-01	-0.07	5.92E-01	0.13	1.79E-01	0.37
1178.8708@0.6442378	1178.8708	0.64	POS	hilic				9.42E-01	5.14E-01	0.22	6.33E-01	0.12	5.20E-01	0.15	3.23E-01	0.26
1179.2795@0.3896538	1179.2795	0.39	NEG	hilic				8.67E-01	2.62E-01	-0.24	8.34E-01	0.05	9.17E-01	-0.03	6.86E-01	0.10
1179.6812@10.507921	1179.6812	10.51	NEG	RP				5.19E-02	1.76E-01	0.39	1.63E-02	-0.72	4.67E-01	-0.19	6.11E-01	-0.16
1180.5212@10.189309	1180.5212	10.19	POS	RP				4.11E-01	8.21E-02	-0.34	6.40E-01	0.13	2.45E-01	0.30	5.65E-01	0.13
1180.597@9.947264	1180.5970	9.95	NEG	RP				6.68E-01	9.04E-01	0.04	1.11E-01	0.47	2.72E-01	0.34	6.46E-01	0.13
1180.6855@1.3011757	1180.6855	1.30	NEG	hilic				4.86E-01	1.24E-01	-0.48	2.96E-01	-0.31	5.44E-01	0.16	7.05E-01	-0.11
1180.8@10.708818	1180.8000	10.71	NEG	RP				2.33E-01	1.12E-01	0.43	3.20E-03	0.76	2.35E-02	0.59	8.98E-02	0.60
1180.8925@0.64433676	1180.8925	0.64	POS	hilic				9.40E-01	5.09E-01	0.22	5.63E-01	0.15	4.58E-01	0.19	3.47E-01	0.27
1181.566@0.49551967	1181.5660	0.50	POS	hilic				8.96E-01	6.98E-01	-0.12	7.52E-01	0.09	3.77E-01	-0.27	7.38E-01	-0.09
1182.247@0.39310956	1182.2470	0.39	NEG	hilic				5.93E-01	8.63E-02	0.49	2.04E-01	0.34	2.48E-01	0.31	8.53E-01	0.05
1182.7596@10.564427	1182.7596	10.56	NEG	RP				5.96E-01	3.09E-01	-0.21	2.10E-01	0.31	6.66E-01	0.10	6.75E-01	0.10
1182.8029@10.708853	1182.8029	10.71	NEG	RP				4.61E-01	1.90E-01	0.40	1.69E-02	0.62	7.84E-02	0.47	1.56E-01	0.50
1183.268@9.708578	1183.2680	9.71	POS	RP				1.92E-01	3.96E-01	-0.25	1.99E-02	0.65	7.05E-01	0.11	7.05E-01	0.12
1183.566@0.49504507	1183.5660	0.50	POS	hilic				8.26E-01	9.41E-01	-0.02	3.86E-01	0.25	7.24E-01	-0.11	4.55E-01	0.23
1183.586@9.960092	1183.5860	9.96	NEG	RP				5.12E-02	4.16E-01	0.23	2.52E-01	-0.37	2.17E-02	-0.60	1.72E-01	0.38
1183.5865@9.936734	1183.5865	9.94	NEG	RP				5.52E-01	2.50E-01	0.38	7.03E-01	-0.11	5.24E-01	-0.16	3.39E-01	-0.28
1183.7625@10.562056	1183.7625	10.56	NEG	RP				3.95E-01	7.46E-01	-0.08	1.24E-01	0.41	1.18E-01	0.39	1.45E-01	0.41
1183.805@10.708	1183.8050	10.71	NEG	RP				3.88E-01	1.14E-01	0.48	3.45E-02	0.58	2.93E-02	0.63	1.45E-01	0.45
1184.2091@0.39220455	1184.2091	0.39	NEG	hilic				4.47E-01	1.07E-01	0.44	2.44E-01	0.27	5.17E-01	-0.12	5.76E-01	0.14
1184.873@11.109691	1184.8730	11.11	POS	RP				6.28E-01	5.01E-01	0.21	3.24E-01	-0.23	4.79E-01	0.20	4.87E-01	-0.17
1185.2806@9.58382	1185.2806	9.58	POS	RP				2.06E-01	5.05E-01	-0.18	2.32E-02	0.61	5.79E-01	-0.16	9.69E-01	-0.01
1185.2819@9.708314	1185.2819	9.71	POS	RP				3.61E-01	2.61E-01	-0.34	1.11E-01	0.47	9.65E-01	0.01	9.55E-01	-0.02
1185.5804@0.5043296	1185.5804	0.50	POS	hilic				9.92E-01	6.83E-01	0.13	7.24E-01	0.09	9.32E-01	0.03	6.55E-01	0.13
1185.5979@10.2321205	1185.5979	10.23	NEG	RP				3.93E-02	3.92E-02	0.64	6.76E-01	-0.11	1.03E-01	-0.38	2.21E-01	0.34
1185.7675@10.562105	1185.7675	10.56	NEG	RP				4.35E-01	9.80E-01	0.01	5.10E-02	0.49	1.79E-01	0.31	1.64E-01	0.40
1186.1626@9.384618	1186.1626	9.38	NEG	RP				5.04E-01	1.36E-01	-0.40	6.42E-01	0.13	3.49E-01	0.25	9.25E-01	-0.03
1186.1635@9.548318	1186.1635	9.55	NEG	RP				3.40E-01	1.83E-01	-0.38	3.93E-01	0.23	7.58E-01	0.08	1.83E-01	-0.45
1186.1646@9.89156	1186.1646	9.89	NEG	RP				4.05E-01	1.85E-02	-0.53	1.31E-01	-0.35	6.77E-01	-0.12	9.94E-01	0.00
1186.165@9.712488	1186.1650	9.71	NEG	RP				3.29E-01	7.76E-02	0.48	2.24E-02	0.73	3.72E-01	0.28	4.82E-01	0.22
1186.5175@10.168068	1186.5175	10.17	POS	RP				4.22E-01	9.37E-01	-0.02	4.67E-02	0.57	3.08E-01	0.22	1.49E-01	0.42
1187.1646@9.548571	1187.1646	9.55	NEG	RP				6.71E-01	3.36E-01	0.29	8.57E-01	0.04	6.26E-01	0.15	3.23E-01	-0.30
1187.1674@9.71282	1187.1674	9.71	NEG	RP				8.14E-02	5.99E-01	0.16	6.31E-02	0.50	2.84E-02	0.69	4.16E-01	-0.24
1187.6176@10.230722	1187.6176	10.23	NEG	RP				6.22E-02	1.47E-01	0.49	3.07E-01	-0.30	9.32E-02	-0.42	1.75E-01	0.39
1188.0013@11.036947	1188.0013	11.04	NEG	RP				6.06E-01	4.46E-01	0.26	2.55E-01	0.31	2.41E-02	0.60	2.69E-01	0.31
1188.5103@10.178998	1188.5103	10.18	POS	RP				2.26E-01	8.41E-02	-0.41	8.39E-01	0.05	3.39E-02	-0.48	6.74E-01	0.12
1189.6497@10.502479	1189.6497	10.50	NEG	RP				1.21E-02	1.59E-03	0.90	3.33E-01	-0.24	5.21E-01	-0.16	3.59E-01	0.30
1189.7751@0.38589743	1189.7751	0.39	POS	hilic				7.98E-01	3.34E-01	-0.23	5.90E-01	0.13	8.76E-01	-0.04	5.41E-01	-0.14
1190.2482@0.39321372	1190.2482	0.39	NEG	hilic				6.33E-01	3.21E-01	0.27	6.30E-02	0.55	6.31E-01	0.13	3.34E-01	0.32
1190.5187@10.163691	1190.5187	10.16	POS	RP				3.96E-02	5.66E-01	-0.13	3.53E-03	0.74	5.64E-02	0.40	6.06E-01	0.13
1190.611@10.13776	1190.6110	10.14	NEG	RP				3.25E-01	9.14E-02	-0.40	6.08E-01	-0.13	4.80E-01	0.20	1.57E-01	-0.35
1190.9998@11.036869	1190.9998	11.04	NEG	RP				4.42E-01	9.90E-02	0.51	8.29E-02	0.52	2.57E-02	0.63	2.56E-01	0.35
1191.649@10.503384	1191.6490	10.50	NEG	RP				5.14E-03	1.79E-03	0.90	1.49E-01	-0.41	6.15E-01	-0.13	2.04E-01	0.38
1191.795@10.599895	1191.7950	10.60	POS	hilic				7.98E-01	3.87E-01	0.25	5.07E-01	-0.19	5.96E-01	-0.15	6.41E-01	-0.16
1191.7957@0.38587162	1191.7957	0.39	POS	hilic				6.90E-01	4.65E-01	-0.23	1.09E-01	-0.50	1.83E-01	-0.41	4.56E-01	-0.22
1191.7957@11.833917	1191.7957	11.83	POS	RP				8.83E-01	9.51E-01	0.02	3.71E-01	0.28	3.52E-01	0.28	4.75E-01	0.22
1192.2618@0.39295495	1192.2618	0.39	NEG	hilic				6.94E-01	4.72E-01	0.20	1.89E-01	0.40	6.39E-01	0.13	1.27E-01	0.47
1192.5677@1.0909811	1192.5677	1.09	POS	hilic				6.50E-01	5.43E-01	0.15	8.06E-01	0.06	8.52E-02	0.46	3.78E-01	0.21
1192.6523@10.502618	1192.6523	10.50	NEG	RP				6.94E-03	4.91E-03	0.82	9.85E-02	-0.46	4.45E-01	-0.19	3.88E-01	0.26
1193.2646@0.3927547	1193.2646	0.39	NEG	hilic				6.17E-01	3.99E-01	0.23	5.66E-01	0.18	1.38E-01	0.39	8.74E-02	0.55
1193.8105@10.59913	1193.8105	10.60	POS	hilic				9.87E-01	7.89E-01	0.08	7.12E-01	-0.11	8.30E-01	0.06	9.79E-01	-0.01
1193.8112@11.833765	1193.8112	11.83	POS	RP				9.01E-01	7.65E-01	0.10	8.86E-01	-0.04	3.09E-01	0.30	9.92E-01	0.00
1193.8123@0.38584334	1193.8123	0.39	POS	hilic				5.89E-01	5.10E-01	-0.21	7.44E-02	-0.55	1.81E-01	-0.41	6.07E-01	-0.15
1194.5219@10.1680155	1194.5219	10.17	POS	RP				2.42E-01	8.71E-01	0.04	2.64E-02	0.65	3.26E-01	0.24	7.43E-02	0.44
1194.6141@9.942633	1194.6141	9.94	NEG	RP				7.24E-02	2.25E-01	-0.34	8.35E-01	0.07	1.96E-02	0.63	2.55E-01	-0.31
1194.8138@10.59982	1194.8138	10.60	POS	hilic				9.19E-01	9.00E-01	0.04	9.61E-01	-0.02	4.02E-01	-0.25	5.63E-01	-0.19
1194.816@0.38587162	1194.8160	0.39	POS	hilic				7.02E-01	4.80E-01	-0.23	1.15E-01	-0.50	2.06E-01	-0.38	5.00E-01	-0.20
1195.5673@0.49742582	1195.5673	0.50	POS	hilic				9.32E-01	3.61E-01	0.25	4.54E-01	0.18	4.29E-01	0.18	6.81E-01	0.10
1196.2415@0.39556396	1196.2415	0.40	NEG	hilic				7.68E-01	3.14E-01	0.34	1.73E-01	0.42	2.52E-01	0.32	6.54E-01	0.14
1197.5754@0.49626622	1197.5754	0.50	POS	hilic				7.00E-01	5.28E-01	0.18	2.21E-01	0.35	2.58E-01	0.34	1.10E-01	0.49
1197.8431@10.708233	1197.8431	10.71	NEG	RP				2.96E-01	2.23E-01	-0.29	5.31E-01	-0.19	8.72E-02	0.45	6.41E-01	-0.12

Supplementary table 2: Metabolomics analysis results

Compound	Mass	Retention Time	Ionization mode	column	Compound_ID	MSMS_spectra	ID_level	ANOVA	Anorexia vs control		overweight vs control		obese vs control		athletes vs control	
								P-FDR	P	Estimate	P	Estimate	P	Estimate	P	Estimate
1198.7657@0.38596657	1198.7657	0.39	POS	hilic				2.50E-01	2.23E-01	-0.35	1.62E-02	-0.70	2.97E-02	-0.58	7.36E-02	-0.49
1198.7668@11.830533	1198.7668	11.83	POS	RP				9.70E-01	5.94E-01	-0.18	9.57E-01	-0.02	9.85E-01	-0.01	7.43E-01	0.10
1200.5654@0.4896046	1200.5654	0.49	POS	hilic				6.23E-01	1.02E-01	-0.36	6.02E-01	0.14	8.34E-01	-0.05	9.20E-01	-0.02
1201.789@4.551504	1201.7890	4.55	NEG	hilic				7.79E-01	2.58E-01	0.35	5.00E-01	0.21	9.80E-01	0.01	7.30E-01	-0.09
1202.2242@0.3905619	1202.2242	0.39	NEG	hilic				9.38E-01	8.04E-01	0.07	4.05E-01	0.25	6.10E-01	0.15	4.12E-01	0.25
1203.575@10.15956	1203.5750	10.16	NEG	RP				7.33E-01	8.21E-01	-0.08	2.20E-01	0.37	3.72E-01	0.21	3.08E-01	0.30
1204.2325@0.3878378	1204.2325	0.39	NEG	hilic				9.23E-01	6.25E-01	0.16	5.42E-01	0.19	8.05E-01	-0.07	5.02E-01	0.20
1204.2332@9.594154	1204.2332	9.59	NEG	RP				4.03E-01	2.92E-02	0.54	1.47E-01	0.42	3.55E-01	0.26	2.56E-02	0.67
1204.5316@9.890331	1204.5316	9.89	POS	RP				2.77E-01	3.48E-02	0.61	7.76E-01	-0.07	7.04E-01	-0.09	4.79E-01	0.20
1205.2365@0.3837692	1205.2365	0.38	NEG	hilic				9.29E-01	4.54E-01	0.25	5.26E-01	0.20	8.97E-01	-0.04	7.31E-01	0.11
1205.8104@0.3866172	1205.8104	0.39	POS	hilic				7.10E-01	4.54E-01	-0.21	1.17E-01	-0.44	2.14E-01	-0.35	3.14E-01	-0.27
1206.565@9.875046	1206.5650	9.88	POS	RP				2.00E-01	2.87E-01	0.32	8.07E-01	-0.06	9.55E-02	-0.37	1.66E-01	-0.30
1206.7474@0.3711728	1206.7474	0.37	NEG	hilic				8.34E-01	6.43E-01	-0.15	5.24E-01	-0.21	1.45E-01	-0.41	8.26E-01	-0.06
1207.585@9.945104	1207.5850	9.95	NEG	RP				3.22E-01	8.84E-01	-0.04	1.82E-01	0.40	1.33E-02	0.66	2.63E-01	0.32
1207.7911@11.318783	1207.7911	11.32	POS	RP				7.64E-01	2.22E-01	-0.27	6.73E-01	-0.10	9.43E-01	0.02	6.58E-01	0.11
1207.825@11.833285	1207.8250	11.83	POS	RP				9.97E-01	9.56E-01	0.02	9.36E-01	-0.02	7.08E-01	-0.09	9.89E-01	0.00
1208.257@0.39137995	1208.2570	0.39	NEG	hilic				7.31E-01	8.60E-01	0.05	1.18E-01	0.45	8.16E-01	0.06	5.95E-01	0.16
1208.5673@10.259308	1208.5673	10.26	POS	RP				3.89E-01	4.97E-02	0.56	8.34E-01	-0.04	4.96E-01	0.15	4.96E-01	0.18
1209.8042@11.378127	1209.8042	11.38	POS	RP				7.56E-01	1.47E-01	-0.47	5.05E-01	-0.19	9.26E-01	-0.03	8.68E-01	-0.05
1209.8052@11.212464	1209.8052	11.21	POS	RP				8.27E-01	3.68E-01	-0.23	5.21E-01	-0.17	5.96E-01	0.13	8.31E-01	-0.06
1209.8268@10.709147	1209.8268	10.71	NEG	RP				7.61E-01	2.12E-01	0.35	7.81E-01	0.09	1.51E-01	0.41	7.53E-01	0.08
1210.2574@0.3911636	1210.2574	0.39	NEG	hilic				8.42E-01	9.44E-01	0.02	3.45E-01	0.29	6.62E-01	-0.12	6.29E-01	0.15
1210.6075@10.238179	1210.6075	10.24	NEG	RP				9.29E-01	7.60E-01	0.10	6.69E-01	-0.12	4.16E-01	-0.21	9.29E-01	0.03
1210.8077@0.386623	1210.8077	0.39	POS	hilic				7.14E-01	4.27E-01	-0.22	2.52E-01	-0.30	2.35E-01	-0.32	1.04E-01	-0.41
1211.5955@0.50846666	1211.5955	0.51	POS	hilic				7.41E-01	8.39E-01	-0.05	3.61E-01	0.21	3.52E-01	-0.19	6.15E-01	0.13
1211.8246@10.709184	1211.8246	10.71	NEG	RP				1.07E-01	3.84E-01	0.27	3.31E-02	0.62	1.29E-02	0.77	9.35E-01	-0.03
1212.2452@0.38757658	1212.2452	0.39	NEG	hilic				7.61E-01	9.13E-01	-0.03	2.46E-01	0.36	9.40E-01	0.02	3.58E-01	0.29
1212.7231@11.828658	1212.7231	11.83	POS	RP				9.67E-01	9.12E-01	-0.03	7.29E-01	-0.09	7.92E-01	-0.07	6.13E-01	0.13
1212.7332@0.38550004	1212.7332	0.39	POS	hilic				5.43E-01	1.59E-01	-0.37	5.69E-02	-0.47	1.89E-01	-0.33	4.95E-01	-0.18
1212.7584@0.4064697	1212.7584	0.41	NEG	hilic				9.33E-01	3.30E-01	-0.25	8.53E-01	-0.05	6.87E-01	-0.10	9.51E-01	0.02
1212.7592@3.5492723	1212.7592	3.55	NEG	hilic				2.08E-01	4.69E-01	-0.27	7.95E-01	0.08	7.06E-01	0.09	2.30E-02	-0.68
1213.415@3.0284107	1213.4150	3.03	POS	hilic				9.63E-01	3.65E-01	0.26	8.67E-01	0.05	7.56E-01	0.10	9.65E-01	0.01
1213.8387@10.710087	1213.8387	10.71	NEG	RP				6.51E-02	9.38E-01	-0.02	6.91E-02	0.49	1.16E-02	0.89	5.78E-01	0.16
1214.1963@9.31399	1214.1963	9.31	NEG	RP				5.43E-01	2.14E-01	-0.39	6.25E-01	-0.14	3.50E-01	0.27	8.37E-01	0.06
1214.2574@9.468589	1214.2574	9.47	NEG	RP				8.01E-01	1.72E-01	0.36	9.93E-01	0.00	7.79E-01	0.09	8.25E-01	-0.05
1214.2574@9.590801	1214.2574	9.59	NEG	RP				8.56E-01	9.02E-01	-0.04	9.02E-01	0.03	2.34E-01	0.35	9.96E-01	0.00
1214.2614@0.38741884	1214.2614	0.39	NEG	hilic				5.29E-01	9.77E-01	0.01	7.02E-02	0.56	6.43E-01	0.12	3.43E-01	0.32
1214.6198@9.954984	1214.6198	9.95	NEG	RP				4.26E-03	9.62E-03	0.79	9.30E-01	-0.02	2.39E-02	-0.44	3.12E-01	0.26
1214.7397@11.828638	1214.7397	11.83	POS	RP				8.79E-01	3.92E-01	-0.27	4.58E-01	-0.20	6.17E-01	-0.14	8.01E-01	0.07
1214.7415@0.3859478	1214.7415	0.39	POS	hilic				5.55E-01	2.89E-01	-0.30	7.55E-02	-0.52	9.24E-02	-0.48	3.81E-01	-0.24
1214.7559@11.212047	1214.7559	11.21	POS	RP				8.06E-01	1.67E-01	-0.32	3.12E-01	-0.24	8.10E-01	-0.06	5.15E-01	-0.16
1215.2642@0.38775674	1215.2642	0.39	NEG	hilic				6.69E-01	8.69E-01	-0.05	3.07E-01	0.31	8.15E-01	-0.07	2.44E-01	0.38
1215.4128@3.0344784	1215.4128	3.03	POS	hilic				6.68E-01	1.17E-01	0.47	1.09E-01	0.47	3.88E-01	0.26	5.25E-01	0.20
1215.7441@11.829002	1215.7441	11.83	POS	RP				9.43E-01	5.09E-01	-0.20	7.15E-01	-0.09	8.32E-01	-0.06	7.46E-01	0.09
1215.7452@0.38529408	1215.7452	0.39	POS	hilic				6.81E-01	2.88E-01	-0.31	9.46E-02	-0.47	1.92E-01	-0.36	3.69E-01	-0.25
1215.7635@0.40624136	1215.7635	0.41	NEG	hilic				6.85E-01	4.03E-01	-0.17	9.44E-01	-0.02	2.68E-01	-0.21	4.77E-01	0.16
1217.409@3.0510411	1217.4090	3.05	POS	hilic				6.42E-01	5.63E-01	-0.19	5.37E-02	-0.53	6.04E-01	-0.16	5.64E-01	-0.15
1218.2358@0.38628203	1218.2358	0.39	NEG	hilic				6.93E-01	9.63E-01	-0.01	1.58E-01	-0.41	4.40E-01	-0.23	1.97E-01	-0.40
1218.6562@10.232889	1218.6562	10.23	NEG	RP				4.59E-03	3.29E-03	0.92	7.74E-01	-0.06	2.29E-01	-0.26	1.20E-01	0.39
1219.8267@11.831924	1219.8267	11.83	POS	RP				8.99E-01	5.31E-01	-0.20	8.74E-01	0.04	9.32E-01	-0.02	5.34E-01	0.17
1220.8177@0.38621536	1220.8177	0.39	POS	hilic				9.41E-01	8.58E-01	-0.05	3.64E-01	-0.25	6.49E-01	-0.12	8.39E-01	-0.06
1221.4633@3.2094579	1221.4633	3.21	NEG	hilic				8.01E-01	7.10E-01	0.13	4.47E-01	-0.22	5.18E-01	0.18	6.11E-01	-0.14
1221.4644@3.2004406	1221.4644	3.20	NEG	hilic				8.18E-01	6.49E-01	0.14	7.25E-01	-0.10	3.11E-01	0.33	9.65E-01	0.01
1221.8254@0.38414282	1221.8254	0.38	POS	hilic				6.60E-01	5.57E-01	-0.15	1.57E-01	-0.34	1.17E-01	-0.37	6.33E-01	-0.12
1221.841@11.831193	1221.8410	11.83	POS	RP				9.73E-01	7.54E-01	-0.10	9.95E-01	0.00	8.92E-01	-0.04	6.10E-01	0.16
1222.1758@9.571532	1222.1758	9.57	NEG	RP				9.48E-01	9.82E-01	-0.01	9.93E-01	0.00	9.65E-01	0.01	4.30E-01	0.24
1222.179@9.71639	1222.1790	9.72	NEG	RP				2.70E-01	3.65E-01	-0.26	5.93E-01	0.17	3.27E-01	0.25	9.58E-02	-0.51
1222.4456@0.42297012	1222.4456	0.42	POS	hilic				2.59E-01	2.19E-01	-0.37	6.29E-01	-0.13	5.81E-03	-0.65	7.75E-01	-0.07
1222.4854@10.280106	1222.4854	10.28	POS	RP				1.48E-01	3.42E-01	0.27	6.70E-03	0.77	5.37E-03	0.68	3.77E-01	0.28
1222.5406@10.160078	1222.5406	10.16	NEG	RP				2.50E-01	6.19E-02	-0.51	3.77E-01	0.27	5.12E-01	0.18	4.18E-01	-0.23
1222.7805@0.3930732	1222.7805	0.39	POS	hilic				7.12E-01	3.93E-01	0.23	7.85E-02	0.40	4.33E-01	0.17	1.92E-01	0.29
1222.8914@3.3243177	1222.8914	3.32	NEG	hilic				8.11E-01	9.70E-01	-0.01	4.12E-01	-0.23	9.64E-01	-0.01	3.91E-01	0.24
1222.9653@3.200402	1222.9653	3.20	NEG	hilic				9.60E-01	6.22E-01	0.17	8.57E-01	0.05	6.26E-01	0.15	3.95E-01	0.24
1223.7874@0.39309618	1223.7874	0.39	POS	hilic				7.69E-01	6.12E-01	0.14	8.17E-02	0.39	7.70E-01	0.07	6.53E-01	0.11
1224.6746@10.240626	1224.6746	10.24	NEG	RP				5.19E-02	1.40E-02	0.74	3.14E-01	-0.25	5.35E-01	-0.17	6.09E-01	0.13
1224.779@0.39247826	1224.7790	0.39	POS	hilic				7.36E-01	5.82E-01	-0.16	3.29E-01	-0.26	5.13E-01	-0.18	1.01E-01	-0.45
1224.893@3.324295	1224.8930	3.32	NEG	hilic				6.32E-01	4.72E-01	0.24	6.70E-01	-0.13	3.92E-01	0.27	1.66E-01	0.40
1225.5905@0.52071726	1225.5905	0.52	POS	hilic				8.65E-01	9.08E-01	-0.03	3.53E-01	-0.26	6.57E-01	-0.13	5.89E-01	0.13
1225.7847@0.39132822	1225.7847	0.39	POS	hilic				9.81E-01	6.71E-01	0.12	7.28E-01	-0.09	9.16E-01	0.03	9.65E-01	-0.01
1226.8845@3.3246603	1226.8845	3.32	NEG	hilic				6.42E-01	5.35E-01	0.22	7.95E-01	-0.07	2.82E-01	0.33	1.11E-01	0.42
1227.2408@0.38618803	1227.2408	0.39	NEG	hilic				5.80E-01	3.57E-01	-0.30	1.36E-01	-0.42	3.55E-01	-0.24	6.85E-02	-0.59
1227.5931@0.52037406	1227.5931	0.52	POS	hilic				8.47E-01	2.44E-01	0.36	9.90E-01	0.00	4.89E-01	0.20	9.34E-01	0.03
1228.706@10.504203	1228.7060	10.50	NEG	RP				4.99E-03	6.49E-03	0.76	1.21E-01	-0.39	3.41E-01	-0.23	1.62E-01	0.42

Supplementary table 2: Metabolomics analysis results

Compound	Mass	Retention Time	Ionization mode	column	Compound_ID	MSMS_spectra	ID_level	ANOVA	Anorexia vs control		overweight vs control		obese vs control		athletes vs control	
								P-FDR	P	Estimate	P	Estimate	P	Estimate	P	Estimate
1228.8674@10.866501	1228.8674	10.87	POS	RP				8.42E-02	3.10E-01	0.28	2.14E-01	-0.32	2.26E-02	-0.52	1.64E-01	-0.34
1229.6027@0.52576	1229.6027	0.53	POS	hilic				2.64E-01	3.77E-01	-0.26	1.49E-02	-0.63	1.73E-01	-0.36	3.67E-02	-0.58
1230.257@0.39030173	1230.2570	0.39	NEG	hilic				3.68E-01	1.71E-02	0.65	6.53E-02	0.56	4.68E-01	0.20	5.89E-01	0.17
1231.2502@9.602039	1231.2502	9.60	NEG	RP				2.31E-01	1.64E-02	-0.60	2.40E-01	-0.30	2.83E-01	-0.31	5.90E-01	0.15
1231.2524@0.39069998	1231.2524	0.39	NEG	hilic				8.13E-01	1.12E-01	0.40	2.42E-01	0.35	5.96E-01	0.15	4.70E-01	0.24
1234.4803@10.0445	1234.4803	10.04	POS	RP				3.83E-01	1.20E-01	-0.36	3.39E-01	-0.22	8.88E-01	-0.04	1.49E-02	-0.50
1235.727@4.465406	1235.7270	4.47	POS	hilic				9.32E-01	8.13E-01	0.08	5.11E-01	-0.18	7.98E-01	0.07	5.89E-01	-0.12
1235.7819@4.55864	1235.7819	4.56	NEG	hilic				4.37E-01	5.39E-02	0.60	8.18E-01	-0.07	4.38E-01	0.23	2.65E-01	0.31
1235.8542@11.833614	1235.8542	11.83	POS	RP				7.64E-01	3.00E-01	-0.32	4.60E-01	-0.22	7.48E-01	-0.10	5.82E-01	0.15
1236.1943@9.717633	1236.1943	9.72	NEG	RP				3.48E-01	4.95E-02	-0.48	9.15E-01	-0.03	1.99E-01	0.36	5.90E-01	-0.16
1236.1945@9.571521	1236.1945	9.57	NEG	RP				5.45E-01	2.34E-01	-0.36	3.06E-01	-0.30	2.95E-01	-0.29	2.41E-02	-0.64
1237.196@9.571418	1237.1960	9.57	NEG	RP				7.68E-01	5.90E-01	-0.17	4.76E-01	-0.22	5.08E-01	-0.17	1.07E-01	-0.49
1237.1978@9.720776	1237.1978	9.72	NEG	RP				7.96E-01	2.67E-01	-0.32	6.33E-01	-0.16	5.56E-01	0.15	5.14E-01	-0.18
1238.253@0.3697637	1238.2530	0.37	NEG	hilic				7.64E-01	4.65E-01	0.24	5.59E-01	-0.17	4.75E-01	-0.21	4.81E-01	-0.20
1238.7524@0.36957273	1238.7524	0.37	NEG	hilic				6.04E-01	3.24E-01	0.31	5.90E-01	-0.17	3.28E-01	-0.28	4.76E-01	-0.20
1239.5946@0.48193556	1239.5946	0.48	POS	hilic				9.93E-01	8.16E-01	-0.07	7.22E-01	0.09	8.70E-01	0.04	9.64E-01	-0.01
1240.1504@9.568658	1240.1504	9.57	NEG	RP				9.77E-01	4.85E-01	0.19	6.37E-01	0.13	5.69E-01	0.15	5.74E-01	0.19
1242.2885@0.3874635	1242.2885	0.39	NEG	hilic				6.99E-01	9.48E-01	-0.02	1.45E-01	0.43	7.33E-01	0.09	3.42E-01	0.30
1242.6313@10.241457	1242.6313	10.24	NEG	RP				1.75E-01	3.08E-01	0.28	2.08E-01	-0.43	2.58E-02	-0.57	5.28E-01	-0.17
1243.6162@0.5543	1243.6162	0.55	POS	hilic				6.64E-01	5.88E-01	0.13	8.56E-01	-0.04	4.61E-01	-0.14	2.78E-01	0.29
1244.0042@11.035856	1244.0042	11.04	NEG	RP				3.84E-01	1.58E-01	-0.34	7.72E-01	-0.08	4.11E-01	0.19	7.35E-02	-0.44
1244.6656@10.509792	1244.6656	10.51	NEG	RP				9.89E-01	6.22E-01	0.12	9.10E-01	-0.03	8.83E-01	-0.03	8.98E-01	0.03
1245.7527@4.41647	1245.7527	4.42	POS	hilic				4.66E-01	9.62E-01	-0.01	4.03E-01	0.21	3.95E-01	-0.26	1.64E-01	-0.45
1246.2546@0.3907719	1246.2546	0.39	NEG	hilic				8.89E-01	8.94E-01	0.03	3.29E-01	0.26	8.85E-01	-0.03	9.89E-01	0.00
1246.6635@10.509687	1246.6635	10.51	NEG	RP				1.33E-01	2.56E-02	0.57	2.32E-01	-0.34	6.11E-01	-0.14	3.89E-01	0.28
1247.2762@9.724983	1247.2762	9.72	POS	RP				1.33E-01	1.92E-01	-0.37	4.69E-02	0.61	3.85E-01	0.23	5.08E-01	0.21
1247.6685@10.51386	1247.6685	10.51	NEG	RP				1.66E-01	1.10E-01	0.45	4.12E-01	-0.18	1.63E-01	-0.31	4.61E-01	0.22
1248.1779@9.730238	1248.1779	9.73	NEG	RP				3.69E-01	7.54E-01	0.09	5.28E-02	0.63	8.60E-01	-0.06	4.43E-01	0.20
1248.1812@9.571474	1248.1812	9.57	NEG	RP				7.31E-01	6.42E-01	-0.17	9.90E-01	0.00	2.02E-01	0.31	3.99E-01	0.26
1249.2928@9.72447	1249.2928	9.72	POS	RP				3.33E-01	5.54E-01	-0.20	5.40E-02	0.58	3.94E-01	0.21	8.11E-01	0.07
1249.2935@9.601441	1249.2935	9.60	POS	RP				4.23E-01	8.01E-01	0.08	6.55E-01	0.12	7.48E-02	-0.48	2.57E-01	-0.36
1250.1761@9.571094	1250.1761	9.57	NEG	RP				6.21E-01	8.32E-01	-0.06	9.49E-01	-0.02	5.87E-02	0.45	4.09E-01	0.29
1250.1761@9.730484	1250.1761	9.73	NEG	RP				4.39E-01	9.42E-01	-0.02	4.02E-02	0.62	3.85E-01	0.28	4.98E-01	0.18
1250.1783@9.89722	1250.1783	9.90	NEG	RP				1.42E-01	1.52E-01	-0.39	7.88E-02	-0.51	4.13E-01	0.24	8.32E-02	-0.45
1250.2361@0.389387	1250.2361	0.39	NEG	hilic				4.63E-01	3.17E-01	0.26	1.44E-01	0.36	5.45E-01	0.14	5.40E-02	0.57
1250.7408@0.73003453	1250.7408	0.73	POS	hilic				7.96E-01	2.22E-01	-0.21	9.94E-01	0.00	8.56E-01	0.04	5.92E-01	0.12
1251.1783@9.727472	1251.1783	9.73	NEG	RP				3.47E-01	9.69E-01	-0.01	9.44E-02	0.52	5.12E-02	0.56	7.26E-01	0.10
1251.179@9.570847	1251.1790	9.57	NEG	RP				7.64E-01	2.47E-01	-0.31	6.74E-01	0.13	7.48E-01	0.09	5.18E-01	-0.20
1252.8914@1.3129399	1252.8914	1.31	POS	hilic				6.27E-01	9.61E-01	0.01	7.66E-01	-0.08	6.76E-01	-0.12	1.77E-01	0.44
1252.8975@1.1525743	1252.8975	1.15	POS	hilic				5.14E-03	4.41E-03	0.70	2.73E-01	-0.27	1.77E-01	-0.36	5.15E-02	0.64
1254.2607@0.38856757	1254.2607	0.39	NEG	hilic				4.75E-01	1.06E-01	0.49	6.57E-02	0.57	1.76E-01	0.33	7.96E-02	0.54
1255.601@10.242964	1255.6010	10.24	NEG	RP				1.42E-01	5.22E-02	0.53	2.64E-01	-0.32	1.68E-01	-0.34	7.94E-01	0.08
1256.274@0.38835904	1256.2740	0.39	NEG	hilic				1.14E-01	3.22E-02	0.65	3.18E-03	0.89	9.95E-03	0.62	7.19E-02	0.59
1257.2769@0.3893	1257.2769	0.39	NEG	hilic				3.78E-01	3.58E-02	0.60	4.21E-02	0.59	2.61E-01	0.31	6.87E-02	0.56
1257.981@11.036501	1257.9810	11.04	NEG	RP				1.55E-01	3.38E-01	0.30	3.22E-01	0.31	7.18E-03	0.66	3.26E-01	-0.25
1259.6368@10.504166	1259.6368	10.50	NEG	RP				2.00E-03	2.10E-03	0.85	1.36E-01	-0.36	3.66E-01	-0.22	1.33E-01	0.40
1260.2538@0.39164585	1260.2538	0.39	NEG	hilic				5.96E-01	1.56E-01	0.46	5.52E-01	0.15	5.02E-01	-0.17	9.62E-01	-0.02
1262.25@0.39247274	1262.2500	0.39	NEG	hilic				1.45E-01	8.58E-02	0.45	7.33E-02	0.45	9.05E-02	0.45	4.42E-01	-0.17
1264.2292@9.760215	1264.2292	9.76	NEG	RP				9.35E-01	9.69E-01	0.01	8.85E-01	-0.04	9.41E-01	0.02	3.53E-01	-0.26
1266.2365@0.38864988	1266.2365	0.39	NEG	hilic				5.03E-01	7.96E-01	-0.06	2.79E-01	0.30	8.66E-01	-0.04	1.42E-01	0.44
1266.4423@3.2081215	1266.4423	3.21	NEG	hilic				8.41E-01	8.19E-01	-0.07	3.67E-01	-0.22	9.52E-01	-0.02	4.95E-01	0.21
1268.2448@0.38655853	1268.2448	0.39	NEG	hilic				8.34E-01	2.71E-01	0.31	5.58E-01	0.19	6.62E-01	0.13	1.52E-01	0.40
1268.2452@9.608779	1268.2452	9.61	NEG	RP				3.65E-01	7.83E-02	0.45	4.16E-01	-0.24	6.87E-01	-0.12	2.99E-01	0.29
1268.2458@9.769639	1268.2458	9.77	NEG	RP				4.40E-01	2.88E-02	0.59	8.75E-01	-0.05	9.71E-01	0.01	3.27E-01	0.31
1269.6046@0.5227291	1269.6046	0.52	POS	hilic				9.07E-01	4.88E-01	0.21	2.65E-01	0.31	8.66E-01	0.05	6.31E-01	0.15
1269.778@4.5493155	1269.7780	4.55	NEG	hilic				4.97E-01	1.57E-01	0.38	1.15E-01	0.48	7.32E-01	-0.08	8.90E-01	0.04
1270.7585@0.36896336	1270.7585	0.37	NEG	hilic				8.89E-01	9.38E-01	-0.02	3.19E-01	-0.29	3.58E-01	-0.28	6.16E-01	-0.15
1271.6143@0.5146488	1271.6143	0.51	POS	hilic				7.76E-01	7.41E-01	-0.09	5.88E-01	0.14	5.42E-01	-0.19	3.96E-01	0.27
1273.6285@0.5247246	1273.6285	0.52	POS	hilic				2.76E-01	4.76E-01	-0.17	8.26E-01	0.05	2.83E-01	-0.24	1.14E-01	0.47
1274.2719@0.39571157	1274.2719	0.40	NEG	hilic				7.34E-01	3.94E-01	0.23	4.59E-01	0.23	5.85E-01	0.14	1.26E-01	0.51
1274.565@10.244172	1274.5650	10.24	NEG	RP				9.92E-01	7.86E-01	0.08	8.32E-01	-0.05	7.74E-01	-0.07	9.64E-01	0.01
1274.874@1.1465064	1274.8740	1.15	POS	hilic				5.10E-02	4.61E-02	0.56	8.41E-01	0.05	1.38E-01	-0.34	1.02E-01	0.50
1276.258@0.3865585	1276.2580	0.39	NEG	hilic				7.02E-01	7.76E-01	0.08	2.72E-01	0.35	5.87E-01	0.15	1.22E-01	0.47
1276.8918@1.1450958	1276.8918	1.15	POS	hilic				1.27E-01	8.07E-02	0.41	3.17E-01	-0.26	1.85E-01	-0.36	2.23E-01	0.41
1277.9084@10.782548	1277.9084	10.78	NEG	RP				2.01E-01	1.96E-01	-0.25	3.86E-01	0.23	5.32E-02	0.47	6.96E-01	-0.09
1278.2054@9.354934	1278.2054	9.35	NEG	RP				9.10E-01	7.26E-01	-0.09	5.18E-01	0.19	5.97E-01	0.16	4.18E-01	0.21
1278.2708@9.608715	1278.2708	9.61	NEG	RP				7.72E-01	9.14E-01	0.03	1.47E-01	0.41	9.39E-01	0.02	5.05E-01	0.20
1278.2736@0.38653845	1278.2736	0.39	NEG	hilic				5.19E-01	5.94E-01	0.16	8.05E-02	0.55	2.41E-01	0.32	1.23E-01	0.51
1278.6002@10.507895	1278.6002	10.51	NEG	RP				1.12E-01	3.25E-02	0.55	1.36E-01	-0.36	6.93E-01	-0.10	7.02E-01	0.12
1278.7705@0.45811117	1278.7705	0.46	POS	hilic				7.15E-01	5.33E-01	-0.15	8.82E-02	-0.32	2.65E-01	-0.23	6.86E-01	-0.09
1278.9045@1.2058825	1278.9045	1.21	POS	hilic				1.61E-01	9.76E-01	0.01	5.04E-01	-0.18	2.03E-01	-0.37	8.21E-02	0.55
1279.2769@0.38673866	1279.2769	0.39	NEG	hilic				5.96E-01	6.02E-01	0.15	1.90E-01	0.39	4.48E-01	0.22	6.46E-02	0.57
1280.924@1.2078333	1280.9240	1.21	POS	hilic				2.17E-01	1.09E-01	0.46	6.08E-01	-0.14	9.52E-01	-0.02	6.47E-02	0.60

Supplementary table 2: Metabolomics analysis results

Compound	Mass	Retention Time	Ionization mode	column	Compound_ID	MSMS_spectra	ID_level	ANOVA P-FDR	Anorexia vs control P	Anorexia vs control Estimate	overweight vs control P	overweight vs control Estimate	obese vs control P	obese vs control Estimate	athletes vs control P	athletes vs control Estimate
1280.9637@11.037515	1280.9637	11.04	NEG	RP				6.89E-01	3.21E-01	-0.25	9.60E-01	0.01	4.69E-01	0.19	3.21E-01	-0.26
1282.2457@9.594045	1282.2457	9.59	NEG	RP				6.34E-01	8.68E-01	0.05	6.44E-01	0.11	5.62E-02	0.49	2.79E-01	0.37
1282.2476@0.38619655	1282.2476	0.39	NEG	hilic				7.27E-01	9.47E-01	-0.02	2.95E-01	-0.29	4.69E-01	0.21	5.06E-01	-0.21
1283.9666@11.037756	1283.9666	11.04	NEG	RP				4.62E-01	5.88E-01	0.17	8.32E-02	0.44	5.42E-02	0.63	3.96E-01	0.23
1285.6284@0.5240694	1285.6284	0.52	POS	hilic				5.56E-01	6.07E-01	0.14	3.41E-01	-0.25	3.36E-01	-0.28	4.14E-01	0.24
1286.1877@9.5928335	1286.1877	9.59	NEG	RP				7.31E-01	5.96E-01	0.16	9.73E-01	0.01	3.68E-01	0.25	3.75E-01	-0.22
1286.8606@10.78224	1286.8606	10.78	NEG	RP				8.32E-02	3.16E-01	-0.19	5.57E-02	0.49	8.77E-02	0.40	6.53E-01	-0.10
1287.6403@0.5248392	1287.6403	0.52	POS	hilic				6.90E-01	7.73E-02	-0.47	8.31E-01	-0.06	5.55E-01	-0.15	2.48E-01	-0.32
1289.1685@7.1878953	1289.1685	7.19	NEG	RP				4.61E-01	3.85E-01	-0.27	1.17E-01	0.43	4.63E-01	0.23	9.89E-01	0.00
1289.6273@0.58962494	1289.6273	0.59	POS	hilic				7.58E-01	1.31E-01	0.30	1.19E-01	0.36	4.68E-01	0.17	3.62E-01	0.23
1290.5411@10.487489	1290.5411	10.49	POS	RP				6.40E-01	7.39E-01	-0.08	5.35E-01	-0.14	3.59E-01	-0.22	3.70E-01	0.26
1290.9146@11.038833	1290.9146	11.04	NEG	RP				4.47E-01	2.40E-02	0.64	9.15E-02	0.49	3.12E-01	0.26	4.13E-01	0.25
1291.1827@7.433966	1291.1827	7.43	NEG	RP				7.12E-01	1.40E-01	-0.36	7.00E-01	-0.09	9.10E-01	0.03	7.03E-01	0.10
1291.184@7.94425	1291.1840	7.94	NEG	RP				6.91E-01	1.87E-01	-0.34	7.73E-01	-0.07	9.35E-01	-0.03	4.15E-01	0.24
1291.1844@6.73514	1291.1844	6.74	NEG	RP				2.00E-02	4.16E-01	-0.16	3.95E-01	0.20	3.52E-02	0.56	2.97E-02	-0.35
1291.252@0.3859316	1291.2520	0.39	NEG	hilic				1.04E-01	4.50E-01	-0.25	4.22E-03	-0.87	1.81E-01	-0.34	1.54E-02	-0.68
1292.184@6.025705	1292.1840	6.03	POS	hilic				4.81E-01	4.88E-01	-0.19	1.88E-01	-0.31	8.27E-01	-0.05	3.07E-01	0.24
1294.2675@0.38741276	1294.2675	0.39	NEG	hilic				7.33E-01	3.25E-01	0.32	5.15E-01	0.19	8.49E-01	0.05	1.15E-01	0.45
1294.9255@11.0386715	1294.9255	11.04	NEG	RP				6.61E-01	7.51E-02	0.48	2.21E-01	0.33	3.59E-01	0.25	9.87E-01	-0.01
1295.2657@0.3882566	1295.2657	0.39	NEG	hilic				8.67E-01	2.72E-01	0.37	3.83E-01	0.27	6.74E-01	0.10	4.19E-01	0.23
1295.925@11.037587	1295.9250	11.04	NEG	RP				6.41E-01	2.70E-01	0.34	6.92E-02	0.55	2.54E-01	0.34	2.16E-01	0.33
1296.83@3.3252528	1296.8300	3.33	NEG	hilic				6.10E-01	3.93E-01	-0.22	7.41E-01	-0.09	5.36E-01	0.20	2.78E-01	0.31
1297.373@3.0301993	1297.3730	3.03	POS	hilic				7.22E-01	9.52E-01	0.02	3.72E-01	0.25	2.52E-01	-0.30	9.34E-01	0.02
1297.3737@3.0301993	1297.3737	3.03	POS	hilic				5.60E-01	4.39E-01	-0.26	8.78E-01	0.04	3.21E-02	-0.50	2.56E-01	-0.35
1298.2072@9.591332	1298.2072	9.59	NEG	RP				7.07E-01	3.08E-01	-0.31	5.28E-01	-0.20	5.35E-01	-0.15	7.92E-02	-0.52
1298.8218@3.3253534	1298.8218	3.33	NEG	hilic				8.67E-01	7.80E-01	-0.08	7.26E-01	-0.10	6.74E-01	0.14	3.79E-01	0.25
1298.9257@11.037861	1298.9257	11.04	NEG	RP				5.76E-01	1.22E-01	0.42	7.48E-02	0.48	1.95E-01	0.31	4.49E-01	0.19
1299.372@3.039964	1299.3720	3.04	POS	hilic				3.85E-01	1.87E-01	-0.43	4.03E-01	0.19	1.10E-01	-0.42	9.09E-01	0.03
1300.2065@9.591332	1300.2065	9.59	NEG	RP				7.29E-01	3.24E-01	-0.29	9.91E-01	0.00	8.89E-01	0.04	1.72E-01	-0.39
1300.2072@9.754205	1300.2072	9.75	NEG	RP				6.02E-01	9.89E-01	0.00	9.75E-01	0.01	2.35E-01	0.31	1.80E-01	-0.35
1300.8184@3.3257892	1300.8184	3.33	NEG	hilic				7.10E-01	2.77E-01	0.30	5.71E-01	-0.15	8.38E-01	0.06	4.29E-01	0.24
1300.8857@1.17725	1300.8857	1.18	POS	hilic				6.74E-01	9.21E-01	-0.03	8.56E-01	-0.05	1.94E-01	-0.41	5.81E-01	0.18
1301.2094@9.590948	1301.2094	9.59	NEG	RP				5.20E-01	2.25E-01	-0.32	4.92E-01	-0.21	4.49E-01	0.20	1.81E-01	-0.42
1301.2096@9.750522	1301.2096	9.75	NEG	RP				4.62E-01	1.07E-01	-0.50	7.81E-01	0.09	3.81E-01	0.21	8.79E-01	0.04
1301.9321@11.037833	1301.9321	11.04	NEG	RP				5.04E-01	1.37E-01	0.42	5.95E-02	0.54	1.76E-01	0.32	9.43E-02	0.47
1302.2635@0.36758184	1302.2635	0.37	NEG	hilic				4.61E-01	5.61E-02	0.57	9.58E-01	-0.02	6.81E-01	-0.12	5.84E-01	0.16
1302.7646@0.36810094	1302.7646	0.37	NEG	hilic				5.80E-01	8.44E-01	-0.06	1.59E-01	-0.46	1.53E-01	-0.39	9.13E-02	-0.44
1302.9061@1.2107254	1302.9061	1.21	POS	hilic				7.31E-01	4.66E-01	0.19	6.64E-01	-0.12	2.39E-01	-0.33	7.25E-01	-0.10
1303.7133@4.4632983	1303.7133	4.46	POS	hilic				8.87E-01	3.15E-01	0.30	3.20E-01	0.30	3.56E-01	0.25	3.54E-01	0.25
1303.7701@4.5525684	1303.7701	4.55	NEG	hilic				1.52E-01	8.40E-01	-0.06	8.09E-02	0.51	1.99E-01	-0.37	2.72E-01	0.29
1305.164@6.0750594	1305.1640	6.08	NEG	RP				2.35E-02	2.59E-01	0.28	8.64E-04	0.84	1.43E-01	0.33	9.81E-01	0.00
1305.4222@3.195729	1305.4222	3.20	NEG	hilic				5.96E-01	2.65E-01	0.36	8.61E-01	-0.05	7.95E-01	-0.07	1.85E-01	0.40
1305.425@3.200486	1305.4250	3.20	NEG	hilic				8.34E-01	7.62E-01	0.10	9.12E-01	-0.03	5.75E-01	-0.15	3.15E-01	0.29
1306.3@0.38653445	1306.3000	0.39	NEG	hilic				4.06E-01	5.97E-01	0.16	1.18E-01	0.45	7.50E-01	-0.09	1.06E-01	0.52
1306.9207@3.2025626	1306.9207	3.20	NEG	hilic				9.40E-01	4.78E-01	-0.23	5.95E-01	-0.16	3.06E-01	-0.28	7.00E-01	-0.11
1307.9236@3.2182498	1307.9236	3.22	NEG	hilic				6.47E-01	5.04E-01	-0.19	3.47E-01	-0.24	4.83E-01	-0.20	4.09E-02	-0.50
1308.425@3.2064507	1308.4250	3.21	NEG	hilic				7.58E-01	4.66E-01	0.24	4.98E-01	-0.18	4.54E-01	0.22	4.78E-01	0.20
1309.9694@11.035918	1309.9694	11.04	NEG	RP				9.86E-01	9.40E-01	0.02	5.61E-01	0.16	8.17E-01	0.06	7.26E-01	0.09
1310.2648@0.38870144	1310.2648	0.39	NEG	hilic				7.92E-01	7.55E-01	0.09	7.48E-01	-0.08	2.42E-01	0.33	9.91E-01	0.00
1311.2896@9.740392	1311.2896	9.74	POS	RP				5.86E-01	4.35E-01	-0.22	1.61E-01	0.41	5.12E-01	0.21	7.34E-01	0.10
1311.6263@0.5033597	1311.6263	0.50	POS	hilic				6.12E-01	6.68E-01	0.10	6.72E-02	0.52	2.26E-01	0.30	4.70E-01	0.23
1311.9937@11.036659	1311.9937	11.04	NEG	RP				2.85E-01	1.46E-02	-0.59	3.47E-01	-0.27	6.47E-01	-0.11	4.41E-02	-0.60
1312.19@9.747984	1312.1900	9.75	NEG	RP				5.51E-01	9.25E-01	-0.03	5.25E-01	0.21	3.49E-01	0.26	2.35E-01	-0.35
1313.3062@9.628231	1313.3062	9.63	POS	RP				1.44E-01	4.01E-01	0.24	5.93E-01	0.16	9.89E-01	0.00	2.56E-02	-0.66
1313.3062@9.740532	1313.3062	9.74	POS	RP				5.08E-01	4.34E-01	-0.26	1.80E-01	0.37	4.24E-01	0.23	7.39E-01	-0.10
1313.6324@0.5044644	1313.6324	0.50	POS	hilic				6.03E-01	4.79E-02	-0.50	9.91E-01	0.00	7.80E-01	-0.09	6.90E-01	0.12
1313.7408@4.41606	1313.7408	4.42	POS	hilic				7.16E-01	6.20E-01	0.15	5.02E-01	-0.17	2.06E-01	-0.36	4.32E-01	-0.25
1313.8759@10.7824135	1313.8759	10.78	NEG	RP				1.55E-01	1.18E-01	-0.38	1.68E-01	0.39	2.56E-01	0.30	5.13E-01	-0.18
1314.1877@9.589993	1314.1877	9.59	NEG	RP				8.30E-01	3.70E-01	-0.23	4.09E-01	0.22	7.50E-01	0.09	8.43E-01	-0.07
1314.1895@9.747812	1314.1895	9.75	NEG	RP				7.76E-01	8.20E-01	-0.07	6.79E-01	0.14	4.33E-01	0.22	4.07E-01	-0.25
1314.19@9.898097	1314.1900	9.90	NEG	RP				2.28E-01	1.33E-01	0.37	1.37E-02	0.69	2.12E-02	0.69	9.00E-02	0.48
1314.247@0.38818863	1314.2470	0.39	NEG	hilic				9.57E-01	8.51E-01	-0.04	5.12E-01	-0.16	7.91E-01	0.06	9.17E-01	0.03
1314.6508@0.45856896	1314.6508	0.46	POS	hilic				3.57E-01	2.45E-01	-0.33	1.80E-01	-0.37	1.51E-02	-0.65	2.29E-01	-0.31
1315.1906@9.590757	1315.1906	9.59	NEG	RP				6.08E-01	8.22E-01	0.07	3.05E-01	0.31	2.90E-01	-0.32	8.68E-01	0.06
1315.1919@9.748903	1315.1919	9.75	NEG	RP				1.17E-01	8.27E-01	0.06	1.50E-02	0.79	4.06E-02	0.61	4.45E-01	0.23
1315.887@10.782802	1315.8870	10.78	NEG	RP				7.70E-02	2.23E-01	-0.33	7.21E-02	0.51	6.14E-02	0.52	9.42E-01	0.02
1316.2754@0.38711604	1316.2754	0.39	NEG	hilic				5.86E-01	6.12E-01	0.14	2.06E-01	0.31	2.71E-01	-0.29	9.54E-01	0.02
1318.2747@0.3870431	1318.2747	0.39	NEG	hilic				4.47E-01	1.28E-01	0.46	2.23E-02	0.66	3.17E-01	0.27	1.88E-01	0.42
1319.2146@7.8475466	1319.2146	7.85	NEG	RP				4.29E-02	9.40E-01	-0.02	1.11E-02	0.69	1.13E-01	0.42	5.04E-01	-0.17
1319.9456@11.038107	1319.9456	11.04	NEG	RP				5.65E-01	1.57E-01	0.39	7.81E-02	0.53	8.41E-02	0.46	1.98E-01	0.37
1320.2852@0.38696578	1320.2852	0.39	NEG	hilic				3.74E-01	1.22E-01	0.46	1.65E-02	0.69	2.67E-01	0.31	9.81E-02	0.51
1320.287@9.433	1320.2870	9.43	NEG	RP				7.82E-01	5.35E-01	0.17	7.59E-01	0.08	9.63E-01	-0.02	3.36E-01	-0.31
1321.2896@0.3875454	1321.2896	0.39	NEG	hilic				6.06E-01	2.47E-01	0.35	9.07E-02	0.50	3.83E-01	0.23	9.35E-02	0.49

Supplementary table 2: Metabolomics analysis results

								ANOVA	Anorexia vs control		overweight vs control		obese vs control		athletes vs control	
Compound	Mass	Retention Time	Ionization mode	column	Compound_ID	MSMS_spectra	ID_level	P-FDR	P	Estimate	P	Estimate	P	Estimate	P	Estimate
1322.705@10.367645	1322.7050	10.37	NEG	RP				2.18E-01	2.07E-01	-0.24	3.23E-01	-0.19	3.06E-01	0.23	2.29E-01	0.29
1324.2675@0.38857013	1324.2675	0.39	NEG	hilic				5.45E-01	1.82E-01	0.41	4.63E-01	0.22	8.74E-01	-0.04	7.11E-02	0.50
1324.9528@11.0357685	1324.9528	11.04	NEG	RP				4.21E-01	1.14E-01	0.52	4.64E-01	0.22	2.70E-02	0.65	4.73E-01	0.22
1325.6191@0.5057902	1325.6191	0.51	POS	hilic				4.91E-01	1.75E-01	-0.38	9.63E-01	0.01	1.33E-01	-0.41	6.57E-01	0.13
1325.6309@0.50747263	1325.6309	0.51	POS	hilic				9.32E-01	3.18E-01	-0.31	6.77E-01	-0.12	4.95E-01	-0.15	7.93E-01	-0.07
1325.9622@11.036015	1325.9622	11.04	NEG	RP				3.39E-01	2.14E-01	0.38	8.29E-01	0.06	2.77E-02	0.65	9.86E-01	-0.01
1326.2618@0.39097062	1326.2618	0.39	NEG	hilic				5.43E-01	8.37E-01	-0.05	4.75E-01	-0.16	4.79E-01	0.18	1.26E-01	-0.32
1326.9705@11.03699	1326.9705	11.04	NEG	RP				4.10E-01	5.29E-01	0.20	7.33E-01	0.10	9.36E-02	0.48	3.81E-01	-0.27
1327.6368@0.5069303	1327.6368	0.51	POS	hilic				7.46E-01	3.30E-01	0.26	2.37E-01	0.34	9.29E-01	0.02	2.51E-01	0.35
1328.6858@0.45842248	1328.6858	0.46	POS	hilic				7.68E-01	8.99E-01	0.03	6.14E-01	-0.12	2.96E-01	0.30	4.85E-01	0.19
1330.253@0.3864506	1330.2530	0.39	NEG	hilic				3.50E-01	2.04E-01	0.33	7.39E-01	0.09	4.78E-01	-0.16	5.91E-02	0.50
1330.3291@10.2314	1330.3291	10.23	POS	RP				6.38E-03	8.33E-02	0.51	4.45E-02	-0.55	1.21E-02	-0.62	9.97E-01	0.00
1330.6602@0.49402267	1330.6602	0.49	POS	hilic				8.81E-01	5.62E-01	-0.17	6.72E-01	0.12	6.87E-01	0.09	5.62E-01	-0.16
1332.2563@9.62255	1332.2563	9.62	NEG	RP				7.27E-01	1.01E-01	0.44	1.42E-01	0.40	3.50E-01	0.30	2.02E-01	0.33
1332.2574@0.3856752	1332.2574	0.39	NEG	hilic				8.16E-01	1.81E-01	0.42	4.23E-01	0.25	5.72E-01	0.16	2.96E-01	0.33
1332.258@9.777736	1332.2580	9.78	NEG	RP				7.79E-01	1.24E-01	0.41	6.73E-01	0.11	3.14E-01	0.37	6.83E-01	0.12
1332.6711@0.50005335	1332.6711	0.50	POS	hilic				7.41E-01	2.27E-01	-0.30	8.85E-01	-0.04	8.95E-01	-0.04	3.95E-01	0.24
1333.8905@1.0378335	1333.8905	1.04	POS	hilic				7.29E-01	6.96E-01	-0.11	7.97E-02	-0.40	3.54E-01	-0.25	6.98E-01	-0.10
1334.7701@0.36691818	1334.7701	0.37	NEG	hilic				7.02E-01	4.19E-01	0.24	3.83E-01	-0.28	4.02E-01	-0.24	7.31E-01	-0.09
1335.2083@7.2325583	1335.2083	7.23	NEG	RP				2.45E-02	1.33E-01	-0.24	9.67E-03	0.65	6.32E-01	0.10	9.89E-01	0.00
1336.2751@0.3865431	1336.2751	0.39	NEG	hilic				8.20E-01	3.01E-01	0.29	3.53E-01	0.26	3.33E-01	0.24	1.57E-01	0.39
1337.7646@4.545775	1337.7646	4.55	NEG	hilic				6.07E-01	3.47E-01	0.28	1.45E-01	0.43	7.22E-01	-0.10	3.30E-01	0.29
1338.2823@0.38675532	1338.2823	0.39	NEG	hilic				8.81E-01	5.66E-01	-0.15	8.42E-01	-0.06	5.42E-01	0.15	5.13E-01	0.19
1339.8077@0.66942555	1339.8077	0.67	POS	hilic				6.53E-01	2.18E-01	-0.40	5.24E-01	-0.18	6.30E-02	-0.50	4.73E-01	-0.20
1340.2697@0.38637832	1340.2697	0.39	NEG	hilic				5.05E-01	1.50E-01	0.43	6.82E-02	0.57	3.44E-01	0.26	8.52E-02	0.52
1341.8267@0.66946137	1341.8267	0.67	POS	hilic				7.97E-01	2.67E-01	-0.42	8.61E-01	-0.05	2.61E-01	-0.30	6.58E-01	-0.13
1341.9233@12.722822	1341.9233	12.72	POS	RP				9.53E-01	9.11E-01	-0.03	8.59E-01	-0.04	5.72E-01	0.15	5.54E-01	0.15
1342.2795@9.627023	1342.2795	9.63	NEG	RP				5.24E-01	6.53E-01	0.14	2.61E-01	-0.29	5.94E-01	0.12	2.76E-01	0.30
1342.2863@0.3863675	1342.2863	0.39	NEG	hilic				2.96E-01	1.25E-01	0.46	1.52E-02	0.76	1.40E-01	0.39	1.05E-01	0.52
1342.8282@0.66933316	1342.8282	0.67	POS	hilic				7.68E-01	2.40E-01	-0.42	7.76E-01	-0.09	2.00E-01	-0.34	6.20E-01	-0.15
1343.2885@0.38637832	1343.2885	0.39	NEG	hilic				5.03E-01	8.88E-02	0.50	1.01E-01	0.51	3.54E-01	0.27	5.93E-02	0.55
1344.6646@0.50779283	1344.6646	0.51	POS	hilic				5.44E-01	8.91E-01	0.04	6.93E-01	0.10	2.30E-01	-0.33	3.10E-01	0.29
1346.2603@9.609282	1346.2603	9.61	NEG	RP				9.10E-01	2.99E-01	0.26	7.21E-01	0.09	7.10E-01	0.11	3.00E-01	0.30
1346.2603@9.945599	1346.2603	9.95	NEG	RP				6.31E-01	4.81E-01	-0.22	1.97E-01	-0.33	4.15E-02	-0.49	2.77E-01	-0.31
1346.2607@0.38601708	1346.2607	0.39	NEG	hilic				9.23E-01	8.12E-01	0.07	9.28E-01	-0.02	9.82E-01	-0.01	4.06E-01	-0.27
1346.2618@9.785274	1346.2618	9.79	NEG	RP				5.82E-01	1.57E-01	0.42	4.82E-01	0.20	7.45E-02	0.46	8.52E-02	0.49
1346.6873@0.5032825	1346.6873	0.50	POS	hilic				8.34E-01	3.79E-01	-0.18	7.58E-01	0.08	9.27E-01	0.02	3.95E-01	0.20
1348.4049@3.206311	1348.4049	3.21	NEG	hilic				7.28E-01	4.30E-01	0.23	7.39E-01	0.09	4.48E-01	0.21	1.00E-01	0.49
1348.9604@11.038995	1348.9604	11.04	NEG	RP				1.35E-01	5.04E-01	-0.20	1.05E-01	-0.51	3.98E-01	0.21	3.73E-02	-0.63
1350.2028@9.759471	1350.2028	9.76	NEG	RP				4.54E-01	6.97E-01	-0.11	4.57E-01	0.21	2.80E-01	0.37	2.10E-01	-0.31
1350.4001@3.204222	1350.4001	3.20	NEG	hilic				3.99E-01	4.38E-01	-0.22	1.34E-01	0.43	3.67E-01	0.25	1.47E-01	0.39
1351.9576@11.039035	1351.9576	11.04	NEG	RP				3.78E-01	2.41E-01	0.30	5.05E-01	0.20	1.51E-02	0.69	8.92E-01	0.04
1355.2653@0.3850598	1355.2653	0.39	NEG	hilic				9.58E-01	7.35E-01	0.10	6.56E-01	-0.14	6.62E-01	-0.11	6.07E-01	-0.15
1356.2758@0.38570774	1356.2758	0.39	NEG	hilic				8.82E-01	2.70E-01	0.36	8.06E-01	0.07	4.54E-01	0.19	4.76E-01	0.19
1357.8191@0.7757637	1357.8191	0.78	POS	hilic				1.65E-01	2.57E-01	-0.30	3.84E-01	-0.22	3.91E-04	-0.76	3.29E-01	-0.26
1358.278@0.38676137	1358.2780	0.39	NEG	hilic				3.71E-01	1.36E-01	0.41	1.74E-01	0.40	7.65E-01	0.08	1.89E-02	0.67
1359.2769@0.38717386	1359.2769	0.39	NEG	hilic				3.97E-01	3.02E-02	0.57	3.95E-02	0.55	4.26E-01	0.24	6.48E-02	0.58
1359.6666@0.56159145	1359.6666	0.56	POS	hilic				5.93E-01	1.59E-01	-0.42	7.99E-01	-0.07	3.14E-01	-0.29	6.30E-01	0.15
1360.4283@4.549422	1360.4283	4.55	NEG	hilic				5.36E-01	2.74E-01	0.27	6.48E-01	0.10	4.15E-02	0.47	1.65E-01	0.33
1362.9402@10.782949	1362.9402	10.78	NEG	RP				3.72E-01	1.10E-01	-0.35	8.18E-01	0.06	2.94E-01	0.29	3.45E-01	-0.21
1363.799@0.670047	1363.7990	0.67	POS	hilic				9.16E-01	4.85E-01	-0.22	5.86E-01	-0.15	2.99E-01	-0.27	3.70E-01	-0.26
1364.2188@9.608007	1364.2188	9.61	NEG	RP				5.38E-01	2.37E-01	-0.38	5.06E-01	-0.17	6.60E-01	0.12	1.22E-01	-0.46
1364.2194@9.766412	1364.2194	9.77	NEG	RP				1.55E-01	1.92E-02	-0.71	9.88E-01	0.00	8.16E-01	0.06	9.79E-02	-0.51
1365.2216@9.763299	1365.2216	9.76	NEG	RP				1.84E-01	6.14E-01	-0.13	1.26E-01	0.46	3.22E-02	0.65	7.58E-01	0.08
1365.8221@0.67040944	1365.8221	0.67	POS	hilic				5.66E-01	2.14E-01	-0.44	9.12E-01	-0.03	8.63E-02	-0.46	7.67E-01	-0.09
1366.2747@0.3668455	1366.2747	0.37	NEG	hilic				7.68E-01	5.25E-01	0.20	3.89E-01	-0.26	4.92E-01	-0.21	9.32E-01	0.02
1366.7762@0.3661905	1366.7762	0.37	NEG	hilic				6.35E-01	9.23E-01	-0.03	1.03E-01	-0.51	3.16E-01	-0.30	4.78E-01	-0.20
1366.83@0.6704227	1366.8300	0.67	POS	hilic				5.48E-01	3.44E-01	-0.28	6.05E-01	-0.13	2.36E-02	-0.55	5.05E-01	-0.18
1368.8387@0.6702382	1368.8387	0.67	POS	hilic				8.84E-01	7.32E-01	-0.11	8.05E-01	0.07	2.74E-01	-0.28	7.89E-01	-0.08
1369.6508@0.49209893	1369.6508	0.49	POS	hilic				9.94E-01	9.17E-01	0.03	7.50E-01	-0.09	8.04E-01	0.06	9.96E-01	0.00
1369.856@0.6705278	1369.8560	0.67	POS	hilic				5.92E-01	7.26E-01	-0.09	3.33E-01	-0.22	2.81E-02	-0.42	2.52E-01	-0.26
1370.3134@0.38626722	1370.3134	0.39	NEG	hilic				5.53E-01	3.36E-01	0.28	4.32E-02	0.60	5.30E-01	0.17	2.30E-01	0.38
1370.3145@9.940852	1370.3145	9.94	NEG	RP				2.44E-01	1.58E-02	0.76	1.06E-02	0.67	1.15E-01	0.45	9.75E-02	0.49
1371.3167@0.38621566	1371.3167	0.39	NEG	hilic				8.48E-01	2.36E-01	0.32	9.91E-01	0.00	8.90E-01	0.03	5.99E-01	0.15
1371.6646@0.56510764	1371.6646	0.57	POS	hilic				6.61E-01	9.53E-01	-0.02	8.40E-01	-0.05	6.67E-01	-0.12	2.05E-01	0.40
1371.7021@4.4630523	1371.7021	4.46	POS	hilic				9.82E-01	6.36E-01	0.13	9.37E-01	-0.02	7.83E-01	-0.06	9.01E-01	0.03
1372.6746@0.4993676	1372.6746	0.50	POS	hilic				5.95E-01	5.83E-01	0.13	5.19E-01	-0.18	8.34E-02	-0.41	4.97E-01	-0.19
1373.0707@0.9253797	1373.0707	0.93	POS	hilic				3.58E-01	9.58E-01	-0.01	3.31E-02	0.52	9.57E-02	0.45	6.90E-01	0.12
1373.6779@0.56386054	1373.6779	0.56	POS	hilic				6.42E-01	8.68E-01	-0.05	1.00E+00	0.00	1.47E-01	0.41	3.36E-01	0.28
1374.2795@0.38741812	1374.2795	0.39	NEG	hilic				9.43E-01	5.39E-01	0.15	7.41E-01	0.09	2.97E-01	0.24	4.17E-01	0.19
1374.9666@10.513289	1374.9666	10.51	NEG	RP				1.62E-01	5.35E-02	-0.49	5.33E-01	-0.18	1.47E-01	0.42	6.25E-01	-0.15
1375.0883@0.923305	1375.0883	0.92	POS	hilic				8.39E-02	6.80E-01	-0.12	6.74E-03	0.68	4.20E-02	0.59	9.45E-01	-0.02
1375.3@9.752628	1375.3000	9.75	POS	RP				2.04E-01	1.02E-01	-0.44	8.44E-02	0.48	8.59E-01	-0.04	6.49E-01	0.15

Supplementary table 2: Metabolomics analysis results

Compound	Mass	Retention Time	Ionization mode	column	Compound_ID	MSMS_spectra	ID_level	ANOVA	Anorexia vs control		overweight vs control		obese vs control		athletes vs control	
								P-FDR	P	Estimate	P	Estimate	P	Estimate	P	Estimate
1376.2076@9.758414	1376.2076	9.76	NEG	RP				8.49E-01	7.90E-01	0.08	5.18E-01	0.20	2.19E-01	0.38	9.04E-01	0.03
1377.3177@9.641225	1377.3177	9.64	POS	RP				2.24E-01	8.86E-01	-0.04	2.66E-02	0.61	6.12E-01	-0.12	6.19E-01	-0.17
1377.3188@9.753409	1377.3188	9.75	POS	RP				1.41E-01	2.44E-01	-0.34	4.38E-02	0.61	7.73E-01	-0.08	9.62E-01	-0.02
1378.2004@9.7593155	1378.2004	9.76	NEG	RP				1.66E-01	8.24E-01	-0.07	4.40E-01	0.23	3.39E-03	0.79	2.24E-01	0.36
1378.2007@9.60727	1378.2007	9.61	NEG	RP				9.68E-01	5.85E-01	0.16	4.16E-01	0.25	7.85E-01	0.08	6.86E-01	0.13
1378.2017@9.892442	1378.2017	9.89	NEG	RP				3.61E-01	4.91E-01	-0.20	1.69E-01	0.40	2.51E-01	0.33	4.58E-01	-0.20
1379.1993@9.609572	1379.1993	9.61	NEG	RP				8.01E-01	6.26E-01	-0.15	3.87E-01	0.21	9.59E-01	-0.02	4.07E-01	-0.23
1379.2046@9.748506	1379.2046	9.75	NEG	RP				5.08E-02	2.49E-01	-0.32	3.51E-01	-0.22	2.20E-02	0.67	7.24E-01	-0.09
1379.2054@9.795752	1379.2054	9.80	NEG	RP				7.86E-01	5.08E-01	-0.18	5.31E-01	0.15	4.07E-01	-0.25	5.21E-01	-0.18
1381.33@3.0411038	1381.3300	3.04	POS	hilic				4.81E-01	7.42E-01	0.11	9.90E-01	0.00	1.42E-01	-0.44	3.62E-01	0.26
1381.3333@3.041166	1381.3333	3.04	POS	hilic				3.91E-01	7.96E-01	-0.09	8.41E-01	-0.06	8.36E-02	-0.50	3.35E-01	0.28
1381.7332@4.413974	1381.7332	4.41	POS	hilic				1.20E-01	1.31E-02	0.78	1.93E-02	0.62	9.76E-01	-0.01	2.94E-01	0.29
1382.284@0.38626313	1382.2840	0.39	NEG	hilic				6.09E-01	4.62E-01	0.21	2.17E-01	0.38	3.22E-01	0.24	6.18E-02	0.58
1383.0898@4.550035	1383.0898	4.55	NEG	hilic				6.60E-01	5.77E-01	0.12	1.28E-01	0.40	1.21E-01	0.38	2.47E-01	0.28
1383.3344@3.0452983	1383.3344	3.05	POS	hilic				3.47E-01	7.82E-01	-0.09	5.22E-01	-0.18	6.52E-02	-0.50	2.45E-01	0.30
1384.2981@9.439139	1384.2981	9.44	NEG	RP				2.24E-01	4.27E-01	0.22	8.21E-01	0.07	6.98E-01	0.10	3.93E-02	-0.55
1384.299@0.38627264	1384.2990	0.39	NEG	hilic				5.41E-01	2.90E-01	0.34	7.70E-02	0.51	4.93E-01	0.18	7.71E-02	0.54
1385.3022@0.38706034	1385.3022	0.39	NEG	hilic				3.24E-01	6.60E-02	0.60	1.78E-02	0.70	1.03E-01	0.42	1.31E-01	0.51
1388.2773@0.38643965	1388.2773	0.39	NEG	hilic				6.47E-01	5.12E-02	0.60	3.21E-01	0.29	3.01E-01	0.28	3.22E-01	0.29
1389.1008@0.91077137	1389.1008	0.91	POS	hilic				6.37E-01	9.19E-01	0.03	7.54E-02	0.42	2.39E-01	0.31	7.44E-01	0.09
1389.3846@3.1988974	1389.3846	3.20	NEG	hilic				6.96E-01	2.84E-01	0.34	4.68E-01	0.20	1.54E-01	0.38	1.38E-01	0.50
1389.3857@3.1921444	1389.3857	3.19	NEG	hilic				5.99E-01	3.35E-01	0.33	9.70E-01	0.01	5.56E-01	0.17	6.14E-02	0.53
1389.8062@0.7449493	1389.8062	0.74	POS	hilic				1.02E-01	1.09E-01	-0.39	3.31E-01	-0.25	9.99E-04	-0.74	9.42E-01	0.02
1389.8159@0.6976501	1389.8159	0.70	POS	hilic				6.06E-01	3.44E-01	-0.25	3.62E-01	-0.22	5.11E-02	-0.47	8.46E-01	-0.05
1390.7198@3.2018197	1390.7198	3.20	NEG	hilic				2.61E-01	3.14E-02	0.63	1.83E-02	0.54	6.86E-02	0.56	1.30E-02	0.60
1390.8864@3.1986434	1390.8864	3.20	NEG	hilic				8.63E-01	4.30E-01	0.27	9.80E-01	0.01	5.67E-01	0.16	6.36E-01	-0.14
1391.9352@11.037355	1391.9352	11.04	NEG	RP				8.18E-01	1.14E-01	0.39	5.60E-01	0.16	2.45E-01	0.35	5.58E-01	0.17
1392.2773@0.38312727	1392.2773	0.38	NEG	hilic				7.21E-01	1.18E-01	0.46	6.60E-01	0.14	9.79E-01	-0.01	2.92E-01	0.29
1394.263@0.38642856	1394.2630	0.39	NEG	hilic				3.80E-01	6.23E-01	-0.11	1.62E-01	0.34	3.64E-01	0.18	6.46E-02	0.45
1396.2673@9.637831	1396.2673	9.64	NEG	RP				3.05E-01	4.49E-01	-0.17	7.62E-01	-0.06	8.20E-01	0.06	8.63E-02	0.48
1396.2686@0.38308108	1396.2686	0.38	NEG	hilic				7.24E-01	1.28E-01	0.45	6.73E-01	0.13	9.64E-01	-0.01	2.75E-01	0.29
1396.2706@9.78173	1396.2706	9.78	NEG	RP				6.08E-01	2.45E-01	0.30	6.07E-02	0.51	8.39E-01	0.07	9.35E-01	0.02
1397.0667@0.90896016	1397.0667	0.91	POS	hilic				2.88E-01	7.07E-01	-0.09	6.29E-02	0.55	8.44E-02	0.50	5.66E-01	0.16
1397.275@0.37740904	1397.2750	0.38	NEG	hilic				7.44E-01	3.77E-01	0.27	6.59E-01	-0.14	3.61E-01	-0.26	8.81E-01	0.04
1398.7823@0.3663684	1398.7823	0.37	NEG	hilic				7.18E-01	9.81E-01	0.01	1.81E-01	-0.41	3.01E-01	-0.30	7.28E-01	-0.09
1400.29@0.38645837	1400.2900	0.39	NEG	hilic				5.57E-01	9.56E-01	0.01	6.43E-01	0.12	2.92E-01	-0.24	8.95E-02	-0.36
1401.6741@0.5328001	1401.6741	0.53	POS	hilic				8.02E-01	2.81E-01	-0.35	9.23E-01	-0.02	4.99E-01	-0.22	8.27E-01	0.06
1402.295@0.3863332	1402.2950	0.39	NEG	hilic				2.95E-02	1.24E-01	0.35	9.81E-02	0.45	1.35E-01	0.34	2.57E-04	1.01
1402.6832@0.48183146	1402.6832	0.48	POS	hilic				6.74E-01	7.53E-01	-0.09	6.10E-01	-0.15	2.46E-01	-0.34	4.43E-01	0.22
1403.1198@0.89649963	1403.1198	0.90	POS	hilic				4.36E-01	4.44E-01	-0.24	3.92E-02	0.50	6.03E-01	0.17	8.70E-01	0.05
1403.69@0.5414575	1403.6900	0.54	POS	hilic				5.08E-01	1.37E-01	-0.41	7.81E-01	-0.07	8.42E-01	0.06	3.43E-01	0.27
1404.2823@0.38628823	1404.2823	0.39	NEG	hilic				5.20E-01	1.41E-01	0.44	1.08E-01	0.49	7.12E-01	0.10	9.76E-02	0.48
1404.6997@0.51765114	1404.6997	0.52	POS	hilic				8.80E-01	9.61E-01	0.01	9.13E-01	-0.03	2.14E-01	-0.27	7.66E-01	-0.07
1405.7542@4.541684	1405.7542	4.54	NEG	hilic				3.06E-01	5.13E-02	0.61	4.46E-02	0.63	4.85E-01	0.16	6.90E-02	0.48
1406.1353@0.89287347	1406.1353	0.89	POS	hilic				6.65E-01	5.24E-01	-0.19	1.11E-01	0.39	8.36E-01	0.07	6.33E-01	0.14
1406.295@9.788089	1406.2950	9.79	NEG	RP				8.01E-01	8.49E-01	-0.06	1.83E-01	0.36	3.88E-01	0.24	7.04E-01	0.12
1406.2979@0.38628203	1406.2979	0.39	NEG	hilic				2.75E-01	1.14E-01	0.48	1.46E-02	0.74	2.84E-01	0.29	6.91E-02	0.56
1407.3011@0.38618916	1407.3011	0.39	NEG	hilic				3.00E-01	3.04E-01	0.31	6.32E-02	0.56	5.93E-01	0.15	1.81E-02	0.69
1410.2723@9.783577	1410.2723	9.78	NEG	RP				4.10E-01	2.91E-01	0.31	8.00E-03	0.62	5.23E-01	0.18	1.82E-01	0.39
1410.274@0.38488886	1410.2740	0.38	NEG	hilic				8.12E-01	3.76E-01	0.30	8.98E-01	0.04	3.63E-01	0.24	1.68E-01	0.37
1414.2166@9.770324	1414.2166	9.77	NEG	RP				3.11E-01	9.18E-01	-0.03	6.22E-01	0.14	1.03E-01	0.47	2.47E-01	-0.35
1414.6898@0.5060727	1414.6898	0.51	POS	hilic				7.15E-01	9.63E-01	-0.01	4.92E-01	0.16	1.08E-01	0.40	7.98E-01	0.06
1415.691@0.5468426	1415.6910	0.55	POS	hilic				6.85E-01	1.72E-01	-0.41	9.94E-01	0.00	9.99E-01	0.00	5.70E-01	0.17
1416.7106@0.50501394	1416.7106	0.51	POS	hilic				5.70E-01	1.10E-01	-0.38	6.72E-01	0.12	5.14E-01	0.17	7.61E-01	0.08
1417.701@0.546125	1417.7010	0.55	POS	hilic				6.98E-01	5.31E-01	0.17	1.03E-01	0.45	1.79E-01	0.39	5.25E-01	0.19
1418.7042@0.50962967	1418.7042	0.51	POS	hilic				7.50E-01	6.92E-01	-0.11	8.95E-01	-0.03	1.04E-01	-0.43	8.28E-01	-0.06
1418.7053@3.2038474	1418.7053	3.20	NEG	hilic				2.70E-01	1.25E-01	-0.48	3.02E-02	-0.57	4.68E-02	-0.57	1.21E-02	-0.66
1419.2762@0.38121355	1419.2762	0.38	NEG	hilic				1.43E-01	3.51E-03	0.83	8.31E-01	-0.06	8.27E-01	0.06	8.26E-01	0.06
1420.2874@0.38371265	1420.2874	0.38	NEG	hilic				8.20E-01	1.40E-01	0.39	3.03E-01	0.29	5.67E-01	0.14	7.76E-01	0.08
1422.2913@0.3859	1422.2913	0.39	NEG	hilic				8.48E-01	2.48E-01	0.34	7.58E-01	-0.09	8.37E-01	0.06	7.69E-01	0.08
1423.0785@0.90009797	1423.0785	0.90	POS	hilic				4.60E-01	3.12E-01	-0.32	9.46E-02	0.39	4.84E-01	0.22	9.09E-01	0.04
1423.2852@9.78196	1423.2852	9.78	NEG	RP				9.25E-02	2.66E-04	0.87	5.21E-01	0.16	1.27E-01	0.46	2.32E-02	0.65
1423.2874@0.38570178	1423.2874	0.39	NEG	hilic				9.65E-01	5.63E-01	0.18	9.45E-01	0.02	9.37E-01	0.02	4.61E-01	0.21
1423.9814@6.188151	1423.9814	6.19	POS	hilic				9.02E-01	2.92E-01	0.34	4.23E-01	0.23	4.86E-01	0.21	3.45E-01	0.27
1426.9976@0.941403	1426.9976	0.94	POS	hilic				1.93E-01	6.01E-01	0.16	6.07E-02	0.49	1.76E-02	0.56	7.15E-01	-0.08
1428.1185@0.8920383	1428.1185	0.89	POS	hilic				9.16E-01	3.97E-01	0.17	3.16E-01	0.20	8.64E-01	0.03	7.42E-01	0.07
1428.231@9.62368	1428.2310	9.62	NEG	RP				5.85E-01	1.61E-01	-0.38	8.60E-01	-0.05	3.81E-01	0.27	4.47E-01	-0.21
1428.232@9.773839	1428.2320	9.77	NEG	RP				1.61E-01	2.51E-02	-0.62	5.19E-01	0.19	5.74E-01	0.16	1.93E-01	-0.37
1428.2823@0.36626607	1428.2823	0.37	NEG	hilic				8.61E-01	5.43E-01	-0.20	2.26E-01	-0.38	3.61E-01	-0.27	5.21E-01	-0.17
1429.1316@0.89063233	1429.1316	0.89	POS	hilic				5.90E-02	8.28E-02	-0.43	1.09E-02	0.59	3.35E-01	0.30	9.32E-01	-0.03
1429.2354@9.623666	1429.2354	9.62	NEG	RP				8.18E-01	2.38E-01	0.37	3.38E-01	0.26	2.19E-01	0.33	2.62E-01	0.36
1430.131@0.89160466	1430.1310	0.89	POS	hilic				4.79E-02	9.24E-01	0.02	2.77E-03	0.66	1.96E-02	0.60	1.30E-01	0.35
1430.288@0.3662818	1430.2880	0.37	NEG	hilic				5.92E-01	9.67E-01	-0.01	9.37E-02	-0.52	2.38E-01	-0.35	3.22E-01	-0.26

Supplementary table 2: Metabolomics analysis results

								ANOVA	Anorexia vs control		overweight vs control		obese vs control		athletes vs control	
Compound	Mass	Retention Time	Ionization mode	column	Compound_ID	MSMS_spectra	ID_level	P-FDR	P	Estimate	P	Estimate	P	Estimate	P	Estimate
1430.7906@0.3662857	1430.7906	0.37	NEG	hilic				6.02E-01	3.66E-01	0.28	3.55E-02	-0.28	3.84E-01	-0.26	9.99E-01	0.00
1431.1462@0.89134663	1431.1462	0.89	POS	hilic				1.13E-01	3.49E-01	-0.22	5.21E-02	0.50	5.19E-02	0.55	6.10E-01	0.13
1432.3668@3.1970434	1432.3668	3.20	NEG	hilic				8.96E-01	4.37E-01	-0.27	9.78E-01	0.01	8.54E-01	0.05	6.86E-01	0.12
1432.8665@3.1977422	1432.8665	3.20	NEG	hilic				5.89E-01	1.45E-01	0.44	2.11E-01	0.34	8.76E-01	-0.04	2.76E-01	0.31
1433.0398@0.6608204	1433.0398	0.66	POS	hilic				8.92E-01	2.82E-01	0.24	4.77E-01	0.17	8.77E-01	0.04	9.91E-01	0.00
1433.9149@0.8258948	1433.9149	0.83	POS	hilic				4.60E-01	6.20E-02	-0.36	3.43E-01	-0.20	1.23E-01	-0.30	8.55E-02	-0.34
1434.3228@9.943443	1434.3228	9.94	NEG	RP				1.29E-01	9.41E-01	0.02	4.11E-01	-0.18	4.67E-01	0.18	2.45E-02	0.65
1434.326@0.38617423	1434.3260	0.39	NEG	hilic				5.40E-01	1.51E-01	0.44	9.22E-02	0.48	4.28E-01	0.21	8.05E-02	0.54
1434.3643@3.2017548	1434.3643	3.20	NEG	hilic				8.94E-01	4.89E-01	0.21	7.80E-01	-0.07	4.19E-01	0.23	9.99E-01	0.00
1438.295@0.38594276	1438.2950	0.39	NEG	hilic				5.56E-01	7.79E-01	0.08	5.30E-02	0.49	4.68E-01	0.16	9.78E-01	0.01
1439.3123@9.773429	1439.3123	9.77	POS	RP				5.11E-01	4.83E-01	0.22	2.46E-02	0.60	3.02E-01	0.33	7.13E-01	0.09
1440.2104@9.774514	1440.2104	9.77	NEG	RP				3.84E-01	7.01E-01	-0.12	1.08E-01	0.45	7.40E-01	0.10	3.31E-01	-0.29
1440.2231@9.625583	1440.2231	9.63	NEG	RP				9.14E-01	8.71E-01	-0.04	5.17E-01	0.18	4.96E-01	0.22	8.47E-01	-0.05
1441.3304@9.661743	1441.3304	9.66	POS	RP				5.55E-01	7.48E-02	0.52	2.10E-01	0.35	1.06E-01	0.47	7.54E-01	0.09
1441.3322@9.771974	1441.3322	9.77	POS	RP				6.07E-01	4.73E-01	0.23	7.24E-02	0.51	1.54E-01	0.44	6.91E-01	0.12
1442.2122@9.625805	1442.2122	9.63	NEG	RP				9.88E-01	7.51E-01	0.08	7.15E-01	0.11	5.22E-01	0.20	7.77E-01	0.08
1442.2133@9.7709675	1442.2133	9.77	NEG	RP				2.40E-01	5.25E-01	0.18	2.07E-01	0.35	1.63E-02	0.81	5.64E-01	0.14
1442.2585@0.38720518	1442.2585	0.39	NEG	hilic				2.66E-01	8.43E-01	-0.04	1.85E-01	0.25	3.51E-02	0.51	1.09E-01	0.41
1443.216@9.77272	1443.2160	9.77	NEG	RP				2.20E-01	4.02E-01	-0.21	9.96E-01	0.00	2.12E-01	0.40	8.44E-02	-0.49
1443.2168@9.676863	1443.2168	9.68	NEG	RP				6.68E-01	5.91E-01	-0.16	7.44E-01	-0.09	8.38E-01	0.07	8.09E-02	-0.46
1443.9471@10.122712	1443.9471	10.12	NEG	RP				1.11E-03	3.43E-02	0.69	9.54E-02	-0.35	9.60E-03	-0.67	1.65E-01	0.37
1444.2925@0.38610944	1444.2925	0.39	NEG	hilic				8.45E-01	1.87E-01	0.35	2.91E-01	0.28	5.48E-01	0.14	5.83E-01	0.14
1446.0272@3.202825	1446.0272	3.20	NEG	hilic				4.52E-01	5.63E-01	-0.18	2.03E-02	-0.57	5.66E-01	-0.15	7.77E-01	-0.07
1446.295@0.38607475	1446.2950	0.39	NEG	hilic				4.29E-01	7.25E-02	0.51	5.90E-02	0.54	5.67E-01	0.16	8.55E-02	0.52
1446.6935@3.2091687	1446.6935	3.21	NEG	hilic				8.34E-01	7.85E-01	0.08	7.31E-01	-0.09	8.73E-01	-0.04	2.83E-01	-0.33
1446.7231@0.5030909	1446.7231	0.50	POS	hilic				7.15E-01	1.98E-01	0.31	5.74E-01	0.14	8.68E-01	-0.04	5.49E-01	-0.13
1447.1051@0.9842161	1447.1051	0.98	NEG	hilic				7.96E-01	4.80E-01	0.20	1.32E-01	0.37	7.33E-01	0.09	8.91E-01	0.04
1447.1075@11.657242	1447.1075	11.66	NEG	RP				1.79E-01	6.07E-01	-0.13	1.29E-01	0.41	3.93E-02	0.58	7.14E-01	-0.11
1447.3538@3.2050824	1447.3538	3.21	NEG	hilic				5.85E-02	1.48E-02	0.70	8.74E-03	0.64	1.81E-02	0.64	1.39E-03	0.89
1448.3116@0.38617265	1448.3116	0.39	NEG	hilic				5.08E-01	1.01E-01	0.49	1.51E-01	0.44	8.27E-01	0.06	9.99E-02	0.46
1449.1215@0.9841028	1449.1215	0.98	NEG	hilic				9.32E-01	7.22E-01	0.10	4.31E-01	0.19	8.81E-01	0.04	7.19E-01	-0.10
1449.3145@0.38616666	1449.3145	0.39	NEG	hilic				7.43E-01	2.53E-01	0.36	2.91E-01	0.32	8.84E-01	0.04	2.00E-01	0.38
1449.716@4.4149904	1449.7160	4.41	POS	hilic				5.22E-01	9.09E-01	0.03	2.09E-01	0.30	3.77E-01	-0.26	3.35E-01	-0.30
1451.112@0.8893588	1451.1120	0.89	POS	hilic				7.70E-02	1.84E-01	-0.23	1.44E-01	0.33	4.43E-02	0.57	7.93E-01	0.06
1451.1383@11.940726	1451.1383	11.94	NEG	RP				4.95E-01	5.34E-01	-0.17	1.01E-01	0.42	7.92E-01	0.07	5.77E-01	-0.15
1452.2924@0.38549992	1452.2924	0.39	NEG	hilic				6.71E-01	9.22E-02	0.50	8.31E-01	0.05	5.00E-01	0.18	2.71E-01	0.34
1455.0577@0.6604782	1455.0577	0.66	POS	hilic				9.74E-01	6.71E-01	0.09	8.36E-01	-0.04	7.56E-01	0.07	6.08E-01	0.14
1456.2668@9.888753	1456.2668	9.89	NEG	RP				3.83E-01	3.58E-01	-0.27	9.67E-01	0.01	8.07E-01	-0.06	3.55E-02	-0.65
1457.08@0.66081256	1457.0800	0.66	POS	hilic				8.57E-01	4.40E-01	0.18	1.78E-01	0.31	3.33E-01	0.26	7.46E-01	0.09
1458.1158@0.8954119	1458.1158	0.90	POS	hilic				8.92E-01	9.70E-01	0.01	4.41E-01	-0.20	4.80E-01	-0.18	7.69E-01	0.08
1458.2842@0.38443393	1458.2842	0.38	NEG	hilic				9.81E-01	8.63E-01	0.04	9.31E-01	-0.02	9.71E-01	-0.01	5.11E-01	0.15
1459.0612@0.6595979	1459.0612	0.66	POS	hilic				8.30E-01	5.16E-01	0.20	5.70E-01	0.15	6.36E-01	0.14	1.50E-01	0.43
1460.2806@0.38024777	1460.2806	0.38	NEG	hilic				6.86E-01	1.26E-01	0.45	4.67E-01	0.24	6.87E-02	0.47	2.33E-01	0.38
1460.284@9.786242	1460.2840	9.79	NEG	RP				4.54E-01	1.33E-02	0.56	1.23E-01	0.47	7.99E-01	0.07	2.15E-01	0.41
1460.7153@0.5041906	1460.7153	0.50	POS	hilic				5.43E-01	1.45E-01	-0.46	9.36E-02	-0.48	5.91E-01	-0.14	6.89E-01	-0.11
1461.0732@0.65625435	1461.0732	0.66	POS	hilic				7.34E-01	8.95E-01	0.04	7.97E-01	0.07	6.31E-01	0.14	1.55E-01	0.48
1461.2856@0.3771363	1461.2856	0.38	NEG	hilic				8.02E-01	4.14E-01	0.25	5.63E-01	-0.19	9.96E-01	0.00	5.04E-01	0.19
1462.7948@0.3662386	1462.7948	0.37	NEG	hilic				7.44E-01	6.50E-01	0.13	2.60E-01	-0.35	5.72E-01	-0.17	5.79E-01	-0.14
1463.0931@0.6594208	1463.0931	0.66	POS	hilic				8.05E-01	3.97E-01	0.26	4.86E-01	0.20	3.94E-01	0.24	1.49E-01	0.45
1464.097@0.65899986	1464.0970	0.66	POS	hilic				7.31E-01	7.67E-01	0.10	4.35E-01	0.21	5.98E-01	0.15	7.60E-02	0.50
1465.2961@3.039991	1465.2961	3.04	POS	hilic				9.31E-01	8.05E-01	-0.07	7.58E-01	-0.09	8.04E-01	-0.08	3.96E-01	0.20
1465.2968@3.039609	1465.2968	3.04	POS	hilic				7.46E-01	3.66E-01	-0.26	3.69E-01	-0.27	5.00E-01	-0.22	5.31E-01	0.16
1467.2953@3.0444734	1467.2953	3.04	POS	hilic				6.99E-01	6.68E-01	-0.13	5.53E-01	-0.18	8.60E-01	0.06	1.55E-01	0.34
1468.2946@0.38609904	1468.2946	0.39	NEG	hilic				5.44E-01	1.49E-01	0.44	1.08E-01	0.49	3.36E-01	0.27	5.95E-02	0.56
1470.3055@9.799037	1470.3055	9.80	NEG	RP				8.01E-01	8.49E-01	-0.06	3.54E-01	0.25	8.97E-01	0.04	4.35E-01	-0.21
1470.3105@0.38610256	1470.3105	0.39	NEG	hilic				3.82E-01	1.21E-01	0.49	2.57E-02	0.68	1.55E-01	0.38	8.75E-02	0.53
1471.0785@0.652488	1471.0785	0.65	POS	hilic				7.03E-01	6.51E-01	0.12	1.42E-01	0.36	2.25E-01	0.33	1.38E-01	0.41
1471.3138@0.38609904	1471.3138	0.39	NEG	hilic				6.74E-01	1.97E-01	0.39	2.73E-01	0.35	7.46E-01	0.09	1.08E-01	0.46
1472.7086@0.51327527	1472.7086	0.51	POS	hilic				2.79E-01	5.18E-02	-0.60	6.35E-01	0.12	5.30E-01	-0.17	5.25E-01	0.19
1473.1049@0.65112805	1473.1049	0.65	POS	hilic				6.21E-01	1.65E-01	0.40	8.97E-01	-0.03	3.40E-01	0.26	2.00E-01	0.37
1473.3466@3.1995764	1473.3466	3.20	NEG	hilic				5.33E-01	2.98E-01	0.30	7.08E-01	-0.10	1.10E-01	0.45	2.37E-01	0.35
1473.3488@3.192372	1473.3488	3.19	NEG	hilic				7.55E-01	3.05E-01	0.29	9.70E-01	0.01	1.48E-01	0.39	3.58E-01	0.30
1473.7393@4.537282	1473.7393	4.54	NEG	hilic				7.03E-01	5.63E-01	0.18	5.35E-01	-0.20	2.98E-01	-0.25	4.90E-01	0.19
1474.0126@3.1974604	1474.0126	3.20	NEG	hilic				9.75E-01	4.63E-01	0.24	6.34E-01	0.14	7.69E-01	0.09	6.01E-01	0.13
1474.2849@9.787587	1474.2849	9.79	NEG	RP				7.40E-01	8.84E-01	0.04	4.46E-01	0.16	1.62E-01	0.37	2.93E-01	0.26
1474.2852@0.38094863	1474.2852	0.38	NEG	hilic				9.36E-01	2.98E-01	0.28	9.44E-01	-0.02	9.61E-01	0.01	9.74E-01	0.01
1474.6794@3.2028804	1474.6794	3.20	NEG	hilic				7.83E-01	6.07E-01	0.14	3.31E-01	-0.24	6.54E-01	-0.11	3.91E-01	-0.24
1474.6809@3.19383	1474.6809	3.19	NEG	hilic				4.29E-01	3.74E-02	0.65	2.08E-01	0.33	1.07E-01	0.45	7.07E-02	0.55
1474.7285@0.5150434	1474.7285	0.52	POS	hilic				9.47E-01	6.92E-01	-0.10	5.97E-01	0.13	6.69E-01	0.12	8.64E-01	-0.04
1474.8466@3.2068188	1474.8466	3.21	NEG	hilic				7.12E-01	8.90E-02	0.51	2.59E-01	0.30	5.05E-01	0.22	1.87E-01	0.34
1475.1045@0.66045946	1475.1045	0.66	POS	hilic				2.37E-01	4.12E-02	-0.43	8.76E-01	-0.04	1.09E-02	-0.53	1.64E-01	-0.36
1475.3455@3.2005274	1475.3455	3.20	NEG	hilic				7.02E-01	7.35E-01	0.10	9.11E-01	0.03	7.23E-01	0.10	1.02E-01	0.50
1475.8431@3.205164	1475.8431	3.21	NEG	hilic				7.98E-01	3.71E-01	-0.24	2.27E-01	-0.29	8.54E-01	0.05	8.82E-01	-0.04

Supplementary table 2: Metabolomics analysis results

Compound	Mass	Retention Time	Ionization mode	column	Compound_ID	MSMS_spectra	ID_level	ANOVA	Anorexia vs control		overweight vs control		obese vs control		athletes vs control	
								P-FDR	P	Estimate	P	Estimate	P	Estimate	P	Estimate
1476.1082@0.6532896	1476.1082	0.65	POS	hilic				9.75E-01	9.55E-01	0.01	7.58E-01	0.08	8.63E-01	-0.05	5.56E-01	0.17
1477.3448@3.2232394	1477.3448	3.22	NEG	hilic				7.65E-01	1.72E-01	-0.34	2.35E-01	-0.29	5.14E-01	-0.18	7.57E-01	-0.08
1477.7393@0.50641865	1477.7393	0.51	POS	hilic				5.04E-01	5.78E-01	0.16	7.29E-01	-0.09	3.06E-01	0.31	6.36E-02	0.47
1478.2292@9.778378	1478.2292	9.78	NEG	RP				7.07E-02	1.30E-02	-0.61	4.99E-01	0.19	9.72E-01	-0.01	1.26E-02	-0.63
1479.0883@0.6709214	1479.0883	0.67	POS	hilic				4.92E-01	6.92E-01	0.10	2.81E-01	0.27	4.27E-01	0.20	4.39E-02	0.61
1481.081@0.67093027	1481.0810	0.67	POS	hilic				5.19E-01	8.32E-01	-0.06	4.30E-01	0.20	2.49E-01	0.29	8.41E-02	0.51
1481.9594@1.318333	1481.9594	1.32	POS	hilic				6.64E-01	5.03E-01	0.20	7.09E-01	0.09	1.11E-01	0.50	9.17E-01	0.03
1482.081@0.6709567	1482.0810	0.67	POS	hilic				2.52E-01	1.80E-01	-0.34	3.77E-01	0.20	6.85E-01	0.10	1.18E-01	0.44
1482.1108@11.991659	1482.1108	11.99	POS	RP				6.66E-01	4.81E-01	0.20	2.16E-01	-0.29	9.38E-01	-0.02	4.00E-01	-0.24
1482.1108@12.108181	1482.1108	12.11	POS	RP				4.43E-01	2.18E-01	0.37	3.85E-02	0.56	9.87E-01	0.00	3.90E-01	0.24
1482.1125@0.8962549	1482.1125	0.90	POS	hilic				8.53E-01	1.91E-01	-0.31	5.84E-01	-0.14	3.69E-01	-0.24	3.21E-01	-0.26
1483.2885@0.3780084	1483.2885	0.38	NEG	hilic				8.64E-01	3.09E-01	0.35	1.86E-01	0.33	5.67E-01	0.15	5.37E-01	0.19
1483.981@1.304522	1483.9810	1.30	POS	hilic				9.38E-02	1.11E-02	0.79	8.08E-01	-0.06	1.32E-01	0.49	5.01E-02	0.56
1484.0955@0.6623086	1484.0955	0.66	POS	hilic				6.34E-01	7.28E-01	0.10	6.60E-01	0.10	8.91E-01	0.03	1.05E-01	0.49
1484.128@12.13052	1484.1280	12.13	POS	RP				6.53E-01	1.21E-01	0.41	8.94E-02	0.47	5.31E-01	0.16	3.68E-01	0.25
1484.1282@11.990395	1484.1282	11.99	POS	RP				3.97E-01	4.05E-01	-0.24	5.50E-02	-0.51	2.12E-02	-0.60	3.52E-01	-0.26
1484.3029@0.38010344	1484.3029	0.38	NEG	hilic				8.01E-01	3.26E-01	-0.24	4.42E-01	-0.20	2.33E-01	-0.27	8.57E-01	0.05
1485.0848@0.65138304	1485.0848	0.65	POS	hilic				8.19E-01	9.68E-01	-0.01	5.63E-01	0.16	6.46E-01	0.14	1.87E-01	0.40
1485.9948@1.1977853	1485.9948	1.20	POS	hilic				4.39E-01	5.17E-01	-0.18	9.86E-02	-0.54	5.04E-01	-0.20	3.19E-02	-0.59
1485.9979@1.3006439	1485.9979	1.30	POS	hilic				1.53E-02	6.25E-02	0.61	6.18E-02	-0.44	4.58E-01	-0.22	5.25E-02	0.51
1485.9987@10.109618	1485.9987	10.11	POS	RP				1.53E-03	4.72E-03	0.81	6.63E-02	-0.45	8.74E-01	-0.04	1.08E-02	0.70
1486.3011@0.38329914	1486.3011	0.38	NEG	hilic				8.23E-01	2.22E-01	0.42	3.09E-01	0.29	3.88E-01	0.21	6.05E-01	0.16
1486.9987@1.2961124	1486.9987	1.30	POS	hilic				4.03E-03	1.89E-02	0.68	2.19E-02	-0.58	3.85E-01	-0.24	1.49E-01	0.42
1487.0938@0.65147	1487.0938	0.65	POS	hilic				8.34E-01	9.60E-01	0.02	4.09E-01	0.22	5.00E-01	0.20	1.95E-01	0.39
1487.2947@9.790987	1487.2947	9.79	NEG	RP				5.52E-01	6.90E-01	-0.10	6.92E-01	-0.10	7.53E-01	0.09	6.16E-02	-0.50
1487.299@0.38205206	1487.2990	0.38	NEG	hilic				9.11E-01	6.87E-01	0.12	9.81E-01	0.01	4.30E-01	-0.21	7.35E-01	0.10
1488.7339@0.49901134	1488.7339	0.50	POS	hilic				3.33E-01	6.60E-01	-0.12	5.21E-01	-0.16	6.05E-01	-0.12	9.18E-02	0.50
1489.1108@0.65043294	1489.1108	0.65	POS	hilic				4.41E-01	6.19E-01	-0.16	9.68E-01	-0.01	1.90E-01	-0.35	2.52E-01	0.38
1490.1097@0.65096635	1490.1097	0.65	POS	hilic				7.61E-01	4.36E-01	0.26	1.70E-01	0.38	1.38E-01	0.43	2.60E-01	0.35
1490.1115@12.188011	1490.1115	12.19	POS	RP				5.47E-01	1.81E-01	0.48	9.02E-02	0.49	7.23E-02	0.48	8.08E-02	0.50
1491.9504@9.988126	1491.9504	9.99	NEG	RP				1.33E-03	3.42E-02	0.62	3.33E-02	-0.47	7.54E-02	-0.44	1.60E-01	0.36
1492.0074@0.9140129	1492.0074	0.91	POS	hilic				1.22E-01	7.92E-01	-0.07	1.09E-02	0.61	5.54E-01	-0.16	6.90E-01	-0.11
1492.2437@9.638171	1492.2437	9.64	NEG	RP				6.06E-01	4.71E-01	-0.23	3.52E-01	-0.25	3.33E-01	0.27	3.88E-01	-0.26
1492.2441@9.781668	1492.2441	9.78	NEG	RP				3.23E-01	6.76E-01	-0.14	7.63E-02	0.50	2.26E-01	0.31	4.98E-01	-0.18
1492.2441@9.90455	1492.2441	9.90	NEG	RP				2.85E-01	5.42E-01	-0.19	5.61E-01	0.17	7.65E-03	0.62	9.72E-01	-0.01
1493.248@9.779527	1493.2480	9.78	NEG	RP				2.22E-01	3.81E-01	-0.24	3.78E-02	0.62	6.75E-01	0.11	9.87E-01	0.00
1494.3029@0.36590913	1494.3029	0.37	NEG	hilic				5.10E-01	5.35E-01	0.18	1.57E-01	-0.46	3.53E-01	-0.27	4.02E-01	-0.23
1494.805@0.3657307	1494.8050	0.37	NEG	hilic				6.02E-01	6.71E-01	0.12	4.19E-01	-0.21	1.70E-01	-0.33	2.03E-01	-0.30
1497.0826@0.6501	1497.0826	0.65	POS	hilic				7.28E-01	7.01E-02	0.42	3.46E-01	0.24	1.65E-01	0.33	5.29E-01	0.18
1497.0966@0.64672583	1497.0966	0.65	POS	hilic				3.37E-01	8.03E-02	-0.42	1.21E-01	-0.36	1.48E-01	-0.35	5.93E-01	0.17
1498.336@0.38617387	1498.3360	0.39	NEG	hilic				1.69E-01	1.09E-02	0.76	1.18E-02	0.77	4.63E-02	0.52	1.44E-01	0.46
1498.3427@9.942604	1498.3427	9.94	NEG	RP				8.72E-01	2.72E-01	0.39	8.17E-01	0.06	5.84E-01	0.16	7.93E-01	0.08
1499.0905@12.221857	1499.0905	12.22	NEG	RP				4.44E-01	8.02E-01	-0.07	5.36E-01	-0.13	5.17E-02	-0.36	3.83E-01	0.21
1499.1226@0.6498315	1499.1226	0.65	POS	hilic				7.61E-01	4.53E-01	-0.19	5.57E-01	-0.15	6.66E-01	-0.11	4.26E-01	0.25
1501.1051@12.204904	1501.1051	12.20	NEG	RP				4.12E-02	8.12E-01	0.07	9.84E-01	-0.01	9.67E-02	-0.42	1.42E-02	0.74
1501.1184@0.6504634	1501.1184	0.65	POS	hilic				5.56E-01	9.12E-01	-0.03	6.01E-01	0.15	5.66E-01	0.15	8.74E-02	0.56
1501.9884@3.2019997	1501.9884	3.20	NEG	hilic				6.59E-01	9.83E-02	0.48	9.83E-01	-0.01	8.74E-01	0.04	3.95E-01	0.25
1502.305@0.38447997	1502.3050	0.38	NEG	hilic				6.93E-01	6.42E-02	-0.43	5.04E-01	-0.16	2.73E-01	-0.26	7.81E-01	-0.07
1502.6663@3.1993794	1502.6663	3.20	NEG	hilic				8.95E-01	9.76E-01	0.01	3.24E-01	-0.25	6.49E-01	-0.14	7.29E-01	0.10
1502.7358@0.472206	1502.7358	0.47	POS	hilic				6.23E-01	2.77E-02	-0.51	7.06E-01	-0.10	8.17E-01	-0.06	7.39E-01	-0.10
1503.331@3.205099	1503.3310	3.21	NEG	hilic				7.98E-01	5.85E-01	0.15	3.09E-01	0.31	7.79E-01	-0.08	3.95E-01	0.28
1503.3326@9.803351	1503.3326	9.80	POS	RP				1.20E-01	1.28E-01	-0.46	3.91E-02	0.54	4.16E-01	0.24	9.09E-01	-0.04
1503.9471@1.319329	1503.9471	1.32	POS	hilic				5.56E-01	7.63E-01	0.08	5.59E-01	-0.13	2.16E-01	0.40	5.96E-01	-0.13
1504.1158@11.926116	1504.1158	11.93	NEG	RP				7.59E-01	1.56E-01	0.42	8.04E-01	0.08	1.99E-01	0.36	6.38E-01	0.13
1504.231@9.781545	1504.2310	9.78	NEG	RP				3.64E-02	2.51E-02	0.75	3.09E-03	0.95	1.03E-02	0.78	3.27E-03	0.81
1504.2657@0.38444448	1504.2657	0.38	NEG	hilic				7.22E-01	5.40E-01	0.14	1.54E-01	0.31	3.00E-01	0.21	1.26E-01	0.39
1504.7408@0.48277992	1504.7408	0.48	POS	hilic				8.92E-01	2.34E-01	-0.35	4.50E-01	-0.21	8.65E-01	-0.06	7.45E-01	-0.09
1505.0834@12.273471	1505.0834	12.27	NEG	RP				3.21E-01	3.10E-02	-0.59	9.16E-01	0.03	7.51E-01	0.08	8.96E-01	0.03
1505.0945@0.6697086	1505.0945	0.67	POS	hilic				1.18E-01	3.37E-01	-0.25	6.41E-02	0.49	1.45E-01	0.40	6.21E-02	0.58
1505.1174@11.926265	1505.1174	11.93	NEG	RP				5.10E-01	1.82E-01	0.38	4.46E-01	-0.22	4.17E-01	0.21	6.67E-01	-0.11
1505.1263@0.6648642	1505.1263	0.66	POS	hilic				5.27E-01	7.73E-01	0.07	1.35E-01	0.38	7.11E-02	0.50	2.10E-01	0.36
1505.3438@9.791657	1505.3438	9.79	POS	RP				4.77E-01	6.38E-01	-0.14	8.25E-02	0.54	5.90E-01	0.15	5.21E-01	0.20
1505.9639@1.307915	1505.9639	1.31	POS	hilic				3.81E-02	3.29E-01	0.28	2.11E-02	-0.56	8.72E-02	-0.45	4.80E-01	0.20
1506.2227@9.657934	1506.2227	9.66	NEG	RP				4.06E-01	8.38E-01	0.06	1.20E-01	0.50	3.74E-02	0.52	9.09E-01	0.03
1506.226@9.782815	1506.2260	9.78	NEG	RP				2.67E-01	6.29E-02	0.53	3.61E-02	0.63	1.41E-02	0.70	3.70E-01	0.28
1506.7585@0.4875139	1506.7585	0.49	POS	hilic				7.76E-01	9.25E-01	-0.03	8.51E-01	0.05	3.60E-01	-0.28	4.55E-01	0.23
1507.0968@12.380704	1507.0968	12.38	NEG	RP				1.66E-01	1.07E-01	-0.45	9.95E-01	0.00	4.10E-01	0.22	1.06E-01	0.50
1507.0979@12.380551	1507.0979	12.38	NEG	RP				2.56E-01	1.93E-01	-0.39	5.27E-01	0.16	9.40E-02	0.47	9.56E-01	-0.02
1507.102@0.6621034	1507.1020	0.66	POS	hilic				9.52E-01	5.39E-01	0.14	8.20E-01	0.06	4.34E-01	0.20	4.75E-01	0.20
1507.2299@9.78253	1507.2299	9.78	NEG	RP				3.84E-01	7.96E-02	0.53	6.85E-02	0.56	1.44E-01	0.50	7.80E-01	0.09
1507.9781@1.1580786	1507.9781	1.16	POS	hilic				1.91E-02	4.81E-02	0.60	7.63E-02	-0.42	7.84E-01	0.08	4.03E-02	0.57
1507.9788@10.12092	1507.9788	10.12	POS	RP				5.94E-01	2.67E-01	0.34	2.22E-01	0.39	2.08E-01	0.36	4.68E-02	0.59
1507.9799@1.2981198	1507.9799	1.30	POS	hilic				6.94E-03	1.40E-01	0.40	1.64E-02	-0.67	8.83E-02	-0.48	2.66E-01	0.36

Supplementary table 2: Metabolomics analysis results

								ANOVA	Anorexia vs control		overweight vs control		obese vs control		athletes vs control	
Compound	Mass	Retention Time	Ionization mode	column	Compound_ID	MSMS_spectra	ID_level	P-FDR	P	Estimate	P	Estimate	P	Estimate	P	Estimate
1508.0988@12.380326	1508.0988	12.38	NEG	RP				6.67E-02	1.77E-01	-0.39	2.27E-01	0.31	4.43E-02	0.51	9.53E-02	0.50
1508.1259@12.057271	1508.1259	12.06	POS	RP				7.55E-01	5.08E-01	0.17	1.35E-01	0.36	7.44E-01	0.08	9.76E-01	0.01
1508.2788@3.050509	1508.2788	3.05	POS	hilic				9.05E-01	7.63E-01	0.10	9.01E-01	-0.03	5.88E-01	0.15	4.83E-01	-0.20
1508.9832@1.2928087	1508.9832	1.29	POS	hilic				6.11E-03	5.60E-01	0.18	1.18E-02	-0.69	8.53E-03	-0.73	3.29E-01	0.30
1508.9832@10.118939	1508.9832	10.12	POS	RP				6.90E-01	2.68E-01	0.35	4.94E-01	0.22	1.46E-01	0.35	7.74E-02	0.52
1509.0992@12.059569	1509.0992	12.06	NEG	RP				7.34E-01	8.27E-02	0.47	5.67E-01	0.16	4.80E-01	0.19	2.37E-01	0.32
1509.1061@0.65053684	1509.1061	0.65	POS	hilic				8.17E-01	7.72E-01	0.09	8.31E-01	-0.06	8.23E-01	-0.07	2.37E-01	0.34
1509.982@10.032531	1509.9820	10.03	POS	RP				6.25E-01	9.21E-01	0.03	1.68E-01	-0.40	1.54E-01	-0.42	8.09E-01	-0.08
1509.9965@1.289222	1509.9965	1.29	POS	hilic				4.99E-03	4.42E-01	0.21	9.74E-03	-0.76	2.66E-03	-0.77	6.16E-01	0.17
1511.0931@11.949492	1511.0931	11.95	POS	RP				7.20E-01	3.96E-01	0.23	2.74E-01	-0.29	9.77E-01	-0.01	7.15E-01	0.12
1511.097@0.6503279	1511.0970	0.65	POS	hilic				4.69E-01	6.11E-01	0.13	2.55E-01	-0.28	5.42E-02	-0.44	8.65E-01	-0.05
1512.325@0.38618174	1512.3250	0.39	NEG	hilic				6.09E-01	2.47E-01	0.38	9.22E-02	0.45	8.33E-01	0.06	1.56E-01	0.41
1513.112@12.122043	1513.1120	12.12	POS	RP				5.20E-01	1.26E-01	0.51	1.53E-01	0.41	4.97E-02	0.55	1.11E-01	0.48
1513.3271@0.38598242	1513.3271	0.39	NEG	hilic				8.14E-01	3.89E-01	0.27	1.43E-01	0.41	7.00E-01	0.10	4.66E-01	0.23
1513.4557@10.107617	1513.4557	10.11	POS	RP				1.72E-02	2.04E-02	0.69	2.96E-01	-0.27	5.40E-01	0.17	9.42E-03	0.76
1514.0236@1.2598171	1514.0236	1.26	POS	hilic				1.28E-02	5.06E-01	0.18	3.48E-02	-0.53	1.37E-03	-0.76	8.36E-01	0.06
1514.1132@12.117056	1514.1132	12.12	POS	RP				5.92E-01	9.75E-01	-0.01	3.65E-01	0.21	8.31E-02	0.42	9.82E-01	-0.01
1514.7393@0.49253196	1514.7393	0.49	POS	hilic				3.21E-01	7.90E-02	-0.49	3.66E-01	0.25	4.38E-01	-0.21	6.67E-01	0.12
1515.127@0.64767	1515.1270	0.65	POS	hilic				2.75E-02	1.26E-01	-0.45	5.33E-01	-0.15	1.19E-02	-0.70	1.55E-01	0.43
1515.127@12.215902	1515.1270	12.22	POS	RP				8.95E-01	9.89E-01	0.00	2.75E-01	0.28	5.01E-01	0.15	9.05E-01	0.03
1515.9493@9.970372	1515.9493	9.97	NEG	RP				2.16E-04	3.49E-02	0.60	1.74E-02	-0.66	3.33E-03	-0.77	2.63E-01	0.30
1516.1335@0.6502826	1516.1335	0.65	POS	hilic				1.67E-01	8.71E-01	-0.05	6.59E-01	-0.11	1.68E-02	-0.62	3.06E-01	0.34
1516.304@0.3826964	1516.3040	0.38	NEG	hilic				2.37E-01	1.46E-02	0.70	9.20E-01	0.03	5.80E-01	0.15	6.62E-02	0.53
1516.326@3.1982992	1516.3260	3.20	NEG	hilic				7.59E-01	4.92E-01	0.19	1.34E-01	0.41	1.99E-01	0.40	2.91E-01	0.30
1516.7443@0.49273866	1516.7443	0.49	POS	hilic				6.90E-01	1.42E-01	-0.42	8.73E-01	-0.05	2.93E-01	-0.27	7.48E-01	0.09
1516.8293@3.1935177	1516.8293	3.19	NEG	hilic				1.74E-01	7.00E-01	0.12	3.32E-01	-0.26	9.17E-01	-0.03	1.25E-02	0.64
1517.1392@12.695237	1517.1392	12.70	POS	RP				8.42E-01	2.47E-01	0.37	4.04E-01	0.23	9.90E-01	0.00	5.98E-01	0.17
1517.7025@4.4044037	1517.7025	4.40	POS	hilic				7.75E-01	5.47E-01	-0.13	3.40E-01	0.24	7.62E-01	-0.07	6.60E-01	0.12
1518.326@3.223459	1518.3260	3.22	NEG	hilic				7.65E-01	2.34E-01	0.31	1.78E-01	0.39	4.87E-01	0.19	9.08E-01	0.03
1518.3278@3.2010477	1518.3278	3.20	NEG	hilic				8.94E-01	6.97E-01	0.12	7.46E-01	0.09	4.72E-01	-0.22	7.39E-01	0.09
1518.7603@0.49342337	1518.7603	0.49	POS	hilic				7.31E-01	1.04E-01	-0.49	8.00E-01	-0.08	7.72E-01	-0.07	9.28E-01	-0.03
1519.7415@4.5669146	1519.7415	4.57	NEG	hilic				5.42E-01	2.50E-01	0.38	9.45E-02	0.50	2.04E-01	0.38	9.41E-01	0.02
1519.7472@4.561787	1519.7472	4.56	NEG	hilic				7.04E-01	9.99E-01	0.00	2.20E-01	0.31	2.64E-01	0.25	1.54E-01	0.32
1519.7603@0.49478948	1519.7603	0.49	POS	hilic				8.34E-01	2.83E-01	-0.27	3.70E-01	-0.26	3.07E-01	-0.26	9.77E-01	0.01
1520.1602@12.695659	1520.1602	12.70	POS	RP				8.69E-01	2.47E-01	0.37	4.45E-01	0.22	9.42E-01	0.02	6.15E-01	0.16
1520.279@9.864479	1520.2790	9.86	NEG	RP				5.95E-01	4.38E-01	0.25	4.78E-01	0.21	2.85E-01	0.30	3.93E-01	-0.27
1521.08@12.123453	1521.0800	12.12	NEG	RP				4.60E-01	7.26E-02	0.42	5.24E-02	0.48	5.48E-01	0.13	1.41E-01	0.38
1521.972@10.105564	1521.9720	10.11	NEG	RP				1.64E-01	2.66E-01	0.39	9.67E-01	-0.01	5.48E-02	0.51	1.27E-02	0.75
1521.9727@1.424305	1521.9727	1.42	NEG	hilic				2.24E-01	5.22E-01	0.22	1.24E-01	0.37	6.21E-03	0.80	7.78E-01	0.09
1521.9965@10.230229	1521.9965	10.23	NEG	RP				1.44E-03	1.26E-01	0.45	2.24E-02	-0.60	9.65E-03	-0.69	3.29E-01	0.27
1522.2836@0.37912354	1522.2836	0.38	NEG	hilic				5.25E-01	2.68E-01	0.29	7.08E-02	0.54	8.21E-01	0.06	2.82E-01	0.29
1522.977@1.4222883	1522.9770	1.42	NEG	hilic				6.56E-01	5.23E-01	0.18	4.95E-01	0.16	6.65E-02	0.49	6.51E-01	0.12
1523.092@12.235814	1523.0920	12.24	NEG	RP				5.44E-02	4.25E-01	0.22	6.65E-01	-0.10	1.65E-01	-0.29	1.80E-02	0.64
1523.122@0.64233327	1523.1220	0.64	POS	hilic				1.10E-02	7.28E-04	-0.73	2.34E-02	-0.50	1.62E-01	-0.35	4.16E-01	0.22
1523.1342@12.416346	1523.1342	12.42	POS	RP				6.60E-01	2.79E-01	0.23	3.53E-01	0.26	8.87E-02	0.39	1.00E-01	0.42
1523.9478@10.106966	1523.9478	10.11	POS	RP				6.98E-01	6.53E-01	0.14	8.62E-01	0.05	2.54E-01	-0.35	5.42E-01	0.18
1523.9556@1.3149999	1523.9556	1.31	POS	hilic				1.99E-01	2.09E-01	-0.29	9.00E-01	-0.03	1.01E-01	0.46	5.25E-01	-0.16
1524.2925@0.37750444	1524.2925	0.38	NEG	hilic				7.97E-01	4.31E-01	0.25	5.18E-01	0.22	8.99E-01	-0.04	1.75E-01	0.37
1524.2935@9.794472	1524.2935	9.79	NEG	RP				2.35E-01	1.46E-03	0.72	1.71E-01	0.42	4.22E-01	0.22	1.56E-02	0.59
1525.1064@12.002801	1525.1064	12.00	POS	RP				2.42E-01	2.38E-01	0.30	6.80E-01	-0.12	4.56E-01	-0.22	6.24E-02	0.56
1525.1427@12.52072	1525.1427	12.52	POS	RP				9.18E-01	4.65E-01	-0.19	9.74E-01	-0.01	5.82E-01	0.15	9.42E-01	-0.02
1525.299@0.3768545	1525.2990	0.38	NEG	hilic				9.13E-01	4.96E-01	0.22	8.50E-01	-0.06	8.15E-01	-0.07	5.29E-01	0.18
1526.1414@0.63909876	1526.1414	0.64	POS	hilic				1.48E-01	5.77E-01	-0.15	3.54E-01	0.25	9.16E-01	-0.03	2.04E-02	0.73
1527.1248@12.747495	1527.1248	12.75	NEG	RP				7.09E-01	1.86E-01	0.33	1.72E-01	0.39	5.64E-01	0.13	2.00E-01	0.35
1527.1628@12.679442	1527.1628	12.68	POS	RP				8.47E-01	1.60E-01	-0.38	1.00E+00	0.00	8.03E-01	-0.07	7.05E-01	-0.11
1527.9517@1.3117802	1527.9517	1.31	POS	hilic				1.35E-01	1.94E-03	-0.96	3.75E-01	-0.23	1.65E-01	-0.42	2.68E-01	-0.34
1528.0425@10.498478	1528.0425	10.50	NEG	RP				8.49E-03	3.57E-02	0.63	1.32E-01	-0.30	2.47E-02	-0.44	9.46E-01	0.02
1528.1154@11.94568	1528.1154	11.95	NEG	RP				7.66E-01	7.36E-01	-0.10	2.92E-01	-0.29	1.57E-01	-0.39	2.64E-01	-0.32
1528.144@0.6417727	1528.1440	0.64	POS	hilic				3.91E-01	3.96E-01	-0.19	1.89E-01	-0.28	1.07E-01	-0.36	4.92E-01	0.20
1528.3221@0.38579985	1528.3221	0.39	NEG	hilic				1.71E-01	1.67E-01	0.36	3.36E-01	0.24	5.18E-02	0.50	4.44E-03	0.79
1529.9637@1.1537215	1529.9637	1.15	POS	hilic				1.32E-01	4.89E-01	0.21	1.11E-02	-0.63	4.62E-01	-0.22	5.20E-01	0.18
1529.9644@1.3196862	1529.9644	1.32	POS	hilic				3.77E-02	1.13E-02	-0.86	5.12E-01	-0.16	1.59E-02	-0.71	8.23E-03	-0.80
1529.9792@3.1951134	1529.9792	3.20	NEG	hilic				9.27E-01	6.22E-01	0.15	7.41E-01	-0.09	5.20E-01	-0.18	9.93E-01	0.00
1529.9832@1.4123532	1529.9832	1.41	NEG	hilic				2.39E-02	4.56E-03	0.82	4.91E-01	-0.16	1.62E-01	0.45	1.52E-02	0.69
1530.1284@12.123837	1530.1284	12.12	NEG	RP				6.07E-01	4.11E-01	0.17	9.12E-02	0.41	3.57E-01	0.19	1.07E-01	0.37
1530.1299@11.965753	1530.1299	11.97	NEG	RP				2.68E-02	9.96E-02	0.45	1.45E-01	-0.38	7.47E-02	-0.50	2.45E-01	0.36
1530.1447@0.6444445	1530.1447	0.64	POS	hilic				7.72E-02	9.19E-03	0.62	9.74E-01	0.00	7.59E-01	0.05	4.86E-01	0.14
1530.3167@0.38598242	1530.3167	0.39	NEG	hilic				8.51E-01	7.92E-01	0.08	2.42E-01	0.33	6.56E-01	0.12	3.05E-01	0.32
1530.649@3.195576	1530.6490	3.20	NEG	hilic				6.35E-01	1.86E-01	0.45	1.65E-01	0.36	6.62E-02	0.51	2.78E-01	0.28
1530.6542@3.1878154	1530.6542	3.19	NEG	hilic				4.04E-01	5.87E-01	0.15	7.30E-01	-0.09	2.13E-01	0.37	3.73E-02	0.53
1530.7618@0.4876093	1530.7618	0.49	POS	hilic				9.87E-01	9.98E-01	0.00	6.76E-01	0.11	8.52E-01	-0.05	7.27E-01	0.11
1531.0911@0.6731283	1531.0911	0.67	NEG	hilic				1.55E-01	7.51E-01	-0.06	3.55E-01	0.22	3.66E-01	0.19	1.45E-02	0.64
1531.1025@12.38046	1531.1025	12.38	NEG	RP				2.70E-01	3.63E-02	-0.45	8.77E-01	-0.04	2.42E-01	0.28	5.58E-01	-0.15

Supplementary table 2: Metabolomics analysis results

Compound	Mass	Retention Time	Ionization mode	column	Compound_ID	MSMS_spectra	ID_level	ANOVA P-FDR	Anorexia vs control P	Anorexia vs control Estimate	overweight vs control P	overweight vs control Estimate	obese vs control P	obese vs control Estimate	athletes vs control P	athletes vs control Estimate
1531.2004@11.036973	1531.2004	11.04	NEG	RP				5.57E-01	7.28E-01	0.08	1.90E-01	0.32	7.84E-02	0.43	5.85E-01	0.11
1531.314@3.1920896	1531.3140	3.19	NEG	hilic				8.91E-01	8.39E-01	-0.06	8.54E-01	0.06	7.69E-01	0.09	3.67E-01	0.30
1531.3188@3.1978	1531.3188	3.20	NEG	hilic				9.75E-01	9.43E-01	-0.02	6.34E-01	-0.13	4.91E-01	-0.19	9.43E-01	-0.02
1531.9764@1.2775642	1531.9764	1.28	POS	hilic				1.84E-02	4.32E-01	-0.20	1.42E-02	-0.71	1.40E-04	-1.01	7.25E-01	-0.12
1531.9775@9.992348	1531.9775	9.99	POS	RP				1.76E-03	3.02E-02	0.60	1.22E-01	-0.45	6.69E-02	-0.46	4.90E-02	0.63
1531.9788@1.1559719	1531.9788	1.16	POS	hilic				2.55E-02	1.76E-01	0.37	1.04E-01	-0.44	1.34E-01	-0.46	1.22E-01	0.43
1532.0013@1.4125254	1532.0013	1.41	NEG	hilic				2.57E-02	4.04E-03	0.83	4.71E-01	-0.17	1.99E-01	0.42	1.60E-02	0.67
1532.002@10.110602	1532.0020	10.11	NEG	RP				2.30E-03	8.61E-03	0.76	6.17E-02	-0.46	7.59E-01	-0.09	1.32E-02	0.67
1532.0035@10.031034	1532.0035	10.03	NEG	RP				9.75E-01	7.29E-01	0.10	6.85E-01	-0.13	7.91E-01	-0.08	7.11E-01	-0.11
1532.3027@9.941914	1532.3027	9.94	NEG	RP				7.38E-01	5.50E-01	-0.17	7.47E-01	0.10	4.14E-01	0.26	5.33E-01	-0.22
1532.3066@0.38609904	1532.3066	0.39	NEG	hilic				6.81E-01	3.04E-01	0.33	2.38E-01	0.36	7.61E-01	0.08	9.10E-02	0.48
1532.758@0.4874876	1532.7580	0.49	POS	hilic				8.18E-01	7.67E-01	0.08	2.61E-01	-0.29	5.51E-01	-0.15	9.15E-01	0.03
1533.004@1.4117451	1533.0040	1.41	NEG	hilic				2.90E-02	3.13E-02	0.70	1.68E-01	-0.31	6.48E-01	0.14	1.54E-02	0.61
1533.0778@11.9338875	1533.0778	11.93	POS	RP				6.16E-01	1.92E-01	0.37	7.58E-01	-0.08	1.44E-01	0.39	4.67E-01	0.22
1533.0999@0.67226577	1533.0999	0.67	NEG	hilic				5.86E-01	2.70E-01	0.33	2.80E-01	0.32	4.47E-01	0.21	3.35E-02	0.60
1533.114@12.491267	1533.1140	12.49	NEG	RP				3.60E-01	7.50E-01	-0.08	9.98E-01	0.00	1.69E-01	0.38	6.31E-02	0.54
1533.9893@10.13497	1533.9893	10.13	POS	RP				8.69E-01	6.39E-01	0.16	6.11E-01	-0.16	5.66E-01	-0.17	6.94E-01	0.11
1533.9941@9.98736	1533.9941	9.99	POS	RP				2.85E-03	1.72E-01	0.37	3.73E-02	-0.62	8.79E-03	-0.67	1.97E-01	0.40
1533.9948@1.2752095	1533.9948	1.28	POS	hilic				5.28E-03	6.02E-01	-0.14	3.09E-03	-0.71	3.00E-04	-0.83	8.08E-01	0.07
1534.1031@0.66822743	1534.1031	0.67	NEG	hilic				9.43E-01	6.14E-01	0.13	6.49E-01	0.11	5.93E-01	0.12	3.04E-01	0.25
1534.1202@12.492989	1534.1202	12.49	NEG	RP				1.15E-01	9.95E-01	0.00	1.57E-01	0.38	6.83E-02	0.49	4.30E-03	0.78
1534.1277@0.6442971	1534.1277	0.64	POS	hilic				7.80E-01	6.83E-01	0.10	2.10E-01	0.34	1.90E-01	0.35	4.79E-01	0.19
1534.3184@9.796605	1534.3184	9.80	NEG	RP				7.65E-01	3.31E-01	0.28	8.78E-02	0.42	2.18E-01	0.34	2.89E-01	0.35
1534.3226@0.38610256	1534.3226	0.39	NEG	hilic				3.99E-01	2.21E-01	0.39	2.60E-02	0.67	3.21E-01	0.27	8.90E-02	0.53
1534.9991@1.2693596	1534.9991	1.27	POS	hilic				9.12E-03	6.45E-01	-0.13	6.23E-03	-0.74	1.24E-04	-0.99	8.23E-01	-0.07
1535.0981@11.928242	1535.0981	11.93	POS	RP				6.81E-01	6.40E-01	0.13	5.46E-01	-0.17	2.30E-01	0.36	8.13E-01	-0.07
1535.101@0.64039445	1535.1010	0.64	POS	hilic				2.14E-01	1.85E-01	-0.39	1.84E-01	-0.41	5.60E-01	-0.15	1.84E-01	0.38
1535.1011@12.067356	1535.1011	12.07	POS	RP				7.12E-02	4.19E-03	0.77	8.06E-01	-0.06	8.71E-01	0.04	2.60E-01	0.28
1535.1147@12.174723	1535.1147	12.17	NEG	RP				5.93E-01	5.93E-02	0.55	1.99E-01	0.33	2.97E-01	0.26	2.62E-01	0.31
1535.3221@9.941971	1535.3221	9.94	NEG	RP				4.52E-01	6.39E-01	0.12	5.24E-02	-0.52	4.04E-01	-0.24	2.89E-01	-0.34
1535.326@0.38600898	1535.3260	0.39	NEG	hilic				3.86E-01	2.02E-01	0.39	4.35E-02	0.62	4.23E-01	0.22	5.42E-02	0.59
1536.008@1.1868548	1536.0080	1.19	POS	hilic				6.64E-02	3.78E-01	0.23	3.67E-02	-0.50	7.68E-02	-0.44	5.92E-01	0.15
1536.7642@0.48915562	1536.7642	0.49	POS	hilic				9.18E-01	5.14E-01	-0.18	7.02E-01	-0.10	6.51E-01	0.12	5.69E-01	-0.17
1536.9023@1.3190436	1536.9023	1.32	POS	hilic				4.02E-01	4.75E-01	0.23	4.45E-02	0.53	3.50E-02	0.66	3.14E-01	0.28
1536.9178@10.125642	1536.9178	10.13	POS	RP				8.16E-01	5.54E-01	0.19	4.39E-01	0.21	3.87E-01	0.22	1.08E-01	0.43
1537.0115@1.2672848	1537.0115	1.27	POS	hilic				3.25E-03	3.03E-01	0.30	6.95E-03	-0.74	4.97E-03	-0.70	5.82E-01	0.16
1537.1053@11.976762	1537.1053	11.98	POS	RP				1.55E-02	1.02E-02	0.61	3.19E-02	-0.58	3.24E-01	-0.27	7.55E-01	0.09
1537.107@12.184175	1537.1070	12.18	POS	RP				2.73E-01	8.07E-01	0.08	3.25E-02	0.65	7.36E-02	0.45	5.82E-02	0.57
1537.1105@12.070502	1537.1105	12.07	POS	RP				8.00E-01	2.12E-01	0.42	3.59E-01	0.27	6.09E-01	0.15	9.07E-01	0.04
1538.0198@1.253153	1538.0198	1.25	POS	hilic				6.37E-03	1.92E-01	0.40	8.98E-02	-0.44	3.68E-03	-0.74	3.09E-01	0.31
1538.1132@12.037519	1538.1132	12.04	POS	RP				9.17E-01	2.12E-01	0.31	9.79E-01	-0.01	9.74E-01	0.01	6.98E-01	0.10
1538.2983@0.37723917	1538.2983	0.38	NEG	hilic				4.54E-01	2.77E-02	0.69	3.25E-01	0.28	6.64E-01	0.12	2.47E-01	0.34
1538.6122@12.359933	1538.6122	12.36	POS	RP				5.67E-01	3.07E-01	0.27	5.60E-02	0.51	1.74E-01	0.31	1.40E-01	0.42
1538.9124@10.112579	1538.9124	10.11	POS	RP				4.51E-02	3.11E-02	0.70	3.35E-02	0.67	2.58E-02	0.62	4.50E-04	1.04
1538.9156@1.3182865	1538.9156	1.32	POS	hilic				5.72E-01	3.85E-01	0.30	5.66E-02	0.48	7.50E-02	0.56	1.55E-01	0.37
1539.1263@12.183874	1539.1263	12.18	POS	RP				7.92E-01	3.32E-01	0.33	2.10E-01	0.37	3.79E-01	0.23	9.07E-01	0.03
1539.1274@0.64044416	1539.1274	0.64	POS	hilic				4.11E-01	8.31E-01	-0.06	8.64E-01	-0.05	2.79E-01	-0.29	1.43E-01	0.47
1539.606@12.365935	1539.6060	12.37	POS	RP				8.89E-01	6.85E-01	0.12	3.09E-01	0.29	8.30E-01	0.05	3.44E-01	0.29
1539.9135@1.317057	1539.9135	1.32	POS	hilic				1.04E-01	4.33E-01	0.23	8.37E-02	0.46	1.25E-03	0.93	2.33E-01	0.31
1539.9156@10.1037655	1539.9156	10.10	POS	RP				3.66E-01	2.04E-01	0.36	2.24E-01	0.37	6.23E-02	0.48	1.95E-02	0.73
1539.9478@9.967116	1539.9478	9.97	NEG	RP				2.17E-01	1.69E-01	0.42	3.86E-01	-0.26	2.99E-01	-0.30	2.03E-01	0.33
1540.0417@1.2375724	1540.0417	1.24	POS	hilic				2.05E-03	8.77E-02	0.52	1.53E-02	-0.64	5.04E-03	-0.74	8.57E-01	0.05
1540.1313@0.64040846	1540.1313	0.64	POS	hilic				1.78E-01	4.05E-01	-0.24	9.53E-01	-0.02	1.31E-01	-0.42	9.30E-02	0.50
1541.1375@12.33612	1541.1375	12.34	POS	RP				1.80E-02	3.16E-01	0.33	9.94E-02	-0.41	9.46E-02	-0.44	4.66E-02	0.54
1541.1385@12.350536	1541.1385	12.35	POS	RP				2.26E-01	9.57E-01	0.02	5.03E-01	-0.17	4.95E-02	-0.65	4.42E-01	0.20
1541.7275@4.5287066	1541.7275	4.53	NEG	hilic				9.23E-01	6.58E-01	-0.14	9.95E-01	0.00	3.00E-01	-0.30	7.89E-01	-0.08
1541.919@10.106153	1541.9190	10.11	POS	RP				2.91E-02	1.14E-02	0.72	5.67E-01	0.18	7.62E-01	0.09	2.15E-04	0.95
1542.143@12.343152	1542.1430	12.34	POS	RP				4.64E-02	5.61E-01	0.17	2.74E-01	-0.28	1.23E-02	-0.71	2.28E-01	0.31
1542.1462@0.6404954	1542.1462	0.64	POS	hilic				4.12E-01	6.02E-01	0.16	5.53E-01	-0.15	1.79E-01	-0.39	2.98E-01	0.33
1542.1469@12.670095	1542.1469	12.67	POS	RP				6.37E-01	4.08E-01	0.26	4.52E-01	-0.18	2.91E-01	-0.26	5.23E-01	-0.17
1542.2393@9.797795	1542.2393	9.80	NEG	RP				7.61E-01	5.65E-01	0.15	3.27E-01	0.29	1.81E-01	0.42	9.29E-01	0.02
1542.5887@12.226191	1542.5887	12.23	POS	RP				2.48E-01	8.96E-01	0.04	6.04E-02	-0.53	9.74E-02	-0.44	5.87E-01	0.16
1543.1519@12.663015	1543.1519	12.66	POS	RP				1.52E-01	2.24E-01	0.37	1.71E-01	-0.29	8.06E-01	0.06	1.07E-01	-0.39
1543.1545@0.6405824	1543.1545	0.64	POS	hilic				5.89E-01	5.35E-01	0.19	6.47E-01	-0.11	2.89E-01	-0.32	3.93E-01	0.27
1543.1552@12.897637	1543.1552	12.90	POS	RP				7.71E-01	2.15E-01	-0.38	6.57E-01	-0.13	4.04E-01	-0.21	7.85E-01	0.08
1544.1556@0.64061517	1544.1556	0.64	POS	hilic				5.89E-01	1.54E-01	0.41	6.71E-01	-0.11	6.36E-01	-0.14	4.49E-01	0.25
1544.1584@12.6787815	1544.1584	12.68	POS	RP				7.49E-01	1.76E-01	0.33	3.08E-01	0.26	2.08E-01	0.29	1.15E-01	0.35
1544.9229@10.113118	1544.9229	10.11	POS	RP				6.65E-01	4.67E-01	0.22	1.14E-01	0.40	2.71E-01	0.25	1.00E-01	0.47
1545.075@12.050873	1545.0750	12.05	NEG	RP				4.53E-01	3.26E-01	0.22	6.42E-01	0.09	3.71E-01	-0.15	1.73E-01	0.35
1545.0844@12.027435	1545.0844	12.03	NEG	RP				2.58E-01	3.96E-01	0.20	6.50E-01	-0.12	2.82E-01	-0.24	1.01E-01	0.45
1545.1335@12.4983635	1545.1335	12.50	NEG	RP				9.80E-01	9.96E-01	0.00	7.45E-01	-0.08	8.60E-01	0.05	6.25E-01	0.12
1545.1687@12.808915	1545.1687	12.81	POS	RP				7.82E-01	5.62E-01	0.16	3.36E-01	0.26	1.08E-01	0.43	4.13E-01	0.23
1545.976@1.3969822	1545.9760	1.40	NEG	hilic				8.19E-01	5.54E-01	-0.18	3.29E-01	-0.26	3.61E-01	-0.27	7.43E-01	0.10

Supplementary table 2: Metabolomics analysis results

								ANOVA	Anorexia vs control		overweight vs control		obese vs control		athletes vs control	
Compound	Mass	Retention Time	Ionization mode	column	Compound_ID	MSMS_spectra	ID_level	P-FDR	P	Estimate	P	Estimate	P	Estimate	P	Estimate
1546.1729@12.897426	1546.1729	12.90	POS	RP				8.60E-01	4.33E-01	0.25	7.98E-01	0.06	1.95E-01	0.31	3.67E-01	0.25
1546.7635@0.5151625	1546.7635	0.52	POS	hilic				9.25E-01	8.47E-01	-0.05	4.29E-01	0.20	7.47E-01	0.09	7.50E-01	-0.08
1547.3018@0.37657794	1547.3018	0.38	NEG	hilic				9.79E-01	5.80E-01	0.16	4.98E-01	0.19	8.80E-01	0.05	5.91E-01	0.16
1548.1451@0.6224917	1548.1451	0.62	POS	hilic				2.39E-02	1.78E-02	-0.54	5.17E-01	0.17	4.41E-02	-0.43	1.61E-01	0.36
1548.315@0.37736547	1548.3150	0.38	NEG	hilic				8.29E-01	3.00E-01	-0.27	1.74E-01	-0.31	3.31E-01	-0.25	3.39E-01	-0.24
1548.7719@0.5199278	1548.7719	0.52	POS	hilic				2.10E-01	2.23E-01	-0.33	3.41E-01	0.25	1.27E-01	-0.40	3.72E-01	0.24
1549.1038@12.057715	1549.1038	12.06	POS	RP				8.39E-01	3.47E-01	-0.20	7.70E-01	0.07	4.89E-01	0.16	8.18E-01	0.06
1549.1084@0.68846357	1549.1084	0.69	NEG	hilic				2.81E-01	3.81E-01	0.21	6.89E-01	0.11	4.40E-01	-0.18	5.34E-02	0.55
1549.2535@3.0455434	1549.2535	3.05	POS	hilic				8.75E-01	9.01E-01	-0.03	8.54E-01	0.05	4.37E-01	-0.26	3.98E-01	-0.25
1549.2585@3.0555139	1549.2585	3.06	POS	hilic				8.41E-01	5.16E-01	0.21	3.47E-01	0.25	6.72E-01	0.13	6.09E-01	-0.14
1550.3173@0.3795306	1550.3173	0.38	NEG	hilic				7.55E-01	2.64E-01	0.32	9.95E-01	0.00	7.22E-01	0.11	1.82E-01	0.38
1551.1031@12.233206	1551.1031	12.23	NEG	RP				7.99E-01	8.12E-01	0.07	7.92E-01	-0.08	5.97E-01	-0.12	2.44E-01	0.32
1551.1494@12.688594	1551.1494	12.69	POS	RP				4.73E-01	1.48E-01	-0.35	7.84E-01	-0.07	6.87E-01	0.10	1.00E-01	-0.36
1551.2548@3.053434	1551.2548	3.05	POS	hilic				8.09E-01	7.07E-01	-0.10	8.07E-01	0.07	4.14E-01	-0.28	2.19E-01	-0.30
1551.3151@0.37861225	1551.3151	0.38	NEG	hilic				8.23E-01	2.45E-01	0.34	8.45E-01	-0.06	8.48E-01	0.05	4.78E-01	0.20
1552.1523@0.6305961	1552.1523	0.63	POS	hilic				1.96E-01	2.96E-02	-0.52	8.08E-01	0.06	8.27E-01	-0.06	1.90E-01	0.40
1553.1722@12.701263	1553.1722	12.70	POS	RP				4.52E-01	5.96E-02	-0.50	7.52E-02	-0.48	3.57E-01	-0.26	9.91E-01	0.00
1553.9624@1.1490794	1553.9624	1.15	POS	hilic				1.40E-01	8.60E-01	0.04	3.87E-02	-0.51	1.87E-01	-0.33	3.87E-01	0.28
1553.9857@1.3952998	1553.9857	1.40	NEG	hilic				3.06E-03	2.12E-01	0.35	1.05E-02	-0.66	3.65E-03	-0.66	3.50E-01	0.29
1554.0754@12.250088	1554.0754	12.25	POS	RP				5.69E-01	2.36E-01	0.33	6.92E-01	0.12	1.95E-01	-0.33	9.19E-01	0.03
1554.1285@11.967	1554.1285	11.97	NEG	RP				7.64E-01	1.43E-01	-0.34	8.34E-01	-0.06	6.74E-01	0.11	6.90E-01	-0.10
1554.1298@12.028767	1554.1298	12.03	NEG	RP				4.46E-01	3.51E-01	-0.26	1.23E-01	0.44	9.06E-01	0.03	9.81E-01	0.01
1554.1595@0.6341396	1554.1595	0.63	POS	hilic				8.41E-01	7.89E-01	0.07	7.03E-01	-0.10	6.19E-01	0.15	3.05E-01	0.29
1554.1772@12.700136	1554.1772	12.70	POS	RP				1.28E-01	7.22E-03	-0.67	8.61E-01	0.05	4.42E-01	-0.19	4.43E-01	0.22
1554.2509@9.875384	1554.2509	9.88	NEG	RP				4.71E-01	8.01E-01	0.08	2.26E-01	0.28	6.29E-01	-0.15	2.06E-01	-0.40
1555.1324@12.667505	1555.1324	12.67	NEG	RP				4.63E-01	2.46E-01	0.37	1.50E-01	0.39	6.78E-01	-0.11	1.55E-01	0.44
1555.9683@9.989579	1555.9683	9.99	POS	RP				2.64E-01	2.09E-01	0.40	2.39E-01	-0.32	1.55E-01	-0.37	7.38E-01	0.10
1555.9764@1.260635	1555.9764	1.26	POS	hilic				2.76E-02	3.61E-01	-0.28	1.62E-02	-0.68	2.09E-04	-1.03	5.38E-01	-0.19
1555.9792@1.2136433	1555.9792	1.21	POS	hilic				1.50E-01	4.24E-03	-0.80	1.33E-01	-0.46	5.75E-02	-0.57	8.59E-01	-0.05
1555.9998@9.997089	1555.9998	10.00	NEG	RP				7.03E-03	2.65E-02	0.64	1.15E-01	-0.47	2.29E-01	-0.33	9.88E-02	0.49
1556.0002@1.394862	1556.0002	1.39	NEG	hilic				5.73E-03	2.22E-01	0.31	3.32E-02	-0.61	2.36E-03	-0.75	4.29E-01	0.26
1556.1761@0.62421626	1556.1761	0.62	POS	hilic				4.94E-01	1.77E-02	-0.56	8.94E-01	-0.03	9.59E-01	-0.02	6.75E-01	-0.11
1556.2563@9.894267	1556.2563	9.89	NEG	RP				5.89E-02	2.92E-01	-0.29	1.11E-01	0.44	8.19E-03	0.67	6.95E-01	-0.12
1556.2574@9.789182	1556.2574	9.79	NEG	RP				7.58E-01	4.53E-01	-0.22	9.37E-01	0.03	2.16E-01	0.33	8.85E-01	0.04
1557.0042@1.3942224	1557.0042	1.39	NEG	hilic				3.19E-03	5.54E-01	0.16	1.20E-02	-0.70	7.72E-04	-0.82	4.21E-01	0.25
1557.075@11.936028	1557.0750	11.94	POS	RP				9.52E-01	5.02E-01	0.20	7.72E-01	-0.08	8.42E-01	0.05	6.96E-01	0.12
1557.0931@0.6608414	1557.0931	0.66	NEG	hilic				2.72E-02	8.31E-01	-0.06	7.74E-02	-0.46	6.28E-03	-0.64	1.87E-01	0.42
1557.098@11.884597	1557.0980	11.88	NEG	RP				8.30E-01	3.49E-01	0.25	2.77E-01	0.27	5.59E-01	0.14	2.16E-01	0.36
1557.2596@9.790481	1557.2596	9.79	NEG	RP				7.98E-01	3.67E-01	0.25	2.96E-01	0.30	1.32E-01	0.40	8.80E-01	0.05
1557.2603@9.901902	1557.2603	9.90	NEG	RP				1.74E-01	8.65E-01	0.05	8.66E-01	-0.05	2.95E-01	0.26	2.27E-02	-0.63
1557.3069@3.1938171	1557.3069	3.19	NEG	hilic				9.57E-01	9.53E-01	0.02	8.76E-01	-0.04	6.40E-01	0.15	4.85E-01	0.20
1557.3077@3.1929467	1557.3077	3.19	NEG	hilic				8.24E-01	9.10E-01	0.04	9.25E-01	-0.03	3.28E-01	0.30	2.93E-01	0.30
1557.973@3.195511	1557.9730	3.20	NEG	hilic				9.70E-01	9.93E-01	0.00	7.42E-01	0.08	7.74E-01	-0.09	5.61E-01	0.15
1557.9742@3.1842442	1557.9742	3.18	NEG	hilic				5.44E-01	3.85E-01	0.27	1.67E-01	-0.31	6.94E-01	0.11	5.17E-01	-0.17
1557.9904@9.959446	1557.9904	9.96	POS	RP				1.07E-02	9.01E-01	0.03	2.31E-02	-0.70	1.43E-03	-0.84	6.08E-01	0.16
1557.9952@1.2397718	1557.9952	1.24	POS	hilic				1.73E-01	8.17E-01	-0.07	3.55E-02	-0.66	1.90E-02	-0.62	8.63E-01	-0.06
1558.0164@10.11879	1558.0164	10.12	NEG	RP				4.29E-03	4.86E-02	0.58	7.24E-02	-0.56	9.87E-03	-0.67	7.54E-01	0.09
1558.0988@11.896276	1558.0988	11.90	NEG	RP				7.01E-01	3.12E-01	0.23	3.20E-01	-0.28	6.86E-01	0.12	7.80E-01	0.08
1558.6385@3.1966245	1558.6385	3.20	NEG	hilic				7.58E-01	3.71E-01	0.26	9.13E-01	-0.03	2.30E-01	0.39	3.82E-01	0.24
1558.643@3.201291	1558.6430	3.20	NEG	hilic				9.06E-01	9.32E-01	-0.03	4.92E-01	-0.18	5.72E-01	0.16	7.83E-01	0.07
1558.7769@0.48867077	1558.7769	0.49	POS	hilic				6.37E-01	6.23E-02	-0.44	5.55E-01	-0.17	9.71E-01	0.01	7.21E-01	0.10
1558.809@3.1997292	1558.8090	3.20	NEG	hilic				9.22E-01	6.67E-01	0.13	3.27E-01	0.30	5.15E-01	0.19	3.72E-01	0.24
1558.9976@1.1966124	1558.9976	1.20	POS	hilic				4.28E-01	4.38E-01	-0.21	3.24E-02	-0.64	1.03E-01	-0.47	4.85E-01	-0.22
1559.0208@10.118957	1559.0208	10.12	NEG	RP				6.04E-03	1.11E-01	0.47	5.68E-02	-0.60	3.89E-03	-0.75	9.82E-01	-0.01
1559.0887@11.911841	1559.0887	11.91	POS	RP				4.55E-01	3.71E-02	0.59	2.06E-01	0.32	5.10E-01	0.18	1.04E-01	0.42
1559.0953@0.6305	1559.0953	0.63	POS	hilic				9.88E-01	8.29E-01	-0.06	6.57E-01	0.13	8.71E-01	0.04	9.43E-01	0.02
1559.1132@12.10469	1559.1132	12.10	NEG	RP				3.05E-01	5.02E-01	0.21	6.30E-01	0.12	3.66E-01	-0.25	4.87E-02	0.53
1560.0063@1.2467028	1560.0063	1.25	POS	hilic				4.58E-02	2.82E-01	0.32	4.79E-02	-0.53	2.98E-02	-0.54	7.33E-01	0.10
1560.116@12.0999775	1560.1160	12.10	NEG	RP				7.76E-01	6.24E-01	0.13	4.56E-01	0.16	1.17E-01	0.40	6.88E-01	0.10
1560.7701@0.5054623	1560.7701	0.51	POS	hilic				5.71E-01	2.26E-01	-0.33	6.49E-01	0.13	4.34E-01	0.22	3.32E-01	0.27
1560.894@1.3229028	1560.8940	1.32	POS	hilic				9.34E-01	5.50E-01	0.18	5.90E-01	0.15	7.46E-01	-0.09	6.16E-01	0.14
1560.9105@10.009291	1560.9105	10.01	POS	RP				3.54E-01	1.56E-01	0.46	9.38E-01	0.02	8.38E-01	-0.06	7.13E-02	0.52
1561.0142@1.2501311	1561.0142	1.25	POS	hilic				1.78E-02	8.28E-01	0.06	1.78E-01	-0.39	9.54E-05	-1.01	8.28E-01	-0.06
1561.102@0.6301539	1561.1020	0.63	POS	hilic				2.01E-01	5.21E-03	0.68	1.38E-01	0.37	2.21E-02	0.66	3.46E-01	0.23
1561.1049@12.148194	1561.1049	12.15	POS	RP				1.28E-02	5.87E-02	-0.59	4.49E-02	0.59	5.00E-02	0.50	6.08E-01	0.14
1561.1053@11.989106	1561.1053	11.99	POS	RP				8.88E-03	8.76E-01	-0.04	1.34E-03	-0.87	3.51E-02	-0.60	4.93E-01	0.20
1561.1282@0.660919	1561.1282	0.66	NEG	hilic				5.29E-02	4.97E-01	-0.21	9.94E-02	-0.40	6.06E-03	-0.75	3.26E-01	0.30
1561.1289@12.201691	1561.1289	12.20	NEG	RP				5.64E-02	7.00E-01	-0.13	1.91E-01	-0.34	2.42E-02	-0.64	1.44E-01	0.44
1561.3062@3.2055	1561.3062	3.21	NEG	hilic				3.36E-01	7.53E-01	-0.07	2.65E-01	0.23	4.61E-01	0.17	5.28E-02	0.55
1562.0284@1.2379818	1562.0284	1.24	POS	hilic				2.29E-02	3.43E-01	0.32	4.44E-02	-0.56	4.02E-03	-0.72	8.66E-01	0.05
1562.1093@11.99802	1562.1093	12.00	POS	RP				5.41E-03	3.32E-01	0.26	5.33E-02	-0.52	3.44E-02	-0.64	9.85E-02	0.49
1562.1309@0.6609318	1562.1309	0.66	NEG	hilic				1.45E-01	8.49E-01	0.06	4.47E-01	-0.19	1.49E-02	-0.64	3.53E-01	0.31
1562.3492@0.38698244	1562.3492	0.39	NEG	hilic				8.36E-01	1.83E-01	0.39	3.46E-01	0.27	7.31E-01	0.09	4.28E-01	0.26

Supplementary table 2: Metabolomics analysis results

Compound	Mass	Retention Time	Ionization mode	column	Compound_ID	MSMS_spectra	ID_level	ANOVA P-FDR	Anorexia vs control P	Anorexia vs control Estimate	overweight vs control P	overweight vs control Estimate	obese vs control P	obese vs control Estimate	athletes vs control P	athletes vs control Estimate
1562.3499@9.943041	1562.3499	9.94	NEG	RP				3.42E-01	6.13E-01	-0.14	5.05E-01	-0.18	2.66E-02	-0.73	7.15E-01	-0.12
1562.7712@0.51144457	1562.7712	0.51	POS	hilic				5.12E-01	4.98E-01	-0.18	2.79E-01	-0.26	8.26E-01	-0.06	2.37E-01	0.34
1562.908@1.3063914	1562.9080	1.31	POS	hilic				9.33E-01	9.07E-01	0.04	6.25E-01	0.12	5.70E-01	-0.15	5.95E-01	0.16
1562.9156@10.000582	1562.9156	10.00	POS	RP				1.39E-03	1.60E-03	0.92	4.32E-01	-0.22	4.75E-01	-0.18	4.08E-03	0.76
1563.029@1.2401718	1563.0290	1.24	POS	hilic				3.27E-03	6.21E-02	0.53	2.69E-02	-0.65	5.13E-03	-0.71	9.45E-01	-0.02
1563.1285@12.144584	1563.1285	12.14	POS	RP				9.43E-01	5.10E-01	0.20	7.01E-01	-0.11	8.48E-01	0.05	7.40E-01	0.11
1563.1447@12.664511	1563.1447	12.66	NEG	RP				8.65E-01	2.70E-01	0.37	6.21E-01	0.13	9.53E-01	-0.02	6.22E-01	0.16
1563.6156@12.367163	1563.6156	12.37	POS	RP				4.62E-01	6.69E-01	0.14	3.48E-02	-0.48	6.98E-01	-0.10	7.31E-01	-0.09
1563.9178@1.3086567	1563.9178	1.31	POS	hilic				5.24E-01	3.80E-01	-0.26	2.53E-01	0.26	3.06E-01	-0.28	3.78E-01	-0.26
1563.919@9.999438	1563.9190	10.00	POS	RP				5.45E-04	2.87E-03	0.91	1.43E-01	-0.36	1.27E-01	-0.36	4.65E-02	0.53
1563.9482@9.9589815	1563.9482	9.96	NEG	RP				8.72E-01	3.34E-01	-0.28	4.08E-01	-0.24	5.04E-01	-0.23	8.97E-01	0.03
1564.039@1.2210529	1564.0390	1.22	POS	hilic				1.65E-02	1.12E-01	0.52	6.05E-02	-0.52	1.58E-02	-0.62	9.84E-01	-0.01
1564.0426@10.232657	1564.0426	10.23	POS	RP				9.91E-03	8.46E-02	0.52	5.18E-02	-0.60	1.75E-02	-0.63	9.17E-01	-0.03
1564.1274@0.63047004	1564.1274	0.63	POS	hilic				2.12E-01	6.08E-02	-0.56	2.75E-01	0.33	4.58E-01	0.19	8.99E-01	0.04
1564.1302@12.13761	1564.1302	12.14	POS	RP				1.14E-02	3.12E-02	-0.62	9.08E-02	0.50	1.04E-02	0.53	9.24E-01	0.03
1564.3892@4.5417285	1564.3892	4.54	NEG	hilic				3.94E-01	3.56E-01	0.24	3.12E-02	0.55	7.83E-01	0.06	1.49E-01	0.37
1565.0472@1.2260535	1565.0472	1.23	POS	hilic				1.50E-02	1.24E-01	0.49	8.24E-02	-0.49	9.46E-03	-0.66	8.29E-01	0.06
1565.0483@10.232479	1565.0483	10.23	POS	RP				8.18E-03	8.24E-02	0.52	5.60E-02	-0.58	1.66E-02	-0.63	9.94E-01	0.00
1565.1436@12.257071	1565.1436	12.26	POS	RP				7.88E-01	3.89E-01	0.30	8.13E-01	0.07	8.05E-01	0.06	1.91E-01	0.39
1565.1584@12.665453	1565.1584	12.67	NEG	RP				8.34E-01	2.30E-01	0.40	5.06E-01	0.18	9.92E-01	0.00	6.25E-01	0.16
1565.5865@12.24812	1565.5865	12.25	POS	RP				3.69E-01	1.17E-01	0.44	4.50E-01	-0.15	8.90E-01	-0.03	3.03E-01	0.29
1565.6302@12.36721	1565.6302	12.37	POS	RP				2.12E-01	9.82E-03	0.79	2.81E-02	0.55	2.02E-01	0.33	1.00E-01	0.48
1566.055@1.2198535	1566.0550	1.22	POS	hilic				1.20E-02	2.43E-02	0.67	7.11E-02	-0.54	8.34E-02	-0.43	9.62E-01	0.01
1566.1469@0.63018256	1566.1469	0.63	POS	hilic				6.19E-01	3.17E-01	-0.32	2.65E-01	0.33	9.08E-01	0.03	9.85E-01	-0.01
1566.1617@12.664913	1566.1617	12.66	NEG	RP				8.09E-01	2.05E-01	0.43	5.21E-01	0.18	9.76E-01	0.01	5.81E-01	0.17
1566.3156@0.38091958	1566.3156	0.38	NEG	hilic				7.37E-01	9.39E-01	0.02	2.08E-01	0.33	7.13E-01	-0.09	3.88E-01	0.25
1566.5908@12.17621	1566.5908	12.18	POS	RP				4.68E-01	7.14E-01	0.09	8.95E-01	-0.03	6.25E-02	0.51	7.79E-01	-0.08
1566.6112@12.363494	1566.6112	12.36	POS	RP				4.20E-01	1.70E-01	0.34	4.34E-01	0.22	3.43E-01	-0.22	2.07E-01	0.31
1566.878@9.9939995	1566.8780	9.99	POS	RP				3.78E-01	3.27E-01	0.19	4.69E-02	0.32	4.02E-01	0.13	2.92E-02	0.45
1567.1501@12.855992	1567.1501	12.86	POS	RP				1.48E-01	9.23E-01	0.02	2.83E-02	0.61	1.35E-01	0.36	4.88E-01	-0.19
1567.1525@0.63253284	1567.1525	0.63	POS	hilic				5.77E-01	1.99E-01	0.38	7.99E-02	0.48	1.24E-01	0.47	7.62E-02	0.49
1567.1534@12.397268	1567.1534	12.40	POS	RP				2.85E-01	8.66E-01	0.05	2.36E-02	-0.62	3.32E-01	-0.23	9.02E-01	0.04
1567.1562@12.419308	1567.1562	12.42	POS	RP				7.99E-01	1.71E-01	0.36	6.93E-01	0.12	2.43E-01	0.30	9.55E-01	-0.02
1567.5891@12.365928	1567.5891	12.37	POS	RP				6.46E-01	2.64E-01	-0.30	2.36E-01	-0.28	1.37E-01	-0.35	8.91E-02	-0.41
1567.5931@12.303644	1567.5931	12.30	POS	RP				7.21E-02	3.10E-01	0.30	7.40E-03	-0.65	1.32E-01	-0.36	6.26E-01	-0.14
1568.0721@1.2157192	1568.0721	1.22	POS	hilic				5.15E-02	8.80E-02	0.56	7.58E-02	-0.49	1.58E-01	-0.37	9.58E-01	-0.02
1568.1595@0.63018256	1568.1595	0.63	POS	hilic				7.31E-01	8.74E-01	0.05	1.72E-01	0.39	2.20E-01	0.35	9.36E-01	0.02
1568.2408@9.837304	1568.2408	9.84	NEG	RP				1.06E-01	3.16E-01	0.27	1.56E-02	0.81	7.98E-03	0.71	1.33E-02	0.60
1568.8647@9.970638	1568.8647	9.97	POS	RP				2.87E-04	9.11E-03	0.86	9.82E-02	-0.39	6.86E-02	-0.42	8.31E-03	0.67
1569.0721@1.2116047	1569.0721	1.21	POS	hilic				1.09E-01	8.35E-02	0.57	1.15E-01	-0.43	3.84E-01	-0.23	8.83E-01	0.04
1569.08@12.0510645	1569.0800	12.05	POS	RP				5.70E-01	8.21E-02	-0.36	4.98E-01	-0.15	6.75E-01	0.10	7.06E-01	0.10
1569.1686@12.798466	1569.1686	12.80	POS	RP				7.77E-01	5.64E-01	0.17	5.03E-01	0.20	2.87E-01	0.32	6.53E-01	-0.13
1569.1729@0.63018256	1569.1729	0.63	POS	hilic				6.36E-01	6.76E-01	0.12	1.53E-01	0.40	1.12E-01	0.45	9.63E-01	0.01
1569.174@12.79613	1569.1740	12.80	POS	RP				7.10E-01	1.40E-01	-0.41	8.91E-01	-0.04	5.72E-01	0.14	8.89E-01	-0.04
1569.356@9.798197	1569.3560	9.80	POS	RP				3.60E-01	4.38E-01	0.26	1.54E-02	0.69	8.30E-02	0.48	9.98E-02	0.50
1569.8665@9.970564	1569.8665	9.97	POS	RP				1.25E-04	5.16E-03	0.89	1.01E-01	-0.36	3.20E-02	-0.49	2.83E-02	0.56
1570.049@12.2407675	1570.0490	12.24	POS	RP				8.85E-01	7.09E-01	0.11	7.56E-01	0.08	4.53E-01	-0.22	6.90E-01	-0.13
1570.0846@1.2099644	1570.0846	1.21	POS	hilic				4.79E-02	1.70E-02	0.74	1.57E-01	-0.40	8.15E-01	0.06	8.10E-01	-0.07
1570.0876@10.516923	1570.0876	10.52	POS	RP				9.51E-02	1.34E-01	0.48	6.21E-02	-0.53	4.07E-01	-0.22	9.68E-01	0.01
1570.1656@12.661795	1570.1656	12.66	POS	RP				9.39E-01	4.44E-01	0.22	8.60E-01	-0.04	7.16E-01	0.09	9.31E-01	-0.02
1570.1724@12.793339	1570.1724	12.79	POS	RP				1.78E-01	1.12E-01	-0.47	4.84E-01	0.20	1.98E-01	0.31	2.59E-01	-0.30
1570.2382@9.793531	1570.2382	9.79	NEG	RP				3.83E-01	6.05E-01	0.15	1.39E-02	0.70	2.39E-01	0.38	2.84E-01	0.25
1570.2404@9.999287	1570.2404	10.00	NEG	RP				3.69E-01	8.03E-01	-0.07	1.24E-02	-0.62	2.86E-01	-0.31	1.82E-01	-0.35
1571.0876@1.2075741	1571.0876	1.21	POS	hilic				1.78E-01	7.27E-02	0.57	2.17E-01	-0.32	6.78E-01	-0.12	7.70E-01	0.08
1571.1486@12.488058	1571.1486	12.49	NEG	RP				4.05E-01	2.23E-01	-0.29	4.82E-01	-0.19	6.74E-02	-0.52	6.25E-01	0.14
1571.1781@0.6301217	1571.1781	0.63	POS	hilic				4.02E-01	4.21E-01	0.21	3.18E-01	0.29	2.09E-02	0.69	7.84E-01	0.08
1571.1833@12.917446	1571.1833	12.92	POS	RP				7.31E-01	2.07E-01	-0.33	1.86E-01	-0.37	9.85E-01	0.01	3.21E-01	-0.29
1571.242@9.789283	1571.2420	9.79	NEG	RP				8.59E-01	2.54E-01	0.31	2.35E-01	0.33	5.09E-01	0.21	7.80E-01	0.07
1571.2441@9.802853	1571.2441	9.80	NEG	RP				7.36E-02	1.36E-01	0.47	3.08E-01	0.30	3.36E-03	0.72	3.35E-01	-0.26
1572.1888@12.921883	1572.1888	12.92	POS	RP				5.55E-01	1.90E-01	-0.38	5.14E-02	-0.49	9.19E-01	-0.03	4.83E-01	-0.21
1572.3217@0.38586554	1572.3217	0.39	NEG	hilic				9.61E-01	8.16E-01	0.06	8.15E-01	0.06	4.59E-01	-0.16	9.19E-01	-0.03
1572.9115@10.227404	1572.9115	10.23	POS	RP				9.25E-03	1.71E-02	0.66	1.35E-01	-0.35	3.49E-01	-0.23	1.85E-01	0.38
1573.0837@12.687788	1573.0837	12.69	POS	RP				7.26E-01	1.36E-01	0.47	3.41E-01	0.24	3.69E-01	0.24	7.99E-01	0.07
1573.1288@0.6805532	1573.1288	0.68	NEG	hilic				6.31E-01	1.93E-01	0.33	8.91E-01	-0.03	8.53E-01	-0.04	3.62E-01	-0.19
1573.1462@0.6198498	1573.1462	0.62	POS	hilic				4.95E-03	6.84E-04	-0.81	1.60E-01	0.39	4.88E-01	0.19	1.56E-01	0.38
1573.1606@12.634622	1573.1606	12.63	NEG	RP				7.40E-01	1.68E-01	-0.33	7.50E-01	0.09	7.86E-01	-0.06	7.47E-01	0.09
1573.8951@1.3241737	1573.8951	1.32	POS	hilic				7.36E-01	2.14E-01	-0.26	5.07E-01	0.17	7.69E-01	0.08	9.18E-01	-0.02
1574.1511@0.61958754	1574.1511	0.62	POS	hilic				3.33E-03	2.93E-04	-0.98	1.61E-01	0.38	1.00E+00	0.00	5.25E-01	0.18
1574.7712@0.50715554	1574.7712	0.51	POS	hilic				5.31E-01	9.46E-01	0.02	8.39E-01	0.06	5.41E-01	0.17	5.04E-02	0.55
1574.9098@10.228697	1574.9098	10.23	POS	RP				1.74E-03	1.99E-02	0.67	7.03E-02	-0.50	1.02E-01	-0.44	1.11E-01	0.47
1574.9504@10.25638	1574.9504	10.26	POS	RP				4.15E-02	5.06E-02	0.57	4.87E-01	-0.15	2.94E-02	-0.41	6.75E-01	0.11
1575.0997@12.162096	1575.0997	12.16	NEG	RP				7.07E-02	8.98E-02	0.50	1.24E-01	-0.41	1.61E-01	-0.33	7.31E-01	0.09
1575.1241@0.68368953	1575.1241	0.68	NEG	hilic				2.60E-01	1.52E-01	0.32	4.97E-02	0.42	6.59E-02	0.41	9.67E-01	-0.01

Supplementary table 2: Metabolomics analysis results

Compound	Mass	Retention Time	Ionization mode	column	Compound_ID	MSMS_spectra	ID_level	ANOVA P-FDR	Anorexia vs control P	Anorexia vs control Estimate	overweight vs control P	overweight vs control Estimate	obese vs control P	obese vs control Estimate	athletes vs control P	athletes vs control Estimate
1575.1584@0.6207661	1575.1584	0.62	POS	hilic				7.74E-01	3.21E-01	-0.20	4.44E-01	0.20	7.90E-01	0.06	6.41E-01	0.11
1575.8909@1.3252032	1575.8909	1.33	POS	hilic				1.57E-01	8.21E-01	0.06	3.57E-02	0.56	1.69E-02	0.59	5.92E-01	0.12
1575.9144@10.228057	1575.9144	10.23	POS	RP				1.90E-03	1.27E-01	0.45	1.69E-02	-0.63	2.13E-02	-0.62	3.25E-01	0.27
1576.1248@12.370056	1576.1248	12.37	POS	RP				5.02E-01	6.15E-01	0.14	9.53E-02	-0.31	1.46E-01	-0.28	8.17E-01	-0.06
1576.1649@0.619824	1576.1649	0.62	POS	hilic				1.86E-01	3.95E-03	-0.68	5.33E-01	0.18	6.98E-01	0.11	8.24E-01	-0.07
1576.336@0.385809	1576.3360	0.39	NEG	hilic				7.88E-01	9.24E-01	0.03	4.22E-01	0.25	6.01E-01	-0.14	3.56E-01	0.28
1576.7711@0.5129375	1576.7711	0.51	POS	hilic				9.46E-01	5.45E-01	0.16	9.06E-01	-0.03	5.90E-01	-0.11	9.02E-01	0.03
1577.1646@0.6200984	1577.1646	0.62	POS	hilic				1.67E-01	1.41E-02	-0.45	3.05E-01	0.27	4.86E-01	0.19	2.34E-01	0.29
1577.3394@0.38564277	1577.3394	0.39	NEG	hilic				8.87E-01	2.46E-01	0.32	5.18E-01	0.19	9.34E-01	0.02	4.13E-01	0.25
1577.7035@4.600785	1577.7035	4.60	NEG	hilic				4.27E-01	5.16E-02	0.57	7.80E-01	0.06	1.18E-01	0.37	6.79E-01	0.11
1577.9637@1.158969	1577.9637	1.16	POS	hilic				2.14E-01	2.17E-01	-0.35	4.03E-02	-0.55	3.99E-03	-0.74	6.55E-01	-0.15
1578.132@12.3299265	1578.1320	12.33	NEG	RP				9.82E-01	6.44E-01	-0.13	8.18E-01	-0.06	7.26E-01	-0.09	8.18E-01	0.06
1578.1716@0.62033343	1578.1716	0.62	POS	hilic				3.10E-01	4.86E-02	-0.37	3.84E-01	0.22	7.16E-01	0.10	2.07E-01	0.30
1579.0634@11.901727	1579.0634	11.90	POS	RP				3.38E-01	4.27E-02	0.53	1.30E-01	0.38	9.86E-01	0.00	1.24E-01	0.44
1579.9738@9.9576845	1579.9738	9.96	POS	RP				1.85E-02	8.90E-01	-0.04	3.45E-02	-0.66	7.73E-04	-0.86	6.80E-01	0.13
1579.9742@1.1632125	1579.9742	1.16	POS	hilic				2.57E-01	4.10E-01	-0.24	2.63E-02	-0.62	9.85E-03	-0.66	6.32E-01	-0.17
1579.9814@1.2470698	1579.9814	1.25	POS	hilic				4.40E-02	8.49E-01	0.05	4.47E-02	-0.56	1.15E-04	-0.91	3.80E-01	-0.27
1580.0009@9.987348	1580.0009	9.99	NEG	RP				6.09E-03	4.84E-01	0.18	2.85E-02	-0.68	3.20E-03	-0.77	3.91E-01	0.27
1580.002@1.3807575	1580.0020	1.38	NEG	hilic				1.23E-02	9.72E-01	0.01	2.24E-02	-0.65	6.83E-04	-0.90	8.39E-01	0.07
1580.1462@12.047971	1580.1462	12.05	NEG	RP				5.84E-02	4.74E-03	-0.65	2.99E-01	0.27	6.43E-01	-0.13	7.86E-02	-0.40
1580.1779@0.6281001	1580.1779	0.63	POS	hilic				7.12E-01	1.41E-01	-0.33	7.27E-01	-0.09	5.21E-01	0.19	8.64E-01	0.05
1580.315@0.37894443	1580.3150	0.38	NEG	hilic				6.09E-01	2.04E-01	0.37	2.56E-01	0.32	3.39E-01	0.25	6.48E-01	-0.15
1580.9792@9.956894	1580.9792	9.96	POS	RP				6.94E-02	6.78E-01	-0.12	5.28E-02	-0.58	2.12E-03	-0.74	6.26E-01	0.15
1581.1108@0.6553789	1581.1108	0.66	NEG	hilic				9.39E-01	6.14E-01	-0.14	7.51E-01	0.10	6.26E-01	-0.13	7.54E-01	0.09
1581.1825@0.6290768	1581.1825	0.63	POS	hilic				5.00E-01	4.54E-02	-0.38	8.21E-01	-0.06	5.78E-01	-0.13	4.82E-01	0.16
1581.9893@1.2293074	1581.9893	1.23	POS	hilic				7.85E-01	5.69E-01	0.17	4.76E-01	-0.17	2.69E-01	-0.23	9.28E-01	0.03
1582.0164@10.136063	1582.0164	10.14	NEG	RP				5.43E-01	4.45E-01	0.26	2.24E-01	-0.39	6.96E-01	0.10	8.97E-01	0.04
1583.0013@1.237937	1583.0013	1.24	POS	hilic				2.24E-02	4.20E-01	0.23	1.97E-01	-0.33	4.61E-04	-0.87	7.63E-01	0.09
1583.0944@12.120144	1583.0944	12.12	POS	RP				2.70E-01	1.24E-01	-0.48	6.22E-02	-0.49	8.19E-02	-0.48	7.60E-01	0.10
1583.1082@11.966696	1583.1082	11.97	NEG	RP				4.70E-01	2.90E-01	0.26	1.65E-01	-0.42	7.56E-01	-0.11	6.45E-01	0.12
1583.1104@0.65695757	1583.1104	0.66	NEG	hilic				5.09E-01	8.28E-01	-0.06	9.57E-01	-0.02	2.83E-01	-0.28	1.98E-01	0.42
1583.1143@12.066614	1583.1143	12.07	NEG	RP				4.06E-01	9.33E-01	-0.03	2.73E-01	0.30	4.73E-02	0.64	6.17E-01	0.14
1584.0071@1.2311001	1584.0071	1.23	POS	hilic				2.29E-01	2.99E-02	0.61	7.49E-02	0.42	1.04E-01	0.36	8.57E-01	0.04
1584.0319@10.218258	1584.0319	10.22	NEG	RP				4.63E-03	1.55E-02	0.72	9.58E-02	-0.45	1.10E-01	-0.42	2.67E-01	0.33
1584.1132@12.3681345	1584.1132	12.37	POS	RP				6.60E-01	2.61E-01	0.29	8.70E-01	0.04	3.40E-01	0.26	4.55E-01	-0.20
1584.898@9.992797	1584.8980	9.99	POS	RP				2.40E-01	3.89E-02	0.63	7.59E-01	0.09	6.75E-01	-0.10	1.38E-01	0.48
1584.9135@10.108308	1584.9135	10.11	NEG	RP				4.17E-01	3.91E-01	0.30	3.62E-01	0.28	4.37E-01	0.22	9.78E-03	0.72
1585.0103@1.2362319	1585.0103	1.24	POS	hilic				3.11E-02	6.76E-01	0.14	5.58E-02	-0.53	1.04E-03	-0.85	9.40E-01	-0.02
1585.1064@12.118756	1585.1064	12.12	POS	RP				5.75E-01	1.01E-01	-0.47	4.90E-01	-0.21	4.12E-02	-0.51	7.44E-01	-0.10
1585.1104@0.6247376	1585.1104	0.62	POS	hilic				3.87E-01	6.75E-02	0.40	5.33E-02	0.54	1.59E-01	0.36	8.04E-01	0.06
1585.1263@0.65672994	1585.1263	0.66	NEG	hilic				5.08E-01	6.01E-01	-0.15	6.42E-01	-0.13	2.67E-01	-0.29	2.19E-01	0.37
1585.1313@12.167183	1585.1313	12.17	NEG	RP				2.58E-02	1.78E-01	-0.42	1.95E-02	0.68	2.11E-02	0.53	4.05E-01	0.24
1585.919@10.111144	1585.9190	10.11	NEG	RP				4.33E-01	3.90E-01	0.22	4.93E-01	0.17	4.59E-01	0.18	2.44E-02	0.63
1585.9565@3.1854584	1585.9565	3.19	NEG	hilic				8.17E-01	3.33E-01	0.27	3.83E-01	0.25	9.16E-01	0.03	2.13E-01	0.35
1586.0212@1.2208687	1586.0212	1.22	POS	hilic				3.92E-02	1.27E-01	0.50	1.03E-01	-0.46	2.88E-02	-0.55	9.71E-01	0.01
1586.0215@10.23461	1586.0215	10.23	POS	RP				1.27E-02	5.14E-02	0.60	9.79E-02	-0.50	2.39E-02	-0.58	8.99E-01	-0.04
1586.132@0.65636957	1586.1320	0.66	NEG	hilic				4.24E-01	5.40E-01	-0.18	5.08E-01	-0.19	2.21E-01	-0.31	1.93E-01	0.39
1586.2966@0.37753698	1586.2966	0.38	NEG	hilic				4.20E-01	1.32E-01	0.44	3.67E-02	0.67	3.40E-01	0.24	1.84E-01	0.39
1586.6254@3.1895092	1586.6254	3.19	NEG	hilic				4.41E-01	1.34E-01	0.54	4.02E-01	0.22	1.34E-01	0.42	5.86E-03	0.62
1586.7596@0.49507087	1586.7596	0.50	POS	hilic				6.94E-01	7.07E-01	-0.12	7.03E-01	0.10	1.56E-01	-0.38	6.83E-01	0.13
1586.9146@9.992213	1586.9146	9.99	POS	RP				1.70E-01	1.49E-02	0.68	9.67E-01	0.01	2.94E-01	-0.25	5.03E-01	0.23
1587.0273@10.234272	1587.0273	10.23	POS	RP				8.11E-03	5.19E-02	0.60	7.08E-02	-0.54	1.72E-02	-0.60	8.81E-01	-0.04
1587.123@12.090871	1587.1230	12.09	POS	RP				7.99E-01	4.10E-01	-0.27	6.79E-01	0.13	9.03E-01	-0.03	4.84E-01	0.19
1587.1259@12.116816	1587.1259	12.12	POS	RP				9.39E-01	8.31E-01	0.07	7.37E-01	-0.10	3.20E-01	-0.24	7.67E-01	-0.08
1587.143@12.317447	1587.1430	12.32	NEG	RP				5.44E-02	9.97E-02	0.48	9.36E-01	0.02	1.25E-01	-0.42	4.18E-02	0.59
1587.2972@3.2017114	1587.2972	3.20	NEG	hilic				7.98E-01	8.00E-01	0.08	3.64E-01	-0.24	3.90E-01	0.19	6.92E-01	-0.13
1587.731@4.569452	1587.7310	4.57	NEG	hilic				3.34E-01	1.59E-01	0.45	3.23E-02	0.61	6.76E-02	0.48	1.56E-02	0.65
1587.919@9.991671	1587.9190	9.99	POS	RP				7.36E-02	3.89E-02	0.60	5.59E-01	-0.16	4.51E-01	-0.17	1.02E-01	0.52
1587.9218@10.111941	1587.9218	10.11	NEG	RP				4.98E-01	2.37E-01	0.39	2.86E-01	0.25	5.36E-02	0.52	7.38E-02	0.55
1588.0413@1.2182758	1588.0413	1.22	POS	hilic				4.50E-02	4.58E-02	0.60	1.06E-01	-0.49	1.66E-01	-0.35	8.90E-01	-0.04
1588.1263@12.074482	1588.1263	12.07	POS	RP				7.04E-01	1.74E-01	-0.34	8.82E-01	-0.04	8.84E-01	0.03	5.80E-01	0.15
1588.1292@0.6266174	1588.1292	0.63	POS	hilic				2.13E-01	4.12E-02	-0.63	3.44E-01	0.27	8.24E-01	-0.07	3.53E-01	-0.25
1588.13@12.120664	1588.1300	12.12	POS	RP				4.44E-01	4.84E-01	-0.24	1.01E-01	0.47	5.84E-01	0.14	4.21E-01	0.23
1588.1451@0.65846175	1588.1451	0.66	NEG	hilic				3.19E-01	1.77E-01	0.37	3.35E-01	-0.25	3.20E-01	-0.29	3.95E-01	0.29
1588.305@0.37654302	1588.3050	0.38	NEG	hilic				5.80E-01	4.51E-02	0.61	2.68E-01	0.34	1.57E-01	0.42	2.86E-01	0.33
1588.3055@9.87399	1588.3055	9.87	NEG	RP				2.44E-01	2.28E-02	0.73	1.53E-02	0.67	1.02E-01	0.40	3.53E-01	0.28
1588.3094@9.807019	1588.3094	9.81	NEG	RP				9.49E-01	7.70E-01	0.08	6.60E-01	-0.10	8.47E-01	0.04	5.78E-01	0.15
1588.7831@0.5154435	1588.7831	0.52	POS	hilic				8.94E-01	4.53E-01	-0.19	8.47E-01	-0.05	4.09E-01	-0.27	7.98E-01	0.08
1589.1545@0.6587569	1589.1545	0.66	NEG	hilic				4.57E-01	5.13E-01	0.19	2.06E-01	-0.33	3.88E-01	-0.25	4.01E-01	0.27
1589.1584@12.640381	1589.1584	12.64	NEG	RP				7.36E-02	1.10E-01	0.46	2.93E-03	0.80	4.91E-03	0.72	1.20E-01	0.33
1589.3116@0.3764818	1589.3116	0.38	NEG	hilic				8.47E-01	2.78E-01	0.34	8.59E-01	-0.05	6.32E-01	0.14	5.31E-01	0.19
1590.0496@10.518847	1590.0496	10.52	POS	RP				6.79E-01	3.06E-01	0.28	7.41E-01	-0.08	9.38E-01	-0.02	2.89E-01	0.29
1590.0522@1.2126195	1590.0522	1.21	POS	hilic				6.76E-01	4.18E-01	0.28	6.49E-01	-0.12	2.73E-01	-0.29	7.04E-01	-0.10

Supplementary table 2: Metabolomics analysis results

Compound	Mass	Retention Time	Ionization mode	column	Compound_ID	MSMS_spectra	ID_level	ANOVA	Anorexia vs control		overweight vs control		obese vs control		athletes vs control	
								P-FDR	P	Estimate	P	Estimate	P	Estimate	P	Estimate
1590.1613@0.65846854	1590.1613	0.66	NEG	hilic				4.85E-01	5.29E-01	0.20	2.23E-01	-0.32	3.00E-01	-0.30	5.00E-01	0.21
1590.586@12.146762	1590.5860	12.15	POS	RP				7.93E-02	8.00E-02	-0.50	1.64E-01	0.37	6.67E-02	0.51	5.34E-01	0.18
1590.7823@0.5134107	1590.7823	0.51	POS	hilic				7.58E-01	2.11E-01	-0.38	7.34E-01	0.09	8.07E-01	-0.07	7.66E-01	0.09
1591.1451@12.406206	1591.1451	12.41	POS	RP				5.10E-01	5.50E-01	0.18	8.17E-02	-0.41	8.57E-01	-0.04	5.08E-01	-0.17
1591.1569@0.6291614	1591.1569	0.63	POS	hilic				2.95E-01	6.03E-01	-0.13	1.83E-01	0.38	6.60E-02	0.54	9.04E-01	-0.03
1591.1672@0.65821725	1591.1672	0.66	NEG	hilic				5.19E-01	1.11E-01	0.46	7.05E-01	-0.09	9.89E-01	0.00	4.67E-01	0.21
1591.1711@12.871727	1591.1711	12.87	NEG	RP				5.38E-01	1.08E-01	0.48	6.07E-01	0.13	4.40E-01	-0.20	9.32E-01	0.03
1592.0656@1.2081649	1592.0656	1.21	POS	hilic				3.62E-01	1.08E-01	0.49	2.90E-01	-0.31	5.90E-01	0.15	8.69E-01	0.05
1592.0706@10.518845	1592.0706	10.52	POS	RP				4.07E-02	9.41E-02	0.52	3.75E-02	-0.63	3.70E-01	-0.23	9.62E-01	-0.01
1592.175@12.87148	1592.1750	12.87	NEG	RP				2.41E-01	1.56E-02	0.68	8.95E-01	0.04	4.65E-01	-0.19	4.58E-01	0.24
1592.3221@0.38406447	1592.3221	0.38	NEG	hilic				9.51E-01	8.88E-01	0.04	9.84E-01	-0.01	3.61E-01	-0.21	9.97E-01	0.00
1592.7961@0.51765656	1592.7961	0.52	POS	hilic				8.38E-01	9.35E-01	0.02	5.69E-01	0.17	7.57E-01	0.09	1.66E-01	0.38
1593.049@12.3385315	1593.0490	12.34	POS	RP				2.03E-01	2.62E-01	0.32	1.92E-01	-0.34	2.33E-02	-0.50	6.87E-01	-0.10
1593.0507@12.176704	1593.0507	12.18	POS	RP				8.82E-01	2.36E-01	-0.34	4.87E-01	-0.19	5.67E-01	-0.15	3.09E-01	-0.23
1593.0713@1.2038908	1593.0713	1.20	POS	hilic				3.87E-01	8.02E-02	0.50	5.60E-01	-0.14	6.93E-01	0.10	8.54E-01	-0.04
1593.076@10.51661	1593.0760	10.52	POS	RP				1.15E-01	1.56E-01	0.45	7.72E-02	-0.49	2.92E-01	-0.28	9.74E-01	0.01
1593.1635@12.899502	1593.1635	12.90	POS	RP				5.87E-01	1.80E-01	-0.38	8.09E-01	-0.07	3.22E-01	0.26	7.88E-01	0.08
1593.1675@12.688036	1593.1675	12.69	POS	RP				4.06E-01	4.51E-02	-0.52	3.84E-01	-0.25	7.78E-01	-0.07	2.44E-02	-0.55
1593.972@9.958454	1593.9720	9.96	NEG	RP				5.80E-03	8.66E-01	0.04	4.77E-02	-0.48	3.73E-04	-0.88	4.96E-01	0.20
1594.1711@12.685363	1594.1711	12.69	POS	RP				6.05E-01	4.60E-02	-0.46	7.42E-01	-0.10	9.14E-01	-0.03	1.38E-01	-0.36
1594.1722@0.6302136	1594.1722	0.63	POS	hilic				2.76E-02	3.27E-02	0.58	1.54E-01	0.41	3.71E-04	0.97	8.63E-01	-0.05
1595.0627@12.348252	1595.0627	12.35	POS	RP				5.29E-01	4.02E-02	0.52	4.80E-01	0.21	7.16E-01	-0.10	2.33E-01	0.33
1595.1497@12.399446	1595.1497	12.40	NEG	RP				2.68E-01	6.27E-02	-0.50	3.62E-01	0.27	5.51E-01	0.16	4.24E-01	0.23
1595.18@12.887313	1595.1800	12.89	POS	RP				7.07E-01	3.65E-01	-0.22	9.17E-01	-0.02	4.78E-01	0.19	3.89E-01	0.24
1596.1545@12.400522	1596.1545	12.40	NEG	RP				1.72E-01	1.50E-03	-0.70	8.73E-01	0.04	4.66E-01	-0.18	8.97E-01	-0.03
1596.1833@12.897507	1596.1833	12.90	POS	RP				6.00E-01	3.53E-01	-0.25	9.96E-01	0.00	1.47E-01	0.40	9.49E-01	-0.02
1596.3195@0.38541025	1596.3195	0.39	NEG	hilic				5.12E-01	2.24E-01	0.40	6.15E-02	0.57	5.89E-01	0.14	1.52E-01	0.45
1597.1215@12.12756	1597.1215	12.13	NEG	RP				6.02E-01	4.49E-01	-0.19	7.82E-01	0.07	1.78E-01	0.43	8.99E-01	0.04
1597.1877@12.889677	1597.1877	12.89	POS	RP				3.87E-01	5.61E-01	-0.14	9.47E-01	-0.02	4.87E-02	0.54	7.33E-01	0.10
1598.0513@10.23781	1598.0513	10.24	NEG	RP				4.95E-02	6.96E-02	0.49	3.64E-01	-0.20	3.25E-02	-0.40	9.28E-01	0.02
1598.1086@11.971257	1598.1086	11.97	NEG	RP				1.11E-01	1.00E+00	0.00	3.15E-02	-0.49	6.54E-03	-0.69	7.58E-01	-0.09
1598.3322@9.857427	1598.3322	9.86	NEG	RP				6.82E-01	2.99E-01	0.34	6.49E-01	0.13	6.48E-01	0.15	4.50E-01	-0.24
1598.335@0.3853603	1598.3350	0.39	NEG	hilic				5.95E-01	2.70E-01	0.34	2.66E-01	0.34	8.07E-01	-0.07	1.36E-01	0.42
1599.106@12.1310835	1599.1060	12.13	NEG	RP				2.45E-02	7.18E-01	-0.11	9.08E-02	0.46	2.29E-03	0.88	7.52E-01	-0.10
1599.1746@12.684809	1599.1746	12.68	NEG	RP				2.04E-01	1.72E-02	-0.66	7.88E-01	-0.08	3.60E-01	-0.27	3.79E-01	0.25
1599.3394@0.38529307	1599.3394	0.39	NEG	hilic				5.47E-01	3.23E-01	0.30	8.09E-02	0.53	8.29E-01	0.06	1.53E-01	0.42
1601.0652@12.153228	1601.0652	12.15	POS	RP				8.44E-01	5.16E-01	0.17	7.15E-01	-0.09	4.68E-01	0.17	3.90E-01	0.20
1601.9865@10.234595	1601.9865	10.23	POS	RP				1.51E-01	5.86E-02	0.64	3.31E-01	-0.22	9.61E-01	-0.01	6.80E-01	0.11
1602.784@0.5189784	1602.7840	0.52	POS	hilic				8.31E-01	4.77E-01	-0.21	5.76E-01	0.17	7.36E-01	-0.09	5.81E-01	0.14
1604.7883@0.51867735	1604.7883	0.52	POS	hilic				6.09E-01	3.55E-01	-0.25	7.20E-01	0.11	7.29E-01	-0.09	2.32E-01	0.34
1606.8077@0.518923	1606.8077	0.52	POS	hilic				7.06E-01	8.53E-01	0.05	4.60E-01	-0.20	7.44E-01	0.09	2.72E-01	0.33
1607.8116@0.5188966	1607.8116	0.52	POS	hilic				7.31E-01	2.30E-01	-0.29	6.48E-01	-0.11	8.43E-01	0.05	4.90E-01	0.21
1608.0391@10.512588	1608.0391	10.51	POS	RP				3.23E-02	2.75E-01	0.31	7.77E-03	-0.69	1.83E-01	-0.33	2.12E-01	-0.35
1608.8975@9.963474	1608.8975	9.96	POS	RP				8.36E-03	2.81E-01	0.32	2.82E-01	-0.30	5.78E-03	-0.76	1.44E-01	0.42
1609.1259@12.942751	1609.1259	12.94	POS	RP				8.49E-01	8.89E-01	0.04	8.97E-01	-0.03	2.19E-01	0.33	8.46E-01	0.06
1610.9092@9.95334	1610.9092	9.95	POS	RP				2.73E-01	5.79E-01	0.16	4.38E-01	-0.24	8.07E-02	-0.46	2.63E-01	0.33
1611.915@9.959828	1611.9150	9.96	POS	RP				1.21E-01	3.18E-01	-0.30	1.19E-03	-0.77	3.11E-03	-0.61	1.06E-01	-0.46
1612.9366@10.239937	1612.9366	10.24	POS	RP				2.05E-03	8.55E-04	0.96	2.91E-01	-0.29	3.07E-01	-0.24	1.99E-01	0.36
1613.9196@9.960119	1613.9196	9.96	POS	RP				1.86E-02	8.47E-01	0.06	5.50E-02	-0.59	1.34E-03	-0.84	5.84E-01	0.16
1614.7812@0.46658087	1614.7812	0.47	POS	hilic				6.58E-01	6.53E-01	-0.13	3.40E-01	0.28	9.66E-01	0.01	1.92E-01	0.38
1614.9445@10.2419615	1614.9445	10.24	POS	RP				6.94E-02	6.72E-02	0.62	5.39E-01	-0.17	1.39E-01	-0.40	3.34E-01	0.28
1616.9497@1.2207981	1616.9497	1.22	POS	hilic				7.72E-01	4.64E-01	0.23	8.81E-01	-0.04	2.63E-01	-0.27	8.73E-01	0.04
1616.9576@10.238896	1616.9576	10.24	POS	RP				1.07E-02	1.48E-02	0.74	2.05E-01	-0.37	1.09E-01	-0.41	3.23E-01	0.30
1617.0295@6.025601	1617.0295	6.03	POS	hilic				5.76E-01	1.31E-01	0.41	3.18E-01	0.21	8.18E-01	-0.04	4.69E-01	0.15
1617.9543@1.2195255	1617.9543	1.22	POS	hilic				1.41E-02	8.67E-03	0.73	7.21E-01	-0.10	6.88E-02	-0.46	2.28E-01	0.33
1617.9637@10.237259	1617.9637	10.24	POS	RP				6.54E-03	1.79E-02	0.70	1.06E-01	-0.46	2.79E-02	-0.54	7.71E-01	0.09
1618.0522@12.144988	1618.0522	12.14	POS	RP				1.68E-01	1.65E-01	-0.36	2.91E-01	0.31	1.97E-01	0.39	3.06E-01	-0.29
1618.9708@1.2151415	1618.9708	1.22	POS	hilic				6.71E-01	5.29E-01	0.20	5.41E-01	-0.18	1.57E-01	-0.38	6.82E-01	-0.13
1618.9729@10.516544	1618.9729	10.52	POS	RP				6.36E-01	5.90E-01	0.14	6.90E-01	0.08	2.53E-01	0.25	1.02E-01	0.44
1619.9677@10.236664	1619.9677	10.24	POS	RP				1.18E-02	4.13E-02	0.64	1.04E-01	-0.50	4.96E-02	-0.51	8.62E-01	0.05
1620.8167@0.5019384	1620.8167	0.50	POS	hilic				5.41E-01	6.60E-02	-0.49	4.56E-01	-0.19	1.10E-01	-0.42	9.13E-01	-0.03
1621.0059@10.522613	1621.0059	10.52	POS	RP				3.00E-02	1.33E-02	0.68	1.70E-01	-0.38	9.74E-01	0.01	1.31E-01	0.49
1621.9912@1.2091938	1621.9912	1.21	POS	hilic				4.07E-01	1.04E-01	0.49	3.56E-01	-0.25	6.96E-01	0.11	8.06E-01	-0.07
1622.076@12.434221	1622.0760	12.43	POS	RP				3.47E-01	7.71E-01	0.08	1.57E-01	-0.39	6.11E-01	-0.15	2.84E-02	-0.54
1622.7996@0.5019893	1622.7996	0.50	POS	hilic				6.34E-01	3.25E-01	-0.29	5.65E-01	-0.14	7.93E-01	0.07	3.96E-01	0.28
1623.0052@10.5197	1623.0052	10.52	POS	RP				4.62E-02	7.38E-02	0.52	3.23E-02	-0.64	6.12E-01	-0.13	8.76E-01	-0.05
1624.0118@10.519721	1624.0118	10.52	POS	RP				3.64E-02	7.16E-02	0.55	4.01E-02	-0.62	4.76E-01	-0.19	8.20E-01	0.07
1625.1125@12.939776	1625.1125	12.94	POS	RP				4.83E-01	9.28E-01	-0.03	5.09E-02	-0.55	4.81E-01	-0.18	2.09E-01	-0.36
1626.0142@10.517666	1626.0142	10.52	POS	RP				4.60E-02	9.22E-02	0.50	3.61E-02	-0.62	3.74E-01	-0.24	9.98E-01	0.00
1626.8152@0.5020701	1626.8152	0.50	POS	hilic				8.64E-01	4.78E-01	-0.19	4.92E-01	0.17	8.91E-01	-0.04	7.84E-01	-0.07
1634.8105@0.5106465	1634.8105	0.51	POS	hilic				7.12E-02	8.68E-01	0.05	6.13E-01	0.13	6.58E-03	0.68	9.55E-03	0.76
1634.9095@9.944632	1634.9095	9.94	POS	RP				4.60E-01	8.12E-01	-0.06	2.67E-01	0.37	6.04E-02	0.56	3.26E-01	0.28
1635.815@0.5118473	1635.8150	0.51	POS	hilic				8.23E-01	3.35E-01	-0.30	5.26E-01	-0.15	4.22E-01	-0.17	7.56E-01	0.09

Supplementary table 2: Metabolomics analysis results

								ANOVA	Anorexia vs control		overweight vs control		obese vs control		athletes vs control	
Compound	Mass	Retention Time	Ionization mode	column	Compound_ID	MSMS_spectra	ID_level	P-FDR	P	Estimate	P	Estimate	P	Estimate	P	Estimate
1635.9117@9.945542	1635.9117	9.95	POS	RP				8.21E-01	3.61E-01	-0.24	8.14E-01	0.08	4.04E-01	0.23	7.71E-01	0.08
1639.0396@12.113133	1639.0396	12.11	POS	RP				7.82E-01	4.64E-01	-0.18	4.62E-01	0.19	5.61E-01	-0.13	8.35E-01	-0.05
1640.9597@1.2080277	1640.9597	1.21	POS	hilic				8.04E-01	7.95E-01	-0.08	2.06E-01	-0.37	3.93E-01	-0.23	9.82E-01	0.01
1641.0424@12.11406	1641.0424	12.11	POS	RP				1.35E-01	2.41E-01	0.33	1.16E-01	-0.47	3.46E-01	-0.28	6.95E-02	-0.50
1641.9666@1.2033187	1641.9666	1.20	POS	hilic				6.77E-01	9.79E-01	-0.01	1.66E-01	0.39	8.21E-01	-0.06	7.77E-01	-0.08
1643.076@12.377259	1643.0760	12.38	POS	RP				7.42E-01	6.22E-01	0.14	7.91E-01	0.07	1.29E-01	0.42	9.77E-01	-0.01
1644.8077@0.5013728	1644.8077	0.50	POS	hilic				9.11E-01	2.29E-01	-0.34	7.39E-01	-0.10	8.50E-01	-0.05	8.68E-01	-0.05
1645.08@12.38516	1645.0800	12.39	POS	RP				1.43E-01	9.55E-01	0.02	3.84E-02	-0.55	2.58E-01	0.32	2.96E-01	-0.27
1645.691@4.600519	1645.6910	4.60	NEG	hilic				7.98E-01	5.19E-01	-0.17	1.02E-01	-0.35	8.39E-01	-0.05	4.82E-01	-0.19
1646.8@0.50127363	1646.8000	0.50	POS	hilic				8.85E-01	3.62E-01	-0.26	8.83E-01	-0.04	7.00E-01	0.10	7.13E-01	0.11
1648.8173@0.5000001	1648.8173	0.50	POS	hilic				7.69E-01	2.32E-01	-0.37	8.62E-01	0.05	3.88E-01	-0.23	8.92E-01	0.04
1649.1049@12.894103	1649.1049	12.89	POS	RP				6.67E-02	9.93E-01	0.00	9.48E-01	0.02	7.92E-03	0.75	6.17E-01	-0.10
1649.8221@0.50344765	1649.8221	0.50	POS	hilic				9.19E-01	3.02E-01	-0.29	7.23E-01	-0.11	3.12E-01	-0.26	7.68E-01	-0.09
1650.814@0.4978677	1650.8140	0.50	POS	hilic				9.45E-01	4.55E-01	-0.18	9.72E-01	0.01	7.01E-01	0.11	9.39E-01	0.02
1654.8282@0.49786368	1654.8282	0.50	POS	hilic				8.41E-01	4.48E-01	-0.22	6.48E-01	0.12	4.98E-01	-0.21	7.45E-01	0.09
1655.7194@4.568835	1655.7194	4.57	NEG	hilic				2.77E-01	4.21E-01	0.27	4.63E-03	0.79	2.34E-01	0.33	1.33E-01	0.40
1655.7216@4.564802	1655.7216	4.56	NEG	hilic				2.21E-01	3.14E-02	0.65	1.04E-02	0.78	9.18E-02	0.49	5.15E-02	0.52
1658.8322@0.5033957	1658.8322	0.50	POS	hilic				8.51E-01	4.32E-01	0.20	1.88E-01	0.36	3.32E-01	0.25	3.59E-01	0.28
1660.82@0.5076168	1660.8200	0.51	POS	hilic				9.79E-01	7.61E-01	-0.10	6.99E-01	0.11	8.51E-01	-0.05	7.31E-01	-0.10
1665.0443@12.0764885	1665.0443	12.08	POS	RP				4.72E-01	2.60E-02	-0.56	9.62E-01	-0.01	8.95E-01	0.04	6.91E-01	-0.08
1672.8293@0.48954058	1672.8293	0.49	POS	hilic				6.34E-01	4.32E-01	-0.20	2.15E-01	0.30	7.93E-01	-0.07	5.00E-01	0.18
1674.8073@0.48998207	1674.8073	0.49	POS	hilic				7.90E-01	6.05E-01	-0.15	2.72E-01	0.28	6.99E-01	-0.11	7.13E-01	0.12
1676.8278@0.49614027	1676.8278	0.50	POS	hilic				6.20E-01	4.47E-01	-0.23	9.62E-01	0.01	1.36E-01	-0.50	2.71E-01	-0.32
1678.8303@0.49235126	1678.8303	0.49	POS	hilic				7.83E-01	2.39E-01	-0.35	9.62E-01	-0.01	2.24E-01	-0.36	6.98E-01	-0.12
1682.8531@0.51385003	1682.8531	0.51	POS	hilic				1.12E-01	9.96E-01	0.00	2.83E-01	-0.21	2.35E-01	0.33	9.41E-03	-0.45
1686.8477@0.49935946	1686.8477	0.50	POS	hilic				9.97E-01	9.69E-01	0.01	7.40E-01	-0.08	9.84E-01	-0.01	9.48E-01	0.02
1687.2751@3.1930628	1687.2751	3.19	NEG	hilic				4.97E-01	4.61E-01	0.24	2.52E-02	0.63	1.39E-01	0.39	1.41E-01	0.39
1687.2765@3.2529879	1687.2765	3.25	NEG	hilic				4.60E-01	6.71E-02	-0.42	3.47E-01	0.25	8.43E-01	0.05	6.02E-01	0.14
1688.8362@0.49871656	1688.8362	0.50	POS	hilic				8.01E-01	2.22E-01	-0.30	4.86E-01	-0.18	2.82E-01	-0.26	9.16E-01	0.03
1689.273@3.2242324	1689.2730	3.22	NEG	hilic				4.28E-01	7.14E-01	0.12	6.13E-02	0.56	9.43E-01	-0.02	1.33E-01	0.41
1702.8337@0.51403844	1702.8337	0.51	POS	hilic				9.43E-01	5.32E-01	-0.16	8.43E-01	-0.05	4.41E-01	-0.19	4.70E-01	-0.20
1703.83@0.49962255	1703.8300	0.50	POS	hilic				6.41E-01	6.59E-01	0.13	1.04E-01	0.46	9.87E-01	0.00	2.61E-01	0.31
1704.8339@0.5132054	1704.8339	0.51	POS	hilic				9.84E-01	8.27E-01	0.05	7.21E-01	0.09	8.18E-01	0.06	5.30E-01	0.19
1706.8538@0.5124939	1706.8538	0.51	POS	hilic				8.00E-01	2.59E-01	-0.29	3.96E-01	-0.24	8.42E-01	0.06	9.36E-01	0.02
1713.6779@4.599616	1713.6779	4.60	NEG	hilic				1.99E-01	9.51E-01	-0.01	6.91E-01	0.10	1.11E-01	0.48	1.48E-01	-0.32
1716.8352@0.5182711	1716.8352	0.52	POS	hilic				6.83E-01	3.68E-01	0.25	5.70E-01	0.15	4.14E-01	-0.18	3.33E-01	0.23
1718.8468@0.51335615	1718.8468	0.51	POS	hilic				5.10E-01	8.90E-01	-0.04	1.43E-01	-0.39	6.07E-02	-0.51	4.16E-01	-0.22
1720.8398@0.5209246	1720.8398	0.52	POS	hilic				9.51E-01	5.17E-01	-0.20	9.94E-01	0.00	5.62E-01	-0.16	4.65E-01	-0.20
1721.843@0.5246604	1721.8430	0.52	POS	hilic				7.31E-01	5.56E-01	-0.20	8.22E-01	0.06	9.65E-01	-0.01	2.08E-01	0.35
1722.8534@0.5209107	1722.8534	0.52	POS	hilic				8.17E-01	4.46E-01	-0.21	7.16E-01	-0.10	3.36E-01	-0.25	1.24E-01	-0.35
1723.7065@4.5693574	1723.7065	4.57	NEG	hilic				2.24E-01	2.39E-02	0.70	1.00E-01	0.49	8.63E-01	-0.05	3.52E-01	0.27
1723.7083@4.5697274	1723.7083	4.57	NEG	hilic				9.90E-02	2.16E-03	0.91	3.47E-02	0.63	2.47E-01	0.29	5.72E-01	0.18
1724.7104@4.5665383	1724.7104	4.57	NEG	hilic				1.01E-01	1.30E-02	0.74	3.35E-02	0.58	8.12E-01	-0.06	7.56E-01	0.09
1724.8525@0.5050948	1724.8525	0.51	POS	hilic				7.69E-01	3.78E-01	-0.26	2.41E-01	-0.32	1.27E-01	-0.44	4.85E-01	-0.21
1732.8365@0.5329093	1732.8365	0.53	POS	hilic				2.04E-01	4.79E-01	-0.19	8.38E-01	0.05	1.52E-01	-0.34	1.20E-01	0.44
1734.8481@0.5283821	1734.8481	0.53	POS	hilic				4.20E-01	9.27E-01	0.03	5.26E-01	0.17	1.88E-01	-0.39	3.24E-01	0.30
1736.8654@0.52984625	1736.8654	0.53	POS	hilic				4.43E-01	7.99E-01	-0.07	3.42E-01	0.25	5.88E-01	0.15	6.68E-02	0.59
1737.868@0.5326191	1737.8680	0.53	POS	hilic				9.18E-01	5.95E-01	-0.15	9.63E-01	0.01	6.29E-01	-0.13	6.27E-01	0.14
1743.6458@4.4032364	1743.6458	4.40	POS	hilic				5.35E-01	3.25E-01	0.34	2.92E-02	0.54	5.03E-01	0.21	9.62E-01	-0.01
1748.856@0.51924133	1748.8560	0.52	POS	hilic				8.57E-01	9.81E-01	0.01	4.84E-01	0.19	8.09E-01	0.07	5.03E-01	-0.21
1756.9432@11.881151	1756.9432	11.88	POS	RP				4.53E-01	2.04E-01	0.36	9.72E-01	0.01	1.70E-01	0.39	5.05E-01	-0.15
1758.8372@0.4925349	1758.8372	0.49	POS	hilic				7.96E-01	6.29E-01	-0.12	7.34E-01	0.09	6.55E-01	0.13	2.43E-01	0.34
1762.8564@0.49220183	1762.8564	0.49	POS	hilic				9.57E-01	4.58E-01	-0.24	9.61E-01	-0.01	5.98E-01	-0.14	9.96E-01	0.00
1764.8665@0.49761263	1764.8665	0.50	POS	hilic				8.81E-01	6.59E-01	-0.13	8.32E-01	0.06	6.41E-01	-0.13	3.88E-01	0.23
1764.9753@12.00664	1764.9753	12.01	POS	RP				3.12E-02	6.96E-01	0.11	1.10E-03	-0.78	4.15E-02	-0.58	2.30E-01	-0.32
1766.8853@0.4955043	1766.8853	0.50	POS	hilic				7.14E-01	3.23E-01	-0.26	6.61E-01	-0.12	5.12E-01	-0.17	4.28E-01	0.27
1767.0059@12.422339	1767.0059	12.42	POS	RP				9.77E-01	9.69E-01	0.01	8.40E-01	0.05	5.02E-01	0.18	9.71E-01	-0.01
1769.0164@12.418924	1769.0164	12.42	POS	RP				9.65E-01	7.57E-01	0.09	7.33E-01	-0.10	6.49E-01	0.13	8.77E-01	-0.05
1769.04@12.966394	1769.0400	12.97	POS	RP				6.58E-01	5.91E-01	-0.15	1.64E-01	-0.37	9.25E-02	-0.44	2.06E-01	-0.36
1771.0352@12.968579	1771.0352	12.97	POS	RP				8.92E-01	7.14E-01	0.08	8.15E-01	0.05	7.79E-01	0.07	2.87E-01	0.29
1773.0446@12.93629	1773.0446	12.94	POS	RP				8.66E-01	3.93E-01	-0.23	3.30E-01	-0.28	2.41E-01	-0.32	3.03E-01	-0.30
1774.873@0.51311845	1774.8730	0.51	POS	hilic				7.05E-01	2.15E-01	-0.34	1.50E-01	-0.35	2.79E-01	-0.30	1.07E-01	-0.44
1776.8628@0.5131415	1776.8628	0.51	POS	hilic				8.98E-01	9.97E-01	0.00	7.01E-01	0.11	6.21E-01	0.15	2.90E-01	0.33
1777.8752@0.5132633	1777.8752	0.51	POS	hilic				8.99E-01	7.94E-01	0.07	4.45E-01	0.20	9.07E-01	0.03	3.72E-01	0.29
1781.6656@4.597471	1781.6656	4.60	NEG	hilic				4.93E-01	2.03E-02	0.58	3.81E-01	0.24	8.13E-01	0.06	6.20E-01	0.13
1786.8655@0.49768054	1786.8655	0.50	POS	hilic				7.80E-01	7.55E-01	-0.09	9.73E-01	-0.01	6.39E-01	-0.13	2.31E-01	0.31
1790.8789@0.49763885	1790.8789	0.50	POS	hilic				6.83E-01	7.72E-01	-0.08	3.97E-01	0.23	8.79E-01	0.04	1.96E-01	0.38
1791.6971@4.5675044	1791.6971	4.57	NEG	hilic				2.77E-01	2.18E-02	0.71	1.44E-01	0.42	7.53E-02	0.56	7.05E-01	0.11
1791.6974@4.5654397	1791.6974	4.57	NEG	hilic				2.42E-01	1.96E-02	0.77	5.94E-02	0.54	7.48E-02	0.56	4.64E-01	0.22
1808.8925@0.57567865	1808.8925	0.58	POS	hilic				9.22E-01	6.37E-01	0.13	7.02E-01	0.10	5.79E-01	0.17	2.87E-01	0.31
1809.8918@0.57922643	1809.8918	0.58	POS	hilic				6.45E-01	6.45E-01	-0.12	4.07E-01	-0.23	1.26E-01	-0.33	6.19E-01	0.13
1810.9778@12.078816	1810.9778	12.08	POS	RP				2.82E-01	2.55E-02	-0.59	9.45E-02	-0.46	2.10E-01	-0.39	6.80E-03	-0.70
1812.9803@12.076583	1812.9803	12.08	POS	RP				2.10E-01	2.17E-02	-0.58	2.00E-01	-0.38	9.31E-01	0.02	1.68E-02	-0.60

Supplementary table 2: Metabolomics analysis results

Compound	Mass	Retention Time	Ionization mode	column	Compound_ID	MSMS_spectra	ID_level	ANOVA P-FDR	Anorexia vs control P	Anorexia vs control Estimate	overweight vs control P	overweight vs control Estimate	obese vs control P	obese vs control Estimate	athletes vs control P	athletes vs control Estimate
1816.882@0.49977335	1816.8820	0.50	POS	hilic				6.65E-01	6.65E-01	-0.11	4.34E-01	-0.18	3.05E-01	-0.29	4.80E-01	0.22
1818.8986@0.49773997	1818.8986	0.50	POS	hilic				7.30E-01	5.93E-01	0.15	2.98E-01	0.27	7.23E-01	0.11	1.52E-01	0.48
1820.8932@0.5771744	1820.8932	0.58	POS	hilic				4.49E-01	2.74E-02	-0.56	1.27E-01	-0.40	1.19E-01	-0.43	7.37E-01	-0.10
1822.8907@0.57938147	1822.8907	0.58	POS	hilic				8.74E-01	7.69E-01	-0.08	8.64E-01	-0.05	2.19E-01	-0.34	9.95E-01	0.00
1823.9023@0.5807468	1823.9023	0.58	POS	hilic				8.96E-01	3.25E-01	-0.25	3.15E-01	-0.27	2.84E-01	-0.28	5.03E-01	-0.18
1824.9163@0.57324994	1824.9163	0.57	POS	hilic				2.13E-01	3.87E-01	-0.20	2.21E-02	-0.47	4.24E-01	-0.21	3.50E-01	0.26
1830.9034@0.5414825	1830.9034	0.54	POS	hilic				2.57E-01	2.41E-02	-0.62	2.49E-01	-0.32	1.02E-02	-0.75	3.50E-01	-0.29
1832.9012@0.5098855	1832.9012	0.51	POS	hilic				4.78E-01	8.73E-02	-0.59	8.55E-02	-0.43	1.32E-01	-0.44	5.09E-01	-0.19
1834.9052@0.51491797	1834.9052	0.51	POS	hilic				3.35E-01	7.09E-02	-0.47	7.94E-01	-0.07	7.17E-01	0.10	3.08E-01	0.26
1836.9022@0.5526111	1836.9022	0.55	POS	hilic				6.09E-01	2.86E-01	-0.27	6.97E-01	-0.10	2.71E-01	0.28	7.02E-01	-0.10
1839.6262@4.6138644	1839.6262	4.61	NEG	hilic				7.58E-01	9.54E-01	-0.01	1.28E-01	0.35	3.46E-01	0.24	4.61E-01	0.18
1846.8853@0.4915437	1846.8853	0.49	POS	hilic				5.47E-01	5.96E-01	-0.17	8.28E-01	-0.05	2.29E-01	-0.36	3.15E-01	0.31
1848.8949@0.5037078	1848.8949	0.50	POS	hilic				5.73E-01	3.29E-01	-0.31	2.37E-01	0.31	5.13E-01	-0.17	8.61E-01	0.05
1849.653@4.5963373	1849.6530	4.60	NEG	hilic				7.70E-01	4.84E-01	0.16	1.18E-01	0.40	3.56E-01	0.25	2.94E-01	0.29
1850.9069@0.5121043	1850.9069	0.51	POS	hilic				7.32E-01	8.53E-01	-0.06	5.41E-01	0.16	4.76E-01	-0.22	3.01E-01	0.30
1851.9056@0.52038455	1851.9056	0.52	POS	hilic				6.31E-01	8.51E-01	-0.05	4.84E-01	0.21	2.84E-01	-0.31	4.27E-01	0.22
1854.9275@0.52268755	1854.9275	0.52	POS	hilic				5.43E-01	3.27E-01	-0.33	2.40E-01	-0.31	6.22E-02	-0.44	7.11E-01	0.11
1855.1956@3.1812334	1855.1956	3.18	NEG	hilic				3.58E-01	1.47E-01	0.47	2.78E-01	-0.29	2.92E-01	0.26	5.68E-01	0.16
1855.1975@3.2371664	1855.1975	3.24	NEG	hilic				6.78E-01	1.60E-01	0.50	4.76E-01	0.17	7.68E-01	0.09	2.20E-01	0.38
1857.1989@3.2358017	1857.1989	3.24	NEG	hilic				9.29E-01	5.00E-01	0.22	4.19E-01	0.22	8.78E-01	0.04	3.61E-01	0.26
1859.1982@3.2281175	1859.1982	3.23	NEG	hilic				6.67E-01	9.85E-01	-0.01	6.02E-01	0.15	3.18E-01	0.27	7.61E-02	0.50
1859.6827@4.566483	1859.6827	4.57	NEG	hilic				1.38E-01	2.86E-03	0.91	4.08E-02	0.66	5.66E-02	0.48	1.94E-01	0.39
1859.6833@4.565561	1859.6833	4.57	NEG	hilic				5.19E-02	3.34E-04	1.05	2.17E-02	0.71	1.34E-01	0.42	8.17E-02	0.47
1860.6855@4.5657296	1860.6855	4.57	NEG	hilic				5.16E-01	5.12E-02	0.50	1.56E-01	0.42	9.75E-01	-0.01	2.67E-01	0.35
1864.9167@0.55215144	1864.9167	0.55	POS	hilic				5.91E-01	6.56E-01	0.13	4.26E-01	0.23	3.70E-01	-0.25	2.99E-01	0.32
1865.9178@0.5537195	1865.9178	0.55	POS	hilic				9.68E-01	4.28E-01	0.21	6.35E-01	0.13	6.13E-01	0.14	9.70E-01	0.01
1866.9294@0.55453	1866.9294	0.55	POS	hilic				8.13E-01	6.26E-01	-0.14	4.20E-01	-0.20	4.28E-01	-0.23	5.65E-01	0.16
1874.908@0.47643256	1874.9080	0.48	POS	hilic				9.02E-01	9.46E-01	-0.02	5.09E-01	0.19	7.98E-01	-0.07	4.28E-01	0.23
1876.9124@0.47677997	1876.9124	0.48	POS	hilic				5.08E-01	6.56E-01	0.15	4.79E-01	0.19	8.33E-01	-0.06	3.29E-02	0.57
1879.6235@4.41014	1879.6235	4.41	POS	hilic				7.13E-01	5.36E-01	0.22	7.16E-01	-0.09	2.02E-01	-0.30	4.49E-01	-0.25
1883.406@11.040622	1883.4060	11.04	NEG	RP				3.69E-01	2.38E-01	0.27	8.55E-01	-0.05	4.41E-02	0.51	7.34E-01	0.08
1886.9388@0.5142988	1886.9388	0.51	POS	hilic				8.98E-01	3.27E-01	-0.25	8.66E-01	0.05	9.23E-01	-0.02	5.19E-01	-0.18
1892.9211@0.5206863	1892.9211	0.52	POS	hilic				8.76E-01	8.98E-01	-0.04	6.10E-01	0.15	7.40E-01	-0.09	3.64E-01	0.26
1893.925@0.52219236	1893.9250	0.52	POS	hilic				5.57E-01	9.76E-01	-0.01	9.12E-01	0.03	9.77E-02	-0.48	6.47E-01	0.12
1894.9327@0.52212626	1894.9327	0.52	POS	hilic				7.50E-01	9.68E-01	0.01	7.96E-01	0.08	7.78E-01	-0.07	1.40E-01	0.40
1900.934@0.51893175	1900.9340	0.52	POS	hilic				9.65E-01	5.15E-01	-0.18	4.51E-01	-0.19	5.64E-01	-0.15	9.41E-01	-0.02
1902.934@0.51862264	1902.9340	0.52	POS	hilic				8.01E-01	8.87E-01	-0.04	2.14E-01	0.36	7.62E-01	0.09	4.07E-01	0.23
1904.9283@0.51812845	1904.9283	0.52	POS	hilic				9.95E-01	9.18E-01	-0.03	8.94E-01	0.04	7.74E-01	0.10	8.98E-01	-0.04
1906.929@0.51656365	1906.9290	0.52	POS	hilic				5.33E-01	1.17E-01	-0.41	7.18E-01	0.10	3.83E-01	-0.28	5.91E-01	0.16
1907.9327@0.5203964	1907.9327	0.52	POS	hilic				3.99E-01	2.45E-01	-0.35	3.34E-01	0.27	4.07E-01	-0.23	4.00E-01	0.25
1908.9406@0.51918757	1908.9406	0.52	POS	hilic				9.01E-01	6.36E-01	-0.13	4.87E-01	0.20	8.82E-01	-0.04	5.71E-01	0.16
1912.9489@0.5034475	1912.9489	0.50	POS	hilic				6.10E-01	7.74E-02	-0.54	8.75E-01	-0.05	8.88E-01	0.04	6.59E-01	-0.11
1914.9445@0.50485843	1914.9445	0.50	POS	hilic				7.31E-01	1.30E-01	-0.46	2.28E-01	-0.34	4.56E-01	-0.23	8.20E-01	-0.07
1916.9445@0.5162683	1916.9445	0.52	POS	hilic				6.89E-01	3.13E-01	-0.27	5.44E-01	0.18	4.07E-01	-0.22	7.59E-01	0.08
1917.6425@4.595864	1917.6425	4.60	NEG	hilic				8.59E-01	5.11E-01	0.17	7.08E-01	0.10	1.43E-01	0.35	6.65E-01	0.12
1920.952@0.5288281	1920.9520	0.53	POS	hilic				7.63E-01	5.31E-01	-0.16	5.77E-01	0.14	6.69E-01	-0.11	3.89E-01	0.24
1927.674@4.5660267	1927.6740	4.57	NEG	hilic				6.66E-01	5.15E-02	0.57	2.84E-01	0.33	2.28E-01	0.33	3.24E-01	0.29
1927.6746@4.560678	1927.6746	4.56	NEG	hilic				5.94E-01	1.85E-01	0.37	1.32E-01	0.42	5.72E-01	0.16	7.94E-01	-0.07
1932.9489@0.5013439	1932.9489	0.50	POS	hilic				7.05E-01	3.19E-01	-0.28	6.22E-01	0.15	4.12E-01	-0.22	6.24E-01	0.14
1934.9323@0.5013425	1934.9323	0.50	POS	hilic				3.54E-01	8.19E-02	-0.50	5.68E-01	0.15	6.74E-02	-0.45	7.57E-01	-0.10
1936.9456@0.502143	1936.9456	0.50	POS	hilic				6.63E-01	3.99E-01	-0.24	3.61E-01	0.27	4.77E-01	-0.20	6.74E-01	0.12
1940.9626@0.5055001	1940.9626	0.51	POS	hilic				5.58E-01	2.03E-01	0.37	8.97E-01	-0.04	9.95E-01	0.00	1.25E-01	0.41
1942.9725@0.5221334	1942.9725	0.52	POS	hilic				8.59E-01	5.40E-01	-0.16	5.48E-01	0.15	7.72E-01	0.08	4.28E-01	0.21
1946.9594@0.5071887	1946.9594	0.51	POS	hilic				4.94E-01	7.82E-03	-0.52	5.96E-01	-0.13	9.81E-01	0.01	8.20E-01	-0.06
1952.9548@0.5941471	1952.9548	0.59	POS	hilic				8.62E-01	2.26E-01	0.29	9.90E-01	0.00	7.74E-01	0.07	9.58E-01	0.01
1962.956@0.5224029	1962.9560	0.52	POS	hilic				7.99E-01	7.26E-01	0.09	7.74E-01	0.08	4.94E-01	-0.19	3.63E-01	0.24
1966.9673@0.5232903	1966.9673	0.52	POS	hilic				9.48E-02	8.99E-02	0.46	2.02E-01	-0.23	1.48E-01	-0.27	8.95E-01	0.03
1975.5948@4.613224	1975.5948	4.61	NEG	hilic				6.40E-01	5.45E-01	0.14	1.43E-01	0.40	8.27E-01	-0.05	9.53E-01	-0.01
1980.9583@0.54510975	1980.9583	0.55	POS	hilic				5.33E-01	9.10E-01	0.03	3.04E-01	0.27	2.21E-01	-0.32	5.41E-01	-0.18
1981.972@0.5625189	1981.9720	0.56	POS	hilic				6.40E-01	8.08E-01	-0.07	4.65E-01	-0.19	8.97E-02	-0.43	7.80E-01	0.08
1985.6298@4.5951586	1985.6298	4.60	NEG	hilic				8.96E-01	6.83E-01	0.11	5.17E-01	0.16	4.95E-01	0.17	2.28E-01	0.31
1988.956@0.5609615	1988.9560	0.56	POS	hilic				9.31E-01	5.58E-01	-0.16	4.79E-01	-0.17	5.24E-01	-0.15	8.75E-01	0.04
1990.9716@0.56240827	1990.9716	0.56	POS	hilic				9.53E-01	9.80E-01	0.01	6.66E-01	0.12	6.23E-01	0.14	4.02E-01	0.23
1992.9615@0.56125236	1992.9615	0.56	POS	hilic				7.20E-01	7.24E-01	0.11	5.59E-01	-0.15	2.95E-01	-0.29	4.81E-01	0.20
1994.97@0.5633444	1994.9700	0.56	POS	hilic				4.43E-01	4.94E-02	-0.54	5.12E-01	-0.17	7.78E-01	-0.07	5.84E-01	0.16
1995.658@4.56545	1995.6580	4.57	NEG	hilic				3.72E-01	1.81E-02	0.75	2.74E-01	0.34	1.67E-01	0.37	7.23E-02	0.50
1995.6594@4.560377	1995.6594	4.56	NEG	hilic				1.63E-01	9.97E-03	0.86	3.79E-02	0.56	5.64E-01	0.16	2.44E-01	0.31
1995.9878@0.5647032	1995.9878	0.56	POS	hilic				7.64E-01	6.94E-01	0.11	1.80E-01	0.36	1.70E-01	0.39	3.44E-01	0.28
1996.6635@4.5630827	1996.6635	4.56	NEG	hilic				9.60E-01	4.10E-01	0.23	4.35E-01	0.22	8.58E-01	0.06	6.69E-01	0.12
1996.9858@0.5643909	1996.9858	0.56	POS	hilic				7.88E-01	3.29E-01	-0.30	5.88E-01	-0.16	2.79E-01	-0.30	7.26E-01	0.10
1997.9948@0.5665305	1997.9948	0.57	POS	hilic				9.02E-01	7.64E-01	0.09	6.47E-01	-0.10	9.16E-01	-0.03	4.43E-01	0.19
2003.2979@10.059815	2003.2979	10.06	POS	RP				9.91E-01	7.72E-01	0.08	8.71E-01	-0.05	7.25E-01	-0.09	8.97E-01	0.04
2003.2983@10.114914	2003.2983	10.11	POS	RP				9.73E-01	7.67E-01	0.08	5.00E-01	0.20	9.61E-01	-0.01	7.06E-01	0.12

Supplementary table 2: Metabolomics analysis results

Compound	Mass	Retention Time	Ionization mode	column	Compound_ID	MSMS_spectra	ID_level	ANOVA	Anorexia vs control		overweight vs control		obese vs control		athletes vs control	
								P-FDR	P	Estimate	P	Estimate	P	Estimate	P	Estimate
2004.304@10.112923	2004.3040	10.11	POS	RP				6.36E-01	9.58E-02	0.43	2.96E-01	0.33	7.92E-01	-0.08	3.27E-01	0.27
2005.007@11.869794	2005.0070	11.87	NEG	RP				5.57E-01	4.20E-01	0.23	5.85E-01	0.13	4.69E-02	0.55	3.35E-01	0.25
2007.0251@3.0306666	2007.0251	3.03	POS	hilic				7.64E-01	9.09E-01	-0.04	5.83E-01	0.16	5.77E-01	-0.15	2.65E-01	-0.32
2008.9796@0.563976	2008.9796	0.56	POS	hilic				4.94E-01	3.99E-01	-0.23	9.13E-01	0.03	1.80E-01	-0.35	3.73E-01	0.26
2009.0175@3.0394173	2009.0175	3.04	POS	hilic				7.89E-01	5.94E-01	0.17	2.89E-01	0.32	7.62E-01	-0.10	8.06E-01	-0.07
2015.5948@4.410972	2015.5948	4.41	POS	hilic				6.67E-01	2.72E-01	0.32	2.10E-01	0.34	8.84E-01	-0.05	2.00E-01	0.38
2017.2375@1.3328668	2017.2375	1.33	POS	hilic				9.60E-01	4.95E-01	0.17	7.04E-01	0.09	9.40E-01	0.02	7.95E-01	-0.06
2019.2758@10.101615	2019.2758	10.10	POS	RP				3.75E-01	5.83E-02	0.51	9.08E-01	0.04	3.83E-01	-0.26	3.66E-01	0.26
2020.9799@0.5040199	2020.9799	0.50	POS	hilic				8.57E-01	5.77E-01	-0.17	7.13E-01	0.11	7.09E-01	0.11	3.23E-01	0.25
2022.9843@0.50207496	2022.9843	0.50	POS	hilic				8.27E-01	3.33E-01	-0.27	9.35E-01	-0.02	7.71E-01	-0.09	4.96E-01	0.20
2023.118@3.2242053	2023.1180	3.22	NEG	hilic				9.18E-01	3.64E-01	0.30	9.60E-01	0.02	4.27E-01	0.19	7.18E-01	0.11
2023.1215@3.173934	2023.1215	3.17	NEG	hilic				2.01E-01	1.09E-01	0.52	3.12E-01	-0.26	9.26E-01	0.03	2.09E-01	-0.34
2025.1185@3.1919992	2025.1185	3.19	NEG	hilic				5.19E-01	7.90E-02	0.56	6.56E-01	0.13	9.78E-01	-0.01	1.82E-01	0.38
2027.0024@0.50674105	2027.0024	0.51	POS	hilic				8.84E-01	5.51E-01	-0.18	6.53E-01	0.12	7.45E-01	-0.10	5.39E-01	0.17
2027.1191@3.2270408	2027.1191	3.23	NEG	hilic				8.58E-01	8.19E-01	0.07	2.27E-01	0.33	9.14E-01	-0.03	6.00E-01	0.14
2027.1215@3.2099354	2027.1215	3.21	NEG	hilic				9.03E-01	3.64E-01	0.24	6.47E-01	0.14	9.39E-01	0.02	2.65E-01	0.30
2027.2979@10.002846	2027.2979	10.00	POS	RP				2.20E-03	1.55E-04	1.04	5.64E-01	-0.17	7.08E-01	-0.10	1.64E-02	0.64
2029.1143@3.2239792	2029.1143	3.22	NEG	hilic				4.21E-02	1.86E-01	0.39	9.83E-03	-0.67	8.09E-01	0.07	3.31E-01	-0.24
2036.2552@10.062419	2036.2552	10.06	POS	RP				8.93E-01	5.66E-01	-0.16	4.55E-01	0.22	8.69E-01	0.05	8.13E-01	-0.08
2036.9886@0.532299	2036.9886	0.53	POS	hilic				5.99E-01	8.05E-01	-0.07	9.55E-02	0.47	5.12E-01	0.21	2.90E-01	0.28
2037.2596@10.108009	2037.2596	10.11	POS	RP				1.32E-01	5.68E-02	0.61	3.58E-01	0.29	1.91E-01	0.37	2.21E-03	0.90
2038.0052@0.5336438	2038.0052	0.53	POS	hilic				8.61E-01	1.42E-01	-0.36	4.97E-01	-0.17	7.86E-01	-0.08	8.72E-01	-0.05
2038.2548@10.107494	2038.2548	10.11	POS	RP				9.18E-01	8.19E-01	0.06	7.37E-01	0.07	2.95E-01	0.25	4.28E-01	0.23
2039.0013@0.5548829	2039.0013	0.55	POS	hilic				7.78E-01	8.02E-01	-0.07	4.34E-01	0.22	4.43E-01	-0.23	6.90E-01	0.12
2040.2535@10.106682	2040.2535	10.11	POS	RP				7.41E-03	9.96E-03	0.80	9.47E-01	-0.02	3.02E-01	0.30	4.33E-04	1.02
2043.2502@10.101452	2043.2502	10.10	POS	RP				3.09E-02	5.37E-03	0.83	9.66E-01	0.01	3.35E-01	-0.28	8.59E-02	0.45
2043.2523@1.3283249	2043.2523	1.33	POS	hilic				1.79E-01	5.76E-01	-0.13	9.91E-01	0.00	4.37E-01	-0.16	6.21E-02	0.51
2045.2697@1.3231974	2045.2697	1.32	POS	hilic				6.65E-01	4.44E-01	-0.24	3.59E-01	-0.27	7.04E-02	-0.53	5.13E-01	-0.19
2046.9778@0.50787854	2046.9778	0.51	POS	hilic				2.61E-01	4.18E-01	0.23	1.51E-02	0.60	7.33E-01	-0.08	7.55E-01	0.08
2047.2784@1.3269849	2047.2784	1.33	POS	hilic				8.29E-01	7.50E-01	-0.09	7.97E-01	0.07	6.10E-01	-0.13	3.86E-01	0.24
2049.2754@10.00041	2049.2754	10.00	POS	RP				4.96E-03	2.76E-03	0.76	1.92E-01	-0.40	1.33E-01	-0.39	1.33E-01	0.41
2049.3098@1.2856857	2049.3098	1.29	POS	hilic				6.41E-01	8.92E-01	0.04	2.33E-01	0.30	8.45E-01	-0.04	4.58E-01	-0.15
2051.0027@0.5072071	2051.0027	0.51	POS	hilic				9.84E-01	7.17E-01	-0.12	8.06E-01	0.07	7.51E-01	-0.09	8.87E-01	0.04
2051.2961@10.0016165	2051.2961	10.00	POS	RP				1.33E-02	7.41E-03	0.70	3.02E-01	-0.32	1.23E-01	-0.41	1.38E-01	0.40
2053.2163@1.3136225	2053.2163	1.31	POS	hilic				5.47E-01	7.98E-01	-0.07	1.38E-01	-0.31	1.53E-01	-0.34	6.23E-01	0.12
2053.6167@4.5912724	2053.6167	4.59	NEG	hilic				9.01E-01	6.34E-01	0.13	5.98E-01	-0.13	5.15E-01	0.16	9.93E-01	0.00
2057.2412@1.314304	2057.2412	1.31	POS	hilic				8.51E-01	7.34E-01	0.10	8.25E-01	0.05	8.52E-01	-0.05	2.05E-01	0.31
2057.2432@1.1527016	2057.2432	1.15	POS	hilic				9.18E-01	8.40E-01	0.07	8.03E-01	-0.05	3.47E-01	0.23	6.28E-01	0.13
2057.2434@10.130915	2057.2434	10.13	POS	RP				3.28E-02	2.63E-02	0.67	8.94E-01	-0.04	8.07E-02	-0.46	1.39E-01	0.41
2058.2422@10.001001	2058.2422	10.00	POS	RP				4.47E-04	9.97E-04	0.99	8.96E-02	-0.39	8.68E-01	-0.05	6.40E-03	0.72
2059.249@10.131264	2059.2490	10.13	POS	RP				2.97E-02	2.45E-02	0.68	8.75E-01	-0.05	8.46E-02	-0.45	1.36E-01	0.41
2059.2522@10.000704	2059.2522	10.00	POS	RP				5.86E-04	7.43E-04	1.00	8.09E-02	-0.40	8.35E-01	0.05	2.21E-02	0.65
2060.255@10.130856	2060.2550	10.13	POS	RP				3.97E-02	3.04E-02	0.67	8.58E-01	-0.06	7.66E-02	-0.45	1.94E-01	0.35
2061.261@10.129291	2061.2610	10.13	POS	RP				2.76E-02	2.75E-02	0.67	6.97E-01	-0.12	5.03E-02	-0.49	2.32E-01	0.33
2061.27@1.2896504	2061.2700	1.29	POS	hilic				2.08E-01	1.14E-01	-0.55	1.38E-02	-0.67	8.91E-03	-0.68	4.19E-01	-0.25
2063.645@4.5599656	2063.6450	4.56	NEG	hilic				7.88E-01	2.57E-01	0.33	2.90E-01	0.34	9.49E-01	-0.02	5.13E-01	0.19
2063.6475@4.5629315	2063.6475	4.56	NEG	hilic				8.09E-01	7.18E-02	0.45	4.60E-01	0.25	5.52E-01	0.15	3.58E-01	0.29
2067.0088@0.58455384	2067.0088	0.58	POS	hilic				8.75E-01	2.66E-01	-0.31	9.52E-01	0.02	9.96E-01	0.00	8.03E-01	-0.07
2069.0034@0.6090468	2069.0034	0.61	POS	hilic				6.19E-01	3.78E-01	-0.21	2.04E-01	0.31	7.12E-01	-0.10	8.84E-01	-0.04
2075.2856@9.997407	2075.2856	10.00	POS	RP				1.92E-01	6.13E-01	-0.13	2.35E-01	-0.29	2.91E-04	-0.71	6.61E-01	-0.12
2077.0137@0.51187754	2077.0137	0.51	POS	hilic				9.18E-01	3.15E-01	-0.31	9.69E-01	-0.01	8.84E-01	-0.04	7.64E-01	-0.09
2079.019@0.5104787	2079.0190	0.51	POS	hilic				1.02E-01	3.56E-02	-0.65	3.02E-01	-0.28	5.82E-01	0.15	4.09E-01	0.25
2081.348@10.222126	2081.3480	10.22	POS	RP				4.11E-01	2.83E-01	-0.35	7.71E-01	-0.07	8.04E-01	0.06	2.15E-01	0.38
2083.02@0.59978265	2083.0200	0.60	POS	hilic				5.96E-01	9.07E-01	-0.03	2.76E-01	-0.26	9.06E-02	-0.42	2.44E-01	-0.32
2084.2517@9.988575	2084.2517	9.99	POS	RP				3.70E-03	2.56E-02	0.63	2.20E-01	-0.34	1.02E-01	-0.39	2.59E-02	0.67
2091.0554@0.569736	2091.0554	0.57	POS	hilic				6.08E-01	2.85E-01	-0.28	9.34E-01	-0.02	2.78E-01	-0.28	9.69E-02	-0.52
2093.0205@0.4936633	2093.0205	0.49	POS	hilic				8.67E-01	2.58E-01	-0.35	4.14E-01	-0.21	3.98E-01	-0.22	9.17E-01	-0.03
2097.2527@9.966207	2097.2527	9.97	POS	RP				1.66E-01	1.43E-01	0.42	6.89E-01	-0.13	9.80E-02	-0.50	4.84E-01	0.21
2099.2893@9.965369	2099.2893	9.97	POS	RP				7.23E-02	3.06E-01	0.29	3.30E-01	-0.31	1.25E-02	-0.72	7.68E-01	0.09
2100.3015@9.965206	2100.3015	9.97	POS	RP				6.12E-02	3.87E-01	0.23	2.62E-01	-0.33	6.46E-03	-0.81	9.28E-01	-0.03
2101.3103@10.240923	2101.3103	10.24	POS	RP				2.38E-01	3.45E-01	0.32	1.57E-01	-0.38	1.38E-01	-0.32	4.94E-01	0.18
2103.33@10.236261	2103.3300	10.24	POS	RP				3.76E-03	2.38E-02	0.67	7.46E-02	-0.52	1.25E-02	-0.61	9.21E-01	0.03
2105.2434@1.173551	2105.2434	1.17	POS	hilic				4.86E-02	1.57E-01	-0.45	9.72E-03	-0.65	7.38E-04	-0.81	9.95E-01	0.00
2105.244@1.274936	2105.2440	1.27	POS	hilic				9.84E-01	8.44E-01	-0.05	5.75E-01	-0.13	5.43E-01	-0.13	9.48E-01	-0.02
2106.2434@9.985746	2106.2434	9.99	POS	RP				8.18E-03	7.46E-02	0.50	1.70E-01	-0.40	2.64E-02	-0.53	9.49E-02	0.50
2107.0103@0.49483487	2107.0103	0.49	POS	hilic				9.11E-01	4.37E-01	-0.25	6.96E-01	-0.11	3.60E-01	-0.27	3.41E-01	-0.27
2107.082@3.1781366	2107.0820	3.18	NEG	hilic				6.36E-01	4.68E-01	-0.17	9.98E-01	0.00	3.13E-01	0.30	5.72E-01	-0.13
2107.244@1.2453622	2107.2440	1.25	POS	hilic				7.72E-01	6.95E-01	-0.10	1.76E-01	-0.37	2.40E-01	-0.31	4.47E-01	-0.22
2107.245@9.985756	2107.2450	9.99	POS	RP				1.01E-02	7.71E-02	0.49	1.90E-01	-0.38	3.16E-02	-0.52	9.51E-02	0.50
2107.3606@10.238194	2107.3606	10.24	POS	RP				8.34E-03	3.31E-02	0.64	1.23E-01	-0.48	1.53E-02	-0.59	9.68E-01	0.01
2108.3623@10.238757	2108.3623	10.24	POS	RP				9.28E-03	3.13E-02	0.65	1.18E-01	-0.48	2.22E-02	-0.57	9.34E-01	-0.02
2109.083@3.2044632	2109.0830	3.20	NEG	hilic				8.23E-01	3.93E-01	-0.20	7.61E-01	-0.08	5.22E-01	0.18	6.89E-01	-0.10
2109.254@9.985311	2109.2540	9.99	POS	RP				1.28E-02	6.54E-02	0.48	8.80E-02	-0.43	3.75E-02	-0.48	2.52E-01	0.38

Supplementary table 2: Metabolomics analysis results

Compound	Mass	Retention Time	Ionization mode	column	Compound_ID	MSMS_spectra	ID_level	ANOVA	Anorexia vs control		overweight vs control		obese vs control		athletes vs control	
								P-FDR	P	Estimate	P	Estimate	P	Estimate	P	Estimate
2111.021@0.5213731	2111.0210	0.52	POS	hilic				9.41E-01	6.03E-01	-0.14	9.08E-01	-0.03	4.61E-01	-0.17	7.61E-01	0.09
2112.2954@10.224264	2112.2954	10.22	POS	RP				1.80E-03	3.51E-03	0.87	1.97E-01	-0.35	1.12E-01	-0.40	1.15E-01	0.45
2113.0305@0.5684047	2113.0305	0.57	POS	hilic				7.19E-01	4.99E-01	0.22	3.41E-01	0.25	5.42E-01	-0.17	3.93E-01	0.25
2115.0508@0.4881042	2115.0508	0.49	POS	hilic				9.42E-01	6.86E-01	-0.13	8.88E-01	-0.04	6.96E-01	0.12	5.82E-01	-0.18
2115.2595@9.951575	2115.2595	9.95	POS	RP				1.65E-01	1.87E-01	-0.34	4.58E-01	-0.19	1.02E-03	-0.70	9.82E-01	-0.01
2115.2954@10.224617	2115.2954	10.22	POS	RP				5.89E-02	9.02E-02	0.53	1.25E-01	-0.35	8.86E-02	-0.37	6.32E-01	0.14
2115.4204@10.514824	2115.4204	10.51	POS	RP				2.49E-02	6.97E-02	0.56	3.94E-02	-0.61	2.11E-01	-0.32	9.41E-01	-0.02
2115.993@6.1820016	2115.9930	6.18	POS	hilic				7.42E-01	6.20E-01	0.14	3.35E-01	-0.22	3.75E-01	-0.20	7.75E-01	0.07
2116.427@10.514659	2116.4270	10.51	POS	RP				2.32E-02	7.87E-02	0.55	3.29E-02	-0.62	1.79E-01	-0.34	8.36E-01	-0.06
2117.3098@9.838779	2117.3098	9.84	NEG	RP				8.69E-01	2.61E-01	0.25	5.12E-01	0.14	9.77E-01	0.00	5.94E-01	0.10
2121.3328@10.23221	2121.3328	10.23	POS	RP				6.35E-03	1.50E-01	0.44	2.18E-02	-0.59	1.47E-02	-0.53	2.85E-02	-0.57
2123.026@0.5845223	2123.0260	0.58	POS	hilic				8.93E-01	4.53E-01	-0.23	9.73E-01	-0.01	7.27E-01	0.09	6.80E-01	0.13
2123.3325@10.23225	2123.3325	10.23	POS	RP				2.97E-01	5.00E-01	0.20	6.99E-01	-0.09	2.27E-01	-0.27	4.85E-02	-0.44
2124.333@10.233966	2124.3330	10.23	POS	RP				9.48E-01	9.35E-01	0.02	4.56E-01	-0.18	5.71E-01	-0.14	5.87E-01	-0.15
2125.036@0.58331734	2125.0360	0.58	POS	hilic				9.94E-01	8.70E-01	-0.05	8.15E-01	0.06	8.04E-01	-0.07	9.98E-01	0.00
2126.0432@0.5834647	2126.0432	0.58	POS	hilic				4.39E-01	8.41E-02	-0.51	4.84E-02	-0.52	1.58E-01	-0.39	5.06E-01	-0.19
2127.0464@0.58675754	2127.0464	0.59	POS	hilic				4.03E-01	5.50E-01	0.17	4.07E-01	-0.21	4.37E-01	-0.20	2.19E-01	0.36
2130.246@9.956094	2130.2460	9.96	POS	RP				3.50E-02	5.59E-01	0.16	1.02E-01	-0.49	6.88E-03	-0.66	3.15E-01	0.31
2131.3965@10.515414	2131.3965	10.52	POS	RP				1.07E-01	2.66E-01	0.37	4.17E-02	-0.59	1.83E-01	-0.33	7.69E-01	-0.08
2131.632@4.5562577	2131.6320	4.56	NEG	hilic				4.19E-01	1.16E-01	0.45	2.69E-01	0.33	5.24E-01	-0.19	7.09E-01	-0.10
2131.6333@4.5572352	2131.6333	4.56	NEG	hilic				2.76E-01	6.20E-03	0.69	4.85E-01	0.22	7.76E-01	0.08	6.25E-01	-0.14
2132.2495@9.9559555	2132.2495	9.96	POS	RP				3.97E-02	5.33E-01	0.18	1.05E-01	-0.48	7.76E-03	-0.66	3.32E-01	0.29
2132.6328@4.5637097	2132.6328	4.56	NEG	hilic				6.36E-01	7.50E-01	-0.09	5.37E-01	0.18	1.38E-01	0.45	8.40E-01	-0.06
2133.256@9.953651	2133.2560	9.95	POS	RP				2.09E-01	6.48E-01	0.13	1.90E-01	-0.40	2.48E-02	-0.56	6.20E-01	0.16
2135.0322@0.48717916	2135.0322	0.49	POS	hilic				7.61E-01	2.45E-01	-0.33	1.03E-01	-0.45	3.12E-01	-0.27	4.39E-01	-0.25
2135.235@9.951721	2135.2350	9.95	POS	RP				3.84E-01	7.16E-01	-0.10	6.24E-01	-0.13	5.49E-02	-0.43	3.39E-01	0.29
2138.303@10.233331	2138.3030	10.23	POS	RP				2.95E-03	2.85E-02	0.66	9.49E-02	-0.47	1.40E-02	-0.64	6.86E-01	0.11
2139.056@0.48742113	2139.0560	0.49	POS	hilic				8.81E-01	7.21E-01	-0.09	4.78E-01	0.19	6.10E-01	-0.14	7.99E-01	0.07
2141.192@10.1539545	2141.1920	10.15	POS	RP				5.18E-01	1.70E-01	0.34	2.39E-01	0.32	6.08E-01	-0.11	1.96E-01	0.32
2142.3186@10.231119	2142.3186	10.23	POS	RP				5.08E-03	4.52E-02	0.58	8.95E-02	-0.44	1.67E-02	-0.58	8.28E-01	0.06
2143.1975@10.152774	2143.1975	10.15	POS	RP				4.52E-01	7.33E-01	-0.10	8.89E-02	0.49	4.91E-01	0.16	1.73E-01	0.39
2143.3135@10.230657	2143.3135	10.23	POS	RP				9.82E-03	6.73E-02	0.51	7.88E-02	-0.43	1.33E-02	-0.57	6.18E-01	-0.13
2144.348@1.2149134	2144.3480	1.21	POS	hilic				1.61E-01	9.79E-01	0.01	1.05E-01	-0.32	5.01E-02	-0.38	2.34E-01	0.31
2144.371@10.512296	2144.3710	10.51	POS	RP				4.46E-01	3.97E-01	0.28	1.08E-01	-0.37	3.84E-01	-0.21	8.95E-01	-0.04
2146.3684@10.517336	2146.3684	10.52	POS	RP				5.67E-02	7.86E-02	0.55	4.86E-02	-0.56	5.37E-01	-0.15	9.19E-01	0.03
2147.3716@10.516691	2147.3716	10.52	POS	RP				6.67E-02	7.87E-02	0.56	5.95E-02	-0.53	5.52E-01	-0.15	9.39E-01	0.02
2147.6333@0.90062857	2147.6333	0.90	POS	hilic				1.47E-01	1.04E-01	-0.43	4.68E-02	0.49	4.05E-01	0.24	6.00E-01	0.15
2149.343@1.2120159	2149.3430	1.21	POS	hilic				7.58E-01	8.81E-01	-0.04	3.10E-01	-0.25	5.01E-01	0.18	5.57E-01	-0.16
2149.6494@0.898842	2149.6494	0.90	POS	hilic				5.29E-01	5.09E-01	-0.16	9.49E-02	0.41	3.85E-01	0.24	5.22E-01	0.18
2151.3816@10.51294	2151.3816	10.51	POS	RP				1.06E-01	1.74E-01	0.43	5.20E-02	-0.50	3.00E-01	-0.25	9.68E-01	-0.01
2151.5686@4.410639	2151.5686	4.41	POS	hilic				4.82E-01	8.81E-02	0.51	5.10E-02	0.55	6.33E-01	0.12	3.29E-01	0.30
2152.2378@9.9518585	2152.2378	9.95	POS	RP				6.63E-01	8.02E-01	-0.08	8.43E-01	-0.06	3.46E-01	-0.24	2.66E-01	0.35
2153.045@0.5535258	2153.0450	0.55	POS	hilic				7.31E-01	8.43E-01	0.05	1.28E-01	0.43	3.33E-01	0.29	3.87E-01	0.30
2154.2412@9.951604	2154.2412	9.95	POS	RP				5.75E-01	9.56E-01	0.01	6.51E-01	-0.15	1.89E-01	-0.34	3.10E-01	0.31
2155.0625@0.5483899	2155.0625	0.55	POS	hilic				1.92E-01	4.07E-01	0.26	2.12E-02	0.70	9.47E-01	0.02	6.28E-02	0.53
2155.2444@9.950887	2155.2444	9.95	POS	RP				9.40E-01	7.57E-01	-0.09	8.49E-01	-0.06	2.19E-01	-0.30	9.15E-01	-0.03
2156.445@0.3763909	2156.4450	0.38	NEG	hilic				8.93E-01	6.45E-01	0.14	9.52E-01	0.02	3.40E-01	-0.22	7.02E-01	0.13
2157.255@9.951537	2157.2550	9.95	POS	RP				4.06E-01	4.97E-01	-0.18	3.61E-01	-0.28	4.14E-02	-0.51	5.43E-01	0.18
2158.2478@9.949776	2158.2478	9.95	POS	RP				2.00E-01	7.04E-01	-0.10	7.36E-01	-0.09	2.82E-03	-0.62	6.92E-01	0.11
2159.275@1.2182046	2159.2750	1.22	POS	hilic				1.49E-01	2.19E-01	-0.36	3.05E-02	-0.49	8.53E-04	-0.71	7.18E-02	-0.43
2159.9558@6.184633	2159.9558	6.18	POS	hilic				6.89E-01	9.65E-01	-0.01	3.26E-01	-0.26	5.18E-01	0.17	4.33E-01	0.23
2161.302@1.2141839	2161.3020	1.21	POS	hilic				8.04E-01	6.50E-01	0.11	2.64E-01	0.26	2.25E-01	0.33	7.05E-01	0.09
2162.303@1.2150965	2162.3030	1.22	POS	hilic				3.96E-01	2.47E-01	0.36	3.49E-01	-0.24	2.52E-01	-0.29	6.92E-01	0.11
2163.317@1.2085112	2163.3170	1.21	POS	hilic				1.35E-01	2.67E-02	-0.59	6.52E-02	-0.52	8.30E-01	-0.07	8.59E-03	-0.69
2164.322@1.2154465	2164.3220	1.22	POS	hilic				6.47E-02	6.21E-02	0.57	5.34E-01	-0.15	2.25E-01	-0.26	1.50E-01	-0.31
2165.0542@0.54509264	2165.0542	0.55	POS	hilic				8.45E-01	7.29E-01	0.11	7.36E-01	-0.09	3.11E-01	-0.29	7.80E-01	0.08
2165.327@1.1975528	2165.3270	1.20	POS	hilic				4.43E-01	2.58E-02	-0.71	3.20E-01	-0.27	2.50E-01	-0.32	4.05E-01	-0.24
2167.0493@0.53897196	2167.0493	0.54	POS	hilic				9.35E-01	5.63E-01	-0.20	6.53E-01	0.13	9.42E-01	-0.02	7.97E-01	0.07
2168.059@0.547901	2168.0590	0.55	POS	hilic				3.07E-01	9.97E-01	0.00	2.65E-02	-0.55	5.75E-02	-0.57	5.78E-01	-0.16
2169.0703@0.548802	2169.0703	0.55	POS	hilic				6.90E-01	1.95E-01	-0.37	8.10E-01	0.07	3.27E-01	-0.26	8.30E-01	0.06
2171.0608@0.5281875	2171.0608	0.53	POS	hilic				8.94E-01	7.59E-01	0.09	9.44E-01	-0.02	4.54E-01	-0.22	6.98E-01	0.11
2174.9363@3.0355415	2174.9363	3.04	POS	hilic				6.45E-01	1.96E-01	0.42	3.21E-01	0.26	1.39E-01	0.50	6.73E-01	0.12
2176.9414@3.0430958	2176.9414	3.04	POS	hilic				8.71E-01	2.82E-01	0.36	5.96E-01	0.15	8.71E-01	0.05	9.70E-01	-0.01
2177.141@10.156943	2177.1410	10.16	POS	RP				7.31E-01	1.98E-01	0.42	2.50E-01	0.28	4.31E-01	0.18	9.79E-01	0.01
2178.2349@9.943334	2178.2349	9.94	POS	RP				2.71E-01	1.52E-01	-0.26	7.35E-01	0.08	8.57E-02	0.42	9.14E-01	-0.02
2183.074@0.59273475	2183.0740	0.59	POS	hilic				5.55E-01	8.42E-01	-0.05	6.79E-01	-0.10	6.63E-01	0.10	1.16E-01	0.40
2191.0403@3.2128088	2191.0403	3.21	NEG	hilic				6.69E-01	3.32E-01	-0.28	5.97E-01	0.14	5.04E-01	0.18	3.31E-01	0.25
2191.0442@3.1618478	2191.0442	3.16	NEG	hilic				6.31E-01	4.24E-02	0.56	6.94E-01	0.10	7.18E-01	0.10	8.50E-01	0.06
2193.0432@3.182342	2193.0432	3.18	NEG	hilic				2.36E-01	8.53E-01	-0.06	1.04E-01	0.45	2.82E-01	0.29	1.07E-02	0.70
2193.0437@3.2090352	2193.0437	3.21	NEG	hilic				5.53E-01	7.18E-01	-0.11	1.87E-01	0.34	3.42E-01	0.24	1.05E-01	0.47
2195.0415@3.19381	2195.0415	3.19	NEG	hilic				7.78E-01	5.45E-01	0.22	5.53E-01	-0.15	2.81E-01	-0.27	8.54E-01	0.05
2195.0525@0.5117386	2195.0525	0.51	POS	hilic				5.72E-01	9.39E-01	-0.02	6.72E-01	0.12	4.39E-01	0.21	6.34E-02	0.55
2197.0398@3.2190444	2197.0398	3.22	NEG	hilic				7.65E-01	2.08E-01	0.37	6.54E-01	-0.13	7.82E-01	0.07	8.89E-01	-0.04

Supplementary table 2: Metabolomics analysis results

								ANOVA	Anorexia vs control		overweight vs control		obese vs control		athletes vs control	
Compound	Mass	Retention Time	Ionization mode	column	Compound_ID	MSMS_spectra	ID_level	P-FDR	P	Estimate	P	Estimate	P	Estimate	P	Estimate
2199.0818@0.5143192	2199.0818	0.51	POS	hilic				6.75E-01	1.67E-01	-0.29	9.42E-01	0.02	1.30E-01	-0.33	9.99E-01	0.00
2199.619@4.561947	2199.6190	4.56	NEG	hilic				6.43E-01	3.89E-01	0.26	8.81E-01	0.05	7.41E-01	0.08	5.81E-02	0.52
2199.6216@4.556484	2199.6216	4.56	NEG	hilic				7.46E-01	4.56E-01	0.22	5.51E-01	-0.16	4.99E-01	-0.18	5.14E-01	0.18
2209.3015@1.1915519	2209.3015	1.19	POS	hilic				4.08E-01	3.39E-01	-0.24	2.05E-02	-0.57	7.99E-01	-0.07	4.18E-01	-0.23
2217.3435@9.852199	2217.3435	9.85	NEG	RP				2.31E-01	4.55E-02	-0.52	1.14E-01	-0.41	5.15E-03	-0.68	5.35E-01	-0.19
2221.4585@0.3746972	2221.4585	0.37	NEG	hilic				7.75E-01	4.33E-01	0.24	6.17E-01	-0.16	6.83E-01	0.11	3.12E-01	0.28
2235.1538@9.956446	2235.1538	9.96	POS	RP				4.60E-01	2.92E-02	0.59	1.12E-01	0.43	4.52E-01	0.17	1.77E-01	0.37
2237.1655@9.9623995	2237.1655	9.96	POS	RP				1.57E-01	5.26E-03	0.78	5.43E-01	0.18	7.16E-01	0.08	4.16E-02	0.56
2241.075@0.59171754	2241.0750	0.59	POS	hilic				9.53E-01	6.35E-01	-0.13	9.50E-01	-0.02	4.15E-01	-0.23	9.43E-01	-0.02
2247.2307@10.237324	2247.2307	10.24	POS	RP				9.39E-03	6.95E-03	0.90	4.06E-01	-0.21	4.08E-01	-0.18	1.33E-01	0.44
2253.0862@0.59499985	2253.0862	0.59	POS	hilic				9.26E-01	2.86E-01	0.30	7.23E-01	0.09	3.72E-01	0.21	6.27E-01	0.16
2255.0906@0.5946131	2255.0906	0.59	POS	hilic				4.78E-01	4.36E-01	-0.22	2.96E-01	-0.31	3.89E-01	-0.27	3.36E-01	0.29
2255.3103@10.515336	2255.3103	10.52	POS	RP				3.40E-01	9.63E-01	-0.02	3.14E-02	-0.52	6.20E-01	-0.12	5.07E-01	0.18
2259.1714@10.255259	2259.1714	10.26	POS	RP				5.93E-01	5.03E-01	-0.19	1.05E-01	-0.34	5.55E-01	0.13	5.78E-01	-0.14
2265.3071@10.520541	2265.3071	10.52	POS	RP				2.14E-01	1.61E-01	0.45	3.18E-01	-0.27	6.23E-01	-0.13	2.29E-01	0.37
2267.2512@10.532277	2267.2512	10.53	POS	RP				2.83E-01	1.13E-01	0.50	2.89E-01	-0.29	6.90E-01	0.11	3.70E-01	0.29
2267.6067@4.5577655	2267.6067	4.56	NEG	hilic				6.62E-02	7.35E-04	0.95	5.90E-03	0.75	1.12E-01	0.39	4.33E-02	0.67
2267.6096@4.5569024	2267.6096	4.56	NEG	hilic				2.07E-01	1.08E-02	0.76	7.75E-02	0.48	4.25E-01	0.20	3.55E-02	0.64
2275.0027@3.1529765	2275.0027	3.15	NEG	hilic				5.96E-01	9.89E-01	0.00	1.54E-01	-0.30	1.30E-01	-0.32	7.79E-01	0.07
2275.004@3.2410908	2275.0040	3.24	NEG	hilic				6.96E-01	1.52E-01	0.44	7.78E-01	0.07	8.87E-01	-0.04	5.24E-01	0.18
2277.0088@3.2154293	2277.0088	3.22	NEG	hilic				2.27E-01	2.43E-02	0.72	2.15E-01	0.32	2.83E-01	0.32	9.47E-03	0.70
2279.0012@3.2213001	2279.0012	3.22	NEG	hilic				4.28E-01	9.93E-02	0.53	9.21E-01	0.02	2.07E-01	0.35	9.42E-02	0.50
2279.2112@10.240833	2279.2112	10.24	POS	RP				5.96E-01	1.04E-01	-0.41	8.98E-01	0.03	7.89E-01	-0.06	7.14E-01	0.10
2281.181@10.234673	2281.1810	10.23	POS	RP				7.07E-03	2.17E-02	0.69	2.76E-01	-0.21	4.56E-02	-0.36	8.21E-01	-0.05
2283.109@0.5091758	2283.1090	0.51	POS	hilic				5.39E-01	9.22E-01	0.03	5.71E-01	0.16	8.07E-02	-0.43	7.43E-01	-0.09
2284.4658@0.37548265	2284.4658	0.38	NEG	hilic				9.65E-01	7.21E-01	0.11	9.17E-01	-0.03	5.48E-01	-0.18	9.43E-01	-0.02
2287.545@4.4124618	2287.5450	4.41	POS	hilic				8.05E-01	3.37E-01	-0.23	6.30E-01	-0.14	6.09E-01	-0.15	1.18E-01	-0.42
2289.269@10.516899	2289.2690	10.52	POS	RP				1.27E-01	1.64E-01	0.45	6.34E-02	-0.53	4.00E-01	-0.23	8.95E-01	-0.04
2299.14@0.57595056	2299.1400	0.58	POS	hilic				7.75E-01	9.23E-01	0.03	3.51E-01	-0.22	3.00E-01	-0.26	6.56E-01	0.13
2324.618@12.242109	2324.6180	12.24	POS	RP				3.97E-01	4.70E-01	-0.21	7.54E-03	-0.60	5.02E-01	-0.17	6.90E-01	-0.11
2326.6062@12.246723	2326.6062	12.25	POS	RP				2.18E-01	8.51E-01	-0.05	9.81E-02	-0.44	1.17E-01	-0.43	3.28E-01	0.31
2327.6074@12.24359	2327.6074	12.24	POS	RP				6.53E-01	6.21E-01	0.16	5.38E-01	-0.17	7.34E-01	-0.09	2.07E-01	0.37
2335.5947@4.5595555	2335.5947	4.56	NEG	hilic				4.08E-01	2.10E-01	0.28	9.45E-02	0.53	5.26E-01	-0.18	5.60E-01	0.18
2335.5952@4.559038	2335.5952	4.56	NEG	hilic				6.32E-01	3.75E-01	0.22	1.21E-01	0.48	8.45E-01	-0.05	6.78E-01	0.13
2342.8545@3.0412686	2342.8545	3.04	POS	hilic				2.37E-01	5.82E-01	0.19	7.50E-01	0.09	1.02E-01	-0.44	9.89E-02	0.42
2344.858@3.0453424	2344.8580	3.05	POS	hilic				7.47E-01	7.95E-01	-0.08	7.65E-01	0.09	8.49E-01	-0.06	7.75E-02	0.40
2346.4717@0.3728878	2346.4717	0.37	NEG	hilic				9.74E-01	8.14E-01	0.07	6.81E-01	-0.12	6.45E-01	-0.15	9.38E-01	-0.02
2346.86@3.0463157	2346.8600	3.05	POS	hilic				8.64E-01	2.64E-01	0.35	3.38E-01	0.24	9.48E-01	0.02	6.57E-01	0.14
2349.4849@0.408	2349.4849	0.41	NEG	hilic				4.62E-01	6.50E-01	0.11	2.51E-02	-0.31	9.01E-01	-0.03	3.73E-01	-0.15
2351.5425@0.40754342	2351.5425	0.41	NEG	hilic				9.95E-01	8.03E-01	-0.07	7.28E-01	-0.08	8.96E-01	0.03	8.90E-01	-0.03
2355.5737@0.40796545	2355.5737	0.41	NEG	hilic				8.38E-01	9.38E-01	0.02	4.15E-01	0.15	7.97E-01	0.05	2.47E-01	0.26
2358.9648@3.2161016	2358.9648	3.22	NEG	hilic				8.31E-01	8.97E-01	0.04	1.79E-01	0.37	8.21E-01	0.06	7.17E-01	0.10
2358.965@3.1766336	2358.9650	3.18	NEG	hilic				8.25E-01	3.82E-01	0.24	7.46E-01	-0.10	5.66E-01	-0.19	5.67E-01	-0.17
2360.963@3.2051888	2360.9630	3.21	NEG	hilic				9.35E-01	6.27E-01	-0.13	4.05E-01	-0.23	8.11E-01	0.08	8.89E-01	-0.04
2362.962@3.2030716	2362.9620	3.20	NEG	hilic				6.38E-01	7.57E-01	-0.09	8.50E-01	-0.05	1.80E-01	0.44	4.98E-01	0.20
2364.9624@3.2096717	2364.9624	3.21	NEG	hilic				7.92E-01	7.36E-01	0.10	3.50E-01	-0.29	8.75E-01	-0.04	5.89E-01	0.16
2374.607@12.162015	2374.6070	12.16	POS	RP				3.61E-01	8.30E-01	0.07	1.15E-01	-0.34	2.29E-02	-0.50	8.14E-01	-0.06
2385.1433@0.59818363	2385.1433	0.60	POS	hilic				8.41E-01	5.26E-01	-0.15	4.76E-01	-0.18	1.85E-01	-0.33	8.56E-01	-0.04
2395.146@0.4972422	2395.1460	0.50	POS	hilic				9.39E-01	5.17E-01	-0.19	6.27E-01	-0.14	7.86E-01	0.07	8.60E-01	0.05
2396.6057@12.143968	2396.6057	12.14	POS	RP				8.42E-01	2.02E-01	-0.34	8.68E-01	-0.05	9.47E-01	0.02	8.05E-01	0.05
2398.6013@12.142676	2398.6013	12.14	POS	RP				8.96E-01	5.26E-01	0.16	7.90E-01	0.08	2.99E-01	0.31	9.82E-01	-0.01
2399.609@12.14378	2399.6090	12.14	POS	RP				4.68E-03	2.31E-03	-0.79	8.42E-01	0.06	4.92E-02	0.57	3.03E-01	-0.24
2403.5818@4.553225	2403.5818	4.55	NEG	hilic				8.41E-01	2.89E-01	0.34	7.14E-01	0.11	7.09E-01	-0.10	9.93E-01	0.00
2403.5837@4.553225	2403.5837	4.55	NEG	hilic				8.34E-01	3.27E-01	0.31	7.89E-01	0.08	5.80E-01	-0.14	9.14E-01	-0.03
2404.5852@4.554112	2404.5852	4.55	NEG	hilic				1.76E-01	1.35E-03	0.92	2.11E-01	0.35	2.56E-01	0.29	4.30E-01	0.25
2412.4917@0.374688	2412.4917	0.37	NEG	hilic				8.02E-01	3.58E-01	0.31	3.06E-01	0.31	3.09E-01	0.27	1.57E-01	0.42
2413.17@0.57611424	2413.1700	0.58	POS	hilic				7.26E-01	3.71E-01	0.26	8.85E-02	0.46	2.68E-01	0.26	3.22E-01	0.24
2423.5215@4.408597	2423.5215	4.41	POS	hilic				6.72E-01	6.45E-01	0.13	3.70E-01	0.22	6.96E-01	-0.12	2.83E-01	-0.28
2424.4905@0.39094228	2424.4905	0.39	NEG	hilic				7.08E-01	9.45E-02	-0.39	1.87E-01	-0.32	7.13E-01	-0.10	6.01E-01	-0.14
2427.1726@0.5734756	2427.1726	0.57	POS	hilic				4.17E-01	7.79E-01	-0.08	4.40E-01	-0.23	4.82E-02	-0.55	7.22E-01	0.10
2427.525@11.829982	2427.5250	11.83	NEG	RP				7.14E-01	5.65E-02	-0.45	4.30E-01	-0.22	7.59E-01	-0.08	5.09E-01	-0.17
2442.9265@3.147785	2442.9265	3.15	NEG	hilic				3.47E-01	9.85E-01	0.00	8.43E-01	-0.04	1.04E-01	0.45	1.30E-01	0.38
2442.9287@3.2459722	2442.9287	3.25	NEG	hilic				6.96E-01	4.98E-01	0.21	8.32E-01	0.05	4.26E-01	-0.22	3.08E-01	-0.25
2444.9287@3.215197	2444.9287	3.22	NEG	hilic				7.31E-01	4.56E-01	0.26	2.16E-01	0.35	9.45E-02	0.50	3.93E-01	0.28
2446.9243@3.2070997	2446.9243	3.21	NEG	hilic				9.42E-01	5.30E-01	0.23	9.81E-01	0.01	4.99E-01	0.21	4.91E-01	0.18
2452.5117@0.3862942	2452.5117	0.39	NEG	hilic				8.46E-01	8.41E-01	-0.05	3.53E-01	-0.20	5.32E-01	-0.14	2.12E-01	-0.28
2458.5044@0.38911632	2458.5044	0.39	NEG	hilic				8.27E-01	5.14E-01	-0.15	7.42E-01	-0.08	7.54E-01	0.08	2.05E-01	-0.26
2468.593@12.077207	2468.5930	12.08	POS	RP				1.62E-01	2.78E-03	-0.72	1.71E-01	-0.37	5.13E-02	-0.51	8.79E-02	-0.42
2470.5913@12.074579	2470.5913	12.07	POS	RP				1.78E-01	1.53E-03	-0.78	3.93E-01	-0.23	3.72E-01	-0.25	8.05E-02	-0.44
2471.5703@4.5574484	2471.5703	4.56	NEG	hilic				8.75E-01	3.24E-01	0.33	8.68E-01	0.05	9.73E-01	0.01	7.68E-01	-0.08
2471.5713@4.5557804	2471.5713	4.56	NEG	hilic				4.66E-01	1.31E-01	0.44	9.77E-01	0.01	2.69E-01	0.30	4.63E-01	-0.22
2474.484@0.36991256	2474.4840	0.37	NEG	hilic				6.21E-01	5.09E-01	0.19	8.77E-01	-0.05	1.71E-01	-0.42	5.79E-01	-0.15
2477.506@0.36991823	2477.5060	0.37	NEG	hilic				7.11E-01	5.69E-01	0.18	3.68E-01	-0.28	3.02E-01	-0.31	7.38E-01	-0.09

Supplementary table 2: Metabolomics analysis results

Compound	Mass	Retention Time	Ionization mode	column	Compound_ID	MSMS_spectra	ID_level	ANOVA	Anorexia vs control		overweight vs control		obese vs control		athletes vs control	
								P-FDR	P	Estimate	P	Estimate	P	Estimate	P	Estimate
2510.7727@3.039165	2510.7727	3.04	POS	hilic				2.17E-01	8.61E-03	0.78	2.14E-02	0.52	1.09E-01	0.46	4.14E-02	0.59
2512.7793@3.0413308	2512.7793	3.04	POS	hilic				1.50E-01	4.08E-02	0.71	6.20E-02	0.51	2.06E-02	0.68	7.17E-03	0.80
2514.611@10.10359	2514.6110	10.10	POS	RP				7.44E-01	2.56E-01	0.31	8.23E-01	-0.07	6.00E-01	-0.17	4.91E-01	0.20
2514.7866@3.0486727	2514.7866	3.05	POS	hilic				5.10E-01	2.64E-01	0.38	1.83E-01	0.35	9.83E-01	0.01	9.07E-02	0.54
2515.2173@0.59455556	2515.2173	0.59	POS	hilic				3.07E-01	1.07E-02	-0.65	3.81E-01	-0.23	7.31E-01	-0.10	1.70E-01	-0.35
2518.532@0.3891145	2518.5320	0.39	NEG	hilic				4.26E-01	1.90E-01	0.43	3.57E-01	0.25	4.62E-01	-0.19	2.28E-01	0.37
2518.6057@1.3192451	2518.6057	1.32	POS	hilic				1.19E-01	3.00E-01	-0.31	4.30E-01	0.21	3.73E-02	0.59	3.61E-01	-0.26
2519.6096@1.3176162	2519.6096	1.32	POS	hilic				1.62E-01	1.42E-01	-0.43	3.27E-01	0.27	1.08E-01	0.45	5.48E-01	-0.16
2520.6255@1.3163859	2520.6255	1.32	POS	hilic				3.90E-02	2.48E-02	-0.65	4.15E-01	0.19	1.23E-01	0.46	3.55E-01	-0.26
2525.641@10.106556	2525.6410	10.11	POS	RP				6.35E-02	7.26E-01	0.08	9.07E-01	0.02	6.10E-02	0.38	6.49E-03	0.77
2526.8875@3.1888046	2526.8875	3.19	NEG	hilic				8.25E-01	9.42E-01	-0.02	8.29E-01	0.06	9.79E-01	-0.01	1.98E-01	0.36
2526.8882@3.2056913	2526.8882	3.21	NEG	hilic				5.24E-01	9.81E-01	0.01	6.99E-01	-0.11	8.93E-01	0.04	9.88E-02	0.49
2527.5784@1.3109248	2527.5784	1.31	POS	hilic				3.91E-01	2.04E-01	0.42	2.29E-01	0.27	1.75E-02	0.68	1.28E-01	0.40
2527.609@10.10575	2527.6090	10.11	POS	RP				4.60E-01	4.07E-01	0.26	8.49E-01	-0.04	5.62E-01	0.16	5.16E-02	0.57
2528.5798@1.3108494	2528.5798	1.31	POS	hilic				6.29E-01	3.87E-01	0.30	4.80E-01	0.17	8.71E-02	0.52	1.15E-01	0.42
2528.8877@3.209351	2528.8877	3.21	NEG	hilic				9.95E-01	9.91E-01	0.00	6.74E-01	0.12	9.80E-01	0.01	9.98E-01	0.00
2529.5696@1.3084745	2529.5696	1.31	POS	hilic				5.56E-01	1.12E-01	0.57	5.08E-01	0.16	2.04E-01	0.38	1.03E-01	0.44
2529.578@10.104915	2529.5780	10.10	POS	RP				1.69E-02	1.02E-02	0.76	9.59E-01	-0.01	5.62E-01	0.17	8.92E-04	0.93
2530.5786@10.106232	2530.5786	10.11	POS	RP				1.26E-02	5.60E-03	0.80	7.95E-01	-0.07	9.39E-01	0.02	2.42E-03	0.84
2530.8877@3.210455	2530.8877	3.21	NEG	hilic				8.27E-01	5.97E-01	-0.16	2.12E-01	-0.33	5.46E-01	-0.19	8.16E-01	0.07
2532.5803@10.106854	2532.5803	10.11	POS	RP				6.93E-03	2.10E-02	0.69	3.48E-01	-0.25	4.38E-01	0.22	9.43E-04	0.91
2532.8828@3.2098653	2532.8828	3.21	NEG	hilic				5.16E-01	4.37E-01	0.27	5.18E-01	-0.17	5.35E-01	0.18	9.84E-02	0.46
2533.5857@10.106574	2533.5857	10.11	POS	RP				8.33E-03	1.85E-02	0.74	2.48E-01	-0.28	6.17E-01	0.15	2.99E-03	0.80
2534.5847@10.107087	2534.5847	10.11	POS	RP				8.11E-03	1.53E-02	0.72	3.80E-01	-0.22	4.21E-01	0.23	1.23E-03	0.89
2535.5862@10.107189	2535.5862	10.11	POS	RP				1.04E-02	1.54E-02	0.71	3.02E-01	-0.25	2.70E-01	0.32	3.23E-03	0.84
2539.553@4.5532393	2539.5530	4.55	NEG	hilic				5.02E-01	4.16E-01	0.24	2.08E-02	0.64	5.09E-01	0.18	2.50E-01	0.36
2539.553@4.554227	2539.5530	4.55	NEG	hilic				5.45E-01	1.15E-01	0.43	3.91E-01	0.27	6.67E-01	-0.11	6.45E-01	-0.13
2540.5144@0.37422407	2540.5144	0.37	NEG	hilic				8.97E-01	2.56E-01	0.33	9.09E-01	0.04	4.45E-01	0.23	5.75E-01	0.17
2540.5542@4.5550103	2540.5542	4.56	NEG	hilic				6.13E-01	5.99E-01	-0.15	8.78E-01	0.05	5.36E-01	0.17	1.47E-01	-0.41
2542.5996@1.3138981	2542.5996	1.31	POS	hilic				6.47E-01	9.49E-02	-0.52	3.74E-01	-0.26	8.79E-01	-0.04	3.11E-01	-0.27
2543.2253@0.52253217	2543.2253	0.52	POS	hilic				2.97E-01	6.09E-02	-0.45	5.68E-01	-0.15	2.10E-02	-0.57	9.52E-01	-0.02
2543.6162@1.3136052	2543.6162	1.31	POS	hilic				8.76E-01	9.37E-01	-0.02	4.02E-01	0.19	8.30E-01	0.05	3.76E-01	0.23
2544.615@1.3091153	2544.6150	1.31	POS	hilic				3.20E-02	3.19E-03	-0.96	1.00E-01	-0.47	1.65E-01	-0.39	8.78E-04	-0.92
2551.5667@1.3037664	2551.5667	1.30	POS	hilic				8.94E-01	4.45E-01	0.28	4.50E-01	0.19	9.72E-01	0.01	3.23E-01	0.27
2552.5547@1.300478	2552.5547	1.30	POS	hilic				1.89E-01	1.50E-01	0.48	7.20E-01	-0.09	1.18E-01	-0.44	4.09E-01	0.21
2553.5696@1.295772	2553.5696	1.30	POS	hilic				1.56E-01	7.46E-01	-0.10	3.09E-01	-0.26	2.02E-03	-0.85	7.29E-01	-0.10
2559.4956@4.4123816	2559.4956	4.41	POS	hilic				8.42E-01	6.55E-01	-0.15	5.88E-01	-0.15	2.76E-01	-0.30	1.79E-01	-0.36
2577.568@1.286678	2577.5680	1.29	POS	hilic				1.74E-01	1.60E-01	-0.38	3.91E-01	-0.21	8.25E-04	-0.75	4.51E-01	-0.21
2602.52@0.36791304	2602.5200	0.37	NEG	hilic				5.41E-01	6.00E-01	0.14	2.89E-01	-0.30	1.63E-01	-0.39	8.54E-01	0.05
2605.5342@0.36761463	2605.5342	0.37	NEG	hilic				4.44E-01	9.57E-01	0.02	1.23E-01	-0.50	9.22E-02	-0.48	1.48E-01	-0.38
2607.5398@4.5535975	2607.5398	4.55	NEG	hilic				3.69E-01	2.53E-01	-0.34	1.60E-01	0.41	6.49E-01	0.12	4.03E-01	0.26
2607.5437@4.5534515	2607.5437	4.55	NEG	hilic				8.14E-01	6.78E-01	0.12	8.92E-01	-0.04	4.18E-01	-0.21	4.20E-01	0.22
2610.8474@3.188474	2610.8474	3.19	NEG	hilic				7.19E-01	2.18E-01	0.40	9.12E-01	-0.03	8.21E-01	-0.06	4.04E-01	0.25
2612.8518@3.2085762	2612.8518	3.21	NEG	hilic				5.84E-01	5.18E-01	0.21	2.52E-01	-0.32	8.48E-01	-0.06	3.62E-01	0.26
2614.8474@3.2181253	2614.8474	3.22	NEG	hilic				6.74E-01	4.61E-01	0.23	4.41E-01	-0.22	7.56E-01	-0.09	3.34E-01	0.27
2634.5952@9.956763	2634.5952	9.96	POS	RP				5.58E-02	4.07E-01	-0.22	6.47E-02	-0.48	3.04E-04	-0.83	6.55E-01	-0.13
2644.6792@10.23199	2644.6792	10.23	POS	RP				7.48E-03	8.40E-02	0.54	5.52E-02	-0.53	1.60E-02	-0.62	8.50E-01	-0.05
2646.553@0.38671124	2646.5530	0.39	NEG	hilic				9.41E-01	6.99E-01	0.12	6.77E-01	0.12	5.57E-01	-0.15	7.76E-01	0.08
2647.5774@9.961041	2647.5774	9.96	POS	RP				2.49E-02	8.64E-01	0.05	6.83E-02	-0.50	3.49E-03	-0.74	4.15E-01	0.25
2649.5713@9.958413	2649.5713	9.96	POS	RP				1.01E-02	5.78E-01	0.15	5.64E-02	-0.53	2.08E-03	-0.79	3.71E-01	0.28
2650.577@9.962195	2650.5770	9.96	POS	RP				3.74E-03	6.45E-01	0.13	2.88E-02	-0.61	8.43E-04	-0.87	3.88E-01	0.26
2659.6497@10.23199	2659.6497	10.23	POS	RP				1.07E-02	8.65E-02	0.53	7.14E-02	-0.50	1.61E-02	-0.60	9.27E-01	0.03
2660.657@10.23198	2660.6570	10.23	POS	RP				6.13E-03	6.68E-02	0.55	6.35E-02	-0.51	1.05E-02	-0.66	9.83E-01	0.01
2668.5337@0.37193096	2668.5337	0.37	NEG	hilic				6.69E-01	2.20E-01	0.35	4.21E-01	-0.26	8.51E-01	0.05	9.11E-01	-0.03
2669.7278@10.515658	2669.7278	10.52	POS	RP				1.49E-01	1.54E-01	0.46	7.70E-02	-0.46	5.03E-01	-0.17	9.23E-01	0.03
2670.735@10.514362	2670.7350	10.51	POS	RP				1.36E-01	1.96E-01	0.41	5.22E-02	-0.48	2.73E-01	-0.28	8.84E-01	-0.04
2675.5288@4.546825	2675.5288	4.55	NEG	hilic				4.90E-01	2.67E-01	0.34	3.66E-02	0.59	6.18E-01	0.15	9.33E-01	0.03
2675.5298@4.5446315	2675.5298	4.54	NEG	hilic				4.01E-01	1.14E-02	0.66	1.41E-01	0.42	5.46E-01	0.20	9.07E-01	0.03
2676.529@4.5513773	2676.5290	4.55	NEG	hilic				9.97E-01	8.87E-01	0.04	8.31E-01	-0.07	8.27E-01	-0.06	9.81E-01	-0.01
2678.7058@3.0425634	2678.7058	3.04	POS	hilic				4.48E-01	3.61E-01	-0.26	1.12E-01	0.47	5.66E-01	0.16	8.12E-01	0.06
2680.7075@3.0477276	2680.7075	3.05	POS	hilic				2.02E-01	7.04E-01	-0.11	8.72E-03	0.70	2.15E-01	0.37	3.02E-01	0.27
2694.8047@3.1954834	2694.8047	3.20	NEG	hilic				7.63E-01	2.27E-01	0.32	8.10E-01	-0.06	3.11E-01	0.26	4.17E-01	0.26
2694.8064@3.2131128	2694.8064	3.21	NEG	hilic				2.12E-01	1.59E-01	-0.37	6.82E-01	0.11	6.33E-01	-0.14	5.47E-02	0.53
2695.469@4.4081635	2695.4690	4.41	POS	hilic				1.38E-01	2.10E-01	0.41	6.58E-01	0.12	9.08E-02	-0.44	2.41E-01	-0.32
2696.8064@3.1967409	2696.8064	3.20	NEG	hilic				7.92E-01	7.35E-01	0.10	4.28E-01	0.19	8.98E-01	-0.04	2.43E-01	0.40
2696.809@3.2119904	2696.8090	3.21	NEG	hilic				4.22E-01	7.16E-01	-0.09	3.00E-01	0.27	9.41E-01	0.02	5.66E-02	0.58
2697.8137@3.1957822	2697.8137	3.20	NEG	hilic				7.43E-01	1.85E-01	0.41	1.41E-01	0.41	3.38E-01	0.28	6.20E-01	0.13
2698.8083@3.2049725	2698.8083	3.20	NEG	hilic				6.80E-01	8.03E-01	-0.07	7.81E-01	0.08	3.89E-01	0.26	6.39E-02	0.44
2700.807@3.2105274	2700.8070	3.21	NEG	hilic				9.16E-01	8.71E-01	0.05	6.15E-01	0.16	4.28E-01	0.23	2.64E-01	0.30
2702.8027@3.2161877	2702.8027	3.22	NEG	hilic				7.18E-01	1.52E-01	0.36	1.54E-01	0.42	1.60E-01	0.38	1.97E-01	0.36
2730.5386@0.3668455	2730.5386	0.37	NEG	hilic				7.65E-01	8.94E-01	0.04	2.07E-01	-0.39	3.92E-01	-0.26	5.65E-01	-0.16
2733.5537@0.36647272	2733.5537	0.37	NEG	hilic				5.95E-01	7.55E-01	-0.09	7.75E-02	-0.54	2.31E-01	-0.36	4.22E-01	-0.22
2743.515@4.5476956	2743.5150	4.55	NEG	hilic				8.21E-01	6.54E-01	-0.15	3.29E-01	0.30	5.96E-01	0.15	7.70E-01	0.09

Supplementary table 2: Metabolomics analysis results

								ANOVA	Anorexia vs control		overweight vs control		obese vs control		athletes vs control	
Compound	Mass	Retention Time	Ionization mode	column	Compound_ID	MSMS_spectra	ID_level	P-FDR	P	Estimate	P	Estimate	P	Estimate	P	Estimate
2743.5154@4.5492697	2743.5154	4.55	NEG	hilic				1.14E-01	3.92E-01	0.30	1.46E-02	0.60	1.99E-03	0.87	4.52E-01	0.19
2746.5625@0.38568878	2746.5625	0.39	NEG	hilic				5.16E-01	9.11E-01	-0.03	9.31E-01	0.02	6.87E-02	-0.44	4.79E-01	0.20
2774.581@0.38337627	2774.5810	0.38	NEG	hilic				9.18E-01	6.92E-01	0.13	8.32E-01	-0.06	3.55E-01	-0.23	9.72E-01	-0.01
2778.77@3.1947823	2778.7700	3.19	NEG	hilic				4.68E-01	2.89E-02	0.64	4.78E-01	0.21	1.49E-01	0.40	1.41E-01	0.47
2778.7725@3.2287633	2778.7725	3.23	NEG	hilic				8.98E-01	8.04E-01	0.08	7.33E-01	-0.08	7.90E-01	0.06	3.28E-01	-0.21
2780.7693@3.1982186	2780.7693	3.20	NEG	hilic				5.76E-01	1.70E-01	0.45	3.28E-01	0.27	3.50E-01	0.27	6.02E-02	0.58
2782.7693@3.2034001	2782.7693	3.20	NEG	hilic				6.69E-01	3.00E-01	0.31	6.53E-01	0.13	1.15E-01	0.47	1.78E-01	0.42
2796.557@0.3708065	2796.5570	0.37	NEG	hilic				8.36E-01	7.45E-01	0.09	9.27E-01	-0.03	6.32E-01	-0.13	2.77E-01	-0.31
2811.5037@4.5422406	2811.5037	4.54	NEG	hilic				8.12E-02	2.32E-02	0.73	3.70E-03	0.88	1.84E-01	0.30	2.79E-02	0.60
2811.5063@4.5425267	2811.5063	4.54	NEG	hilic				1.57E-01	1.67E-02	0.73	2.80E-02	0.68	5.62E-01	0.14	3.71E-02	0.54
2812.5005@4.5491343	2812.5005	4.55	NEG	hilic				9.92E-01	8.57E-01	0.05	8.18E-01	-0.05	6.87E-01	-0.10	9.28E-01	-0.02
2831.4478@4.407291	2831.4478	4.41	POS	hilic				4.91E-01	9.81E-01	-0.01	1.29E-01	-0.44	3.22E-01	-0.27	6.59E-01	0.13
2848.6255@3.052209	2848.6255	3.05	POS	hilic				6.34E-01	7.05E-01	-0.13	3.78E-01	-0.24	8.04E-02	-0.53	1.52E-01	-0.41
2861.5813@0.36618185	2861.5813	0.37	NEG	hilic				6.82E-01	9.62E-01	0.01	1.98E-01	-0.41	1.85E-01	-0.39	4.75E-01	-0.19
2862.725@3.1893365	2862.7250	3.19	NEG	hilic				7.11E-01	7.85E-01	0.09	6.39E-01	0.13	7.55E-02	0.47	2.77E-01	0.36
2862.7312@3.2067018	2862.7312	3.21	NEG	hilic				5.77E-01	1.11E-01	-0.46	7.68E-01	-0.07	5.16E-01	0.18	8.18E-01	0.08
2864.7317@3.2028794	2864.7317	3.20	NEG	hilic				5.93E-01	5.28E-01	0.22	2.04E-01	0.35	4.41E-02	0.54	1.30E-01	0.47
2864.7334@3.1933913	2864.7334	3.19	NEG	hilic				7.19E-01	3.78E-01	0.26	7.53E-01	-0.09	1.84E-01	0.37	4.31E-01	0.24
2866.729@3.207613	2866.7290	3.21	NEG	hilic				7.48E-01	5.01E-01	0.20	8.47E-01	0.05	1.43E-01	0.40	2.10E-01	0.37
2868.734@3.2052586	2868.7340	3.21	NEG	hilic				9.10E-01	2.88E-01	0.34	8.74E-01	0.04	6.20E-01	0.14	9.14E-01	0.03
2870.7224@3.2169409	2870.7224	3.22	NEG	hilic				5.96E-01	2.64E-01	0.32	5.02E-01	-0.15	2.68E-01	0.29	9.78E-01	0.01
2874.5923@0.38308328	2874.5923	0.38	NEG	hilic				4.55E-01	6.67E-02	0.51	8.99E-01	-0.03	1.79E-01	0.38	8.82E-01	0.04
2879.4895@4.5391884	2879.4895	4.54	NEG	hilic				9.87E-01	8.70E-01	-0.05	6.20E-01	0.13	9.11E-01	-0.03	9.81E-01	-0.01
2879.4912@4.541535	2879.4912	4.54	NEG	hilic				9.70E-01	9.22E-01	0.03	5.97E-01	0.14	7.94E-01	-0.08	7.65E-01	-0.09
2902.599@0.38461316	2902.5990	0.38	NEG	hilic				9.79E-01	6.58E-01	0.14	5.12E-01	0.19	4.44E-01	0.19	7.05E-01	0.11
2924.583@0.37209085	2924.5830	0.37	NEG	hilic				6.14E-01	3.47E-01	-0.27	2.83E-01	-0.28	2.17E-01	-0.32	6.53E-01	0.12
2932.5894@3.0427144	2932.5894	3.04	POS	hilic				7.06E-01	9.15E-01	0.02	4.59E-01	0.15	1.49E-01	0.36	2.42E-01	0.27
2934.5889@3.0447211	2934.5889	3.04	POS	hilic				7.88E-01	8.31E-01	0.05	6.36E-01	0.10	1.88E-01	0.32	3.16E-01	0.25
2946.6902@3.22771	2946.6902	3.23	NEG	hilic				7.34E-01	5.16E-01	0.18	9.60E-01	-0.01	1.67E-01	0.39	4.31E-01	0.21
2946.6907@3.199782	2946.6907	3.20	NEG	hilic				2.83E-01	9.62E-02	0.46	2.60E-01	-0.27	9.69E-01	-0.01	2.41E-01	0.38
2947.4753@4.5370793	2947.4753	4.54	NEG	hilic				8.50E-01	3.05E-01	0.31	4.85E-01	0.18	8.45E-01	-0.06	4.71E-01	0.21
2947.4805@4.5350394	2947.4805	4.54	NEG	hilic				9.61E-01	4.32E-01	0.24	6.12E-01	0.15	9.26E-01	0.03	6.50E-01	0.13
2948.6936@3.197855	2948.6936	3.20	NEG	hilic				7.76E-01	2.46E-01	0.33	5.89E-01	0.15	1.10E-01	0.45	6.18E-01	0.17
2950.6868@3.201609	2950.6868	3.20	NEG	hilic				8.06E-01	7.64E-01	0.09	9.93E-01	0.00	5.10E-01	0.20	1.54E-01	0.40
2952.6904@3.2018747	2952.6904	3.20	NEG	hilic				7.88E-01	2.04E-01	0.31	4.56E-01	0.17	6.48E-01	0.12	7.55E-01	-0.07
2967.42@4.4062114	2967.4200	4.41	POS	hilic				5.04E-01	1.19E-01	0.48	3.47E-01	0.24	5.42E-01	0.18	5.03E-01	-0.15
2989.605@0.3658018	2989.6050	0.37	NEG	hilic				7.46E-01	4.62E-01	0.21	4.35E-01	-0.25	7.69E-01	-0.08	3.81E-01	-0.26
3002.6052@0.3793334	3002.6052	0.38	NEG	hilic				8.87E-01	6.37E-01	0.14	4.34E-01	-0.22	6.09E-01	-0.13	8.20E-01	-0.06
3009.9397@10.106555	3009.9397	10.11	POS	RP				9.02E-01	9.13E-01	-0.02	4.01E-01	0.18	9.04E-01	0.02	4.64E-01	0.19
3015.4663@4.540101	3015.4663	4.54	NEG	hilic				3.97E-01	8.56E-01	0.06	1.07E-02	0.64	4.34E-01	0.21	1.71E-01	0.42
3016.5537@3.0479474	3016.5537	3.05	POS	hilic				7.70E-01	2.41E-01	0.40	9.87E-01	0.00	6.01E-01	0.15	8.13E-01	-0.07
3018.5542@3.0539463	3018.5542	3.05	POS	hilic				9.22E-01	9.39E-01	0.03	5.30E-01	0.16	7.53E-01	-0.10	5.45E-01	-0.17
3024.9082@10.108828	3024.9082	10.11	POS	RP				4.74E-02	1.85E-01	0.47	6.59E-01	0.10	1.44E-02	0.57	1.98E-03	0.92
3028.9204@10.107771	3028.9204	10.11	POS	RP				3.73E-03	1.61E-02	0.73	2.65E-01	-0.27	5.85E-01	0.15	1.27E-03	0.91
3030.6394@0.37928867	3030.6394	0.38	NEG	hilic				4.76E-01	3.02E-01	0.31	2.34E-01	-0.37	9.25E-01	-0.02	5.78E-01	0.13
3030.6453@3.2117434	3030.6453	3.21	NEG	hilic				3.19E-01	4.49E-02	0.54	3.44E-02	0.57	1.80E-01	0.43	1.79E-02	0.71
3030.6538@3.1855583	3030.6538	3.19	NEG	hilic				2.78E-01	7.04E-02	0.50	5.19E-02	0.59	8.41E-01	-0.06	2.13E-01	0.32
3032.6504@3.2014794	3032.6504	3.20	NEG	hilic				2.95E-01	6.04E-02	0.54	2.92E-02	0.63	5.04E-02	0.62	2.75E-02	0.62
3032.6565@3.2067778	3032.6565	3.21	NEG	hilic				5.93E-01	2.39E-01	0.34	3.59E-01	0.26	7.23E-02	0.47	6.24E-02	0.54
3034.6516@3.2079556	3034.6516	3.21	NEG	hilic				3.97E-01	3.05E-01	0.30	8.26E-01	0.06	9.21E-02	0.45	3.22E-02	0.63
3036.651@3.2116208	3036.6510	3.21	NEG	hilic				8.77E-01	8.95E-01	0.04	3.89E-01	-0.25	6.66E-01	-0.13	6.57E-01	0.12
3037.948@1.31192	3037.9480	1.31	POS	hilic				7.49E-01	9.10E-01	0.03	4.18E-01	-0.21	6.05E-01	-0.13	1.36E-01	-0.39
3038.645@3.2045248	3038.6450	3.20	NEG	hilic				6.97E-01	1.52E-01	0.40	2.30E-01	0.29	3.76E-01	0.24	1.04E-01	0.50
3039.9663@1.3080002	3039.9663	1.31	POS	hilic				3.95E-02	4.33E-01	0.20	1.34E-01	0.32	7.81E-04	0.91	7.50E-01	0.08
3047.9126@1.3051864	3047.9126	1.31	POS	hilic				6.19E-02	9.21E-01	0.03	9.86E-02	0.36	1.29E-02	0.68	4.84E-01	-0.16
3052.608@0.37415296	3052.6080	0.37	NEG	hilic				3.34E-01	1.06E-01	0.50	6.36E-01	0.12	7.81E-01	-0.08	5.75E-02	0.49
3066.1553@11.930556	3066.1553	11.93	POS	RP				9.15E-01	8.13E-01	-0.06	5.09E-01	-0.16	5.84E-01	0.14	7.84E-01	0.08
3074.312@12.674787	3074.3120	12.67	POS	RP				2.71E-01	4.73E-01	-0.24	2.08E-01	-0.30	1.67E-01	0.32	2.75E-01	0.29
3076.267@12.681978	3076.2670	12.68	POS	RP				8.97E-01	8.57E-01	0.06	5.03E-01	0.20	6.50E-01	-0.12	4.88E-01	0.20
3078.2656@12.677796	3078.2656	12.68	POS	RP				8.42E-01	3.59E-01	0.30	9.31E-01	-0.02	9.04E-01	-0.03	7.25E-01	-0.09
3078.2722@12.686839	3078.2722	12.69	POS	RP				7.14E-01	2.14E-01	0.38	1.31E-01	0.43	7.18E-01	0.09	4.81E-01	0.20
3080.2605@12.692108	3080.2605	12.69	POS	RP				9.61E-01	4.58E-01	0.25	7.17E-01	0.11	6.23E-01	0.12	4.56E-01	0.21
3083.4558@4.5200453	3083.4558	4.52	NEG	hilic				5.03E-01	3.81E-01	0.25	4.06E-01	0.23	1.92E-01	-0.38	9.90E-01	0.00
3083.458@4.534902	3083.4580	4.53	NEG	hilic				7.46E-01	4.66E-01	-0.24	2.98E-01	-0.31	7.58E-02	-0.49	3.51E-01	-0.27
3100.5227@3.0536182	3100.5227	3.05	POS	hilic				2.71E-01	3.57E-01	-0.23	9.03E-01	-0.03	5.85E-02	-0.56	2.51E-02	-0.55
3102.5122@3.0561216	3102.5122	3.06	POS	hilic				4.29E-01	3.18E-01	-0.26	4.90E-01	-0.19	2.58E-02	-0.64	1.55E-01	-0.37
3103.391@4.40947	3103.3910	4.41	POS	hilic				8.56E-01	4.94E-01	-0.23	3.15E-01	-0.28	9.93E-01	0.00	8.79E-01	0.05
3108.1812@12.194464	3108.1812	12.19	POS	RP				8.53E-01	4.37E-01	-0.20	5.86E-01	-0.14	8.61E-01	-0.04	5.63E-01	0.17
3114.614@3.200944	3114.6140	3.20	NEG	hilic				7.20E-01	1.54E-01	0.42	5.37E-01	0.16	2.55E-01	0.34	1.81E-01	0.40
3114.6145@3.1968672	3114.6145	3.20	NEG	hilic				6.95E-01	4.21E-01	0.23	8.14E-01	-0.06	5.36E-01	-0.20	3.05E-01	0.29
3116.1604@11.9513	3116.1604	11.95	POS	RP				6.28E-01	2.73E-01	0.32	6.25E-01	-0.11	7.75E-01	-0.08	3.17E-01	-0.30
3116.6118@3.1951194	3116.6118	3.20	NEG	hilic				9.29E-01	9.00E-01	0.04	9.24E-01	-0.03	6.26E-01	0.16	3.48E-01	0.26
3116.6123@3.1968086	3116.6123	3.20	NEG	hilic				7.10E-01	6.82E-01	0.11	6.46E-01	-0.13	5.39E-01	0.19	2.05E-01	0.39

Supplementary table 2: Metabolomics analysis results

								ANOVA	Anorexia vs control		overweight vs control		obese vs control		athletes vs control	
Compound	Mass	Retention Time	Ionization mode	column	Compound_ID	MSMS_spectra	ID_level	P-FDR	P	Estimate	P	Estimate	P	Estimate	P	Estimate
3116.6233@0.3656364	3116.6233	0.37	NEG	hilic				7.33E-01	6.96E-01	-0.12	1.60E-01	-0.46	2.21E-01	-0.35	3.21E-01	-0.28
3117.6255@0.3655189	3117.6255	0.37	NEG	hilic				8.38E-01	8.19E-01	0.07	3.53E-01	-0.30	3.94E-01	-0.25	5.97E-01	-0.16
3118.615@3.1985505	3118.6150	3.20	NEG	hilic				8.18E-01	4.89E-01	0.21	9.24E-01	0.03	3.84E-01	0.26	1.94E-01	0.38
3120.1914@11.988333	3120.1914	11.99	POS	RP				1.01E-02	5.00E-01	0.19	1.45E-02	-0.68	1.28E-01	-0.44	1.43E-01	0.42
3120.6118@3.2031603	3120.6118	3.20	NEG	hilic				7.72E-01	5.36E-01	0.16	3.15E-01	-0.26	5.30E-01	-0.17	8.95E-01	0.04
3124.0354@10.22975	3124.0354	10.23	POS	RP				1.15E-02	5.74E-01	0.16	3.61E-03	-0.68	2.58E-03	-0.68	4.65E-01	-0.19
3130.6453@0.3780367	3130.6453	0.38	NEG	hilic				2.85E-01	3.97E-02	0.59	8.45E-01	-0.05	2.25E-01	0.29	1.17E-01	0.44
3144.218@12.367776	3144.2180	12.37	POS	RP				8.60E-01	7.74E-01	0.08	3.88E-01	0.25	5.77E-01	-0.16	8.10E-01	0.07
3146.2422@12.368482	3146.2422	12.37	POS	RP				9.17E-01	8.71E-01	0.05	4.12E-01	0.23	7.05E-01	0.09	7.04E-01	-0.11
3150.2317@12.36957	3150.2317	12.37	POS	RP				9.61E-01	9.22E-01	-0.03	8.27E-01	-0.06	8.46E-01	-0.06	3.73E-01	-0.25
3151.2527@12.370184	3151.2527	12.37	POS	RP				7.38E-01	5.07E-01	-0.12	5.08E-01	0.16	2.60E-01	0.26	8.25E-01	0.04
3151.4407@4.5364857	3151.4407	4.54	NEG	hilic				6.68E-01	2.24E-01	0.35	2.50E-01	0.33	6.06E-01	0.16	1.09E-01	0.52
3152.2488@12.370209	3152.2488	12.37	POS	RP				9.33E-01	7.52E-01	0.08	8.11E-01	-0.07	8.31E-01	-0.06	4.63E-01	0.22
3153.2446@12.3706875	3153.2446	12.37	POS	RP				4.05E-01	1.50E-01	-0.37	2.31E-01	-0.29	4.60E-02	-0.44	1.85E-02	-0.49
3154.2112@12.169572	3154.2112	12.17	POS	RP				4.44E-01	2.63E-01	0.30	4.52E-01	-0.21	1.73E-01	0.42	3.60E-01	0.22
3154.2578@12.372022	3154.2578	12.37	POS	RP				8.66E-01	4.44E-01	-0.21	9.75E-01	0.01	2.35E-01	-0.32	7.80E-01	-0.07
3155.1719@12.169055	3155.1719	12.17	POS	RP				2.64E-02	6.98E-02	0.51	1.61E-01	0.40	3.39E-04	1.00	9.66E-01	-0.01
3156.1804@12.167585	3156.1804	12.17	POS	RP				8.68E-01	6.74E-01	0.11	4.00E-01	-0.21	6.56E-01	0.13	8.52E-01	-0.05
3157.171@12.16815	3157.1710	12.17	POS	RP				5.34E-01	6.09E-02	0.57	1.57E-01	0.39	7.66E-01	0.09	5.13E-01	0.17
3158.274@12.367857	3158.2740	12.37	POS	RP				6.87E-01	7.57E-01	0.09	9.01E-01	0.03	4.66E-01	-0.17	2.00E-01	0.33
3158.6472@0.37867454	3158.6472	0.38	NEG	hilic				9.06E-01	8.93E-01	-0.04	5.79E-01	-0.16	8.53E-01	-0.05	2.86E-01	-0.31
3159.266@12.367618	3159.2660	12.37	POS	RP				5.25E-01	5.37E-01	0.19	2.66E-01	-0.25	1.37E-01	-0.30	3.86E-01	-0.20
3160.2344@12.367769	3160.2344	12.37	POS	RP				4.27E-01	4.25E-01	0.24	2.24E-01	-0.28	1.12E-01	-0.37	7.39E-01	-0.09
3160.2463@12.367744	3160.2463	12.37	POS	RP				9.51E-01	8.55E-01	-0.05	7.69E-01	-0.09	8.62E-01	-0.05	3.30E-01	-0.27
3162.188@12.148859	3162.1880	12.15	POS	RP				3.08E-02	6.72E-01	0.14	1.25E-02	0.74	1.38E-03	0.83	9.18E-01	-0.02
3162.2273@12.36864	3162.2273	12.37	POS	RP				2.50E-01	4.09E-01	0.23	7.33E-02	-0.48	1.32E-01	-0.41	8.42E-01	-0.06
3163.218@12.36973	3163.2180	12.37	POS	RP				5.22E-01	2.07E-01	0.40	3.93E-01	-0.25	5.43E-01	0.16	4.27E-01	0.23
3164.182@12.12312	3164.1820	12.12	POS	RP				8.28E-01	5.27E-01	0.17	8.28E-01	0.06	1.64E-01	0.39	5.74E-01	0.15
3165.2424@12.370232	3165.2424	12.37	POS	RP				4.38E-01	4.78E-01	0.21	1.11E-01	-0.47	6.54E-01	0.12	8.12E-01	-0.07
3166.2295@12.368372	3166.2295	12.37	POS	RP				3.87E-01	4.09E-01	0.21	7.08E-02	0.53	3.61E-02	0.54	6.06E-01	0.13
3167.2253@12.367832	3167.2253	12.37	POS	RP				7.68E-01	4.09E-01	-0.22	7.19E-01	0.10	8.34E-01	0.05	3.08E-01	-0.27
3168.2212@12.404277	3168.2212	12.40	POS	RP				8.05E-01	3.40E-01	0.25	7.08E-01	0.12	6.79E-01	-0.12	5.26E-01	-0.16
3169.225@12.368335	3169.2250	12.37	POS	RP				6.77E-01	2.94E-01	0.29	3.74E-01	0.27	7.59E-01	-0.09	5.25E-01	-0.16
3172.2158@12.380787	3172.2158	12.38	POS	RP				9.51E-01	5.18E-01	-0.15	9.49E-01	-0.02	5.49E-01	-0.17	3.66E-01	-0.24
3173.215@12.369796	3173.2150	12.37	POS	RP				8.28E-01	7.88E-01	-0.07	5.02E-01	0.19	6.39E-01	-0.13	4.48E-01	-0.19
3174.23@12.4066105	3174.2300	12.41	POS	RP				7.64E-01	4.07E-01	0.21	6.82E-01	0.13	5.84E-01	-0.16	3.71E-01	-0.23
3180.6306@0.3739636	3180.6306	0.37	NEG	hilic				7.55E-01	6.49E-01	0.14	2.57E-01	-0.36	5.96E-01	-0.16	7.68E-01	-0.08
3184.4695@3.0539305	3184.4695	3.05	POS	hilic				5.65E-01	1.80E-01	0.47	8.21E-01	-0.07	3.08E-01	0.29	7.97E-01	-0.07
3186.4663@3.0533683	3186.4663	3.05	POS	hilic				9.16E-01	3.76E-01	0.34	5.66E-01	0.16	6.11E-01	0.13	8.79E-01	0.04
3187.2246@12.370239	3187.2246	12.37	POS	RP				9.68E-01	8.51E-01	0.05	6.45E-01	0.10	9.60E-01	0.01	4.03E-01	0.19
3192.2307@12.369685	3192.2307	12.37	POS	RP				7.20E-01	6.86E-01	0.11	7.22E-01	0.09	3.05E-01	0.29	3.79E-01	-0.20
3198.5515@3.2003334	3198.5515	3.20	NEG	hilic				8.35E-01	6.34E-01	0.14	9.62E-01	-0.01	7.60E-01	0.09	2.34E-01	0.35
